# Supplementary material for: Identifying and profiling structural similarities between Spike of SARS-CoV-2 and other viral or host proteins with Machaon
Source: Commun Biol. 2023 Jul 19;6:752. doi: 10.1038/s42003-023-05076-7 (PMC10356814; doi:10.1038/s42003-023-05076-7)
Supplement: Supplementary file 2 — Supplementary Information [file 42003_2023_5076_MOESM2_ESM.pdf]

Supplementary Tables

Metrics	Rounded median (%)	
	Gaps included	Gaps excluded
Protein 1D identity	12.98/5.51/3.99	29.82/27.37/28.05
Protein 2D identity (PDB)	32.72/9.96/14.13	88.08/88.52/87.90
5-UTR identity	23.60/22.48/23.60	79.04/82.22/76.72
CDS identity	38.54/18.42/18.45	77.79/78.91/79.36
3-UTR identity	23.63/32.23/21.18	80.19/79.90/80.65
3D similarity	22.06/8.07/8.74	
Chemical similarity	86.07/85.38/82.96	

Table 1: Sequence identity from global alignments on the final output sets from the comparisons with the viral dataset by the method's different modes (**whole structure/domain/binding sites**), targeting native SARS-CoV-2 Spike protein monomer. Protein 2D secondary structure is treated as a sequence of different secondary structure states as defined by DSSP. Evaluations on three-dimensional similarity by TM-Align and on chemical similarity by Tanimoto Index are also displayed.

Protein	Associated GO terms	Details
DNA damage-binding protein 1 (DDB1)	Biological processes:<br>• histone H2A mono**ubiquit**ination<br>• proteasome-mediated **ubiquit**in-dependent protein catabolic process<br>• protein **ubiquit**ination<br>• **ubiquit**in-dependent protein catabolic process<br><br>Cellular components:<br>• Cul4-RING E3 **ubiquit**in ligase complex<br>• Cul4A-RING E3 **ubiquit**in ligase complex<br>• Cul4B-RING E3 **ubiquit**in ligase complex	Reported in the top 100 results for all three Spikes (Native / Delta / Omicron)<br><br>Positions in the results for each Spike:<br>18 / 20 / 28<br><br>[b-phipsi, w-rdist, t-alpha] for each Spike:<br>[0.007194, 1.107738, 0.023709]/<br>[0.015288, 1.27945, 0.036705]/<br>[0.011142, 1.263002, 0.045724]<br><br>2D similarity for each Spike:<br>30.72% / 34.66% / 32.76%<br><br>3D similarity for each Spike: 20.02% / 19.53% /17.4%<br><br>Genomic similarity with Spike - 5-UTR|CDS|3-UTR identity (%):<br>27.05 | 40.25 | 20.28

Pre-mRNA-processing-splicing factor 8 (PRPF8)	Molecular function:<br><br>• K63-linked poly**ubiquit**in modification-dependent protein binding	Reported in the top 100 results for the variant Spikes (Delta / Omicron)<br><br>Positions in the results for each Spike:<br>23 / 40<br><br>[b-phipsi, w-rdist, t-alpha] for each Spike:<br>[0.041555, 0.80366, 0.070024]/<br>[0.032376, 0.889346, 0.101215]<br><br>2D similarity for each Spike:<br>N/A because of the large length of the PRPF8's sequence (2211 residues in PDB)<br><br>3D similarity for each Spike:<br>4.85% / 5.34%<br><br>Genomic similarity with Spike - 5-UTR|CDS|3-UTR identity (%):<br>20.92 | 34.86 | 33.45
Cullin-4A (CUL4A)	Molecular functions:<br><br>• **ubiquit**in protein ligase binding<br>• **ubiquit**in-protein transferase activity<br><br>Biological processes:<br><br>• proteasome-mediated **ubiquit**in-dependent protein catabolic process<br>• protein **ubiquit**ination<br>• SCF-dependent proteasomal **ubiquit**in-dependent protein catabolic process<br><br>Cellular components:<br><br>• Cul4-RING E3 **ubiquit**in ligase complex<br>• Cul4A-RING E3 **ubiquit**in ligase complex<br>• cullin-RING **ubiquit**in ligase complex<br>• nucleoplasm<br>• SCF **ubiquit**in ligase complex	Reported in the top 100 results for the variant Spikes (Delta / Omicron)<br><br>Positions in the results for each Spike:<br>50 / 50<br><br>[b-phipsi, w-rdist, t-alpha] for each Spike:<br>[0.091276, 0.806863, 0.369612]/<br>[0.093818, 0.791059, 0.330819]<br><br>2D similarity for each Spike:<br>20.15% / 18.98%<br><br>3D similarity for each Spike:<br>17.4% / 19.43%<br><br>Genomic similarity with Spike - 5-UTR|CDS|3-UTR identity (%):<br>N/A

Ubiquitin carboxyl-terminal hydrolase 7 (USP7)	Molecular functions:<br><br>• **ubiquit**inyl hydrolase activity<br>• Lys48-specific de**ubiquit**inase activity<br>• thiol-dependent **ubiquit**in-specific protease activity<br>• **ubiquit**in protein ligase binding<br><br>Biological processes:<br><br>• histone H2B conserved C-terminal lysine de**ubiquit**ination<br>• mono**ubiquit**inated protein de**ubiquit**ination<br>• protein de**ubiquit**ination<br>• protein K63-linked de**ubiquit**ination<br>• protein **ubiquit**ination<br>• **ubiquit**in-dependent protein catabolic process	Reported in the top 100 results for the Omicron variant Spike<br><br>Position in the results:<br>26<br><br>[b-phipsi, w-rdist, t-alpha]:<br>[0.003217, 0.96409, 0.352683]<br><br>2D similarity:<br>23.62%<br><br>3D similarity:<br>1.04%<br><br>Genomic similarity with Spike - 5-UTR|CDS|3-UTR identity (%):<br>8.07 | 42.68 | 7.82

Table 2: The proteins in the final sets of whole structure comparisons between the viral dataset and the Spike monomers of native SARS-CoV-2 and Delta/Omicron variants, that were found to be associated with Gene Ontology terms that contain "**ubiquit**" substring.

	b-phipsi	w-rdist	t-alpha
b-phipsi	1.000000	0.118785	0.075813
w-rdist	0.118785	1.000000	0.089612
t-alpha	0.075813	0.089612	1.000000

Table 3: Correlation matrix of b-phipsi, w-rdist and t-alpha based on whole structure comparisons between SARS-CoV-2 Spike monomer (native) and the proteins in the paper's target dataset. We see that the metrics are segregated and therefore there is no redundancy of information.

Alternative solution	Results
RCSB PDB	SARS-CoV Spike protein is included in the results of the new structural similarity search in RCSB PDB along with SARS-CoV Spike protein - Escherichia T4 viral protein chimeric entries.
SCOP database	Only the associated Coronavirus proteins are enlisted.
CATH databse	Only the associated Coronavirus proteins are enlisted.
Aquaria-COVID	Murine Coronavirus Spike protein is listed together with other Coronavirus family proteins in the matching structures displayed for Spike protein entry.
DALI server	Server's results encompass Human Coronavirus family proteins and ACE2 receptor.
BLAST	It was mainly able to pinpoint proteins of Coronaviruses by sequence similarity.

Table 4: The results in alternative methods on finding similar structures with Spike protein during the writing of the manuscript.

Hydrophobicity group	Aminoacids
0	Ile, Val
1	Leu
2	Phe,Cys,Met,Ala
3	Gly,Thr,Ser,Trp,Tyr,Pro
4	His,Asn,Gln,Asp,Glu,Lys
5	Arg

Table 5: Hydrophobicity groups based on Hydropathy Index, which are encoded into the mixed sequence alignments in the method's constrained mode search.

Site Index	Residue positions
0	1, 38, 39, 40, 41, 42, 43, 44, 45, 46, 47, 48, 49, 50, 53, 195, 197, 198, 200, 202, 203, 204, 225, 227, 228, 274, 276, 281, 291, 298, 301, 302, 303, 304, 305, 312, 313, 314, 315, 316, 317, 319, 320, 321, 322, 355, 378, 379, 380, 381, 382, 383, 384, 386, 393, 396, 412, 426, 427, 428, 429, 430, 464, 514, 515, 516, 517, 518, 520, 540, 547, 548, 549, 550, 567, 568, 569, 570, 571, 574, 589, 590, 592, 596, 613, 735, 736, 737, 738, 739, 740, 743, 744, 745, 746, 747, 748, 750, 752, 753, 754, 755, 756, 757, 758, 759, 760, 761, 762, 763, 764, 765, 766, 767, 768, 769, 770, 773, 854, 855, 856, 858, 951, 954, 957, 958, 959, 960, 961, 962, 963, 964, 965, 967, 968, 969, 970, 971, 972, 973, 978, 981, 982, 983, 984, 985, 986, 987, 988, 989, 990, 991, 992, 993, 994, 995, 998, 999, 1001, 1002, 1003, 1005, 1006, 1007, 1008, 1009, 1010, 1012, 1013, 1014, 1015, 1016, 1017, 1019

1	37, 38, 39, 40, 41, 42, 43, 44, 45, 47, 48, 49, 50, 53, 195, 197, 198, 200, 202, 203, 204, 225, 226, 227, 228, 230, 274, 291, 298, 301, 302, 303, 304, 312, 313, 314, 315, 316, 317, 318, 319, 320, 321, 322, 355, 357, 380, 381, 382, 390, 391, 392, 393, 394, 396, 412, 426, 427, 428, 429, 430, 464, 514, 515, 516, 517, 518, 519, 520, 521, 522, 523, 541, 543, 544, 545, 546, 547, 548, 549, 550, 557, 565, 567, 568, 569, 571, 574, 589, 590, 591, 592, 595, 596, 611, 612, 613, 614, 646, 647, 661, 662, 665, 666, 667, 697, 698, 699, 700, 733, 735, 736, 737, 738, 739, 740, 743, 744, 745, 746, 747, 750, 754, 755, 757, 758, 761, 762, 763, 764, 765, 767, 768, 771, 772, 775, 776, 779, 783, 785, 786, 854, 861, 862, 864, 865, 873, 957, 960, 961, 964, 965, 967, 968, 969, 973, 974, 975, 976, 978, 979, 982, 983, 1004
2	1, 725, 727, 728, 769, 770, 772, 773, 776, 777, 780, 781, 784, 785, 786, 789, 888, 889, 891, 947, 950, 951, 954, 1012, 1013, 1014, 1015, 1016, 1017, 1018, 1019, 1020, 1021, 1022, 1023, 1024, 1026, 1027, 1028, 1030, 1031, 1034, 1039, 1040, 1041, 1042, 1043, 1044, 1045, 1064
3	86, 87, 88, 89, 107, 108, 109, 113, 114, 115, 130, 132, 167, 168, 195, 196, 197, 198, 199, 200, 229, 230, 231, 232, 233, 234, 235, 236, 353, 354, 355, 356, 357, 396, 454, 456, 457, 458, 459, 460, 461, 462, 463, 464, 465, 466, 467, 468, 469, 470, 471, 472, 473, 474, 475, 476, 477, 478, 479, 480, 486, 487, 488, 489, 490, 491, 492
4	41, 197, 198, 200, 202, 228, 355, 368, 369, 370, 371, 372, 373, 374, 375, 376, 377, 378, 379, 380, 381, 382, 383, 384, 390, 391, 392, 393, 396, 403, 405, 406, 407, 408, 409, 410, 411, 412, 413, 414, 415, 416, 417, 421, 424, 426, 427, 428, 429, 430, 433, 436, 437, 439, 440, 464, 505, 514, 515, 516, 517, 518, 519, 520, 522, 544, 545, 546, 547, 565, 567, 571, 973, 974, 976, 978, 979, 982, 983, 984, 985, 986, 987, 988, 991, 992

Table 6: Predicted binding sites by Schrödinger SiteMap for SARS-CoV-2 Spike protein (native). The predictions derive from PDB 6VXX.A that was prepared with Protein Preparation Wizard of Schrödinger Maestro Suite.

Supplementary Figures

a

Structural Comparison Report for 6VXX_A - whole structures (total: 58)

1

- **Protein name:** Spike glycoprotein
- **Organism:** Severe acute respiratory syndrome coronavirus
- **Uniprot Accession Number:** P59594
- **Protein sequence length:** 1255 aa
- **1D identity (%):** 76.35
- **1D identity (%) [Gaps excluded]:** 77.94
- **1D identity - Alignment Gaps:** 26
- **Common reported functions (%):** 100.0
- **Common reported locations (%):** 62.5
- **Common reported processes (%):** 90.0

- **PDB ID:** 6NB6
- **Chain:** A
- **Crystallized protein length:** 1052 aa
- **Resolution:** 4.2 Å
- **b-phipsi:** 0.001058
- **w-rdist:** 0.5121
- **t-alpha:** 0.002481
- **Chemical similarity (Tanimoto Index) (%):** 94.56
- **1D identity (%) [PDB]:** 69.31
- **1D identity (%) [Gaps excluded][PDB]:** 78.58
- **1D identity - Alignment Gaps [PDB]:** 128
- **2D identity (%) [PDB]:** 72.68
- **2D identity (%) [Gaps excluded][PDB]:** 86.39
- **2D identity - Alignment Gaps [PDB]:** 176
- **3D similarity (TM-Score) (%) [PDB]:** 96.46

- **Gene name:** S
- **RefSeq ID:** NC_004718
- **Genomic sequence length:** 29751
- **5-UTR|CDS|3-UTR identity (%):** 88.52 | 73.15 | 22.38
- **5-UTR|CDS|3-UTR identity (%) [Gaps excluded]:** 92.28 | 78.79 | 98.18
- **5-UTR|CDS|3-UTR identity [Alignment Gaps]:** 11 | 282 | 745

Uniprot Description:

Spike glycoprotein May down-regulate host tetherin (BST2) by lysosomal degradation, thereby counteracting its antiviral activity.

Homotrimer; each monomer consists of a S1 and a S2 subunit. The resulting peplomers protrude from the virus surface as spikes (By similarity). Binds to human and palm civet ACE2 and human CLEC4M/DC-SIGNR. Interacts with the accessory proteins 3a and 7a.

Gene Ontology Information:

Molecular Function

- host cell surface receptor binding
- identical protein binding

Location

- host cell endoplasmic reticulum-Golgi intermediate compartment membrane
- host cell plasma membrane
- integral component of membrane
- viral envelope
- virion membrane

Biological process

- endocytosis involved in viral entry into host cell
- fusion of virus membrane with host endosome membrane
- fusion of virus membrane with host plasma membrane
- pathogenesis
- receptor-mediated virion attachment to host cell
- suppression by virus of host tetherin activity
- suppression by virus of host type I interferon-mediated signaling pathway
- viral protein processing
- viral translation

b

Structural Comparison Report for 6VXX_A - whole structures (total: 58)

1

Protein name: Spike glycoprotein **Organism:** Severe acute respiratory syndrome coronavirus **Uniprot Accession Number:** P59594 **Protein sequence length:** 1255 aa **1D identity (%):** 76.35 **1D identity (%) [Gaps excluded]:** 77.94 **1D identity - Alignment Gaps:** 26 **Common reported functions (%):** 100.0 **Common reported locations (%):** 62.5 **Common reported processes (%):** 90.0

PDB ID: 6NB6 **Chain:** A **Crystallized protein length:** 1052 aa **Resolution:** 4.2 Å **b-phipsi:** 0.001058 **w-rdist:** 0.5121 **t-alpha:** 0.002481 **Chemical similarity (Tanimoto Index) (%):** 94.56 **1D identity (%) [PDB]:** 69.31 **1D identity (%) [Gaps excluded][PDB]:** 78.58 **1D identity - Alignment Gaps [PDB]:** 128 **2D identity (%) [PDB]:** 72.68 **2D identity (%) [Gaps excluded][PDB]:** 86.39 **2D identity - Alignment Gaps [PDB]:** 176 **3D similarity (TM-Score) (%) [PDB]:** 96.46

Gene name: S **RefSeq ID:** NC_004718 **Genomic sequence length:** 29751 **5-UTR|CDS|3-UTR identity (%):** 88.52 | 73.15 | 22.38 **5-UTR|CDS|3-UTR identity (%) [Gaps excluded]:** 92.28 | 78.79 | 98.18 **5-UTR|CDS|3-UTR identity [Alignment Gaps]:** 11 | 282 | 745

Figure 1: Sample final report of Machaon in printer-friendly, readable formats. This is a formatted output for a protein profile built by Machaon in Hypertext Markup Language (HMTL) and Cascading Style Sheets (CSS). Figure **a** is the browser display format and **b** is the compact printed PDF view format.

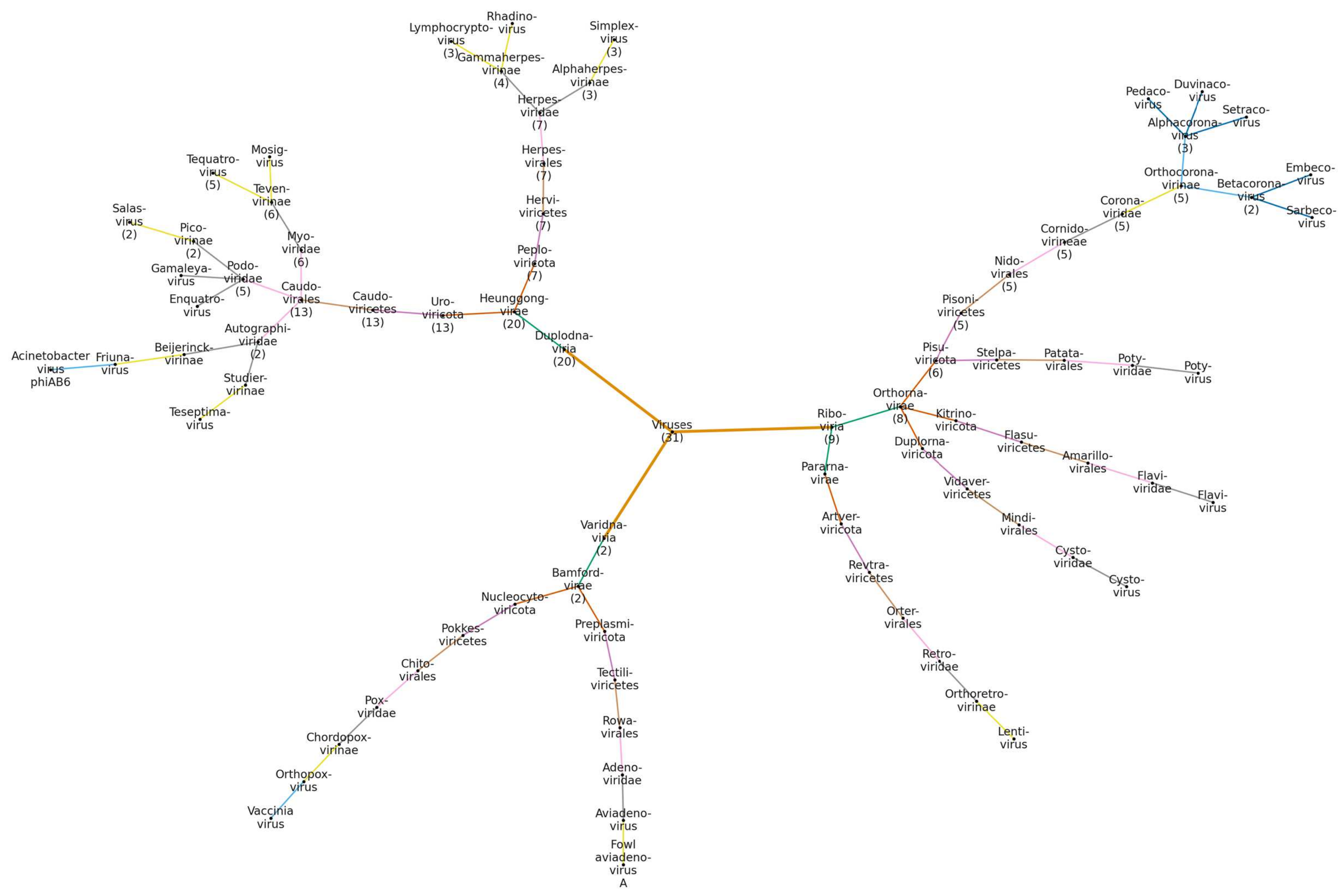
Viruses (31)
Duplodna-viria (20)
Heunggong-virae (20)
Uro-viricota (13)
Caudo-viricetes (13)
Caudo-virales (13)
Myo-viridae (6)
Teven-virinae (6)
Tequatro-virus (5)
Mosig-virus
Podo-viridae (5)
Pico-virinae (2)
Salas-virus (2)
Gamaleya-virus
Enquatro-virus
Autographi-viridae (2)
Beijerinck-virinae
Friuna-virus
Acinetobacter virus phiAB6
Studier-virinae
Teseptima-virus
Peplo-viricota (7)
Hervi-viricetes (7)
Herpes-virales (7)
Herpes-viridae (7)
Gammaherpes-virinae (4)
Lymphocrypto-virus (3)
Rhadino-virus
Alphaherpes-virinae (3)
Simplex-virus (3)
Varidna-viria (2)
Bamford-virae (2)
Nucleocyto-viricota
Pokkes-viricetes
Chito-virales
Pox-viridae
Chordopox-virinae
Orthopox-virus
Vaccinia virus
Preplasmi-viricota
Tectili-viricetes
Rowa-virales
Adeno-viridae
Aviadeno-virus
Fowl aviadeno-virus A
Ribo-viria (9)
Pararna-virae
Artver-viricota
Revtra-viricetes
Orter-virales
Retro-viridae
Orthoretro-virinae
Lenti-virus
Orthorna-virae (8)
Pisu-viricota (6)
Pisoni-viricetes (5)
Nido-virales (5)
Cornido-virineae (5)
Corona-viridae (5)
Orthocorona-virinae (5)
Alphacorona-virus (3)
Pedaco-virus
Duvinaco-virus
Setraco-virus
Betacorona-virus (2)
Embeco-virus
Sarbeco-virus
Stelpa-viricetes
Patata-virales
Poty-viridae
Poty-virus
Kitrino-viricota
Flasu-viricetes
Amarillo-virales
Flavi-viridae
Flavi-virus
Duplorna-viricota
Vidaver-viricetes
Mindi-virales
Cysto-viridae
Cysto-virus

Figure 2: The lineage tree of the viral proteins in the final set of the whole structural comparisons with Delta variant's Spike monomer and viral dataset. These trees are generated by Machaon's presentation module. Each family name carries also a population number if there are more than one protein categorized under it. The root of the tree starts with thicker branches and the colors designated the branch levels. The tree refers to the viral proteins that were found to be the most structurally relevant. These trees could also be considered as distant evolutionary trees according to the structural traits of the proteins in the results. The lineage information is retrieved from UniProt. (Zoom to review)

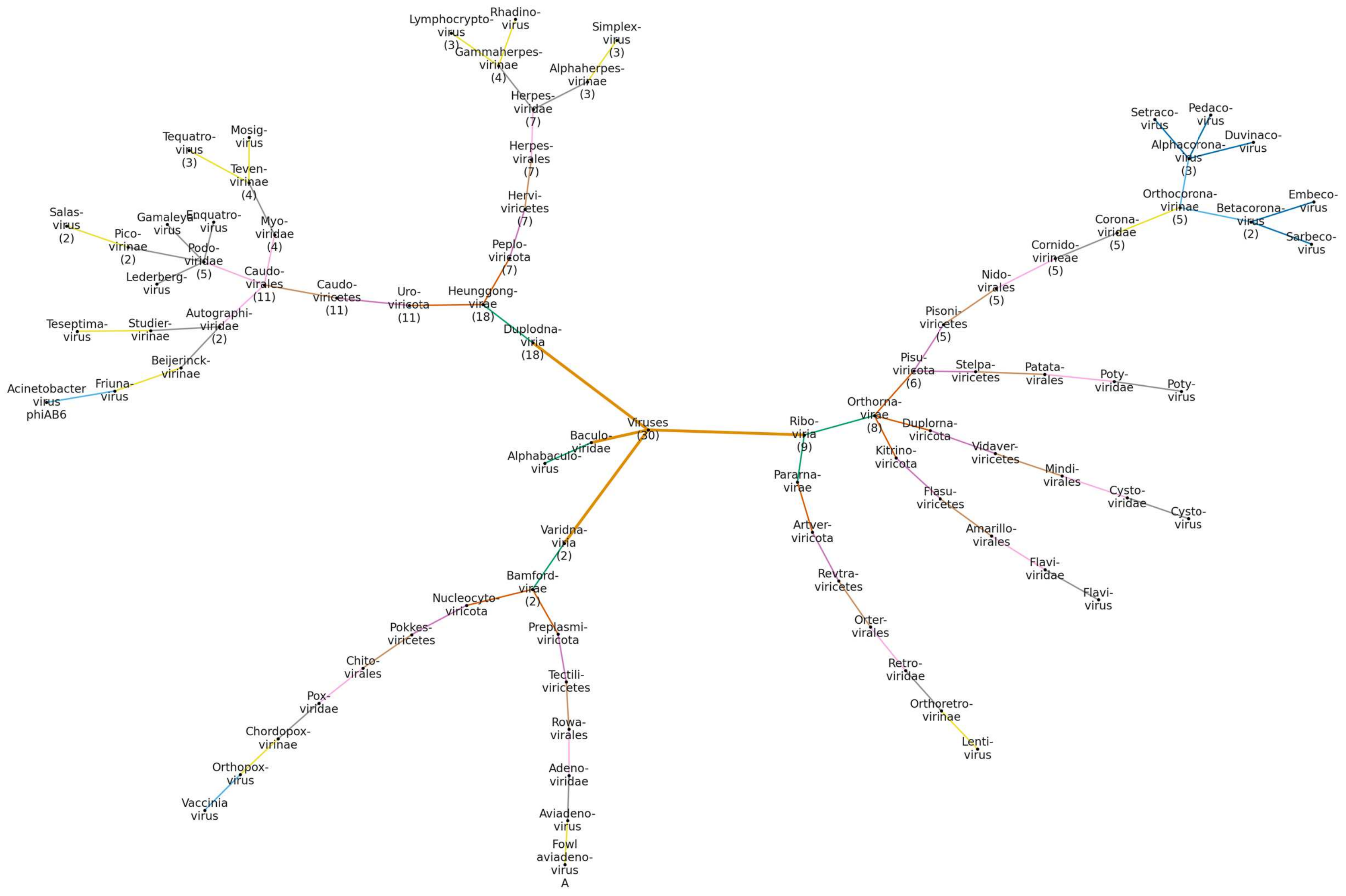

Viruses (30)
Duplodnaviria (18)
Heunggongvirae (18)
Uroviricota (11)
Caudoviricetes (11)
Caudovirales (11)
Myoviridae (4)
Tevenvirinae (4)
Tequatrovirus (3)
Mosigvirus
Podoviridae (5)
Picovirinae (2)
Salasvirus (2)
Gamaleyavirus
Enquatrovirus
Lederbergvirus
Autographiviridae (2)
Studiervirinae
Teseptimavirus
Beijerinckvirinae
Friunavirus
Acinetobacter virus phiAB6
Peploviricota (7)
Herviviricetes (7)
Herpesvirales (7)
Herpesviridae (7)
Gammaherpesvirinae (4)
Lymphocryptovirus (3)
Rhadinovirus
Alphaherpesvirinae (3)
Simplexvirus (3)
Baculoviridae
Alphabaculovirus
Varidnaviria (2)
Bamfordvirae (2)
Nucleocytoviricota
Pokkesviricetes
Chitovirales
Poxviridae
Chordopoxvirinae
Orthopoxvirus
Vaccinia virus
Preplasmiviricota
Tectiliviricetes
Rowavirales
Adenoviridae
Aviadenovirus
Fowl aviadenovirus A
Riboviria (9)
Pararnavirae
Artverviricota
Revtraviricetes
Ortervirales
Retroviridae
Orthoretrovirinae
Lentivirus
Orthornavirae (8)
Pisuviricota (6)
Pisoniviricetes (5)
Nidovirales (5)
Cornidovirineae (5)
Coronaviridae (5)
Orthocoronavirinae (5)
Alphacoronavirus (3)
Setracovirus
Pedacovirus
Duvinacovirus
Betacoronavirus (2)
Embecovirus
Sarbecovirus
Stelpaviricetes
Patatavirales
Potyviridae
Potyvirus
Duplornaviricota
Vidaverviricetes
Mindivirales
Cystoviridae
Cystovirus
Kitrinoviricota
Flasuviricetes
Amarillovirales
Flaviviridae
Flavivirus

Figure 3: The lineage tree of the viral proteins in the final set of the whole structural comparisons with Omicron variant's Spike monomer and viral dataset. These trees are generated by Machaon's presentation module. Each family name carries also a population number if there are more than one protein categorized under it. The root of the tree starts with thicker branches and the colors designated the branch levels. The tree refers to the viral proteins that were found most structurally relevant. These trees could also be considered as distant evolutionary trees according to the structural traits of the proteins in the results. The lineage information is retrieved from UniProt. (Zoom to review)

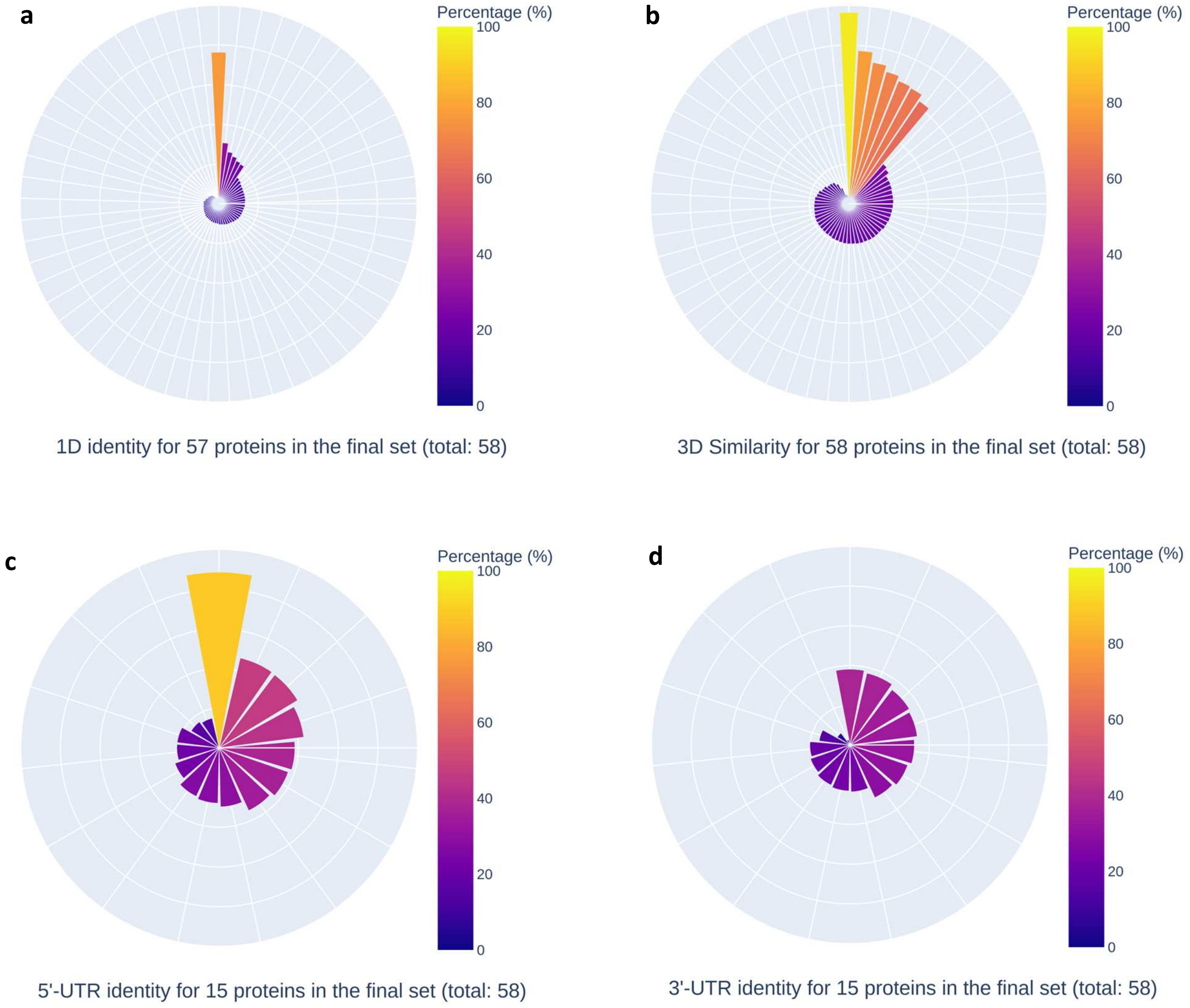

Figure 4: Evaluation of whole structure comparison results with SARS-CoV-2 Spike protein monomer (native) and viral dataset. Each protruding bar from the center of each radial plot represents a protein entry from the final set yielded by Machaon. The plots are generated by the presentation module. The measurements were carried out by the evaluation module wherever the underlying data allowed it (missing or malformed relevant data). **a)** Protein 1D sequence identities **b)** 3D similarities (TM-Score) **c)** 5'-end Untranslated Region identities **d)** 3'-end Untranslated Region sequence identities.

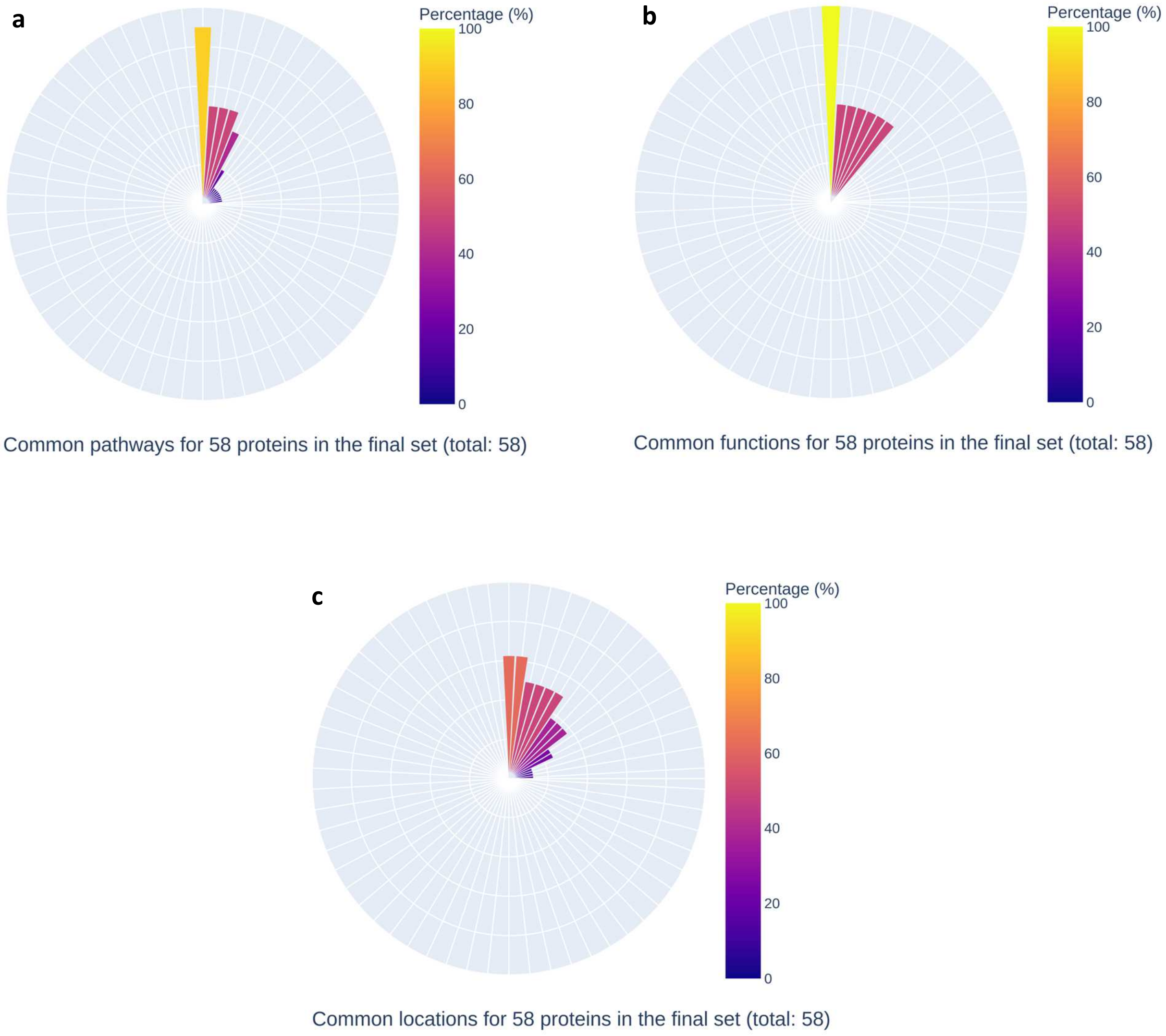

Figure 5: Common Gene Ontology terms on whole structure comparison results with SARS-CoV-2 Spike protein monomer (native) and viral dataset. Each protruding bar from the center of each radial plot represents a protein entry from the final set yielded by Machaon. The plots are generated by the presentation module. **a-c)** Common Gene Ontology terms retrieved from UniProt/EMBL QuickGo: **a** for biological processes, **b** for molecular functions and **c** for cellular locations.

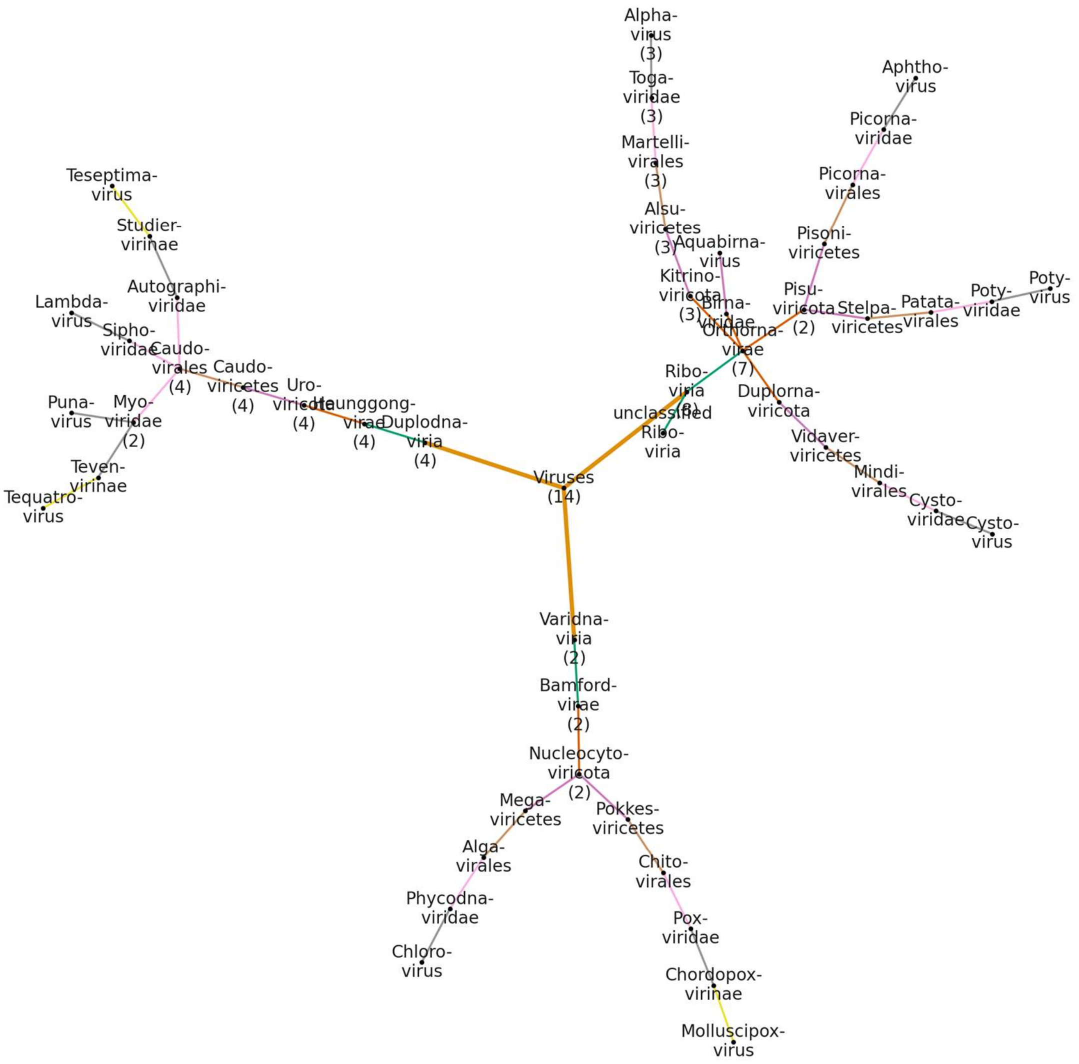

Figure 6: The lineage tree of the viral proteins in the final set of the constrained structural comparisons with S1 N-terminal domain (NTD) of Spike protein monomer (native) and viral dataset. These trees are generated by Machaon's presentation module. Each family name carries also a population number if there are more than one protein categorized under it. The root of the tree starts with thicker branches and the colors designated the branch levels. The tree refers to the viral proteins that were found most structurally relevant. These trees could also be considered as distant evolutionary trees according to the structural traits of the proteins in the results. The lineage information is retrieved from UniProt. (Zoom to review)

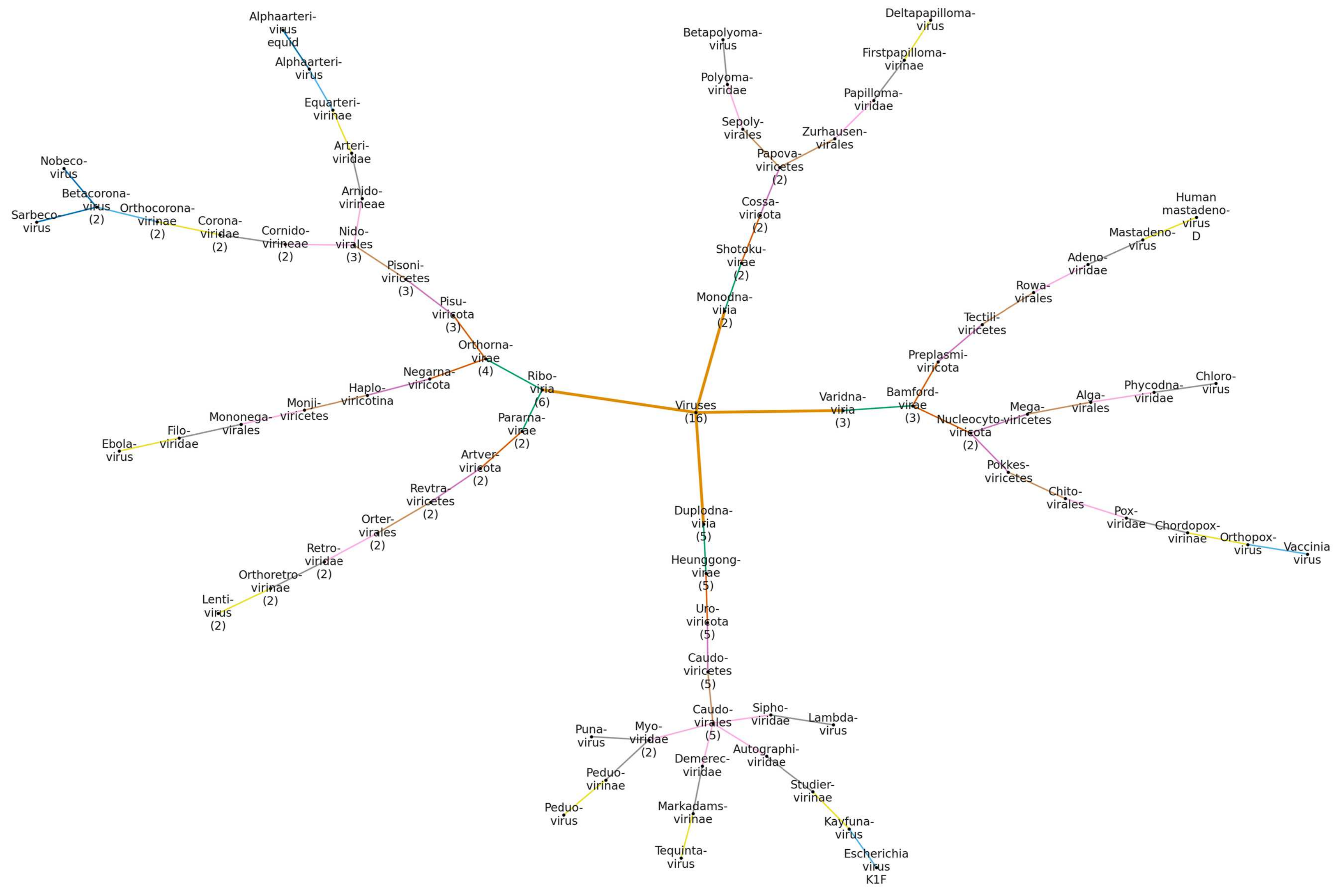
Viruses (16)
Riboviria (6)
Orthornavirae (4)
Pisuviricota (3)
Pisoniviricetes (3)
Nidovirales (3)
Cornidovirineae (2)
Coronaviridae (2)
Orthocoronavirinae (2)
Betacoronavirus (2)
Nobecovirus
Sarbecovirus
Arnidovirineae
Arteriviridae
Equarterivirinae
Alphaarterivirus
Alphaarterivirus equid
Negarnaviricota
Haploviricotina
Monjiviricetes
Mononegavirales
Filoviridae
Ebolavirus
Pararnavirae (2)
Artverviricota (2)
Revtraviricetes (2)
Ortervirales (2)
Retroviridae (2)
Orthoretrovirinae (2)
Lentivirus (2)
Monodnaviria (2)
Shotokuvirae (2)
Cossaviricota (2)
Papovaviricetes (2)
Sepolyvirales
Polyomaviridae
Betapolyomavirus
Zurhausenvirales
Papillomaviridae
Firstpapillomavirinae
Deltapapillomavirus
Varidnaviria (3)
Bamfordvirae (3)
Preplasmiviricota
Tectiliviricetes
Rowavirales
Adenoviridae
Mastadenovirus
Human mastadenovirus D
Nucleocytoviricota (2)
Megaviricetes
Algavirales
Phycodnaviridae
Chlorovirus
Pokkesviricetes
Chitovirales
Poxviridae
Chordopoxvirinae
Orthopoxvirus
Vaccinia virus
Duplodnaviria (5)
Heunggongvirae (5)
Uroviricota (5)
Caudoviricetes (5)
Caudovirales (5)
Myoviridae (2)
Punavirus
Peduovirinae
Peduovirus
Demerecviridae
Markadamsvirinae
Tequintavirus
Siphoviridae
Lambdavirus
Autographiviridae
Studiervirinae
Kayfunavirus
Escherichia virus K1F

Figure 7: The lineage tree of the viral proteins in the final set of the constrained structural comparisons with S1 C-terminal domain (CTD) of Spike protein monomer (native) and viral dataset. These trees are generated by Machaon's presentation module. Each family name carries also a population number if there are more than one protein categorized under it. The root of the tree starts with thicker branches and the colors designated the branch levels. The tree refers to the viral proteins that were found most structurally relevant. These trees could also be considered as distant evolutionary trees according to the structural traits of the proteins in the results. The lineage information is retrieved from UniProt. (Zoom to review)

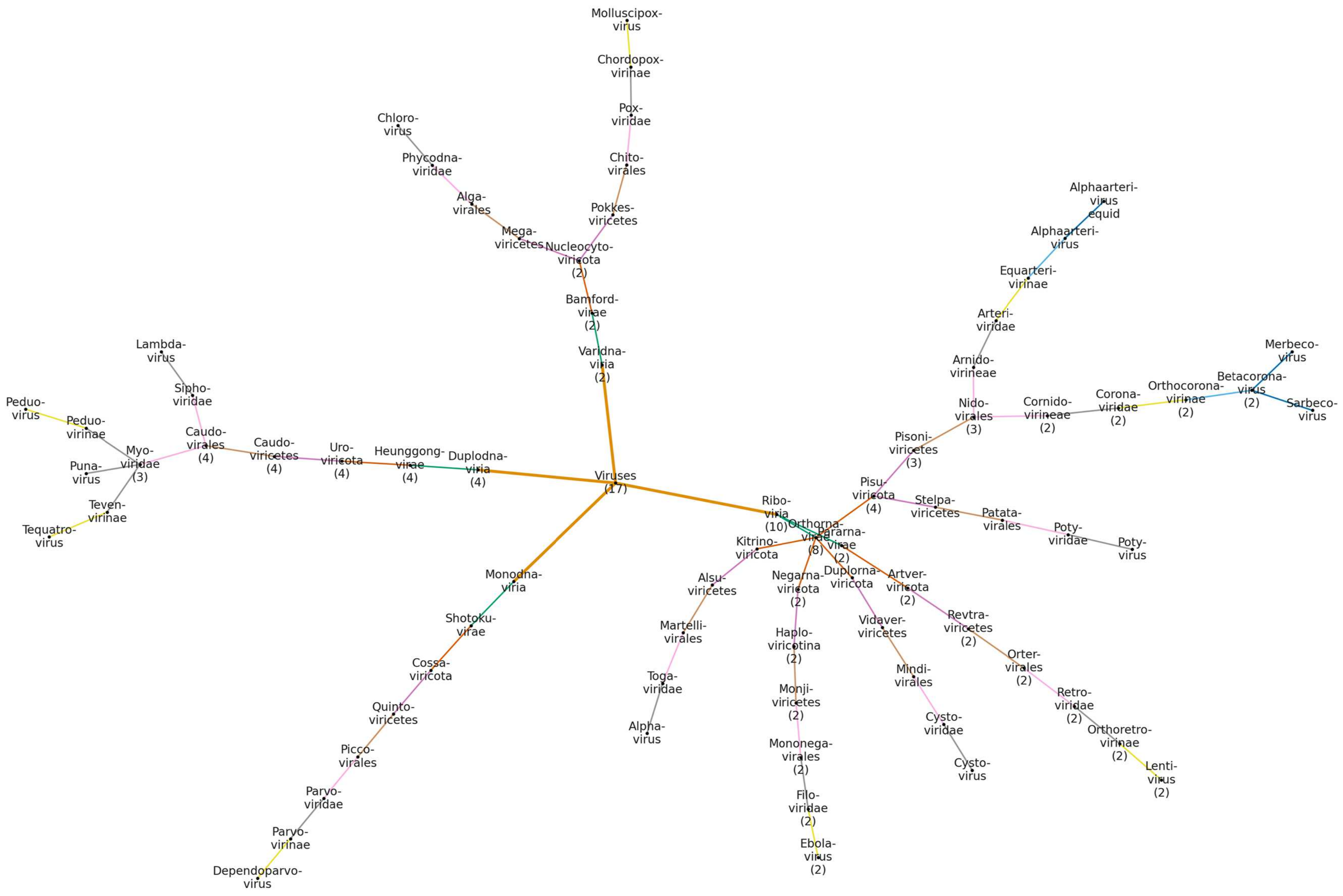
Viruses (17)
Duplodna-viria (4)
Heunggong-virae (4)
Uro-viricota (4)
Caudo-viricetes (4)
Caudo-virales (4)
Myo-viridae (3)
Peduo-virinae
Peduo-virus
Puna-virus
Teven-virinae
Tequatro-virus
Sipho-viridae
Lambda-virus
Varidna-viria (2)
Bamford-virae (2)
Nucleocyto-viricota (2)
Mega-viricetes
Alga-virales
Phycodna-viridae
Chloro-virus
Pokkes-viricetes
Chito-virales
Pox-viridae
Chordopox-virinae
Molluscipox-virus
Monodna-viria
Shotoku-virae
Cossa-viricota
Quinto-viricetes
Picco-virales
Parvo-viridae
Parvo-virinae
Dependoparvo-virus
Ribo-viria (10)
Orthorna-virae (8)
Pararna-virae (2)
Kitrino-viricota
Alsu-viricetes
Martelli-virales
Toga-viridae
Alpha-virus
Negarna-viricota (2)
Haplo-viricotina (2)
Monji-viricetes (2)
Mononega-virales (2)
Filo-viridae (2)
Ebola-virus (2)
Duplorna-viricota
Vidaver-viricetes
Mindi-virales
Cysto-viridae
Cysto-virus
Artver-viricota (2)
Revtra-viricetes (2)
Orter-virales (2)
Retro-viridae (2)
Orthoretro-virinae (2)
Lenti-virus (2)
Pisu-viricota (4)
Stelpa-viricetes
Patata-virales
Poty-viridae
Poty-virus
Pisoni-viricetes (3)
Nido-virales (3)
Arnido-virineae
Arteri-viridae
Equarteri-virinae
Alphaarteri-virus
Alphaarteri-virus equid
Cornido-virineae (2)
Corona-viridae (2)
Orthocorona-virinae (2)
Betacorona-virus (2)
Merbeco-virus
Sarbeco-virus

Figure 8: The lineage tree of the viral proteins in the final set of the constrained structural comparisons with S1 receptor-binding domain (RBD) of Spike protein monomer (native) and viral dataset. These trees are generated by Machaon's presentation module. Each family name carries also a population number if there are more than one protein categorized under it. The root of the tree starts with thicker branches and the colors designated the branch levels. The tree refers to the viral proteins that were found most structurally relevant. These trees could also be considered as distant evolutionary trees according to the structural traits of the proteins in the results. The lineage information is retrieved from UniProt. (Zoom to review)

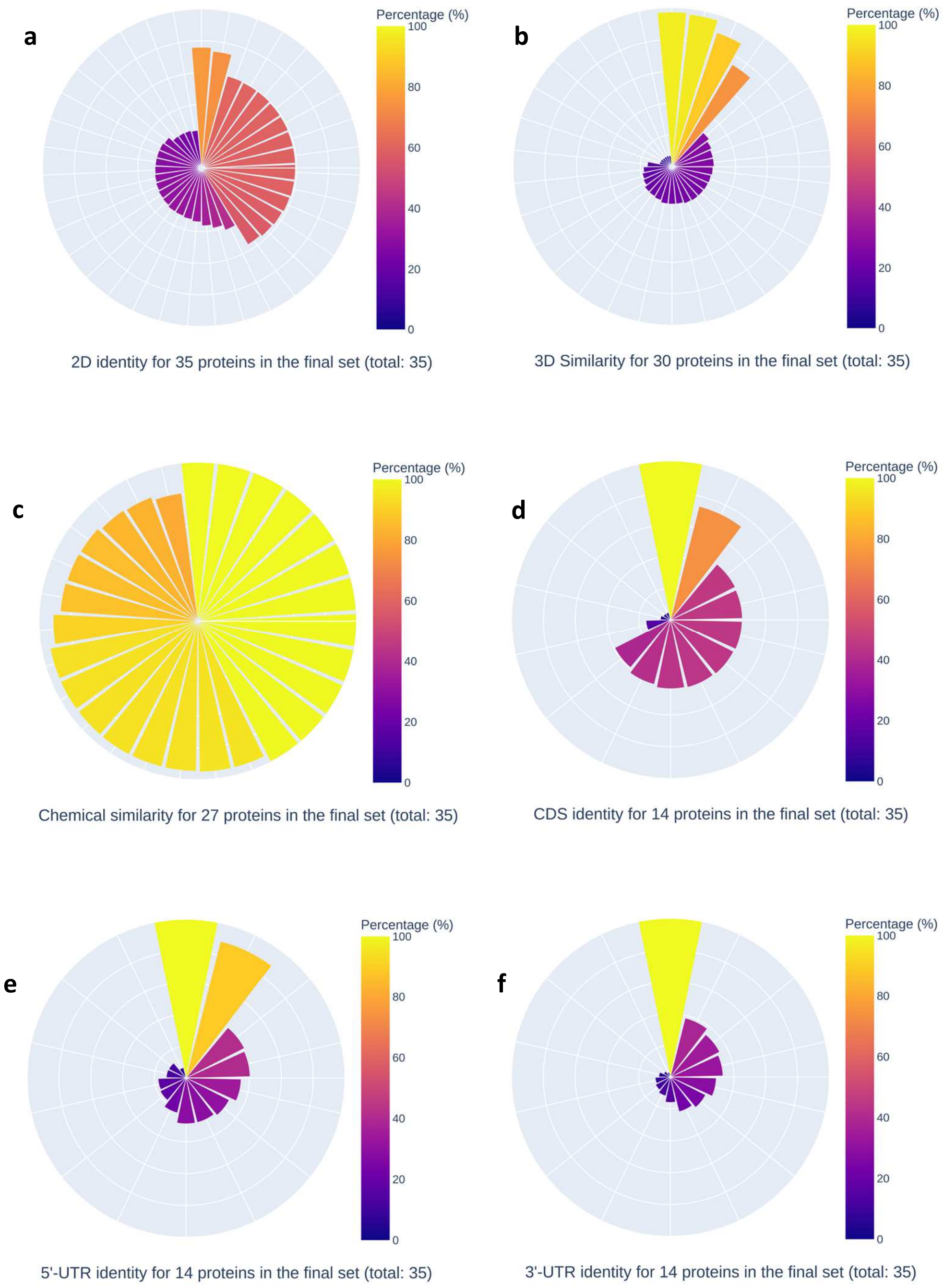

Figure 9: Evaluation of whole structure comparison results with SARS-CoV-2 Spike protein monomer (native) and experimental human dataset. Each protruding bar from the center of each radial plot represents a protein entry from the final set yielded by Machaon. The plots are generated by the presentation module. The measurements were carried out by the evaluation module wherever the underlying data allowed it (missing or malformed relevant data). **a)** Protein 2D folds sequence identities **b)** tertiary structure (3D) similarities (TM-Score) **c)** chemical similarities (Tanimoto Index) **d)** gene coding region identities **e)** 5'-end Untranslated Region identities **f)** 3'-end Untranslated Region sequence identities.

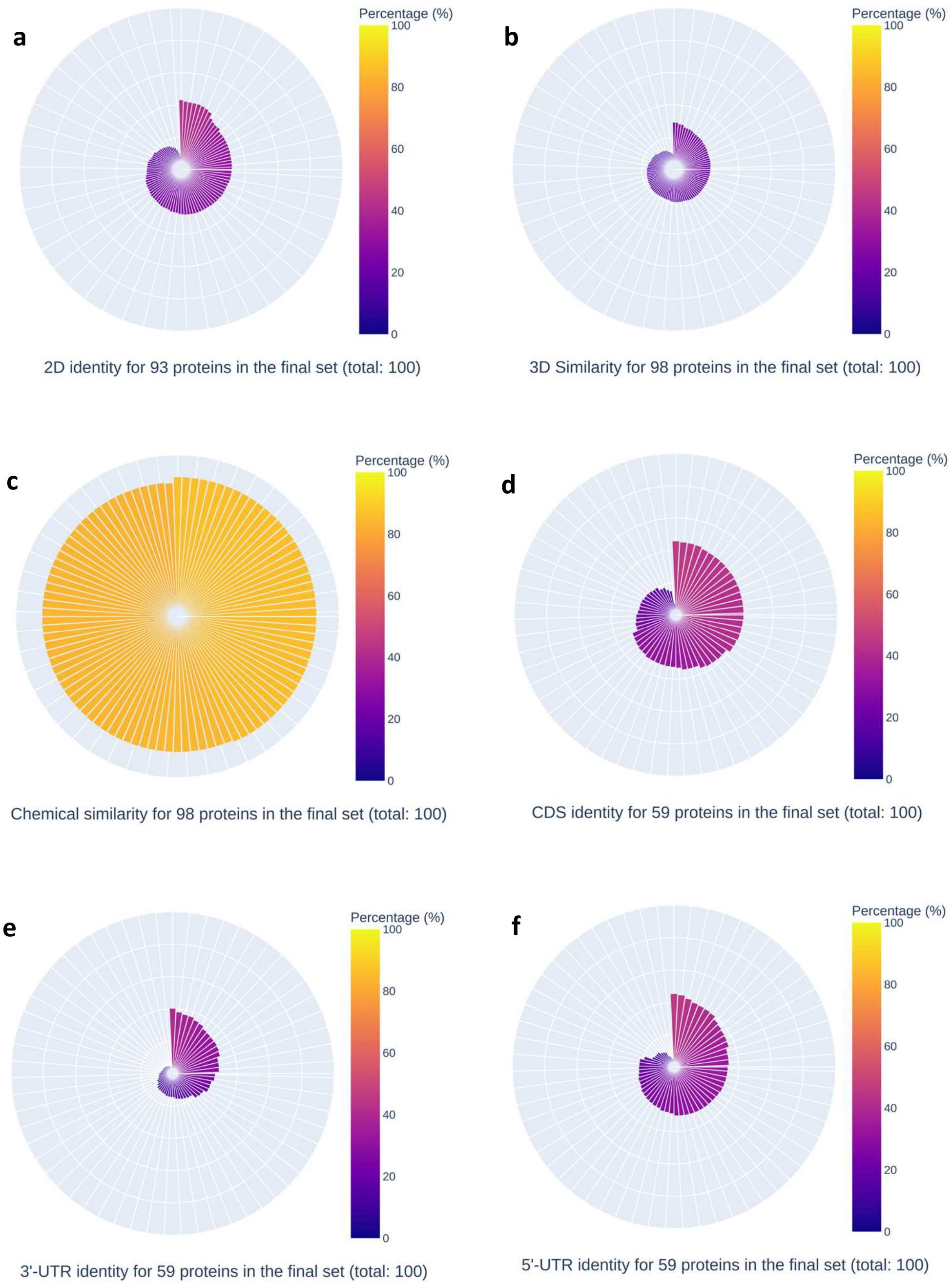

Figure 10: Evaluation of whole structure comparison results with SARS-CoV-2 Spike protein monomer (native) and predicted human dataset by AlphaFold. Each protruding bar from the center of each radial plot represents a protein entry from the final set yielded by Machaon. The plots are generated by the presentation module. The measurements were carried out by the evaluation module wherever the underlying data allowed it (missing or malformed relevant data). **a)** Protein 2D folds sequence identities **b)** tertiary structure (3D) similarities (TM-Score) **c)** chemical similarities (Tanimoto Index) **d)** gene coding region identities **e)** 5'-end Untranslated Region identities **f)** 3'-end Untranslated Region sequence identities.

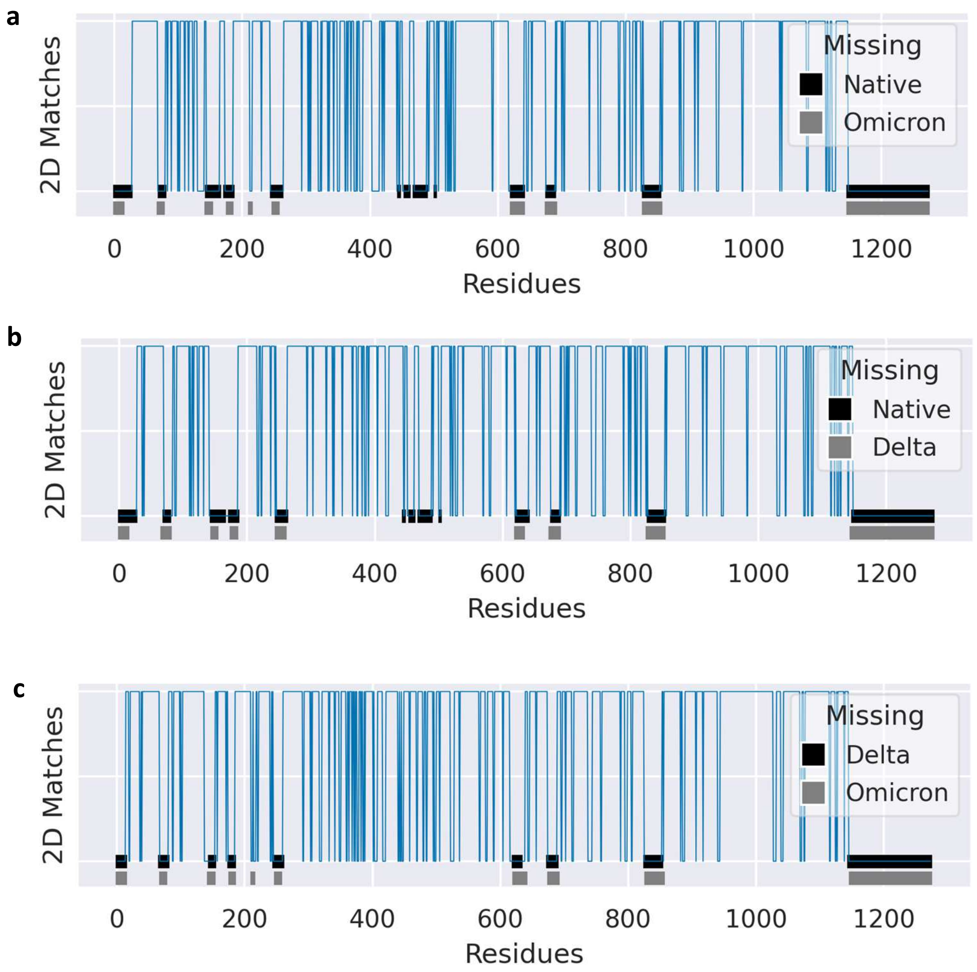

Figure 11: The difference in secondary proteins structures between the variants of SARS-CoV-2 Spike protein (native, Delta, Omicron) and viral dataset. The depicted comparisons are between: **a)** Native and Delta (6VXX.A & 7V7Q.A), **b)** Native and Delta (6VXX.A & 7T9K.A), **c)** Delta and Omicron (7V7Q.A & 7T9K.A). These data were produced by using Machaon's modules programmatically. Areas with missing residues in the reference PDB file are annotated with black and gray boxes on the x-axis.

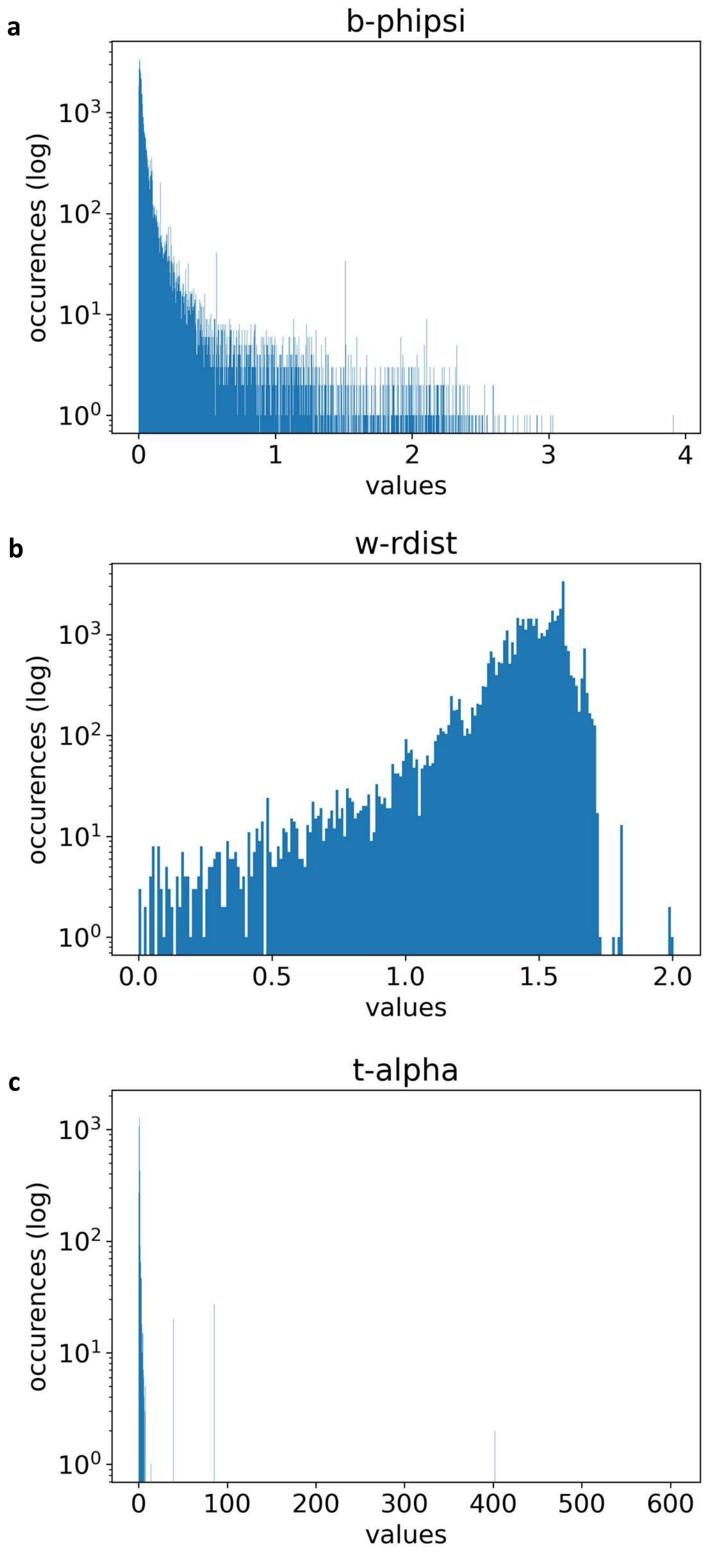

a
b-phipsi
occurences (log)
values
b
w-rdist
occurences (log)
values
c
t-alpha
occurences (log)
values

Figure 12: The numerical space of the metrics as calculated between SARS-CoV-2 Spike protein (native strain) and our study's viral dataset. The histograms include computed metrics on more than 41000 PDB chains and their bins are determined by Freedman-Diaconis rule; **a** plot refers to b-phipsi, **b** to w-rdist and **c** to t-alpha metric. We can notice that the metrics' distributions have peaks localizing near to zero and outliers within a finite small margin.

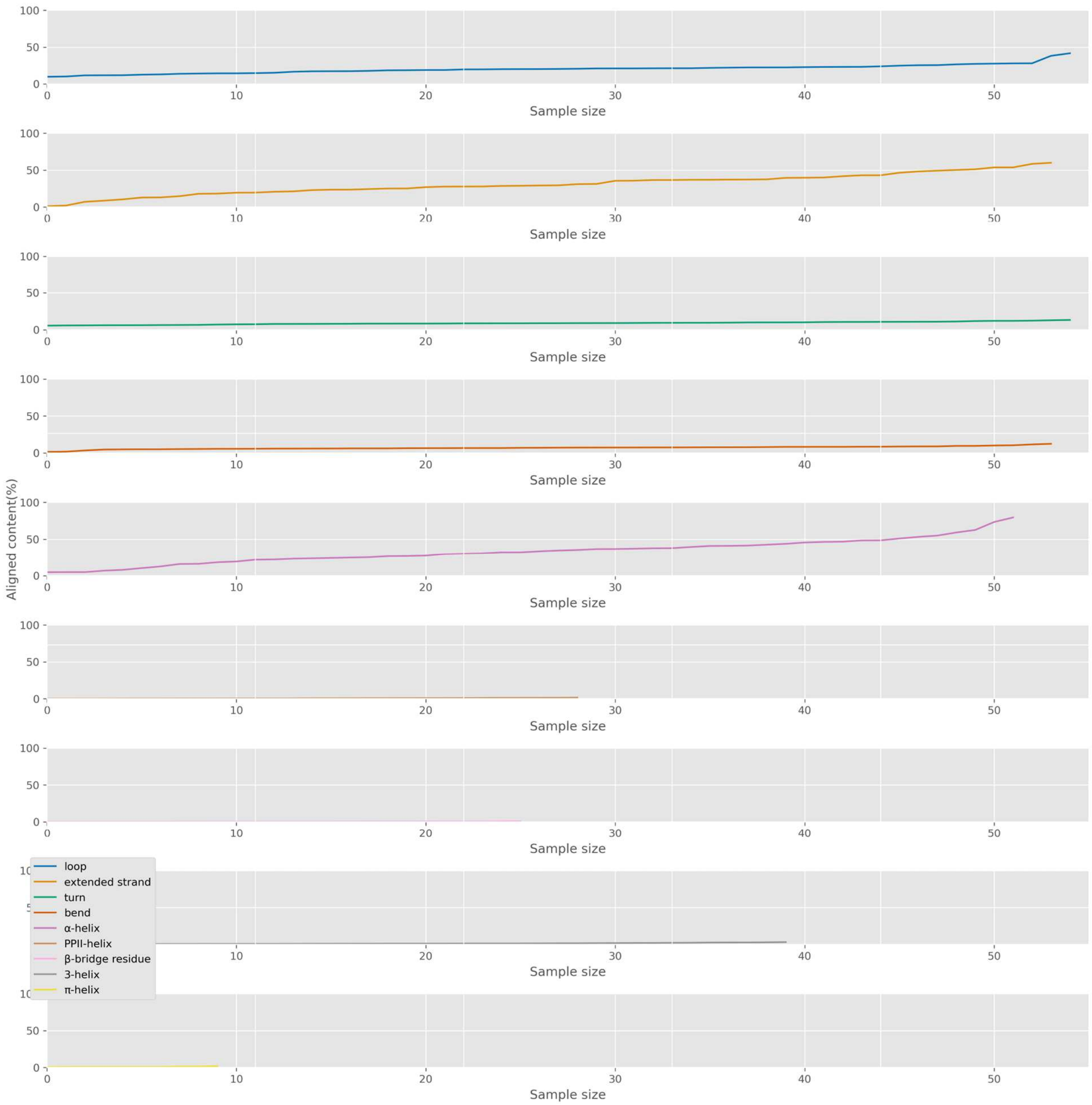

Figure 13: The matched 2D folding types among the proteins in the final set from the comparisons between SARS-CoV-2 Spike protein monomer (native strain) and our study's viral dataset. Each subplot corresponds to a 2D folding type and depicts the number of the proteins (sample size) that have this type matched and in what percentage of their aligned secondary structure.

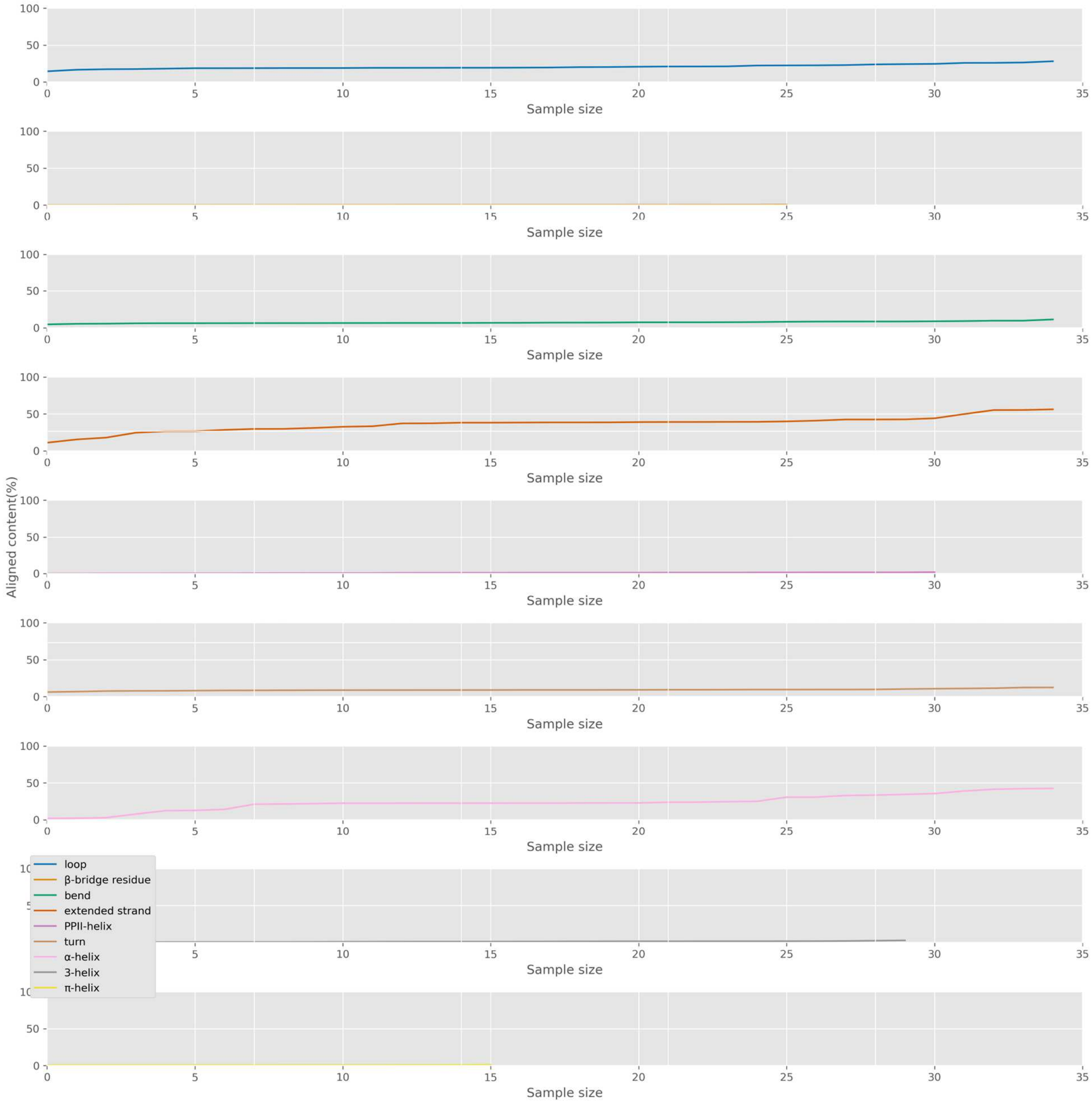

Figure 14: The matched 2D folding types among the proteins in the final set from the comparisons between SARS-CoV-2 Spike protein monomer (native strain) and experimental human dataset. Each subplot corresponds to a 2D folding type and depicts the number of the proteins (sample size) that have this type matched and in what percentage of their aligned secondary structure.

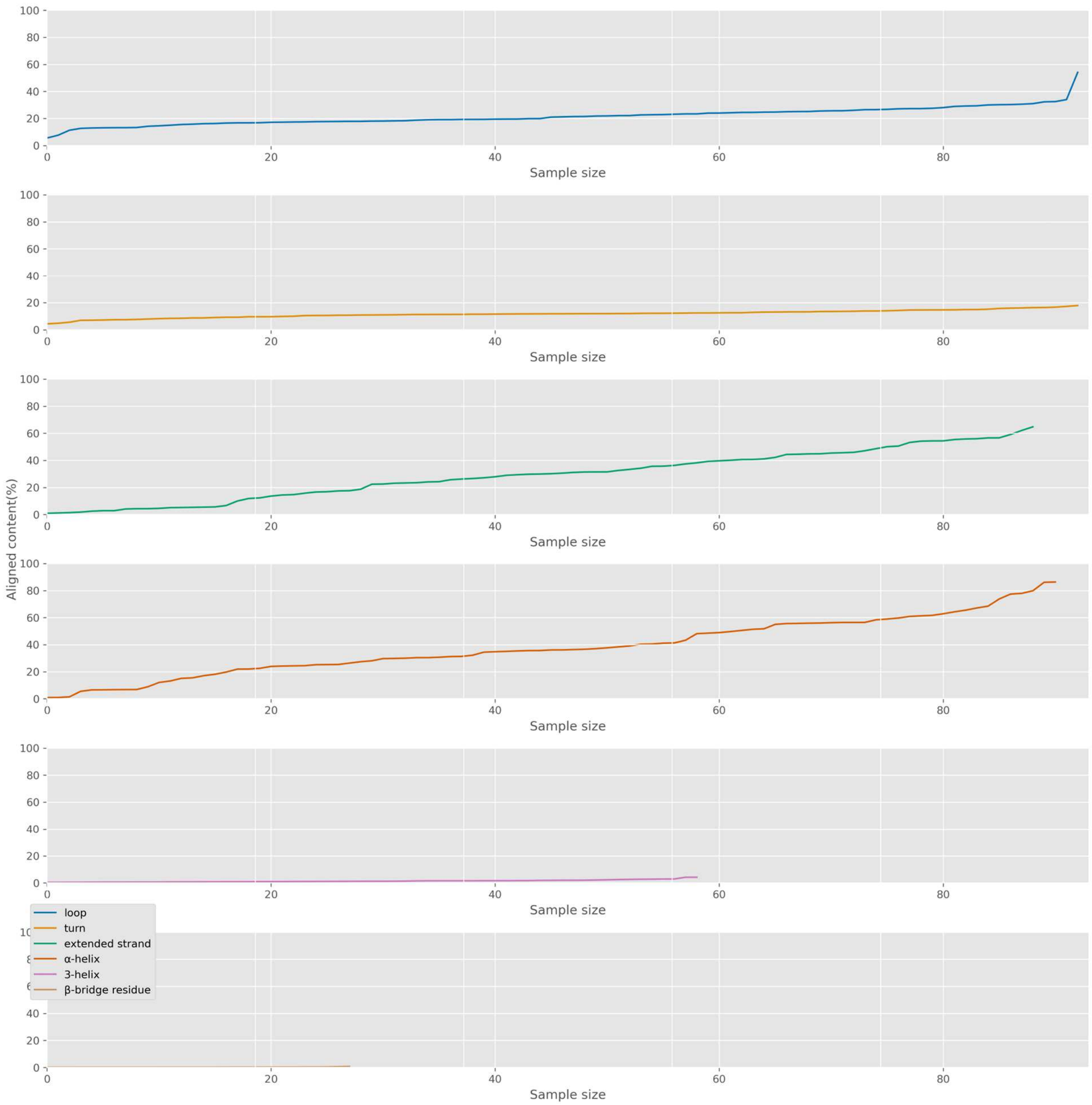

Figure 15: The matched 2D folding types among the proteins in the final set from the comparisons between SARS-CoV-2 Spike protein monomer (native strain) and predicted human dataset by AlphaFold. Each subplot corresponds to a 2D folding type and depicts the number of the proteins (sample size) that have this type matched and in what percentage of their aligned secondary structure.

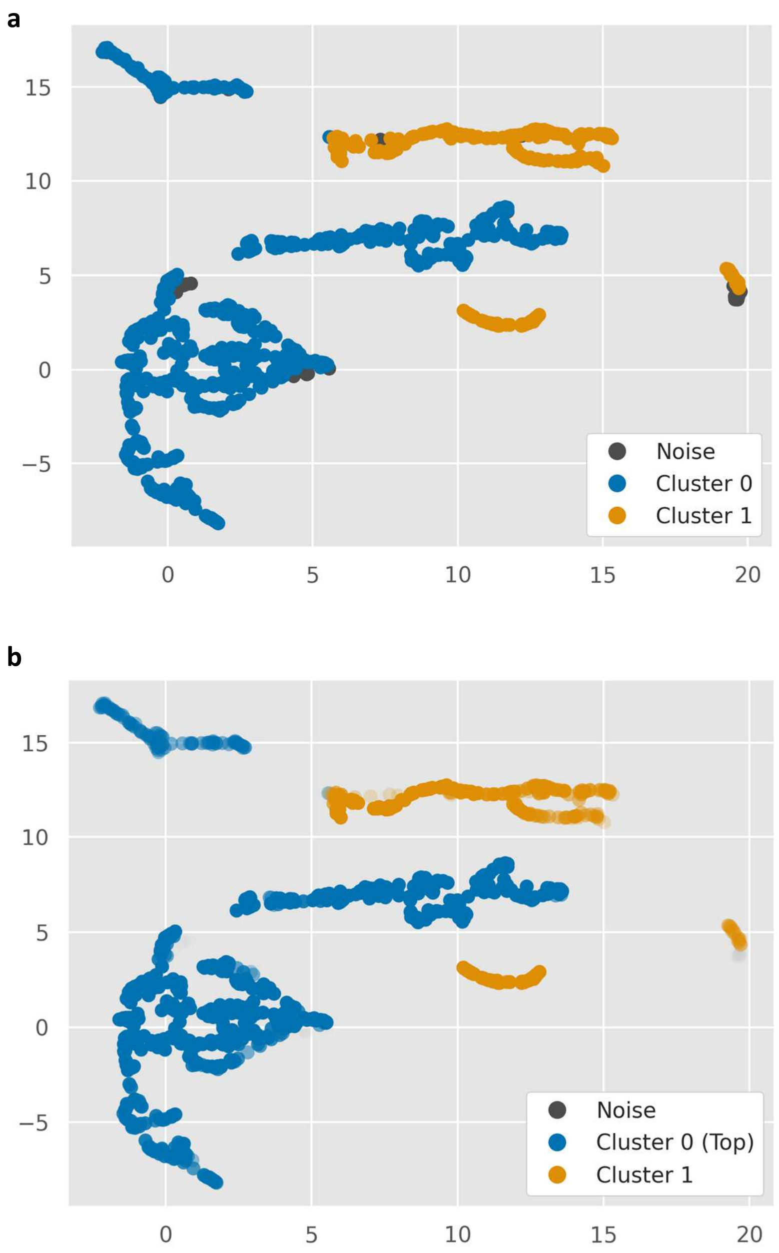

Figure 16: The clustering result on whole structure comparisons with SARS-CoV-2 Spike protein (native) and viral dataset. Each point corresponds to a PDB structure in the dataset, which is represented by a three-dimensional vector (the values of the proposed metrics). Some proteins participate with multiple PDBs in the dataset. The visualization is generated by UMAP method by reducing the dimensions of the data to 2D. **a** depicts the clustering result by HDSCAN and **b** the final selection and form of the top cluster determined by the ranking via b-phipsi, w-rdist and t-alpha. The blue cluster ('Top') is chosen as the preferred one, a selection based on the order of the vectors derived from rank aggregation. The transparency of a point matches its clustering probability. The light greyed data points are interpreted as noise by Hierarchical DBSCAN (not visible in b plot due to their low clustering probability). Also, every point that has clustering probability below 0.1 is discarded from the clusters, labelled as noise. The figure displays the separation of the clusters.

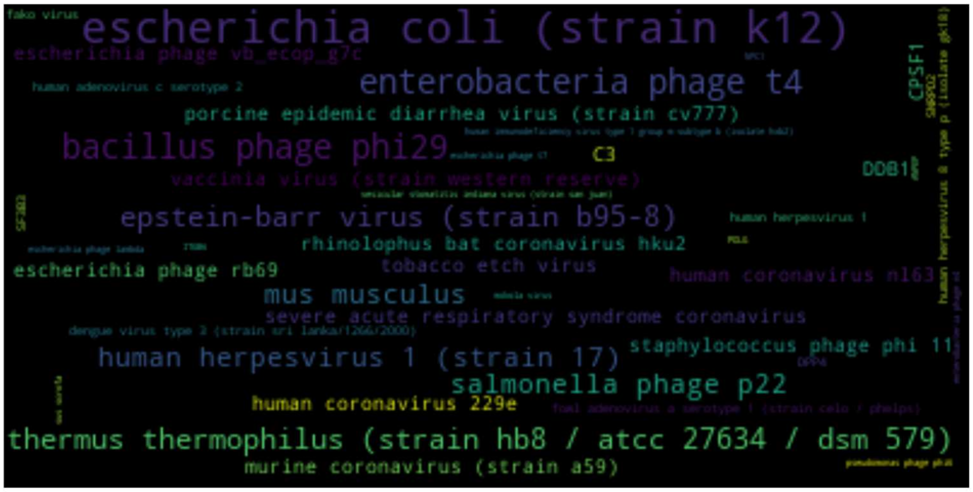

Figure 17: The world cloud visualization of the whole structure comparisons with SARS-CoV-2 Spike protein (native) and viral dataset. The figure is based on the ‘organism’ and ‘gene’ metadata fields of the PDB files containing the data of structurally concordant proteins to the reference (Spike) as determined by Machaon. Non-human proteins are represented by the ‘organism’ metadata and human proteins by the ‘gene’ metadata. The large letter font is correlated with the increased frequency of appearance in the final output set.

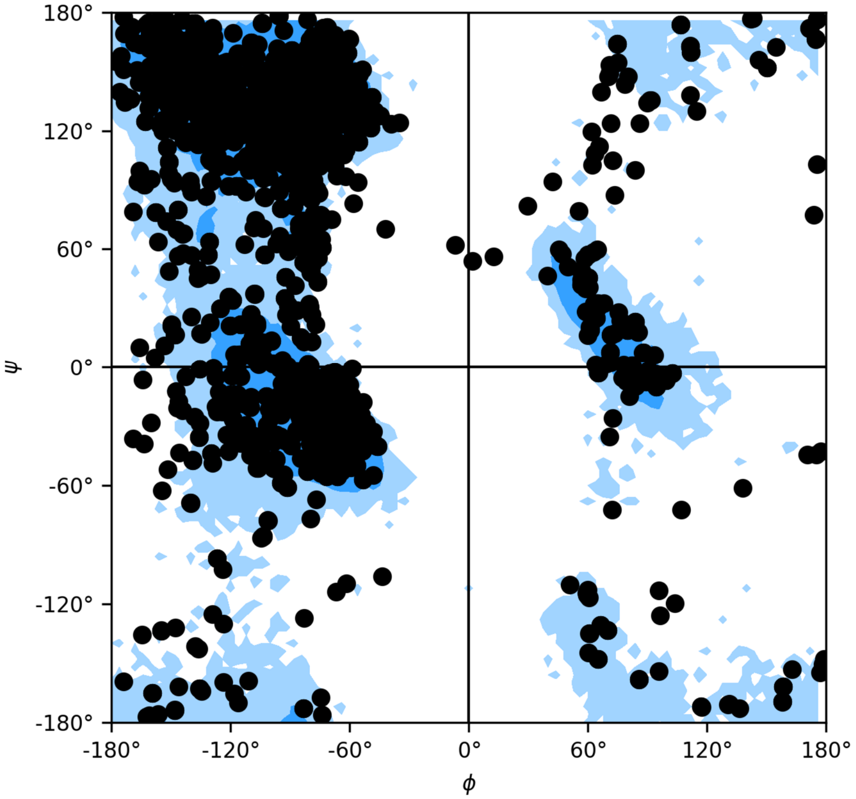

Figure 18: Ramachandran plot for preprocessed PDB data on SARS-CoV-2 Spike protein (native). This plot depicts the backbone phi-psi angles of the residues listed in the preprocessed 6VXX.A PDB file. The colored areas refer to the most favorable spatial arrangements regarding minimum potential energy. The figure was generated with MDAnalysis Python package.

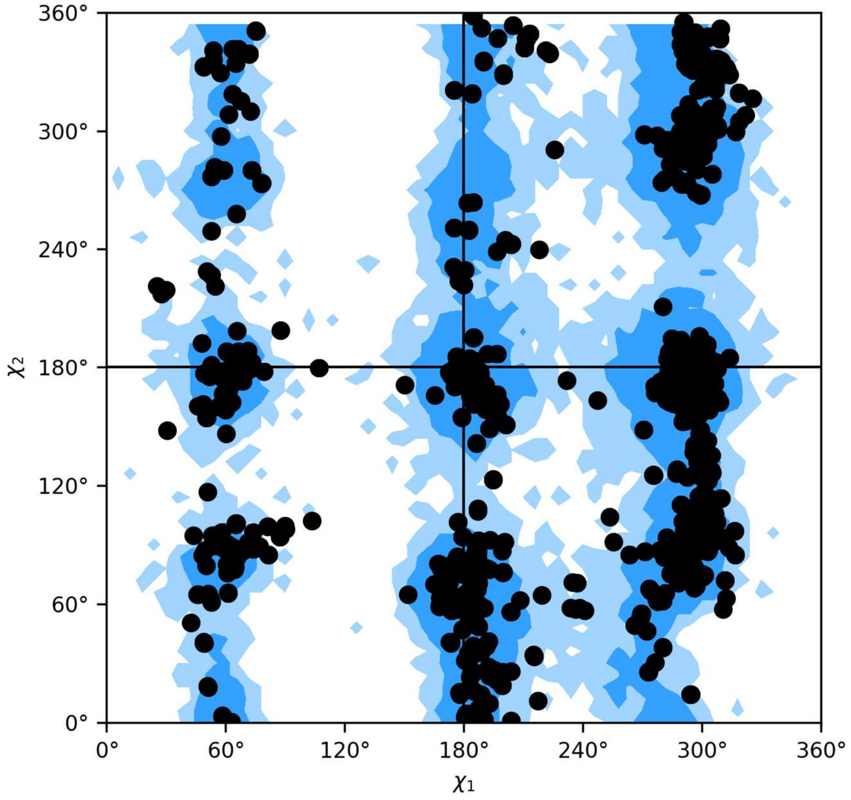

Figure 19: Janin plot for preprocessed PDB data on SARS-CoV-2 Spike protein (native). This plot depicts the side chain chi1-chi2 angles of the residues listed in the preprocessed 6VXX.A PDB file. The colored areas refer to the most favorable spatial arrangements regarding minimum potential energy. The figure was generated with MDAnalysis Python package.
