# Supplementary material for: Identifying and profiling structural similarities between Spike of SARS-CoV-2 and other viral or host proteins with Machaon
Source: Commun Biol. 2023 Jul 19;6:752. doi: 10.1038/s42003-023-05076-7 (PMC10356814; doi:10.1038/s42003-023-05076-7)
Supplement: Supplementary file 3 — Description of Additional Supplementary Files [file 42003_2023_5076_MOESM3_ESM.pdf]

Description of Additional Supplementary Files

File name: Supplementary Data 1
Description: The source data behind the figures in the paper

File name: Supplementary Data 2
Description PDB files of native, Delta and Omicron Spike monomers that contain the intersection of the available residue positions per monomer.

File name: Supplementary Data 3
Description Outputs of whole structure comparisons for viral dataset and native, Delta and Omicron Spike monomers

File name: Supplementary Data 4
Description Outputs of constrained comparisons for viral dataset and native Spike monomer (domain, segment)

File name: Supplementary Data 5
Description Outputs of whole structure comparisons for experimental human dataset and native Spike monomer

File name: Supplementary Data 6
Description Outputs of whole structure comparisons for AlphaFold human dataset and native Spike monomer

File name: Supplementary Data 7
Description Metrics on different PDBs of native Spike monomer

File name: Supplementary Data 8
Description Outputs of whole structure comparisons for viral dataset with raw and preprocessed PDB files of native Spike monomer
