# Supplementary material for: Identifying and profiling structural similarities between Spike of SARS-CoV-2 and other viral or host proteins with Machaon
Source: Commun Biol. 2023 Jul 19;6:752. doi: 10.1038/s42003-023-05076-7 (PMC10356814; doi:10.1038/s42003-023-05076-7)
Supplement: Supplementary file 6 — Supplementary Data 3 [file 42003_2023_5076_MOESM6_ESM.zip › 6VXX_A_whole/candidates/6VXX_A-merged-enriched_eval_report_trunc.html]

 

# Structural Comparison Report for 6VXX\_A - whole structures (total: 58)

---

1

- **Protein name:** Spike glycoprotein
- **Organism:** Severe acute respiratory syndrome coronavirus
- **Uniprot Accession Number:** P59594
- **Protein sequence length:** 1255 aa
- **1D identity (%):** 76.35
- **1D identity (%) [Gaps excluded]:** 77.94
- **1D identity - Alignment Gaps:** 26
- **Common reported functions (%):** 100.0
- **Common reported locations (%):** 62.5
- **Common reported processes (%):** 90.0

- **PDB ID:** 6NB6
- **Chain:** A
- **Crystallized protein length:** 1052 aa
- **Resolution:** 4.2 Å
- **b-phipsi:** 0.001058
- **w-rdist:** 0.5121
- **t-alpha:** 0.002481
- **Chemical similarity (Tanimoto Index) (%):** 94.56
- **1D identity (%) [PDB]:** 69.31
- **1D identity (%) [Gaps excluded][PDB]:** 78.58
- **1D identity - Alignment Gaps [PDB]:** 128
- **2D identity (%) [PDB]:** 72.68
- **2D identity (%) [Gaps excluded][PDB]:** 86.39
- **2D identity - Alignment Gaps [PDB]:** 176
- **3D similarity (TM-Score) (%) [PDB]:** 96.46

- **Gene name:** S
- **RefSeq ID:** NC\_004718
- **Genomic sequence length:** 29751
- **5-UTR|CDS|3-UTR identity (%):** 88.52 | 73.15 | 22.38
- **5-UTR|CDS|3-UTR identity (%) [Gaps excluded]:** 92.28 | 78.79 | 98.18
- **5-UTR|CDS|3-UTR identity [Alignment Gaps]:** 11 | 282 | 745

**Uniprot Description:**  
  
Spike glycoprotein
May down-regulate host tetherin (BST2) by lysosomal degradation, thereby counteracting its antiviral activity.  
  
Homotrimer; each monomer consists of a S1 and a S2 subunit. The resulting peplomers protrude from the virus surface as spikes (By similarity). Binds to human and palm civet ACE2 and human CLEC4M/DC-SIGNR. Interacts with the accessory proteins 3a and 7a.  
  
**Gene Ontology Information:**

Molecular Function

- host cell surface receptor binding
- identical protein binding

Location

- host cell endoplasmic reticulum-Golgi intermediate compartment membrane
- host cell plasma membrane
- integral component of membrane
- viral envelope
- virion membrane

Biological process

- endocytosis involved in viral entry into host cell
- fusion of virus membrane with host endosome membrane
- fusion of virus membrane with host plasma membrane
- pathogenesis
- receptor-mediated virion attachment to host cell
- suppression by virus of host tetherin activity
- suppression by virus of host type I interferon-mediated signaling pathway
- viral protein processing
- viral translation
