# Supplementary material for: Identifying and profiling structural similarities between Spike of SARS-CoV-2 and other viral or host proteins with Machaon
Source: Commun Biol. 2023 Jul 19;6:752. doi: 10.1038/s42003-023-05076-7 (PMC10356814; doi:10.1038/s42003-023-05076-7)
Supplement: Supplementary file 6 — Supplementary Data 3 [file 42003_2023_5076_MOESM6_ESM.zip › 7T9K_A_whole/candidates/7T9K_A-merged-enriched_eval_report.html]

 

# Structural Comparison Report for 7T9K\_A - whole structures (total: 53)

---

1

- **Protein name:** Spike glycoprotein
- **Organism:** Severe acute respiratory syndrome coronavirus
- **Uniprot Accession Number:** P59594
- **Protein sequence length:** 1255 aa
- **1D identity (%):** 76.35
- **1D identity (%) [Gaps excluded]:** 77.94
- **1D identity - Alignment Gaps:** 26
- **Common reported functions (%):** 100.0
- **Common reported locations (%):** 100.0
- **Common reported processes (%):** 85.71

- **PDB ID:** 6NB7
- **Chain:** B
- **Crystallized protein length:** 1032 aa
- **Resolution:** 4.5 Å
- **b-phipsi:** 0.003091
- **w-rdist:** 0.179976
- **t-alpha:** 0.002429
- **Chemical similarity (Tanimoto Index) (%):** 94.5
- **1D identity (%) [PDB]:** 64.61
- **1D identity (%) [Gaps excluded][PDB]:** 77.22
- **1D identity - Alignment Gaps [PDB]:** 185
- **2D identity (%) [PDB]:** 72.71
- **2D identity (%) [Gaps excluded][PDB]:** 83.76
- **2D identity - Alignment Gaps [PDB]:** 147
- **3D similarity (TM-Score) (%) [PDB]:** 90.71

- **Gene name:** S
- **RefSeq ID:** NC\_004718
- **Genomic sequence length:** 29751
- **5-UTR|CDS|3-UTR identity (%):** 88.52 | 73.15 | 22.38
- **5-UTR|CDS|3-UTR identity (%) [Gaps excluded]:** 92.28 | 78.79 | 98.18
- **5-UTR|CDS|3-UTR identity [Alignment Gaps]:** 11 | 282 | 745

**Uniprot Description:**  
  
Spike glycoprotein
May down-regulate host tetherin (BST2) by lysosomal degradation, thereby counteracting its antiviral activity.  
  
Homotrimer; each monomer consists of a S1 and a S2 subunit. The resulting peplomers protrude from the virus surface as spikes (By similarity). Binds to human and palm civet ACE2 and human CLEC4M/DC-SIGNR. Interacts with the accessory proteins 3a and 7a.  
  
**Gene Ontology Information:**

Molecular Function

- host cell surface receptor binding
- identical protein binding

Location

- host cell endoplasmic reticulum-Golgi intermediate compartment membrane
- host cell plasma membrane
- integral component of membrane
- viral envelope
- virion membrane

Biological process

- endocytosis involved in viral entry into host cell
- fusion of virus membrane with host endosome membrane
- fusion of virus membrane with host plasma membrane
- pathogenesis
- receptor-mediated virion attachment to host cell
- suppression by virus of host tetherin activity
- suppression by virus of host type I interferon-mediated signaling pathway
- viral protein processing
- viral translation

---

2

- **Protein name:** Spike glycoprotein
- **Organism:** Human coronavirus 229E
- **Uniprot Accession Number:** P15423
- **Protein sequence length:** 1173 aa
- **1D identity (%):** 26.53
- **1D identity (%) [Gaps excluded]:** 36.78
- **1D identity - Alignment Gaps:** 396
- **Common reported functions (%):** 0.0
- **Common reported locations (%):** 0.0
- **Common reported processes (%):** 0.0

- **PDB ID:** 6IXA
- **Chain:** B
- **Crystallized protein length:** 959 aa
- **Resolution:** 3.21 Å
- **b-phipsi:** 0.000888
- **w-rdist:** 0.856635
- **t-alpha:** 0.004858
- **Chemical similarity (Tanimoto Index) (%):** N/A
- **1D identity (%) [PDB]:** 0.0
- **1D identity (%) [Gaps excluded][PDB]:** 0.0
- **1D identity - Alignment Gaps [PDB]:** 2002
- **2D identity (%) [PDB]:** 56.32
- **2D identity (%) [Gaps excluded][PDB]:** 86.22
- **2D identity - Alignment Gaps [PDB]:** 420
- **3D similarity (TM-Score) (%) [PDB]:** 61.76

- **Gene name:** S
- **RefSeq ID:** NC\_002645
- **Genomic sequence length:** 27317
- **5-UTR|CDS|3-UTR identity (%):** 46.55 | 46.24 | 32.46
- **5-UTR|CDS|3-UTR identity (%) [Gaps excluded]:** 77.51 | 79.68 | 84.38
- **5-UTR|CDS|3-UTR identity [Alignment Gaps]:** 139 | 1950 | 307

**Uniprot Description:**  
  
S1 region attaches the virion to the cell membrane by interacting with host ANPEP/aminopeptidase N, initiating the infection. Binding to the receptor probably induces conformational changes in the S glycoprotein unmasking the fusion peptide of S2 region and activating membranes fusion. S2 region belongs to the class I viral fusion protein. Under the current model, the protein has at least 3 conformational states: pre-fusion native state, pre-hairpin intermediate state, and post-fusion hairpin state. During viral and target cell membrane fusion, the coiled coil regions (heptad repeats) regions assume a trimer-of-hairpins structure, positioning the fusion peptide in close proximity to the C-terminal region of the ectodomain. The formation of this structure appears to drive apposition and subsequent fusion of viral and target cell membranes.  
  
Homotrimer. During virus morphogenesis, found in a complex with M and HE proteins. Interacts with host ANPEP.  
  
**Gene Ontology Information:**

Molecular Function  
  
N/A

Location  
  
N/A

Biological process  
  
N/A

---

3

- **Protein name:** Fibritin
- **Organism:** Enterobacteria phage T4
- **Uniprot Accession Number:** P10104
- **Protein sequence length:** 487 aa
- **1D identity (%):** 7.64
- **1D identity (%) [Gaps excluded]:** 26.13
- **1D identity - Alignment Gaps:** 964
- **Common reported functions (%):** 0.0
- **Common reported locations (%):** 0.0
- **Common reported processes (%):** 0.0

- **PDB ID:** 6JX7
- **Chain:** C
- **Crystallized protein length:** 1245 aa
- **Resolution:** 3.31 Å
- **b-phipsi:** 0.011279
- **w-rdist:** 0.56793
- **t-alpha:** 0.003239
- **Chemical similarity (Tanimoto Index) (%):** 94.27
- **1D identity (%) [PDB]:** 0.0
- **1D identity (%) [Gaps excluded][PDB]:** 0.0
- **1D identity - Alignment Gaps [PDB]:** 2287
- **2D identity (%) [PDB]:** 50.7
- **2D identity (%) [Gaps excluded][PDB]:** 85.55
- **2D identity - Alignment Gaps [PDB]:** 585
- **3D similarity (TM-Score) (%) [PDB]:** 59.17

- **Gene name:** wac
- **RefSeq ID:** NC\_000866
- **Genomic sequence length:** 168903
- **5-UTR|CDS|3-UTR identity (%):** N/A | 27.1 | N/A
- **5-UTR|CDS|3-UTR identity (%) [Gaps excluded]:** N/A | 80.92 | N/A
- **5-UTR|CDS|3-UTR identity [Alignment Gaps]:** N/A | 2634 | N/A

**Uniprot Description:**  
  
Chaperone involved in tail fiber assembly and retraction. Acts as a chaperone helping to attach the long tail fibers to the virus during the assembly process. During phage assembly, twelve fibritin molecules attach to the phage neck via gp13: six molecules forming the collar and six molecules forming the whiskers.  
  
Homotrimer (PubMed:9261070, PubMed:15033360, PubMed:19361528). Interacts (via N-terminal domain) with neck protein gp13; this interaction allows attachment of the fibrous collar and wiskers (PubMed:23434847).  
  
**Gene Ontology Information:**

Molecular Function  
  
N/A

Location

- virion

Biological process  
  
N/A

---

4

- **Protein name:** Complement C3
- **Organism:** Homo sapiens
- **Uniprot Accession Number:** P01024
- **Protein sequence length:** 1663 aa
- **1D identity (%):** 14.7
- **1D identity (%) [Gaps excluded]:** 27.41
- **1D identity - Alignment Gaps:** 886
- **Common reported functions (%):** 0.0
- **Common reported locations (%):** 0.0
- **Common reported processes (%):** 0.0

- **PDB ID:** 5FOB
- **Chain:** B
- **Crystallized protein length:** 902 aa
- **Resolution:** 2.6 Å
- **b-phipsi:** 0.006029
- **w-rdist:** 0.701738
- **t-alpha:** 0.009812
- **Chemical similarity (Tanimoto Index) (%):** 89.02
- **1D identity (%) [PDB]:** 0.0
- **1D identity (%) [Gaps excluded][PDB]:** 0.0
- **1D identity - Alignment Gaps [PDB]:** 1943
- **2D identity (%) [PDB]:** 31.81
- **2D identity (%) [Gaps excluded][PDB]:** 91.8
- **2D identity - Alignment Gaps [PDB]:** 943
- **3D similarity (TM-Score) (%) [PDB]:** 20.35

- **Gene name:** C3
- **RefSeq ID:** NM\_000064
- **Transcript sequence length:** 5231
- **5-UTR|CDS|3-UTR identity (%):** 16.48 | 37.2 | 29.35
- **5-UTR|CDS|3-UTR identity (%) [Gaps excluded]:** 84.91 | 76.72 | 75.44
- **5-UTR|CDS|3-UTR identity [Alignment Gaps]:** 220 | 3058 | 179

**Uniprot Description:**  
  
C3 plays a central role in the activation of the complement system. Its processing by C3 convertase is the central reaction in both classical and alternative complement pathways. After activation C3b can bind covalently, via its reactive thioester, to cell surface carbohydrates or immune aggregates.  
  
C3 precursor is first processed by the removal of 4 Arg residues, forming two chains, beta and alpha, linked by a disulfide bond. C3 convertase activates C3 by cleaving the alpha chain, releasing C3a anaphylatoxin and generating C3b (beta chain + alpha' chain). Forms the pro-C3-convertase enzyme complex by interacting with Complement factor B Bb fragment (Bb), which is then stabilized by binding CFP, allowing the complex to become active (PubMed:28264884, PubMed:31507604). The interaction with Bb is dependent on Mg2+ (PubMed:31507604). C3b interacts with CR1 (via Sushi 8 and Sushi 9 domains) (PubMed:8175757, PubMed:2972794). C3b interacts with CFH (PubMed:21285368). C3d interacts with CFH (PubMed:21285368, PubMed:21317894). C3dg interacts with CR2 (via the N-terminal Sushi domains 1 and 2). During pregnancy, C3dg exists as a complex (probably a 2:2:2 heterohexamer) with AGT and the proform of PRG2. Interacts with VSIG4. Interacts (both C3a and ASP) with C5AR2; the interaction occurs with higher affinity for ASP, enhancing the phosphorylation and activation of C5AR2, recruitment of ARRB2 to the cell surface and endocytosis of GRP77.  
  
**Gene Ontology Information:**

Molecular Function

- C5L2 anaphylatoxin chemotactic receptor binding
- endopeptidase inhibitor activity
- signaling receptor binding

Location

- azurophil granule lumen
- blood microparticle
- cell surface
- endoplasmic reticulum lumen
- extracellular exosome
- extracellular region
- extracellular space
- plasma membrane
- protein-containing complex
- secretory granule lumen

Biological process

- amyloid-beta clearance
- cell surface receptor signaling pathway involved in cell-cell signaling
- cellular protein metabolic process
- complement activation
- complement activation, alternative pathway
- complement activation, classical pathway
- complement-dependent cytotoxicity
- complement-mediated synapse pruning
- fatty acid metabolic process
- G protein-coupled receptor signaling pathway
- immune response
- inflammatory response
- neuron remodeling
- neutrophil degranulation
- oviduct epithelium development
- positive regulation of activation of membrane attack complex
- positive regulation of angiogenesis
- positive regulation of apoptotic cell clearance
- positive regulation of G protein-coupled receptor signaling pathway
- positive regulation of glucose transmembrane transport
- positive regulation of lipid storage
- positive regulation of phagocytosis, engulfment
- positive regulation of protein phosphorylation
- positive regulation of receptor-mediated endocytosis
- positive regulation of type IIa hypersensitivity
- positive regulation of vascular endothelial growth factor production
- post-translational protein modification
- purinergic nucleotide receptor signaling pathway
- regulation of complement activation
- regulation of immune response
- regulation of triglyceride biosynthetic process
- response to bacterium
- signal transduction
- vertebrate eye-specific patterning

---

5

- **Protein name:** Spike glycoprotein
- **Organism:** Porcine epidemic diarrhea virus (strain CV777)
- **Uniprot Accession Number:** Q91AV1
- **Protein sequence length:** 1383 aa
- **1D identity (%):** 22.74
- **1D identity (%) [Gaps excluded]:** 34.02
- **1D identity - Alignment Gaps:** 528
- **Common reported functions (%):** 0.0
- **Common reported locations (%):** 80.0
- **Common reported processes (%):** 57.14

- **PDB ID:** 6U7K
- **Chain:** A
- **Crystallized protein length:** 1064 aa
- **Resolution:** 3.14 Å
- **b-phipsi:** 0.000643
- **w-rdist:** 0.909262
- **t-alpha:** 0.012295
- **Chemical similarity (Tanimoto Index) (%):** N/A
- **1D identity (%) [PDB]:** 0.43
- **1D identity (%) [Gaps excluded][PDB]:** 64.29
- **1D identity - Alignment Gaps [PDB]:** 2082
- **2D identity (%) [PDB]:** 51.39
- **2D identity (%) [Gaps excluded][PDB]:** 88.16
- **2D identity - Alignment Gaps [PDB]:** 556
- **3D similarity (TM-Score) (%) [PDB]:** 61.73

- **Gene name:** S
- **RefSeq ID:** NC\_003436
- **Genomic sequence length:** 28033
- **5-UTR|CDS|3-UTR identity (%):** N/A | 45.12 | N/A
- **5-UTR|CDS|3-UTR identity (%) [Gaps excluded]:** N/A | 78.14 | N/A
- **5-UTR|CDS|3-UTR identity [Alignment Gaps]:** N/A | 2136 | N/A

**Uniprot Description:**  
  
S1 region attaches the virion to the cell membrane by interacting with host ANPEP/aminopeptidase N, initiating the infection. Binding to the receptor probably induces conformational changes in the S glycoprotein unmasking the fusion peptide of S2 region and activating membranes fusion. S2 region belongs to the class I viral fusion protein. Under the current model, the protein has at least 3 conformational states: pre-fusion native state, pre-hairpin intermediate state, and post-fusion hairpin state. During viral and target cell membrane fusion, the coiled coil regions (heptad repeats) regions assume a trimer-of-hairpins structure, positioning the fusion peptide in close proximity to the C-terminal region of the ectodomain. The formation of this structure appears to drive apposition and subsequent fusion of viral and target cell membranes.  
  
Homotrimer. During virus morphogenesis, found in a complex with M and HE proteins. Interacts with host ANPEP.  
  
**Gene Ontology Information:**

Molecular Function  
  
N/A

Location

- host cell endoplasmic reticulum-Golgi intermediate compartment membrane
- integral component of membrane
- viral envelope
- virion membrane

Biological process

- endocytosis involved in viral entry into host cell
- fusion of virus membrane with host endosome membrane
- fusion of virus membrane with host plasma membrane
- pathogenesis
- receptor-mediated virion attachment to host cell

---

6

- **Protein name:** Spike glycoprotein
- **Organism:** Human coronavirus NL63
- **Uniprot Accession Number:** Q6Q1S2
- **Protein sequence length:** 1356 aa
- **1D identity (%):** 23.33
- **1D identity (%) [Gaps excluded]:** 35.71
- **1D identity - Alignment Gaps:** 551
- **Common reported functions (%):** 50.0
- **Common reported locations (%):** 80.0
- **Common reported processes (%):** 57.14

- **PDB ID:** 5SZS
- **Chain:** A
- **Crystallized protein length:** 1177 aa
- **Resolution:** 3.4 Å
- **b-phipsi:** 0.001712
- **w-rdist:** 0.877031
- **t-alpha:** 0.012955
- **Chemical similarity (Tanimoto Index) (%):** N/A
- **1D identity (%) [PDB]:** 0.0
- **1D identity (%) [Gaps excluded][PDB]:** 0.0
- **1D identity - Alignment Gaps [PDB]:** 2220
- **2D identity (%) [PDB]:** 50.75
- **2D identity (%) [Gaps excluded][PDB]:** 86.15
- **2D identity - Alignment Gaps [PDB]:** 574
- **3D similarity (TM-Score) (%) [PDB]:** 65.13

- **Gene name:** S
- **RefSeq ID:** NC\_005831
- **Genomic sequence length:** 27553
- **5-UTR|CDS|3-UTR identity (%):** 46.29 | 46.82 | 37.18
- **5-UTR|CDS|3-UTR identity (%) [Gaps excluded]:** 80.6 | 78.15 | 81.99
- **5-UTR|CDS|3-UTR identity [Alignment Gaps]:** 149 | 1979 | 194

**Uniprot Description:**  
  
S1 region attaches the virion to the cell membrane by interacting with host ANPEP/aminopeptidase N, initiating the infection. Binding to the receptor probably induces conformational changes in the S glycoprotein unmasking the fusion peptide of S2 region and activating membranes fusion. S2 region belongs to the class I viral fusion protein. Under the current model, the protein has at least 3 conformational states: pre-fusion native state, pre-hairpin intermediate state, and post-fusion hairpin state. During viral and target cell membrane fusion, the coiled coil regions (heptad repeats) regions assume a trimer-of-hairpins structure, positioning the fusion peptide in close proximity to the C-terminal region of the ectodomain. The formation of this structure appears to drive apposition and subsequent fusion of viral and target cell membranes.  
  
Homotrimer. During virus morphogenesis, found in a complex with M and HE proteins. Interacts with host ANPEP.  
  
**Gene Ontology Information:**

Molecular Function

- host cell surface receptor binding

Location

- host cell endoplasmic reticulum-Golgi intermediate compartment membrane
- integral component of membrane
- viral envelope
- virion membrane

Biological process

- endocytosis involved in viral entry into host cell
- fusion of virus membrane with host endosome membrane
- fusion of virus membrane with host plasma membrane
- pathogenesis
- receptor-mediated virion attachment to host cell

---

7

- **Protein name:** Spike glycoprotein
- **Organism:** Murine coronavirus (strain A59)
- **Uniprot Accession Number:** P11224
- **Protein sequence length:** 1324 aa
- **1D identity (%):** 30.78
- **1D identity (%) [Gaps excluded]:** 38.07
- **1D identity - Alignment Gaps:** 275
- **Common reported functions (%):** 50.0
- **Common reported locations (%):** 100.0
- **Common reported processes (%):** 57.14

- **PDB ID:** 6VSJ
- **Chain:** A
- **Crystallized protein length:** 1122 aa
- **Resolution:** 3.94 Å
- **b-phipsi:** 0.006332
- **w-rdist:** 0.685803
- **t-alpha:** 0.013126
- **Chemical similarity (Tanimoto Index) (%):** 100.0
- **1D identity (%) [PDB]:** 0.0
- **1D identity (%) [Gaps excluded][PDB]:** 0.0
- **1D identity - Alignment Gaps [PDB]:** 2164
- **2D identity (%) [PDB]:** 48.55
- **2D identity (%) [Gaps excluded][PDB]:** 85.11
- **2D identity - Alignment Gaps [PDB]:** 592
- **3D similarity (TM-Score) (%) [PDB]:** 72.27

- **Gene name:** S
- **RefSeq ID:** NC\_001846
- **Genomic sequence length:** 31357
- **5-UTR|CDS|3-UTR identity (%):** 43.0 | 48.17 | 38.17
- **5-UTR|CDS|3-UTR identity (%) [Gaps excluded]:** 79.04 | 76.7 | 77.6
- **5-UTR|CDS|3-UTR identity [Alignment Gaps]:** 140 | 1781 | 189

**Uniprot Description:**  
  
Spike protein S1
attaches the virion to the cell membrane by interacting with host receptor, initiating the infection. Interacts with murine CEACAM1 to mediate viral entry.  
  
Homotrimer; each monomer consists of a S1 and a S2 subunit. The resulting peplomers protrude from the virus surface as spikes (By similarity). Cytoplasmic tail interacts with M protein. S1 interacts with murine CEACAM1, and weakly with murine CEACAM2 in tissue culture.  
  
**Gene Ontology Information:**

Molecular Function

- identical protein binding

Location

- host cell endoplasmic reticulum-Golgi intermediate compartment membrane
- host cell Golgi apparatus
- host cell plasma membrane
- integral component of membrane
- viral envelope
- virion membrane

Biological process

- endocytosis involved in viral entry into host cell
- fusion of virus membrane with host endosome membrane
- fusion of virus membrane with host plasma membrane
- pathogenesis
- receptor-mediated virion attachment to host cell

---

8

- **Protein name:** Major capsid protein
- **Organism:** Epstein-Barr virus (strain B95-8)
- **Uniprot Accession Number:** P03226
- **Protein sequence length:** 1381 aa
- **1D identity (%):** 6.12
- **1D identity (%) [Gaps excluded]:** 25.49
- **1D identity - Alignment Gaps:** 1626
- **Common reported functions (%):** 0.0
- **Common reported locations (%):** 0.0
- **Common reported processes (%):** 0.0

- **PDB ID:** 7BR8
- **Chain:** W
- **Crystallized protein length:** 1331 aa
- **Resolution:** 3.8 Å
- **b-phipsi:** 0.0233
- **w-rdist:** 0.770442
- **t-alpha:** 0.00081
- **Chemical similarity (Tanimoto Index) (%):** N/A
- **1D identity (%) [PDB]:** 0.0
- **1D identity (%) [Gaps excluded][PDB]:** 0.0
- **1D identity - Alignment Gaps [PDB]:** 2375
- **2D identity (%) [PDB]:** 30.55
- **2D identity (%) [Gaps excluded][PDB]:** 92.22
- **2D identity - Alignment Gaps [PDB]:** 1193
- **3D similarity (TM-Score) (%) [PDB]:** 4.85

- **Gene name:** MCP
- **RefSeq ID:** NC\_007605
- **Genomic sequence length:** 171823
- **5-UTR|CDS|3-UTR identity (%):** N/A | 38.94 | N/A
- **5-UTR|CDS|3-UTR identity (%) [Gaps excluded]:** N/A | 76.61 | N/A
- **5-UTR|CDS|3-UTR identity [Alignment Gaps]:** N/A | 2598 | N/A

**Uniprot Description:**  
  
Self-assembles to form an icosahedral capsid with a T=16 symmetry, about 200 nm in diameter, and consisting of 150 hexons and 12 pentons (total of 162 capsomers). Hexons form the edges and faces of the capsid and are each composed of six MCP molecules. In contrast, one penton is found at each of the 12 vertices. Eleven of the pentons are MCP pentamers, while the last vertex is occupied by the portal complex. The capsid is surrounded by a layer of proteinaceous material designated the tegument which, in turn, is enclosed in an envelope of host cell-derived lipids containing virus-encoded glycoproteins.  
  
Homomultimer. Makes the hexons and eleven out of twelve pentons. Interacts with triplex proteins 1/TRX1 and 2/TRX2; adjacent capsomers are linked together in groups of three by triplexes, heterotrimeric complexes composed of one molecule of TRX1 and two molecules of TRX2. Interacts with scaffold protein; this interaction allows efficient MCP transport to the host nucleus. Interacts with capsid vertex component 2/CVC2. Interacts with the small capsomere-interacting protein/SCP.  
  
**Gene Ontology Information:**

Molecular Function

- structural molecule activity

Location

- host cell nucleus
- T=16 icosahedral viral capsid

Biological process  
  
N/A

---

9

- **Protein name:** Envelope glycoprotein B
- **Organism:** Human herpesvirus 1 (strain 17)
- **Uniprot Accession Number:** P10211
- **Protein sequence length:** 904 aa
- **1D identity (%):** 9.99
- **1D identity (%) [Gaps excluded]:** 27.78
- **1D identity - Alignment Gaps:** 1025
- **Common reported functions (%):** 0.0
- **Common reported locations (%):** 80.0
- **Common reported processes (%):** 14.29

- **PDB ID:** 6BM8
- **Chain:** A
- **Crystallized protein length:** 665 aa
- **Resolution:** 4.1 Å
- **b-phipsi:** 0.002902
- **w-rdist:** 0.936174
- **t-alpha:** 0.017814
- **Chemical similarity (Tanimoto Index) (%):** 94.73
- **1D identity (%) [PDB]:** 0.0
- **1D identity (%) [Gaps excluded][PDB]:** 0.0
- **1D identity - Alignment Gaps [PDB]:** 1707
- **2D identity (%) [PDB]:** 31.5
- **2D identity (%) [Gaps excluded][PDB]:** 87.2
- **2D identity - Alignment Gaps [PDB]:** 801
- **3D similarity (TM-Score) (%) [PDB]:** 15.52

- **Gene name:** gB
- **RefSeq ID:** NC\_001806
- **Genomic sequence length:** 152222
- **5-UTR|CDS|3-UTR identity (%):** N/A | 32.33 | N/A
- **5-UTR|CDS|3-UTR identity (%) [Gaps excluded]:** N/A | 74.19 | N/A
- **5-UTR|CDS|3-UTR identity [Alignment Gaps]:** N/A | 2569 | N/A

**Uniprot Description:**  
  
Envelope glycoprotein that forms spikes at the surface of virion envelope. Essential for the initial attachment to heparan sulfate moieties of the host cell surface proteoglycans. Involved in fusion of viral and cellular membranes leading to virus entry into the host cell. Following initial binding to its host receptors, membrane fusion is mediated by the fusion machinery composed at least of gB and the heterodimer gH/gL. May be involved in the fusion between the virion envelope and the outer nuclear membrane during virion egress (By similarity). Also plays a role, together with gK, in virus-induced cell-to-cell fusion (syncytia formation).  
  
Homotrimer; disulfide-linked. Binds to heparan sulfate proteoglycans. Interacts with gH/gL heterodimer.  
  
**Gene Ontology Information:**

Molecular Function  
  
N/A

Location

- host cell endosome membrane
- host cell Golgi membrane
- host cell plasma membrane
- integral component of membrane
- viral envelope
- virion membrane

Biological process

- viral entry into host cell
- virion attachment to host cell

---

10

- **Protein name:** DNA-directed RNA polymerase subunit beta
- **Organism:** Thermus thermophilus (strain HB8 / ATCC 27634 / DSM 579)
- **Uniprot Accession Number:** Q8RQE9
- **Protein sequence length:** 1119 aa
- **1D identity (%):** 13.58
- **1D identity (%) [Gaps excluded]:** 26.58
- **1D identity - Alignment Gaps:** 774
- **Common reported functions (%):** 0.0
- **Common reported locations (%):** 0.0
- **Common reported processes (%):** 0.0

- **PDB ID:** 5XJ0
- **Chain:** C
- **Crystallized protein length:** 1114 aa
- **Resolution:** 4.0 Å
- **b-phipsi:** 0.00534
- **w-rdist:** 1.145668
- **t-alpha:** 0.004882
- **Chemical similarity (Tanimoto Index) (%):** 83.83
- **1D identity (%) [PDB]:** 0.0
- **1D identity (%) [Gaps excluded][PDB]:** 0.0
- **1D identity - Alignment Gaps [PDB]:** 2154
- **2D identity (%) [PDB]:** 32.29
- **2D identity (%) [Gaps excluded][PDB]:** 87.44
- **2D identity - Alignment Gaps [PDB]:** 992
- **3D similarity (TM-Score) (%) [PDB]:** 20.98

- **Gene name:** rpoB
- **RefSeq ID:** N/A
- **Sequence length:** N/A
- **5-UTR|CDS|3-UTR identity (%):** N/A | N/A | N/A
- **5-UTR|CDS|3-UTR identity (%) [Gaps excluded]:** N/A | N/A | N/A
- **5-UTR|CDS|3-UTR identity [Alignment Gaps]:** N/A | N/A | N/A

**Uniprot Description:**  
  
DNA-dependent RNA polymerase catalyzes the transcription of DNA into RNA using the four ribonucleoside triphosphates as substrates.  
  
The RNAP catalytic core consists of 2 alpha, 1 beta, 1 beta' and 1 omega subunit. When a sigma factor is associated with the core the holoenzyme is formed, which can initiate transcription.  
  
**Gene Ontology Information:**

Molecular Function

- DNA binding
- DNA-directed 5'-3' RNA polymerase activity
- ribonucleoside binding

Location  
  
N/A

Biological process

- transcription, DNA-templated

---

11

- **Protein name:** Genome polyprotein
- **Organism:** Tobacco etch virus
- **Uniprot Accession Number:** P04517
- **Protein sequence length:** 3054 aa
- **1D identity (%):** 8.63
- **1D identity (%) [Gaps excluded]:** 27.83
- **1D identity - Alignment Gaps:** 2279
- **Common reported functions (%):** 0.0
- **Common reported locations (%):** 0.0
- **Common reported processes (%):** 0.0

- **PDB ID:** 6SUQ
- **Chain:** A
- **Crystallized protein length:** 2134 aa
- **Resolution:** 3.7 Å
- **b-phipsi:** 0.005664
- **w-rdist:** 0.827428
- **t-alpha:** 0.072874
- **Chemical similarity (Tanimoto Index) (%):** 85.75
- **1D identity (%) [PDB]:** 0.03
- **1D identity (%) [Gaps excluded][PDB]:** 100.0
- **1D identity - Alignment Gaps [PDB]:** 3174
- **2D identity (%) [PDB]:** N/A
- **2D identity (%) [Gaps excluded][PDB]:** N/A
- **2D identity - Alignment Gaps [PDB]:** N/A
- **3D similarity (TM-Score) (%) [PDB]:** 27.2

- **Gene name:** N/A
- **RefSeq ID:** NC\_001555
- **Genomic sequence length:** 9494
- **5-UTR|CDS|3-UTR identity (%):** N/A | 28.55 | N/A
- **5-UTR|CDS|3-UTR identity (%) [Gaps excluded]:** N/A | 81.18 | N/A
- **5-UTR|CDS|3-UTR identity [Alignment Gaps]:** N/A | 6229 | N/A

**Uniprot Description:**  
  
Capsid protein
involved in aphid transmission, cell-to-cell and systemis movement, encapsidation of the viral RNA and in the regulation of viral RNA amplification.  
  
Nuclear inclusion protein A protease is a dimer; disulfide-linked.  
  
**Gene Ontology Information:**

Molecular Function

- ATP binding
- cysteine-type endopeptidase activity
- helicase activity
- hydrolase activity, acting on acid anhydrides, in phosphorus-containing anhydrides
- RNA binding
- RNA-directed 5'-3' RNA polymerase activity
- serine-type peptidase activity
- structural molecule activity

Location

- helical viral capsid

Biological process

- RNA-protein covalent cross-linking
- transcription, DNA-templated
- viral RNA genome replication

---

12

- **Protein name:** Envelope glycoprotein B
- **Organism:** Epstein-Barr virus (strain B95-8)
- **Uniprot Accession Number:** P03188
- **Protein sequence length:** 857 aa
- **1D identity (%):** 13.35
- **1D identity (%) [Gaps excluded]:** 28.66
- **1D identity - Alignment Gaps:** 776
- **Common reported functions (%):** 0.0
- **Common reported locations (%):** 80.0
- **Common reported processes (%):** 14.29

- **PDB ID:** 3FVC
- **Chain:** A
- **Crystallized protein length:** 559 aa
- **Resolution:** 3.2 Å
- **b-phipsi:** 0.003876
- **w-rdist:** 0.826649
- **t-alpha:** 0.197866
- **Chemical similarity (Tanimoto Index) (%):** 99.87
- **1D identity (%) [PDB]:** 0.0
- **1D identity (%) [Gaps excluded][PDB]:** 0.0
- **1D identity - Alignment Gaps [PDB]:** 1604
- **2D identity (%) [PDB]:** 33.76
- **2D identity (%) [Gaps excluded][PDB]:** 90.16
- **2D identity - Alignment Gaps [PDB]:** 730
- **3D similarity (TM-Score) (%) [PDB]:** 17.56

- **Gene name:** gB
- **RefSeq ID:** NC\_007605
- **Genomic sequence length:** 171823
- **5-UTR|CDS|3-UTR identity (%):** N/A | 36.98 | N/A
- **5-UTR|CDS|3-UTR identity (%) [Gaps excluded]:** N/A | 77.33 | N/A
- **5-UTR|CDS|3-UTR identity [Alignment Gaps]:** N/A | 2258 | N/A

**Uniprot Description:**  
  
Envelope glycoprotein that forms spikes at the surface of virion envelope. Essential for the initial attachment to heparan sulfate moieties of the host cell surface proteoglycans. Involved in fusion of viral and cellular membranes leading to virus entry into the host cell. Following initial binding to its host receptors, membrane fusion is mediated by the fusion machinery composed at least of gB and the heterodimer gH/gL. May be involved in the fusion between the virion envelope and the outer nuclear membrane during virion egress.  
  
Homotrimer; disulfide-linked. Binds to heparan sulfate proteoglycans. Interacts with gH/gL heterodimer.  
  
**Gene Ontology Information:**

Molecular Function  
  
N/A

Location

- host cell endosome membrane
- host cell Golgi membrane
- host cell plasma membrane
- integral component of membrane
- viral envelope
- virion membrane

Biological process

- viral entry into host cell
- virion attachment to host cell

---

13

- **Protein name:** DNA-directed RNA polymerase subunit beta'
- **Organism:** Thermus thermophilus (strain HB8 / ATCC 27634 / DSM 579)
- **Uniprot Accession Number:** Q8RQE8
- **Protein sequence length:** 1524 aa
- **1D identity (%):** 12.47
- **1D identity (%) [Gaps excluded]:** 27.16
- **1D identity - Alignment Gaps:** 1037
- **Common reported functions (%):** 0.0
- **Common reported locations (%):** 0.0
- **Common reported processes (%):** 0.0

- **PDB ID:** 5XJ0
- **Chain:** D
- **Crystallized protein length:** 1462 aa
- **Resolution:** 4.0 Å
- **b-phipsi:** 0.015482
- **w-rdist:** 0.941675
- **t-alpha:** 0.027454
- **Chemical similarity (Tanimoto Index) (%):** 83.21
- **1D identity (%) [PDB]:** 0.04
- **1D identity (%) [Gaps excluded][PDB]:** 50.0
- **1D identity - Alignment Gaps [PDB]:** 2500
- **2D identity (%) [PDB]:** N/A
- **2D identity (%) [Gaps excluded][PDB]:** N/A
- **2D identity - Alignment Gaps [PDB]:** N/A
- **3D similarity (TM-Score) (%) [PDB]:** 21.1

- **Gene name:** rpoC
- **RefSeq ID:** N/A
- **Sequence length:** N/A
- **5-UTR|CDS|3-UTR identity (%):** N/A | N/A | N/A
- **5-UTR|CDS|3-UTR identity (%) [Gaps excluded]:** N/A | N/A | N/A
- **5-UTR|CDS|3-UTR identity [Alignment Gaps]:** N/A | N/A | N/A

**Uniprot Description:**  
  
DNA-dependent RNA polymerase catalyzes the transcription of DNA into RNA using the four ribonucleoside triphosphates as substrates.  
  
The RNAP catalytic core consists of 2 alpha, 1 beta, 1 beta' and 1 omega subunit. When a sigma factor is associated with the core the holoenzyme is formed, which can initiate transcription.  
  
**Gene Ontology Information:**

Molecular Function

- DNA binding
- DNA-directed 5'-3' RNA polymerase activity
- magnesium ion binding
- zinc ion binding

Location  
  
N/A

Biological process

- transcription, DNA-templated

---

14

- **Protein name:** Elongation factor Ts
- **Organism:** Escherichia coli (strain K12)
- **Uniprot Accession Number:** P0A6P1
- **Protein sequence length:** 283 aa
- **1D identity (%):** 4.19
- **1D identity (%) [Gaps excluded]:** 25.69
- **1D identity - Alignment Gaps:** 1120
- **Common reported functions (%):** 0.0
- **Common reported locations (%):** 0.0
- **Common reported processes (%):** 0.0

- **PDB ID:** 3AVX
- **Chain:** A
- **Crystallized protein length:** 1203 aa
- **Resolution:** 2.41 Å
- **b-phipsi:** 0.010613
- **w-rdist:** 1.158069
- **t-alpha:** 0.019433
- **Chemical similarity (Tanimoto Index) (%):** 78.69
- **1D identity (%) [PDB]:** 0.0
- **1D identity (%) [Gaps excluded][PDB]:** 0.0
- **1D identity - Alignment Gaps [PDB]:** 2247
- **2D identity (%) [PDB]:** 33.03
- **2D identity (%) [Gaps excluded][PDB]:** 92.55
- **2D identity - Alignment Gaps [PDB]:** 1065
- **3D similarity (TM-Score) (%) [PDB]:** 20.39

- **Gene name:** tsf
- **RefSeq ID:** N/A
- **Sequence length:** N/A
- **5-UTR|CDS|3-UTR identity (%):** N/A | N/A | N/A
- **5-UTR|CDS|3-UTR identity (%) [Gaps excluded]:** N/A | N/A | N/A
- **5-UTR|CDS|3-UTR identity [Alignment Gaps]:** N/A | N/A | N/A

**Uniprot Description:**  
  
Associates with the EF-Tu.GDP complex and induces the exchange of GDP to GTP. It remains bound to the aminoacyl-tRNA.EF-Tu.GTP complex up to the GTP hydrolysis stage on the ribosome.  
  
Heterotetramer composed of two EF-Ts.EF-Tu dimer complexes.  
  
**Gene Ontology Information:**

Molecular Function

- guanyl-nucleotide exchange factor activity
- translation elongation factor activity
- zinc ion binding

Location

- cytoplasm
- cytosol
- membrane

Biological process

- translational elongation

---

15

- **Protein name:** DNA-directed RNA polymerase 133 kDa polypeptide
- **Organism:** Vaccinia virus (strain Western Reserve)
- **Uniprot Accession Number:** Q76ZP7
- **Protein sequence length:** 1164 aa
- **1D identity (%):** 11.17
- **1D identity (%) [Gaps excluded]:** 31.5
- **1D identity - Alignment Gaps:** 1161
- **Common reported functions (%):** 0.0
- **Common reported locations (%):** 0.0
- **Common reported processes (%):** 0.0

- **PDB ID:** 6RIE
- **Chain:** B
- **Crystallized protein length:** 1129 aa
- **Resolution:** 3.1 Å
- **b-phipsi:** 0.007829
- **w-rdist:** 1.124034
- **t-alpha:** 0.02831
- **Chemical similarity (Tanimoto Index) (%):** 83.52
- **1D identity (%) [PDB]:** 0.09
- **1D identity (%) [Gaps excluded][PDB]:** 50.0
- **1D identity - Alignment Gaps [PDB]:** 2164
- **2D identity (%) [PDB]:** 24.74
- **2D identity (%) [Gaps excluded][PDB]:** 91.56
- **2D identity - Alignment Gaps [PDB]:** 1248
- **3D similarity (TM-Score) (%) [PDB]:** 20.41

- **Gene name:** RPO132
- **RefSeq ID:** NC\_006998
- **Genomic sequence length:** 194711
- **5-UTR|CDS|3-UTR identity (%):** N/A | 42.9 | N/A
- **5-UTR|CDS|3-UTR identity (%) [Gaps excluded]:** N/A | 78.1 | N/A
- **5-UTR|CDS|3-UTR identity [Alignment Gaps]:** N/A | 2129 | N/A

**Uniprot Description:**  
  
Part of the DNA-dependent RNA polymerase which catalyzes the transcription of viral DNA into RNA using the four ribonucleoside triphosphates as substrates. Responsible for the transcription of early, intermediate and late genes. DNA-dependent RNA polymerase associates with the early transcription factor (ETF), itself composed of D6 and A7, thereby allowing the early genes transcription. Late transcription, and probably also intermediate transcription, require newly synthesized RNA polymerase.  
  
The DNA-dependent RNA polymerase used for intermediate and late genes expression consists of eight subunits 147 kDa, 133 kDa, 35 kDa, 30 kDa, 22 kDa, 19 kDa, 18 kDa and 7 kDa totalling more than 500 kDa in mass. The same holoenzyme, with the addition of the transcription-specificity factor RAP94, is used for early gene expression.  
  
**Gene Ontology Information:**

Molecular Function

- DNA binding
- DNA-directed 5'-3' RNA polymerase activity
- metal ion binding
- ribonucleoside binding

Location

- virion

Biological process

- transcription, DNA-templated

---

16

- **Protein name:** Triplex capsid protein 2
- **Organism:** Human herpesvirus 1
- **Uniprot Accession Number:** G8H8D9
- **Protein sequence length:** 318 aa
- **1D identity (%):** 5.54
- **1D identity (%) [Gaps excluded]:** 29.02
- **1D identity - Alignment Gaps:** 1081
- **Common reported functions (%):** 0.0
- **Common reported locations (%):** 0.0
- **Common reported processes (%):** 0.0

- **PDB ID:** 6ODM
- **Chain:** S
- **Crystallized protein length:** 1328 aa
- **Resolution:** 4.3 Å
- **b-phipsi:** 0.016292
- **w-rdist:** 0.774807
- **t-alpha:** 0.146704
- **Chemical similarity (Tanimoto Index) (%):** 83.9
- **1D identity (%) [PDB]:** 0.0
- **1D identity (%) [Gaps excluded][PDB]:** 0.0
- **1D identity - Alignment Gaps [PDB]:** 2369
- **2D identity (%) [PDB]:** 27.73
- **2D identity (%) [Gaps excluded][PDB]:** 89.8
- **2D identity - Alignment Gaps [PDB]:** 1251
- **3D similarity (TM-Score) (%) [PDB]:** 4.35

- **Gene name:** UL18
- **RefSeq ID:** NC\_001806
- **Genomic sequence length:** 152222
- **5-UTR|CDS|3-UTR identity (%):** N/A | 13.67 | N/A
- **5-UTR|CDS|3-UTR identity (%) [Gaps excluded]:** N/A | 74.39 | N/A
- **5-UTR|CDS|3-UTR identity [Alignment Gaps]:** N/A | 3295 | N/A

**Uniprot Description:**  
  
Structural component of the T=16 icosahedral capsid. The capsid is composed of pentamers and hexamers of major capsid protein/MCP, which are linked together by heterotrimers called triplexes. These triplexes are formed by a single molecule of triplex protein 1/TRX1 and two copies of triplex protein 2/TRX2. Additionally, TRX1 is required for efficient transport of TRX2 to the nucleus, which is the site of capsid assembly.  
  
Interacts with TRX1 and major capisd protein/MCP.  
  
**Gene Ontology Information:**

Molecular Function  
  
N/A

Location  
  
N/A

Biological process  
  
N/A

---

17

- **Protein name:** DNA-directed RNA polymerase subunit beta
- **Organism:** Escherichia coli (strain K12)
- **Uniprot Accession Number:** P0A8V2
- **Protein sequence length:** 1342 aa
- **1D identity (%):** 13.69
- **1D identity (%) [Gaps excluded]:** 28.22
- **1D identity - Alignment Gaps:** 907
- **Common reported functions (%):** 0.0
- **Common reported locations (%):** 0.0
- **Common reported processes (%):** 0.0

- **PDB ID:** 6R9G
- **Chain:** C
- **Crystallized protein length:** 1341 aa
- **Resolution:** 3.7 Å
- **b-phipsi:** 0.018637
- **w-rdist:** 0.93933
- **t-alpha:** 0.031746
- **Chemical similarity (Tanimoto Index) (%):** 83.9
- **1D identity (%) [PDB]:** 0.0
- **1D identity (%) [Gaps excluded][PDB]:** 0.0
- **1D identity - Alignment Gaps [PDB]:** 2381
- **2D identity (%) [PDB]:** 29.83
- **2D identity (%) [Gaps excluded][PDB]:** 89.73
- **2D identity - Alignment Gaps [PDB]:** 1193
- **3D similarity (TM-Score) (%) [PDB]:** 23.34

- **Gene name:** rpoB
- **RefSeq ID:** N/A
- **Sequence length:** N/A
- **5-UTR|CDS|3-UTR identity (%):** N/A | N/A | N/A
- **5-UTR|CDS|3-UTR identity (%) [Gaps excluded]:** N/A | N/A | N/A
- **5-UTR|CDS|3-UTR identity [Alignment Gaps]:** N/A | N/A | N/A

**Uniprot Description:**  
  
DNA-dependent RNA polymerase (RNAP) catalyzes the transcription of DNA into RNA using the four ribonucleoside triphosphates as substrates.  
  
The RNAP catalytic core consists of 2 alpha, 1 beta, 1 beta' and 1 omega subunit. When a sigma factor is associated with the core the holoenzyme is formed, which can initiate transcription. The rRNA transcription and antitermination complex (rrnTAC) consists of RNAP, NusA, NusB, NusE (rpsJ), NusG, SubB, ribosomal protein S4, DNA and precursor rRNA; S4 is more flexible than other subunits (PubMed:32871103).  
  
**Gene Ontology Information:**

Molecular Function

- DNA binding
- DNA-directed 5'-3' RNA polymerase activity
- ribonucleoside binding

Location

- cytoplasm
- cytosol
- membrane

Biological process

- response to antibiotic
- transcription, DNA-templated

---

18

- **Protein name:** Tail fiber protein
- **Organism:** Escherichia phage vB\_EcoP\_G7C
- **Uniprot Accession Number:** G0XNW5
- **Protein sequence length:** 851 aa
- **1D identity (%):** 12.09
- **1D identity (%) [Gaps excluded]:** 27.84
- **1D identity - Alignment Gaps:** 838
- **Common reported functions (%):** 0.0
- **Common reported locations (%):** 0.0
- **Common reported processes (%):** 0.0

- **PDB ID:** 4QNL
- **Chain:** A
- **Crystallized protein length:** 826 aa
- **Resolution:** 2.41 Å
- **b-phipsi:** 0.004623
- **w-rdist:** 0.933927
- **t-alpha:** 0.216749
- **Chemical similarity (Tanimoto Index) (%):** 83.83
- **1D identity (%) [PDB]:** 0.0
- **1D identity (%) [Gaps excluded][PDB]:** 0.0
- **1D identity - Alignment Gaps [PDB]:** 1887
- **2D identity (%) [PDB]:** 25.77
- **2D identity (%) [Gaps excluded][PDB]:** 90.0
- **2D identity - Alignment Gaps [PDB]:** 1047
- **3D similarity (TM-Score) (%) [PDB]:** 19.79

- **Gene name:** gp63.1
- **RefSeq ID:** NC\_015933
- **Genomic sequence length:** 72917
- **5-UTR|CDS|3-UTR identity (%):** N/A | 40.17 | N/A
- **5-UTR|CDS|3-UTR identity (%) [Gaps excluded]:** N/A | 79.34 | N/A
- **5-UTR|CDS|3-UTR identity [Alignment Gaps]:** N/A | 2090 | N/A

**Uniprot Description:**  
  
N/A  
  
**Gene Ontology Information:**

Molecular Function  
  
N/A

Location  
  
N/A

Biological process  
  
N/A

---

19

- **Protein name:** Major envelope glycoprotein
- **Organism:** Autographa californica nuclear polyhedrosis virus
- **Uniprot Accession Number:** P17501
- **Protein sequence length:** 512 aa
- **1D identity (%):** 8.55
- **1D identity (%) [Gaps excluded]:** 27.1
- **1D identity - Alignment Gaps:** 929
- **Common reported functions (%):** 50.0
- **Common reported locations (%):** 80.0
- **Common reported processes (%):** 28.57

- **PDB ID:** 3DUZ
- **Chain:** A
- **Crystallized protein length:** 418 aa
- **Resolution:** 2.95 Å
- **b-phipsi:** 0.001906
- **w-rdist:** 0.973596
- **t-alpha:** 0.228856
- **Chemical similarity (Tanimoto Index) (%):** 84.82
- **1D identity (%) [PDB]:** 0.0
- **1D identity (%) [Gaps excluded][PDB]:** 0.0
- **1D identity - Alignment Gaps [PDB]:** 1460
- **2D identity (%) [PDB]:** 25.15
- **2D identity (%) [Gaps excluded][PDB]:** 87.16
- **2D identity - Alignment Gaps [PDB]:** 806
- **3D similarity (TM-Score) (%) [PDB]:** 12.06

- **Gene name:** GP64
- **RefSeq ID:** NC\_001623
- **Genomic sequence length:** 133894
- **5-UTR|CDS|3-UTR identity (%):** N/A | 23.48 | N/A
- **5-UTR|CDS|3-UTR identity (%) [Gaps excluded]:** N/A | 77.86 | N/A
- **5-UTR|CDS|3-UTR identity [Alignment Gaps]:** N/A | 2877 | N/A

**Uniprot Description:**  
  
Class III viral fusion protein. Envelope phosphoglycoprotein which mediates the fusion of viral and host endosomal membranes leading to virus entry into the host cell. After receptor-mediated internalization of the virus, gp64 undergoes conformational change into a fusion-competent state at low pH, and the nucleocapsid is released into the cytoplasm after cell fusion. May also play role in budding.  
  
Homotrimer; disulfide-linked.  
  
**Gene Ontology Information:**

Molecular Function

- identical protein binding

Location

- host cell plasma membrane
- integral component of membrane
- viral envelope
- virion membrane

Biological process

- fusion of virus membrane with host endosome membrane
- membrane fusion involved in viral entry into host cell
- modulation by virus of host process
- viral budding from plasma membrane
- viral entry into host cell

---

20

- **Protein name:** Pre-neck appendage protein
- **Organism:** Bacillus phage phi29
- **Uniprot Accession Number:** P20345
- **Protein sequence length:** 854 aa
- **1D identity (%):** N/A
- **1D identity (%) [Gaps excluded]:** N/A
- **1D identity - Alignment Gaps:** N/A
- **Common reported functions (%):** N/A
- **Common reported locations (%):** N/A
- **Common reported processes (%):** N/A

- **PDB ID:** 3SUC
- **Chain:** A
- **Crystallized protein length:** 767 aa
- **Resolution:** 2.15 Å
- **b-phipsi:** 0.029204
- **w-rdist:** 0.859276
- **t-alpha:** 0.057363
- **Chemical similarity (Tanimoto Index) (%):** N/A
- **1D identity (%) [PDB]:** N/A
- **1D identity (%) [Gaps excluded][PDB]:** N/A
- **1D identity - Alignment Gaps [PDB]:** N/A
- **2D identity (%) [PDB]:** N/A
- **2D identity (%) [Gaps excluded][PDB]:** N/A
- **2D identity - Alignment Gaps [PDB]:** N/A
- **3D similarity (TM-Score) (%) [PDB]:** N/A

- **Gene name:** 12
- **RefSeq ID:** N/A
- **Sequence length:** N/A
- **5-UTR|CDS|3-UTR identity (%):** N/A | N/A | N/A
- **5-UTR|CDS|3-UTR identity (%) [Gaps excluded]:** N/A | N/A | N/A
- **5-UTR|CDS|3-UTR identity [Alignment Gaps]:** N/A | N/A | N/A

**Uniprot Description:**  
  
Structural component of the 12 appendages that hang from the lower collar. Adhesion protein that binds to the host cell surface during virus attachment and mediates teichoic acids degradation.  
  
Homotrimer. Each appendage is a homotrimer of gp12\*.  
  
**Gene Ontology Information:**

Molecular Function

- ATP binding
- metal ion binding

Location

- virus tail, fiber

Biological process

- adhesion receptor-mediated virion attachment to host cell
- disruption of host cell envelope during viral entry
- virion attachment to host cell

---

21

- **Protein name:** Envelope glycoprotein H
- **Organism:** Epstein-Barr virus (strain B95-8)
- **Uniprot Accession Number:** P03231
- **Protein sequence length:** 706 aa
- **1D identity (%):** 5.74
- **1D identity (%) [Gaps excluded]:** 29.41
- **1D identity - Alignment Gaps:** 1333
- **Common reported functions (%):** 0.0
- **Common reported locations (%):** 80.0
- **Common reported processes (%):** 14.29

- **PDB ID:** 5W0K
- **Chain:** A
- **Crystallized protein length:** 655 aa
- **Resolution:** 3.1 Å
- **b-phipsi:** 0.014537
- **w-rdist:** 1.366706
- **t-alpha:** 0.007341
- **Chemical similarity (Tanimoto Index) (%):** N/A
- **1D identity (%) [PDB]:** 0.06
- **1D identity (%) [Gaps excluded][PDB]:** 50.0
- **1D identity - Alignment Gaps [PDB]:** 1691
- **2D identity (%) [PDB]:** 31.61
- **2D identity (%) [Gaps excluded][PDB]:** 90.43
- **2D identity - Alignment Gaps [PDB]:** 817
- **3D similarity (TM-Score) (%) [PDB]:** 17.44

- **Gene name:** gH
- **RefSeq ID:** NC\_007605
- **Genomic sequence length:** 171823
- **5-UTR|CDS|3-UTR identity (%):** N/A | 29.21 | N/A
- **5-UTR|CDS|3-UTR identity (%) [Gaps excluded]:** N/A | 78.43 | N/A
- **5-UTR|CDS|3-UTR identity [Alignment Gaps]:** N/A | 2717 | N/A

**Uniprot Description:**  
  
The heterodimer glycoprotein H-glycoprotein L is required for the fusion of viral and plasma membranes leading to virus entry into the host cell. Following initial binding to host receptor, membrane fusion is mediated by the fusion machinery composed of gB and the heterodimer gH/gL. May also be involved in the fusion between the virion envelope and the outer nuclear membrane during virion morphogenesis. The heterodimer gH/gL targets also host EPHA2 to promote viral entry.  
  
Interacts with glycoprotein L (gL); this interaction is necessary for the correct processing and cell surface expression of gH. The heterodimer gH/gL seems to interact with gB trimers during fusion. The heterodimer gH/gL interacts with host EPHA2 to facilitate virus internalization and fusion (PubMed:29292384). Interacts with glycoprotein 42/BZLF2 (PubMed:27929061).  
  
**Gene Ontology Information:**

Molecular Function  
  
N/A

Location

- host cell endosome membrane
- host cell plasma membrane
- integral component of membrane
- viral envelope
- virion membrane

Biological process

- fusion of virus membrane with host plasma membrane

---

22

- **Protein name:** Major capsid protein
- **Organism:** Human herpesvirus 8 type P (isolate GK18)
- **Uniprot Accession Number:** Q2HRA7
- **Protein sequence length:** 1376 aa
- **1D identity (%):** 16.12
- **1D identity (%) [Gaps excluded]:** 28.0
- **1D identity - Alignment Gaps:** 713
- **Common reported functions (%):** 0.0
- **Common reported locations (%):** 0.0
- **Common reported processes (%):** 0.0

- **PDB ID:** 6PPD
- **Chain:** T
- **Crystallized protein length:** 1360 aa
- **Resolution:** 3.7 Å
- **b-phipsi:** 0.030168
- **w-rdist:** 0.681971
- **t-alpha:** 0.118659
- **Chemical similarity (Tanimoto Index) (%):** 83.67
- **1D identity (%) [PDB]:** 0.0
- **1D identity (%) [Gaps excluded][PDB]:** 0.0
- **1D identity - Alignment Gaps [PDB]:** 2402
- **2D identity (%) [PDB]:** 28.62
- **2D identity (%) [Gaps excluded][PDB]:** 90.31
- **2D identity - Alignment Gaps [PDB]:** 1246
- **3D similarity (TM-Score) (%) [PDB]:** 9.17

- **Gene name:** MCP
- **RefSeq ID:** NC\_009333
- **Genomic sequence length:** 137969
- **5-UTR|CDS|3-UTR identity (%):** N/A | 39.87 | N/A
- **5-UTR|CDS|3-UTR identity (%) [Gaps excluded]:** N/A | 77.0 | N/A
- **5-UTR|CDS|3-UTR identity [Alignment Gaps]:** N/A | 2527 | N/A

**Uniprot Description:**  
  
Self-assembles to form an icosahedral capsid with a T=16 symmetry, about 200 nm in diameter, and consisting of 150 hexons and 12 pentons (total of 162 capsomers). Hexons form the edges and faces of the capsid and are each composed of six MCP molecules. In contrast, one penton is found at each of the 12 vertices. Eleven of the pentons are MCP pentamers, while the last vertex is occupied by the portal complex. The capsid is surrounded by a layer of proteinaceous material designated the tegument which, in turn, is enclosed in an envelope of host cell-derived lipids containing virus-encoded glycoproteins.  
  
Homomultimer. Makes the hexons and eleven out of twelve pentons. Interacts with triplex proteins 1/TRX1 and 2/TRX2; adjacent capsomers are linked together in groups of three by triplexes, heterotrimeric complexes composed of one molecule of TRX1 and two molecules of TRX2. Interacts with scaffold protein; this interaction allows efficient MCP transport to the host nucleus. Interacts with capsid vertex component 2/CVC2. Interacts with the small capsomere-interacting protein/SCP.  
  
**Gene Ontology Information:**

Molecular Function

- structural molecule activity

Location

- host cell nucleus
- T=16 icosahedral viral capsid
- virion

Biological process  
  
N/A

---

23

- **Protein name:** DNA-directed DNA polymerase
- **Organism:** Escherichia phage RB69
- **Uniprot Accession Number:** Q38087
- **Protein sequence length:** 903 aa
- **1D identity (%):** 12.76
- **1D identity (%) [Gaps excluded]:** 27.66
- **1D identity - Alignment Gaps:** 802
- **Common reported functions (%):** 0.0
- **Common reported locations (%):** 0.0
- **Common reported processes (%):** 0.0

- **PDB ID:** 2DTU
- **Chain:** A
- **Crystallized protein length:** 896 aa
- **Resolution:** 2.37 Å
- **b-phipsi:** 0.014287
- **w-rdist:** 1.300705
- **t-alpha:** 0.020661
- **Chemical similarity (Tanimoto Index) (%):** 83.83
- **1D identity (%) [PDB]:** 0.0
- **1D identity (%) [Gaps excluded][PDB]:** 0.0
- **1D identity - Alignment Gaps [PDB]:** 1937
- **2D identity (%) [PDB]:** 29.97
- **2D identity (%) [Gaps excluded][PDB]:** 90.46
- **2D identity - Alignment Gaps [PDB]:** 973
- **3D similarity (TM-Score) (%) [PDB]:** 18.88

- **Gene name:** 43
- **RefSeq ID:** NC\_004928
- **Genomic sequence length:** 167560
- **5-UTR|CDS|3-UTR identity (%):** N/A | 40.03 | N/A
- **5-UTR|CDS|3-UTR identity (%) [Gaps excluded]:** N/A | 78.33 | N/A
- **5-UTR|CDS|3-UTR identity [Alignment Gaps]:** N/A | 2114 | N/A

**Uniprot Description:**  
  
Replicates the viral genomic DNA. This polymerase possesses two enzymatic activities: DNA synthesis (polymerase) and an exonucleolytic activity that degrades single-stranded DNA in the 3'- to 5'-direction for proofreading purpose.  
  
Part of the replicase complex that includes the DNA polymerase, the polymerase clamp, the clamp loader complex, the single-stranded DNA binding protein, and the primase/helicase (By similarity). Interacts with the polymerase clamp; this interaction constitutes the polymerase holoenzyme (PubMed:10535734).  
  
**Gene Ontology Information:**

Molecular Function

- 3'-5' exonuclease activity
- DNA binding
- DNA-directed DNA polymerase activity
- metal ion binding
- nucleotide binding

Location  
  
N/A

Biological process

- bidirectional double-stranded viral DNA replication
- DNA replication

---

24

- **Protein name:** Splicing factor 3B subunit 3
- **Organism:** Homo sapiens
- **Uniprot Accession Number:** Q15393
- **Protein sequence length:** 1217 aa
- **1D identity (%):** 9.88
- **1D identity (%) [Gaps excluded]:** 30.59
- **1D identity - Alignment Gaps:** 1274
- **Common reported functions (%):** 0.0
- **Common reported locations (%):** 0.0
- **Common reported processes (%):** 0.0

- **PDB ID:** 5Z58
- **Chain:** 3
- **Crystallized protein length:** 1177 aa
- **Resolution:** 4.9 Å
- **b-phipsi:** 0.005688
- **w-rdist:** 1.236768
- **t-alpha:** 0.068339
- **Chemical similarity (Tanimoto Index) (%):** 83.9
- **1D identity (%) [PDB]:** 0.0
- **1D identity (%) [Gaps excluded][PDB]:** 0.0
- **1D identity - Alignment Gaps [PDB]:** 2217
- **2D identity (%) [PDB]:** N/A
- **2D identity (%) [Gaps excluded][PDB]:** N/A
- **2D identity - Alignment Gaps [PDB]:** N/A
- **3D similarity (TM-Score) (%) [PDB]:** 23.47

- **Gene name:** SF3B3
- **RefSeq ID:** NM\_012426
- **Transcript sequence length:** 9692
- **5-UTR|CDS|3-UTR identity (%):** 34.95 | 42.2 | 2.75
- **5-UTR|CDS|3-UTR identity (%) [Gaps excluded]:** 77.7 | 77.76 | 83.51
- **5-UTR|CDS|3-UTR identity [Alignment Gaps]:** 170 | 2216 | 5696

**Uniprot Description:**  
  
Involved in pre-mRNA splicing as a component of the splicing factor SF3B complex, a constituent of the spliceosome (PubMed:10490618, PubMed:10882114, PubMed:27720643, PubMed:28781166). SF3B complex is required for 'A' complex assembly formed by the stable binding of U2 snRNP to the branchpoint sequence (BPS) in pre-mRNA. Sequence independent binding of SF3A/SF3B complex upstream of the branch site is essential, it may anchor U2 snRNP to the pre-mRNA (PubMed:12234937). May also be involved in the assembly of the 'E' complex (PubMed:10882114). Belongs also to the minor U12-dependent spliceosome, which is involved in the splicing of rare class of nuclear pre-mRNA intron (PubMed:15146077).  
  
Identified in the spliceosome A complex; remains associated with the spliceosome throughout the splicing process (PubMed:10490618). Component of the spliceosome B complex (PubMed:28781166). Identified in the spliceosome C complex (PubMed:11991638). Identified in the spliceosome E complex (PubMed:10882114). Component of the U11/U12 snRNPs that are part of the U12-type spliceosome (PubMed:15146077). Component of splicing factor SF3B complex which is composed of at least eight subunits; SF3B1, SF3B2, SF3B3, SF3B4, SF3B5, SF3B6, PHF5A and DDX42 (PubMed:12234937, PubMed:12738865, PubMed:28541300, PubMed:27720643). SF3B associates with the splicing factor SF3A and a 12S RNA unit to form the U2 small nuclear ribonucleoproteins complex (U2 snRNP). Interaction between SF3B3 and SF3B1 is tighter than the interaction between SF3B3 and SF3B2 (PubMed:12234937). Within the SF3B complex interacts directly with SF3B1 (via HEAT domain), SF3B5 and PHF5A (PubMed:27720643). The SF3B complex composed of SF3B1, SF3B2, SF3B3, SF3B4, SF3B5, SF3B6 and PHF5A interacts with U2AF2 (PubMed:27720643). Associates with the STAGA transcription coactivator-HAT complex. Interacts with SUPT3H (PubMed:11564863). Interacts with TAF3 (PubMed:11438666).  
  
**Gene Ontology Information:**

Molecular Function

- protein-containing complex binding
- U2 snRNA binding

Location

- catalytic step 2 spliceosome
- nucleolus
- nucleoplasm
- nucleus
- U12-type spliceosomal complex
- U2 snRNP
- U2-type precatalytic spliceosome

Biological process

- mRNA splicing, via spliceosome
- negative regulation of protein catabolic process
- RNA splicing
- RNA splicing, via transesterification reactions

---

25

- **Protein name:** DNA-directed RNA polymerase subunit beta'
- **Organism:** Escherichia coli (strain K12)
- **Uniprot Accession Number:** P0A8T7
- **Protein sequence length:** 1407 aa
- **1D identity (%):** 13.96
- **1D identity (%) [Gaps excluded]:** 28.12
- **1D identity - Alignment Gaps:** 902
- **Common reported functions (%):** 0.0
- **Common reported locations (%):** 0.0
- **Common reported processes (%):** 0.0

- **PDB ID:** 6K4Y
- **Chain:** D
- **Crystallized protein length:** 1335 aa
- **Resolution:** 3.79 Å
- **b-phipsi:** 0.026009
- **w-rdist:** 0.980531
- **t-alpha:** 0.024291
- **Chemical similarity (Tanimoto Index) (%):** 83.32
- **1D identity (%) [PDB]:** 0.08
- **1D identity (%) [Gaps excluded][PDB]:** 50.0
- **1D identity - Alignment Gaps [PDB]:** 2369
- **2D identity (%) [PDB]:** 27.09
- **2D identity (%) [Gaps excluded][PDB]:** 90.0
- **2D identity - Alignment Gaps [PDB]:** 1277
- **3D similarity (TM-Score) (%) [PDB]:** 19.3

- **Gene name:** rpoC
- **RefSeq ID:** N/A
- **Sequence length:** N/A
- **5-UTR|CDS|3-UTR identity (%):** N/A | N/A | N/A
- **5-UTR|CDS|3-UTR identity (%) [Gaps excluded]:** N/A | N/A | N/A
- **5-UTR|CDS|3-UTR identity [Alignment Gaps]:** N/A | N/A | N/A

**Uniprot Description:**  
  
DNA-dependent RNA polymerase (RNAP) catalyzes the transcription of DNA into RNA using the four ribonucleoside triphosphates as substrates.  
  
The RNAP catalytic core consists of 2 alpha, 1 beta, 1 beta' and 1 omega subunit. When a sigma factor is associated with the core the holoenzyme is formed, which can initiate transcription. The rRNA transcription and antitermination complex (rrnTAC) consists of RNAP, NusA, NusB, NusE (rpsJ), NusG, SubB, ribosomal protein S4, DNA and precursor rRNA; S4 is more flexible than other subunits (PubMed:32871103).  
  
**Gene Ontology Information:**

Molecular Function

- DNA binding
- DNA-directed 5'-3' RNA polymerase activity
- magnesium ion binding
- zinc ion binding

Location

- cytoplasm
- cytosol
- membrane

Biological process

- response to antibiotic
- transcription, DNA-templated

---

26

- **Protein name:** Ubiquitin carboxyl-terminal hydrolase 7
- **Organism:** Homo sapiens
- **Uniprot Accession Number:** Q93009
- **Protein sequence length:** 1102 aa
- **1D identity (%):** 12.39
- **1D identity (%) [Gaps excluded]:** 26.28
- **1D identity - Alignment Gaps:** 853
- **Common reported functions (%):** 0.0
- **Common reported locations (%):** 0.0
- **Common reported processes (%):** 0.0

- **PDB ID:** 5C56
- **Chain:** A
- **Crystallized protein length:** 528 aa
- **Resolution:** 2.69 Å
- **b-phipsi:** 0.003217
- **w-rdist:** 0.96409
- **t-alpha:** 0.352683
- **Chemical similarity (Tanimoto Index) (%):** 83.51
- **1D identity (%) [PDB]:** 0.13
- **1D identity (%) [Gaps excluded][PDB]:** 50.0
- **1D identity - Alignment Gaps [PDB]:** 1560
- **2D identity (%) [PDB]:** 23.62
- **2D identity (%) [Gaps excluded][PDB]:** 87.95
- **2D identity - Alignment Gaps [PDB]:** 904
- **3D similarity (TM-Score) (%) [PDB]:** 1.04

- **Gene name:** USP7
- **RefSeq ID:** NM\_003470
- **Transcript sequence length:** 5831
- **5-UTR|CDS|3-UTR identity (%):** 8.07 | 42.68 | 7.82
- **5-UTR|CDS|3-UTR identity (%) [Gaps excluded]:** 80.25 | 78.87 | 76.26
- **5-UTR|CDS|3-UTR identity [Alignment Gaps]:** 724 | 2123 | 1734

**Uniprot Description:**  
  
Hydrolase that deubiquitinates target proteins such as FOXO4, p53/TP53, MDM2, ERCC6, DNMT1, UHRF1, PTEN, KMT2E/MLL5 and DAXX (PubMed:11923872, PubMed:15053880, PubMed:16964248, PubMed:18716620, PubMed:25283148, PubMed:26678539, PubMed:28655758). Together with DAXX, prevents MDM2 self-ubiquitination and enhances the E3 ligase activity of MDM2 towards p53/TP53, thereby promoting p53/TP53 ubiquitination and proteasomal degradation (PubMed:15053880, PubMed:16845383, PubMed:18566590, PubMed:20153724). Deubiquitinates p53/TP53, preventing degradation of p53/TP53, and enhances p53/TP53-dependent transcription regulation, cell growth repression and apoptosis (PubMed:25283148). Deubiquitinates p53/TP53 and MDM2 and strongly stabilizes p53/TP53 even in the presence of excess MDM2, and also induces p53/TP53-dependent cell growth repression and apoptosis (PubMed:11923872). Deubiquitination of FOXO4 in presence of hydrogen peroxide is not dependent on p53/TP53 and inhibits FOXO4-induced transcriptional activity (PubMed:16964248). In association with DAXX, is involved in the deubiquitination and translocation of PTEN from the nucleus to the cytoplasm, both processes that are counteracted by PML (PubMed:18716620). Deubiquitinates KMT2E/MLL5 preventing KMT2E/MLL5 proteasomal-mediated degradation (PubMed:26678539). Involved in cell proliferation during early embryonic development. Involved in transcription-coupled nucleotide excision repair (TC-NER) in response to UV damage: recruited to DNA damage sites following interaction with KIAA1530/UVSSA and promotes deubiquitination of ERCC6, preventing UV-induced degradation of ERCC6 (PubMed:22466611, PubMed:22466612). Involved in maintenance of DNA methylation via its interaction with UHRF1 and DNMT1: acts by mediating deubiquitination of UHRF1 and DNMT1, preventing their degradation and promoting DNA methylation by DNMT1 (PubMed:21745816, PubMed:22411829). Deubiquitinates alkylation repair enzyme ALKBH3. OTUD4 recruits USP7 and USP9X to stabilize ALKBH3, thereby promoting the repair of alkylated DNA lesions (PubMed:25944111). Acts as a chromatin regulator via its association with the Polycomb group (PcG) multiprotein PRC1-like complex; may act by deubiquitinating components of the PRC1-like complex (PubMed:20601937). Able to mediate deubiquitination of histone H2B; it is however unsure whether this activity takes place in vivo (PubMed:20601937). Exhibits a preference towards 'Lys-48'-linked ubiquitin chains (PubMed:22689415). Increases regulatory T-cells (Treg) suppressive capacity by deubiquitinating and stabilizing the transcription factor FOXP3 which is crucial for Treg cell function (PubMed:23973222). Plays a role in the maintenance of the circadian clock periodicity via deubiquitination and stabilization of the CRY1 and CRY2 proteins (PubMed:27123980). Deubiquitinates REST, thereby stabilizing REST and promoting the maintenance of neural progenitor cells (PubMed:21258371). Deubiquitinates SIRT7, inhibiting SIRT7 histone deacetylase activity and regulating gluconeogenesis (PubMed:28655758).  
  
Monomer. Homodimer. Part of a complex with DAXX, MDM2, RASSF1 and USP7 (PubMed:18566590). Part of a complex with DAXX, MDM2 and USP7 (PubMed:16845383). Interacts with MDM2; the interaction is independent of p53/TP53. Interacts with DAXX; the interaction is direct and independent of MDM2 and p53/TP53 (PubMed:16845383). Component of a complex composed of KMT2E/MLL5 (isoform 3), OGT (isoform 1) and USP7; the complex stabilizes KMT2E/MLL5, preventing KMT2E/MLL5 ubiquitination and proteosomal-mediated degradation (PubMed:26678539). Interacts (via MATH domain) with KMT2E/MLL5 isoform 3 (PubMed:26678539). Interacts with OGT isoform 1 (PubMed:26678539). Interacts with FOXO4; the interaction is enhanced in presence of hydrogen peroxide and occurs independently of p53/TP53 (PubMed:16964248). Interacts with p53/TP53; the interaction is enhanced in response to DNA damage (PubMed:25283148). Interacts with TSPYL5; this impairs interaction with p53/TP53 (PubMed:21170034). Interacts with PTEN; the interaction is direct (PubMed:18716620). Interacts with ATXN1 and the strength of interaction is influenced by the length of the poly-Gln region in ATXN1 (PubMed:12093161). A weaker interaction seen with mutants having longer poly-Gln regions (PubMed:12093161). Interacts with KIAA1530/UVSSA (PubMed:22466611, PubMed:22466612). Interacts with ABRAXAS2; the interaction is direct (PubMed:25283148). Identified in a complex with TP53/p53 and ABRAXAS2 (PubMed:25283148). Interacts with MEX3C and antagonizes its ability to degrade mRNA (PubMed:22863774). Interacts with DNMT1 and UHRF1 (PubMed:21745816, PubMed:22411829). Interacts with FOXP3 (PubMed:23973222). Interacts (via MATH domain) with RNF220. Associated component of the Polycomb group (PcG) multiprotein PRC1-like complex (PubMed:20601937). Interacts with EPOP (By similarity). Interacts with OTUD4 and USP9X; the interaction is direct (PubMed:25944111). Interacts with CRY2 (PubMed:27123980). Interacts with REST (PubMed:21258371). Interacts with ERCC6 (PubMed:26030138).  
  
**Gene Ontology Information:**

Molecular Function

- cysteine-type endopeptidase activity
- ubiquitinyl hydrolase activity
- Lys48-specific deubiquitinase activity
- p53 binding
- protein C-terminus binding
- thiol-dependent ubiquitin-specific protease activity
- transcription factor binding
- ubiquitin protein ligase binding

Location

- chromosome
- cytosol
- nuclear body
- nucleoplasm
- nucleus
- PML body
- protein-containing complex

Biological process

- histone H2B conserved C-terminal lysine deubiquitination
- maintenance of DNA methylation
- monoubiquitinated protein deubiquitination
- multicellular organism development
- negative regulation of NF-kappaB transcription factor activity
- negative regulation of proteasomal ubiquitin-dependent protein catabolic process
- positive regulation of DNA demethylation
- protein deubiquitination
- protein K63-linked deubiquitination
- protein stabilization
- protein ubiquitination
- regulation of circadian rhythm
- regulation of DNA-binding transcription factor activity
- regulation of gluconeogenesis
- regulation of protein stability
- regulation of retrograde transport, endosome to Golgi
- regulation of signal transduction by p53 class mediator
- regulation of telomere capping
- rhythmic process
- transcription-coupled nucleotide-excision repair
- ubiquitin-dependent protein catabolic process
- viral process

---

27

- **Protein name:** Cleavage and polyadenylation specificity factor subunit 1
- **Organism:** Homo sapiens
- **Uniprot Accession Number:** Q10570
- **Protein sequence length:** 1443 aa
- **1D identity (%):** 1.5
- **1D identity (%) [Gaps excluded]:** 32.23
- **1D identity - Alignment Gaps:** 2474
- **Common reported functions (%):** 0.0
- **Common reported locations (%):** 0.0
- **Common reported processes (%):** 0.0

- **PDB ID:** 6FUW
- **Chain:** A
- **Crystallized protein length:** 1181 aa
- **Resolution:** 3.07 Å
- **b-phipsi:** 0.012328
- **w-rdist:** 1.259212
- **t-alpha:** 0.039562
- **Chemical similarity (Tanimoto Index) (%):** 83.67
- **1D identity (%) [PDB]:** 0.0
- **1D identity (%) [Gaps excluded][PDB]:** 0.0
- **1D identity - Alignment Gaps [PDB]:** 2232
- **2D identity (%) [PDB]:** 32.92
- **2D identity (%) [Gaps excluded][PDB]:** 85.21
- **2D identity - Alignment Gaps [PDB]:** 988
- **3D similarity (TM-Score) (%) [PDB]:** 20.77

- **Gene name:** CPSF1
- **RefSeq ID:** NM\_013291
- **Transcript sequence length:** 4480
- **5-UTR|CDS|3-UTR identity (%):** 15.38 | 36.42 | 23.21
- **5-UTR|CDS|3-UTR identity (%) [Gaps excluded]:** 79.25 | 76.03 | 69.62
- **5-UTR|CDS|3-UTR identity [Alignment Gaps]:** 220 | 2872 | 158

**Uniprot Description:**  
  
Component of the cleavage and polyadenylation specificity factor (CPSF) complex that plays a key role in pre-mRNA 3'-end formation, recognizing the AAUAAA signal sequence and interacting with poly(A) polymerase and other factors to bring about cleavage and poly(A) addition. This subunit is involved in the RNA recognition step of the polyadenylation reaction (PubMed:14749727). May play a role in eye morphogenesis and the development of retinal ganglion cell projections to the midbrain (By similarity).  
  
Component of the cleavage and polyadenylation specificity factor (CPSF) complex, composed of CPSF1, CPSF2, CPSF3, CPSF4 and FIP1L1. Found in a complex with CPSF1, FIP1L1 and PAPOLA. Interacts with FIP1L1, TENT2/GLD2 and SRRM1. Interacts with TUT1; the interaction is direct and mediates the recruitment of the CPSF complex on the 3'UTR of selected pre-mRNAs.  
  
**Gene Ontology Information:**

Molecular Function

- enzyme binding
- mRNA 3'-UTR AU-rich region binding

Location

- mRNA cleavage and polyadenylation specificity factor complex
- nucleoplasm
- nucleus

Biological process

- mRNA 3'-end processing
- mRNA export from nucleus
- mRNA polyadenylation
- mRNA splicing, via spliceosome
- pre-mRNA cleavage required for polyadenylation
- termination of RNA polymerase II transcription
- tRNA splicing, via endonucleolytic cleavage and ligation

---

28

- **Protein name:** DNA damage-binding protein 1
- **Organism:** Homo sapiens
- **Uniprot Accession Number:** Q16531
- **Protein sequence length:** 1140 aa
- **1D identity (%):** 15.51
- **1D identity (%) [Gaps excluded]:** 27.41
- **1D identity - Alignment Gaps:** 669
- **Common reported functions (%):** 0.0
- **Common reported locations (%):** 0.0
- **Common reported processes (%):** 0.0

- **PDB ID:** 5JK7
- **Chain:** B
- **Crystallized protein length:** 1133 aa
- **Resolution:** 3.49 Å
- **b-phipsi:** 0.011142
- **w-rdist:** 1.263002
- **t-alpha:** 0.045724
- **Chemical similarity (Tanimoto Index) (%):** N/A
- **1D identity (%) [PDB]:** 0.05
- **1D identity (%) [Gaps excluded][PDB]:** 50.0
- **1D identity - Alignment Gaps [PDB]:** 2171
- **2D identity (%) [PDB]:** 32.76
- **2D identity (%) [Gaps excluded][PDB]:** 85.41
- **2D identity - Alignment Gaps [PDB]:** 969
- **3D similarity (TM-Score) (%) [PDB]:** 19.53

- **Gene name:** DDB1
- **RefSeq ID:** NM\_001923
- **Transcript sequence length:** 4245
- **5-UTR|CDS|3-UTR identity (%):** 27.05 | 40.25 | 20.28
- **5-UTR|CDS|3-UTR identity (%) [Gaps excluded]:** 71.82 | 77.47 | 75.26
- **5-UTR|CDS|3-UTR identity [Alignment Gaps]:** 182 | 2291 | 526

**Uniprot Description:**  
  
Protein, which is both involved in DNA repair and protein ubiquitination, as part of the UV-DDB complex and DCX (DDB1-CUL4-X-box) complexes, respectively (PubMed:15448697, PubMed:14739464, PubMed:16260596, PubMed:16482215, PubMed:17079684, PubMed:16407242, PubMed:16407252, PubMed:16940174). Core component of the UV-DDB complex (UV-damaged DNA-binding protein complex), a complex that recognizes UV-induced DNA damage and recruit proteins of the nucleotide excision repair pathway (the NER pathway) to initiate DNA repair (PubMed:15448697, PubMed:16260596, PubMed:16407242, PubMed:16940174). The UV-DDB complex preferentially binds to cyclobutane pyrimidine dimers (CPD), 6-4 photoproducts (6-4 PP), apurinic sites and short mismatches (PubMed:15448697, PubMed:16260596, PubMed:16407242, PubMed:16940174). Also functions as a component of numerous distinct DCX (DDB1-CUL4-X-box) E3 ubiquitin-protein ligase complexes which mediate the ubiquitination and subsequent proteasomal degradation of target proteins (PubMed:14739464, PubMed:16407252, PubMed:16482215, PubMed:17079684, PubMed:25043012, PubMed:25108355, PubMed:18332868, PubMed:18381890, PubMed:19966799, PubMed:22118460, PubMed:28886238). The functional specificity of the DCX E3 ubiquitin-protein ligase complex is determined by the variable substrate recognition component recruited by DDB1 (PubMed:14739464, PubMed:16407252, PubMed:16482215, PubMed:17079684, PubMed:25043012, PubMed:25108355, PubMed:18332868, PubMed:18381890, PubMed:19966799, PubMed:22118460). DCX(DDB2) (also known as DDB1-CUL4-ROC1, CUL4-DDB-ROC1 and CUL4-DDB-RBX1) may ubiquitinate histone H2A, histone H3 and histone H4 at sites of UV-induced DNA damage (PubMed:16678110, PubMed:17041588, PubMed:16473935, PubMed:18593899). The ubiquitination of histones may facilitate their removal from the nucleosome and promote subsequent DNA repair (PubMed:16678110, PubMed:17041588, PubMed:16473935, PubMed:18593899). DCX(DDB2) also ubiquitinates XPC, which may enhance DNA-binding by XPC and promote NER (PubMed:15882621). DCX(DTL) plays a role in PCNA-dependent polyubiquitination of CDT1 and MDM2-dependent ubiquitination of TP53 in response to radiation-induced DNA damage and during DNA replication (PubMed:17041588). DCX(ERCC8) (the CSA complex) plays a role in transcription-coupled repair (TCR) (PubMed:12732143). The DDB1-CUL4A-DTL E3 ligase complex regulates the circadian clock function by mediating the ubiquitination and degradation of CRY1 (PubMed:26431207). DDB1-mediated CRY1 degradation promotes FOXO1 protein stability and FOXO1-mediated gluconeogenesis in the liver (By similarity).  
  
Component of the UV-DDB complex which includes DDB1 and DDB2; the heterodimer dimerizes to give rise to a heterotetramer when bound to damaged DNA (PubMed:9632823, PubMed:16223728, PubMed:16527807, PubMed:19109893, PubMed:22822215). The UV-DDB complex interacts with monoubiquitinated histone H2A and binds to XPC via the DDB2 subunit (PubMed:16473935). Component of numerous DCX (DDB1-CUL4-X-box) E3 ubiquitin-protein ligase complexes which consist of a core of DDB1, CUL4A or CUL4B and RBX1 (PubMed:11673459, PubMed:12732143, PubMed:15882621, PubMed:16678110, PubMed:18593899, PubMed:28886238, PubMed:28437394, PubMed:28302793, PubMed:31693891, PubMed:31686031, PubMed:31819272, PubMed:31693911). DDB1 may recruit specific substrate targeting subunits to the DCX complex (PubMed:11673459, PubMed:12732143, PubMed:15882621, PubMed:18593899, PubMed:28886238). These substrate targeting subunits are generally known as DCAF (DDB1- and CUL4-associated factor) or CDW (CUL4-DDB1-associated WD40-repeat) proteins (PubMed:17079684, PubMed:16949367, PubMed:18606781, PubMed:19608861, PubMed:16964240, PubMed:19966799). Interacts with AMBRA1, ATG16L1, BTRC, CRBN, DCAF1, DCAF4, DCAF5, DCAF6, DCAF7, DCAF8, DCAF9, DCAF10, DCAF11, DCAF12, DCAF15, DCAF16, DCAF17, DDA1, DET1, DTL, ERCC8, FBXW5, FBXW8, GRWD1, KATNB1, NLE1, NUP43, PAFAH1B1, PHIP, PWP1, RBBP4, RBBP5, RBBP7, COP1, SNRNP40, DCAF1, WDR5, WDR5B, WDR12, WDR26, WDR39, WDR42, WDR53, WDR59, WDR61, WSB1, WSB2, LRWD1 and WDTC1 (PubMed:14739464, PubMed:17079684, PubMed:16949367, PubMed:17041588, PubMed:18606781, PubMed:22935713, PubMed:23478445, PubMed:22118460, PubMed:25043012, PubMed:25108355). DCX complexes may associate with the COP9 signalosome, and this inhibits the E3 ubiquitin-protein ligase activity of the complex (PubMed:15448697, PubMed:16260596). Interacts with NF2, TSC1 and TSC2 (PubMed:18332868, PubMed:18381890). Interacts with AGO1 and AGO2 (PubMed:17932509). Associates with the E3 ligase complex containing DYRK2, EDD/UBR5, DDB1 and DCAF1 proteins (EDVP complex) (PubMed:19287380). Interacts directly with DYRK2 (PubMed:19287380). DCX(DTL) complex interacts with FBXO11; does not ubiquitinate and degradate FBXO11 (PubMed:19287380). Interacts with TRPC4AP (PubMed:19966799). Interacts with CRY1 and CRY2 (By similarity). The DDB1-CUL4A complex interacts with CRY1 (PubMed:26431207). May also interact with DCUN1D1, DCUN1D2, DCUN1D3 and DCUN1D5 (PubMed:26906416).  
  
**Gene Ontology Information:**

Molecular Function

- cullin family protein binding
- damaged DNA binding
- DNA binding
- protein-containing complex binding
- protein-macromolecule adaptor activity
- WD40-repeat domain binding

Location

- chromosome, telomeric region
- Cul4-RING E3 ubiquitin ligase complex
- Cul4A-RING E3 ubiquitin ligase complex
- Cul4B-RING E3 ubiquitin ligase complex
- cytoplasm
- extracellular exosome
- extracellular space
- nucleoplasm
- nucleus
- protein-containing complex
- site of double-strand break

Biological process

- biological process involved in interaction with symbiont
- cellular response to DNA damage stimulus
- DNA damage response, detection of DNA damage
- DNA repair
- global genome nucleotide-excision repair
- histone H2A monoubiquitination
- nucleotide-excision repair
- nucleotide-excision repair, DNA damage recognition
- nucleotide-excision repair, DNA duplex unwinding
- nucleotide-excision repair, DNA incision
- nucleotide-excision repair, DNA incision, 3'-to lesion
- nucleotide-excision repair, DNA incision, 5'-to lesion
- nucleotide-excision repair, preincision complex assembly
- nucleotide-excision repair, preincision complex stabilization
- positive regulation by virus of viral protein levels in host cell
- positive regulation of gluconeogenesis
- positive regulation of protein catabolic process
- positive regulation of viral genome replication
- positive regulation of viral release from host cell
- post-translational protein modification
- proteasomal protein catabolic process
- proteasome-mediated ubiquitin-dependent protein catabolic process
- protein ubiquitination
- regulation of mitotic cell cycle phase transition
- rhythmic process
- transcription-coupled nucleotide-excision repair
- ubiquitin-dependent protein catabolic process
- UV-damage excision repair
- viral process

---

29

- **Protein name:** Endolysin
- **Organism:** Enterobacteria phage T4
- **Uniprot Accession Number:** P00720
- **Protein sequence length:** 164 aa
- **1D identity (%):** 3.27
- **1D identity (%) [Gaps excluded]:** 27.81
- **1D identity - Alignment Gaps:** 1135
- **Common reported functions (%):** 0.0
- **Common reported locations (%):** 0.0
- **Common reported processes (%):** 0.0

- **PDB ID:** 6QAJ
- **Chain:** A
- **Crystallized protein length:** 439 aa
- **Resolution:** 2.9 Å
- **b-phipsi:** 0.071431
- **w-rdist:** 0.968947
- **t-alpha:** 0.006478
- **Chemical similarity (Tanimoto Index) (%):** 78.61
- **1D identity (%) [PDB]:** 0.0
- **1D identity (%) [Gaps excluded][PDB]:** 0.0
- **1D identity - Alignment Gaps [PDB]:** 1369
- **2D identity (%) [PDB]:** 17.2
- **2D identity (%) [Gaps excluded][PDB]:** 90.83
- **2D identity - Alignment Gaps [PDB]:** 933
- **3D similarity (TM-Score) (%) [PDB]:** 11.16

- **Gene name:** E
- **RefSeq ID:** NC\_000866
- **Genomic sequence length:** 168903
- **5-UTR|CDS|3-UTR identity (%):** N/A | 9.19 | N/A
- **5-UTR|CDS|3-UTR identity (%) [Gaps excluded]:** N/A | 80.18 | N/A
- **5-UTR|CDS|3-UTR identity [Alignment Gaps]:** N/A | 3429 | N/A

**Uniprot Description:**  
  
Endolysin with lysozyme activity that degrades host peptidoglycans and participates with the holin and spanin proteins in the sequential events which lead to the programmed host cell lysis releasing the mature viral particles. Once the holin has permeabilized the host cell membrane, the endolysin can reach the periplasm and break down the peptidoglycan layer.  
  
**Gene Ontology Information:**

Molecular Function

- lysozyme activity

Location

- host cell cytoplasm

Biological process

- cell wall macromolecule catabolic process
- cytolysis
- defense response to bacterium
- peptidoglycan catabolic process
- viral release from host cell by cytolysis

---

30

- **Protein name:** Small nuclear ribonucleoprotein Sm D2
- **Organism:** Homo sapiens
- **Uniprot Accession Number:** P62316
- **Protein sequence length:** 118 aa
- **1D identity (%):** 2.18
- **1D identity (%) [Gaps excluded]:** 26.92
- **1D identity - Alignment Gaps:** 1183
- **Common reported functions (%):** 0.0
- **Common reported locations (%):** 0.0
- **Common reported processes (%):** 0.0

- **PDB ID:** 5Z58
- **Chain:** D
- **Crystallized protein length:** 1722 aa
- **Resolution:** 4.9 Å
- **b-phipsi:** 0.026414
- **w-rdist:** 0.89961
- **t-alpha:** 0.091954
- **Chemical similarity (Tanimoto Index) (%):** 32.03
- **1D identity (%) [PDB]:** 0.0
- **1D identity (%) [Gaps excluded][PDB]:** 0.0
- **1D identity - Alignment Gaps [PDB]:** 2762
- **2D identity (%) [PDB]:** N/A
- **2D identity (%) [Gaps excluded][PDB]:** N/A
- **2D identity - Alignment Gaps [PDB]:** N/A
- **3D similarity (TM-Score) (%) [PDB]:** 5.0

- **Gene name:** SNRPD2
- **RefSeq ID:** NM\_177542
- **Transcript sequence length:** 689
- **5-UTR|CDS|3-UTR identity (%):** 36.89 | 6.22 | 35.0
- **5-UTR|CDS|3-UTR identity (%) [Gaps excluded]:** 76.65 | 77.35 | 82.35
- **5-UTR|CDS|3-UTR identity [Alignment Gaps]:** 180 | 3531 | 138

**Uniprot Description:**  
  
Plays role in pre-mRNA splicing as core component of the SMN-Sm complex that mediates spliceosomal snRNP assembly and as component of the spliceosomal U1, U2, U4 and U5 small nuclear ribonucleoproteins (snRNPs), the building blocks of the spliceosome (PubMed:11991638, PubMed:18984161, PubMed:19325628, PubMed:23333303, PubMed:25555158, PubMed:26912367, PubMed:28502770, PubMed:28781166, PubMed:28076346). Component of both the pre-catalytic spliceosome B complex and activated spliceosome C complexes (PubMed:11991638, PubMed:28502770, PubMed:28781166, PubMed:28076346). Is also a component of the minor U12 spliceosome (PubMed:15146077).  
  
Core component of the spliceosomal U1, U2, U4 and U5 small nuclear ribonucleoproteins (snRNPs), the building blocks of the spliceosome (PubMed:11991638, PubMed:19325628, PubMed:21516107, PubMed:25555158, PubMed:26912367, PubMed:28502770, PubMed:28781166, PubMed:28076346). Most spliceosomal snRNPs contain a common set of Sm proteins, SNRPB, SNRPD1, SNRPD2, SNRPD3, SNRPE, SNRPF and SNRPG that assemble in a heptameric protein ring on the Sm site of the small nuclear RNA to form the core snRNP (PubMed:10025403, PubMed:19325628, PubMed:21516107, PubMed:25555158, PubMed:26912367, PubMed:28502770, PubMed:28781166, PubMed:28076346). Component of the U1 snRNP (PubMed:19325628, PubMed:25555158). The U1 snRNP is composed of the U1 snRNA and the 7 core Sm proteins SNRPB, SNRPD1, SNRPD2, SNRPD3, SNRPE, SNRPF and SNRPG, and at least three U1 snRNP-specific proteins SNRNP70/U1-70K, SNRPA/U1-A and SNRPC/U1-C (PubMed:19325628, PubMed:25555158). Component of the U4/U6-U5 tri-snRNP complex composed of the U4, U6 and U5 snRNAs and at least PRPF3, PRPF4, PRPF6, PRPF8, PRPF31, SNRNP200, TXNL4A, SNRNP40, SNRPB, SNRPD1, SNRPD2, SNRPD3, SNRPE, SNRPF, SNRPG, DDX23, CD2BP2, PPIH, SNU13, EFTUD2, SART1 and USP39, plus LSM2, LSM3, LSM4, LSM5, LSM6, LSM7 and LSM8 (PubMed:26912367). Component of the U11/U12 snRNPs that are part of the U12-type spliceosome (PubMed:15146077). Part of the SMN-Sm complex that contains SMN1, GEMIN2/SIP1, DDX20/GEMIN3, GEMIN4, GEMIN5, GEMIN6, GEMIN7, GEMIN8, STRAP/UNRIP and the Sm proteins SNRPB, SNRPD1, SNRPD2, SNRPD3, SNRPE, SNRPF and SNRPG; catalyzes core snRNPs assembly. Forms a 6S pICln-Sm complex composed of CLNS1A/pICln, SNRPD1, SNRPD2, SNRPE, SNRPF and SNRPG; ring-like structure where CLNS1A/pICln mimics additional Sm proteins and which is unable to assemble into the core snRNP.  
  
**Gene Ontology Information:**

Molecular Function

- RNA binding
- U1 snRNP binding

Location

- catalytic step 2 spliceosome
- cytosol
- extracellular exosome
- methylosome
- nucleoplasm
- nucleus
- pICln-Sm protein complex
- precatalytic spliceosome
- small nuclear ribonucleoprotein complex
- SMN-Sm protein complex
- spliceosomal complex
- U1 snRNP
- U12-type spliceosomal complex
- U2 snRNP
- U2-type catalytic step 2 spliceosome
- U2-type precatalytic spliceosome
- U4 snRNP
- U4/U6 x U5 tri-snRNP complex
- U5 snRNP

Biological process

- import into nucleus
- mRNA splicing, via spliceosome
- RNA splicing
- spliceosomal complex assembly
- spliceosomal snRNP assembly

---

31

- **Protein name:** Dipeptidyl peptidase 4
- **Organism:** Homo sapiens
- **Uniprot Accession Number:** P27487
- **Protein sequence length:** 766 aa
- **1D identity (%):** 7.63
- **1D identity (%) [Gaps excluded]:** 28.87
- **1D identity - Alignment Gaps:** 1187
- **Common reported functions (%):** 50.0
- **Common reported locations (%):** 20.0
- **Common reported processes (%):** 0.0

- **PDB ID:** 4L72
- **Chain:** A
- **Crystallized protein length:** 728 aa
- **Resolution:** 3.0 Å
- **b-phipsi:** 0.002035
- **w-rdist:** 1.429925
- **t-alpha:** 0.064655
- **Chemical similarity (Tanimoto Index) (%):** 94.67
- **1D identity (%) [PDB]:** 0.06
- **1D identity (%) [Gaps excluded][PDB]:** 100.0
- **1D identity - Alignment Gaps [PDB]:** 1766
- **2D identity (%) [PDB]:** 33.36
- **2D identity (%) [Gaps excluded][PDB]:** 85.31
- **2D identity - Alignment Gaps [PDB]:** 774
- **3D similarity (TM-Score) (%) [PDB]:** 16.43

- **Gene name:** DPP4
- **RefSeq ID:** NM\_001935
- **Transcript sequence length:** 3573
- **5-UTR|CDS|3-UTR identity (%):** 29.75 | 38.54 | 15.66
- **5-UTR|CDS|3-UTR identity (%) [Gaps excluded]:** 71.92 | 78.23 | 80.19
- **5-UTR|CDS|3-UTR identity [Alignment Gaps]:** 207 | 2081 | 853

**Uniprot Description:**  
  
Cell surface glycoprotein receptor involved in the costimulatory signal essential for T-cell receptor (TCR)-mediated T-cell activation (PubMed:10951221, PubMed:10900005, PubMed:11772392, PubMed:17287217). Acts as a positive regulator of T-cell coactivation, by binding at least ADA, CAV1, IGF2R, and PTPRC (PubMed:10951221, PubMed:10900005, PubMed:11772392, PubMed:14691230). Its binding to CAV1 and CARD11 induces T-cell proliferation and NF-kappa-B activation in a T-cell receptor/CD3-dependent manner (PubMed:17287217). Its interaction with ADA also regulates lymphocyte-epithelial cell adhesion (PubMed:11772392). In association with FAP is involved in the pericellular proteolysis of the extracellular matrix (ECM), the migration and invasion of endothelial cells into the ECM (PubMed:16651416, PubMed:10593948). May be involved in the promotion of lymphatic endothelial cells adhesion, migration and tube formation (PubMed:18708048). When overexpressed, enhanced cell proliferation, a process inhibited by GPC3 (PubMed:17549790). Acts also as a serine exopeptidase with a dipeptidyl peptidase activity that regulates various physiological processes by cleaving peptides in the circulation, including many chemokines, mitogenic growth factors, neuropeptides and peptide hormones such as brain natriuretic peptide 32 (PubMed:16254193, PubMed:10570924). Removes N-terminal dipeptides sequentially from polypeptides having unsubstituted N-termini provided that the penultimate residue is proline (PubMed:10593948).  
  
Monomer. Homodimer (PubMed:12832764, PubMed:15448155, PubMed:17287217, PubMed:12646248, PubMed:12483204, PubMed:12906826). Heterodimer with Seprase (FAP) (PubMed:16651416). Requires homodimerization for optimal dipeptidyl peptidase activity and T-cell costimulation. Found in a membrane raft complex, at least composed of BCL10, CARD11, DPP4 and IKBKB (PubMed:17287217). Associates with collagen (PubMed:8526932). Interacts with PTPRC; the interaction is enhanced in an interleukin-12-dependent manner in activated lymphocytes (PubMed:12676959). Interacts (via extracellular domain) with ADA; does not inhibit its dipeptidyl peptidase activity (PubMed:15016824, PubMed:10951221, PubMed:14691230, PubMed:7907293, PubMed:8101391). Interacts with CAV1 (via the N-terminus); the interaction is direct (PubMed:17287217). Interacts (via cytoplasmic tail) with CARD11 (via PDZ domain); its homodimerization is necessary for interaction with CARD11 (PubMed:17287217). Interacts with IGF2R; the interaction is direct (PubMed:10900005). Interacts with GPC3 (PubMed:17549790). Interacts with human coronavirus-EMC spike protein and acts as a receptor for this virus (PubMed:23486063).  
  
**Gene Ontology Information:**

Molecular Function

- aminopeptidase activity
- chemorepellent activity
- dipeptidyl-peptidase activity
- identical protein binding
- protease binding
- protein homodimerization activity
- serine-type endopeptidase activity
- serine-type peptidase activity
- signaling receptor binding
- virus receptor activity

Location

- apical plasma membrane
- cell surface
- endocytic vesicle
- extracellular exosome
- extracellular region
- focal adhesion
- integral component of membrane
- intercellular canaliculus
- lamellipodium
- lamellipodium membrane
- lysosomal membrane
- membrane
- membrane raft
- plasma membrane

Biological process

- behavioral fear response
- cell adhesion
- endothelial cell migration
- locomotory exploration behavior
- negative regulation of extracellular matrix disassembly
- negative regulation of neutrophil chemotaxis
- positive regulation of cell population proliferation
- proteolysis
- psychomotor behavior
- regulation of cell-cell adhesion mediated by integrin
- regulation of insulin secretion
- response to hypoxia
- T cell activation
- T cell costimulation

---

32

- **Protein name:** RecBCD enzyme subunit RecC
- **Organism:** Escherichia coli (strain K12)
- **Uniprot Accession Number:** P07648
- **Protein sequence length:** 1122 aa
- **1D identity (%):** 13.31
- **1D identity (%) [Gaps excluded]:** 29.2
- **1D identity - Alignment Gaps:** 895
- **Common reported functions (%):** 0.0
- **Common reported locations (%):** 0.0
- **Common reported processes (%):** 0.0

- **PDB ID:** 5MBV
- **Chain:** C
- **Crystallized protein length:** 1121 aa
- **Resolution:** 3.8 Å
- **b-phipsi:** 0.034605
- **w-rdist:** 0.932265
- **t-alpha:** 0.05587
- **Chemical similarity (Tanimoto Index) (%):** 83.9
- **1D identity (%) [PDB]:** 0.05
- **1D identity (%) [Gaps excluded][PDB]:** 100.0
- **1D identity - Alignment Gaps [PDB]:** 2159
- **2D identity (%) [PDB]:** 26.78
- **2D identity (%) [Gaps excluded][PDB]:** 91.8
- **2D identity - Alignment Gaps [PDB]:** 1185
- **3D similarity (TM-Score) (%) [PDB]:** 17.1

- **Gene name:** recC
- **RefSeq ID:** N/A
- **Sequence length:** N/A
- **5-UTR|CDS|3-UTR identity (%):** N/A | N/A | N/A
- **5-UTR|CDS|3-UTR identity (%) [Gaps excluded]:** N/A | N/A | N/A
- **5-UTR|CDS|3-UTR identity [Alignment Gaps]:** N/A | N/A | N/A

**Uniprot Description:**  
  
A helicase/nuclease that prepares dsDNA breaks (DSB) for recombinational DNA repair. Binds to DSBs and unwinds DNA via a rapid (>1 kb/second) and highly processive (>30 kb) ATP-dependent bidirectional helicase. Unwinds dsDNA until it encounters a Chi (crossover hotspot instigator, 5'-GCTGGTGG-3') sequence from the 3' direction. Cuts ssDNA a few nucleotides 3' to Chi site, by nicking one strand or switching the strand degraded (depending on the reaction conditions). The properties and activities of the enzyme are changed at Chi. The Chi-altered holoenzyme produces a long 3'-ssDNA overhang which facilitates RecA-binding to the ssDNA for homologous DNA recombination and repair. Holoenzyme degrades any linearized DNA that is unable to undergo homologous recombination (PubMed:4562392, PubMed:4552016, PubMed:123277). In the holoenzyme this subunit almost certainly recognizes the wild-type Chi sequence, when added to isolated RecB increases its ATP-dependent helicase processivity. The RecBC complex requires the RecD subunit for nuclease activity, but can translocate along ssDNA in both directions.  
  
Heterotrimer of RecB, RecC and RecD. All subunits contribute to DNA-binding. Interacts with YgbT (Cas1) (PubMed:21219465).  
  
**Gene Ontology Information:**

Molecular Function

- ATP binding
- DNA binding
- DNA helicase activity
- exodeoxyribonuclease V activity

Location

- exodeoxyribonuclease V complex

Biological process

- clearance of foreign intracellular DNA
- DNA recombination
- double-strand break repair via homologous recombination
- response to radiation

---

33

- **Protein name:** T7 RNA polymerase
- **Organism:** Escherichia phage T7
- **Uniprot Accession Number:** P00573
- **Protein sequence length:** 883 aa
- **1D identity (%):** 14.33
- **1D identity (%) [Gaps excluded]:** 26.32
- **1D identity - Alignment Gaps:** 636
- **Common reported functions (%):** 0.0
- **Common reported locations (%):** 0.0
- **Common reported processes (%):** 0.0

- **PDB ID:** 1MSW
- **Chain:** D
- **Crystallized protein length:** 863 aa
- **Resolution:** 2.1 Å
- **b-phipsi:** 0.055153
- **w-rdist:** 1.314775
- **t-alpha:** 0.0
- **Chemical similarity (Tanimoto Index) (%):** 83.86
- **1D identity (%) [PDB]:** 0.05
- **1D identity (%) [Gaps excluded][PDB]:** 50.0
- **1D identity - Alignment Gaps [PDB]:** 1901
- **2D identity (%) [PDB]:** 18.19
- **2D identity (%) [Gaps excluded][PDB]:** 89.44
- **2D identity - Alignment Gaps [PDB]:** 1261
- **3D similarity (TM-Score) (%) [PDB]:** 18.78

- **Gene name:** 1
- **RefSeq ID:** NC\_001604
- **Genomic sequence length:** 39937
- **5-UTR|CDS|3-UTR identity (%):** N/A | 38.21 | N/A
- **5-UTR|CDS|3-UTR identity (%) [Gaps excluded]:** N/A | 78.09 | N/A
- **5-UTR|CDS|3-UTR identity [Alignment Gaps]:** N/A | 2220 | N/A

**Uniprot Description:**  
  
Highly processive DNA-dependent RNA polymerase that catalyzes the transcription of class II and class III viral genes. Recognizes a specific promoter sequence and enters first into an 'abortive phase' where very short transcripts are synthesized and released before proceeding to the processive transcription of long RNA chains. Unwinds the double-stranded DNA to expose the coding strand for templating. Participates in the initiation of viral DNA replication presumably by making primers accessible to the DNA polymerase, thus facilitating the DNA opening. Plays also a role in viral DNA packaging, probably by pausing the transcription at the right end of concatemer junction to allow packaging complex recruitment and beginning of the packaging process.  
  
Monomer. Interacts with T7 lysozyme; this interaction inhibits transcriptional function of T7 RNA polymerase.  
  
**Gene Ontology Information:**

Molecular Function

- DNA binding
- DNA-directed 5'-3' RNA polymerase activity

Location  
  
N/A

Biological process

- DNA-templated viral transcription
- transcription, DNA-templated

---

34

- **Protein name:** Baseplate wedge protein gp7
- **Organism:** Enterobacteria phage T4
- **Uniprot Accession Number:** P19061
- **Protein sequence length:** 1032 aa
- **1D identity (%):** 10.1
- **1D identity (%) [Gaps excluded]:** 26.74
- **1D identity - Alignment Gaps:** 1041
- **Common reported functions (%):** 0.0
- **Common reported locations (%):** 0.0
- **Common reported processes (%):** 0.0

- **PDB ID:** 5HX2
- **Chain:** A
- **Crystallized protein length:** 1030 aa
- **Resolution:** 3.8 Å
- **b-phipsi:** 0.053932
- **w-rdist:** 1.042223
- **t-alpha:** 0.018137
- **Chemical similarity (Tanimoto Index) (%):** N/A
- **1D identity (%) [PDB]:** 0.0
- **1D identity (%) [Gaps excluded][PDB]:** 0.0
- **1D identity - Alignment Gaps [PDB]:** 2070
- **2D identity (%) [PDB]:** 29.26
- **2D identity (%) [Gaps excluded][PDB]:** 86.1
- **2D identity - Alignment Gaps [PDB]:** 1020
- **3D similarity (TM-Score) (%) [PDB]:** 17.57

- **Gene name:** 7
- **RefSeq ID:** NC\_000866
- **Genomic sequence length:** 168903
- **5-UTR|CDS|3-UTR identity (%):** N/A | 42.06 | N/A
- **5-UTR|CDS|3-UTR identity (%) [Gaps excluded]:** N/A | 79.42 | N/A
- **5-UTR|CDS|3-UTR identity [Alignment Gaps]:** N/A | 2129 | N/A

**Uniprot Description:**  
  
Intermediate/inner baseplate protein (PubMed:27193680, PubMed:15315755). The gp25-(gp6)2-gp7 module is involved in sheath contraction (PubMed:27193680). Involved in the tail assembly (PubMed:21129200).  
  
Heterotrimer with gp6; assembles as a (gp6)2-gp7 heterotrimeric molecule. The (gp6)2-gp7 heterotrimeric molecule further interacts with gp25 and gp53. The gp25-(gp6)2-gp7 module is involved in sheath contraction. Interacts with gp8. Binds to gp10 homotrimer; disulfide-linked. Heteromultimer with gp10; a gp10 molecule is disulfide-linked to gp7 and the other two remaining gp10 molecules form a disulfide bond. Part of the baseplate macromolecular complex which consists of gp5, gp5.4, gp27 (central spike complex); gp6, gp25, gp53 (inner baseplate); gp7, gp8 (intermediate baseplate); gp9, gp10, gp11, gp12 (peripheral); gp48 and gp54 (proximal region of the tail tube).  
  
**Gene Ontology Information:**

Molecular Function  
  
N/A

Location

- virion
- virus tail, baseplate

Biological process

- viral tail assembly

---

35

- **Protein name:** Hexon protein
- **Organism:** Fowl adenovirus A serotype 1 (strain CELO / Phelps)
- **Uniprot Accession Number:** P42671
- **Protein sequence length:** 942 aa
- **1D identity (%):** 13.84
- **1D identity (%) [Gaps excluded]:** 30.09
- **1D identity - Alignment Gaps:** 819
- **Common reported functions (%):** 0.0
- **Common reported locations (%):** 0.0
- **Common reported processes (%):** 14.29

- **PDB ID:** 2INY
- **Chain:** A
- **Crystallized protein length:** 941 aa
- **Resolution:** 3.9 Å
- **b-phipsi:** 0.054789
- **w-rdist:** 1.113888
- **t-alpha:** 0.015625
- **Chemical similarity (Tanimoto Index) (%):** 83.78
- **1D identity (%) [PDB]:** 0.1
- **1D identity (%) [Gaps excluded][PDB]:** 66.67
- **1D identity - Alignment Gaps [PDB]:** 1975
- **2D identity (%) [PDB]:** 22.86
- **2D identity (%) [Gaps excluded][PDB]:** 85.2
- **2D identity - Alignment Gaps [PDB]:** 1143
- **3D similarity (TM-Score) (%) [PDB]:** 21.34

- **Gene name:** L3
- **RefSeq ID:** NC\_001720
- **Genomic sequence length:** 43804
- **5-UTR|CDS|3-UTR identity (%):** N/A | 10.77 | N/A
- **5-UTR|CDS|3-UTR identity (%) [Gaps excluded]:** N/A | 77.35 | N/A
- **5-UTR|CDS|3-UTR identity [Alignment Gaps]:** N/A | 3357 | N/A

**Uniprot Description:**  
  
Major capsid protein that self-associates to form 240 hexon trimers, each in the shape of a hexagon, building most of the pseudo T=25 capsid. Assembled into trimeric units with the help of the chaperone shutoff protein. Transported by pre-protein VI to the nucleus where it associates with other structural proteins to form an empty capsid. Might be involved, through its interaction with host dyneins, in the intracellular microtubule-dependent transport of incoming viral capsid to the nucleus.  
  
Homotrimer. Interacts with the capsid vertex protein; this interaction binds the peripentonal hexons to the neighboring penton base. Interacts with the hexon-linking protein; this interaction tethers the hexons surrounding the penton to those situated in the central plate of the facet. Interacts with the hexon-interlacing protein; this interaction lashes the hexons together. Interacts with host dyneins DYNC1LI1 and DYNC1I2; this interaction might be involved in intracellular microtubule-dependent transport of incoming viral capsid. Interacts with the shutoff protein; this interaction allows folding and formation of hexons trimers. Interacts with pre-protein VI; this interaction probably allows nuclear import of hexon trimers and possibly pre-capsid assembly.  
  
**Gene Ontology Information:**

Molecular Function

- structural molecule activity

Location

- host cell nucleus
- T=25 icosahedral viral capsid

Biological process

- microtubule-dependent intracellular transport of viral material towards nucleus
- viral entry into host cell

---

36

- **Protein name:** Interferon-induced helicase C domain-containing protein 1
- **Organism:** Mus musculus
- **Uniprot Accession Number:** Q8R5F7
- **Protein sequence length:** 1025 aa
- **1D identity (%):** 6.88
- **1D identity (%) [Gaps excluded]:** 26.98
- **1D identity - Alignment Gaps:** 1364
- **Common reported functions (%):** 50.0
- **Common reported locations (%):** 0.0
- **Common reported processes (%):** 0.0

- **PDB ID:** 6G19
- **Chain:** A
- **Crystallized protein length:** 682 aa
- **Resolution:** 3.68 Å
- **b-phipsi:** 0.033201
- **w-rdist:** 1.382629
- **t-alpha:** 0.00081
- **Chemical similarity (Tanimoto Index) (%):** 83.77
- **1D identity (%) [PDB]:** 0.29
- **1D identity (%) [Gaps excluded][PDB]:** 71.43
- **1D identity - Alignment Gaps [PDB]:** 1711
- **2D identity (%) [PDB]:** 27.9
- **2D identity (%) [Gaps excluded][PDB]:** 88.62
- **2D identity - Alignment Gaps [PDB]:** 899
- **3D similarity (TM-Score) (%) [PDB]:** 14.98

- **Gene name:** Ifih1
- **RefSeq ID:** N/A
- **Sequence length:** N/A
- **5-UTR|CDS|3-UTR identity (%):** N/A | N/A | N/A
- **5-UTR|CDS|3-UTR identity (%) [Gaps excluded]:** N/A | N/A | N/A
- **5-UTR|CDS|3-UTR identity [Alignment Gaps]:** N/A | N/A | N/A

**Uniprot Description:**  
  
Innate immune receptor which acts as a cytoplasmic sensor of viral nucleic acids and plays a major role in sensing viral infection and in the activation of a cascade of antiviral responses including the induction of type I interferons and proinflammatory cytokines. Its ligands include mRNA lacking 2'-O-methylation at their 5' cap and long-dsRNA (>1 kb in length). Upon ligand binding it associates with mitochondria antiviral signaling protein (MAVS/IPS1) which activates the IKK-related kinases: TBK1 and IKBKE which phosphorylate interferon regulatory factors: IRF3 and IRF7 which in turn activate transcription of antiviral immunological genes, including interferons (IFNs); IFN-alpha and IFN-beta. Responsible for detecting the Picornaviridae family members such as encephalomyocarditis virus (EMCV), mengo encephalomyocarditis virus (ENMG), and theiler's murine encephalomyelitis virus (TMEV). Can also detect other viruses such as dengue virus (DENV), west Nile virus (WNV), and reovirus. Also involved in antiviral signaling in response to viruses containing a dsDNA genome, such as vaccinia virus. Plays an important role in amplifying innate immune signaling through recognition of RNA metabolites that are produced during virus infection by ribonuclease L (RNase L). May play an important role in enhancing natural killer cell function and may be involved in growth inhibition and apoptosis in several tumor cell lines.  
  
Monomer in the absence of ligands and homodimerizes in the presence of dsRNA ligands. Can assemble into helical or linear polymeric filaments on long dsRNA. Interacts with MAVS/IPS1. Interacts with PCBP2. Interacts with NLRC5. Interacts with PIAS2-beta. Interacts with DDX60. Interacts with ANKRD17. Interacts with IKBKE. Interacts (via the CARD domains) with TKFC, the interaction is inhibited by viral infection (By similarity). Interacts with ATG5 and ATG12, either as ATG5 and ATG12 monomers or as ATG12-ATG5 conjugates (By similarity). Interacts with ZCCHC3; leading to activate IFIH1/MDA5 (By similarity). Interacts with RNF123 (By similarity). Interacts with DDX3X (By similarity).  
  
**Gene Ontology Information:**

Molecular Function

- ATP binding
- DNA binding
- double-stranded RNA binding
- hydrolase activity
- identical protein binding
- ribonucleoprotein complex binding
- RNA helicase activity
- single-stranded RNA binding
- zinc ion binding

Location

- cytoplasm
- nucleus

Biological process

- cellular response to exogenous dsRNA
- defense response to virus
- innate immune response
- MDA-5 signaling pathway
- positive regulation of interferon-alpha production
- positive regulation of interferon-beta production
- positive regulation of interleukin-6 production
- positive regulation of response to cytokine stimulus
- positive regulation of tumor necrosis factor production
- protein sumoylation
- response to virus

---

37

- **Protein name:** Gag-Pol polyprotein
- **Organism:** Human immunodeficiency virus type 1 group M subtype B (isolate HXB2)
- **Uniprot Accession Number:** P04585
- **Protein sequence length:** 1435 aa
- **1D identity (%):** 12.98
- **1D identity (%) [Gaps excluded]:** 28.67
- **1D identity - Alignment Gaps:** 1020
- **Common reported functions (%):** 50.0
- **Common reported locations (%):** 40.0
- **Common reported processes (%):** 14.29

- **PDB ID:** 6BSJ
- **Chain:** A
- **Crystallized protein length:** 543 aa
- **Resolution:** 2.89 Å
- **b-phipsi:** 0.007528
- **w-rdist:** 1.282844
- **t-alpha:** 0.083333
- **Chemical similarity (Tanimoto Index) (%):** 81.17
- **1D identity (%) [PDB]:** 0.0
- **1D identity (%) [Gaps excluded][PDB]:** 0.0
- **1D identity - Alignment Gaps [PDB]:** 1584
- **2D identity (%) [PDB]:** 16.75
- **2D identity (%) [Gaps excluded][PDB]:** 93.36
- **2D identity - Alignment Gaps [PDB]:** 1102
- **3D similarity (TM-Score) (%) [PDB]:** 16.1

- **Gene name:** gag-pol
- **RefSeq ID:** NC\_001802
- **Genomic sequence length:** 9181
- **5-UTR|CDS|3-UTR identity (%):** 23.6 | 40.46 | 23.63
- **5-UTR|CDS|3-UTR identity (%) [Gaps excluded]:** 75.9 | 77.79 | 80.65
- **5-UTR|CDS|3-UTR identity [Alignment Gaps]:** 184 | 2566 | 374

**Uniprot Description:**  
  
Gag-Pol polyprotein
Mediates, with Gag polyprotein, the essential events in virion assembly, including binding the plasma membrane, making the protein-protein interactions necessary to create spherical particles, recruiting the viral Env proteins, and packaging the genomic RNA via direct interactions with the RNA packaging sequence (Psi). Gag-Pol polyprotein may regulate its own translation, by the binding genomic RNA in the 5'-UTR. At low concentration, the polyprotein would promote translation, whereas at high concentration, the polyprotein would encapsidate genomic RNA and then shut off translation.  
  
Matrix protein p17
Homotrimer; further assembles as hexamers of trimers (PubMed:19327811). Interacts with gp41 (via C-terminus) (By similarity). Interacts with host CALM1; this interaction induces a conformational change in the Matrix protein, triggering exposure of the myristate group (PubMed:24500712). Interacts with host AP3D1; this interaction allows the polyprotein trafficking to multivesicular bodies during virus assembly (By similarity). Part of the pre-integration complex (PIC) which is composed of viral genome, matrix protein, Vpr and integrase (By similarity).  
  
**Gene Ontology Information:**

Molecular Function

- aspartic-type endopeptidase activity
- DNA binding
- DNA-directed DNA polymerase activity
- exoribonuclease H activity
- identical protein binding
- lipid binding
- RNA binding
- RNA-directed DNA polymerase activity
- RNA-DNA hybrid ribonuclease activity
- structural molecule activity
- zinc ion binding

Location

- host cell nucleus
- host cell plasma membrane
- host multivesicular body
- viral nucleocapsid
- virion membrane

Biological process

- DNA integration
- DNA recombination
- entry into host
- establishment of integrated proviral latency
- fusion of virus membrane with host plasma membrane
- induction by virus of host cysteine-type endopeptidase activity involved in apoptotic process
- RNA-dependent DNA biosynthetic process
- suppression by virus of host gene expression
- uncoating of virus
- viral genome integration into host DNA
- viral genome packaging
- viral life cycle
- viral penetration into host nucleus
- virion assembly

---

38

- **Protein name:** RecBCD enzyme subunit RecB
- **Organism:** Escherichia coli (strain K12)
- **Uniprot Accession Number:** P08394
- **Protein sequence length:** 1180 aa
- **1D identity (%):** 9.26
- **1D identity (%) [Gaps excluded]:** 30.31
- **1D identity - Alignment Gaps:** 1305
- **Common reported functions (%):** 0.0
- **Common reported locations (%):** 0.0
- **Common reported processes (%):** 0.0

- **PDB ID:** 5MBV
- **Chain:** B
- **Crystallized protein length:** 1127 aa
- **Resolution:** 3.8 Å
- **b-phipsi:** 0.031663
- **w-rdist:** 0.878623
- **t-alpha:** 0.11417
- **Chemical similarity (Tanimoto Index) (%):** 83.9
- **1D identity (%) [PDB]:** 0.0
- **1D identity (%) [Gaps excluded][PDB]:** 0.0
- **1D identity - Alignment Gaps [PDB]:** 2169
- **2D identity (%) [PDB]:** 27.68
- **2D identity (%) [Gaps excluded][PDB]:** 89.82
- **2D identity - Alignment Gaps [PDB]:** 1147
- **3D similarity (TM-Score) (%) [PDB]:** 20.77

- **Gene name:** recB
- **RefSeq ID:** N/A
- **Sequence length:** N/A
- **5-UTR|CDS|3-UTR identity (%):** N/A | N/A | N/A
- **5-UTR|CDS|3-UTR identity (%) [Gaps excluded]:** N/A | N/A | N/A
- **5-UTR|CDS|3-UTR identity [Alignment Gaps]:** N/A | N/A | N/A

**Uniprot Description:**  
  
A helicase/nuclease that prepares dsDNA breaks (DSB) for recombinational DNA repair. Binds to DSBs and unwinds DNA via a rapid (>1 kb/second) and highly processive (>30 kb) ATP-dependent bidirectional helicase. Unwinds dsDNA until it encounters a Chi (crossover hotspot instigator, 5'-GCTGGTGG-3') sequence from the 3' direction. Cuts ssDNA a few nucleotides 3' to Chi site, by nicking one strand or switching the strand degraded (depending on the reaction conditions). The properties and activities of the enzyme are changed at Chi. The Chi-altered holoenzyme produces a long 3'-ssDNA overhang which facilitates RecA-binding to the ssDNA for homologous DNA recombination and repair. Holoenzyme degrades any linearized DNA that is unable to undergo homologous recombination (PubMed:4562392, PubMed:4552016, PubMed:123277). In the holoenzyme this subunit contributes ATPase, 3'-5' helicase, exonuclease activity and loads RecA onto ssDNA. The RecBC complex requires the RecD subunit for nuclease activity, but can translocate along ssDNA in both directions.  
  
Heterotrimer of RecB, RecC and RecD. All subunits contribute to DNA-binding. The C-terminus interacts with RecA (PubMed:16483938). Interacts with YgbT (Cas1) (PubMed:21219465).  
  
**Gene Ontology Information:**

Molecular Function

- 3'-5' DNA helicase activity
- ATP binding
- DNA-dependent ATPase activity
- DNA binding
- DNA helicase activity
- DNA translocase activity
- endodeoxyribonuclease activity
- exodeoxyribonuclease V activity
- magnesium ion binding

Location

- cytosol
- exodeoxyribonuclease V complex

Biological process

- clearance of foreign intracellular DNA
- DNA recombination
- double-strand break repair via homologous recombination
- recombinational repair
- response to radiation

---

39

- **Protein name:** Major DNA-binding protein
- **Organism:** Human herpesvirus 1 (strain 17)
- **Uniprot Accession Number:** P04296
- **Protein sequence length:** 1196 aa
- **1D identity (%):** 3.15
- **1D identity (%) [Gaps excluded]:** 28.69
- **1D identity - Alignment Gaps:** 1981
- **Common reported functions (%):** 0.0
- **Common reported locations (%):** 0.0
- **Common reported processes (%):** 0.0

- **PDB ID:** 1URJ
- **Chain:** A
- **Crystallized protein length:** 1029 aa
- **Resolution:** 3.0 Å
- **b-phipsi:** 0.018198
- **w-rdist:** 1.3091
- **t-alpha:** 0.030885
- **Chemical similarity (Tanimoto Index) (%):** 82.22
- **1D identity (%) [PDB]:** 0.0
- **1D identity (%) [Gaps excluded][PDB]:** 0.0
- **1D identity - Alignment Gaps [PDB]:** 2079
- **2D identity (%) [PDB]:** 30.11
- **2D identity (%) [Gaps excluded][PDB]:** 88.45
- **2D identity - Alignment Gaps [PDB]:** 1023
- **3D similarity (TM-Score) (%) [PDB]:** 22.71

- **Gene name:** DBP
- **RefSeq ID:** NC\_001806
- **Genomic sequence length:** 152222
- **5-UTR|CDS|3-UTR identity (%):** N/A | 34.99 | N/A
- **5-UTR|CDS|3-UTR identity (%) [Gaps excluded]:** N/A | 73.86 | N/A
- **5-UTR|CDS|3-UTR identity [Alignment Gaps]:** N/A | 2647 | N/A

**Uniprot Description:**  
  
Plays several crucial roles in viral infection. Participates in the opening of the viral DNA origin to initiate replication by interacting with the origin-binding protein. May disrupt loops, hairpins and other secondary structures present on ssDNA to reduce and eliminate pausing of viral DNA polymerase at specific sites during elongation. Promotes viral DNA recombination by performing strand-transfer, characterized by the ability to transfer a DNA strand from a linear duplex to a complementary single-stranded DNA circle. Can also catalyze the renaturation of complementary single strands. Additionally, reorganizes the host cell nucleus, leading to the formation of prereplicative sites and replication compartments. This process is driven by the protein which can form double-helical filaments in the absence of DNA.  
  
Homooligomers. Forms double-helical filaments necessary for the formation of replication compartments within the host nucleus (PubMed:26676794). Interacts with the origin-binding protein (PubMed:7961904). Interacts with the helicase primase complex; this interaction stimulates primer synthesis activity of the helicase-primase complex (PubMed:9129659). Interacts with the DNA polymerase (PubMed:3031068). Interacts with the alkaline exonuclease; this interaction increases its nuclease processivity (PubMed:15078942). Interacts with ICP27; this interaction plays a role in the stimulation of late gene transcription (PubMed:15582656).  
  
**Gene Ontology Information:**

Molecular Function

- DNA binding
- metal ion binding
- single-stranded DNA binding

Location

- host cell nucleus
- nuclear viral factory

Biological process

- bidirectional double-stranded viral DNA replication
- DNA replication

---

40

- **Protein name:** Pre-mRNA-processing-splicing factor 8
- **Organism:** Homo sapiens
- **Uniprot Accession Number:** Q6P2Q9
- **Protein sequence length:** 2335 aa
- **1D identity (%):** 7.61
- **1D identity (%) [Gaps excluded]:** 28.09
- **1D identity - Alignment Gaps:** 2070
- **Common reported functions (%):** 0.0
- **Common reported locations (%):** 0.0
- **Common reported processes (%):** 0.0

- **PDB ID:** 5Z58
- **Chain:** A
- **Crystallized protein length:** 2211 aa
- **Resolution:** 4.9 Å
- **b-phipsi:** 0.032376
- **w-rdist:** 0.889346
- **t-alpha:** 0.101215
- **Chemical similarity (Tanimoto Index) (%):** 84.93
- **1D identity (%) [PDB]:** 0.03
- **1D identity (%) [Gaps excluded][PDB]:** 50.0
- **1D identity - Alignment Gaps [PDB]:** 3252
- **2D identity (%) [PDB]:** N/A
- **2D identity (%) [Gaps excluded][PDB]:** N/A
- **2D identity - Alignment Gaps [PDB]:** N/A
- **3D similarity (TM-Score) (%) [PDB]:** 5.34

- **Gene name:** PRPF8
- **RefSeq ID:** NM\_006445
- **Transcript sequence length:** 7280
- **5-UTR|CDS|3-UTR identity (%):** 20.92 | 34.86 | 33.45
- **5-UTR|CDS|3-UTR identity (%) [Gaps excluded]:** 71.95 | 78.93 | 80.51
- **5-UTR|CDS|3-UTR identity [Alignment Gaps]:** 200 | 4194 | 166

**Uniprot Description:**  
  
Plays role in pre-mRNA splicing as core component of precatalytic, catalytic and postcatalytic spliceosomal complexes, both of the predominant U2-type spliceosome and the minor U12-type spliceosome (PubMed:10411133, PubMed:11971955, PubMed:28502770, PubMed:28781166, PubMed:28076346, PubMed:29361316, PubMed:30315277, PubMed:29360106, PubMed:29301961, PubMed:30728453, PubMed:30705154). Functions as a scaffold that mediates the ordered assembly of spliceosomal proteins and snRNAs. Required for the assembly of the U4/U6-U5 tri-snRNP complex, a building block of the spliceosome. Functions as scaffold that positions spliceosomal U2, U5 and U6 snRNAs at splice sites on pre-mRNA substrates, so that splicing can occur. Interacts with both the 5' and the 3' splice site.  
  
Part of the U5 snRNP complex (PubMed:2532307, PubMed:2527369). Component of the U4/U6-U5 tri-snRNP complex composed of the U4, U6 and U5 snRNAs and at least PRPF3, PRPF4, PRPF6, PRPF8, PRPF31, SNRNP200, TXNL4A, SNRNP40, DDX23, CD2BP2, PPIH, SNU13, EFTUD2, SART1 and USP39 (PubMed:2479028, PubMed:16723661, PubMed:26912367). Component of the U5.U4atac/U6atac snRNP complexes in U12-dependent spliceosomes (PubMed:11971955). Core component of U2-type precatalytic, catalytic and postcatalytic spliceosomal complexes (PubMed:10411133, PubMed:28502770, PubMed:28781166, PubMed:28076346, PubMed:29361316, PubMed:30315277, PubMed:29360106, PubMed:29301961, PubMed:30728453, PubMed:30705154). Found in a mRNA splicing-dependent exon junction complex (EJC) with SRRM1. Interacts with U5 snRNP proteins SNRP116 and SNRNP40. Interacts with EFTUD2 and SNRNP200. Interacts (via the MPN (JAB/Mov34) domain) with PRPF3 ('Lys-63'-linked polyubiquitinated); may stabilize the U4/U6-U5 tri-snRNP complex. Interacts (via RNase H homology domain) with AAR2 (PubMed:26527271). Interacts with RPAP3 and URI1 in a ZNHIT2-dependent manner (PubMed:28561026).  
  
**Gene Ontology Information:**

Molecular Function

- isopeptidase activity
- K63-linked polyubiquitin modification-dependent protein binding
- pre-mRNA intronic binding
- RNA binding
- U1 snRNA binding
- U2 snRNA binding
- U5 snRNA binding
- U6 snRNA binding

Location

- catalytic step 2 spliceosome
- membrane
- nuclear speck
- nucleoplasm
- nucleus
- U2-type catalytic step 1 spliceosome
- U2-type catalytic step 2 spliceosome
- U2-type precatalytic spliceosome
- U4/U6 x U5 tri-snRNP complex
- U5 snRNP

Biological process

- cellular response to lipopolysaccharide
- cellular response to tumor necrosis factor
- mRNA processing
- mRNA splicing, via spliceosome
- RNA splicing
- RNA splicing, via transesterification reactions
- spliceosomal tri-snRNP complex assembly

---

41

- **Protein name:** DNA polymerase
- **Organism:** Bacillus phage phi29
- **Uniprot Accession Number:** P03680
- **Protein sequence length:** 575 aa
- **1D identity (%):** 7.96
- **1D identity (%) [Gaps excluded]:** 26.34
- **1D identity - Alignment Gaps:** 990
- **Common reported functions (%):** 0.0
- **Common reported locations (%):** 0.0
- **Common reported processes (%):** 0.0

- **PDB ID:** 1XHZ
- **Chain:** D
- **Crystallized protein length:** 571 aa
- **Resolution:** 2.7 Å
- **b-phipsi:** 0.001266
- **w-rdist:** 1.443392
- **t-alpha:** 0.128885
- **Chemical similarity (Tanimoto Index) (%):** 83.76
- **1D identity (%) [PDB]:** 0.0
- **1D identity (%) [Gaps excluded][PDB]:** 0.0
- **1D identity - Alignment Gaps [PDB]:** 1611
- **2D identity (%) [PDB]:** 22.9
- **2D identity (%) [Gaps excluded][PDB]:** 94.59
- **2D identity - Alignment Gaps [PDB]:** 983
- **3D similarity (TM-Score) (%) [PDB]:** 16.77

- **Gene name:** 2
- **RefSeq ID:** NC\_011048
- **Genomic sequence length:** 19282
- **5-UTR|CDS|3-UTR identity (%):** N/A | 28.35 | N/A
- **5-UTR|CDS|3-UTR identity (%) [Gaps excluded]:** N/A | 78.73 | N/A
- **5-UTR|CDS|3-UTR identity [Alignment Gaps]:** N/A | 2607 | N/A

**Uniprot Description:**  
  
Polymerase responsible for protein-primed viral DNA replication by strand displacement with high processivity and fidelity (PubMed:3863101) (PubMed:2498321). To start replication, the DNA polymerase forms a heterodimer with a free primer terminal protein (TP), recognizes the replication origins at both 5' ends of the linear chromosome, and initiates replication using as primer the OH-group of Ser-232 of the TP (PubMed:22210885). This polymerase possesses three enzymatic activities: DNA synthesis (polymerase), primer terminal protein (TP) deoxynucleotidylation, which is the formation of a covalent linkage (phosphoester) between the hydroxyl group of a specific serine residue in TP and 5'-dAMP, a reaction directed by the second T at the 3' end, and 3' to 5' exonuclease activity (PubMed:2790959). Exonuclease activity has a proofreading purpose (PubMed:2790959). DNA polymerase edits the polymerization errors using an intramolecular pathway as the primer terminus travels from one active site to the other without dissociation from the DNA (PubMed:10493855). DNA polymerization catalyzed by the DNA polymerase is a highly accurate process, but the protein-primed initiation is a quite inaccurate reaction (PubMed:8428945). Since the polymerase initiates the replication on the second thymine, the TP-dAMP initiation product translocates backwards to recover the template information of the first nucleotide (sliding back-mechanism) (PubMed:19011105).  
  
Interacts with the primer terminal protein; this interaction allows the initiation of TP-primed DNA replication at both viral DNA ends. Interacts with DNA.  
  
**Gene Ontology Information:**

Molecular Function

- DNA binding
- DNA-directed DNA polymerase activity
- exonuclease activity
- metal ion binding
- nucleoside binding
- nucleotide binding

Location  
  
N/A

Biological process

- DNA replication
- viral DNA genome replication

---

42

- **Protein name:** Tail fiber
- **Organism:** Acinetobacter phage phiAB6
- **Uniprot Accession Number:** A0A159BDB5
- **Protein sequence length:** 699 aa
- **1D identity (%):** 10.24
- **1D identity (%) [Gaps excluded]:** 31.15
- **1D identity - Alignment Gaps:** 996
- **Common reported functions (%):** 0.0
- **Common reported locations (%):** 0.0
- **Common reported processes (%):** 0.0

- **PDB ID:** 5JSE
- **Chain:** B
- **Crystallized protein length:** 548 aa
- **Resolution:** 1.89 Å
- **b-phipsi:** 0.021791
- **w-rdist:** 0.921643
- **t-alpha:** 0.231306
- **Chemical similarity (Tanimoto Index) (%):** 87.26
- **1D identity (%) [PDB]:** 0.0
- **1D identity (%) [Gaps excluded][PDB]:** 0.0
- **1D identity - Alignment Gaps [PDB]:** 1588
- **2D identity (%) [PDB]:** 27.89
- **2D identity (%) [Gaps excluded][PDB]:** 86.56
- **2D identity - Alignment Gaps [PDB]:** 814
- **3D similarity (TM-Score) (%) [PDB]:** 16.22

- **Gene name:** phiAB6\_gp40
- **RefSeq ID:** NC\_031086
- **Genomic sequence length:** 40570
- **5-UTR|CDS|3-UTR identity (%):** N/A | 36.03 | N/A
- **5-UTR|CDS|3-UTR identity (%) [Gaps excluded]:** N/A | 79.11 | N/A
- **5-UTR|CDS|3-UTR identity [Alignment Gaps]:** N/A | 2216 | N/A

**Uniprot Description:**  
  
N/A  
  
**Gene Ontology Information:**

Molecular Function  
  
N/A

Location  
  
N/A

Biological process  
  
N/A

---

43

- **Protein name:** Genome polyprotein
- **Organism:** Dengue virus type 3 (strain Sri Lanka/1266/2000)
- **Uniprot Accession Number:** Q6YMS4
- **Protein sequence length:** 3390 aa
- **1D identity (%):** 7.89
- **1D identity (%) [Gaps excluded]:** 29.82
- **1D identity - Alignment Gaps:** 2711
- **Common reported functions (%):** 0.0
- **Common reported locations (%):** 60.0
- **Common reported processes (%):** 28.57

- **PDB ID:** 5DTO
- **Chain:** A
- **Crystallized protein length:** 852 aa
- **Resolution:** 2.6 Å
- **b-phipsi:** 0.021713
- **w-rdist:** 1.355527
- **t-alpha:** 0.030025
- **Chemical similarity (Tanimoto Index) (%):** 80.79
- **1D identity (%) [PDB]:** 0.0
- **1D identity (%) [Gaps excluded][PDB]:** 0.0
- **1D identity - Alignment Gaps [PDB]:** 1894
- **2D identity (%) [PDB]:** 25.92
- **2D identity (%) [Gaps excluded][PDB]:** 90.95
- **2D identity - Alignment Gaps [PDB]:** 1054
- **3D similarity (TM-Score) (%) [PDB]:** 20.95

- **Gene name:** pol
- **RefSeq ID:** NC\_001475
- **Genomic sequence length:** 10707
- **5-UTR|CDS|3-UTR identity (%):** 21.35 | 25.26 | 30.8
- **5-UTR|CDS|3-UTR identity (%) [Gaps excluded]:** 76.92 | 79.2 | 82.42
- **5-UTR|CDS|3-UTR identity [Alignment Gaps]:** 203 | 7227 | 305

**Uniprot Description:**  
  
Capsid protein C
Plays a role in virus budding by binding to the cell membrane and gathering the viral RNA into a nucleocapsid that forms the core of a mature virus particle. During virus entry, may induce genome penetration into the host cytoplasm after hemifusion induced by the surface proteins. Can migrate to the cell nucleus where it modulates host functions. Overcomes the anti-viral effects of host EXOC1 by sequestering and degrading the latter through the proteasome degradation pathway.  
  
Capsid protein C
Homodimer. Interacts (via N-terminus) with host EXOC1 (via C-terminus); this interaction results in EXOC1 degradation through the proteasome degradation pathway (By similarity).  
  
**Gene Ontology Information:**

Molecular Function

- ATP binding
- double-stranded RNA binding
- ion channel activity
- metal ion binding
- mRNA (guanine-N7-)-methyltransferase activity
- mRNA (nucleoside-2'-O-)-methyltransferase activity
- nucleoside-triphosphatase activity
- protein dimerization activity
- RNA helicase activity
- RNA-directed 5'-3' RNA polymerase activity
- serine-type endopeptidase activity
- structural molecule activity

Location

- extracellular region
- host cell endoplasmic reticulum membrane
- host cell mitochondrion
- host cell nucleus
- host cell perinuclear region of cytoplasm
- integral component of membrane
- integral to membrane of host cell
- viral capsid
- viral envelope
- virion membrane

Biological process

- clathrin-dependent endocytosis of virus by host cell
- fusion of virus membrane with host endosome membrane
- induction by virus of host autophagy
- pore formation by virus in membrane of host cell
- protein complex oligomerization
- suppression by virus of host MAVS activity
- suppression by virus of host STAT2 activity
- suppression by virus of host TYK2 activity
- suppression by virus of host type I interferon-mediated signaling pathway
- viral RNA genome replication
- virion attachment to host cell

---

44

- **Protein name:** Major inner protein P1
- **Organism:** Pseudomonas phage phi6
- **Uniprot Accession Number:** P11126
- **Protein sequence length:** 769 aa
- **1D identity (%):** 8.42
- **1D identity (%) [Gaps excluded]:** 29.71
- **1D identity - Alignment Gaps:** 1140
- **Common reported functions (%):** 50.0
- **Common reported locations (%):** 0.0
- **Common reported processes (%):** 0.0

- **PDB ID:** 4BTQ
- **Chain:** B
- **Crystallized protein length:** 761 aa
- **Resolution:** 7.5 Å
- **b-phipsi:** 0.065989
- **w-rdist:** 1.311284
- **t-alpha:** 0.004065
- **Chemical similarity (Tanimoto Index) (%):** 30.08
- **1D identity (%) [PDB]:** 0.0
- **1D identity (%) [Gaps excluded][PDB]:** 0.0
- **1D identity - Alignment Gaps [PDB]:** 1801
- **2D identity (%) [PDB]:** 26.73
- **2D identity (%) [Gaps excluded][PDB]:** 88.73
- **2D identity - Alignment Gaps [PDB]:** 967
- **3D similarity (TM-Score) (%) [PDB]:** 16.63

- **Gene name:** P1
- **RefSeq ID:** NC\_003715
- **Genomic sequence length:** 6374
- **5-UTR|CDS|3-UTR identity (%):** N/A | 34.85 | N/A
- **5-UTR|CDS|3-UTR identity (%) [Gaps excluded]:** N/A | 76.33 | N/A
- **5-UTR|CDS|3-UTR identity [Alignment Gaps]:** N/A | 2288 | N/A

**Uniprot Description:**  
  
P1 is the major inner capsid (core) protein of the polyhedral procapsid, which is responsible for genomic replication and transcription. Forms a dodecahedral shell from 60 asymmetric dimers. Binds to RNA and may be involved in genomic packaging.  
  
Homodimer. Associates with the polymerase complex.  
  
**Gene Ontology Information:**

Molecular Function

- identical protein binding
- RNA binding

Location

- T=2 icosahedral viral capsid
- viral inner capsid
- viral nucleocapsid
- virion

Biological process  
  
N/A

---

45

- **Protein name:** N/A
- **Organism:** N/A
- **Uniprot Accession Number:** A0A0A0U7Z7
- **Protein sequence length:** N/A
- **1D identity (%):** N/A
- **1D identity (%) [Gaps excluded]:** N/A
- **1D identity - Alignment Gaps:** N/A
- **Common reported functions (%):** 0.0
- **Common reported locations (%):** 0.0
- **Common reported processes (%):** 0.0

- **PDB ID:** 6DJY
- **Chain:** B
- **Crystallized protein length:** 1121 aa
- **Resolution:** 3.9 Å
- **b-phipsi:** 0.017865
- **w-rdist:** 0.957524
- **t-alpha:** 0.247475
- **Chemical similarity (Tanimoto Index) (%):** 83.58
- **1D identity (%) [PDB]:** 0.0
- **1D identity (%) [Gaps excluded][PDB]:** 0.0
- **1D identity - Alignment Gaps [PDB]:** 2161
- **2D identity (%) [PDB]:** 28.41
- **2D identity (%) [Gaps excluded][PDB]:** 88.74
- **2D identity - Alignment Gaps [PDB]:** 1113
- **3D similarity (TM-Score) (%) [PDB]:** 20.44

- **Gene name:** N/A
- **RefSeq ID:** N/A
- **Sequence length:** N/A
- **5-UTR|CDS|3-UTR identity (%):** N/A | N/A | N/A
- **5-UTR|CDS|3-UTR identity (%) [Gaps excluded]:** N/A | N/A | N/A
- **5-UTR|CDS|3-UTR identity [Alignment Gaps]:** N/A | N/A | N/A

**Uniprot Description:**  
  
N/A  
  
**Gene Ontology Information:**

Molecular Function  
  
N/A

Location  
  
N/A

Biological process  
  
N/A

---

46

- **Protein name:** Aminopeptidase N
- **Organism:** Homo sapiens
- **Uniprot Accession Number:** P15144
- **Protein sequence length:** 967 aa
- **1D identity (%):** 11.89
- **1D identity (%) [Gaps excluded]:** 26.59
- **1D identity - Alignment Gaps:** 856
- **Common reported functions (%):** 0.0
- **Common reported locations (%):** 20.0
- **Common reported processes (%):** 0.0

- **PDB ID:** 6U7G
- **Chain:** A
- **Crystallized protein length:** 898 aa
- **Resolution:** 2.35 Å
- **b-phipsi:** 0.026987
- **w-rdist:** 1.393062
- **t-alpha:** 0.0266
- **Chemical similarity (Tanimoto Index) (%):** 88.61
- **1D identity (%) [PDB]:** 0.05
- **1D identity (%) [Gaps excluded][PDB]:** 50.0
- **1D identity - Alignment Gaps [PDB]:** 1935
- **2D identity (%) [PDB]:** 35.71
- **2D identity (%) [Gaps excluded][PDB]:** 89.51
- **2D identity - Alignment Gaps [PDB]:** 833
- **3D similarity (TM-Score) (%) [PDB]:** 21.41

- **Gene name:** ANPEP
- **RefSeq ID:** N/A
- **Sequence length:** N/A
- **5-UTR|CDS|3-UTR identity (%):** N/A | N/A | N/A
- **5-UTR|CDS|3-UTR identity (%) [Gaps excluded]:** N/A | N/A | N/A
- **5-UTR|CDS|3-UTR identity [Alignment Gaps]:** N/A | N/A | N/A

**Uniprot Description:**  
  
Broad specificity aminopeptidase which plays a role in the final digestion of peptides generated from hydrolysis of proteins by gastric and pancreatic proteases. Also involved in the processing of various peptides including peptide hormones, such as angiotensin III and IV, neuropeptides, and chemokines. May also be involved the cleavage of peptides bound to major histocompatibility complex class II molecules of antigen presenting cells. May have a role in angiogenesis and promote cholesterol crystallization. May have a role in amino acid transport by acting as binding partner of amino acid transporter SLC6A19 and regulating its activity (By similarity).  
  
Homodimer. Interacts with SLC6A19 (By similarity).  
  
**Gene Ontology Information:**

Molecular Function

- aminopeptidase activity
- metalloaminopeptidase activity
- metallopeptidase activity
- peptide binding
- signaling receptor activity
- virus receptor activity
- zinc ion binding

Location

- cytoplasm
- endoplasmic reticulum-Golgi intermediate compartment
- extracellular exosome
- extracellular space
- integral component of membrane
- lysosomal membrane
- plasma membrane
- secretory granule membrane

Biological process

- angiogenesis
- cell differentiation
- neutrophil degranulation
- peptide catabolic process
- proteolysis
- regulation of blood pressure
- signal transduction

---

47

- **Protein name:** Aminopeptidase N
- **Organism:** Sus scrofa
- **Uniprot Accession Number:** P15145
- **Protein sequence length:** 963 aa
- **1D identity (%):** 12.67
- **1D identity (%) [Gaps excluded]:** 26.24
- **1D identity - Alignment Gaps:** 780
- **Common reported functions (%):** 0.0
- **Common reported locations (%):** 20.0
- **Common reported processes (%):** 0.0

- **PDB ID:** 4F5C
- **Chain:** B
- **Crystallized protein length:** 900 aa
- **Resolution:** 3.2 Å
- **b-phipsi:** 0.025424
- **w-rdist:** 1.360116
- **t-alpha:** 0.043074
- **Chemical similarity (Tanimoto Index) (%):** 88.62
- **1D identity (%) [PDB]:** 0.1
- **1D identity (%) [Gaps excluded][PDB]:** 50.0
- **1D identity - Alignment Gaps [PDB]:** 1934
- **2D identity (%) [PDB]:** 36.57
- **2D identity (%) [Gaps excluded][PDB]:** 89.36
- **2D identity - Alignment Gaps [PDB]:** 814
- **3D similarity (TM-Score) (%) [PDB]:** 20.79

- **Gene name:** ANPEP
- **RefSeq ID:** N/A
- **Sequence length:** N/A
- **5-UTR|CDS|3-UTR identity (%):** N/A | N/A | N/A
- **5-UTR|CDS|3-UTR identity (%) [Gaps excluded]:** N/A | N/A | N/A
- **5-UTR|CDS|3-UTR identity [Alignment Gaps]:** N/A | N/A | N/A

**Uniprot Description:**  
  
Broad specificity aminopeptidase which plays a role in the final digestion of peptides generated from hydrolysis of proteins by gastric and pancreatic proteases. Also involved in the processing of various peptides including peptide hormones, such as angiotensin III and IV, neuropeptides, and chemokines. May also be involved the cleavage of peptides bound to major histocompatibility complex class II molecules of antigen presenting cells. May have a role in angiogenesis and promote cholesterol crystallization (By similarity). It is able to degrade Leu-enkephalin and Met-enkephalin but not cholecystokinin CCK8, neuromedin C (GRP-10), somatostatin-14, substance P and vasoactive intestinal peptide (PubMed:8963385). May have a role in amino acid transport by acting as binding partner of amino acid transporter SLC6A19 and regulating its activity (By similarity).  
  
Homodimer. Interacts with SLC6A19 (By similarity).  
  
**Gene Ontology Information:**

Molecular Function

- metalloaminopeptidase activity
- peptide binding
- virus receptor activity
- zinc ion binding

Location

- cytoplasm
- integral component of membrane
- plasma membrane

Biological process

- angiogenesis
- cell differentiation
- peptide catabolic process
- proteolysis
- regulation of blood pressure
- signal transduction

---

48

- **Protein name:** Dynein heavy chain, cytoplasmic
- **Organism:** Saccharomyces cerevisiae (strain ATCC 204508 / S288c)
- **Uniprot Accession Number:** P36022
- **Protein sequence length:** 4092 aa
- **1D identity (%):** 6.67
- **1D identity (%) [Gaps excluded]:** 31.9
- **1D identity - Alignment Gaps:** 3509
- **Common reported functions (%):** 0.0
- **Common reported locations (%):** 0.0
- **Common reported processes (%):** 0.0

- **PDB ID:** 4W8F
- **Chain:** B
- **Crystallized protein length:** 2609 aa
- **Resolution:** 3.54 Å
- **b-phipsi:** 0.061438
- **w-rdist:** 0.819686
- **t-alpha:** 0.391903
- **Chemical similarity (Tanimoto Index) (%):** 81.8
- **1D identity (%) [PDB]:** 0.0
- **1D identity (%) [Gaps excluded][PDB]:** 0.0
- **1D identity - Alignment Gaps [PDB]:** 3654
- **2D identity (%) [PDB]:** N/A
- **2D identity (%) [Gaps excluded][PDB]:** N/A
- **2D identity - Alignment Gaps [PDB]:** N/A
- **3D similarity (TM-Score) (%) [PDB]:** 25.5

- **Gene name:** DYN1
- **RefSeq ID:** N/A
- **Sequence length:** N/A
- **5-UTR|CDS|3-UTR identity (%):** N/A | N/A | N/A
- **5-UTR|CDS|3-UTR identity (%) [Gaps excluded]:** N/A | N/A | N/A
- **5-UTR|CDS|3-UTR identity [Alignment Gaps]:** N/A | N/A | N/A

**Uniprot Description:**  
  
Cytoplasmic dynein acts as a motor for the intracellular retrograde motility of vesicles and organelles along microtubules. Dynein has ATPase activity; the force-producing power stroke is thought to occur on release of ADP. Required to maintain uniform nuclear distribution in hyphae. May play an important role in the proper orientation of the mitotic spindle into the budding daughter cell yeast. Probably required for normal progression of the cell cycle.  
  
The dynein complex consists of at least two heavy chains and a number of intermediate and light chains. Interacts with DYN3.  
  
**Gene Ontology Information:**

Molecular Function

- ATP binding
- ATP-dependent microtubule motor activity, minus-end-directed
- dynein intermediate chain binding
- dynein light intermediate chain binding

Location

- astral microtubule
- cell cortex
- cytoplasmic dynein complex
- cytoplasmic microtubule
- dynein complex
- spindle pole body

Biological process

- cytoplasmic microtubule organization
- establishment of mitotic spindle localization
- establishment of mitotic spindle orientation
- establishment of spindle localization
- karyogamy
- microtubule-based movement
- minus-end-directed vesicle transport along microtubule
- mitotic cell cycle
- mitotic sister chromatid segregation
- nuclear migration
- nuclear migration along microtubule

---

49

- **Protein name:** DNA polymerase subunit gamma-1
- **Organism:** Homo sapiens
- **Uniprot Accession Number:** P54098
- **Protein sequence length:** 1239 aa
- **1D identity (%):** 12.78
- **1D identity (%) [Gaps excluded]:** 27.85
- **1D identity - Alignment Gaps:** 932
- **Common reported functions (%):** 0.0
- **Common reported locations (%):** 0.0
- **Common reported processes (%):** 0.0

- **PDB ID:** 5C51
- **Chain:** A
- **Crystallized protein length:** 983 aa
- **Resolution:** 3.43 Å
- **b-phipsi:** 0.027981
- **w-rdist:** 1.240313
- **t-alpha:** 0.07298
- **Chemical similarity (Tanimoto Index) (%):** N/A
- **1D identity (%) [PDB]:** 0.0
- **1D identity (%) [Gaps excluded][PDB]:** 0.0
- **1D identity - Alignment Gaps [PDB]:** 2029
- **2D identity (%) [PDB]:** 24.62
- **2D identity (%) [Gaps excluded][PDB]:** 91.84
- **2D identity - Alignment Gaps [PDB]:** 1171
- **3D similarity (TM-Score) (%) [PDB]:** 20.2

- **Gene name:** POLG
- **RefSeq ID:** NM\_002693
- **Transcript sequence length:** 4462
- **5-UTR|CDS|3-UTR identity (%):** 27.95 | 34.47 | 34.19
- **5-UTR|CDS|3-UTR identity (%) [Gaps excluded]:** 72.05 | 77.05 | 83.33
- **5-UTR|CDS|3-UTR identity [Alignment Gaps]:** 254 | 2880 | 276

**Uniprot Description:**  
  
Involved in the replication of mitochondrial DNA. Associates with mitochondrial DNA.  
  
Heterotrimer composed of a catalytic subunit and a homodimer of accessory subunits (By similarity). Interacts with TTC3 (PubMed:29290964).  
  
**Gene Ontology Information:**

Molecular Function

- 3'-5' exonuclease activity
- chromatin binding
- DNA binding
- DNA-directed DNA polymerase activity
- protease binding

Location

- gamma DNA polymerase complex
- mitochondrial nucleoid
- mitochondrion
- protein-containing complex
- terminal bouton

Biological process

- aging
- base-excision repair, gap-filling
- cellular response to glucose stimulus
- DNA metabolic process
- DNA-dependent DNA replication
- mitochondrial DNA replication
- response to gamma radiation
- response to hyperoxia
- response to light stimulus

---

50

- **Protein name:** Cullin-4A
- **Organism:** Homo sapiens
- **Uniprot Accession Number:** Q13619
- **Protein sequence length:** 759 aa
- **1D identity (%):** 3.99
- **1D identity (%) [Gaps excluded]:** 25.27
- **1D identity - Alignment Gaps:** 1478
- **Common reported functions (%):** 0.0
- **Common reported locations (%):** 0.0
- **Common reported processes (%):** 0.0

- **PDB ID:** 2HYE
- **Chain:** C
- **Crystallized protein length:** 719 aa
- **Resolution:** 3.1 Å
- **b-phipsi:** 0.093818
- **w-rdist:** 0.791059
- **t-alpha:** 0.330819
- **Chemical similarity (Tanimoto Index) (%):** N/A
- **1D identity (%) [PDB]:** 0.06
- **1D identity (%) [Gaps excluded][PDB]:** 50.0
- **1D identity - Alignment Gaps [PDB]:** 1755
- **2D identity (%) [PDB]:** 18.98
- **2D identity (%) [Gaps excluded][PDB]:** 94.56
- **2D identity - Alignment Gaps [PDB]:** 1171
- **3D similarity (TM-Score) (%) [PDB]:** 19.43

- **Gene name:** CUL4A
- **RefSeq ID:** N/A
- **Sequence length:** N/A
- **5-UTR|CDS|3-UTR identity (%):** N/A | N/A | N/A
- **5-UTR|CDS|3-UTR identity (%) [Gaps excluded]:** N/A | N/A | N/A
- **5-UTR|CDS|3-UTR identity [Alignment Gaps]:** N/A | N/A | N/A

**Uniprot Description:**  
  
Core component of multiple cullin-RING-based E3 ubiquitin-protein ligase complexes which mediate the ubiquitination of target proteins (PubMed:14578910, PubMed:15811626, PubMed:15548678, PubMed:15448697, PubMed:14739464, PubMed:16678110, PubMed:17041588, PubMed:24209620, PubMed:30166453, PubMed:33854232, PubMed:33854239). As a scaffold protein may contribute to catalysis through positioning of the substrate and the ubiquitin-conjugating enzyme (PubMed:14578910, PubMed:15811626, PubMed:15548678, PubMed:15448697, PubMed:14739464, PubMed:16678110, PubMed:17041588, PubMed:24209620). The E3 ubiquitin-protein ligase activity of the complex is dependent on the neddylation of the cullin subunit and is inhibited by the association of the deneddylated cullin subunit with TIP120A/CAND1 (PubMed:14578910, PubMed:15811626, PubMed:15548678, PubMed:15448697, PubMed:14739464, PubMed:16678110, PubMed:17041588, PubMed:24209620). The functional specificity of the E3 ubiquitin-protein ligase complex depends on the variable substrate recognition component (PubMed:14578910, PubMed:15811626, PubMed:15548678, PubMed:15448697, PubMed:14739464, PubMed:16678110, PubMed:17041588, PubMed:24209620). DCX(DET1-COP1) directs ubiquitination of JUN (PubMed:14739464). DCX(DDB2) directs ubiquitination of XPC (PubMed:15811626). DCX(DDB2) ubiquitinates histones H3-H4 and is required for efficient histone deposition during replication-coupled (H3.1) and replication-independent (H3.3) nucleosome assembly, probably by facilitating the transfer of H3 from ASF1A/ASF1B to other chaperones involved in histone deposition (PubMed:16678110, PubMed:17041588, PubMed:24209620). DCX(DTL) plays a role in PCNA-dependent polyubiquitination of CDT1 and MDM2-dependent ubiquitination of p53/TP53 in response to radiation-induced DNA damage and during DNA replication (PubMed:14578910, PubMed:15548678, PubMed:15448697). DCX(DTL) directs autoubiquitination of DTL (PubMed:23478445). In association with DDB1 and SKP2 probably is involved in ubiquitination of CDKN1B/p27kip (PubMed:16537899). Is involved in ubiquitination of HOXA9 (PubMed:14609952). The DDB1-CUL4A-DTL E3 ligase complex regulates the circadian clock function by mediating the ubiquitination and degradation of CRY1 (PubMed:26431207). A number of DCX complexes (containing either TRPC4AP or DCAF12 as substrate-recognition component) are part of the DesCEND (destruction via C-end degrons) pathway, which recognizes a C-degron located at the extreme C terminus of target proteins, leading to their ubiquitination and degradation (PubMed:29779948). The DCX(AMBRA1) complex is a master regulator of the transition from G1 to S cell phase by mediating ubiquitination of phosphorylated cyclin-D (CCND1, CCND2 and CCND3) (PubMed:33854232, PubMed:33854239). The DCX(AMBRA1) complex also acts as a regulator of Cul5-RING (CRL5) E3 ubiquitin-protein ligase complexes by mediating ubiquitination and degradation of Elongin-C (ELOC) component of CRL5 complexes (PubMed:30166453). With CUL4B, contributes to ribosome biogenesis (PubMed:26711351).  
  
Can self-associate (PubMed:17254749). Component of multiple DCX (DDB1-CUL4-X-box) E3 ubiquitin-protein ligase complexes that seem to consist of DDB1, CUL4A or CUL4B, RBX1 and a variable substrate recognition component which seems to belong to a protein family described as DCAF (Ddb1- and Cul4-associated factor) or CDW (CUL4-DDB1-associated WD40-repeat) proteins (PubMed:14578910, PubMed:12732143, PubMed:15548678, PubMed:14739464, PubMed:29779948, PubMed:30166453). Component of the CSA complex (DCX(ERCC8) complex) containing ERCC8, RBX1, DDB1 and CUL4A; the CSA complex interacts with RNA polymerase II; upon UV irradiation it interacts with the COP9 signalosome and preferentially with the hyperphosphorylated form of RNA polymerase II (PubMed:12732143). Component of the DCX(DET1-COP1) complex with the substrate recognition component DET1 and COP1 (PubMed:14739464). Component of the DCX(DDB2) complex with the substrate recognition component DDB2 (PubMed:15811626, PubMed:16678110). Component of the DCX(DTL) complex with the putative substrate recognition component DTL (PubMed:14578910, PubMed:15448697, PubMed:15548678). Component of DCX complexes part of the DesCEND (destruction via C-end degrons) pathway, which contain either TRPC4AP or DCAF12 as substrate-recognition component (PubMed:29779948). Component of the DCX(AMBRA1) complex with the substrate recognition component AMBRA1 (PubMed:30166453, PubMed:33854232, PubMed:33854239). Interacts with DDB1, RBX1, RNF7, CDT1, TIP120A/CAND1, SKP2, CDKN1B, MDM2, TP53 and HOXA9 (PubMed:16964240, PubMed:16537899, PubMed:22118460, PubMed:16482215, PubMed:12609982, PubMed:10230407, PubMed:14609952, PubMed:16678110). Interacts with DDB2; the interactions with DDB2 and CAND1 are mutually exclusive (PubMed:16678110, PubMed:22118460, PubMed:16482215). Interacts with DCAF1, DTL, DDA1, DCAF6, DCAF4, DCAF16, DCAF17, DET1, WDTC1, DCAF5, DCAF11, WDR24A, COP1, PAFAH1B1, ERCC8, GRWD1, FBXW5, RBBP7, GNB2, WSB1, WSB2, NUP43, PWP1, FBXW8, ATG16L1, KATNB1, RBBP4, RBBP5, LRWD1 and DCAF8 (PubMed:16949367, PubMed:22935713, PubMed:17079684). May interact with WDR26, WDR51B, SNRNP40, WDR61, WDR76, WDR5 (PubMed:17041588). Interacts (when neddylated) with ARIH1; leading to activate the E3 ligase activity of ARIH1 (PubMed:24076655). The DDB1-CUL4A complex interacts with CRY1 (PubMed:26431207). Interacts (unneddylated form) with DCUN1D1, DCUN1D2, DCUN1D3, DCUN1D4 and DCUN1D5; these interacti ons promote the cullin neddylation (PubMed:23201271, PubMed:26906416).  
  
**Gene Ontology Information:**

Molecular Function

- ubiquitin protein ligase binding
- ubiquitin-protein transferase activity

Location

- Cul4-RING E3 ubiquitin ligase complex
- Cul4A-RING E3 ubiquitin ligase complex
- cullin-RING ubiquitin ligase complex
- nucleoplasm
- SCF ubiquitin ligase complex

Biological process

- cellular response to DNA damage stimulus
- DNA damage response, detection of DNA damage
- G1/S transition of mitotic cell cycle
- global genome nucleotide-excision repair
- hemopoiesis
- in utero embryonic development
- intrinsic apoptotic signaling pathway
- negative regulation of granulocyte differentiation
- nucleotide-excision repair, DNA damage recognition
- nucleotide-excision repair, DNA duplex unwinding
- nucleotide-excision repair, DNA incision
- nucleotide-excision repair, DNA incision, 3'-to lesion
- nucleotide-excision repair, DNA incision, 5'-to lesion
- nucleotide-excision repair, preincision complex assembly
- nucleotide-excision repair, preincision complex stabilization
- positive regulation of cell population proliferation
- positive regulation of G1/S transition of mitotic cell cycle
- post-translational protein modification
- proteasome-mediated ubiquitin-dependent protein catabolic process
- protein ubiquitination
- regulation of DNA damage checkpoint
- regulation of nucleotide-excision repair
- regulation of protein metabolic process
- rhythmic process
- ribosome biogenesis
- SCF-dependent proteasomal ubiquitin-dependent protein catabolic process
- somatic stem cell population maintenance
- transcription-coupled nucleotide-excision repair
- viral process

---

51

- **Protein name:** Tail spike protein
- **Organism:** Salmonella phage P22
- **Uniprot Accession Number:** P12528
- **Protein sequence length:** 667 aa
- **1D identity (%):** 9.51
- **1D identity (%) [Gaps excluded]:** 26.67
- **1D identity - Alignment Gaps:** 920
- **Common reported functions (%):** 0.0
- **Common reported locations (%):** 0.0
- **Common reported processes (%):** 0.0

- **PDB ID:** 5GAI
- **Chain:** 0
- **Crystallized protein length:** 662 aa
- **Resolution:** 10.5 Å
- **b-phipsi:** 0.043167
- **w-rdist:** 0.977132
- **t-alpha:** 0.171727
- **Chemical similarity (Tanimoto Index) (%):** 83.77
- **1D identity (%) [PDB]:** 0.0
- **1D identity (%) [Gaps excluded][PDB]:** 0.0
- **1D identity - Alignment Gaps [PDB]:** 1702
- **2D identity (%) [PDB]:** N/A
- **2D identity (%) [Gaps excluded][PDB]:** N/A
- **2D identity - Alignment Gaps [PDB]:** N/A
- **3D similarity (TM-Score) (%) [PDB]:** 17.43

- **Gene name:** 9
- **RefSeq ID:** NC\_002371
- **Genomic sequence length:** 41724
- **5-UTR|CDS|3-UTR identity (%):** N/A | 32.17 | N/A
- **5-UTR|CDS|3-UTR identity (%) [Gaps excluded]:** N/A | 77.43 | N/A
- **5-UTR|CDS|3-UTR identity [Alignment Gaps]:** N/A | 2406 | N/A

**Uniprot Description:**  
  
Structural component of the short non-contractile tail. The tail comprises six spikes that mediate primary attachment to the host cell lipopolysaccharides (LPS) and display endorhamnosidase enzymatic activity, hydrolyzing the alpha-1,3-O-glycosidic linkage between rhamnose and galactose of the O-antigen polysaccharide. Digestion of the LPS brings the capsid near the cell outer membrane.  
  
Homotrimer. Interacts with the host O-antigen lipopolysaccharides; this interaction induces cleavage of host O-antigen.  
  
**Gene Ontology Information:**

Molecular Function

- endo-1,3-alpha-L-rhamnosidase activity

Location

- virus tail, fiber

Biological process

- adhesion receptor-mediated virion attachment to host cell
- disruption by virus of host envelope lipopolysaccharide during virus entry
- entry into host
- metabolic process
- pathogenesis
- virion attachment to host cell

---

52

- **Protein name:** NPC intracellular cholesterol transporter 1
- **Organism:** Homo sapiens
- **Uniprot Accession Number:** O15118
- **Protein sequence length:** 1278 aa
- **1D identity (%):** 10.68
- **1D identity (%) [Gaps excluded]:** 30.04
- **1D identity - Alignment Gaps:** 1213
- **Common reported functions (%):** 0.0
- **Common reported locations (%):** 20.0
- **Common reported processes (%):** 14.29

- **PDB ID:** 5JNX
- **Chain:** A
- **Crystallized protein length:** 1133 aa
- **Resolution:** 6.56 Å
- **b-phipsi:** 0.069032
- **w-rdist:** 0.964467
- **t-alpha:** 0.146704
- **Chemical similarity (Tanimoto Index) (%):** N/A
- **1D identity (%) [PDB]:** 0.05
- **1D identity (%) [Gaps excluded][PDB]:** 50.0
- **1D identity - Alignment Gaps [PDB]:** 2172
- **2D identity (%) [PDB]:** 25.01
- **2D identity (%) [Gaps excluded][PDB]:** 92.04
- **2D identity - Alignment Gaps [PDB]:** 1246
- **3D similarity (TM-Score) (%) [PDB]:** 22.44

- **Gene name:** NPC1
- **RefSeq ID:** NM\_000271
- **Transcript sequence length:** 4760
- **5-UTR|CDS|3-UTR identity (%):** 21.36 | 41.65 | 21.12
- **5-UTR|CDS|3-UTR identity (%) [Gaps excluded]:** 79.12 | 78.27 | 81.77
- **5-UTR|CDS|3-UTR identity [Alignment Gaps]:** 246 | 2339 | 583

**Uniprot Description:**  
  
Intracellular cholesterol transporter which acts in concert with NPC2 and plays an important role in the egress of cholesterol from the endosomal/lysosomal compartment (PubMed:9211849, PubMed:9927649, PubMed:10821832, PubMed:18772377, PubMed:27238017, PubMed:12554680). Unesterified cholesterol that has been released from LDLs in the lumen of the late endosomes/lysosomes is transferred by NPC2 to the cholesterol-binding pocket in the N-terminal domain of NPC1 (PubMed:9211849, PubMed:9927649, PubMed:18772377, PubMed:19563754, PubMed:27238017, PubMed:28784760). Cholesterol binds to NPC1 with the hydroxyl group buried in the binding pocket (PubMed:19563754). Binds oxysterol with higher affinity than cholesterol. May play a role in vesicular trafficking in glia, a process that may be crucial for maintaining the structural and functional integrity of nerve terminals (Probable).  
  
Interacts (via the second lumenal domain) with NPC2 (PubMed:18772377, PubMed:27238017, PubMed:27551080). Interacts with TMEM97 (PubMed:19583955). Interacts with TIM1 (PubMed:25855742).  
  
**Gene Ontology Information:**

Molecular Function

- cholesterol binding
- signaling receptor activity
- sterol transporter activity
- transmembrane signaling receptor activity
- virus receptor activity

Location

- endoplasmic reticulum
- extracellular exosome
- extracellular region
- Golgi apparatus
- integral component of lysosomal membrane
- integral component of membrane
- integral component of plasma membrane
- late endosome membrane
- lysosomal membrane
- lysosome
- membrane
- membrane raft
- nuclear envelope
- perinuclear region of cytoplasm

Biological process

- adult walking behavior
- autophagy
- bile acid metabolic process
- cellular response to low-density lipoprotein particle stimulus
- cellular response to steroid hormone stimulus
- cholesterol efflux
- cholesterol homeostasis
- cholesterol metabolic process
- cholesterol transport
- endocytosis
- establishment of protein localization to membrane
- gene expression
- intracellular cholesterol transport
- low-density lipoprotein particle clearance
- lysosomal transport
- membrane raft organization
- negative regulation of cell death
- negative regulation of macroautophagy
- protein glycosylation
- response to cadmium ion
- response to drug
- viral entry into host cell

---

53

- **Protein name:** Virion DNA-directed RNA polymerase
- **Organism:** Enterobacteria phage N4
- **Uniprot Accession Number:** Q859P9
- **Protein sequence length:** 3500 aa
- **1D identity (%):** 7.66
- **1D identity (%) [Gaps excluded]:** 26.25
- **1D identity - Alignment Gaps:** 2617
- **Common reported functions (%):** 0.0
- **Common reported locations (%):** 0.0
- **Common reported processes (%):** 0.0

- **PDB ID:** 4FF1
- **Chain:** B
- **Crystallized protein length:** 1095 aa
- **Resolution:** 2.47 Å
- **b-phipsi:** 0.061553
- **w-rdist:** 1.310001
- **t-alpha:** 0.058269
- **Chemical similarity (Tanimoto Index) (%):** 82.2
- **1D identity (%) [PDB]:** 0.05
- **1D identity (%) [Gaps excluded][PDB]:** 100.0
- **1D identity - Alignment Gaps [PDB]:** 2133
- **2D identity (%) [PDB]:** 23.17
- **2D identity (%) [Gaps excluded][PDB]:** 89.52
- **2D identity - Alignment Gaps [PDB]:** 1257
- **3D similarity (TM-Score) (%) [PDB]:** 20.02

- **Gene name:** 50
- **RefSeq ID:** NC\_008720
- **Genomic sequence length:** 70153
- **5-UTR|CDS|3-UTR identity (%):** N/A | 24.71 | N/A
- **5-UTR|CDS|3-UTR identity (%) [Gaps excluded]:** N/A | 80.35 | N/A
- **5-UTR|CDS|3-UTR identity [Alignment Gaps]:** N/A | 7587 | N/A

**Uniprot Description:**  
  
DNA-dependent RNA polymerase, which is injected into the host upon infection and transcribes the phage early genes from promoters that have a 5-bp stem-3 nt loop hairpin structure.  
  
**Gene Ontology Information:**

Molecular Function

- ATP binding
- DNA-directed 5'-3' RNA polymerase activity
- GTP binding
- metal ion binding

Location

- virion

Biological process  
  
N/A

---
