# Supplementary figures and images for: Identifying and profiling structural similarities between Spike of SARS-CoV-2 and other viral or host proteins with Machaon (part 1 of 2)
Source: Commun Biol. 2023 Jul 19;6:752. doi: 10.1038/s42003-023-05076-7 (PMC10356814; doi:10.1038/s42003-023-05076-7)

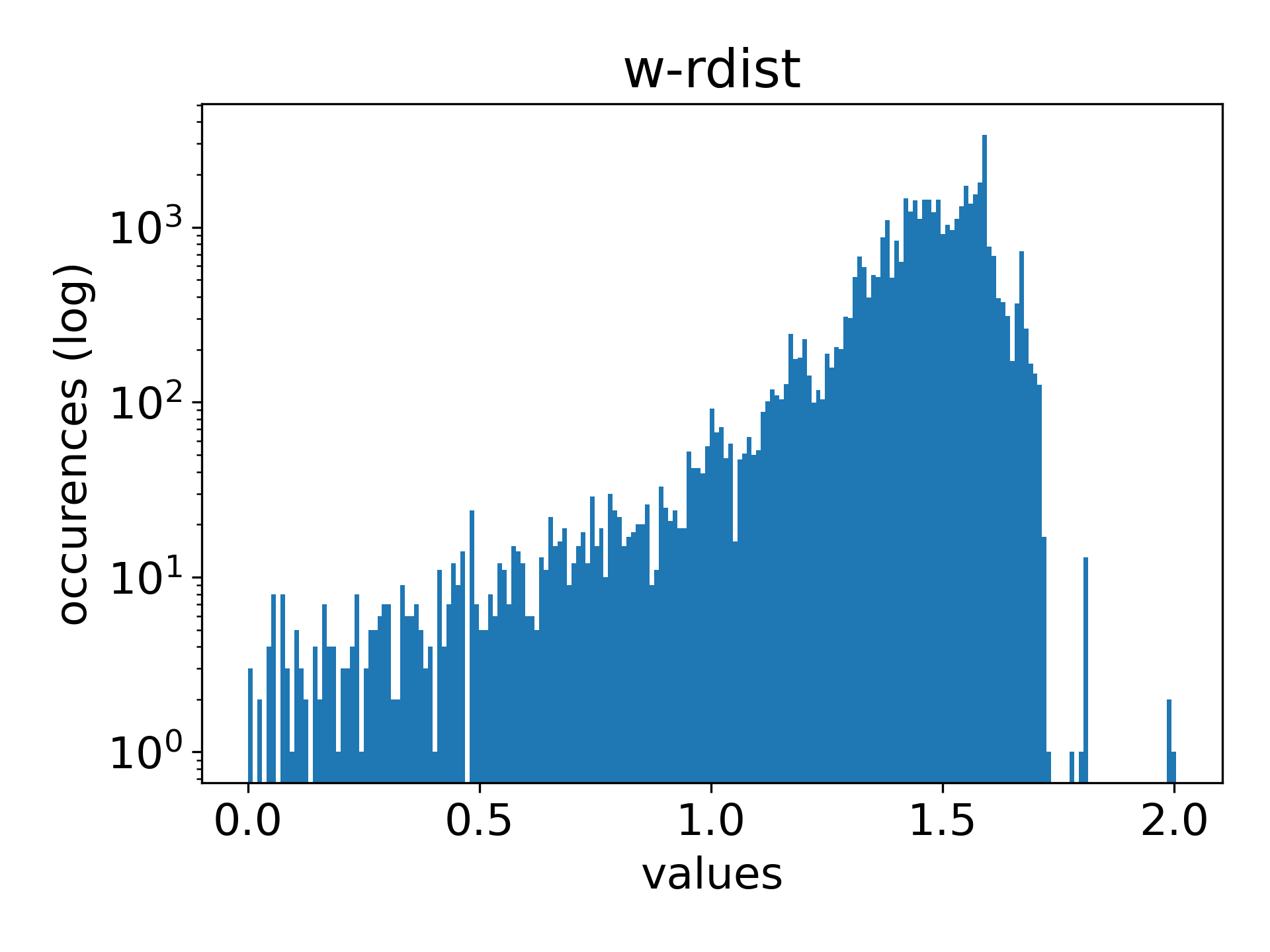

Supplement: Supplementary file 6 — Supplementary Data 3 [file 42003_2023_5076_MOESM6_ESM.zip › 6VXX_A_whole/metrics/6VXX_A_w-rdist.png]

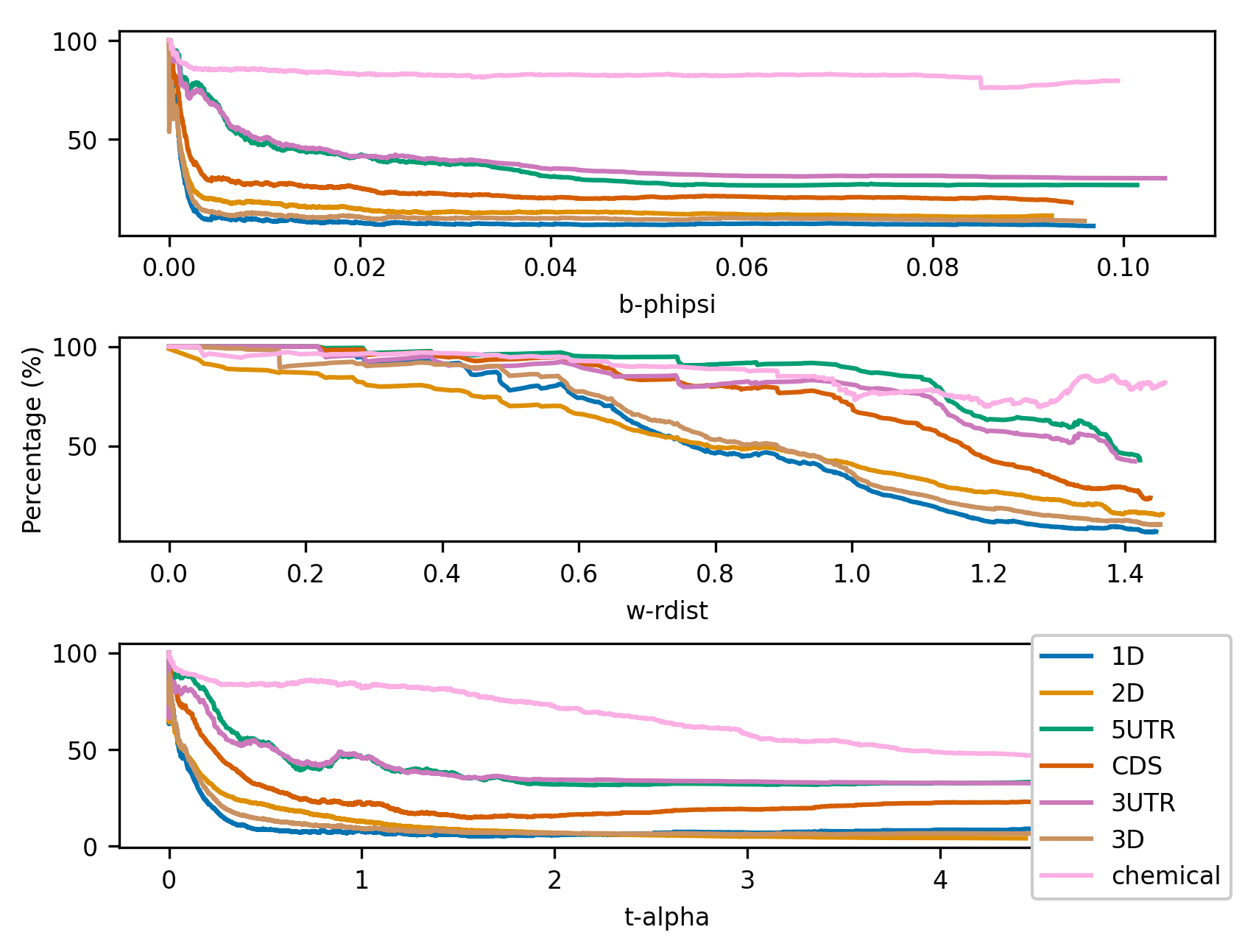

Supplement: Supplementary file 6 — Supplementary Data 3 [file 42003_2023_5076_MOESM6_ESM.zip › 6VXX_A_whole/metrics/6VXX_A-metrics.png]

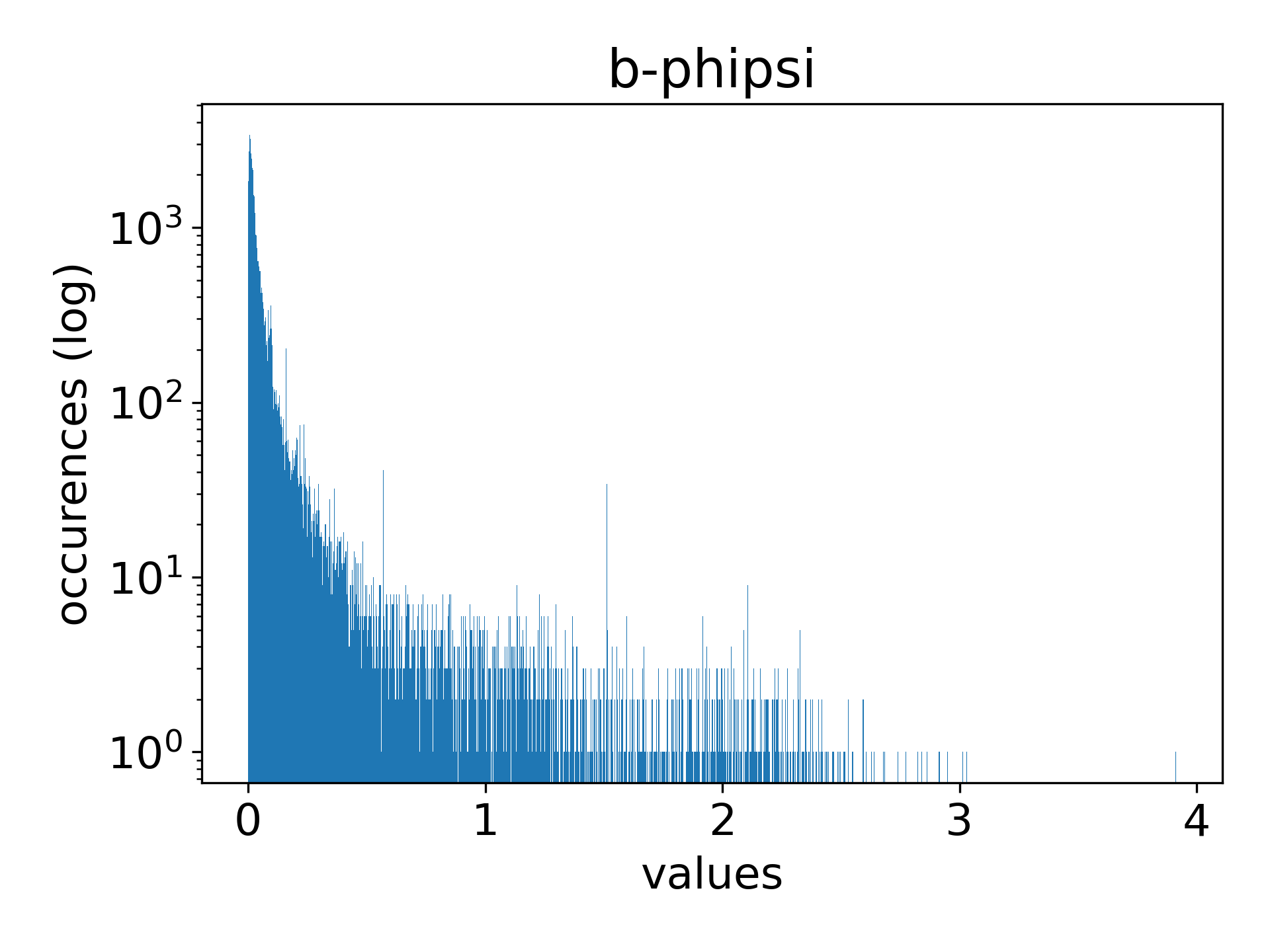

Supplement: Supplementary file 6 — Supplementary Data 3 [file 42003_2023_5076_MOESM6_ESM.zip › 6VXX_A_whole/metrics/6VXX_A_b-phipsi.png]

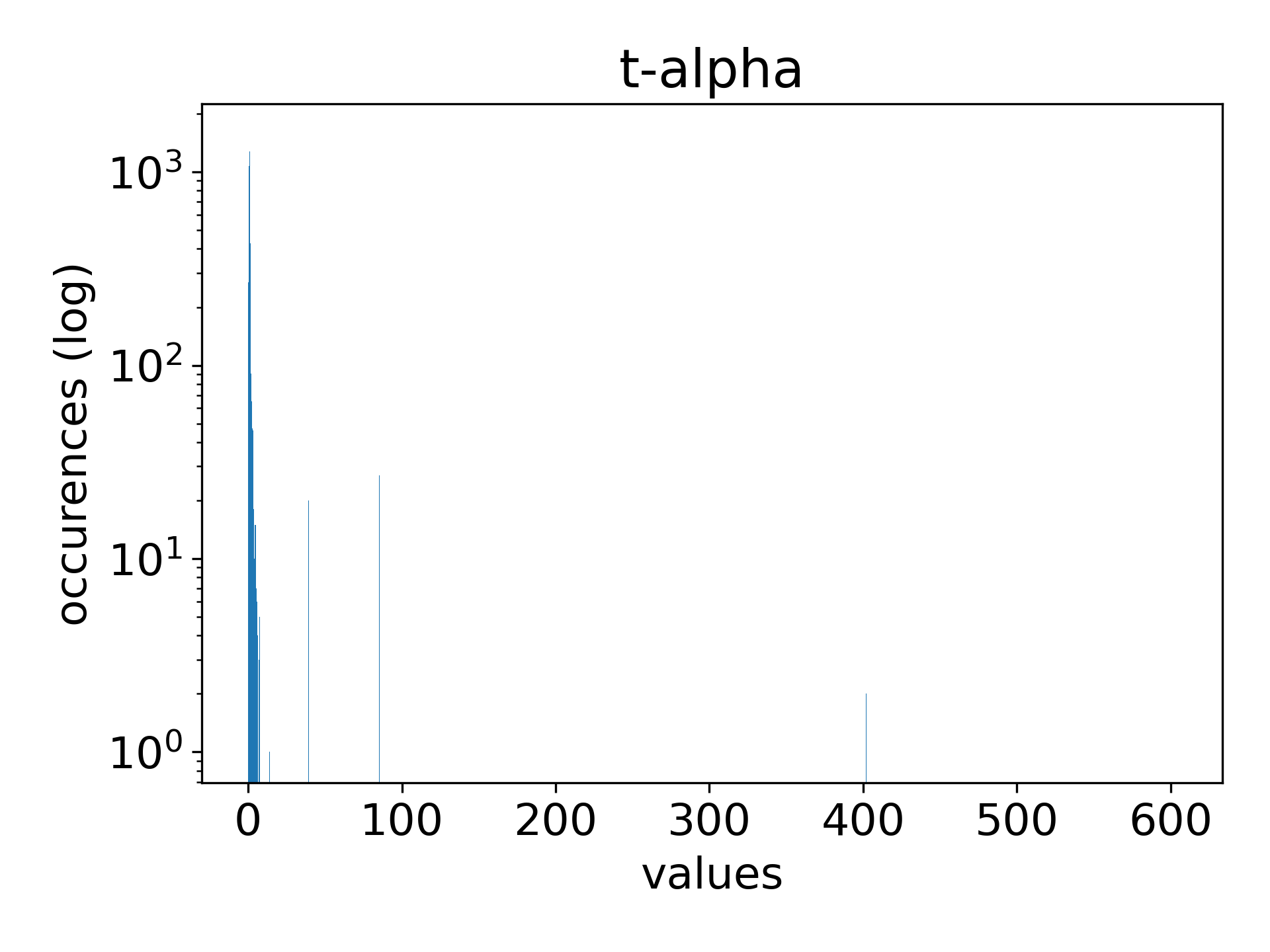

Supplement: Supplementary file 6 — Supplementary Data 3 [file 42003_2023_5076_MOESM6_ESM.zip › 6VXX_A_whole/metrics/6VXX_A_t-alpha.png]

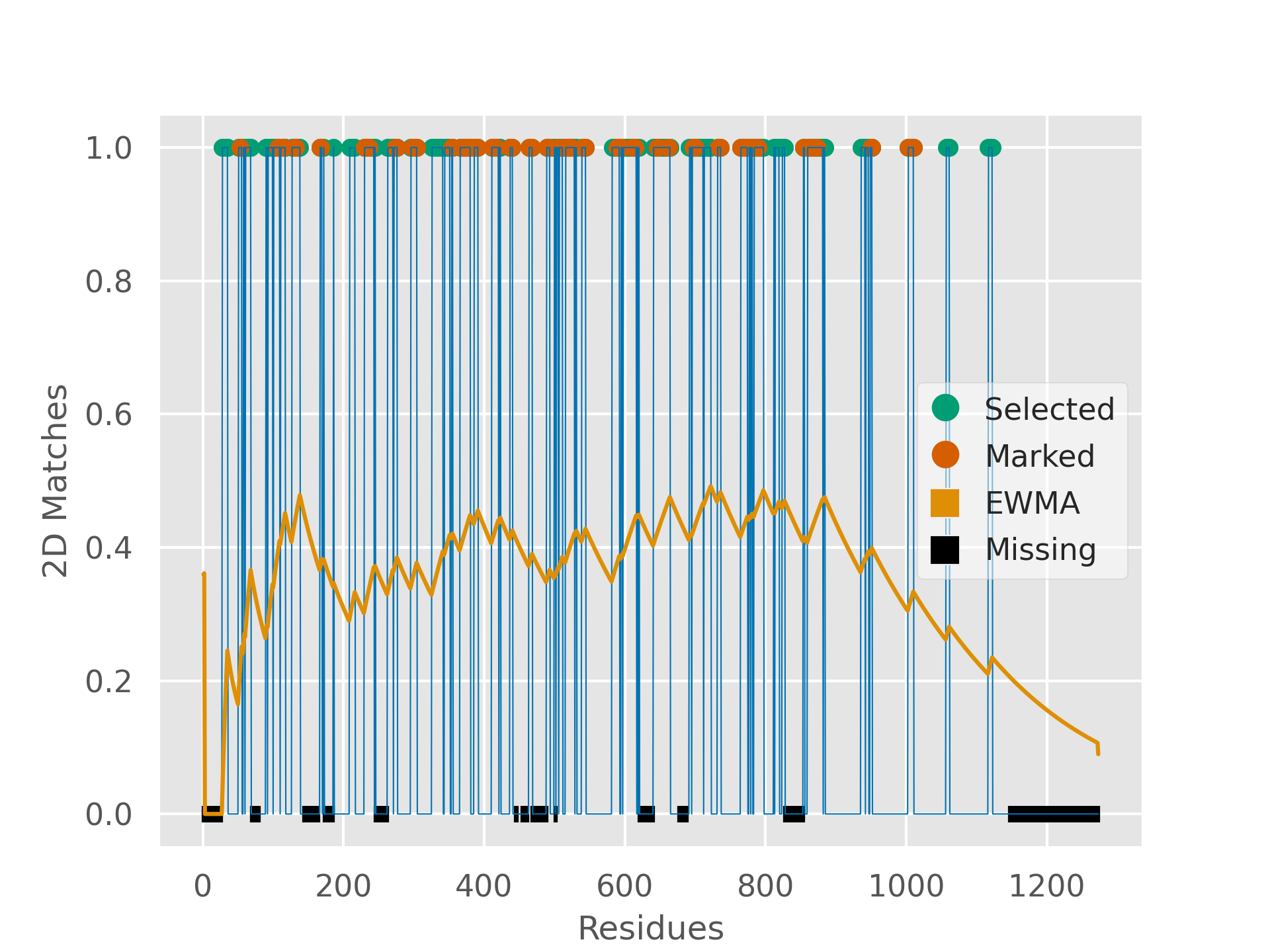

Supplement: Supplementary file 6 — Supplementary Data 3 [file 42003_2023_5076_MOESM6_ESM.zip › 6VXX_A_whole/go/6VXX_A_ubiquit_7aa0d1281fc74ceaa3a6f33371f43263.png]

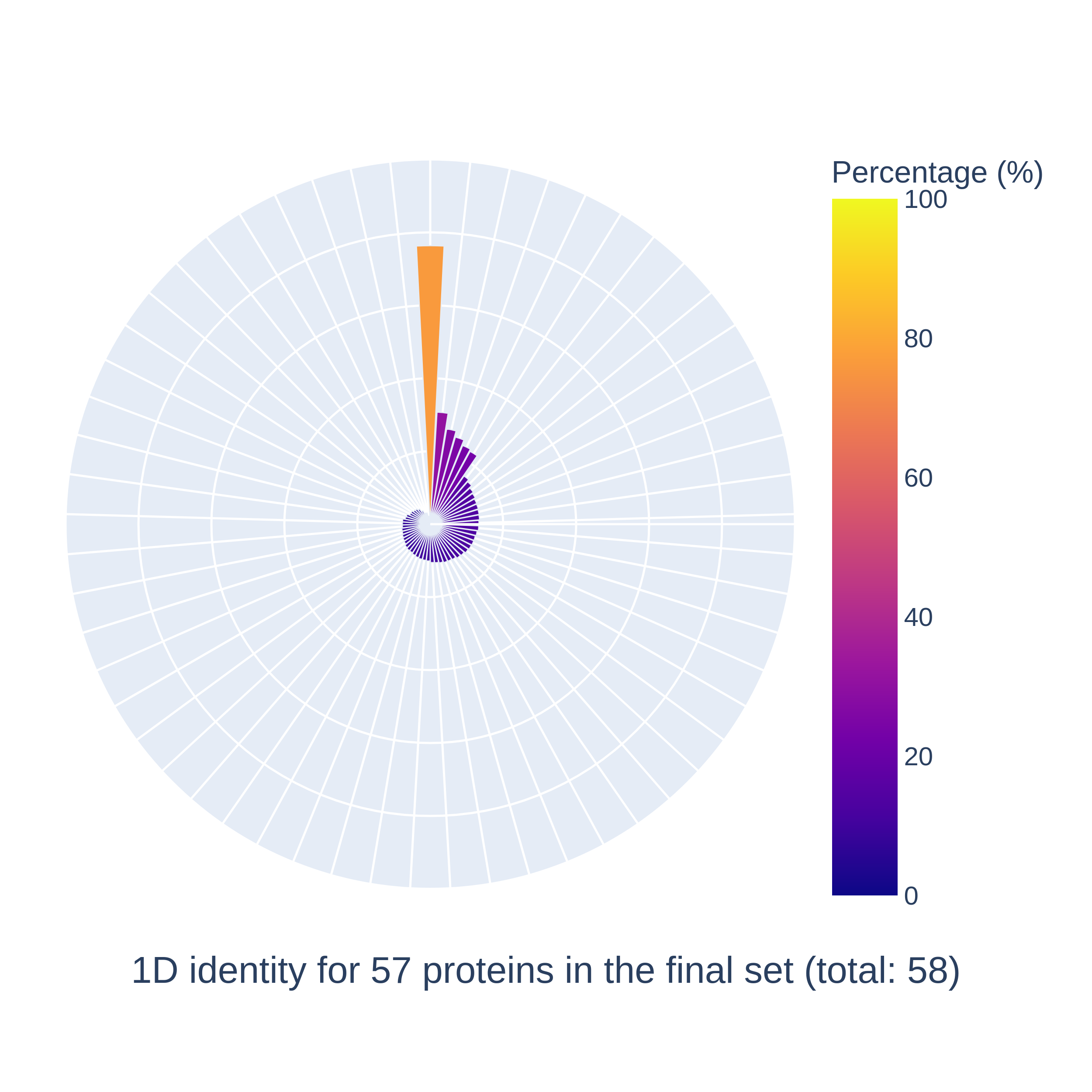

Supplement: Supplementary file 6 — Supplementary Data 3 [file 42003_2023_5076_MOESM6_ESM.zip › 6VXX_A_whole/plots/6VXX_A_1D-identity.png]

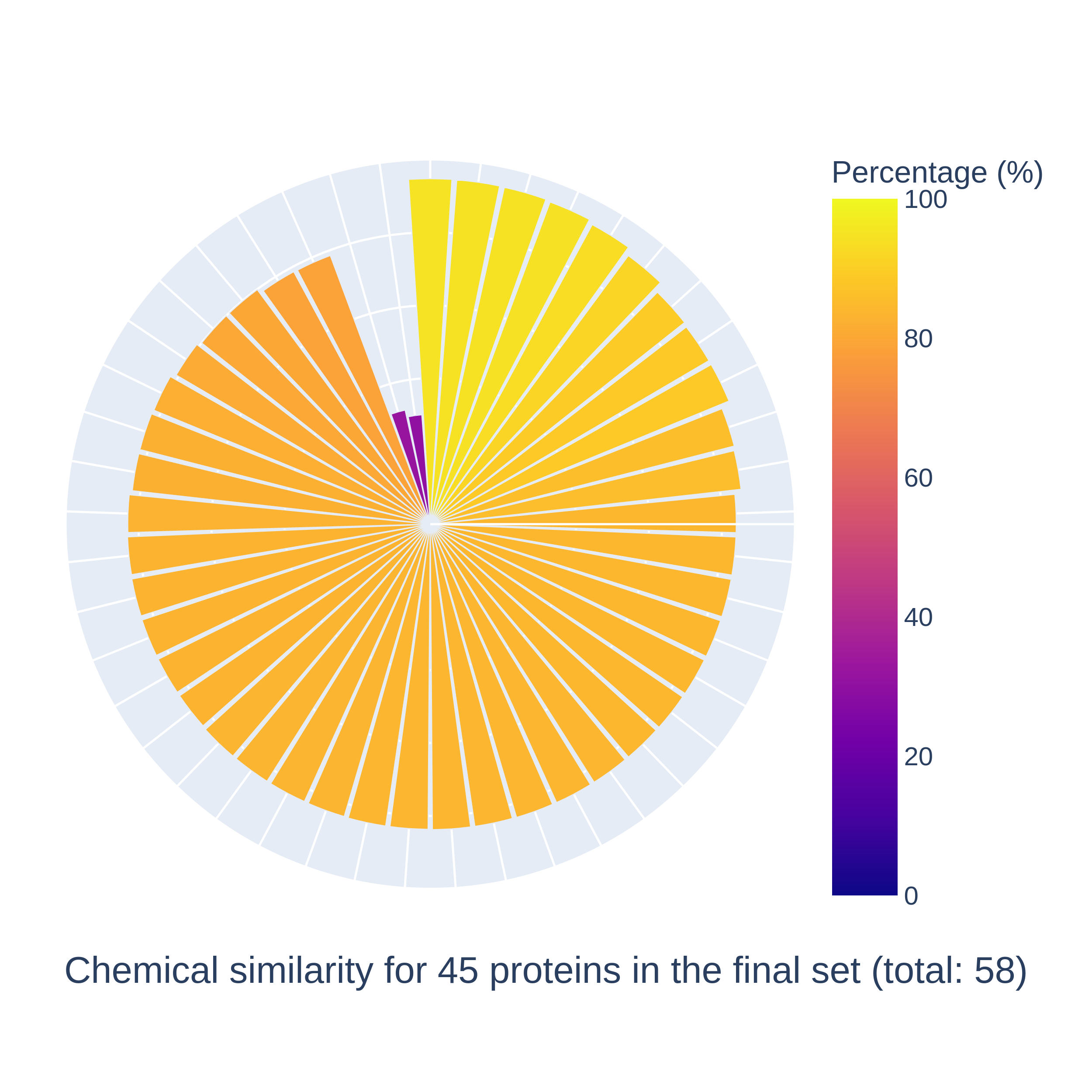

Supplement: Supplementary file 6 — Supplementary Data 3 [file 42003_2023_5076_MOESM6_ESM.zip › 6VXX_A_whole/plots/6VXX_A_chemSim.png]

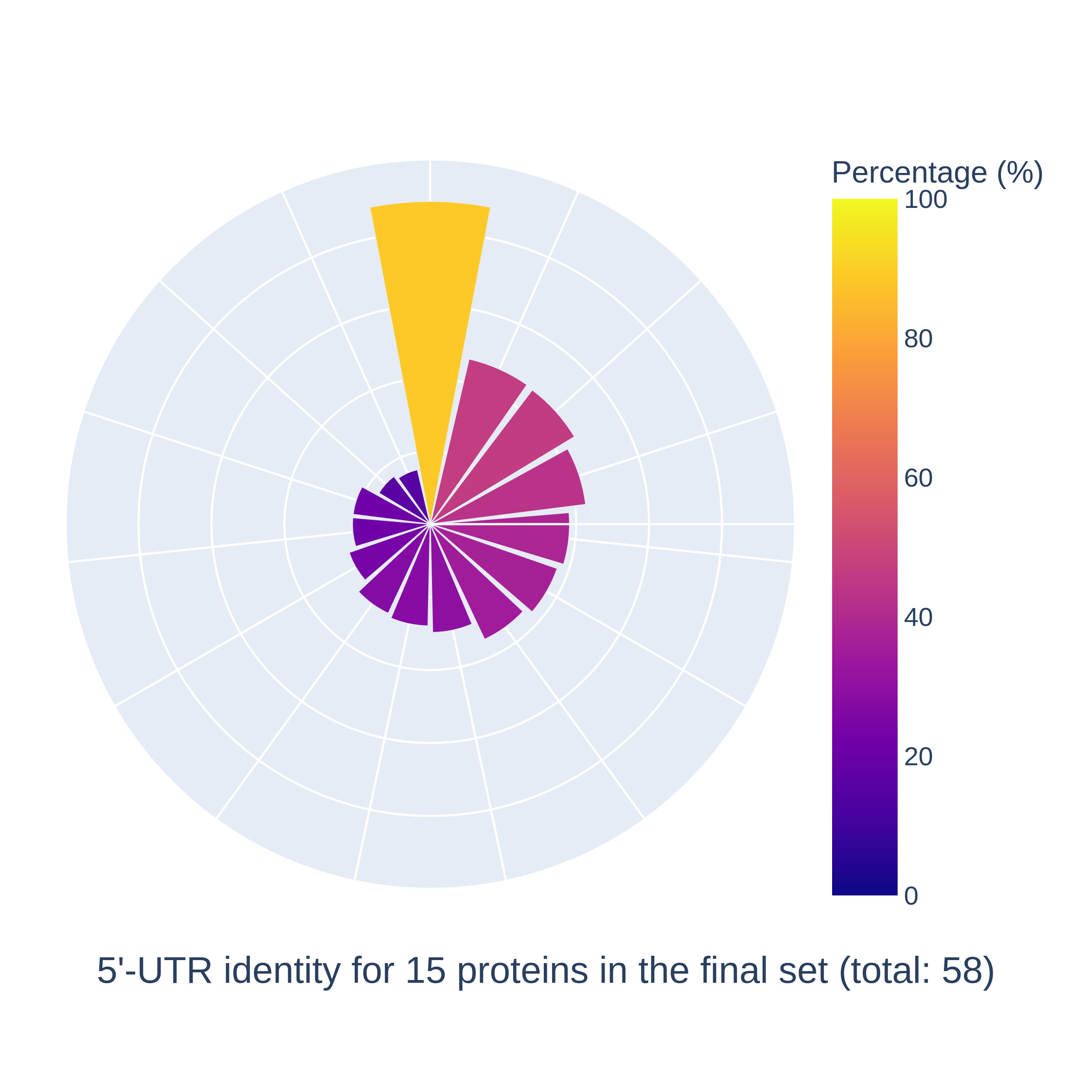

Supplement: Supplementary file 6 — Supplementary Data 3 [file 42003_2023_5076_MOESM6_ESM.zip › 6VXX_A_whole/plots/6VXX_A_5UTR-identity.png]

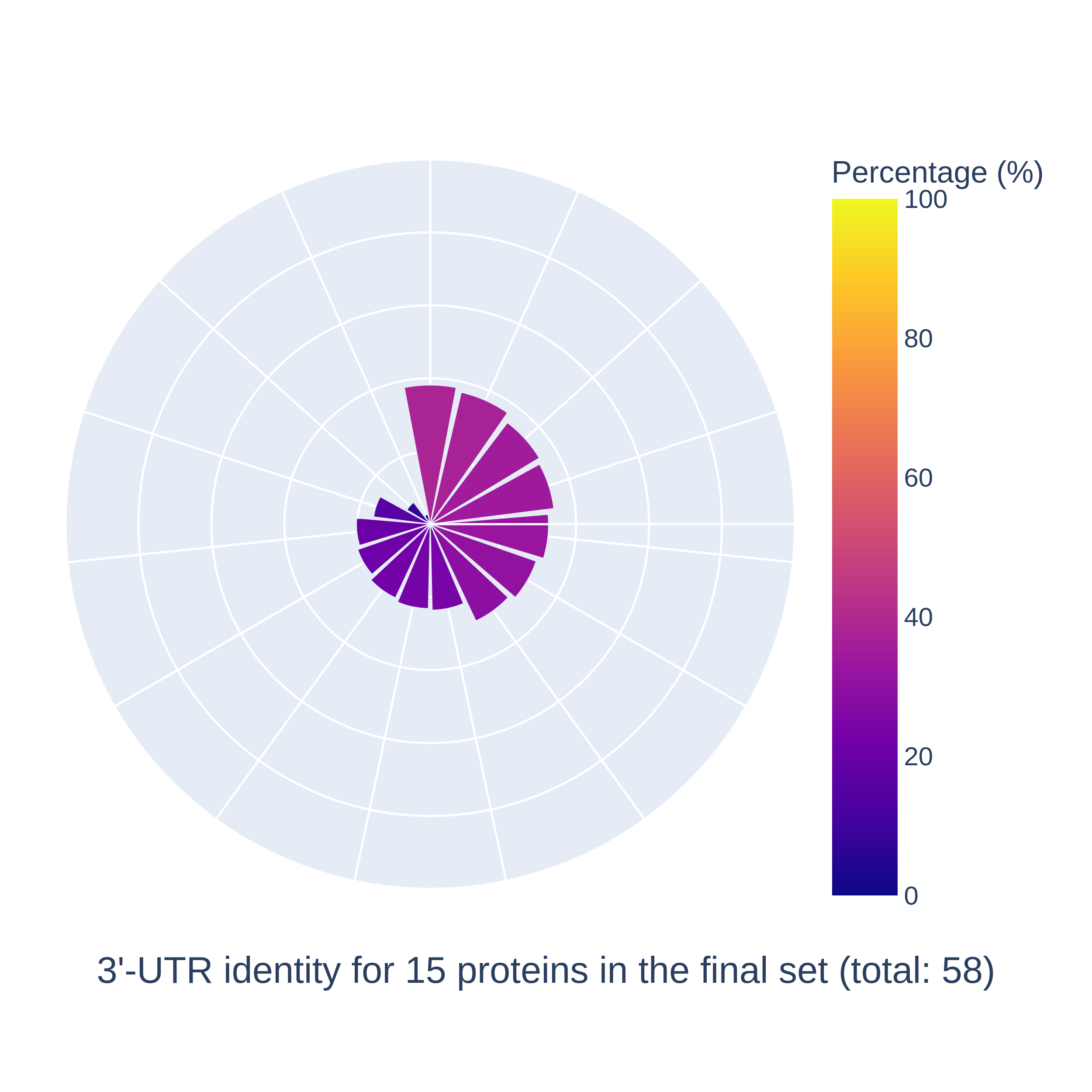

Supplement: Supplementary file 6 — Supplementary Data 3 [file 42003_2023_5076_MOESM6_ESM.zip › 6VXX_A_whole/plots/6VXX_A_3UTR-identity.png]

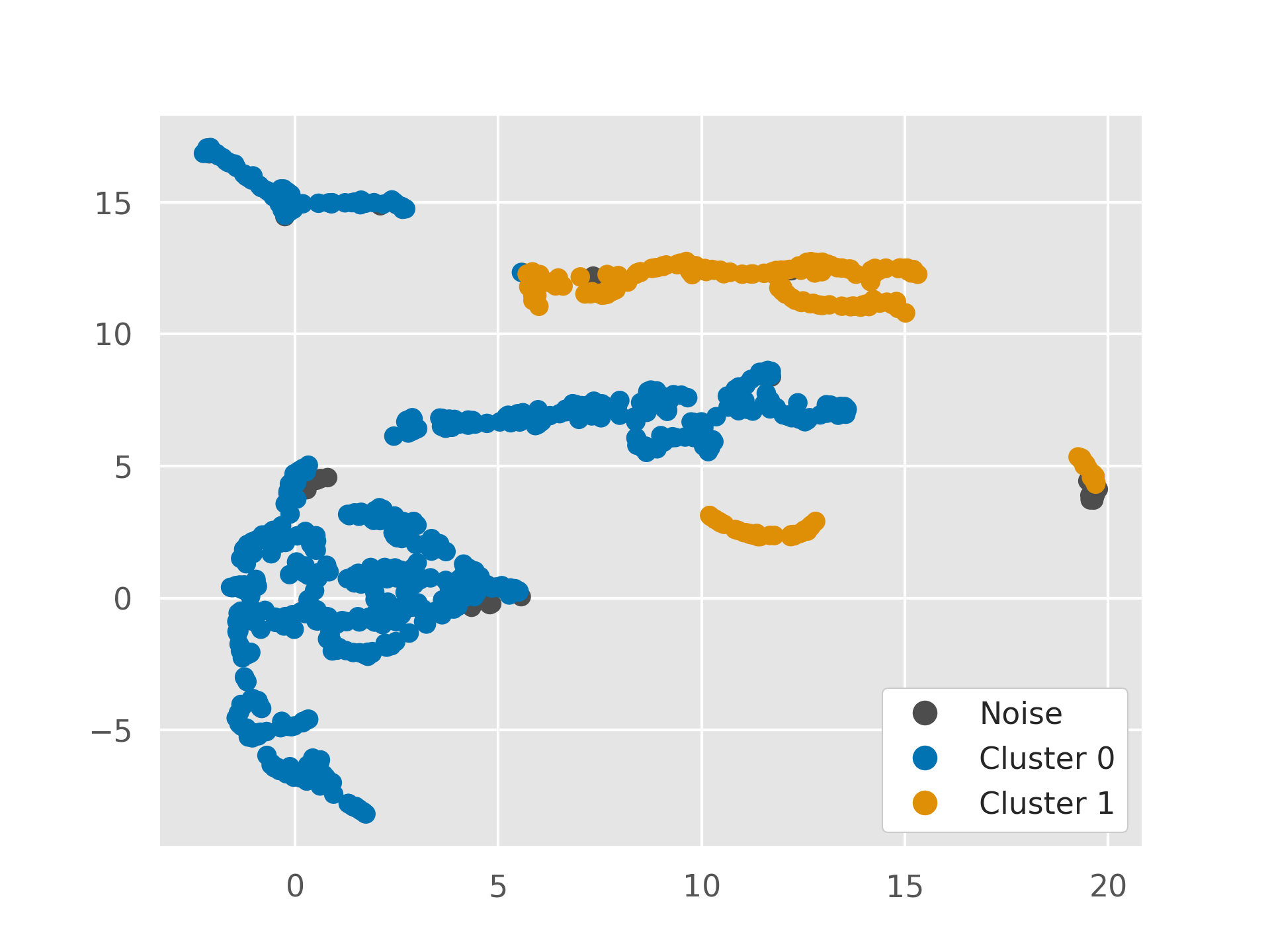

Supplement: Supplementary file 6 — Supplementary Data 3 [file 42003_2023_5076_MOESM6_ESM.zip › 6VXX_A_whole/plots/6VXX_A-clusters-initial.png]

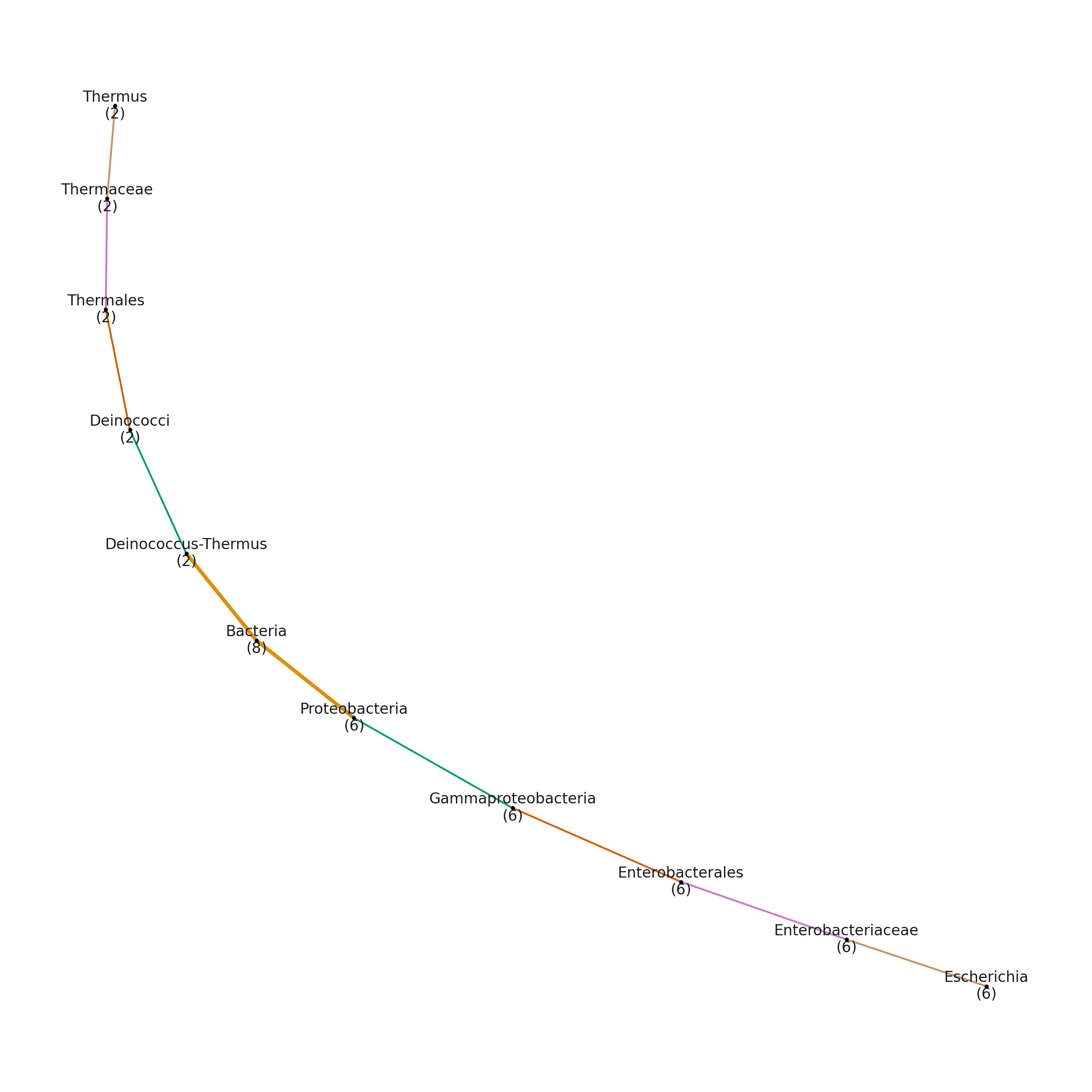

Supplement: Supplementary file 6 — Supplementary Data 3 [file 42003_2023_5076_MOESM6_ESM.zip › 6VXX_A_whole/plots/6VXX_A-Bacteria-tree.png]

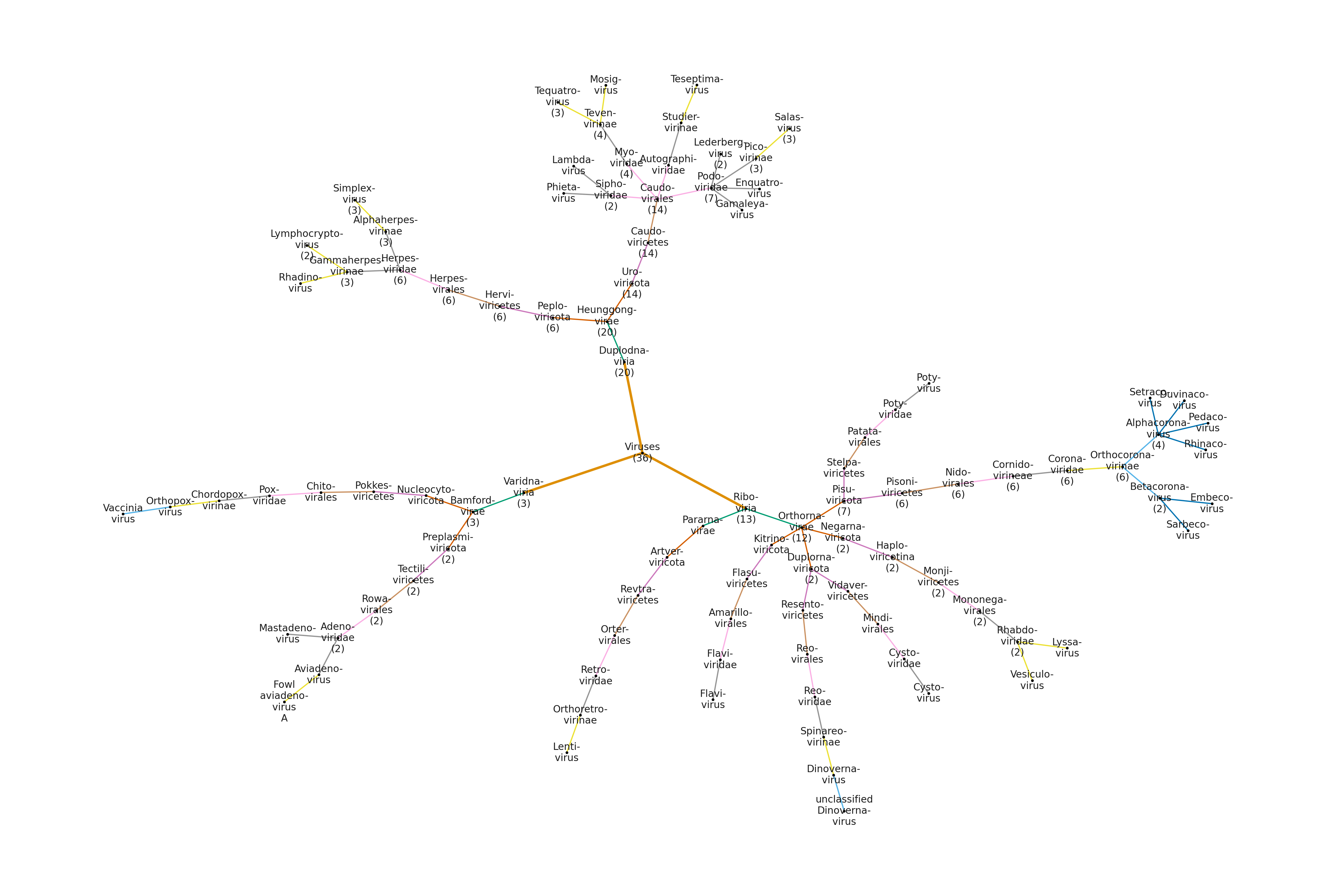

Supplement: Supplementary file 6 — Supplementary Data 3 [file 42003_2023_5076_MOESM6_ESM.zip › 6VXX_A_whole/plots/6VXX_A-Viruses-tree.png]

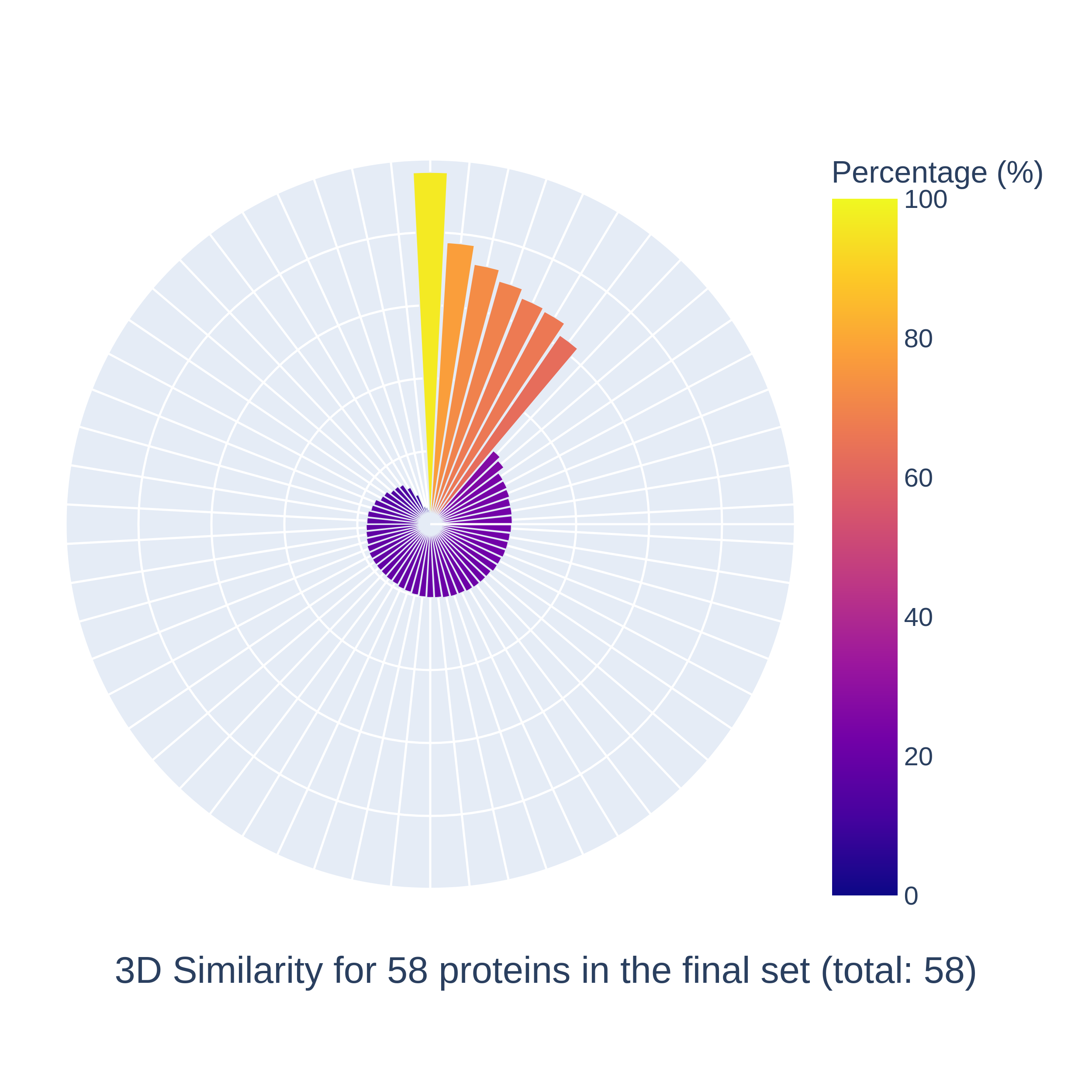

Supplement: Supplementary file 6 — Supplementary Data 3 [file 42003_2023_5076_MOESM6_ESM.zip › 6VXX_A_whole/plots/6VXX_A_3D-score.png]

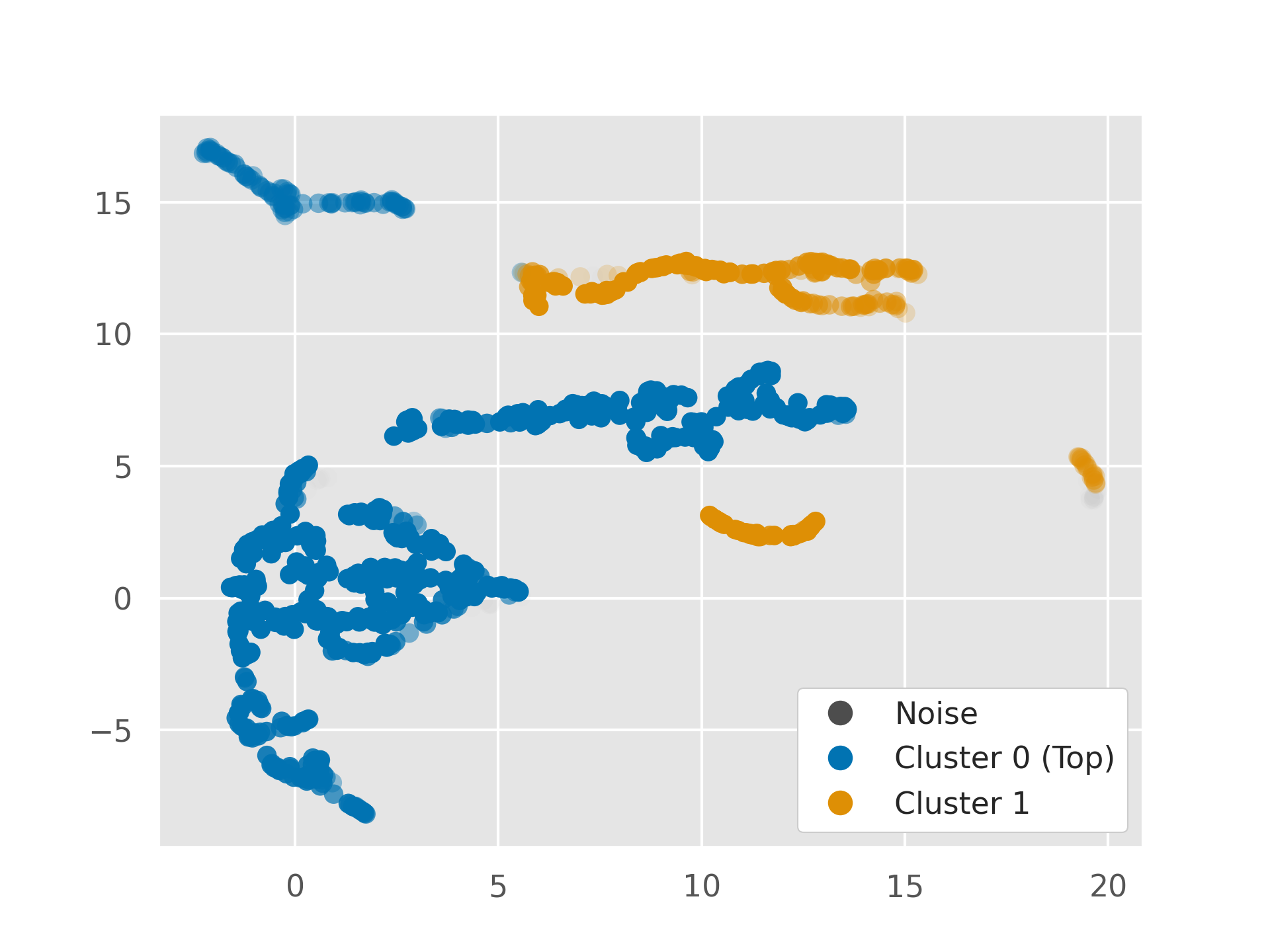

Supplement: Supplementary file 6 — Supplementary Data 3 [file 42003_2023_5076_MOESM6_ESM.zip › 6VXX_A_whole/plots/6VXX_A-clusters.png]

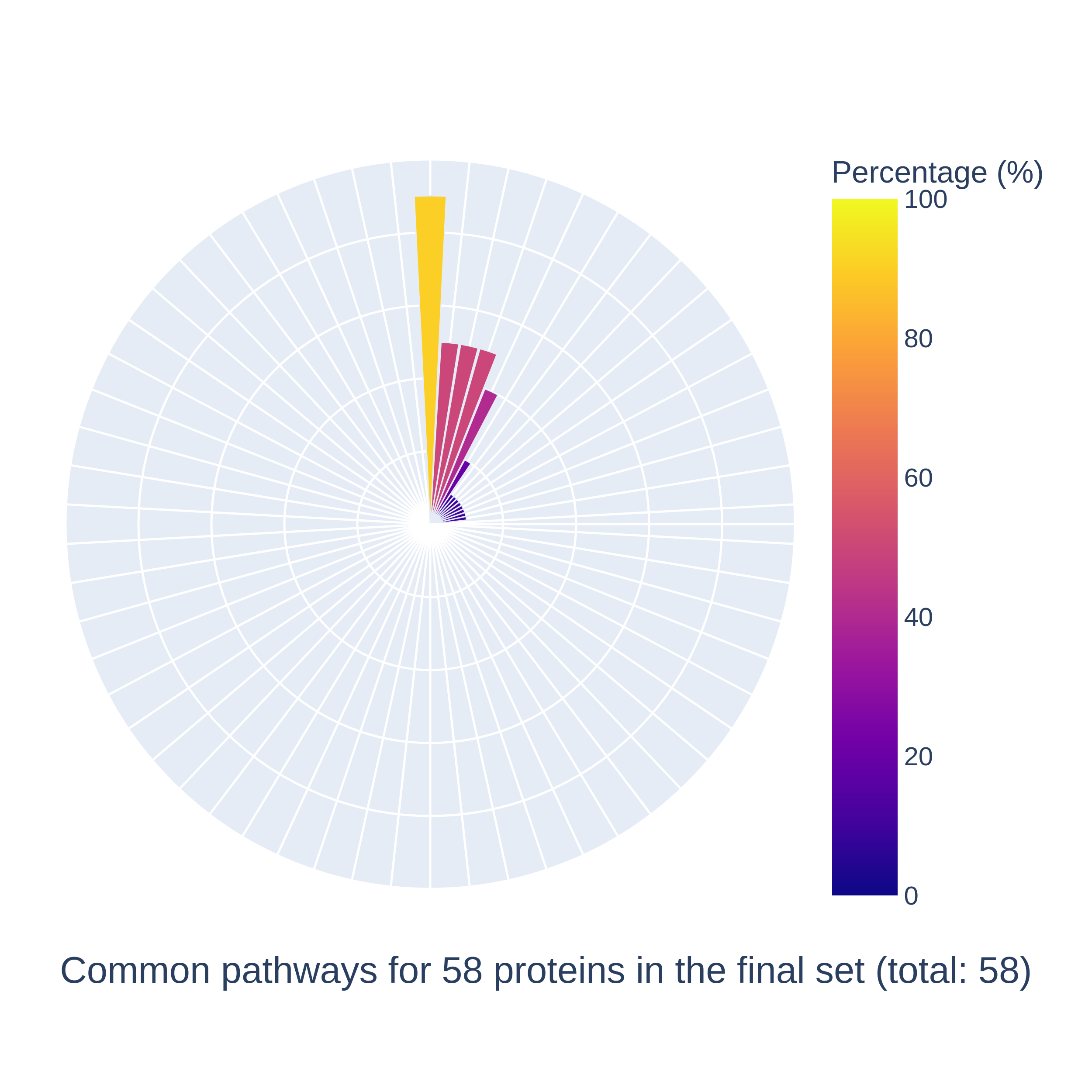

Supplement: Supplementary file 6 — Supplementary Data 3 [file 42003_2023_5076_MOESM6_ESM.zip › 6VXX_A_whole/plots/6VXX_A_biologicalProcessSim.png]

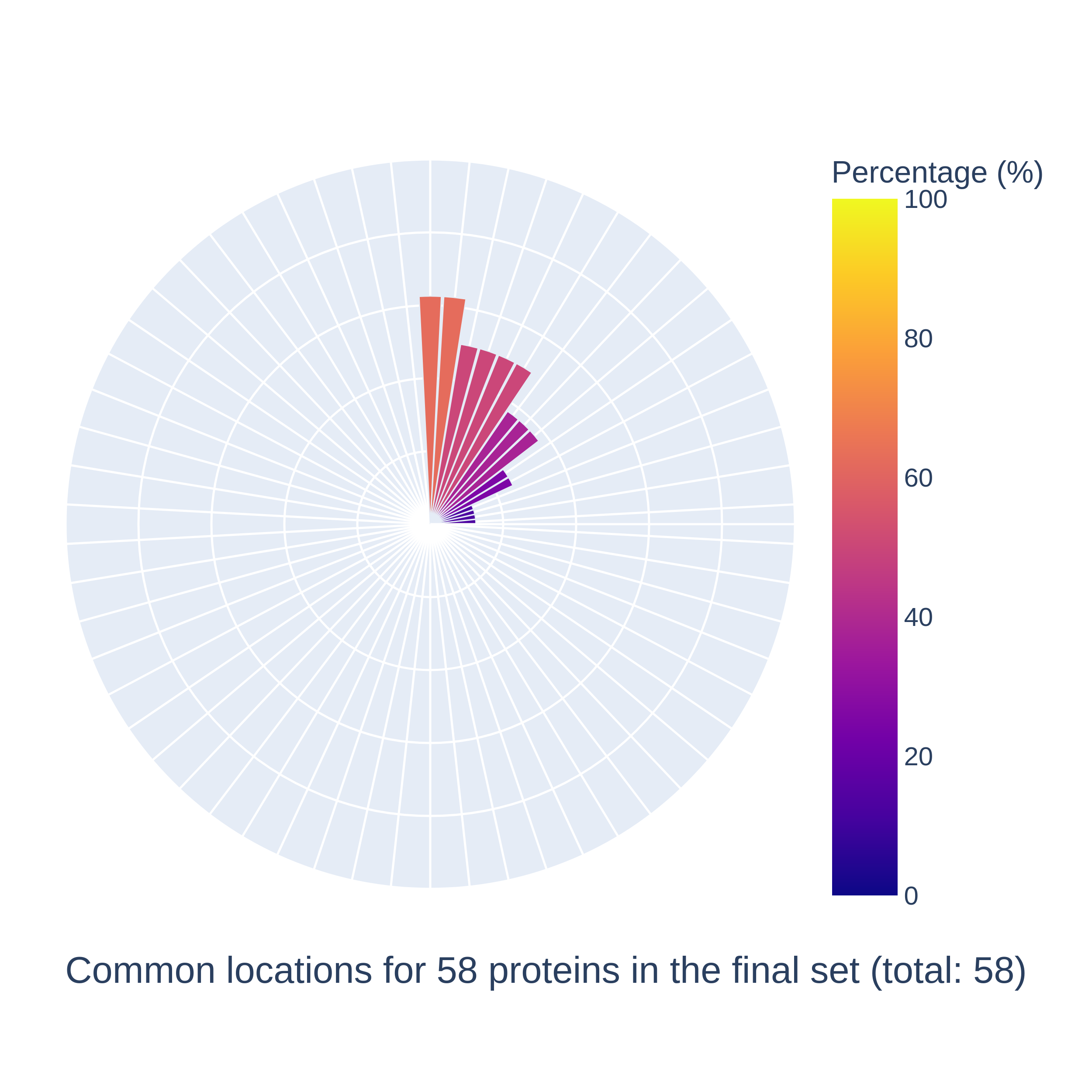

Supplement: Supplementary file 6 — Supplementary Data 3 [file 42003_2023_5076_MOESM6_ESM.zip › 6VXX_A_whole/plots/6VXX_A_cellularComponentSim.png]

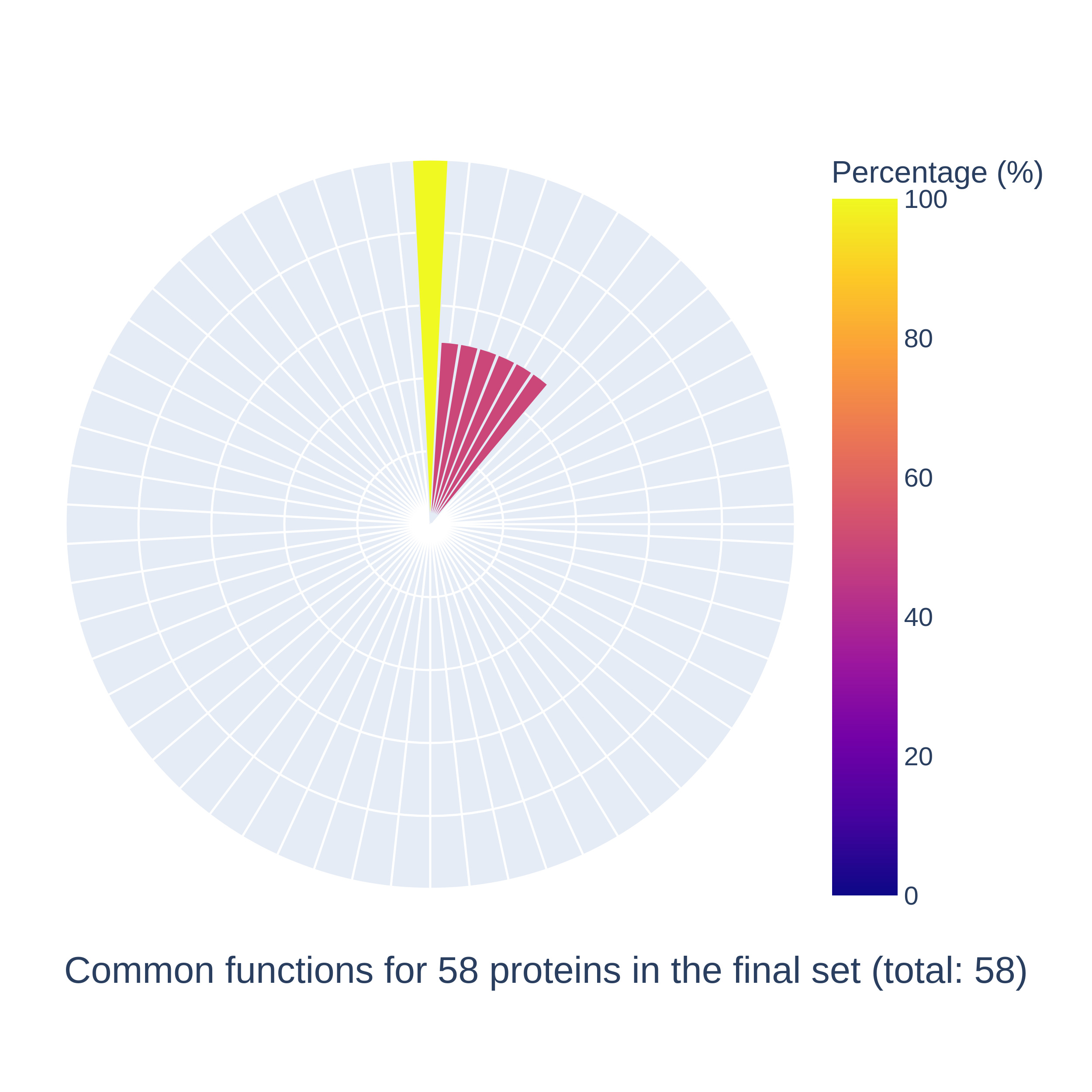

Supplement: Supplementary file 6 — Supplementary Data 3 [file 42003_2023_5076_MOESM6_ESM.zip › 6VXX_A_whole/plots/6VXX_A_molecularFunctionSim.png]

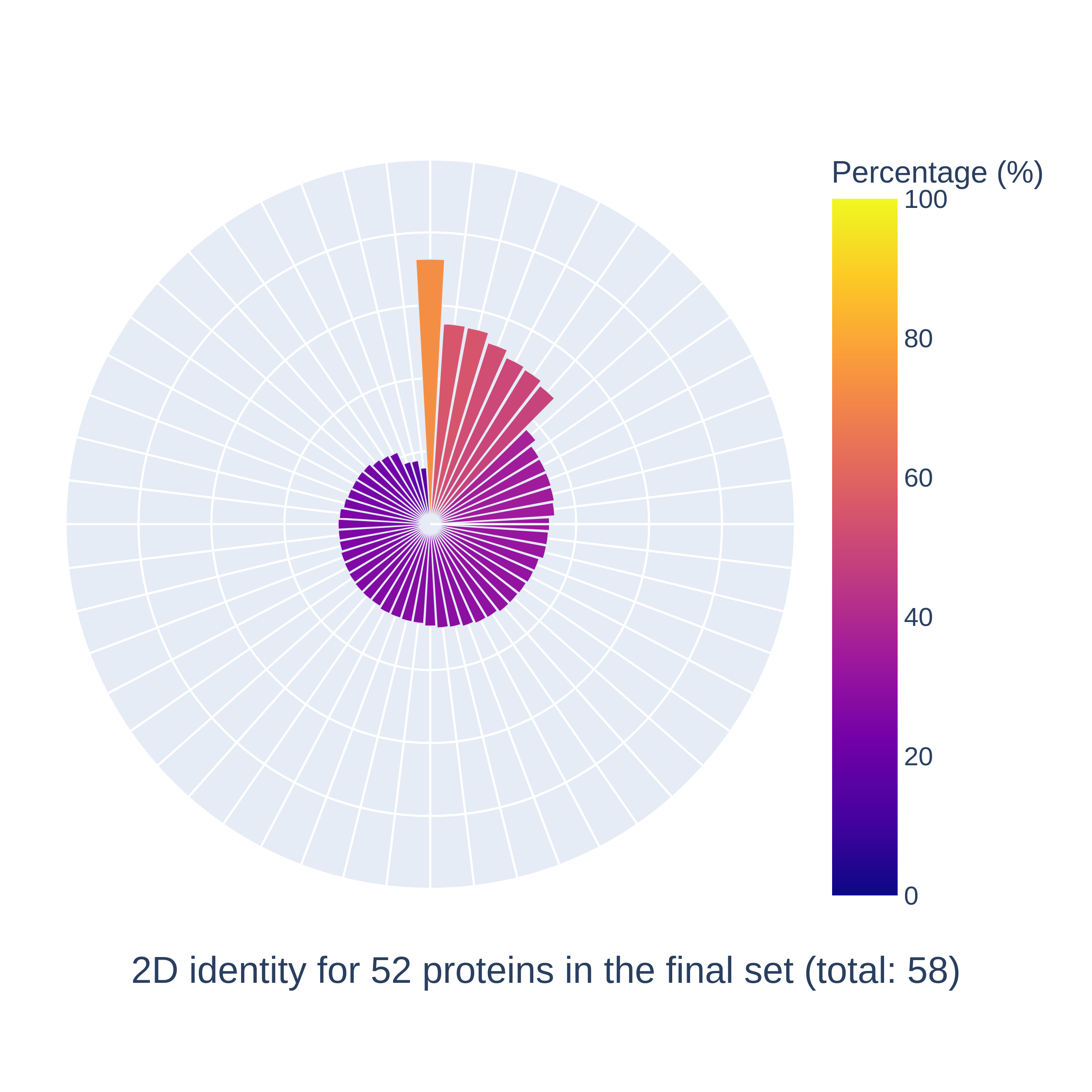

Supplement: Supplementary file 6 — Supplementary Data 3 [file 42003_2023_5076_MOESM6_ESM.zip › 6VXX_A_whole/plots/6VXX_A_2D-identity.png]

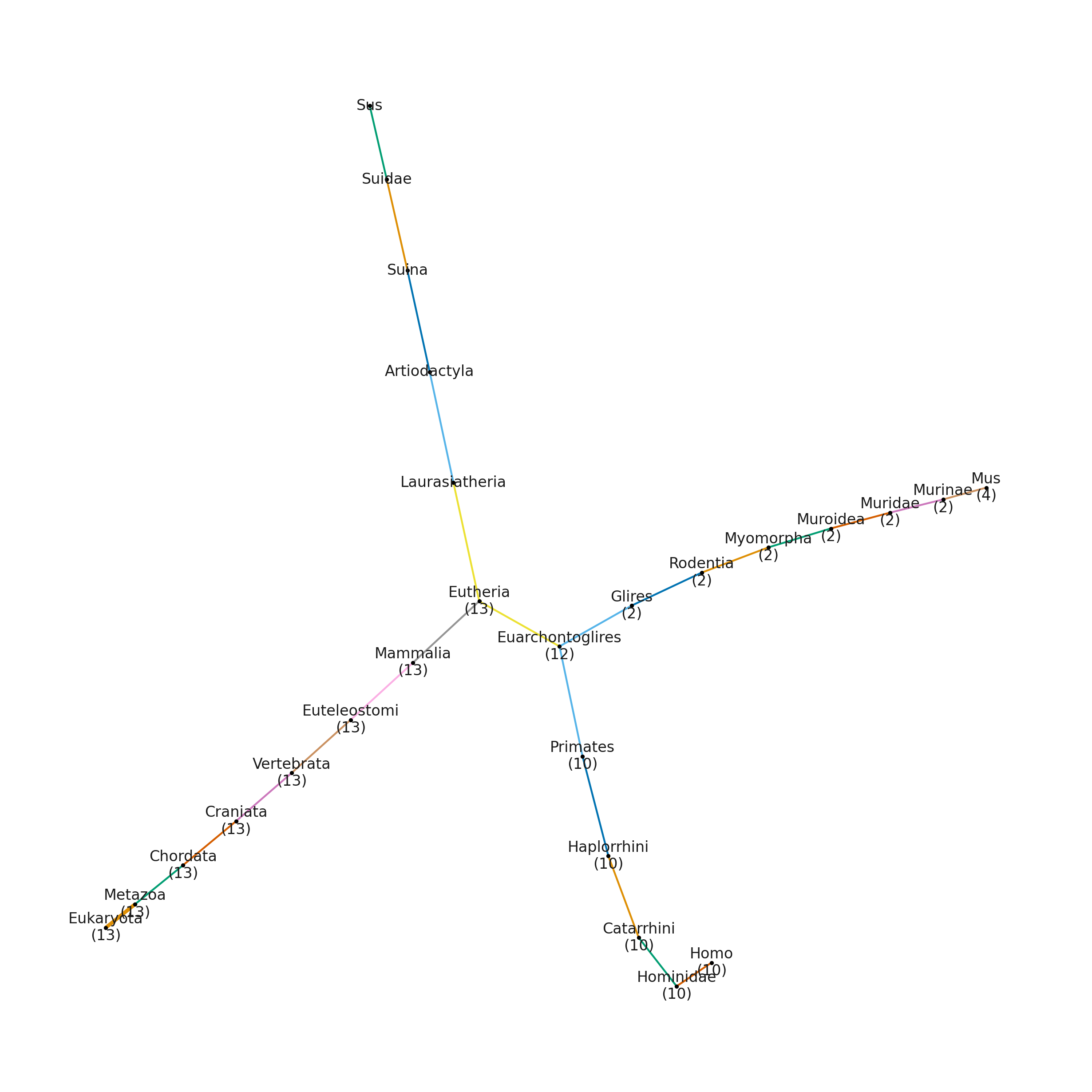

Supplement: Supplementary file 6 — Supplementary Data 3 [file 42003_2023_5076_MOESM6_ESM.zip › 6VXX_A_whole/plots/6VXX_A-Eukaryota-tree.png]

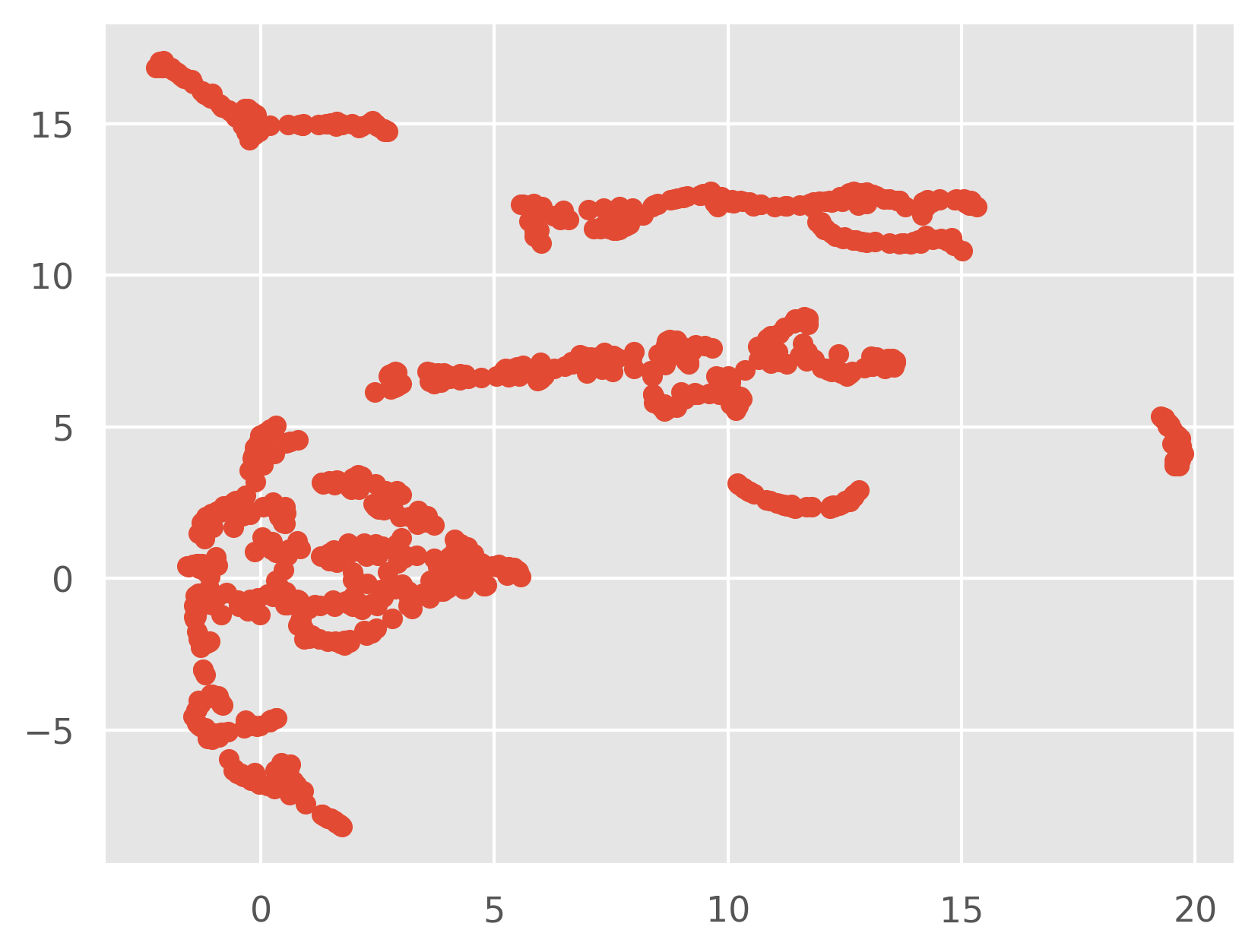

Supplement: Supplementary file 6 — Supplementary Data 3 [file 42003_2023_5076_MOESM6_ESM.zip › 6VXX_A_whole/plots/6VXX_A-UMAP-.png]

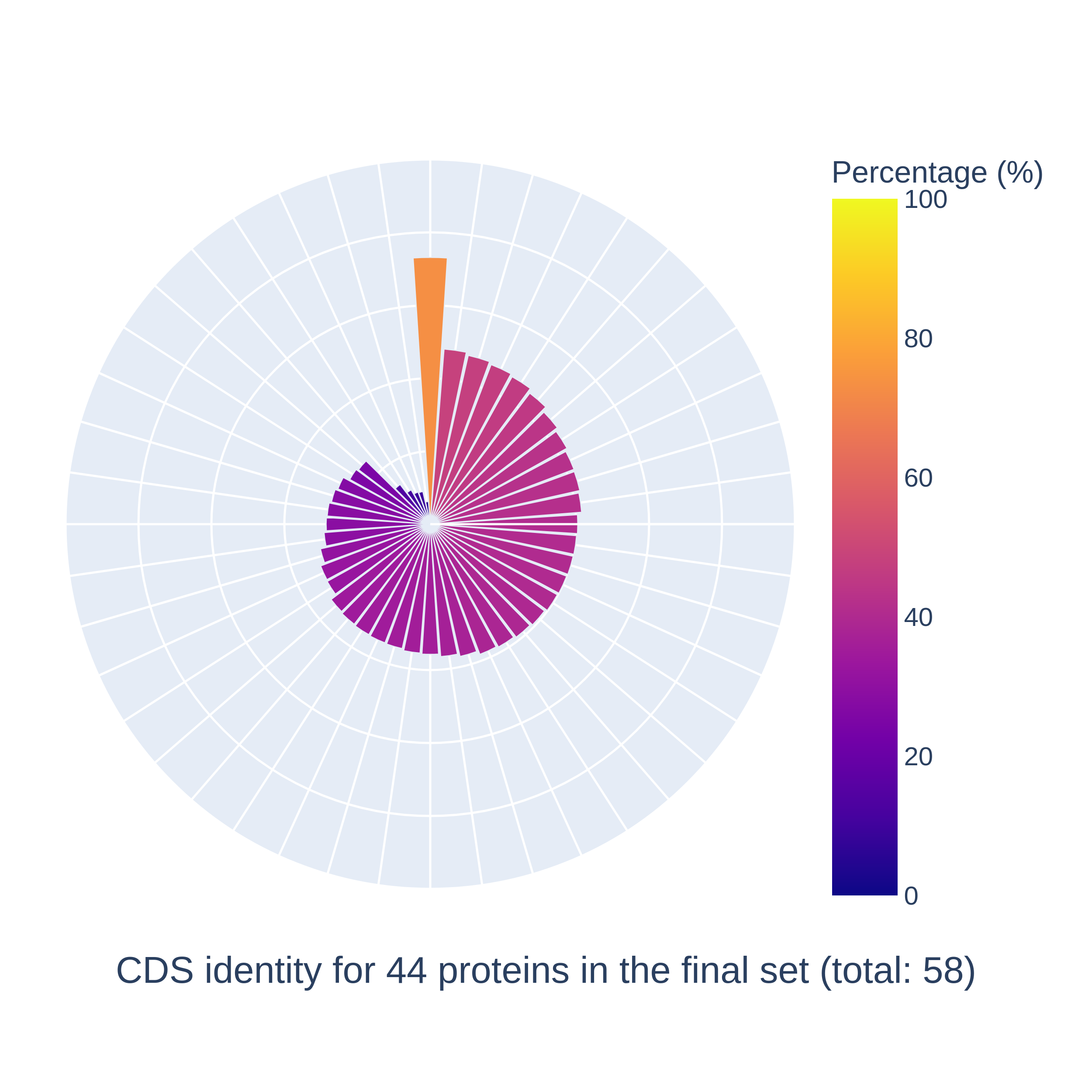

Supplement: Supplementary file 6 — Supplementary Data 3 [file 42003_2023_5076_MOESM6_ESM.zip › 6VXX_A_whole/plots/6VXX_A_CDS-identity.png]

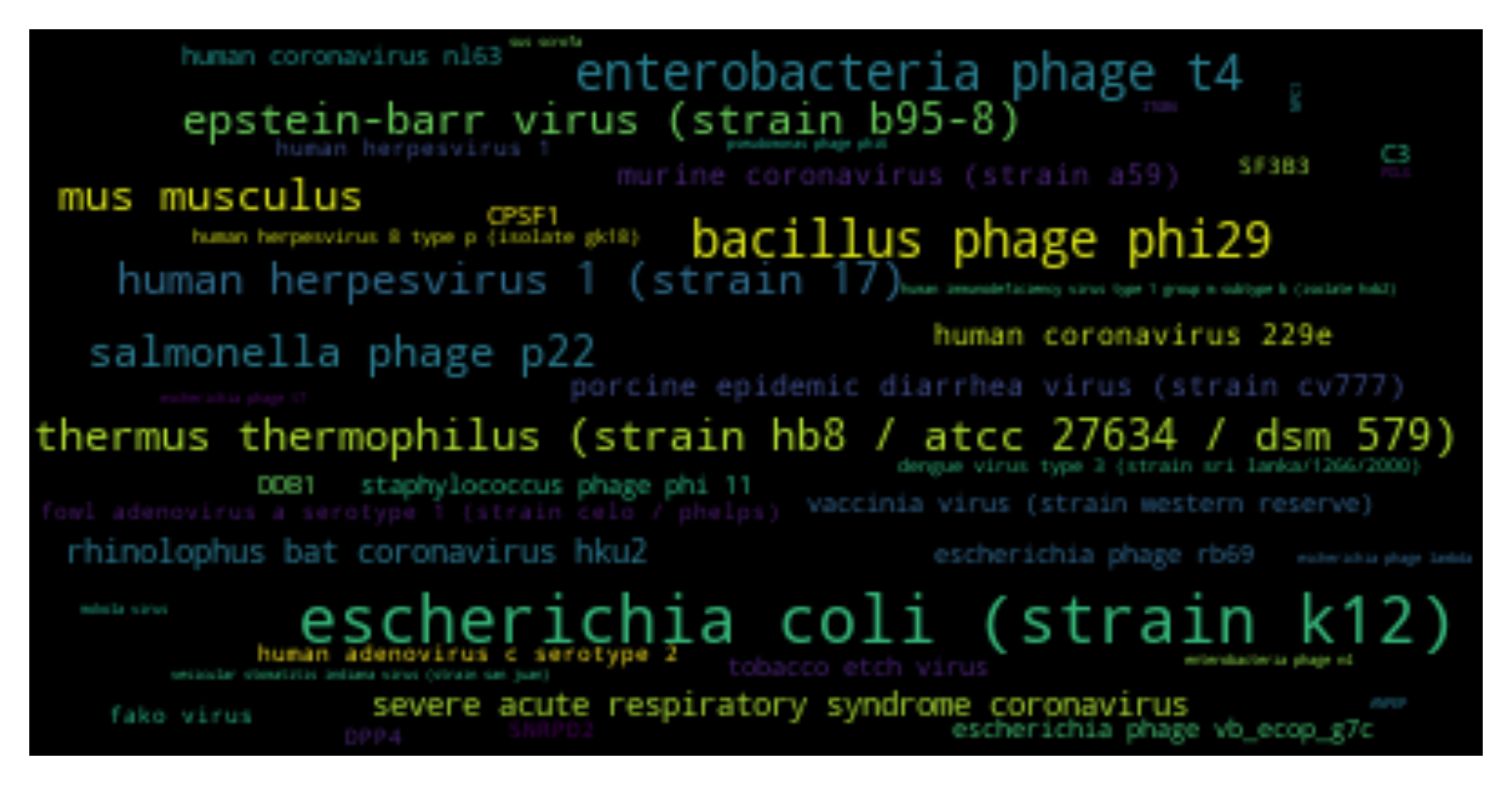

Supplement: Supplementary file 6 — Supplementary Data 3 [file 42003_2023_5076_MOESM6_ESM.zip › 6VXX_A_whole/plots/6VXX_A-wordcloud.png]

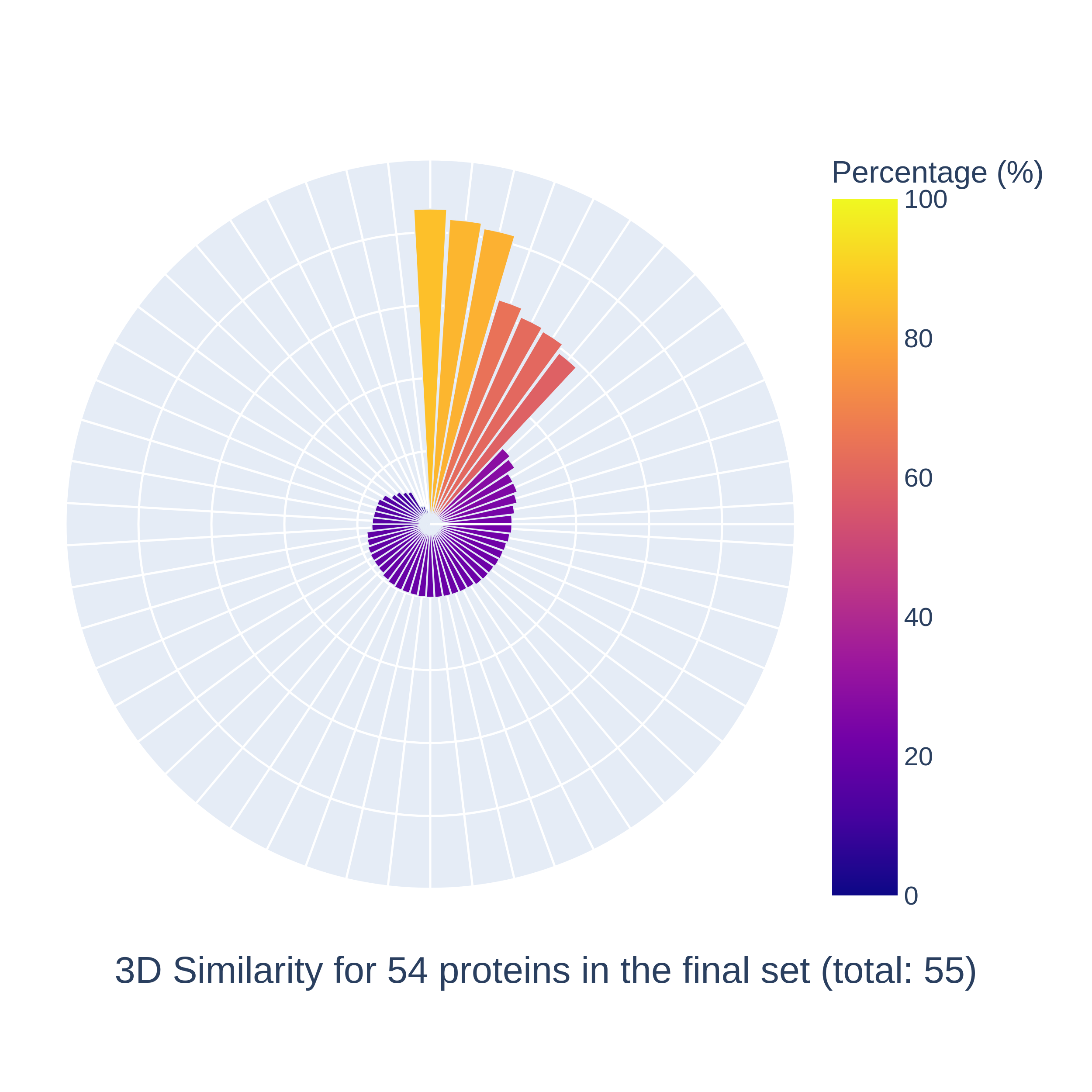

Supplement: Supplementary file 6 — Supplementary Data 3 [file 42003_2023_5076_MOESM6_ESM.zip › 6VXXp_A_whole/plots/6VXXp_A_3D-score.png]

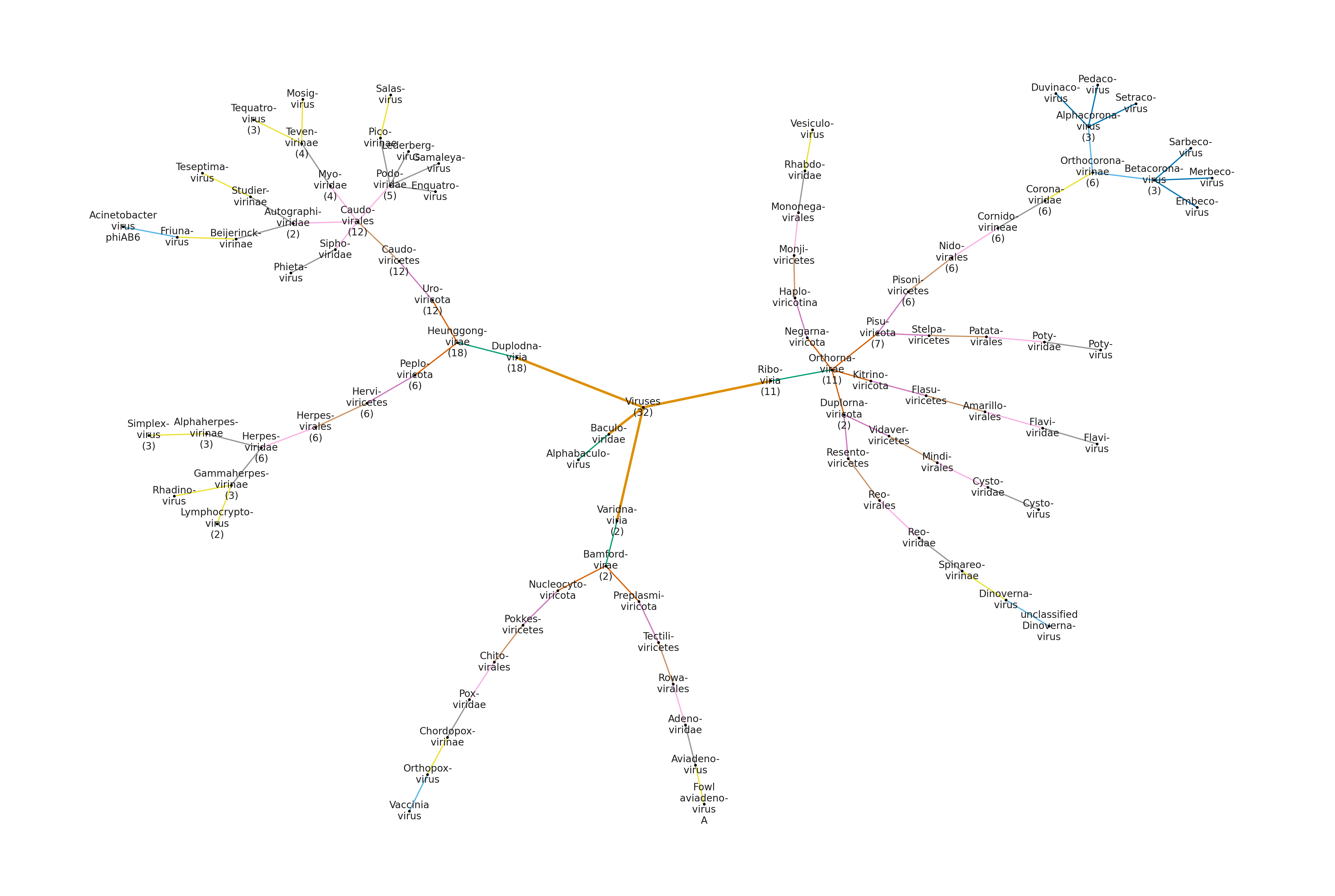

Supplement: Supplementary file 6 — Supplementary Data 3 [file 42003_2023_5076_MOESM6_ESM.zip › 6VXXp_A_whole/plots/6VXXp_A-Viruses-tree.png]

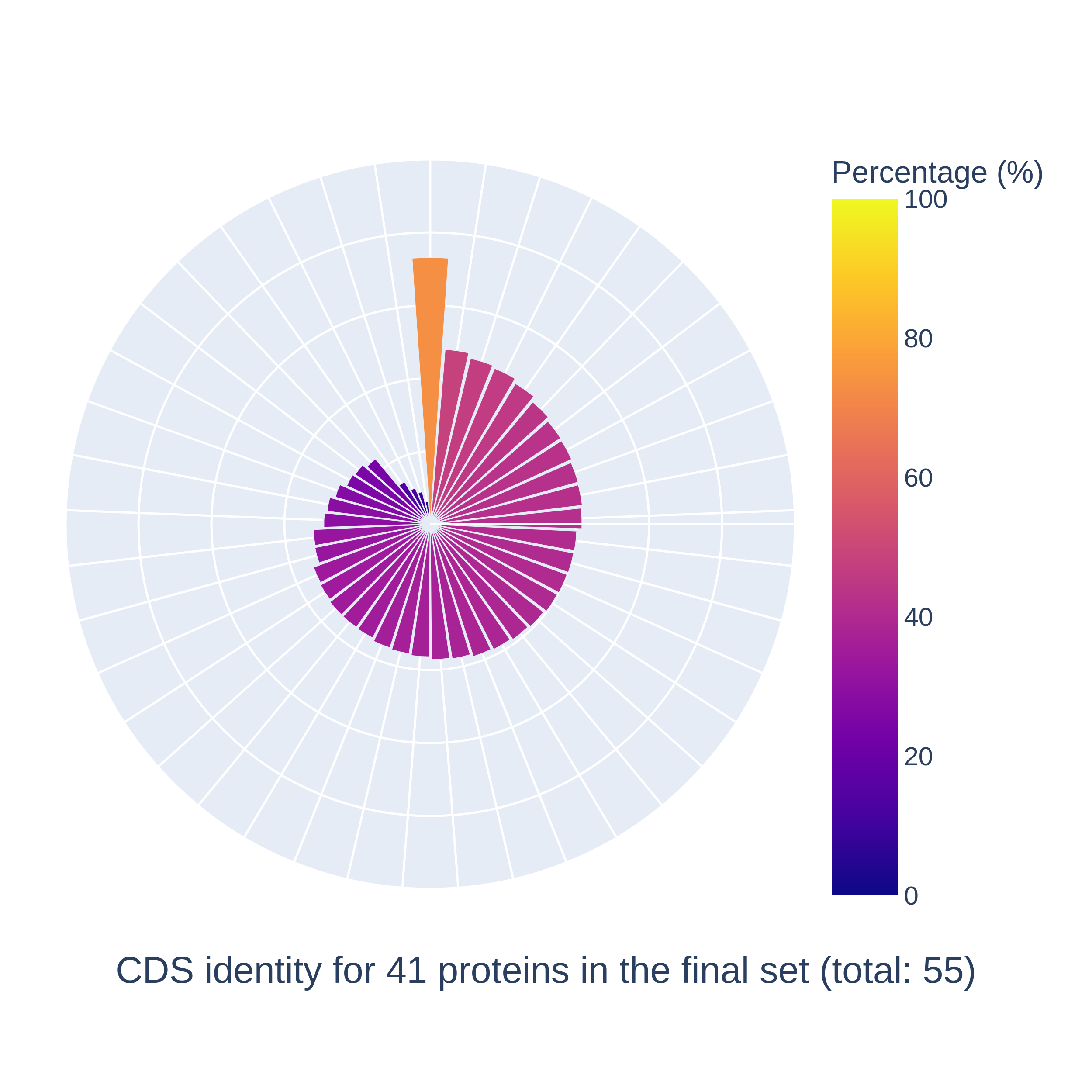

Supplement: Supplementary file 6 — Supplementary Data 3 [file 42003_2023_5076_MOESM6_ESM.zip › 6VXXp_A_whole/plots/6VXXp_A_CDS-identity.png]

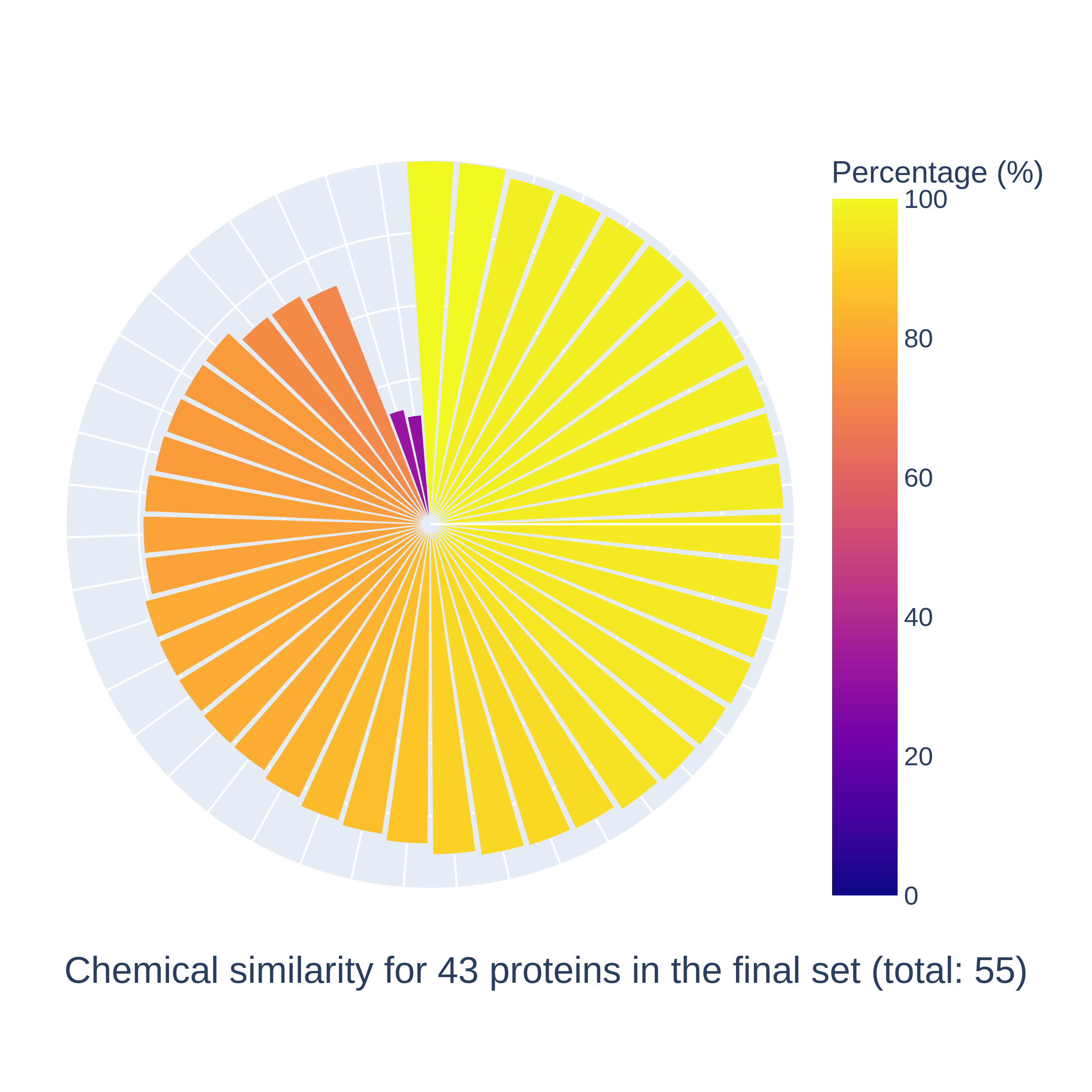

Supplement: Supplementary file 6 — Supplementary Data 3 [file 42003_2023_5076_MOESM6_ESM.zip › 6VXXp_A_whole/plots/6VXXp_A_chemSim.png]

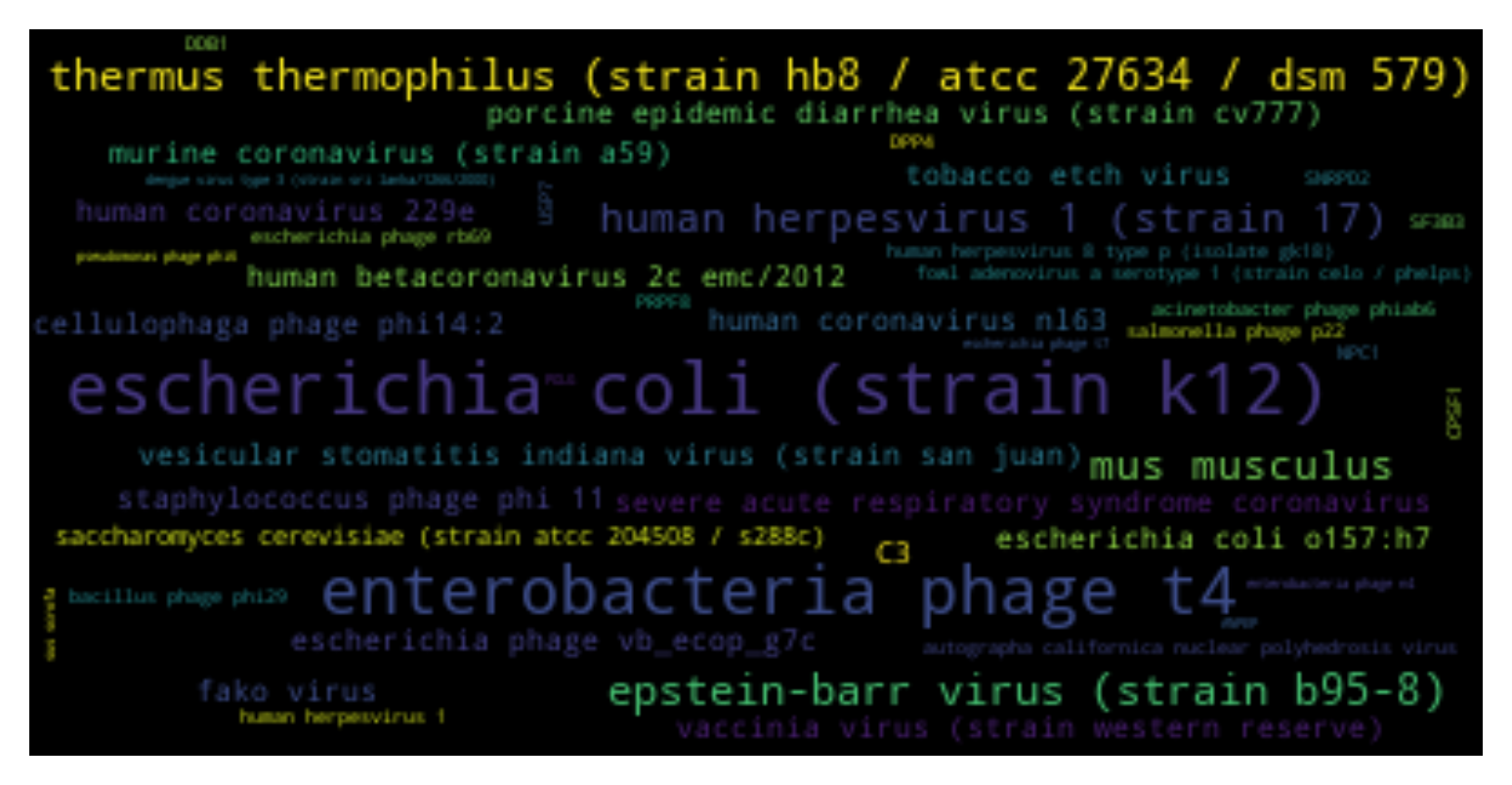

Supplement: Supplementary file 6 — Supplementary Data 3 [file 42003_2023_5076_MOESM6_ESM.zip › 6VXXp_A_whole/plots/6VXXp_A-wordcloud.png]

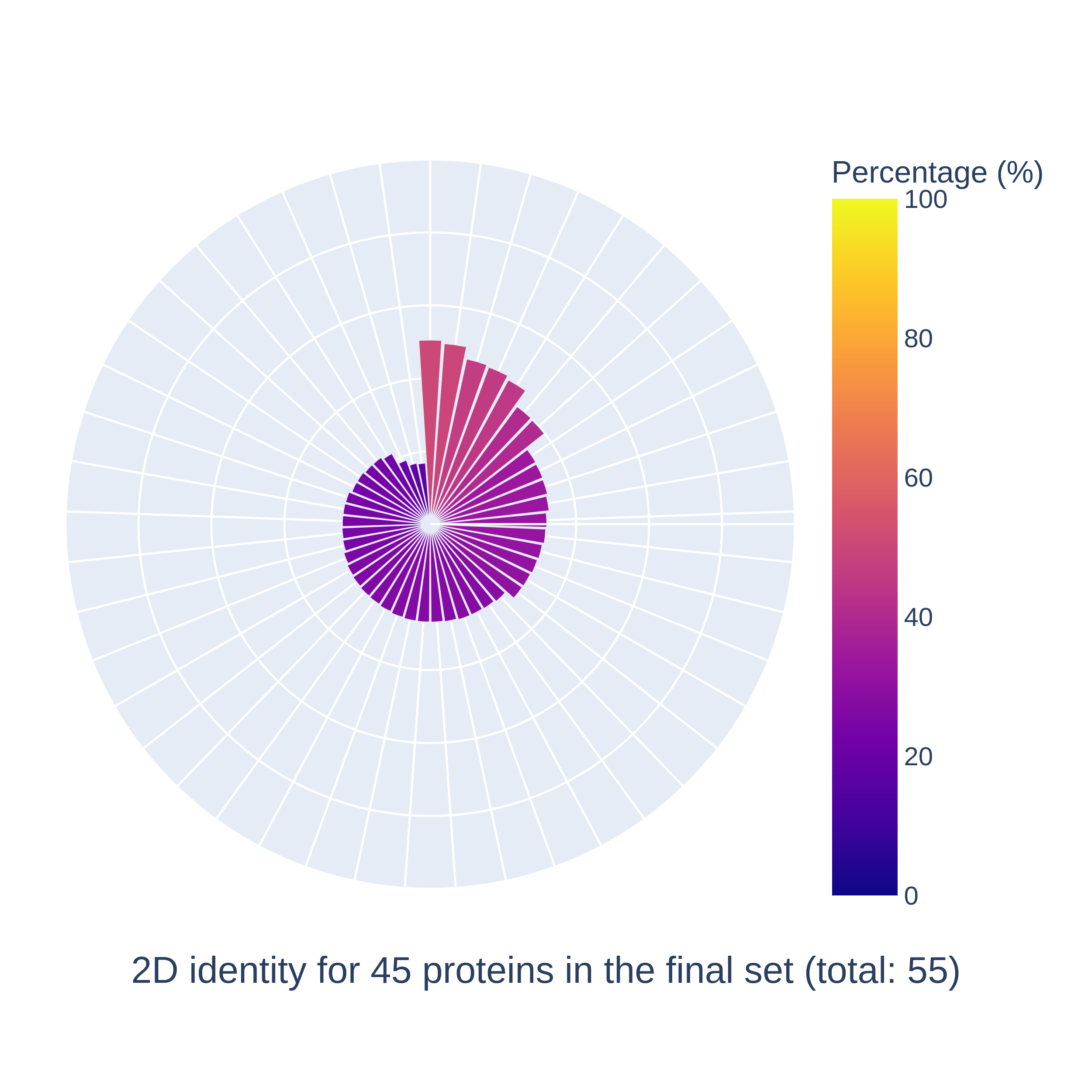

Supplement: Supplementary file 6 — Supplementary Data 3 [file 42003_2023_5076_MOESM6_ESM.zip › 6VXXp_A_whole/plots/6VXXp_A_2D-identity.png]

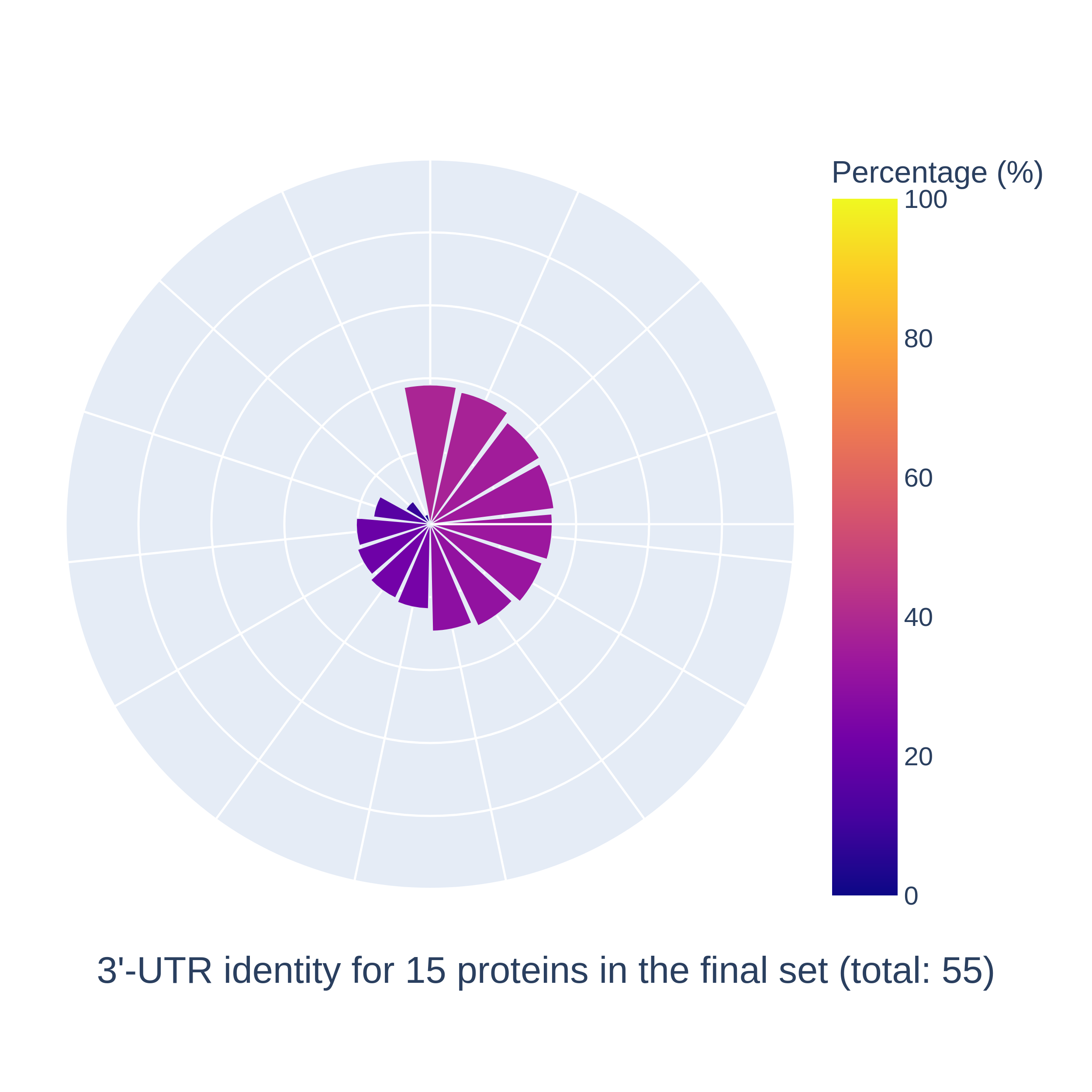

Supplement: Supplementary file 6 — Supplementary Data 3 [file 42003_2023_5076_MOESM6_ESM.zip › 6VXXp_A_whole/plots/6VXXp_A_3UTR-identity.png]

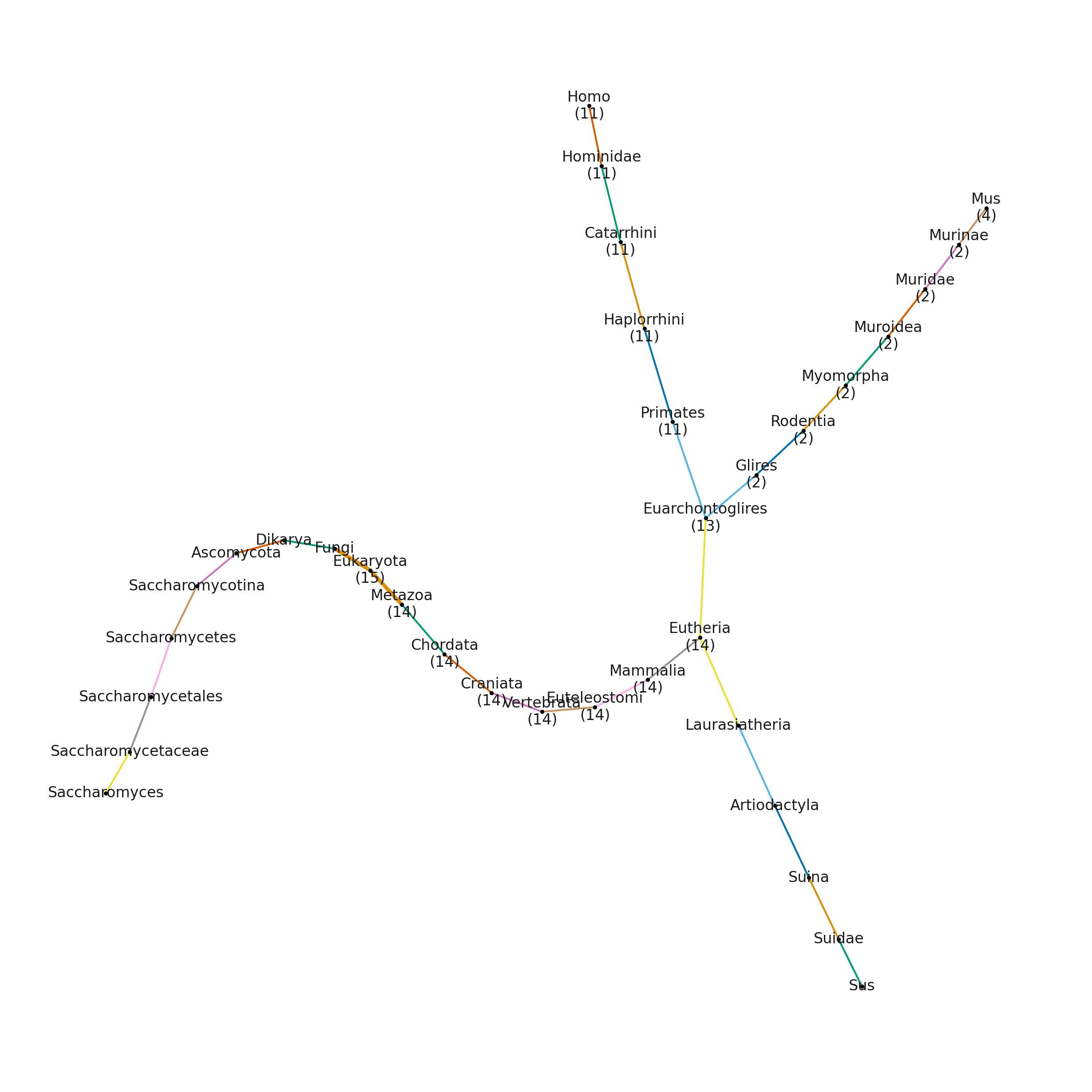

Supplement: Supplementary file 6 — Supplementary Data 3 [file 42003_2023_5076_MOESM6_ESM.zip › 6VXXp_A_whole/plots/6VXXp_A-Eukaryota-tree.png]

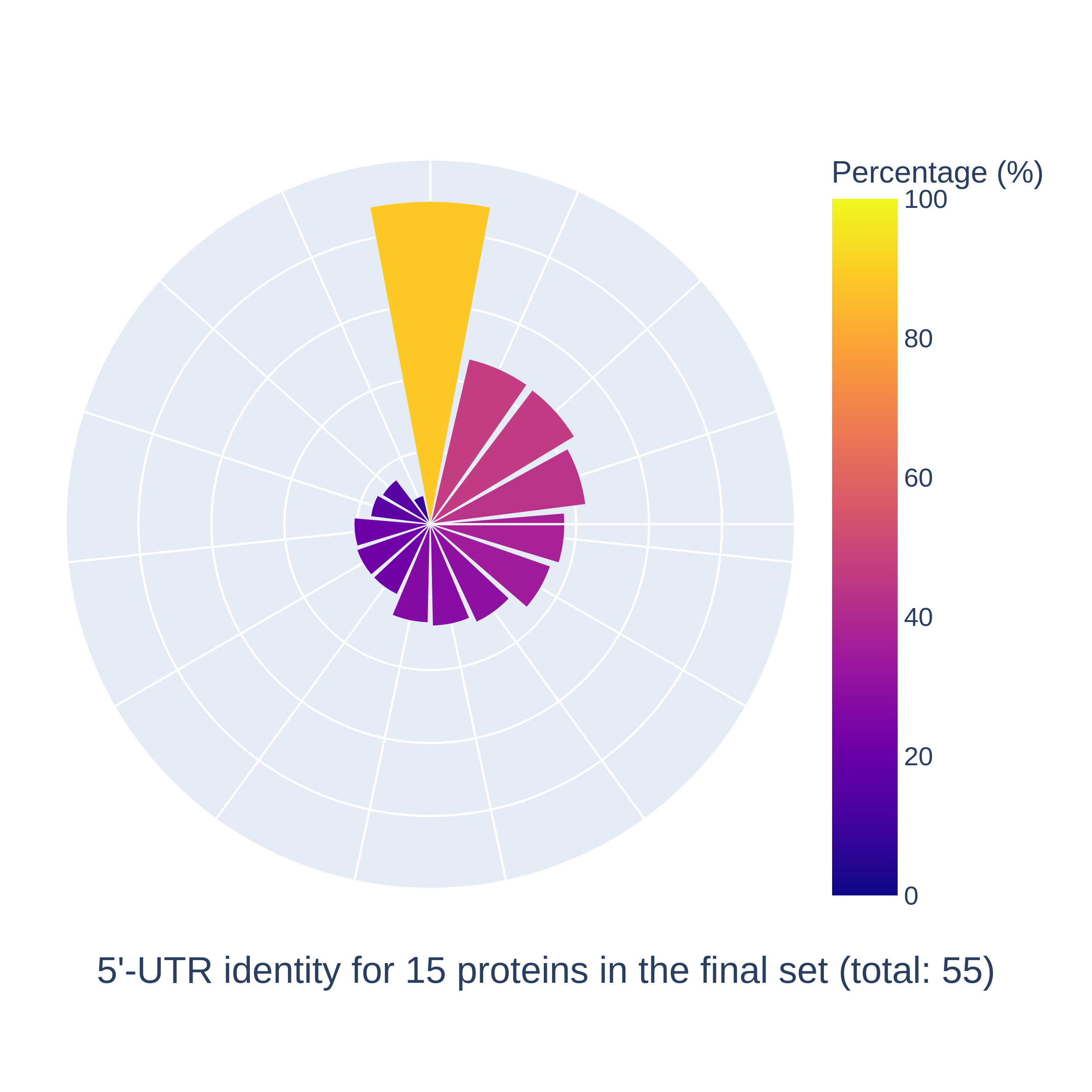

Supplement: Supplementary file 6 — Supplementary Data 3 [file 42003_2023_5076_MOESM6_ESM.zip › 6VXXp_A_whole/plots/6VXXp_A_5UTR-identity.png]

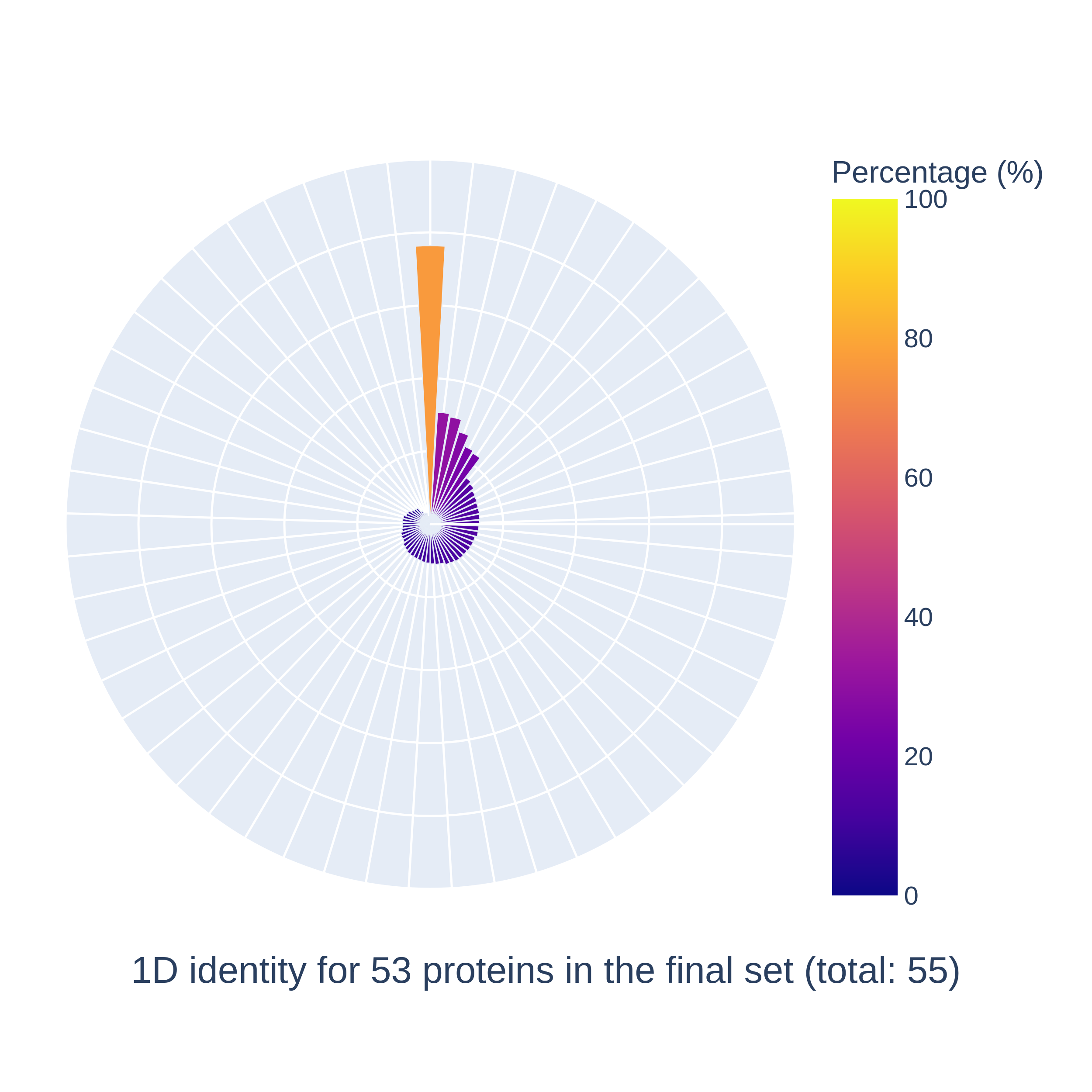

Supplement: Supplementary file 6 — Supplementary Data 3 [file 42003_2023_5076_MOESM6_ESM.zip › 6VXXp_A_whole/plots/6VXXp_A_1D-identity.png]

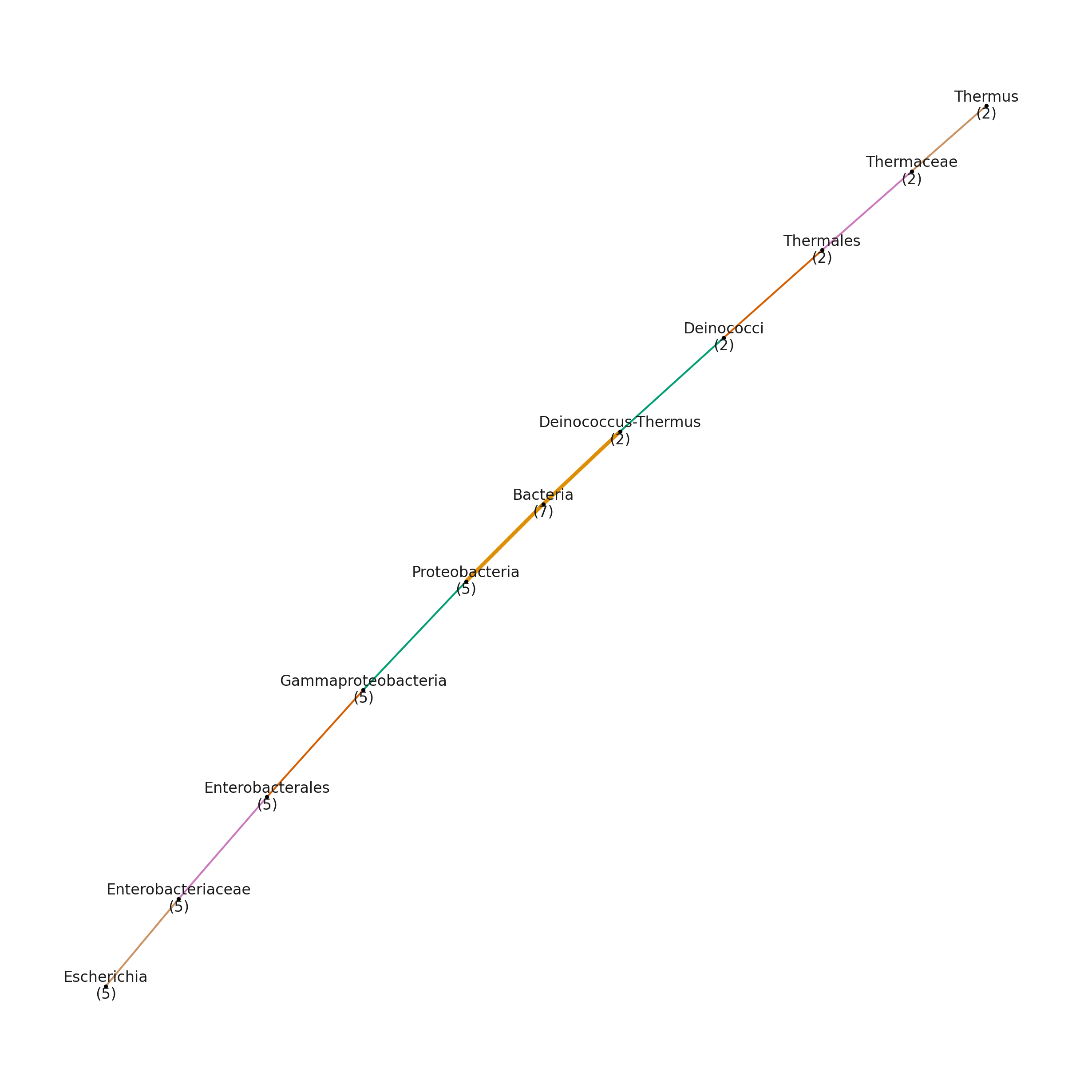

Supplement: Supplementary file 6 — Supplementary Data 3 [file 42003_2023_5076_MOESM6_ESM.zip › 6VXXp_A_whole/plots/6VXXp_A-Bacteria-tree.png]

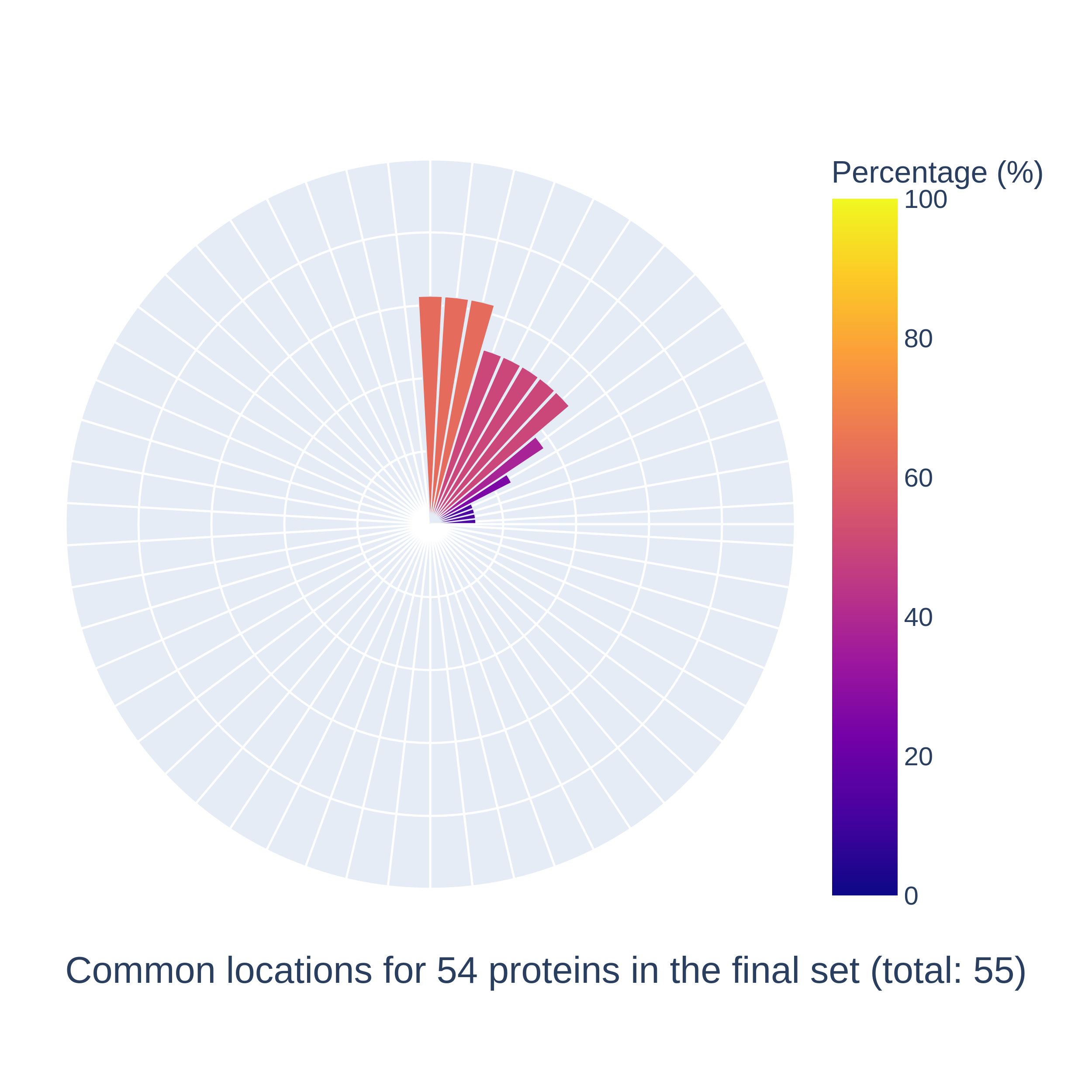

Supplement: Supplementary file 6 — Supplementary Data 3 [file 42003_2023_5076_MOESM6_ESM.zip › 6VXXp_A_whole/plots/6VXXp_A_cellularComponentSim.png]

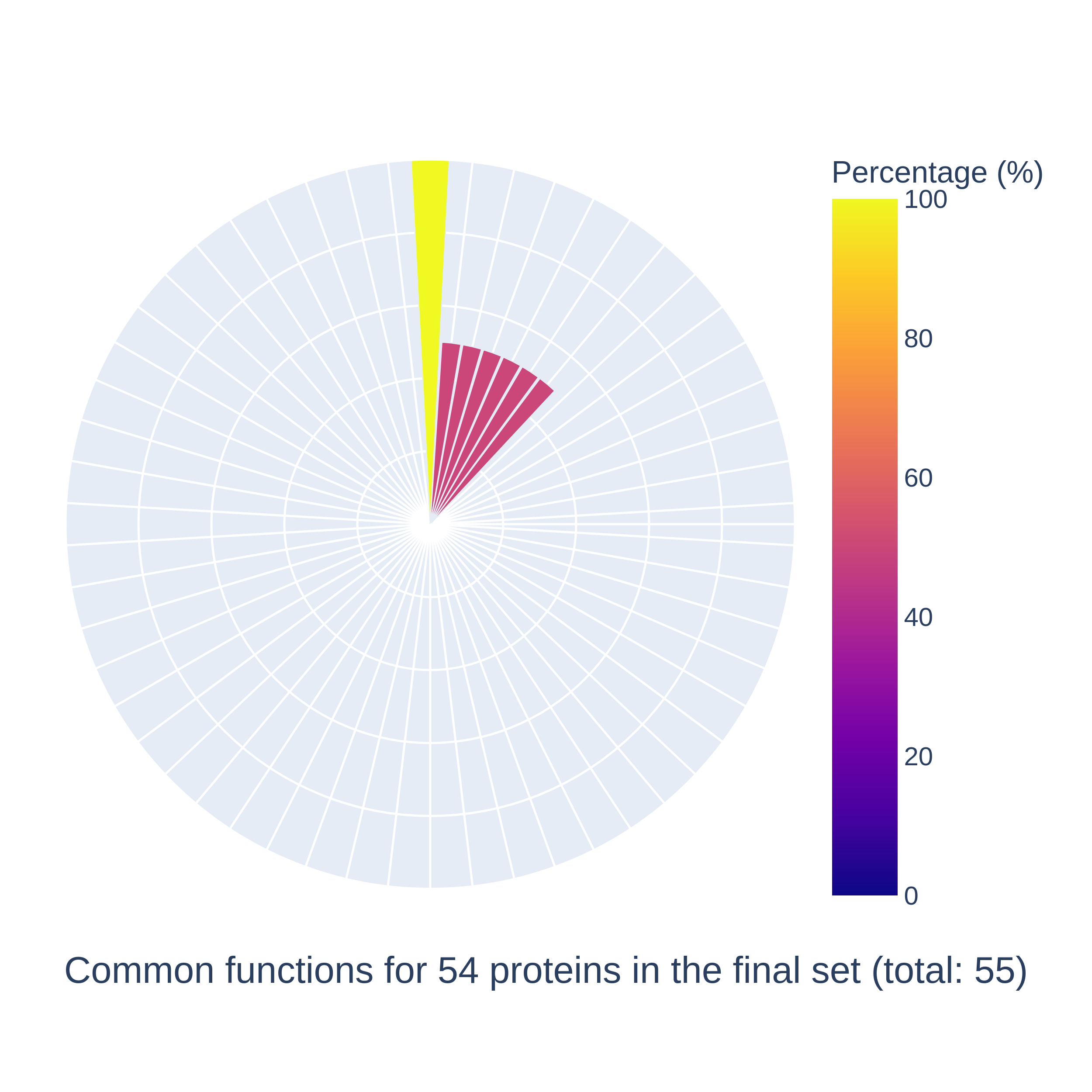

Supplement: Supplementary file 6 — Supplementary Data 3 [file 42003_2023_5076_MOESM6_ESM.zip › 6VXXp_A_whole/plots/6VXXp_A_molecularFunctionSim.png]

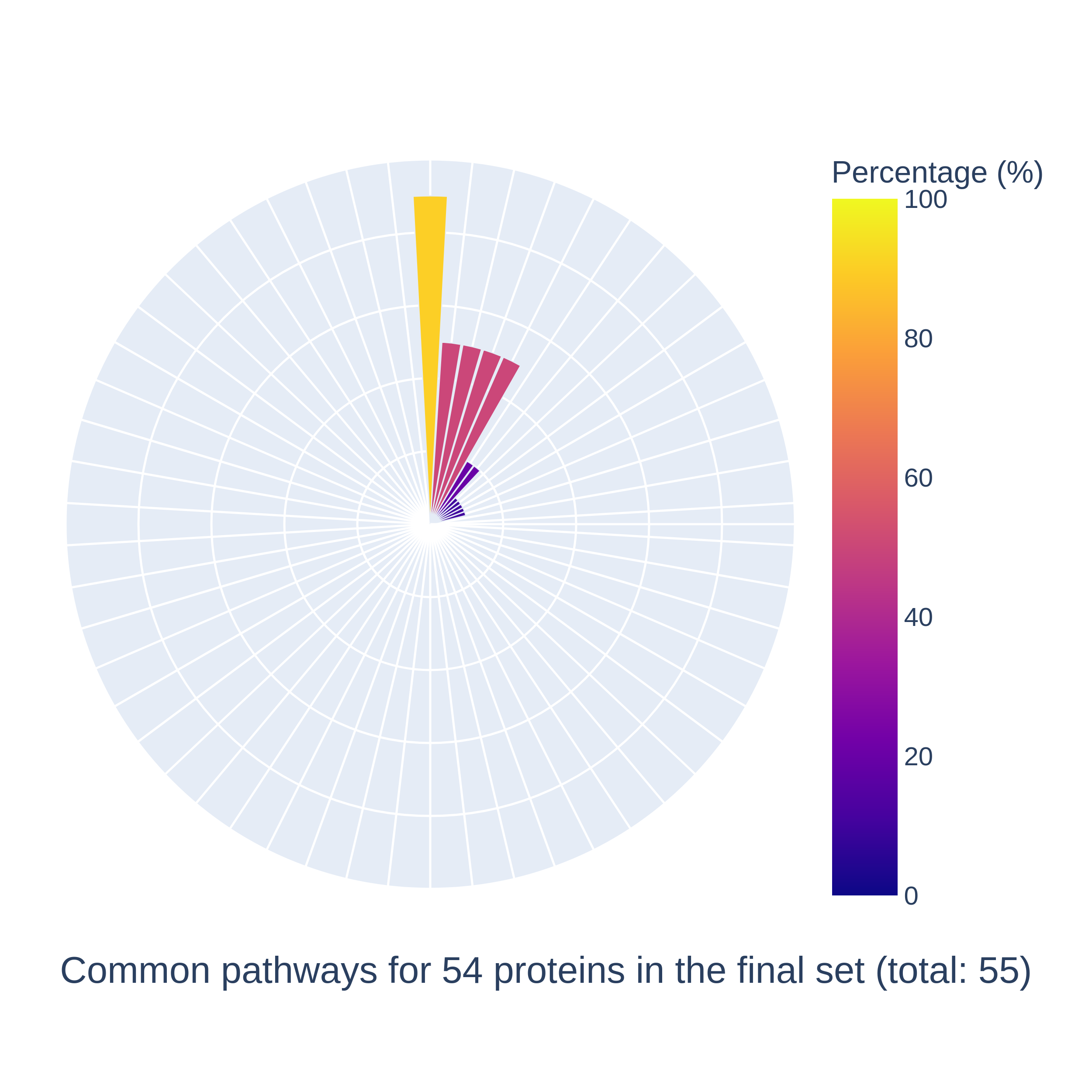

Supplement: Supplementary file 6 — Supplementary Data 3 [file 42003_2023_5076_MOESM6_ESM.zip › 6VXXp_A_whole/plots/6VXXp_A_biologicalProcessSim.png]

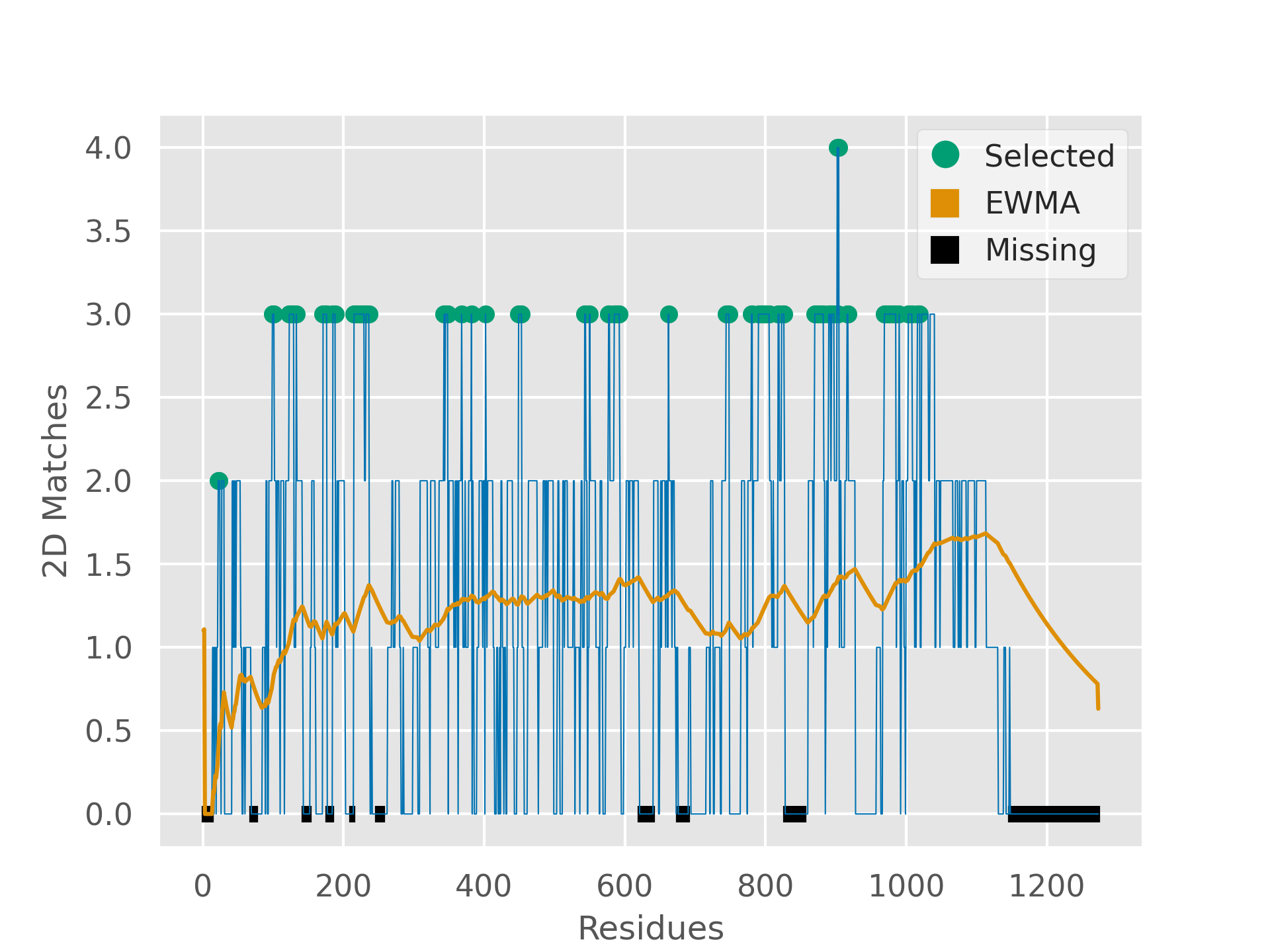

Supplement: Supplementary file 6 — Supplementary Data 3 [file 42003_2023_5076_MOESM6_ESM.zip › 7T9K_A_whole/go/7T9K_A_ubiquit_b4eaf75427a44fb8a52943c1e64b31b5.png]

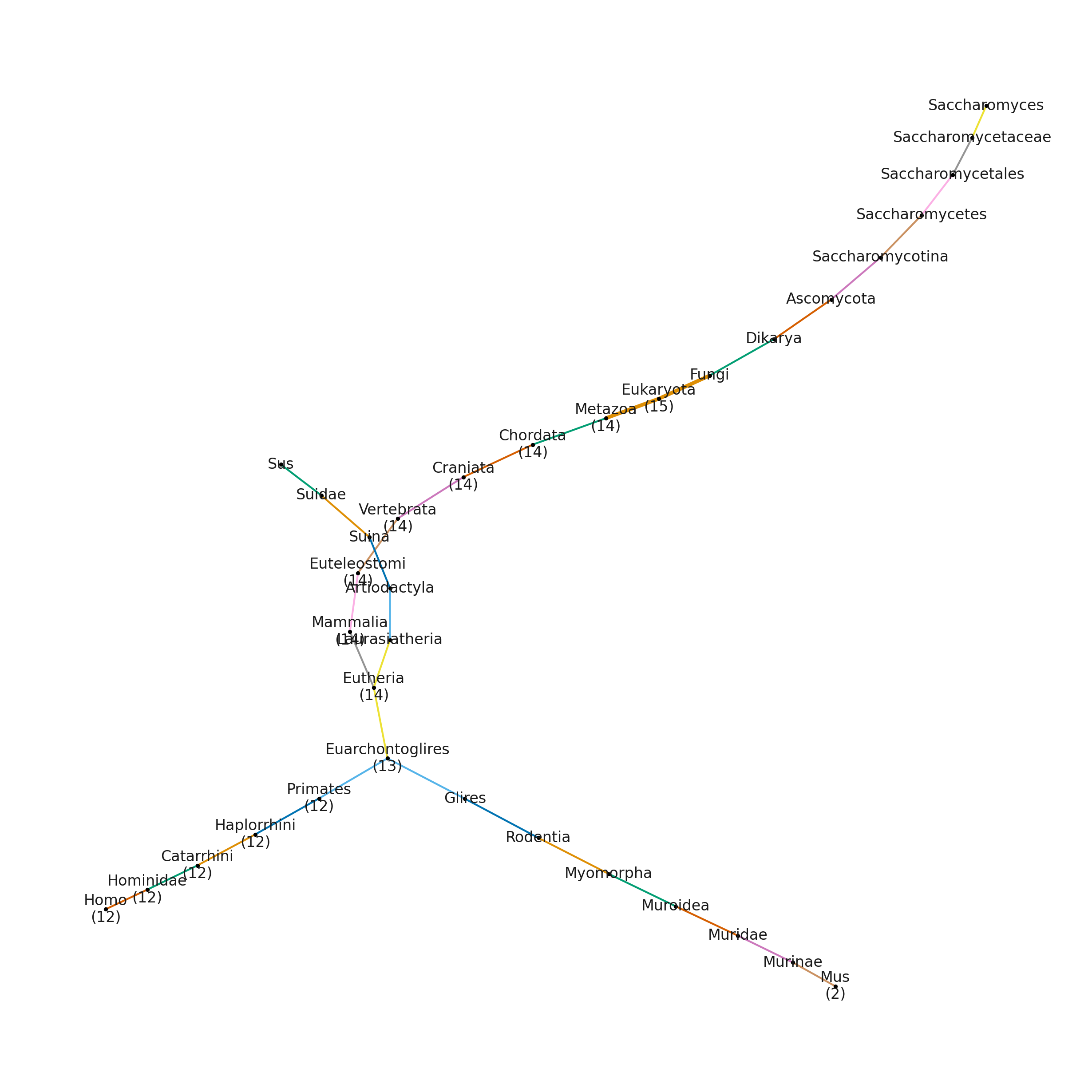

Supplement: Supplementary file 6 — Supplementary Data 3 [file 42003_2023_5076_MOESM6_ESM.zip › 7T9K_A_whole/plots/7T9K_A-Eukaryota-tree.png]

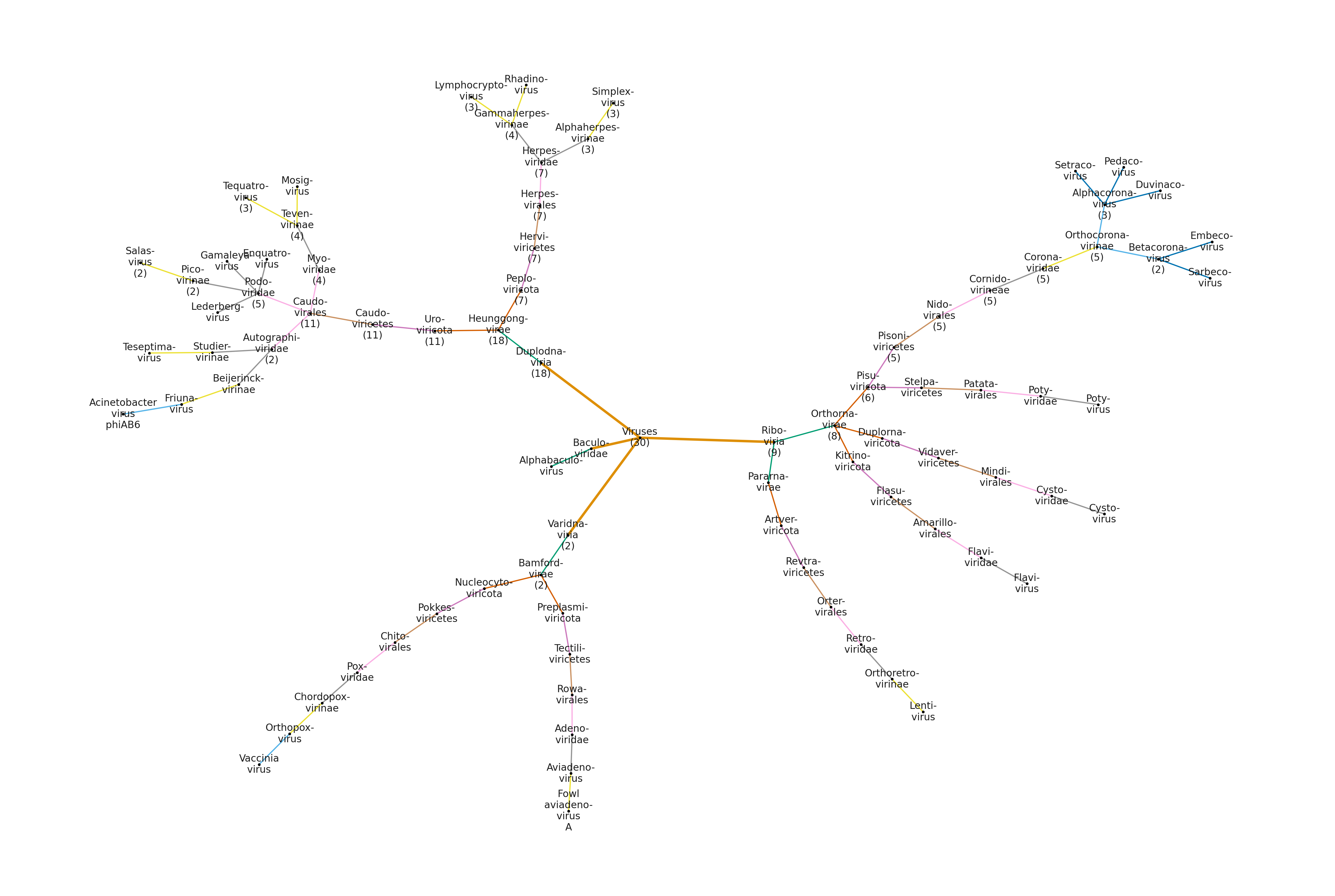

Supplement: Supplementary file 6 — Supplementary Data 3 [file 42003_2023_5076_MOESM6_ESM.zip › 7T9K_A_whole/plots/7T9K_A-Viruses-tree.png]

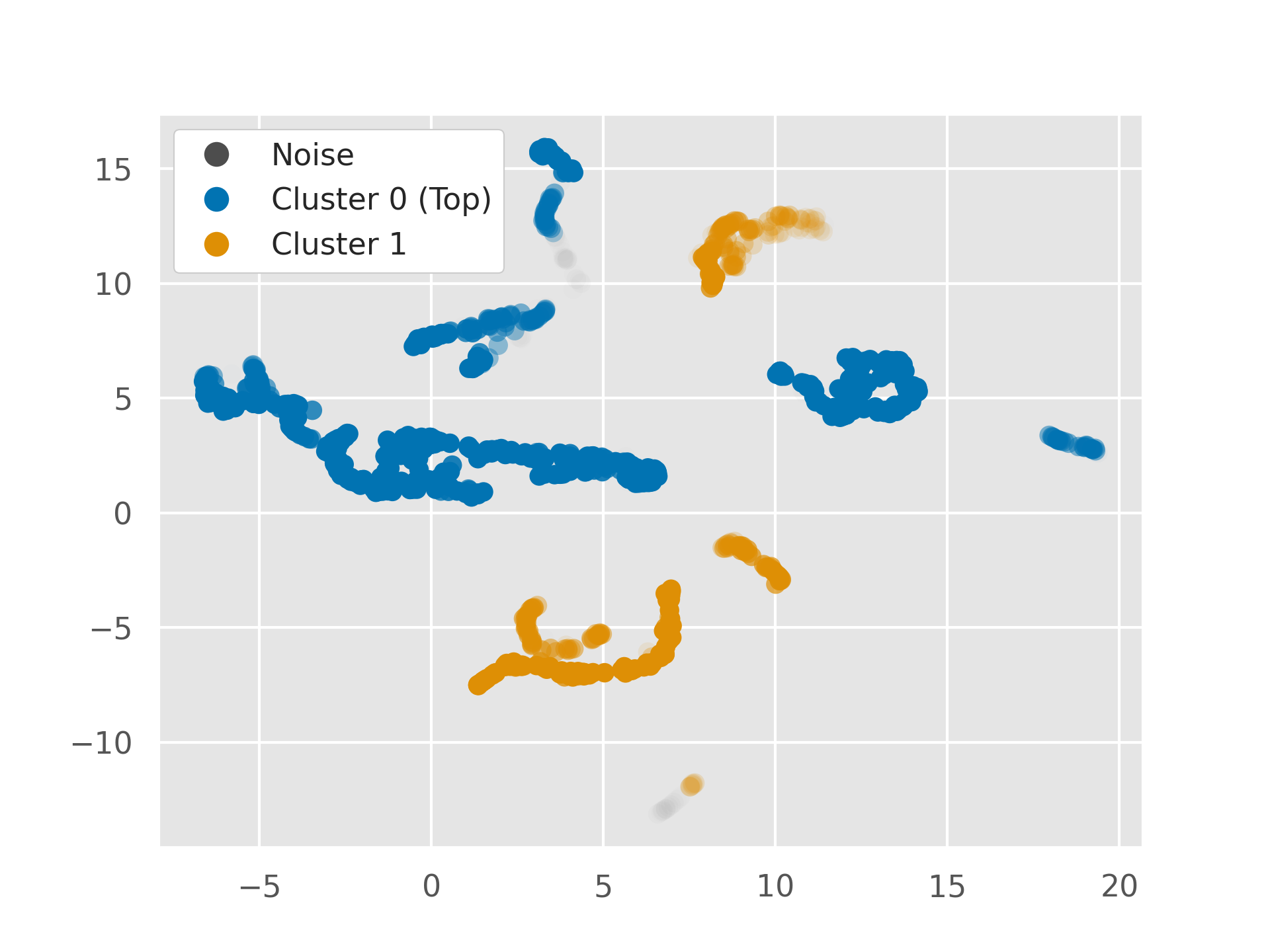

Supplement: Supplementary file 6 — Supplementary Data 3 [file 42003_2023_5076_MOESM6_ESM.zip › 7T9K_A_whole/plots/7T9K_A-clusters.png]

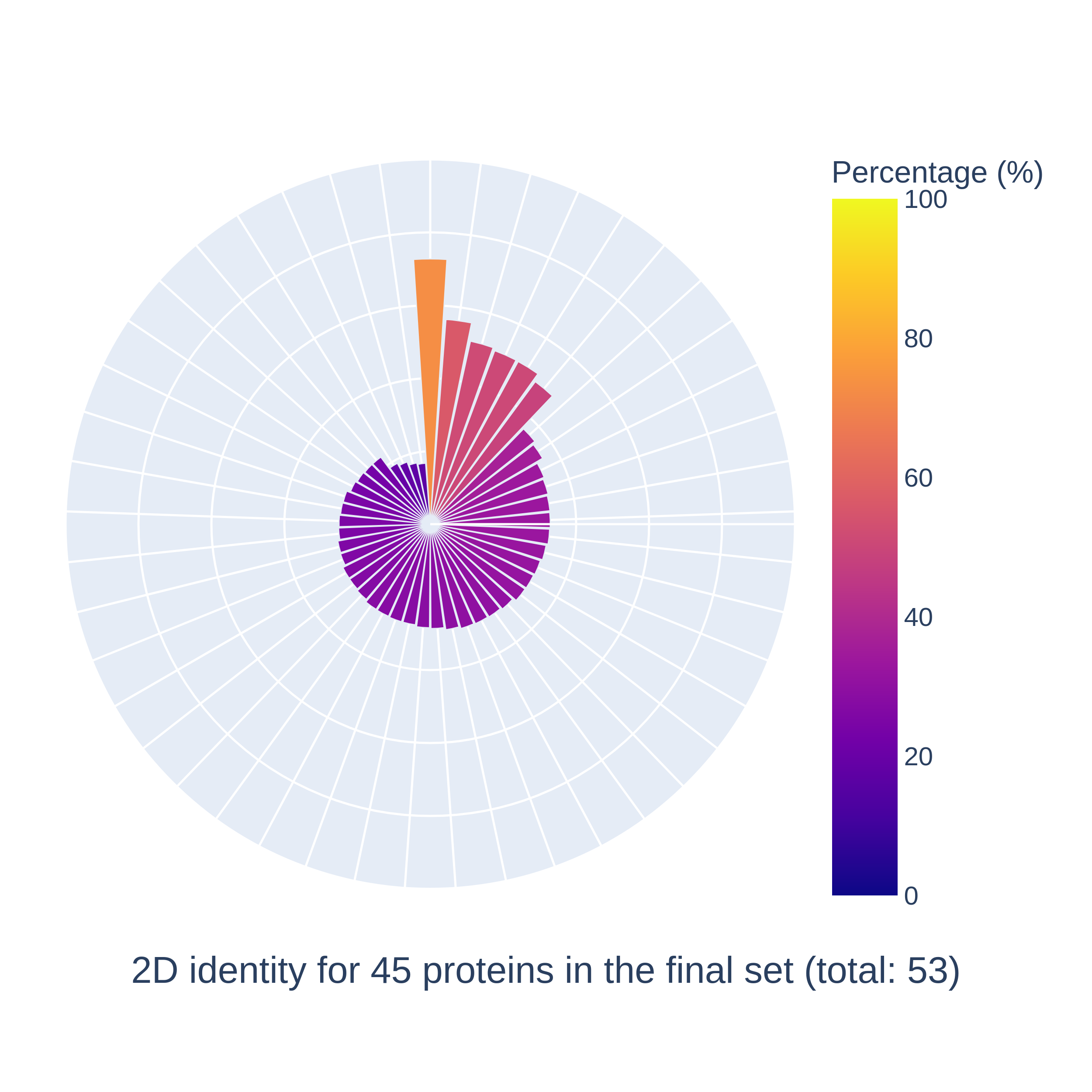

Supplement: Supplementary file 6 — Supplementary Data 3 [file 42003_2023_5076_MOESM6_ESM.zip › 7T9K_A_whole/plots/7T9K_A_2D-identity.png]

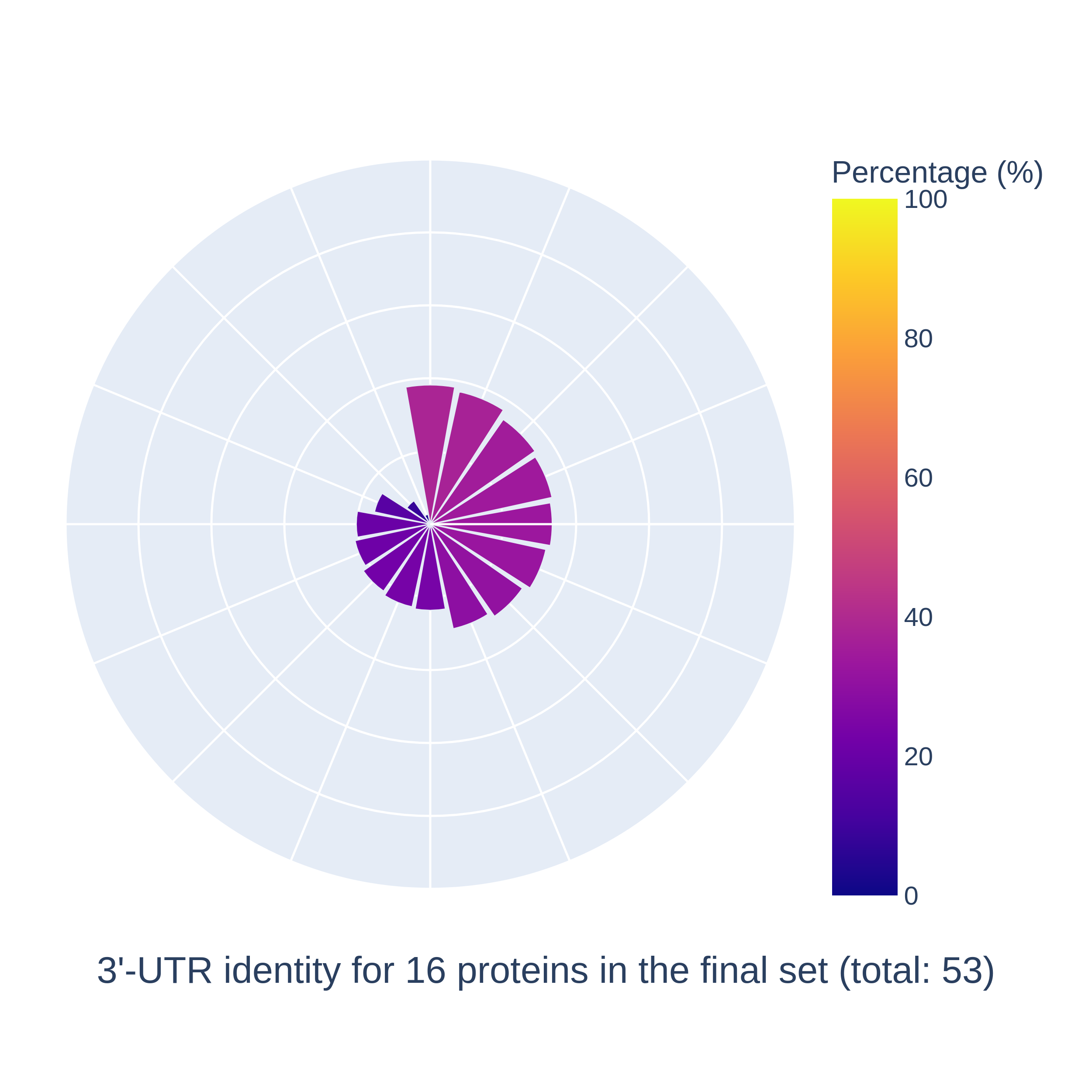

Supplement: Supplementary file 6 — Supplementary Data 3 [file 42003_2023_5076_MOESM6_ESM.zip › 7T9K_A_whole/plots/7T9K_A_3UTR-identity.png]

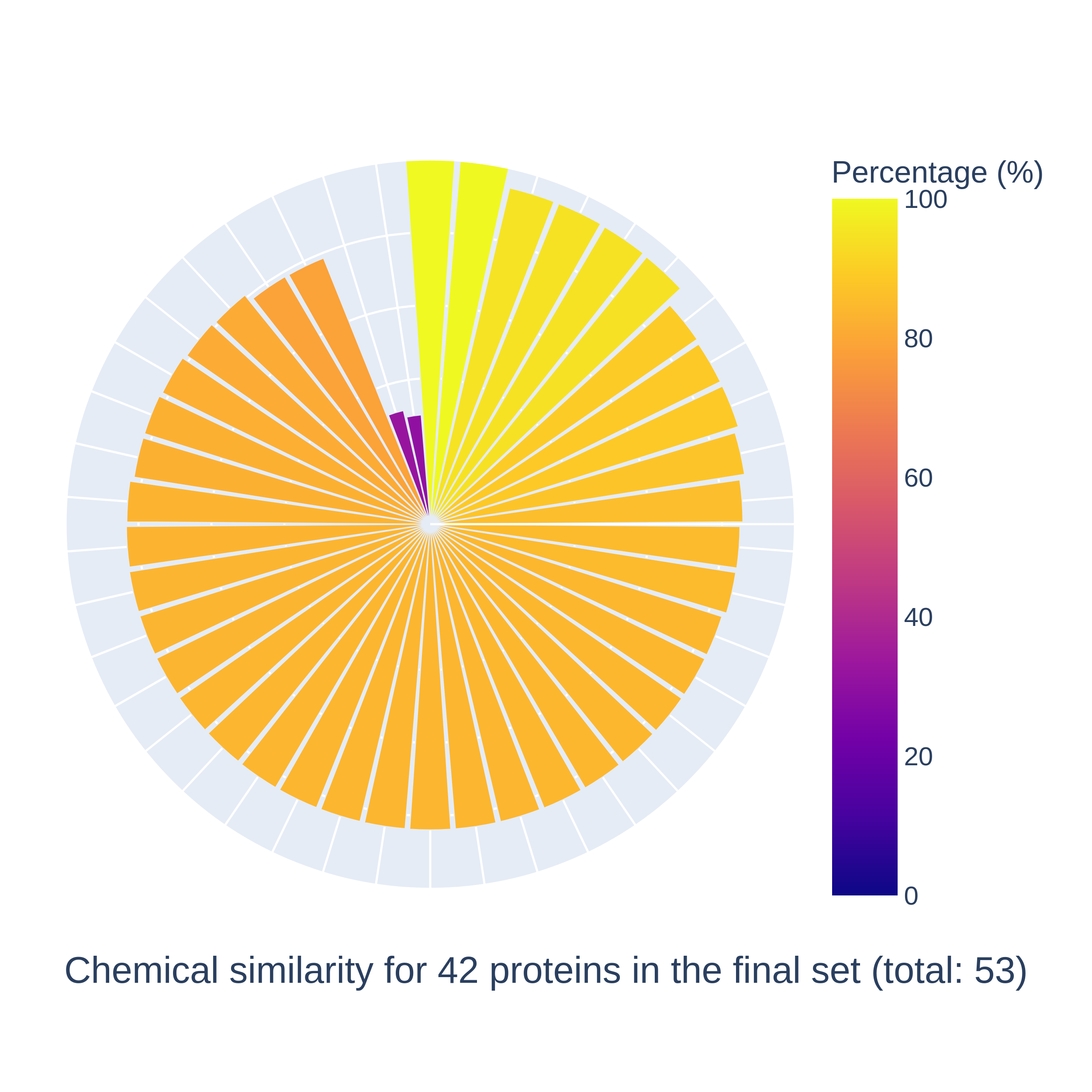

Supplement: Supplementary file 6 — Supplementary Data 3 [file 42003_2023_5076_MOESM6_ESM.zip › 7T9K_A_whole/plots/7T9K_A_chemSim.png]

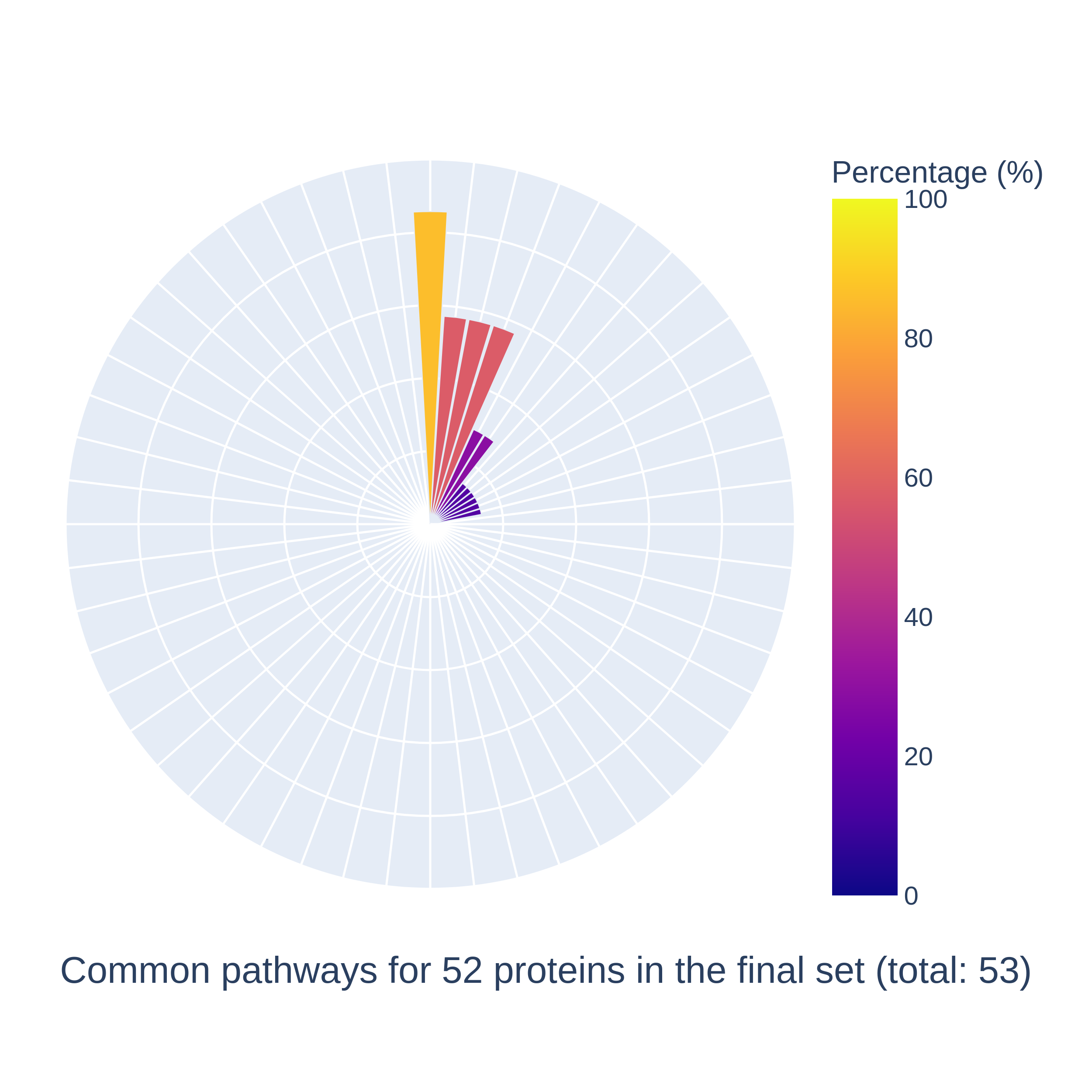

Supplement: Supplementary file 6 — Supplementary Data 3 [file 42003_2023_5076_MOESM6_ESM.zip › 7T9K_A_whole/plots/7T9K_A_biologicalProcessSim.png]

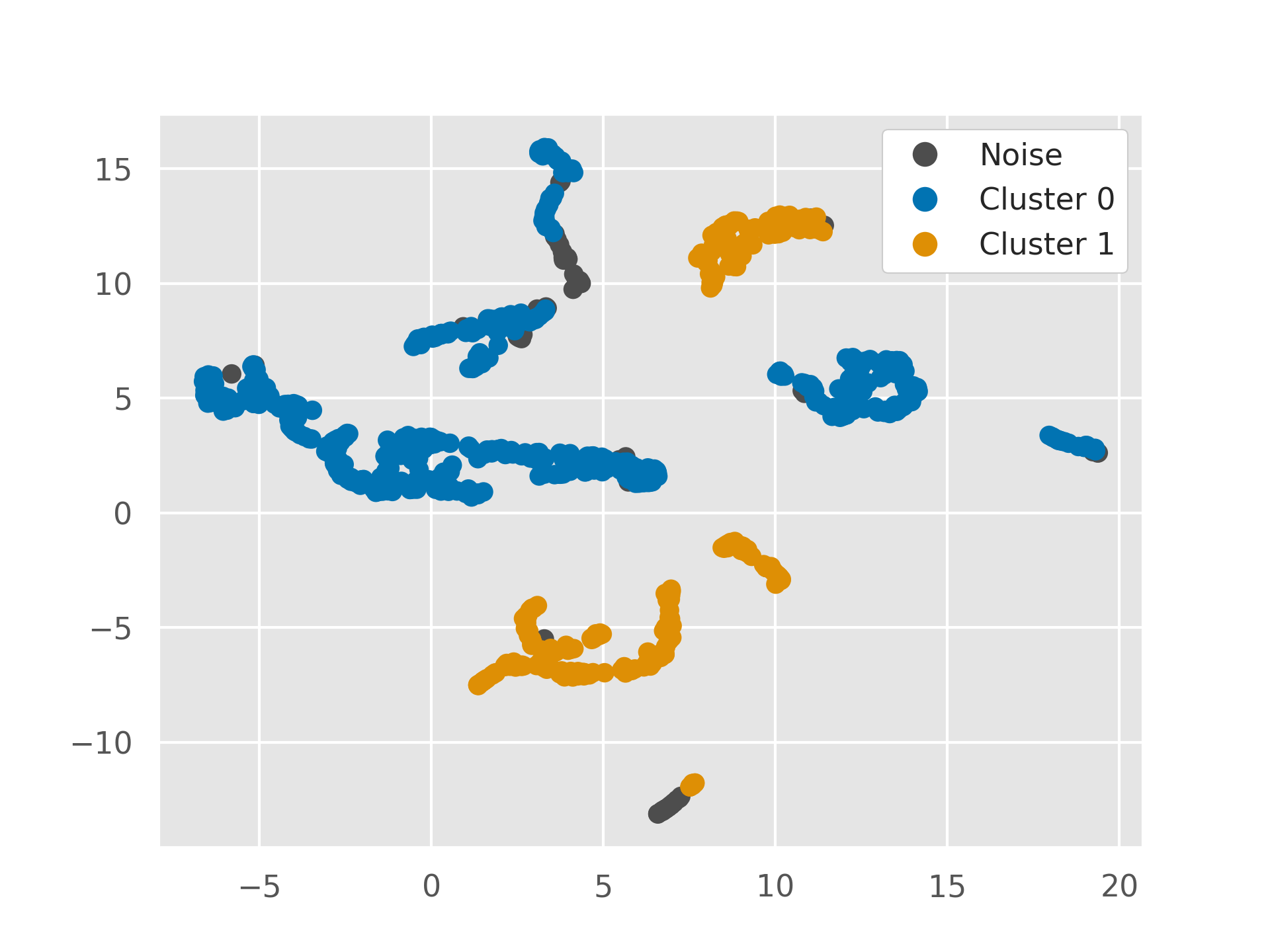

Supplement: Supplementary file 6 — Supplementary Data 3 [file 42003_2023_5076_MOESM6_ESM.zip › 7T9K_A_whole/plots/7T9K_A-clusters-initial.png]

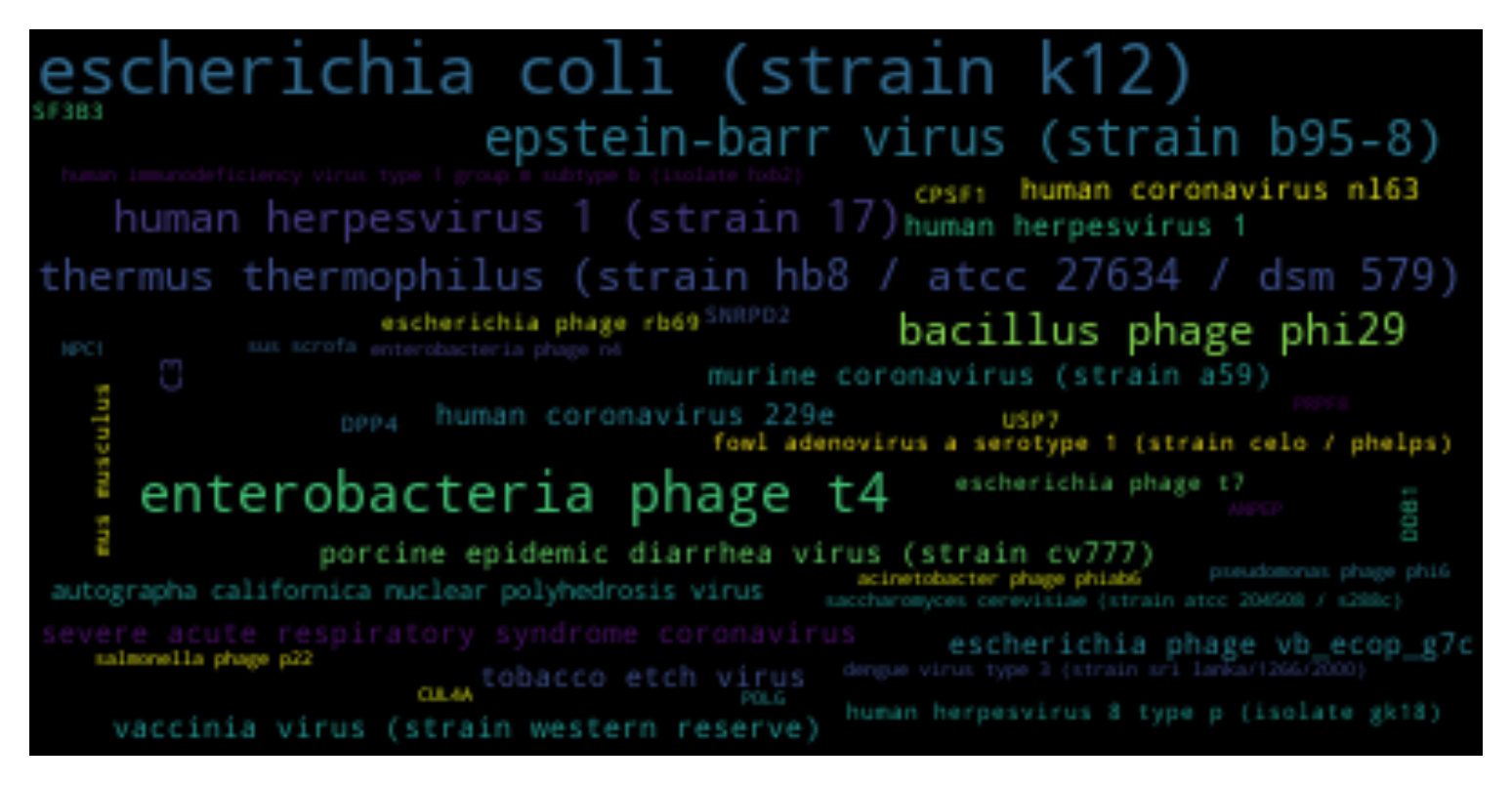

Supplement: Supplementary file 6 — Supplementary Data 3 [file 42003_2023_5076_MOESM6_ESM.zip › 7T9K_A_whole/plots/7T9K_A-wordcloud.png]

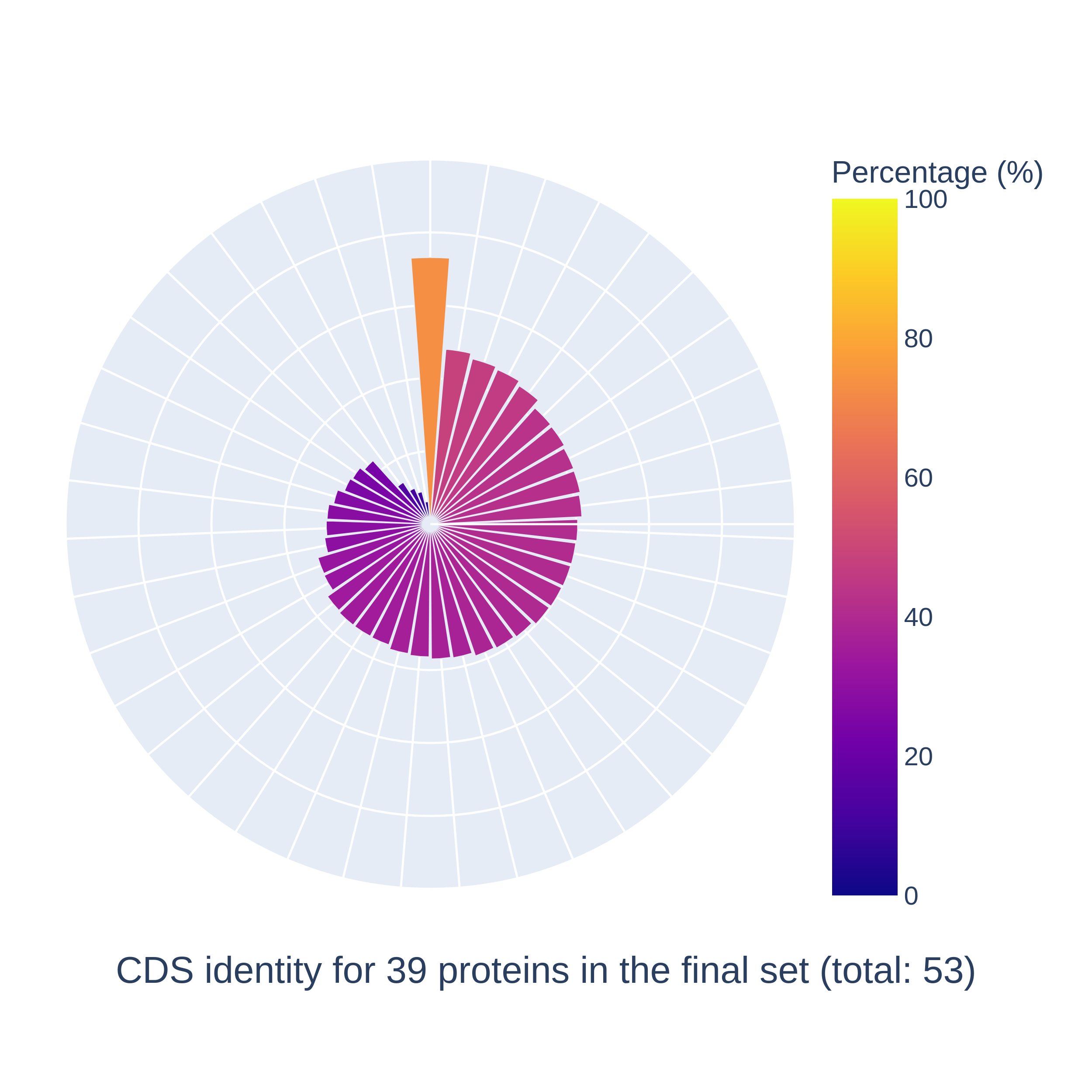

Supplement: Supplementary file 6 — Supplementary Data 3 [file 42003_2023_5076_MOESM6_ESM.zip › 7T9K_A_whole/plots/7T9K_A_CDS-identity.png]

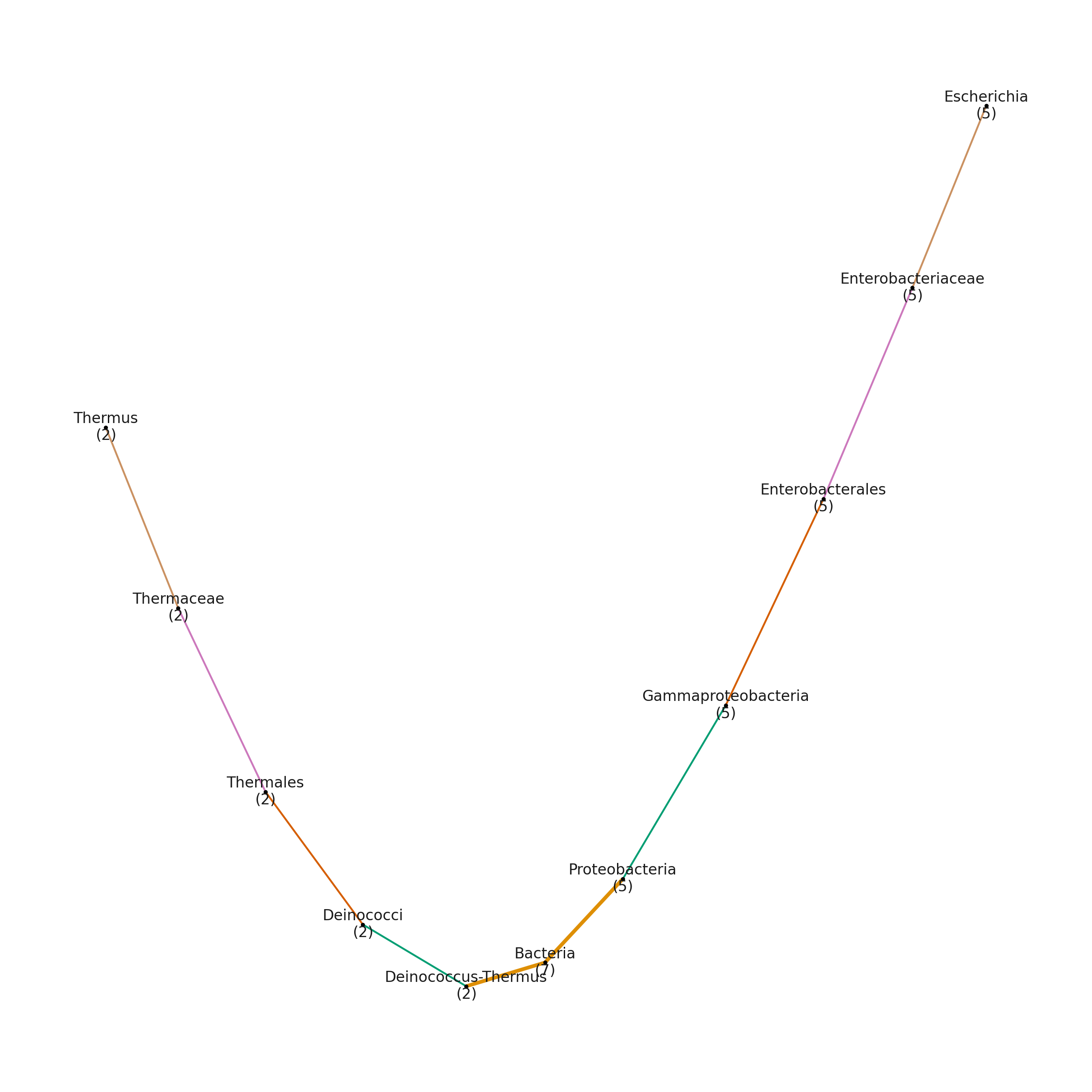

Supplement: Supplementary file 6 — Supplementary Data 3 [file 42003_2023_5076_MOESM6_ESM.zip › 7T9K_A_whole/plots/7T9K_A-Bacteria-tree.png]

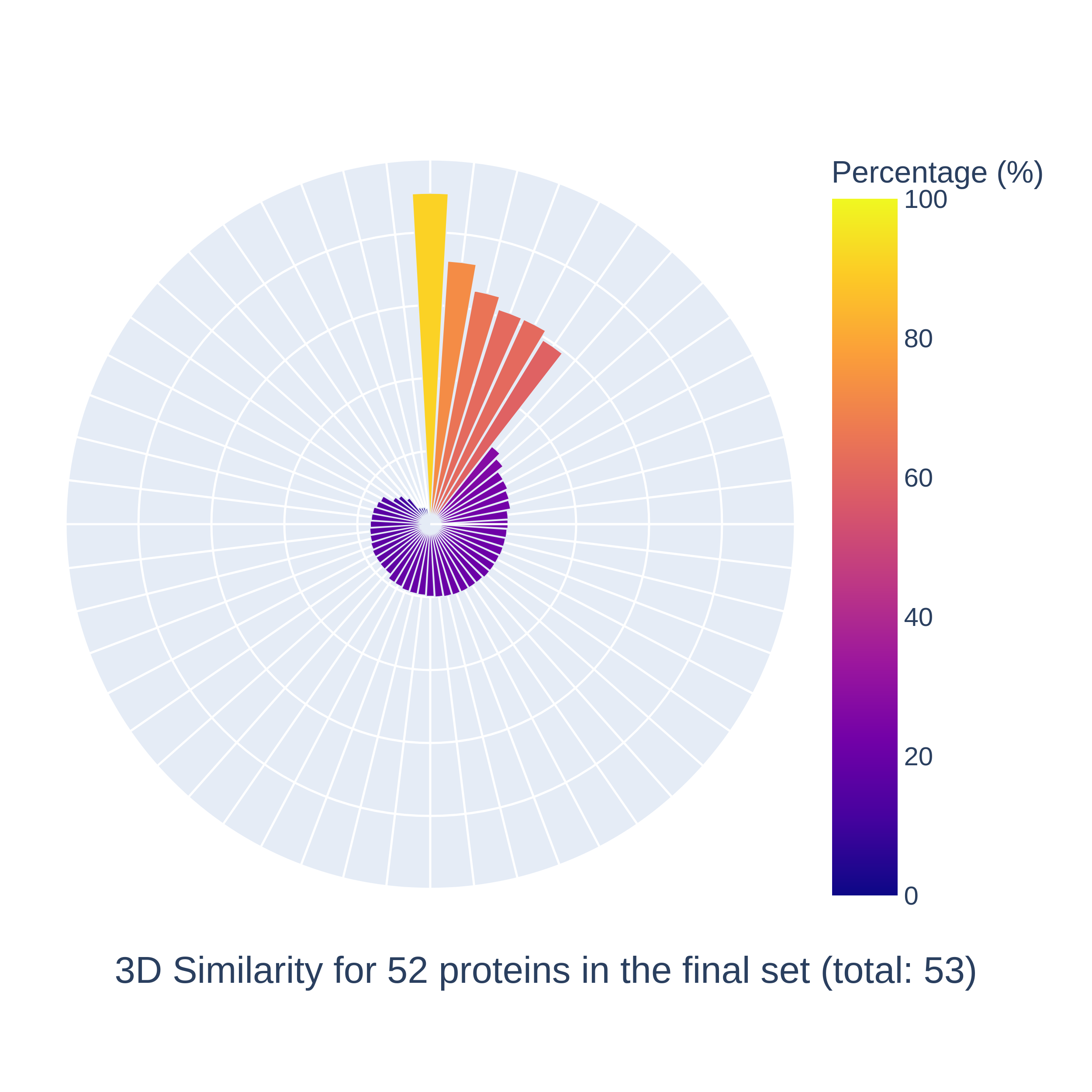

Supplement: Supplementary file 6 — Supplementary Data 3 [file 42003_2023_5076_MOESM6_ESM.zip › 7T9K_A_whole/plots/7T9K_A_3D-score.png]

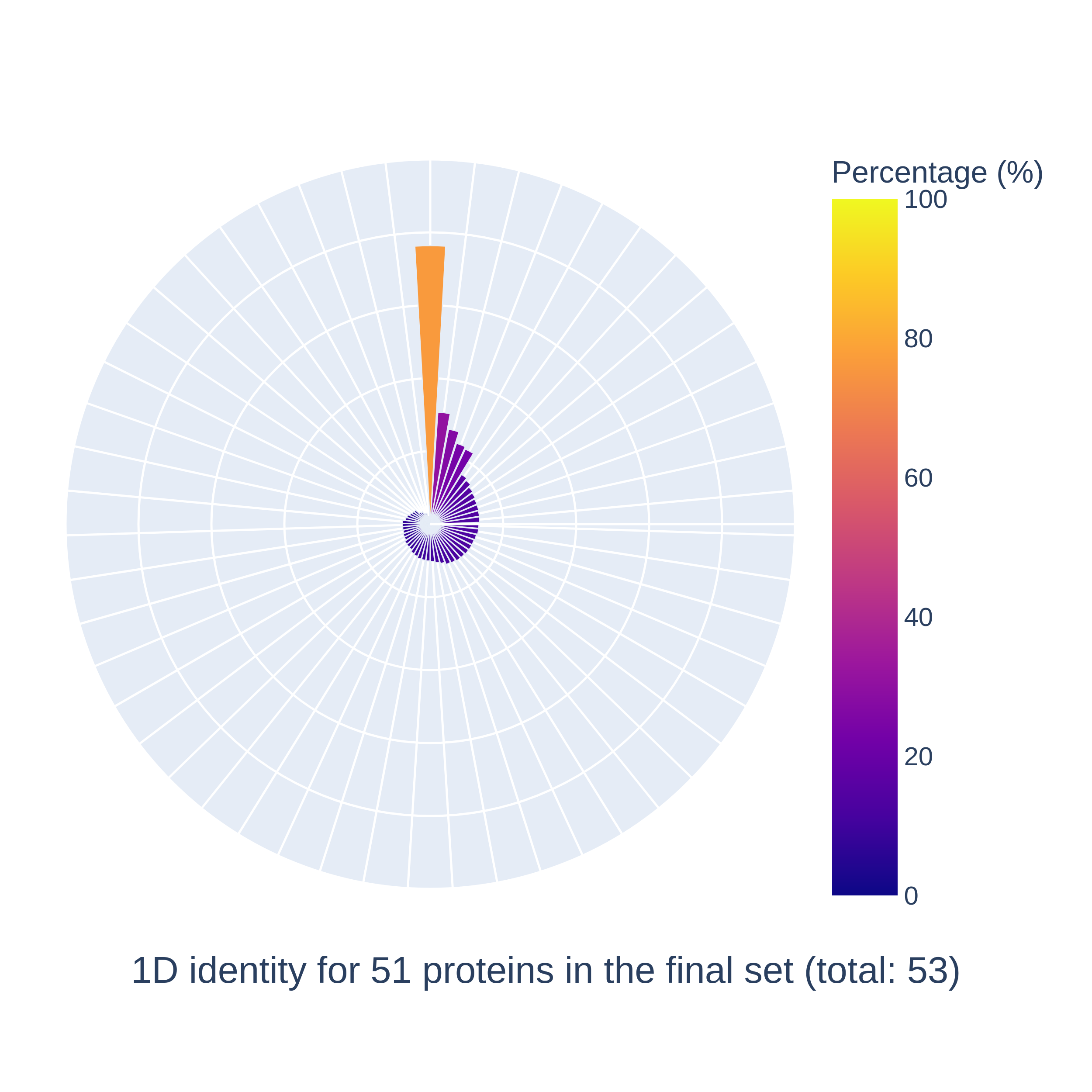

Supplement: Supplementary file 6 — Supplementary Data 3 [file 42003_2023_5076_MOESM6_ESM.zip › 7T9K_A_whole/plots/7T9K_A_1D-identity.png]

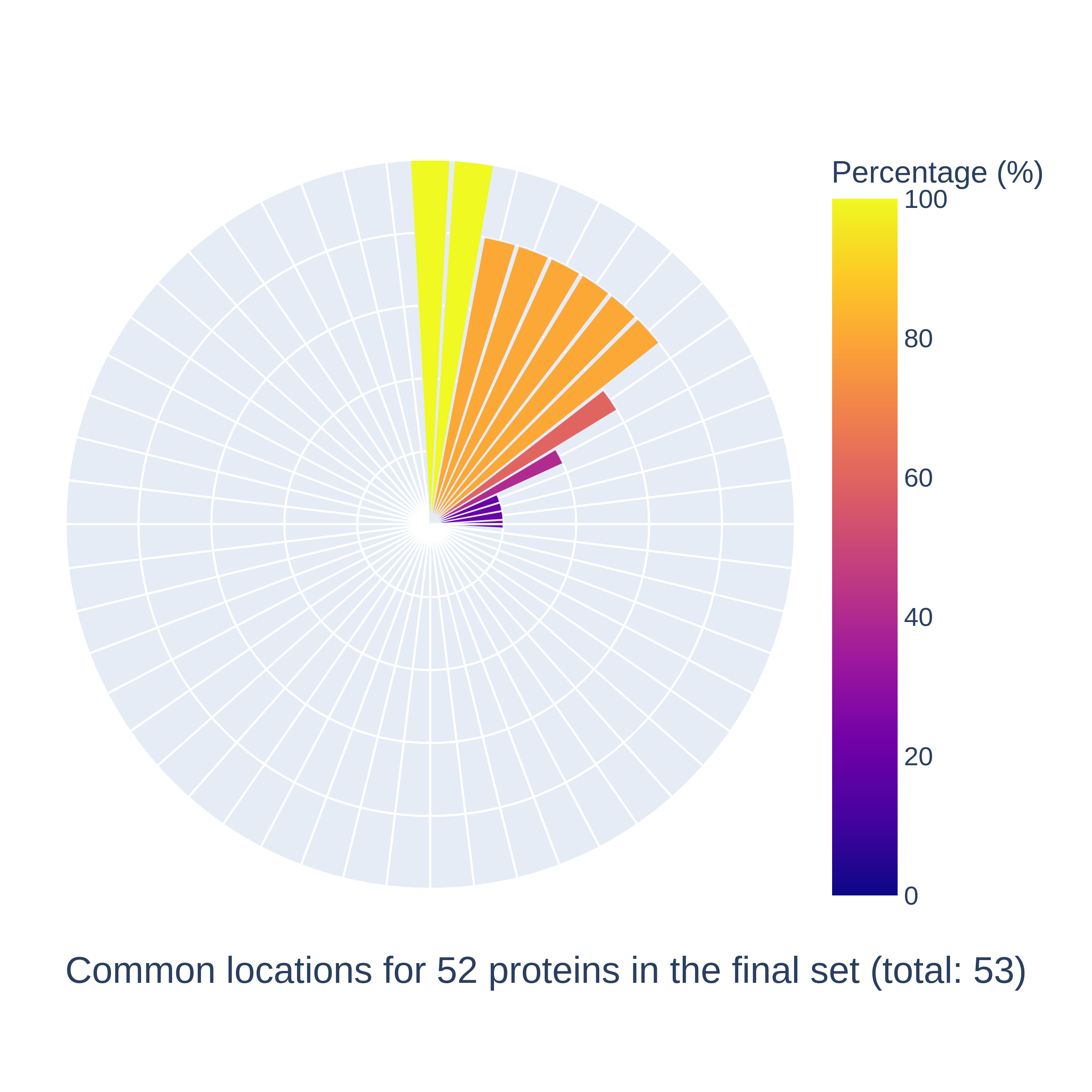

Supplement: Supplementary file 6 — Supplementary Data 3 [file 42003_2023_5076_MOESM6_ESM.zip › 7T9K_A_whole/plots/7T9K_A_cellularComponentSim.png]

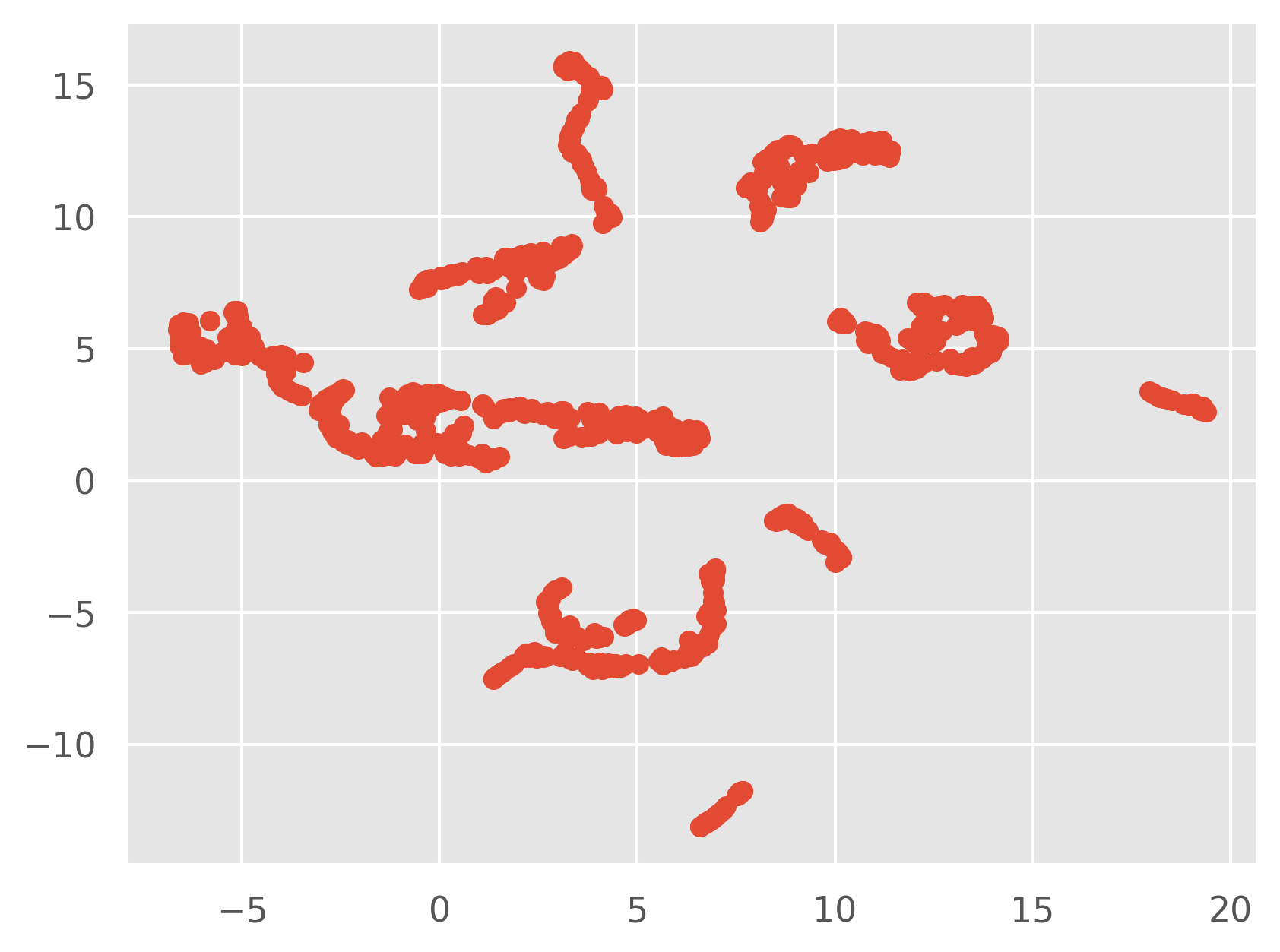

Supplement: Supplementary file 6 — Supplementary Data 3 [file 42003_2023_5076_MOESM6_ESM.zip › 7T9K_A_whole/plots/7T9K_A-UMAP-.png]

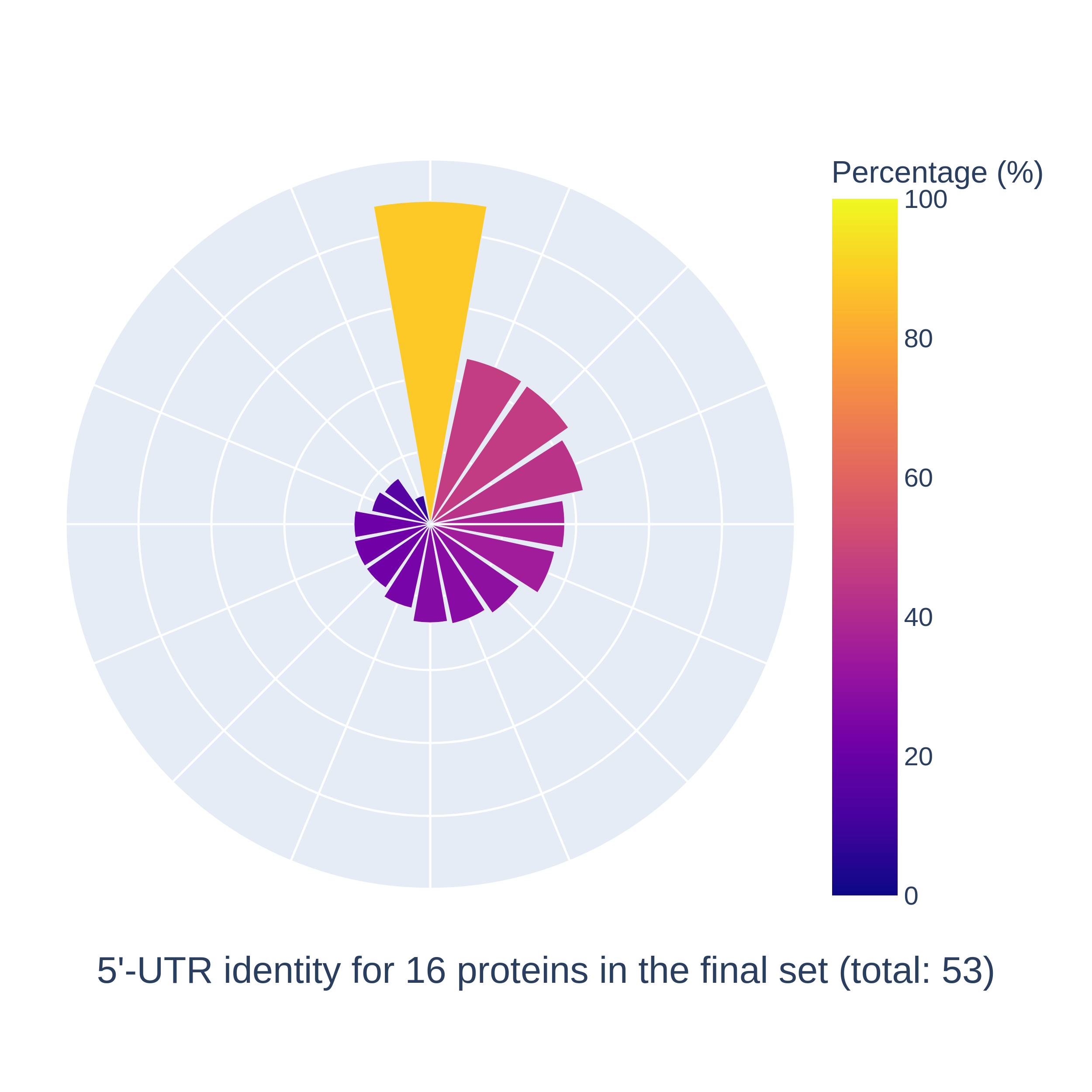

Supplement: Supplementary file 6 — Supplementary Data 3 [file 42003_2023_5076_MOESM6_ESM.zip › 7T9K_A_whole/plots/7T9K_A_5UTR-identity.png]

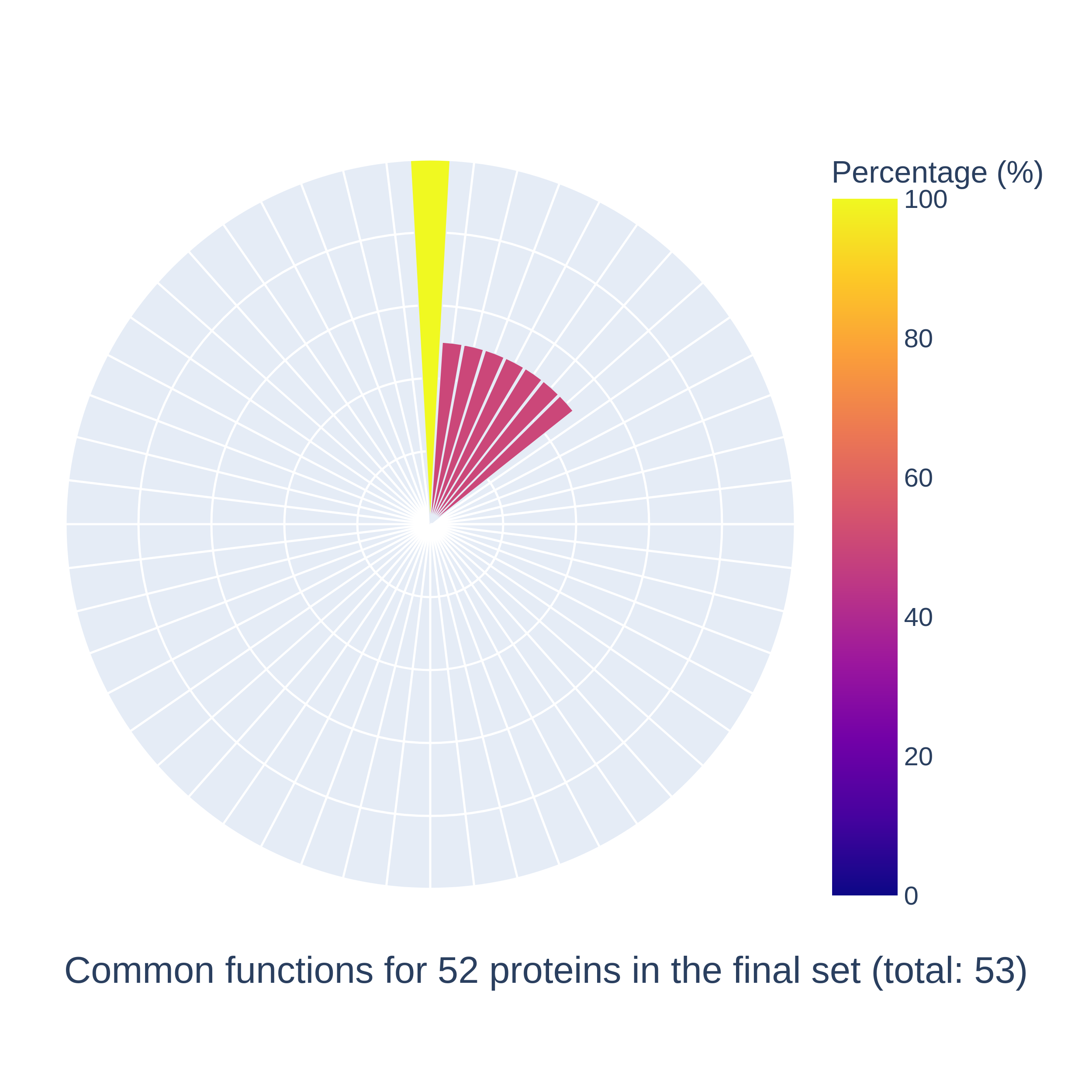

Supplement: Supplementary file 6 — Supplementary Data 3 [file 42003_2023_5076_MOESM6_ESM.zip › 7T9K_A_whole/plots/7T9K_A_molecularFunctionSim.png]

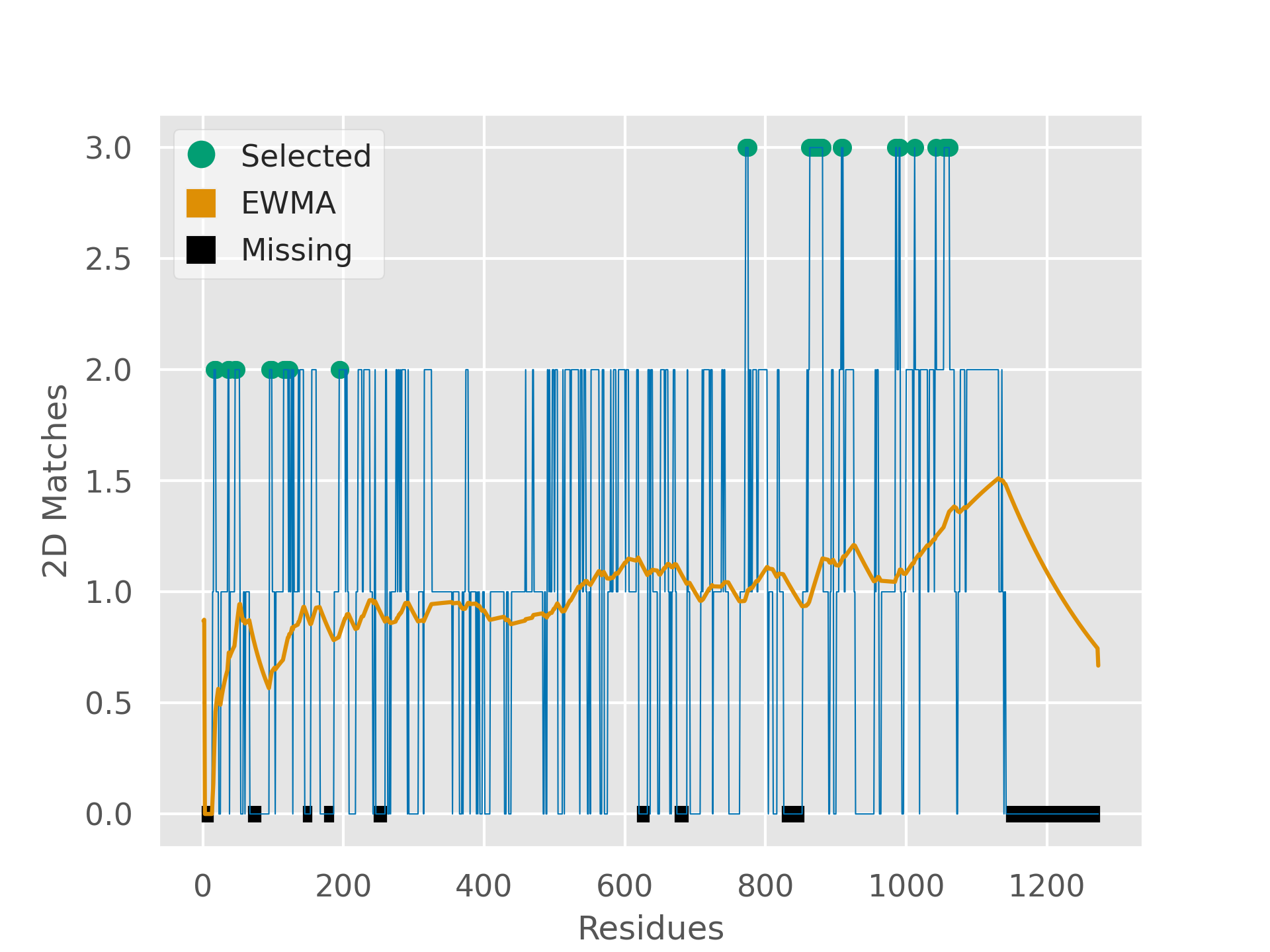

Supplement: Supplementary file 6 — Supplementary Data 3 [file 42003_2023_5076_MOESM6_ESM.zip › 7V7Q_A_whole/go/7V7Q_A_ubiquit_28aec8173aa9414bb8d0cd7d0a2b4be3.png]

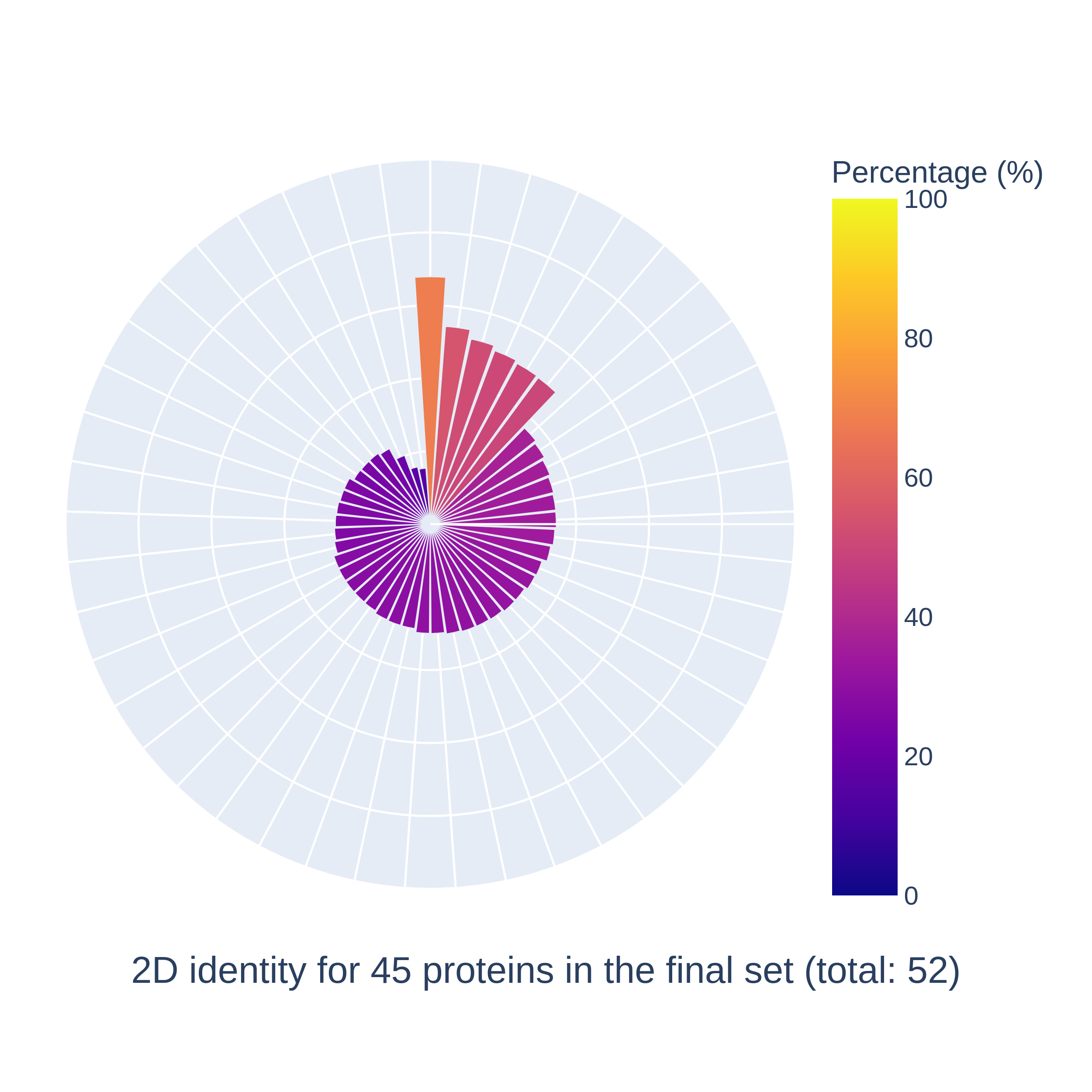

Supplement: Supplementary file 6 — Supplementary Data 3 [file 42003_2023_5076_MOESM6_ESM.zip › 7V7Q_A_whole/plots/7V7Q_A_2D-identity.png]

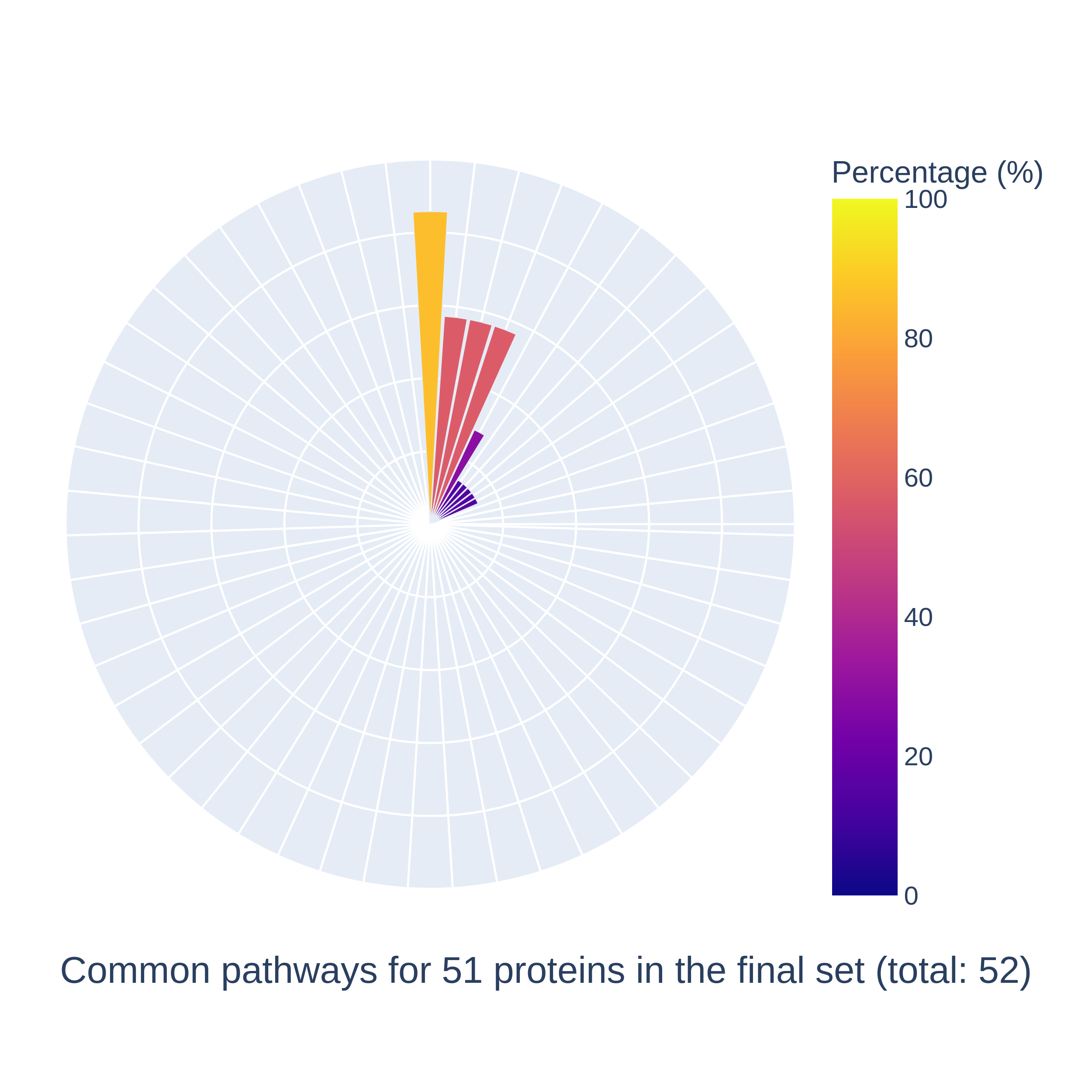

Supplement: Supplementary file 6 — Supplementary Data 3 [file 42003_2023_5076_MOESM6_ESM.zip › 7V7Q_A_whole/plots/7V7Q_A_biologicalProcessSim.png]

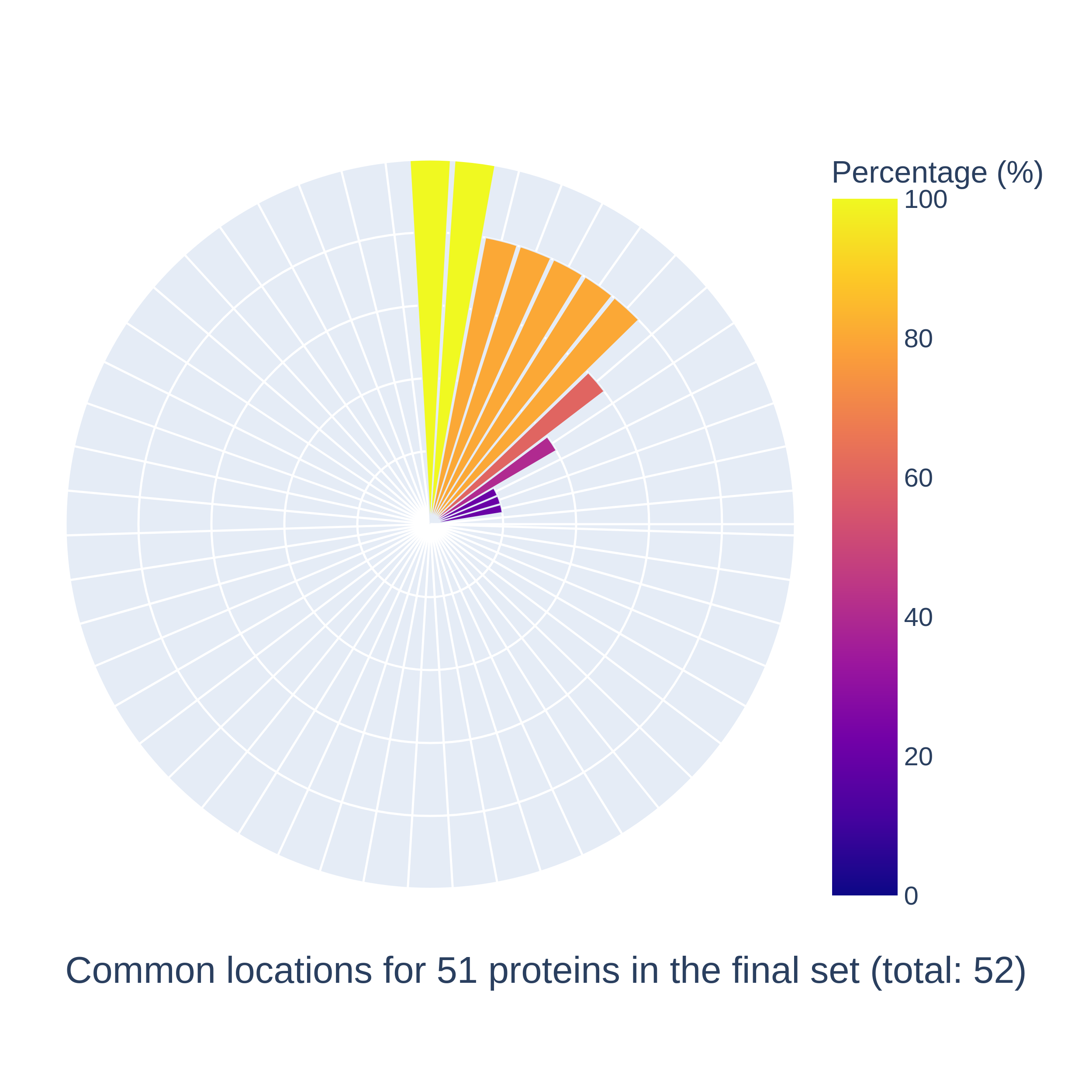

Supplement: Supplementary file 6 — Supplementary Data 3 [file 42003_2023_5076_MOESM6_ESM.zip › 7V7Q_A_whole/plots/7V7Q_A_cellularComponentSim.png]

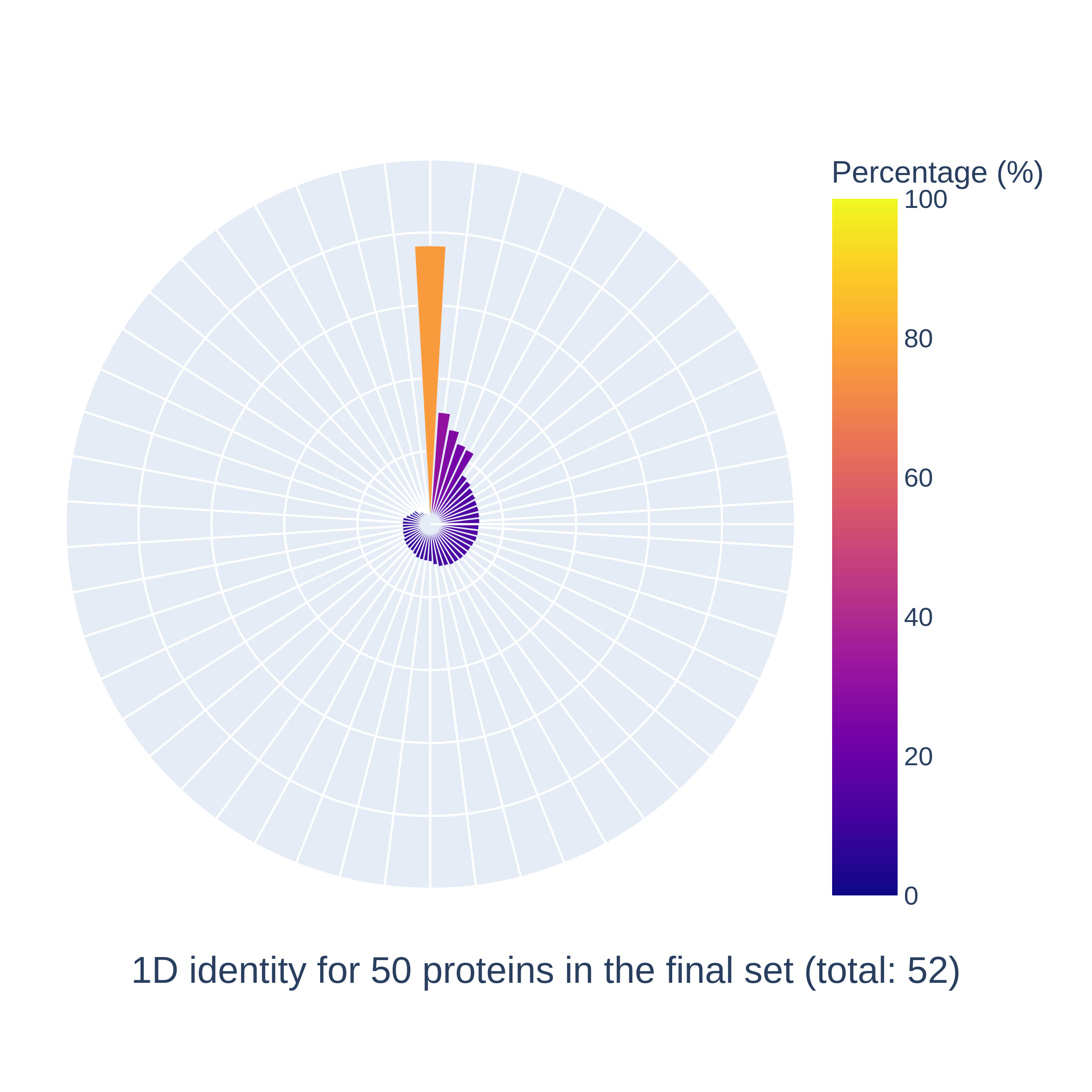

Supplement: Supplementary file 6 — Supplementary Data 3 [file 42003_2023_5076_MOESM6_ESM.zip › 7V7Q_A_whole/plots/7V7Q_A_1D-identity.png]

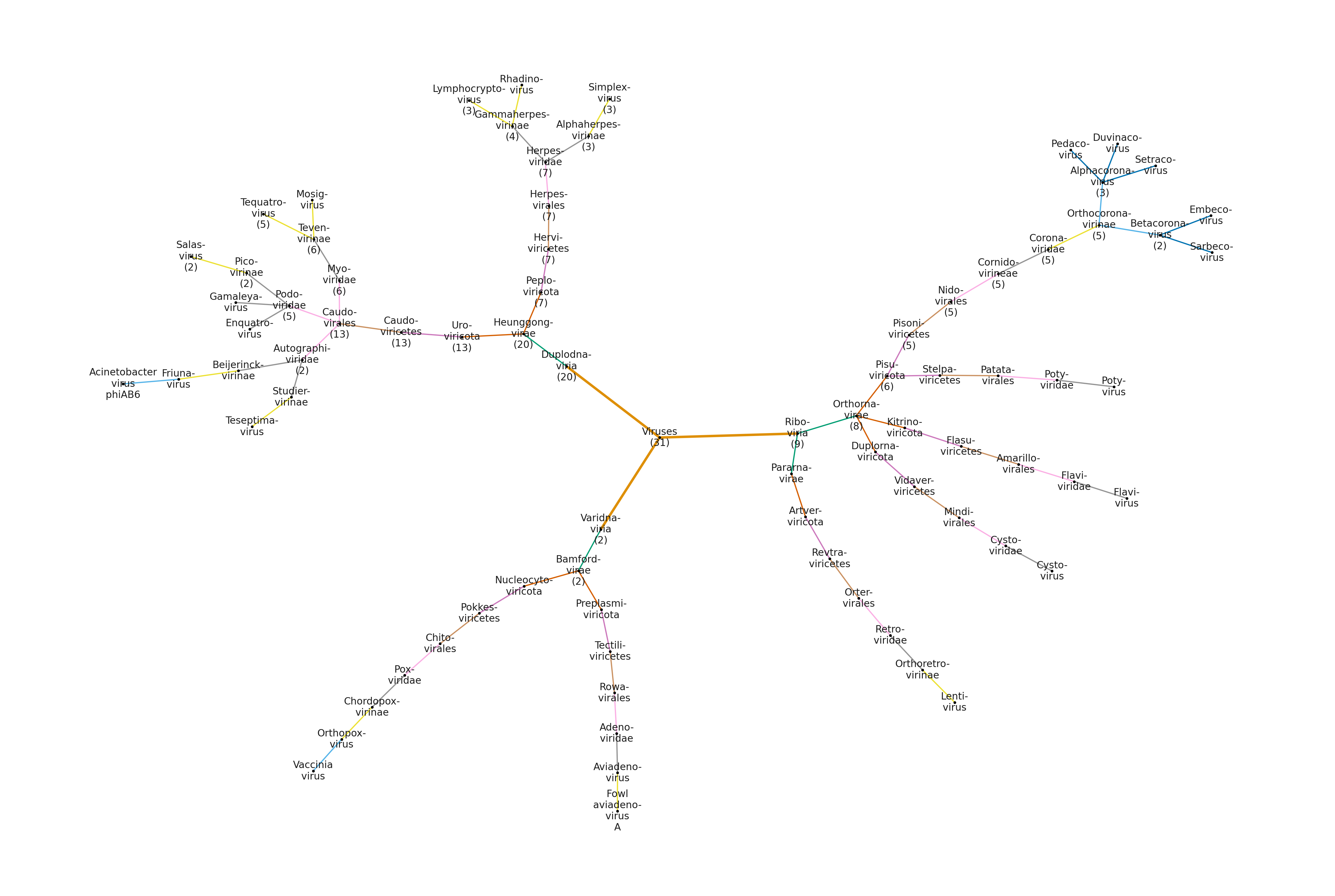

Supplement: Supplementary file 6 — Supplementary Data 3 [file 42003_2023_5076_MOESM6_ESM.zip › 7V7Q_A_whole/plots/7V7Q_A-Viruses-tree.png]

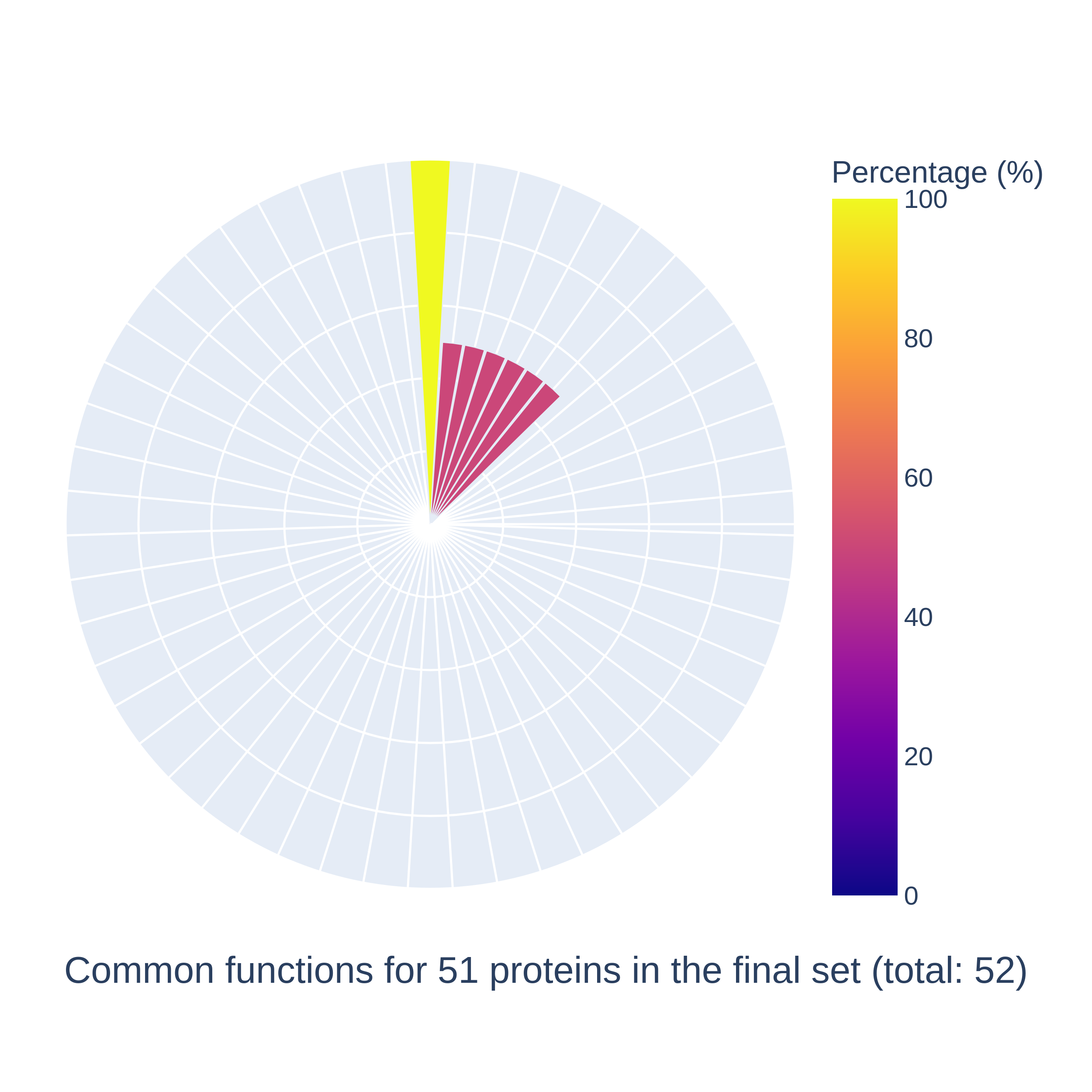

Supplement: Supplementary file 6 — Supplementary Data 3 [file 42003_2023_5076_MOESM6_ESM.zip › 7V7Q_A_whole/plots/7V7Q_A_molecularFunctionSim.png]

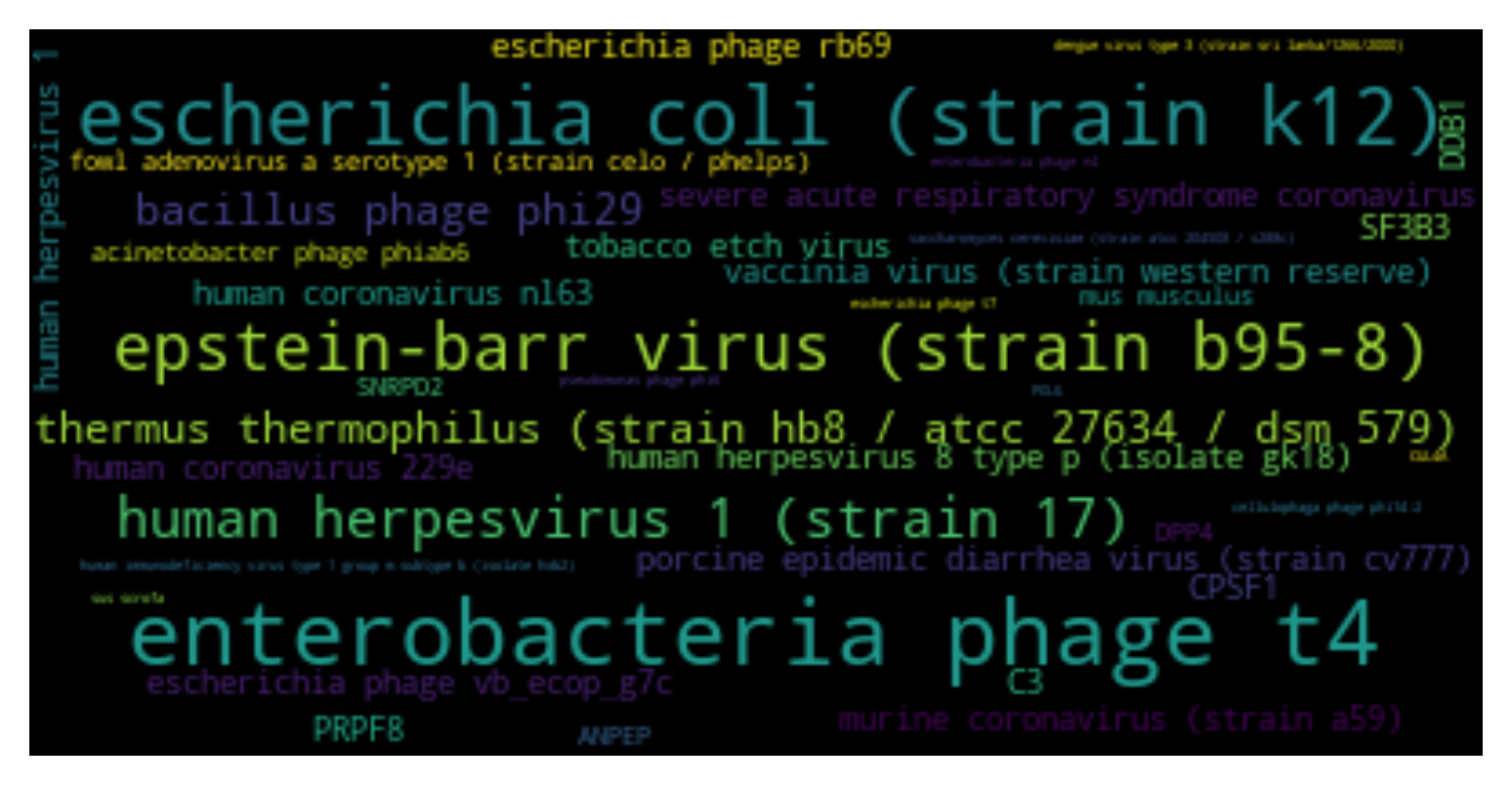

Supplement: Supplementary file 6 — Supplementary Data 3 [file 42003_2023_5076_MOESM6_ESM.zip › 7V7Q_A_whole/plots/7V7Q_A-wordcloud.png]

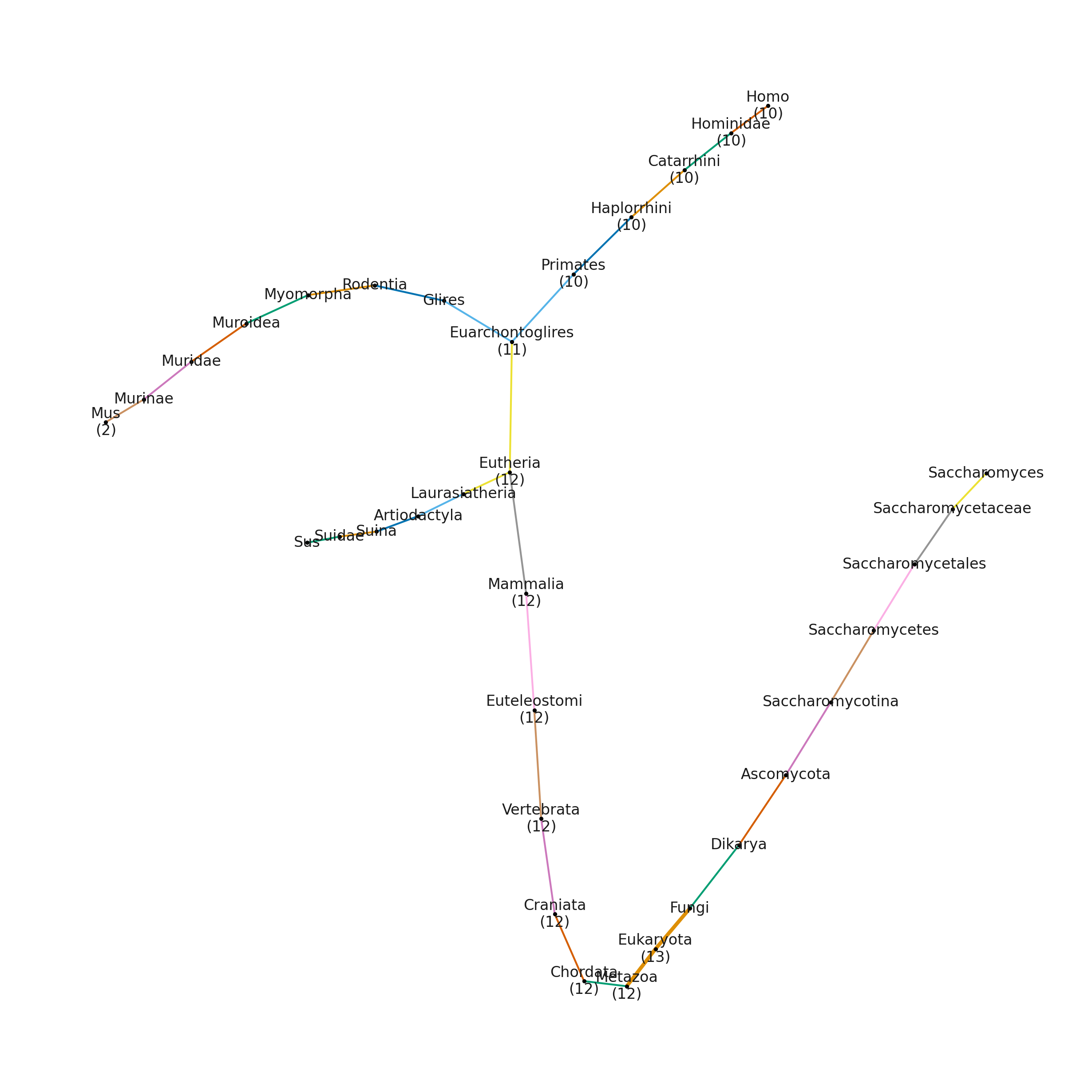

Supplement: Supplementary file 6 — Supplementary Data 3 [file 42003_2023_5076_MOESM6_ESM.zip › 7V7Q_A_whole/plots/7V7Q_A-Eukaryota-tree.png]

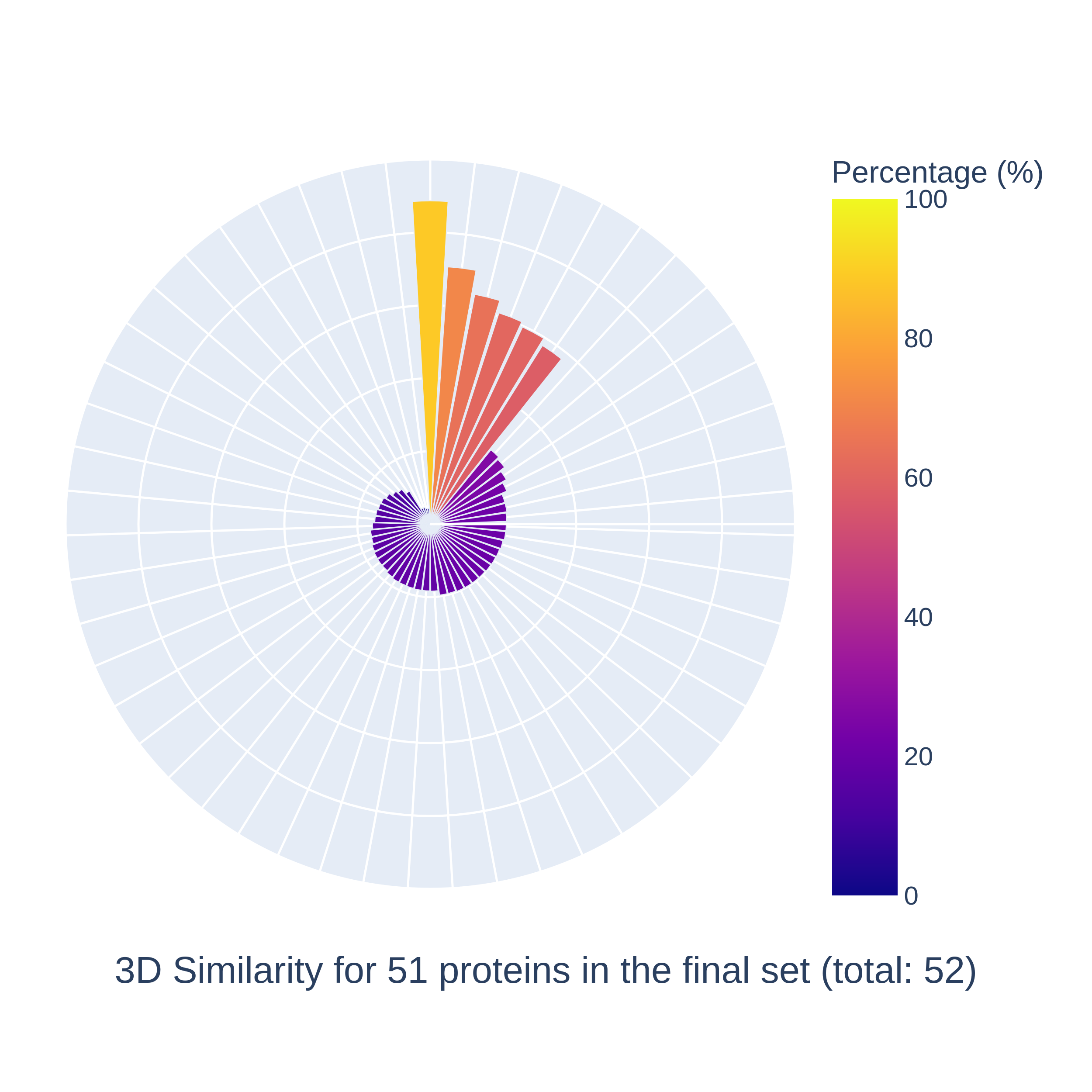

Supplement: Supplementary file 6 — Supplementary Data 3 [file 42003_2023_5076_MOESM6_ESM.zip › 7V7Q_A_whole/plots/7V7Q_A_3D-score.png]

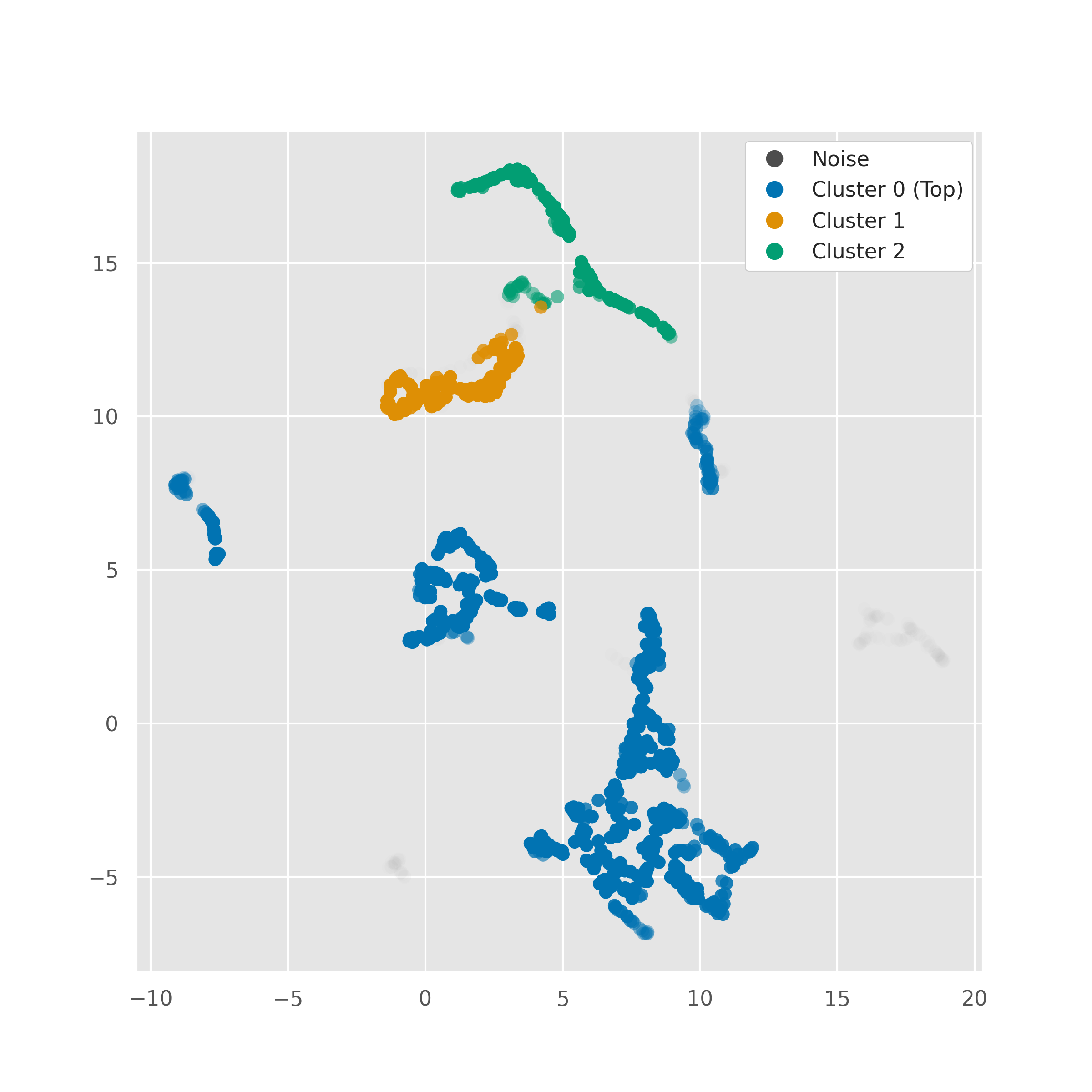

Supplement: Supplementary file 6 — Supplementary Data 3 [file 42003_2023_5076_MOESM6_ESM.zip › 7V7Q_A_whole/plots/7V7Q_A-clusters.png]

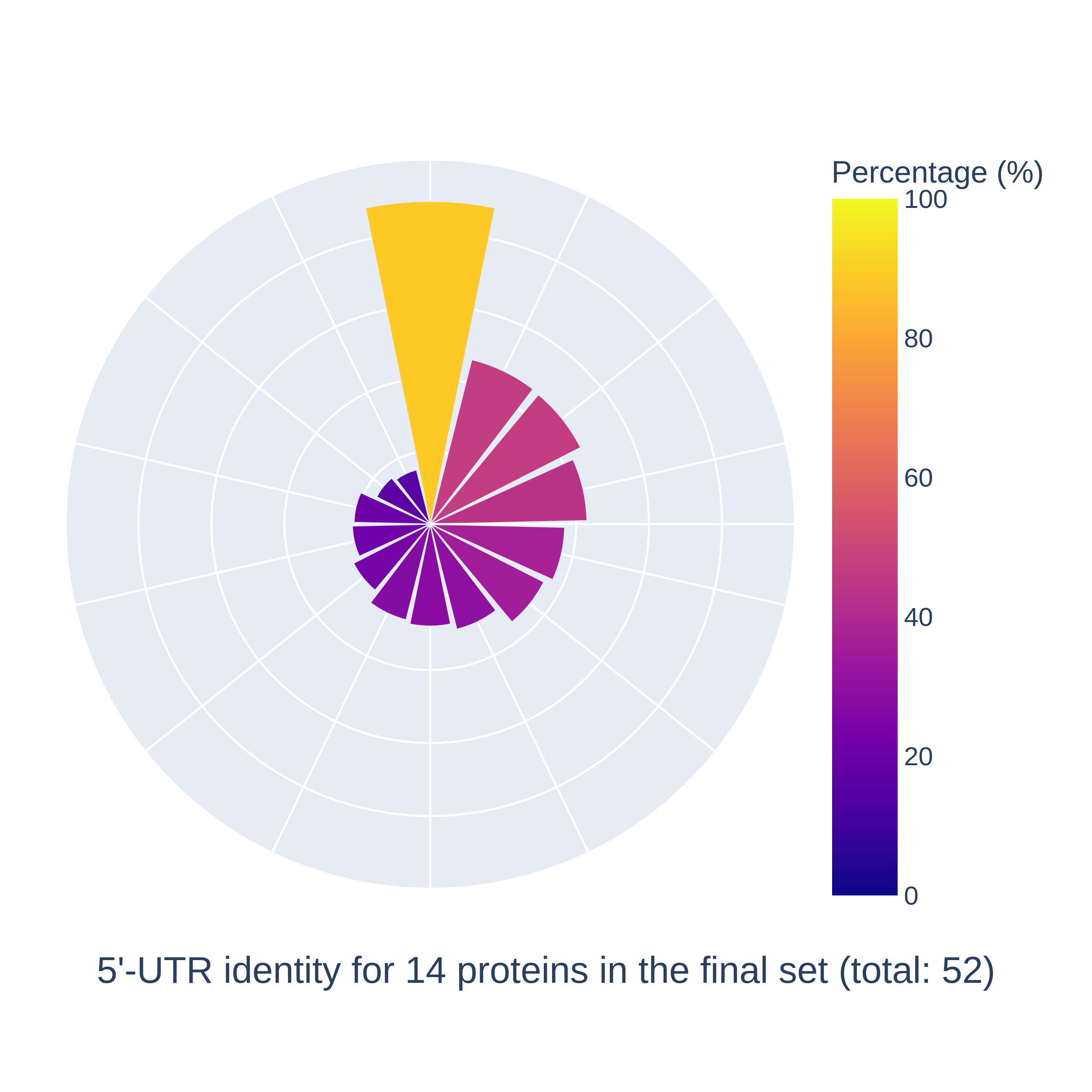

Supplement: Supplementary file 6 — Supplementary Data 3 [file 42003_2023_5076_MOESM6_ESM.zip › 7V7Q_A_whole/plots/7V7Q_A_5UTR-identity.png]

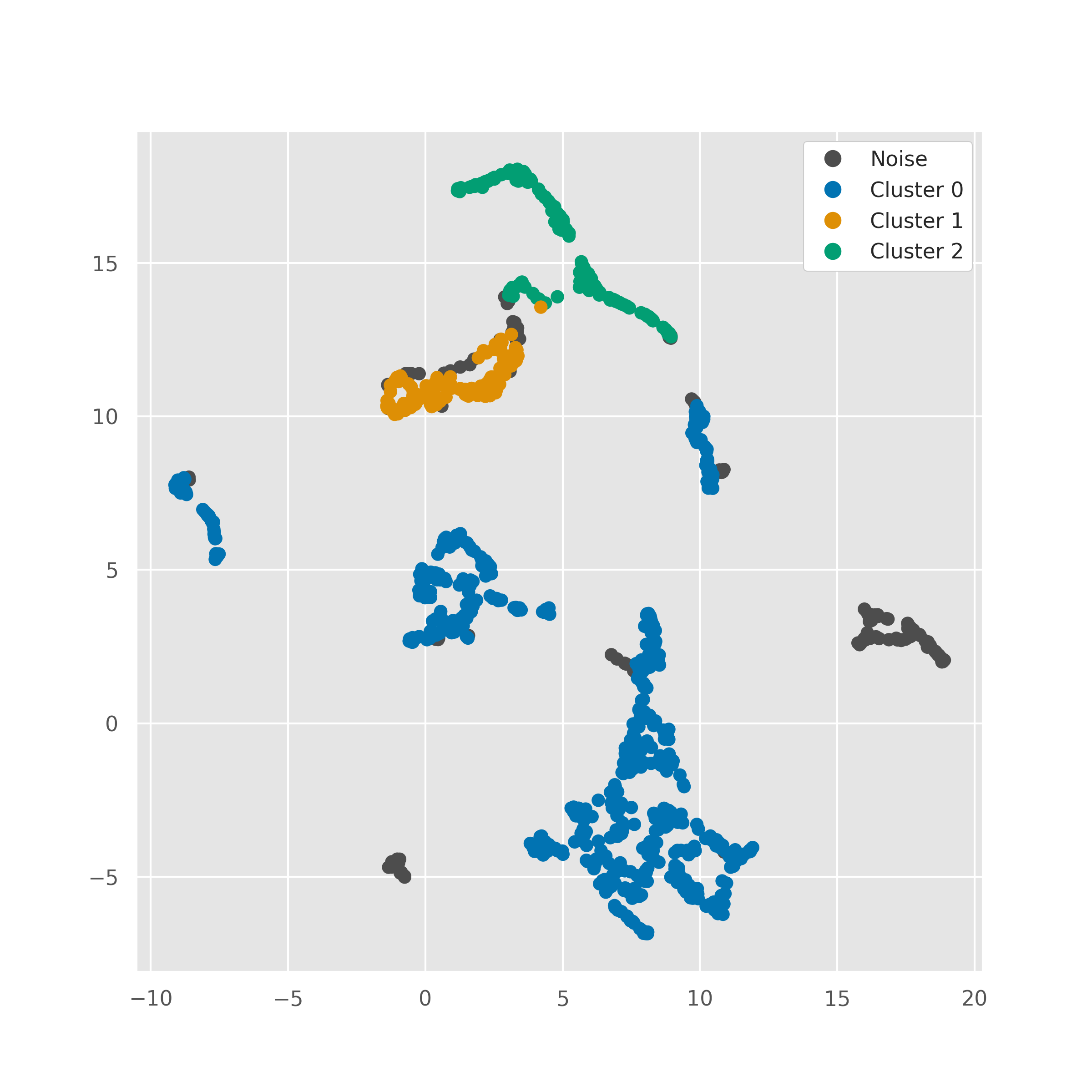

Supplement: Supplementary file 6 — Supplementary Data 3 [file 42003_2023_5076_MOESM6_ESM.zip › 7V7Q_A_whole/plots/7V7Q_A-clusters-initial.png]

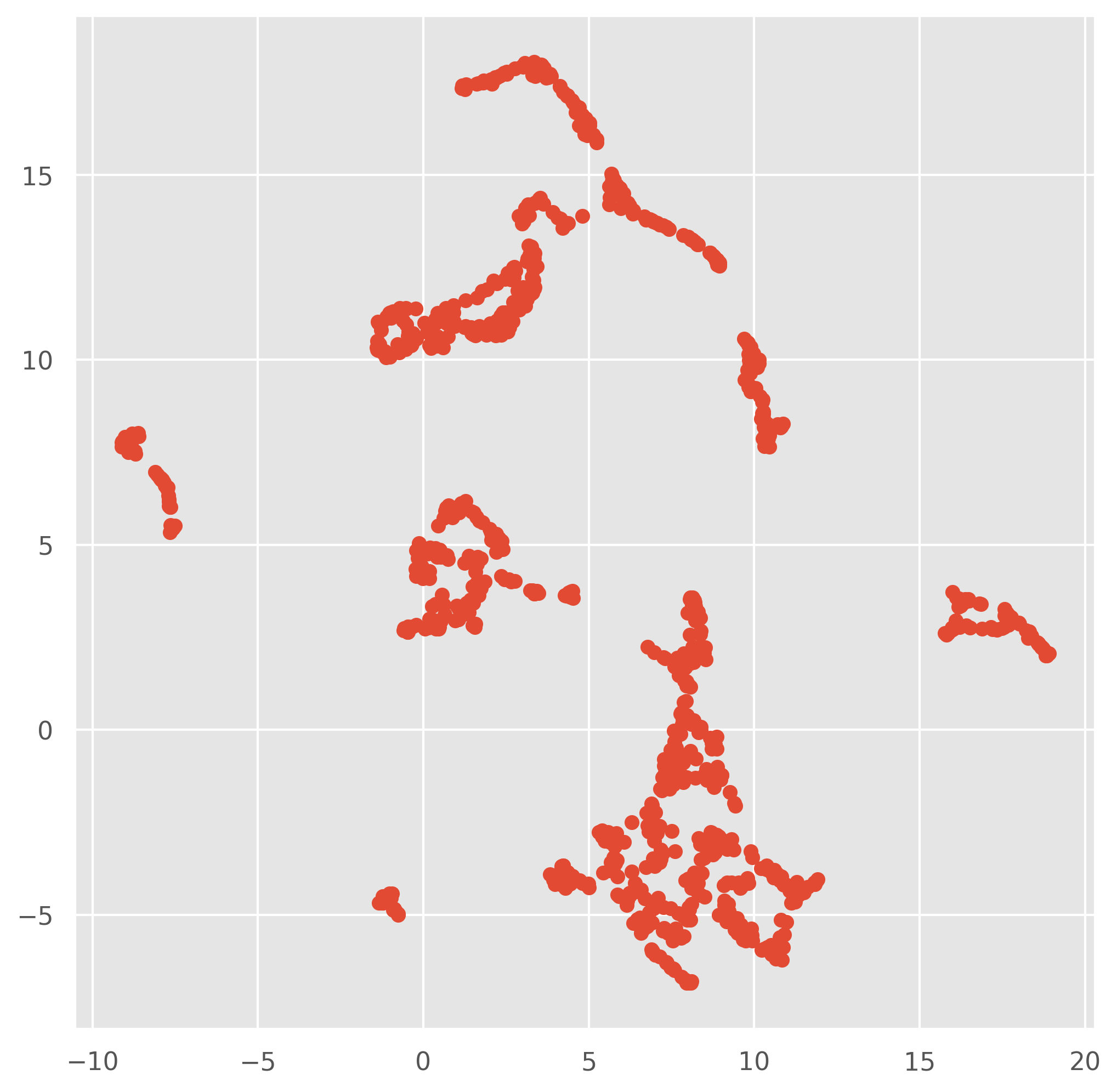

Supplement: Supplementary file 6 — Supplementary Data 3 [file 42003_2023_5076_MOESM6_ESM.zip › 7V7Q_A_whole/plots/7V7Q_A-UMAP-.png]

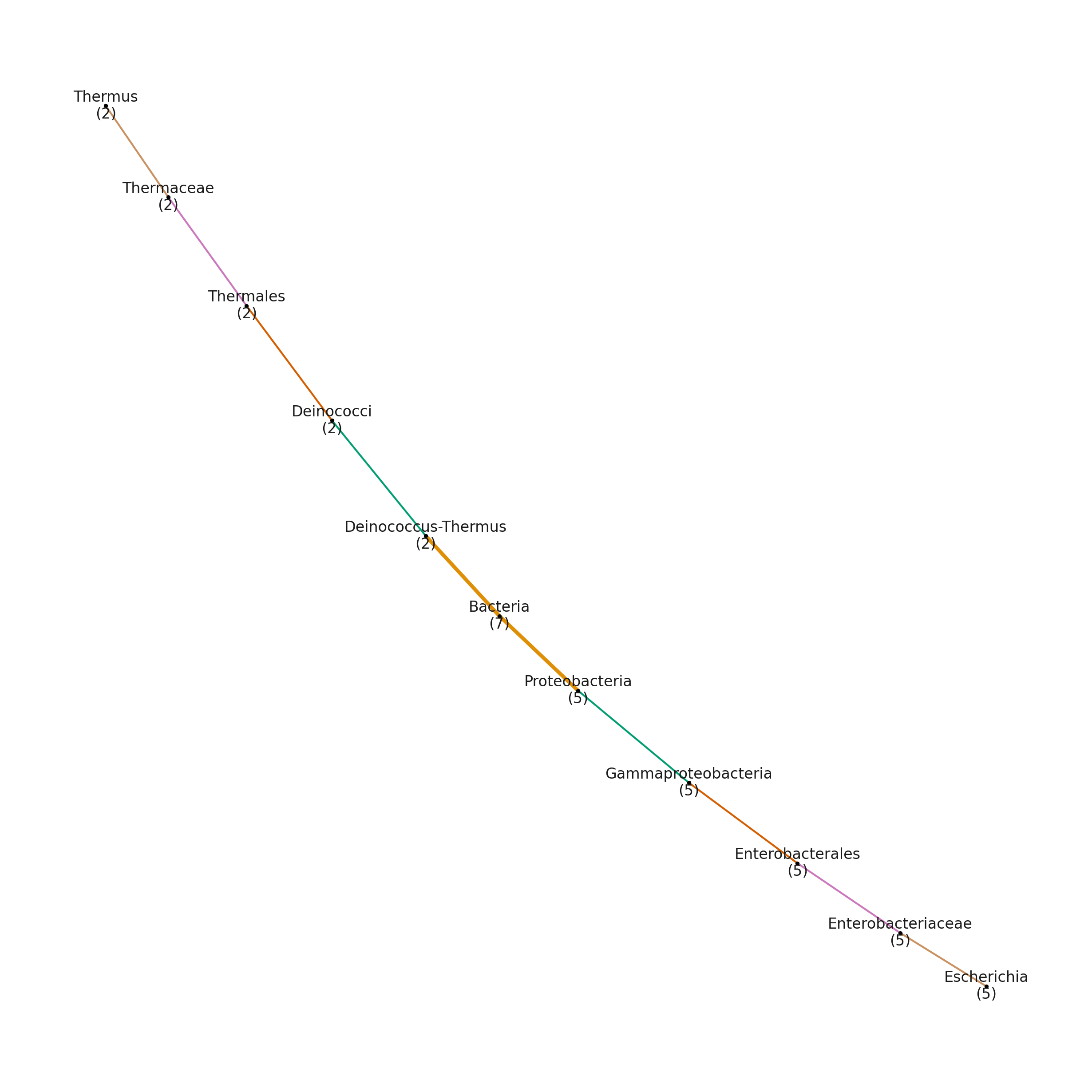

Supplement: Supplementary file 6 — Supplementary Data 3 [file 42003_2023_5076_MOESM6_ESM.zip › 7V7Q_A_whole/plots/7V7Q_A-Bacteria-tree.png]

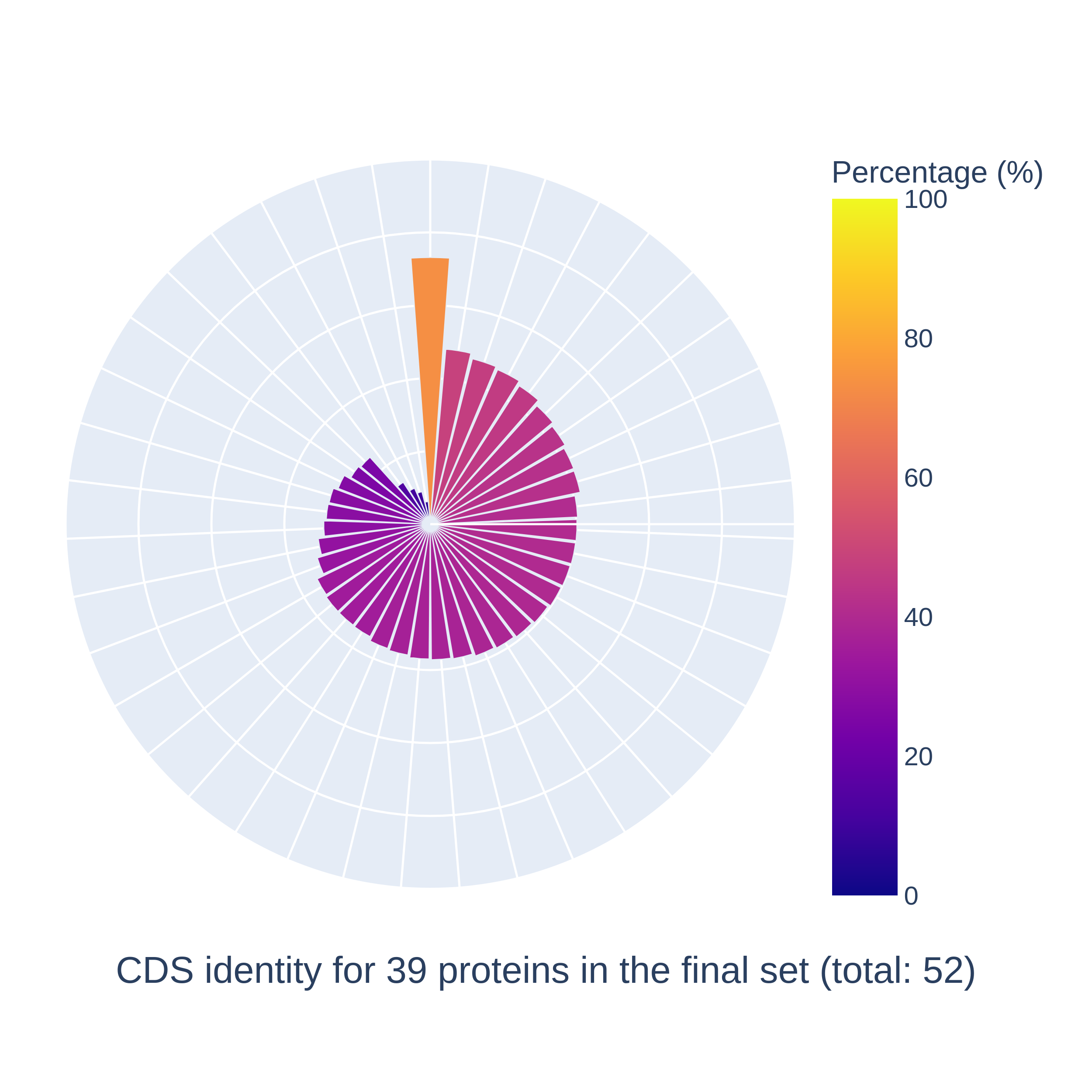

Supplement: Supplementary file 6 — Supplementary Data 3 [file 42003_2023_5076_MOESM6_ESM.zip › 7V7Q_A_whole/plots/7V7Q_A_CDS-identity.png]

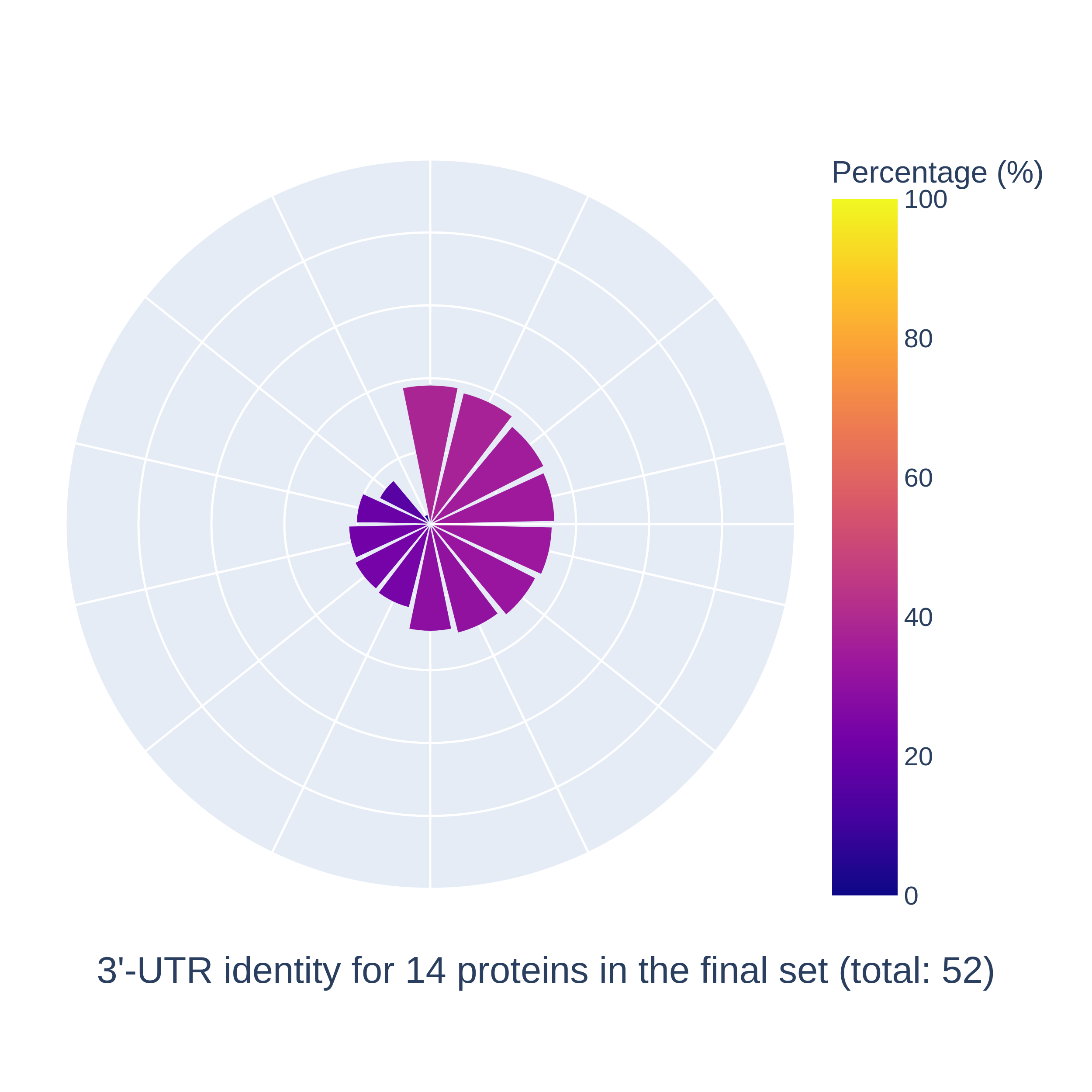

Supplement: Supplementary file 6 — Supplementary Data 3 [file 42003_2023_5076_MOESM6_ESM.zip › 7V7Q_A_whole/plots/7V7Q_A_3UTR-identity.png]

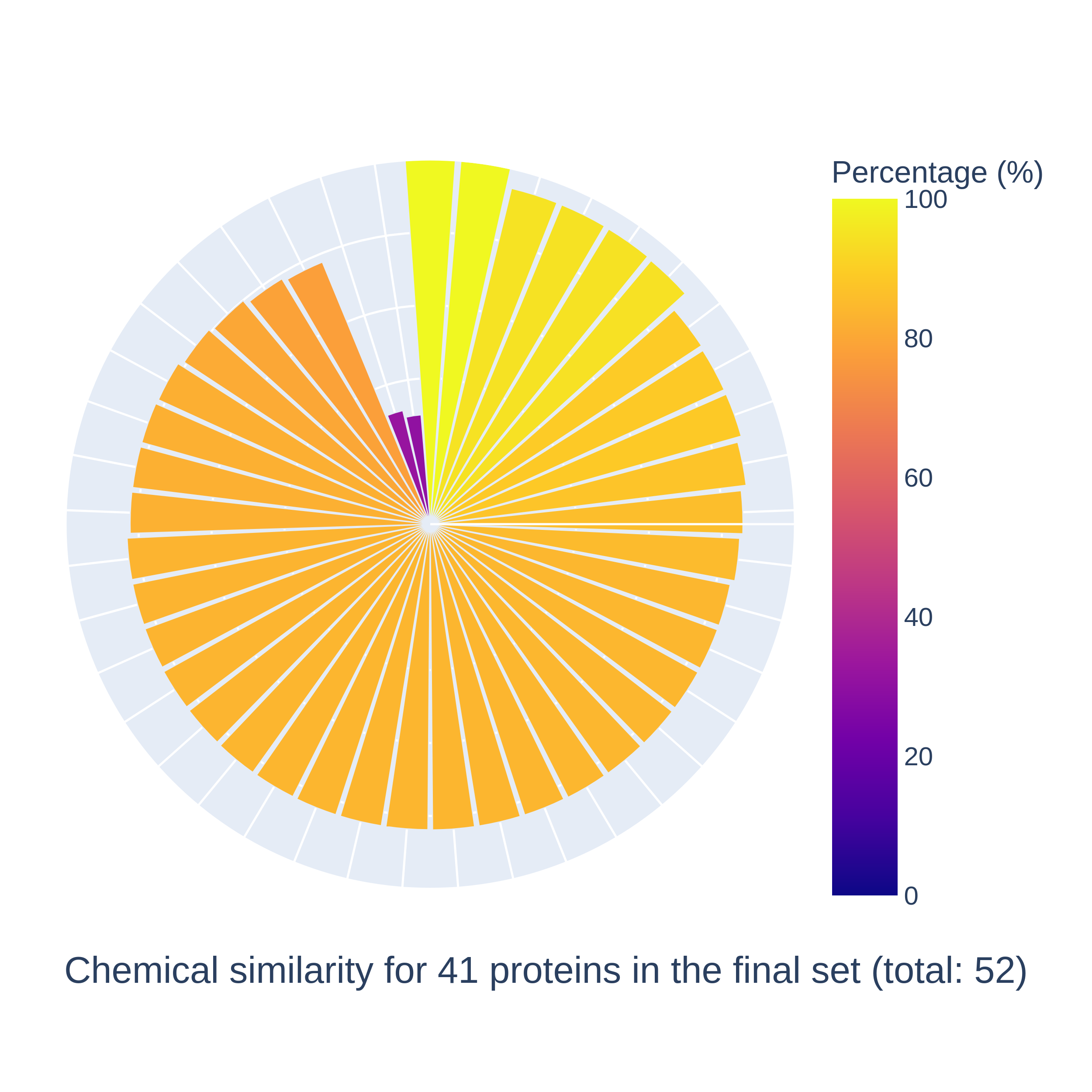

Supplement: Supplementary file 6 — Supplementary Data 3 [file 42003_2023_5076_MOESM6_ESM.zip › 7V7Q_A_whole/plots/7V7Q_A_chemSim.png]

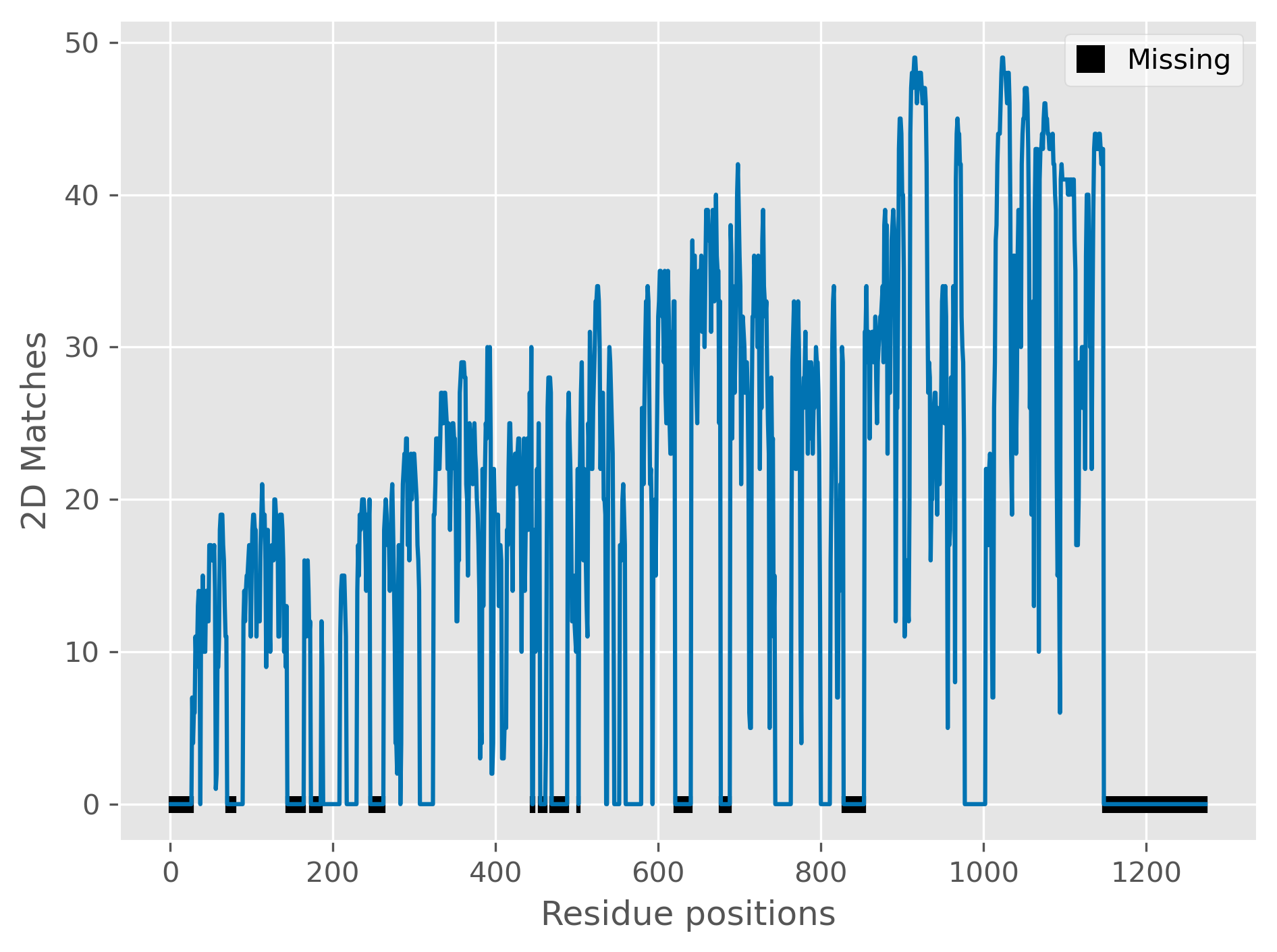

Supplement: Supplementary file 6 — Supplementary Data 3 [file 42003_2023_5076_MOESM6_ESM.zip › 6VXX_A_whole/plots/6VXX_A-2Dmatches.png]

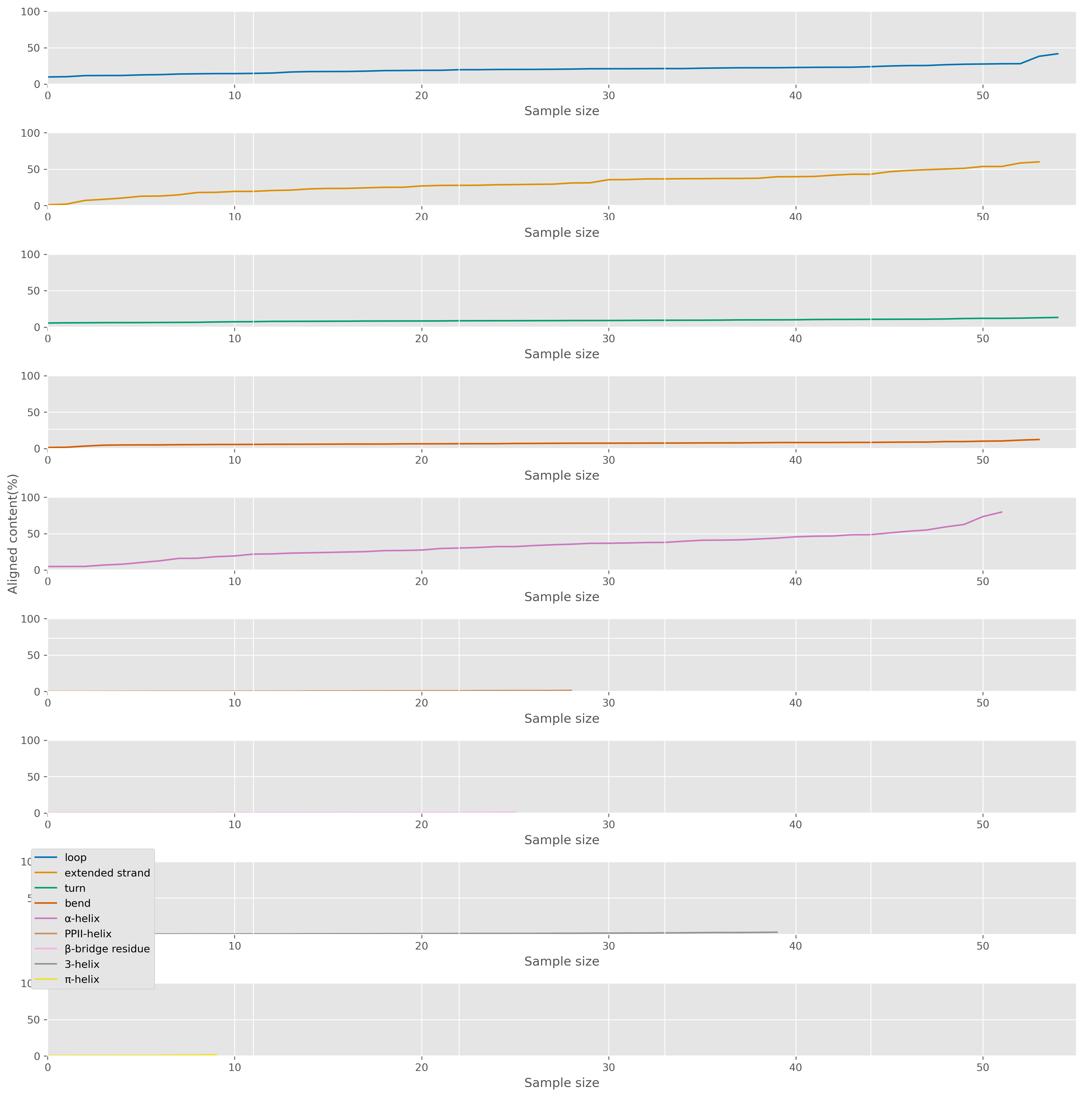

Supplement: Supplementary file 6 — Supplementary Data 3 [file 42003_2023_5076_MOESM6_ESM.zip › 6VXX_A_whole/plots/6VXX_A-2Dfold_coverage.png]

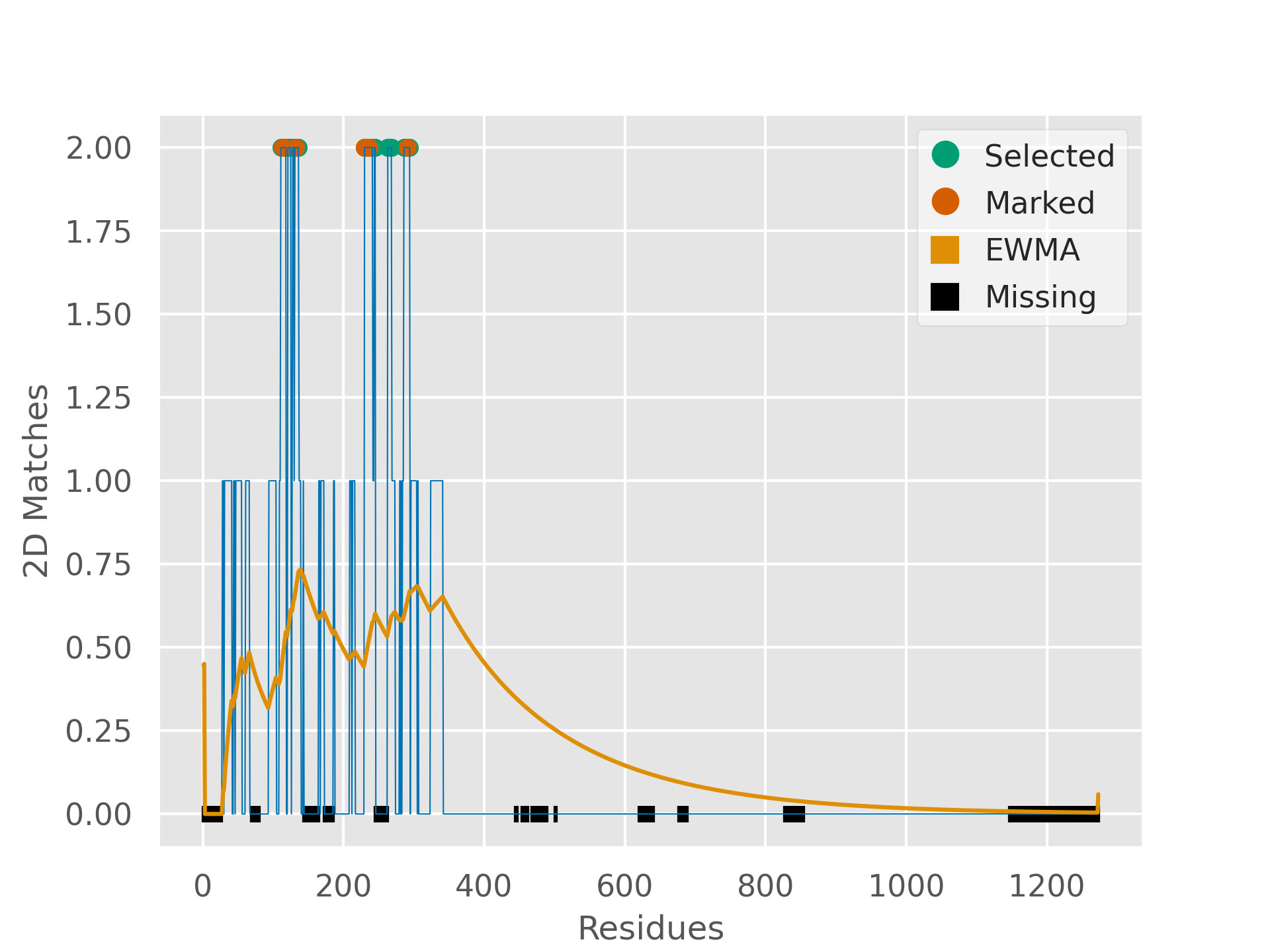

Supplement: Supplementary file 7 — Supplementary Data 4 [file 42003_2023_5076_MOESM7_ESM.zip › 6VXX_A_domain/go/6VXX_A_BetaCoV-S1-CTD_angiogen_f93fb8e3849e46a79caacb66662bf0a9.png]

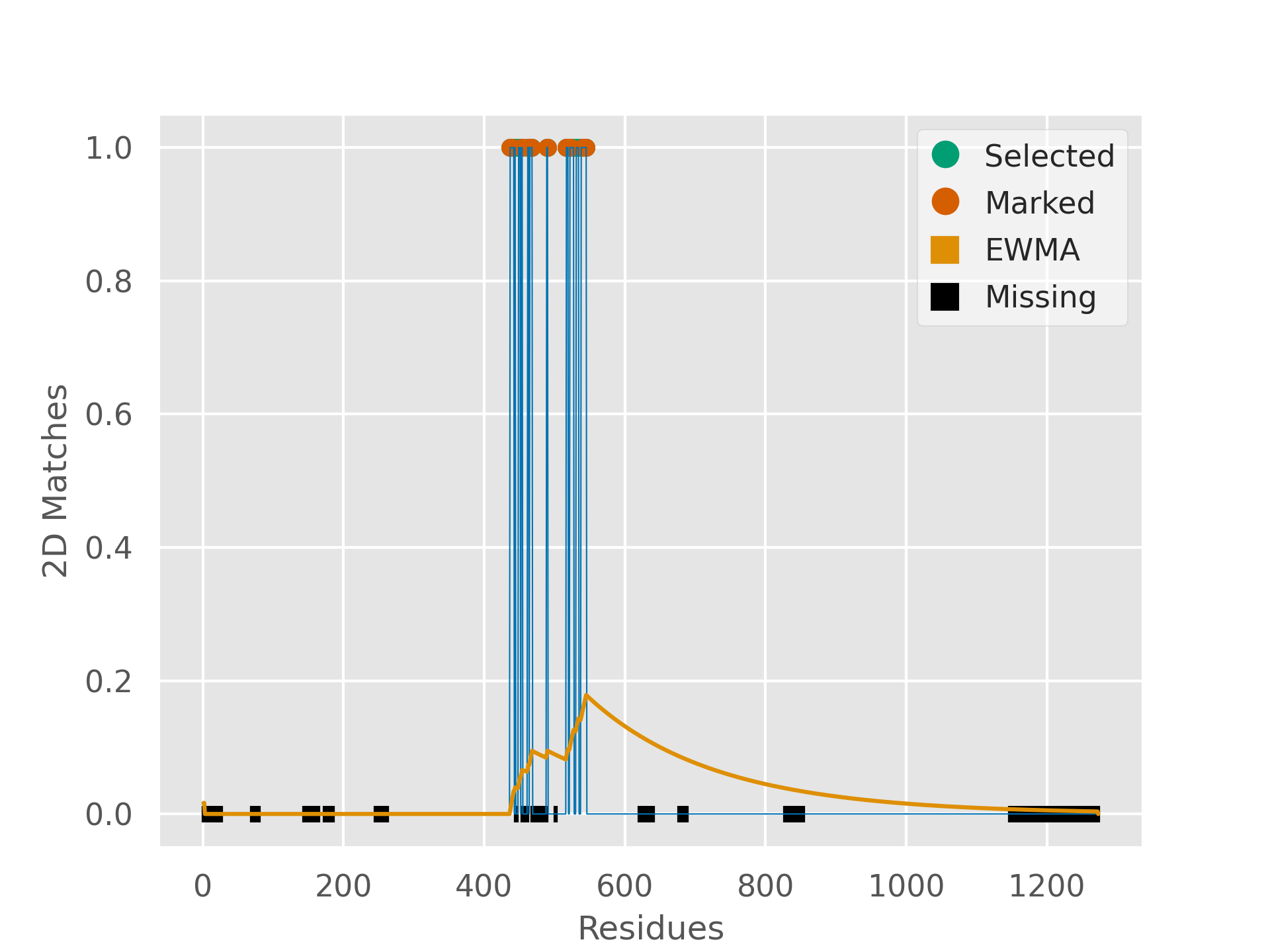

Supplement: Supplementary file 7 — Supplementary Data 4 [file 42003_2023_5076_MOESM7_ESM.zip › 6VXX_A_domain/go/6VXX_A_BetaCoV-S1-NTD_coagulation_6ad422107fc54289ad9e7d74c8c618f0.png]

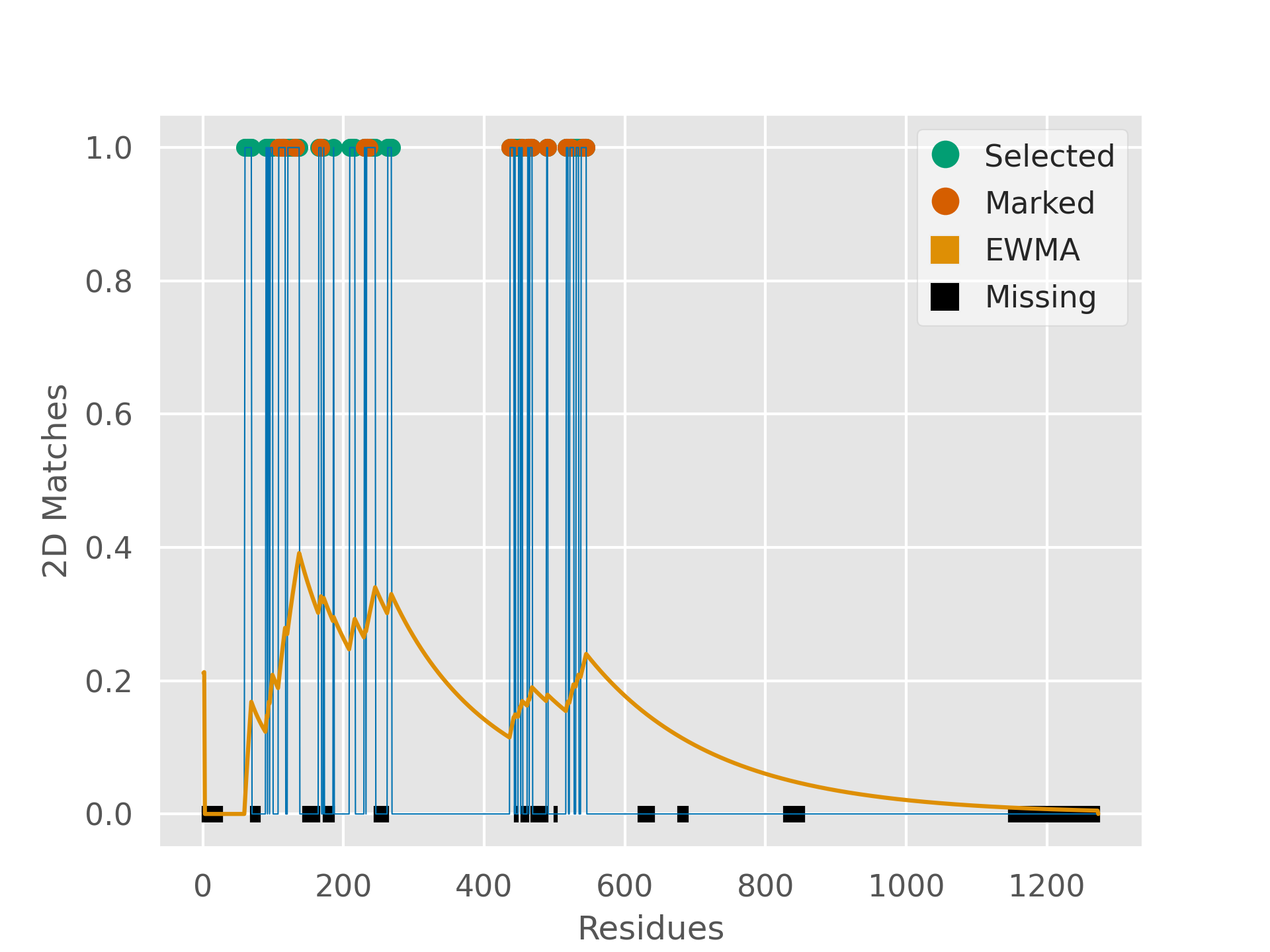

Supplement: Supplementary file 7 — Supplementary Data 4 [file 42003_2023_5076_MOESM7_ESM.zip › 6VXX_A_domain/go/6VXX_A_BetaCoV-S1-NTD_heart_3dd837ef7eac46c3a0599e7671456770.png]

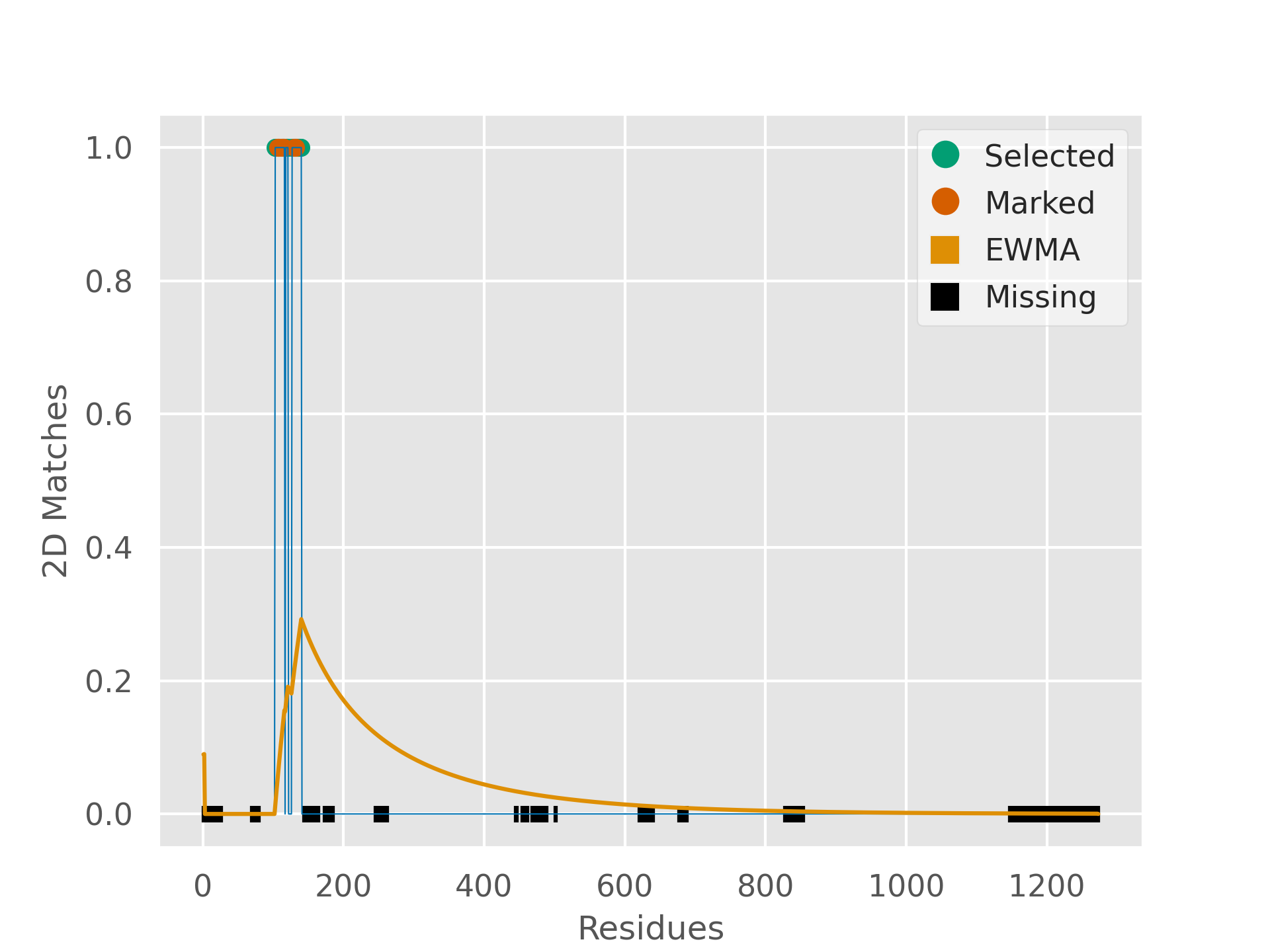

Supplement: Supplementary file 7 — Supplementary Data 4 [file 42003_2023_5076_MOESM7_ESM.zip › 6VXX_A_domain/go/6VXX_A_RBD_coagulation_bc26f14f69054fc0815793ba10f7b930.png]

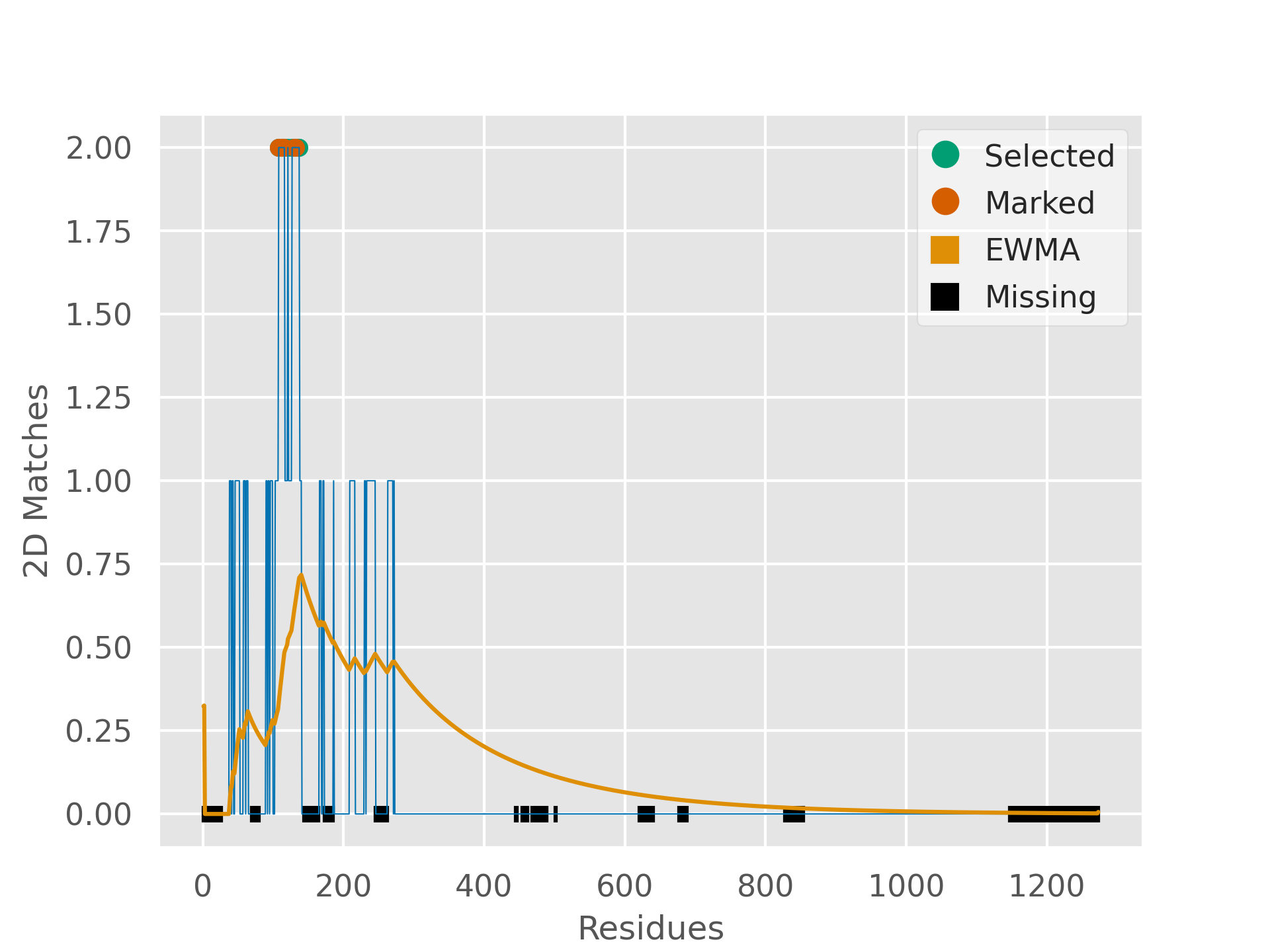

Supplement: Supplementary file 7 — Supplementary Data 4 [file 42003_2023_5076_MOESM7_ESM.zip › 6VXX_A_domain/go/6VXX_A_RBD_heart_f95e76cd80fc4cceb2c3af28d29cb4b9.png]

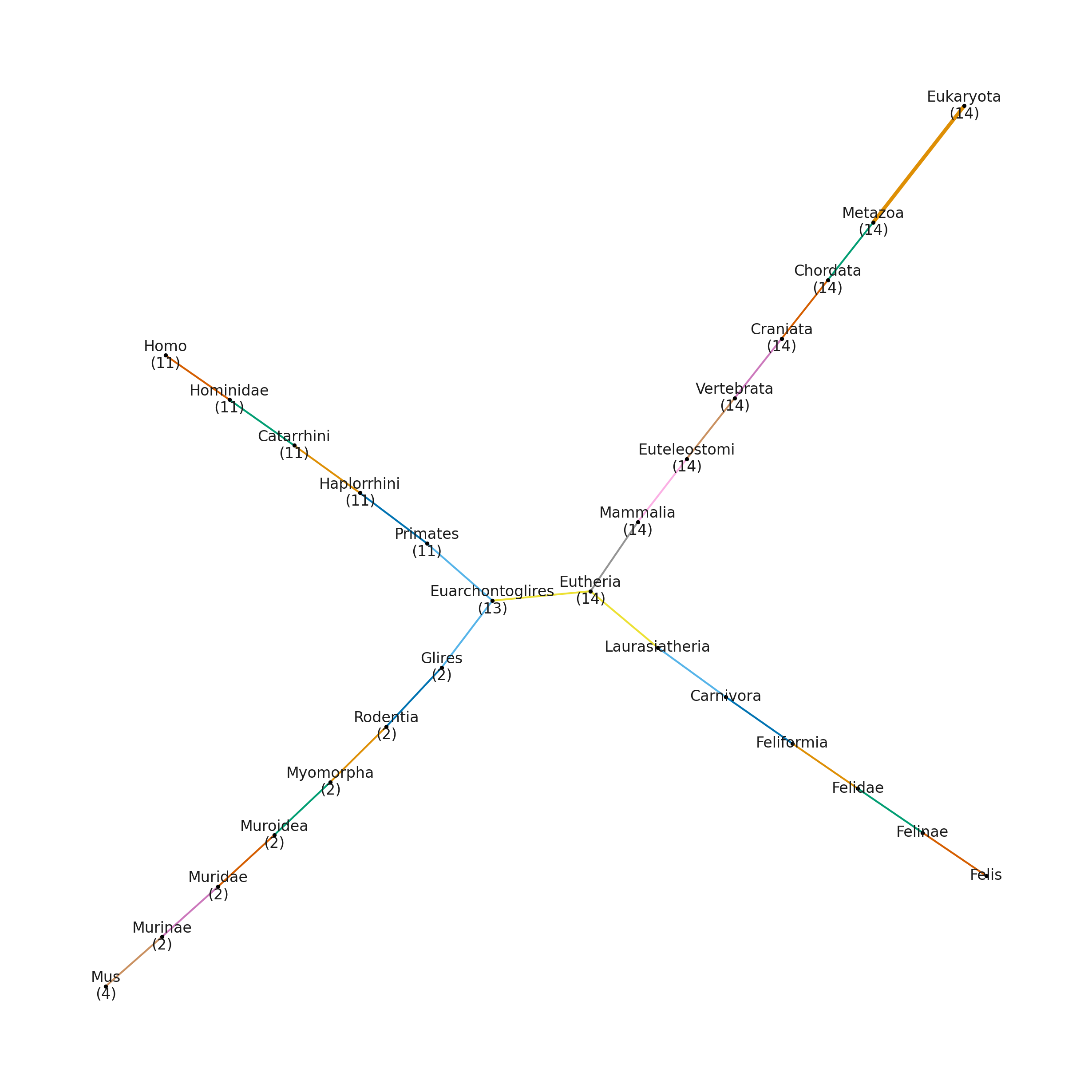

Supplement: Supplementary file 7 — Supplementary Data 4 [file 42003_2023_5076_MOESM7_ESM.zip › 6VXX_A_domain/plots/6VXX_A_BetaCoV-S1-CTD-Eukaryota-tree.png]

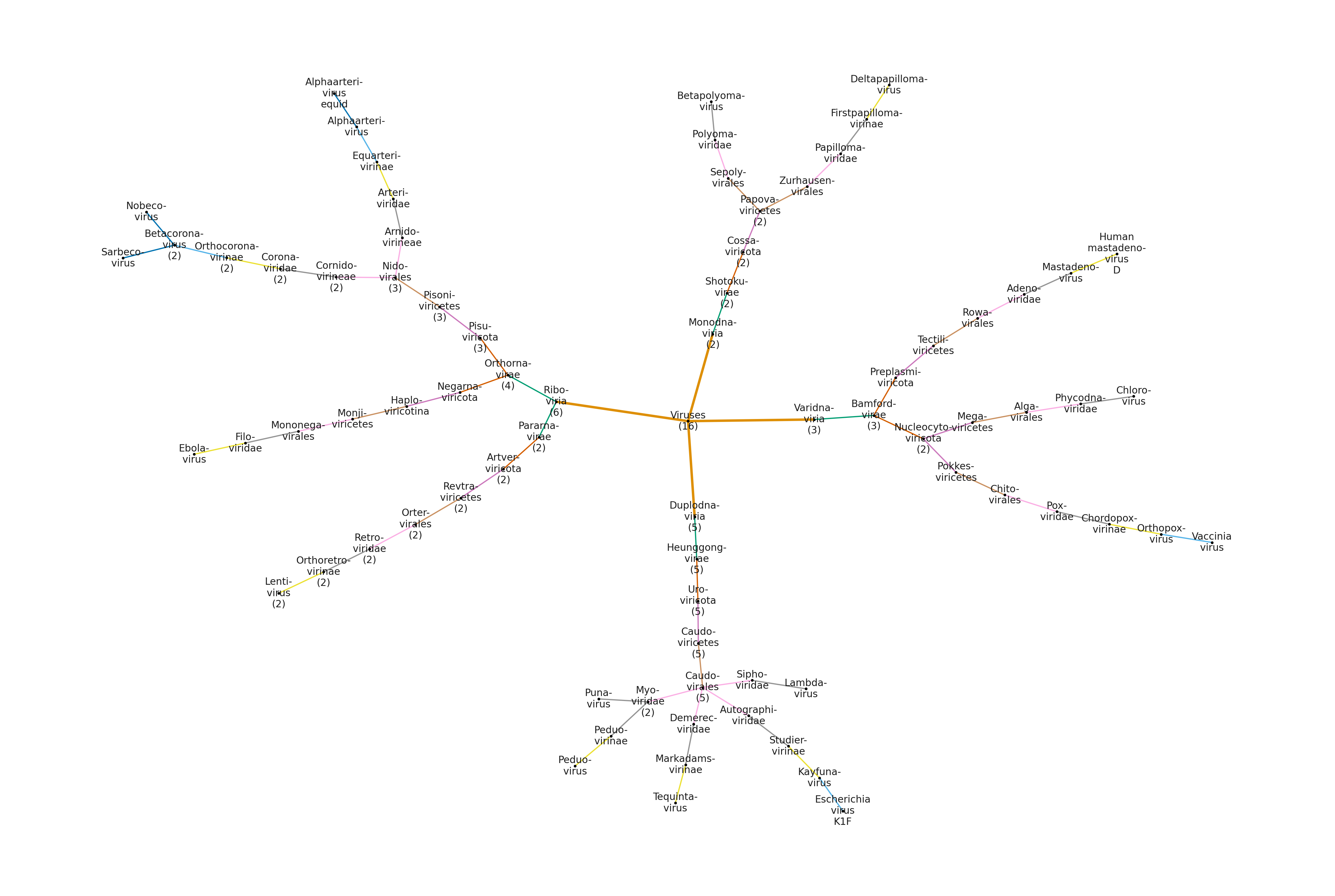

Supplement: Supplementary file 7 — Supplementary Data 4 [file 42003_2023_5076_MOESM7_ESM.zip › 6VXX_A_domain/plots/6VXX_A_BetaCoV-S1-CTD-Viruses-tree.png]

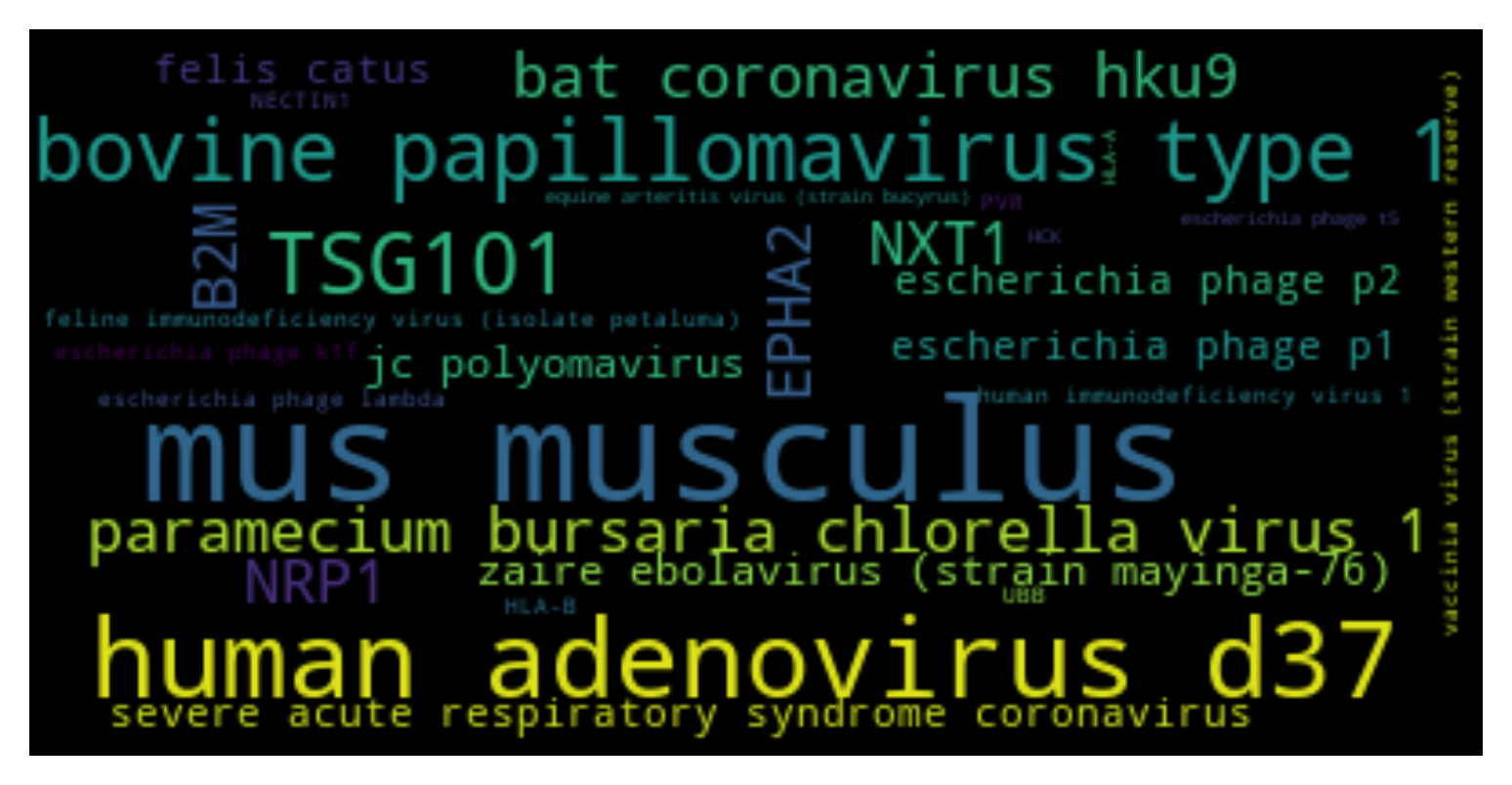

Supplement: Supplementary file 7 — Supplementary Data 4 [file 42003_2023_5076_MOESM7_ESM.zip › 6VXX_A_domain/plots/6VXX_A_BetaCoV-S1-CTD-wordcloud.png]

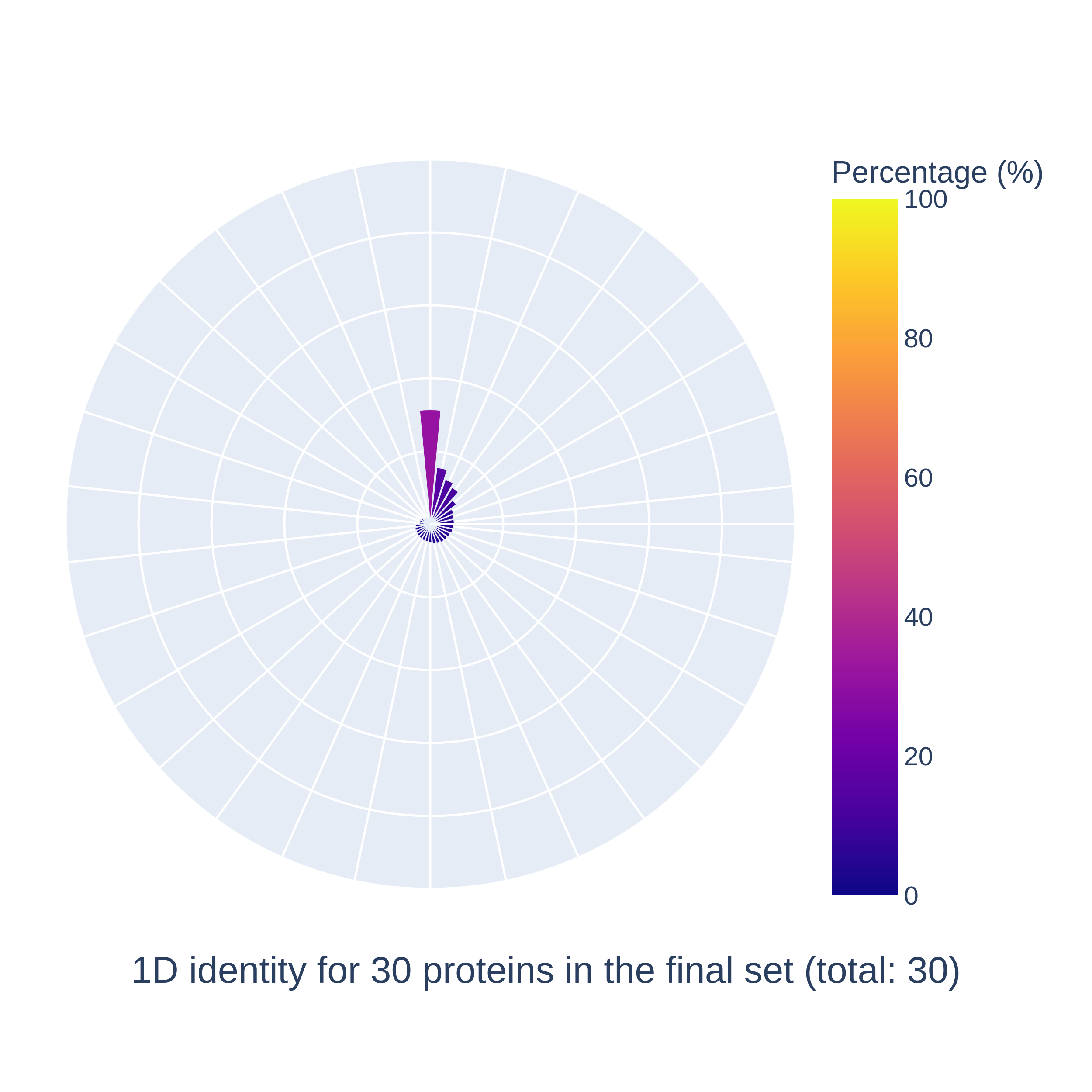

Supplement: Supplementary file 7 — Supplementary Data 4 [file 42003_2023_5076_MOESM7_ESM.zip › 6VXX_A_domain/plots/6VXX_A_BetaCoV-S1-CTD_1D-identity.png]

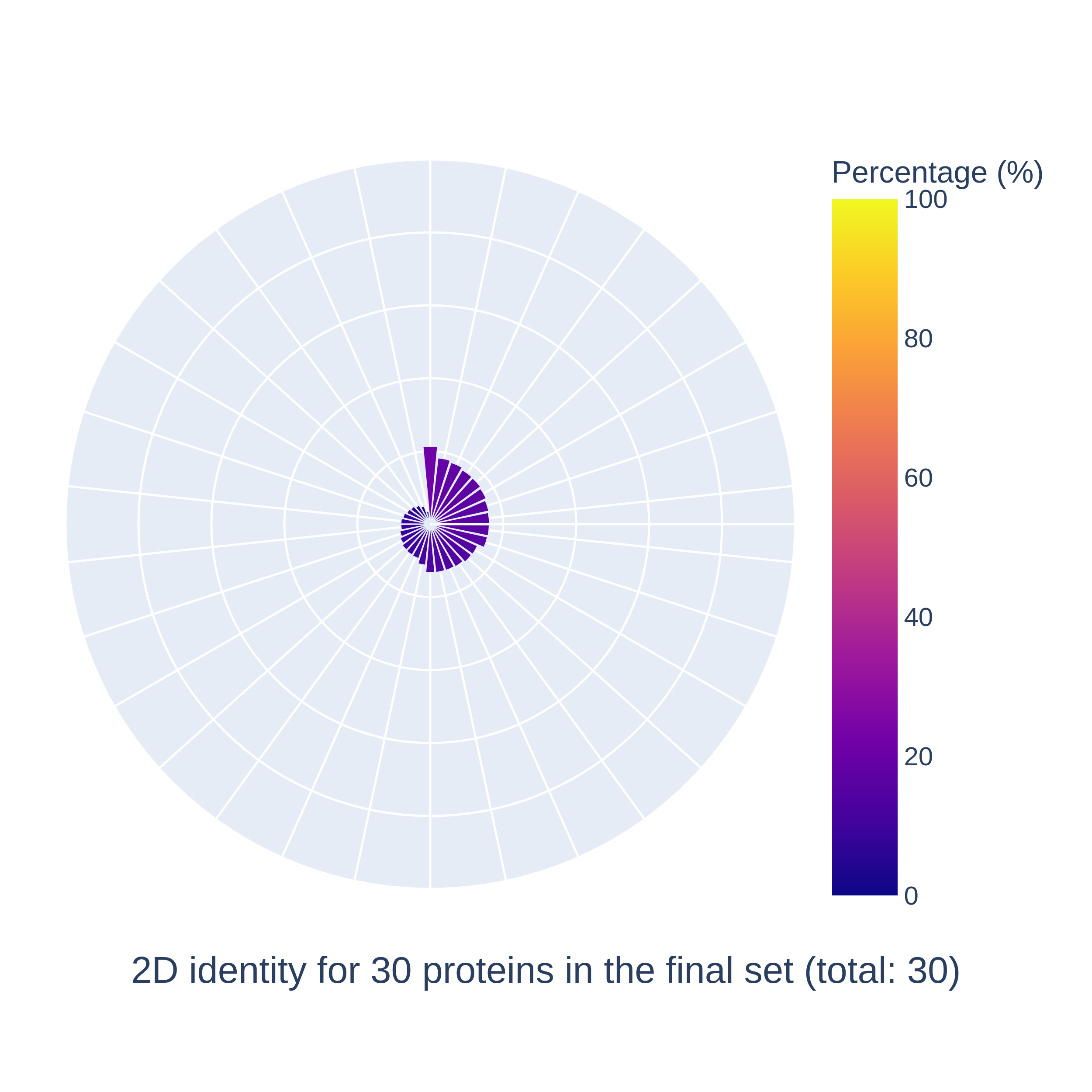

Supplement: Supplementary file 7 — Supplementary Data 4 [file 42003_2023_5076_MOESM7_ESM.zip › 6VXX_A_domain/plots/6VXX_A_BetaCoV-S1-CTD_2D-identity.png]

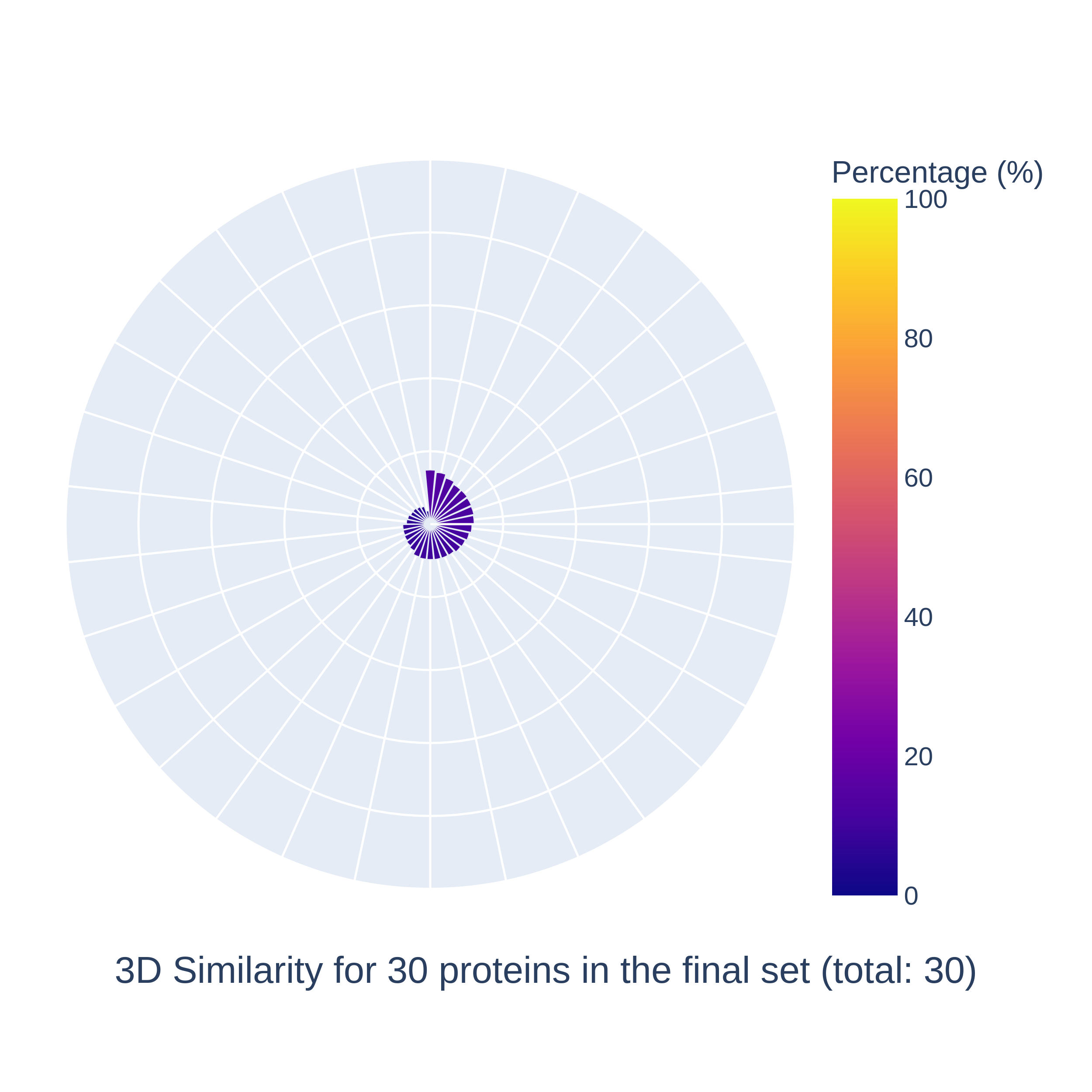

Supplement: Supplementary file 7 — Supplementary Data 4 [file 42003_2023_5076_MOESM7_ESM.zip › 6VXX_A_domain/plots/6VXX_A_BetaCoV-S1-CTD_3D-score.png]

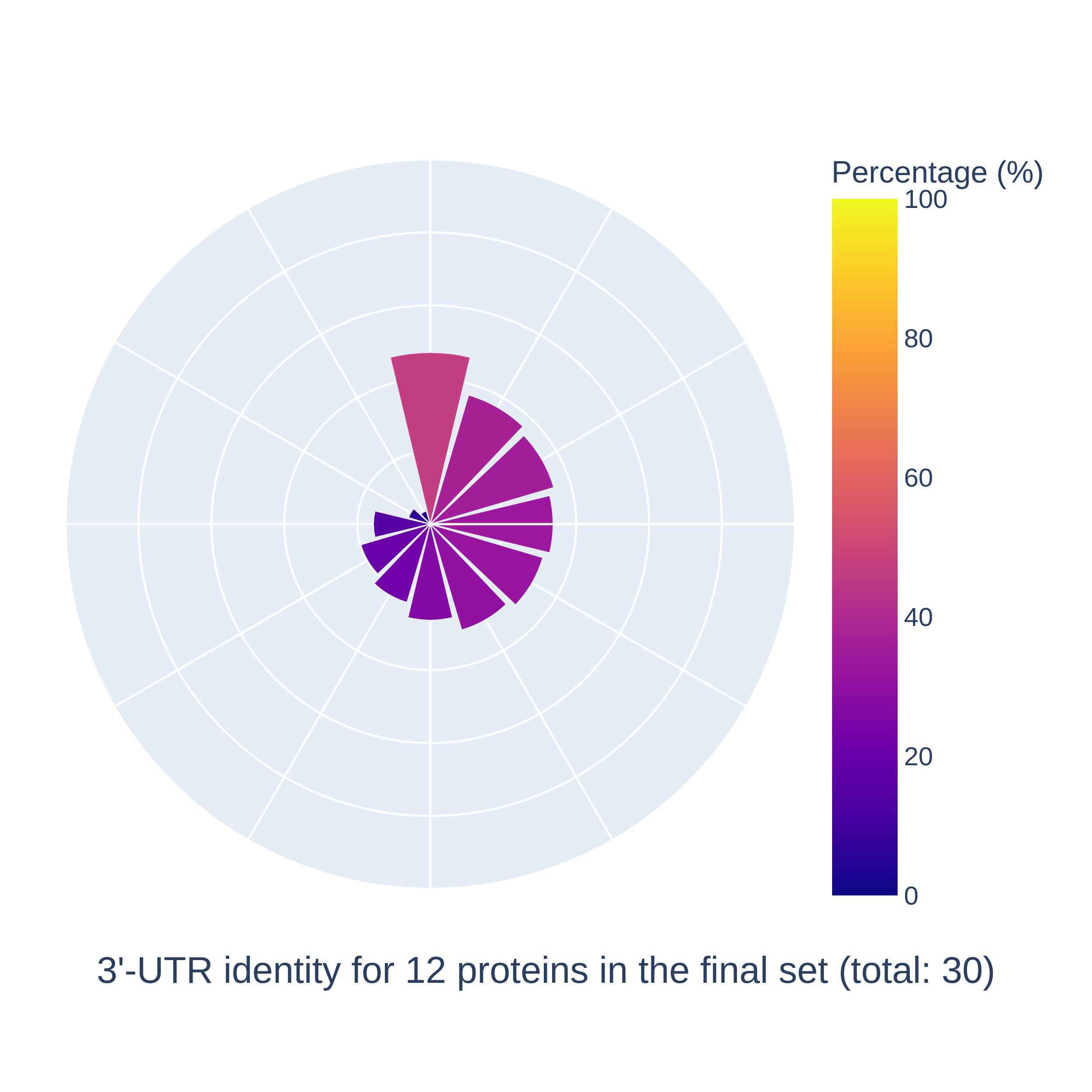

Supplement: Supplementary file 7 — Supplementary Data 4 [file 42003_2023_5076_MOESM7_ESM.zip › 6VXX_A_domain/plots/6VXX_A_BetaCoV-S1-CTD_3UTR-identity.png]

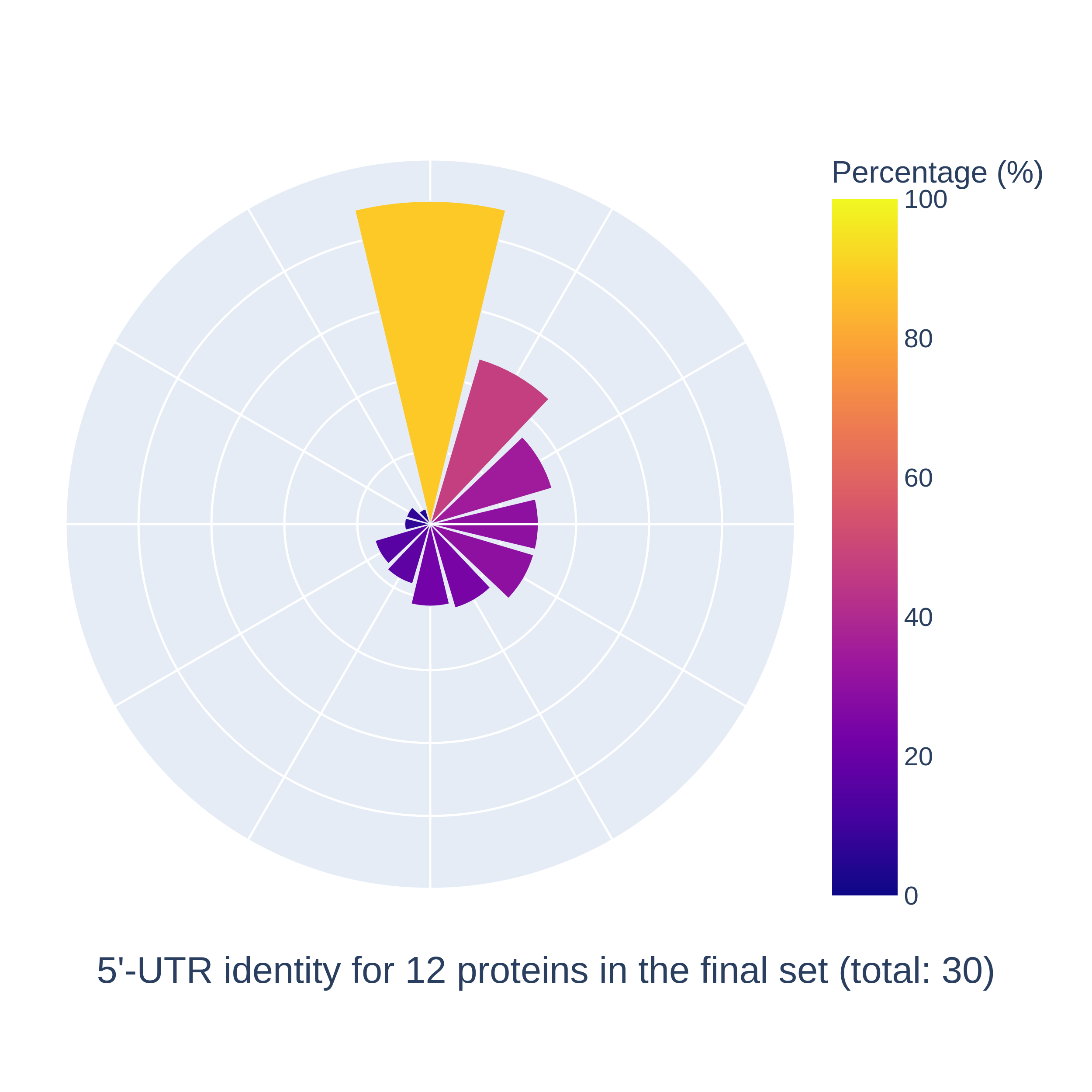

Supplement: Supplementary file 7 — Supplementary Data 4 [file 42003_2023_5076_MOESM7_ESM.zip › 6VXX_A_domain/plots/6VXX_A_BetaCoV-S1-CTD_5UTR-identity.png]

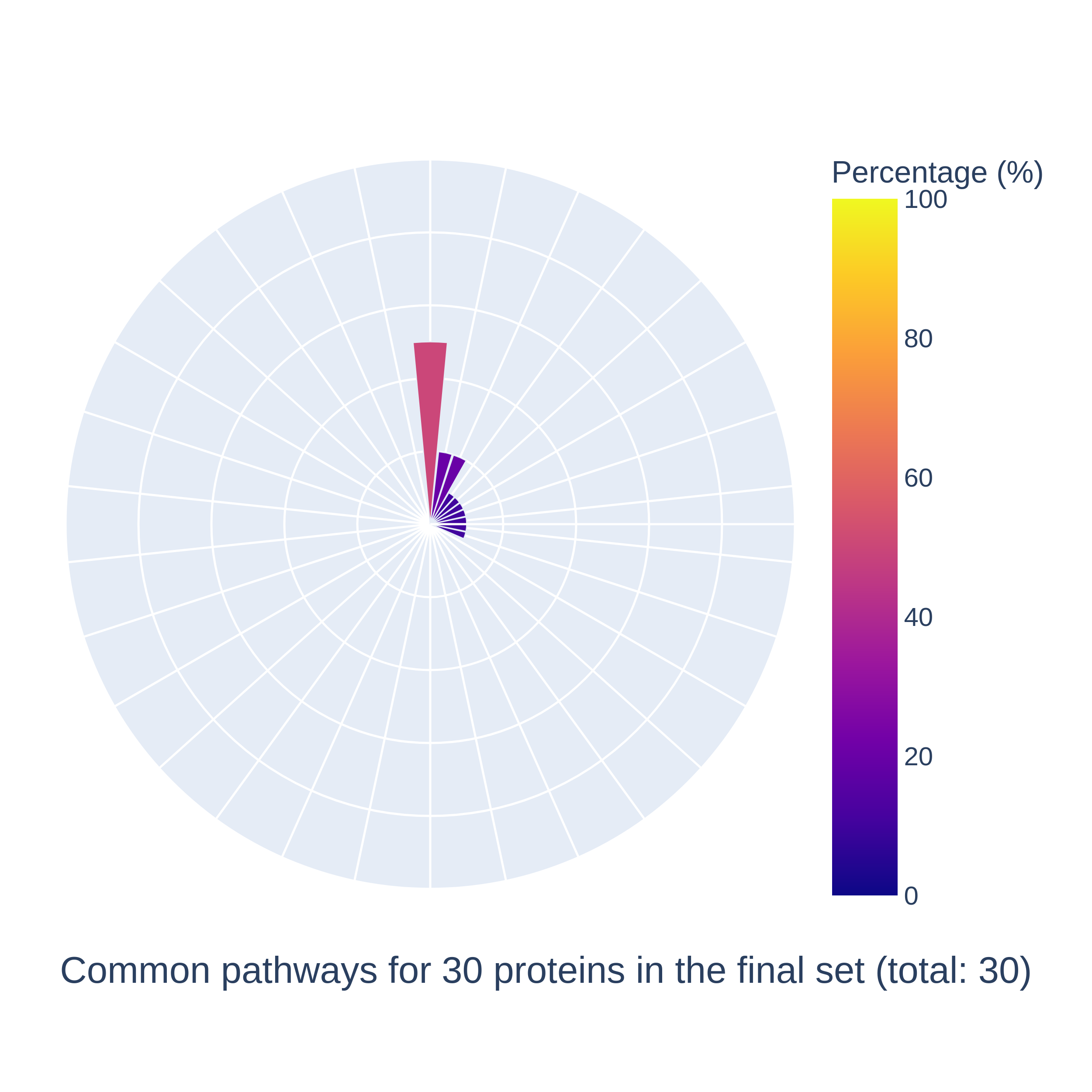

Supplement: Supplementary file 7 — Supplementary Data 4 [file 42003_2023_5076_MOESM7_ESM.zip › 6VXX_A_domain/plots/6VXX_A_BetaCoV-S1-CTD_biologicalProcessSim.png]

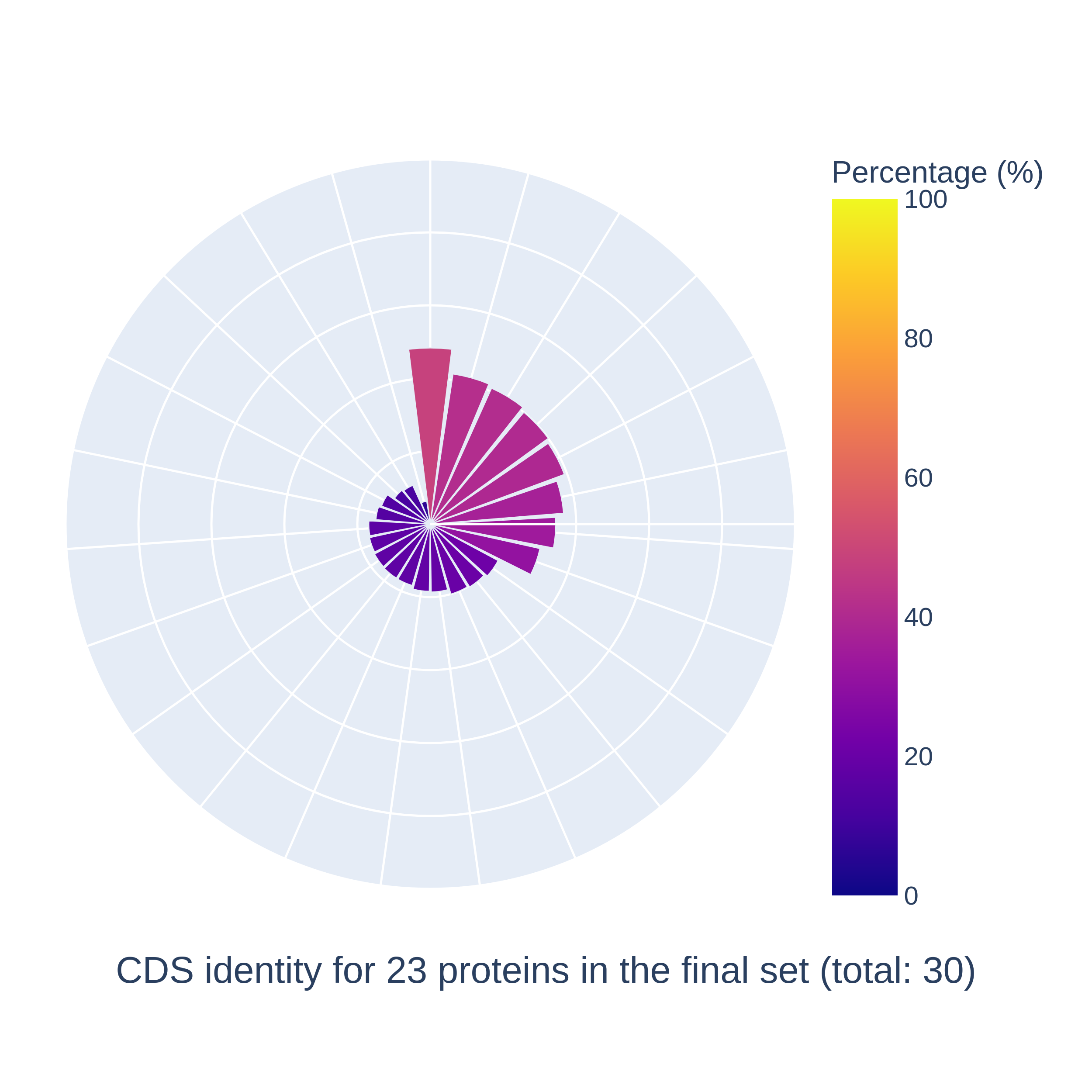

Supplement: Supplementary file 7 — Supplementary Data 4 [file 42003_2023_5076_MOESM7_ESM.zip › 6VXX_A_domain/plots/6VXX_A_BetaCoV-S1-CTD_CDS-identity.png]

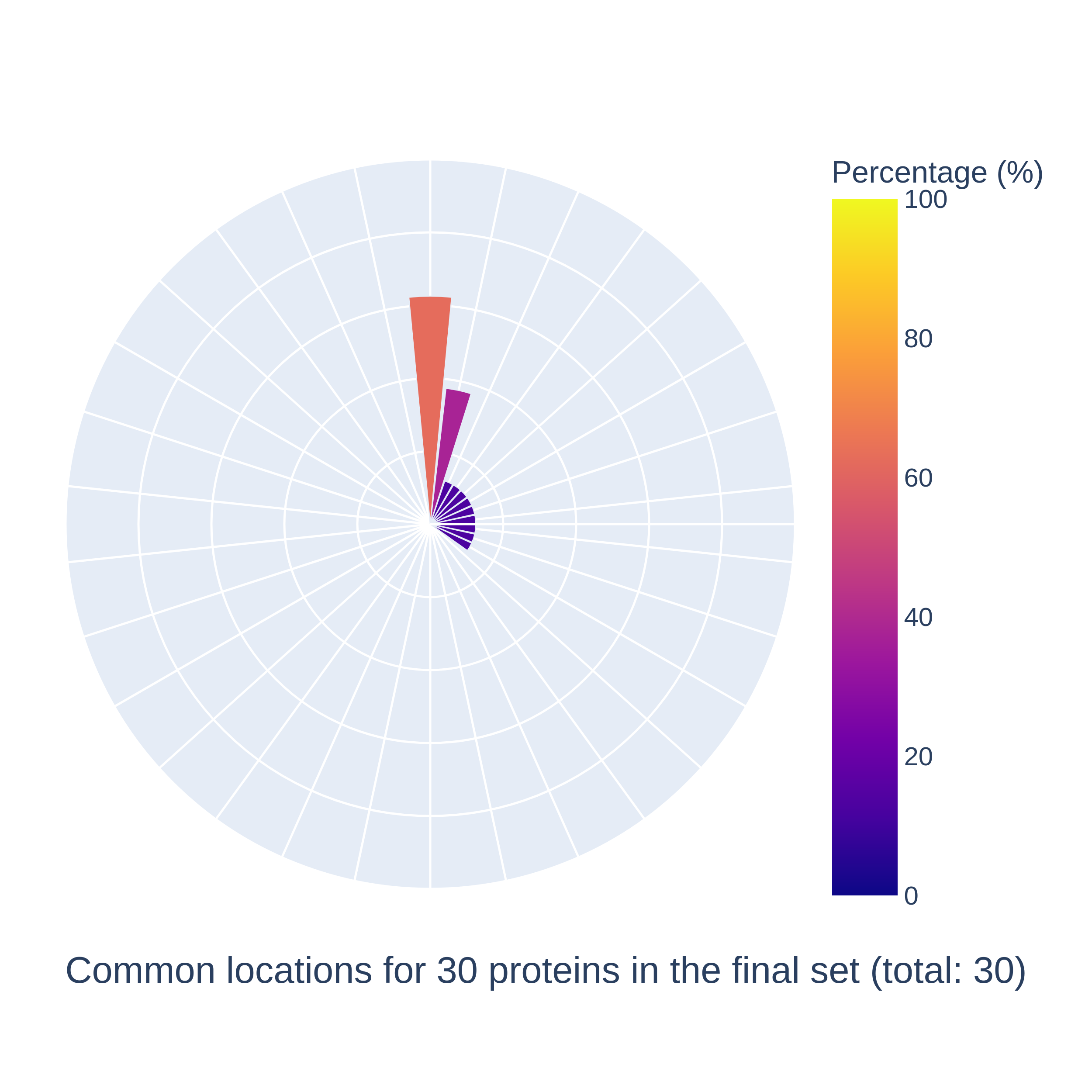

Supplement: Supplementary file 7 — Supplementary Data 4 [file 42003_2023_5076_MOESM7_ESM.zip › 6VXX_A_domain/plots/6VXX_A_BetaCoV-S1-CTD_cellularComponentSim.png]

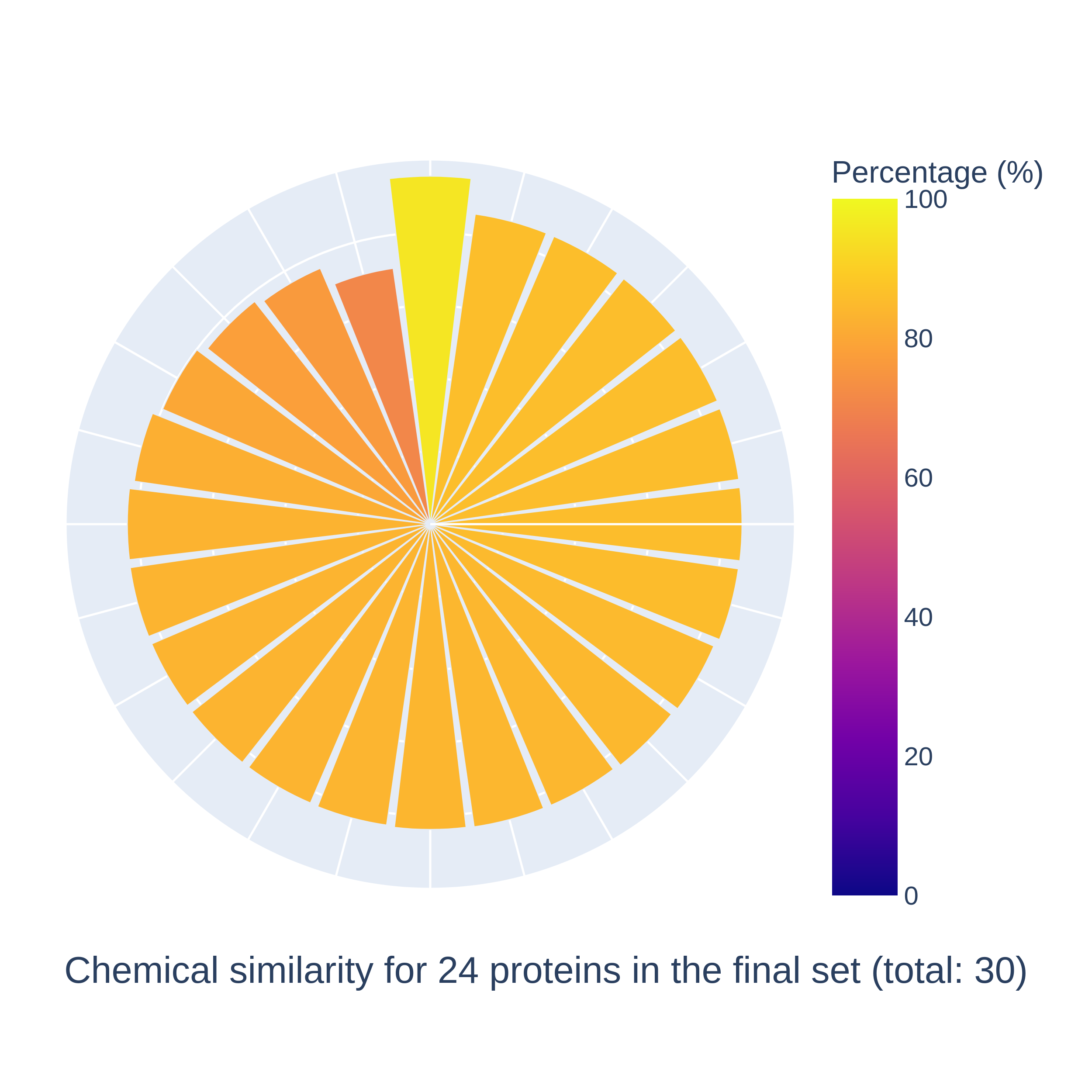

Supplement: Supplementary file 7 — Supplementary Data 4 [file 42003_2023_5076_MOESM7_ESM.zip › 6VXX_A_domain/plots/6VXX_A_BetaCoV-S1-CTD_chemSim.png]

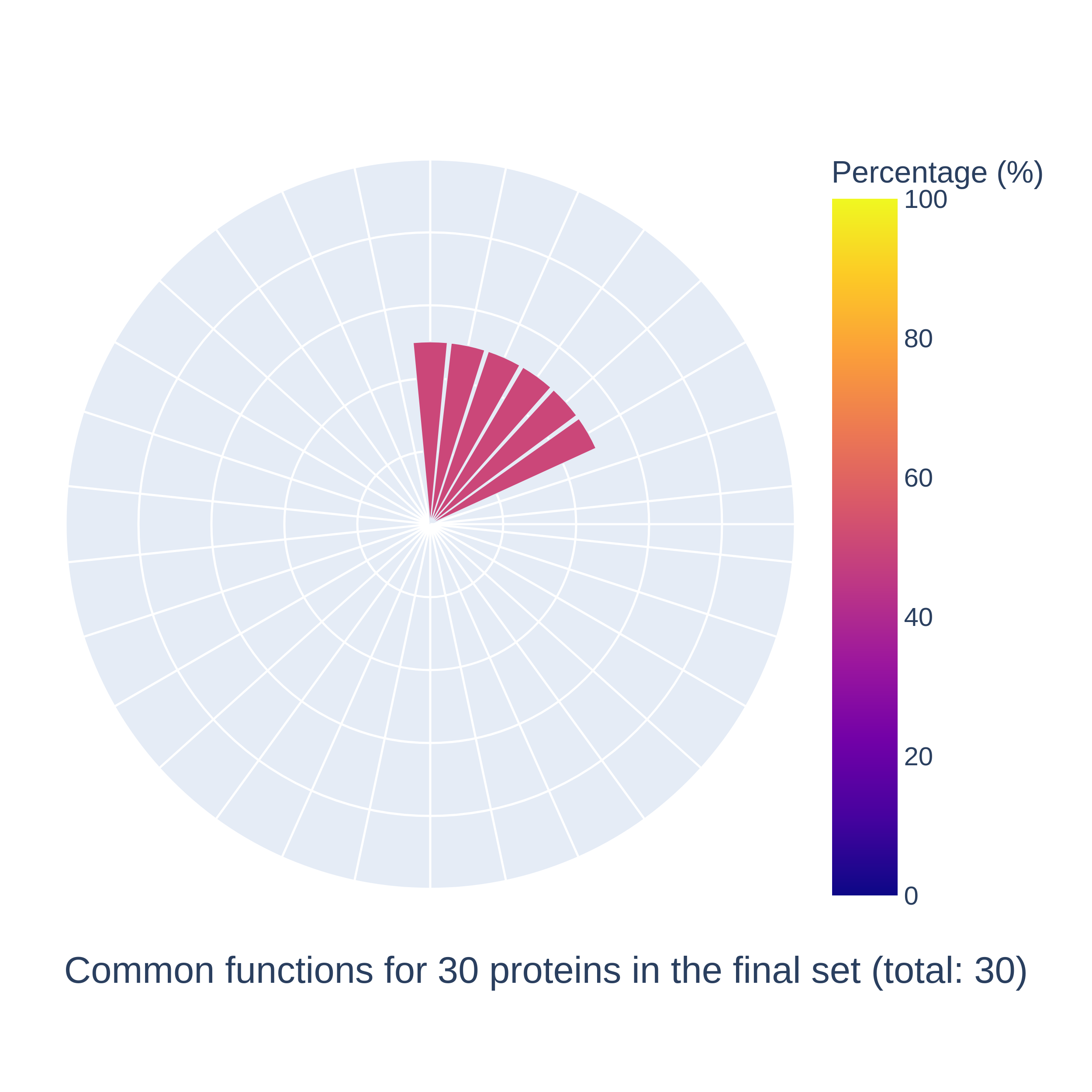

Supplement: Supplementary file 7 — Supplementary Data 4 [file 42003_2023_5076_MOESM7_ESM.zip › 6VXX_A_domain/plots/6VXX_A_BetaCoV-S1-CTD_molecularFunctionSim.png]

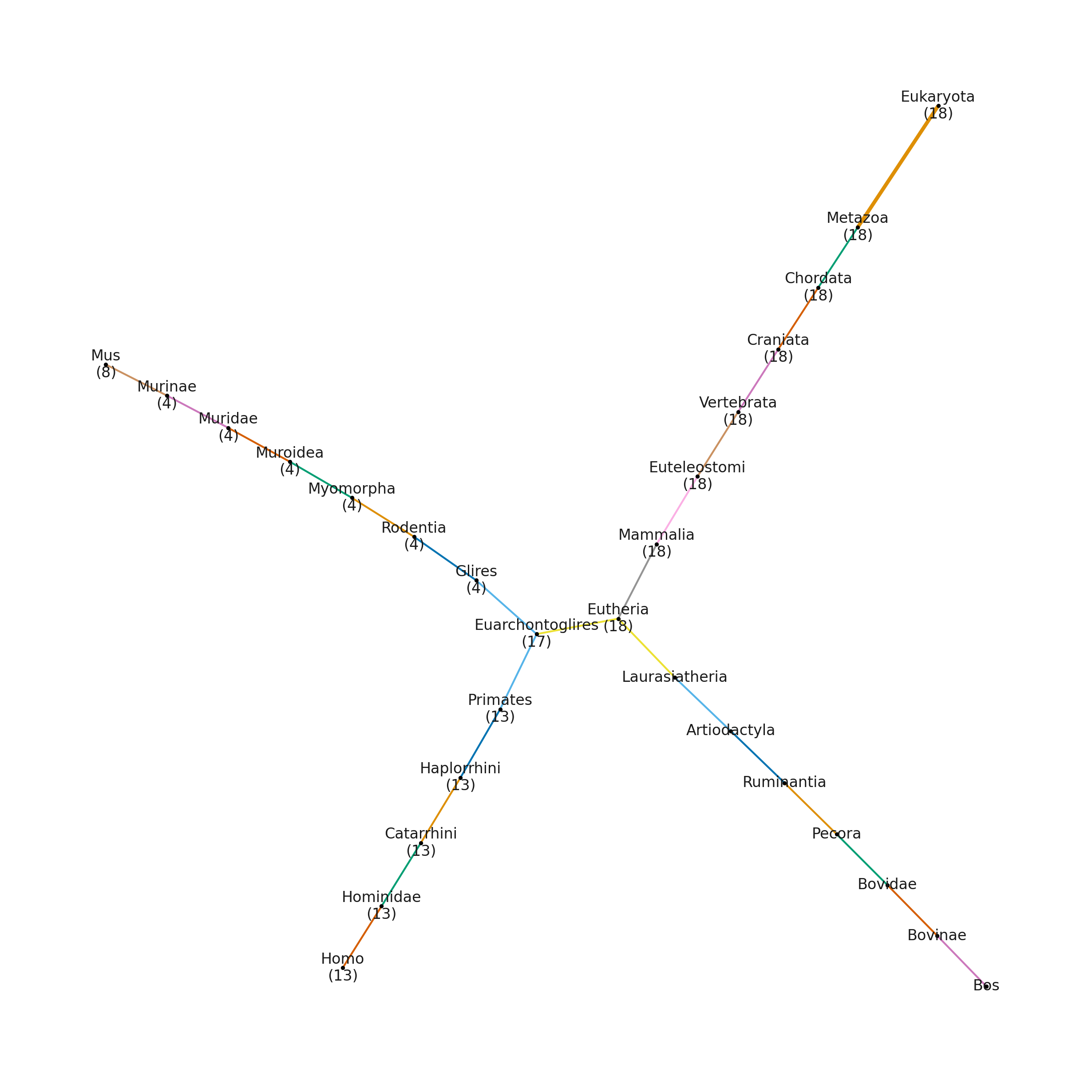

Supplement: Supplementary file 7 — Supplementary Data 4 [file 42003_2023_5076_MOESM7_ESM.zip › 6VXX_A_domain/plots/6VXX_A_BetaCoV-S1-NTD-Eukaryota-tree.png]

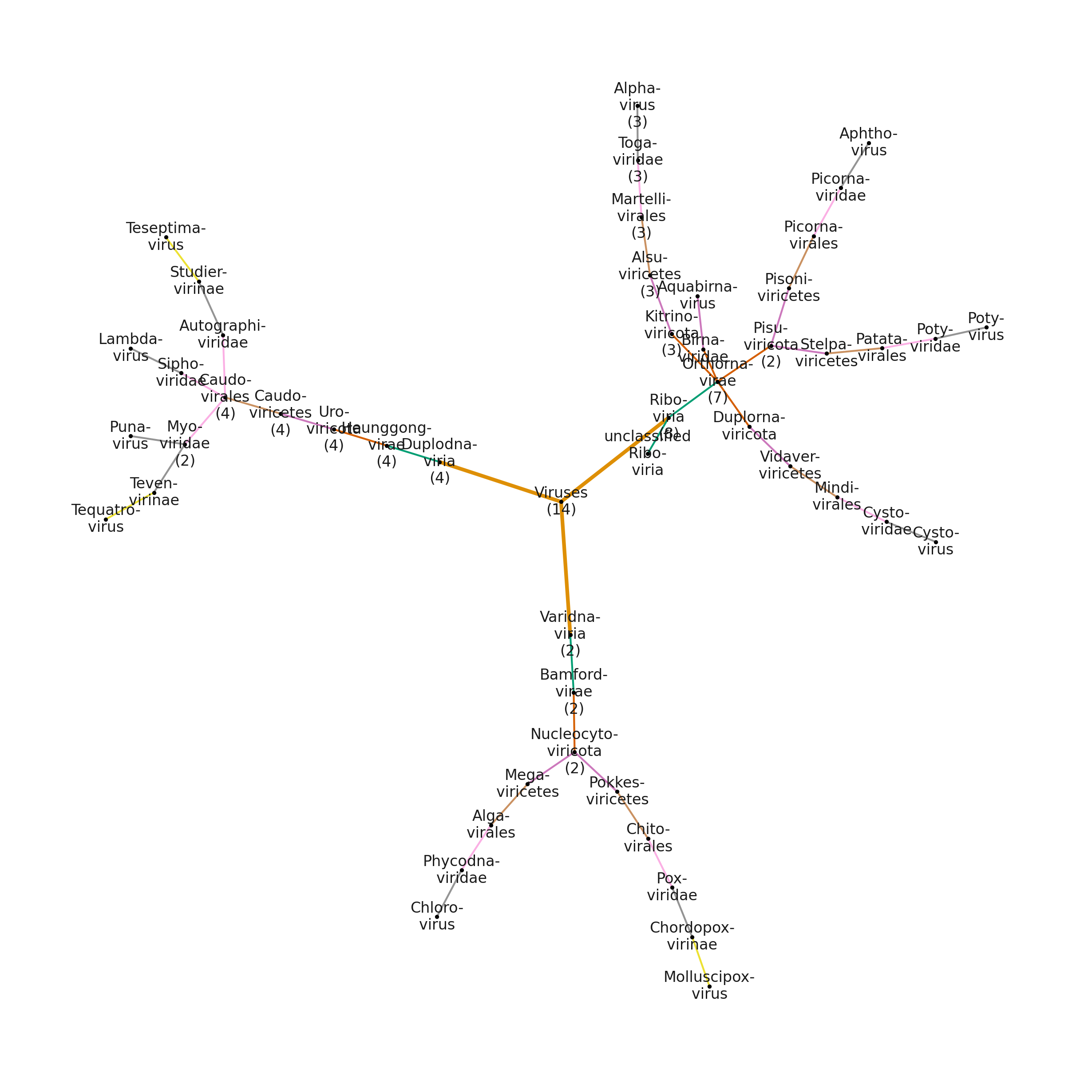

Supplement: Supplementary file 7 — Supplementary Data 4 [file 42003_2023_5076_MOESM7_ESM.zip › 6VXX_A_domain/plots/6VXX_A_BetaCoV-S1-NTD-Viruses-tree.png]

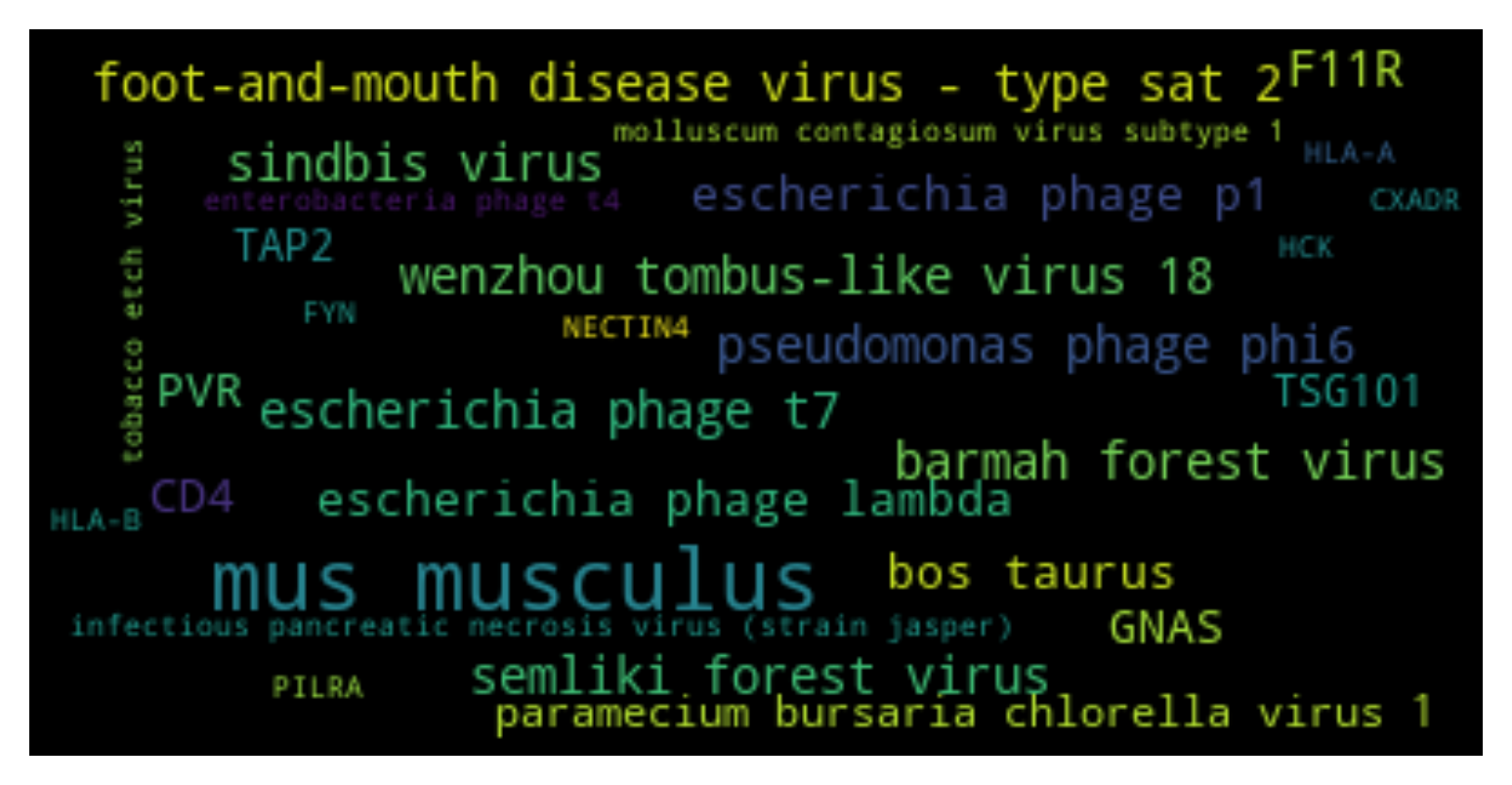

Supplement: Supplementary file 7 — Supplementary Data 4 [file 42003_2023_5076_MOESM7_ESM.zip › 6VXX_A_domain/plots/6VXX_A_BetaCoV-S1-NTD-wordcloud.png]

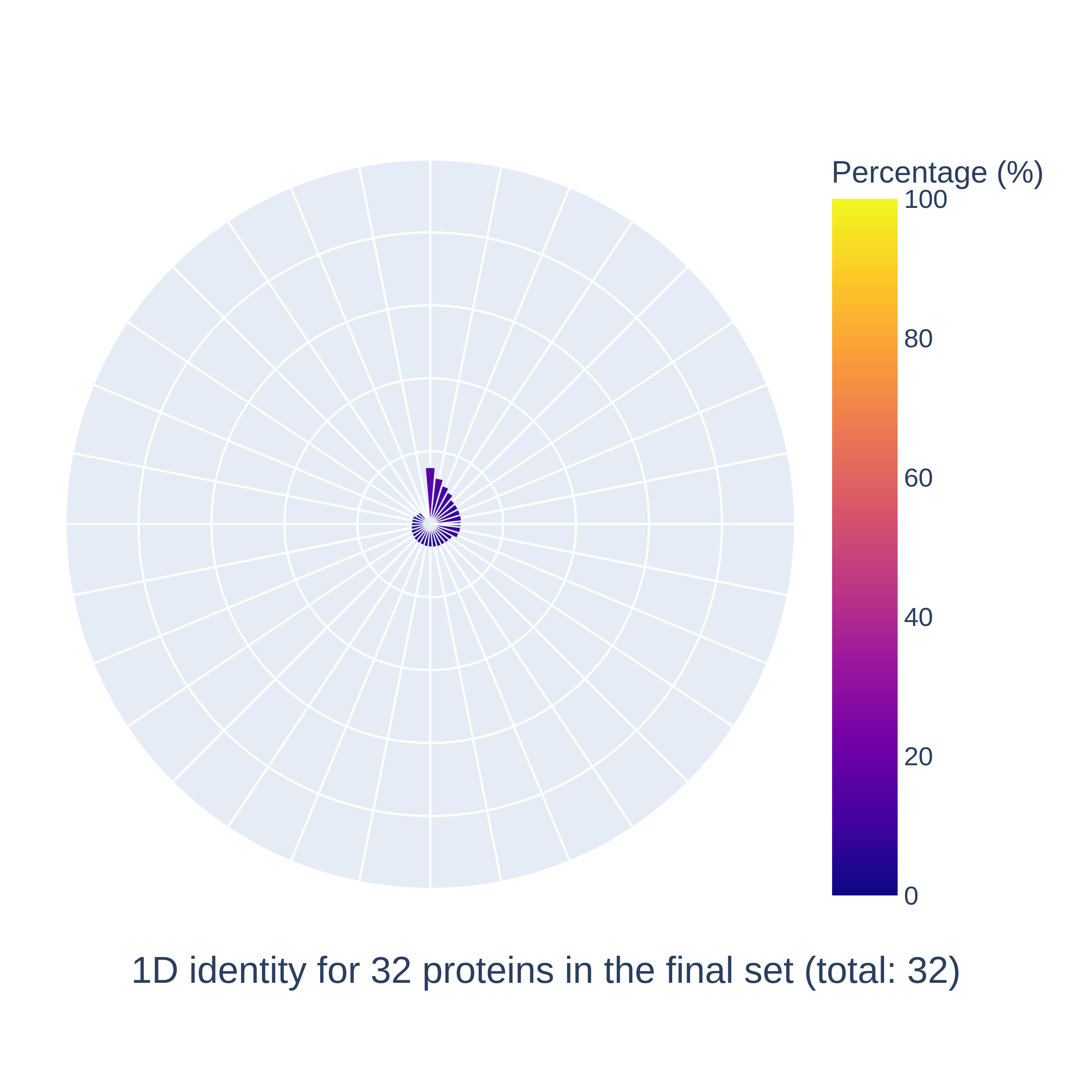

Supplement: Supplementary file 7 — Supplementary Data 4 [file 42003_2023_5076_MOESM7_ESM.zip › 6VXX_A_domain/plots/6VXX_A_BetaCoV-S1-NTD_1D-identity.png]

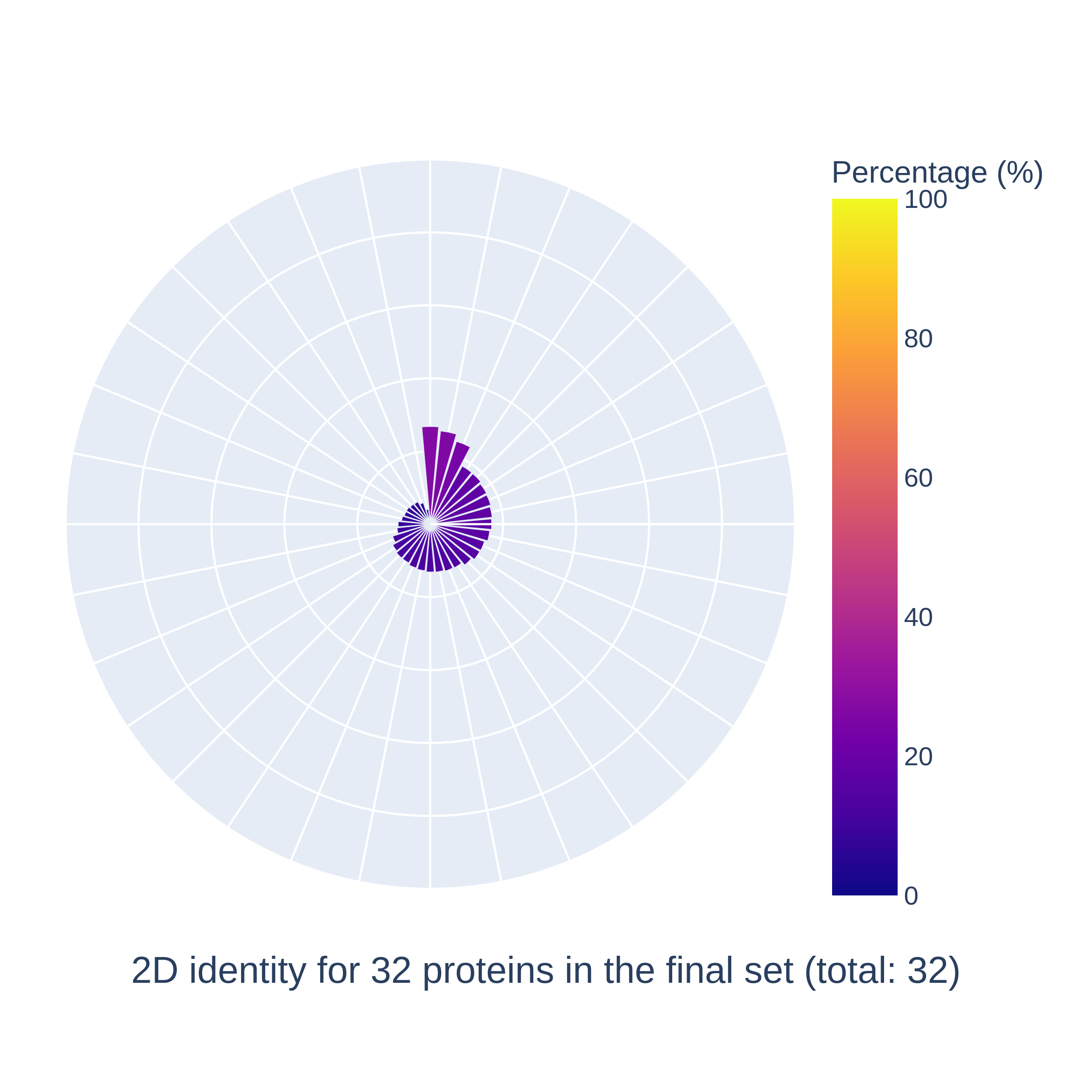

Supplement: Supplementary file 7 — Supplementary Data 4 [file 42003_2023_5076_MOESM7_ESM.zip › 6VXX_A_domain/plots/6VXX_A_BetaCoV-S1-NTD_2D-identity.png]

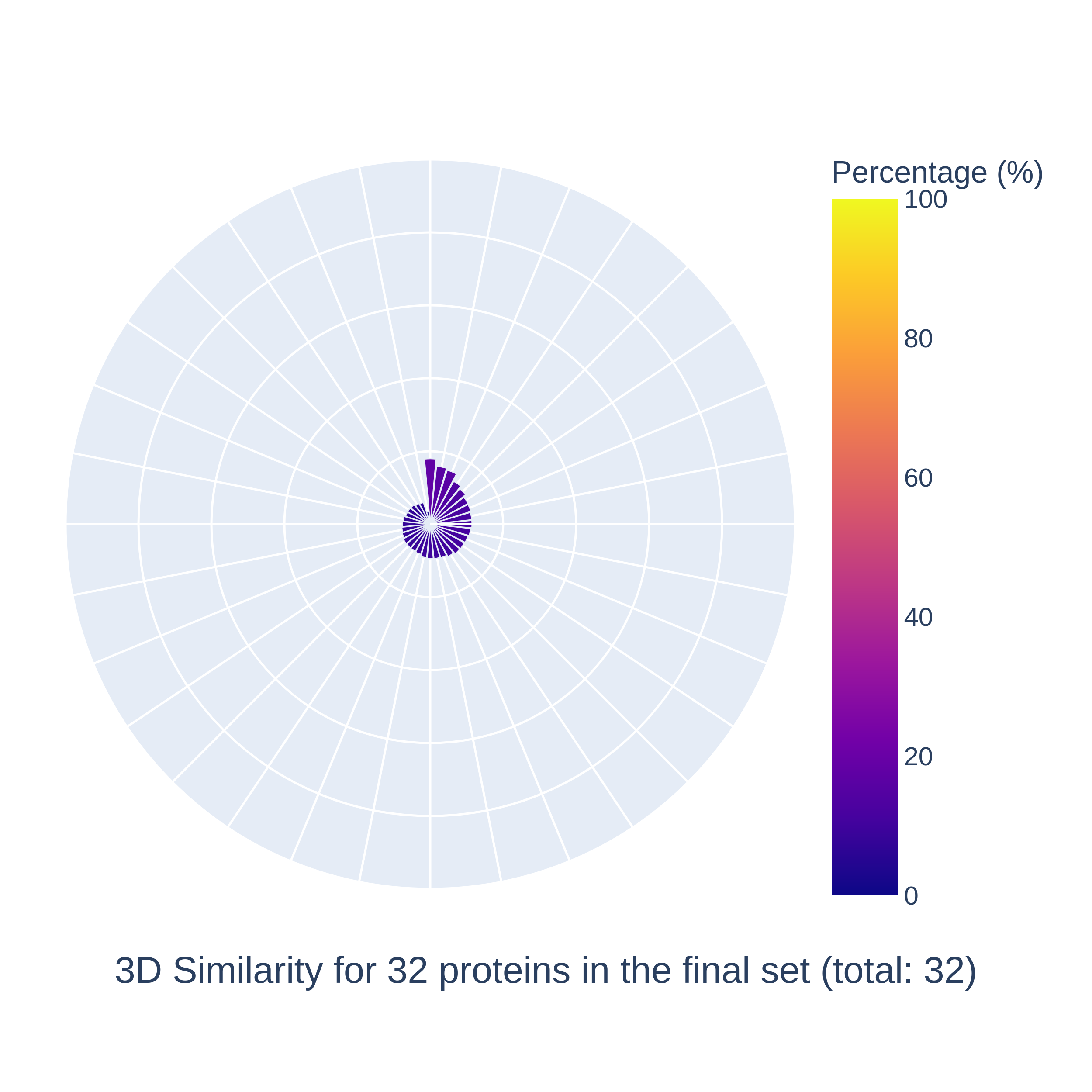

Supplement: Supplementary file 7 — Supplementary Data 4 [file 42003_2023_5076_MOESM7_ESM.zip › 6VXX_A_domain/plots/6VXX_A_BetaCoV-S1-NTD_3D-score.png]

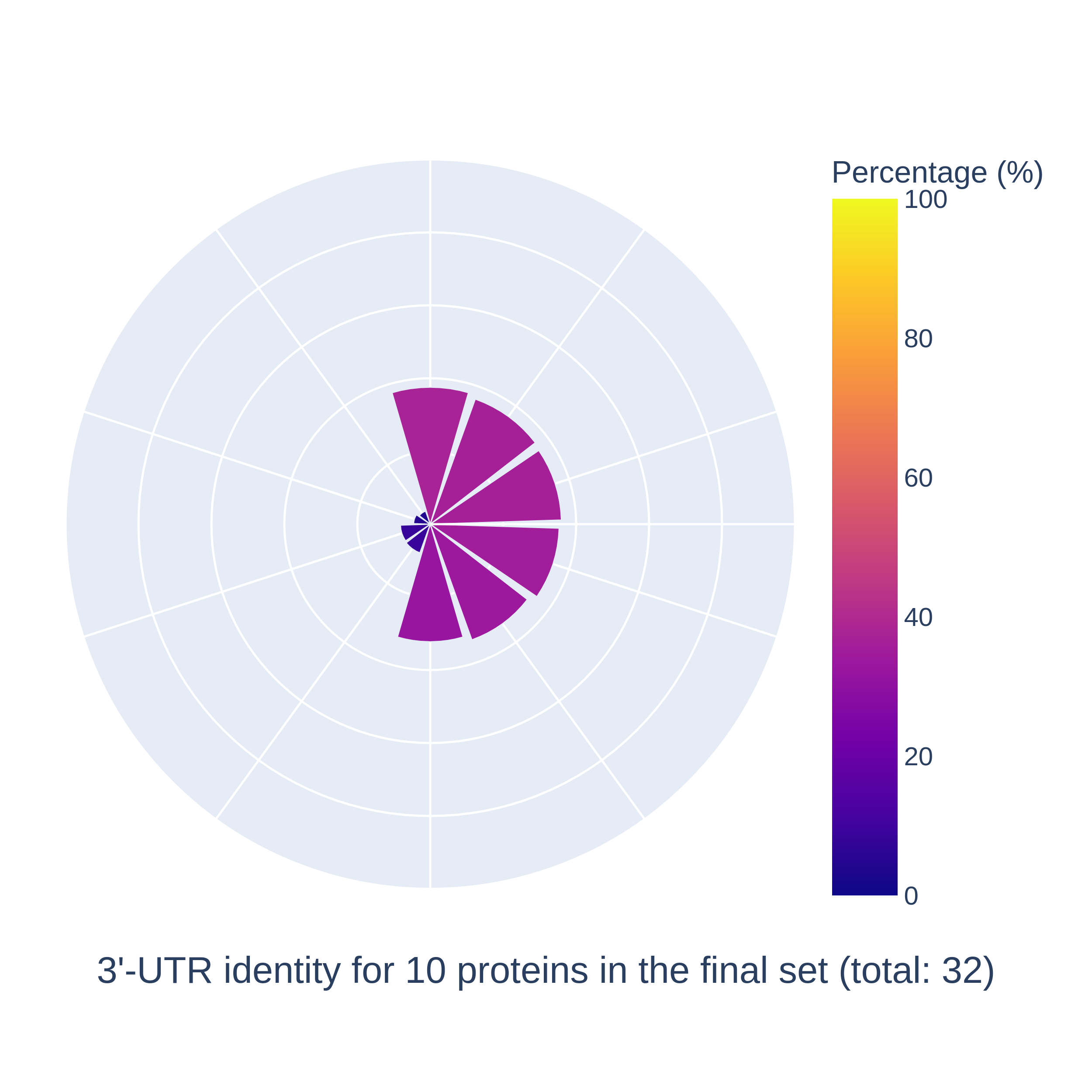

Supplement: Supplementary file 7 — Supplementary Data 4 [file 42003_2023_5076_MOESM7_ESM.zip › 6VXX_A_domain/plots/6VXX_A_BetaCoV-S1-NTD_3UTR-identity.png]

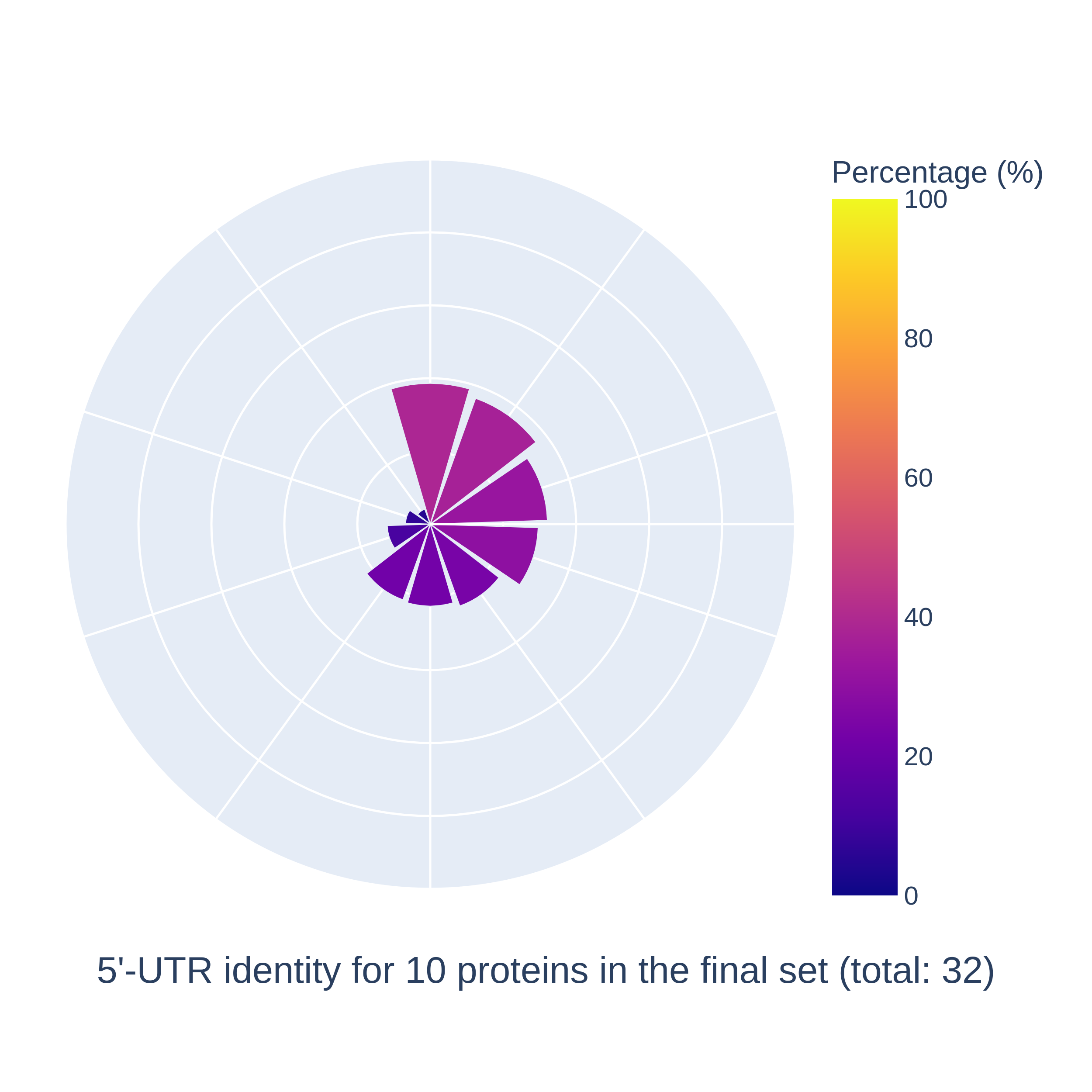

Supplement: Supplementary file 7 — Supplementary Data 4 [file 42003_2023_5076_MOESM7_ESM.zip › 6VXX_A_domain/plots/6VXX_A_BetaCoV-S1-NTD_5UTR-identity.png]
