# Supplementary material for: Identifying and profiling structural similarities between Spike of SARS-CoV-2 and other viral or host proteins with Machaon
Source: Commun Biol. 2023 Jul 19;6:752. doi: 10.1038/s42003-023-05076-7 (PMC10356814; doi:10.1038/s42003-023-05076-7)
Supplement: Supplementary file 7 — Supplementary Data 4 [file 42003_2023_5076_MOESM7_ESM.zip › 6VXX_A_domain/candidates/6VXX_A_BetaCoV-S1-CTD-merged-enriched_eval_report.html]

 

# Structural Comparison Report for 6VXX\_A\_BetaCoV-S1-CTD - domains (total: 30)

---

1

- **Protein name:** Fiber
- **Organism:** Human adenovirus D37
- **Uniprot Accession Number:** Q64823
- **Protein sequence length:** 365 aa
- **1D identity (%):** 5.81
- **1D identity (%) [Gaps excluded]:** 24.68
- **1D identity - Alignment Gaps:** 1014
- **Common reported functions (%):** 0.0
- **Common reported locations (%):** 0.0
- **Common reported processes (%):** 10.0

- **PDB ID:** 4K6T
- **Chain:** A
- **Crystallized protein length:** 186 aa
- **Resolution:** 2.0 Å
- **Associated domain:** Adeno-knob
- **b-phipsi:** 0.005451
- **w-rdist:** 0.205589
- **t-alpha:** 0.0
- **Chemical similarity (Tanimoto Index) (%):** 85.91
- **1D identity (%) [PDB]:** 0.0
- **1D identity (%) [Gaps excluded][PDB]:** 0.0
- **1D identity - Alignment Gaps [PDB]:** 1169
- **2D identity (%) [PDB]:** 13.33
- **2D identity (%) [Gaps excluded][PDB]:** 86.54
- **2D identity - Alignment Gaps [PDB]:** 857
- **3D similarity (TM-Score) (%) [PDB]:** 7.62

- **Gene name:** L5
- **RefSeq ID:** N/A
- **Sequence length:** N/A
- **5-UTR|CDS|3-UTR identity (%):** N/A | N/A | N/A
- **5-UTR|CDS|3-UTR identity (%) [Gaps excluded]:** N/A | N/A | N/A
- **5-UTR|CDS|3-UTR identity [Alignment Gaps]:** N/A | N/A | N/A

**Uniprot Description:**  
  
N/A  
  
**Gene Ontology Information:**

Molecular Function

- metal ion binding

Location

- host cell nucleus
- viral capsid

Biological process

- adhesion receptor-mediated virion attachment to host cell
- cell adhesion
- viral entry into host cell

---

2

- **Protein name:** Replication protein E1
- **Organism:** Bovine papillomavirus type 1
- **Uniprot Accession Number:** P03116
- **Protein sequence length:** 605 aa
- **1D identity (%):** 5.32
- **1D identity (%) [Gaps excluded]:** 26.1
- **1D identity - Alignment Gaps:** 1242
- **Common reported functions (%):** 0.0
- **Common reported locations (%):** 0.0
- **Common reported processes (%):** 0.0

- **PDB ID:** 5A9K
- **Chain:** A
- **Crystallized protein length:** 272 aa
- **Resolution:** 19.0 Å
- **Associated domain:** SF3-helicase
- **b-phipsi:** 0.027337
- **w-rdist:** 0.263196
- **t-alpha:** 0.0
- **Chemical similarity (Tanimoto Index) (%):** 81.98
- **1D identity (%) [PDB]:** 0.0
- **1D identity (%) [Gaps excluded][PDB]:** 0.0
- **1D identity - Alignment Gaps [PDB]:** 1257
- **2D identity (%) [PDB]:** 14.11
- **2D identity (%) [Gaps excluded][PDB]:** 88.44
- **2D identity - Alignment Gaps [PDB]:** 911
- **3D similarity (TM-Score) (%) [PDB]:** 9.91

- **Gene name:** E1
- **RefSeq ID:** NC\_001522
- **Genomic sequence length:** 7945
- **5-UTR|CDS|3-UTR identity (%):** N/A | 30.82 | N/A
- **5-UTR|CDS|3-UTR identity (%) [Gaps excluded]:** N/A | 79.34 | N/A
- **5-UTR|CDS|3-UTR identity [Alignment Gaps]:** N/A | 2484 | N/A

**Uniprot Description:**  
  
ATP-dependent DNA helicase required for initiation of viral DNA replication. It forms a complex with the viral E2 protein. The E1-E2 complex binds to the replication origin which contains binding sites for both proteins. During the initial step, a dimer of E1 interacts with a dimer of protein E2 leading to a complex that binds the viral origin of replication with high specificity. Then, a second dimer of E1 displaces the E2 dimer in an ATP-dependent manner to form the E1 tetramer. Following this, two E1 monomers are added to each half of the site, which results in the formation of two E1 trimers on the viral ori. Subsequently, two hexamers will be created. The double hexamer acts as a bi-directional helicase machinery and unwinds the viral DNA and then recruits the host DNA polymerase to start replication.  
  
Can form hexamers. Interacts with E2 protein; this interaction increases E1 DNA binding specificity. Interacts with host DNA polymerase subunit POLA2. Interacts with host single stranded DNA-binding protein RPA1. Interacts with host TOP1; this interaction stimulates the enzymatic activity of TOP1.  
  
**Gene Ontology Information:**

Molecular Function

- ATP binding
- DNA binding
- DNA helicase activity
- hydrolase activity, acting on acid anhydrides

Location

- host cell nucleus

Biological process

- DNA replication

---

3

- **Protein name:** Tumor susceptibility gene 101 protein
- **Organism:** Homo sapiens
- **Uniprot Accession Number:** Q99816
- **Protein sequence length:** 390 aa
- **1D identity (%):** 5.51
- **1D identity (%) [Gaps excluded]:** 26.76
- **1D identity - Alignment Gaps:** 1095
- **Common reported functions (%):** 0.0
- **Common reported locations (%):** 0.0
- **Common reported processes (%):** 0.0

- **PDB ID:** 3P9H
- **Chain:** A
- **Crystallized protein length:** 141 aa
- **Resolution:** 1.8 Å
- **Associated domain:** UEV
- **b-phipsi:** 0.008926
- **w-rdist:** 0.353696
- **t-alpha:** 0.001808
- **Chemical similarity (Tanimoto Index) (%):** 83.73
- **1D identity (%) [PDB]:** 0.09
- **1D identity (%) [Gaps excluded][PDB]:** 100.0
- **1D identity - Alignment Gaps [PDB]:** 1123
- **2D identity (%) [PDB]:** 8.07
- **2D identity (%) [Gaps excluded][PDB]:** 86.46
- **2D identity - Alignment Gaps [PDB]:** 933
- **3D similarity (TM-Score) (%) [PDB]:** 7.56

- **Gene name:** TSG101
- **RefSeq ID:** NM\_006292
- **Transcript sequence length:** 1534
- **5-UTR|CDS|3-UTR identity (%):** 22.48 | 21.13 | 32.23
- **5-UTR|CDS|3-UTR identity (%) [Gaps excluded]:** 72.04 | 80.54 | 81.06
- **5-UTR|CDS|3-UTR identity [Alignment Gaps]:** 205 | 2919 | 200

**Uniprot Description:**  
  
Component of the ESCRT-I complex, a regulator of vesicular trafficking process. Binds to ubiquitinated cargo proteins and is required for the sorting of endocytic ubiquitinated cargos into multivesicular bodies (MVBs). Mediates the association between the ESCRT-0 and ESCRT-I complex. Required for completion of cytokinesis; the function requires CEP55. May be involved in cell growth and differentiation. Acts as a negative growth regulator. Involved in the budding of many viruses through an interaction with viral proteins that contain a late-budding motif P-[ST]-A-P. This interaction is essential for viral particle budding of numerous retroviruses. Required for the exosomal release of SDCBP, CD63 and syndecan (PubMed:22660413). It may also play a role in the extracellular release of microvesicles that differ from the exosomes (PubMed:22315426).  
  
Component of the ESCRT-I complex (endosomal sorting complex required for transport I) which consists of TSG101, VPS28, a VPS37 protein (VPS37A to -D) and MVB12A or MVB12B in a 1:1:1:1 stoichiometry (PubMed:18005716). Interacts with VPS37A, VPS37B and VPS37C (PubMed:15218037, PubMed:15509564). Interacts with DMAP1 (PubMed:10888872). Interacts with ubiquitin (PubMed:11595185). Interacts with stathmin, GMCL and AATF (By similarity). Component of an ESCRT-I complex (endosomal sorting complex required for transport I) which consists of TSG101, VPS28, VPS37A and UBAP1 in a 1:1:1:1 stoichiometry (PubMed:21757351). Interacts with HGS; the interaction mediates the association with the ESCRT-0 complex. Interacts with GGA1 and GGA3 (PubMed:15143060, PubMed:15039775). Interacts (via UEV domain) with PDCD6IP/AIP1 (PubMed:14505570, PubMed:14519844). Interacts with VPS28, SNF8 and VPS36 (PubMed:14505570). Self-associates (PubMed:14505570, PubMed:14519844). Interacts with MVB12A; the association appears to be mediated by the TSG101-VPS37 binary subcomplex. Interacts with VPS37D. Interacts with LRSAM1. Interacts with CEP55; the interaction is required for cytokinesis but not for viral budding (PubMed:17853893). Interacts with PDCD6 (PubMed:18256029). Interacts with LITAF (PubMed:23166352). Interacts with MGRN1 (PubMed:17229889). Interacts with ARRDC1; recruits TSG101 to the plasma membrane (PubMed:21191027, PubMed:22315426).  
  
**Gene Ontology Information:**

Molecular Function

- calcium-dependent protein binding
- DNA binding
- nuclear receptor coactivator activity
- protein homodimerization activity
- protein-containing complex binding
- transcription corepressor activity
- ubiquitin binding
- ubiquitin protein ligase binding
- virion binding

Location

- cytoplasm
- cytosol
- early endosome
- early endosome membrane
- endosome
- endosome membrane
- ESCRT I complex
- extracellular exosome
- Flemming body
- host cell
- late endosome
- late endosome membrane
- microtubule organizing center
- multivesicular body
- nucleolus
- plasma membrane

Biological process

- autophagosome maturation
- cell cycle arrest
- cell division
- endosomal transport
- endosome to lysosome transport
- exosomal secretion
- extracellular transport
- intracellular transport of virus
- keratinocyte differentiation
- macroautophagy
- multivesicular body assembly
- negative regulation of cell population proliferation
- negative regulation of epidermal growth factor receptor signaling pathway
- negative regulation of epidermal growth factor-activated receptor activity
- negative regulation of transcription by RNA polymerase II
- positive regulation of exosomal secretion
- positive regulation of ubiquitin-dependent endocytosis
- positive regulation of viral budding via host ESCRT complex
- positive regulation of viral release from host cell
- protein monoubiquitination
- protein transport
- regulation of cell growth
- regulation of extracellular exosome assembly
- regulation of MAP kinase activity
- ubiquitin-dependent protein catabolic process via the multivesicular body sorting pathway
- viral budding
- viral budding via host ESCRT complex
- viral life cycle

---

4

- **Protein name:** Arginine/Ornithine decarboxylase
- **Organism:** Paramecium bursaria Chlorella virus 1
- **Uniprot Accession Number:** Q84527
- **Protein sequence length:** 372 aa
- **1D identity (%):** 6.34
- **1D identity (%) [Gaps excluded]:** 29.76
- **1D identity - Alignment Gaps:** 1067
- **Common reported functions (%):** 0.0
- **Common reported locations (%):** 0.0
- **Common reported processes (%):** 0.0

- **PDB ID:** 2NV9
- **Chain:** C
- **Crystallized protein length:** 367 aa
- **Resolution:** 1.95 Å
- **Associated domain:** Orn-DAP-Arg-deC
- **b-phipsi:** 0.012463
- **w-rdist:** 0.693528
- **t-alpha:** 0.0
- **Chemical similarity (Tanimoto Index) (%):** 84.2
- **1D identity (%) [PDB]:** 0.75
- **1D identity (%) [Gaps excluded][PDB]:** 83.33
- **1D identity - Alignment Gaps [PDB]:** 1328
- **2D identity (%) [PDB]:** 16.92
- **2D identity (%) [Gaps excluded][PDB]:** 88.48
- **2D identity - Alignment Gaps [PDB]:** 918
- **3D similarity (TM-Score) (%) [PDB]:** 12.65

- **Gene name:** A207R
- **RefSeq ID:** NC\_000852
- **Genomic sequence length:** 330611
- **5-UTR|CDS|3-UTR identity (%):** N/A | 18.61 | N/A
- **5-UTR|CDS|3-UTR identity (%) [Gaps excluded]:** N/A | 77.78 | N/A
- **5-UTR|CDS|3-UTR identity [Alignment Gaps]:** N/A | 3033 | N/A

**Uniprot Description:**  
  
N/A  
  
**Gene Ontology Information:**

Molecular Function

- catalytic activity

Location  
  
N/A

Biological process

- polyamine biosynthetic process

---

5

- **Protein name:** NTF2-related export protein 1
- **Organism:** Homo sapiens
- **Uniprot Accession Number:** Q9UKK6
- **Protein sequence length:** 140 aa
- **1D identity (%):** 3.04
- **1D identity (%) [Gaps excluded]:** 30.47
- **1D identity - Alignment Gaps:** 1157
- **Common reported functions (%):** 0.0
- **Common reported locations (%):** 0.0
- **Common reported processes (%):** 0.0

- **PDB ID:** 6E5U
- **Chain:** H
- **Crystallized protein length:** 138 aa
- **Resolution:** 3.8 Å
- **Associated domain:** NTF2
- **b-phipsi:** 0.00079
- **w-rdist:** 0.447514
- **t-alpha:** 0.025316
- **Chemical similarity (Tanimoto Index) (%):** N/A
- **1D identity (%) [PDB]:** 0.09
- **1D identity (%) [Gaps excluded][PDB]:** 100.0
- **1D identity - Alignment Gaps [PDB]:** 1119
- **2D identity (%) [PDB]:** 9.11
- **2D identity (%) [Gaps excluded][PDB]:** 93.0
- **2D identity - Alignment Gaps [PDB]:** 921
- **3D similarity (TM-Score) (%) [PDB]:** 12.28

- **Gene name:** NXT1
- **RefSeq ID:** N/A
- **Sequence length:** N/A
- **5-UTR|CDS|3-UTR identity (%):** N/A | N/A | N/A
- **5-UTR|CDS|3-UTR identity (%) [Gaps excluded]:** N/A | N/A | N/A
- **5-UTR|CDS|3-UTR identity [Alignment Gaps]:** N/A | N/A | N/A

**Uniprot Description:**  
  
Stimulator of protein export for NES-containing proteins (PubMed:10567585). Also plays a role in the nuclear export of U1 snRNA, tRNA, and mRNA (PubMed:10848583). The NXF1-NXT1 heterodimer is involved in the export of HSP70 mRNA in conjunction with ALYREF/THOC4 and THOC5 (PubMed:19165146, PubMed:11259602).  
  
Heterodimer with NXF1 (PubMed:11583626). Forms a complex with RANGAP1, RANBP2/NUP358 and NXF1 (PubMed:14729961). Interacts (via NTF2 domain) with NXF1 (PubMed:11583626). Stabilizes the NTF2 domain of NXF1 by heterodimerization (PubMed:11583626). The formation of NXF1-NXT1 heterodimers is required for the NXF1-mediated nuclear mRNA export (PubMed:11583626). Preferentially binds Ran-GTP (PubMed:10567585). Associates with NXF2, NXF3 and NXF5. Does not bind nucleoporins (NPC) directly, its association to NPC is mediated by NXF1 (PubMed:11583626).  
  
**Gene Ontology Information:**

Molecular Function

- small GTPase binding

Location

- cytoplasm
- cytosol
- nuclear pore
- nuclear pore central transport channel
- nuclear speck
- nucleoplasm

Biological process

- mRNA export from nucleus
- nucleocytoplasmic transport
- protein import into nucleus

---

6

- **Protein name:** Spike glycoprotein
- **Organism:** Bat coronavirus HKU9
- **Uniprot Accession Number:** A3EXG6
- **Protein sequence length:** 1274 aa
- **1D identity (%):** 31.4
- **1D identity (%) [Gaps excluded]:** 37.07
- **1D identity - Alignment Gaps:** 211
- **Common reported functions (%):** 0.0
- **Common reported locations (%):** 62.5
- **Common reported processes (%):** 50.0

- **PDB ID:** 5GYQ
- **Chain:** A
- **Crystallized protein length:** 169 aa
- **Resolution:** 2.1 Å
- **Associated domain:** BetaCoV-S1-NTD
- **b-phipsi:** 0.013456
- **w-rdist:** 0.085748
- **t-alpha:** 0.045208
- **Chemical similarity (Tanimoto Index) (%):** 95.43
- **1D identity (%) [PDB]:** 0.09
- **1D identity (%) [Gaps excluded][PDB]:** 100.0
- **1D identity - Alignment Gaps [PDB]:** 1150
- **2D identity (%) [PDB]:** 11.34
- **2D identity (%) [Gaps excluded][PDB]:** 89.92
- **2D identity - Alignment Gaps [PDB]:** 894
- **3D similarity (TM-Score) (%) [PDB]:** 14.89

- **Gene name:** S
- **RefSeq ID:** NC\_009021
- **Genomic sequence length:** 29114
- **5-UTR|CDS|3-UTR identity (%):** 47.28 | 48.33 | 47.08
- **5-UTR|CDS|3-UTR identity (%) [Gaps excluded]:** 82.22 | 76.52 | 80.59
- **5-UTR|CDS|3-UTR identity [Alignment Gaps]:** 133 | 1727 | 121

**Uniprot Description:**  
  
Spike protein S1
attaches the virion to the cell membrane by interacting with host receptor, initiating the infection.  
  
Homotrimer; each monomer consists of a S1 and a S2 subunit. The resulting peplomers protrude from the virus surface as spikes.  
  
**Gene Ontology Information:**

Molecular Function  
  
N/A

Location

- host cell endoplasmic reticulum-Golgi intermediate compartment membrane
- host cell plasma membrane
- integral component of membrane
- viral envelope
- virion membrane

Biological process

- endocytosis involved in viral entry into host cell
- fusion of virus membrane with host endosome membrane
- fusion of virus membrane with host plasma membrane
- pathogenesis
- receptor-mediated virion attachment to host cell

---

7

- **Protein name:** Neuropilin-1
- **Organism:** Homo sapiens
- **Uniprot Accession Number:** O14786
- **Protein sequence length:** 923 aa
- **1D identity (%):** 15.68
- **1D identity (%) [Gaps excluded]:** 27.74
- **1D identity - Alignment Gaps:** 610
- **Common reported functions (%):** 0.0
- **Common reported locations (%):** 12.5
- **Common reported processes (%):** 0.0

- **PDB ID:** 7JJC
- **Chain:** D
- **Crystallized protein length:** 166 aa
- **Resolution:** 2.36 Å
- **Associated domain:** F5-8-type-C-1
- **b-phipsi:** 0.029795
- **w-rdist:** 0.509125
- **t-alpha:** 0.001808
- **Chemical similarity (Tanimoto Index) (%):** 85.7
- **1D identity (%) [PDB]:** 0.0
- **1D identity (%) [Gaps excluded][PDB]:** 0.0
- **1D identity - Alignment Gaps [PDB]:** 1138
- **2D identity (%) [PDB]:** 9.96
- **2D identity (%) [Gaps excluded][PDB]:** 89.47
- **2D identity - Alignment Gaps [PDB]:** 910
- **3D similarity (TM-Score) (%) [PDB]:** 8.07

- **Gene name:** NRP1
- **RefSeq ID:** NM\_003873
- **Transcript sequence length:** 5640
- **5-UTR|CDS|3-UTR identity (%):** 34.83 | 40.01 | 6.19
- **5-UTR|CDS|3-UTR identity (%) [Gaps excluded]:** 77.19 | 78.15 | 75.94
- **5-UTR|CDS|3-UTR identity [Alignment Gaps]:** 208 | 2128 | 2388

**Uniprot Description:**  
  
Cell-surface receptor involved in the development of the cardiovascular system, in angiogenesis, in the formation of certain neuronal circuits and in organogenesis outside the nervous system. Mediates the chemorepulsant activity of semaphorins (PubMed:9288753, PubMed:9529250, PubMed:10688880). Recognizes a C-end rule (CendR) motif R/KXXR/K on its ligands which causes cellular internalization and vascular leakage (PubMed:19805273). It binds to semaphorin 3A, the PLGF-2 isoform of PGF, the VEGF165 isoform of VEGFA and VEGFB (PubMed:9288753, PubMed:9529250, PubMed:10688880, PubMed:19805273). Coexpression with KDR results in increased VEGF165 binding to KDR as well as increased chemotaxis. Regulates VEGF-induced angiogenesis. Binding to VEGFA initiates a signaling pathway needed for motor neuron axon guidance and cell body migration, including for the caudal migration of facial motor neurons from rhombomere 4 to rhombomere 6 during embryonic development (By similarity). Regulates mitochondrial iron transport via interaction with ABCB8/MITOSUR (PubMed:30623799).  
  
Homodimer, and heterodimer with NRP2 (PubMed:17989695). Interacts with FER (By similarity). Interacts with PLXNB1 (PubMed:10520995). Interacts with VEGFA (PubMed:26503042, PubMed:19805273). Interacts with ABCB8/MITOSUR in mitochondria (PubMed:30623799).  
  
**Gene Ontology Information:**

Molecular Function

- coreceptor activity
- cytokine binding
- growth factor binding
- GTPase activator activity
- heparin binding
- metal ion binding
- protein kinase binding
- semaphorin receptor activity
- vascular endothelial growth factor binding
- vascular endothelial growth factor-activated receptor activity

Location

- axon
- cell surface
- cytoplasmic vesicle
- cytosol
- early endosome
- extracellular space
- focal adhesion
- glutamatergic synapse
- growth cone
- integral component of membrane
- mitochondrial membrane
- neurofilament
- neuron projection
- neuronal cell body
- plasma membrane
- receptor complex
- semaphorin receptor complex
- sorting endosome

Biological process

- actin cytoskeleton reorganization
- angiogenesis
- angiogenesis involved in coronary vascular morphogenesis
- animal organ morphogenesis
- artery morphogenesis
- axon extension involved in axon guidance
- axon guidance
- axonal fasciculation
- axonogenesis involved in innervation
- basal dendrite arborization
- basal dendrite development
- branching involved in blood vessel morphogenesis
- branchiomotor neuron axon guidance
- cell migration involved in sprouting angiogenesis
- cell-cell signaling
- cellular response to hepatocyte growth factor stimulus
- cellular response to vascular endothelial growth factor stimulus
- commissural neuron axon guidance
- coronary artery morphogenesis
- dichotomous subdivision of terminal units involved in salivary gland branching
- dorsal root ganglion morphogenesis
- endothelial cell chemotaxis
- endothelial cell migration
- endothelial tip cell fate specification
- facial nerve structural organization
- facioacoustic ganglion development
- gonadotrophin-releasing hormone neuronal migration to the hypothalamus
- hepatocyte growth factor receptor signaling pathway
- integrin-mediated signaling pathway
- motor neuron migration
- negative regulation of axon extension involved in axon guidance
- negative regulation of extrinsic apoptotic signaling pathway
- negative regulation of neuron apoptotic process
- nerve development
- neural crest cell migration involved in autonomic nervous system development
- neuron migration
- neuropilin signaling pathway
- otic placode development
- outflow tract septum morphogenesis
- platelet-derived growth factor receptor signaling pathway
- positive chemotaxis
- positive regulation of actin cytoskeleton reorganization
- positive regulation of axon extension involved in axon guidance
- positive regulation of cell migration involved in sprouting angiogenesis
- positive regulation of cytokine activity
- positive regulation of endothelial cell migration
- positive regulation of endothelial cell proliferation
- positive regulation of ERK1 and ERK2 cascade
- positive regulation of filopodium assembly
- positive regulation of focal adhesion assembly
- positive regulation of peptidyl-tyrosine phosphorylation
- positive regulation of phosphorylation
- positive regulation of retinal ganglion cell axon guidance
- positive regulation of smooth muscle cell migration
- positive regulation of stress fiber assembly
- positive regulation of substrate adhesion-dependent cell spreading
- postsynapse organization
- protein localization to early endosome
- regulation of Cdc42 protein signal transduction
- regulation of retinal ganglion cell axon guidance
- regulation of vesicle-mediated transport
- renal artery morphogenesis
- response to wounding
- retina vasculature morphogenesis in camera-type eye
- retinal ganglion cell axon guidance
- semaphorin-plexin signaling pathway
- semaphorin-plexin signaling pathway involved in axon guidance
- semaphorin-plexin signaling pathway involved in neuron projection guidance
- sensory neuron axon guidance
- signal transduction
- sprouting angiogenesis
- substrate adhesion-dependent cell spreading
- substrate-dependent cell migration, cell extension
- sympathetic ganglion development
- sympathetic neuron projection extension
- sympathetic neuron projection guidance
- toxin transport
- trigeminal ganglion development
- trigeminal nerve structural organization
- vascular endothelial growth factor receptor signaling pathway
- VEGF-activated neuropilin signaling pathway
- VEGF-activated neuropilin signaling pathway involved in axon guidance
- ventral trunk neural crest cell migration
- vestibulocochlear nerve structural organization

---

8

- **Protein name:** Ephrin type-A receptor 2
- **Organism:** Homo sapiens
- **Uniprot Accession Number:** P29317
- **Protein sequence length:** 976 aa
- **1D identity (%):** 11.73
- **1D identity (%) [Gaps excluded]:** 29.1
- **1D identity - Alignment Gaps:** 957
- **Common reported functions (%):** 0.0
- **Common reported locations (%):** 0.0
- **Common reported processes (%):** 0.0

- **PDB ID:** 7CZF
- **Chain:** D
- **Crystallized protein length:** 158 aa
- **Resolution:** 3.2 Å
- **Associated domain:** Eph-LBD
- **b-phipsi:** 0.023931
- **w-rdist:** 0.088714
- **t-alpha:** 0.003617
- **Chemical similarity (Tanimoto Index) (%):** N/A
- **1D identity (%) [PDB]:** 0.09
- **1D identity (%) [Gaps excluded][PDB]:** 50.0
- **1D identity - Alignment Gaps [PDB]:** 1138
- **2D identity (%) [PDB]:** 9.81
- **2D identity (%) [Gaps excluded][PDB]:** 90.18
- **2D identity - Alignment Gaps [PDB]:** 918
- **3D similarity (TM-Score) (%) [PDB]:** 9.51

- **Gene name:** EPHA2
- **RefSeq ID:** NM\_004431
- **Transcript sequence length:** 3946
- **5-UTR|CDS|3-UTR identity (%):** 29.59 | 36.69 | 15.59
- **5-UTR|CDS|3-UTR identity (%) [Gaps excluded]:** 80.56 | 75.58 | 75.26
- **5-UTR|CDS|3-UTR identity [Alignment Gaps]:** 186 | 2339 | 727

**Uniprot Description:**  
  
Receptor tyrosine kinase which binds promiscuously membrane-bound ephrin-A family ligands residing on adjacent cells, leading to contact-dependent bidirectional signaling into neighboring cells. The signaling pathway downstream of the receptor is referred to as forward signaling while the signaling pathway downstream of the ephrin ligand is referred to as reverse signaling. Activated by the ligand ephrin-A1/EFNA1 regulates migration, integrin-mediated adhesion, proliferation and differentiation of cells. Regulates cell adhesion and differentiation through DSG1/desmoglein-1 and inhibition of the ERK1/ERK2 (MAPK3/MAPK1, respectively) signaling pathway. May also participate in UV radiation-induced apoptosis and have a ligand-independent stimulatory effect on chemotactic cell migration. During development, may function in distinctive aspects of pattern formation and subsequently in development of several fetal tissues. Involved for instance in angiogenesis, in early hindbrain development and epithelial proliferation and branching morphogenesis during mammary gland development. Engaged by the ligand ephrin-A5/EFNA5 may regulate lens fiber cells shape and interactions and be important for lens transparency development and maintenance. With ephrin-A2/EFNA2 may play a role in bone remodeling through regulation of osteoclastogenesis and osteoblastogenesis.  
  
Homodimer. Interacts with SLA. Interacts (phosphorylated form) with VAV2, VAV3 and PI3-kinase p85 subunit (PIK3R1, PIK3R2 or PIK3R3); critical for the EFNA1-induced activation of RAC1 which stimulates cell migration (By similarity). Interacts with INPPL1; regulates activated EPHA2 endocytosis and degradation. Interacts (inactivated form) with PTK2/FAK1 and interacts (EFNA1 ligand-activated form) with PTPN11; regulates integrin-mediated adhesion. Interacts with ARHGEF16, DOCK4 and ELMO2; mediates ligand-independent activation of RAC1 which stimulates cell migration. Interacts with CLDN4; phosphorylates CLDN4 and may regulate tight junctions. Interacts with ACP1. Interacts (via SAM domain) with ANKS1A (via SAM domain). Interacts with CEMIP. Interacts with NCK1; may regulate EPHA2 activity in cell migration and adhesion.  
  
**Gene Ontology Information:**

Molecular Function

- ATP binding
- cadherin binding
- growth factor binding
- transmembrane receptor protein tyrosine kinase activity
- transmembrane-ephrin receptor activity
- virus receptor activity

Location

- cell surface
- focal adhesion
- integral component of plasma membrane
- lamellipodium
- lamellipodium membrane
- leading edge membrane
- neuron projection
- plasma membrane
- receptor complex
- ruffle membrane
- tight junction

Biological process

- activation of GTPase activity
- axial mesoderm formation
- axon guidance
- blood vessel endothelial cell proliferation involved in sprouting angiogenesis
- bone remodeling
- branching involved in mammary gland duct morphogenesis
- cAMP metabolic process
- cell adhesion
- cell chemotaxis
- cell migration
- cell motility
- defense response to Gram-positive bacterium
- ephrin receptor signaling pathway
- inflammatory response
- intrinsic apoptotic signaling pathway in response to DNA damage
- keratinocyte differentiation
- lens fiber cell morphogenesis
- mammary gland epithelial cell proliferation
- multicellular organism development
- negative regulation of angiogenesis
- negative regulation of chemokine production
- negative regulation of lymphangiogenesis
- negative regulation of protein kinase B signaling
- neural tube development
- notochord cell development
- notochord formation
- osteoblast differentiation
- osteoclast differentiation
- pericyte cell differentiation
- positive regulation of bicellular tight junction assembly
- positive regulation of kinase activity
- positive regulation of protein localization to plasma membrane
- post-anal tail morphogenesis
- protein kinase B signaling
- protein localization to plasma membrane
- regulation of angiogenesis
- regulation of blood vessel endothelial cell migration
- regulation of cell adhesion mediated by integrin
- regulation of ERK1 and ERK2 cascade
- regulation of lamellipodium assembly
- response to growth factor
- skeletal system development
- transmembrane receptor protein tyrosine kinase signaling pathway
- vasculogenesis

---

9

- **Protein name:** H-2 class I histocompatibility antigen, D-B alpha chain
- **Organism:** Mus musculus
- **Uniprot Accession Number:** P01899
- **Protein sequence length:** 362 aa
- **1D identity (%):** 6.68
- **1D identity (%) [Gaps excluded]:** 26.2
- **1D identity - Alignment Gaps:** 971
- **Common reported functions (%):** 0.0
- **Common reported locations (%):** 0.0
- **Common reported processes (%):** 0.0

- **PDB ID:** 4HUX
- **Chain:** A
- **Crystallized protein length:** 273 aa
- **Resolution:** 2.2 Å
- **Associated domain:** Ig-like-C1-type
- **b-phipsi:** 0.0333
- **w-rdist:** 0.683558
- **t-alpha:** 0.0
- **Chemical similarity (Tanimoto Index) (%):** 85.69
- **1D identity (%) [PDB]:** 0.0
- **1D identity (%) [Gaps excluded][PDB]:** 0.0
- **1D identity - Alignment Gaps [PDB]:** 1257
- **2D identity (%) [PDB]:** 18.33
- **2D identity (%) [Gaps excluded][PDB]:** 88.84
- **2D identity - Alignment Gaps [PDB]:** 827
- **3D similarity (TM-Score) (%) [PDB]:** 9.69

- **Gene name:** H2-D1
- **RefSeq ID:** N/A
- **Sequence length:** N/A
- **5-UTR|CDS|3-UTR identity (%):** N/A | N/A | N/A
- **5-UTR|CDS|3-UTR identity (%) [Gaps excluded]:** N/A | N/A | N/A
- **5-UTR|CDS|3-UTR identity [Alignment Gaps]:** N/A | N/A | N/A

**Uniprot Description:**  
  
Involved in the presentation of foreign antigens to the immune system.  
  
Heterodimer of an alpha chain and a beta chain (beta-2-microglobulin). Interacts with murid herpesvirus 4 protein K3 (mK3).  
  
**Gene Ontology Information:**

Molecular Function

- beta-2-microglobulin binding
- CD8 receptor binding
- peptide antigen binding
- peptide binding
- protein-containing complex binding
- signaling receptor binding
- T cell receptor binding
- TAP binding
- TAP complex binding

Location

- cell surface
- endoplasmic reticulum
- endoplasmic reticulum exit site
- external side of plasma membrane
- extracellular space
- Golgi apparatus
- Golgi medial cisterna
- integral component of lumenal side of endoplasmic reticulum membrane
- MHC class I peptide loading complex
- MHC class I protein complex
- phagocytic vesicle membrane
- plasma membrane

Biological process

- antigen processing and presentation of endogenous peptide antigen via MHC class I via ER pathway, TAP-dependent
- antigen processing and presentation of endogenous peptide antigen via MHC class Ib
- immune response
- negative regulation of neuron projection development
- positive regulation of T cell mediated cytotoxicity

---

10

- **Protein name:** Beta-2-microglobulin
- **Organism:** Homo sapiens
- **Uniprot Accession Number:** P61769
- **Protein sequence length:** 119 aa
- **1D identity (%):** 1.97
- **1D identity (%) [Gaps excluded]:** 35.62
- **1D identity - Alignment Gaps:** 1246
- **Common reported functions (%):** 50.0
- **Common reported locations (%):** 12.5
- **Common reported processes (%):** 0.0

- **PDB ID:** 3SJV
- **Chain:** L
- **Crystallized protein length:** 100 aa
- **Resolution:** 3.1 Å
- **Associated domain:** Ig-like-C1-type
- **b-phipsi:** 0.054889
- **w-rdist:** 0.623644
- **t-alpha:** 0.0
- **Chemical similarity (Tanimoto Index) (%):** 85.38
- **1D identity (%) [PDB]:** 0.0
- **1D identity (%) [Gaps excluded][PDB]:** 0.0
- **1D identity - Alignment Gaps [PDB]:** 1082
- **2D identity (%) [PDB]:** 5.29
- **2D identity (%) [Gaps excluded][PDB]:** 88.52
- **2D identity - Alignment Gaps [PDB]:** 960
- **3D similarity (TM-Score) (%) [PDB]:** 5.93

- **Gene name:** B2M
- **RefSeq ID:** NM\_004048
- **Transcript sequence length:** 943
- **5-UTR|CDS|3-UTR identity (%):** 6.99 | 6.38 | 26.36
- **5-UTR|CDS|3-UTR identity (%) [Gaps excluded]:** 82.61 | 78.91 | 79.9
- **5-UTR|CDS|3-UTR identity [Alignment Gaps]:** 249 | 3556 | 394

**Uniprot Description:**  
  
Component of the class I major histocompatibility complex (MHC). Involved in the presentation of peptide antigens to the immune system. Exogenously applied M.tuberculosis EsxA or EsxA-EsxB (or EsxA expressed in host) binds B2M and decreases its export to the cell surface (total protein levels do not change), probably leading to defects in class I antigen presentation (PubMed:25356553).  
  
Heterodimer of an alpha chain and a beta chain. Beta-2-microglobulin is the beta-chain of major histocompatibility complex class I molecules. Polymers of beta 2-microglobulin can be found in tissues from patients on long-term hemodialysis. B2M alone (not in complex with HLA-I) interacts with M.tuberculosis EsxA (ESAT-6) and an EsxA-EsxB (CFP-10) complex; the tripartite complex can be detected in the host endoplasmic reticulum (PubMed:25356553). The B2M-EsxA complex can be detected in patients with pleural tuberculosis and is stable from pH 4.0 to 8.0 and in the presence of 2M NaCl (PubMed:25356553). Forms a heterotrimer with HLA-E and a self- or a foreign peptide (PubMed:9427624). Forms a heterotrimer with HLA-G and a self-peptide (PubMed:17056715). Forms a heterotrimer with HLA-F and a self-peptide (PubMed:10605026). Forms a heterotrimer with MR1 and a metabolite antigen.  
  
**Gene Ontology Information:**

Molecular Function

- identical protein binding
- protein homodimerization activity

Location

- cytosol
- early endosome lumen
- early endosome membrane
- endoplasmic reticulum lumen
- ER to Golgi transport vesicle membrane
- external side of plasma membrane
- extracellular exosome
- extracellular region
- extracellular space
- focal adhesion
- Golgi apparatus
- Golgi membrane
- HFE-transferrin receptor complex
- membrane
- MHC class I peptide loading complex
- MHC class I protein complex
- phagocytic vesicle membrane
- plasma membrane
- recycling endosome membrane
- specific granule lumen
- tertiary granule lumen

Biological process

- amyloid fibril formation
- antibacterial humoral response
- antigen processing and presentation of endogenous peptide antigen via MHC class I
- antigen processing and presentation of exogenous peptide antigen via MHC class I, TAP-dependent
- antigen processing and presentation of exogenous peptide antigen via MHC class I, TAP-independent
- antigen processing and presentation of exogenous protein antigen via MHC class Ib, TAP-dependent
- antigen processing and presentation of peptide antigen via MHC class I
- antimicrobial humoral immune response mediated by antimicrobial peptide
- cellular response to iron ion
- cellular response to iron(III) ion
- cellular response to lipopolysaccharide
- cellular response to nicotine
- defense response to Gram-negative bacterium
- defense response to Gram-positive bacterium
- innate immune response
- interferon-gamma-mediated signaling pathway
- iron ion homeostasis
- iron ion transport
- learning or memory
- modulation by symbiont of host defense response
- modulation of age-related behavioral decline
- negative regulation of epithelial cell proliferation
- negative regulation of forebrain neuron differentiation
- negative regulation of neurogenesis
- negative regulation of neuron projection development
- negative regulation of receptor binding
- neutrophil degranulation
- positive regulation of cellular senescence
- positive regulation of ferrous iron binding
- positive regulation of protein binding
- positive regulation of receptor binding
- positive regulation of receptor-mediated endocytosis
- positive regulation of T cell cytokine production
- positive regulation of T cell mediated cytotoxicity
- positive regulation of transferrin receptor binding
- protein homotetramerization
- protein refolding
- regulation of defense response to virus by virus
- regulation of erythrocyte differentiation
- regulation of immune response
- regulation of iron ion transport
- regulation of membrane depolarization
- response to cadmium ion
- response to drug
- response to molecule of bacterial origin
- retina homeostasis
- T cell differentiation in thymus

---

11

- **Protein name:** Polymerase cofactor VP35
- **Organism:** Zaire ebolavirus (strain Mayinga-76)
- **Uniprot Accession Number:** Q05127
- **Protein sequence length:** 340 aa
- **1D identity (%):** 6.61
- **1D identity (%) [Gaps excluded]:** 29.29
- **1D identity - Alignment Gaps:** 1019
- **Common reported functions (%):** 0.0
- **Common reported locations (%):** 0.0
- **Common reported processes (%):** 0.0

- **PDB ID:** 3FKE
- **Chain:** B
- **Crystallized protein length:** 127 aa
- **Resolution:** 1.4 Å
- **Associated domain:** VP35-IID
- **b-phipsi:** 0.039325
- **w-rdist:** 0.438762
- **t-alpha:** 0.001812
- **Chemical similarity (Tanimoto Index) (%):** 83.23
- **1D identity (%) [PDB]:** 0.0
- **1D identity (%) [Gaps excluded][PDB]:** 0.0
- **1D identity - Alignment Gaps [PDB]:** 1110
- **2D identity (%) [PDB]:** 7.08
- **2D identity (%) [Gaps excluded][PDB]:** 92.41
- **2D identity - Alignment Gaps [PDB]:** 952
- **3D similarity (TM-Score) (%) [PDB]:** 5.94

- **Gene name:** VP35
- **RefSeq ID:** NC\_002549
- **Genomic sequence length:** 18959
- **5-UTR|CDS|3-UTR identity (%):** 15.79 | 18.42 | 19.86
- **5-UTR|CDS|3-UTR identity (%) [Gaps excluded]:** 77.78 | 80.87 | 74.35
- **5-UTR|CDS|3-UTR identity [Alignment Gaps]:** 212 | 3047 | 524

**Uniprot Description:**  
  
Plays an essential role in viral RNA synthesis and also a role in suppressing innate immune signaling (PubMed:11027311). Acts as a polymerase cofactor in the RNA polymerase transcription and replication complexes (PubMed:9971816, PubMed:16495261, PubMed:24495995). Serves as nucleoprotein/NP monomer chaperone prior to the formation of the large oligomeric RNA-bound complexes (By similarity). Regulates RNA synthesis by modulating NP-RNA interactions and interacting with DYNLL1 (PubMed:25741013). VP35-NP interaction controls the switch between RNA-bound NP and free NP and thus the switch between genome replication and genome packaging into the nucleocapsid (PubMed:25865894). Prevents establishment of cellular antiviral state, thereby suppressing host DC maturation (PubMed:26962215). Acts by inhibiting host DDX58/RIG-I activation both by shielding dsRNA from detection and by preventing PRKRA binding to DDX58 (PubMed:23870315). Blocks virus-induced phosphorylation and activation of interferon regulatory factor 3/IRF3, a transcription factor critical for the induction of interferons alpha and beta (PubMed:12829834). This blockage is produced through the interaction with and inhibition of host IKBKE and TBK1, producing a strong inhibition of the phosphorylation and activation of IRF3 (PubMed:12829834). Also inhibits the antiviral effect mediated by the host interferon-induced, double-stranded RNA-activated protein kinase EIF2AK2/PKR (PubMed:17065211). Increases PIAS1-mediated SUMOylation of IRF7, thereby repressing interferon transcription (PubMed:19557165). Also acts as a suppressor of RNA silencing by interacting with host DICER1, TARBP2/TRBP and PRKRA/PACT (By similarity). As a dimer, binds and sequesters dsRNA contributing to the inhibition of interferon production (By similarity).  
  
Homodimer (By similarity). Homooligomer; via the coiled coil domain (PubMed:16095644). Interacts with nucleoprotein NP and polymerase L; VP35 bridges L and NP and allows the formation of the polymerase complex (PubMed:25865894) (Probable). Also interacts with VP30; this interaction is regulated by VP30 phosphorylation (PubMed:23493393). Interacts with host IKBKE and TBK1; the interactions lead to inhibition of cellular antiviral response by blocking necessary interactions of IKBKE and TBK1 with their substrate IRF3. Interacts with host DYNLL1; this interaction stabilizes VP35 N-terminal oligomerization domain, enhances viral RNA synthesis but does not participate in suppressing the host innate immune response (PubMed:19403681, PubMed:25741013). Interacts with host PRKRA; this interaction inhibits the interaction between DDX58 and PRKRA. Interacts with dsRNA (PubMed:19122151, PubMed:20071589, PubMed:23870315). Interacts with host TRIM6; this interaction plays an important role in promoting efficient viral replication (PubMed:28679761). Interacts with host STAU1 (PubMed:30301857). Interacts with host IRF7, PIAS1 and UBE2I/UBC9; these interactions mediate the sumoylation of IRF7 and contribute to the inhibition of IFN-type I production (PubMed:19557165). Interacts with host DICER1; this interaction prevents TARBP2/TRBP binding to DICER1 and thus allows the virus to counteract host RNA silencing (By similarity). Interacts with host TARBP2/TRBP and PRKRA/PACT; these interactions prevent TARBP2 and PRKRA binding to DICER1 and thus allows the virus to counteract host RNA silencing (By similarity).  
  
**Gene Ontology Information:**

Molecular Function

- RNA binding

Location

- host cell cytoplasm
- viral nucleocapsid

Biological process

- negative regulation of gene expression
- negative regulation of gene silencing by miRNA
- positive regulation of protein sumoylation
- suppression by virus of host antigen processing and presentation of peptide antigen via MHC class II
- suppression by virus of host cytokine production
- suppression by virus of host IKBKE activity
- suppression by virus of host IRF7 activity
- suppression by virus of host protein phosphorylation
- suppression by virus of host TBK1 activity
- suppression by virus of host toll-like receptor signaling pathway
- suppression by virus of host type I interferon production
- suppression of host defenses by symbiont

---

12

- **Protein name:** Beta-2-microglobulin
- **Organism:** Mus musculus
- **Uniprot Accession Number:** P01887
- **Protein sequence length:** 119 aa
- **1D identity (%):** 2.18
- **1D identity (%) [Gaps excluded]:** 26.67
- **1D identity - Alignment Gaps:** 1182
- **Common reported functions (%):** 50.0
- **Common reported locations (%):** 0.0
- **Common reported processes (%):** 0.0

- **PDB ID:** 1S7Q
- **Chain:** B
- **Crystallized protein length:** 99 aa
- **Resolution:** 1.99 Å
- **Associated domain:** Ig-like-C1-type
- **b-phipsi:** 0.039593
- **w-rdist:** 0.644356
- **t-alpha:** 0.001808
- **Chemical similarity (Tanimoto Index) (%):** 85.5
- **1D identity (%) [PDB]:** 0.0
- **1D identity (%) [Gaps excluded][PDB]:** 0.0
- **1D identity - Alignment Gaps [PDB]:** 1082
- **2D identity (%) [PDB]:** 8.06
- **2D identity (%) [Gaps excluded][PDB]:** 89.89
- **2D identity - Alignment Gaps [PDB]:** 904
- **3D similarity (TM-Score) (%) [PDB]:** 6.37

- **Gene name:** B2m
- **RefSeq ID:** N/A
- **Sequence length:** N/A
- **5-UTR|CDS|3-UTR identity (%):** N/A | N/A | N/A
- **5-UTR|CDS|3-UTR identity (%) [Gaps excluded]:** N/A | N/A | N/A
- **5-UTR|CDS|3-UTR identity [Alignment Gaps]:** N/A | N/A | N/A

**Uniprot Description:**  
  
Component of the class I major histocompatibility complex (MHC). Involved in the presentation of peptide antigens to the immune system.  
  
Heterodimer of an alpha chain and a beta chain. Beta-2-microglobulin is the beta-chain of major histocompatibility complex class I molecules. Forms a heterotrimer with MR1 and a metabolite antigen.  
  
**Gene Ontology Information:**

Molecular Function

- identical protein binding
- protein homodimerization activity

Location

- cytosol
- external side of plasma membrane
- extracellular space
- Golgi apparatus
- HFE-transferrin receptor complex
- MHC class I peptide loading complex
- MHC class I protein complex
- phagocytic vesicle membrane
- plasma membrane

Biological process

- amyloid fibril formation
- antibacterial humoral response
- antigen processing and presentation of endogenous peptide antigen via MHC class I
- antigen processing and presentation of exogenous protein antigen via MHC class Ib, TAP-dependent
- antimicrobial humoral immune response mediated by antimicrobial peptide
- cellular defense response
- cellular response to iron ion
- cellular response to iron(III) ion
- cellular response to lipopolysaccharide
- cellular response to nicotine
- defense response to Gram-negative bacterium
- defense response to Gram-positive bacterium
- innate immune response
- iron ion homeostasis
- iron ion transport
- learning or memory
- modulation of age-related behavioral decline
- multicellular organism development
- negative regulation of epithelial cell proliferation
- negative regulation of forebrain neuron differentiation
- negative regulation of neurogenesis
- negative regulation of neuron projection development
- negative regulation of receptor binding
- positive regulation of cellular senescence
- positive regulation of ferrous iron binding
- positive regulation of protein binding
- positive regulation of receptor binding
- positive regulation of receptor-mediated endocytosis
- positive regulation of T cell cytokine production
- positive regulation of T cell mediated cytotoxicity
- positive regulation of transferrin receptor binding
- protein homotetramerization
- protein refolding
- regulation of erythrocyte differentiation
- regulation of iron ion transport
- regulation of membrane depolarization
- response to cadmium ion
- response to drug
- response to molecule of bacterial origin
- T cell differentiation in thymus

---

13

- **Protein name:** Large T antigen
- **Organism:** JC polyomavirus
- **Uniprot Accession Number:** P03072
- **Protein sequence length:** 688 aa
- **1D identity (%):** 2.64
- **1D identity (%) [Gaps excluded]:** 33.8
- **1D identity - Alignment Gaps:** 1677
- **Common reported functions (%):** 0.0
- **Common reported locations (%):** 0.0
- **Common reported processes (%):** 10.0

- **PDB ID:** 5J47
- **Chain:** A
- **Crystallized protein length:** 360 aa
- **Resolution:** 1.99 Å
- **Associated domain:** SF3-helicase
- **b-phipsi:** 0.044738
- **w-rdist:** 0.048108
- **t-alpha:** 0.066908
- **Chemical similarity (Tanimoto Index) (%):** 79.85
- **1D identity (%) [PDB]:** 0.0
- **1D identity (%) [Gaps excluded][PDB]:** 0.0
- **1D identity - Alignment Gaps [PDB]:** 1344
- **2D identity (%) [PDB]:** 17.31
- **2D identity (%) [Gaps excluded][PDB]:** 95.63
- **2D identity - Alignment Gaps [PDB]:** 932
- **3D similarity (TM-Score) (%) [PDB]:** 13.39

- **Gene name:** N/A
- **RefSeq ID:** NC\_001699
- **Genomic sequence length:** 5130
- **5-UTR|CDS|3-UTR identity (%):** N/A | 34.42 | N/A
- **5-UTR|CDS|3-UTR identity (%) [Gaps excluded]:** N/A | 79.02 | N/A
- **5-UTR|CDS|3-UTR identity [Alignment Gaps]:** N/A | 2315 | N/A

**Uniprot Description:**  
  
Isoform large T antigen is a key early protein essential for both driving viral replication and inducing cellular transformation. Plays a role in viral genome replication by driving entry of quiescent cells into the cell cycle and by autoregulating the synthesis of viral early mRNA. Displays highly oncogenic activities by corrupting the host cellular checkpoint mechanisms that guard cell division and the transcription, replication, and repair of DNA. Participates in the modulation of cellular gene expression preceeding viral DNA replication. This step involves binding to host key cell cycle regulators retinoblastoma protein RB1/pRb and TP53. Induces the disassembly of host E2F1 transcription factors from RB1, thus promoting transcriptional activation of E2F1-regulated S-phase genes. Inhibits host TP53 binding to DNA, abrogating the ability of TP53 to stimulate gene expression. Plays the role of a TFIID-associated factor (TAF) in transcription initiation for all three RNA polymerases, by stabilizing the TBP-TFIIA complex on promoters. Initiates viral DNA replication and unwinding via interactions with the viral origin of replication. Binds two adjacent sites in the SV40 origin. The replication fork movement is facilitated by Large T antigen helicase activity. Activates the transcription of viral late mRNA, through host TBP and TFIIA stabilization. Interferes with histone deacetylation mediated by HDAC1, leading to activation of transcription.  
  
Forms homohexamers in the presence of ATP. Interacts with host HDAC1. Interacts (via LXCXE domain) with host RB1; the interaction induces the aberrant dissociation of RB1-E2F1 complex thereby disrupting RB1's activity. Interacts (via LXCXE domain) with host pRB-related proteins RBL1 and RBL2. Interacts (via C-terminus) with host TOP1 and POLA1 allowing DNA replication. Interacts with host TP53, inhibiting TP53 binding to DNA. Interacts with host preinitiation complex components TBP, TFIIA and TFIID to regulate transcription initiation.  
  
**Gene Ontology Information:**

Molecular Function

- ATP binding
- DNA replication origin binding
- hydrolase activity
- metal ion binding

Location

- host cell nucleus

Biological process

- DNA replication
- modulation by virus of host G1/S transition checkpoint
- suppression by virus of host JAK1 activity
- suppression by virus of host type I interferon-mediated signaling pathway

---

14

- **Protein name:** Replicase polyprotein 1ab
- **Organism:** Severe acute respiratory syndrome coronavirus
- **Uniprot Accession Number:** P0C6X7
- **Protein sequence length:** 7073 aa
- **1D identity (%):** 4.01
- **1D identity (%) [Gaps excluded]:** 27.37
- **1D identity - Alignment Gaps:** 6212
- **Common reported functions (%):** 50.0
- **Common reported locations (%):** 12.5
- **Common reported processes (%):** 20.0

- **PDB ID:** 1Z1J
- **Chain:** B
- **Crystallized protein length:** 306 aa
- **Resolution:** 2.8 Å
- **Associated domain:** Macro
- **b-phipsi:** 0.035243
- **w-rdist:** 0.077998
- **t-alpha:** 0.292948
- **Chemical similarity (Tanimoto Index) (%):** 83.88
- **1D identity (%) [PDB]:** 0.08
- **1D identity (%) [Gaps excluded][PDB]:** 100.0
- **1D identity - Alignment Gaps [PDB]:** 1287
- **2D identity (%) [PDB]:** 21.32
- **2D identity (%) [Gaps excluded][PDB]:** 83.59
- **2D identity - Alignment Gaps [PDB]:** 765
- **3D similarity (TM-Score) (%) [PDB]:** 12.66

- **Gene name:** rep
- **RefSeq ID:** NC\_004718
- **Genomic sequence length:** 29751
- **5-UTR|CDS|3-UTR identity (%):** 88.52 | 19.99 | 22.38
- **5-UTR|CDS|3-UTR identity (%) [Gaps excluded]:** 92.28 | 80.71 | 98.18
- **5-UTR|CDS|3-UTR identity [Alignment Gaps]:** 11 | 10233 | 745

**Uniprot Description:**  
  
Isoform Replicase polyprotein 1ab
Multifunctional protein involved in the transcription and replication of viral RNAs. Contains the proteinases responsible for the cleavages of the polyprotein.  
  
Non-structural protein 2
Interacts with host PHB and PHB2.  
  
**Gene Ontology Information:**

Molecular Function

- 3'-5'-exoribonuclease activity
- ATP binding
- cysteine-type endopeptidase activity
- DNA helicase activity
- double-stranded RNA binding
- endonuclease activity
- G-quadruplex RNA binding
- helicase activity
- identical protein binding
- ISG15-specific protease activity
- Lys48-specific deubiquitinase activity
- methyltransferase activity
- mRNA (guanine-N7-)-methyltransferase activity
- mRNA (nucleoside-2'-O-)-methyltransferase activity
- protein dimerization activity
- RNA helicase activity
- RNA-directed 5'-3' RNA polymerase activity
- single-stranded RNA binding
- thiol-dependent ubiquitin-specific protease activity
- zinc ion binding

Location

- cytoplasmic viral factory
- double membrane vesicle viral factory outer membrane
- host cell cytoplasm
- host cell endoplasmic reticulum-Golgi intermediate compartment
- host cell perinuclear region of cytoplasm
- integral component of membrane

Biological process

- 7-methylguanosine mRNA capping
- induction by virus of catabolism of host mRNA
- induction by virus of host autophagy
- methylation
- modulation by virus of host protein ubiquitination
- mRNA methylation
- positive regulation of ubiquitin-specific protease activity
- positive stranded viral RNA replication
- protein autoprocessing
- protein K48-linked deubiquitination
- protein K63-linked deubiquitination
- RNA phosphodiester bond hydrolysis, exonucleolytic
- suppression by virus of host IRF3 activity
- suppression by virus of host ISG15 activity
- suppression by virus of host NF-kappaB transcription factor activity
- suppression by virus of host toll-like receptor signaling pathway
- suppression by virus of host TRAF activity
- suppression by virus of host translation
- suppression by virus of host type I interferon production
- suppression by virus of host type I interferon-mediated signaling pathway
- transcription, DNA-templated
- transcription, RNA-templated
- viral protein processing
- viral RNA genome replication
- viral transcription

---

15

- **Protein name:** Recombinase cre
- **Organism:** Escherichia phage P1
- **Uniprot Accession Number:** P06956
- **Protein sequence length:** 343 aa
- **1D identity (%):** 5.89
- **1D identity (%) [Gaps excluded]:** 24.92
- **1D identity - Alignment Gaps:** 998
- **Common reported functions (%):** 0.0
- **Common reported locations (%):** 0.0
- **Common reported processes (%):** 0.0

- **PDB ID:** 2HOF
- **Chain:** A
- **Crystallized protein length:** 309 aa
- **Resolution:** 2.4 Å
- **Associated domain:** Core-binding-CB
- **b-phipsi:** 0.236558
- **w-rdist:** 0.62418
- **t-alpha:** 0.0
- **Chemical similarity (Tanimoto Index) (%):** 83.13
- **1D identity (%) [PDB]:** 0.08
- **1D identity (%) [Gaps excluded][PDB]:** 100.0
- **1D identity - Alignment Gaps [PDB]:** 1291
- **2D identity (%) [PDB]:** 17.88
- **2D identity (%) [Gaps excluded][PDB]:** 93.27
- **2D identity - Alignment Gaps [PDB]:** 877
- **3D similarity (TM-Score) (%) [PDB]:** 12.44

- **Gene name:** cre
- **RefSeq ID:** NC\_005856
- **Genomic sequence length:** 94800
- **5-UTR|CDS|3-UTR identity (%):** N/A | 17.47 | N/A
- **5-UTR|CDS|3-UTR identity (%) [Gaps excluded]:** N/A | 79.91 | N/A
- **5-UTR|CDS|3-UTR identity [Alignment Gaps]:** N/A | 3112 | N/A

**Uniprot Description:**  
  
Catalyzes site-specific recombination between two 34-base-pair LOXP sites. Its role is to maintain the phage genome as a monomeric unit-copy plasmid in the lysogenic state.  
  
Homotetramer when bound to DNA.  
  
**Gene Ontology Information:**

Molecular Function

- DNA binding

Location  
  
N/A

Biological process

- DNA integration
- DNA recombination

---

16

- **Protein name:** Junctional adhesion molecule A
- **Organism:** Felis catus
- **Uniprot Accession Number:** Q2WGK2
- **Protein sequence length:** 298 aa
- **1D identity (%):** 4.79
- **1D identity (%) [Gaps excluded]:** 27.23
- **1D identity - Alignment Gaps:** 1101
- **Common reported functions (%):** 0.0
- **Common reported locations (%):** 12.5
- **Common reported processes (%):** 0.0

- **PDB ID:** 6GSI
- **Chain:** G
- **Crystallized protein length:** 200 aa
- **Resolution:** 3.75 Å
- **Associated domain:** Ig-like-V-type-1
- **b-phipsi:** 0.046865
- **w-rdist:** 0.655709
- **t-alpha:** 0.001808
- **Chemical similarity (Tanimoto Index) (%):** N/A
- **1D identity (%) [PDB]:** 0.0
- **1D identity (%) [Gaps excluded][PDB]:** 0.0
- **1D identity - Alignment Gaps [PDB]:** 1183
- **2D identity (%) [PDB]:** 13.33
- **2D identity (%) [Gaps excluded][PDB]:** 88.39
- **2D identity - Alignment Gaps [PDB]:** 873
- **3D similarity (TM-Score) (%) [PDB]:** 14.37

- **Gene name:** F11R
- **RefSeq ID:** N/A
- **Sequence length:** N/A
- **5-UTR|CDS|3-UTR identity (%):** N/A | N/A | N/A
- **5-UTR|CDS|3-UTR identity (%) [Gaps excluded]:** N/A | N/A | N/A
- **5-UTR|CDS|3-UTR identity [Alignment Gaps]:** N/A | N/A | N/A

**Uniprot Description:**  
  
Seems to play a role in epithelial tight junction formation. Appears early in primordial forms of cell junctions and recruits PARD3. The association of the PARD6-PARD3 complex may prevent the interaction of PARD3 with JAM1, thereby preventing tight junction assembly. Plays a role in regulating monocyte transmigration involved in integrity of epithelial barrier. Ligand for integrin alpha-L/beta-2 involved in memory T-cell and neutrophil transmigration. Involved in platelet activation.  
  
Interacts with the ninth PDZ domain of MPDZ. Interacts with the first PDZ domain of PARD3. The association between PARD3 and PARD6B probably disrupts this interaction. Interacts with ITGAL (via I-domain).  
  
**Gene Ontology Information:**

Molecular Function  
  
N/A

Location

- bicellular tight junction
- integral component of membrane
- plasma membrane

Biological process

- cell adhesion
- establishment of endothelial intestinal barrier
- intestinal absorption
- regulation of membrane permeability
- viral process

---

17

- **Protein name:** Integrase
- **Organism:** Escherichia phage P2
- **Uniprot Accession Number:** P36932
- **Protein sequence length:** 337 aa
- **1D identity (%):** 4.57
- **1D identity (%) [Gaps excluded]:** 30.33
- **1D identity - Alignment Gaps:** 1188
- **Common reported functions (%):** 0.0
- **Common reported locations (%):** 0.0
- **Common reported processes (%):** 10.0

- **PDB ID:** 5DOR
- **Chain:** A
- **Crystallized protein length:** 162 aa
- **Resolution:** 2.5 Å
- **Associated domain:** Tyr-recombinase
- **b-phipsi:** 0.066726
- **w-rdist:** 0.092679
- **t-alpha:** 0.001812
- **Chemical similarity (Tanimoto Index) (%):** 83.07
- **1D identity (%) [PDB]:** 0.09
- **1D identity (%) [Gaps excluded][PDB]:** 50.0
- **1D identity - Alignment Gaps [PDB]:** 1143
- **2D identity (%) [PDB]:** 9.63
- **2D identity (%) [Gaps excluded][PDB]:** 91.74
- **2D identity - Alignment Gaps [PDB]:** 929
- **3D similarity (TM-Score) (%) [PDB]:** 7.8

- **Gene name:** int
- **RefSeq ID:** NC\_001895
- **Genomic sequence length:** 33593
- **5-UTR|CDS|3-UTR identity (%):** N/A | 17.07 | N/A
- **5-UTR|CDS|3-UTR identity (%) [Gaps excluded]:** N/A | 79.14 | N/A
- **5-UTR|CDS|3-UTR identity [Alignment Gaps]:** N/A | 3120 | N/A

**Uniprot Description:**  
  
Integrase is necessary for integration of the phage into the host genome by site-specific recombination.  
  
**Gene Ontology Information:**

Molecular Function

- DNA binding
- hydrolase activity
- transferase activity

Location  
  
N/A

Biological process

- DNA integration
- DNA recombination
- establishment of integrated proviral latency
- viral entry into host cell
- viral genome integration into host DNA

---

18

- **Protein name:** Pol polyprotein
- **Organism:** Feline immunodeficiency virus (isolate Petaluma)
- **Uniprot Accession Number:** P16088
- **Protein sequence length:** 1124 aa
- **1D identity (%):** 4.81
- **1D identity (%) [Gaps excluded]:** 29.2
- **1D identity - Alignment Gaps:** 1719
- **Common reported functions (%):** 0.0
- **Common reported locations (%):** 0.0
- **Common reported processes (%):** 10.0

- **PDB ID:** 1B11
- **Chain:** A
- **Crystallized protein length:** 113 aa
- **Resolution:** 1.9 Å
- **Associated domain:** Peptidase-A2
- **b-phipsi:** 0.002939
- **w-rdist:** 0.691693
- **t-alpha:** 0.385965
- **Chemical similarity (Tanimoto Index) (%):** 77.77
- **1D identity (%) [PDB]:** 0.0
- **1D identity (%) [Gaps excluded][PDB]:** 0.0
- **1D identity - Alignment Gaps [PDB]:** 1096
- **2D identity (%) [PDB]:** 8.57
- **2D identity (%) [Gaps excluded][PDB]:** 92.47
- **2D identity - Alignment Gaps [PDB]:** 910
- **3D similarity (TM-Score) (%) [PDB]:** 5.57

- **Gene name:** pol
- **RefSeq ID:** NC\_001482
- **Genomic sequence length:** 9474
- **5-UTR|CDS|3-UTR identity (%):** N/A | 40.89 | N/A
- **5-UTR|CDS|3-UTR identity (%) [Gaps excluded]:** N/A | 77.95 | N/A
- **5-UTR|CDS|3-UTR identity [Alignment Gaps]:** N/A | 2125 | N/A

**Uniprot Description:**  
  
During replicative cycle of retroviruses, the reverse-transcribed viral DNA is integrated into the host chromosome by the viral integrase enzyme. RNase H activity is associated with the reverse transcriptase.  
  
**Gene Ontology Information:**

Molecular Function

- aspartic-type endopeptidase activity
- DNA binding
- dUTP diphosphatase activity
- exoribonuclease H activity
- magnesium ion binding
- RNA-directed DNA polymerase activity
- RNA-DNA hybrid ribonuclease activity
- zinc ion binding

Location  
  
N/A

Biological process

- DNA integration
- DNA recombination
- dUMP biosynthetic process
- dUTP catabolic process
- establishment of integrated proviral latency
- viral entry into host cell
- viral genome integration into host DNA

---

19

- **Protein name:** Poliovirus receptor
- **Organism:** Homo sapiens
- **Uniprot Accession Number:** P15151
- **Protein sequence length:** 417 aa
- **1D identity (%):** 1.27
- **1D identity (%) [Gaps excluded]:** 52.5
- **1D identity - Alignment Gaps:** 1610
- **Common reported functions (%):** 0.0
- **Common reported locations (%):** 12.5
- **Common reported processes (%):** 0.0

- **PDB ID:** 3EPC
- **Chain:** R
- **Crystallized protein length:** 213 aa
- **Resolution:** 8.0 Å
- **Associated domain:** Ig-like-C2-type-1
- **b-phipsi:** 0.087722
- **w-rdist:** 0.598665
- **t-alpha:** 0.001808
- **Chemical similarity (Tanimoto Index) (%):** N/A
- **1D identity (%) [PDB]:** 0.51
- **1D identity (%) [Gaps excluded][PDB]:** 60.0
- **1D identity - Alignment Gaps [PDB]:** 1176
- **2D identity (%) [PDB]:** 16.47
- **2D identity (%) [Gaps excluded][PDB]:** 85.05
- **2D identity - Alignment Gaps [PDB]:** 808
- **3D similarity (TM-Score) (%) [PDB]:** 10.6

- **Gene name:** PVR
- **RefSeq ID:** NM\_006505
- **Transcript sequence length:** 5792
- **5-UTR|CDS|3-UTR identity (%):** 29.63 | 20.35 | 3.79
- **5-UTR|CDS|3-UTR identity (%) [Gaps excluded]:** 75.0 | 75.28 | 81.37
- **5-UTR|CDS|3-UTR identity [Alignment Gaps]:** 196 | 2916 | 4172

**Uniprot Description:**  
  
Mediates NK cell adhesion and triggers NK cell effector functions. Binds two different NK cell receptors: CD96 and CD226. These interactions accumulates at the cell-cell contact site, leading to the formation of a mature immunological synapse between NK cell and target cell. This may trigger adhesion and secretion of lytic granules and IFN-gamma and activate cytotoxicity of activated NK cells. May also promote NK cell-target cell modular exchange, and PVR transfer to the NK cell. This transfer is more important in some tumor cells expressing a lot of PVR, and may trigger fratricide NK cell activation, providing tumors with a mechanism of immunoevasion. Plays a role in mediating tumor cell invasion and migration.  
  
Can form trans-heterodimers with NECTIN3. The extracellular domain interacts with VTN, CD226 and CD96. The cytoplasmic domain interacts with DYNLT1. Binds with high affinity to TIGIT.  
  
**Gene Ontology Information:**

Molecular Function

- cell adhesion molecule binding
- signaling receptor activity
- virus receptor activity

Location

- adherens junction
- cell surface
- cytoplasm
- extracellular space
- focal adhesion
- integral component of membrane
- plasma membrane

Biological process

- adherens junction organization
- heterophilic cell-cell adhesion via plasma membrane cell adhesion molecules
- homophilic cell adhesion via plasma membrane adhesion molecules
- positive regulation of natural killer cell mediated cytotoxicity
- positive regulation of natural killer cell mediated cytotoxicity directed against tumor cell target
- regulation of immune response
- susceptibility to natural killer cell mediated cytotoxicity
- susceptibility to T cell mediated cytotoxicity

---

20

- **Protein name:** Tail tube protein
- **Organism:** Escherichia phage lambda
- **Uniprot Accession Number:** P03733
- **Protein sequence length:** 246 aa
- **1D identity (%):** 4.32
- **1D identity (%) [Gaps excluded]:** 25.11
- **1D identity - Alignment Gaps:** 1073
- **Common reported functions (%):** 0.0
- **Common reported locations (%):** 0.0
- **Common reported processes (%):** 0.0

- **PDB ID:** 6P3E
- **Chain:** K
- **Crystallized protein length:** 246 aa
- **Resolution:** 5.4 Å
- **Associated domain:** BIG2
- **b-phipsi:** 0.05894
- **w-rdist:** 0.696757
- **t-alpha:** 0.0
- **Chemical similarity (Tanimoto Index) (%):** N/A
- **1D identity (%) [PDB]:** 0.08
- **1D identity (%) [Gaps excluded][PDB]:** 50.0
- **1D identity - Alignment Gaps [PDB]:** 1225
- **2D identity (%) [PDB]:** 16.25
- **2D identity (%) [Gaps excluded][PDB]:** 89.42
- **2D identity - Alignment Gaps [PDB]:** 851
- **3D similarity (TM-Score) (%) [PDB]:** 9.84

- **Gene name:** V
- **RefSeq ID:** NC\_001416
- **Genomic sequence length:** 48502
- **5-UTR|CDS|3-UTR identity (%):** N/A | 12.14 | N/A
- **5-UTR|CDS|3-UTR identity (%) [Gaps excluded]:** N/A | 78.95 | N/A
- **5-UTR|CDS|3-UTR identity [Alignment Gaps]:** N/A | 3347 | N/A

**Uniprot Description:**  
  
Forms the phage's tail tube composed of 32 hexameric disks. When it encounters the appropriate initiation complex gpM and gpL, it assembles in hexameric rings that stack on top of each others. Multimerization ceases when the correct tail length is achieved through a mechanism dependent on tail terminator protein.  
  
Multimerizes into a structure formed by 32 hexameric rings stacked on phage baseplate gpM and gpL. Does not multimerize in solution without the tail initiation complex. Soluble major tail protein interacts with tail assembly protein GT during tail assembly.  
  
**Gene Ontology Information:**

Molecular Function  
  
N/A

Location

- host cell cytoplasm
- virus tail, tube

Biological process

- viral genome ejection through host cell envelope, long flexible tail mechanism
- viral tail assembly

---

21

- **Protein name:** Complement control protein C3
- **Organism:** Vaccinia virus (strain Western Reserve)
- **Uniprot Accession Number:** P68638
- **Protein sequence length:** 263 aa
- **1D identity (%):** 4.5
- **1D identity (%) [Gaps excluded]:** 33.89
- **1D identity - Alignment Gaps:** 1176
- **Common reported functions (%):** 0.0
- **Common reported locations (%):** 37.5
- **Common reported processes (%):** 0.0

- **PDB ID:** 1VVC
- **Chain:** A
- **Crystallized protein length:** 118 aa
- **Resolution:** -1.0 Å
- **Associated domain:** Sushi-1
- **b-phipsi:** 0.162816
- **w-rdist:** 0.571895
- **t-alpha:** 0.001808
- **Chemical similarity (Tanimoto Index) (%):** 84.64
- **1D identity (%) [PDB]:** 0.0
- **1D identity (%) [Gaps excluded][PDB]:** 0.0
- **1D identity - Alignment Gaps [PDB]:** 1101
- **2D identity (%) [PDB]:** 7.65
- **2D identity (%) [Gaps excluded][PDB]:** 81.05
- **2D identity - Alignment Gaps [PDB]:** 911
- **3D similarity (TM-Score) (%) [PDB]:** 6.55

- **Gene name:** VACWR025
- **RefSeq ID:** NC\_006998
- **Genomic sequence length:** 194711
- **5-UTR|CDS|3-UTR identity (%):** N/A | 14.25 | N/A
- **5-UTR|CDS|3-UTR identity (%) [Gaps excluded]:** N/A | 80.06 | N/A
- **5-UTR|CDS|3-UTR identity [Alignment Gaps]:** N/A | 3220 | N/A

**Uniprot Description:**  
  
Serves to protect the virus against complement attack by inhibiting both classical and alternative pathways of complement activation. Binds C3b and C4b.  
  
Heterodimer with A56 protein; disulfide-linked.  
  
**Gene Ontology Information:**

Molecular Function

- complement binding

Location

- extracellular region
- host cell plasma membrane
- integral component of membrane
- virion membrane

Biological process

- suppression by virus of host complement activation

---

22

- **Protein name:** HLA class I histocompatibility antigen, B alpha chain
- **Organism:** Homo sapiens
- **Uniprot Accession Number:** P01889
- **Protein sequence length:** 362 aa
- **1D identity (%):** 5.07
- **1D identity (%) [Gaps excluded]:** 25.27
- **1D identity - Alignment Gaps:** 1089
- **Common reported functions (%):** 0.0
- **Common reported locations (%):** 0.0
- **Common reported processes (%):** 0.0

- **PDB ID:** 6VMX
- **Chain:** F
- **Crystallized protein length:** 267 aa
- **Resolution:** 3.1 Å
- **Associated domain:** Ig-like-C1-type
- **b-phipsi:** 0.026676
- **w-rdist:** 0.694991
- **t-alpha:** 0.001812
- **Chemical similarity (Tanimoto Index) (%):** 85.89
- **1D identity (%) [PDB]:** 0.08
- **1D identity (%) [Gaps excluded][PDB]:** 50.0
- **1D identity - Alignment Gaps [PDB]:** 1247
- **2D identity (%) [PDB]:** 16.09
- **2D identity (%) [Gaps excluded][PDB]:** 90.96
- **2D identity - Alignment Gaps [PDB]:** 875
- **3D similarity (TM-Score) (%) [PDB]:** 9.71

- **Gene name:** HLA-B
- **RefSeq ID:** NM\_005514
- **Transcript sequence length:** 1536
- **5-UTR|CDS|3-UTR identity (%):** 6.79 | 16.87 | 33.71
- **5-UTR|CDS|3-UTR identity (%) [Gaps excluded]:** 85.71 | 75.47 | 72.95
- **5-UTR|CDS|3-UTR identity [Alignment Gaps]:** 244 | 3117 | 241

**Uniprot Description:**  
  
Antigen-presenting major histocompatibility complex class I (MHCI) molecule. In complex with B2M/beta 2 microglobulin displays primarily viral and tumor-derived peptides on antigen-presenting cells for recognition by alpha-beta T cell receptor (TCR) on HLA-B-restricted CD8-positive T cells, guiding antigen-specific T cell immune response to eliminate infected or transformed cells (PubMed:25808313, PubMed:29531227, PubMed:9620674, PubMed:23209413). May also present self-peptides derived from the signal sequence of secreted or membrane proteins, although T cells specific for these peptides are usually inactivated to prevent autoreactivity (PubMed:7743181, PubMed:18991276). Both the peptide and the MHC molecule are recognized by TCR, the peptide is responsible for the fine specificity of antigen recognition and MHC residues account for the MHC restriction of T cells (PubMed:29531227, PubMed:9620674, PubMed:24600035). Typically presents intracellular peptide antigens of 8 to 13 amino acids that arise from cytosolic proteolysis via constitutive proteasome and IFNG-induced immunoproteasome (PubMed:23209413). Can bind different peptides containing allele-specific binding motifs, which are mainly defined by anchor residues at position 2 and 9 (PubMed:25808313, PubMed:29531227).  
  
Heterotrimer that consists of an alpha chain HLA-B, a beta chain B2M and a peptide (peptide-HLA-B-B2M) (PubMed:25808313, PubMed:29531227, PubMed:15657948, PubMed:17057332, PubMed:22020283, PubMed:24600035). Early in biogenesis, HLA-B-B2M dimer interacts with the components of the peptide-loading complex composed of TAPBP, TAP1-TAP2, TAPBPL, PDIA3/ERP57 and CALR (PubMed:9036970, PubMed:9620674, PubMed:26439010, PubMed:26416272). Interacts with TAP1-TAP2 transporter via TAPBP; this interaction is obligatory for the loading of peptide epitopes delivered to the ER by TAP1-TAP2 transporter (PubMed:9036970, PubMed:9620674). Interacts with TAPBPL; TAPBPL binds peptide-free HLA-B-B2M complexes or those loaded with low affinity peptides, likely facilitating peptide exchange for higher affinity peptides (PubMed:26439010). Only optimally assembled peptide-HLA-B-B2M trimer translocates to the surface of antigen-presenting cells, where it interacts with TCR and CD8 coreceptor on the surface of T cells. HLA-B (via polymorphic alpha-1 and alpha-2 domains) interacts with antigen-specific TCR (via CDR1, CDR2 and CDR3 domains) (PubMed:29531227, PubMed:24600035). One HLA-B molecule (mainly via nonpolymorphic alpha-3 domain) interacts with one CD8A homodimer (via CDR-like loop); this interaction insures peptide-HLA-B-B2M recognition by CD8-positive T cells only (PubMed:29531227). Allele B\*57:01 interacts (via Bw4 motif) with KIR3DL1 (via Ig-like C2-type domain); this interaction may interfere with peptide binding (PubMed:22020283, PubMed:25480565). Allele B\*46:01 interacts with KIR2DL3 (PubMed:28514659).  
  
**Gene Ontology Information:**

Molecular Function

- chaperone binding
- peptide antigen binding
- signaling receptor binding
- TAP binding

Location

- cell surface
- early endosome membrane
- endoplasmic reticulum
- ER to Golgi transport vesicle membrane
- extracellular exosome
- Golgi apparatus
- Golgi membrane
- integral component of lumenal side of endoplasmic reticulum membrane
- integral component of plasma membrane
- membrane
- MHC class I protein complex
- phagocytic vesicle membrane
- plasma membrane
- recycling endosome membrane
- secretory granule membrane

Biological process

- adaptive immune response
- antigen processing and presentation of endogenous peptide antigen via MHC class I via ER pathway, TAP-independent
- antigen processing and presentation of exogenous peptide antigen via MHC class I, TAP-dependent
- antigen processing and presentation of exogenous peptide antigen via MHC class I, TAP-independent
- antigen processing and presentation of peptide antigen via MHC class I
- defense response
- detection of bacterium
- immune response
- interferon-gamma-mediated signaling pathway
- neutrophil degranulation
- positive regulation of T cell mediated cytotoxicity
- protection from natural killer cell mediated cytotoxicity
- regulation of dendritic cell differentiation
- regulation of immune response
- regulation of interleukin-12 production
- regulation of interleukin-6 production
- regulation of T cell anergy
- type I interferon signaling pathway
- viral process

---

23

- **Protein name:** Nectin-1
- **Organism:** Homo sapiens
- **Uniprot Accession Number:** Q15223
- **Protein sequence length:** 517 aa
- **1D identity (%):** 6.49
- **1D identity (%) [Gaps excluded]:** 27.57
- **1D identity - Alignment Gaps:** 1108
- **Common reported functions (%):** 50.0
- **Common reported locations (%):** 12.5
- **Common reported processes (%):** 10.0

- **PDB ID:** 3SKU
- **Chain:** D
- **Crystallized protein length:** 181 aa
- **Resolution:** 4.0 Å
- **Associated domain:** Ig-like-C2-type-1
- **b-phipsi:** 0.050732
- **w-rdist:** 0.812352
- **t-alpha:** 0.0
- **Chemical similarity (Tanimoto Index) (%):** N/A
- **1D identity (%) [PDB]:** 0.52
- **1D identity (%) [Gaps excluded][PDB]:** 85.71
- **1D identity - Alignment Gaps [PDB]:** 1153
- **2D identity (%) [PDB]:** 13.39
- **2D identity (%) [Gaps excluded][PDB]:** 84.91
- **2D identity - Alignment Gaps [PDB]:** 849
- **3D similarity (TM-Score) (%) [PDB]:** 8.54

- **Gene name:** NECTIN1
- **RefSeq ID:** NM\_203286
- **Transcript sequence length:** 1909
- **5-UTR|CDS|3-UTR identity (%):** 17.07 | 17.34 | 30.28
- **5-UTR|CDS|3-UTR identity (%) [Gaps excluded]:** 79.01 | 78.95 | 83.48
- **5-UTR|CDS|3-UTR identity [Alignment Gaps]:** 588 | 3123 | 202

**Uniprot Description:**  
  
Promotes cell-cell contacts by forming homophilic or heterophilic trans-dimers. Heterophilic interactions have been detected between NECTIN1 and NECTIN3 and between NECTIN1 and NECTIN4. Has some neurite outgrowth-promoting activity.  
  
Interacts (via Ig-like C2-type domain 2) with FGFR1, FGFR2 and FGFR3 (By similarity). Cis- and trans-homodimer. Can form trans-heterodimers with NECTIN3 and with NECTIN4. Interaction between NECTIN1 and NECTIN3 on the pre- and postsynaptic sites, respectively, initiates the formation of puncta adherentia junctions between axons and dendrites. Interacts (via cytoplasmic domain) with AFDN (via PDZ domain); this interaction recruits NECTIN1 to cadherin-based adherens junctions and provides a connection with the actin cytoskeleton. Interacts with integrin alphaV/beta3.  
  
**Gene Ontology Information:**

Molecular Function

- carbohydrate binding
- cell adhesion molecule binding
- coreceptor activity
- identical protein binding
- protein homodimerization activity
- protein-containing complex binding
- virion binding
- virus receptor activity

Location

- adherens junction
- apical junction complex
- cell-cell contact zone
- dendrite
- extracellular region
- growth cone membrane
- hippocampal mossy fiber to CA3 synapse
- integral component of membrane
- integral component of presynaptic active zone membrane
- intracellular membrane-bounded organelle
- membrane
- plasma membrane

Biological process

- adherens junction organization
- axon guidance
- cell adhesion
- cell-cell adhesion
- desmosome organization
- enamel mineralization
- heterophilic cell-cell adhesion via plasma membrane cell adhesion molecules
- homophilic cell adhesion via plasma membrane adhesion molecules
- immune response
- iron ion transport
- lens morphogenesis in camera-type eye
- protein localization to cell junction
- regulation of synapse assembly
- retina development in camera-type eye
- viral entry into host cell
- virion attachment to host cell

---

24

- **Protein name:** Polyubiquitin-B
- **Organism:** Homo sapiens
- **Uniprot Accession Number:** P0CG47
- **Protein sequence length:** 229 aa
- **1D identity (%):** 2.98
- **1D identity (%) [Gaps excluded]:** 24.84
- **1D identity - Alignment Gaps:** 1180
- **Common reported functions (%):** 0.0
- **Common reported locations (%):** 12.5
- **Common reported processes (%):** 10.0

- **PDB ID:** 5WFI
- **Chain:** C
- **Crystallized protein length:** 76 aa
- **Resolution:** 1.85 Å
- **Associated domain:** Ubiquitin-like-1
- **b-phipsi:** 0.018163
- **w-rdist:** 0.805738
- **t-alpha:** 0.001812
- **Chemical similarity (Tanimoto Index) (%):** 70.88
- **1D identity (%) [PDB]:** 0.0
- **1D identity (%) [Gaps excluded][PDB]:** 0.0
- **1D identity - Alignment Gaps [PDB]:** 1059
- **2D identity (%) [PDB]:** 6.08
- **2D identity (%) [Gaps excluded][PDB]:** 83.33
- **2D identity - Alignment Gaps [PDB]:** 915
- **3D similarity (TM-Score) (%) [PDB]:** 5.18

- **Gene name:** UBB
- **RefSeq ID:** NM\_018955
- **Transcript sequence length:** 933
- **5-UTR|CDS|3-UTR identity (%):** 23.99 | 11.72 | 36.96
- **5-UTR|CDS|3-UTR identity (%) [Gaps excluded]:** 69.15 | 79.62 | 82.61
- **5-UTR|CDS|3-UTR identity [Alignment Gaps]:** 177 | 3354 | 142

**Uniprot Description:**  
  
Ubiquitin
Exists either covalently attached to another protein, or free (unanchored). When covalently bound, it is conjugated to target proteins via an isopeptide bond either as a monomer (monoubiquitin), a polymer linked via different Lys residues of the ubiquitin (polyubiquitin chains) or a linear polymer linked via the initiator Met of the ubiquitin (linear polyubiquitin chains). Polyubiquitin chains, when attached to a target protein, have different functions depending on the Lys residue of the ubiquitin that is linked: Lys-6-linked may be involved in DNA repair; Lys-11-linked is involved in ERAD (endoplasmic reticulum-associated degradation) and in cell-cycle regulation; Lys-29-linked is involved in lysosomal degradation; Lys-33-linked is involved in kinase modification; Lys-48-linked is involved in protein degradation via the proteasome; Lys-63-linked is involved in endocytosis, DNA-damage responses as well as in signaling processes leading to activation of the transcription factor NF-kappa-B. Linear polymer chains formed via attachment by the initiator Met lead to cell signaling. Ubiquitin is usually conjugated to Lys residues of target proteins, however, in rare cases, conjugation to Cys or Ser residues has been observed. When polyubiquitin is free (unanchored-polyubiquitin), it also has distinct roles, such as in activation of protein kinases, and in signaling.  
  
Interacts with SKP1-KMD2A and SKP1-KMD2B complexes.  
  
**Gene Ontology Information:**

Molecular Function

- protein tag
- ubiquitin protein ligase binding

Location

- cytoplasm
- cytosol
- endocytic vesicle membrane
- endoplasmic reticulum membrane
- endoplasmic reticulum quality control compartment
- endosome membrane
- extracellular exosome
- extracellular space
- host cell
- mitochondrial outer membrane
- mitochondrion
- neuron projection
- neuronal cell body
- nucleoplasm
- nucleus
- plasma membrane
- vesicle

Biological process

- activation of MAPK activity
- aggrephagy
- amyloid fibril formation
- anaphase-promoting complex-dependent catabolic process
- cytokine-mediated signaling pathway
- cytoplasmic pattern recognition receptor signaling pathway
- DNA damage response, detection of DNA damage
- endoplasmic reticulum mannose trimming
- endosomal transport
- energy homeostasis
- error-free translesion synthesis
- error-prone translesion synthesis
- fat pad development
- female gonad development
- female meiosis I
- global genome nucleotide-excision repair
- hypothalamus gonadotrophin-releasing hormone neuron development
- I-kappaB kinase/NF-kappaB signaling
- interleukin-1-mediated signaling pathway
- interstrand cross-link repair
- intracellular transport of virus
- JNK cascade
- male meiosis I
- membrane organization
- mitochondrion transport along microtubule
- modification-dependent protein catabolic process
- modulation by symbiont of host defense response
- MyD88-dependent toll-like receptor signaling pathway
- MyD88-independent toll-like receptor signaling pathway
- negative regulation of apoptotic process
- negative regulation of transcription by RNA polymerase II
- negative regulation of transforming growth factor beta receptor signaling pathway
- neuron projection morphogenesis
- nucleotide-binding oligomerization domain containing signaling pathway
- nucleotide-excision repair, DNA damage recognition
- nucleotide-excision repair, DNA duplex unwinding
- nucleotide-excision repair, DNA gap filling
- nucleotide-excision repair, DNA incision
- nucleotide-excision repair, DNA incision, 5'-to lesion
- nucleotide-excision repair, preincision complex assembly
- positive regulation of apoptotic process
- positive regulation of intrinsic apoptotic signaling pathway by p53 class mediator
- positive regulation of NF-kappaB transcription factor activity
- positive regulation of protein monoubiquitination
- positive regulation of protein ubiquitination
- positive regulation of transcription by RNA polymerase II
- pre-replicative complex assembly
- protein deubiquitination
- protein localization
- protein polyubiquitination
- protein ubiquitination
- regulation of exit from mitosis
- regulation of mitochondrial membrane potential
- regulation of mRNA stability
- regulation of neuron death
- regulation of proteasomal protein catabolic process
- regulation of transcription from RNA polymerase II promoter in response to hypoxia
- seminiferous tubule development
- stress-activated MAPK cascade
- transcription-coupled nucleotide-excision repair
- transforming growth factor beta receptor signaling pathway
- translesion synthesis
- transmembrane transport
- TRIF-dependent toll-like receptor signaling pathway
- viral life cycle
- viral translation
- virion assembly
- Wnt signaling pathway

---

25

- **Protein name:** Envelope glycoprotein gp160
- **Organism:** Human immunodeficiency virus 1
- **Uniprot Accession Number:** B3UES2
- **Protein sequence length:** 860 aa
- **1D identity (%):** 12.91
- **1D identity (%) [Gaps excluded]:** 26.88
- **1D identity - Alignment Gaps:** 749
- **Common reported functions (%):** 50.0
- **Common reported locations (%):** 12.5
- **Common reported processes (%):** 10.0

- **PDB ID:** 6OPP
- **Chain:** C
- **Crystallized protein length:** 97 aa
- **Resolution:** 3.7 Å
- **Associated domain:** GP120
- **b-phipsi:** 0.004075
- **w-rdist:** 0.771227
- **t-alpha:** 0.081374
- **Chemical similarity (Tanimoto Index) (%):** 83.39
- **1D identity (%) [PDB]:** 0.0
- **1D identity (%) [Gaps excluded][PDB]:** 0.0
- **1D identity - Alignment Gaps [PDB]:** 1080
- **2D identity (%) [PDB]:** 6.67
- **2D identity (%) [Gaps excluded][PDB]:** 89.33
- **2D identity - Alignment Gaps [PDB]:** 930
- **3D similarity (TM-Score) (%) [PDB]:** 11.47

- **Gene name:** env
- **RefSeq ID:** N/A
- **Sequence length:** N/A
- **5-UTR|CDS|3-UTR identity (%):** N/A | N/A | N/A
- **5-UTR|CDS|3-UTR identity (%) [Gaps excluded]:** N/A | N/A | N/A
- **5-UTR|CDS|3-UTR identity [Alignment Gaps]:** N/A | N/A | N/A

**Uniprot Description:**  
  
Envelope glycoprotein gp160: Oligomerizes in the host endoplasmic reticulum into predominantly trimers. In a second time, gp160 transits in the host Golgi, where glycosylation is completed. The precursor is then proteolytically cleaved in the trans-Golgi and thereby activated by cellular furin or furin-like proteases to produce gp120 and gp41.  
  
The mature envelope protein (Env) consists of a homotrimer of non-covalently associated gp120-gp41 heterodimers. The resulting complex protrudes from the virus surface as a spike. There seems to be as few as 10 spikes on the average virion. Surface protein gp120 interacts with host CD4, CCR5 and CXCR4. Gp120 also interacts with the C-type lectins CD209/DC-SIGN and CLEC4M/DC-SIGNR (collectively referred to as DC-SIGN(R)). Gp120 and gp41 interact with GalCer. Gp120 interacts with host ITGA4/ITGB7 complex; on CD4+ T-cells, this interaction results in rapid activation of integrin ITGAL/LFA-1, which facilitates efficient cell-to-cell spreading of HIV-1. Gp120 interacts with cell-associated heparan sulfate; this interaction increases virus infectivity on permissive cells and may be involved in infection of CD4- cells.  
  
**Gene Ontology Information:**

Molecular Function

- coreceptor activity
- enzyme binding
- extracellular matrix structural constituent
- identical protein binding
- immunoglobulin binding
- interleukin-16 binding
- interleukin-16 receptor activity
- MHC class II protein binding
- MHC class II protein complex binding
- protein homodimerization activity
- protein kinase binding
- protein tyrosine kinase binding
- signaling receptor activity
- transmembrane signaling receptor activity
- virus receptor activity
- zinc ion binding

Location

- clathrin-coated endocytic vesicle membrane
- early endosome
- endoplasmic reticulum lumen
- endoplasmic reticulum membrane
- external side of plasma membrane
- integral component of plasma membrane
- membrane raft
- plasma membrane
- T cell receptor complex

Biological process

- adaptive immune response
- cell adhesion
- cell surface receptor signaling pathway
- cellular response to granulocyte macrophage colony-stimulating factor stimulus
- cytokine-mediated signaling pathway
- defense response to Gram-negative bacterium
- entry into host
- enzyme linked receptor protein signaling pathway
- fusion of virus membrane with host plasma membrane
- helper T cell enhancement of adaptive immune response
- immune response
- induction by virus of host cell-cell fusion
- interleukin-15-mediated signaling pathway
- macrophage differentiation
- maintenance of protein location in cell
- membrane organization
- positive regulation of calcium ion transport into cytosol
- positive regulation of calcium-mediated signaling
- positive regulation of ERK1 and ERK2 cascade
- positive regulation of I-kappaB kinase/NF-kappaB signaling
- positive regulation of interleukin-2 production
- positive regulation of kinase activity
- positive regulation of MAPK cascade
- positive regulation of monocyte differentiation
- positive regulation of peptidyl-tyrosine phosphorylation
- positive regulation of protein kinase activity
- positive regulation of protein phosphorylation
- positive regulation of T cell proliferation
- positive regulation of transcription, DNA-templated
- positive regulation of viral entry into host cell
- regulation of calcium ion transport
- regulation of defense response to virus by virus
- regulation of T cell activation
- response to estradiol
- response to vitamin D
- signal transduction
- T cell activation
- T cell differentiation
- T cell receptor signaling pathway
- T cell selection
- transmembrane receptor protein tyrosine kinase signaling pathway

---

26

- **Protein name:** Tail spike protein
- **Organism:** Escherichia phage K1F
- **Uniprot Accession Number:** Q04830
- **Protein sequence length:** 1064 aa
- **1D identity (%):** 8.96
- **1D identity (%) [Gaps excluded]:** 29.76
- **1D identity - Alignment Gaps:** 1255
- **Common reported functions (%):** 50.0
- **Common reported locations (%):** 0.0
- **Common reported processes (%):** 0.0

- **PDB ID:** 3GW6
- **Chain:** F
- **Crystallized protein length:** 257 aa
- **Resolution:** 2.6 Å
- **Associated domain:** Peptidase-S74
- **b-phipsi:** 0.016997
- **w-rdist:** 1.170646
- **t-alpha:** 0.001812
- **Chemical similarity (Tanimoto Index) (%):** 83.21
- **1D identity (%) [PDB]:** 0.0
- **1D identity (%) [Gaps excluded][PDB]:** 0.0
- **1D identity - Alignment Gaps [PDB]:** 1242
- **2D identity (%) [PDB]:** 14.27
- **2D identity (%) [Gaps excluded][PDB]:** 90.0
- **2D identity - Alignment Gaps [PDB]:** 902
- **3D similarity (TM-Score) (%) [PDB]:** 12.08

- **Gene name:** N/A
- **RefSeq ID:** NC\_007456
- **Genomic sequence length:** 39704
- **5-UTR|CDS|3-UTR identity (%):** N/A | 41.66 | N/A
- **5-UTR|CDS|3-UTR identity (%) [Gaps excluded]:** N/A | 78.64 | N/A
- **5-UTR|CDS|3-UTR identity [Alignment Gaps]:** N/A | 2157 | N/A

**Uniprot Description:**  
  
Tail spike protein
Responsible for initial absorption of the phage to the host bacterium. Degrades the alpha-2,8-linked polysialic acid K1 capsule by cleaving within the polymer chain of polysialic acid.  
  
Tail spike protein
Homotrimer (PubMed:3546309, PubMed:20124697). Interacts with sialic acid (PubMed:15608653, PubMed:20096705).  
  
**Gene Ontology Information:**

Molecular Function

- endo-alpha-(2,8)-sialidase activity
- identical protein binding

Location

- virus tail, fiber

Biological process

- adhesion receptor-mediated virion attachment to host cell
- disruption of host cell glycocalyx during viral entry
- entry into host
- metabolic process
- virion attachment to host cell

---

27

- **Protein name:** Replicase polyprotein 1ab
- **Organism:** Equine arteritis virus (strain Bucyrus)
- **Uniprot Accession Number:** P19811
- **Protein sequence length:** 3175 aa
- **1D identity (%):** 7.13
- **1D identity (%) [Gaps excluded]:** 31.74
- **1D identity - Alignment Gaps:** 2816
- **Common reported functions (%):** 0.0
- **Common reported locations (%):** 12.5
- **Common reported processes (%):** 20.0

- **PDB ID:** 5HBZ
- **Chain:** F
- **Crystallized protein length:** 211 aa
- **Resolution:** 3.1 Å
- **Associated domain:** Peptidase-C32
- **b-phipsi:** 0.003941
- **w-rdist:** 0.837159
- **t-alpha:** 0.112676
- **Chemical similarity (Tanimoto Index) (%):** 83.91
- **1D identity (%) [PDB]:** 0.08
- **1D identity (%) [Gaps excluded][PDB]:** 100.0
- **1D identity - Alignment Gaps [PDB]:** 1191
- **2D identity (%) [PDB]:** 13.58
- **2D identity (%) [Gaps excluded][PDB]:** 86.42
- **2D identity - Alignment Gaps [PDB]:** 869
- **3D similarity (TM-Score) (%) [PDB]:** 9.78

- **Gene name:** rep
- **RefSeq ID:** NC\_002532
- **Genomic sequence length:** 12704
- **5-UTR|CDS|3-UTR identity (%):** N/A | 39.23 | N/A
- **5-UTR|CDS|3-UTR identity (%) [Gaps excluded]:** N/A | 77.64 | N/A
- **5-UTR|CDS|3-UTR identity [Alignment Gaps]:** N/A | 2960 | N/A

**Uniprot Description:**  
  
The replicase polyprotein 1ab is a multifunctional protein: it contains the activities necessary for the transcription of negative stranded RNA, leader RNA, subgenomic mRNAs and progeny virion RNA as well as proteinases responsible for the cleavage of the polyprotein into functional products.  
  
Nsp1 interacts with cellular transcription cofactor SND1/p100.  
  
**Gene Ontology Information:**

Molecular Function

- ATP binding
- cysteine-type endopeptidase activity
- DNA helicase activity
- hydrolase activity, acting on acid anhydrides
- RNA binding
- RNA helicase activity
- RNA-directed 5'-3' RNA polymerase activity
- serine-type endopeptidase activity
- serine-type exopeptidase activity
- thiol-dependent ubiquitin-specific protease activity
- zinc ion binding

Location

- host cell membrane
- host cell nucleus
- host cell perinuclear region of cytoplasm
- integral component of membrane

Biological process

- modulation by virus of host protein ubiquitination
- suppression by virus of host ISG15 activity
- suppression by virus of host type I interferon-mediated signaling pathway
- transcription, DNA-templated
- viral protein processing
- viral RNA genome replication

---

28

- **Protein name:** HLA class I histocompatibility antigen, A alpha chain
- **Organism:** Homo sapiens
- **Uniprot Accession Number:** P04439
- **Protein sequence length:** 365 aa
- **1D identity (%):** 6.03
- **1D identity (%) [Gaps excluded]:** 24.09
- **1D identity - Alignment Gaps:** 982
- **Common reported functions (%):** 0.0
- **Common reported locations (%):** 0.0
- **Common reported processes (%):** 0.0

- **PDB ID:** 5HGB
- **Chain:** A
- **Crystallized protein length:** 274 aa
- **Resolution:** 2.4 Å
- **Associated domain:** Ig-like-C1-type
- **b-phipsi:** 0.048283
- **w-rdist:** 0.676972
- **t-alpha:** 0.001812
- **Chemical similarity (Tanimoto Index) (%):** 85.5
- **1D identity (%) [PDB]:** 0.08
- **1D identity (%) [Gaps excluded][PDB]:** 50.0
- **1D identity - Alignment Gaps [PDB]:** 1253
- **2D identity (%) [PDB]:** 17.21
- **2D identity (%) [Gaps excluded][PDB]:** 88.29
- **2D identity - Alignment Gaps [PDB]:** 847
- **3D similarity (TM-Score) (%) [PDB]:** 10.4

- **Gene name:** HLA-A
- **RefSeq ID:** NM\_002116
- **Transcript sequence length:** 1535
- **5-UTR|CDS|3-UTR identity (%):** 4.4 | 17.53 | 35.36
- **5-UTR|CDS|3-UTR identity (%) [Gaps excluded]:** 85.71 | 75.0 | 78.5
- **5-UTR|CDS|3-UTR identity [Alignment Gaps]:** 259 | 3056 | 244

**Uniprot Description:**  
  
Antigen-presenting major histocompatibility complex class I (MHCI) molecule. In complex with B2M/beta 2 microglobulin displays primarily viral and tumor-derived peptides on antigen-presenting cells for recognition by alpha-beta T cell receptor (TCR) on HLA-A-restricted CD8-positive T cells, guiding antigen-specific T cell immune response to eliminate infected or transformed cells (PubMed:2456340, PubMed:2784196, PubMed:1402688, PubMed:7504010, PubMed:9862734, PubMed:10449296, PubMed:12138174, PubMed:12393434, PubMed:15893615, PubMed:17189421, PubMed:19543285, PubMed:21498667, PubMed:24192765, PubMed:7694806, PubMed:24395804, PubMed:28250417). May also present self-peptides derived from the signal sequence of secreted or membrane proteins, although T cells specific for these peptides are usually inactivated to prevent autoreactivity (PubMed:25880248, PubMed:7506728, PubMed:7679507). Both the peptide and the MHC molecule are recognized by TCR, the peptide is responsible for the fine specificity of antigen recognition and MHC residues account for the MHC restriction of T cells (PubMed:12796775, PubMed:18275829, PubMed:19542454, PubMed:28250417). Typically presents intracellular peptide antigens of 8 to 13 amino acids that arise from cytosolic proteolysis via IFNG-induced immunoproteasome or via endopeptidase IDE/insulin-degrading enzyme (PubMed:17189421, PubMed:20364150, PubMed:17079320, PubMed:26929325, PubMed:27049119). Can bind different peptides containing allele-specific binding motifs, which are mainly defined by anchor residues at position 2 and 9 (PubMed:7504010, PubMed:9862734).  
  
Heterotrimer that consists of an alpha chain HLA-A, a beta chain B2M and a peptide (peptide-HLA-A-B2M) (PubMed:7504010, PubMed:7679507, PubMed:21943705, PubMed:19177349, PubMed:24395804, PubMed:26758806, PubMed:7504010, PubMed:7506728, PubMed:8805302, PubMed:7694806, PubMed:7935798, PubMed:9177355, PubMed:18275829, PubMed:22245737, PubMed:28250417, PubMed:11502003, PubMed:8906788, PubMed:19542454). Early in biogenesis, HLA-A-B2M dimer interacts with the components of the peptide-loading complex composed of TAPBP, TAP1-TAP2, TAPBPL, PDIA3/ERP57 and CALR (PubMed:21263072). Interacts with TAP1-TAP2 transporter via TAPBP; this interaction is obligatory for the loading of peptide epitopes delivered to the ER by TAP1-TAP2 transporter (PubMed:8805302, PubMed:8630735, PubMed:21263072). Interacts with TAPBPL; TAPBPL binds peptide-free HLA-A-B2M complexes or those loaded with low affinity peptides, likely facilitating peptide exchange for higher affinity peptides (PubMed:26869717). Only optimally assembled peptide-HLA-B2M trimer translocates to the surface of antigen-presenting cells, where it interacts with TCR and CD8 coreceptor on the surface of T cells. HLA-A (via polymorphic alpha-1 and alpha-2 domains) interacts with antigen-specific TCR (via CDR3 domains) (PubMed:22245737, PubMed:12796775, PubMed:18275829). One HLA-A molecule (mainly via nonpolymorphic alpha-3 domain) interacts with one CD8A homodimer (via CDR-like loop); this interaction insures peptide-HLA-A-B2M recognition by CD8-positive T cells only (PubMed:9177355, PubMed:2784196). Alleles A\*23:01; A\*24:02 and A\*32:01 interact (via Bw4 motif) with KIR3DL1 on NK cells; this interaction is direct.  
  
**Gene Ontology Information:**

Molecular Function

- beta-2-microglobulin binding
- CD8 receptor binding
- peptide antigen binding
- RNA binding
- signaling receptor binding
- T cell receptor binding
- TAP binding
- TAP complex binding

Location

- cell surface
- early endosome membrane
- endoplasmic reticulum
- endoplasmic reticulum exit site
- endoplasmic reticulum membrane
- ER to Golgi transport vesicle membrane
- extracellular exosome
- Golgi apparatus
- Golgi medial cisterna
- Golgi membrane
- integral component of lumenal side of endoplasmic reticulum membrane
- integral component of plasma membrane
- membrane
- MHC class I peptide loading complex
- MHC class I protein complex
- phagocytic vesicle membrane
- plasma membrane
- recycling endosome membrane

Biological process

- antibacterial humoral response
- antigen processing and presentation of endogenous peptide antigen via MHC class I
- antigen processing and presentation of endogenous peptide antigen via MHC class I via ER pathway, TAP-dependent
- antigen processing and presentation of endogenous peptide antigen via MHC class I via ER pathway, TAP-independent
- antigen processing and presentation of exogenous peptide antigen via MHC class I
- antigen processing and presentation of exogenous peptide antigen via MHC class I, TAP-dependent
- antigen processing and presentation of exogenous peptide antigen via MHC class I, TAP-independent
- antigen processing and presentation of peptide antigen via MHC class I
- CD8-positive, alpha-beta T cell activation
- defense response to Gram-positive bacterium
- detection of bacterium
- immune response
- interferon-gamma-mediated signaling pathway
- positive regulation of CD8-positive, alpha-beta T cell activation
- positive regulation of CD8-positive, alpha-beta T cell proliferation
- positive regulation of interferon-gamma production
- positive regulation of memory T cell activation
- positive regulation of T cell cytokine production
- positive regulation of T cell mediated cytotoxicity
- protection from natural killer cell mediated cytotoxicity
- protein ubiquitination
- regulation of immune response
- T cell mediated cytotoxicity
- T cell mediated cytotoxicity directed against tumor cell target
- T cell receptor signaling pathway
- type I interferon signaling pathway
- viral process

---

29

- **Protein name:** Tyrosine-protein kinase HCK
- **Organism:** Homo sapiens
- **Uniprot Accession Number:** P08631
- **Protein sequence length:** 526 aa
- **1D identity (%):** 0.45
- **1D identity (%) [Gaps excluded]:** 27.59
- **1D identity - Alignment Gaps:** 1741
- **Common reported functions (%):** 0.0
- **Common reported locations (%):** 0.0
- **Common reported processes (%):** 0.0

- **PDB ID:** 3RBB
- **Chain:** D
- **Crystallized protein length:** 58 aa
- **Resolution:** 2.35 Å
- **Associated domain:** SH3
- **b-phipsi:** 0.040512
- **w-rdist:** 0.926146
- **t-alpha:** 0.001808
- **Chemical similarity (Tanimoto Index) (%):** 76.36
- **1D identity (%) [PDB]:** 0.1
- **1D identity (%) [Gaps excluded][PDB]:** 50.0
- **1D identity - Alignment Gaps [PDB]:** 1037
- **2D identity (%) [PDB]:** 3.5
- **2D identity (%) [Gaps excluded][PDB]:** 87.5
- **2D identity - Alignment Gaps [PDB]:** 961
- **3D similarity (TM-Score) (%) [PDB]:** 3.72

- **Gene name:** HCK
- **RefSeq ID:** N/A
- **Sequence length:** N/A
- **5-UTR|CDS|3-UTR identity (%):** N/A | N/A | N/A
- **5-UTR|CDS|3-UTR identity (%) [Gaps excluded]:** N/A | N/A | N/A
- **5-UTR|CDS|3-UTR identity [Alignment Gaps]:** N/A | N/A | N/A

**Uniprot Description:**  
  
Non-receptor tyrosine-protein kinase found in hematopoietic cells that transmits signals from cell surface receptors and plays an important role in the regulation of innate immune responses, including neutrophil, monocyte, macrophage and mast cell functions, phagocytosis, cell survival and proliferation, cell adhesion and migration. Acts downstream of receptors that bind the Fc region of immunoglobulins, such as FCGR1A and FCGR2A, but also CSF3R, PLAUR, the receptors for IFNG, IL2, IL6 and IL8, and integrins, such as ITGB1 and ITGB2. During the phagocytic process, mediates mobilization of secretory lysosomes, degranulation, and activation of NADPH oxidase to bring about the respiratory burst. Plays a role in the release of inflammatory molecules. Promotes reorganization of the actin cytoskeleton and actin polymerization, formation of podosomes and cell protrusions. Inhibits TP73-mediated transcription activation and TP73-mediated apoptosis. Phosphorylates CBL in response to activation of immunoglobulin gamma Fc region receptors. Phosphorylates ADAM15, BCR, ELMO1, FCGR2A, GAB1, GAB2, RAPGEF1, STAT5B, TP73, VAV1 and WAS.  
  
Interacts (via SH2 domain) with FLT3 (tyrosine phosphorylated). Interacts with VAV1, WAS and RAPGEF1 (By similarity). This interaction stimulates its tyrosine-kinase activity. Interacts with ARRB1 and ARRB2. Interacts with ADAM15. Interacts with FASLG. Interacts with CBL. Interacts with FCGR1A; the interaction may be indirect. Interacts with IL6ST. Interacts (via SH3 domain) with ELMO1. Interacts (via SH3 domain) with TP73. Interacts with YAP1. Interacts with ABL1 and ITGB1, and thereby recruits ABL1 to activated ITGB1. Interacts (via SH3 domain) with WDCP.  
  
**Gene Ontology Information:**

Molecular Function

- ATP binding
- non-membrane spanning protein tyrosine kinase activity
- phosphotyrosine residue binding
- protein tyrosine kinase activity
- signaling receptor binding
- transmembrane receptor protein tyrosine kinase activity

Location

- caveola
- cell projection
- cytoskeleton
- cytosol
- extrinsic component of cytoplasmic side of plasma membrane
- focal adhesion
- Golgi apparatus
- intracellular membrane-bounded organelle
- lysosome
- nucleus
- plasma membrane
- transport vesicle

Biological process

- cell adhesion
- cell differentiation
- cytokine-mediated signaling pathway
- Fc-gamma receptor signaling pathway involved in phagocytosis
- inflammatory response
- innate immune response
- innate immune response-activating signal transduction
- integrin-mediated signaling pathway
- interferon-gamma-mediated signaling pathway
- leukocyte degranulation
- leukocyte migration involved in immune response
- lipopolysaccharide-mediated signaling pathway
- mesoderm development
- negative regulation of apoptotic process
- negative regulation of inflammatory response to antigenic stimulus
- peptidyl-tyrosine phosphorylation
- positive regulation of actin cytoskeleton reorganization
- positive regulation of actin filament polymerization
- positive regulation of cell population proliferation
- protein autophosphorylation
- protein phosphorylation
- regulation of cell shape
- regulation of defense response to virus by virus
- regulation of DNA-binding transcription factor activity
- regulation of inflammatory response
- regulation of phagocytosis
- regulation of podosome assembly
- respiratory burst after phagocytosis
- transmembrane receptor protein tyrosine kinase signaling pathway

---

30

- **Protein name:** Flap endonuclease
- **Organism:** Escherichia phage T5
- **Uniprot Accession Number:** P06229
- **Protein sequence length:** 291 aa
- **1D identity (%):** 4.35
- **1D identity (%) [Gaps excluded]:** 25.11
- **1D identity - Alignment Gaps:** 1102
- **Common reported functions (%):** 0.0
- **Common reported locations (%):** 0.0
- **Common reported processes (%):** 0.0

- **PDB ID:** 1UT8
- **Chain:** B
- **Crystallized protein length:** 272 aa
- **Resolution:** 2.75 Å
- **Associated domain:** 5-3-exonuclease
- **b-phipsi:** 0.119085
- **w-rdist:** 0.851105
- **t-alpha:** 0.001812
- **Chemical similarity (Tanimoto Index) (%):** 83.12
- **1D identity (%) [PDB]:** 0.0
- **1D identity (%) [Gaps excluded][PDB]:** 0.0
- **1D identity - Alignment Gaps [PDB]:** 1255
- **2D identity (%) [PDB]:** 16.2
- **2D identity (%) [Gaps excluded][PDB]:** 92.51
- **2D identity - Alignment Gaps [PDB]:** 881
- **3D similarity (TM-Score) (%) [PDB]:** 10.99

- **Gene name:** D15
- **RefSeq ID:** NC\_005859
- **Genomic sequence length:** 121750
- **5-UTR|CDS|3-UTR identity (%):** N/A | 14.96 | N/A
- **5-UTR|CDS|3-UTR identity (%) [Gaps excluded]:** N/A | 79.78 | N/A
- **5-UTR|CDS|3-UTR identity [Alignment Gaps]:** N/A | 3214 | N/A

**Uniprot Description:**  
  
Catalyzes both the 5'-exonucleolytic and structure-specific endonucleolytic hydrolysis of DNA branched nucleic acid molecules and probably plays a role in viral genome replication (PubMed:9874768, PubMed:15077103, PubMed:10364212). Active on flap (branched duplex DNA containing a free single-stranded 5'-end), 5'overhangs and pseudo-Y structures (PubMed:9874768, PubMed:15077103, PubMed:10364212). The substrates require a free, single-stranded 5' end, with endonucleolytic hydrolysis occurring at the junction of double- and single-stranded DNA (PubMed:9874768). This function may be used for example to trim such branched molecules generated by Okazaki fragments synthesis during replication.  
  
**Gene Ontology Information:**

Molecular Function

- 5'-3' exodeoxyribonuclease activity
- 5'-3' exonuclease activity
- 5'-flap endonuclease activity
- DNA binding
- double-stranded DNA endodeoxyribonuclease activity
- exodeoxyribonuclease activity
- metal ion binding

Location

- viral replication complex

Biological process

- DNA replication, Okazaki fragment processing
- late viral transcription
- viral DNA genome replication

---
