# Supplementary material for: Identifying and profiling structural similarities between Spike of SARS-CoV-2 and other viral or host proteins with Machaon
Source: Commun Biol. 2023 Jul 19;6:752. doi: 10.1038/s42003-023-05076-7 (PMC10356814; doi:10.1038/s42003-023-05076-7)
Supplement: Supplementary file 7 — Supplementary Data 4 [file 42003_2023_5076_MOESM7_ESM.zip › 6VXX_A_domain/candidates/6VXX_A_BetaCoV-S1-NTD-merged-enriched_eval_report.html]

 

# Structural Comparison Report for 6VXX\_A\_BetaCoV-S1-NTD - domains (total: 32)

---

1

- **Protein name:** Capsid protein VP0
- **Organism:** Foot-and-mouth disease virus - type SAT 2
- **Uniprot Accession Number:** Q1L764
- **Protein sequence length:** 740 aa
- **1D identity (%):** 8.47
- **1D identity (%) [Gaps excluded]:** 32.22
- **1D identity - Alignment Gaps:** 1175
- **Common reported functions (%):** 0.0
- **Common reported locations (%):** 0.0
- **Common reported processes (%):** 0.0

- **PDB ID:** 5ACA
- **Chain:** 3
- **Crystallized protein length:** 222 aa
- **Resolution:** 3.5 Å
- **Associated domain:** Rhv
- **b-phipsi:** 0.012292
- **w-rdist:** 0.262466
- **t-alpha:** 0.0
- **Chemical similarity (Tanimoto Index) (%):** 84.13
- **1D identity (%) [PDB]:** 0.0
- **1D identity (%) [Gaps excluded][PDB]:** 0.0
- **1D identity - Alignment Gaps [PDB]:** 1205
- **2D identity (%) [PDB]:** 13.57
- **2D identity (%) [Gaps excluded][PDB]:** 84.94
- **2D identity - Alignment Gaps [PDB]:** 873
- **3D similarity (TM-Score) (%) [PDB]:** 9.49

- **Gene name:** N/A
- **RefSeq ID:** N/A
- **Sequence length:** N/A
- **5-UTR|CDS|3-UTR identity (%):** N/A | N/A | N/A
- **5-UTR|CDS|3-UTR identity (%) [Gaps excluded]:** N/A | N/A | N/A
- **5-UTR|CDS|3-UTR identity [Alignment Gaps]:** N/A | N/A | N/A

**Uniprot Description:**  
  
N/A  
  
**Gene Ontology Information:**

Molecular Function  
  
N/A

Location  
  
N/A

Biological process  
  
N/A

---

2

- **Protein name:** Interferon-activable protein 204
- **Organism:** Mus musculus
- **Uniprot Accession Number:** P0DOV2
- **Protein sequence length:** 619 aa
- **1D identity (%):** 6.47
- **1D identity (%) [Gaps excluded]:** 24.68
- **1D identity - Alignment Gaps:** 1106
- **Common reported functions (%):** 50.0
- **Common reported locations (%):** 0.0
- **Common reported processes (%):** 0.0

- **PDB ID:** 5Z7D
- **Chain:** A
- **Crystallized protein length:** 395 aa
- **Resolution:** 4.5 Å
- **Associated domain:** HIN-200-1
- **b-phipsi:** 0.009429
- **w-rdist:** 0.171214
- **t-alpha:** 0.001493
- **Chemical similarity (Tanimoto Index) (%):** 83.19
- **1D identity (%) [PDB]:** 0.0
- **1D identity (%) [Gaps excluded][PDB]:** 0.0
- **1D identity - Alignment Gaps [PDB]:** 1378
- **2D identity (%) [PDB]:** 23.87
- **2D identity (%) [Gaps excluded][PDB]:** 88.4
- **2D identity - Alignment Gaps [PDB]:** 792
- **3D similarity (TM-Score) (%) [PDB]:** 13.4

- **Gene name:** Ifi204
- **RefSeq ID:** N/A
- **Sequence length:** N/A
- **5-UTR|CDS|3-UTR identity (%):** N/A | N/A | N/A
- **5-UTR|CDS|3-UTR identity (%) [Gaps excluded]:** N/A | N/A | N/A
- **5-UTR|CDS|3-UTR identity [Alignment Gaps]:** N/A | N/A | N/A

**Uniprot Description:**  
  
Inhibits the transcription of ribosomal RNA. May inhibit DNA binding by UBTF. Inhibits cell growth via p53/TP53 and RB1-dependent and independent pathways. Acts as a coactivator of RUNX2 during osteogenesis. May be involved in macrophage differentiation. Enables skeletal muscle and cardiac myocyte differentiation by sequestring Id proteins in the cytosol and promoting their ubiquitination and subsequent degradation.  
  
Interacts with UBTF. Interacts with RUNX2. Interacts with ID1, ID2 and ID3.  
  
**Gene Ontology Information:**

Molecular Function

- DNA-binding transcription repressor activity, RNA polymerase II-specific
- double-stranded DNA binding
- identical protein binding
- RNA polymerase II cis-regulatory region sequence-specific DNA binding
- transcription coregulator activity
- transcription factor binding

Location

- cytosol
- nuclear inclusion body
- nuclear speck
- nucleolus
- nucleoplasm
- nucleus

Biological process

- activation of innate immune response
- cellular response to interferon-alpha
- cellular response to interferon-beta
- inner ear development
- intrinsic apoptotic signaling pathway in response to DNA damage by p53 class mediator
- positive regulation of interleukin-1 beta production
- positive regulation of osteoblast differentiation
- regulation of transcription by RNA polymerase II
- response to bacterium

---

3

- **Protein name:** Capsid protein
- **Organism:** Wenzhou tombus-like virus 18
- **Uniprot Accession Number:** A0A1L3KFA2
- **Protein sequence length:** 337 aa
- **1D identity (%):** 6.93
- **1D identity (%) [Gaps excluded]:** 30.64
- **1D identity - Alignment Gaps:** 1016
- **Common reported functions (%):** 0.0
- **Common reported locations (%):** 0.0
- **Common reported processes (%):** 0.0

- **PDB ID:** 6IZL
- **Chain:** B
- **Crystallized protein length:** 201 aa
- **Resolution:** 3.3 Å
- **Associated domain:** ICOSAH-VIR-COAT-S
- **b-phipsi:** 0.025392
- **w-rdist:** 0.324052
- **t-alpha:** 0.0
- **Chemical similarity (Tanimoto Index) (%):** 83.45
- **1D identity (%) [PDB]:** 0.0
- **1D identity (%) [Gaps excluded][PDB]:** 0.0
- **1D identity - Alignment Gaps [PDB]:** 1184
- **2D identity (%) [PDB]:** 13.27
- **2D identity (%) [Gaps excluded][PDB]:** 85.53
- **2D identity - Alignment Gaps [PDB]:** 866
- **3D similarity (TM-Score) (%) [PDB]:** 8.51

- **Gene name:** N/A
- **RefSeq ID:** NC\_033431
- **Genomic sequence length:** 3883
- **5-UTR|CDS|3-UTR identity (%):** N/A | 17.49 | N/A
- **5-UTR|CDS|3-UTR identity (%) [Gaps excluded]:** N/A | 79.86 | N/A
- **5-UTR|CDS|3-UTR identity [Alignment Gaps]:** N/A | 3098 | N/A

**Uniprot Description:**  
  
N/A  
  
**Gene Ontology Information:**

Molecular Function

- structural molecule activity

Location

- T=3 icosahedral viral capsid

Biological process  
  
N/A

---

4

- **Protein name:** Structural polyprotein
- **Organism:** Sindbis virus
- **Uniprot Accession Number:** P03316
- **Protein sequence length:** 1245 aa
- **1D identity (%):** 12.7
- **1D identity (%) [Gaps excluded]:** 30.12
- **1D identity - Alignment Gaps:** 1024
- **Common reported functions (%):** 0.0
- **Common reported locations (%):** 50.0
- **Common reported processes (%):** 10.0

- **PDB ID:** 2SNW
- **Chain:** A
- **Crystallized protein length:** 158 aa
- **Resolution:** 2.7 Å
- **Associated domain:** Peptidase-S3
- **b-phipsi:** 0.014848
- **w-rdist:** 0.669278
- **t-alpha:** 0.0
- **Chemical similarity (Tanimoto Index) (%):** 81.72
- **1D identity (%) [PDB]:** 0.0
- **1D identity (%) [Gaps excluded][PDB]:** 0.0
- **1D identity - Alignment Gaps [PDB]:** 1141
- **2D identity (%) [PDB]:** 12.2
- **2D identity (%) [Gaps excluded][PDB]:** 92.48
- **2D identity - Alignment Gaps [PDB]:** 875
- **3D similarity (TM-Score) (%) [PDB]:** 6.24

- **Gene name:** N/A
- **RefSeq ID:** NC\_001547
- **Genomic sequence length:** 11703
- **5-UTR|CDS|3-UTR identity (%):** N/A | 40.48 | N/A
- **5-UTR|CDS|3-UTR identity (%) [Gaps excluded]:** N/A | 77.26 | N/A
- **5-UTR|CDS|3-UTR identity [Alignment Gaps]:** N/A | 2362 | N/A

**Uniprot Description:**  
  
Capsid protein
Forms an icosahedral capsid with a T=4 symmetry composed of 240 copies of the capsid protein surrounded by a lipid membrane through which penetrate 80 spikes composed of trimers of E1-E2 heterodimers (PubMed:8415660). The capsid protein binds to the viral RNA genome at a site adjacent to a ribosome binding site for viral genome translation following genome release (By similarity). Possesses a protease activity that results in its autocatalytic cleavage from the nascent structural protein (PubMed:1944569). Following its self-cleavage, the capsid protein transiently associates with ribosomes, and within several minutes the protein binds to viral RNA and rapidly assembles into icosahedric core particles (By similarity). The resulting nucleocapsid eventually associates with the cytoplasmic domain of the spike glycoprotein E2 at the cell membrane, leading to budding and formation of mature virions (PubMed:9143274). In case of infection, new virions attach to target cells and after clathrin-mediated endocytosis their membrane fuses with the host endosomal membrane (By similarity). This leads to the release of the nucleocapsid into the cytoplasm, followed by an uncoating event necessary for the genomic RNA to become accessible (By similarity). The uncoating might be triggered by the interaction of capsid proteins with ribosomes (PubMed:3656418). Binding of ribosomes would release the genomic RNA since the same region is genomic RNA-binding and ribosome-binding (By similarity).  
  
Capsid protein
Homomultimer (Probable). Interacts with host karyopherin KPNA4; this interaction allows the nuclear import of the viral capsid protein (By similarity). Interacts with spike glycoprotein E2 (PubMed:9143274).  
  
**Gene Ontology Information:**

Molecular Function

- RNA binding
- serine-type endopeptidase activity
- structural molecule activity
- ubiquitin-like protein ligase binding

Location

- host cell cytoplasm
- host cell nucleus
- host cell plasma membrane
- icosahedral viral capsid, spike
- integral component of membrane
- T=4 icosahedral viral capsid
- viral envelope
- virion membrane

Biological process

- clathrin-dependent endocytosis of virus by host cell
- fusion of virus membrane with host endosome membrane
- membrane fusion
- virion attachment to host cell

---

5

- **Protein name:** Structural polyprotein
- **Organism:** Semliki forest virus
- **Uniprot Accession Number:** P03315
- **Protein sequence length:** 1253 aa
- **1D identity (%):** 8.19
- **1D identity (%) [Gaps excluded]:** 28.8
- **1D identity - Alignment Gaps:** 1408
- **Common reported functions (%):** 0.0
- **Common reported locations (%):** 50.0
- **Common reported processes (%):** 10.0

- **PDB ID:** 1VCP
- **Chain:** C
- **Crystallized protein length:** 149 aa
- **Resolution:** 3.0 Å
- **Associated domain:** Peptidase-S3
- **b-phipsi:** 0.008345
- **w-rdist:** 0.701273
- **t-alpha:** 0.001493
- **Chemical similarity (Tanimoto Index) (%):** 81.14
- **1D identity (%) [PDB]:** 0.0
- **1D identity (%) [Gaps excluded][PDB]:** 0.0
- **1D identity - Alignment Gaps [PDB]:** 1132
- **2D identity (%) [PDB]:** 12.66
- **2D identity (%) [Gaps excluded][PDB]:** 86.21
- **2D identity - Alignment Gaps [PDB]:** 842
- **3D similarity (TM-Score) (%) [PDB]:** 7.07

- **Gene name:** N/A
- **RefSeq ID:** NC\_003215
- **Genomic sequence length:** 11442
- **5-UTR|CDS|3-UTR identity (%):** N/A | 40.5 | N/A
- **5-UTR|CDS|3-UTR identity (%) [Gaps excluded]:** N/A | 76.59 | N/A
- **5-UTR|CDS|3-UTR identity [Alignment Gaps]:** N/A | 2338 | N/A

**Uniprot Description:**  
  
Capsid protein
Forms an icosahedral capsid with a T=4 symmetry composed of 240 copies of the capsid protein surrounded by a lipid membrane through which penetrate 80 spikes composed of trimers of E1-E2 heterodimers (By similarity). The capsid protein binds to the viral RNA genome at a site adjacent to a ribosome binding site for viral genome translation following genome release (By similarity). Possesses a protease activity that results in its autocatalytic cleavage from the nascent structural protein (PubMed:3553612, PubMed:9642067). Following its self-cleavage, the capsid protein transiently associates with ribosomes, and within several minutes the protein binds to viral RNA and rapidly assembles into icosahedric core particles (PubMed:516447). The resulting nucleocapsid eventually associates with the cytoplasmic domain of the spike glycoprotein E2 at the cell membrane, leading to budding and formation of mature virions (By similarity). In case of infection, new virions attach to target cells and after clathrin-mediated endocytosis their membrane fuses with the host endosomal membrane (PubMed:15954801). This leads to the release of the nucleocapsid into the cytoplasm, followed by an uncoating event necessary for the genomic RNA to become accessible (PubMed:1433506). The uncoating might be triggered by the interaction of capsid proteins with ribosomes (PubMed:1433506). Binding of ribosomes would release the genomic RNA since the same region is genomic RNA-binding and ribosome-binding (PubMed:1433506).  
  
Capsid protein
Homodimer (By similarity). Homomultimer (Probable). Interacts with host karyopherin KPNA4; this interaction allows the nuclear import of the viral capsid protein (By similarity). Interacts with spike glycoprotein E2 (By similarity).  
  
**Gene Ontology Information:**

Molecular Function

- RNA binding
- serine-type endopeptidase activity
- structural molecule activity

Location

- host cell endosome
- host cell nucleus
- host cell plasma membrane
- integral component of membrane
- T=4 icosahedral viral capsid
- viral envelope
- virion membrane

Biological process

- clathrin-dependent endocytosis of virus by host cell
- fusion of virus membrane with host endosome membrane
- virion assembly
- virion attachment to host cell

---

6

- **Protein name:** DNA primase/helicase
- **Organism:** Escherichia phage T7
- **Uniprot Accession Number:** P03692
- **Protein sequence length:** 566 aa
- **1D identity (%):** 11.03
- **1D identity (%) [Gaps excluded]:** 30.53
- **1D identity - Alignment Gaps:** 863
- **Common reported functions (%):** 50.0
- **Common reported locations (%):** 0.0
- **Common reported processes (%):** 0.0

- **PDB ID:** 6N7V
- **Chain:** C
- **Crystallized protein length:** 276 aa
- **Resolution:** 3.8 Å
- **Associated domain:** SF4-helicase
- **b-phipsi:** 0.075877
- **w-rdist:** 0.327446
- **t-alpha:** 0.001493
- **Chemical similarity (Tanimoto Index) (%):** 83.2
- **1D identity (%) [PDB]:** 0.08
- **1D identity (%) [Gaps excluded][PDB]:** 50.0
- **1D identity - Alignment Gaps [PDB]:** 1258
- **2D identity (%) [PDB]:** 15.66
- **2D identity (%) [Gaps excluded][PDB]:** 92.35
- **2D identity - Alignment Gaps [PDB]:** 896
- **3D similarity (TM-Score) (%) [PDB]:** 9.67

- **Gene name:** 4
- **RefSeq ID:** NC\_001604
- **Genomic sequence length:** 39937
- **5-UTR|CDS|3-UTR identity (%):** N/A | 29.08 | N/A
- **5-UTR|CDS|3-UTR identity (%) [Gaps excluded]:** N/A | 78.0 | N/A
- **5-UTR|CDS|3-UTR identity [Alignment Gaps]:** N/A | 2523 | N/A

**Uniprot Description:**  
  
Synthesizes short RNA primers for DNA replication. Unwinds the DNA at the replication forks and generates single-stranded DNA for both leading and lagging strand synthesis. The primase synthesizes short RNA primers on the lagging strand that the polymerase elongates using dNTPs.  
  
Homohexamer. Present in a mixture of heptamers and hexamers in the absence of DNA, and assembles onto ssDNA as a hexamer. Interacts with the DNA polymerase gp5; this interaction is essential to initiate leading-strand DNA synthesis. Interacts with single-stranded DNA-binding protein gp2.5.  
  
**Gene Ontology Information:**

Molecular Function

- ATP binding
- DNA helicase activity
- DNA primase activity
- identical protein binding
- zinc ion binding

Location  
  
N/A

Biological process  
  
N/A

---

7

- **Protein name:** RNA-directed RNA polymerase
- **Organism:** Pseudomonas phage phi6
- **Uniprot Accession Number:** P11124
- **Protein sequence length:** 665 aa
- **1D identity (%):** 9.98
- **1D identity (%) [Gaps excluded]:** 27.57
- **1D identity - Alignment Gaps:** 908
- **Common reported functions (%):** 0.0
- **Common reported locations (%):** 0.0
- **Common reported processes (%):** 0.0

- **PDB ID:** 2JLG
- **Chain:** C
- **Crystallized protein length:** 651 aa
- **Resolution:** 2.8 Å
- **Associated domain:** RdRp-catalytic
- **b-phipsi:** 0.10991
- **w-rdist:** 0.431161
- **t-alpha:** 0.0
- **Chemical similarity (Tanimoto Index) (%):** 84.58
- **1D identity (%) [PDB]:** 0.0
- **1D identity (%) [Gaps excluded][PDB]:** 0.0
- **1D identity - Alignment Gaps [PDB]:** 1635
- **2D identity (%) [PDB]:** 26.84
- **2D identity (%) [Gaps excluded][PDB]:** 91.13
- **2D identity - Alignment Gaps [PDB]:** 891
- **3D similarity (TM-Score) (%) [PDB]:** 15.55

- **Gene name:** P2
- **RefSeq ID:** NC\_003715
- **Genomic sequence length:** 6374
- **5-UTR|CDS|3-UTR identity (%):** N/A | 31.76 | N/A
- **5-UTR|CDS|3-UTR identity (%) [Gaps excluded]:** N/A | 77.29 | N/A
- **5-UTR|CDS|3-UTR identity [Alignment Gaps]:** N/A | 2430 | N/A

**Uniprot Description:**  
  
Rna-dependent RNA polymerase part of the packaging complex that packages the viral RNA segments, replicate them into a double-stranded form and transcribe them.  
  
Part of the packaging complex composed of RDRP, P4 and P7. Interacts with P7 (Probable).  
  
**Gene Ontology Information:**

Molecular Function

- metal ion binding
- nucleotide binding
- RNA binding
- RNA uridylyltransferase activity
- RNA-directed 5'-3' RNA polymerase activity

Location

- virion

Biological process

- transcription, DNA-templated
- viral RNA genome replication

---

8

- **Protein name:** Structural polyprotein
- **Organism:** Barmah forest virus
- **Uniprot Accession Number:** P89946
- **Protein sequence length:** 1239 aa
- **1D identity (%):** 15.55
- **1D identity (%) [Gaps excluded]:** 26.18
- **1D identity - Alignment Gaps:** 640
- **Common reported functions (%):** 0.0
- **Common reported locations (%):** 37.5
- **Common reported processes (%):** 10.0

- **PDB ID:** 2YEW
- **Chain:** D
- **Crystallized protein length:** 171 aa
- **Resolution:** 5.0 Å
- **Associated domain:** Peptidase-S3
- **b-phipsi:** 0.011505
- **w-rdist:** 0.682985
- **t-alpha:** 0.001495
- **Chemical similarity (Tanimoto Index) (%):** 81.71
- **1D identity (%) [PDB]:** 0.0
- **1D identity (%) [Gaps excluded][PDB]:** 0.0
- **1D identity - Alignment Gaps [PDB]:** 1154
- **2D identity (%) [PDB]:** 12.2
- **2D identity (%) [Gaps excluded][PDB]:** 89.86
- **2D identity - Alignment Gaps [PDB]:** 878
- **3D similarity (TM-Score) (%) [PDB]:** 7.07

- **Gene name:** N/A
- **RefSeq ID:** NC\_001786
- **Genomic sequence length:** 11488
- **5-UTR|CDS|3-UTR identity (%):** N/A | 40.54 | N/A
- **5-UTR|CDS|3-UTR identity (%) [Gaps excluded]:** N/A | 77.89 | N/A
- **5-UTR|CDS|3-UTR identity [Alignment Gaps]:** N/A | 2378 | N/A

**Uniprot Description:**  
  
Capsid protein
Forms an icosahedral capsid with a T=4 symmetry composed of 240 copies of the capsid protein surrounded by a lipid membrane through which penetrate 80 spikes composed of trimers of E1-E2 heterodimers (By similarity). The capsid protein binds to the viral RNA genome at a site adjacent to a ribosome binding site for viral genome translation following genome release (By similarity). Possesses a protease activity that results in its autocatalytic cleavage from the nascent structural protein (By similarity). Following its self-cleavage, the capsid protein transiently associates with ribosomes, and within several minutes the protein binds to viral RNA and rapidly assembles into icosahedric core particles (By similarity). The resulting nucleocapsid eventually associates with the cytoplasmic domain of the spike glycoprotein E2 at the cell membrane, leading to budding and formation of mature virions (By similarity). In case of infection, new virions attach to target cells and after clathrin-mediated endocytosis their membrane fuses with the host endosomal membrane (By similarity). This leads to the release of the nucleocapsid into the cytoplasm, followed by an uncoating event necessary for the genomic RNA to become accessible (By similarity). The uncoating might be triggered by the interaction of capsid proteins with ribosomes (By similarity). Binding of ribosomes would release the genomic RNA since the same region is genomic RNA-binding and ribosome-binding (By similarity).  
  
Capsid protein
Homodimer (By similarity). Homomultimer (Probable). Interacts with host karyopherin KPNA4; this interaction allows the nuclear import of the viral capsid protein (By similarity).  
  
**Gene Ontology Information:**

Molecular Function

- RNA binding
- serine-type endopeptidase activity
- structural molecule activity

Location

- host cell cytoplasm
- host cell nucleus
- host cell plasma membrane
- integral component of membrane
- T=4 icosahedral viral capsid
- virion membrane

Biological process

- fusion of virus membrane with host endosome membrane
- virion attachment to host cell

---

9

- **Protein name:** Junctional adhesion molecule A
- **Organism:** Homo sapiens
- **Uniprot Accession Number:** Q9Y624
- **Protein sequence length:** 299 aa
- **1D identity (%):** 5.26
- **1D identity (%) [Gaps excluded]:** 26.44
- **1D identity - Alignment Gaps:** 1050
- **Common reported functions (%):** 0.0
- **Common reported locations (%):** 12.5
- **Common reported processes (%):** 0.0

- **PDB ID:** 3EOY
- **Chain:** H
- **Crystallized protein length:** 102 aa
- **Resolution:** 3.4 Å
- **Associated domain:** Ig-like-V-type-1
- **b-phipsi:** 0.000916
- **w-rdist:** 0.843117
- **t-alpha:** 0.066879
- **Chemical similarity (Tanimoto Index) (%):** 85.42
- **1D identity (%) [PDB]:** 0.09
- **1D identity (%) [Gaps excluded][PDB]:** 100.0
- **1D identity - Alignment Gaps [PDB]:** 1083
- **2D identity (%) [PDB]:** 7.25
- **2D identity (%) [Gaps excluded][PDB]:** 93.59
- **2D identity - Alignment Gaps [PDB]:** 929
- **3D similarity (TM-Score) (%) [PDB]:** 6.96

- **Gene name:** F11R
- **RefSeq ID:** NM\_016946
- **Transcript sequence length:** 4639
- **5-UTR|CDS|3-UTR identity (%):** 23.88 | 16.37 | 4.57
- **5-UTR|CDS|3-UTR identity (%) [Gaps excluded]:** 84.21 | 80.15 | 79.62
- **5-UTR|CDS|3-UTR identity [Alignment Gaps]:** 192 | 3120 | 3467

**Uniprot Description:**  
  
Seems to play a role in epithelial tight junction formation. Appears early in primordial forms of cell junctions and recruits PARD3 (PubMed:11489913). The association of the PARD6-PARD3 complex may prevent the interaction of PARD3 with JAM1, thereby preventing tight junction assembly (By similarity). Plays a role in regulating monocyte transmigration involved in integrity of epithelial barrier (By similarity). Ligand for integrin alpha-L/beta-2 involved in memory T-cell and neutrophil transmigration (PubMed:11812992). Involved in platelet activation (PubMed:10753840).  
  
Interacts with the ninth PDZ domain of MPDZ (PubMed:11489913). Interacts with the first PDZ domain of PARD3 (PubMed:11489913). The association between PARD3 and PARD6B probably disrupts this interaction (By similarity). Interacts with ITGAL (via I-domain) (PubMed:15528364).  
  
**Gene Ontology Information:**

Molecular Function

- cadherin binding
- integrin binding
- PDZ domain binding
- protein homodimerization activity
- virus receptor activity

Location

- bicellular tight junction
- cell junction
- cell-cell junction
- cytoplasmic vesicle
- extracellular exosome
- integral component of membrane
- plasma membrane
- protein-containing complex
- slit diaphragm
- tight junction

Biological process

- actomyosin structure organization
- bicellular tight junction assembly
- cell-cell adhesion
- cellular response to mechanical stimulus
- establishment of endothelial intestinal barrier
- extracellular matrix organization
- inflammatory response
- intestinal absorption
- leukocyte cell-cell adhesion
- leukocyte migration
- maintenance of blood-brain barrier
- memory T cell extravasation
- negative regulation of GTPase activity
- negative regulation of stress fiber assembly
- positive regulation of blood pressure
- positive regulation of establishment of endothelial barrier
- positive regulation of GTPase activity
- positive regulation of platelet aggregation
- positive regulation of Rho protein signal transduction
- protein localization to bicellular tight junction
- protein localization to plasma membrane
- regulation of actin cytoskeleton organization
- regulation of actin cytoskeleton reorganization
- regulation of bicellular tight junction assembly
- regulation of cell shape
- regulation of cytokine production
- regulation of cytoskeleton organization
- regulation of membrane permeability
- response to radiation
- transforming growth factor beta receptor signaling pathway

---

10

- **Protein name:** Integrase
- **Organism:** Escherichia phage lambda
- **Uniprot Accession Number:** P03700
- **Protein sequence length:** 356 aa
- **1D identity (%):** 5.08
- **1D identity (%) [Gaps excluded]:** 27.78
- **1D identity - Alignment Gaps:** 1125
- **Common reported functions (%):** 0.0
- **Common reported locations (%):** 0.0
- **Common reported processes (%):** 10.0

- **PDB ID:** 5J0N
- **Chain:** G
- **Crystallized protein length:** 345 aa
- **Resolution:** 11.0 Å
- **Associated domain:** Tyr-recombinase
- **b-phipsi:** 0.116844
- **w-rdist:** 0.489289
- **t-alpha:** 0.0
- **Chemical similarity (Tanimoto Index) (%):** N/A
- **1D identity (%) [PDB]:** 0.0
- **1D identity (%) [Gaps excluded][PDB]:** 0.0
- **1D identity - Alignment Gaps [PDB]:** 1329
- **2D identity (%) [PDB]:** 16.46
- **2D identity (%) [Gaps excluded][PDB]:** 90.24
- **2D identity - Alignment Gaps [PDB]:** 919
- **3D similarity (TM-Score) (%) [PDB]:** 11.46

- **Gene name:** int
- **RefSeq ID:** NC\_001416
- **Genomic sequence length:** 48502
- **5-UTR|CDS|3-UTR identity (%):** N/A | N/A | N/A
- **5-UTR|CDS|3-UTR identity (%) [Gaps excluded]:** N/A | N/A | N/A
- **5-UTR|CDS|3-UTR identity [Alignment Gaps]:** N/A | N/A | N/A

**Uniprot Description:**  
  
Integrase is necessary for integration of the phage into the host genome by site-specific recombination. In conjunction with excisionase, integrase is also necessary for excision of the prophage from the host genome.  
  
Homotetramer. Interacts (via N-terminus) with the excisionase (via C-terminus) (PubMed:12832614). Part of the excision complex made of the integrase tetramer, IHF, Fis and Xis.  
  
**Gene Ontology Information:**

Molecular Function

- DNA binding
- hydrolase activity
- integrase activity
- transferase activity

Location  
  
N/A

Biological process

- DNA integration
- DNA recombination
- establishment of integrated proviral latency
- provirus excision
- viral entry into host cell
- viral genome integration into host DNA

---

11

- **Protein name:** Recombinase cre
- **Organism:** Escherichia phage P1
- **Uniprot Accession Number:** P06956
- **Protein sequence length:** 343 aa
- **1D identity (%):** 5.89
- **1D identity (%) [Gaps excluded]:** 24.92
- **1D identity - Alignment Gaps:** 998
- **Common reported functions (%):** 0.0
- **Common reported locations (%):** 0.0
- **Common reported processes (%):** 0.0

- **PDB ID:** 1NZB
- **Chain:** A
- **Crystallized protein length:** 332 aa
- **Resolution:** 3.1 Å
- **Associated domain:** Tyr-recombinase
- **b-phipsi:** 0.114161
- **w-rdist:** 0.06967
- **t-alpha:** 0.014925
- **Chemical similarity (Tanimoto Index) (%):** 82.21
- **1D identity (%) [PDB]:** 0.0
- **1D identity (%) [Gaps excluded][PDB]:** 0.0
- **1D identity - Alignment Gaps [PDB]:** 1315
- **2D identity (%) [PDB]:** 17.13
- **2D identity (%) [Gaps excluded][PDB]:** 92.23
- **2D identity - Alignment Gaps [PDB]:** 903
- **3D similarity (TM-Score) (%) [PDB]:** 11.69

- **Gene name:** cre
- **RefSeq ID:** NC\_005856
- **Genomic sequence length:** 94800
- **5-UTR|CDS|3-UTR identity (%):** N/A | 17.47 | N/A
- **5-UTR|CDS|3-UTR identity (%) [Gaps excluded]:** N/A | 79.91 | N/A
- **5-UTR|CDS|3-UTR identity [Alignment Gaps]:** N/A | 3112 | N/A

**Uniprot Description:**  
  
Catalyzes site-specific recombination between two 34-base-pair LOXP sites. Its role is to maintain the phage genome as a monomeric unit-copy plasmid in the lysogenic state.  
  
Homotetramer when bound to DNA.  
  
**Gene Ontology Information:**

Molecular Function

- DNA binding

Location  
  
N/A

Biological process

- DNA integration
- DNA recombination

---

12

- **Protein name:** T-cell surface glycoprotein CD4
- **Organism:** Homo sapiens
- **Uniprot Accession Number:** P01730
- **Protein sequence length:** 458 aa
- **1D identity (%):** 6.23
- **1D identity (%) [Gaps excluded]:** 27.59
- **1D identity - Alignment Gaps:** 1093
- **Common reported functions (%):** 50.0
- **Common reported locations (%):** 12.5
- **Common reported processes (%):** 10.0

- **PDB ID:** 4R4H
- **Chain:** B
- **Crystallized protein length:** 178 aa
- **Resolution:** 4.28 Å
- **Associated domain:** Ig-like-V-type
- **b-phipsi:** 0.001653
- **w-rdist:** 0.595043
- **t-alpha:** 0.547344
- **Chemical similarity (Tanimoto Index) (%):** N/A
- **1D identity (%) [PDB]:** 0.09
- **1D identity (%) [Gaps excluded][PDB]:** 100.0
- **1D identity - Alignment Gaps [PDB]:** 1159
- **2D identity (%) [PDB]:** 11.82
- **2D identity (%) [Gaps excluded][PDB]:** 88.32
- **2D identity - Alignment Gaps [PDB]:** 887
- **3D similarity (TM-Score) (%) [PDB]:** 10.6

- **Gene name:** CD4
- **RefSeq ID:** NM\_000616
- **Transcript sequence length:** 3049
- **5-UTR|CDS|3-UTR identity (%):** 36.71 | 23.32 | 8.36
- **5-UTR|CDS|3-UTR identity (%) [Gaps excluded]:** 76.82 | 77.54 | 76.65
- **5-UTR|CDS|3-UTR identity [Alignment Gaps]:** 165 | 2795 | 1365

**Uniprot Description:**  
  
Integral membrane glycoprotein that plays an essential role in the immune response and serves multiple functions in responses against both external and internal offenses. In T-cells, functions primarily as a coreceptor for MHC class II molecule:peptide complex. The antigens presented by class II peptides are derived from extracellular proteins while class I peptides are derived from cytosolic proteins. Interacts simultaneously with the T-cell receptor (TCR) and the MHC class II presented by antigen presenting cells (APCs). In turn, recruits the Src kinase LCK to the vicinity of the TCR-CD3 complex. LCK then initiates different intracellular signaling pathways by phosphorylating various substrates ultimately leading to lymphokine production, motility, adhesion and activation of T-helper cells. In other cells such as macrophages or NK cells, plays a role in differentiation/activation, cytokine expression and cell migration in a TCR/LCK-independent pathway. Participates in the development of T-helper cells in the thymus and triggers the differentiation of monocytes into functional mature macrophages.  
  
Forms disulfide-linked homodimers at the cell surface. Interacts with LCK (PubMed:16888650). Interacts with PTK2/FAK1 (PubMed:18078954). Binds to P4HB/PDI. Interacts with IL16; this interaction induces a CD4-dependent signaling in lymphocytes (PubMed:1673145). Interacts (via Ig-like V-type domain) with MHCII alpha chain (via alpha-2 domain) and beta chain (via beta-2 domain); this interaction increases the affinity of TCR for peptide-MHCII. CD4 oligomerization via Ig-like C2-type 2 and 3 domains appears to be required for stable binding to MHCII and adhesion between T cells and APCs (PubMed:27114505, PubMed:21900604, PubMed:7604010).  
  
**Gene Ontology Information:**

Molecular Function

- coreceptor activity
- enzyme binding
- extracellular matrix structural constituent
- identical protein binding
- immunoglobulin binding
- interleukin-16 binding
- interleukin-16 receptor activity
- MHC class II protein binding
- MHC class II protein complex binding
- protein homodimerization activity
- protein kinase binding
- protein tyrosine kinase binding
- signaling receptor activity
- transmembrane signaling receptor activity
- virus receptor activity
- zinc ion binding

Location

- clathrin-coated endocytic vesicle membrane
- early endosome
- endoplasmic reticulum lumen
- endoplasmic reticulum membrane
- external side of plasma membrane
- integral component of plasma membrane
- membrane raft
- plasma membrane
- T cell receptor complex

Biological process

- adaptive immune response
- cell adhesion
- cell surface receptor signaling pathway
- cellular response to granulocyte macrophage colony-stimulating factor stimulus
- cytokine-mediated signaling pathway
- defense response to Gram-negative bacterium
- entry into host
- enzyme linked receptor protein signaling pathway
- fusion of virus membrane with host plasma membrane
- helper T cell enhancement of adaptive immune response
- immune response
- induction by virus of host cell-cell fusion
- interleukin-15-mediated signaling pathway
- macrophage differentiation
- maintenance of protein location in cell
- membrane organization
- positive regulation of calcium ion transport into cytosol
- positive regulation of calcium-mediated signaling
- positive regulation of ERK1 and ERK2 cascade
- positive regulation of I-kappaB kinase/NF-kappaB signaling
- positive regulation of interleukin-2 production
- positive regulation of kinase activity
- positive regulation of MAPK cascade
- positive regulation of monocyte differentiation
- positive regulation of peptidyl-tyrosine phosphorylation
- positive regulation of protein kinase activity
- positive regulation of protein phosphorylation
- positive regulation of T cell proliferation
- positive regulation of transcription, DNA-templated
- positive regulation of viral entry into host cell
- regulation of calcium ion transport
- regulation of defense response to virus by virus
- regulation of T cell activation
- response to estradiol
- response to vitamin D
- signal transduction
- T cell activation
- T cell differentiation
- T cell receptor signaling pathway
- T cell selection
- transmembrane receptor protein tyrosine kinase signaling pathway

---

13

- **Protein name:** Guanine nucleotide-binding protein G(s) subunit alpha isoforms short
- **Organism:** Homo sapiens
- **Uniprot Accession Number:** P63092
- **Protein sequence length:** 394 aa
- **1D identity (%):** 5.1
- **1D identity (%) [Gaps excluded]:** 28.35
- **1D identity - Alignment Gaps:** 1159
- **Common reported functions (%):** 0.0
- **Common reported locations (%):** 0.0
- **Common reported processes (%):** 0.0

- **PDB ID:** 6NI3
- **Chain:** A
- **Crystallized protein length:** 234 aa
- **Resolution:** 3.8 Å
- **Associated domain:** G-alpha
- **b-phipsi:** 0.086418
- **w-rdist:** 0.127053
- **t-alpha:** 0.161194
- **Chemical similarity (Tanimoto Index) (%):** 83.58
- **1D identity (%) [PDB]:** 0.0
- **1D identity (%) [Gaps excluded][PDB]:** 0.0
- **1D identity - Alignment Gaps [PDB]:** 1219
- **2D identity (%) [PDB]:** 13.73
- **2D identity (%) [Gaps excluded][PDB]:** 93.59
- **2D identity - Alignment Gaps [PDB]:** 907
- **3D similarity (TM-Score) (%) [PDB]:** 9.27

- **Gene name:** GNAS
- **RefSeq ID:** NM\_080426
- **Transcript sequence length:** 1809
- **5-UTR|CDS|3-UTR identity (%):** 11.81 | 19.86 | 35.98
- **5-UTR|CDS|3-UTR identity (%) [Gaps excluded]:** 72.5 | 79.46 | 77.96
- **5-UTR|CDS|3-UTR identity [Alignment Gaps]:** 411 | 2979 | 217

**Uniprot Description:**  
  
Guanine nucleotide-binding proteins (G proteins) function as transducers in numerous signaling pathways controlled by G protein-coupled receptors (GPCRs) (PubMed:17110384). Signaling involves the activation of adenylyl cyclases, resulting in increased levels of the signaling molecule cAMP (PubMed:26206488, PubMed:8702665). GNAS functions downstream of several GPCRs, including beta-adrenergic receptors (PubMed:21488135). Stimulates the Ras signaling pathway via RAPGEF2 (PubMed:12391161).  
  
Heterotrimeric G proteins are composed of 3 units; alpha, beta and gamma. The alpha chain contains the guanine nucleotide binding site. Interacts with CRY1; the interaction may block GPCR-mediated regulation of cAMP concentrations (PubMed:20852621). Interacts with ADCY5 and stimulates its adenylyl cyclase activity (PubMed:17110384, PubMed:26206488). Interacts with ADCY6 and stimulates its adenylyl cyclase activity (PubMed:17110384). Interacts with ADCY2 (By similarity). Interaction with SASH1 (PubMed:23333244). Interacts with GAS2L2 (PubMed:23994616).  
  
**Gene Ontology Information:**

Molecular Function

- adenylate cyclase activator activity
- G-protein beta/gamma-subunit complex binding
- GTP binding
- GTPase activity
- metal ion binding

Location

- cytoplasm
- cytosol
- extracellular exosome
- heterotrimeric G-protein complex
- intrinsic component of membrane
- membrane
- plasma membrane
- trans-Golgi network membrane

Biological process

- activation of adenylate cyclase activity
- adenylate cyclase-activating adrenergic receptor signaling pathway
- adenylate cyclase-activating dopamine receptor signaling pathway
- adenylate cyclase-activating G protein-coupled receptor signaling pathway
- bone development
- cellular response to catecholamine stimulus
- cellular response to glucagon stimulus
- cellular response to prostaglandin E stimulus
- cognition
- developmental growth
- G protein-coupled receptor signaling pathway
- hair follicle placode formation
- intracellular transport
- negative regulation of inflammatory response to antigenic stimulus
- platelet aggregation
- positive regulation of cAMP-mediated signaling
- positive regulation of cold-induced thermogenesis
- positive regulation of GTPase activity
- regulation of insulin secretion
- renal water homeostasis
- sensory perception of smell

---

14

- **Protein name:** Poliovirus receptor
- **Organism:** Homo sapiens
- **Uniprot Accession Number:** P15151
- **Protein sequence length:** 417 aa
- **1D identity (%):** 1.27
- **1D identity (%) [Gaps excluded]:** 52.5
- **1D identity - Alignment Gaps:** 1610
- **Common reported functions (%):** 0.0
- **Common reported locations (%):** 12.5
- **Common reported processes (%):** 0.0

- **PDB ID:** 3J9F
- **Chain:** 7
- **Crystallized protein length:** 116 aa
- **Resolution:** 9.0 Å
- **Associated domain:** Ig-like-V-type
- **b-phipsi:** 0.00181
- **w-rdist:** 0.793252
- **t-alpha:** 0.205036
- **Chemical similarity (Tanimoto Index) (%):** N/A
- **1D identity (%) [PDB]:** 0.09
- **1D identity (%) [Gaps excluded][PDB]:** 100.0
- **1D identity - Alignment Gaps [PDB]:** 1097
- **2D identity (%) [PDB]:** 7.52
- **2D identity (%) [Gaps excluded][PDB]:** 86.36
- **2D identity - Alignment Gaps [PDB]:** 923
- **3D similarity (TM-Score) (%) [PDB]:** 8.68

- **Gene name:** PVR
- **RefSeq ID:** NM\_006505
- **Transcript sequence length:** 5792
- **5-UTR|CDS|3-UTR identity (%):** 29.63 | 20.35 | 3.79
- **5-UTR|CDS|3-UTR identity (%) [Gaps excluded]:** 75.0 | 75.28 | 81.37
- **5-UTR|CDS|3-UTR identity [Alignment Gaps]:** 196 | 2916 | 4172

**Uniprot Description:**  
  
Mediates NK cell adhesion and triggers NK cell effector functions. Binds two different NK cell receptors: CD96 and CD226. These interactions accumulates at the cell-cell contact site, leading to the formation of a mature immunological synapse between NK cell and target cell. This may trigger adhesion and secretion of lytic granules and IFN-gamma and activate cytotoxicity of activated NK cells. May also promote NK cell-target cell modular exchange, and PVR transfer to the NK cell. This transfer is more important in some tumor cells expressing a lot of PVR, and may trigger fratricide NK cell activation, providing tumors with a mechanism of immunoevasion. Plays a role in mediating tumor cell invasion and migration.  
  
Can form trans-heterodimers with NECTIN3. The extracellular domain interacts with VTN, CD226 and CD96. The cytoplasmic domain interacts with DYNLT1. Binds with high affinity to TIGIT.  
  
**Gene Ontology Information:**

Molecular Function

- cell adhesion molecule binding
- signaling receptor activity
- virus receptor activity

Location

- adherens junction
- cell surface
- cytoplasm
- extracellular space
- focal adhesion
- integral component of membrane
- plasma membrane

Biological process

- adherens junction organization
- heterophilic cell-cell adhesion via plasma membrane cell adhesion molecules
- homophilic cell adhesion via plasma membrane adhesion molecules
- positive regulation of natural killer cell mediated cytotoxicity
- positive regulation of natural killer cell mediated cytotoxicity directed against tumor cell target
- regulation of immune response
- susceptibility to natural killer cell mediated cytotoxicity
- susceptibility to T cell mediated cytotoxicity

---

15

- **Protein name:** Guanine nucleotide-binding protein G(i) subunit alpha-1
- **Organism:** Bos taurus
- **Uniprot Accession Number:** P63097
- **Protein sequence length:** 354 aa
- **1D identity (%):** 5.67
- **1D identity (%) [Gaps excluded]:** 28.62
- **1D identity - Alignment Gaps:** 1089
- **Common reported functions (%):** 0.0
- **Common reported locations (%):** 0.0
- **Common reported processes (%):** 0.0

- **PDB ID:** 6K42
- **Chain:** A
- **Crystallized protein length:** 218 aa
- **Resolution:** 4.1 Å
- **Associated domain:** G-alpha
- **b-phipsi:** 0.11343
- **w-rdist:** 0.156468
- **t-alpha:** 0.045242
- **Chemical similarity (Tanimoto Index) (%):** 83.04
- **1D identity (%) [PDB]:** 0.08
- **1D identity (%) [Gaps excluded][PDB]:** 50.0
- **1D identity - Alignment Gaps [PDB]:** 1199
- **2D identity (%) [PDB]:** 10.88
- **2D identity (%) [Gaps excluded][PDB]:** 84.67
- **2D identity - Alignment Gaps [PDB]:** 929
- **3D similarity (TM-Score) (%) [PDB]:** 9.52

- **Gene name:** GNAI1
- **RefSeq ID:** N/A
- **Sequence length:** N/A
- **5-UTR|CDS|3-UTR identity (%):** N/A | N/A | N/A
- **5-UTR|CDS|3-UTR identity (%) [Gaps excluded]:** N/A | N/A | N/A
- **5-UTR|CDS|3-UTR identity [Alignment Gaps]:** N/A | N/A | N/A

**Uniprot Description:**  
  
Guanine nucleotide-binding proteins (G proteins) function as transducers downstream of G protein-coupled receptors (GPCRs) in numerous signaling cascades. The alpha chain contains the guanine nucleotide binding site and alternates between an active, GTP-bound state and an inactive, GDP-bound state. Signaling by an activated GPCR promotes GDP release and GTP binding. The alpha subunit has a low GTPase activity that converts bound GTP to GDP, thereby terminating the signal. Both GDP release and GTP hydrolysis are modulated by numerous regulatory proteins (By similarity). Signaling is mediated via effector proteins, such as adenylate cyclase. Inhibits adenylate cyclase activity, leading to decreased intracellular cAMP levels (By similarity). The inactive GDP-bound form prevents the association of RGS14 with centrosomes and is required for the translocation of RGS14 from the cytoplasm to the plasma membrane. Required for normal cytokinesis during mitosis (By similarity). Required for cortical dynein-dynactin complex recruitment during metaphase (By similarity).  
  
Heterotrimeric G proteins are composed of 3 units; alpha, beta and gamma. The alpha chain contains the guanine nucleotide binding site. Part of a spindle orientation complex at least composed of GNAI1, GPSM2 and NUMA1. Identified in complex with the beta subunit GNB1 and the gamma subunit GNG1. Identified in complex with the beta subunit GNB1 and the gamma subunit GNG2. GTP binding causes dissociation of the heterotrimer, liberating the individual subunits so that they can interact with downstream effector proteins. Interacts (GDP-bound form) with GPSM1; this inhibits guanine nucleotide exchange and GTP binding. Interacts (GDP-bound form) with GPSM2 (via GoLoco domains); this inhibits guanine nucleotide exchange. Interacts with RGS10; this strongly enhances GTP hydrolysis. Interacts with RGS1 and RGS16; this strongly enhances GTPase activity. Interacts with RGS4. Interacts with RGS12. Interacts (via active GTP- or inactive GDP-bound forms) with RGS14 (via RGS and GoLoco domains). Interacts with RGS3, RGS6, RGS7, RGS8, RGS17, RGS18 and RGS20 (in vitro). Interacts (GDP-bound form) with RIC8A (via C-terminus). Interacts (inactive GDP-bound form) with NUCB1 (via GBA motif); the interaction leads to activation of GNAI1 (By similarity). Interacts (inactive GDP-bound form) with CCDC88C/DAPLE (via GBA motif); the interaction leads to activation of GNAI1 (By similarity). Interacts (inactive GDP-bound form) with CCDC8A/GIV (via GBA motif) (By similarity).  
  
**Gene Ontology Information:**

Molecular Function

- G protein-coupled receptor binding
- G protein-coupled serotonin receptor binding
- G-protein beta/gamma-subunit complex binding
- GDP binding
- GTP binding
- GTPase activity
- magnesium ion binding

Location

- cell cortex region
- centrosome
- cytoplasm
- heterotrimeric G-protein complex
- midbody
- nucleus
- plasma membrane

Biological process

- adenylate cyclase-modulating G protein-coupled receptor signaling pathway
- cell cycle
- cell division
- cellular response to forskolin
- G protein-coupled receptor signaling pathway
- positive regulation of protein localization to cell cortex
- regulation of cAMP-mediated signaling
- regulation of mitotic spindle organization

---

16

- **Protein name:** Arginine/Ornithine decarboxylase
- **Organism:** Paramecium bursaria Chlorella virus 1
- **Uniprot Accession Number:** Q84527
- **Protein sequence length:** 372 aa
- **1D identity (%):** 6.34
- **1D identity (%) [Gaps excluded]:** 29.76
- **1D identity - Alignment Gaps:** 1067
- **Common reported functions (%):** 0.0
- **Common reported locations (%):** 0.0
- **Common reported processes (%):** 0.0

- **PDB ID:** 2NV9
- **Chain:** C
- **Crystallized protein length:** 367 aa
- **Resolution:** 1.95 Å
- **Associated domain:** Orn-Arg-deC-N
- **b-phipsi:** 0.10278
- **w-rdist:** 0.370972
- **t-alpha:** 0.004498
- **Chemical similarity (Tanimoto Index) (%):** 84.2
- **1D identity (%) [PDB]:** 0.75
- **1D identity (%) [Gaps excluded][PDB]:** 83.33
- **1D identity - Alignment Gaps [PDB]:** 1328
- **2D identity (%) [PDB]:** 16.92
- **2D identity (%) [Gaps excluded][PDB]:** 88.48
- **2D identity - Alignment Gaps [PDB]:** 918
- **3D similarity (TM-Score) (%) [PDB]:** 12.65

- **Gene name:** A207R
- **RefSeq ID:** NC\_000852
- **Genomic sequence length:** 330611
- **5-UTR|CDS|3-UTR identity (%):** N/A | 18.61 | N/A
- **5-UTR|CDS|3-UTR identity (%) [Gaps excluded]:** N/A | 77.78 | N/A
- **5-UTR|CDS|3-UTR identity [Alignment Gaps]:** N/A | 3033 | N/A

**Uniprot Description:**  
  
N/A  
  
**Gene Ontology Information:**

Molecular Function

- catalytic activity

Location  
  
N/A

Biological process

- polyamine biosynthetic process

---

17

- **Protein name:** Tumor susceptibility gene 101 protein
- **Organism:** Homo sapiens
- **Uniprot Accession Number:** Q99816
- **Protein sequence length:** 390 aa
- **1D identity (%):** 5.51
- **1D identity (%) [Gaps excluded]:** 26.76
- **1D identity - Alignment Gaps:** 1095
- **Common reported functions (%):** 0.0
- **Common reported locations (%):** 0.0
- **Common reported processes (%):** 0.0

- **PDB ID:** 4EJE
- **Chain:** B
- **Crystallized protein length:** 142 aa
- **Resolution:** 2.2 Å
- **Associated domain:** UEV
- **b-phipsi:** 0.066204
- **w-rdist:** 0.647906
- **t-alpha:** 0.004498
- **Chemical similarity (Tanimoto Index) (%):** 83.19
- **1D identity (%) [PDB]:** 0.09
- **1D identity (%) [Gaps excluded][PDB]:** 50.0
- **1D identity - Alignment Gaps [PDB]:** 1121
- **2D identity (%) [PDB]:** 7.53
- **2D identity (%) [Gaps excluded][PDB]:** 87.64
- **2D identity - Alignment Gaps [PDB]:** 947
- **3D similarity (TM-Score) (%) [PDB]:** 7.69

- **Gene name:** TSG101
- **RefSeq ID:** NM\_006292
- **Transcript sequence length:** 1534
- **5-UTR|CDS|3-UTR identity (%):** 22.48 | 21.13 | 32.23
- **5-UTR|CDS|3-UTR identity (%) [Gaps excluded]:** 72.04 | 80.54 | 81.06
- **5-UTR|CDS|3-UTR identity [Alignment Gaps]:** 205 | 2919 | 200

**Uniprot Description:**  
  
Component of the ESCRT-I complex, a regulator of vesicular trafficking process. Binds to ubiquitinated cargo proteins and is required for the sorting of endocytic ubiquitinated cargos into multivesicular bodies (MVBs). Mediates the association between the ESCRT-0 and ESCRT-I complex. Required for completion of cytokinesis; the function requires CEP55. May be involved in cell growth and differentiation. Acts as a negative growth regulator. Involved in the budding of many viruses through an interaction with viral proteins that contain a late-budding motif P-[ST]-A-P. This interaction is essential for viral particle budding of numerous retroviruses. Required for the exosomal release of SDCBP, CD63 and syndecan (PubMed:22660413). It may also play a role in the extracellular release of microvesicles that differ from the exosomes (PubMed:22315426).  
  
Component of the ESCRT-I complex (endosomal sorting complex required for transport I) which consists of TSG101, VPS28, a VPS37 protein (VPS37A to -D) and MVB12A or MVB12B in a 1:1:1:1 stoichiometry (PubMed:18005716). Interacts with VPS37A, VPS37B and VPS37C (PubMed:15218037, PubMed:15509564). Interacts with DMAP1 (PubMed:10888872). Interacts with ubiquitin (PubMed:11595185). Interacts with stathmin, GMCL and AATF (By similarity). Component of an ESCRT-I complex (endosomal sorting complex required for transport I) which consists of TSG101, VPS28, VPS37A and UBAP1 in a 1:1:1:1 stoichiometry (PubMed:21757351). Interacts with HGS; the interaction mediates the association with the ESCRT-0 complex. Interacts with GGA1 and GGA3 (PubMed:15143060, PubMed:15039775). Interacts (via UEV domain) with PDCD6IP/AIP1 (PubMed:14505570, PubMed:14519844). Interacts with VPS28, SNF8 and VPS36 (PubMed:14505570). Self-associates (PubMed:14505570, PubMed:14519844). Interacts with MVB12A; the association appears to be mediated by the TSG101-VPS37 binary subcomplex. Interacts with VPS37D. Interacts with LRSAM1. Interacts with CEP55; the interaction is required for cytokinesis but not for viral budding (PubMed:17853893). Interacts with PDCD6 (PubMed:18256029). Interacts with LITAF (PubMed:23166352). Interacts with MGRN1 (PubMed:17229889). Interacts with ARRDC1; recruits TSG101 to the plasma membrane (PubMed:21191027, PubMed:22315426).  
  
**Gene Ontology Information:**

Molecular Function

- calcium-dependent protein binding
- DNA binding
- nuclear receptor coactivator activity
- protein homodimerization activity
- protein-containing complex binding
- transcription corepressor activity
- ubiquitin binding
- ubiquitin protein ligase binding
- virion binding

Location

- cytoplasm
- cytosol
- early endosome
- early endosome membrane
- endosome
- endosome membrane
- ESCRT I complex
- extracellular exosome
- Flemming body
- host cell
- late endosome
- late endosome membrane
- microtubule organizing center
- multivesicular body
- nucleolus
- plasma membrane

Biological process

- autophagosome maturation
- cell cycle arrest
- cell division
- endosomal transport
- endosome to lysosome transport
- exosomal secretion
- extracellular transport
- intracellular transport of virus
- keratinocyte differentiation
- macroautophagy
- multivesicular body assembly
- negative regulation of cell population proliferation
- negative regulation of epidermal growth factor receptor signaling pathway
- negative regulation of epidermal growth factor-activated receptor activity
- negative regulation of transcription by RNA polymerase II
- positive regulation of exosomal secretion
- positive regulation of ubiquitin-dependent endocytosis
- positive regulation of viral budding via host ESCRT complex
- positive regulation of viral release from host cell
- protein monoubiquitination
- protein transport
- regulation of cell growth
- regulation of extracellular exosome assembly
- regulation of MAP kinase activity
- ubiquitin-dependent protein catabolic process via the multivesicular body sorting pathway
- viral budding
- viral budding via host ESCRT complex
- viral life cycle

---

18

- **Protein name:** H-2 class I histocompatibility antigen, D-B alpha chain
- **Organism:** Mus musculus
- **Uniprot Accession Number:** P01899
- **Protein sequence length:** 362 aa
- **1D identity (%):** 6.68
- **1D identity (%) [Gaps excluded]:** 26.2
- **1D identity - Alignment Gaps:** 971
- **Common reported functions (%):** 0.0
- **Common reported locations (%):** 0.0
- **Common reported processes (%):** 0.0

- **PDB ID:** 1S7W
- **Chain:** A
- **Crystallized protein length:** 276 aa
- **Resolution:** 2.4 Å
- **Associated domain:** Ig-like-C1-type
- **b-phipsi:** 0.001313
- **w-rdist:** 0.84382
- **t-alpha:** 0.220401
- **Chemical similarity (Tanimoto Index) (%):** 85.5
- **1D identity (%) [PDB]:** 0.0
- **1D identity (%) [Gaps excluded][PDB]:** 0.0
- **1D identity - Alignment Gaps [PDB]:** 1259
- **2D identity (%) [PDB]:** 18.23
- **2D identity (%) [Gaps excluded][PDB]:** 90.52
- **2D identity - Alignment Gaps [PDB]:** 837
- **3D similarity (TM-Score) (%) [PDB]:** 10.15

- **Gene name:** H2-D1
- **RefSeq ID:** N/A
- **Sequence length:** N/A
- **5-UTR|CDS|3-UTR identity (%):** N/A | N/A | N/A
- **5-UTR|CDS|3-UTR identity (%) [Gaps excluded]:** N/A | N/A | N/A
- **5-UTR|CDS|3-UTR identity [Alignment Gaps]:** N/A | N/A | N/A

**Uniprot Description:**  
  
Involved in the presentation of foreign antigens to the immune system.  
  
Heterodimer of an alpha chain and a beta chain (beta-2-microglobulin). Interacts with murid herpesvirus 4 protein K3 (mK3).  
  
**Gene Ontology Information:**

Molecular Function

- beta-2-microglobulin binding
- CD8 receptor binding
- peptide antigen binding
- peptide binding
- protein-containing complex binding
- signaling receptor binding
- T cell receptor binding
- TAP binding
- TAP complex binding

Location

- cell surface
- endoplasmic reticulum
- endoplasmic reticulum exit site
- external side of plasma membrane
- extracellular space
- Golgi apparatus
- Golgi medial cisterna
- integral component of lumenal side of endoplasmic reticulum membrane
- MHC class I peptide loading complex
- MHC class I protein complex
- phagocytic vesicle membrane
- plasma membrane

Biological process

- antigen processing and presentation of endogenous peptide antigen via MHC class I via ER pathway, TAP-dependent
- antigen processing and presentation of endogenous peptide antigen via MHC class Ib
- immune response
- negative regulation of neuron projection development
- positive regulation of T cell mediated cytotoxicity

---

19

- **Protein name:** Antigen peptide transporter 2
- **Organism:** Homo sapiens
- **Uniprot Accession Number:** Q03519
- **Protein sequence length:** 686 aa
- **1D identity (%):** 6.48
- **1D identity (%) [Gaps excluded]:** 26.42
- **1D identity - Alignment Gaps:** 1187
- **Common reported functions (%):** 0.0
- **Common reported locations (%):** 12.5
- **Common reported processes (%):** 0.0

- **PDB ID:** 5U1D
- **Chain:** B
- **Crystallized protein length:** 552 aa
- **Resolution:** 3.97 Å
- **Associated domain:** ABC-transporter
- **b-phipsi:** 0.05605
- **w-rdist:** 0.095087
- **t-alpha:** 0.332836
- **Chemical similarity (Tanimoto Index) (%):** 83.51
- **1D identity (%) [PDB]:** 0.07
- **1D identity (%) [Gaps excluded][PDB]:** 50.0
- **1D identity - Alignment Gaps [PDB]:** 1531
- **2D identity (%) [PDB]:** 18.15
- **2D identity (%) [Gaps excluded][PDB]:** 95.12
- **2D identity - Alignment Gaps [PDB]:** 1043
- **3D similarity (TM-Score) (%) [PDB]:** 15.99

- **Gene name:** TAP2
- **RefSeq ID:** NM\_018833
- **Transcript sequence length:** 2511
- **5-UTR|CDS|3-UTR identity (%):** 22.06 | 30.12 | 36.46
- **5-UTR|CDS|3-UTR identity (%) [Gaps excluded]:** 71.43 | 78.7 | 78.44
- **5-UTR|CDS|3-UTR identity [Alignment Gaps]:** 188 | 2582 | 251

**Uniprot Description:**  
  
ABC transporter associated with antigen processing. In complex with TAP1 mediates unidirectional translocation of peptide antigens from cytosol to endoplasmic reticulum (ER) for loading onto MHC class I (MHCI) molecules (PubMed:25656091, PubMed:25377891). Uses the chemical energy of ATP to export peptides against the concentration gradient (PubMed:25377891). During the transport cycle alternates between 'inward-facing' state with peptide binding site facing the cytosol to 'outward-facing' state with peptide binding site facing the ER lumen. Peptide antigen binding to ATP-loaded TAP1-TAP2 induces a switch to hydrolysis-competent 'outward-facing' conformation ready for peptide loading onto nascent MHCI molecules. Subsequently ATP hydrolysis resets the transporter to the 'inward facing' state for a new cycle (PubMed:25377891, PubMed:25656091, PubMed:11274390). Typically transports intracellular peptide antigens of 8 to 13 amino acids that arise from cytosolic proteolysis via IFNG-induced immunoproteasome. Binds peptides with free N- and C-termini, the first three and the C-terminal residues being critical. Preferentially selects peptides having a highly hydrophobic residue at position 3 and hydrophobic or charged residues at the C-terminal anchor. Proline at position 2 has the most destabilizing effect (PubMed:7500034, PubMed:9256420, PubMed:11274390). As a component of the peptide loading complex (PLC), acts as a molecular scaffold essential for peptide-MHCI assembly and antigen presentation (PubMed:26611325, PubMed:1538751, PubMed:25377891).  
  
Heterodimer of TAP1 and TAP2 (TAP1-TAP2) (PubMed:1538751). A component of the peptide loading complex (PLC), interacts via TAPBP with MHCI heterodimer; this interaction mediates peptide-MHCI assembly (PubMed:26611325). Recruits TAPBP in a 1:1 stoichiometry (PubMed:22638925). Interacts with classical MHCI such as HLA-A\*02-B2M; this interaction is obligatory for the loading of peptide epitopes (PubMed:8805302, PubMed:8630735). Interacts with non-classical MHCI molecules including HLA-E-B2M and HLA-F-B2M as well as PLC component CALR before the peptide loading (PubMed:9427624, PubMed:10605026).  
  
**Gene Ontology Information:**

Molecular Function

- ABC-type peptide antigen transporter activity
- ABC-type peptide transporter activity
- ATP binding
- ATPase-coupled transmembrane transporter activity
- metal ion binding
- MHC class Ib protein binding
- peptide antigen binding
- TAP1 binding
- tapasin binding

Location

- endoplasmic reticulum
- endoplasmic reticulum membrane
- endoplasmic reticulum-Golgi intermediate compartment membrane
- integral component of endoplasmic reticulum membrane
- integral component of membrane
- membrane
- MHC class I peptide loading complex
- nuclear speck
- phagocytic vesicle membrane
- TAP complex

Biological process

- adaptive immune response
- antigen processing and presentation of endogenous peptide antigen via MHC class I
- antigen processing and presentation of endogenous peptide antigen via MHC class Ib via ER pathway, TAP-dependent
- antigen processing and presentation of exogenous peptide antigen via MHC class I, TAP-dependent
- antigen processing and presentation of peptide antigen via MHC class I
- cytosol to endoplasmic reticulum transport
- peptide antigen transport
- peptide transport
- protein transport
- transmembrane transport
- vesicle fusion with endoplasmic reticulum-Golgi intermediate compartment (ERGIC) membrane
- viral process

---

20

- **Protein name:** RNA-directed RNA polymerase
- **Organism:** Infectious pancreatic necrosis virus (strain Jasper)
- **Uniprot Accession Number:** P22173
- **Protein sequence length:** 845 aa
- **1D identity (%):** 8.37
- **1D identity (%) [Gaps excluded]:** 32.49
- **1D identity - Alignment Gaps:** 1250
- **Common reported functions (%):** 0.0
- **Common reported locations (%):** 0.0
- **Common reported processes (%):** 0.0

- **PDB ID:** 2YI9
- **Chain:** D
- **Crystallized protein length:** 771 aa
- **Resolution:** 2.2 Å
- **Associated domain:** RdRp-catalytic
- **b-phipsi:** 0.062164
- **w-rdist:** 0.78729
- **t-alpha:** 0.001493
- **Chemical similarity (Tanimoto Index) (%):** 80.92
- **1D identity (%) [PDB]:** 0.0
- **1D identity (%) [Gaps excluded][PDB]:** 0.0
- **1D identity - Alignment Gaps [PDB]:** 1755
- **2D identity (%) [PDB]:** 25.77
- **2D identity (%) [Gaps excluded][PDB]:** 90.49
- **2D identity - Alignment Gaps [PDB]:** 977
- **3D similarity (TM-Score) (%) [PDB]:** 17.95

- **Gene name:** VP1
- **RefSeq ID:** NC\_001916
- **Genomic sequence length:** 2784
- **5-UTR|CDS|3-UTR identity (%):** N/A | 32.98 | N/A
- **5-UTR|CDS|3-UTR identity (%) [Gaps excluded]:** N/A | 78.12 | N/A
- **5-UTR|CDS|3-UTR identity [Alignment Gaps]:** N/A | 2584 | N/A

**Uniprot Description:**  
  
RNA-dependent RNA polymerase which is found both free and covalently attached to the genomic RNA. May also contain guanylyl and methyl transferase activities (By similarity).  
  
Interacts with VP3 in the cytoplasm.  
  
**Gene Ontology Information:**

Molecular Function

- GTP binding
- RNA-directed 5'-3' RNA polymerase activity

Location

- virion

Biological process

- RNA-protein covalent cross-linking
- viral genome replication

---

21

- **Protein name:** Coxsackievirus and adenovirus receptor
- **Organism:** Homo sapiens
- **Uniprot Accession Number:** P78310
- **Protein sequence length:** 365 aa
- **1D identity (%):** 6.0
- **1D identity (%) [Gaps excluded]:** 30.26
- **1D identity - Alignment Gaps:** 1096
- **Common reported functions (%):** 50.0
- **Common reported locations (%):** 0.0
- **Common reported processes (%):** 0.0

- **PDB ID:** 2W9L
- **Chain:** B
- **Crystallized protein length:** 117 aa
- **Resolution:** 2.91 Å
- **Associated domain:** Ig-like-C2-type-1
- **b-phipsi:** 0.001921
- **w-rdist:** 0.776533
- **t-alpha:** 0.326733
- **Chemical similarity (Tanimoto Index) (%):** 85.43
- **1D identity (%) [PDB]:** 0.09
- **1D identity (%) [Gaps excluded][PDB]:** 50.0
- **1D identity - Alignment Gaps [PDB]:** 1096
- **2D identity (%) [PDB]:** 7.94
- **2D identity (%) [Gaps excluded][PDB]:** 86.96
- **2D identity - Alignment Gaps [PDB]:** 916
- **3D similarity (TM-Score) (%) [PDB]:** 8.46

- **Gene name:** CXADR
- **RefSeq ID:** N/A
- **Sequence length:** N/A
- **5-UTR|CDS|3-UTR identity (%):** N/A | N/A | N/A
- **5-UTR|CDS|3-UTR identity (%) [Gaps excluded]:** N/A | N/A | N/A
- **5-UTR|CDS|3-UTR identity [Alignment Gaps]:** N/A | N/A | N/A

**Uniprot Description:**  
  
Component of the epithelial apical junction complex that may function as a homophilic cell adhesion molecule and is essential for tight junction integrity. Also involved in transepithelial migration of leukocytes through adhesive interactions with JAML a transmembrane protein of the plasma membrane of leukocytes. The interaction between both receptors also mediates the activation of gamma-delta T-cells, a subpopulation of T-cells residing in epithelia and involved in tissue homeostasis and repair. Upon epithelial CXADR-binding, JAML induces downstream cell signaling events in gamma-delta T-cells through PI3-kinase and MAP kinases. It results in proliferation and production of cytokines and growth factors by T-cells that in turn stimulate epithelial tissues repair.  
  
Monomer. May form homodimer. Interacts with LNX, MAGI1, DLG4, PRKCABP, TJP1 and CTNNB1. Interacts with MPDZ; recruits MPDZ to intercellular contact sites. Interacts with JAML (homodimeric form). Secreted isoform 3, isoform 4 and isoform 5 can interact with the extracellular domain of the receptor.  
  
**Gene Ontology Information:**

Molecular Function

- beta-catenin binding
- cell adhesion molecule binding
- cell adhesive protein binding involved in AV node cell-bundle of His cell communication
- connexin binding
- identical protein binding
- integrin binding
- PDZ domain binding
- signaling receptor binding
- virus receptor activity

Location

- acrosomal vesicle
- adherens junction
- apicolateral plasma membrane
- basolateral plasma membrane
- bicellular tight junction
- cell body
- cell junction
- cell-cell junction
- cytoplasm
- extracellular region
- extracellular space
- filopodium
- growth cone
- integral component of plasma membrane
- intercalated disc
- membrane raft
- neuromuscular junction
- neuron projection
- nucleoplasm
- plasma membrane
- protein-containing complex

Biological process

- actin cytoskeleton reorganization
- AV node cell to bundle of His cell communication
- AV node cell-bundle of His cell adhesion involved in cell communication
- cardiac muscle fiber development
- cell-cell junction organization
- defense response to virus
- epithelial structure maintenance
- gamma-delta T cell activation
- germ cell migration
- heart development
- heterophilic cell-cell adhesion via plasma membrane cell adhesion molecules
- homotypic cell-cell adhesion
- leukocyte migration
- mitochondrion organization
- neutrophil chemotaxis
- regulation of AV node cell action potential
- regulation of immune response
- transepithelial transport

---

22

- **Protein name:** Paired immunoglobulin-like type 2 receptor alpha
- **Organism:** Homo sapiens
- **Uniprot Accession Number:** Q9UKJ1
- **Protein sequence length:** 303 aa
- **1D identity (%):** 4.47
- **1D identity (%) [Gaps excluded]:** 32.8
- **1D identity - Alignment Gaps:** 1198
- **Common reported functions (%):** 0.0
- **Common reported locations (%):** 12.5
- **Common reported processes (%):** 0.0

- **PDB ID:** 5XO2
- **Chain:** A
- **Crystallized protein length:** 120 aa
- **Resolution:** 2.2 Å
- **Associated domain:** Ig-like-V-type
- **b-phipsi:** 0.002227
- **w-rdist:** 0.729961
- **t-alpha:** 0.285988
- **Chemical similarity (Tanimoto Index) (%):** 83.13
- **1D identity (%) [PDB]:** 0.0
- **1D identity (%) [Gaps excluded][PDB]:** 0.0
- **1D identity - Alignment Gaps [PDB]:** 1103
- **2D identity (%) [PDB]:** 8.86
- **2D identity (%) [Gaps excluded][PDB]:** 90.82
- **2D identity - Alignment Gaps [PDB]:** 907
- **3D similarity (TM-Score) (%) [PDB]:** 7.51

- **Gene name:** PILRA
- **RefSeq ID:** NM\_178273
- **Transcript sequence length:** 1018
- **5-UTR|CDS|3-UTR identity (%):** 32.15 | 9.29 | 37.53
- **5-UTR|CDS|3-UTR identity (%) [Gaps excluded]:** 80.65 | 81.76 | 79.55
- **5-UTR|CDS|3-UTR identity [Alignment Gaps]:** 187 | 3462 | 197

**Uniprot Description:**  
  
Paired receptors consist of highly related activating and inhibitory receptors and are widely involved in the regulation of the immune system. PILRA is thought to act as a cellular signaling inhibitory receptor by recruiting cytoplasmic phosphatases like PTPN6/SHP-1 and PTPN11/SHP-2 via their SH2 domains that block signal transduction through dephosphorylation of signaling molecules. Receptor for PIANP.  
  
Monomer. Interacts with PTPN6/SHP-1 and PTPN11/SHP-2 upon tyrosine phosphorylation.  
  
**Gene Ontology Information:**

Molecular Function

- MHC class I protein binding

Location

- extracellular exosome
- integral component of membrane
- plasma membrane

Biological process

- regulation of immune response
- signal transduction
- viral process

---

23

- **Protein name:** Tyrosine-protein kinase HCK
- **Organism:** Homo sapiens
- **Uniprot Accession Number:** P08631
- **Protein sequence length:** 526 aa
- **1D identity (%):** 0.45
- **1D identity (%) [Gaps excluded]:** 27.59
- **1D identity - Alignment Gaps:** 1741
- **Common reported functions (%):** 0.0
- **Common reported locations (%):** 0.0
- **Common reported processes (%):** 0.0

- **PDB ID:** 4U5W
- **Chain:** B
- **Crystallized protein length:** 163 aa
- **Resolution:** 1.86 Å
- **Associated domain:** SH2
- **b-phipsi:** 0.026222
- **w-rdist:** 0.82951
- **t-alpha:** 0.001495
- **Chemical similarity (Tanimoto Index) (%):** 83.13
- **1D identity (%) [PDB]:** 0.09
- **1D identity (%) [Gaps excluded][PDB]:** 50.0
- **1D identity - Alignment Gaps [PDB]:** 1143
- **2D identity (%) [PDB]:** 9.24
- **2D identity (%) [Gaps excluded][PDB]:** 88.89
- **2D identity - Alignment Gaps [PDB]:** 931
- **3D similarity (TM-Score) (%) [PDB]:** 8.08

- **Gene name:** HCK
- **RefSeq ID:** N/A
- **Sequence length:** N/A
- **5-UTR|CDS|3-UTR identity (%):** N/A | N/A | N/A
- **5-UTR|CDS|3-UTR identity (%) [Gaps excluded]:** N/A | N/A | N/A
- **5-UTR|CDS|3-UTR identity [Alignment Gaps]:** N/A | N/A | N/A

**Uniprot Description:**  
  
Non-receptor tyrosine-protein kinase found in hematopoietic cells that transmits signals from cell surface receptors and plays an important role in the regulation of innate immune responses, including neutrophil, monocyte, macrophage and mast cell functions, phagocytosis, cell survival and proliferation, cell adhesion and migration. Acts downstream of receptors that bind the Fc region of immunoglobulins, such as FCGR1A and FCGR2A, but also CSF3R, PLAUR, the receptors for IFNG, IL2, IL6 and IL8, and integrins, such as ITGB1 and ITGB2. During the phagocytic process, mediates mobilization of secretory lysosomes, degranulation, and activation of NADPH oxidase to bring about the respiratory burst. Plays a role in the release of inflammatory molecules. Promotes reorganization of the actin cytoskeleton and actin polymerization, formation of podosomes and cell protrusions. Inhibits TP73-mediated transcription activation and TP73-mediated apoptosis. Phosphorylates CBL in response to activation of immunoglobulin gamma Fc region receptors. Phosphorylates ADAM15, BCR, ELMO1, FCGR2A, GAB1, GAB2, RAPGEF1, STAT5B, TP73, VAV1 and WAS.  
  
Interacts (via SH2 domain) with FLT3 (tyrosine phosphorylated). Interacts with VAV1, WAS and RAPGEF1 (By similarity). This interaction stimulates its tyrosine-kinase activity. Interacts with ARRB1 and ARRB2. Interacts with ADAM15. Interacts with FASLG. Interacts with CBL. Interacts with FCGR1A; the interaction may be indirect. Interacts with IL6ST. Interacts (via SH3 domain) with ELMO1. Interacts (via SH3 domain) with TP73. Interacts with YAP1. Interacts with ABL1 and ITGB1, and thereby recruits ABL1 to activated ITGB1. Interacts (via SH3 domain) with WDCP.  
  
**Gene Ontology Information:**

Molecular Function

- ATP binding
- non-membrane spanning protein tyrosine kinase activity
- phosphotyrosine residue binding
- protein tyrosine kinase activity
- signaling receptor binding
- transmembrane receptor protein tyrosine kinase activity

Location

- caveola
- cell projection
- cytoskeleton
- cytosol
- extrinsic component of cytoplasmic side of plasma membrane
- focal adhesion
- Golgi apparatus
- intracellular membrane-bounded organelle
- lysosome
- nucleus
- plasma membrane
- transport vesicle

Biological process

- cell adhesion
- cell differentiation
- cytokine-mediated signaling pathway
- Fc-gamma receptor signaling pathway involved in phagocytosis
- inflammatory response
- innate immune response
- innate immune response-activating signal transduction
- integrin-mediated signaling pathway
- interferon-gamma-mediated signaling pathway
- leukocyte degranulation
- leukocyte migration involved in immune response
- lipopolysaccharide-mediated signaling pathway
- mesoderm development
- negative regulation of apoptotic process
- negative regulation of inflammatory response to antigenic stimulus
- peptidyl-tyrosine phosphorylation
- positive regulation of actin cytoskeleton reorganization
- positive regulation of actin filament polymerization
- positive regulation of cell population proliferation
- protein autophosphorylation
- protein phosphorylation
- regulation of cell shape
- regulation of defense response to virus by virus
- regulation of DNA-binding transcription factor activity
- regulation of inflammatory response
- regulation of phagocytosis
- regulation of podosome assembly
- respiratory burst after phagocytosis
- transmembrane receptor protein tyrosine kinase signaling pathway

---

24

- **Protein name:** Genome polyprotein
- **Organism:** Tobacco etch virus
- **Uniprot Accession Number:** P04517
- **Protein sequence length:** 3054 aa
- **1D identity (%):** 8.63
- **1D identity (%) [Gaps excluded]:** 27.83
- **1D identity - Alignment Gaps:** 2279
- **Common reported functions (%):** 0.0
- **Common reported locations (%):** 0.0
- **Common reported processes (%):** 0.0

- **PDB ID:** 1Q31
- **Chain:** B
- **Crystallized protein length:** 226 aa
- **Resolution:** 2.7 Å
- **Associated domain:** Peptidase-S30
- **b-phipsi:** 0.002374
- **w-rdist:** 0.722257
- **t-alpha:** 0.482301
- **Chemical similarity (Tanimoto Index) (%):** 85.86
- **1D identity (%) [PDB]:** 0.0
- **1D identity (%) [Gaps excluded][PDB]:** 0.0
- **1D identity - Alignment Gaps [PDB]:** 1210
- **2D identity (%) [PDB]:** 14.73
- **2D identity (%) [Gaps excluded][PDB]:** 89.47
- **2D identity - Alignment Gaps [PDB]:** 868
- **3D similarity (TM-Score) (%) [PDB]:** 7.86

- **Gene name:** N/A
- **RefSeq ID:** NC\_001555
- **Genomic sequence length:** 9494
- **5-UTR|CDS|3-UTR identity (%):** N/A | 28.55 | N/A
- **5-UTR|CDS|3-UTR identity (%) [Gaps excluded]:** N/A | 81.18 | N/A
- **5-UTR|CDS|3-UTR identity [Alignment Gaps]:** N/A | 6229 | N/A

**Uniprot Description:**  
  
Capsid protein
involved in aphid transmission, cell-to-cell and systemis movement, encapsidation of the viral RNA and in the regulation of viral RNA amplification.  
  
Nuclear inclusion protein A protease is a dimer; disulfide-linked.  
  
**Gene Ontology Information:**

Molecular Function

- ATP binding
- cysteine-type endopeptidase activity
- helicase activity
- hydrolase activity, acting on acid anhydrides, in phosphorus-containing anhydrides
- RNA binding
- RNA-directed 5'-3' RNA polymerase activity
- serine-type peptidase activity
- structural molecule activity

Location

- helical viral capsid

Biological process

- RNA-protein covalent cross-linking
- transcription, DNA-templated
- viral RNA genome replication

---

25

- **Protein name:** H-2 class I histocompatibility antigen, K-B alpha chain
- **Organism:** Mus musculus
- **Uniprot Accession Number:** P01901
- **Protein sequence length:** 369 aa
- **1D identity (%):** 6.17
- **1D identity (%) [Gaps excluded]:** 30.0
- **1D identity - Alignment Gaps:** 1082
- **Common reported functions (%):** 0.0
- **Common reported locations (%):** 0.0
- **Common reported processes (%):** 0.0

- **PDB ID:** 1S7T
- **Chain:** A
- **Crystallized protein length:** 276 aa
- **Resolution:** 2.3 Å
- **Associated domain:** Ig-like-C1-type
- **b-phipsi:** 0.001377
- **w-rdist:** 0.846433
- **t-alpha:** 0.384298
- **Chemical similarity (Tanimoto Index) (%):** 85.5
- **1D identity (%) [PDB]:** 0.0
- **1D identity (%) [Gaps excluded][PDB]:** 0.0
- **1D identity - Alignment Gaps [PDB]:** 1259
- **2D identity (%) [PDB]:** 17.46
- **2D identity (%) [Gaps excluded][PDB]:** 86.73
- **2D identity - Alignment Gaps [PDB]:** 837
- **3D similarity (TM-Score) (%) [PDB]:** 10.68

- **Gene name:** H2-K1
- **RefSeq ID:** N/A
- **Sequence length:** N/A
- **5-UTR|CDS|3-UTR identity (%):** N/A | N/A | N/A
- **5-UTR|CDS|3-UTR identity (%) [Gaps excluded]:** N/A | N/A | N/A
- **5-UTR|CDS|3-UTR identity [Alignment Gaps]:** N/A | N/A | N/A

**Uniprot Description:**  
  
Involved in the presentation of foreign antigens to the immune system.  
  
Heterodimer of an alpha chain and a beta chain (beta-2-microglobulin).  
  
**Gene Ontology Information:**

Molecular Function

- beta-2-microglobulin binding
- CD8 receptor binding
- peptide antigen binding
- peptide binding
- protein-containing complex binding
- signaling receptor binding
- T cell receptor binding
- TAP binding
- TAP complex binding

Location

- cell surface
- endoplasmic reticulum
- endoplasmic reticulum exit site
- external side of plasma membrane
- extracellular space
- Golgi apparatus
- Golgi medial cisterna
- integral component of lumenal side of endoplasmic reticulum membrane
- MHC class I peptide loading complex
- MHC class I protein complex
- phagocytic vesicle membrane
- plasma membrane

Biological process

- antigen processing and presentation of endogenous peptide antigen via MHC class I via ER pathway, TAP-dependent
- antigen processing and presentation of endogenous peptide antigen via MHC class Ib
- antigen processing and presentation of exogenous peptide antigen via MHC class I
- defense response to bacterium
- immune response
- inner ear development
- negative regulation of neuron projection development
- positive regulation of T cell mediated cytotoxicity

---

26

- **Protein name:** ATP-dependent DNA helicase uvsW
- **Organism:** Enterobacteria phage T4
- **Uniprot Accession Number:** P20703
- **Protein sequence length:** 587 aa
- **1D identity (%):** 8.79
- **1D identity (%) [Gaps excluded]:** 27.56
- **1D identity - Alignment Gaps:** 960
- **Common reported functions (%):** 0.0
- **Common reported locations (%):** 0.0
- **Common reported processes (%):** 0.0

- **PDB ID:** 1RIF
- **Chain:** A
- **Crystallized protein length:** 282 aa
- **Resolution:** 2.0 Å
- **Associated domain:** Helicase-ATP-binding
- **b-phipsi:** 0.115743
- **w-rdist:** 0.702891
- **t-alpha:** 0.002994
- **Chemical similarity (Tanimoto Index) (%):** 83.16
- **1D identity (%) [PDB]:** 0.0
- **1D identity (%) [Gaps excluded][PDB]:** 0.0
- **1D identity - Alignment Gaps [PDB]:** 1265
- **2D identity (%) [PDB]:** 13.18
- **2D identity (%) [Gaps excluded][PDB]:** 92.99
- **2D identity - Alignment Gaps [PDB]:** 951
- **3D similarity (TM-Score) (%) [PDB]:** 11.46

- **Gene name:** uvsW
- **RefSeq ID:** NC\_000866
- **Genomic sequence length:** 168903
- **5-UTR|CDS|3-UTR identity (%):** N/A | 32.04 | N/A
- **5-UTR|CDS|3-UTR identity (%) [Gaps excluded]:** N/A | 79.81 | N/A
- **5-UTR|CDS|3-UTR identity [Alignment Gaps]:** N/A | 2386 | N/A

**Uniprot Description:**  
  
Plays important roles in recombination-dependent DNA repair and the reorganization of stalled replication forks during viral DNA synthesis.  
  
Interacts with gp32.  
  
**Gene Ontology Information:**

Molecular Function

- ATP binding
- DNA binding
- DNA helicase activity
- helicase activity

Location  
  
N/A

Biological process  
  
N/A

---

27

- **Protein name:** CMRF35-like molecule 1
- **Organism:** Mus musculus
- **Uniprot Accession Number:** Q6SJQ7
- **Protein sequence length:** 337 aa
- **1D identity (%):** 1.87
- **1D identity (%) [Gaps excluded]:** 25.69
- **1D identity - Alignment Gaps:** 1392
- **Common reported functions (%):** 0.0
- **Common reported locations (%):** 12.5
- **Common reported processes (%):** 0.0

- **PDB ID:** 6H6L
- **Chain:** G
- **Crystallized protein length:** 88 aa
- **Resolution:** 2.5 Å
- **Associated domain:** Ig-like-V-type
- **b-phipsi:** 0.002628
- **w-rdist:** 0.929852
- **t-alpha:** 0.089431
- **Chemical similarity (Tanimoto Index) (%):** 78.08
- **1D identity (%) [PDB]:** 0.09
- **1D identity (%) [Gaps excluded][PDB]:** 50.0
- **1D identity - Alignment Gaps [PDB]:** 1070
- **2D identity (%) [PDB]:** 6.2
- **2D identity (%) [Gaps excluded][PDB]:** 83.78
- **2D identity - Alignment Gaps [PDB]:** 926
- **3D similarity (TM-Score) (%) [PDB]:** 6.47

- **Gene name:** Cd300lf
- **RefSeq ID:** N/A
- **Sequence length:** N/A
- **5-UTR|CDS|3-UTR identity (%):** N/A | N/A | N/A
- **5-UTR|CDS|3-UTR identity (%) [Gaps excluded]:** N/A | N/A | N/A
- **5-UTR|CDS|3-UTR identity [Alignment Gaps]:** N/A | N/A | N/A

**Uniprot Description:**  
  
Acts as an inhibitory receptor for myeloid cells and mast cells (PubMed:17438331). Positively regulates the phagocytosis of apoptotic cells (efferocytosis) via phosphatidylserine (PS) recognition; recognizes and binds PS as a ligand which is expressed on the surface of apoptotic cells (PubMed:21865548). Plays an important role in the maintenance of immune homeostasis, by promoting macrophage-mediated efferocytosis and by inhibiting dendritic cell-mediated efferocytosis (PubMed:26768664). Negatively regulates Fc epsilon receptor-dependent mast cell activation and allergic responses via binding to ceramide which acts as a ligand (PubMed:23123064). May act as a coreceptor for interleukin 4 (IL-4). Associates with and regulates IL-4 receptor alpha-mediated responses by augmenting IL-4- and IL-13-induced signaling (PubMed:26124135). Negatively regulates the Toll-like receptor (TLR) signaling mediated by MYD88 and TRIF through activation of PTPN6/SHP-1 and PTPN11/SHP-2 (By similarity). Inhibits osteoclast formation (PubMed:14662855). Induces macrophage cell death upon engagement (PubMed:18097021).  
  
Interacts with PTPN6/SHP-1 in a tyrosine phosphorylation dependent manner (PubMed:14662855). Interacts with IL4R (PubMed:26124135).  
  
**Gene Ontology Information:**

Molecular Function

- ceramide binding
- interleukin-4 receptor binding
- phosphatidylserine binding
- transmembrane signaling receptor activity
- virus receptor activity

Location

- integral component of membrane
- plasma membrane

Biological process

- interleukin-13-mediated signaling pathway
- negative regulation of apoptotic cell clearance
- negative regulation of mast cell activation
- negative regulation of MyD88-dependent toll-like receptor signaling pathway
- osteoclast differentiation
- positive regulation of apoptotic cell clearance
- positive regulation of interleukin-4-mediated signaling pathway
- TRIF-dependent toll-like receptor signaling pathway

---

28

- **Protein name:** HLA class I histocompatibility antigen, B alpha chain
- **Organism:** Homo sapiens
- **Uniprot Accession Number:** P01889
- **Protein sequence length:** 362 aa
- **1D identity (%):** 5.07
- **1D identity (%) [Gaps excluded]:** 25.27
- **1D identity - Alignment Gaps:** 1089
- **Common reported functions (%):** 0.0
- **Common reported locations (%):** 0.0
- **Common reported processes (%):** 0.0

- **PDB ID:** 3MV9
- **Chain:** A
- **Crystallized protein length:** 276 aa
- **Resolution:** 2.7 Å
- **Associated domain:** Ig-like-C1-type
- **b-phipsi:** 0.001967
- **w-rdist:** 0.833237
- **t-alpha:** 1.189542
- **Chemical similarity (Tanimoto Index) (%):** 86.01
- **1D identity (%) [PDB]:** 0.08
- **1D identity (%) [Gaps excluded][PDB]:** 50.0
- **1D identity - Alignment Gaps [PDB]:** 1255
- **2D identity (%) [PDB]:** 15.55
- **2D identity (%) [Gaps excluded][PDB]:** 90.27
- **2D identity - Alignment Gaps [PDB]:** 889
- **3D similarity (TM-Score) (%) [PDB]:** 11.82

- **Gene name:** HLA-B
- **RefSeq ID:** NM\_005514
- **Transcript sequence length:** 1536
- **5-UTR|CDS|3-UTR identity (%):** 6.79 | 16.87 | 33.71
- **5-UTR|CDS|3-UTR identity (%) [Gaps excluded]:** 85.71 | 75.47 | 72.95
- **5-UTR|CDS|3-UTR identity [Alignment Gaps]:** 244 | 3117 | 241

**Uniprot Description:**  
  
Antigen-presenting major histocompatibility complex class I (MHCI) molecule. In complex with B2M/beta 2 microglobulin displays primarily viral and tumor-derived peptides on antigen-presenting cells for recognition by alpha-beta T cell receptor (TCR) on HLA-B-restricted CD8-positive T cells, guiding antigen-specific T cell immune response to eliminate infected or transformed cells (PubMed:25808313, PubMed:29531227, PubMed:9620674, PubMed:23209413). May also present self-peptides derived from the signal sequence of secreted or membrane proteins, although T cells specific for these peptides are usually inactivated to prevent autoreactivity (PubMed:7743181, PubMed:18991276). Both the peptide and the MHC molecule are recognized by TCR, the peptide is responsible for the fine specificity of antigen recognition and MHC residues account for the MHC restriction of T cells (PubMed:29531227, PubMed:9620674, PubMed:24600035). Typically presents intracellular peptide antigens of 8 to 13 amino acids that arise from cytosolic proteolysis via constitutive proteasome and IFNG-induced immunoproteasome (PubMed:23209413). Can bind different peptides containing allele-specific binding motifs, which are mainly defined by anchor residues at position 2 and 9 (PubMed:25808313, PubMed:29531227).  
  
Heterotrimer that consists of an alpha chain HLA-B, a beta chain B2M and a peptide (peptide-HLA-B-B2M) (PubMed:25808313, PubMed:29531227, PubMed:15657948, PubMed:17057332, PubMed:22020283, PubMed:24600035). Early in biogenesis, HLA-B-B2M dimer interacts with the components of the peptide-loading complex composed of TAPBP, TAP1-TAP2, TAPBPL, PDIA3/ERP57 and CALR (PubMed:9036970, PubMed:9620674, PubMed:26439010, PubMed:26416272). Interacts with TAP1-TAP2 transporter via TAPBP; this interaction is obligatory for the loading of peptide epitopes delivered to the ER by TAP1-TAP2 transporter (PubMed:9036970, PubMed:9620674). Interacts with TAPBPL; TAPBPL binds peptide-free HLA-B-B2M complexes or those loaded with low affinity peptides, likely facilitating peptide exchange for higher affinity peptides (PubMed:26439010). Only optimally assembled peptide-HLA-B-B2M trimer translocates to the surface of antigen-presenting cells, where it interacts with TCR and CD8 coreceptor on the surface of T cells. HLA-B (via polymorphic alpha-1 and alpha-2 domains) interacts with antigen-specific TCR (via CDR1, CDR2 and CDR3 domains) (PubMed:29531227, PubMed:24600035). One HLA-B molecule (mainly via nonpolymorphic alpha-3 domain) interacts with one CD8A homodimer (via CDR-like loop); this interaction insures peptide-HLA-B-B2M recognition by CD8-positive T cells only (PubMed:29531227). Allele B\*57:01 interacts (via Bw4 motif) with KIR3DL1 (via Ig-like C2-type domain); this interaction may interfere with peptide binding (PubMed:22020283, PubMed:25480565). Allele B\*46:01 interacts with KIR2DL3 (PubMed:28514659).  
  
**Gene Ontology Information:**

Molecular Function

- chaperone binding
- peptide antigen binding
- signaling receptor binding
- TAP binding

Location

- cell surface
- early endosome membrane
- endoplasmic reticulum
- ER to Golgi transport vesicle membrane
- extracellular exosome
- Golgi apparatus
- Golgi membrane
- integral component of lumenal side of endoplasmic reticulum membrane
- integral component of plasma membrane
- membrane
- MHC class I protein complex
- phagocytic vesicle membrane
- plasma membrane
- recycling endosome membrane
- secretory granule membrane

Biological process

- adaptive immune response
- antigen processing and presentation of endogenous peptide antigen via MHC class I via ER pathway, TAP-independent
- antigen processing and presentation of exogenous peptide antigen via MHC class I, TAP-dependent
- antigen processing and presentation of exogenous peptide antigen via MHC class I, TAP-independent
- antigen processing and presentation of peptide antigen via MHC class I
- defense response
- detection of bacterium
- immune response
- interferon-gamma-mediated signaling pathway
- neutrophil degranulation
- positive regulation of T cell mediated cytotoxicity
- protection from natural killer cell mediated cytotoxicity
- regulation of dendritic cell differentiation
- regulation of immune response
- regulation of interleukin-12 production
- regulation of interleukin-6 production
- regulation of T cell anergy
- type I interferon signaling pathway
- viral process

---

29

- **Protein name:** Nectin-4
- **Organism:** Homo sapiens
- **Uniprot Accession Number:** Q96NY8
- **Protein sequence length:** 510 aa
- **1D identity (%):** 8.65
- **1D identity (%) [Gaps excluded]:** 27.15
- **1D identity - Alignment Gaps:** 921
- **Common reported functions (%):** 50.0
- **Common reported locations (%):** 12.5
- **Common reported processes (%):** 0.0

- **PDB ID:** 4GJT
- **Chain:** B
- **Crystallized protein length:** 114 aa
- **Resolution:** 3.1 Å
- **Associated domain:** Ig-like-V-type
- **b-phipsi:** 0.002476
- **w-rdist:** 0.851564
- **t-alpha:** 0.283525
- **Chemical similarity (Tanimoto Index) (%):** N/A
- **1D identity (%) [PDB]:** 0.0
- **1D identity (%) [Gaps excluded][PDB]:** 0.0
- **1D identity - Alignment Gaps [PDB]:** 1097
- **2D identity (%) [PDB]:** 7.08
- **2D identity (%) [Gaps excluded][PDB]:** 90.0
- **2D identity - Alignment Gaps [PDB]:** 937
- **3D similarity (TM-Score) (%) [PDB]:** 11.14

- **Gene name:** NECTIN4
- **RefSeq ID:** NM\_030916
- **Transcript sequence length:** 3458
- **5-UTR|CDS|3-UTR identity (%):** 38.64 | 23.79 | 8.18
- **5-UTR|CDS|3-UTR identity (%) [Gaps excluded]:** 77.71 | 76.54 | 77.78
- **5-UTR|CDS|3-UTR identity [Alignment Gaps]:** 177 | 2815 | 1532

**Uniprot Description:**  
  
Seems to be involved in cell adhesion through trans-homophilic and -heterophilic interactions, the latter including specifically interactions with NECTIN1. Does not act as receptor for alpha-herpesvirus entry into cells.  
  
Self-associates. Interacts via its Ig-like V-type domain with NECTIN1 Ig-like V-type domain. Interacts via its C-terminus with AFDN.  
  
**Gene Ontology Information:**

Molecular Function

- identical protein binding
- virus receptor activity

Location

- adherens junction
- extracellular exosome
- integral component of membrane
- plasma membrane

Biological process

- adherens junction organization
- heterophilic cell-cell adhesion via plasma membrane cell adhesion molecules
- homophilic cell adhesion via plasma membrane adhesion molecules

---

30

- **Protein name:** Tyrosine-protein kinase Fyn
- **Organism:** Homo sapiens
- **Uniprot Accession Number:** P06241
- **Protein sequence length:** 537 aa
- **1D identity (%):** 8.7
- **1D identity (%) [Gaps excluded]:** 27.91
- **1D identity - Alignment Gaps:** 950
- **Common reported functions (%):** 50.0
- **Common reported locations (%):** 0.0
- **Common reported processes (%):** 0.0

- **PDB ID:** 4D8D
- **Chain:** A
- **Crystallized protein length:** 57 aa
- **Resolution:** 2.52 Å
- **Associated domain:** SH3
- **b-phipsi:** 0.002564
- **w-rdist:** 1.037969
- **t-alpha:** 0.133672
- **Chemical similarity (Tanimoto Index) (%):** 79.77
- **1D identity (%) [PDB]:** 0.0
- **1D identity (%) [Gaps excluded][PDB]:** 0.0
- **1D identity - Alignment Gaps [PDB]:** 1040
- **2D identity (%) [PDB]:** 4.15
- **2D identity (%) [Gaps excluded][PDB]:** 80.39
- **2D identity - Alignment Gaps [PDB]:** 938
- **3D similarity (TM-Score) (%) [PDB]:** 3.52

- **Gene name:** FYN
- **RefSeq ID:** N/A
- **Sequence length:** N/A
- **5-UTR|CDS|3-UTR identity (%):** N/A | N/A | N/A
- **5-UTR|CDS|3-UTR identity (%) [Gaps excluded]:** N/A | N/A | N/A
- **5-UTR|CDS|3-UTR identity [Alignment Gaps]:** N/A | N/A | N/A

**Uniprot Description:**  
  
Non-receptor tyrosine-protein kinase that plays a role in many biological processes including regulation of cell growth and survival, cell adhesion, integrin-mediated signaling, cytoskeletal remodeling, cell motility, immune response and axon guidance. Inactive FYN is phosphorylated on its C-terminal tail within the catalytic domain. Following activation by PKA, the protein subsequently associates with PTK2/FAK1, allowing PTK2/FAK1 phosphorylation, activation and targeting to focal adhesions. Involved in the regulation of cell adhesion and motility through phosphorylation of CTNNB1 (beta-catenin) and CTNND1 (delta-catenin). Regulates cytoskeletal remodeling by phosphorylating several proteins including the actin regulator WAS and the microtubule-associated proteins MAP2 and MAPT. Promotes cell survival by phosphorylating AGAP2/PIKE-A and preventing its apoptotic cleavage. Participates in signal transduction pathways that regulate the integrity of the glomerular slit diaphragm (an essential part of the glomerular filter of the kidney) by phosphorylating several slit diaphragm components including NPHS1, KIRREL1 and TRPC6. Plays a role in neural processes by phosphorylating DPYSL2, a multifunctional adapter protein within the central nervous system, ARHGAP32, a regulator for Rho family GTPases implicated in various neural functions, and SNCA, a small pre-synaptic protein. Participates in the downstream signaling pathways that lead to T-cell differentiation and proliferation following T-cell receptor (TCR) stimulation. Phosphorylates PTK2B/PYK2 in response to T-cell receptor activation. Also participates in negative feedback regulation of TCR signaling through phosphorylation of PAG1, thereby promoting interaction between PAG1 and CSK and recruitment of CSK to lipid rafts. CSK maintains LCK and FYN in an inactive form. Promotes CD28-induced phosphorylation of VAV1. In mast cells, phosphorylates CLNK after activation of immunoglobulin epsilon receptor signaling (By similarity).  
  
Interacts (via its SH3 domain) with PIK3R1 and PRMT8. Interacts with FYB1, PAG1, and SH2D1A. Interacts with CD79A (tyrosine-phosphorylated form); the interaction increases FYN activity. Interacts (via SH2 domain) with CSF1R (tyrosine phosphorylated) (By similarity). Interacts with TOM1L1 (phosphorylated form). Interacts with KDR (tyrosine phosphorylated). Interacts (via SH3 domain) with KLHL2 (via N-terminus) (By similarity). Interacts with SH2D1A and SLAMF1. Interacts with ITCH; the interaction phosphorylates ITCH and negatively regulates its activity. Interacts with FASLG. Interacts with RUNX3. Interacts with KIT. Interacts with EPHA8; possible downstream effector of EPHA8 in regulation of cell adhesion. Interacts with PTK2/FAK1; this interaction leads to PTK2/FAK1 phosphorylation and activation. Interacts with CAV1; this interaction couples integrins to the Ras-ERK pathway. Interacts with UNC119. Interacts (via SH2 domain) with PTPRH (phosphorylated form) (By similarity). Interacts with PTPRO (phosphorylated form) (By similarity). Interacts with PTPRB (phosphorylated form) (By similarity). Interacts with FYB2 (PubMed:27335501). Interacts with DSCAM (By similarity). Interacts with SKAP1 and FYB1; this interaction promotes the phosphorylation of CLNK (By similarity).  
  
**Gene Ontology Information:**

Molecular Function

- alpha-tubulin binding
- ATP binding
- CD4 receptor binding
- CD8 receptor binding
- disordered domain specific binding
- enzyme binding
- ephrin receptor binding
- growth factor receptor binding
- identical protein binding
- ion channel binding
- metal ion binding
- non-membrane spanning protein tyrosine kinase activity
- peptide hormone receptor binding
- phosphatidylinositol 3-kinase binding
- phospholipase activator activity
- phospholipase binding
- protein tyrosine kinase activity
- signaling receptor binding
- T cell receptor binding
- tau protein binding
- tau-protein kinase activity
- transmembrane receptor protein tyrosine kinase activity
- type 5 metabotropic glutamate receptor binding

Location

- actin filament
- cell body
- cytosol
- dendrite
- endosome
- extrinsic component of cytoplasmic side of plasma membrane
- glial cell projection
- glutamatergic synapse
- membrane raft
- mitochondrion
- nucleus
- perinuclear endoplasmic reticulum
- perinuclear region of cytoplasm
- plasma membrane
- postsynaptic density
- postsynaptic density, intracellular component
- Schaffer collateral - CA1 synapse

Biological process

- activated T cell proliferation
- adaptive immune response
- axon guidance
- blood coagulation
- calcium ion transport
- cell differentiation
- cellular response to amyloid-beta
- cellular response to glycine
- cellular response to L-glutamate
- cellular response to peptide hormone stimulus
- cellular response to platelet-derived growth factor stimulus
- cellular response to transforming growth factor beta stimulus
- cytokine-mediated signaling pathway
- dendrite morphogenesis
- dendritic spine maintenance
- detection of mechanical stimulus involved in sensory perception of pain
- ephrin receptor signaling pathway
- Fc-gamma receptor signaling pathway involved in phagocytosis
- feeding behavior
- forebrain development
- heart process
- innate immune response
- intracellular signal transduction
- learning
- leukocyte migration
- MAPK cascade
- modulation of chemical synaptic transmission
- negative regulation of dendritic spine maintenance
- negative regulation of extrinsic apoptotic signaling pathway in absence of ligand
- negative regulation of gene expression
- negative regulation of hydrogen peroxide biosynthetic process
- negative regulation of inflammatory response to antigenic stimulus
- negative regulation of neuron apoptotic process
- negative regulation of oxidative stress-induced cell death
- negative regulation of protein catabolic process
- negative regulation of protein ubiquitination
- neuron migration
- peptidyl-tyrosine phosphorylation
- platelet activation
- positive regulation of cysteine-type endopeptidase activity
- positive regulation of I-kappaB kinase/NF-kappaB signaling
- positive regulation of neuron death
- positive regulation of neuron projection development
- positive regulation of non-membrane spanning protein tyrosine kinase activity
- positive regulation of phosphatidylinositol 3-kinase signaling
- positive regulation of protein kinase B signaling
- positive regulation of protein localization to membrane
- positive regulation of protein localization to nucleus
- positive regulation of protein targeting to membrane
- positive regulation of tyrosine phosphorylation of STAT protein
- protein autophosphorylation
- protein phosphorylation
- regulation of calcium ion import across plasma membrane
- regulation of cell shape
- regulation of defense response to virus by virus
- regulation of glutamate receptor signaling pathway
- regulation of peptidyl-tyrosine phosphorylation
- response to amyloid-beta
- response to drug
- response to ethanol
- response to hydrogen peroxide
- response to singlet oxygen
- stimulatory C-type lectin receptor signaling pathway
- T cell costimulation
- T cell receptor signaling pathway
- transmembrane receptor protein tyrosine kinase signaling pathway
- vascular endothelial growth factor receptor signaling pathway

---

31

- **Protein name:** HLA class I histocompatibility antigen, A alpha chain
- **Organism:** Homo sapiens
- **Uniprot Accession Number:** P04439
- **Protein sequence length:** 365 aa
- **1D identity (%):** 6.03
- **1D identity (%) [Gaps excluded]:** 24.09
- **1D identity - Alignment Gaps:** 982
- **Common reported functions (%):** 0.0
- **Common reported locations (%):** 0.0
- **Common reported processes (%):** 0.0

- **PDB ID:** 1B0R
- **Chain:** A
- **Crystallized protein length:** 269 aa
- **Resolution:** 2.9 Å
- **Associated domain:** Ig-like-C1-type
- **b-phipsi:** 0.002005
- **w-rdist:** 0.866632
- **t-alpha:** 0.332008
- **Chemical similarity (Tanimoto Index) (%):** 85.31
- **1D identity (%) [PDB]:** 0.08
- **1D identity (%) [Gaps excluded][PDB]:** 50.0
- **1D identity - Alignment Gaps [PDB]:** 1249
- **2D identity (%) [PDB]:** 17.68
- **2D identity (%) [Gaps excluded][PDB]:** 86.79
- **2D identity - Alignment Gaps [PDB]:** 829
- **3D similarity (TM-Score) (%) [PDB]:** 10.81

- **Gene name:** HLA-A
- **RefSeq ID:** NM\_002116
- **Transcript sequence length:** 1535
- **5-UTR|CDS|3-UTR identity (%):** 4.4 | 17.53 | 35.36
- **5-UTR|CDS|3-UTR identity (%) [Gaps excluded]:** 85.71 | 75.0 | 78.5
- **5-UTR|CDS|3-UTR identity [Alignment Gaps]:** 259 | 3056 | 244

**Uniprot Description:**  
  
Antigen-presenting major histocompatibility complex class I (MHCI) molecule. In complex with B2M/beta 2 microglobulin displays primarily viral and tumor-derived peptides on antigen-presenting cells for recognition by alpha-beta T cell receptor (TCR) on HLA-A-restricted CD8-positive T cells, guiding antigen-specific T cell immune response to eliminate infected or transformed cells (PubMed:2456340, PubMed:2784196, PubMed:1402688, PubMed:7504010, PubMed:9862734, PubMed:10449296, PubMed:12138174, PubMed:12393434, PubMed:15893615, PubMed:17189421, PubMed:19543285, PubMed:21498667, PubMed:24192765, PubMed:7694806, PubMed:24395804, PubMed:28250417). May also present self-peptides derived from the signal sequence of secreted or membrane proteins, although T cells specific for these peptides are usually inactivated to prevent autoreactivity (PubMed:25880248, PubMed:7506728, PubMed:7679507). Both the peptide and the MHC molecule are recognized by TCR, the peptide is responsible for the fine specificity of antigen recognition and MHC residues account for the MHC restriction of T cells (PubMed:12796775, PubMed:18275829, PubMed:19542454, PubMed:28250417). Typically presents intracellular peptide antigens of 8 to 13 amino acids that arise from cytosolic proteolysis via IFNG-induced immunoproteasome or via endopeptidase IDE/insulin-degrading enzyme (PubMed:17189421, PubMed:20364150, PubMed:17079320, PubMed:26929325, PubMed:27049119). Can bind different peptides containing allele-specific binding motifs, which are mainly defined by anchor residues at position 2 and 9 (PubMed:7504010, PubMed:9862734).  
  
Heterotrimer that consists of an alpha chain HLA-A, a beta chain B2M and a peptide (peptide-HLA-A-B2M) (PubMed:7504010, PubMed:7679507, PubMed:21943705, PubMed:19177349, PubMed:24395804, PubMed:26758806, PubMed:7504010, PubMed:7506728, PubMed:8805302, PubMed:7694806, PubMed:7935798, PubMed:9177355, PubMed:18275829, PubMed:22245737, PubMed:28250417, PubMed:11502003, PubMed:8906788, PubMed:19542454). Early in biogenesis, HLA-A-B2M dimer interacts with the components of the peptide-loading complex composed of TAPBP, TAP1-TAP2, TAPBPL, PDIA3/ERP57 and CALR (PubMed:21263072). Interacts with TAP1-TAP2 transporter via TAPBP; this interaction is obligatory for the loading of peptide epitopes delivered to the ER by TAP1-TAP2 transporter (PubMed:8805302, PubMed:8630735, PubMed:21263072). Interacts with TAPBPL; TAPBPL binds peptide-free HLA-A-B2M complexes or those loaded with low affinity peptides, likely facilitating peptide exchange for higher affinity peptides (PubMed:26869717). Only optimally assembled peptide-HLA-B2M trimer translocates to the surface of antigen-presenting cells, where it interacts with TCR and CD8 coreceptor on the surface of T cells. HLA-A (via polymorphic alpha-1 and alpha-2 domains) interacts with antigen-specific TCR (via CDR3 domains) (PubMed:22245737, PubMed:12796775, PubMed:18275829). One HLA-A molecule (mainly via nonpolymorphic alpha-3 domain) interacts with one CD8A homodimer (via CDR-like loop); this interaction insures peptide-HLA-A-B2M recognition by CD8-positive T cells only (PubMed:9177355, PubMed:2784196). Alleles A\*23:01; A\*24:02 and A\*32:01 interact (via Bw4 motif) with KIR3DL1 on NK cells; this interaction is direct.  
  
**Gene Ontology Information:**

Molecular Function

- beta-2-microglobulin binding
- CD8 receptor binding
- peptide antigen binding
- RNA binding
- signaling receptor binding
- T cell receptor binding
- TAP binding
- TAP complex binding

Location

- cell surface
- early endosome membrane
- endoplasmic reticulum
- endoplasmic reticulum exit site
- endoplasmic reticulum membrane
- ER to Golgi transport vesicle membrane
- extracellular exosome
- Golgi apparatus
- Golgi medial cisterna
- Golgi membrane
- integral component of lumenal side of endoplasmic reticulum membrane
- integral component of plasma membrane
- membrane
- MHC class I peptide loading complex
- MHC class I protein complex
- phagocytic vesicle membrane
- plasma membrane
- recycling endosome membrane

Biological process

- antibacterial humoral response
- antigen processing and presentation of endogenous peptide antigen via MHC class I
- antigen processing and presentation of endogenous peptide antigen via MHC class I via ER pathway, TAP-dependent
- antigen processing and presentation of endogenous peptide antigen via MHC class I via ER pathway, TAP-independent
- antigen processing and presentation of exogenous peptide antigen via MHC class I
- antigen processing and presentation of exogenous peptide antigen via MHC class I, TAP-dependent
- antigen processing and presentation of exogenous peptide antigen via MHC class I, TAP-independent
- antigen processing and presentation of peptide antigen via MHC class I
- CD8-positive, alpha-beta T cell activation
- defense response to Gram-positive bacterium
- detection of bacterium
- immune response
- interferon-gamma-mediated signaling pathway
- positive regulation of CD8-positive, alpha-beta T cell activation
- positive regulation of CD8-positive, alpha-beta T cell proliferation
- positive regulation of interferon-gamma production
- positive regulation of memory T cell activation
- positive regulation of T cell cytokine production
- positive regulation of T cell mediated cytotoxicity
- protection from natural killer cell mediated cytotoxicity
- protein ubiquitination
- regulation of immune response
- T cell mediated cytotoxicity
- T cell mediated cytotoxicity directed against tumor cell target
- T cell receptor signaling pathway
- type I interferon signaling pathway
- viral process

---

32

- **Protein name:** Viral CASP8 and FADD-like apoptosis regulator
- **Organism:** Molluscum contagiosum virus subtype 1
- **Uniprot Accession Number:** Q98325
- **Protein sequence length:** 241 aa
- **1D identity (%):** 4.26
- **1D identity (%) [Gaps excluded]:** 24.66
- **1D identity - Alignment Gaps:** 1068
- **Common reported functions (%):** 0.0
- **Common reported locations (%):** 0.0
- **Common reported processes (%):** 0.0

- **PDB ID:** 2BBZ
- **Chain:** D
- **Crystallized protein length:** 190 aa
- **Resolution:** 3.8 Å
- **Associated domain:** DED-2
- **b-phipsi:** 0.250425
- **w-rdist:** 0.946815
- **t-alpha:** 0.0
- **Chemical similarity (Tanimoto Index) (%):** 72.27
- **1D identity (%) [PDB]:** 0.17
- **1D identity (%) [Gaps excluded][PDB]:** 66.67
- **1D identity - Alignment Gaps [PDB]:** 1167
- **2D identity (%) [PDB]:** 12.92
- **2D identity (%) [Gaps excluded][PDB]:** 87.42
- **2D identity - Alignment Gaps [PDB]:** 871
- **3D similarity (TM-Score) (%) [PDB]:** 8.46

- **Gene name:** MC159L
- **RefSeq ID:** NC\_001731
- **Genomic sequence length:** 190289
- **5-UTR|CDS|3-UTR identity (%):** N/A | 12.58 | N/A
- **5-UTR|CDS|3-UTR identity (%) [Gaps excluded]:** N/A | 77.36 | N/A
- **5-UTR|CDS|3-UTR identity [Alignment Gaps]:** N/A | 3276 | N/A

**Uniprot Description:**  
  
Inhibits TNFRSF1A, TNFRSF6/FAS and TNFRSF12 induced apoptosis. Directs the degradation of host NFKBIB but not NFKBIA. Suppresses also host NF-kappa-B activation by interacting with and preventing ubiquitination of host NEMO/IKBKG, the NF-kappa-B essential modulator subunit of the IKK complex (PubMed:28515292). Interferes with host CASP8/caspase-8 recruitment and activation at the death-inducing signaling complex (DISC). May lead to higher virus production and contribute to virus persistence and oncogenicity. Participates also in the inhibition of host autophagy by interacting with host SH3BP4 (PubMed:30842330).  
  
Associates with the death-inducing signaling complex (DISC) formed by TNFRSF6/FAS, FADD and CASP8. Interacts with FADD (PubMed:16364918). Interacts with host TRAF2 (PubMed:16040075). Interacts with host NEMO/IKBKG (via N-terminus)(PubMed:28515292). Interacts with host SH3BP4; this interaction plays an important in the suppression of host autophagy (PubMed:30842330).  
  
**Gene Ontology Information:**

Molecular Function

- cysteine-type endopeptidase activity

Location

- host cell cytoplasm
- host cell nucleus

Biological process

- positive regulation of necroptotic process
- suppression by virus of host apoptotic process
- suppression by virus of host autophagy
- suppression by virus of host NF-kappaB transcription factor activity

---
