# Supplementary material for: Identifying and profiling structural similarities between Spike of SARS-CoV-2 and other viral or host proteins with Machaon
Source: Commun Biol. 2023 Jul 19;6:752. doi: 10.1038/s42003-023-05076-7 (PMC10356814; doi:10.1038/s42003-023-05076-7)
Supplement: Supplementary file 7 — Supplementary Data 4 [file 42003_2023_5076_MOESM7_ESM.zip › 6VXX_A_domain/candidates/6VXX_A_RBD-merged-enriched_eval_report.html]

 

# Structural Comparison Report for 6VXX\_A\_RBD - domains (total: 34)

---

1

- **Protein name:** Spike glycoprotein
- **Organism:** Middle East respiratory syndrome-related coronavirus
- **Uniprot Accession Number:** W5ZZF5
- **Protein sequence length:** 1353 aa
- **1D identity (%):** 29.92
- **1D identity (%) [Gaps excluded]:** 37.12
- **1D identity - Alignment Gaps:** 282
- **Common reported functions (%):** 0.0
- **Common reported locations (%):** 62.5
- **Common reported processes (%):** 50.0

- **PDB ID:** 5W9L
- **Chain:** J
- **Crystallized protein length:** 726 aa
- **Resolution:** 4.8 Å
- **Associated domain:** BetaCoV-S1-CTD
- **b-phipsi:** 0.003446
- **w-rdist:** 0.323229
- **t-alpha:** 0.0
- **Chemical similarity (Tanimoto Index) (%):** N/A
- **1D identity (%) [PDB]:** 0.0
- **1D identity (%) [Gaps excluded][PDB]:** 0.0
- **1D identity - Alignment Gaps [PDB]:** 1709
- **2D identity (%) [PDB]:** 30.74
- **2D identity (%) [Gaps excluded][PDB]:** 83.48
- **2D identity - Alignment Gaps [PDB]:** 789
- **3D similarity (TM-Score) (%) [PDB]:** 40.26

- **Gene name:** S
- **RefSeq ID:** N/A
- **Sequence length:** N/A
- **5-UTR|CDS|3-UTR identity (%):** N/A | N/A | N/A
- **5-UTR|CDS|3-UTR identity (%) [Gaps excluded]:** N/A | N/A | N/A
- **5-UTR|CDS|3-UTR identity [Alignment Gaps]:** N/A | N/A | N/A

**Uniprot Description:**  
  
Spike protein S1: attaches the virion to the cell membrane by interacting with host receptor, initiating the infection.  
  
Homotrimer; each monomer consists of a S1 and a S2 subunit. The resulting peplomers protrude from the virus surface as spikes.  
  
**Gene Ontology Information:**

Molecular Function  
  
N/A

Location

- host cell endoplasmic reticulum-Golgi intermediate compartment membrane
- host cell plasma membrane
- integral component of membrane
- viral envelope
- virion membrane

Biological process

- endocytosis involved in viral entry into host cell
- fusion of virus membrane with host endosome membrane
- fusion of virus membrane with host plasma membrane
- pathogenesis
- receptor-mediated virion attachment to host cell

---

2

- **Protein name:** Genome polyprotein
- **Organism:** Tobacco etch virus
- **Uniprot Accession Number:** P04517
- **Protein sequence length:** 3054 aa
- **1D identity (%):** 8.63
- **1D identity (%) [Gaps excluded]:** 27.83
- **1D identity - Alignment Gaps:** 2279
- **Common reported functions (%):** 0.0
- **Common reported locations (%):** 0.0
- **Common reported processes (%):** 0.0

- **PDB ID:** 6SUQ
- **Chain:** A
- **Crystallized protein length:** 2134 aa
- **Resolution:** 3.7 Å
- **Associated domain:** Peptidase-S30
- **b-phipsi:** 0.014723
- **w-rdist:** 0.584186
- **t-alpha:** 0.0
- **Chemical similarity (Tanimoto Index) (%):** 85.75
- **1D identity (%) [PDB]:** 0.03
- **1D identity (%) [Gaps excluded][PDB]:** 100.0
- **1D identity - Alignment Gaps [PDB]:** 3117
- **2D identity (%) [PDB]:** N/A
- **2D identity (%) [Gaps excluded][PDB]:** N/A
- **2D identity - Alignment Gaps [PDB]:** N/A
- **3D similarity (TM-Score) (%) [PDB]:** 25.52

- **Gene name:** N/A
- **RefSeq ID:** NC\_001555
- **Genomic sequence length:** 9494
- **5-UTR|CDS|3-UTR identity (%):** N/A | 28.55 | N/A
- **5-UTR|CDS|3-UTR identity (%) [Gaps excluded]:** N/A | 81.18 | N/A
- **5-UTR|CDS|3-UTR identity [Alignment Gaps]:** N/A | 6229 | N/A

**Uniprot Description:**  
  
Capsid protein
involved in aphid transmission, cell-to-cell and systemis movement, encapsidation of the viral RNA and in the regulation of viral RNA amplification.  
  
Nuclear inclusion protein A protease is a dimer; disulfide-linked.  
  
**Gene Ontology Information:**

Molecular Function

- ATP binding
- cysteine-type endopeptidase activity
- helicase activity
- hydrolase activity, acting on acid anhydrides, in phosphorus-containing anhydrides
- RNA binding
- RNA-directed 5'-3' RNA polymerase activity
- serine-type peptidase activity
- structural molecule activity

Location

- helical viral capsid

Biological process

- RNA-protein covalent cross-linking
- transcription, DNA-templated
- viral RNA genome replication

---

3

- **Protein name:** Nuclear RNA export factor 1
- **Organism:** Homo sapiens
- **Uniprot Accession Number:** Q9UBU9
- **Protein sequence length:** 619 aa
- **1D identity (%):** 4.21
- **1D identity (%) [Gaps excluded]:** 30.57
- **1D identity - Alignment Gaps:** 1434
- **Common reported functions (%):** 0.0
- **Common reported locations (%):** 0.0
- **Common reported processes (%):** 0.0

- **PDB ID:** 6E5U
- **Chain:** A
- **Crystallized protein length:** 339 aa
- **Resolution:** 3.8 Å
- **Associated domain:** NTF2
- **b-phipsi:** 0.017273
- **w-rdist:** 0.773548
- **t-alpha:** 0.0
- **Chemical similarity (Tanimoto Index) (%):** N/A
- **1D identity (%) [PDB]:** 0.08
- **1D identity (%) [Gaps excluded][PDB]:** 100.0
- **1D identity - Alignment Gaps [PDB]:** 1321
- **2D identity (%) [PDB]:** 16.32
- **2D identity (%) [Gaps excluded][PDB]:** 90.59
- **2D identity - Alignment Gaps [PDB]:** 919
- **3D similarity (TM-Score) (%) [PDB]:** 12.28

- **Gene name:** NXF1
- **RefSeq ID:** NM\_006362
- **Transcript sequence length:** 2290
- **5-UTR|CDS|3-UTR identity (%):** 18.86 | 31.29 | 33.17
- **5-UTR|CDS|3-UTR identity (%) [Gaps excluded]:** 77.94 | 78.0 | 78.36
- **5-UTR|CDS|3-UTR identity [Alignment Gaps]:** 213 | 2428 | 233

**Uniprot Description:**  
  
Involved in the nuclear export of mRNA species bearing retroviral constitutive transport elements (CTE) and in the export of mRNA from the nucleus to the cytoplasm (TAP/NFX1 pathway) (PubMed:10924507). The NXF1-NXT1 heterodimer is involved in the export of HSP70 mRNA in conjunction with ALYREF/THOC4 and THOC5 components of the TREX complex (PubMed:18364396, PubMed:19165146, PubMed:9660949). ALYREF/THOC4-bound mRNA is thought to be transferred to the NXF1-NXT1 heterodimer for export (PubMed:18364396, PubMed:19165146, PubMed:9660949). Also involved in nuclear export of m6A-containing mRNAs: interaction between SRSF3 and YTHDC1 facilitates m6A-containing mRNA-binding to both SRSF3 and NXF1, promoting mRNA nuclear export (PubMed:28984244).  
  
Heterodimer (via NTF2 domain) with NXT1 (PubMed:11583626). The formation of NXF1-NXT1 heterodimers is required for the NXF1-mediated nuclear mRNA export (PubMed:11583626). Forms a complex with RANBP2/NUP358, NXT1 and RANGAP1 (PubMed:14729961). Associates with the exon junction complex (EJC) and with the transcription/export (TREX) complex (PubMed:11707413, PubMed:22893130). Found in a mRNA complex with UPF3A and UPF3B (PubMed:11546873). Found in a post-splicing complex with RBM8A, UPF1, UPF2, UPF3A, UPF3B and RNPS1 (PubMed:11546874). Interacts (via N-terminus) with DHX9 (via N-terminus); this interaction is direct and negatively regulates NXF1-mediated nuclear export of constitutive transport element (CTE)-containing cellular mRNAs (PubMed:10924507). Interacts with ALYREF/THOC4 (PubMed:11707413, PubMed:14730019, PubMed:19165146, PubMed:23299939). Interacts with FYTTD1/UIF (PubMed:19836239). Interacts with EIF4A3 (PubMed:14730019). Interacts with NUP42 (PubMed:10228171). Interacts with THOC5 (PubMed:19165146, PubMed:23299939). Interacts with CHTOP (PubMed:23299939, PubMed:23826332). Interacts with FRG1 (via N-terminus) (PubMed:21699900). Interacts with LUZP4 (PubMed:25662211). Interacts with FMR1; the interaction occurs in a mRNA-dependent and polyribosomes-independent manner in the nucleus (PubMed:18936162). Interacts with CPSF6 (via N-terminus); this interaction is direct (PubMed:19864460). Interacts with RBM15 (PubMed:17001072). Interacts with RBM15B (PubMed:19586903). Interacts with MCM3AP isoform GANP; this interaction is not mediated by RNA (PubMed:20005110). Interacts with DDX3X (via C-terminus); this interaction may be partly involved in DDX3X nuclear export and in NXF1 localization to stress granules (PubMed:18596238). Interacts with PABPC1/PABP1 (PubMed:18596238).  
  
**Gene Ontology Information:**

Molecular Function

- mRNA binding
- RNA binding

Location

- cytoplasm
- cytoplasmic stress granule
- cytosol
- nuclear inclusion body
- nuclear pore
- nuclear speck
- nucleoplasm
- nucleus

Biological process

- mRNA export from nucleus
- poly(A)+ mRNA export from nucleus
- RNA export from nucleus
- viral process

---

4

- **Protein name:** Probable flavin-dependent thymidylate synthase
- **Organism:** Paramecium bursaria Chlorella virus 1
- **Uniprot Accession Number:** O41156
- **Protein sequence length:** 216 aa
- **1D identity (%):** 4.41
- **1D identity (%) [Gaps excluded]:** 28.93
- **1D identity - Alignment Gaps:** 1095
- **Common reported functions (%):** 0.0
- **Common reported locations (%):** 0.0
- **Common reported processes (%):** 0.0

- **PDB ID:** 4FZB
- **Chain:** H
- **Crystallized protein length:** 179 aa
- **Resolution:** 2.59 Å
- **Associated domain:** ThyX
- **b-phipsi:** 0.078887
- **w-rdist:** 0.305261
- **t-alpha:** 0.0
- **Chemical similarity (Tanimoto Index) (%):** 82.92
- **1D identity (%) [PDB]:** 0.09
- **1D identity (%) [Gaps excluded][PDB]:** 50.0
- **1D identity - Alignment Gaps [PDB]:** 1160
- **2D identity (%) [PDB]:** 13.21
- **2D identity (%) [Gaps excluded][PDB]:** 89.33
- **2D identity - Alignment Gaps [PDB]:** 864
- **3D similarity (TM-Score) (%) [PDB]:** 7.63

- **Gene name:** A674R
- **RefSeq ID:** NC\_000852
- **Genomic sequence length:** 330611
- **5-UTR|CDS|3-UTR identity (%):** N/A | 12.3 | N/A
- **5-UTR|CDS|3-UTR identity (%) [Gaps excluded]:** N/A | 81.43 | N/A
- **5-UTR|CDS|3-UTR identity [Alignment Gaps]:** N/A | 3299 | N/A

**Uniprot Description:**  
  
Catalyzes the reductive methylation of 2'-deoxyuridine-5'-monophosphate (dUMP) to 2'-deoxythymidine-5'-monophosphate (dTMP) while utilizing 5,10-methylenetetrahydrofolate (mTHF) as the methyl donor, and NADPH and FADH(2) as the reductant.  
  
Homotetramer.  
  
**Gene Ontology Information:**

Molecular Function

- flavin adenine dinucleotide binding
- thymidylate synthase (FAD) activity

Location  
  
N/A

Biological process

- dTMP biosynthetic process
- dTTP biosynthetic process
- methylation

---

5

- **Protein name:** DNA dC->dU-editing enzyme APOBEC-3F
- **Organism:** Homo sapiens
- **Uniprot Accession Number:** Q8IUX4
- **Protein sequence length:** 373 aa
- **1D identity (%):** 6.37
- **1D identity (%) [Gaps excluded]:** 29.15
- **1D identity - Alignment Gaps:** 1056
- **Common reported functions (%):** 50.0
- **Common reported locations (%):** 0.0
- **Common reported processes (%):** 0.0

- **PDB ID:** 6NIL
- **Chain:** A
- **Crystallized protein length:** 177 aa
- **Resolution:** 3.9 Å
- **Associated domain:** CMP-dCMP-type-deaminase-2
- **b-phipsi:** 0.041498
- **w-rdist:** 0.787699
- **t-alpha:** 0.0
- **Chemical similarity (Tanimoto Index) (%):** 82.98
- **1D identity (%) [PDB]:** 0.0
- **1D identity (%) [Gaps excluded][PDB]:** 0.0
- **1D identity - Alignment Gaps [PDB]:** 1161
- **2D identity (%) [PDB]:** 9.32
- **2D identity (%) [Gaps excluded][PDB]:** 89.91
- **2D identity - Alignment Gaps [PDB]:** 943
- **3D similarity (TM-Score) (%) [PDB]:** 7.41

- **Gene name:** APOBEC3F
- **RefSeq ID:** N/A
- **Sequence length:** N/A
- **5-UTR|CDS|3-UTR identity (%):** N/A | N/A | N/A
- **5-UTR|CDS|3-UTR identity (%) [Gaps excluded]:** N/A | N/A | N/A
- **5-UTR|CDS|3-UTR identity [Alignment Gaps]:** N/A | N/A | N/A

**Uniprot Description:**  
  
DNA deaminase (cytidine deaminase) which acts as an inhibitor of retrovirus replication and retrotransposon mobility via deaminase-dependent and -independent mechanisms. Exhibits antiviral activity against Vif-deficient HIV-1 (PubMed:15152192, PubMed:23001005). After the penetration of retroviral nucleocapsids into target cells of infection and the initiation of reverse transcription, it can induce the conversion of cytosine to uracil in the minus-sense single-strand viral DNA, leading to G-to-A hypermutations in the subsequent plus-strand viral DNA. The resultant detrimental levels of mutations in the proviral genome, along with a deamination-independent mechanism that works prior to the proviral integration, together exert efficient antiretroviral effects in infected target cells. Selectively targets single-stranded DNA and does not deaminate double-stranded DNA or single- or double-stranded RNA. Exhibits antiviral activity also against hepatitis B virus (HBV), equine infectious anemia virus (EIAV), xenotropic MuLV-related virus (XMRV) and simian foamy virus (SFV) and may inhibit the mobility of LTR and non-LTR retrotransposons. May also play a role in the epigenetic regulation of gene expression through the process of active DNA demethylation.  
  
Interacts with APOBEC3G in an RNA-dependent manner (PubMed:16699599). Interacts with AGO1, AGO2 and AGO3 (PubMed:22915799).  
  
**Gene Ontology Information:**

Molecular Function

- cytidine deaminase activity
- deoxycytidine deaminase activity
- identical protein binding
- RNA binding
- zinc ion binding

Location

- apolipoprotein B mRNA editing enzyme complex
- cytoplasm
- nucleus
- P-body
- ribonucleoprotein complex

Biological process

- base conversion or substitution editing
- cytidine to uridine editing
- defense response to virus
- DNA cytosine deamination
- DNA demethylation
- innate immune response
- negative regulation of single stranded viral RNA replication via double stranded DNA intermediate
- negative regulation of transposition
- negative regulation of viral genome replication
- negative regulation of viral process
- positive regulation of defense response to virus by host

---

6

- **Protein name:** B- and T-lymphocyte attenuator
- **Organism:** Homo sapiens
- **Uniprot Accession Number:** Q7Z6A9
- **Protein sequence length:** 289 aa
- **1D identity (%):** 3.19
- **1D identity (%) [Gaps excluded]:** 29.41
- **1D identity - Alignment Gaps:** 1256
- **Common reported functions (%):** 0.0
- **Common reported locations (%):** 0.0
- **Common reported processes (%):** 0.0

- **PDB ID:** 6NYP
- **Chain:** A
- **Crystallized protein length:** 102 aa
- **Resolution:** 2.7 Å
- **Associated domain:** Ig-like-V-type
- **b-phipsi:** 0.022435
- **w-rdist:** 0.919067
- **t-alpha:** 0.0
- **Chemical similarity (Tanimoto Index) (%):** 82.09
- **1D identity (%) [PDB]:** 0.0
- **1D identity (%) [Gaps excluded][PDB]:** 0.0
- **1D identity - Alignment Gaps [PDB]:** 1085
- **2D identity (%) [PDB]:** 8.93
- **2D identity (%) [Gaps excluded][PDB]:** 88.0
- **2D identity - Alignment Gaps [PDB]:** 885
- **3D similarity (TM-Score) (%) [PDB]:** 6.78

- **Gene name:** BTLA
- **RefSeq ID:** N/A
- **Sequence length:** N/A
- **5-UTR|CDS|3-UTR identity (%):** N/A | N/A | N/A
- **5-UTR|CDS|3-UTR identity (%) [Gaps excluded]:** N/A | N/A | N/A
- **5-UTR|CDS|3-UTR identity [Alignment Gaps]:** N/A | N/A | N/A

**Uniprot Description:**  
  
Inhibitory receptor on lymphocytes that negatively regulates antigen receptor signaling via PTPN6/SHP-1 and PTPN11/SHP-2 (PubMed:12796776, PubMed:14652006, PubMed:15568026, PubMed:18193050). May interact in cis (on the same cell) or in trans (on other cells) with TNFRSF14 (PubMed:19915044). In cis interactions, appears to play an immune regulatory role inhibiting in trans interactions in naive T cells to maintain a resting state. In trans interactions, can predominate during adaptive immune response to provide survival signals to effector T cells (PubMed:19915044).  
  
Interacts with tyrosine phosphatases PTPN6/SHP-1 and PTPN11/SHP-2 (PubMed:12796776, PubMed:14652006). Interacts with TNFRSF14/HVEM (via cysteine-rich domain 1) (PubMed:15568026, PubMed:18193050, PubMed:19915044).  
  
**Gene Ontology Information:**

Molecular Function

- signaling receptor activity

Location

- integral component of plasma membrane
- plasma membrane

Biological process

- adaptive immune response
- immune response-regulating cell surface receptor signaling pathway
- T cell costimulation

---

7

- **Protein name:** Replicase polyprotein 1ab
- **Organism:** Equine arteritis virus (strain Bucyrus)
- **Uniprot Accession Number:** P19811
- **Protein sequence length:** 3175 aa
- **1D identity (%):** 7.13
- **1D identity (%) [Gaps excluded]:** 31.74
- **1D identity - Alignment Gaps:** 2816
- **Common reported functions (%):** 12.5
- **Common reported locations (%):** 20.0
- **Common reported processes (%):** 1270400.0

- **PDB ID:** 4N0O
- **Chain:** G
- **Crystallized protein length:** 393 aa
- **Resolution:** 2.65 Å
- **Associated domain:** Peptidase-C33
- **b-phipsi:** 0.021432
- **w-rdist:** 0.658778
- **t-alpha:** 0.001712
- **Chemical similarity (Tanimoto Index) (%):** N/A
- **1D identity (%) [PDB]:** 0.0
- **1D identity (%) [Gaps excluded][PDB]:** 0.0
- **1D identity - Alignment Gaps [PDB]:** 1379
- **2D identity (%) [PDB]:** 15.53
- **2D identity (%) [Gaps excluded][PDB]:** 91.04
- **2D identity - Alignment Gaps [PDB]:** 977
- **3D similarity (TM-Score) (%) [PDB]:** N/A

- **Gene name:** rep
- **RefSeq ID:** N/A
- **Sequence length:** NC\_002532
- **5-UTR|CDS|3-UTR identity (%):** N/A | 39.23 | N/A
- **5-UTR|CDS|3-UTR identity (%) [Gaps excluded]:** N/A | 77.64 | N/A
- **5-UTR|CDS|3-UTR identity [Alignment Gaps]:** N/A | 2960 | N/A

**Uniprot Description:**  
  
The replicase polyprotein 1ab is a multifunctional protein: it contains the activities necessary for the transcription of negative stranded RNA, leader RNA, subgenomic mRNAs and progeny virion RNA as well as proteinases responsible for the cleavage of the polyprotein into functional products.  
  
Nsp1 interacts with cellular transcription cofactor SND1/p100.  
  
**Gene Ontology Information:**

Molecular Function

- ATP binding
- cysteine-type endopeptidase activity
- DNA helicase activity
- hydrolase activity, acting on acid anhydrides
- RNA binding
- RNA helicase activity
- RNA-directed 5'-3' RNA polymerase activity
- serine-type endopeptidase activity
- serine-type exopeptidase activity
- thiol-dependent ubiquitin-specific protease activity
- zinc ion binding

Location

- host cell membrane
- host cell nucleus
- host cell perinuclear region of cytoplasm
- integral component of membrane

Biological process

- modulation by virus of host protein ubiquitination
- suppression by virus of host ISG15 activity
- suppression by virus of host type I interferon-mediated signaling pathway
- transcription, DNA-templated
- viral protein processing
- viral RNA genome replication

---

8

- **Protein name:** Nucleoprotein
- **Organism:** Severe acute respiratory syndrome coronavirus
- **Uniprot Accession Number:** P59595
- **Protein sequence length:** 422 aa
- **1D identity (%):** 6.48
- **1D identity (%) [Gaps excluded]:** 27.64
- **1D identity - Alignment Gaps:** 1051
- **Common reported functions (%):** 50.0
- **Common reported locations (%):** 0.0
- **Common reported processes (%):** 20.0

- **PDB ID:** 2CJR
- **Chain:** F
- **Crystallized protein length:** 112 aa
- **Resolution:** 2.5 Å
- **Associated domain:** CoV-N-CTD
- **b-phipsi:** 0.08259
- **w-rdist:** 0.753327
- **t-alpha:** 0.0
- **Chemical similarity (Tanimoto Index) (%):** 81.98
- **1D identity (%) [PDB]:** 0.18
- **1D identity (%) [Gaps excluded][PDB]:** 50.0
- **1D identity - Alignment Gaps [PDB]:** 1087
- **2D identity (%) [PDB]:** 5.94
- **2D identity (%) [Gaps excluded][PDB]:** 89.71
- **2D identity - Alignment Gaps [PDB]:** 959
- **3D similarity (TM-Score) (%) [PDB]:** 5.91

- **Gene name:** N
- **RefSeq ID:** NC\_004718
- **Genomic sequence length:** 29751
- **5-UTR|CDS|3-UTR identity (%):** 88.52 | 4.33 | 22.38
- **5-UTR|CDS|3-UTR identity (%) [Gaps excluded]:** 92.28 | 82.18 | 98.18
- **5-UTR|CDS|3-UTR identity [Alignment Gaps]:** 11 | 3631 | 745

**Uniprot Description:**  
  
Packages the positive strand viral genome RNA into a helical ribonucleocapsid (RNP) and plays a fundamental role during virion assembly through its interactions with the viral genome and membrane protein M. Plays an important role in enhancing the efficiency of subgenomic viral RNA transcription as well as viral replication (PubMed:17210170). May modulate transforming growth factor-beta signaling by binding host SMAD3 (PubMed:18055455).  
  
Homooligomer. Both monomeric and oligomeric forms interact with RNA. Interacts with protein M (PubMed:15351485). Interacts with protein E (PubMed:24766657). May bind to host HNRNPA1 (Probable). Interacts with NSP3; this interaction serves to tether the genome to the newly translated replicase-transcriptase complex at a very early stage of infection (By similarity). May interact with host SMAD3 (Probable). Interacts with host PPIA/CYPA (PubMed:15688292).  
  
**Gene Ontology Information:**

Molecular Function

- identical protein binding
- RNA binding

Location

- host cell endoplasmic reticulum-Golgi intermediate compartment
- host cell Golgi apparatus
- host cell perinuclear region of cytoplasm
- viral capsid
- viral nucleocapsid

Biological process

- viral protein processing
- viral translation

---

9

- **Protein name:** Polyubiquitin-B
- **Organism:** Homo sapiens
- **Uniprot Accession Number:** P0CG47
- **Protein sequence length:** 229 aa
- **1D identity (%):** 2.98
- **1D identity (%) [Gaps excluded]:** 24.84
- **1D identity - Alignment Gaps:** 1180
- **Common reported functions (%):** 0.0
- **Common reported locations (%):** 12.5
- **Common reported processes (%):** 10.0

- **PDB ID:** 6XAA
- **Chain:** B
- **Crystallized protein length:** 75 aa
- **Resolution:** 2.7 Å
- **Associated domain:** Ubiquitin-like-1
- **b-phipsi:** 0.018757
- **w-rdist:** 1.03718
- **t-alpha:** 0.0
- **Chemical similarity (Tanimoto Index) (%):** 70.61
- **1D identity (%) [PDB]:** 0.0
- **1D identity (%) [Gaps excluded][PDB]:** 0.0
- **1D identity - Alignment Gaps [PDB]:** 1058
- **2D identity (%) [PDB]:** 4.59
- **2D identity (%) [Gaps excluded][PDB]:** 83.64
- **2D identity - Alignment Gaps [PDB]:** 948
- **3D similarity (TM-Score) (%) [PDB]:** 5.1

- **Gene name:** UBB
- **RefSeq ID:** NM\_018955
- **Transcript sequence length:** 933
- **5-UTR|CDS|3-UTR identity (%):** 23.99 | 11.72 | 36.96
- **5-UTR|CDS|3-UTR identity (%) [Gaps excluded]:** 69.15 | 79.62 | 82.61
- **5-UTR|CDS|3-UTR identity [Alignment Gaps]:** 177 | 3354 | 142

**Uniprot Description:**  
  
Ubiquitin
Exists either covalently attached to another protein, or free (unanchored). When covalently bound, it is conjugated to target proteins via an isopeptide bond either as a monomer (monoubiquitin), a polymer linked via different Lys residues of the ubiquitin (polyubiquitin chains) or a linear polymer linked via the initiator Met of the ubiquitin (linear polyubiquitin chains). Polyubiquitin chains, when attached to a target protein, have different functions depending on the Lys residue of the ubiquitin that is linked: Lys-6-linked may be involved in DNA repair; Lys-11-linked is involved in ERAD (endoplasmic reticulum-associated degradation) and in cell-cycle regulation; Lys-29-linked is involved in lysosomal degradation; Lys-33-linked is involved in kinase modification; Lys-48-linked is involved in protein degradation via the proteasome; Lys-63-linked is involved in endocytosis, DNA-damage responses as well as in signaling processes leading to activation of the transcription factor NF-kappa-B. Linear polymer chains formed via attachment by the initiator Met lead to cell signaling. Ubiquitin is usually conjugated to Lys residues of target proteins, however, in rare cases, conjugation to Cys or Ser residues has been observed. When polyubiquitin is free (unanchored-polyubiquitin), it also has distinct roles, such as in activation of protein kinases, and in signaling.  
  
Interacts with SKP1-KMD2A and SKP1-KMD2B complexes.  
  
**Gene Ontology Information:**

Molecular Function

- protein tag
- ubiquitin protein ligase binding

Location

- cytoplasm
- cytosol
- endocytic vesicle membrane
- endoplasmic reticulum membrane
- endoplasmic reticulum quality control compartment
- endosome membrane
- extracellular exosome
- extracellular space
- host cell
- mitochondrial outer membrane
- mitochondrion
- neuron projection
- neuronal cell body
- nucleoplasm
- nucleus
- plasma membrane
- vesicle

Biological process

- activation of MAPK activity
- aggrephagy
- amyloid fibril formation
- anaphase-promoting complex-dependent catabolic process
- cytokine-mediated signaling pathway
- cytoplasmic pattern recognition receptor signaling pathway
- DNA damage response, detection of DNA damage
- endoplasmic reticulum mannose trimming
- endosomal transport
- energy homeostasis
- error-free translesion synthesis
- error-prone translesion synthesis
- fat pad development
- female gonad development
- female meiosis I
- global genome nucleotide-excision repair
- hypothalamus gonadotrophin-releasing hormone neuron development
- I-kappaB kinase/NF-kappaB signaling
- interleukin-1-mediated signaling pathway
- interstrand cross-link repair
- intracellular transport of virus
- JNK cascade
- male meiosis I
- membrane organization
- mitochondrion transport along microtubule
- modification-dependent protein catabolic process
- modulation by symbiont of host defense response
- MyD88-dependent toll-like receptor signaling pathway
- MyD88-independent toll-like receptor signaling pathway
- negative regulation of apoptotic process
- negative regulation of transcription by RNA polymerase II
- negative regulation of transforming growth factor beta receptor signaling pathway
- neuron projection morphogenesis
- nucleotide-binding oligomerization domain containing signaling pathway
- nucleotide-excision repair, DNA damage recognition
- nucleotide-excision repair, DNA duplex unwinding
- nucleotide-excision repair, DNA gap filling
- nucleotide-excision repair, DNA incision
- nucleotide-excision repair, DNA incision, 5'-to lesion
- nucleotide-excision repair, preincision complex assembly
- positive regulation of apoptotic process
- positive regulation of intrinsic apoptotic signaling pathway by p53 class mediator
- positive regulation of NF-kappaB transcription factor activity
- positive regulation of protein monoubiquitination
- positive regulation of protein ubiquitination
- positive regulation of transcription by RNA polymerase II
- pre-replicative complex assembly
- protein deubiquitination
- protein localization
- protein polyubiquitination
- protein ubiquitination
- regulation of exit from mitosis
- regulation of mitochondrial membrane potential
- regulation of mRNA stability
- regulation of neuron death
- regulation of proteasomal protein catabolic process
- regulation of transcription from RNA polymerase II promoter in response to hypoxia
- seminiferous tubule development
- stress-activated MAPK cascade
- transcription-coupled nucleotide-excision repair
- transforming growth factor beta receptor signaling pathway
- translesion synthesis
- transmembrane transport
- TRIF-dependent toll-like receptor signaling pathway
- viral life cycle
- viral translation
- virion assembly
- Wnt signaling pathway

---

10

- **Protein name:** Ubiquitin-like protein ISG15
- **Organism:** Mus musculus
- **Uniprot Accession Number:** Q64339
- **Protein sequence length:** 161 aa
- **1D identity (%):** 2.94
- **1D identity (%) [Gaps excluded]:** 27.14
- **1D identity - Alignment Gaps:** 1154
- **Common reported functions (%):** 0.0
- **Common reported locations (%):** 0.0
- **Common reported processes (%):** 0.0

- **PDB ID:** 6J62
- **Chain:** A
- **Crystallized protein length:** 150 aa
- **Resolution:** 2.49 Å
- **Associated domain:** Ubiquitin-like-2
- **b-phipsi:** 0.018029
- **w-rdist:** 1.045332
- **t-alpha:** 0.0
- **Chemical similarity (Tanimoto Index) (%):** 82.81
- **1D identity (%) [PDB]:** 0.0
- **1D identity (%) [Gaps excluded][PDB]:** 0.0
- **1D identity - Alignment Gaps [PDB]:** 1133
- **2D identity (%) [PDB]:** 10.58
- **2D identity (%) [Gaps excluded][PDB]:** 87.7
- **2D identity - Alignment Gaps [PDB]:** 889
- **3D similarity (TM-Score) (%) [PDB]:** 7.89

- **Gene name:** Isg15
- **RefSeq ID:** N/A
- **Sequence length:** N/A
- **5-UTR|CDS|3-UTR identity (%):** N/A | N/A | N/A
- **5-UTR|CDS|3-UTR identity (%) [Gaps excluded]:** N/A | N/A | N/A
- **5-UTR|CDS|3-UTR identity [Alignment Gaps]:** N/A | N/A | N/A

**Uniprot Description:**  
  
Ubiquitin-like protein which plays a key role in the innate immune response to viral infection either via its conjugation to a target protein (ISGylation) or via its action as a free or unconjugated protein. ISGylation involves a cascade of enzymatic reactions involving E1, E2, and E3 enzymes which catalyze the conjugation of ISG15 to a lysine residue in the target protein. Its target proteins include SERPINA3G/SPI2A, JAK1, MAPK3/ERK1, PLCG1, TRIM25, STAT5A, MAPK1/ERK2 and globin. Can also isgylate: DDX58/RIG-I which inhibits its function in antiviral signaling response, IRF3 which inhibits its ubiquitination and degradation as well as EIF4E2 which enhances its cap structure-binding activity and translation-inhibition activity. Exhibits antiviral activity towards both DNA and RNA viruses, including influenza A and B virus, sindbis virus (SV) and herpes simplex type-1 (HHV-1). Plays a significant role in the control of neonatal Chikungunya virus (CHIKV) infection by acting as a putative immunomodulator of proinflammatory cytokines. Protects mice against the consequences of Chikungunya virus infection by downregulating the pathogenic cytokine response, often denoted as the cytokine storm. Plays a role in erythroid differentiation. The secreted form of ISG15 can: induce natural killer cell proliferation, act as a chemotactic factor for neutrophils and act as a IFN-gamma-inducing cytokine playing an essential role in antimycobacterial immunity. The secreted form acts through the integrin ITGAL/ITGB2 receptor to initiate activation of SRC family tyrosine kinases including LYN, HCK and FGR which leads to secretion of IFNG and IL10; the interaction is mediated by ITGAL (By similarity).  
  
Homodimer; disulfide-linked (By similarity). Interacts with, and is conjugated to its targets by the UBE1L (E1 enzyme) and UBE2E2 (E2 enzyme) (By similarity). Interacts with NEDD4 (By similarity).  
  
**Gene Ontology Information:**

Molecular Function

- integrin binding
- protein tag
- ubiquitin protein ligase binding

Location

- cytoplasm
- cytosolic small ribosomal subunit
- extracellular region
- nucleus

Biological process

- defense response to bacterium
- defense response to virus
- integrin-mediated signaling pathway
- ISG15-protein conjugation
- modification-dependent protein catabolic process
- negative regulation of protein ubiquitination
- negative regulation of type I interferon-mediated signaling pathway
- negative regulation of viral genome replication
- positive regulation of bone mineralization
- positive regulation of erythrocyte differentiation
- positive regulation of interferon-gamma production
- positive regulation of interleukin-10 production
- regulation of interferon-gamma production
- response to bacterium
- response to type I interferon

---

11

- **Protein name:** Structural polyprotein
- **Organism:** Semliki forest virus
- **Uniprot Accession Number:** P03315
- **Protein sequence length:** 1253 aa
- **1D identity (%):** 8.19
- **1D identity (%) [Gaps excluded]:** 28.8
- **1D identity - Alignment Gaps:** 1408
- **Common reported functions (%):** 0.0
- **Common reported locations (%):** 50.0
- **Common reported processes (%):** 10.0

- **PDB ID:** 1DYL
- **Chain:** D
- **Crystallized protein length:** 149 aa
- **Resolution:** 9.0 Å
- **Associated domain:** Peptidase-S3
- **b-phipsi:** 0.076328
- **w-rdist:** 0.836188
- **t-alpha:** 0.0
- **Chemical similarity (Tanimoto Index) (%):** N/A
- **1D identity (%) [PDB]:** 0.09
- **1D identity (%) [Gaps excluded][PDB]:** 50.0
- **1D identity - Alignment Gaps [PDB]:** 1128
- **2D identity (%) [PDB]:** 9.6
- **2D identity (%) [Gaps excluded][PDB]:** 88.29
- **2D identity - Alignment Gaps [PDB]:** 910
- **3D similarity (TM-Score) (%) [PDB]:** 6.85

- **Gene name:** N/A
- **RefSeq ID:** NC\_003215
- **Genomic sequence length:** 11442
- **5-UTR|CDS|3-UTR identity (%):** N/A | 40.5 | N/A
- **5-UTR|CDS|3-UTR identity (%) [Gaps excluded]:** N/A | 76.59 | N/A
- **5-UTR|CDS|3-UTR identity [Alignment Gaps]:** N/A | 2338 | N/A

**Uniprot Description:**  
  
Capsid protein
Forms an icosahedral capsid with a T=4 symmetry composed of 240 copies of the capsid protein surrounded by a lipid membrane through which penetrate 80 spikes composed of trimers of E1-E2 heterodimers (By similarity). The capsid protein binds to the viral RNA genome at a site adjacent to a ribosome binding site for viral genome translation following genome release (By similarity). Possesses a protease activity that results in its autocatalytic cleavage from the nascent structural protein (PubMed:3553612, PubMed:9642067). Following its self-cleavage, the capsid protein transiently associates with ribosomes, and within several minutes the protein binds to viral RNA and rapidly assembles into icosahedric core particles (PubMed:516447). The resulting nucleocapsid eventually associates with the cytoplasmic domain of the spike glycoprotein E2 at the cell membrane, leading to budding and formation of mature virions (By similarity). In case of infection, new virions attach to target cells and after clathrin-mediated endocytosis their membrane fuses with the host endosomal membrane (PubMed:15954801). This leads to the release of the nucleocapsid into the cytoplasm, followed by an uncoating event necessary for the genomic RNA to become accessible (PubMed:1433506). The uncoating might be triggered by the interaction of capsid proteins with ribosomes (PubMed:1433506). Binding of ribosomes would release the genomic RNA since the same region is genomic RNA-binding and ribosome-binding (PubMed:1433506).  
  
Capsid protein
Homodimer (By similarity). Homomultimer (Probable). Interacts with host karyopherin KPNA4; this interaction allows the nuclear import of the viral capsid protein (By similarity). Interacts with spike glycoprotein E2 (By similarity).  
  
**Gene Ontology Information:**

Molecular Function

- RNA binding
- serine-type endopeptidase activity
- structural molecule activity

Location

- host cell endosome
- host cell nucleus
- host cell plasma membrane
- integral component of membrane
- T=4 icosahedral viral capsid
- viral envelope
- virion membrane

Biological process

- clathrin-dependent endocytosis of virus by host cell
- fusion of virus membrane with host endosome membrane
- virion assembly
- virion attachment to host cell

---

12

- **Protein name:** NTF2-related export protein 1
- **Organism:** Homo sapiens
- **Uniprot Accession Number:** Q9UKK6
- **Protein sequence length:** 140 aa
- **1D identity (%):** 3.04
- **1D identity (%) [Gaps excluded]:** 30.47
- **1D identity - Alignment Gaps:** 1157
- **Common reported functions (%):** 0.0
- **Common reported locations (%):** 0.0
- **Common reported processes (%):** 0.0

- **PDB ID:** 6E5U
- **Chain:** B
- **Crystallized protein length:** 138 aa
- **Resolution:** 3.8 Å
- **Associated domain:** NTF2
- **b-phipsi:** 0.002641
- **w-rdist:** 0.844082
- **t-alpha:** 0.017391
- **Chemical similarity (Tanimoto Index) (%):** N/A
- **1D identity (%) [PDB]:** 0.09
- **1D identity (%) [Gaps excluded][PDB]:** 100.0
- **1D identity - Alignment Gaps [PDB]:** 1119
- **2D identity (%) [PDB]:** 9.32
- **2D identity (%) [Gaps excluded][PDB]:** 93.14
- **2D identity - Alignment Gaps [PDB]:** 917
- **3D similarity (TM-Score) (%) [PDB]:** 12.28

- **Gene name:** NXT1
- **RefSeq ID:** N/A
- **Sequence length:** N/A
- **5-UTR|CDS|3-UTR identity (%):** N/A | N/A | N/A
- **5-UTR|CDS|3-UTR identity (%) [Gaps excluded]:** N/A | N/A | N/A
- **5-UTR|CDS|3-UTR identity [Alignment Gaps]:** N/A | N/A | N/A

**Uniprot Description:**  
  
Stimulator of protein export for NES-containing proteins (PubMed:10567585). Also plays a role in the nuclear export of U1 snRNA, tRNA, and mRNA (PubMed:10848583). The NXF1-NXT1 heterodimer is involved in the export of HSP70 mRNA in conjunction with ALYREF/THOC4 and THOC5 (PubMed:19165146, PubMed:11259602).  
  
Heterodimer with NXF1 (PubMed:11583626). Forms a complex with RANGAP1, RANBP2/NUP358 and NXF1 (PubMed:14729961). Interacts (via NTF2 domain) with NXF1 (PubMed:11583626). Stabilizes the NTF2 domain of NXF1 by heterodimerization (PubMed:11583626). The formation of NXF1-NXT1 heterodimers is required for the NXF1-mediated nuclear mRNA export (PubMed:11583626). Preferentially binds Ran-GTP (PubMed:10567585). Associates with NXF2, NXF3 and NXF5. Does not bind nucleoporins (NPC) directly, its association to NPC is mediated by NXF1 (PubMed:11583626).  
  
**Gene Ontology Information:**

Molecular Function

- small GTPase binding

Location

- cytoplasm
- cytosol
- nuclear pore
- nuclear pore central transport channel
- nuclear speck
- nucleoplasm

Biological process

- mRNA export from nucleus
- nucleocytoplasmic transport
- protein import into nucleus

---

13

- **Protein name:** Ephrin-B1
- **Organism:** Homo sapiens
- **Uniprot Accession Number:** P98172
- **Protein sequence length:** 346 aa
- **1D identity (%):** 1.3
- **1D identity (%) [Gaps excluded]:** 24.39
- **1D identity - Alignment Gaps:** 1455
- **Common reported functions (%):** 0.0
- **Common reported locations (%):** 0.0
- **Common reported processes (%):** 0.0

- **PDB ID:** 6THG
- **Chain:** J
- **Crystallized protein length:** 136 aa
- **Resolution:** 4.07 Å
- **Associated domain:** Ephrin-RBD
- **b-phipsi:** 0.018357
- **w-rdist:** 0.855183
- **t-alpha:** 0.001712
- **Chemical similarity (Tanimoto Index) (%):** 99.87
- **1D identity (%) [PDB]:** 0.0
- **1D identity (%) [Gaps excluded][PDB]:** 0.0
- **1D identity - Alignment Gaps [PDB]:** 1119
- **2D identity (%) [PDB]:** 8.0
- **2D identity (%) [Gaps excluded][PDB]:** 87.23
- **2D identity - Alignment Gaps [PDB]:** 931
- **3D similarity (TM-Score) (%) [PDB]:** 7.22

- **Gene name:** EFNB1
- **RefSeq ID:** NM\_004429
- **Transcript sequence length:** 3303
- **5-UTR|CDS|3-UTR identity (%):** 20.81 | 17.7 | 10.94
- **5-UTR|CDS|3-UTR identity (%) [Gaps excluded]:** 72.37 | 77.63 | 80.29
- **5-UTR|CDS|3-UTR identity [Alignment Gaps]:** 565 | 3057 | 1319

**Uniprot Description:**  
  
Cell surface transmembrane ligand for Eph receptors, a family of receptor tyrosine kinases which are crucial for migration, repulsion and adhesion during neuronal, vascular and epithelial development (PubMed:8070404, PubMed:7973638). Binding to Eph receptors residing on adjacent cells leads to contact-dependent bidirectional signaling into neighboring cells (PubMed:8070404, PubMed:7973638). Shows high affinity for the receptor tyrosine kinase EPHB1/ELK (PubMed:8070404, PubMed:7973638). Can also bind EPHB2 and EPHB3 (PubMed:8070404). Binds to, and induces collapse of, commissural axons/growth cones in vitro (By similarity). May play a role in constraining the orientation of longitudinally projecting axons (By similarity).  
  
Interacts (via PDZ-binding motif) with GRIP1 and GRIP2 (via PDZ domain 6) (PubMed:10197531). Interacts with TLE1 (PubMed:21429299). The intracellular domain peptide interacts with ZHX2; the interaction enhances ZHX2 transcriptional repression activity (By similarity).  
  
**Gene Ontology Information:**

Molecular Function

- ephrin receptor binding

Location

- cytoplasm
- extracellular exosome
- integral component of plasma membrane
- membrane raft
- nucleus
- plasma membrane
- synapse

Biological process

- axon guidance
- cell adhesion
- cell-cell signaling
- embryonic pattern specification
- ephrin receptor signaling pathway
- neural crest cell migration
- positive regulation of T cell proliferation
- T cell costimulation

---

14

- **Protein name:** U1 small nuclear ribonucleoprotein A
- **Organism:** Homo sapiens
- **Uniprot Accession Number:** P09012
- **Protein sequence length:** 282 aa
- **1D identity (%):** 4.59
- **1D identity (%) [Gaps excluded]:** 26.87
- **1D identity - Alignment Gaps:** 1101
- **Common reported functions (%):** 50.0
- **Common reported locations (%):** 0.0
- **Common reported processes (%):** 0.0

- **PDB ID:** 6XH3
- **Chain:** A
- **Crystallized protein length:** 88 aa
- **Resolution:** 2.35 Å
- **Associated domain:** RRM-1
- **b-phipsi:** 0.036139
- **w-rdist:** 1.029225
- **t-alpha:** 0.0
- **Chemical similarity (Tanimoto Index) (%):** 71.44
- **1D identity (%) [PDB]:** 0.0
- **1D identity (%) [Gaps excluded][PDB]:** 0.0
- **1D identity - Alignment Gaps [PDB]:** 1071
- **2D identity (%) [PDB]:** 5.85
- **2D identity (%) [Gaps excluded][PDB]:** 93.65
- **2D identity - Alignment Gaps [PDB]:** 945
- **3D similarity (TM-Score) (%) [PDB]:** 5.46

- **Gene name:** SNRPA
- **RefSeq ID:** NM\_004596
- **Transcript sequence length:** 1277
- **5-UTR|CDS|3-UTR identity (%):** 33.64 | 14.12 | 26.25
- **5-UTR|CDS|3-UTR identity (%) [Gaps excluded]:** 74.15 | 75.96 | 79.46
- **5-UTR|CDS|3-UTR identity [Alignment Gaps]:** 177 | 3207 | 227

**Uniprot Description:**  
  
Component of the spliceosomal U1 snRNP, which is essential for recognition of the pre-mRNA 5' splice-site and the subsequent assembly of the spliceosome. U1 snRNP is the first snRNP to interact with pre-mRNA. This interaction is required for the subsequent binding of U2 snRNP and the U4/U6/U5 tri-snRNP. SNRPA binds stem loop II of U1 snRNA. In a snRNP-free form (SF-A) may be involved in coupled pre-mRNA splicing and polyadenylation process. May bind preferentially to the 5'-UGCAC-3' motif on RNAs.  
  
U1 snRNP is composed of the 7 core Sm proteins SNRPB, SNRPD1, SNRPD2, SNRPD3, SNRPE, SNRPF and SNRPG that assemble in a heptameric protein ring on the Sm site of the small nuclear RNA to form the core snRNP, and at least three U1 snRNP-specific proteins SNRNP70/U1-70K, SNRPA/U1-A and SNRPC/U1-C. Interacts with SFPQ; component of a snRNP-free complex with SFPQ.  
  
**Gene Ontology Information:**

Molecular Function

- identical protein binding
- RNA binding
- U1 snRNA binding
- U1 snRNP binding

Location

- nucleoplasm
- spliceosomal complex
- U1 snRNP

Biological process

- mRNA splicing, via spliceosome
- regulation of mRNA polyadenylation

---

15

- **Protein name:** Integrase
- **Organism:** Escherichia phage P2
- **Uniprot Accession Number:** P36932
- **Protein sequence length:** 337 aa
- **1D identity (%):** 4.57
- **1D identity (%) [Gaps excluded]:** 30.33
- **1D identity - Alignment Gaps:** 1188
- **Common reported functions (%):** 0.0
- **Common reported locations (%):** 0.0
- **Common reported processes (%):** 10.0

- **PDB ID:** 5C6K
- **Chain:** A
- **Crystallized protein length:** 165 aa
- **Resolution:** 1.9 Å
- **Associated domain:** Tyr-recombinase
- **b-phipsi:** 0.077239
- **w-rdist:** 0.712698
- **t-alpha:** 0.001709
- **Chemical similarity (Tanimoto Index) (%):** 83.07
- **1D identity (%) [PDB]:** 0.0
- **1D identity (%) [Gaps excluded][PDB]:** 0.0
- **1D identity - Alignment Gaps [PDB]:** 1148
- **2D identity (%) [PDB]:** 9.72
- **2D identity (%) [Gaps excluded][PDB]:** 92.66
- **2D identity - Alignment Gaps [PDB]:** 930
- **3D similarity (TM-Score) (%) [PDB]:** 7.31

- **Gene name:** int
- **RefSeq ID:** NC\_001895
- **Genomic sequence length:** 33593
- **5-UTR|CDS|3-UTR identity (%):** N/A | 17.07 | N/A
- **5-UTR|CDS|3-UTR identity (%) [Gaps excluded]:** N/A | 79.14 | N/A
- **5-UTR|CDS|3-UTR identity [Alignment Gaps]:** N/A | 3120 | N/A

**Uniprot Description:**  
  
Integrase is necessary for integration of the phage into the host genome by site-specific recombination.  
  
**Gene Ontology Information:**

Molecular Function

- DNA binding
- hydrolase activity
- transferase activity

Location  
  
N/A

Biological process

- DNA integration
- DNA recombination
- establishment of integrated proviral latency
- viral entry into host cell
- viral genome integration into host DNA

---

16

- **Protein name:** Deoxycytidylate deaminase
- **Organism:** Enterobacteria phage T4
- **Uniprot Accession Number:** P16006
- **Protein sequence length:** 193 aa
- **1D identity (%):** 3.97
- **1D identity (%) [Gaps excluded]:** 33.12
- **1D identity - Alignment Gaps:** 1152
- **Common reported functions (%):** 0.0
- **Common reported locations (%):** 0.0
- **Common reported processes (%):** 0.0

- **PDB ID:** 1VQ2
- **Chain:** A
- **Crystallized protein length:** 173 aa
- **Resolution:** 2.2 Å
- **Associated domain:** CMP-dCMP-type-deaminase
- **b-phipsi:** 0.036319
- **w-rdist:** 0.754638
- **t-alpha:** 0.001712
- **Chemical similarity (Tanimoto Index) (%):** 80.77
- **1D identity (%) [PDB]:** 0.0
- **1D identity (%) [Gaps excluded][PDB]:** 0.0
- **1D identity - Alignment Gaps [PDB]:** 1157
- **2D identity (%) [PDB]:** 8.79
- **2D identity (%) [Gaps excluded][PDB]:** 93.94
- **2D identity - Alignment Gaps [PDB]:** 959
- **3D similarity (TM-Score) (%) [PDB]:** 6.32

- **Gene name:** CD
- **RefSeq ID:** NC\_000866
- **Genomic sequence length:** 168903
- **5-UTR|CDS|3-UTR identity (%):** N/A | 10.82 | N/A
- **5-UTR|CDS|3-UTR identity (%) [Gaps excluded]:** N/A | 80.61 | N/A
- **5-UTR|CDS|3-UTR identity [Alignment Gaps]:** N/A | 3362 | N/A

**Uniprot Description:**  
  
Supplies the nucleotide substrate for thymidylate synthetase.  
  
Homohexamer.  
  
**Gene Ontology Information:**

Molecular Function

- dCMP deaminase activity
- zinc ion binding

Location  
  
N/A

Biological process

- nucleotide biosynthetic process
- pyrimidine nucleotide metabolic process

---

17

- **Protein name:** Protein Rep68
- **Organism:** Adeno-associated virus 2 (isolate Srivastava/1982)
- **Uniprot Accession Number:** P03132
- **Protein sequence length:** 536 aa
- **1D identity (%):** 6.34
- **1D identity (%) [Gaps excluded]:** 28.75
- **1D identity - Alignment Gaps:** 1155
- **Common reported functions (%):** 0.0
- **Common reported locations (%):** 0.0
- **Common reported processes (%):** 0.0

- **PDB ID:** 1S9H
- **Chain:** A
- **Crystallized protein length:** 268 aa
- **Resolution:** 2.4 Å
- **Associated domain:** SF3-helicase
- **b-phipsi:** 0.044212
- **w-rdist:** 0.744052
- **t-alpha:** 0.001712
- **Chemical similarity (Tanimoto Index) (%):** 83.46
- **1D identity (%) [PDB]:** 0.0
- **1D identity (%) [Gaps excluded][PDB]:** 0.0
- **1D identity - Alignment Gaps [PDB]:** 1251
- **2D identity (%) [PDB]:** 14.07
- **2D identity (%) [Gaps excluded][PDB]:** 88.89
- **2D identity - Alignment Gaps [PDB]:** 909
- **3D similarity (TM-Score) (%) [PDB]:** 10.21

- **Gene name:** Rep68
- **RefSeq ID:** NC\_001401
- **Genomic sequence length:** 4679
- **5-UTR|CDS|3-UTR identity (%):** N/A | 26.47 | N/A
- **5-UTR|CDS|3-UTR identity (%) [Gaps excluded]:** N/A | 77.81 | N/A
- **5-UTR|CDS|3-UTR identity [Alignment Gaps]:** N/A | 2675 | N/A

**Uniprot Description:**  
  
Plays an essential role in the initiation of viral DNA synthesis. Binds specifically to an inverted terminal repeat element (ITR) on the 3' and 5' ends of the viral DNA, where it cleaves a site specifically to generate a priming site for initiation of the synthesis of a complementary strand. Plays also a role as transcriptional regulator, DNA helicase and as key factor in site-specific integration of the viral genome. Inhibits the host cell cycle G1/S and G2/M transitions. These arrests may provide essential cellular factors for viral DNA replication.  
  
Interacts with host TOPORS. Interacts with host KCTD5.  
  
**Gene Ontology Information:**

Molecular Function

- ATP binding
- DNA helicase activity
- hydrolase activity

Location  
  
N/A

Biological process

- DNA replication
- modulation by virus of host G1/S transition checkpoint
- permeabilization of host organelle membrane involved in viral entry into host cell
- suppression by virus of G2/M transition of host mitotic cell cycle
- viral DNA genome replication
- viral entry via permeabilization of inner membrane

---

18

- **Protein name:** Ubiquitin-like protein ISG15
- **Organism:** Homo sapiens
- **Uniprot Accession Number:** P05161
- **Protein sequence length:** 165 aa
- **1D identity (%):** 2.69
- **1D identity (%) [Gaps excluded]:** 25.93
- **1D identity - Alignment Gaps:** 1168
- **Common reported functions (%):** 0.0
- **Common reported locations (%):** 0.0
- **Common reported processes (%):** 0.0

- **PDB ID:** 3RT3
- **Chain:** B
- **Crystallized protein length:** 153 aa
- **Resolution:** 2.01 Å
- **Associated domain:** Ubiquitin-like-2
- **b-phipsi:** 0.020515
- **w-rdist:** 1.037105
- **t-alpha:** 0.001709
- **Chemical similarity (Tanimoto Index) (%):** 81.95
- **1D identity (%) [PDB]:** 0.0
- **1D identity (%) [Gaps excluded][PDB]:** 0.0
- **1D identity - Alignment Gaps [PDB]:** 1136
- **2D identity (%) [PDB]:** 10.25
- **2D identity (%) [Gaps excluded][PDB]:** 85.95
- **2D identity - Alignment Gaps [PDB]:** 894
- **3D similarity (TM-Score) (%) [PDB]:** 7.05

- **Gene name:** ISG15
- **RefSeq ID:** NM\_005101
- **Transcript sequence length:** 637
- **5-UTR|CDS|3-UTR identity (%):** 21.32 | 8.61 | 20.43
- **5-UTR|CDS|3-UTR identity (%) [Gaps excluded]:** 82.86 | 78.09 | 77.05
- **5-UTR|CDS|3-UTR identity [Alignment Gaps]:** 202 | 3462 | 169

**Uniprot Description:**  
  
Ubiquitin-like protein which plays a key role in the innate immune response to viral infection either via its conjugation to a target protein (ISGylation) or via its action as a free or unconjugated protein. ISGylation involves a cascade of enzymatic reactions involving E1, E2, and E3 enzymes which catalyze the conjugation of ISG15 to a lysine residue in the target protein. Its target proteins include IFIT1, MX1/MxA, PPM1B, UBE2L6, UBA7, CHMP5, CHMP2A, CHMP4B and CHMP6. Can also isgylate: EIF2AK2/PKR which results in its activation, DDX58/RIG-I which inhibits its function in antiviral signaling response, EIF4E2 which enhances its cap structure-binding activity and translation-inhibition activity, UBE2N and UBE2E1 which negatively regulates their activity, IRF3 which inhibits its ubiquitination and degradation and FLNB which prevents its ability to interact with the upstream activators of the JNK cascade thereby inhibiting IFNA-induced JNK signaling. Exhibits antiviral activity towards both DNA and RNA viruses, including influenza A, HIV-1 and Ebola virus. Restricts HIV-1 and ebola virus via disruption of viral budding. Inhibits the ubiquitination of HIV-1 Gag and host TSG101 and disrupts their interaction, thereby preventing assembly and release of virions from infected cells. Inhibits Ebola virus budding mediated by the VP40 protein by disrupting ubiquitin ligase activity of NEDD4 and its ability to ubiquitinate VP40. ISGylates influenza A virus NS1 protein which causes a loss of function of the protein and the inhibition of virus replication. The secreted form of ISG15 can: induce natural killer cell proliferation, act as a chemotactic factor for neutrophils and act as a IFN-gamma-inducing cytokine playing an essential role in antimycobacterial immunity. The secreted form acts through the integrin ITGAL/ITGB2 receptor to initiate activation of SRC family tyrosine kinases including LYN, HCK and FGR which leads to secretion of IFNG and IL10; the interaction is mediated by ITGAL (PubMed:29100055).  
  
Homodimer; disulfide-linked (PubMed:2440890). Interacts with, and is conjugated to its targets by UBE1L (E1 enzyme) and UBE2E2 (E2 enzyme) (PubMed:11157743, PubMed:15131269). Interacts with NEDD4 (PubMed:18305167).  
  
**Gene Ontology Information:**

Molecular Function

- integrin binding
- protein tag
- ubiquitin protein ligase binding

Location

- cytoplasm
- cytosol
- cytosolic small ribosomal subunit
- extracellular region
- nucleoplasm
- nucleus

Biological process

- defense response to bacterium
- defense response to virus
- integrin-mediated signaling pathway
- ISG15-protein conjugation
- modification-dependent protein catabolic process
- negative regulation of protein ubiquitination
- negative regulation of type I interferon production
- negative regulation of type I interferon-mediated signaling pathway
- negative regulation of viral genome replication
- positive regulation of bone mineralization
- positive regulation of erythrocyte differentiation
- positive regulation of interferon-gamma production
- positive regulation of interleukin-10 production
- regulation of interferon-gamma production
- response to type I interferon
- response to virus
- translesion synthesis
- type I interferon signaling pathway
- viral process

---

19

- **Protein name:** B-cell lymphoma 6 protein
- **Organism:** Homo sapiens
- **Uniprot Accession Number:** P41182
- **Protein sequence length:** 706 aa
- **1D identity (%):** 12.04
- **1D identity (%) [Gaps excluded]:** 28.21
- **1D identity - Alignment Gaps:** 795
- **Common reported functions (%):** 50.0
- **Common reported locations (%):** 0.0
- **Common reported processes (%):** 0.0

- **PDB ID:** 5H7G
- **Chain:** B
- **Crystallized protein length:** 122 aa
- **Resolution:** 1.85 Å
- **Associated domain:** BTB
- **b-phipsi:** 0.055533
- **w-rdist:** 1.00683
- **t-alpha:** 0.0
- **Chemical similarity (Tanimoto Index) (%):** 72.73
- **1D identity (%) [PDB]:** 0.09
- **1D identity (%) [Gaps excluded][PDB]:** 100.0
- **1D identity - Alignment Gaps [PDB]:** 1103
- **2D identity (%) [PDB]:** 8.51
- **2D identity (%) [Gaps excluded][PDB]:** 90.53
- **2D identity - Alignment Gaps [PDB]:** 915
- **3D similarity (TM-Score) (%) [PDB]:** 6.13

- **Gene name:** BCL6
- **RefSeq ID:** N/A
- **Sequence length:** N/A
- **5-UTR|CDS|3-UTR identity (%):** N/A | N/A | N/A
- **5-UTR|CDS|3-UTR identity (%) [Gaps excluded]:** N/A | N/A | N/A
- **5-UTR|CDS|3-UTR identity [Alignment Gaps]:** N/A | N/A | N/A

**Uniprot Description:**  
  
Transcriptional repressor mainly required for germinal center (GC) formation and antibody affinity maturation which has different mechanisms of action specific to the lineage and biological functions. Forms complexes with different corepressors and histone deacetylases to repress the transcriptional expression of different subsets of target genes. Represses its target genes by binding directly to the DNA sequence 5'-TTCCTAGAA-3' (BCL6-binding site) or indirectly by repressing the transcriptional activity of transcription factors. In GC B-cells, represses genes that function in differentiation, inflammation, apoptosis and cell cycle control, also autoregulates its transcriptional expression and up-regulates, indirectly, the expression of some genes important for GC reactions, such as AICDA, through the repression of microRNAs expression, like miR155. An important function is to allow GC B-cells to proliferate very rapidly in response to T-cell dependent antigens and tolerate the physiological DNA breaks required for immunglobulin class switch recombination and somatic hypermutation without inducing a p53/TP53-dependent apoptotic response. In follicular helper CD4(+) T-cells (T(FH) cells), promotes the expression of T(FH)-related genes but inhibits the differentiation of T(H)1, T(H)2 and T(H)17 cells. Also required for the establishment and maintenance of immunological memory for both T- and B-cells. Suppresses macrophage proliferation through competition with STAT5 for STAT-binding motifs binding on certain target genes, such as CCL2 and CCND2. In response to genotoxic stress, controls cell cycle arrest in GC B-cells in both p53/TP53-dependedent and -independent manners. Besides, also controls neurogenesis through the alteration of the composition of NOTCH-dependent transcriptional complexes at selective NOTCH targets, such as HES5, including the recruitment of the deacetylase SIRT1 and resulting in an epigenetic silencing leading to neuronal differentiation.  
  
Homodimer. Interacts (via BTB domain) with the corepressors BCOR, NCOR1 and SMRT/NCOR2; the interactions are direct. Forms preferably ternary complexes with BCOR and SMRT/NCOR2 on target gene promoters but, on enhancer elements, interacts with SMRT/NCOR2 and HDAC3 to repress proximal gene expression. Interacts with histone deacetylases HDAC2, HDAC5 and HDAC9 (via the catalytic domain). Interacts with ZBTB7 and BCL6B. Interacts with SCF(FBXO11) complex; the interaction is independent of phosphorylation and promotes ubiquitination. Interacts (when phosphorylated) with PIN1; the interaction is required for BCL6 degradation upon genotoxic stress. Interacts with ZBTB17; inhibits ZBTB17 transcriptional activity. Interacts with CTBP1, autoinhibits its transcriptional expression. Interacts with NOTCH1 NCID and SIRT1; leads to a epigenetic repression of selective NOTCH1-target genes. Interacts (nor via BTB domain neither acetylated) with the NuRD complex components CHD4, HDAC1, MBD3 and MTA3; the interaction with MTA3 inhibits BCL6 acetylation and is required for BCL6 transpriptional repression.  
  
**Gene Ontology Information:**

Molecular Function

- chromatin binding
- chromatin DNA binding
- DNA-binding transcription factor activity
- DNA-binding transcription repressor activity, RNA polymerase II-specific
- identical protein binding
- intronic transcription regulatory region sequence-specific DNA binding
- metal ion binding
- RNA polymerase II cis-regulatory region sequence-specific DNA binding
- sequence-specific DNA binding
- sequence-specific double-stranded DNA binding

Location

- Golgi apparatus
- nucleolus
- nucleoplasm
- nucleus

Biological process

- actin cytoskeleton organization
- B cell differentiation
- cell morphogenesis
- cellular response to DNA damage stimulus
- cytokine-mediated signaling pathway
- erythrocyte development
- germinal center formation
- inflammatory response
- negative regulation of B cell apoptotic process
- negative regulation of cell growth
- negative regulation of cell population proliferation
- negative regulation of cell-matrix adhesion
- negative regulation of cellular senescence
- negative regulation of isotype switching to IgE isotypes
- negative regulation of mast cell cytokine production
- negative regulation of mitotic cell cycle DNA replication
- negative regulation of Notch signaling pathway
- negative regulation of Rho protein signal transduction
- negative regulation of T-helper 2 cell differentiation
- negative regulation of transcription by RNA polymerase II
- negative regulation of transcription, DNA-templated
- positive regulation of apoptotic process
- positive regulation of B cell proliferation
- positive regulation of cellular component movement
- positive regulation of histone deacetylation
- positive regulation of neuron differentiation
- positive regulation of regulatory T cell differentiation
- protein localization
- regulation of apoptotic process
- regulation of cell differentiation
- regulation of cell population proliferation
- regulation of cytokine production
- regulation of germinal center formation
- regulation of GTPase activity
- regulation of immune response
- regulation of immune system process
- regulation of inflammatory response
- regulation of memory T cell differentiation
- regulation of transcription by RNA polymerase II
- Rho protein signal transduction
- spermatogenesis
- type 2 immune response

---

20

- **Protein name:** Peptidyl-prolyl cis-trans isomerase A
- **Organism:** Homo sapiens
- **Uniprot Accession Number:** P62937
- **Protein sequence length:** 165 aa
- **1D identity (%):** 3.52
- **1D identity (%) [Gaps excluded]:** 34.59
- **1D identity - Alignment Gaps:** 1172
- **Common reported functions (%):** 0.0
- **Common reported locations (%):** 0.0
- **Common reported processes (%):** 10.0

- **PDB ID:** 1M9C
- **Chain:** B
- **Crystallized protein length:** 163 aa
- **Resolution:** 2.0 Å
- **Associated domain:** PPIase-cyclophilin-type
- **b-phipsi:** 0.096585
- **w-rdist:** 0.870991
- **t-alpha:** 0.0
- **Chemical similarity (Tanimoto Index) (%):** 82.89
- **1D identity (%) [PDB]:** 0.0
- **1D identity (%) [Gaps excluded][PDB]:** 0.0
- **1D identity - Alignment Gaps [PDB]:** 1146
- **2D identity (%) [PDB]:** 11.09
- **2D identity (%) [Gaps excluded][PDB]:** 88.98
- **2D identity - Alignment Gaps [PDB]:** 892
- **3D similarity (TM-Score) (%) [PDB]:** 7.2

- **Gene name:** PPIA
- **RefSeq ID:** NM\_021130
- **Transcript sequence length:** 2237
- **5-UTR|CDS|3-UTR identity (%):** 10.14 | 8.96 | 9.28
- **5-UTR|CDS|3-UTR identity (%) [Gaps excluded]:** 84.85 | 84.34 | 75.71
- **5-UTR|CDS|3-UTR identity [Alignment Gaps]:** 243 | 3490 | 1504

**Uniprot Description:**  
  
Catalyzes the cis-trans isomerization of proline imidic peptide bonds in oligopeptides (PubMed:2001362, PubMed:20676357, PubMed:21245143, PubMed:25678563, PubMed:21593166). Exerts a strong chemotactic effect on leukocytes partly through activation of one of its membrane receptors BSG/CD147, initiating a signaling cascade that culminates in MAPK/ERK activation (PubMed:11943775, PubMed:21245143). Activates endothelial cells (ECs) in a proinflammatory manner by stimulating activation of NF-kappa-B and ERK, JNK and p38 MAP-kinases and by inducing expression of adhesion molecules including SELE and VCAM1 (PubMed:15130913). Induces apoptosis in ECs by promoting the FOXO1-dependent expression of CCL2 and BCL2L11 which are involved in EC chemotaxis and apoptosis (PubMed:31063815). In response to oxidative stress, initiates proapoptotic and antiapoptotic signaling in ECs via activation of NF-kappa-B and AKT1 and up-regulation of antiapoptotic protein BCL2 (PubMed:23180369). Negatively regulates MAP3K5/ASK1 kinase activity, autophosphorylation and oxidative stress-induced apoptosis mediated by MAP3K5/ASK1 (PubMed:26095851). Necessary for the assembly of TARDBP in heterogeneous nuclear ribonucleoprotein (hnRNP) complexes and regulates TARDBP binding to RNA UG repeats and TARDBP-dependent expression of HDAC6, ATG7 and VCP which are involved in clearance of protein aggregates (PubMed:25678563). Plays an important role in platelet activation and aggregation (By similarity). Regulates calcium mobilization and integrin ITGA2B:ITGB3 bidirectional signaling via increased ROS production as well as by facilitating the interaction between integrin and the cell cytoskeleton (By similarity). Binds heparan sulfate glycosaminoglycans (PubMed:11943775). Inhibits replication of influenza A virus (IAV) (PubMed:19207730). Inhibits ITCH/AIP4-mediated ubiquitination of matrix protein 1 (M1) of IAV by impairing the interaction of ITCH/AIP4 with M1, followed by the suppression of the nuclear export of M1, and finally reduction of the replication of IAV (PubMed:30328013, PubMed:22347431).  
  
Interacts with protein phosphatase PPP3CA/calcineurin A (PubMed:12218175, PubMed:12357034). Interacts with PRPF19 isoform 2 (via N-terminus) (By similarity). Interacts with isoform 2 of BSG/CD147 (PubMed:15688292, PubMed:11353871, PubMed:11943775, PubMed:21245143). Interacts with FOXO1; the interaction promotes FOXO1 dephosphorylation, nuclear accumulation and transcriptional activity (PubMed:31063815). Interacts with integrin ITGA2B:ITGB3; the interaction is ROS and peptidyl-prolyl cis-trans isomerase (PPIase) activity-dependent and is increased in the presence of thrombin (By similarity). Interacts with MAP3K5 (PubMed:26095851). Interacts with TARDBP; the interaction is dependent on the RNA-binding activity of TARDBP and the PPIase activity of PPIA/CYPA and the acetylation of PPIA/CYPA at Lys-125 favors the interaction (PubMed:25678563). Interacts with HNRNPA1, HNRNPA2B1, HNRNPC, RBMX, HNRNPK and HNRNPM (PubMed:25678563).  
  
**Gene Ontology Information:**

Molecular Function

- cyclosporin A binding
- heparan sulfate binding
- integrin binding
- peptidyl-prolyl cis-trans isomerase activity
- RNA binding
- unfolded protein binding
- virion binding

Location

- cytoplasm
- cytosol
- extracellular exosome
- extracellular region
- extracellular space
- ficolin-1-rich granule lumen
- focal adhesion
- intracellular membrane-bounded organelle
- membrane
- nucleus
- protein-containing complex
- secretory granule lumen
- vesicle

Biological process

- activation of MAPK activity
- activation of protein kinase B activity
- apoptotic process
- cell adhesion molecule production
- cellular response to oxidative stress
- endothelial cell activation
- entry into host
- establishment of integrated proviral latency
- fusion of virus membrane with host plasma membrane
- interleukin-12-mediated signaling pathway
- leukocyte chemotaxis
- leukocyte migration
- lipid droplet organization
- negative regulation of oxidative stress-induced intrinsic apoptotic signaling pathway
- negative regulation of protein K48-linked ubiquitination
- negative regulation of protein kinase activity
- negative regulation of protein phosphorylation
- negative regulation of stress-activated MAPK cascade
- negative regulation of viral life cycle
- neutrophil chemotaxis
- neutrophil degranulation
- platelet activation
- platelet aggregation
- positive regulation of NF-kappaB transcription factor activity
- positive regulation of protein dephosphorylation
- positive regulation of protein phosphorylation
- positive regulation of protein secretion
- positive regulation of viral genome replication
- protein folding
- protein peptidyl-prolyl isomerization
- regulation of apoptotic signaling pathway
- regulation of viral genome replication
- RNA-dependent DNA biosynthetic process
- uncoating of virus
- viral life cycle
- viral release from host cell
- virion assembly

---

21

- **Protein name:** Polymerase cofactor VP35
- **Organism:** Reston ebolavirus (strain Reston-89)
- **Uniprot Accession Number:** Q8JPY0
- **Protein sequence length:** 329 aa
- **1D identity (%):** 5.62
- **1D identity (%) [Gaps excluded]:** 25.96
- **1D identity - Alignment Gaps:** 1032
- **Common reported functions (%):** 0.0
- **Common reported locations (%):** 0.0
- **Common reported processes (%):** 0.0

- **PDB ID:** 3KS8
- **Chain:** B
- **Crystallized protein length:** 124 aa
- **Resolution:** 2.4 Å
- **Associated domain:** VP35-IID
- **b-phipsi:** 0.064991
- **w-rdist:** 0.862621
- **t-alpha:** 0.001709
- **Chemical similarity (Tanimoto Index) (%):** 83.23
- **1D identity (%) [PDB]:** 0.0
- **1D identity (%) [Gaps excluded][PDB]:** 0.0
- **1D identity - Alignment Gaps [PDB]:** 1107
- **2D identity (%) [PDB]:** 7.21
- **2D identity (%) [Gaps excluded][PDB]:** 92.5
- **2D identity - Alignment Gaps [PDB]:** 947
- **3D similarity (TM-Score) (%) [PDB]:** 7.6

- **Gene name:** VP35
- **RefSeq ID:** NC\_004161
- **Genomic sequence length:** 18891
- **5-UTR|CDS|3-UTR identity (%):** 13.55 | 16.83 | N/A
- **5-UTR|CDS|3-UTR identity (%) [Gaps excluded]:** 78.72 | 79.33 | N/A
- **5-UTR|CDS|3-UTR identity [Alignment Gaps]:** 226 | 3128 | N/A

**Uniprot Description:**  
  
Plays an essential role in viral RNA synthesis and also a role in suppressing innate immune signaling. Acts as a polymerase cofactor in the RNA polymerase transcription and replication complexes (By similarity). Serves as nucleoprotein/NP monomer chaperone prior to the formation of the large oligomeric RNA-bound complexes (By similarity). Regulates RNA synthesis by modulating NP-RNA interactions and interacting with DYNLL1. VP35-NP interaction controls the switch between RNA-bound NP and free NP and thus the switch between genome replication and genome packaging into the nucleocapsid. Prevents establishment of cellular antiviral state, thereby suppressing host DC maturation. Acts by inhibiting host DDX58/RIG-I activation both by shielding dsRNA from detection and by preventing PRKRA binding to DDX58. Blocks virus-induced phosphorylation and activation of interferon regulatory factor 3/IRF3, a transcription factor critical for the induction of interferons alpha and beta. This blockage is produced through the interaction with and inhibition of host IKBKE and TBK1, producing a strong inhibition of the phosphorylation and activation of IRF3. Also inhibits the antiviral effect mediated by the host interferon-induced, double-stranded RNA-activated protein kinase EIF2AK2/PKR. Increases PIAS1-mediated SUMOylation of IRF7, thereby repressing interferon transcription (By similarity). Also acts as a suppressor of RNA silencing by interacting with host DICER1, TARBP2/TRBP and PRKRA/PACT (By similarity). As a dimer, binds and sequesters dsRNA contributing to the inhibition of interferon production (PubMed:20018665).  
  
Homodimer (PubMed:20018665). Homooligomer; via the coiled coil domain (PubMed:30482729). Interacts with nucleoprotein NP and polymerase L; VP35 bridges L and NP and allows the formation of the polymerase complex. Also interacts with VP30; this interaction is regulated by VP30 phosphorylation. Interacts with host IKBKE and TBK1; the interactions lead to inhibition of cellular antiviral response by blocking necessary interactions of IKBKE and TBK1 with their substrate IRF3. Interacts with host DYNLL1; this interaction stabilizes VP35 N-terminal oligomerization domain, enhances viral RNA synthesis but does not participate in suppressing the host innate immune response. Interacts with host PRKRA; this interaction inhibits the interaction between DDX58 and PRKRA. Interacts with dsRNA. Interacts with host TRIM6; this interaction plays an important role in promoting efficient viral replication. Interacts with host STAU1. Interacts with host IRF7, PIAS1 and UBE2I/UBC9; these interactions mediate the sumoylation of IRF7 and contribute to the inhibition of IFN-type I production (By similarity). Interacts with host DICER1; this interaction prevents TARBP2/TRBP binding to DICER1 and thus allows the virus to counteract host RNA silencing. Interacts with host TARBP2/TRBP and PRKRA/PACT; these interactions prevent TARBP2 and PRKRA binding to DICER1 and thus allows the virus to counteract host RNA silencing (By similarity).  
  
**Gene Ontology Information:**

Molecular Function

- RNA binding

Location

- host cell cytoplasm
- virion

Biological process

- suppression by virus of host IKBKE activity
- suppression by virus of host IRF7 activity
- suppression by virus of host TBK1 activity
- suppression by virus of host toll-like receptor signaling pathway

---

22

- **Protein name:** RNA-directed RNA polymerase
- **Organism:** Pseudomonas phage phi6
- **Uniprot Accession Number:** P11124
- **Protein sequence length:** 665 aa
- **1D identity (%):** 9.98
- **1D identity (%) [Gaps excluded]:** 27.57
- **1D identity - Alignment Gaps:** 908
- **Common reported functions (%):** 0.0
- **Common reported locations (%):** 0.0
- **Common reported processes (%):** 637400.0

- **PDB ID:** 1HHT
- **Chain:** Q
- **Crystallized protein length:** 664 aa
- **Resolution:** 2.9 Å
- **Associated domain:** RdRp-catalytic
- **b-phipsi:** 0.076366
- **w-rdist:** 0.275751
- **t-alpha:** 0.022222
- **Chemical similarity (Tanimoto Index) (%):** N/A
- **1D identity (%) [PDB]:** 0.0
- **1D identity (%) [Gaps excluded][PDB]:** 0.0
- **1D identity - Alignment Gaps [PDB]:** 1647
- **2D identity (%) [PDB]:** 26.97
- **2D identity (%) [Gaps excluded][PDB]:** 93.75
- **2D identity - Alignment Gaps [PDB]:** 911
- **3D similarity (TM-Score) (%) [PDB]:** N/A

- **Gene name:** P2
- **RefSeq ID:** N/A
- **Sequence length:** NC\_003715
- **5-UTR|CDS|3-UTR identity (%):** N/A | 31.76 | N/A
- **5-UTR|CDS|3-UTR identity (%) [Gaps excluded]:** N/A | 77.29 | N/A
- **5-UTR|CDS|3-UTR identity [Alignment Gaps]:** N/A | 2430 | N/A

**Uniprot Description:**  
  
Rna-dependent RNA polymerase part of the packaging complex that packages the viral RNA segments, replicate them into a double-stranded form and transcribe them.  
  
Part of the packaging complex composed of RDRP, P4 and P7. Interacts with P7 (Probable).  
  
**Gene Ontology Information:**

Molecular Function

- metal ion binding
- nucleotide binding
- RNA binding
- RNA uridylyltransferase activity
- RNA-directed 5'-3' RNA polymerase activity

Location

- virion

Biological process

- transcription, DNA-templated
- viral RNA genome replication

---

23

- **Protein name:** Pol polyprotein
- **Organism:** Feline immunodeficiency virus (isolate Petaluma)
- **Uniprot Accession Number:** P16088
- **Protein sequence length:** 1124 aa
- **1D identity (%):** 4.81
- **1D identity (%) [Gaps excluded]:** 29.2
- **1D identity - Alignment Gaps:** 1719
- **Common reported functions (%):** 0.0
- **Common reported locations (%):** 0.0
- **Common reported processes (%):** 10.0

- **PDB ID:** 2HAH
- **Chain:** A
- **Crystallized protein length:** 112 aa
- **Resolution:** 1.7 Å
- **Associated domain:** Peptidase-A2
- **b-phipsi:** 0.002031
- **w-rdist:** 0.964221
- **t-alpha:** 0.477273
- **Chemical similarity (Tanimoto Index) (%):** 77.94
- **1D identity (%) [PDB]:** 0.0
- **1D identity (%) [Gaps excluded][PDB]:** 0.0
- **1D identity - Alignment Gaps [PDB]:** 1095
- **2D identity (%) [PDB]:** 9.26
- **2D identity (%) [Gaps excluded][PDB]:** 90.2
- **2D identity - Alignment Gaps [PDB]:** 891
- **3D similarity (TM-Score) (%) [PDB]:** 5.52

- **Gene name:** pol
- **RefSeq ID:** NC\_001482
- **Genomic sequence length:** 9474
- **5-UTR|CDS|3-UTR identity (%):** N/A | 40.89 | N/A
- **5-UTR|CDS|3-UTR identity (%) [Gaps excluded]:** N/A | 77.95 | N/A
- **5-UTR|CDS|3-UTR identity [Alignment Gaps]:** N/A | 2125 | N/A

**Uniprot Description:**  
  
During replicative cycle of retroviruses, the reverse-transcribed viral DNA is integrated into the host chromosome by the viral integrase enzyme. RNase H activity is associated with the reverse transcriptase.  
  
**Gene Ontology Information:**

Molecular Function

- aspartic-type endopeptidase activity
- DNA binding
- dUTP diphosphatase activity
- exoribonuclease H activity
- magnesium ion binding
- RNA-directed DNA polymerase activity
- RNA-DNA hybrid ribonuclease activity
- zinc ion binding

Location  
  
N/A

Biological process

- DNA integration
- DNA recombination
- dUMP biosynthetic process
- dUTP catabolic process
- establishment of integrated proviral latency
- viral entry into host cell
- viral genome integration into host DNA

---

24

- **Protein name:** HLA class I histocompatibility antigen, B alpha chain
- **Organism:** Homo sapiens
- **Uniprot Accession Number:** P01889
- **Protein sequence length:** 362 aa
- **1D identity (%):** 5.07
- **1D identity (%) [Gaps excluded]:** 25.27
- **1D identity - Alignment Gaps:** 1089
- **Common reported functions (%):** 0.0
- **Common reported locations (%):** 0.0
- **Common reported processes (%):** 0.0

- **PDB ID:** 2YPK
- **Chain:** A
- **Crystallized protein length:** 274 aa
- **Resolution:** 1.95 Å
- **Associated domain:** Ig-like-C1-type
- **b-phipsi:** 0.030743
- **w-rdist:** 0.957612
- **t-alpha:** 0.001712
- **Chemical similarity (Tanimoto Index) (%):** 85.12
- **1D identity (%) [PDB]:** 0.08
- **1D identity (%) [Gaps excluded][PDB]:** 50.0
- **1D identity - Alignment Gaps [PDB]:** 1253
- **2D identity (%) [PDB]:** 17.64
- **2D identity (%) [Gaps excluded][PDB]:** 85.98
- **2D identity - Alignment Gaps [PDB]:** 829
- **3D similarity (TM-Score) (%) [PDB]:** 10.64

- **Gene name:** HLA-B
- **RefSeq ID:** NM\_005514
- **Transcript sequence length:** 1536
- **5-UTR|CDS|3-UTR identity (%):** 6.79 | 16.87 | 33.71
- **5-UTR|CDS|3-UTR identity (%) [Gaps excluded]:** 85.71 | 75.47 | 72.95
- **5-UTR|CDS|3-UTR identity [Alignment Gaps]:** 244 | 3117 | 241

**Uniprot Description:**  
  
Antigen-presenting major histocompatibility complex class I (MHCI) molecule. In complex with B2M/beta 2 microglobulin displays primarily viral and tumor-derived peptides on antigen-presenting cells for recognition by alpha-beta T cell receptor (TCR) on HLA-B-restricted CD8-positive T cells, guiding antigen-specific T cell immune response to eliminate infected or transformed cells (PubMed:25808313, PubMed:29531227, PubMed:9620674, PubMed:23209413). May also present self-peptides derived from the signal sequence of secreted or membrane proteins, although T cells specific for these peptides are usually inactivated to prevent autoreactivity (PubMed:7743181, PubMed:18991276). Both the peptide and the MHC molecule are recognized by TCR, the peptide is responsible for the fine specificity of antigen recognition and MHC residues account for the MHC restriction of T cells (PubMed:29531227, PubMed:9620674, PubMed:24600035). Typically presents intracellular peptide antigens of 8 to 13 amino acids that arise from cytosolic proteolysis via constitutive proteasome and IFNG-induced immunoproteasome (PubMed:23209413). Can bind different peptides containing allele-specific binding motifs, which are mainly defined by anchor residues at position 2 and 9 (PubMed:25808313, PubMed:29531227).  
  
Heterotrimer that consists of an alpha chain HLA-B, a beta chain B2M and a peptide (peptide-HLA-B-B2M) (PubMed:25808313, PubMed:29531227, PubMed:15657948, PubMed:17057332, PubMed:22020283, PubMed:24600035). Early in biogenesis, HLA-B-B2M dimer interacts with the components of the peptide-loading complex composed of TAPBP, TAP1-TAP2, TAPBPL, PDIA3/ERP57 and CALR (PubMed:9036970, PubMed:9620674, PubMed:26439010, PubMed:26416272). Interacts with TAP1-TAP2 transporter via TAPBP; this interaction is obligatory for the loading of peptide epitopes delivered to the ER by TAP1-TAP2 transporter (PubMed:9036970, PubMed:9620674). Interacts with TAPBPL; TAPBPL binds peptide-free HLA-B-B2M complexes or those loaded with low affinity peptides, likely facilitating peptide exchange for higher affinity peptides (PubMed:26439010). Only optimally assembled peptide-HLA-B-B2M trimer translocates to the surface of antigen-presenting cells, where it interacts with TCR and CD8 coreceptor on the surface of T cells. HLA-B (via polymorphic alpha-1 and alpha-2 domains) interacts with antigen-specific TCR (via CDR1, CDR2 and CDR3 domains) (PubMed:29531227, PubMed:24600035). One HLA-B molecule (mainly via nonpolymorphic alpha-3 domain) interacts with one CD8A homodimer (via CDR-like loop); this interaction insures peptide-HLA-B-B2M recognition by CD8-positive T cells only (PubMed:29531227). Allele B\*57:01 interacts (via Bw4 motif) with KIR3DL1 (via Ig-like C2-type domain); this interaction may interfere with peptide binding (PubMed:22020283, PubMed:25480565). Allele B\*46:01 interacts with KIR2DL3 (PubMed:28514659).  
  
**Gene Ontology Information:**

Molecular Function

- chaperone binding
- peptide antigen binding
- signaling receptor binding
- TAP binding

Location

- cell surface
- early endosome membrane
- endoplasmic reticulum
- ER to Golgi transport vesicle membrane
- extracellular exosome
- Golgi apparatus
- Golgi membrane
- integral component of lumenal side of endoplasmic reticulum membrane
- integral component of plasma membrane
- membrane
- MHC class I protein complex
- phagocytic vesicle membrane
- plasma membrane
- recycling endosome membrane
- secretory granule membrane

Biological process

- adaptive immune response
- antigen processing and presentation of endogenous peptide antigen via MHC class I via ER pathway, TAP-independent
- antigen processing and presentation of exogenous peptide antigen via MHC class I, TAP-dependent
- antigen processing and presentation of exogenous peptide antigen via MHC class I, TAP-independent
- antigen processing and presentation of peptide antigen via MHC class I
- defense response
- detection of bacterium
- immune response
- interferon-gamma-mediated signaling pathway
- neutrophil degranulation
- positive regulation of T cell mediated cytotoxicity
- protection from natural killer cell mediated cytotoxicity
- regulation of dendritic cell differentiation
- regulation of immune response
- regulation of interleukin-12 production
- regulation of interleukin-6 production
- regulation of T cell anergy
- type I interferon signaling pathway
- viral process

---

25

- **Protein name:** Coxsackievirus and adenovirus receptor
- **Organism:** Homo sapiens
- **Uniprot Accession Number:** P78310
- **Protein sequence length:** 365 aa
- **1D identity (%):** 6.0
- **1D identity (%) [Gaps excluded]:** 30.26
- **1D identity - Alignment Gaps:** 1096
- **Common reported functions (%):** 50.0
- **Common reported locations (%):** 0.0
- **Common reported processes (%):** 0.0

- **PDB ID:** 2W9L
- **Chain:** K
- **Crystallized protein length:** 123 aa
- **Resolution:** 2.91 Å
- **Associated domain:** Ig-like-C2-type-1
- **b-phipsi:** 0.038786
- **w-rdist:** 0.892942
- **t-alpha:** 0.001712
- **Chemical similarity (Tanimoto Index) (%):** 85.43
- **1D identity (%) [PDB]:** 0.0
- **1D identity (%) [Gaps excluded][PDB]:** 0.0
- **1D identity - Alignment Gaps [PDB]:** 1106
- **2D identity (%) [PDB]:** 9.08
- **2D identity (%) [Gaps excluded][PDB]:** 87.5
- **2D identity - Alignment Gaps [PDB]:** 898
- **3D similarity (TM-Score) (%) [PDB]:** 8.42

- **Gene name:** CXADR
- **RefSeq ID:** N/A
- **Sequence length:** N/A
- **5-UTR|CDS|3-UTR identity (%):** N/A | N/A | N/A
- **5-UTR|CDS|3-UTR identity (%) [Gaps excluded]:** N/A | N/A | N/A
- **5-UTR|CDS|3-UTR identity [Alignment Gaps]:** N/A | N/A | N/A

**Uniprot Description:**  
  
Component of the epithelial apical junction complex that may function as a homophilic cell adhesion molecule and is essential for tight junction integrity. Also involved in transepithelial migration of leukocytes through adhesive interactions with JAML a transmembrane protein of the plasma membrane of leukocytes. The interaction between both receptors also mediates the activation of gamma-delta T-cells, a subpopulation of T-cells residing in epithelia and involved in tissue homeostasis and repair. Upon epithelial CXADR-binding, JAML induces downstream cell signaling events in gamma-delta T-cells through PI3-kinase and MAP kinases. It results in proliferation and production of cytokines and growth factors by T-cells that in turn stimulate epithelial tissues repair.  
  
Monomer. May form homodimer. Interacts with LNX, MAGI1, DLG4, PRKCABP, TJP1 and CTNNB1. Interacts with MPDZ; recruits MPDZ to intercellular contact sites. Interacts with JAML (homodimeric form). Secreted isoform 3, isoform 4 and isoform 5 can interact with the extracellular domain of the receptor.  
  
**Gene Ontology Information:**

Molecular Function

- beta-catenin binding
- cell adhesion molecule binding
- cell adhesive protein binding involved in AV node cell-bundle of His cell communication
- connexin binding
- identical protein binding
- integrin binding
- PDZ domain binding
- signaling receptor binding
- virus receptor activity

Location

- acrosomal vesicle
- adherens junction
- apicolateral plasma membrane
- basolateral plasma membrane
- bicellular tight junction
- cell body
- cell junction
- cell-cell junction
- cytoplasm
- extracellular region
- extracellular space
- filopodium
- growth cone
- integral component of plasma membrane
- intercalated disc
- membrane raft
- neuromuscular junction
- neuron projection
- nucleoplasm
- plasma membrane
- protein-containing complex

Biological process

- actin cytoskeleton reorganization
- AV node cell to bundle of His cell communication
- AV node cell-bundle of His cell adhesion involved in cell communication
- cardiac muscle fiber development
- cell-cell junction organization
- defense response to virus
- epithelial structure maintenance
- gamma-delta T cell activation
- germ cell migration
- heart development
- heterophilic cell-cell adhesion via plasma membrane cell adhesion molecules
- homotypic cell-cell adhesion
- leukocyte migration
- mitochondrion organization
- neutrophil chemotaxis
- regulation of AV node cell action potential
- regulation of immune response
- transepithelial transport

---

26

- **Protein name:** Elongin-B
- **Organism:** Homo sapiens
- **Uniprot Accession Number:** Q15370
- **Protein sequence length:** 118 aa
- **1D identity (%):** 2.48
- **1D identity (%) [Gaps excluded]:** 31.68
- **1D identity - Alignment Gaps:** 1189
- **Common reported functions (%):** 0.0
- **Common reported locations (%):** 0.0
- **Common reported processes (%):** 0.0

- **PDB ID:** 4N9F
- **Chain:** s
- **Crystallized protein length:** 95 aa
- **Resolution:** 3.3 Å
- **Associated domain:** Ubiquitin-like
- **b-phipsi:** 0.018469
- **w-rdist:** 1.04449
- **t-alpha:** 0.001712
- **Chemical similarity (Tanimoto Index) (%):** N/A
- **1D identity (%) [PDB]:** 0.0
- **1D identity (%) [Gaps excluded][PDB]:** 0.0
- **1D identity - Alignment Gaps [PDB]:** 1078
- **2D identity (%) [PDB]:** N/A
- **2D identity (%) [Gaps excluded][PDB]:** N/A
- **2D identity - Alignment Gaps [PDB]:** N/A
- **3D similarity (TM-Score) (%) [PDB]:** 11.24

- **Gene name:** ELOB
- **RefSeq ID:** NM\_207013
- **Transcript sequence length:** 585
- **5-UTR|CDS|3-UTR identity (%):** 10.29 | 7.58 | 18.99
- **5-UTR|CDS|3-UTR identity (%) [Gaps excluded]:** 82.35 | 78.63 | 90.0
- **5-UTR|CDS|3-UTR identity [Alignment Gaps]:** 238 | 3550 | 187

**Uniprot Description:**  
  
SIII, also known as elongin, is a general transcription elongation factor that increases the RNA polymerase II transcription elongation past template-encoded arresting sites. Subunit A is transcriptionally active and its transcription activity is strongly enhanced by binding to the dimeric complex of the SIII regulatory subunits B and C (elongin BC complex) (PubMed:7638163). In embryonic stem cells, the elongin BC complex is recruited by EPOP to Polycomb group (PcG) target genes in order generate genomic region that display both active and repressive chromatin properties, an important feature of pluripotent stem cells (By similarity).  
  
Heterotrimer of an A (ELOA, ELOA2 or ELOA3P), ELOB and ELOC subunit (PubMed:10205047, PubMed:17997974). The elongin BC complex interacts with EPOP; leading to recruit the elongin BC complex to Polycomb group (PcG) target genes, thereby restricting excessive activity of the PRC2/EED-EZH2 complex (By similarity). Part of E3 ubiquitin ligase complexes with CUL5 or CUL2, RBX1 and a substrate adapter protein that can be either SOCS1, SOCS5, ELOA, VHL or WSB1 (PubMed:15590694, PubMed:22286099). Interacts with VHL (PubMed:10205047, PubMed:11006129). Found in a complex composed of LIMD1, VHL, EGLN1/PHD2, ELOB and CUL2. Interacts with SPSB1 (PubMed:17189197). Interacts with KLHDC10; which may be an E3 ubiquitin ligase complex substrate recognition component (PubMed:23102700). May also interact with DCUN1D1, DCUN1D2, DCUN1D3 and DCUN1D5 (PubMed:26906416).  
  
**Gene Ontology Information:**

Molecular Function

- ubiquitin protein ligase binding

Location

- Cul2-RING ubiquitin ligase complex
- Cul5-RING ubiquitin ligase complex
- cytosol
- elongin complex
- nucleoplasm
- VCB complex

Biological process

- positive regulation of proteasomal ubiquitin-dependent protein catabolic process
- post-translational protein modification
- protein ubiquitination
- protein-containing complex assembly
- regulation of transcription from RNA polymerase II promoter in response to hypoxia
- transcription by RNA polymerase II
- transcription elongation from RNA polymerase II promoter
- viral process

---

27

- **Protein name:** Ubiquitin-40S ribosomal protein S27a
- **Organism:** Bos taurus
- **Uniprot Accession Number:** P62992
- **Protein sequence length:** 156 aa
- **1D identity (%):** 1.86
- **1D identity (%) [Gaps excluded]:** 17.52
- **1D identity - Alignment Gaps:** 1155
- **Common reported functions (%):** 0.0
- **Common reported locations (%):** 0.0
- **Common reported processes (%):** 0.0

- **PDB ID:** 4M0W
- **Chain:** B
- **Crystallized protein length:** 76 aa
- **Resolution:** 1.4 Å
- **Associated domain:** Ubiquitin-like
- **b-phipsi:** 0.022545
- **w-rdist:** 1.027513
- **t-alpha:** 0.001712
- **Chemical similarity (Tanimoto Index) (%):** 70.88
- **1D identity (%) [PDB]:** 0.0
- **1D identity (%) [Gaps excluded][PDB]:** 0.0
- **1D identity - Alignment Gaps [PDB]:** 1059
- **2D identity (%) [PDB]:** 4.49
- **2D identity (%) [Gaps excluded][PDB]:** 80.36
- **2D identity - Alignment Gaps [PDB]:** 947
- **3D similarity (TM-Score) (%) [PDB]:** 5.18

- **Gene name:** RPS27A
- **RefSeq ID:** N/A
- **Sequence length:** N/A
- **5-UTR|CDS|3-UTR identity (%):** N/A | N/A | N/A
- **5-UTR|CDS|3-UTR identity (%) [Gaps excluded]:** N/A | N/A | N/A
- **5-UTR|CDS|3-UTR identity [Alignment Gaps]:** N/A | N/A | N/A

**Uniprot Description:**  
  
Ubiquitin
Exists either covalently attached to another protein, or free (unanchored). When covalently bound, it is conjugated to target proteins via an isopeptide bond either as a monomer (monoubiquitin), a polymer linked via different Lys residues of the ubiquitin (polyubiquitin chains) or a linear polymer linked via the initiator Met of the ubiquitin (linear polyubiquitin chains). Polyubiquitin chains, when attached to a target protein, have different functions depending on the Lys residue of the ubiquitin that is linked: Lys-6-linked may be involved in DNA repair; Lys-11-linked is involved in ERAD (endoplasmic reticulum-associated degradation) and in cell-cycle regulation; Lys-29-linked is involved in lysosomal degradation; Lys-33-linked is involved in kinase modification; Lys-48-linked is involved in protein degradation via the proteasome; Lys-63-linked is involved in endocytosis, DNA-damage responses as well as in signaling processes leading to activation of the transcription factor NF-kappa-B. Linear polymer chains formed via attachment by the initiator Met lead to cell signaling. Ubiquitin is usually conjugated to Lys residues of target proteins, however, in rare cases, conjugation to Cys or Ser residues has been observed. When polyubiquitin is free (unanchored-polyubiquitin), it also has distinct roles, such as in activation of protein kinases, and in signaling (By similarity).  
  
Ribosomal protein S27a is part of the 40S ribosomal subunit.  
  
**Gene Ontology Information:**

Molecular Function

- metal ion binding
- protein tag
- structural constituent of ribosome
- ubiquitin protein ligase binding

Location

- cytoplasm
- cytosolic small ribosomal subunit
- nucleus

Biological process

- modification-dependent protein catabolic process
- protein ubiquitination
- translation

---

28

- **Protein name:** Envelope glycoprotein gp160
- **Organism:** Human immunodeficiency virus 1
- **Uniprot Accession Number:** Q2N0S7
- **Protein sequence length:** 860 aa
- **1D identity (%):** 12.29
- **1D identity (%) [Gaps excluded]:** 30.61
- **1D identity - Alignment Gaps:** 911
- **Common reported functions (%):** 50.0
- **Common reported locations (%):** 50.0
- **Common reported processes (%):** 30.0

- **PDB ID:** 6DE7
- **Chain:** B
- **Crystallized protein length:** 129 aa
- **Resolution:** 4.12 Å
- **Associated domain:** GP41
- **b-phipsi:** 0.152618
- **w-rdist:** 0.261307
- **t-alpha:** 0.054054
- **Chemical similarity (Tanimoto Index) (%):** N/A
- **1D identity (%) [PDB]:** 0.0
- **1D identity (%) [Gaps excluded][PDB]:** 0.0
- **1D identity - Alignment Gaps [PDB]:** 1113
- **2D identity (%) [PDB]:** 9.06
- **2D identity (%) [Gaps excluded][PDB]:** 94.85
- **2D identity - Alignment Gaps [PDB]:** 919
- **3D similarity (TM-Score) (%) [PDB]:** 7.06

- **Gene name:** env
- **RefSeq ID:** N/A
- **Sequence length:** N/A
- **5-UTR|CDS|3-UTR identity (%):** N/A | N/A | N/A
- **5-UTR|CDS|3-UTR identity (%) [Gaps excluded]:** N/A | N/A | N/A
- **5-UTR|CDS|3-UTR identity [Alignment Gaps]:** N/A | N/A | N/A

**Uniprot Description:**  
  
Envelope glycoprotein gp160: Oligomerizes in the host endoplasmic reticulum into predominantly trimers. In a second time, gp160 transits in the host Golgi, where glycosylation is completed. The precursor is then proteolytically cleaved in the trans-Golgi and thereby activated by cellular furin or furin-like proteases to produce gp120 and gp41.  
  
The mature envelope protein (Env) consists of a homotrimer of non-covalently associated gp120-gp41 heterodimers. The resulting complex protrudes from the virus surface as a spike. There seems to be as few as 10 spikes on the average virion. Surface protein gp120 interacts with host CD4, CCR5 and CXCR4. Gp120 also interacts with the C-type lectins CD209/DC-SIGN and CLEC4M/DC-SIGNR (collectively referred to as DC-SIGN(R)). Gp120 and gp41 interact with GalCer. Gp120 interacts with host ITGA4/ITGB7 complex; on CD4+ T-cells, this interaction results in rapid activation of integrin ITGAL/LFA-1, which facilitates efficient cell-to-cell spreading of HIV-1. Gp120 interacts with cell-associated heparan sulfate; this interaction increases virus infectivity on permissive cells and may be involved in infection of CD4- cells.  
  
**Gene Ontology Information:**

Molecular Function

- identical protein binding
- structural molecule activity

Location

- host cell endosome membrane
- host cell plasma membrane
- integral component of membrane
- viral envelope
- virion
- virion membrane

Biological process

- actin filament reorganization
- clathrin-dependent endocytosis of virus by host cell
- entry into host
- fusion of virus membrane with host endosome membrane
- fusion of virus membrane with host plasma membrane
- mitigation of host immune response by virus
- positive regulation of establishment of T cell polarity
- positive regulation of plasma membrane raft polarization
- positive regulation of receptor clustering
- stimulatory C-type lectin receptor signaling pathway
- viral life cycle
- viral protein processing
- virion assembly
- virion attachment to host cell

---

29

- **Protein name:** Interferon-induced helicase C domain-containing protein 1
- **Organism:** Mus musculus
- **Uniprot Accession Number:** Q8R5F7
- **Protein sequence length:** 1025 aa
- **1D identity (%):** 6.88
- **1D identity (%) [Gaps excluded]:** 26.98
- **1D identity - Alignment Gaps:** 1364
- **Common reported functions (%):** 50.0
- **Common reported locations (%):** 0.0
- **Common reported processes (%):** 0.0

- **PDB ID:** 6G1X
- **Chain:** A
- **Crystallized protein length:** 648 aa
- **Resolution:** 3.93 Å
- **Associated domain:** Helicase-C-terminal
- **b-phipsi:** 0.095333
- **w-rdist:** 0.279918
- **t-alpha:** 0.107692
- **Chemical similarity (Tanimoto Index) (%):** 83.23
- **1D identity (%) [PDB]:** 0.31
- **1D identity (%) [Gaps excluded][PDB]:** 71.43
- **1D identity - Alignment Gaps [PDB]:** 1628
- **2D identity (%) [PDB]:** 22.51
- **2D identity (%) [Gaps excluded][PDB]:** 90.52
- **2D identity - Alignment Gaps [PDB]:** 988
- **3D similarity (TM-Score) (%) [PDB]:** 16.14

- **Gene name:** Ifih1
- **RefSeq ID:** N/A
- **Sequence length:** N/A
- **5-UTR|CDS|3-UTR identity (%):** N/A | N/A | N/A
- **5-UTR|CDS|3-UTR identity (%) [Gaps excluded]:** N/A | N/A | N/A
- **5-UTR|CDS|3-UTR identity [Alignment Gaps]:** N/A | N/A | N/A

**Uniprot Description:**  
  
Innate immune receptor which acts as a cytoplasmic sensor of viral nucleic acids and plays a major role in sensing viral infection and in the activation of a cascade of antiviral responses including the induction of type I interferons and proinflammatory cytokines. Its ligands include mRNA lacking 2'-O-methylation at their 5' cap and long-dsRNA (>1 kb in length). Upon ligand binding it associates with mitochondria antiviral signaling protein (MAVS/IPS1) which activates the IKK-related kinases: TBK1 and IKBKE which phosphorylate interferon regulatory factors: IRF3 and IRF7 which in turn activate transcription of antiviral immunological genes, including interferons (IFNs); IFN-alpha and IFN-beta. Responsible for detecting the Picornaviridae family members such as encephalomyocarditis virus (EMCV), mengo encephalomyocarditis virus (ENMG), and theiler's murine encephalomyelitis virus (TMEV). Can also detect other viruses such as dengue virus (DENV), west Nile virus (WNV), and reovirus. Also involved in antiviral signaling in response to viruses containing a dsDNA genome, such as vaccinia virus. Plays an important role in amplifying innate immune signaling through recognition of RNA metabolites that are produced during virus infection by ribonuclease L (RNase L). May play an important role in enhancing natural killer cell function and may be involved in growth inhibition and apoptosis in several tumor cell lines.  
  
Monomer in the absence of ligands and homodimerizes in the presence of dsRNA ligands. Can assemble into helical or linear polymeric filaments on long dsRNA. Interacts with MAVS/IPS1. Interacts with PCBP2. Interacts with NLRC5. Interacts with PIAS2-beta. Interacts with DDX60. Interacts with ANKRD17. Interacts with IKBKE. Interacts (via the CARD domains) with TKFC, the interaction is inhibited by viral infection (By similarity). Interacts with ATG5 and ATG12, either as ATG5 and ATG12 monomers or as ATG12-ATG5 conjugates (By similarity). Interacts with ZCCHC3; leading to activate IFIH1/MDA5 (By similarity). Interacts with RNF123 (By similarity). Interacts with DDX3X (By similarity).  
  
**Gene Ontology Information:**

Molecular Function

- ATP binding
- DNA binding
- double-stranded RNA binding
- hydrolase activity
- identical protein binding
- ribonucleoprotein complex binding
- RNA helicase activity
- single-stranded RNA binding
- zinc ion binding

Location

- cytoplasm
- nucleus

Biological process

- cellular response to exogenous dsRNA
- defense response to virus
- innate immune response
- MDA-5 signaling pathway
- positive regulation of interferon-alpha production
- positive regulation of interferon-beta production
- positive regulation of interleukin-6 production
- positive regulation of response to cytokine stimulus
- positive regulation of tumor necrosis factor production
- protein sumoylation
- response to virus

---

30

- **Protein name:** Polymerase cofactor VP35
- **Organism:** Zaire ebolavirus (strain Mayinga-76)
- **Uniprot Accession Number:** Q05127
- **Protein sequence length:** 340 aa
- **1D identity (%):** 6.61
- **1D identity (%) [Gaps excluded]:** 29.29
- **1D identity - Alignment Gaps:** 1019
- **Common reported functions (%):** 0.0
- **Common reported locations (%):** 0.0
- **Common reported processes (%):** 0.0

- **PDB ID:** 3L26
- **Chain:** B
- **Crystallized protein length:** 123 aa
- **Resolution:** 2.4 Å
- **Associated domain:** VP35-IID
- **b-phipsi:** 0.057326
- **w-rdist:** 0.874189
- **t-alpha:** 0.001712
- **Chemical similarity (Tanimoto Index) (%):** 81.75
- **1D identity (%) [PDB]:** 0.09
- **1D identity (%) [Gaps excluded][PDB]:** 50.0
- **1D identity - Alignment Gaps [PDB]:** 1102
- **2D identity (%) [PDB]:** 7.02
- **2D identity (%) [Gaps excluded][PDB]:** 90.0
- **2D identity - Alignment Gaps [PDB]:** 946
- **3D similarity (TM-Score) (%) [PDB]:** 6.73

- **Gene name:** VP35
- **RefSeq ID:** NC\_002549
- **Genomic sequence length:** 18959
- **5-UTR|CDS|3-UTR identity (%):** 15.79 | 18.42 | 19.86
- **5-UTR|CDS|3-UTR identity (%) [Gaps excluded]:** 77.78 | 80.87 | 74.35
- **5-UTR|CDS|3-UTR identity [Alignment Gaps]:** 212 | 3047 | 524

**Uniprot Description:**  
  
Plays an essential role in viral RNA synthesis and also a role in suppressing innate immune signaling (PubMed:11027311). Acts as a polymerase cofactor in the RNA polymerase transcription and replication complexes (PubMed:9971816, PubMed:16495261, PubMed:24495995). Serves as nucleoprotein/NP monomer chaperone prior to the formation of the large oligomeric RNA-bound complexes (By similarity). Regulates RNA synthesis by modulating NP-RNA interactions and interacting with DYNLL1 (PubMed:25741013). VP35-NP interaction controls the switch between RNA-bound NP and free NP and thus the switch between genome replication and genome packaging into the nucleocapsid (PubMed:25865894). Prevents establishment of cellular antiviral state, thereby suppressing host DC maturation (PubMed:26962215). Acts by inhibiting host DDX58/RIG-I activation both by shielding dsRNA from detection and by preventing PRKRA binding to DDX58 (PubMed:23870315). Blocks virus-induced phosphorylation and activation of interferon regulatory factor 3/IRF3, a transcription factor critical for the induction of interferons alpha and beta (PubMed:12829834). This blockage is produced through the interaction with and inhibition of host IKBKE and TBK1, producing a strong inhibition of the phosphorylation and activation of IRF3 (PubMed:12829834). Also inhibits the antiviral effect mediated by the host interferon-induced, double-stranded RNA-activated protein kinase EIF2AK2/PKR (PubMed:17065211). Increases PIAS1-mediated SUMOylation of IRF7, thereby repressing interferon transcription (PubMed:19557165). Also acts as a suppressor of RNA silencing by interacting with host DICER1, TARBP2/TRBP and PRKRA/PACT (By similarity). As a dimer, binds and sequesters dsRNA contributing to the inhibition of interferon production (By similarity).  
  
Homodimer (By similarity). Homooligomer; via the coiled coil domain (PubMed:16095644). Interacts with nucleoprotein NP and polymerase L; VP35 bridges L and NP and allows the formation of the polymerase complex (PubMed:25865894) (Probable). Also interacts with VP30; this interaction is regulated by VP30 phosphorylation (PubMed:23493393). Interacts with host IKBKE and TBK1; the interactions lead to inhibition of cellular antiviral response by blocking necessary interactions of IKBKE and TBK1 with their substrate IRF3. Interacts with host DYNLL1; this interaction stabilizes VP35 N-terminal oligomerization domain, enhances viral RNA synthesis but does not participate in suppressing the host innate immune response (PubMed:19403681, PubMed:25741013). Interacts with host PRKRA; this interaction inhibits the interaction between DDX58 and PRKRA. Interacts with dsRNA (PubMed:19122151, PubMed:20071589, PubMed:23870315). Interacts with host TRIM6; this interaction plays an important role in promoting efficient viral replication (PubMed:28679761). Interacts with host STAU1 (PubMed:30301857). Interacts with host IRF7, PIAS1 and UBE2I/UBC9; these interactions mediate the sumoylation of IRF7 and contribute to the inhibition of IFN-type I production (PubMed:19557165). Interacts with host DICER1; this interaction prevents TARBP2/TRBP binding to DICER1 and thus allows the virus to counteract host RNA silencing (By similarity). Interacts with host TARBP2/TRBP and PRKRA/PACT; these interactions prevent TARBP2 and PRKRA binding to DICER1 and thus allows the virus to counteract host RNA silencing (By similarity).  
  
**Gene Ontology Information:**

Molecular Function

- RNA binding

Location

- host cell cytoplasm
- viral nucleocapsid

Biological process

- negative regulation of gene expression
- negative regulation of gene silencing by miRNA
- positive regulation of protein sumoylation
- suppression by virus of host antigen processing and presentation of peptide antigen via MHC class II
- suppression by virus of host cytokine production
- suppression by virus of host IKBKE activity
- suppression by virus of host IRF7 activity
- suppression by virus of host protein phosphorylation
- suppression by virus of host TBK1 activity
- suppression by virus of host toll-like receptor signaling pathway
- suppression by virus of host type I interferon production
- suppression of host defenses by symbiont

---

31

- **Protein name:** Tyrosine-protein kinase Fyn
- **Organism:** Homo sapiens
- **Uniprot Accession Number:** P06241
- **Protein sequence length:** 537 aa
- **1D identity (%):** 8.7
- **1D identity (%) [Gaps excluded]:** 27.91
- **1D identity - Alignment Gaps:** 950
- **Common reported functions (%):** 50.0
- **Common reported locations (%):** 0.0
- **Common reported processes (%):** 0.0

- **PDB ID:** 1EFN
- **Chain:** C
- **Crystallized protein length:** 57 aa
- **Resolution:** 2.5 Å
- **Associated domain:** SH3
- **b-phipsi:** 0.038499
- **w-rdist:** 1.109511
- **t-alpha:** 0.001709
- **Chemical similarity (Tanimoto Index) (%):** 79.36
- **1D identity (%) [PDB]:** 0.0
- **1D identity (%) [Gaps excluded][PDB]:** 0.0
- **1D identity - Alignment Gaps [PDB]:** 1040
- **2D identity (%) [PDB]:** 3.92
- **2D identity (%) [Gaps excluded][PDB]:** 86.67
- **2D identity - Alignment Gaps [PDB]:** 950
- **3D similarity (TM-Score) (%) [PDB]:** 3.62

- **Gene name:** FYN
- **RefSeq ID:** N/A
- **Sequence length:** N/A
- **5-UTR|CDS|3-UTR identity (%):** N/A | N/A | N/A
- **5-UTR|CDS|3-UTR identity (%) [Gaps excluded]:** N/A | N/A | N/A
- **5-UTR|CDS|3-UTR identity [Alignment Gaps]:** N/A | N/A | N/A

**Uniprot Description:**  
  
Non-receptor tyrosine-protein kinase that plays a role in many biological processes including regulation of cell growth and survival, cell adhesion, integrin-mediated signaling, cytoskeletal remodeling, cell motility, immune response and axon guidance. Inactive FYN is phosphorylated on its C-terminal tail within the catalytic domain. Following activation by PKA, the protein subsequently associates with PTK2/FAK1, allowing PTK2/FAK1 phosphorylation, activation and targeting to focal adhesions. Involved in the regulation of cell adhesion and motility through phosphorylation of CTNNB1 (beta-catenin) and CTNND1 (delta-catenin). Regulates cytoskeletal remodeling by phosphorylating several proteins including the actin regulator WAS and the microtubule-associated proteins MAP2 and MAPT. Promotes cell survival by phosphorylating AGAP2/PIKE-A and preventing its apoptotic cleavage. Participates in signal transduction pathways that regulate the integrity of the glomerular slit diaphragm (an essential part of the glomerular filter of the kidney) by phosphorylating several slit diaphragm components including NPHS1, KIRREL1 and TRPC6. Plays a role in neural processes by phosphorylating DPYSL2, a multifunctional adapter protein within the central nervous system, ARHGAP32, a regulator for Rho family GTPases implicated in various neural functions, and SNCA, a small pre-synaptic protein. Participates in the downstream signaling pathways that lead to T-cell differentiation and proliferation following T-cell receptor (TCR) stimulation. Phosphorylates PTK2B/PYK2 in response to T-cell receptor activation. Also participates in negative feedback regulation of TCR signaling through phosphorylation of PAG1, thereby promoting interaction between PAG1 and CSK and recruitment of CSK to lipid rafts. CSK maintains LCK and FYN in an inactive form. Promotes CD28-induced phosphorylation of VAV1. In mast cells, phosphorylates CLNK after activation of immunoglobulin epsilon receptor signaling (By similarity).  
  
Interacts (via its SH3 domain) with PIK3R1 and PRMT8. Interacts with FYB1, PAG1, and SH2D1A. Interacts with CD79A (tyrosine-phosphorylated form); the interaction increases FYN activity. Interacts (via SH2 domain) with CSF1R (tyrosine phosphorylated) (By similarity). Interacts with TOM1L1 (phosphorylated form). Interacts with KDR (tyrosine phosphorylated). Interacts (via SH3 domain) with KLHL2 (via N-terminus) (By similarity). Interacts with SH2D1A and SLAMF1. Interacts with ITCH; the interaction phosphorylates ITCH and negatively regulates its activity. Interacts with FASLG. Interacts with RUNX3. Interacts with KIT. Interacts with EPHA8; possible downstream effector of EPHA8 in regulation of cell adhesion. Interacts with PTK2/FAK1; this interaction leads to PTK2/FAK1 phosphorylation and activation. Interacts with CAV1; this interaction couples integrins to the Ras-ERK pathway. Interacts with UNC119. Interacts (via SH2 domain) with PTPRH (phosphorylated form) (By similarity). Interacts with PTPRO (phosphorylated form) (By similarity). Interacts with PTPRB (phosphorylated form) (By similarity). Interacts with FYB2 (PubMed:27335501). Interacts with DSCAM (By similarity). Interacts with SKAP1 and FYB1; this interaction promotes the phosphorylation of CLNK (By similarity).  
  
**Gene Ontology Information:**

Molecular Function

- alpha-tubulin binding
- ATP binding
- CD4 receptor binding
- CD8 receptor binding
- disordered domain specific binding
- enzyme binding
- ephrin receptor binding
- growth factor receptor binding
- identical protein binding
- ion channel binding
- metal ion binding
- non-membrane spanning protein tyrosine kinase activity
- peptide hormone receptor binding
- phosphatidylinositol 3-kinase binding
- phospholipase activator activity
- phospholipase binding
- protein tyrosine kinase activity
- signaling receptor binding
- T cell receptor binding
- tau protein binding
- tau-protein kinase activity
- transmembrane receptor protein tyrosine kinase activity
- type 5 metabotropic glutamate receptor binding

Location

- actin filament
- cell body
- cytosol
- dendrite
- endosome
- extrinsic component of cytoplasmic side of plasma membrane
- glial cell projection
- glutamatergic synapse
- membrane raft
- mitochondrion
- nucleus
- perinuclear endoplasmic reticulum
- perinuclear region of cytoplasm
- plasma membrane
- postsynaptic density
- postsynaptic density, intracellular component
- Schaffer collateral - CA1 synapse

Biological process

- activated T cell proliferation
- adaptive immune response
- axon guidance
- blood coagulation
- calcium ion transport
- cell differentiation
- cellular response to amyloid-beta
- cellular response to glycine
- cellular response to L-glutamate
- cellular response to peptide hormone stimulus
- cellular response to platelet-derived growth factor stimulus
- cellular response to transforming growth factor beta stimulus
- cytokine-mediated signaling pathway
- dendrite morphogenesis
- dendritic spine maintenance
- detection of mechanical stimulus involved in sensory perception of pain
- ephrin receptor signaling pathway
- Fc-gamma receptor signaling pathway involved in phagocytosis
- feeding behavior
- forebrain development
- heart process
- innate immune response
- intracellular signal transduction
- learning
- leukocyte migration
- MAPK cascade
- modulation of chemical synaptic transmission
- negative regulation of dendritic spine maintenance
- negative regulation of extrinsic apoptotic signaling pathway in absence of ligand
- negative regulation of gene expression
- negative regulation of hydrogen peroxide biosynthetic process
- negative regulation of inflammatory response to antigenic stimulus
- negative regulation of neuron apoptotic process
- negative regulation of oxidative stress-induced cell death
- negative regulation of protein catabolic process
- negative regulation of protein ubiquitination
- neuron migration
- peptidyl-tyrosine phosphorylation
- platelet activation
- positive regulation of cysteine-type endopeptidase activity
- positive regulation of I-kappaB kinase/NF-kappaB signaling
- positive regulation of neuron death
- positive regulation of neuron projection development
- positive regulation of non-membrane spanning protein tyrosine kinase activity
- positive regulation of phosphatidylinositol 3-kinase signaling
- positive regulation of protein kinase B signaling
- positive regulation of protein localization to membrane
- positive regulation of protein localization to nucleus
- positive regulation of protein targeting to membrane
- positive regulation of tyrosine phosphorylation of STAT protein
- protein autophosphorylation
- protein phosphorylation
- regulation of calcium ion import across plasma membrane
- regulation of cell shape
- regulation of defense response to virus by virus
- regulation of glutamate receptor signaling pathway
- regulation of peptidyl-tyrosine phosphorylation
- response to amyloid-beta
- response to drug
- response to ethanol
- response to hydrogen peroxide
- response to singlet oxygen
- stimulatory C-type lectin receptor signaling pathway
- T cell costimulation
- T cell receptor signaling pathway
- transmembrane receptor protein tyrosine kinase signaling pathway
- vascular endothelial growth factor receptor signaling pathway

---

32

- **Protein name:** Tail tube protein
- **Organism:** Escherichia phage lambda
- **Uniprot Accession Number:** P03733
- **Protein sequence length:** 246 aa
- **1D identity (%):** 4.32
- **1D identity (%) [Gaps excluded]:** 25.11
- **1D identity - Alignment Gaps:** 1073
- **Common reported functions (%):** 0.0
- **Common reported locations (%):** 0.0
- **Common reported processes (%):** 0.0

- **PDB ID:** 6P3E
- **Chain:** N
- **Crystallized protein length:** 246 aa
- **Resolution:** 5.4 Å
- **Associated domain:** BIG2
- **b-phipsi:** 0.055018
- **w-rdist:** 0.963581
- **t-alpha:** 0.001712
- **Chemical similarity (Tanimoto Index) (%):** N/A
- **1D identity (%) [PDB]:** 0.08
- **1D identity (%) [Gaps excluded][PDB]:** 50.0
- **1D identity - Alignment Gaps [PDB]:** 1225
- **2D identity (%) [PDB]:** 16.25
- **2D identity (%) [Gaps excluded][PDB]:** 89.42
- **2D identity - Alignment Gaps [PDB]:** 851
- **3D similarity (TM-Score) (%) [PDB]:** 9.84

- **Gene name:** V
- **RefSeq ID:** NC\_001416
- **Genomic sequence length:** 48502
- **5-UTR|CDS|3-UTR identity (%):** N/A | 12.14 | N/A
- **5-UTR|CDS|3-UTR identity (%) [Gaps excluded]:** N/A | 78.95 | N/A
- **5-UTR|CDS|3-UTR identity [Alignment Gaps]:** N/A | 3347 | N/A

**Uniprot Description:**  
  
Forms the phage's tail tube composed of 32 hexameric disks. When it encounters the appropriate initiation complex gpM and gpL, it assembles in hexameric rings that stack on top of each others. Multimerization ceases when the correct tail length is achieved through a mechanism dependent on tail terminator protein.  
  
Multimerizes into a structure formed by 32 hexameric rings stacked on phage baseplate gpM and gpL. Does not multimerize in solution without the tail initiation complex. Soluble major tail protein interacts with tail assembly protein GT during tail assembly.  
  
**Gene Ontology Information:**

Molecular Function  
  
N/A

Location

- host cell cytoplasm
- virus tail, tube

Biological process

- viral genome ejection through host cell envelope, long flexible tail mechanism
- viral tail assembly

---

33

- **Protein name:** Recombinase cre
- **Organism:** Escherichia phage P1
- **Uniprot Accession Number:** P06956
- **Protein sequence length:** 343 aa
- **1D identity (%):** 5.89
- **1D identity (%) [Gaps excluded]:** 24.92
- **1D identity - Alignment Gaps:** 998
- **Common reported functions (%):** 0.0
- **Common reported locations (%):** 0.0
- **Common reported processes (%):** 9480000.0

- **PDB ID:** 1OUQ
- **Chain:** F
- **Crystallized protein length:** 322 aa
- **Resolution:** 3.2 Å
- **Associated domain:** Core-binding-CB
- **b-phipsi:** 0.235416
- **w-rdist:** 0.925476
- **t-alpha:** 0.001712
- **Chemical similarity (Tanimoto Index) (%):** N/A
- **1D identity (%) [PDB]:** 0.08
- **1D identity (%) [Gaps excluded][PDB]:** 100.0
- **1D identity - Alignment Gaps [PDB]:** 1303
- **2D identity (%) [PDB]:** 17.21
- **2D identity (%) [Gaps excluded][PDB]:** 91.3
- **2D identity - Alignment Gaps [PDB]:** 891
- **3D similarity (TM-Score) (%) [PDB]:** N/A

- **Gene name:** cre
- **RefSeq ID:** N/A
- **Sequence length:** NC\_005856
- **5-UTR|CDS|3-UTR identity (%):** N/A | 17.47 | N/A
- **5-UTR|CDS|3-UTR identity (%) [Gaps excluded]:** N/A | 79.91 | N/A
- **5-UTR|CDS|3-UTR identity [Alignment Gaps]:** N/A | 3112 | N/A

**Uniprot Description:**  
  
Catalyzes site-specific recombination between two 34-base-pair LOXP sites. Its role is to maintain the phage genome as a monomeric unit-copy plasmid in the lysogenic state.  
  
Homotetramer when bound to DNA.  
  
**Gene Ontology Information:**

Molecular Function

- DNA binding

Location  
  
N/A

Biological process

- DNA integration
- DNA recombination

---

34

- **Protein name:** Viral CASP8 and FADD-like apoptosis regulator
- **Organism:** Molluscum contagiosum virus subtype 1
- **Uniprot Accession Number:** Q98325
- **Protein sequence length:** 241 aa
- **1D identity (%):** 4.26
- **1D identity (%) [Gaps excluded]:** 24.66
- **1D identity - Alignment Gaps:** 1068
- **Common reported functions (%):** 0.0
- **Common reported locations (%):** 0.0
- **Common reported processes (%):** 0.0

- **PDB ID:** 2BBZ
- **Chain:** A
- **Crystallized protein length:** 190 aa
- **Resolution:** 3.8 Å
- **Associated domain:** DED-1
- **b-phipsi:** 0.261881
- **w-rdist:** 1.039421
- **t-alpha:** 0.001709
- **Chemical similarity (Tanimoto Index) (%):** 72.27
- **1D identity (%) [PDB]:** 0.17
- **1D identity (%) [Gaps excluded][PDB]:** 66.67
- **1D identity - Alignment Gaps [PDB]:** 1167
- **2D identity (%) [PDB]:** 12.82
- **2D identity (%) [Gaps excluded][PDB]:** 86.75
- **2D identity - Alignment Gaps [PDB]:** 871
- **3D similarity (TM-Score) (%) [PDB]:** 8.07

- **Gene name:** MC159L
- **RefSeq ID:** NC\_001731
- **Genomic sequence length:** 190289
- **5-UTR|CDS|3-UTR identity (%):** N/A | 12.58 | N/A
- **5-UTR|CDS|3-UTR identity (%) [Gaps excluded]:** N/A | 77.36 | N/A
- **5-UTR|CDS|3-UTR identity [Alignment Gaps]:** N/A | 3276 | N/A

**Uniprot Description:**  
  
Inhibits TNFRSF1A, TNFRSF6/FAS and TNFRSF12 induced apoptosis. Directs the degradation of host NFKBIB but not NFKBIA. Suppresses also host NF-kappa-B activation by interacting with and preventing ubiquitination of host NEMO/IKBKG, the NF-kappa-B essential modulator subunit of the IKK complex (PubMed:28515292). Interferes with host CASP8/caspase-8 recruitment and activation at the death-inducing signaling complex (DISC). May lead to higher virus production and contribute to virus persistence and oncogenicity. Participates also in the inhibition of host autophagy by interacting with host SH3BP4 (PubMed:30842330).  
  
Associates with the death-inducing signaling complex (DISC) formed by TNFRSF6/FAS, FADD and CASP8. Interacts with FADD (PubMed:16364918). Interacts with host TRAF2 (PubMed:16040075). Interacts with host NEMO/IKBKG (via N-terminus)(PubMed:28515292). Interacts with host SH3BP4; this interaction plays an important in the suppression of host autophagy (PubMed:30842330).  
  
**Gene Ontology Information:**

Molecular Function

- cysteine-type endopeptidase activity

Location

- host cell cytoplasm
- host cell nucleus

Biological process

- positive regulation of necroptotic process
- suppression by virus of host apoptotic process
- suppression by virus of host autophagy
- suppression by virus of host NF-kappaB transcription factor activity

---
