# Supplementary material for: Identifying and profiling structural similarities between Spike of SARS-CoV-2 and other viral or host proteins with Machaon
Source: Commun Biol. 2023 Jul 19;6:752. doi: 10.1038/s42003-023-05076-7 (PMC10356814; doi:10.1038/s42003-023-05076-7)
Supplement: Supplementary file 7 — Supplementary Data 4 [file 42003_2023_5076_MOESM7_ESM.zip › 6VXX_A_segment/candidates/6VXX_A_site0-metrics-merged-enriched_eval_report.html]

 

# Structural Comparison Report for 6VXX\_A\_site0 - segments (total: 78)

---

1

- **Protein name:** Spike glycoprotein
- **Organism:** Severe acute respiratory syndrome coronavirus
- **Uniprot Accession Number:** P59594
- **Protein sequence length:** 1255 aa
- **1D identity (%):** 76.35
- **1D identity (%) [Gaps excluded]:** 77.94
- **1D identity - Alignment Gaps:** 26
- **Common reported functions (%):** 100.0
- **Common reported locations (%):** 62.5
- **Common reported processes (%):** 90.0

- **PDB ID:** 6NB6
- **Chain:** B
- **Crystallized protein length:** 1026 aa
- **Resolution:** 4.2 Å
- **Alinged residues range:** 42-57, 199-208, 268-306, 342-440, 517-599, 717-755, 933-1001
- **Aligned to segment part (indices):** 3, 5, 6, 1, 4, 0, 2
- **Alinged residues range of reference:** 38-53, 204-215, 281-319, 355-449, 531-613, 735-773, 951-1019
- **b-phipsi:** 0.002062
- **w-rdist:** 0.453611
- **t-alpha:** 0.185853
- **Chemical similarity (Tanimoto Index) (%):** 94.56
- **1D identity (%) [PDB]:** 69.3
- **1D identity (%) [Gaps excluded][PDB]:** 79.09
- **1D identity - Alignment Gaps [PDB]:** 133
- **2D identity (%) [PDB]:** 75.4
- **2D identity (%) [Gaps excluded][PDB]:** 85.7
- **2D identity - Alignment Gaps [PDB]:** 129
- **3D similarity (TM-Score) (%) [PDB]:** 81.03

- **Gene name:** S
- **RefSeq ID:** NC\_004718
- **Genomic sequence length:** 29751
- **5-UTR|CDS|3-UTR identity (%):** 88.52 | 73.15 | 22.38
- **5-UTR|CDS|3-UTR identity (%) [Gaps excluded]:** 92.28 | 78.79 | 98.18
- **5-UTR|CDS|3-UTR identity [Alignment Gaps]:** 11 | 282 | 745

**Uniprot Description:**  
  
Spike glycoprotein
May down-regulate host tetherin (BST2) by lysosomal degradation, thereby counteracting its antiviral activity.  
  
Homotrimer; each monomer consists of a S1 and a S2 subunit. The resulting peplomers protrude from the virus surface as spikes (By similarity). Binds to human and palm civet ACE2 and human CLEC4M/DC-SIGNR. Interacts with the accessory proteins 3a and 7a.  
  
**Gene Ontology Information:**

Molecular Function

- host cell surface receptor binding
- identical protein binding

Location

- host cell endoplasmic reticulum-Golgi intermediate compartment membrane
- host cell plasma membrane
- integral component of membrane
- viral envelope
- virion membrane

Biological process

- endocytosis involved in viral entry into host cell
- fusion of virus membrane with host endosome membrane
- fusion of virus membrane with host plasma membrane
- pathogenesis
- receptor-mediated virion attachment to host cell
- suppression by virus of host tetherin activity
- suppression by virus of host type I interferon-mediated signaling pathway
- viral protein processing
- viral translation

---

2

- **Protein name:** Spike glycoprotein
- **Organism:** Bat coronavirus RaTG13
- **Uniprot Accession Number:** A0A6B9WHD3
- **Protein sequence length:** 1269 aa
- **1D identity (%):** 97.41
- **1D identity (%) [Gaps excluded]:** 97.71
- **1D identity - Alignment Gaps:** 4
- **Common reported functions (%):** 0.0
- **Common reported locations (%):** 50.0
- **Common reported processes (%):** 50.0

- **PDB ID:** 6ZGF
- **Chain:** A
- **Crystallized protein length:** 1060 aa
- **Resolution:** 3.1 Å
- **Alinged residues range:** 38-53, 195-228, 281-310, 355-438, 514-612, 757-769, 947-1015
- **Aligned to segment part (indices):** 3, 5, 6, 1, 4, 0, 2
- **Alinged residues range of reference:** 38-53, 195-228, 281-310, 355-438, 514-612, 761-773, 951-1019
- **b-phipsi:** 0.006343
- **w-rdist:** 0.199298
- **t-alpha:** 0.019883
- **Chemical similarity (Tanimoto Index) (%):** 100.0
- **1D identity (%) [PDB]:** 85.91
- **1D identity (%) [Gaps excluded][PDB]:** 94.08
- **1D identity - Alignment Gaps [PDB]:** 93
- **2D identity (%) [PDB]:** 68.41
- **2D identity (%) [Gaps excluded][PDB]:** 87.14
- **2D identity - Alignment Gaps [PDB]:** 247
- **3D similarity (TM-Score) (%) [PDB]:** 88.88

- **Gene name:** S
- **RefSeq ID:** N/A
- **Sequence length:** N/A
- **5-UTR|CDS|3-UTR identity (%):** N/A | N/A | N/A
- **5-UTR|CDS|3-UTR identity (%) [Gaps excluded]:** N/A | N/A | N/A
- **5-UTR|CDS|3-UTR identity [Alignment Gaps]:** N/A | N/A | N/A

**Uniprot Description:**  
  
Spike protein S1: attaches the virion to the cell membrane by interacting with host receptor, initiating the infection.  
  
Homotrimer; each monomer consists of a S1 and a S2 subunit. The resulting peplomers protrude from the virus surface as spikes.  
  
**Gene Ontology Information:**

Molecular Function  
  
N/A

Location

- host cell plasma membrane
- integral component of membrane
- viral envelope
- virion membrane

Biological process

- endocytosis involved in viral entry into host cell
- fusion of virus membrane with host endosome membrane
- fusion of virus membrane with host plasma membrane
- pathogenesis
- receptor-mediated virion attachment to host cell

---

3

- **Protein name:** Pre-glycoprotein polyprotein GP complex
- **Organism:** Lassa virus (strain Mouse/Sierra Leone/Josiah/1976)
- **Uniprot Accession Number:** P08669
- **Protein sequence length:** 491 aa
- **1D identity (%):** 1.42
- **1D identity (%) [Gaps excluded]:** 30.38
- **1D identity - Alignment Gaps:** 1606
- **Common reported functions (%):** 0.0
- **Common reported locations (%):** 50.0
- **Common reported processes (%):** 10.0

- **PDB ID:** 5VK2
- **Chain:** C
- **Crystallized protein length:** 185 aa
- **Resolution:** 3.2 Å
- **Alinged residues range:** 68-72, 223-228, 222-226, 67-71, 161-167, 132-136, 119-124
- **Aligned to segment part (indices):** 3, 5, 6, 1, 4, 0, 2
- **Alinged residues range of reference:** 48-52, 206-211, 312-316, 448-452, 607-613, 766-770, 985-990
- **b-phipsi:** 0.000593
- **w-rdist:** 1.877355
- **t-alpha:** 0.403941
- **Chemical similarity (Tanimoto Index) (%):** 94.55
- **1D identity (%) [PDB]:** 0.09
- **1D identity (%) [Gaps excluded][PDB]:** 50.0
- **1D identity - Alignment Gaps [PDB]:** 1166
- **2D identity (%) [PDB]:** 11.28
- **2D identity (%) [Gaps excluded][PDB]:** 87.97
- **2D identity - Alignment Gaps [PDB]:** 904
- **3D similarity (TM-Score) (%) [PDB]:** 7.63

- **Gene name:** GPC
- **RefSeq ID:** NC\_004296
- **Genomic sequence length:** 3402
- **5-UTR|CDS|3-UTR identity (%):** N/A | 25.44 | N/A
- **5-UTR|CDS|3-UTR identity (%) [Gaps excluded]:** N/A | 81.11 | N/A
- **5-UTR|CDS|3-UTR identity [Alignment Gaps]:** N/A | 2768 | N/A

**Uniprot Description:**  
  
Glycoprotein G1
interacts with the host receptor (By similarity). Mediates virus attachment to host receptor alpha-dystroglycan DAG1. This attachment induces virion internalization predominantly through clathrin- and caveolin-independent endocytosis (PubMed:11967329).  
  
Glycoprotein G1
homotetramer; disulfide-linked (By similarity). Interacts with host DAG1 (PubMed:11967329).  
  
**Gene Ontology Information:**

Molecular Function

- metal ion binding

Location

- host cell endoplasmic reticulum membrane
- host cell Golgi membrane
- host cell plasma membrane
- integral component of membrane
- viral envelope
- virion membrane

Biological process

- fusion of virus membrane with host endosome membrane
- receptor-mediated endocytosis of virus by host cell
- virion attachment to host cell

---

4

- **Protein name:** N/A
- **Organism:** N/A
- **Uniprot Accession Number:** Q14653
- **Protein sequence length:** N/A
- **1D identity (%):** N/A
- **1D identity (%) [Gaps excluded]:** N/A
- **1D identity - Alignment Gaps:** N/A
- **Common reported functions (%):** 50.0
- **Common reported locations (%):** 0.0
- **Common reported processes (%):** 0.0

- **PDB ID:** 5JER
- **Chain:** A
- **Crystallized protein length:** 234 aa
- **Resolution:** 2.91 Å
- **Alinged residues range:** 303-305, 204-207, 407-411, 205-208, 241-244, 330-333, 406-416
- **Aligned to segment part (indices):** 3, 5, 6, 1, 4, 0, 2
- **Alinged residues range of reference:** 38-40, 201-204, 298-302, 394-397, 593-596, 738-741, 954-963
- **b-phipsi:** 0.00031
- **w-rdist:** 3.88482
- **t-alpha:** 0.259205
- **Chemical similarity (Tanimoto Index) (%):** 83.83
- **1D identity (%) [PDB]:** 0.0
- **1D identity (%) [Gaps excluded][PDB]:** 0.0
- **1D identity - Alignment Gaps [PDB]:** 1217
- **2D identity (%) [PDB]:** 14.75
- **2D identity (%) [Gaps excluded][PDB]:** 89.02
- **2D identity - Alignment Gaps [PDB]:** 871
- **3D similarity (TM-Score) (%) [PDB]:** 1.02

- **Gene name:** N/A
- **RefSeq ID:** N/A
- **Sequence length:** N/A
- **5-UTR|CDS|3-UTR identity (%):** N/A | N/A | N/A
- **5-UTR|CDS|3-UTR identity (%) [Gaps excluded]:** N/A | N/A | N/A
- **5-UTR|CDS|3-UTR identity [Alignment Gaps]:** N/A | N/A | N/A

**Uniprot Description:**  
  
N/A  
  
**Gene Ontology Information:**

Molecular Function

- DNA binding
- DNA-binding transcription activator activity, RNA polymerase II-specific
- DNA-binding transcription factor activity, RNA polymerase II-specific
- DNA-binding transcription repressor activity, RNA polymerase II-specific
- identical protein binding
- protein domain specific binding
- protein homodimerization activity
- RNA polymerase II cis-regulatory region sequence-specific DNA binding
- sequence-specific DNA binding
- sequence-specific double-stranded DNA binding

Location

- chromatin
- cytoplasm
- cytosol
- mitochondrion
- nucleoplasm
- nucleus

Biological process

- apoptotic process
- cellular response to DNA damage stimulus
- cellular response to exogenous dsRNA
- cellular response to virus
- defense response to virus
- immune system process
- interferon-gamma-mediated signaling pathway
- lipopolysaccharide-mediated signaling pathway
- macrophage apoptotic process
- MDA-5 signaling pathway
- negative regulation of type I interferon production
- positive regulation of I-kappaB kinase/NF-kappaB signaling
- positive regulation of interferon-alpha production
- positive regulation of interferon-beta production
- positive regulation of transcription by RNA polymerase II
- positive regulation of type I interferon production
- positive regulation of type I interferon-mediated signaling pathway
- programmed necrotic cell death
- regulation of apoptotic process
- regulation of inflammatory response
- regulation of transcription by RNA polymerase II
- regulation of type I interferon production
- TRIF-dependent toll-like receptor signaling pathway
- type I interferon signaling pathway
- viral process

---

5

- **Protein name:** 40S ribosomal protein S15a
- **Organism:** Homo sapiens
- **Uniprot Accession Number:** P62244
- **Protein sequence length:** 130 aa
- **1D identity (%):** 2.61
- **1D identity (%) [Gaps excluded]:** 34.69
- **1D identity - Alignment Gaps:** 1207
- **Common reported functions (%):** 0.0
- **Common reported locations (%):** 0.0
- **Common reported processes (%):** 0.0

- **PDB ID:** 6ZMT
- **Chain:** W
- **Crystallized protein length:** 129 aa
- **Resolution:** 3.0 Å
- **Alinged residues range:** 103-106, 29-31, 86-90, 119-125, 123-127, 36-43, 8-13
- **Aligned to segment part (indices):** 3, 5, 6, 1, 4, 0, 2
- **Alinged residues range of reference:** 47-50, 219-221, 296-300, 427-433, 549-553, 765-773, 956-961
- **b-phipsi:** 0.000445
- **w-rdist:** 2.068819
- **t-alpha:** 0.429766
- **Chemical similarity (Tanimoto Index) (%):** 82.78
- **1D identity (%) [PDB]:** 0.0
- **1D identity (%) [Gaps excluded][PDB]:** 0.0
- **1D identity - Alignment Gaps [PDB]:** 1112
- **2D identity (%) [PDB]:** 8.86
- **2D identity (%) [Gaps excluded][PDB]:** 93.75
- **2D identity - Alignment Gaps [PDB]:** 920
- **3D similarity (TM-Score) (%) [PDB]:** 7.52

- **Gene name:** RPS15A
- **RefSeq ID:** NM\_001030009
- **Transcript sequence length:** 2137
- **5-UTR|CDS|3-UTR identity (%):** 7.46 | 7.21 | 8.4
- **5-UTR|CDS|3-UTR identity (%) [Gaps excluded]:** 74.07 | 81.34 | 75.77
- **5-UTR|CDS|3-UTR identity [Alignment Gaps]:** 241 | 3529 | 1555

**Uniprot Description:**  
  
Structural component of the ribosome (PubMed:23636399). Required for proper erythropoiesis (PubMed:27909223).  
  
Component of the 40S ribosomal subunit.  
  
**Gene Ontology Information:**

Molecular Function

- RNA binding
- structural constituent of ribosome

Location

- cytoplasm
- cytosol
- cytosolic ribosome
- cytosolic small ribosomal subunit
- extracellular exosome
- membrane
- nucleoplasm

Biological process

- cytoplasmic translation
- nuclear-transcribed mRNA catabolic process, nonsense-mediated decay
- positive regulation of cell cycle
- positive regulation of cell population proliferation
- response to virus
- SRP-dependent cotranslational protein targeting to membrane
- translation
- translational initiation
- viral transcription

---

6

- **Protein name:** Early 35 kDa protein
- **Organism:** Autographa californica nuclear polyhedrosis virus
- **Uniprot Accession Number:** P08160
- **Protein sequence length:** 299 aa
- **1D identity (%):** 4.49
- **1D identity (%) [Gaps excluded]:** 25.53
- **1D identity - Alignment Gaps:** 1102
- **Common reported functions (%):** 0.0
- **Common reported locations (%):** 0.0
- **Common reported processes (%):** 0.0

- **PDB ID:** 1I3P
- **Chain:** A
- **Crystallized protein length:** 274 aa
- **Resolution:** 3.1 Å
- **Alinged residues range:** 231-233, 284-287, 242-247, 263-270, 269-286, 213-215, 108-135
- **Aligned to segment part (indices):** 3, 5, 6, 1, 4, 0, 2
- **Alinged residues range of reference:** 50-52, 202-205, 285-290, 394-401, 575-588, 771-773, 998-1011
- **b-phipsi:** 0.000458
- **w-rdist:** 5.375138
- **t-alpha:** 0.161685
- **Chemical similarity (Tanimoto Index) (%):** 82.81
- **1D identity (%) [PDB]:** 0.0
- **1D identity (%) [Gaps excluded][PDB]:** 0.0
- **1D identity - Alignment Gaps [PDB]:** 1260
- **2D identity (%) [PDB]:** 19.86
- **2D identity (%) [Gaps excluded][PDB]:** 89.91
- **2D identity - Alignment Gaps [PDB]:** 804
- **3D similarity (TM-Score) (%) [PDB]:** 9.75

- **Gene name:** P35
- **RefSeq ID:** NC\_001623
- **Genomic sequence length:** 133894
- **5-UTR|CDS|3-UTR identity (%):** N/A | 17.04 | N/A
- **5-UTR|CDS|3-UTR identity (%) [Gaps excluded]:** N/A | 81.2 | N/A
- **5-UTR|CDS|3-UTR identity [Alignment Gaps]:** N/A | 3084 | N/A

**Uniprot Description:**  
  
Functions as an inhibitor of the host RNA interference antiviral response. Inhibits the insect host cell apoptotic response initiated by the viral infection. Blocks as well the activity of members of the caspase family of proteases. Required for late and very late gene expression.  
  
**Gene Ontology Information:**

Molecular Function

- cysteine-type endopeptidase inhibitor activity

Location  
  
N/A

Biological process

- negative regulation of apoptotic process
- negative regulation of cysteine-type endopeptidase activity involved in apoptotic process
- negative regulation of RNA interference
- suppression by virus of host apoptotic process
- suppression by virus of host cysteine-type endopeptidase activity involved in apoptotic process

---

7

- **Protein name:** Replicase polyprotein 1ab
- **Organism:** Severe acute respiratory syndrome coronavirus
- **Uniprot Accession Number:** P0C6X7
- **Protein sequence length:** 7073 aa
- **1D identity (%):** 4.01
- **1D identity (%) [Gaps excluded]:** 27.37
- **1D identity - Alignment Gaps:** 6212
- **Common reported functions (%):** 50.0
- **Common reported locations (%):** 12.5
- **Common reported processes (%):** 20.0

- **PDB ID:** 2XYQ
- **Chain:** A
- **Crystallized protein length:** 287 aa
- **Resolution:** 2.0 Å
- **Alinged residues range:** 163-166, 191-195, 166-170, 207-211, 164-167, 80-84, 220-227
- **Aligned to segment part (indices):** 3, 5, 6, 1, 4, 0, 2
- **Alinged residues range of reference:** 48-51, 200-204, 286-290, 395-399, 548-551, 768-772, 985-992
- **b-phipsi:** 0.001509
- **w-rdist:** 2.251029
- **t-alpha:** 0.403941
- **Chemical similarity (Tanimoto Index) (%):** 82.52
- **1D identity (%) [PDB]:** 0.08
- **1D identity (%) [Gaps excluded][PDB]:** 50.0
- **1D identity - Alignment Gaps [PDB]:** 1267
- **2D identity (%) [PDB]:** 16.03
- **2D identity (%) [Gaps excluded][PDB]:** 86.87
- **2D identity - Alignment Gaps [PDB]:** 875
- **3D similarity (TM-Score) (%) [PDB]:** 9.85

- **Gene name:** rep
- **RefSeq ID:** NC\_004718
- **Genomic sequence length:** 29751
- **5-UTR|CDS|3-UTR identity (%):** 88.52 | 19.99 | 22.38
- **5-UTR|CDS|3-UTR identity (%) [Gaps excluded]:** 92.28 | 80.71 | 98.18
- **5-UTR|CDS|3-UTR identity [Alignment Gaps]:** 11 | 10233 | 745

**Uniprot Description:**  
  
Isoform Replicase polyprotein 1ab
Multifunctional protein involved in the transcription and replication of viral RNAs. Contains the proteinases responsible for the cleavages of the polyprotein.  
  
Non-structural protein 2
Interacts with host PHB and PHB2.  
  
**Gene Ontology Information:**

Molecular Function

- 3'-5'-exoribonuclease activity
- ATP binding
- cysteine-type endopeptidase activity
- DNA helicase activity
- double-stranded RNA binding
- endonuclease activity
- G-quadruplex RNA binding
- helicase activity
- identical protein binding
- ISG15-specific protease activity
- Lys48-specific deubiquitinase activity
- methyltransferase activity
- mRNA (guanine-N7-)-methyltransferase activity
- mRNA (nucleoside-2'-O-)-methyltransferase activity
- protein dimerization activity
- RNA helicase activity
- RNA-directed 5'-3' RNA polymerase activity
- single-stranded RNA binding
- thiol-dependent ubiquitin-specific protease activity
- zinc ion binding

Location

- cytoplasmic viral factory
- double membrane vesicle viral factory outer membrane
- host cell cytoplasm
- host cell endoplasmic reticulum-Golgi intermediate compartment
- host cell perinuclear region of cytoplasm
- integral component of membrane

Biological process

- 7-methylguanosine mRNA capping
- induction by virus of catabolism of host mRNA
- induction by virus of host autophagy
- methylation
- modulation by virus of host protein ubiquitination
- mRNA methylation
- positive regulation of ubiquitin-specific protease activity
- positive stranded viral RNA replication
- protein autoprocessing
- protein K48-linked deubiquitination
- protein K63-linked deubiquitination
- RNA phosphodiester bond hydrolysis, exonucleolytic
- suppression by virus of host IRF3 activity
- suppression by virus of host ISG15 activity
- suppression by virus of host NF-kappaB transcription factor activity
- suppression by virus of host toll-like receptor signaling pathway
- suppression by virus of host TRAF activity
- suppression by virus of host translation
- suppression by virus of host type I interferon production
- suppression by virus of host type I interferon-mediated signaling pathway
- transcription, DNA-templated
- transcription, RNA-templated
- viral protein processing
- viral RNA genome replication
- viral transcription

---

8

- **Protein name:** H-2 class I histocompatibility antigen, K-B alpha chain
- **Organism:** Mus musculus
- **Uniprot Accession Number:** P01901
- **Protein sequence length:** 369 aa
- **1D identity (%):** 6.17
- **1D identity (%) [Gaps excluded]:** 30.0
- **1D identity - Alignment Gaps:** 1082
- **Common reported functions (%):** 0.0
- **Common reported locations (%):** 0.0
- **Common reported processes (%):** 0.0

- **PDB ID:** 1NAM
- **Chain:** H
- **Crystallized protein length:** 275 aa
- **Resolution:** 2.7 Å
- **Alinged residues range:** 110-115, 96-116, 171-178, 7-12, 31-35, 56-61, 142-145
- **Aligned to segment part (indices):** 3, 5, 6, 1, 4, 0, 2
- **Alinged residues range of reference:** 48-53, 196-208, 295-302, 396-401, 573-577, 746-751, 1008-1011
- **b-phipsi:** 0.002153
- **w-rdist:** 1.6252
- **t-alpha:** 0.372392
- **Chemical similarity (Tanimoto Index) (%):** 85.5
- **1D identity (%) [PDB]:** 0.0
- **1D identity (%) [Gaps excluded][PDB]:** 0.0
- **1D identity - Alignment Gaps [PDB]:** 1258
- **2D identity (%) [PDB]:** 17.23
- **2D identity (%) [Gaps excluded][PDB]:** 90.1
- **2D identity - Alignment Gaps [PDB]:** 854
- **3D similarity (TM-Score) (%) [PDB]:** 10.45

- **Gene name:** H2-K1
- **RefSeq ID:** N/A
- **Sequence length:** N/A
- **5-UTR|CDS|3-UTR identity (%):** N/A | N/A | N/A
- **5-UTR|CDS|3-UTR identity (%) [Gaps excluded]:** N/A | N/A | N/A
- **5-UTR|CDS|3-UTR identity [Alignment Gaps]:** N/A | N/A | N/A

**Uniprot Description:**  
  
Involved in the presentation of foreign antigens to the immune system.  
  
Heterodimer of an alpha chain and a beta chain (beta-2-microglobulin).  
  
**Gene Ontology Information:**

Molecular Function

- beta-2-microglobulin binding
- CD8 receptor binding
- peptide antigen binding
- peptide binding
- protein-containing complex binding
- signaling receptor binding
- T cell receptor binding
- TAP binding
- TAP complex binding

Location

- cell surface
- endoplasmic reticulum
- endoplasmic reticulum exit site
- external side of plasma membrane
- extracellular space
- Golgi apparatus
- Golgi medial cisterna
- integral component of lumenal side of endoplasmic reticulum membrane
- MHC class I peptide loading complex
- MHC class I protein complex
- phagocytic vesicle membrane
- plasma membrane

Biological process

- antigen processing and presentation of endogenous peptide antigen via MHC class I via ER pathway, TAP-dependent
- antigen processing and presentation of endogenous peptide antigen via MHC class Ib
- antigen processing and presentation of exogenous peptide antigen via MHC class I
- defense response to bacterium
- immune response
- inner ear development
- negative regulation of neuron projection development
- positive regulation of T cell mediated cytotoxicity

---

9

- **Protein name:** Gag-Pol polyprotein
- **Organism:** Human immunodeficiency virus type 1 group M subtype B (isolate HXB2)
- **Uniprot Accession Number:** P04585
- **Protein sequence length:** 1435 aa
- **1D identity (%):** 12.98
- **1D identity (%) [Gaps excluded]:** 28.67
- **1D identity - Alignment Gaps:** 1020
- **Common reported functions (%):** 50.0
- **Common reported locations (%):** 25.0
- **Common reported processes (%):** 10.0

- **PDB ID:** 6UK0
- **Chain:** A
- **Crystallized protein length:** 532 aa
- **Resolution:** 2.76 Å
- **Alinged residues range:** 349-353, 338-352, 103-107, 438-442, 455-468, 262-267, 396-404
- **Aligned to segment part (indices):** 3, 5, 6, 1, 4, 0, 2
- **Alinged residues range of reference:** 48-52, 200-209, 309-313, 398-402, 592-599, 761-766, 1002-1010
- **b-phipsi:** 0.001296
- **w-rdist:** 9.410314
- **t-alpha:** 0.007067
- **Chemical similarity (Tanimoto Index) (%):** 82.59
- **1D identity (%) [PDB]:** 0.07
- **1D identity (%) [Gaps excluded][PDB]:** 100.0
- **1D identity - Alignment Gaps [PDB]:** 1517
- **2D identity (%) [PDB]:** 23.3
- **2D identity (%) [Gaps excluded][PDB]:** 85.28
- **2D identity - Alignment Gaps [PDB]:** 867
- **3D similarity (TM-Score) (%) [PDB]:** 16.58

- **Gene name:** gag-pol
- **RefSeq ID:** NC\_001802
- **Genomic sequence length:** 9181
- **5-UTR|CDS|3-UTR identity (%):** 23.6 | 40.46 | 23.63
- **5-UTR|CDS|3-UTR identity (%) [Gaps excluded]:** 75.9 | 77.79 | 80.65
- **5-UTR|CDS|3-UTR identity [Alignment Gaps]:** 184 | 2566 | 374

**Uniprot Description:**  
  
Gag-Pol polyprotein
Mediates, with Gag polyprotein, the essential events in virion assembly, including binding the plasma membrane, making the protein-protein interactions necessary to create spherical particles, recruiting the viral Env proteins, and packaging the genomic RNA via direct interactions with the RNA packaging sequence (Psi). Gag-Pol polyprotein may regulate its own translation, by the binding genomic RNA in the 5'-UTR. At low concentration, the polyprotein would promote translation, whereas at high concentration, the polyprotein would encapsidate genomic RNA and then shut off translation.  
  
Matrix protein p17
Homotrimer; further assembles as hexamers of trimers (PubMed:19327811). Interacts with gp41 (via C-terminus) (By similarity). Interacts with host CALM1; this interaction induces a conformational change in the Matrix protein, triggering exposure of the myristate group (PubMed:24500712). Interacts with host AP3D1; this interaction allows the polyprotein trafficking to multivesicular bodies during virus assembly (By similarity). Part of the pre-integration complex (PIC) which is composed of viral genome, matrix protein, Vpr and integrase (By similarity).  
  
**Gene Ontology Information:**

Molecular Function

- aspartic-type endopeptidase activity
- DNA binding
- DNA-directed DNA polymerase activity
- exoribonuclease H activity
- identical protein binding
- lipid binding
- RNA binding
- RNA-directed DNA polymerase activity
- RNA-DNA hybrid ribonuclease activity
- structural molecule activity
- zinc ion binding

Location

- host cell nucleus
- host cell plasma membrane
- host multivesicular body
- viral nucleocapsid
- virion membrane

Biological process

- DNA integration
- DNA recombination
- entry into host
- establishment of integrated proviral latency
- fusion of virus membrane with host plasma membrane
- induction by virus of host cysteine-type endopeptidase activity involved in apoptotic process
- RNA-dependent DNA biosynthetic process
- suppression by virus of host gene expression
- uncoating of virus
- viral genome integration into host DNA
- viral genome packaging
- viral life cycle
- viral penetration into host nucleus
- virion assembly

---

10

- **Protein name:** Replicase polyprotein 1ab
- **Organism:** Murine coronavirus (strain A59)
- **Uniprot Accession Number:** P0C6X9
- **Protein sequence length:** 7176 aa
- **1D identity (%):** 3.92
- **1D identity (%) [Gaps excluded]:** 30.79
- **1D identity - Alignment Gaps:** 6539
- **Common reported functions (%):** 0.0
- **Common reported locations (%):** 12.5
- **Common reported processes (%):** 20.0

- **PDB ID:** 6JIJ
- **Chain:** C
- **Crystallized protein length:** 298 aa
- **Resolution:** 2.65 Å
- **Alinged residues range:** 151-155, 124-127, 18-22, 28-31, 9-12, 289-295, 289-295
- **Aligned to segment part (indices):** 3, 5, 6, 1, 4, 0, 2
- **Alinged residues range of reference:** 38-42, 201-204, 312-316, 450-453, 600-603, 747-753, 1006-1012
- **b-phipsi:** 0.000427
- **w-rdist:** 7.337982
- **t-alpha:** 0.25
- **Chemical similarity (Tanimoto Index) (%):** 83.71
- **1D identity (%) [PDB]:** 0.08
- **1D identity (%) [Gaps excluded][PDB]:** 100.0
- **1D identity - Alignment Gaps [PDB]:** 1279
- **2D identity (%) [PDB]:** 20.66
- **2D identity (%) [Gaps excluded][PDB]:** 87.35
- **2D identity - Alignment Gaps [PDB]:** 791
- **3D similarity (TM-Score) (%) [PDB]:** 12.01

- **Gene name:** rep
- **RefSeq ID:** NC\_001846
- **Genomic sequence length:** 31357
- **5-UTR|CDS|3-UTR identity (%):** 43.0 | 19.37 | 38.17
- **5-UTR|CDS|3-UTR identity (%) [Gaps excluded]:** 79.04 | 80.1 | 77.6
- **5-UTR|CDS|3-UTR identity [Alignment Gaps]:** 140 | 10523 | 189

**Uniprot Description:**  
  
The replicase polyprotein of coronaviruses is a multifunctional protein: it contains the activities necessary for the transcription of negative stranded RNA, leader RNA, subgenomic mRNAs and progeny virion RNA as well as proteinases responsible for the cleavage of the polyprotein into functional products.  
  
Nsp2 interacts with host PHB and PHB2. 3CL-PRO exists as monomer and homodimer. Nsp4 interacts with PL-PRO and nsp6. Only the homodimer shows catalytic activity. Eight copies of nsp7 and eight copies of nsp8 assemble to form a heterohexadecamer dsRNA-encircling ring structure. Nsp9 is a dimer. Nsp10 forms a dodecamer and interacts with nsp14 and nsp16; these interactions enhance nsp14 and nsp16 enzymatic activities. Nsp14 interacts (via N-terminus) with DDX1.  
  
**Gene Ontology Information:**

Molecular Function

- ATP binding
- cysteine-type endopeptidase activity
- DNA helicase activity
- endonuclease activity
- exoribonuclease activity
- methyltransferase activity
- RNA helicase activity
- RNA-directed 5'-3' RNA polymerase activity
- single-stranded RNA binding
- thiol-dependent ubiquitin-specific protease activity
- zinc ion binding

Location

- cytoplasmic viral factory
- host cell endoplasmic reticulum-Golgi intermediate compartment
- host cell membrane
- host cell perinuclear region of cytoplasm
- integral component of membrane

Biological process

- induction by virus of catabolism of host mRNA
- induction by virus of host autophagy
- methylation
- modulation by virus of host protein ubiquitination
- suppression by virus of host ISG15 activity
- suppression by virus of host NF-kappaB transcription factor activity
- suppression by virus of host type I interferon-mediated signaling pathway
- transcription, DNA-templated
- viral protein processing
- viral RNA genome replication

---

11

- **Protein name:** RIIA-RIIB membrane-associated protein
- **Organism:** Synechococcus phage S-SSM7
- **Uniprot Accession Number:** E3SLL2
- **Protein sequence length:** 128 aa
- **1D identity (%):** 1.82
- **1D identity (%) [Gaps excluded]:** 30.38
- **1D identity - Alignment Gaps:** 1243
- **Common reported functions (%):** 0.0
- **Common reported locations (%):** 0.0
- **Common reported processes (%):** 0.0

- **PDB ID:** 3UWA
- **Chain:** A
- **Crystallized protein length:** 144 aa
- **Resolution:** 1.95 Å
- **Alinged residues range:** 119-121, 77-81, 119-123, 109-113, 76-84, 25-29, 131-139
- **Aligned to segment part (indices):** 3, 5, 6, 1, 4, 0, 2
- **Alinged residues range of reference:** 51-53, 202-206, 313-317, 394-398, 549-557, 748-752, 951-959
- **b-phipsi:** 0.001421
- **w-rdist:** 2.388336
- **t-alpha:** 0.425
- **Chemical similarity (Tanimoto Index) (%):** 81.61
- **1D identity (%) [PDB]:** 0.09
- **1D identity (%) [Gaps excluded][PDB]:** 100.0
- **1D identity - Alignment Gaps [PDB]:** 1129
- **2D identity (%) [PDB]:** 10.8
- **2D identity (%) [Gaps excluded][PDB]:** 89.34
- **2D identity - Alignment Gaps [PDB]:** 887
- **3D similarity (TM-Score) (%) [PDB]:** 7.01

- **Gene name:** SSSM7\_299
- **RefSeq ID:** NC\_015287
- **Genomic sequence length:** 232878
- **5-UTR|CDS|3-UTR identity (%):** N/A | 7.04 | N/A
- **5-UTR|CDS|3-UTR identity (%) [Gaps excluded]:** N/A | 82.73 | N/A
- **5-UTR|CDS|3-UTR identity [Alignment Gaps]:** N/A | 3549 | N/A

**Uniprot Description:**  
  
N/A  
  
**Gene Ontology Information:**

Molecular Function

- metal ion binding

Location  
  
N/A

Biological process  
  
N/A

---

12

- **Protein name:** Capsid vertex component 1
- **Organism:** Epstein-Barr virus (strain B95-8)
- **Uniprot Accession Number:** P03222
- **Protein sequence length:** 507 aa
- **1D identity (%):** 1.61
- **1D identity (%) [Gaps excluded]:** 27.0
- **1D identity - Alignment Gaps:** 1580
- **Common reported functions (%):** 0.0
- **Common reported locations (%):** 0.0
- **Common reported processes (%):** 0.0

- **PDB ID:** 7BR7
- **Chain:** C
- **Crystallized protein length:** 346 aa
- **Resolution:** 4.3 Å
- **Alinged residues range:** 427-429, 18-20, 118-122, 433-438, 77-82, 340-346, 336-344
- **Aligned to segment part (indices):** 3, 5, 6, 1, 4, 0, 2
- **Alinged residues range of reference:** 39-41, 226-228, 295-299, 429-434, 592-597, 765-771, 953-961
- **b-phipsi:** 0.001941
- **w-rdist:** 2.873257
- **t-alpha:** 0.305344
- **Chemical similarity (Tanimoto Index) (%):** 83.83
- **1D identity (%) [PDB]:** 0.0
- **1D identity (%) [Gaps excluded][PDB]:** 0.0
- **1D identity - Alignment Gaps [PDB]:** 1334
- **2D identity (%) [PDB]:** 20.72
- **2D identity (%) [Gaps excluded][PDB]:** 88.54
- **2D identity - Alignment Gaps [PDB]:** 828
- **3D similarity (TM-Score) (%) [PDB]:** 5.41

- **Gene name:** CVC1
- **RefSeq ID:** NC\_007605
- **Genomic sequence length:** 171823
- **5-UTR|CDS|3-UTR identity (%):** N/A | 23.59 | N/A
- **5-UTR|CDS|3-UTR identity (%) [Gaps excluded]:** N/A | 76.83 | N/A
- **5-UTR|CDS|3-UTR identity [Alignment Gaps]:** N/A | 2834 | N/A

**Uniprot Description:**  
  
Capsid vertex-specific component that plays a role during viral DNA encapsidation, assuring correct genome cleavage and presumably stabilizing capsids that contain full-length viral genomes.  
  
Interacts (via C-terminus) with capsid vertex component 2/CVC2.  
  
**Gene Ontology Information:**

Molecular Function  
  
N/A

Location

- host cell nucleus
- viral capsid

Biological process

- DNA packaging
- viral release from host cell

---

13

- **Protein name:** Repair DNA polymerase X
- **Organism:** African swine fever virus (strain Badajoz 1971 Vero-adapted)
- **Uniprot Accession Number:** P42494
- **Protein sequence length:** 174 aa
- **1D identity (%):** 2.94
- **1D identity (%) [Gaps excluded]:** 32.23
- **1D identity - Alignment Gaps:** 1205
- **Common reported functions (%):** 0.0
- **Common reported locations (%):** 0.0
- **Common reported processes (%):** 0.0

- **PDB ID:** 1JQR
- **Chain:** A
- **Crystallized protein length:** 3654 aa
- **Resolution:** -1.0 Å
- **Alinged residues range:** 95-98, 26-28, 95-98, 34-36, 20-24, 110-113, 62-67
- **Aligned to segment part (indices):** 3, 5, 6, 1, 4, 0, 2
- **Alinged residues range of reference:** 49-52, 226-228, 314-317, 433-435, 541-545, 769-772, 977-982
- **b-phipsi:** 0.001315
- **w-rdist:** 2.66933
- **t-alpha:** 0.471601
- **Chemical similarity (Tanimoto Index) (%):** 85.11
- **1D identity (%) [PDB]:** 0.0
- **1D identity (%) [Gaps excluded][PDB]:** 0.0
- **1D identity - Alignment Gaps [PDB]:** 1157
- **2D identity (%) [PDB]:** 13.06
- **2D identity (%) [Gaps excluded][PDB]:** 95.68
- **2D identity - Alignment Gaps [PDB]:** 879
- **3D similarity (TM-Score) (%) [PDB]:** 7.5

- **Gene name:** Ba71V-97
- **RefSeq ID:** NC\_001659
- **Genomic sequence length:** 170101
- **5-UTR|CDS|3-UTR identity (%):** N/A | 9.71 | N/A
- **5-UTR|CDS|3-UTR identity (%) [Gaps excluded]:** N/A | 79.32 | N/A
- **5-UTR|CDS|3-UTR identity [Alignment Gaps]:** N/A | 3399 | N/A

**Uniprot Description:**  
  
Error-prone polymerase lacking a proofreading 3'-5' exonuclease which plays a role in viral DNA repair. Specifically binds intermediates in the single-nucleotide base-excision repair process. Also catalyzes DNA polymerization with low nucleotide-insertion fidelity. Together with the viral DNA ligase, fills the single nucleotide gaps generated by the AP endonuclease.  
  
**Gene Ontology Information:**

Molecular Function

- DNA binding
- DNA-directed DNA polymerase activity
- metal ion binding

Location  
  
N/A

Biological process

- DNA repair

---

14

- **Protein name:** Polyprotein P1234
- **Organism:** Chikungunya virus (strain S27-African prototype)
- **Uniprot Accession Number:** Q8JUX6
- **Protein sequence length:** 2474 aa
- **1D identity (%):** 10.2
- **1D identity (%) [Gaps excluded]:** 28.75
- **1D identity - Alignment Gaps:** 1785
- **Common reported functions (%):** 0.0
- **Common reported locations (%):** 12.5
- **Common reported processes (%):** 10.0

- **PDB ID:** 3TRK
- **Chain:** A
- **Crystallized protein length:** 314 aa
- **Resolution:** 2.4 Å
- **Alinged residues range:** 1251-1254, 1027-1029, 1075-1079, 1093-1103, 1253-1258, 1319-1323, 1315-1322
- **Aligned to segment part (indices):** 3, 5, 6, 1, 4, 0, 2
- **Alinged residues range of reference:** 48-51, 210-212, 312-316, 361-368, 547-552, 762-766, 951-958
- **b-phipsi:** 0.00183
- **w-rdist:** 6.129295
- **t-alpha:** 0.114733
- **Chemical similarity (Tanimoto Index) (%):** 83.91
- **1D identity (%) [PDB]:** 0.08
- **1D identity (%) [Gaps excluded][PDB]:** 50.0
- **1D identity - Alignment Gaps [PDB]:** 1300
- **2D identity (%) [PDB]:** 13.58
- **2D identity (%) [Gaps excluded][PDB]:** 86.44
- **2D identity - Alignment Gaps [PDB]:** 950
- **3D similarity (TM-Score) (%) [PDB]:** 10.83

- **Gene name:** N/A
- **RefSeq ID:** NC\_004162
- **Genomic sequence length:** 11826
- **5-UTR|CDS|3-UTR identity (%):** N/A | 33.61 | N/A
- **5-UTR|CDS|3-UTR identity (%) [Gaps excluded]:** N/A | 78.5 | N/A
- **5-UTR|CDS|3-UTR identity [Alignment Gaps]:** N/A | 4503 | N/A

**Uniprot Description:**  
  
Polyprotein P1234
Inactive precursor of the viral replicase, which is activated by cleavages carried out by the viral protease nsP2.  
  
mRNA-capping enzyme nsP1
Interacts with non-structural protein 3 (PubMed:22951312). Interacts with RNA-directed RNA polymerase nsP4 (PubMed:22951312). Interacts with protease nsP2 (PubMed:22951312). interacts with itself (PubMed:22951312).  
  
**Gene Ontology Information:**

Molecular Function

- ADP-ribosyl-[dinitrogen reductase] hydrolase activity
- ATP binding
- cysteine-type peptidase activity
- GTP binding
- helicase activity
- metal ion binding
- mRNA methyltransferase activity
- nucleoside-triphosphatase activity
- polynucleotide 5'-phosphatase activity
- polynucleotide adenylyltransferase activity
- RNA binding
- RNA helicase activity
- RNA-directed 5'-3' RNA polymerase activity

Location

- host cell cytoplasmic vesicle membrane
- host cell filopodium
- host cell nucleus
- host cell plasma membrane
- membrane

Biological process

- 7-methylguanosine mRNA capping
- positive stranded viral RNA replication
- regulation of cytoskeleton organization
- stress granule disassembly
- suppression by virus of host RNA polymerase II activity
- suppression by virus of host STAT activity
- suppression by virus of host STAT1 activity
- suppression by virus of host type I interferon-mediated signaling pathway
- transcription, DNA-templated
- viral RNA genome replication

---

15

- **Protein name:** Genome polyprotein
- **Organism:** Yellow fever virus (strain 17D vaccine)
- **Uniprot Accession Number:** P03314
- **Protein sequence length:** 3411 aa
- **1D identity (%):** 7.05
- **1D identity (%) [Gaps excluded]:** 28.12
- **1D identity - Alignment Gaps:** 2806
- **Common reported functions (%):** 0.0
- **Common reported locations (%):** 37.5
- **Common reported processes (%):** 20.0

- **PDB ID:** 1YKS
- **Chain:** A
- **Crystallized protein length:** 431 aa
- **Resolution:** 1.8 Å
- **Alinged residues range:** 474-476, 223-225, 233-237, 520-525, 475-486, 296-299, 370-375
- **Aligned to segment part (indices):** 3, 5, 6, 1, 4, 0, 2
- **Alinged residues range of reference:** 49-51, 225-227, 296-300, 380-385, 547-558, 739-742, 1013-1018
- **b-phipsi:** 0.001646
- **w-rdist:** 5.904707
- **t-alpha:** 0.195804
- **Chemical similarity (Tanimoto Index) (%):** 83.75
- **1D identity (%) [PDB]:** 0.07
- **1D identity (%) [Gaps excluded][PDB]:** 100.0
- **1D identity - Alignment Gaps [PDB]:** 1413
- **2D identity (%) [PDB]:** 22.16
- **2D identity (%) [Gaps excluded][PDB]:** 87.11
- **2D identity - Alignment Gaps [PDB]:** 841
- **3D similarity (TM-Score) (%) [PDB]:** 13.23

- **Gene name:** N/A
- **RefSeq ID:** NC\_002031
- **Genomic sequence length:** 10862
- **5-UTR|CDS|3-UTR identity (%):** N/A | 23.34 | N/A
- **5-UTR|CDS|3-UTR identity (%) [Gaps excluded]:** N/A | 79.01 | N/A
- **5-UTR|CDS|3-UTR identity [Alignment Gaps]:** N/A | 7646 | N/A

**Uniprot Description:**  
  
Capsid protein C
Plays a role in virus budding by binding to the cell membrane and gathering the viral RNA into a nucleocapsid that forms the core of a mature virus particle. During virus entry, may induce genome penetration into the host cytoplasm after hemifusion induced by the surface proteins. Can migrate to the cell nucleus where it modulates host functions.  
  
Capsid protein C
Homodimer (PubMed:12768036). Interacts (via N-terminus) with host EXOC1 (via C-terminus); this interaction results in EXOC1 degradation through the proteasome degradation pathway (By similarity).  
  
**Gene Ontology Information:**

Molecular Function

- ATP binding
- double-stranded RNA binding
- GTP binding
- metal ion binding
- mRNA (guanine-N7-)-methyltransferase activity
- mRNA (nucleoside-2'-O-)-methyltransferase activity
- nucleoside-triphosphatase activity
- protein dimerization activity
- RNA helicase activity
- RNA-directed 5'-3' RNA polymerase activity
- serine-type endopeptidase activity
- structural molecule activity

Location

- extracellular region
- host cell endoplasmic reticulum membrane
- host cell nucleus
- host cell perinuclear region of cytoplasm
- integral component of membrane
- viral capsid
- viral envelope
- virion membrane

Biological process

- clathrin-dependent endocytosis of virus by host cell
- fusion of virus membrane with host endosome membrane
- induction by virus of host autophagy
- negative regulation of RNA interference
- suppression by virus of host STAT2 activity
- suppression by virus of host type I interferon-mediated signaling pathway
- viral budding from endoplasmic reticulum membrane
- viral RNA genome replication
- virion attachment to host cell

---

16

- **Protein name:** Genome polyprotein
- **Organism:** Kokobera virus
- **Uniprot Accession Number:** Q32ZD5
- **Protein sequence length:** 3410 aa
- **1D identity (%):** 4.27
- **1D identity (%) [Gaps excluded]:** 27.24
- **1D identity - Alignment Gaps:** 3413
- **Common reported functions (%):** 0.0
- **Common reported locations (%):** 25.0
- **Common reported processes (%):** 20.0

- **PDB ID:** 2V6J
- **Chain:** A
- **Crystallized protein length:** 421 aa
- **Resolution:** 2.3 Å
- **Alinged residues range:** 257-259, 255-259, 257-261, 252-260, 288-294, 107-110, 348-363
- **Aligned to segment part (indices):** 3, 5, 6, 1, 4, 0, 2
- **Alinged residues range of reference:** 49-51, 205-209, 314-318, 393-401, 526-532, 739-742, 1011-1018
- **b-phipsi:** 0.002123
- **w-rdist:** 3.969791
- **t-alpha:** 0.185853
- **Chemical similarity (Tanimoto Index) (%):** 83.58
- **1D identity (%) [PDB]:** 0.0
- **1D identity (%) [Gaps excluded][PDB]:** 0.0
- **1D identity - Alignment Gaps [PDB]:** 1405
- **2D identity (%) [PDB]:** 19.39
- **2D identity (%) [Gaps excluded][PDB]:** 87.45
- **2D identity - Alignment Gaps [PDB]:** 895
- **3D similarity (TM-Score) (%) [PDB]:** 12.84

- **Gene name:** N/A
- **RefSeq ID:** NC\_009029
- **Genomic sequence length:** 10874
- **5-UTR|CDS|3-UTR identity (%):** N/A | 24.11 | N/A
- **5-UTR|CDS|3-UTR identity (%) [Gaps excluded]:** N/A | 77.51 | N/A
- **5-UTR|CDS|3-UTR identity [Alignment Gaps]:** N/A | 7385 | N/A

**Uniprot Description:**  
  
Capsid protein C
Plays a role in virus budding by binding to the cell membrane and gathering the viral RNA into a nucleocapsid that forms the core of a mature virus particle. During virus entry, may induce genome penetration into the host cytoplasm after hemifusion induced by the surface proteins. Can migrate to the cell nucleus where it modulates host functions. Overcomes the anti-viral effects of host EXOC1 by sequestering and degrading the latter through the proteasome degradation pathway.  
  
Capsid protein C
Homodimer (By similarity). Interacts (via N-terminus) with host EXOC1 (via C-terminus); this interaction results in EXOC1 degradation through the proteasome degradation pathway (By similarity).  
  
**Gene Ontology Information:**

Molecular Function

- ATP binding
- double-stranded RNA binding
- metal ion binding
- mRNA (guanine-N7-)-methyltransferase activity
- mRNA (nucleoside-2'-O-)-methyltransferase activity
- nucleoside-triphosphatase activity
- protein dimerization activity
- RNA helicase activity
- RNA-directed 5'-3' RNA polymerase activity
- serine-type endopeptidase activity
- structural molecule activity

Location

- extracellular region
- host cell endoplasmic reticulum membrane
- host cell nucleus
- host cell perinuclear region of cytoplasm
- integral component of membrane
- viral capsid
- virion membrane

Biological process

- fusion of virus membrane with host endosome membrane
- induction by virus of host autophagy
- suppression by virus of host STAT1 activity
- suppression by virus of host STAT2 activity
- suppression by virus of host type I interferon-mediated signaling pathway
- viral RNA genome replication
- virion attachment to host cell

---

17

- **Protein name:** Core-binding factor subunit beta
- **Organism:** Homo sapiens
- **Uniprot Accession Number:** Q13951
- **Protein sequence length:** 182 aa
- **1D identity (%):** 3.0
- **1D identity (%) [Gaps excluded]:** 25.32
- **1D identity - Alignment Gaps:** 1147
- **Common reported functions (%):** 0.0
- **Common reported locations (%):** 0.0
- **Common reported processes (%):** 0.0

- **PDB ID:** 6NIL
- **Chain:** E
- **Crystallized protein length:** 140 aa
- **Resolution:** 3.9 Å
- **Alinged residues range:** 109-115, 104-113, 109-113, 94-102, 107-111, 10-13, 10-13
- **Aligned to segment part (indices):** 3, 5, 6, 1, 4, 0, 2
- **Alinged residues range of reference:** 47-53, 198-209, 312-316, 394-401, 609-613, 761-764, 987-990
- **b-phipsi:** 0.002205
- **w-rdist:** 1.985635
- **t-alpha:** 0.403941
- **Chemical similarity (Tanimoto Index) (%):** 82.94
- **1D identity (%) [PDB]:** 0.0
- **1D identity (%) [Gaps excluded][PDB]:** 0.0
- **1D identity - Alignment Gaps [PDB]:** 1124
- **2D identity (%) [PDB]:** 8.28
- **2D identity (%) [Gaps excluded][PDB]:** 87.63
- **2D identity - Alignment Gaps [PDB]:** 930
- **3D similarity (TM-Score) (%) [PDB]:** 7.7

- **Gene name:** CBFB
- **RefSeq ID:** NM\_022845
- **Transcript sequence length:** 3103
- **5-UTR|CDS|3-UTR identity (%):** 19.66 | 9.18 | 7.11
- **5-UTR|CDS|3-UTR identity (%) [Gaps excluded]:** 72.32 | 81.53 | 81.59
- **5-UTR|CDS|3-UTR identity [Alignment Gaps]:** 300 | 3498 | 2107

**Uniprot Description:**  
  
Forms the heterodimeric complex core-binding factor (CBF) with RUNX family proteins (RUNX1, RUNX2, and RUNX3). RUNX members modulate the transcription of their target genes through recognizing the core consensus binding sequence 5'-TGTGGT-3', or very rarely, 5'-TGCGGT-3', within their regulatory regions via their runt domain, while CBFB is a non-DNA-binding regulatory subunit that allosterically enhances the sequence-specific DNA-binding capacity of RUNX. The heterodimers bind to the core site of a number of enhancers and promoters, including murine leukemia virus, polyomavirus enhancer, T-cell receptor enhancers, LCK, IL3 and GM-CSF promoters. CBF complexes repress ZBTB7B transcription factor during cytotoxic (CD8+) T cell development. They bind to RUNX-binding sequence within the ZBTB7B locus acting as transcriptional silencer and allowing for cytotoxic T cell differentiation.  
  
Heterodimer with RUNX1, RUNX2 and RUNX3. Interacts with COPRS. Found in a complex with PRMT5 and RUNX1.  
  
**Gene Ontology Information:**

Molecular Function

- sequence-specific DNA binding
- transcription coactivator activity

Location

- core-binding factor complex
- membrane
- nucleoplasm

Biological process

- cell maturation
- definitive hemopoiesis
- lymphocyte differentiation
- myeloid cell differentiation
- negative regulation of CD4-positive, alpha-beta T cell differentiation
- negative regulation of transcription by RNA polymerase II
- osteoblast differentiation
- positive regulation of CD8-positive, alpha-beta T cell differentiation
- positive regulation of transcription by RNA polymerase II
- protein polyubiquitination
- regulation of B cell receptor signaling pathway
- regulation of bicellular tight junction assembly
- regulation of cytokine-mediated signaling pathway
- regulation of hematopoietic stem cell differentiation
- regulation of intracellular estrogen receptor signaling pathway
- regulation of keratinocyte differentiation
- regulation of megakaryocyte differentiation
- regulation of myeloid cell differentiation
- regulation of regulatory T cell differentiation
- regulation of transcription by RNA polymerase II
- regulation of transcription initiation from RNA polymerase II promoter
- regulation of Wnt signaling pathway
- transcription by RNA polymerase II

---

18

- **Protein name:** Hemagglutinin
- **Organism:** Influenza B virus (strain B/Lee/1940)
- **Uniprot Accession Number:** P03460
- **Protein sequence length:** 584 aa
- **1D identity (%):** 10.71
- **1D identity (%) [Gaps excluded]:** 29.55
- **1D identity - Alignment Gaps:** 869
- **Common reported functions (%):** 50.0
- **Common reported locations (%):** 50.0
- **Common reported processes (%):** 30.0

- **PDB ID:** 4NRJ
- **Chain:** C
- **Crystallized protein length:** 342 aa
- **Resolution:** 2.53 Å
- **Alinged residues range:** 220-222, 189-194, 187-191, 134-137, 216-224, 62-67, 196-201
- **Aligned to segment part (indices):** 3, 5, 6, 1, 4, 0, 2
- **Alinged residues range of reference:** 50-52, 203-208, 312-316, 430-433, 543-551, 762-767, 985-990
- **b-phipsi:** 0.006144
- **w-rdist:** 3.205345
- **t-alpha:** 0.00117
- **Chemical similarity (Tanimoto Index) (%):** 94.73
- **1D identity (%) [PDB]:** 0.08
- **1D identity (%) [Gaps excluded][PDB]:** 50.0
- **1D identity - Alignment Gaps [PDB]:** 1321
- **2D identity (%) [PDB]:** 21.69
- **2D identity (%) [Gaps excluded][PDB]:** 83.21
- **2D identity - Alignment Gaps [PDB]:** 777
- **3D similarity (TM-Score) (%) [PDB]:** 14.35

- **Gene name:** HA
- **RefSeq ID:** NC\_002207
- **Genomic sequence length:** 1882
- **5-UTR|CDS|3-UTR identity (%):** N/A | 33.2 | N/A
- **5-UTR|CDS|3-UTR identity (%) [Gaps excluded]:** N/A | 80.53 | N/A
- **5-UTR|CDS|3-UTR identity [Alignment Gaps]:** N/A | 2321 | N/A

**Uniprot Description:**  
  
Binds to sialic acid-containing receptors on the cell surface, bringing about the attachment of the virus particle to the cell. Plays a major role in the determination of host range restriction and virulence. Class I viral fusion protein. Responsible for penetration of the virus into the cell cytoplasm by mediating the fusion of the membrane of the endocytosed virus particle with the endosomal membrane. Low pH in endosomes induce an irreversible conformational change in HA2, releasing the fusion hydrophobic peptide. Several trimers are required to form a competent fusion pore.  
  
Homotrimer of disulfide-linked HA1-HA2.  
  
**Gene Ontology Information:**

Molecular Function

- host cell surface receptor binding

Location

- host cell plasma membrane
- integral component of membrane
- viral envelope
- virion membrane

Biological process

- endocytosis involved in viral entry into host cell
- fusion of virus membrane with host endosome membrane
- fusion of virus membrane with host plasma membrane
- viral budding from plasma membrane
- virion attachment to host cell

---

19

- **Protein name:** Lysozyme
- **Organism:** Streptococcus phage Cp-1
- **Uniprot Accession Number:** P15057
- **Protein sequence length:** 339 aa
- **1D identity (%):** 4.05
- **1D identity (%) [Gaps excluded]:** 32.22
- **1D identity - Alignment Gaps:** 1252
- **Common reported functions (%):** 0.0
- **Common reported locations (%):** 0.0
- **Common reported processes (%):** 0.0

- **PDB ID:** 2J8F
- **Chain:** A
- **Crystallized protein length:** 338 aa
- **Resolution:** 1.84 Å
- **Alinged residues range:** 201-204, 201-204, 122-127, 20-25, 263-266, 20-22, 20-25
- **Aligned to segment part (indices):** 3, 5, 6, 1, 4, 0, 2
- **Alinged residues range of reference:** 50-53, 204-207, 311-316, 365-370, 549-552, 768-770, 1006-1011
- **b-phipsi:** 0.003761
- **w-rdist:** 0.693331
- **t-alpha:** 0.947608
- **Chemical similarity (Tanimoto Index) (%):** 88.9
- **1D identity (%) [PDB]:** 0.08
- **1D identity (%) [Gaps excluded][PDB]:** 100.0
- **1D identity - Alignment Gaps [PDB]:** 1319
- **2D identity (%) [PDB]:** 18.81
- **2D identity (%) [Gaps excluded][PDB]:** 88.74
- **2D identity - Alignment Gaps [PDB]:** 859
- **3D similarity (TM-Score) (%) [PDB]:** 13.91

- **Gene name:** CPL1
- **RefSeq ID:** NC\_001825
- **Genomic sequence length:** 19343
- **5-UTR|CDS|3-UTR identity (%):** N/A | 15.9 | N/A
- **5-UTR|CDS|3-UTR identity (%) [Gaps excluded]:** N/A | 82.06 | N/A
- **5-UTR|CDS|3-UTR identity [Alignment Gaps]:** N/A | 3270 | N/A

**Uniprot Description:**  
  
Responsible for the separation of the host daughter cells at the end of cell division and participates in the liberation of progeny bacteriophage into the medium. Strictly depends on the presence of choline-containing cell walls for activity.  
  
**Gene Ontology Information:**

Molecular Function

- lysozyme activity

Location  
  
N/A

Biological process

- cell wall macromolecule catabolic process
- cytolysis
- defense response to bacterium
- peptidoglycan catabolic process
- viral release from host cell by cytolysis

---

20

- **Protein name:** Ubiquitin-like protein ISG15
- **Organism:** Bos taurus
- **Uniprot Accession Number:** O02741
- **Protein sequence length:** 154 aa
- **1D identity (%):** 2.1
- **1D identity (%) [Gaps excluded]:** 30.77
- **1D identity - Alignment Gaps:** 1245
- **Common reported functions (%):** 0.0
- **Common reported locations (%):** 0.0
- **Common reported processes (%):** 0.0

- **PDB ID:** 6JH1
- **Chain:** D
- **Crystallized protein length:** 147 aa
- **Resolution:** 3.0 Å
- **Alinged residues range:** 92-94, 92-94, 90-94, 120-122, 78-81, 26-31, 29-35
- **Aligned to segment part (indices):** 3, 5, 6, 1, 4, 0, 2
- **Alinged residues range of reference:** 50-52, 204-206, 313-317, 451-453, 594-597, 761-766, 954-960
- **b-phipsi:** 0.002216
- **w-rdist:** 0.776529
- **t-alpha:** 1.060241
- **Chemical similarity (Tanimoto Index) (%):** 83.45
- **1D identity (%) [PDB]:** 0.0
- **1D identity (%) [Gaps excluded][PDB]:** 0.0
- **1D identity - Alignment Gaps [PDB]:** 1130
- **2D identity (%) [PDB]:** 10.56
- **2D identity (%) [Gaps excluded][PDB]:** 91.45
- **2D identity - Alignment Gaps [PDB]:** 896
- **3D similarity (TM-Score) (%) [PDB]:** 7.57

- **Gene name:** ISG15
- **RefSeq ID:** N/A
- **Sequence length:** N/A
- **5-UTR|CDS|3-UTR identity (%):** N/A | N/A | N/A
- **5-UTR|CDS|3-UTR identity (%) [Gaps excluded]:** N/A | N/A | N/A
- **5-UTR|CDS|3-UTR identity [Alignment Gaps]:** N/A | N/A | N/A

**Uniprot Description:**  
  
Ubiquitin-like protein which plays a key role in the innate immune response to viral infection either via its conjugation to a target protein (ISGylation) or via its action as a free or unconjugated protein. ISGylation involves a cascade of enzymatic reactions involving E1, E2, and E3 enzymes which catalyze the conjugation of ISG15 to a lysine residue in the target protein. Exhibits antiviral activity towards both DNA and RNA viruses. The secreted form of ISG15 can: induce natural killer cell proliferation, augment lymphokine-activated-killer (LAK) activity, induce dendritic cell maturation, act as a chemotactic factor for neutrophils and act as a IFN-gamma-inducing cytokine playing an essential role in antimycobacterial immunity (By similarity). The secreted form acts through the integrin ITGAL/ITGB2 receptor to initiate activation of SRC family tyrosine kinases including LYN, HCK and FGR which leads to secretion of IFNG and IL10; the interaction is mediated by ITGAL (By similarity). In response to IFN-tau secreted by the conceptus, may ligate to and regulate proteins involved in the release of prostaglandin F2-alpha (PGF), and thus prevent lysis of the corpus luteum and maintain the pregnancy (PubMed:9546718).  
  
Homodimer; disulfide-linked (By similarity). Interacts with, and is conjugated to its targets by the UBE1L (E1 enzyme) and UBE2E2 (E2 enzyme) (By similarity). Interacts with NEDD4 (By similarity).  
  
**Gene Ontology Information:**

Molecular Function

- integrin binding
- protein tag
- ubiquitin protein ligase binding

Location

- cytoplasm
- cytosolic small ribosomal subunit
- extracellular region
- nucleus

Biological process

- defense response to bacterium
- defense response to virus
- integrin-mediated signaling pathway
- ISG15-protein conjugation
- modification-dependent protein catabolic process
- negative regulation of protein ubiquitination
- negative regulation of type I interferon-mediated signaling pathway
- negative regulation of viral genome replication
- positive regulation of bone mineralization
- positive regulation of erythrocyte differentiation
- positive regulation of interferon-gamma production
- positive regulation of interleukin-10 production
- regulation of interferon-gamma production
- response to type I interferon

---

21

- **Protein name:** Pre-glycoprotein polyprotein GP complex
- **Organism:** Morogoro mammarenavirus
- **Uniprot Accession Number:** C6ZK00
- **Protein sequence length:** 489 aa
- **1D identity (%):** 5.16
- **1D identity (%) [Gaps excluded]:** 24.27
- **1D identity - Alignment Gaps:** 1144
- **Common reported functions (%):** 0.0
- **Common reported locations (%):** 50.0
- **Common reported processes (%):** 10.0

- **PDB ID:** 5NFF
- **Chain:** P
- **Crystallized protein length:** 156 aa
- **Resolution:** 2.62 Å
- **Alinged residues range:** 220-224, 221-224, 220-224, 160-165, 209-213, 129-135, 130-136
- **Aligned to segment part (indices):** 3, 5, 6, 1, 4, 0, 2
- **Alinged residues range of reference:** 47-51, 206-209, 312-316, 394-399, 596-600, 764-770, 957-963
- **b-phipsi:** 0.002161
- **w-rdist:** 3.286276
- **t-alpha:** 0.230216
- **Chemical similarity (Tanimoto Index) (%):** 85.3
- **1D identity (%) [PDB]:** 0.0
- **1D identity (%) [Gaps excluded][PDB]:** 0.0
- **1D identity - Alignment Gaps [PDB]:** 1139
- **2D identity (%) [PDB]:** 10.58
- **2D identity (%) [Gaps excluded][PDB]:** 91.53
- **2D identity - Alignment Gaps [PDB]:** 903
- **3D similarity (TM-Score) (%) [PDB]:** 6.9

- **Gene name:** GPC
- **RefSeq ID:** NC\_013057
- **Genomic sequence length:** 3383
- **5-UTR|CDS|3-UTR identity (%):** N/A | 27.05 | N/A
- **5-UTR|CDS|3-UTR identity (%) [Gaps excluded]:** N/A | 80.03 | N/A
- **5-UTR|CDS|3-UTR identity [Alignment Gaps]:** N/A | 2618 | N/A

**Uniprot Description:**  
  
Glycoprotein G1: interacts with the host receptor.  
  
homotetramer; disulfide-linked.  
  
**Gene Ontology Information:**

Molecular Function

- metal ion binding

Location

- host cell endoplasmic reticulum membrane
- host cell Golgi membrane
- host cell plasma membrane
- integral component of membrane
- viral envelope
- virion membrane

Biological process

- fusion of virus membrane with host endosome membrane
- receptor-mediated endocytosis of virus by host cell
- virion attachment to host cell

---

22

- **Protein name:** N/A
- **Organism:** N/A
- **Uniprot Accession Number:** N/A
- **Protein sequence length:** N/A
- **1D identity (%):** N/A
- **1D identity (%) [Gaps excluded]:** N/A
- **1D identity - Alignment Gaps:** N/A
- **Common reported functions (%):** 0.0
- **Common reported locations (%):** 12.5
- **Common reported processes (%):** 0.0

- **PDB ID:** 6CQQ
- **Chain:** G
- **Crystallized protein length:** 189 aa
- **Resolution:** 2.8 Å
- **Alinged residues range:** 161-163, 156-159, 117-121, 155-159, 99-104, 83-85, 82-86
- **Aligned to segment part (indices):** 3, 5, 6, 1, 4, 0, 2
- **Alinged residues range of reference:** 48-50, 224-227, 312-316, 397-401, 595-600, 768-770, 1005-1009
- **b-phipsi:** 0.016491
- **w-rdist:** 0.839574
- **t-alpha:** 0.73428
- **Chemical similarity (Tanimoto Index) (%):** 99.62
- **1D identity (%) [PDB]:** 0.09
- **1D identity (%) [Gaps excluded][PDB]:** 100.0
- **1D identity - Alignment Gaps [PDB]:** 1170
- **2D identity (%) [PDB]:** 14.16
- **2D identity (%) [Gaps excluded][PDB]:** 88.27
- **2D identity - Alignment Gaps [PDB]:** 848
- **3D similarity (TM-Score) (%) [PDB]:** 8.07

- **Gene name:** N/A
- **RefSeq ID:** N/A
- **Sequence length:** N/A
- **5-UTR|CDS|3-UTR identity (%):** N/A | N/A | N/A
- **5-UTR|CDS|3-UTR identity (%) [Gaps excluded]:** N/A | N/A | N/A
- **5-UTR|CDS|3-UTR identity [Alignment Gaps]:** N/A | N/A | N/A

**Uniprot Description:**  
  
N/A  
  
**Gene Ontology Information:**

Molecular Function

- CD4 receptor binding
- MHC class II protein complex binding
- MHC class II receptor activity
- peptide antigen binding
- polysaccharide binding
- structural constituent of cytoskeleton
- T cell receptor binding

Location

- cell surface
- clathrin-coated endocytic vesicle membrane
- endocytic vesicle membrane
- ER to Golgi transport vesicle membrane
- external side of plasma membrane
- extracellular exosome
- extracellular space
- Golgi membrane
- immunological synapse
- integral component of lumenal side of endoplasmic reticulum membrane
- integral component of plasma membrane
- intermediate filament
- late endosome membrane
- lysosomal membrane
- membrane
- MHC class II protein complex
- plasma membrane
- trans-Golgi network membrane
- transport vesicle membrane

Biological process

- antigen processing and presentation of endogenous peptide antigen via MHC class II
- antigen processing and presentation of exogenous peptide antigen via MHC class II
- detection of bacterium
- epidermis development
- humoral immune response
- immune response
- inflammatory response to antigenic stimulus
- interferon-gamma-mediated signaling pathway
- macrophage differentiation
- myeloid dendritic cell antigen processing and presentation
- negative regulation of inflammatory response to antigenic stimulus
- negative regulation of interferon-gamma production
- negative regulation of T cell proliferation
- peptide antigen assembly with MHC class II protein complex
- positive regulation of CD4-positive, alpha-beta T cell activation
- positive regulation of CD4-positive, CD25-positive, alpha-beta regulatory T cell differentiation
- positive regulation of ERK1 and ERK2 cascade
- positive regulation of I-kappaB kinase/NF-kappaB signaling
- positive regulation of insulin secretion involved in cellular response to glucose stimulus
- positive regulation of kinase activity
- positive regulation of MAPK cascade
- positive regulation of memory T cell differentiation
- positive regulation of monocyte differentiation
- positive regulation of protein phosphorylation
- positive regulation of T cell mediated cytotoxicity
- positive regulation of T cell mediated immune response to tumor cell
- positive regulation of transcription, DNA-templated
- positive regulation of viral entry into host cell
- protein tetramerization
- regulation of interleukin-10 production
- regulation of interleukin-4 production
- regulation of T-helper cell differentiation
- signal transduction
- T cell receptor signaling pathway
- T-helper 1 type immune response

---

23

- **Protein name:** 40S ribosomal protein S11
- **Organism:** Homo sapiens
- **Uniprot Accession Number:** P62280
- **Protein sequence length:** 158 aa
- **1D identity (%):** 3.68
- **1D identity (%) [Gaps excluded]:** 30.72
- **1D identity - Alignment Gaps:** 1125
- **Common reported functions (%):** 0.0
- **Common reported locations (%):** 0.0
- **Common reported processes (%):** 0.0

- **PDB ID:** 5OA3
- **Chain:** L
- **Crystallized protein length:** 151 aa
- **Resolution:** 4.3 Å
- **Alinged residues range:** 142-146, 91-93, 127-130, 85-89, 142-146, 50-52, 48-50
- **Aligned to segment part (indices):** 3, 5, 6, 1, 4, 0, 2
- **Alinged residues range of reference:** 47-51, 224-226, 311-314, 399-403, 584-588, 771-773, 954-956
- **b-phipsi:** 0.018603
- **w-rdist:** 1.085331
- **t-alpha:** 0.565934
- **Chemical similarity (Tanimoto Index) (%):** 73.04
- **1D identity (%) [PDB]:** 0.18
- **1D identity (%) [Gaps excluded][PDB]:** 50.0
- **1D identity - Alignment Gaps [PDB]:** 1126
- **2D identity (%) [PDB]:** 12.45
- **2D identity (%) [Gaps excluded][PDB]:** 84.25
- **2D identity - Alignment Gaps [PDB]:** 842
- **3D similarity (TM-Score) (%) [PDB]:** 6.83

- **Gene name:** RPS11
- **RefSeq ID:** NM\_001015
- **Transcript sequence length:** 68689
- **5-UTR|CDS|3-UTR identity (%):** 30.75 | 3.61 | 0.24
- **5-UTR|CDS|3-UTR identity (%) [Gaps excluded]:** 75.51 | 77.35 | 84.77
- **5-UTR|CDS|3-UTR identity [Alignment Gaps]:** 214 | 3694 | 68047

**Uniprot Description:**  
  
N/A  
  
**Gene Ontology Information:**

Molecular Function

- RNA binding
- rRNA binding
- structural constituent of ribosome

Location

- cytoplasm
- cytosol
- cytosolic ribosome
- cytosolic small ribosomal subunit
- extracellular exosome
- focal adhesion
- membrane
- nucleolus
- nucleoplasm
- ribosome

Biological process

- cytoplasmic translation
- nuclear-transcribed mRNA catabolic process, nonsense-mediated decay
- SRP-dependent cotranslational protein targeting to membrane
- translation
- translational initiation
- viral transcription

---

24

- **Protein name:** Replicase polyprotein 1ab
- **Organism:** Human coronavirus 229E
- **Uniprot Accession Number:** P0C6X1
- **Protein sequence length:** 6758 aa
- **1D identity (%):** 3.98
- **1D identity (%) [Gaps excluded]:** 27.43
- **1D identity - Alignment Gaps:** 5997
- **Common reported functions (%):** 50.0
- **Common reported locations (%):** 12.5
- **Common reported processes (%):** 10.0

- **PDB ID:** 4S1T
- **Chain:** E
- **Crystallized protein length:** 342 aa
- **Resolution:** 2.5 Å
- **Alinged residues range:** 129-133, 280-284, 289-291, 286-295, 32-37, 220-224, 211-216
- **Aligned to segment part (indices):** 3, 5, 6, 1, 4, 0, 2
- **Alinged residues range of reference:** 38-42, 201-205, 315-317, 427-436, 575-580, 769-773, 955-960
- **b-phipsi:** 0.00081
- **w-rdist:** 7.30415
- **t-alpha:** 0.34858
- **Chemical similarity (Tanimoto Index) (%):** 83.34
- **1D identity (%) [PDB]:** 0.0
- **1D identity (%) [Gaps excluded][PDB]:** 0.0
- **1D identity - Alignment Gaps [PDB]:** 1325
- **2D identity (%) [PDB]:** 18.74
- **2D identity (%) [Gaps excluded][PDB]:** 88.74
- **2D identity - Alignment Gaps [PDB]:** 863
- **3D similarity (TM-Score) (%) [PDB]:** 15.65

- **Gene name:** rep
- **RefSeq ID:** NC\_002645
- **Genomic sequence length:** 27317
- **5-UTR|CDS|3-UTR identity (%):** 46.55 | 20.59 | 32.46
- **5-UTR|CDS|3-UTR identity (%) [Gaps excluded]:** 77.51 | 80.83 | 84.38
- **5-UTR|CDS|3-UTR identity [Alignment Gaps]:** 139 | 9550 | 307

**Uniprot Description:**  
  
The replicase polyprotein of coronaviruses is a multifunctional protein: it contains the activities necessary for the transcription of negative stranded RNA, leader RNA, subgenomic mRNAs and progeny virion RNA as well as proteinases responsible for the cleavage of the polyprotein into functional products.  
  
3CL-PRO exists as monomer and homodimer. Eight copies of nsp7 and eight copies of nsp8 assemble to form a heterohexadecamer. Nsp9 is a dimer. Nsp10 forms a dodecamer (By similarity).  
  
**Gene Ontology Information:**

Molecular Function

- ATP binding
- cysteine-type endopeptidase activity
- DNA helicase activity
- endonuclease activity
- exoribonuclease activity
- identical protein binding
- methyltransferase activity
- RNA binding
- RNA helicase activity
- RNA-directed 5'-3' RNA polymerase activity
- thiol-dependent ubiquitin-specific protease activity
- zinc ion binding

Location

- host cell endoplasmic reticulum-Golgi intermediate compartment
- host cell membrane
- host cell perinuclear region of cytoplasm
- integral component of membrane

Biological process

- induction by virus of host autophagy
- methylation
- modulation by virus of host protein ubiquitination
- suppression by virus of host IRF3 activity
- transcription, DNA-templated
- viral protein processing
- viral RNA genome replication

---

25

- **Protein name:** Pre-glycoprotein polyprotein GP complex
- **Organism:** Machupo virus
- **Uniprot Accession Number:** Q8AZ57
- **Protein sequence length:** 496 aa
- **1D identity (%):** 6.84
- **1D identity (%) [Gaps excluded]:** 23.86
- **1D identity - Alignment Gaps:** 981
- **Common reported functions (%):** 0.0
- **Common reported locations (%):** 0.0
- **Common reported processes (%):** 0.0

- **PDB ID:** 5W1M
- **Chain:** S
- **Crystallized protein length:** 151 aa
- **Resolution:** 3.91 Å
- **Alinged residues range:** 216-219, 216-219, 120-125, 106-111, 96-100, 142-146, 187-193
- **Aligned to segment part (indices):** 3, 5, 6, 1, 4, 0, 2
- **Alinged residues range of reference:** 50-53, 204-207, 281-286, 398-403, 544-548, 769-773, 1008-1014
- **b-phipsi:** 0.002499
- **w-rdist:** 2.95044
- **t-alpha:** 0.233766
- **Chemical similarity (Tanimoto Index) (%):** 94.25
- **1D identity (%) [PDB]:** 0.27
- **1D identity (%) [Gaps excluded][PDB]:** 75.0
- **1D identity - Alignment Gaps [PDB]:** 1126
- **2D identity (%) [PDB]:** 8.31
- **2D identity (%) [Gaps excluded][PDB]:** 86.87
- **2D identity - Alignment Gaps [PDB]:** 936
- **3D similarity (TM-Score) (%) [PDB]:** 7.24

- **Gene name:** GPC
- **RefSeq ID:** NC\_005078
- **Genomic sequence length:** 3439
- **5-UTR|CDS|3-UTR identity (%):** N/A | 26.01 | N/A
- **5-UTR|CDS|3-UTR identity (%) [Gaps excluded]:** N/A | 80.05 | N/A
- **5-UTR|CDS|3-UTR identity [Alignment Gaps]:** N/A | 2707 | N/A

**Uniprot Description:**  
  
Glycoprotein G1: interacts with the host receptor.  
  
homotetramer; disulfide-linked.  
  
**Gene Ontology Information:**

Molecular Function  
  
N/A

Location  
  
N/A

Biological process  
  
N/A

---

26

- **Protein name:** Major capsid protein
- **Organism:** Enterobacteria phage HK97
- **Uniprot Accession Number:** P49861
- **Protein sequence length:** 385 aa
- **1D identity (%):** 6.11
- **1D identity (%) [Gaps excluded]:** 25.87
- **1D identity - Alignment Gaps:** 1024
- **Common reported functions (%):** 50.0
- **Common reported locations (%):** 0.0
- **Common reported processes (%):** 0.0

- **PDB ID:** 2GP1
- **Chain:** G
- **Crystallized protein length:** 254 aa
- **Resolution:** 5.2 Å
- **Alinged residues range:** 376-380, 173-180, 376-380, 173-181, 298-301, 353-355, 203-211
- **Aligned to segment part (indices):** 3, 5, 6, 1, 4, 0, 2
- **Alinged residues range of reference:** 47-51, 202-208, 312-316, 394-400, 600-603, 771-773, 1004-1012
- **b-phipsi:** 0.001555
- **w-rdist:** 6.588868
- **t-alpha:** 0.281859
- **Chemical similarity (Tanimoto Index) (%):** N/A
- **1D identity (%) [PDB]:** 0.0
- **1D identity (%) [Gaps excluded][PDB]:** 0.0
- **1D identity - Alignment Gaps [PDB]:** 1238
- **2D identity (%) [PDB]:** 17.1
- **2D identity (%) [Gaps excluded][PDB]:** 87.19
- **2D identity - Alignment Gaps [PDB]:** 832
- **3D similarity (TM-Score) (%) [PDB]:** 9.9

- **Gene name:** 5
- **RefSeq ID:** NC\_002167
- **Genomic sequence length:** 39732
- **5-UTR|CDS|3-UTR identity (%):** N/A | 20.54 | N/A
- **5-UTR|CDS|3-UTR identity (%) [Gaps excluded]:** N/A | 81.46 | N/A
- **5-UTR|CDS|3-UTR identity [Alignment Gaps]:** N/A | 2974 | N/A

**Uniprot Description:**  
  
Assembles to form an icosahedral capsid of 66 nm, with a T=7 laevo symmetry (PubMed:11000116, PubMed:21276801). Responsible for its self-assembly into a procapsid. The phage does not need to encode a separate scaffolfing protein because its capsid protein contains the delta domain that carries that function.  
  
Homopentamer and homohexamer; isoaspartyl lysine isopeptide-linked.  
  
**Gene Ontology Information:**

Molecular Function

- identical protein binding

Location

- T=7 icosahedral viral capsid
- viral capsid

Biological process

- viral procapsid maturation

---

27

- **Protein name:** RNA silencing suppressor p19
- **Organism:** Carnation Italian ringspot virus
- **Uniprot Accession Number:** Q66104
- **Protein sequence length:** 172 aa
- **1D identity (%):** 4.52
- **1D identity (%) [Gaps excluded]:** 35.8
- **1D identity - Alignment Gaps:** 1121
- **Common reported functions (%):** 0.0
- **Common reported locations (%):** 0.0
- **Common reported processes (%):** 0.0

- **PDB ID:** 1RPU
- **Chain:** A
- **Crystallized protein length:** 139 aa
- **Resolution:** 2.5 Å
- **Alinged residues range:** 61-63, 58-63, 120-125, 19-22, 120-125, 83-85, 131-136
- **Aligned to segment part (indices):** 3, 5, 6, 1, 4, 0, 2
- **Alinged residues range of reference:** 49-51, 204-209, 311-316, 413-416, 594-599, 763-765, 953-958
- **b-phipsi:** 0.002342
- **w-rdist:** 1.720868
- **t-alpha:** 0.481802
- **Chemical similarity (Tanimoto Index) (%):** 83.0
- **1D identity (%) [PDB]:** 0.0
- **1D identity (%) [Gaps excluded][PDB]:** 0.0
- **1D identity - Alignment Gaps [PDB]:** 1123
- **2D identity (%) [PDB]:** 8.29
- **2D identity (%) [Gaps excluded][PDB]:** 86.73
- **2D identity - Alignment Gaps [PDB]:** 927
- **3D similarity (TM-Score) (%) [PDB]:** 7.31

- **Gene name:** ORF4
- **RefSeq ID:** NC\_003500
- **Genomic sequence length:** 4763
- **5-UTR|CDS|3-UTR identity (%):** N/A | 9.2 | N/A
- **5-UTR|CDS|3-UTR identity (%) [Gaps excluded]:** N/A | 79.38 | N/A
- **5-UTR|CDS|3-UTR identity [Alignment Gaps]:** N/A | 3439 | N/A

**Uniprot Description:**  
  
Acts as a suppressor of RNA-mediated gene silencing, also known as post-transcriptional gene silencing (PTGS), a mechanism of plant viral defense that limits the accumulation of viral RNAs. Binds to short interfering RNAs (siRNAs) with high affinity. Acts as a molecular caliper to specifically select siRNAs based on the length of the duplex region of the RNA.  
  
Homodimer.  
  
**Gene Ontology Information:**

Molecular Function

- RNA binding

Location

- virion

Biological process  
  
N/A

---

28

- **Protein name:** Endolysin
- **Organism:** Enterobacteria phage T4
- **Uniprot Accession Number:** P00720
- **Protein sequence length:** 164 aa
- **1D identity (%):** 3.27
- **1D identity (%) [Gaps excluded]:** 27.81
- **1D identity - Alignment Gaps:** 1135
- **Common reported functions (%):** 0.0
- **Common reported locations (%):** 0.0
- **Common reported processes (%):** 0.0

- **PDB ID:** 5VBA
- **Chain:** A
- **Crystallized protein length:** 408 aa
- **Resolution:** 2.27 Å
- **Alinged residues range:** 1015-1019, 18-22, 94-98, 201-206, 242-249, 252-257, 251-262
- **Aligned to segment part (indices):** 3, 5, 6, 1, 4, 0, 2
- **Alinged residues range of reference:** 48-52, 200-204, 315-319, 432-437, 548-555, 762-767, 954-964
- **b-phipsi:** 0.00327
- **w-rdist:** 5.133174
- **t-alpha:** 0.016647
- **Chemical similarity (Tanimoto Index) (%):** 82.11
- **1D identity (%) [PDB]:** 0.14
- **1D identity (%) [Gaps excluded][PDB]:** 66.67
- **1D identity - Alignment Gaps [PDB]:** 1389
- **2D identity (%) [PDB]:** 25.92
- **2D identity (%) [Gaps excluded][PDB]:** 90.35
- **2D identity - Alignment Gaps [PDB]:** 773
- **3D similarity (TM-Score) (%) [PDB]:** 13.72

- **Gene name:** E
- **RefSeq ID:** NC\_000866
- **Genomic sequence length:** 168903
- **5-UTR|CDS|3-UTR identity (%):** N/A | 9.19 | N/A
- **5-UTR|CDS|3-UTR identity (%) [Gaps excluded]:** N/A | 80.18 | N/A
- **5-UTR|CDS|3-UTR identity [Alignment Gaps]:** N/A | 3429 | N/A

**Uniprot Description:**  
  
Endolysin with lysozyme activity that degrades host peptidoglycans and participates with the holin and spanin proteins in the sequential events which lead to the programmed host cell lysis releasing the mature viral particles. Once the holin has permeabilized the host cell membrane, the endolysin can reach the periplasm and break down the peptidoglycan layer.  
  
**Gene Ontology Information:**

Molecular Function

- lysozyme activity

Location

- host cell cytoplasm

Biological process

- cell wall macromolecule catabolic process
- cytolysis
- defense response to bacterium
- peptidoglycan catabolic process
- viral release from host cell by cytolysis

---

29

- **Protein name:** L-alanyl-D-glutamate peptidase
- **Organism:** Escherichia phage T5
- **Uniprot Accession Number:** Q6QGP7
- **Protein sequence length:** 137 aa
- **1D identity (%):** 3.02
- **1D identity (%) [Gaps excluded]:** 32.5
- **1D identity - Alignment Gaps:** 1170
- **Common reported functions (%):** 0.0
- **Common reported locations (%):** 0.0
- **Common reported processes (%):** 0.0

- **PDB ID:** 2MXZ
- **Chain:** A
- **Crystallized protein length:** 2740 aa
- **Resolution:** -1.0 Å
- **Alinged residues range:** 106-108, 105-107, 128-130, 75-78, 92-94, 18-23
- **Aligned to segment part (indices):** 5, 6, 1, 4, 0, 2
- **Alinged residues range of reference:** 210-212, 307-309, 415-417, 592-595, 771-773, 975-980
- **b-phipsi:** 0.026523
- **w-rdist:** 1.089638
- **t-alpha:** 0.492147
- **Chemical similarity (Tanimoto Index) (%):** 81.34
- **1D identity (%) [PDB]:** 0.09
- **1D identity (%) [Gaps excluded][PDB]:** 50.0
- **1D identity - Alignment Gaps [PDB]:** 1116
- **2D identity (%) [PDB]:** 8.28
- **2D identity (%) [Gaps excluded][PDB]:** 91.4
- **2D identity - Alignment Gaps [PDB]:** 934
- **3D similarity (TM-Score) (%) [PDB]:** 6.94

- **Gene name:** lys
- **RefSeq ID:** NC\_005859
- **Genomic sequence length:** 121750
- **5-UTR|CDS|3-UTR identity (%):** N/A | 8.16 | N/A
- **5-UTR|CDS|3-UTR identity (%) [Gaps excluded]:** N/A | 80.51 | N/A
- **5-UTR|CDS|3-UTR identity [Alignment Gaps]:** N/A | 3456 | N/A

**Uniprot Description:**  
  
Endolysin with L-alanyl-D-glutamate peptidase activity that degrades host peptidoglycans and participates with the holin in the sequential events which lead to the programmed host cell lysis releasing the mature viral particles. Once the holin has permeabilized the host cell membrane, the endolysin can reach the periplasm and break down the peptidoglycan layer by hydrolyzing the link between L-alanine and D-glutamate residues.  
  
**Gene Ontology Information:**

Molecular Function

- metal ion binding
- peptidase activity

Location  
  
N/A

Biological process

- cell wall organization
- cytolysis
- defense response to bacterium
- viral release from host cell by cytolysis

---

30

- **Protein name:** HLA class I histocompatibility antigen, A alpha chain
- **Organism:** Homo sapiens
- **Uniprot Accession Number:** P04439
- **Protein sequence length:** 365 aa
- **1D identity (%):** 6.03
- **1D identity (%) [Gaps excluded]:** 24.09
- **1D identity - Alignment Gaps:** 982
- **Common reported functions (%):** 0.0
- **Common reported locations (%):** 0.0
- **Common reported processes (%):** 0.0

- **PDB ID:** 4N8V
- **Chain:** A
- **Crystallized protein length:** 274 aa
- **Resolution:** 2.5 Å
- **Alinged residues range:** 99-102, 94-102, 89-94, 88-99, 31-35, 56-61, 68-75
- **Aligned to segment part (indices):** 3, 5, 6, 1, 4, 0, 2
- **Alinged residues range of reference:** 50-53, 200-207, 281-286, 426-436, 573-577, 746-751, 988-995
- **b-phipsi:** 0.027796
- **w-rdist:** 0.888279
- **t-alpha:** 0.689723
- **Chemical similarity (Tanimoto Index) (%):** 84.93
- **1D identity (%) [PDB]:** 0.08
- **1D identity (%) [Gaps excluded][PDB]:** 50.0
- **1D identity - Alignment Gaps [PDB]:** 1253
- **2D identity (%) [PDB]:** 17.14
- **2D identity (%) [Gaps excluded][PDB]:** 86.96
- **2D identity - Alignment Gaps [PDB]:** 843
- **3D similarity (TM-Score) (%) [PDB]:** 8.82

- **Gene name:** HLA-A
- **RefSeq ID:** NM\_002116
- **Transcript sequence length:** 1535
- **5-UTR|CDS|3-UTR identity (%):** 4.4 | 17.53 | 35.36
- **5-UTR|CDS|3-UTR identity (%) [Gaps excluded]:** 85.71 | 75.0 | 78.5
- **5-UTR|CDS|3-UTR identity [Alignment Gaps]:** 259 | 3056 | 244

**Uniprot Description:**  
  
Antigen-presenting major histocompatibility complex class I (MHCI) molecule. In complex with B2M/beta 2 microglobulin displays primarily viral and tumor-derived peptides on antigen-presenting cells for recognition by alpha-beta T cell receptor (TCR) on HLA-A-restricted CD8-positive T cells, guiding antigen-specific T cell immune response to eliminate infected or transformed cells (PubMed:2456340, PubMed:2784196, PubMed:1402688, PubMed:7504010, PubMed:9862734, PubMed:10449296, PubMed:12138174, PubMed:12393434, PubMed:15893615, PubMed:17189421, PubMed:19543285, PubMed:21498667, PubMed:24192765, PubMed:7694806, PubMed:24395804, PubMed:28250417). May also present self-peptides derived from the signal sequence of secreted or membrane proteins, although T cells specific for these peptides are usually inactivated to prevent autoreactivity (PubMed:25880248, PubMed:7506728, PubMed:7679507). Both the peptide and the MHC molecule are recognized by TCR, the peptide is responsible for the fine specificity of antigen recognition and MHC residues account for the MHC restriction of T cells (PubMed:12796775, PubMed:18275829, PubMed:19542454, PubMed:28250417). Typically presents intracellular peptide antigens of 8 to 13 amino acids that arise from cytosolic proteolysis via IFNG-induced immunoproteasome or via endopeptidase IDE/insulin-degrading enzyme (PubMed:17189421, PubMed:20364150, PubMed:17079320, PubMed:26929325, PubMed:27049119). Can bind different peptides containing allele-specific binding motifs, which are mainly defined by anchor residues at position 2 and 9 (PubMed:7504010, PubMed:9862734).  
  
Heterotrimer that consists of an alpha chain HLA-A, a beta chain B2M and a peptide (peptide-HLA-A-B2M) (PubMed:7504010, PubMed:7679507, PubMed:21943705, PubMed:19177349, PubMed:24395804, PubMed:26758806, PubMed:7504010, PubMed:7506728, PubMed:8805302, PubMed:7694806, PubMed:7935798, PubMed:9177355, PubMed:18275829, PubMed:22245737, PubMed:28250417, PubMed:11502003, PubMed:8906788, PubMed:19542454). Early in biogenesis, HLA-A-B2M dimer interacts with the components of the peptide-loading complex composed of TAPBP, TAP1-TAP2, TAPBPL, PDIA3/ERP57 and CALR (PubMed:21263072). Interacts with TAP1-TAP2 transporter via TAPBP; this interaction is obligatory for the loading of peptide epitopes delivered to the ER by TAP1-TAP2 transporter (PubMed:8805302, PubMed:8630735, PubMed:21263072). Interacts with TAPBPL; TAPBPL binds peptide-free HLA-A-B2M complexes or those loaded with low affinity peptides, likely facilitating peptide exchange for higher affinity peptides (PubMed:26869717). Only optimally assembled peptide-HLA-B2M trimer translocates to the surface of antigen-presenting cells, where it interacts with TCR and CD8 coreceptor on the surface of T cells. HLA-A (via polymorphic alpha-1 and alpha-2 domains) interacts with antigen-specific TCR (via CDR3 domains) (PubMed:22245737, PubMed:12796775, PubMed:18275829). One HLA-A molecule (mainly via nonpolymorphic alpha-3 domain) interacts with one CD8A homodimer (via CDR-like loop); this interaction insures peptide-HLA-A-B2M recognition by CD8-positive T cells only (PubMed:9177355, PubMed:2784196). Alleles A\*23:01; A\*24:02 and A\*32:01 interact (via Bw4 motif) with KIR3DL1 on NK cells; this interaction is direct.  
  
**Gene Ontology Information:**

Molecular Function

- beta-2-microglobulin binding
- CD8 receptor binding
- peptide antigen binding
- RNA binding
- signaling receptor binding
- T cell receptor binding
- TAP binding
- TAP complex binding

Location

- cell surface
- early endosome membrane
- endoplasmic reticulum
- endoplasmic reticulum exit site
- endoplasmic reticulum membrane
- ER to Golgi transport vesicle membrane
- extracellular exosome
- Golgi apparatus
- Golgi medial cisterna
- Golgi membrane
- integral component of lumenal side of endoplasmic reticulum membrane
- integral component of plasma membrane
- membrane
- MHC class I peptide loading complex
- MHC class I protein complex
- phagocytic vesicle membrane
- plasma membrane
- recycling endosome membrane

Biological process

- antibacterial humoral response
- antigen processing and presentation of endogenous peptide antigen via MHC class I
- antigen processing and presentation of endogenous peptide antigen via MHC class I via ER pathway, TAP-dependent
- antigen processing and presentation of endogenous peptide antigen via MHC class I via ER pathway, TAP-independent
- antigen processing and presentation of exogenous peptide antigen via MHC class I
- antigen processing and presentation of exogenous peptide antigen via MHC class I, TAP-dependent
- antigen processing and presentation of exogenous peptide antigen via MHC class I, TAP-independent
- antigen processing and presentation of peptide antigen via MHC class I
- CD8-positive, alpha-beta T cell activation
- defense response to Gram-positive bacterium
- detection of bacterium
- immune response
- interferon-gamma-mediated signaling pathway
- positive regulation of CD8-positive, alpha-beta T cell activation
- positive regulation of CD8-positive, alpha-beta T cell proliferation
- positive regulation of interferon-gamma production
- positive regulation of memory T cell activation
- positive regulation of T cell cytokine production
- positive regulation of T cell mediated cytotoxicity
- protection from natural killer cell mediated cytotoxicity
- protein ubiquitination
- regulation of immune response
- T cell mediated cytotoxicity
- T cell mediated cytotoxicity directed against tumor cell target
- T cell receptor signaling pathway
- type I interferon signaling pathway
- viral process

---

31

- **Protein name:** Prohead core protein protease
- **Organism:** Enterobacteria phage T4
- **Uniprot Accession Number:** P06807
- **Protein sequence length:** 212 aa
- **1D identity (%):** 4.83
- **1D identity (%) [Gaps excluded]:** 34.62
- **1D identity - Alignment Gaps:** 1121
- **Common reported functions (%):** 0.0
- **Common reported locations (%):** 0.0
- **Common reported processes (%):** 0.0

- **PDB ID:** 5JBL
- **Chain:** C
- **Crystallized protein length:** 166 aa
- **Resolution:** 1.94 Å
- **Alinged residues range:** 104-106, 109-112, 111-115, 141-145, 143-150, 65-67, 124-132
- **Aligned to segment part (indices):** 3, 5, 6, 1, 4, 0, 2
- **Alinged residues range of reference:** 50-52, 202-205, 315-319, 449-453, 548-555, 771-773, 1009-1019
- **b-phipsi:** 0.001138
- **w-rdist:** 2.376662
- **t-alpha:** 0.689723
- **Chemical similarity (Tanimoto Index) (%):** 81.85
- **1D identity (%) [PDB]:** 0.0
- **1D identity (%) [Gaps excluded][PDB]:** 0.0
- **1D identity - Alignment Gaps [PDB]:** 1150
- **2D identity (%) [PDB]:** 11.59
- **2D identity (%) [Gaps excluded][PDB]:** 89.39
- **2D identity - Alignment Gaps [PDB]:** 886
- **3D similarity (TM-Score) (%) [PDB]:** 8.62

- **Gene name:** 21
- **RefSeq ID:** NC\_000866
- **Genomic sequence length:** 168903
- **5-UTR|CDS|3-UTR identity (%):** N/A | 11.63 | N/A
- **5-UTR|CDS|3-UTR identity (%) [Gaps excluded]:** N/A | 78.88 | N/A
- **5-UTR|CDS|3-UTR identity [Alignment Gaps]:** N/A | 3315 | N/A

**Uniprot Description:**  
  
The pathway of bacteriophage T4 head assembly begins with the formation of a prohead bound to the bacterial cell membrane which is later converted to the mature, DNA-containing head. During maturation, all but one of the prohead proteins are proteolytically processed by a phage-coded protease which is formed by autocatalytic cleavage of the product of gene 21 (gp21). Protease gp21 has been tentatively located in the center of the prohead core.  
  
**Gene Ontology Information:**

Molecular Function

- peptidase activity

Location

- virion

Biological process

- viral procapsid maturation

---

32

- **Protein name:** Gag-Pol polyprotein
- **Organism:** Moloney murine leukemia virus (isolate Shinnick)
- **Uniprot Accession Number:** P03355
- **Protein sequence length:** 1738 aa
- **1D identity (%):** 3.8
- **1D identity (%) [Gaps excluded]:** 26.53
- **1D identity - Alignment Gaps:** 2257
- **Common reported functions (%):** 0.0
- **Common reported locations (%):** 12.5
- **Common reported processes (%):** 10.0

- **PDB ID:** 1NND
- **Chain:** A
- **Crystallized protein length:** 251 aa
- **Resolution:** 2.3 Å
- **Alinged residues range:** 180-182, 180-185, 246-249, 73-77, 145-149, 233-243, 75-81
- **Aligned to segment part (indices):** 3, 5, 6, 1, 4, 0, 2
- **Alinged residues range of reference:** 50-52, 204-209, 295-298, 366-370, 548-552, 762-767, 990-996
- **b-phipsi:** 0.002317
- **w-rdist:** 3.890771
- **t-alpha:** 0.281859
- **Chemical similarity (Tanimoto Index) (%):** 83.51
- **1D identity (%) [PDB]:** 0.0
- **1D identity (%) [Gaps excluded][PDB]:** 0.0
- **1D identity - Alignment Gaps [PDB]:** 1234
- **2D identity (%) [PDB]:** 8.42
- **2D identity (%) [Gaps excluded][PDB]:** 89.62
- **2D identity - Alignment Gaps [PDB]:** 1022
- **3D similarity (TM-Score) (%) [PDB]:** 10.41

- **Gene name:** gag-pol
- **RefSeq ID:** NC\_001501
- **Genomic sequence length:** 8332
- **5-UTR|CDS|3-UTR identity (%):** N/A | 38.4 | N/A
- **5-UTR|CDS|3-UTR identity (%) [Gaps excluded]:** N/A | 76.68 | N/A
- **5-UTR|CDS|3-UTR identity [Alignment Gaps]:** N/A | 3006 | N/A

**Uniprot Description:**  
  
Gag-Pol polyprotein
Plays a role in budding and is processed by the viral protease during virion maturation outside the cell. During budding, it recruits, in a PPXY-dependent or independent manner, Nedd4-like ubiquitin ligases that conjugate ubiquitin molecules to Gag-Pol, or to Gag-Pol binding host factors. Interaction with HECT ubiquitin ligases probably links the viral protein to the host ESCRT pathway and facilitates release.  
  
Capsid protein p30
Homohexamer; further associates as homomultimer (By similarity). The virus core is composed of a lattice formed from hexagonal rings, each containing six capsid monomers (PubMed:12093170). Interacts with mouse UBE2I and mouse PIAS4 (PubMed:16352559).  
  
**Gene Ontology Information:**

Molecular Function

- aspartic-type endopeptidase activity
- DNA binding
- DNA-directed DNA polymerase activity
- retroviral 3' processing activity
- RNA binding
- RNA-directed DNA polymerase activity
- RNA-DNA hybrid ribonuclease activity
- structural constituent of virion
- zinc ion binding

Location

- host cell late endosome membrane
- host cell plasma membrane
- host multivesicular body
- membrane
- protein-DNA complex
- viral nucleocapsid

Biological process

- DNA catabolic process
- DNA recombination
- establishment of integrated proviral latency
- suppression by virus of host gene expression
- viral entry into host cell
- viral genome integration into host DNA
- viral translational readthrough
- virion assembly

---

33

- **Protein name:** DDB1- and CUL4-associated factor 1
- **Organism:** Homo sapiens
- **Uniprot Accession Number:** Q9Y4B6
- **Protein sequence length:** 1507 aa
- **1D identity (%):** 8.57
- **1D identity (%) [Gaps excluded]:** 29.11
- **1D identity - Alignment Gaps:** 1516
- **Common reported functions (%):** 0.0
- **Common reported locations (%):** 0.0
- **Common reported processes (%):** 0.0

- **PDB ID:** 4CC9
- **Chain:** A
- **Crystallized protein length:** 307 aa
- **Resolution:** 2.47 Å
- **Alinged residues range:** 1115-1121, 1371-1374, 1179-1182, 1340-1343, 1080-1085, 1340-1342
- **Aligned to segment part (indices):** 3, 5, 6, 1, 4, 0
- **Alinged residues range of reference:** 46-52, 206-209, 309-312, 421-424, 592-597, 744-746
- **b-phipsi:** 0.010555
- **w-rdist:** 4.638822
- **t-alpha:** 0.007067
- **Chemical similarity (Tanimoto Index) (%):** 83.54
- **1D identity (%) [PDB]:** 0.0
- **1D identity (%) [Gaps excluded][PDB]:** 0.0
- **1D identity - Alignment Gaps [PDB]:** 1291
- **2D identity (%) [PDB]:** 23.35
- **2D identity (%) [Gaps excluded][PDB]:** 91.25
- **2D identity - Alignment Gaps [PDB]:** 765
- **3D similarity (TM-Score) (%) [PDB]:** 10.59

- **Gene name:** DCAF1
- **RefSeq ID:** N/A
- **Sequence length:** N/A
- **5-UTR|CDS|3-UTR identity (%):** N/A | N/A | N/A
- **5-UTR|CDS|3-UTR identity (%) [Gaps excluded]:** N/A | N/A | N/A
- **5-UTR|CDS|3-UTR identity [Alignment Gaps]:** N/A | N/A | N/A

**Uniprot Description:**  
  
Acts both as a substrate recognition component of E3 ubiquitin-protein ligase complexes and as an atypical serine/threonine-protein kinase, playing key roles in various processes such as cell cycle, telomerase regulation and histone modification. Probable substrate-specific adapter of a DCX (DDB1-CUL4-X-box) E3 ubiquitin-protein ligase complex, named CUL4A-RBX1-DDB1-DCAF1/VPRBP complex, which mediates ubiquitination and proteasome-dependent degradation of proteins such as NF2. Involved in the turnover of methylated proteins: recognizes and binds methylated proteins via its chromo domain, leading to ubiquitination of target proteins by the RBX1-DDB1-DCAF1/VPRBP complex (PubMed:23063525). The CUL4A-RBX1-DDB1-DCAF1/VPRBP complex is also involved in B-cell development: DCAF1 is recruited by RAG1 to ubiquitinate proteins, leading to limit error-prone repair during V(D)J recombination. Also part of the EDVP complex, an E3 ligase complex that mediates ubiquitination of proteins such as TERT, leading to TERT degradation and telomerase inhibition (PubMed:23362280). Also acts as an atypical serine/threonine-protein kinase that specifically mediates phosphorylation of 'Thr-120' of histone H2A (H2AT120ph) in a nucleosomal context, thereby repressing transcription. H2AT120ph is present in the regulatory region of many tumor suppresor genes, down-regulates their transcription and is present at high level in a number of tumors (PubMed:24140421). Involved in JNK-mediated apoptosis during cell competition process via its interaction with LLGL1 and LLGL2 (PubMed:20644714).  
  
Component of the DCX (DDB1-CUL4-X-box) E3 ubiquitin-protein ligase complex, named CUL4A-RBX1-DDB1-DCAF1/VPRBP complex. Interacts with DDB1; the interaction is direct. Also forms a ternary complex with DDA1 and DDB1. Interacts with NF2 (via FERM domain). Component of the EDVP complex, a E3 ligase complex containing DYRK2, EDD/UBR5, DDB1 and DCAF1. Interacts with DYRK2; the interaction is direct. Interacts with RAG1; the interaction is direct. Interacts with LLGL1 and LLGL2. Interacts with histone H3. Interacts with ESR1 and LATS1; probably recruited by LATS1 to promote ESR1 ubiquitination and ubiquitin-mediated proteasomal degradation (PubMed:28068668).  
  
**Gene Ontology Information:**

Molecular Function

- ATP binding
- estrogen receptor binding
- histone kinase activity (H2A-T120 specific)
- protein serine kinase activity
- protein serine/threonine kinase activity

Location

- Cul4-RING E3 ubiquitin ligase complex
- cytoplasm
- fibrillar center
- nucleoplasm
- nucleus

Biological process

- B cell differentiation
- cell competition in a multicellular organism
- histone H2A-T120 phosphorylation
- negative regulation of transcription by RNA polymerase II
- protein ubiquitination
- V(D)J recombination
- viral process

---

34

- **Protein name:** Aly/REF export factor 2
- **Organism:** Mus musculus
- **Uniprot Accession Number:** Q9JJW6
- **Protein sequence length:** 218 aa
- **1D identity (%):** 3.58
- **1D identity (%) [Gaps excluded]:** 32.21
- **1D identity - Alignment Gaps:** 1193
- **Common reported functions (%):** 0.0
- **Common reported locations (%):** 0.0
- **Common reported processes (%):** 0.0

- **PDB ID:** 2KT5
- **Chain:** A
- **Crystallized protein length:** 2480 aa
- **Resolution:** -1.0 Å
- **Alinged residues range:** 144-146, 144-147, 105-107, 116-119, 56-59, 129-132, 88-92
- **Aligned to segment part (indices):** 3, 5, 6, 1, 4, 0, 2
- **Alinged residues range of reference:** 51-53, 200-203, 288-290, 375-378, 555-558, 763-766, 988-992
- **b-phipsi:** 0.070887
- **w-rdist:** 0.822797
- **t-alpha:** 0.586271
- **Chemical similarity (Tanimoto Index) (%):** 82.56
- **1D identity (%) [PDB]:** 0.0
- **1D identity (%) [Gaps excluded][PDB]:** 0.0
- **1D identity - Alignment Gaps [PDB]:** 1107
- **2D identity (%) [PDB]:** 9.72
- **2D identity (%) [Gaps excluded][PDB]:** 88.99
- **2D identity - Alignment Gaps [PDB]:** 889
- **3D similarity (TM-Score) (%) [PDB]:** 6.17

- **Gene name:** Alyref2
- **RefSeq ID:** N/A
- **Sequence length:** N/A
- **5-UTR|CDS|3-UTR identity (%):** N/A | N/A | N/A
- **5-UTR|CDS|3-UTR identity (%) [Gaps excluded]:** N/A | N/A | N/A
- **5-UTR|CDS|3-UTR identity [Alignment Gaps]:** N/A | N/A | N/A

**Uniprot Description:**  
  
Export adapter involved in spliced and unspliced mRNA nuclear export. Binds mRNA which is transferred to the NXF1-NXT1 heterodimer for export (TAP/NFX1 pathway); enhances NXF1-NXT1 RNA-binding activity.  
  
Interacts (via N-terminus and RRM domain) with DDX39B and th NXF1-NXT1 heterodimer. Interacts with HHV-1 ICP27 and HVS ORF57 proteins.  
  
**Gene Ontology Information:**

Molecular Function

- RNA binding
- single-stranded DNA binding

Location

- cytoplasm
- spliceosomal complex

Biological process

- mRNA processing
- mRNA transport
- RNA splicing

---

35

- **Protein name:** PC4 domain-containing protein
- **Organism:** Escherichia phage T5
- **Uniprot Accession Number:** Q6QGH2
- **Protein sequence length:** 102 aa
- **1D identity (%):** 1.56
- **1D identity (%) [Gaps excluded]:** 22.47
- **1D identity - Alignment Gaps:** 1197
- **Common reported functions (%):** 0.0
- **Common reported locations (%):** 0.0
- **Common reported processes (%):** 0.0

- **PDB ID:** 4BG7
- **Chain:** B
- **Crystallized protein length:** 88 aa
- **Resolution:** 1.9 Å
- **Alinged residues range:** 21-23, 56-60, 57-60, 78-83, 17-22, 92-94, 90-98
- **Aligned to segment part (indices):** 3, 5, 6, 1, 4, 0, 2
- **Alinged residues range of reference:** 51-53, 205-209, 313-316, 365-370, 549-554, 764-766, 952-960
- **b-phipsi:** 0.001183
- **w-rdist:** 1.838788
- **t-alpha:** 0.819149
- **Chemical similarity (Tanimoto Index) (%):** 81.66
- **1D identity (%) [PDB]:** 0.09
- **1D identity (%) [Gaps excluded][PDB]:** 50.0
- **1D identity - Alignment Gaps [PDB]:** 1067
- **2D identity (%) [PDB]:** 7.67
- **2D identity (%) [Gaps excluded][PDB]:** 95.0
- **2D identity - Alignment Gaps [PDB]:** 911
- **3D similarity (TM-Score) (%) [PDB]:** 5.03

- **Gene name:** T5.115
- **RefSeq ID:** NC\_005859
- **Genomic sequence length:** 121750
- **5-UTR|CDS|3-UTR identity (%):** N/A | 6.22 | N/A
- **5-UTR|CDS|3-UTR identity (%) [Gaps excluded]:** N/A | 82.41 | N/A
- **5-UTR|CDS|3-UTR identity [Alignment Gaps]:** N/A | 3551 | N/A

**Uniprot Description:**  
  
N/A  
  
**Gene Ontology Information:**

Molecular Function

- DNA binding

Location

- host cell nucleus

Biological process

- regulation of transcription, DNA-templated

---

36

- **Protein name:** Decoration protein
- **Organism:** Thermus virus P23-45
- **Uniprot Accession Number:** A7XXC1
- **Protein sequence length:** 146 aa
- **1D identity (%):** 2.66
- **1D identity (%) [Gaps excluded]:** 33.33
- **1D identity - Alignment Gaps:** 1209
- **Common reported functions (%):** 0.0
- **Common reported locations (%):** 0.0
- **Common reported processes (%):** 0.0

- **PDB ID:** 6I9E
- **Chain:** J
- **Crystallized protein length:** 146 aa
- **Resolution:** 3.74 Å
- **Alinged residues range:** 46-48, 4-6, 96-102, 98-104, 14-19, 55-57, 43-47
- **Aligned to segment part (indices):** 3, 5, 6, 1, 4, 0, 2
- **Alinged residues range of reference:** 38-40, 210-212, 312-318, 395-401, 529-534, 735-737, 971-975
- **b-phipsi:** 0.012632
- **w-rdist:** 0.999651
- **t-alpha:** 0.88326
- **Chemical similarity (Tanimoto Index) (%):** 82.22
- **1D identity (%) [PDB]:** 0.18
- **1D identity (%) [Gaps excluded][PDB]:** 50.0
- **1D identity - Alignment Gaps [PDB]:** 1121
- **2D identity (%) [PDB]:** 8.23
- **2D identity (%) [Gaps excluded][PDB]:** 88.54
- **2D identity - Alignment Gaps [PDB]:** 937
- **3D similarity (TM-Score) (%) [PDB]:** 6.71

- **Gene name:** P23p88
- **RefSeq ID:** NC\_009803
- **Genomic sequence length:** 84201
- **5-UTR|CDS|3-UTR identity (%):** N/A | 7.79 | N/A
- **5-UTR|CDS|3-UTR identity (%) [Gaps excluded]:** N/A | 78.65 | N/A
- **5-UTR|CDS|3-UTR identity [Alignment Gaps]:** N/A | 3495 | N/A

**Uniprot Description:**  
  
Cooperatively binds the expanded capsid, thereby stabilizing the mature capsid shell and allowing the large viral DNA to be packaged (PubMed:30737287). Trimers of capsid decoration proteins molecules are located at local and icosahedral threefold axes and stabilize the expanded capsid, which shows increased spacing between capsomers (PubMed:30737287).  
  
Homotrimer (PubMed:30737287). Interacts with the major capsid protein (PubMed:30737287).  
  
**Gene Ontology Information:**

Molecular Function  
  
N/A

Location

- viral capsid, decoration

Biological process  
  
N/A

---

37

- **Protein name:** Replication protein E1
- **Organism:** Bovine papillomavirus type 1
- **Uniprot Accession Number:** P03116
- **Protein sequence length:** 605 aa
- **1D identity (%):** 5.32
- **1D identity (%) [Gaps excluded]:** 26.1
- **1D identity - Alignment Gaps:** 1242
- **Common reported functions (%):** 0.0
- **Common reported locations (%):** 0.0
- **Common reported processes (%):** 0.0

- **PDB ID:** 1KSX
- **Chain:** E
- **Crystallized protein length:** 145 aa
- **Resolution:** 3.2 Å
- **Alinged residues range:** 238-241, 226-233, 165-169, 235-240, 217-221, 160-162, 251-254
- **Aligned to segment part (indices):** 3, 5, 6, 1, 4, 0, 2
- **Alinged residues range of reference:** 49-52, 198-204, 295-299, 395-400, 538-542, 769-771, 956-959
- **b-phipsi:** 0.001847
- **w-rdist:** 2.997079
- **t-alpha:** 0.577491
- **Chemical similarity (Tanimoto Index) (%):** 83.18
- **1D identity (%) [PDB]:** 0.27
- **1D identity (%) [Gaps excluded][PDB]:** 75.0
- **1D identity - Alignment Gaps [PDB]:** 1120
- **2D identity (%) [PDB]:** 9.05
- **2D identity (%) [Gaps excluded][PDB]:** 93.0
- **2D identity - Alignment Gaps [PDB]:** 928
- **3D similarity (TM-Score) (%) [PDB]:** 8.73

- **Gene name:** E1
- **RefSeq ID:** NC\_001522
- **Genomic sequence length:** 7945
- **5-UTR|CDS|3-UTR identity (%):** N/A | 30.82 | N/A
- **5-UTR|CDS|3-UTR identity (%) [Gaps excluded]:** N/A | 79.34 | N/A
- **5-UTR|CDS|3-UTR identity [Alignment Gaps]:** N/A | 2484 | N/A

**Uniprot Description:**  
  
ATP-dependent DNA helicase required for initiation of viral DNA replication. It forms a complex with the viral E2 protein. The E1-E2 complex binds to the replication origin which contains binding sites for both proteins. During the initial step, a dimer of E1 interacts with a dimer of protein E2 leading to a complex that binds the viral origin of replication with high specificity. Then, a second dimer of E1 displaces the E2 dimer in an ATP-dependent manner to form the E1 tetramer. Following this, two E1 monomers are added to each half of the site, which results in the formation of two E1 trimers on the viral ori. Subsequently, two hexamers will be created. The double hexamer acts as a bi-directional helicase machinery and unwinds the viral DNA and then recruits the host DNA polymerase to start replication.  
  
Can form hexamers. Interacts with E2 protein; this interaction increases E1 DNA binding specificity. Interacts with host DNA polymerase subunit POLA2. Interacts with host single stranded DNA-binding protein RPA1. Interacts with host TOP1; this interaction stimulates the enzymatic activity of TOP1.  
  
**Gene Ontology Information:**

Molecular Function

- ATP binding
- DNA binding
- DNA helicase activity
- hydrolase activity, acting on acid anhydrides

Location

- host cell nucleus

Biological process

- DNA replication

---

38

- **Protein name:** Major structural protein 1
- **Organism:** Lactococcus phage Tuc2009
- **Uniprot Accession Number:** Q38610
- **Protein sequence length:** 173 aa
- **1D identity (%):** 3.03
- **1D identity (%) [Gaps excluded]:** 24.84
- **1D identity - Alignment Gaps:** 1132
- **Common reported functions (%):** 0.0
- **Common reported locations (%):** 0.0
- **Common reported processes (%):** 0.0

- **PDB ID:** 5E7F
- **Chain:** I
- **Crystallized protein length:** 128 aa
- **Resolution:** 2.7 Å
- **Alinged residues range:** 160-163, 160-164, 113-118, 132-139, 133-137, 52-55, 50-58
- **Aligned to segment part (indices):** 3, 5, 6, 1, 4, 0, 2
- **Alinged residues range of reference:** 50-53, 204-208, 313-318, 429-436, 572-576, 747-750, 952-960
- **b-phipsi:** 0.028579
- **w-rdist:** 1.069584
- **t-alpha:** 0.641075
- **Chemical similarity (Tanimoto Index) (%):** 83.32
- **1D identity (%) [PDB]:** 0.09
- **1D identity (%) [Gaps excluded][PDB]:** 100.0
- **1D identity - Alignment Gaps [PDB]:** 1109
- **2D identity (%) [PDB]:** 11.22
- **2D identity (%) [Gaps excluded][PDB]:** 90.98
- **2D identity - Alignment Gaps [PDB]:** 867
- **3D similarity (TM-Score) (%) [PDB]:** 7.26

- **Gene name:** N/A
- **RefSeq ID:** NC\_002703
- **Genomic sequence length:** 38347
- **5-UTR|CDS|3-UTR identity (%):** N/A | 8.94 | N/A
- **5-UTR|CDS|3-UTR identity (%) [Gaps excluded]:** N/A | 79.14 | N/A
- **5-UTR|CDS|3-UTR identity [Alignment Gaps]:** N/A | 3462 | N/A

**Uniprot Description:**  
  
N/A  
  
**Gene Ontology Information:**

Molecular Function  
  
N/A

Location  
  
N/A

Biological process  
  
N/A

---

39

- **Protein name:** Genome polyprotein
- **Organism:** Seneca Valley virus (isolate -/United States/SSV-001/2002)
- **Uniprot Accession Number:** Q155Z9
- **Protein sequence length:** 2181 aa
- **1D identity (%):** 11.12
- **1D identity (%) [Gaps excluded]:** 29.17
- **1D identity - Alignment Gaps:** 1548
- **Common reported functions (%):** 0.0
- **Common reported locations (%):** 0.0
- **Common reported processes (%):** 10.0

- **PDB ID:** 6CX1
- **Chain:** C
- **Crystallized protein length:** 268 aa
- **Resolution:** 3.8 Å
- **Alinged residues range:** 114-116, 111-116, 114-116, 40-43, 265-268, 89-91, 248-254
- **Aligned to segment part (indices):** 3, 5, 6, 1, 4, 0, 2
- **Alinged residues range of reference:** 50-52, 201-206, 315-317, 412-415, 547-550, 766-768, 968-974
- **b-phipsi:** 0.006094
- **w-rdist:** 1.24493
- **t-alpha:** 0.663424
- **Chemical similarity (Tanimoto Index) (%):** 83.51
- **1D identity (%) [PDB]:** 0.08
- **1D identity (%) [Gaps excluded][PDB]:** 50.0
- **1D identity - Alignment Gaps [PDB]:** 1247
- **2D identity (%) [PDB]:** 17.05
- **2D identity (%) [Gaps excluded][PDB]:** 85.99
- **2D identity - Alignment Gaps [PDB]:** 837
- **3D similarity (TM-Score) (%) [PDB]:** 11.01

- **Gene name:** N/A
- **RefSeq ID:** NC\_011349
- **Genomic sequence length:** 7310
- **5-UTR|CDS|3-UTR identity (%):** N/A | 36.71 | N/A
- **5-UTR|CDS|3-UTR identity (%) [Gaps excluded]:** N/A | 77.7 | N/A
- **5-UTR|CDS|3-UTR identity [Alignment Gaps]:** N/A | 3714 | N/A

**Uniprot Description:**  
  
Capsid protein VP1
Forms an icosahedral capsid of pseudo T=3 symmetry with capsid proteins VP2 and VP3 (PubMed:18940610). Together they form an icosahedral capsid composed of 60 copies of each VP1, VP2, and VP3, with a diameter of approximately 325 Angstroms (Probable). VP4 lies on the inner surface of the protein shell formed by VP1, VP2 and VP3 (By similarity). All the three latter proteins contain a beta-sheet structure called beta-barrel jelly roll (By similarity). VP1 is situated at the 12 fivefold axes, whereas VP2 and VP3 are located at the quasi-sixfold axes (PubMed:18940610).  
  
Protease 3C
Interacts with host IRF3; this interaction is involved in the suppression of IRF3 and IRF7 expression and phosphorylation by the virus (PubMed:29427864). Interacts with host IRF7; this interaction is involved in the suppression of IRF3 and IRF7 expression and phosphorylation by the virus (PubMed:29427864). Interacts with host MAVS; this interaction allows the cleavage of MAVS and subsequent suppression of host immunity (PubMed:28566380). Interacts with host TRIF; this interaction allows the cleavage of TRIF and subsequent suppression of host immunity (PubMed:28566380). Interacts with host TANK; this interaction allows the cleavage of TANK and subsequent suppression of host immunity (PubMed:28566380). Interacts with host DDX58 (PubMed:30408499). Interacts with host TBK1 (PubMed:30408499). Interacts with host TRAF3 (PubMed:30408499).  
  
**Gene Ontology Information:**

Molecular Function

- ATP binding
- cysteine-type endopeptidase activity
- ion channel activity
- Lys48-specific deubiquitinase activity
- Lys63-specific deubiquitinase activity
- RNA binding
- RNA helicase activity
- RNA-directed 5'-3' RNA polymerase activity
- structural molecule activity
- thiol-dependent ubiquitin-specific protease activity

Location

- host cell cytoplasmic vesicle membrane
- host cell nucleolus
- integral to membrane of host cell
- membrane
- T=3 icosahedral viral capsid
- T=pseudo3 icosahedral viral capsid

Biological process

- pore formation by virus in membrane of host cell
- protein complex oligomerization
- RNA-protein covalent cross-linking
- suppression by virus of host IRF3 activity
- suppression by virus of host IRF7 activity
- suppression by virus of host MAVS activity
- suppression by virus of host RIG-I activity
- suppression by virus of host TBK1 activity
- suppression by virus of host toll-like receptor signaling pathway
- suppression by virus of host TRAF activity
- transcription, DNA-templated
- viral entry into host cell
- viral RNA genome replication
- virion attachment to host cell

---

40

- **Protein name:** Putative capsid protein V20
- **Organism:** Sputnik virophage
- **Uniprot Accession Number:** B4YNG0
- **Protein sequence length:** 595 aa
- **1D identity (%):** 6.98
- **1D identity (%) [Gaps excluded]:** 32.04
- **1D identity - Alignment Gaps:** 1200
- **Common reported functions (%):** 0.0
- **Common reported locations (%):** 0.0
- **Common reported processes (%):** 0.0

- **PDB ID:** 3J26
- **Chain:** L
- **Crystallized protein length:** 508 aa
- **Resolution:** 3.5 Å
- **Alinged residues range:** 465-469, 70-76, 465-469, 73-78, 200-205, 502-505, 373-376
- **Aligned to segment part (indices):** 3, 5, 6, 1, 4, 0, 2
- **Alinged residues range of reference:** 47-51, 202-208, 312-316, 431-436, 549-554, 762-765, 1009-1012
- **b-phipsi:** 0.008001
- **w-rdist:** 9.31674
- **t-alpha:** 0.001171
- **Chemical similarity (Tanimoto Index) (%):** N/A
- **1D identity (%) [PDB]:** 0.07
- **1D identity (%) [Gaps excluded][PDB]:** 50.0
- **1D identity - Alignment Gaps [PDB]:** 1487
- **2D identity (%) [PDB]:** 28.04
- **2D identity (%) [Gaps excluded][PDB]:** 84.64
- **2D identity - Alignment Gaps [PDB]:** 749
- **3D similarity (TM-Score) (%) [PDB]:** 13.74

- **Gene name:** ORF20
- **RefSeq ID:** NC\_011132
- **Genomic sequence length:** 18343
- **5-UTR|CDS|3-UTR identity (%):** N/A | 32.62 | N/A
- **5-UTR|CDS|3-UTR identity (%) [Gaps excluded]:** N/A | 80.84 | N/A
- **5-UTR|CDS|3-UTR identity [Alignment Gaps]:** N/A | 2384 | N/A

**Uniprot Description:**  
  
May self assemble to form an icosahedral capsid. Most abundant protein in the virion.  
  
**Gene Ontology Information:**

Molecular Function  
  
N/A

Location

- viral capsid

Biological process  
  
N/A

---

41

- **Protein name:** RNA2 polyprotein
- **Organism:** Cowpea mosaic virus (strain SB)
- **Uniprot Accession Number:** P03599
- **Protein sequence length:** 1046 aa
- **1D identity (%):** 15.28
- **1D identity (%) [Gaps excluded]:** 27.93
- **1D identity - Alignment Gaps:** 679
- **Common reported functions (%):** 0.0
- **Common reported locations (%):** 0.0
- **Common reported processes (%):** 0.0

- **PDB ID:** 5A32
- **Chain:** B
- **Crystallized protein length:** 368 aa
- **Resolution:** 3.44 Å
- **Alinged residues range:** 168-171, 248-251, 169-171, 78-85, 149-156, 105-108, 233-236
- **Aligned to segment part (indices):** 3, 5, 6, 1, 4, 0, 2
- **Alinged residues range of reference:** 48-51, 201-204, 314-316, 423-430, 594-601, 768-771, 1005-1008
- **b-phipsi:** 0.011923
- **w-rdist:** 1.307139
- **t-alpha:** 0.535009
- **Chemical similarity (Tanimoto Index) (%):** 83.64
- **1D identity (%) [PDB]:** 0.0
- **1D identity (%) [Gaps excluded][PDB]:** 0.0
- **1D identity - Alignment Gaps [PDB]:** 1351
- **2D identity (%) [PDB]:** 22.05
- **2D identity (%) [Gaps excluded][PDB]:** 89.51
- **2D identity - Alignment Gaps [PDB]:** 817
- **3D similarity (TM-Score) (%) [PDB]:** 10.78

- **Gene name:** N/A
- **RefSeq ID:** NC\_003550
- **Genomic sequence length:** 3481
- **5-UTR|CDS|3-UTR identity (%):** N/A | 42.09 | N/A
- **5-UTR|CDS|3-UTR identity (%) [Gaps excluded]:** N/A | 78.66 | N/A
- **5-UTR|CDS|3-UTR identity [Alignment Gaps]:** N/A | 2109 | N/A

**Uniprot Description:**  
  
VP58
Responsible for viral RNA2 accumulation. May function by recruiting the RNA1-encoded polyprotein that contains the replication protein to RNA2 and enable its replication.  
  
Mature small capsid protein
Interacts with the large capsid protein (PubMed:26657148, PubMed:10603314).  
  
**Gene Ontology Information:**

Molecular Function

- DNA binding
- GTP binding
- RNA binding
- structural molecule activity

Location

- host cell nucleus
- host cell plasmodesma
- T=3 icosahedral viral capsid

Biological process

- transport of virus in host, cell to cell

---

42

- **Protein name:** Genome polyprotein
- **Organism:** Bovine viral diarrhea virus (isolate NADL)
- **Uniprot Accession Number:** P19711
- **Protein sequence length:** 3988 aa
- **1D identity (%):** 6.66
- **1D identity (%) [Gaps excluded]:** 29.55
- **1D identity - Alignment Gaps:** 3325
- **Common reported functions (%):** 50.0
- **Common reported locations (%):** 25.0
- **Common reported processes (%):** 20.0

- **PDB ID:** 4ILD
- **Chain:** B
- **Crystallized protein length:** 237 aa
- **Resolution:** 3.27 Å
- **Alinged residues range:** 990-993, 894-899, 990-993, 986-990, 954-959, 970-973
- **Aligned to segment part (indices):** 3, 5, 6, 1, 4, 2
- **Alinged residues range of reference:** 49-52, 203-208, 314-317, 374-378, 593-598, 972-975
- **b-phipsi:** 0.031569
- **w-rdist:** 0.689931
- **t-alpha:** 0.819149
- **Chemical similarity (Tanimoto Index) (%):** N/A
- **1D identity (%) [PDB]:** 0.08
- **1D identity (%) [Gaps excluded][PDB]:** 50.0
- **1D identity - Alignment Gaps [PDB]:** 1217
- **2D identity (%) [PDB]:** 15.03
- **2D identity (%) [Gaps excluded][PDB]:** 85.25
- **2D identity - Alignment Gaps [PDB]:** 855
- **3D similarity (TM-Score) (%) [PDB]:** 11.94

- **Gene name:** N/A
- **RefSeq ID:** NC\_001461
- **Genomic sequence length:** 12573
- **5-UTR|CDS|3-UTR identity (%):** N/A | 21.77 | N/A
- **5-UTR|CDS|3-UTR identity (%) [Gaps excluded]:** N/A | 79.67 | N/A
- **5-UTR|CDS|3-UTR identity [Alignment Gaps]:** N/A | 9011 | N/A

**Uniprot Description:**  
  
N-terminal protease
Leader cysteine autoprotease that cleaves itself from the nascent polyprotein during translation of the viral mRNA. Once released, plays a role in the inhibition of host innate immune response by interacting with host IRF3 and inducing its proteasomal degradation.  
  
E(rns) glycoprotein
Homodimer; disulfide-linked.  
  
**Gene Ontology Information:**

Molecular Function

- ATP binding
- cysteine-type endopeptidase activity
- DNA binding
- DNA/DNA annealing activity
- identical protein binding
- ion channel activity
- nucleoside-triphosphatase activity
- ribonuclease T2 activity
- RNA binding
- RNA helicase activity
- RNA strand annealing activity
- RNA-directed 5'-3' RNA polymerase activity
- serine-type endopeptidase activity
- serine-type exopeptidase activity

Location

- host cell cytoplasm
- host cell cytoplasmic vesicle
- host cell Golgi apparatus
- host cell mitochondrion
- host cell nucleus
- host cell surface
- integral component of membrane
- integral to membrane of host cell
- protein-DNA complex
- ribonucleoprotein complex
- virion membrane

Biological process

- clathrin-dependent endocytosis of virus by host cell
- fusion of virus membrane with host endosome membrane
- induction by virus of host autophagy
- pore formation by virus in membrane of host cell
- protein complex oligomerization
- RNA stabilization
- suppression by virus of host IRF3 activity
- viral protein processing
- viral RNA genome replication
- virion attachment to host cell

---

43

- **Protein name:** Polymerase basic protein 2
- **Organism:** Thogoto virus (isolate SiAr 126)
- **Uniprot Accession Number:** Q9YNA4
- **Protein sequence length:** 769 aa
- **1D identity (%):** 13.75
- **1D identity (%) [Gaps excluded]:** 29.25
- **1D identity - Alignment Gaps:** 736
- **Common reported functions (%):** 0.0
- **Common reported locations (%):** 0.0
- **Common reported processes (%):** 0.0

- **PDB ID:** 4CHE
- **Chain:** A
- **Crystallized protein length:** 161 aa
- **Resolution:** 1.8 Å
- **Alinged residues range:** 337-340, 370-376, 359-363, 371-375, 359-363, 406-414, 404-409
- **Aligned to segment part (indices):** 3, 5, 6, 1, 4, 0, 2
- **Alinged residues range of reference:** 47-50, 201-207, 312-316, 394-398, 595-599, 761-769, 959-964
- **b-phipsi:** 0.057499
- **w-rdist:** 0.832039
- **t-alpha:** 0.67319
- **Chemical similarity (Tanimoto Index) (%):** 83.45
- **1D identity (%) [PDB]:** 0.0
- **1D identity (%) [Gaps excluded][PDB]:** 0.0
- **1D identity - Alignment Gaps [PDB]:** 1144
- **2D identity (%) [PDB]:** 12.1
- **2D identity (%) [Gaps excluded][PDB]:** 89.71
- **2D identity - Alignment Gaps [PDB]:** 872
- **3D similarity (TM-Score) (%) [PDB]:** 7.14

- **Gene name:** Segment 1
- **RefSeq ID:** NC\_006508
- **Genomic sequence length:** 2375
- **5-UTR|CDS|3-UTR identity (%):** N/A | 32.97 | N/A
- **5-UTR|CDS|3-UTR identity (%) [Gaps excluded]:** N/A | 78.17 | N/A
- **5-UTR|CDS|3-UTR identity [Alignment Gaps]:** N/A | 2494 | N/A

**Uniprot Description:**  
  
subunit of the RNA-dependent RNA polymerase which is responsible for replication and transcription of virus RNA segments. The transcription of viral mRNAs occurs by a unique mechanism called cap-snatching. 5' methylated caps of cellular mRNAs are cleaved after 10-13 nucleotides by PA. In turn, these short capped RNAs are used as primers by PB1 for transcription of viral mRNAs. During virus replication, PB1 initiates RNA synthesis and copy vRNA into complementary RNA (cRNA) which in turn serves as a template for the production of more vRNAs.  
  
RNA polymerase is composed of three subunits: PA, PB1 and PB2.  
  
**Gene Ontology Information:**

Molecular Function

- RNA binding

Location

- host cell nucleus
- virion

Biological process

- 7-methylguanosine mRNA capping
- cap snatching
- viral RNA genome replication

---

44

- **Protein name:** Capsid protein alpha
- **Organism:** Flock house virus
- **Uniprot Accession Number:** P12870
- **Protein sequence length:** 407 aa
- **1D identity (%):** 4.96
- **1D identity (%) [Gaps excluded]:** 31.44
- **1D identity - Alignment Gaps:** 1222
- **Common reported functions (%):** 0.0
- **Common reported locations (%):** 0.0
- **Common reported processes (%):** 0.0

- **PDB ID:** 4FTE
- **Chain:** A
- **Crystallized protein length:** 328 aa
- **Resolution:** 3.5 Å
- **Alinged residues range:** 312-316, 170-174, 189-192, 103-113, 173-178, 349-351, 362-375
- **Aligned to segment part (indices):** 3, 5, 6, 1, 4, 0, 2
- **Alinged residues range of reference:** 47-51, 200-204, 311-314, 395-401, 595-600, 769-771, 954-964
- **b-phipsi:** 0.020651
- **w-rdist:** 1.193658
- **t-alpha:** 0.598131
- **Chemical similarity (Tanimoto Index) (%):** 83.91
- **1D identity (%) [PDB]:** 0.0
- **1D identity (%) [Gaps excluded][PDB]:** 0.0
- **1D identity - Alignment Gaps [PDB]:** 1311
- **2D identity (%) [PDB]:** 22.11
- **2D identity (%) [Gaps excluded][PDB]:** 86.84
- **2D identity - Alignment Gaps [PDB]:** 779
- **3D similarity (TM-Score) (%) [PDB]:** 10.33

- **Gene name:** alpha
- **RefSeq ID:** NC\_004144
- **Genomic sequence length:** 1400
- **5-UTR|CDS|3-UTR identity (%):** N/A | 20.67 | N/A
- **5-UTR|CDS|3-UTR identity (%) [Gaps excluded]:** N/A | 77.35 | N/A
- **5-UTR|CDS|3-UTR identity [Alignment Gaps]:** N/A | 2918 | N/A

**Uniprot Description:**  
  
Capsid protein alpha self-assembles to form an icosahedral procapsid with a T=3 symmetry, about 30 nm in diameter, and consisting of 60 capsid proteins trimers. In addition, 240 calcium ions are incorporated per capsid during assembly. The capsid encapsulates the two genomic RNAs. Capsid maturation occurs via autoproteolytic cleavage of capsid protein alpha generating capsid protein beta and the membrane-active peptide gamma.  
  
**Gene Ontology Information:**

Molecular Function

- aspartic-type endopeptidase activity
- metal ion binding

Location

- T=3 icosahedral viral capsid

Biological process

- permeabilization of host organelle membrane involved in viral entry into host cell
- viral entry via permeabilization of inner membrane

---

45

- **Protein name:** Polymerase acidic protein
- **Organism:** Thogoto virus (isolate SiAr 126)
- **Uniprot Accession Number:** P27194
- **Protein sequence length:** 622 aa
- **1D identity (%):** 7.7
- **1D identity (%) [Gaps excluded]:** 22.71
- **1D identity - Alignment Gaps:** 935
- **Common reported functions (%):** 0.0
- **Common reported locations (%):** 0.0
- **Common reported processes (%):** 0.0

- **PDB ID:** 4CGX
- **Chain:** B
- **Crystallized protein length:** 158 aa
- **Resolution:** 2.7 Å
- **Alinged residues range:** 114-116, 114-116, 37-39, 94-97, 105-109, 136-152, 133-154
- **Aligned to segment part (indices):** 3, 5, 6, 1, 4, 0, 2
- **Alinged residues range of reference:** 51-53, 205-207, 297-299, 434-437, 527-531, 761-770, 984-1009
- **b-phipsi:** 0.029846
- **w-rdist:** 1.031276
- **t-alpha:** 0.720322
- **Chemical similarity (Tanimoto Index) (%):** 81.66
- **1D identity (%) [PDB]:** 0.0
- **1D identity (%) [Gaps excluded][PDB]:** 0.0
- **1D identity - Alignment Gaps [PDB]:** 1143
- **2D identity (%) [PDB]:** 9.94
- **2D identity (%) [Gaps excluded][PDB]:** 87.18
- **2D identity - Alignment Gaps [PDB]:** 909
- **3D similarity (TM-Score) (%) [PDB]:** 7.37

- **Gene name:** Segment 3
- **RefSeq ID:** NC\_006496
- **Genomic sequence length:** 1927
- **5-UTR|CDS|3-UTR identity (%):** N/A | 31.25 | N/A
- **5-UTR|CDS|3-UTR identity (%) [Gaps excluded]:** N/A | 79.61 | N/A
- **5-UTR|CDS|3-UTR identity [Alignment Gaps]:** N/A | 2483 | N/A

**Uniprot Description:**  
  
subunit of the RNA-dependent RNA polymerase which is responsible for replication and transcription of virus RNA segments. The transcription of viral mRNAs occurs by a unique mechanism called cap-snatching. 5' methylated caps of cellular mRNAs are cleaved after 10-13 nucleotides by PA. In turn, these short capped RNAs are used as primers by PB1 for transcription of viral mRNAs. During virus replication, PB1 initiates RNA synthesis and copy vRNA into complementary RNA (cRNA) which in turn serves as a template for the production of more vRNAs.  
  
RNA polymerase is composed of three subunits: PA, PB1 and PB2.  
  
**Gene Ontology Information:**

Molecular Function

- RNA binding

Location

- host cell nucleus
- virion

Biological process

- viral RNA genome replication

---

46

- **Protein name:** PHIKZ029
- **Organism:** Pseudomonas phage phiKZ
- **Uniprot Accession Number:** Q8SDD3
- **Protein sequence length:** 695 aa
- **1D identity (%):** 11.71
- **1D identity (%) [Gaps excluded]:** 28.87
- **1D identity - Alignment Gaps:** 832
- **Common reported functions (%):** 0.0
- **Common reported locations (%):** 0.0
- **Common reported processes (%):** 0.0

- **PDB ID:** 3J0H
- **Chain:** D
- **Crystallized protein length:** 252 aa
- **Resolution:** 18.0 Å
- **Alinged residues range:** 149-152, 205-210, 149-154, 177-184, 208-213, 278-283, 312-317
- **Aligned to segment part (indices):** 3, 5, 6, 1, 4, 0, 2
- **Alinged residues range of reference:** 49-52, 200-205, 314-319, 438-449, 597-602, 768-773, 1011-1016
- **b-phipsi:** 0.01401
- **w-rdist:** 1.245737
- **t-alpha:** 0.601124
- **Chemical similarity (Tanimoto Index) (%):** 82.24
- **1D identity (%) [PDB]:** 0.0
- **1D identity (%) [Gaps excluded][PDB]:** 0.0
- **1D identity - Alignment Gaps [PDB]:** 1237
- **2D identity (%) [PDB]:** 15.03
- **2D identity (%) [Gaps excluded][PDB]:** 84.95
- **2D identity - Alignment Gaps [PDB]:** 865
- **3D similarity (TM-Score) (%) [PDB]:** 11.2

- **Gene name:** N/A
- **RefSeq ID:** NC\_004629
- **Genomic sequence length:** 280334
- **5-UTR|CDS|3-UTR identity (%):** N/A | 36.54 | N/A
- **5-UTR|CDS|3-UTR identity (%) [Gaps excluded]:** N/A | 78.21 | N/A
- **5-UTR|CDS|3-UTR identity [Alignment Gaps]:** N/A | 2146 | N/A

**Uniprot Description:**  
  
N/A  
  
**Gene Ontology Information:**

Molecular Function  
  
N/A

Location  
  
N/A

Biological process  
  
N/A

---

47

- **Protein name:** Uncharacterized protein
- **Organism:** Sulfolobus turreted icosahedral virus 1
- **Uniprot Accession Number:** Q6Q0L5
- **Protein sequence length:** 197 aa
- **1D identity (%):** 3.38
- **1D identity (%) [Gaps excluded]:** 26.35
- **1D identity - Alignment Gaps:** 1136
- **Common reported functions (%):** 0.0
- **Common reported locations (%):** 0.0
- **Common reported processes (%):** 0.0

- **PDB ID:** 2C0N
- **Chain:** A
- **Crystallized protein length:** 187 aa
- **Resolution:** 1.86 Å
- **Alinged residues range:** 168-172, 166-171, 169-173, 84-89, 162-166, 37-40, 73-80
- **Aligned to segment part (indices):** 3, 5, 6, 1, 4, 0, 2
- **Alinged residues range of reference:** 48-52, 204-209, 314-318, 433-438, 549-553, 770-773, 953-960
- **b-phipsi:** 0.002408
- **w-rdist:** 3.019987
- **t-alpha:** 0.489547
- **Chemical similarity (Tanimoto Index) (%):** 81.51
- **1D identity (%) [PDB]:** 0.0
- **1D identity (%) [Gaps excluded][PDB]:** 0.0
- **1D identity - Alignment Gaps [PDB]:** 1174
- **2D identity (%) [PDB]:** 12.04
- **2D identity (%) [Gaps excluded][PDB]:** 80.92
- **2D identity - Alignment Gaps [PDB]:** 870
- **3D similarity (TM-Score) (%) [PDB]:** 9.36

- **Gene name:** A197
- **RefSeq ID:** NC\_005892
- **Genomic sequence length:** 17663
- **5-UTR|CDS|3-UTR identity (%):** N/A | 10.66 | N/A
- **5-UTR|CDS|3-UTR identity (%) [Gaps excluded]:** N/A | 80.93 | N/A
- **5-UTR|CDS|3-UTR identity [Alignment Gaps]:** N/A | 3388 | N/A

**Uniprot Description:**  
  
N/A  
  
**Gene Ontology Information:**

Molecular Function  
  
N/A

Location  
  
N/A

Biological process  
  
N/A

---

48

- **Protein name:** Ubiquitin-like protein ISG15
- **Organism:** Mus musculus
- **Uniprot Accession Number:** Q64339
- **Protein sequence length:** 161 aa
- **1D identity (%):** 2.94
- **1D identity (%) [Gaps excluded]:** 27.14
- **1D identity - Alignment Gaps:** 1154
- **Common reported functions (%):** 0.0
- **Common reported locations (%):** 0.0
- **Common reported processes (%):** 0.0

- **PDB ID:** 6YVA
- **Chain:** C
- **Crystallized protein length:** 155 aa
- **Resolution:** 3.18 Å
- **Alinged residues range:** 92-95, 92-94, 91-95, 121-123, 124-128, 26-29, 103-111
- **Aligned to segment part (indices):** 3, 5, 6, 1, 4, 0, 2
- **Alinged residues range of reference:** 49-52, 202-204, 313-317, 451-453, 543-547, 761-764, 1002-1010
- **b-phipsi:** 0.001734
- **w-rdist:** 1.49571
- **t-alpha:** 0.846652
- **Chemical similarity (Tanimoto Index) (%):** 82.81
- **1D identity (%) [PDB]:** 0.0
- **1D identity (%) [Gaps excluded][PDB]:** 0.0
- **1D identity - Alignment Gaps [PDB]:** 1138
- **2D identity (%) [PDB]:** 11.06
- **2D identity (%) [Gaps excluded][PDB]:** 89.6
- **2D identity - Alignment Gaps [PDB]:** 888
- **3D similarity (TM-Score) (%) [PDB]:** 7.41

- **Gene name:** Isg15
- **RefSeq ID:** N/A
- **Sequence length:** N/A
- **5-UTR|CDS|3-UTR identity (%):** N/A | N/A | N/A
- **5-UTR|CDS|3-UTR identity (%) [Gaps excluded]:** N/A | N/A | N/A
- **5-UTR|CDS|3-UTR identity [Alignment Gaps]:** N/A | N/A | N/A

**Uniprot Description:**  
  
Ubiquitin-like protein which plays a key role in the innate immune response to viral infection either via its conjugation to a target protein (ISGylation) or via its action as a free or unconjugated protein. ISGylation involves a cascade of enzymatic reactions involving E1, E2, and E3 enzymes which catalyze the conjugation of ISG15 to a lysine residue in the target protein. Its target proteins include SERPINA3G/SPI2A, JAK1, MAPK3/ERK1, PLCG1, TRIM25, STAT5A, MAPK1/ERK2 and globin. Can also isgylate: DDX58/RIG-I which inhibits its function in antiviral signaling response, IRF3 which inhibits its ubiquitination and degradation as well as EIF4E2 which enhances its cap structure-binding activity and translation-inhibition activity. Exhibits antiviral activity towards both DNA and RNA viruses, including influenza A and B virus, sindbis virus (SV) and herpes simplex type-1 (HHV-1). Plays a significant role in the control of neonatal Chikungunya virus (CHIKV) infection by acting as a putative immunomodulator of proinflammatory cytokines. Protects mice against the consequences of Chikungunya virus infection by downregulating the pathogenic cytokine response, often denoted as the cytokine storm. Plays a role in erythroid differentiation. The secreted form of ISG15 can: induce natural killer cell proliferation, act as a chemotactic factor for neutrophils and act as a IFN-gamma-inducing cytokine playing an essential role in antimycobacterial immunity. The secreted form acts through the integrin ITGAL/ITGB2 receptor to initiate activation of SRC family tyrosine kinases including LYN, HCK and FGR which leads to secretion of IFNG and IL10; the interaction is mediated by ITGAL (By similarity).  
  
Homodimer; disulfide-linked (By similarity). Interacts with, and is conjugated to its targets by the UBE1L (E1 enzyme) and UBE2E2 (E2 enzyme) (By similarity). Interacts with NEDD4 (By similarity).  
  
**Gene Ontology Information:**

Molecular Function

- integrin binding
- protein tag
- ubiquitin protein ligase binding

Location

- cytoplasm
- cytosolic small ribosomal subunit
- extracellular region
- nucleus

Biological process

- defense response to bacterium
- defense response to virus
- integrin-mediated signaling pathway
- ISG15-protein conjugation
- modification-dependent protein catabolic process
- negative regulation of protein ubiquitination
- negative regulation of type I interferon-mediated signaling pathway
- negative regulation of viral genome replication
- positive regulation of bone mineralization
- positive regulation of erythrocyte differentiation
- positive regulation of interferon-gamma production
- positive regulation of interleukin-10 production
- regulation of interferon-gamma production
- response to bacterium
- response to type I interferon

---

49

- **Protein name:** DNA-directed RNA polymerase subunit beta
- **Organism:** Thermus thermophilus (strain HB8 / ATCC 27634 / DSM 579)
- **Uniprot Accession Number:** Q8RQE9
- **Protein sequence length:** 1119 aa
- **1D identity (%):** 13.58
- **1D identity (%) [Gaps excluded]:** 26.58
- **1D identity - Alignment Gaps:** 774
- **Common reported functions (%):** 0.0
- **Common reported locations (%):** 0.0
- **Common reported processes (%):** 0.0

- **PDB ID:** 3WOF
- **Chain:** G
- **Crystallized protein length:** 129 aa
- **Resolution:** 3.3 Å
- **Alinged residues range:** 706-709, 708-711, 711-713, 794-797, 798-800, 771-777, 771-773
- **Aligned to segment part (indices):** 3, 5, 6, 1, 4, 0, 2
- **Alinged residues range of reference:** 49-52, 225-228, 317-319, 412-415, 596-598, 764-770, 994-996
- **b-phipsi:** 0.02651
- **w-rdist:** 0.97018
- **t-alpha:** 0.904232
- **Chemical similarity (Tanimoto Index) (%):** 67.8
- **1D identity (%) [PDB]:** 0.0
- **1D identity (%) [Gaps excluded][PDB]:** 0.0
- **1D identity - Alignment Gaps [PDB]:** 1112
- **2D identity (%) [PDB]:** 9.07
- **2D identity (%) [Gaps excluded][PDB]:** 75.0
- **2D identity - Alignment Gaps [PDB]:** 872
- **3D similarity (TM-Score) (%) [PDB]:** 6.51

- **Gene name:** rpoB
- **RefSeq ID:** N/A
- **Sequence length:** N/A
- **5-UTR|CDS|3-UTR identity (%):** N/A | N/A | N/A
- **5-UTR|CDS|3-UTR identity (%) [Gaps excluded]:** N/A | N/A | N/A
- **5-UTR|CDS|3-UTR identity [Alignment Gaps]:** N/A | N/A | N/A

**Uniprot Description:**  
  
DNA-dependent RNA polymerase catalyzes the transcription of DNA into RNA using the four ribonucleoside triphosphates as substrates.  
  
The RNAP catalytic core consists of 2 alpha, 1 beta, 1 beta' and 1 omega subunit. When a sigma factor is associated with the core the holoenzyme is formed, which can initiate transcription.  
  
**Gene Ontology Information:**

Molecular Function

- DNA binding
- DNA-directed 5'-3' RNA polymerase activity
- ribonucleoside binding

Location  
  
N/A

Biological process

- transcription, DNA-templated

---

50

- **Protein name:** Envelope glycoprotein
- **Organism:** Lake Victoria marburgvirus (strain Ravn-87)
- **Uniprot Accession Number:** Q1PDC7
- **Protein sequence length:** 681 aa
- **1D identity (%):** 9.94
- **1D identity (%) [Gaps excluded]:** 28.46
- **1D identity - Alignment Gaps:** 942
- **Common reported functions (%):** 0.0
- **Common reported locations (%):** 50.0
- **Common reported processes (%):** 10.0

- **PDB ID:** 6BP2
- **Chain:** A
- **Crystallized protein length:** 148 aa
- **Resolution:** 3.17 Å
- **Alinged residues range:** 116-124, 124-128, 161-163, 159-162, 81-84, 175-177, 174-178
- **Aligned to segment part (indices):** 3, 5, 6, 1, 4, 0, 2
- **Alinged residues range of reference:** 45-53, 202-206, 313-315, 434-437, 524-527, 764-766, 993-997
- **b-phipsi:** 0.01514
- **w-rdist:** 1.167727
- **t-alpha:** 0.8
- **Chemical similarity (Tanimoto Index) (%):** N/A
- **1D identity (%) [PDB]:** 0.18
- **1D identity (%) [Gaps excluded][PDB]:** 66.67
- **1D identity - Alignment Gaps [PDB]:** 1125
- **2D identity (%) [PDB]:** 9.39
- **2D identity (%) [Gaps excluded][PDB]:** 88.07
- **2D identity - Alignment Gaps [PDB]:** 913
- **3D similarity (TM-Score) (%) [PDB]:** 7.54

- **Gene name:** GP
- **RefSeq ID:** NC\_024781
- **Genomic sequence length:** 19114
- **5-UTR|CDS|3-UTR identity (%):** N/A | 32.8 | N/A
- **5-UTR|CDS|3-UTR identity (%) [Gaps excluded]:** N/A | 78.78 | N/A
- **5-UTR|CDS|3-UTR identity [Alignment Gaps]:** N/A | 2418 | N/A

**Uniprot Description:**  
  
GP1 is responsible for binding to the receptor(s) on target cells. Interacts with CD209/DC-SIGN and CLEC4M/DC-SIGNR which act as cofactors for virus entry into the host cell. Binding to CD209 and CLEC4M, which are respectively found on dendritic cells (DCs), and on endothelial cells of liver sinusoids and lymph node sinuses, facilitate infection of macrophages and endothelial cells. These interactions not only facilitate virus cell entry, but also allow capture of viral particles by DCs and subsequent transmission to susceptible cells without DCs infection (trans infection) (By similarity).  
  
Homotrimer; each monomer consists of a GP1 and a GP2 subunit linked by disulfide bonds. The resulting peplomers (GP1,2) protrude from the virus surface as spikes. GP1,2 interacts with human CD209 and CLEC4M (collectively referred to as DC-SIGN(R)). Asialoglycoprotein receptor (ASGP-R) may be a liver-specific receptor for GP1,2. Members of the Tyro3 receptor tyrosine kinase family may be cell entry factors interacting with GP1,2 (By similarity).  
  
**Gene Ontology Information:**

Molecular Function  
  
N/A

Location

- host cell plasma membrane
- integral component of membrane
- viral envelope
- virion membrane

Biological process

- fusion of virus membrane with host endosome membrane
- virion attachment to host cell

---

51

- **Protein name:** Epstein-Barr nuclear antigen 1
- **Organism:** Epstein-Barr virus (strain B95-8)
- **Uniprot Accession Number:** P03211
- **Protein sequence length:** 641 aa
- **1D identity (%):** 0.0
- **1D identity (%) [Gaps excluded]:** 0.0
- **1D identity - Alignment Gaps:** 1914
- **Common reported functions (%):** 0.0
- **Common reported locations (%):** 0.0
- **Common reported processes (%):** 0.0

- **PDB ID:** 5WMF
- **Chain:** C
- **Crystallized protein length:** 138 aa
- **Resolution:** 1.9 Å
- **Alinged residues range:** 599-602, 599-603, 516-519, 498-506, 595-599, 518-522, 514-519
- **Aligned to segment part (indices):** 3, 5, 6, 1, 4, 0, 2
- **Alinged residues range of reference:** 50-53, 204-208, 295-298, 426-436, 595-599, 761-765, 1005-1010
- **b-phipsi:** 0.031223
- **w-rdist:** 1.303453
- **t-alpha:** 0.40625
- **Chemical similarity (Tanimoto Index) (%):** 83.51
- **1D identity (%) [PDB]:** 0.0
- **1D identity (%) [Gaps excluded][PDB]:** 0.0
- **1D identity - Alignment Gaps [PDB]:** 1121
- **2D identity (%) [PDB]:** 8.35
- **2D identity (%) [Gaps excluded][PDB]:** 94.51
- **2D identity - Alignment Gaps [PDB]:** 939
- **3D similarity (TM-Score) (%) [PDB]:** 6.61

- **Gene name:** EBNA1
- **RefSeq ID:** NC\_007605
- **Genomic sequence length:** 171823
- **5-UTR|CDS|3-UTR identity (%):** N/A | N/A | N/A
- **5-UTR|CDS|3-UTR identity (%) [Gaps excluded]:** N/A | N/A | N/A
- **5-UTR|CDS|3-UTR identity [Alignment Gaps]:** N/A | N/A | N/A

**Uniprot Description:**  
  
Plays an essential role in replication and partitioning of viral genomic DNA during latent viral infection. During this phase, the circular double-stranded viral DNA undergoes replication once per cell cycle and is efficiently partitioned to the daughter cells. EBNA1 activates the initiation of viral DNA replication through binding to specific sites in the viral latent origin of replication, oriP. Additionally, it governs the segregation of viral episomes by mediating their attachment to host cell metaphase chromosomes. Also activates the transcription of several viral latency genes. Finally, it can counteract the stabilization of host p53/TP53 by host USP7, thereby decreasing apoptosis and increasing host cell survival.  
  
Interacts with human USP7. Interacts with human EBP2; this interaction is important for the stable segregation of EBV episomes during cell division but not for the replication of the episomes. Interacts with BGLF4; this interaction facilitates the switch from latent to lytic DNA replication by down-regulating EBNA1 replication function. Interacts with human PAX5; this interaction promotes EBNA1-dependent transcription.  
  
**Gene Ontology Information:**

Molecular Function

- DNA binding
- DNA-binding transcription factor activity

Location

- host cell nucleus

Biological process

- positive regulation of transcription, DNA-templated
- regulation of DNA replication
- suppression by virus of host adaptive immune response
- suppression by virus of host antigen processing and presentation
- suppression by virus of host NF-kappaB transcription factor activity
- viral latency

---

52

- **Protein name:** Capsid protein
- **Organism:** Tobacco mosaic virus (strain vulgare)
- **Uniprot Accession Number:** P69687
- **Protein sequence length:** 159 aa
- **1D identity (%):** 2.57
- **1D identity (%) [Gaps excluded]:** 31.78
- **1D identity - Alignment Gaps:** 1218
- **Common reported functions (%):** 0.0
- **Common reported locations (%):** 0.0
- **Common reported processes (%):** 0.0

- **PDB ID:** 4GQH
- **Chain:** K
- **Crystallized protein length:** 143 aa
- **Resolution:** 3.06 Å
- **Alinged residues range:** 2-4, 106-108, 68-70, 68-70, 118-121, 76-80
- **Aligned to segment part (indices):** 5, 6, 1, 4, 0, 2
- **Alinged residues range of reference:** 208-210, 294-296, 394-396, 586-588, 769-772, 959-963
- **b-phipsi:** 0.133398
- **w-rdist:** 1.08669
- **t-alpha:** 0.521352
- **Chemical similarity (Tanimoto Index) (%):** 81.73
- **1D identity (%) [PDB]:** 0.09
- **1D identity (%) [Gaps excluded][PDB]:** 100.0
- **1D identity - Alignment Gaps [PDB]:** 1123
- **2D identity (%) [PDB]:** 9.86
- **2D identity (%) [Gaps excluded][PDB]:** 90.09
- **2D identity - Alignment Gaps [PDB]:** 903
- **3D similarity (TM-Score) (%) [PDB]:** 8.48

- **Gene name:** CP
- **RefSeq ID:** NC\_001367
- **Genomic sequence length:** 6395
- **5-UTR|CDS|3-UTR identity (%):** N/A | 8.63 | N/A
- **5-UTR|CDS|3-UTR identity (%) [Gaps excluded]:** N/A | 79.76 | N/A
- **5-UTR|CDS|3-UTR identity [Alignment Gaps]:** N/A | 3462 | N/A

**Uniprot Description:**  
  
Capsid protein self-assembles to form rod-shaped virions about 18 nm in diameter with a central canal enclosing the viral genomic RNA.  
  
**Gene Ontology Information:**

Molecular Function

- structural molecule activity

Location

- helical viral capsid

Biological process  
  
N/A

---

53

- **Protein name:** Ephrin type-A receptor 2
- **Organism:** Homo sapiens
- **Uniprot Accession Number:** P29317
- **Protein sequence length:** 976 aa
- **1D identity (%):** 11.73
- **1D identity (%) [Gaps excluded]:** 29.1
- **1D identity - Alignment Gaps:** 957
- **Common reported functions (%):** 0.0
- **Common reported locations (%):** 0.0
- **Common reported processes (%):** 0.0

- **PDB ID:** 7CZF
- **Chain:** A
- **Crystallized protein length:** 170 aa
- **Resolution:** 3.2 Å
- **Alinged residues range:** 122-125, 119-126, 63-68, 123-126, 199-206
- **Aligned to segment part (indices):** 3, 5, 6, 1, 4
- **Alinged residues range of reference:** 50-53, 201-208, 311-316, 396-399, 528-535
- **b-phipsi:** 0.027951
- **w-rdist:** 1.115159
- **t-alpha:** 0.770186
- **Chemical similarity (Tanimoto Index) (%):** N/A
- **1D identity (%) [PDB]:** 0.0
- **1D identity (%) [Gaps excluded][PDB]:** 0.0
- **1D identity - Alignment Gaps [PDB]:** 1154
- **2D identity (%) [PDB]:** 14.1
- **2D identity (%) [Gaps excluded][PDB]:** 91.56
- **2D identity - Alignment Gaps [PDB]:** 846
- **3D similarity (TM-Score) (%) [PDB]:** 9.51

- **Gene name:** EPHA2
- **RefSeq ID:** NM\_004431
- **Transcript sequence length:** 3946
- **5-UTR|CDS|3-UTR identity (%):** 29.59 | 36.69 | 15.59
- **5-UTR|CDS|3-UTR identity (%) [Gaps excluded]:** 80.56 | 75.58 | 75.26
- **5-UTR|CDS|3-UTR identity [Alignment Gaps]:** 186 | 2339 | 727

**Uniprot Description:**  
  
Receptor tyrosine kinase which binds promiscuously membrane-bound ephrin-A family ligands residing on adjacent cells, leading to contact-dependent bidirectional signaling into neighboring cells. The signaling pathway downstream of the receptor is referred to as forward signaling while the signaling pathway downstream of the ephrin ligand is referred to as reverse signaling. Activated by the ligand ephrin-A1/EFNA1 regulates migration, integrin-mediated adhesion, proliferation and differentiation of cells. Regulates cell adhesion and differentiation through DSG1/desmoglein-1 and inhibition of the ERK1/ERK2 (MAPK3/MAPK1, respectively) signaling pathway. May also participate in UV radiation-induced apoptosis and have a ligand-independent stimulatory effect on chemotactic cell migration. During development, may function in distinctive aspects of pattern formation and subsequently in development of several fetal tissues. Involved for instance in angiogenesis, in early hindbrain development and epithelial proliferation and branching morphogenesis during mammary gland development. Engaged by the ligand ephrin-A5/EFNA5 may regulate lens fiber cells shape and interactions and be important for lens transparency development and maintenance. With ephrin-A2/EFNA2 may play a role in bone remodeling through regulation of osteoclastogenesis and osteoblastogenesis.  
  
Homodimer. Interacts with SLA. Interacts (phosphorylated form) with VAV2, VAV3 and PI3-kinase p85 subunit (PIK3R1, PIK3R2 or PIK3R3); critical for the EFNA1-induced activation of RAC1 which stimulates cell migration (By similarity). Interacts with INPPL1; regulates activated EPHA2 endocytosis and degradation. Interacts (inactivated form) with PTK2/FAK1 and interacts (EFNA1 ligand-activated form) with PTPN11; regulates integrin-mediated adhesion. Interacts with ARHGEF16, DOCK4 and ELMO2; mediates ligand-independent activation of RAC1 which stimulates cell migration. Interacts with CLDN4; phosphorylates CLDN4 and may regulate tight junctions. Interacts with ACP1. Interacts (via SAM domain) with ANKS1A (via SAM domain). Interacts with CEMIP. Interacts with NCK1; may regulate EPHA2 activity in cell migration and adhesion.  
  
**Gene Ontology Information:**

Molecular Function

- ATP binding
- cadherin binding
- growth factor binding
- transmembrane receptor protein tyrosine kinase activity
- transmembrane-ephrin receptor activity
- virus receptor activity

Location

- cell surface
- focal adhesion
- integral component of plasma membrane
- lamellipodium
- lamellipodium membrane
- leading edge membrane
- neuron projection
- plasma membrane
- receptor complex
- ruffle membrane
- tight junction

Biological process

- activation of GTPase activity
- axial mesoderm formation
- axon guidance
- blood vessel endothelial cell proliferation involved in sprouting angiogenesis
- bone remodeling
- branching involved in mammary gland duct morphogenesis
- cAMP metabolic process
- cell adhesion
- cell chemotaxis
- cell migration
- cell motility
- defense response to Gram-positive bacterium
- ephrin receptor signaling pathway
- inflammatory response
- intrinsic apoptotic signaling pathway in response to DNA damage
- keratinocyte differentiation
- lens fiber cell morphogenesis
- mammary gland epithelial cell proliferation
- multicellular organism development
- negative regulation of angiogenesis
- negative regulation of chemokine production
- negative regulation of lymphangiogenesis
- negative regulation of protein kinase B signaling
- neural tube development
- notochord cell development
- notochord formation
- osteoblast differentiation
- osteoclast differentiation
- pericyte cell differentiation
- positive regulation of bicellular tight junction assembly
- positive regulation of kinase activity
- positive regulation of protein localization to plasma membrane
- post-anal tail morphogenesis
- protein kinase B signaling
- protein localization to plasma membrane
- regulation of angiogenesis
- regulation of blood vessel endothelial cell migration
- regulation of cell adhesion mediated by integrin
- regulation of ERK1 and ERK2 cascade
- regulation of lamellipodium assembly
- response to growth factor
- skeletal system development
- transmembrane receptor protein tyrosine kinase signaling pathway
- vasculogenesis

---

54

- **Protein name:** Genome polyprotein
- **Organism:** Human rhinovirus 14
- **Uniprot Accession Number:** P03303
- **Protein sequence length:** 2179 aa
- **1D identity (%):** 11.46
- **1D identity (%) [Gaps excluded]:** 28.13
- **1D identity - Alignment Gaps:** 1454
- **Common reported functions (%):** 0.0
- **Common reported locations (%):** 0.0
- **Common reported processes (%):** 10.0

- **PDB ID:** 2HWC
- **Chain:** 1
- **Crystallized protein length:** 273 aa
- **Resolution:** 3.0 Å
- **Alinged residues range:** 204-207, 100-105, 126-129, 125-128, 126-131, 68-71, 114-116
- **Aligned to segment part (indices):** 3, 5, 6, 1, 4, 0, 2
- **Alinged residues range of reference:** 38-41, 206-211, 313-316, 448-451, 547-552, 764-767, 1010-1012
- **b-phipsi:** 0.004193
- **w-rdist:** 1.213058
- **t-alpha:** 1.002342
- **Chemical similarity (Tanimoto Index) (%):** N/A
- **1D identity (%) [PDB]:** 0.08
- **1D identity (%) [Gaps excluded][PDB]:** 50.0
- **1D identity - Alignment Gaps [PDB]:** 1252
- **2D identity (%) [PDB]:** 19.01
- **2D identity (%) [Gaps excluded][PDB]:** 87.11
- **2D identity - Alignment Gaps [PDB]:** 806
- **3D similarity (TM-Score) (%) [PDB]:** 10.58

- **Gene name:** N/A
- **RefSeq ID:** NC\_001490
- **Genomic sequence length:** 7212
- **5-UTR|CDS|3-UTR identity (%):** N/A | 34.41 | N/A
- **5-UTR|CDS|3-UTR identity (%) [Gaps excluded]:** N/A | 78.71 | N/A
- **5-UTR|CDS|3-UTR identity [Alignment Gaps]:** N/A | 4058 | N/A

**Uniprot Description:**  
  
Capsid protein VP1
Forms an icosahedral capsid of pseudo T=3 symmetry with capsid proteins VP2 and VP3. The capsid is 300 Angstroms in diameter, composed of 60 copies of each capsid protein and enclosing the viral positive strand RNA genome (By similarity). Capsid protein VP1 mainly forms the vertices of the capsid. Capsid protein VP1 interacts with host ICAM1 to provide virion attachment to target host cells (PubMed:10562537). This attachment induces virion internalization (By similarity). Tyrosine kinases are probably involved in the entry process. After binding to its receptor, the capsid undergoes conformational changes (By similarity). Capsid protein VP1 N-terminus (that contains an amphipathic alpha-helix) and capsid protein VP4 are externalized (Probable). Together, they shape a pore in the host membrane through which viral genome is translocated to host cell cytoplasm (PubMed:28696310). After genome has been released, the channel shrinks.  
  
Capsid protein VP0
Interacts with capsid protein VP1 and capsid protein VP3 to form heterotrimeric protomers.  
  
**Gene Ontology Information:**

Molecular Function

- ATP binding
- cysteine-type endopeptidase activity
- ion channel activity
- metal ion binding
- nucleoside-triphosphatase activity
- RNA binding
- RNA helicase activity
- RNA-directed 5'-3' RNA polymerase activity
- structural molecule activity

Location

- host cell cytoplasmic vesicle membrane
- host cell nucleus
- integral to membrane of host cell
- membrane
- T=pseudo3 icosahedral viral capsid

Biological process

- DNA replication
- endocytosis involved in viral entry into host cell
- induction by virus of host autophagy
- lysis of host organelle involved in viral entry into host cell
- pore formation by virus in membrane of host cell
- pore-mediated entry of viral genome into host cell
- positive stranded viral RNA replication
- protein complex oligomerization
- RNA-protein covalent cross-linking
- suppression by virus of host gene expression
- suppression by virus of host mRNA export from nucleus
- suppression by virus of host RIG-I activity
- suppression by virus of host translation initiation factor activity
- transcription, DNA-templated
- viral RNA genome replication
- virion attachment to host cell

---

55

- **Protein name:** DNA-directed RNA polymerase II subunit RPB11-a
- **Organism:** Sus scrofa
- **Uniprot Accession Number:** F1RKE4
- **Protein sequence length:** 117 aa
- **1D identity (%):** 1.55
- **1D identity (%) [Gaps excluded]:** 20.62
- **1D identity - Alignment Gaps:** 1196
- **Common reported functions (%):** 0.0
- **Common reported locations (%):** 0.0
- **Common reported processes (%):** 0.0

- **PDB ID:** 6GML
- **Chain:** K
- **Crystallized protein length:** 115 aa
- **Resolution:** 3.2 Å
- **Alinged residues range:** 60-62, 19-23, 41-44, 70-72, 78-82, 96-110, 109-112
- **Aligned to segment part (indices):** 3, 5, 6, 1, 4, 0, 2
- **Alinged residues range of reference:** 50-52, 203-207, 295-298, 432-434, 601-605, 761-773, 952-955
- **b-phipsi:** 0.032517
- **w-rdist:** 1.053954
- **t-alpha:** 0.826923
- **Chemical similarity (Tanimoto Index) (%):** N/A
- **1D identity (%) [PDB]:** 0.0
- **1D identity (%) [Gaps excluded][PDB]:** 0.0
- **1D identity - Alignment Gaps [PDB]:** 1098
- **2D identity (%) [PDB]:** 7.92
- **2D identity (%) [Gaps excluded][PDB]:** 90.91
- **2D identity - Alignment Gaps [PDB]:** 922
- **3D similarity (TM-Score) (%) [PDB]:** 23.68

- **Gene name:** POLR2J
- **RefSeq ID:** N/A
- **Sequence length:** N/A
- **5-UTR|CDS|3-UTR identity (%):** N/A | N/A | N/A
- **5-UTR|CDS|3-UTR identity (%) [Gaps excluded]:** N/A | N/A | N/A
- **5-UTR|CDS|3-UTR identity [Alignment Gaps]:** N/A | N/A | N/A

**Uniprot Description:**  
  
DNA-dependent RNA polymerase catalyzes the transcription of DNA into RNA using the four ribonucleoside triphosphates as substrates. Component of RNA polymerase II which synthesizes mRNA precursors and many functional non-coding RNAs. Pol II is the central component of the basal RNA polymerase II transcription machinery. It is composed of mobile elements that move relative to each other. RPB11 is part of the core element with the central large cleft.  
  
**Gene Ontology Information:**

Molecular Function  
  
N/A

Location  
  
N/A

Biological process  
  
N/A

---

56

- **Protein name:** Tumor necrosis factor
- **Organism:** Homo sapiens
- **Uniprot Accession Number:** P01375
- **Protein sequence length:** 233 aa
- **1D identity (%):** 3.57
- **1D identity (%) [Gaps excluded]:** 24.61
- **1D identity - Alignment Gaps:** 1124
- **Common reported functions (%):** 50.0
- **Common reported locations (%):** 0.0
- **Common reported processes (%):** 0.0

- **PDB ID:** 3IT8
- **Chain:** B
- **Crystallized protein length:** 152 aa
- **Resolution:** 2.8 Å
- **Alinged residues range:** 57-61, 58-61, 117-124, 116-121, 104-107
- **Aligned to segment part (indices):** 3, 5, 6, 1, 4
- **Alinged residues range of reference:** 48-52, 203-206, 311-318, 448-453, 529-532
- **b-phipsi:** 0.063509
- **w-rdist:** 1.098041
- **t-alpha:** 0.634799
- **Chemical similarity (Tanimoto Index) (%):** 84.15
- **1D identity (%) [PDB]:** 0.09
- **1D identity (%) [Gaps excluded][PDB]:** 50.0
- **1D identity - Alignment Gaps [PDB]:** 1131
- **2D identity (%) [PDB]:** 11.3
- **2D identity (%) [Gaps excluded][PDB]:** 90.48
- **2D identity - Alignment Gaps [PDB]:** 883
- **3D similarity (TM-Score) (%) [PDB]:** 7.87

- **Gene name:** TNF
- **RefSeq ID:** NM\_000594
- **Transcript sequence length:** 1678
- **5-UTR|CDS|3-UTR identity (%):** 22.82 | 11.98 | 19.98
- **5-UTR|CDS|3-UTR identity (%) [Gaps excluded]:** 69.72 | 78.33 | 84.26
- **5-UTR|CDS|3-UTR identity [Alignment Gaps]:** 224 | 3324 | 634

**Uniprot Description:**  
  
Cytokine that binds to TNFRSF1A/TNFR1 and TNFRSF1B/TNFBR. It is mainly secreted by macrophages and can induce cell death of certain tumor cell lines. It is potent pyrogen causing fever by direct action or by stimulation of interleukin-1 secretion and is implicated in the induction of cachexia, Under certain conditions it can stimulate cell proliferation and induce cell differentiation. Impairs regulatory T-cells (Treg) function in individuals with rheumatoid arthritis via FOXP3 dephosphorylation. Upregulates the expression of protein phosphatase 1 (PP1), which dephosphorylates the key 'Ser-418' residue of FOXP3, thereby inactivating FOXP3 and rendering Treg cells functionally defective (PubMed:23396208). Key mediator of cell death in the anticancer action of BCG-stimulated neutrophils in combination with DIABLO/SMAC mimetic in the RT4v6 bladder cancer cell line (PubMed:22517918, PubMed:16829952, PubMed:23396208). Induces insulin resistance in adipocytes via inhibition of insulin-induced IRS1 tyrosine phosphorylation and insulin-induced glucose uptake. Induces GKAP42 protein degradation in adipocytes which is partially responsible for TNF-induced insulin resistance (By similarity). Plays a role in angiogenesis by inducing VEGF production synergistically with IL1B and IL6 (PubMed:12794819).  
  
Homotrimer. Interacts with SPPL2B.  
  
**Gene Ontology Information:**

Molecular Function

- cytokine activity
- identical protein binding
- protease binding
- transcription regulatory region sequence-specific DNA binding
- tumor necrosis factor receptor binding

Location

- cell surface
- external side of plasma membrane
- extracellular region
- extracellular space
- integral component of plasma membrane
- membrane
- membrane raft
- phagocytic cup
- plasma membrane
- protein-containing complex
- recycling endosome

Biological process

- activation of cysteine-type endopeptidase activity involved in apoptotic process
- activation of MAPK activity
- activation of MAPKKK activity
- astrocyte activation
- cellular response to amino acid stimulus
- cellular response to nicotine
- cellular response to organic cyclic compound
- chronic inflammatory response to antigenic stimulus
- cognition
- cortical actin cytoskeleton organization
- cytokine-mediated signaling pathway
- death-inducing signaling complex assembly
- defense response to Gram-positive bacterium
- embryonic digestive tract development
- endothelial cell apoptotic process
- epithelial cell proliferation involved in salivary gland morphogenesis
- extracellular matrix organization
- extrinsic apoptotic signaling pathway
- extrinsic apoptotic signaling pathway via death domain receptors
- glucose metabolic process
- humoral immune response
- I-kappaB kinase/NF-kappaB signaling
- inflammatory response
- intrinsic apoptotic signaling pathway in response to DNA damage
- JNK cascade
- leukocyte tethering or rolling
- lipopolysaccharide-mediated signaling pathway
- MAPK cascade
- microglial cell activation
- necroptotic signaling pathway
- negative regulation of alkaline phosphatase activity
- negative regulation of amyloid-beta clearance
- negative regulation of apoptotic signaling pathway
- negative regulation of bicellular tight junction assembly
- negative regulation of blood vessel endothelial cell migration
- negative regulation of branching involved in lung morphogenesis
- negative regulation of cysteine-type endopeptidase activity involved in apoptotic process
- negative regulation of cytokine production involved in immune response
- negative regulation of endothelial cell proliferation
- negative regulation of extrinsic apoptotic signaling pathway in absence of ligand
- negative regulation of fat cell differentiation
- negative regulation of gene expression
- negative regulation of glucose import
- negative regulation of interleukin-6 production
- negative regulation of lipid catabolic process
- negative regulation of lipid storage
- negative regulation of mitotic cell cycle
- negative regulation of myoblast differentiation
- negative regulation of myosin-light-chain-phosphatase activity
- negative regulation of neurogenesis
- negative regulation of osteoblast differentiation
- negative regulation of production of miRNAs involved in gene silencing by miRNA
- negative regulation of protein-containing complex disassembly
- negative regulation of signaling receptor activity
- negative regulation of transcription by RNA polymerase II
- negative regulation of transcription, DNA-templated
- negative regulation of vascular wound healing
- negative regulation of viral genome replication
- osteoclast differentiation
- positive regulation of amyloid-beta formation
- positive regulation of apoptotic process
- positive regulation of blood microparticle formation
- positive regulation of calcidiol 1-monooxygenase activity
- positive regulation of calcineurin-NFAT signaling cascade
- positive regulation of cell adhesion
- positive regulation of ceramide biosynthetic process
- positive regulation of chemokine (C-X-C motif) ligand 2 production
- positive regulation of chemokine production
- positive regulation of chronic inflammatory response to antigenic stimulus
- positive regulation of cysteine-type endopeptidase activity involved in apoptotic process
- positive regulation of cytokine production
- positive regulation of cytokine production involved in inflammatory response
- positive regulation of DNA-binding transcription factor activity
- positive regulation of ERK1 and ERK2 cascade
- positive regulation of extrinsic apoptotic signaling pathway
- positive regulation of fever generation
- positive regulation of fractalkine production
- positive regulation of gene expression
- positive regulation of glial cell proliferation
- positive regulation of hair follicle development
- positive regulation of heterotypic cell-cell adhesion
- positive regulation of humoral immune response mediated by circulating immunoglobulin
- positive regulation of I-kappaB kinase/NF-kappaB signaling
- positive regulation of I-kappaB phosphorylation
- positive regulation of inflammatory response
- positive regulation of interferon-gamma production
- positive regulation of interleukin-1 beta production
- positive regulation of interleukin-33 production
- positive regulation of interleukin-6 production
- positive regulation of interleukin-8 production
- positive regulation of JNK cascade
- positive regulation of JUN kinase activity
- positive regulation of leukocyte adhesion to arterial endothelial cell
- positive regulation of leukocyte adhesion to vascular endothelial cell
- positive regulation of MAP kinase activity
- positive regulation of membrane protein ectodomain proteolysis
- positive regulation of mononuclear cell migration
- positive regulation of neuroinflammatory response
- positive regulation of neuron apoptotic process
- positive regulation of NF-kappaB transcription factor activity
- positive regulation of NIK/NF-kappaB signaling
- positive regulation of nitric oxide biosynthetic process
- positive regulation of nitric-oxide synthase activity
- positive regulation of nitrogen compound metabolic process
- positive regulation of osteoclast differentiation
- positive regulation of peptidyl-serine phosphorylation
- positive regulation of phagocytosis
- positive regulation of phosphatidylinositol 3-kinase signaling
- positive regulation of podosome assembly
- positive regulation of pri-miRNA transcription by RNA polymerase II
- positive regulation of programmed cell death
- positive regulation of protein catabolic process
- positive regulation of protein kinase activity
- positive regulation of protein kinase B signaling
- positive regulation of protein localization to cell surface
- positive regulation of protein localization to plasma membrane
- positive regulation of protein phosphorylation
- positive regulation of protein transport
- positive regulation of protein-containing complex assembly
- positive regulation of protein-containing complex disassembly
- positive regulation of receptor signaling pathway via JAK-STAT
- positive regulation of smooth muscle cell proliferation
- positive regulation of superoxide dismutase activity
- positive regulation of synaptic transmission
- positive regulation of synoviocyte proliferation
- positive regulation of transcription by RNA polymerase II
- positive regulation of transcription, DNA-templated
- positive regulation of translational initiation by iron
- positive regulation of tyrosine phosphorylation of STAT protein
- positive regulation of vascular associated smooth muscle cell proliferation
- positive regulation of vitamin D biosynthetic process
- protein kinase B signaling
- protein localization to plasma membrane
- regulation of branching involved in salivary gland morphogenesis
- regulation of endothelial cell apoptotic process
- regulation of establishment of endothelial barrier
- regulation of fat cell differentiation
- regulation of I-kappaB kinase/NF-kappaB signaling
- regulation of immunoglobulin production
- regulation of insulin secretion
- regulation of reactive oxygen species metabolic process
- regulation of synapse organization
- regulation of synaptic transmission, glutamatergic
- regulation of transcription by RNA polymerase II
- regulation of tumor necrosis factor-mediated signaling pathway
- response to glucocorticoid
- response to salt stress
- response to virus
- sequestering of triglyceride
- tumor necrosis factor-mediated signaling pathway
- vascular endothelial growth factor production

---

57

- **Protein name:** Host-nuclease inhibitor protein gam
- **Organism:** Escherichia phage lambda
- **Uniprot Accession Number:** P03702
- **Protein sequence length:** 138 aa
- **1D identity (%):** 3.01
- **1D identity (%) [Gaps excluded]:** 33.33
- **1D identity - Alignment Gaps:** 1177
- **Common reported functions (%):** 0.0
- **Common reported locations (%):** 0.0
- **Common reported processes (%):** 0.0

- **PDB ID:** 2UUZ
- **Chain:** B
- **Crystallized protein length:** 86 aa
- **Resolution:** 2.3 Å
- **Alinged residues range:** 58-60, 77-80, 126-130, 129-135
- **Aligned to segment part (indices):** 6, 1, 0, 2
- **Alinged residues range of reference:** 298-300, 420-423, 769-773, 988-994
- **b-phipsi:** 0.414057
- **w-rdist:** 1.044803
- **t-alpha:** 0.647399
- **Chemical similarity (Tanimoto Index) (%):** 81.6
- **1D identity (%) [PDB]:** 0.09
- **1D identity (%) [Gaps excluded][PDB]:** 50.0
- **1D identity - Alignment Gaps [PDB]:** 1065
- **2D identity (%) [PDB]:** 6.81
- **2D identity (%) [Gaps excluded][PDB]:** 97.14
- **2D identity - Alignment Gaps [PDB]:** 929
- **3D similarity (TM-Score) (%) [PDB]:** 5.35

- **Gene name:** gam
- **RefSeq ID:** NC\_001416
- **Genomic sequence length:** 48502
- **5-UTR|CDS|3-UTR identity (%):** N/A | 7.78 | N/A
- **5-UTR|CDS|3-UTR identity (%) [Gaps excluded]:** N/A | 78.33 | N/A
- **5-UTR|CDS|3-UTR identity [Alignment Gaps]:** N/A | 3473 | N/A

**Uniprot Description:**  
  
Binds to host RecBCD nuclease and inhibits it thereby protecting the viral DNA against recBCD mediated degradation.  
  
Interacts with host RecBCD; this interaction prevents RecBCD from binding to DNA.  
  
**Gene Ontology Information:**

Molecular Function

- deoxyribonuclease inhibitor activity

Location  
  
N/A

Biological process

- DNA end degradation evasion by virus

---

58

- **Protein name:** Thymidine kinase
- **Organism:** Human herpesvirus 1 (strain 17)
- **Uniprot Accession Number:** P0DTH5
- **Protein sequence length:** 376 aa
- **1D identity (%):** 5.57
- **1D identity (%) [Gaps excluded]:** 26.76
- **1D identity - Alignment Gaps:** 1081
- **Common reported functions (%):** 0.0
- **Common reported locations (%):** 0.0
- **Common reported processes (%):** 0.0

- **PDB ID:** 1KI2
- **Chain:** A
- **Crystallized protein length:** 308 aa
- **Resolution:** 2.2 Å
- **Alinged residues range:** 144-146, 143-146, 144-146, 200-206, 78-82, 359-362, 211-217
- **Aligned to segment part (indices):** 3, 5, 6, 1, 4, 0, 2
- **Alinged residues range of reference:** 50-52, 203-206, 315-317, 430-436, 597-601, 768-771, 990-996
- **b-phipsi:** 0.104967
- **w-rdist:** 1.007801
- **t-alpha:** 0.8
- **Chemical similarity (Tanimoto Index) (%):** 83.4
- **1D identity (%) [PDB]:** 0.0
- **1D identity (%) [Gaps excluded][PDB]:** 0.0
- **1D identity - Alignment Gaps [PDB]:** 1291
- **2D identity (%) [PDB]:** 17.95
- **2D identity (%) [Gaps excluded][PDB]:** 91.51
- **2D identity - Alignment Gaps [PDB]:** 869
- **3D similarity (TM-Score) (%) [PDB]:** 9.86

- **Gene name:** TK
- **RefSeq ID:** NC\_001806
- **Genomic sequence length:** 152222
- **5-UTR|CDS|3-UTR identity (%):** N/A | 17.71 | N/A
- **5-UTR|CDS|3-UTR identity (%) [Gaps excluded]:** N/A | 78.14 | N/A
- **5-UTR|CDS|3-UTR identity [Alignment Gaps]:** N/A | 3123 | N/A

**Uniprot Description:**  
  
Catalyzes the transfer of the gamma-phospho group of ATP to thymidine to generate dTMP in the salvage pathway of pyrimidine synthesis. The dTMP serves as a substrate for DNA polymerase during viral DNA replication. Allows the virus to be reactivated and to grow in non-proliferative cells lacking a high concentration of phosphorylated nucleic acid precursors.  
  
Homodimer.  
  
**Gene Ontology Information:**

Molecular Function

- ATP binding
- thymidine kinase activity

Location  
  
N/A

Biological process

- DNA biosynthetic process
- TMP biosynthetic process

---

59

- **Protein name:** Distal tail protein
- **Organism:** Bacillus phage SPP1
- **Uniprot Accession Number:** O48459
- **Protein sequence length:** 253 aa
- **1D identity (%):** 4.52
- **1D identity (%) [Gaps excluded]:** 30.15
- **1D identity - Alignment Gaps:** 1128
- **Common reported functions (%):** 0.0
- **Common reported locations (%):** 0.0
- **Common reported processes (%):** 0.0

- **PDB ID:** 2X8K
- **Chain:** A
- **Crystallized protein length:** 242 aa
- **Resolution:** 2.95 Å
- **Alinged residues range:** 135-140, 138-144, 137-144, 243-250, 103-117, 75-79, 80-85
- **Aligned to segment part (indices):** 3, 5, 6, 1, 4, 0, 2
- **Alinged residues range of reference:** 48-53, 200-206, 311-317, 430-437, 545-556, 761-765, 1000-1005
- **b-phipsi:** 0.050294
- **w-rdist:** 1.062134
- **t-alpha:** 0.834764
- **Chemical similarity (Tanimoto Index) (%):** 83.32
- **1D identity (%) [PDB]:** 0.0
- **1D identity (%) [Gaps excluded][PDB]:** 0.0
- **1D identity - Alignment Gaps [PDB]:** 1226
- **2D identity (%) [PDB]:** 18.49
- **2D identity (%) [Gaps excluded][PDB]:** 89.95
- **2D identity - Alignment Gaps [PDB]:** 808
- **3D similarity (TM-Score) (%) [PDB]:** 9.31

- **Gene name:** N/A
- **RefSeq ID:** NC\_004166
- **Genomic sequence length:** 44010
- **5-UTR|CDS|3-UTR identity (%):** N/A | 12.98 | N/A
- **5-UTR|CDS|3-UTR identity (%) [Gaps excluded]:** N/A | 80.0 | N/A
- **5-UTR|CDS|3-UTR identity [Alignment Gaps]:** N/A | 3304 | N/A

**Uniprot Description:**  
  
Forms a 40 Angstroms wide channel at the distal tip of the tail. Remains associated to the tail after DNA ejection.  
  
Homohexamer.  
  
**Gene Ontology Information:**

Molecular Function  
  
N/A

Location

- virus tail

Biological process

- viral genome ejection through host cell envelope, long flexible tail mechanism

---

60

- **Protein name:** 40S ribosomal protein S11
- **Organism:** Oryctolagus cuniculus
- **Uniprot Accession Number:** G1TRM4
- **Protein sequence length:** 158 aa
- **1D identity (%):** 3.68
- **1D identity (%) [Gaps excluded]:** 30.72
- **1D identity - Alignment Gaps:** 1125
- **Common reported functions (%):** 0.0
- **Common reported locations (%):** 0.0
- **Common reported processes (%):** 0.0

- **PDB ID:** 6P4G
- **Chain:** M
- **Crystallized protein length:** 143 aa
- **Resolution:** 3.1 Å
- **Alinged residues range:** 142-146, 91-93, 127-130, 85-89, 142-146, 50-52, 48-50
- **Aligned to segment part (indices):** 3, 5, 6, 1, 4, 0, 2
- **Alinged residues range of reference:** 47-51, 224-226, 311-314, 399-403, 584-588, 771-773, 954-956
- **b-phipsi:** 0.015543
- **w-rdist:** 1.196964
- **t-alpha:** 0.921348
- **Chemical similarity (Tanimoto Index) (%):** 73.04
- **1D identity (%) [PDB]:** 0.09
- **1D identity (%) [Gaps excluded][PDB]:** 50.0
- **1D identity - Alignment Gaps [PDB]:** 1123
- **2D identity (%) [PDB]:** 11.59
- **2D identity (%) [Gaps excluded][PDB]:** 92.06
- **2D identity - Alignment Gaps [PDB]:** 875
- **3D similarity (TM-Score) (%) [PDB]:** 6.63

- **Gene name:** RPS11
- **RefSeq ID:** N/A
- **Sequence length:** N/A
- **5-UTR|CDS|3-UTR identity (%):** N/A | N/A | N/A
- **5-UTR|CDS|3-UTR identity (%) [Gaps excluded]:** N/A | N/A | N/A
- **5-UTR|CDS|3-UTR identity [Alignment Gaps]:** N/A | N/A | N/A

**Uniprot Description:**  
  
N/A  
  
**Gene Ontology Information:**

Molecular Function

- rRNA binding
- structural constituent of ribosome

Location

- ribosome

Biological process

- translation

---

61

- **Protein name:** Uncharacterized protein
- **Organism:** Enterobacteria phage YYZ-2008
- **Uniprot Accession Number:** B6DZC0
- **Protein sequence length:** 213 aa
- **1D identity (%):** 4.36
- **1D identity (%) [Gaps excluded]:** 27.72
- **1D identity - Alignment Gaps:** 1082
- **Common reported functions (%):** 0.0
- **Common reported locations (%):** 0.0
- **Common reported processes (%):** 0.0

- **PDB ID:** 5VGC
- **Chain:** A
- **Crystallized protein length:** 211 aa
- **Resolution:** 2.6 Å
- **Alinged residues range:** 162-164, 36-38, 110-113, 95-99, 45-55, 112-115
- **Aligned to segment part (indices):** 3, 5, 6, 1, 4, 0
- **Alinged residues range of reference:** 41-43, 201-203, 297-300, 366-370, 595-605, 761-764
- **b-phipsi:** 0.022493
- **w-rdist:** 1.132384
- **t-alpha:** 0.943182
- **Chemical similarity (Tanimoto Index) (%):** 74.63
- **1D identity (%) [PDB]:** 0.0
- **1D identity (%) [Gaps excluded][PDB]:** 0.0
- **1D identity - Alignment Gaps [PDB]:** 1194
- **2D identity (%) [PDB]:** 10.16
- **2D identity (%) [Gaps excluded][PDB]:** 82.44
- **2D identity - Alignment Gaps [PDB]:** 932
- **3D similarity (TM-Score) (%) [PDB]:** 9.04

- **Gene name:** N/A
- **RefSeq ID:** NC\_011356
- **Genomic sequence length:** 54896
- **5-UTR|CDS|3-UTR identity (%):** N/A | 11.74 | N/A
- **5-UTR|CDS|3-UTR identity (%) [Gaps excluded]:** N/A | 81.35 | N/A
- **5-UTR|CDS|3-UTR identity [Alignment Gaps]:** N/A | 3338 | N/A

**Uniprot Description:**  
  
N/A  
  
**Gene Ontology Information:**

Molecular Function

- ubiquitin-protein transferase activity

Location  
  
N/A

Biological process

- biological process involved in symbiotic interaction

---

62

- **Protein name:** KLLA0F09812p
- **Organism:** Kluyveromyces lactis (strain ATCC 8585 / CBS 2359 / DSM 70799 / NBRC 1267 / NRRL Y-1140 / WM37)
- **Uniprot Accession Number:** Q6CKL3
- **Protein sequence length:** 259 aa
- **1D identity (%):** 3.64
- **1D identity (%) [Gaps excluded]:** 22.64
- **1D identity - Alignment Gaps:** 1108
- **Common reported functions (%):** 0.0
- **Common reported locations (%):** 0.0
- **Common reported processes (%):** 0.0

- **PDB ID:** 5IT9
- **Chain:** C
- **Crystallized protein length:** 217 aa
- **Resolution:** 3.8 Å
- **Alinged residues range:** 89-93, 83-92, 67-70, 114-123, 109-117, 130-134, 189-194
- **Aligned to segment part (indices):** 3, 5, 6, 1, 4, 0, 2
- **Alinged residues range of reference:** 49-53, 195-207, 307-310, 393-400, 576-588, 738-742, 952-957
- **b-phipsi:** 0.012501
- **w-rdist:** 1.18988
- **t-alpha:** 1.080292
- **Chemical similarity (Tanimoto Index) (%):** N/A
- **1D identity (%) [PDB]:** 0.25
- **1D identity (%) [Gaps excluded][PDB]:** 75.0
- **1D identity - Alignment Gaps [PDB]:** 1192
- **2D identity (%) [PDB]:** 11.85
- **2D identity (%) [Gaps excluded][PDB]:** 91.97
- **2D identity - Alignment Gaps [PDB]:** 926
- **3D similarity (TM-Score) (%) [PDB]:** 8.81

- **Gene name:** KLLA0\_F09812g
- **RefSeq ID:** N/A
- **Sequence length:** N/A
- **5-UTR|CDS|3-UTR identity (%):** N/A | N/A | N/A
- **5-UTR|CDS|3-UTR identity (%) [Gaps excluded]:** N/A | N/A | N/A
- **5-UTR|CDS|3-UTR identity [Alignment Gaps]:** N/A | N/A | N/A

**Uniprot Description:**  
  
N/A  
  
**Gene Ontology Information:**

Molecular Function

- structural constituent of ribosome

Location

- cytosolic small ribosomal subunit

Biological process

- positive regulation of nuclear-transcribed mRNA catabolic process, deadenylation-dependent decay
- rRNA export from nucleus
- translation

---

63

- **Protein name:** dUTP diphosphatase
- **Organism:** Staphylococcus phage phi 11
- **Uniprot Accession Number:** Q8SDV3
- **Protein sequence length:** 169 aa
- **1D identity (%):** 3.71
- **1D identity (%) [Gaps excluded]:** 32.21
- **1D identity - Alignment Gaps:** 1144
- **Common reported functions (%):** 0.0
- **Common reported locations (%):** 0.0
- **Common reported processes (%):** 0.0

- **PDB ID:** 4WRK
- **Chain:** B
- **Crystallized protein length:** 155 aa
- **Resolution:** 2.9 Å
- **Alinged residues range:** 115-118, 103-105, 111-120, 65-70, 117-121, 65-67, 65-70
- **Aligned to segment part (indices):** 3, 5, 6, 1, 4, 0, 2
- **Alinged residues range of reference:** 49-52, 203-205, 282-289, 365-370, 573-577, 768-770, 1006-1011
- **b-phipsi:** 0.018888
- **w-rdist:** 1.088805
- **t-alpha:** 1.209302
- **Chemical similarity (Tanimoto Index) (%):** 82.54
- **1D identity (%) [PDB]:** 0.53
- **1D identity (%) [Gaps excluded][PDB]:** 66.67
- **1D identity - Alignment Gaps [PDB]:** 1121
- **2D identity (%) [PDB]:** 10.57
- **2D identity (%) [Gaps excluded][PDB]:** 92.31
- **2D identity - Alignment Gaps [PDB]:** 905
- **3D similarity (TM-Score) (%) [PDB]:** 8.38

- **Gene name:** N/A
- **RefSeq ID:** NC\_004615
- **Genomic sequence length:** 43604
- **5-UTR|CDS|3-UTR identity (%):** N/A | 9.07 | N/A
- **5-UTR|CDS|3-UTR identity (%) [Gaps excluded]:** N/A | 80.59 | N/A
- **5-UTR|CDS|3-UTR identity [Alignment Gaps]:** N/A | 3456 | N/A

**Uniprot Description:**  
  
N/A  
  
**Gene Ontology Information:**

Molecular Function

- dUTP diphosphatase activity
- magnesium ion binding

Location  
  
N/A

Biological process

- dUMP biosynthetic process
- dUTP catabolic process

---

64

- **Protein name:** mRNA export factor ICP27 homolog
- **Organism:** Saimiriine herpesvirus 2 (strain 11)
- **Uniprot Accession Number:** P13199
- **Protein sequence length:** 417 aa
- **1D identity (%):** 4.9
- **1D identity (%) [Gaps excluded]:** 32.58
- **1D identity - Alignment Gaps:** 1248
- **Common reported functions (%):** 0.0
- **Common reported locations (%):** 0.0
- **Common reported processes (%):** 0.0

- **PDB ID:** 6HAU
- **Chain:** B
- **Crystallized protein length:** 273 aa
- **Resolution:** 1.86 Å
- **Alinged residues range:** 403-405, 381-384, 387-390, 179-181, 395-401, 376-386
- **Aligned to segment part (indices):** 3, 6, 1, 4, 0, 2
- **Alinged residues range of reference:** 38-40, 295-298, 427-430, 605-607, 766-772, 1002-1012
- **b-phipsi:** 0.198457
- **w-rdist:** 3.204648
- **t-alpha:** 0.002345
- **Chemical similarity (Tanimoto Index) (%):** 82.48
- **1D identity (%) [PDB]:** 0.0
- **1D identity (%) [Gaps excluded][PDB]:** 0.0
- **1D identity - Alignment Gaps [PDB]:** 1256
- **2D identity (%) [PDB]:** 17.38
- **2D identity (%) [Gaps excluded][PDB]:** 90.15
- **2D identity - Alignment Gaps [PDB]:** 850
- **3D similarity (TM-Score) (%) [PDB]:** 9.72

- **Gene name:** EJRF1
- **RefSeq ID:** NC\_001350
- **Genomic sequence length:** 112930
- **5-UTR|CDS|3-UTR identity (%):** N/A | 21.96 | N/A
- **5-UTR|CDS|3-UTR identity (%) [Gaps excluded]:** N/A | 79.74 | N/A
- **5-UTR|CDS|3-UTR identity [Alignment Gaps]:** N/A | 2884 | N/A

**Uniprot Description:**  
  
Probably acts as a viral splicing factor that regulates viral RNA splicing. Functions as a multifunctional regulator of the expression of viral lytic genes (By similarity). Early protein that promotes the accumulation and nuclear export of viral intronless RNA transcripts by interacting with mRNAs and cellular export proteins.  
  
Homodimer. Homodimerization is required for transactivation (By similarity). Interacts with host ALYREF and with mouse ALYREF2. Associates in a complex with RNA, and host export factors NXF1/TAP and ALYREF or ALYREF2; these interactions allow nuclear export of viral transcripts.  
  
**Gene Ontology Information:**

Molecular Function

- metal ion binding
- RNA binding

Location

- host cell cytoplasm
- host cell nucleus

Biological process

- regulation of transcription, DNA-templated

---

65

- **Protein name:** Nuclear egress protein 2
- **Organism:** Human cytomegalovirus (strain Merlin)
- **Uniprot Accession Number:** Q6SW81
- **Protein sequence length:** 398 aa
- **1D identity (%):** 3.21
- **1D identity (%) [Gaps excluded]:** 27.12
- **1D identity - Alignment Gaps:** 1317
- **Common reported functions (%):** 0.0
- **Common reported locations (%):** 12.5
- **Common reported processes (%):** 0.0

- **PDB ID:** 5D5N
- **Chain:** A
- **Crystallized protein length:** 162 aa
- **Resolution:** 2.44 Å
- **Alinged residues range:** 111-113, 157-160, 133-136, 131-134, 161-167, 157-164
- **Aligned to segment part (indices):** 5, 6, 1, 4, 0, 2
- **Alinged residues range of reference:** 225-227, 297-300, 400-403, 573-576, 762-768, 1007-1014
- **b-phipsi:** 0.020299
- **w-rdist:** 1.077171
- **t-alpha:** 1.261905
- **Chemical similarity (Tanimoto Index) (%):** 82.62
- **1D identity (%) [PDB]:** 0.09
- **1D identity (%) [Gaps excluded][PDB]:** 100.0
- **1D identity - Alignment Gaps [PDB]:** 1144
- **2D identity (%) [PDB]:** 8.67
- **2D identity (%) [Gaps excluded][PDB]:** 93.81
- **2D identity - Alignment Gaps [PDB]:** 952
- **3D similarity (TM-Score) (%) [PDB]:** 7.25

- **Gene name:** NEC2
- **RefSeq ID:** NC\_006273
- **Genomic sequence length:** 235646
- **5-UTR|CDS|3-UTR identity (%):** N/A | 18.9 | N/A
- **5-UTR|CDS|3-UTR identity (%) [Gaps excluded]:** N/A | 74.12 | N/A
- **5-UTR|CDS|3-UTR identity [Alignment Gaps]:** N/A | 2979 | N/A

**Uniprot Description:**  
  
Plays an essential role in virion nuclear egress, the first step of virion release from infected cell. Within the host nucleus, NEC1 interacts with the newly formed capsid through the vertexes and directs it to the inner nuclear membrane by associating with NEC2. Induces the budding of the capsid at the inner nuclear membrane as well as its envelopment into the perinuclear space. There, the NEC1/NEC2 complex promotes the fusion of the enveloped capsid with the outer nuclear membrane and the subsequent release of the viral capsid into the cytoplasm where it will reach the secondary budding sites in the host Golgi or trans-Golgi network.  
  
Forms a heterohexameric complex with NEC1.  
  
**Gene Ontology Information:**

Molecular Function  
  
N/A

Location

- host cell nuclear inner membrane
- integral component of membrane
- viral tegument

Biological process

- viral budding from nuclear membrane
- viral life cycle
- viral transcription

---

66

- **Protein name:** Negative elongation factor C/D
- **Organism:** Homo sapiens
- **Uniprot Accession Number:** Q8IXH7
- **Protein sequence length:** 590 aa
- **1D identity (%):** 1.73
- **1D identity (%) [Gaps excluded]:** 42.47
- **1D identity - Alignment Gaps:** 1717
- **Common reported functions (%):** 0.0
- **Common reported locations (%):** 0.0
- **Common reported processes (%):** 0.0

- **PDB ID:** 6GML
- **Chain:** W
- **Crystallized protein length:** 538 aa
- **Resolution:** 3.2 Å
- **Alinged residues range:** 445-447, 413-416, 412-416, 348-351, 386-391, 426-435
- **Aligned to segment part (indices):** 3, 6, 1, 4, 0, 2
- **Alinged residues range of reference:** 39-41, 295-298, 366-370, 534-537, 768-773, 999-1008
- **b-phipsi:** 0.101203
- **w-rdist:** 0.937582
- **t-alpha:** 0.956522
- **Chemical similarity (Tanimoto Index) (%):** N/A
- **1D identity (%) [PDB]:** 0.0
- **1D identity (%) [Gaps excluded][PDB]:** 0.0
- **1D identity - Alignment Gaps [PDB]:** 1522
- **2D identity (%) [PDB]:** 17.97
- **2D identity (%) [Gaps excluded][PDB]:** 90.12
- **2D identity - Alignment Gaps [PDB]:** 1016
- **3D similarity (TM-Score) (%) [PDB]:** 23.68

- **Gene name:** NELFCD
- **RefSeq ID:** N/A
- **Sequence length:** N/A
- **5-UTR|CDS|3-UTR identity (%):** N/A | N/A | N/A
- **5-UTR|CDS|3-UTR identity (%) [Gaps excluded]:** N/A | N/A | N/A
- **5-UTR|CDS|3-UTR identity [Alignment Gaps]:** N/A | N/A | N/A

**Uniprot Description:**  
  
Essential component of the NELF complex, a complex that negatively regulates the elongation of transcription by RNA polymerase II (PubMed:12612062). The NELF complex, which acts via an association with the DSIF complex and causes transcriptional pausing, is counteracted by the P-TEFb kinase complex (PubMed:10199401).  
  
The NELF complex is composed of NELFA, NELFB, NELFCD (isoform NELF-C or isoform NELF-D) and NELFE; NELFA and NELFCD form a stable subcomplex that binds primarily through NELFCD to the N-terminus of NELFB (PubMed:27282391, PubMed:12612062). Binds RNA which may help to stabilize the NELF complex on nucleic acid (PubMed:27282391). In vitro, the NELFA:NELFCD subcomplex binds to ssDNA and ssRNA in a sequence- and structure-dependent manner (PubMed:27282391). Interacts with ARAF (PubMed:11952167). Interacts with PCF11 (PubMed:23884411). Interacts with KAT8 (By similarity).  
  
**Gene Ontology Information:**

Molecular Function

- RNA binding

Location

- membrane
- NELF complex
- nucleoplasm

Biological process

- negative regulation of transcription elongation from RNA polymerase II promoter
- positive regulation of viral transcription
- transcription by RNA polymerase II
- transcription elongation from RNA polymerase II promoter

---

67

- **Protein name:** Interferon stimulated gene 17
- **Organism:** Ovis aries
- **Uniprot Accession Number:** Q9GKP4
- **Protein sequence length:** 157 aa
- **1D identity (%):** 3.49
- **1D identity (%) [Gaps excluded]:** 32.14
- **1D identity - Alignment Gaps:** 1150
- **Common reported functions (%):** 0.0
- **Common reported locations (%):** 0.0
- **Common reported processes (%):** 0.0

- **PDB ID:** 6OAT
- **Chain:** D
- **Crystallized protein length:** 153 aa
- **Resolution:** 3.17 Å
- **Alinged residues range:** 95-97, 79-81, 93-97, 80-84, 86-91, 26-31, 29-35
- **Aligned to segment part (indices):** 3, 5, 6, 1, 4, 0, 2
- **Alinged residues range of reference:** 50-52, 219-221, 313-317, 429-433, 576-581, 761-766, 954-960
- **b-phipsi:** 0.004767
- **w-rdist:** 1.365781
- **t-alpha:** 1.016509
- **Chemical similarity (Tanimoto Index) (%):** N/A
- **1D identity (%) [PDB]:** 0.09
- **1D identity (%) [Gaps excluded][PDB]:** 50.0
- **1D identity - Alignment Gaps [PDB]:** 1132
- **2D identity (%) [PDB]:** 9.94
- **2D identity (%) [Gaps excluded][PDB]:** 84.17
- **2D identity - Alignment Gaps [PDB]:** 896
- **3D similarity (TM-Score) (%) [PDB]:** 8.44

- **Gene name:** ISG17
- **RefSeq ID:** N/A
- **Sequence length:** N/A
- **5-UTR|CDS|3-UTR identity (%):** N/A | N/A | N/A
- **5-UTR|CDS|3-UTR identity (%) [Gaps excluded]:** N/A | N/A | N/A
- **5-UTR|CDS|3-UTR identity [Alignment Gaps]:** N/A | N/A | N/A

**Uniprot Description:**  
  
N/A  
  
**Gene Ontology Information:**

Molecular Function  
  
N/A

Location  
  
N/A

Biological process  
  
N/A

---

68

- **Protein name:** Ubiquitin carboxyl-terminal hydrolase 7
- **Organism:** Homo sapiens
- **Uniprot Accession Number:** Q93009
- **Protein sequence length:** 1102 aa
- **1D identity (%):** 12.39
- **1D identity (%) [Gaps excluded]:** 26.28
- **1D identity - Alignment Gaps:** 853
- **Common reported functions (%):** 0.0
- **Common reported locations (%):** 0.0
- **Common reported processes (%):** 0.0

- **PDB ID:** 2XXN
- **Chain:** A
- **Crystallized protein length:** 143 aa
- **Resolution:** 1.6 Å
- **Alinged residues range:** 96-98, 190-193, 112-116, 188-195, 137-140, 173-175
- **Aligned to segment part (indices):** 3, 5, 6, 1, 4, 2
- **Alinged residues range of reference:** 50-52, 200-203, 309-313, 394-401, 584-587, 986-988
- **b-phipsi:** 0.041254
- **w-rdist:** 1.355133
- **t-alpha:** 0.631679
- **Chemical similarity (Tanimoto Index) (%):** 82.98
- **1D identity (%) [PDB]:** 0.09
- **1D identity (%) [Gaps excluded][PDB]:** 50.0
- **1D identity - Alignment Gaps [PDB]:** 1122
- **2D identity (%) [PDB]:** 10.42
- **2D identity (%) [Gaps excluded][PDB]:** 88.98
- **2D identity - Alignment Gaps [PDB]:** 890
- **3D similarity (TM-Score) (%) [PDB]:** 7.59

- **Gene name:** USP7
- **RefSeq ID:** NM\_003470
- **Transcript sequence length:** 5831
- **5-UTR|CDS|3-UTR identity (%):** 8.07 | 42.68 | 7.82
- **5-UTR|CDS|3-UTR identity (%) [Gaps excluded]:** 80.25 | 78.87 | 76.26
- **5-UTR|CDS|3-UTR identity [Alignment Gaps]:** 724 | 2123 | 1734

**Uniprot Description:**  
  
Hydrolase that deubiquitinates target proteins such as FOXO4, p53/TP53, MDM2, ERCC6, DNMT1, UHRF1, PTEN, KMT2E/MLL5 and DAXX (PubMed:11923872, PubMed:15053880, PubMed:16964248, PubMed:18716620, PubMed:25283148, PubMed:26678539, PubMed:28655758). Together with DAXX, prevents MDM2 self-ubiquitination and enhances the E3 ligase activity of MDM2 towards p53/TP53, thereby promoting p53/TP53 ubiquitination and proteasomal degradation (PubMed:15053880, PubMed:16845383, PubMed:18566590, PubMed:20153724). Deubiquitinates p53/TP53, preventing degradation of p53/TP53, and enhances p53/TP53-dependent transcription regulation, cell growth repression and apoptosis (PubMed:25283148). Deubiquitinates p53/TP53 and MDM2 and strongly stabilizes p53/TP53 even in the presence of excess MDM2, and also induces p53/TP53-dependent cell growth repression and apoptosis (PubMed:11923872). Deubiquitination of FOXO4 in presence of hydrogen peroxide is not dependent on p53/TP53 and inhibits FOXO4-induced transcriptional activity (PubMed:16964248). In association with DAXX, is involved in the deubiquitination and translocation of PTEN from the nucleus to the cytoplasm, both processes that are counteracted by PML (PubMed:18716620). Deubiquitinates KMT2E/MLL5 preventing KMT2E/MLL5 proteasomal-mediated degradation (PubMed:26678539). Involved in cell proliferation during early embryonic development. Involved in transcription-coupled nucleotide excision repair (TC-NER) in response to UV damage: recruited to DNA damage sites following interaction with KIAA1530/UVSSA and promotes deubiquitination of ERCC6, preventing UV-induced degradation of ERCC6 (PubMed:22466611, PubMed:22466612). Involved in maintenance of DNA methylation via its interaction with UHRF1 and DNMT1: acts by mediating deubiquitination of UHRF1 and DNMT1, preventing their degradation and promoting DNA methylation by DNMT1 (PubMed:21745816, PubMed:22411829). Deubiquitinates alkylation repair enzyme ALKBH3. OTUD4 recruits USP7 and USP9X to stabilize ALKBH3, thereby promoting the repair of alkylated DNA lesions (PubMed:25944111). Acts as a chromatin regulator via its association with the Polycomb group (PcG) multiprotein PRC1-like complex; may act by deubiquitinating components of the PRC1-like complex (PubMed:20601937). Able to mediate deubiquitination of histone H2B; it is however unsure whether this activity takes place in vivo (PubMed:20601937). Exhibits a preference towards 'Lys-48'-linked ubiquitin chains (PubMed:22689415). Increases regulatory T-cells (Treg) suppressive capacity by deubiquitinating and stabilizing the transcription factor FOXP3 which is crucial for Treg cell function (PubMed:23973222). Plays a role in the maintenance of the circadian clock periodicity via deubiquitination and stabilization of the CRY1 and CRY2 proteins (PubMed:27123980). Deubiquitinates REST, thereby stabilizing REST and promoting the maintenance of neural progenitor cells (PubMed:21258371). Deubiquitinates SIRT7, inhibiting SIRT7 histone deacetylase activity and regulating gluconeogenesis (PubMed:28655758).  
  
Monomer. Homodimer. Part of a complex with DAXX, MDM2, RASSF1 and USP7 (PubMed:18566590). Part of a complex with DAXX, MDM2 and USP7 (PubMed:16845383). Interacts with MDM2; the interaction is independent of p53/TP53. Interacts with DAXX; the interaction is direct and independent of MDM2 and p53/TP53 (PubMed:16845383). Component of a complex composed of KMT2E/MLL5 (isoform 3), OGT (isoform 1) and USP7; the complex stabilizes KMT2E/MLL5, preventing KMT2E/MLL5 ubiquitination and proteosomal-mediated degradation (PubMed:26678539). Interacts (via MATH domain) with KMT2E/MLL5 isoform 3 (PubMed:26678539). Interacts with OGT isoform 1 (PubMed:26678539). Interacts with FOXO4; the interaction is enhanced in presence of hydrogen peroxide and occurs independently of p53/TP53 (PubMed:16964248). Interacts with p53/TP53; the interaction is enhanced in response to DNA damage (PubMed:25283148). Interacts with TSPYL5; this impairs interaction with p53/TP53 (PubMed:21170034). Interacts with PTEN; the interaction is direct (PubMed:18716620). Interacts with ATXN1 and the strength of interaction is influenced by the length of the poly-Gln region in ATXN1 (PubMed:12093161). A weaker interaction seen with mutants having longer poly-Gln regions (PubMed:12093161). Interacts with KIAA1530/UVSSA (PubMed:22466611, PubMed:22466612). Interacts with ABRAXAS2; the interaction is direct (PubMed:25283148). Identified in a complex with TP53/p53 and ABRAXAS2 (PubMed:25283148). Interacts with MEX3C and antagonizes its ability to degrade mRNA (PubMed:22863774). Interacts with DNMT1 and UHRF1 (PubMed:21745816, PubMed:22411829). Interacts with FOXP3 (PubMed:23973222). Interacts (via MATH domain) with RNF220. Associated component of the Polycomb group (PcG) multiprotein PRC1-like complex (PubMed:20601937). Interacts with EPOP (By similarity). Interacts with OTUD4 and USP9X; the interaction is direct (PubMed:25944111). Interacts with CRY2 (PubMed:27123980). Interacts with REST (PubMed:21258371). Interacts with ERCC6 (PubMed:26030138).  
  
**Gene Ontology Information:**

Molecular Function

- cysteine-type endopeptidase activity
- ubiquitinyl hydrolase activity
- Lys48-specific deubiquitinase activity
- p53 binding
- protein C-terminus binding
- thiol-dependent ubiquitin-specific protease activity
- transcription factor binding
- ubiquitin protein ligase binding

Location

- chromosome
- cytosol
- nuclear body
- nucleoplasm
- nucleus
- PML body
- protein-containing complex

Biological process

- histone H2B conserved C-terminal lysine deubiquitination
- maintenance of DNA methylation
- monoubiquitinated protein deubiquitination
- multicellular organism development
- negative regulation of NF-kappaB transcription factor activity
- negative regulation of proteasomal ubiquitin-dependent protein catabolic process
- positive regulation of DNA demethylation
- protein deubiquitination
- protein K63-linked deubiquitination
- protein stabilization
- protein ubiquitination
- regulation of circadian rhythm
- regulation of DNA-binding transcription factor activity
- regulation of gluconeogenesis
- regulation of protein stability
- regulation of retrograde transport, endosome to Golgi
- regulation of signal transduction by p53 class mediator
- regulation of telomere capping
- rhythmic process
- transcription-coupled nucleotide-excision repair
- ubiquitin-dependent protein catabolic process
- viral process

---

69

- **Protein name:** B-cell lymphoma 6 protein
- **Organism:** Homo sapiens
- **Uniprot Accession Number:** P41182
- **Protein sequence length:** 706 aa
- **1D identity (%):** 12.04
- **1D identity (%) [Gaps excluded]:** 28.21
- **1D identity - Alignment Gaps:** 795
- **Common reported functions (%):** 50.0
- **Common reported locations (%):** 0.0
- **Common reported processes (%):** 0.0

- **PDB ID:** 5H7H
- **Chain:** A
- **Crystallized protein length:** 130 aa
- **Resolution:** 1.95 Å
- **Alinged residues range:** 70-73, 1-3, 42-44, 46-50, 9-11, 58-61, 55-60
- **Aligned to segment part (indices):** 3, 5, 6, 1, 4, 0, 2
- **Alinged residues range of reference:** 46-49, 200-202, 317-319, 364-368, 539-541, 768-771, 1003-1008
- **b-phipsi:** 0.041201
- **w-rdist:** 0.996935
- **t-alpha:** 1.244094
- **Chemical similarity (Tanimoto Index) (%):** 73.09
- **1D identity (%) [PDB]:** 0.0
- **1D identity (%) [Gaps excluded][PDB]:** 0.0
- **1D identity - Alignment Gaps [PDB]:** 1112
- **2D identity (%) [PDB]:** 9.24
- **2D identity (%) [Gaps excluded][PDB]:** 88.57
- **2D identity - Alignment Gaps [PDB]:** 902
- **3D similarity (TM-Score) (%) [PDB]:** 5.73

- **Gene name:** BCL6
- **RefSeq ID:** N/A
- **Sequence length:** N/A
- **5-UTR|CDS|3-UTR identity (%):** N/A | N/A | N/A
- **5-UTR|CDS|3-UTR identity (%) [Gaps excluded]:** N/A | N/A | N/A
- **5-UTR|CDS|3-UTR identity [Alignment Gaps]:** N/A | N/A | N/A

**Uniprot Description:**  
  
Transcriptional repressor mainly required for germinal center (GC) formation and antibody affinity maturation which has different mechanisms of action specific to the lineage and biological functions. Forms complexes with different corepressors and histone deacetylases to repress the transcriptional expression of different subsets of target genes. Represses its target genes by binding directly to the DNA sequence 5'-TTCCTAGAA-3' (BCL6-binding site) or indirectly by repressing the transcriptional activity of transcription factors. In GC B-cells, represses genes that function in differentiation, inflammation, apoptosis and cell cycle control, also autoregulates its transcriptional expression and up-regulates, indirectly, the expression of some genes important for GC reactions, such as AICDA, through the repression of microRNAs expression, like miR155. An important function is to allow GC B-cells to proliferate very rapidly in response to T-cell dependent antigens and tolerate the physiological DNA breaks required for immunglobulin class switch recombination and somatic hypermutation without inducing a p53/TP53-dependent apoptotic response. In follicular helper CD4(+) T-cells (T(FH) cells), promotes the expression of T(FH)-related genes but inhibits the differentiation of T(H)1, T(H)2 and T(H)17 cells. Also required for the establishment and maintenance of immunological memory for both T- and B-cells. Suppresses macrophage proliferation through competition with STAT5 for STAT-binding motifs binding on certain target genes, such as CCL2 and CCND2. In response to genotoxic stress, controls cell cycle arrest in GC B-cells in both p53/TP53-dependedent and -independent manners. Besides, also controls neurogenesis through the alteration of the composition of NOTCH-dependent transcriptional complexes at selective NOTCH targets, such as HES5, including the recruitment of the deacetylase SIRT1 and resulting in an epigenetic silencing leading to neuronal differentiation.  
  
Homodimer. Interacts (via BTB domain) with the corepressors BCOR, NCOR1 and SMRT/NCOR2; the interactions are direct. Forms preferably ternary complexes with BCOR and SMRT/NCOR2 on target gene promoters but, on enhancer elements, interacts with SMRT/NCOR2 and HDAC3 to repress proximal gene expression. Interacts with histone deacetylases HDAC2, HDAC5 and HDAC9 (via the catalytic domain). Interacts with ZBTB7 and BCL6B. Interacts with SCF(FBXO11) complex; the interaction is independent of phosphorylation and promotes ubiquitination. Interacts (when phosphorylated) with PIN1; the interaction is required for BCL6 degradation upon genotoxic stress. Interacts with ZBTB17; inhibits ZBTB17 transcriptional activity. Interacts with CTBP1, autoinhibits its transcriptional expression. Interacts with NOTCH1 NCID and SIRT1; leads to a epigenetic repression of selective NOTCH1-target genes. Interacts (nor via BTB domain neither acetylated) with the NuRD complex components CHD4, HDAC1, MBD3 and MTA3; the interaction with MTA3 inhibits BCL6 acetylation and is required for BCL6 transpriptional repression.  
  
**Gene Ontology Information:**

Molecular Function

- chromatin binding
- chromatin DNA binding
- DNA-binding transcription factor activity
- DNA-binding transcription repressor activity, RNA polymerase II-specific
- identical protein binding
- intronic transcription regulatory region sequence-specific DNA binding
- metal ion binding
- RNA polymerase II cis-regulatory region sequence-specific DNA binding
- sequence-specific DNA binding
- sequence-specific double-stranded DNA binding

Location

- Golgi apparatus
- nucleolus
- nucleoplasm
- nucleus

Biological process

- actin cytoskeleton organization
- B cell differentiation
- cell morphogenesis
- cellular response to DNA damage stimulus
- cytokine-mediated signaling pathway
- erythrocyte development
- germinal center formation
- inflammatory response
- negative regulation of B cell apoptotic process
- negative regulation of cell growth
- negative regulation of cell population proliferation
- negative regulation of cell-matrix adhesion
- negative regulation of cellular senescence
- negative regulation of isotype switching to IgE isotypes
- negative regulation of mast cell cytokine production
- negative regulation of mitotic cell cycle DNA replication
- negative regulation of Notch signaling pathway
- negative regulation of Rho protein signal transduction
- negative regulation of T-helper 2 cell differentiation
- negative regulation of transcription by RNA polymerase II
- negative regulation of transcription, DNA-templated
- positive regulation of apoptotic process
- positive regulation of B cell proliferation
- positive regulation of cellular component movement
- positive regulation of histone deacetylation
- positive regulation of neuron differentiation
- positive regulation of regulatory T cell differentiation
- protein localization
- regulation of apoptotic process
- regulation of cell differentiation
- regulation of cell population proliferation
- regulation of cytokine production
- regulation of germinal center formation
- regulation of GTPase activity
- regulation of immune response
- regulation of immune system process
- regulation of inflammatory response
- regulation of memory T cell differentiation
- regulation of transcription by RNA polymerase II
- Rho protein signal transduction
- spermatogenesis
- type 2 immune response

---

70

- **Protein name:** Gag polyprotein
- **Organism:** Human immunodeficiency virus type 1 group M subtype B (isolate HXB2)
- **Uniprot Accession Number:** P04591
- **Protein sequence length:** 500 aa
- **1D identity (%):** 7.06
- **1D identity (%) [Gaps excluded]:** 29.53
- **1D identity - Alignment Gaps:** 1089
- **Common reported functions (%):** 0.0
- **Common reported locations (%):** 25.0
- **Common reported processes (%):** 20.0

- **PDB ID:** 6EC2
- **Chain:** G
- **Crystallized protein length:** 201 aa
- **Resolution:** 3.4 Å
- **Alinged residues range:** 156-158, 85-87, 102-108, 156-160, 37-56, 67-79
- **Aligned to segment part (indices):** 3, 6, 1, 4, 0, 2
- **Alinged residues range of reference:** 38-40, 307-309, 369-375, 526-530, 738-767, 1002-1017
- **b-phipsi:** 0.137642
- **w-rdist:** 1.090443
- **t-alpha:** 0.819149
- **Chemical similarity (Tanimoto Index) (%):** 83.45
- **1D identity (%) [PDB]:** 0.0
- **1D identity (%) [Gaps excluded][PDB]:** 0.0
- **1D identity - Alignment Gaps [PDB]:** 1187
- **2D identity (%) [PDB]:** 14.63
- **2D identity (%) [Gaps excluded][PDB]:** 97.42
- **2D identity - Alignment Gaps [PDB]:** 877
- **3D similarity (TM-Score) (%) [PDB]:** 8.66

- **Gene name:** gag
- **RefSeq ID:** NC\_001802
- **Genomic sequence length:** 9181
- **5-UTR|CDS|3-UTR identity (%):** 23.6 | 23.81 | 23.63
- **5-UTR|CDS|3-UTR identity (%) [Gaps excluded]:** 75.9 | 79.95 | 80.65
- **5-UTR|CDS|3-UTR identity [Alignment Gaps]:** 184 | 2881 | 374

**Uniprot Description:**  
  
Gag polyprotein
Mediates, with Gag-Pol polyprotein, the essential events in virion assembly, including binding the plasma membrane, making the protein-protein interactions necessary to create spherical particles, recruiting the viral Env proteins, and packaging the genomic RNA via direct interactions with the RNA packaging sequence (Psi).  
  
Gag polyprotein
Homotrimer; further assembles as hexamers of trimers (By similarity). Oligomerization possibly creates a central hole into which the cytoplasmic tail of the gp41 envelope protein may be inserted. Interacts with host TRIM22; this interaction seems to disrupt proper trafficking of Gag polyprotein and may interfere with budding (PubMed:18389079). Interacts with host PDZD8 (PubMed:20573829). When ubiquitinated, interacts (via p6-gag domain) with host PACSIN2; this interaction allows PACSIN2 recruitment to viral assembly sites and its subsequent incorporation into virions (By similarity).  
  
**Gene Ontology Information:**

Molecular Function

- RNA binding
- structural molecule activity
- zinc ion binding

Location

- cytoplasm
- host cell nucleus
- host cell plasma membrane
- host multivesicular body
- nuclear membrane
- nucleolus
- viral nucleocapsid
- virion membrane

Biological process

- entry into host
- establishment of integrated proviral latency
- fusion of virus membrane with host plasma membrane
- intracellular transport of virus
- RNA-dependent DNA biosynthetic process
- uncoating of virus
- viral budding via host ESCRT complex
- viral life cycle
- viral protein processing
- virion assembly

---

71

- **Protein name:** Matrix protein VP40
- **Organism:** Zaire ebolavirus (strain Mayinga-76)
- **Uniprot Accession Number:** Q05128
- **Protein sequence length:** 326 aa
- **1D identity (%):** 6.31
- **1D identity (%) [Gaps excluded]:** 27.42
- **1D identity - Alignment Gaps:** 1001
- **Common reported functions (%):** 50.0
- **Common reported locations (%):** 25.0
- **Common reported processes (%):** 0.0

- **PDB ID:** 7JZT
- **Chain:** C
- **Crystallized protein length:** 233 aa
- **Resolution:** 3.77 Å
- **Alinged residues range:** 74-76, 173-177, 121-124, 301-304, 120-123, 111-115, 265-269
- **Aligned to segment part (indices):** 3, 5, 6, 1, 4, 0, 2
- **Alinged residues range of reference:** 47-49, 200-204, 311-314, 423-426, 593-596, 768-772, 960-964
- **b-phipsi:** 0.030699
- **w-rdist:** 1.231661
- **t-alpha:** 0.908482
- **Chemical similarity (Tanimoto Index) (%):** 82.3
- **1D identity (%) [PDB]:** 0.08
- **1D identity (%) [Gaps excluded][PDB]:** 50.0
- **1D identity - Alignment Gaps [PDB]:** 1216
- **2D identity (%) [PDB]:** 13.62
- **2D identity (%) [Gaps excluded][PDB]:** 88.34
- **2D identity - Alignment Gaps [PDB]:** 894
- **3D similarity (TM-Score) (%) [PDB]:** 9.11

- **Gene name:** VP40
- **RefSeq ID:** NC\_002549
- **Genomic sequence length:** 18959
- **5-UTR|CDS|3-UTR identity (%):** 15.79 | 17.43 | 19.86
- **5-UTR|CDS|3-UTR identity (%) [Gaps excluded]:** 77.78 | 79.12 | 74.35
- **5-UTR|CDS|3-UTR identity [Alignment Gaps]:** 212 | 3069 | 524

**Uniprot Description:**  
  
Plays an essential role virus particle assembly and budding (PubMed:16719918). Acts by interacting with viral ribonucleocapsid and host members of the ESCRT (endosomal sorting complex required for transport) system such as host VPS4, PDCD6IP/ALIX, NEDD4 or TGS101 (PubMed:15892969, PubMed:16719918, PubMed:23637409, PubMed:25786915, PubMed:26753796, PubMed:27489272). May play a role in immune cell dysfunction by being packaged into exosomes that can decrease the viability of recipient cells (via RNAi suppression and exosome-bystander apoptosis) (PubMed:27872619).  
  
Homodimer (PubMed:23953110). Homohexamer (PubMed:23953110, PubMed:11118208). Homooctamer (PubMed:12919741). Exists as a dimer until it reorganizes at the plasma membrane into a hexameric form using phosphatidylinositol 4,5-bisphosphate (PI(4,5)P2) (PubMed:23953110, PubMed:25159197, PubMed:26753796, PubMed:29950600). Hexamers are critical for budding (PubMed:23953110). Octamers function in genome replication and RNA binding (PubMed:12919741). Interacts with host TSG101 (PubMed:12559917). As a homohexamer, interacts with the WW domain 3 of host NEDD4 (PubMed:11095724, PubMed:12559917). Interacts with the nucleoprotein/NP (PubMed:17229682, PubMed:21987757). Interacts (via YPx(n)L/I motif) with host PDCD6IP/ALIX; this interaction supports efficient egress of viral particles (PubMed:25786915). Interacts with VP35 (PubMed:16698994). Interacts with host ITCH; this interaction is required for efficient egress (PubMed:27489272).  
  
**Gene Ontology Information:**

Molecular Function

- identical protein binding
- RNA binding
- structural constituent of virion

Location

- extracellular region
- host cell endomembrane system
- host cell late endosome membrane
- host cell plasma membrane
- integral to membrane of host cell
- membrane raft
- virion membrane

Biological process

- intracellular transport of virus
- mitigation of host immune response by virus
- suppression of host defenses by symbiont
- viral budding
- viral budding from plasma membrane
- viral budding via host ESCRT complex

---

72

- **Protein name:** Coat protein
- **Organism:** Acinetobacter phage AP205
- **Uniprot Accession Number:** Q9AZ42
- **Protein sequence length:** 131 aa
- **1D identity (%):** 2.83
- **1D identity (%) [Gaps excluded]:** 27.69
- **1D identity - Alignment Gaps:** 1144
- **Common reported functions (%):** 0.0
- **Common reported locations (%):** 0.0
- **Common reported processes (%):** 0.0

- **PDB ID:** 5FS4
- **Chain:** A
- **Crystallized protein length:** 118 aa
- **Resolution:** 1.73 Å
- **Alinged residues range:** 125-127, 45-49, 52-57, 14-19, 80-83, 96-99, 89-100
- **Aligned to segment part (indices):** 3, 5, 6, 1, 4, 0, 2
- **Alinged residues range of reference:** 50-52, 202-206, 311-316, 432-437, 596-599, 761-764, 1001-1011
- **b-phipsi:** 0.048382
- **w-rdist:** 1.21678
- **t-alpha:** 0.875
- **Chemical similarity (Tanimoto Index) (%):** 81.47
- **1D identity (%) [PDB]:** 0.0
- **1D identity (%) [Gaps excluded][PDB]:** 0.0
- **1D identity - Alignment Gaps [PDB]:** 1101
- **2D identity (%) [PDB]:** 8.44
- **2D identity (%) [Gaps excluded][PDB]:** 90.43
- **2D identity - Alignment Gaps [PDB]:** 913
- **3D similarity (TM-Score) (%) [PDB]:** 6.51

- **Gene name:** N/A
- **RefSeq ID:** NC\_002700
- **Genomic sequence length:** 4268
- **5-UTR|CDS|3-UTR identity (%):** N/A | 7.05 | N/A
- **5-UTR|CDS|3-UTR identity (%) [Gaps excluded]:** N/A | 79.36 | N/A
- **5-UTR|CDS|3-UTR identity [Alignment Gaps]:** N/A | 3530 | N/A

**Uniprot Description:**  
  
N/A  
  
**Gene Ontology Information:**

Molecular Function  
  
N/A

Location

- viral capsid

Biological process  
  
N/A

---

73

- **Protein name:** Cellular tumor antigen p53
- **Organism:** Homo sapiens
- **Uniprot Accession Number:** P04637
- **Protein sequence length:** 393 aa
- **1D identity (%):** 6.24
- **1D identity (%) [Gaps excluded]:** 29.86
- **1D identity - Alignment Gaps:** 1090
- **Common reported functions (%):** 50.0
- **Common reported locations (%):** 0.0
- **Common reported processes (%):** 0.0

- **PDB ID:** 2H1L
- **Chain:** O
- **Crystallized protein length:** 199 aa
- **Resolution:** 3.16 Å
- **Alinged residues range:** 230-233, 269-272, 268-273, 266-273, 217-220, 240-242, 284-286
- **Aligned to segment part (indices):** 3, 5, 6, 1, 4, 0, 2
- **Alinged residues range of reference:** 50-53, 200-203, 314-319, 396-403, 597-600, 757-759, 1009-1011
- **b-phipsi:** 0.029651
- **w-rdist:** 1.218193
- **t-alpha:** 1.23822
- **Chemical similarity (Tanimoto Index) (%):** 82.08
- **1D identity (%) [PDB]:** 0.08
- **1D identity (%) [Gaps excluded][PDB]:** 100.0
- **1D identity - Alignment Gaps [PDB]:** 1180
- **2D identity (%) [PDB]:** 11.42
- **2D identity (%) [Gaps excluded][PDB]:** 91.6
- **2D identity - Alignment Gaps [PDB]:** 920
- **3D similarity (TM-Score) (%) [PDB]:** 9.59

- **Gene name:** TP53
- **RefSeq ID:** N/A
- **Sequence length:** N/A
- **5-UTR|CDS|3-UTR identity (%):** N/A | N/A | N/A
- **5-UTR|CDS|3-UTR identity (%) [Gaps excluded]:** N/A | N/A | N/A
- **5-UTR|CDS|3-UTR identity [Alignment Gaps]:** N/A | N/A | N/A

**Uniprot Description:**  
  
Acts as a tumor suppressor in many tumor types; induces growth arrest or apoptosis depending on the physiological circumstances and cell type. Involved in cell cycle regulation as a trans-activator that acts to negatively regulate cell division by controlling a set of genes required for this process. One of the activated genes is an inhibitor of cyclin-dependent kinases. Apoptosis induction seems to be mediated either by stimulation of BAX and FAS antigen expression, or by repression of Bcl-2 expression. Its pro-apoptotic activity is activated via its interaction with PPP1R13B/ASPP1 or TP53BP2/ASPP2 (PubMed:12524540). However, this activity is inhibited when the interaction with PPP1R13B/ASPP1 or TP53BP2/ASPP2 is displaced by PPP1R13L/iASPP (PubMed:12524540). In cooperation with mitochondrial PPIF is involved in activating oxidative stress-induced necrosis; the function is largely independent of transcription. Induces the transcription of long intergenic non-coding RNA p21 (lincRNA-p21) and lincRNA-Mkln1. LincRNA-p21 participates in TP53-dependent transcriptional repression leading to apoptosis and seems to have an effect on cell-cycle regulation. Implicated in Notch signaling cross-over. Prevents CDK7 kinase activity when associated to CAK complex in response to DNA damage, thus stopping cell cycle progression. Isoform 2 enhances the transactivation activity of isoform 1 from some but not all TP53-inducible promoters. Isoform 4 suppresses transactivation activity and impairs growth suppression mediated by isoform 1. Isoform 7 inhibits isoform 1-mediated apoptosis. Regulates the circadian clock by repressing CLOCK-ARNTL/BMAL1-mediated transcriptional activation of PER2 (PubMed:24051492).  
  
Forms homodimers and homotetramers (PubMed:19011621). Binds DNA as a homotetramer. Interacts with AXIN1. Probably part of a complex consisting of TP53, HIPK2 and AXIN1 (By similarity). Interacts with histone acetyltransferases EP300 and methyltransferases HRMT1L2 and CARM1, and recruits them to promoters. Interacts (via C-terminus) with TAF1; when TAF1 is part of the TFIID complex. Interacts with ING4; this interaction may be indirect. Found in a complex with CABLES1 and TP73. Interacts with HIPK1, HIPK2, and TP53INP1. Interacts with WWOX. May interact with HCV core protein. Interacts with USP7 and SYVN1. Interacts with HSP90AB1. Interacts with CHD8; leading to recruit histone H1 and prevent transactivation activity (By similarity). Interacts with ARMC10, BANP, CDKN2AIP, NUAK1, STK11/LKB1, UHRF2 and E4F1. Interacts with YWHAZ; the interaction enhances TP53 transcriptional activity. Phosphorylation of YWHAZ on 'Ser-58' inhibits this interaction. Interacts (via DNA-binding domain) with MAML1 (via N-terminus). Interacts with MKRN1. Interacts with PML (via C-terminus). Interacts with MDM2; leading to ubiquitination and proteasomal degradation of TP53. Directly interacts with FBXO42; leading to ubiquitination and degradation of TP53. Interacts (phosphorylated at Ser-15 by ATM) with the phosphatase PP2A-PPP2R5C holoenzyme; regulates stress-induced TP53-dependent inhibition of cell proliferation. Interacts with PPP2R2A. Interacts with AURKA, DAXX, BRD7 and TRIM24. Interacts (when monomethylated at Lys-382) with L3MBTL1. Isoform 1 interacts with isoform 2 and with isoform 4. Interacts with GRK5. Binds to the CAK complex (CDK7, cyclin H and MAT1) in response to DNA damage. Interacts with CDK5 in neurons. Interacts with AURKB, SETD2, UHRF2 and NOC2L. Interacts (via N-terminus) with PTK2/FAK1; this promotes ubiquitination by MDM2. Interacts with PTK2B/PYK2; this promotes ubiquitination by MDM2. Interacts with PRKCG. Interacts with PPIF; the association implicates preferentially tetrameric TP53, is induced by oxidative stress and is impaired by cyclosporin A (CsA). Interacts with SNAI1; the interaction induces SNAI1 degradation via MDM2-mediated ubiquitination and inhibits SNAI1-induced cell invasion. Interacts with KAT6A. Interacts with UBC9. Interacts with ZNF385B; the interaction is direct. Interacts (via DNA-binding domain) with ZNF385A; the interaction is direct and enhances p53/TP53 transactivation functions on cell-cycle arrest target genes, resulting in growth arrest. Interacts with ANKRD2. Interacts with RFFL and RNF34; involved in p53/TP53 ubiquitination. Interacts with MTA1 and COP1. Interacts with CCAR2 (via N-terminus). Interacts with MORC3 (PubMed:17332504). Interacts (via C-terminus) with POU4F2 isoform 1 (via C-terminus) (PubMed:17145718). Interacts (via oligomerization region) with NOP53; the interaction is direct and may prevent the MDM2-mediated proteasomal degradation of TP53 (PubMed:22522597). Interacts with AFG1L; mediates mitochondrial translocation of TP53 (PubMed:27323408). Interacts with UBD (PubMed:25422469). Interacts with TAF6 isoform 1 and isoform 4 (PubMed:20096117). Interacts with C10orf90/FATS; the interaction inhibits binding of TP53 and MDM2 (By similarity). Interacts with NUPR1; interaction is stress-dependent (PubMed:18690848). Forms a complex with EP300 and NUPR1; this complex binds CDKN1A promoter leading to transcriptional induction of CDKN1A (PubMed:18690848). Interacts with PRMT5 in response to DNA damage; the interaction is STRAP dependent (PubMed:19011621). Interacts with PPP1R13L (via SH3 domain and ANK repeats); the interaction inhibits pro-apoptotic activity of p53/TP53 (PubMed:12524540). Interacts with PPP1R13B/ASPP1 and TP53BP2/ASPP2; the interactions promotes pro-apoptotic activity (PubMed:12524540). When phosphorylated at Ser-15, interacts with DDX3X and gamma-tubulin (PubMed:28842590). Interacts with KAT7/HBO1; leading to inhibit histone acetyltransferase activity of KAT7/HBO1 (PubMed:17954561). Interacts (via N-terminus) with E3 ubiquitin-protein ligase MUL1; the interaction results in ubiquitination of cytoplasmic TP53 at Lys-24 and subsequent proteasomal degradation (PubMed:21597459).  
  
**Gene Ontology Information:**

Molecular Function

- chaperone binding
- chromatin binding
- cis-regulatory region sequence-specific DNA binding
- copper ion binding
- core promoter sequence-specific DNA binding
- disordered domain specific binding
- DNA binding
- DNA-binding transcription activator activity
- DNA-binding transcription activator activity, RNA polymerase II-specific
- DNA-binding transcription factor activity
- DNA-binding transcription factor activity, RNA polymerase II-specific
- enzyme binding
- histone acetyltransferase binding
- histone deacetylase binding
- histone deacetylase regulator activity
- identical protein binding
- MDM2/MDM4 family protein binding
- mRNA 3'-UTR binding
- p53 binding
- promoter-specific chromatin binding
- protease binding
- protein heterodimerization activity
- protein kinase binding
- protein N-terminus binding
- protein phosphatase 2A binding
- protein phosphatase binding
- protein self-association
- receptor tyrosine kinase binding
- RNA polymerase II cis-regulatory region sequence-specific DNA binding
- RNA polymerase II transcription factor binding
- TFIID-class transcription factor complex binding
- transcription factor binding
- transcription regulatory region sequence-specific DNA binding
- ubiquitin protein ligase binding
- zinc ion binding

Location

- centrosome
- chromatin
- cytoplasm
- cytosol
- endoplasmic reticulum
- mitochondrial matrix
- mitochondrion
- nuclear matrix
- nucleolus
- nucleoplasm
- nucleus
- PML body
- protein-containing complex
- replication fork
- site of double-strand break
- transcription regulator complex

Biological process

- autophagy
- B cell lineage commitment
- bone marrow development
- cardiac septum morphogenesis
- cell aging
- cell cycle arrest
- cellular protein localization
- cellular response to actinomycin D
- cellular response to DNA damage stimulus
- cellular response to drug
- cellular response to gamma radiation
- cellular response to glucose starvation
- cellular response to hypoxia
- cellular response to ionizing radiation
- cellular response to UV
- cellular response to UV-C
- cerebellum development
- chromatin assembly
- circadian behavior
- cytokine-mediated signaling pathway
- determination of adult lifespan
- DNA damage response, signal transduction by p53 class mediator
- DNA damage response, signal transduction by p53 class mediator resulting in cell cycle arrest
- DNA damage response, signal transduction by p53 class mediator resulting in transcription of p21 class mediator
- DNA strand renaturation
- double-strand break repair
- embryonic organ development
- entrainment of circadian clock by photoperiod
- ER overload response
- gastrulation
- hematopoietic progenitor cell differentiation
- hematopoietic stem cell differentiation
- in utero embryonic development
- interferon-gamma-mediated signaling pathway
- intrinsic apoptotic signaling pathway
- intrinsic apoptotic signaling pathway by p53 class mediator
- intrinsic apoptotic signaling pathway in response to DNA damage by p53 class mediator
- intrinsic apoptotic signaling pathway in response to endoplasmic reticulum stress
- intrinsic apoptotic signaling pathway in response to hypoxia
- mitochondrial DNA repair
- mitotic cell cycle arrest
- mitotic G1 DNA damage checkpoint
- mRNA transcription
- multicellular organism growth
- necroptotic process
- negative regulation of apoptotic process
- negative regulation of cell growth
- negative regulation of cell population proliferation
- negative regulation of DNA replication
- negative regulation of fibroblast proliferation
- negative regulation of glucose catabolic process to lactate via pyruvate
- negative regulation of helicase activity
- negative regulation of mitophagy
- negative regulation of neuroblast proliferation
- negative regulation of pentose-phosphate shunt
- negative regulation of production of miRNAs involved in gene silencing by miRNA
- negative regulation of proteolysis
- negative regulation of reactive oxygen species metabolic process
- negative regulation of telomerase activity
- negative regulation of transcription by RNA polymerase II
- negative regulation of transcription, DNA-templated
- negative regulation of transforming growth factor beta receptor signaling pathway
- neuron apoptotic process
- nucleotide-excision repair
- oligodendrocyte apoptotic process
- oxidative stress-induced premature senescence
- positive regulation of apoptotic process
- positive regulation of cardiac muscle cell apoptotic process
- positive regulation of cell aging
- positive regulation of cell cycle arrest
- positive regulation of execution phase of apoptosis
- positive regulation of gene expression
- positive regulation of histone deacetylation
- positive regulation of intrinsic apoptotic signaling pathway
- positive regulation of mitochondrial membrane permeability
- positive regulation of neuron apoptotic process
- positive regulation of peptidyl-tyrosine phosphorylation
- positive regulation of pri-miRNA transcription by RNA polymerase II
- positive regulation of production of miRNAs involved in gene silencing by miRNA
- positive regulation of programmed necrotic cell death
- positive regulation of protein export from nucleus
- positive regulation of protein insertion into mitochondrial membrane involved in apoptotic signaling pathway
- positive regulation of reactive oxygen species metabolic process
- positive regulation of release of cytochrome c from mitochondria
- positive regulation of RNA polymerase II transcription preinitiation complex assembly
- positive regulation of thymocyte apoptotic process
- positive regulation of transcription by RNA polymerase II
- positive regulation of transcription from RNA polymerase II promoter in response to endoplasmic reticulum stress
- positive regulation of transcription from RNA polymerase II promoter in response to hypoxia
- positive regulation of transcription from RNA polymerase II promoter in response to stress
- positive regulation of transcription, DNA-templated
- protein deubiquitination
- protein import into nucleus
- protein localization
- protein stabilization
- protein tetramerization
- protein-containing complex assembly
- Ras protein signal transduction
- regulation of apoptotic process
- regulation of cell cycle G2/M phase transition
- regulation of cellular senescence
- regulation of DNA damage response, signal transduction by p53 class mediator
- regulation of fibroblast apoptotic process
- regulation of intrinsic apoptotic signaling pathway by p53 class mediator
- regulation of mitochondrial membrane permeability involved in apoptotic process
- regulation of signal transduction by p53 class mediator
- regulation of tissue remodeling
- regulation of transcription by RNA polymerase II
- regulation of transcription from RNA polymerase II promoter in response to DNA damage
- regulation of transcription initiation from RNA polymerase II promoter
- regulation of transcription, DNA-templated
- release of cytochrome c from mitochondria
- replicative senescence
- response to antibiotic
- response to gamma radiation
- response to ischemia
- response to salt stress
- response to X-ray
- rRNA transcription
- signal transduction by p53 class mediator
- somitogenesis
- T cell differentiation in thymus
- T cell lineage commitment
- T cell proliferation involved in immune response
- transforming growth factor beta receptor signaling pathway
- tumor necrosis factor-mediated signaling pathway
- viral process

---

74

- **Protein name:** Histone H3.3
- **Organism:** Homo sapiens
- **Uniprot Accession Number:** P84243
- **Protein sequence length:** 136 aa
- **1D identity (%):** 3.2
- **1D identity (%) [Gaps excluded]:** 32.28
- **1D identity - Alignment Gaps:** 1155
- **Common reported functions (%):** 0.0
- **Common reported locations (%):** 0.0
- **Common reported processes (%):** 0.0

- **PDB ID:** 5KDM
- **Chain:** A
- **Crystallized protein length:** 99 aa
- **Resolution:** 3.5 Å
- **Alinged residues range:** 42-44, 106-108, 79-82, 80-82, 105-109, 68-71
- **Aligned to segment part (indices):** 3, 6, 1, 4, 0, 2
- **Alinged residues range of reference:** 44-46, 298-300, 422-425, 531-533, 748-752, 994-997
- **b-phipsi:** 0.220425
- **w-rdist:** 1.169973
- **t-alpha:** 0.908482
- **Chemical similarity (Tanimoto Index) (%):** 72.59
- **1D identity (%) [PDB]:** 0.09
- **1D identity (%) [Gaps excluded][PDB]:** 100.0
- **1D identity - Alignment Gaps [PDB]:** 1080
- **2D identity (%) [PDB]:** 8.29
- **2D identity (%) [Gaps excluded][PDB]:** 88.17
- **2D identity - Alignment Gaps [PDB]:** 896
- **3D similarity (TM-Score) (%) [PDB]:** 6.31

- **Gene name:** H3-3A
- **RefSeq ID:** NM\_005324
- **Transcript sequence length:** 2705
- **5-UTR|CDS|3-UTR identity (%):** 28.28 | 7.13 | 6.97
- **5-UTR|CDS|3-UTR identity (%) [Gaps excluded]:** 75.93 | 79.6 | 78.87
- **5-UTR|CDS|3-UTR identity [Alignment Gaps]:** 182 | 3537 | 2002

**Uniprot Description:**  
  
Variant histone H3 which replaces conventional H3 in a wide range of nucleosomes in active genes. Constitutes the predominant form of histone H3 in non-dividing cells and is incorporated into chromatin independently of DNA synthesis. Deposited at sites of nucleosomal displacement throughout transcribed genes, suggesting that it represents an epigenetic imprint of transcriptionally active chromatin. Nucleosomes wrap and compact DNA into chromatin, limiting DNA accessibility to the cellular machineries which require DNA as a template. Histones thereby play a central role in transcription regulation, DNA repair, DNA replication and chromosomal stability. DNA accessibility is regulated via a complex set of post-translational modifications of histones, also called histone code, and nucleosome remodeling.  
  
The nucleosome is a histone octamer containing two molecules each of H2A, H2B, H3 and H4 assembled in one H3-H4 heterotetramer and two H2A-H2B heterodimers. The octamer wraps approximately 147 bp of DNA. Interacts with HIRA, a chaperone required for its incorporation into nucleosomes. Interacts with ZMYND11; when trimethylated at 'Lys-36' (H3.3K36me3).  
  
**Gene Ontology Information:**

Molecular Function

- nucleosomal DNA binding
- protein heterodimerization activity
- RNA polymerase II cis-regulatory region sequence-specific DNA binding
- RNA polymerase II core promoter sequence-specific DNA binding

Location

- Barr body
- chromosome, telomeric region
- extracellular exosome
- extracellular region
- nuclear chromosome
- nucleoplasm
- nucleosome
- nucleus
- protein-containing complex

Biological process

- amyloid fibril formation
- blood coagulation
- cell population proliferation
- DNA replication-independent nucleosome assembly
- embryo implantation
- male gonad development
- multicellular organism growth
- muscle cell differentiation
- negative regulation of chromosome condensation
- negative regulation of gene expression, epigenetic
- nucleosome assembly
- nucleus organization
- oogenesis
- osteoblast differentiation
- pericentric heterochromatin assembly
- positive regulation of cell growth
- positive regulation of histone exchange
- rDNA heterochromatin assembly
- regulation of androgen receptor signaling pathway
- regulation of centromere complex assembly
- regulation of gene silencing by miRNA
- regulation of megakaryocyte differentiation
- single fertilization
- spermatid development
- subtelomeric heterochromatin assembly
- telomere organization

---

75

- **Protein name:** Major capsid protein
- **Organism:** Escherichia phage T7
- **Uniprot Accession Number:** P19726
- **Protein sequence length:** 345 aa
- **1D identity (%):** 5.53
- **1D identity (%) [Gaps excluded]:** 28.63
- **1D identity - Alignment Gaps:** 1094
- **Common reported functions (%):** 50.0
- **Common reported locations (%):** 0.0
- **Common reported processes (%):** 0.0

- **PDB ID:** 3J7V
- **Chain:** D
- **Crystallized protein length:** 298 aa
- **Resolution:** 4.5 Å
- **Alinged residues range:** 167-169, 91-93, 126-129, 123-128, 248-254, 124-130, 121-135
- **Aligned to segment part (indices):** 3, 5, 6, 1, 4, 0, 2
- **Alinged residues range of reference:** 38-40, 195-197, 296-299, 365-370, 524-530, 747-753, 1005-1017
- **b-phipsi:** 0.031519
- **w-rdist:** 1.21977
- **t-alpha:** 1.705696
- **Chemical similarity (Tanimoto Index) (%):** N/A
- **1D identity (%) [PDB]:** 0.08
- **1D identity (%) [Gaps excluded][PDB]:** 50.0
- **1D identity - Alignment Gaps [PDB]:** 1279
- **2D identity (%) [PDB]:** 14.68
- **2D identity (%) [Gaps excluded][PDB]:** 86.56
- **2D identity - Alignment Gaps [PDB]:** 911
- **3D similarity (TM-Score) (%) [PDB]:** 9.65

- **Gene name:** 10
- **RefSeq ID:** NC\_001604
- **Genomic sequence length:** 39937
- **5-UTR|CDS|3-UTR identity (%):** N/A | 18.97 | N/A
- **5-UTR|CDS|3-UTR identity (%) [Gaps excluded]:** N/A | 79.4 | N/A
- **5-UTR|CDS|3-UTR identity [Alignment Gaps]:** N/A | 2986 | N/A

**Uniprot Description:**  
  
Assembles with the minor capsid protein to form an icosahedral capsid with a T=7 symmetry, about 60 nm in diameter, and consisting of 415 capsid proteins. The major and minor capsid proteins are incorporated into the capsid in about a 90/10 ratio respectively. Once the capsid is formed, encapsidates one single copy of the viral genome.  
  
Homohexamer (PubMed:25313071). Interacts with the connector protein and the minor capsid protein (PubMed:20962334, PubMed:23580619). Interacts with the capsid assembly scaffolding protein; capsid proteins and scaffolding proteins form building blocks that assemble to form the procapsid, each hexamer of the major capsid protein interacting with 2 scaffolding proteins (PubMed:25313071).  
  
**Gene Ontology Information:**

Molecular Function

- identical protein binding

Location

- viral capsid

Biological process  
  
N/A

---

76

- **Protein name:** Single-stranded DNA-binding protein
- **Organism:** Escherichia phage T7
- **Uniprot Accession Number:** P03696
- **Protein sequence length:** 232 aa
- **1D identity (%):** 4.27
- **1D identity (%) [Gaps excluded]:** 28.72
- **1D identity - Alignment Gaps:** 1115
- **Common reported functions (%):** 0.0
- **Common reported locations (%):** 0.0
- **Common reported processes (%):** 0.0

- **PDB ID:** 1JE5
- **Chain:** A
- **Crystallized protein length:** 181 aa
- **Resolution:** 1.9 Å
- **Alinged residues range:** 122-124, 177-179, 36-39, 94-100, 99-102, 69-71, 58-65
- **Aligned to segment part (indices):** 3, 5, 6, 1, 4, 0, 2
- **Alinged residues range of reference:** 51-53, 226-228, 284-287, 374-380, 578-581, 771-773, 951-958
- **b-phipsi:** 0.057506
- **w-rdist:** 1.24901
- **t-alpha:** 1.040573
- **Chemical similarity (Tanimoto Index) (%):** 82.88
- **1D identity (%) [PDB]:** 0.0
- **1D identity (%) [Gaps excluded][PDB]:** 0.0
- **1D identity - Alignment Gaps [PDB]:** 1167
- **2D identity (%) [PDB]:** 11.99
- **2D identity (%) [Gaps excluded][PDB]:** 87.23
- **2D identity - Alignment Gaps [PDB]:** 885
- **3D similarity (TM-Score) (%) [PDB]:** 7.19

- **Gene name:** 2.5
- **RefSeq ID:** NC\_001604
- **Genomic sequence length:** 39937
- **5-UTR|CDS|3-UTR identity (%):** N/A | 11.68 | N/A
- **5-UTR|CDS|3-UTR identity (%) [Gaps excluded]:** N/A | 81.77 | N/A
- **5-UTR|CDS|3-UTR identity [Alignment Gaps]:** N/A | 3391 | N/A

**Uniprot Description:**  
  
Single-stranded DNA-binding protein that participates in viral DNA replication, formation of concatemers, recombination and repair of double-stranded breaks (PubMed:16807232, PubMed:8617248, PubMed:9079662, PubMed:11222583). Coats the lagging-strand ssDNA as the replication fork advances and stimulates the activities of viral DNA polymerase and primase/helicase (PubMed:8617248). Coordinates simultaneous synthesis of leading- and lagging-strands (PubMed:9651583). Together with DNA primase/helicase, promotes pairing of two homologous DNA molecules containing complementary single-stranded regions and mediates homologous DNA strand exchange (PubMed:8617248). Promotes also the formation of joint molecules (PubMed:8617248, PubMed:9079662). Disrupts loops, hairpins and other secondary structures present on ssDNA to reduce and eliminate pausing of viral DNA polymerase at specific sites during elongation (PubMed:1634538).  
  
Homodimer (PubMed:1634538, PubMed:11481454). Interacts (via C-terminus) with the viral DNA polymerase (PubMed:15795374, PubMed:1634539, PubMed:8106511, PubMed:12766155). Interacts with the viral helicase/primase (PubMed:1634539). Part of the replicase complex that includes the DNA polymerase, host thioredoxin, the primase/helicase and the single-stranded DNA binding protein (PubMed:22977246).  
  
**Gene Ontology Information:**

Molecular Function

- DNA binding

Location  
  
N/A

Biological process

- DNA repair
- DNA replication
- viral DNA genome replication

---

77

- **Protein name:** Glutaredoxin-2
- **Organism:** Vaccinia virus (strain Western Reserve)
- **Uniprot Accession Number:** P68460
- **Protein sequence length:** 124 aa
- **1D identity (%):** 2.41
- **1D identity (%) [Gaps excluded]:** 27.43
- **1D identity - Alignment Gaps:** 1171
- **Common reported functions (%):** 0.0
- **Common reported locations (%):** 0.0
- **Common reported processes (%):** 0.0

- **PDB ID:** 2G2Q
- **Chain:** B
- **Crystallized protein length:** 118 aa
- **Resolution:** 2.5 Å
- **Alinged residues range:** 32-34, 71-76, 72-76, 6-9, 32-35, 20-24, 104-113
- **Aligned to segment part (indices):** 3, 5, 6, 1, 4, 0, 2
- **Alinged residues range of reference:** 47-49, 203-208, 311-315, 433-436, 584-587, 769-773, 1001-1010
- **b-phipsi:** 0.108726
- **w-rdist:** 1.284661
- **t-alpha:** 1.045455
- **Chemical similarity (Tanimoto Index) (%):** 83.75
- **1D identity (%) [PDB]:** 0.46
- **1D identity (%) [Gaps excluded][PDB]:** 62.5
- **1D identity - Alignment Gaps [PDB]:** 1087
- **2D identity (%) [PDB]:** 7.01
- **2D identity (%) [Gaps excluded][PDB]:** 94.74
- **2D identity - Alignment Gaps [PDB]:** 951
- **3D similarity (TM-Score) (%) [PDB]:** 7.56

- **Gene name:** VACWR081
- **RefSeq ID:** NC\_006998
- **Genomic sequence length:** 194711
- **5-UTR|CDS|3-UTR identity (%):** N/A | 6.64 | N/A
- **5-UTR|CDS|3-UTR identity (%) [Gaps excluded]:** N/A | 79.32 | N/A
- **5-UTR|CDS|3-UTR identity [Alignment Gaps]:** N/A | 3549 | N/A

**Uniprot Description:**  
  
Glutaredoxin necessary for virion morphogenesis and virus replication. Functions as a thiol-disulfide transfer protein between membrane-associated A2.5 and substrates L1 or F9. The complete pathway for formation of disulfide bonds in intracellular virion membrane proteins sequentially involves oxidation of E10, A2.5 and G4. Exhibit thioltransferase and dehydroascorbate reductase activities in vitro.  
  
Homodimer (Probable). Interacts with A2.5; this interaction involves formation of a transient disulfide-bonded intermediate, allowing disulfide bond transfer. Interacts with L1; this interaction involves formation of a transient disulfide-bonded intermediate, allowing disulfide bond transfer.  
  
**Gene Ontology Information:**

Molecular Function  
  
N/A

Location

- host cell cytoplasm

Biological process  
  
N/A

---

78

- **Protein name:** Coat protein
- **Organism:** Physalis mottle virus
- **Uniprot Accession Number:** P36351
- **Protein sequence length:** 188 aa
- **1D identity (%):** 3.24
- **1D identity (%) [Gaps excluded]:** 25.3
- **1D identity - Alignment Gaps:** 1129
- **Common reported functions (%):** 0.0
- **Common reported locations (%):** 0.0
- **Common reported processes (%):** 0.0

- **PDB ID:** 1E57
- **Chain:** B
- **Crystallized protein length:** 183 aa
- **Resolution:** 3.2 Å
- **Alinged residues range:** 161-163, 102-104, 181-183, 141-147, 23-26, 61-63, 59-62
- **Aligned to segment part (indices):** 3, 5, 6, 1, 4, 0, 2
- **Alinged residues range of reference:** 38-40, 219-221, 313-315, 377-383, 578-581, 761-763, 1007-1010
- **b-phipsi:** 0.058381
- **w-rdist:** 1.349918
- **t-alpha:** 1.583082
- **Chemical similarity (Tanimoto Index) (%):** 83.25
- **1D identity (%) [PDB]:** 0.09
- **1D identity (%) [Gaps excluded][PDB]:** 50.0
- **1D identity - Alignment Gaps [PDB]:** 1162
- **2D identity (%) [PDB]:** 12.52
- **2D identity (%) [Gaps excluded][PDB]:** 83.55
- **2D identity - Alignment Gaps [PDB]:** 862
- **3D similarity (TM-Score) (%) [PDB]:** 8.02

- **Gene name:** N/A
- **RefSeq ID:** NC\_003634
- **Genomic sequence length:** 6673
- **5-UTR|CDS|3-UTR identity (%):** N/A | 9.9 | N/A
- **5-UTR|CDS|3-UTR identity (%) [Gaps excluded]:** N/A | 75.29 | N/A
- **5-UTR|CDS|3-UTR identity [Alignment Gaps]:** N/A | 3369 | N/A

**Uniprot Description:**  
  
N/A  
  
The virus coat is composed of 180 copies of the coat protein arranged in an icosahedral shell.  
  
**Gene Ontology Information:**

Molecular Function

- structural molecule activity

Location

- T=3 icosahedral viral capsid

Biological process  
  
N/A

---
