# Supplementary material for: Identifying and profiling structural similarities between Spike of SARS-CoV-2 and other viral or host proteins with Machaon
Source: Commun Biol. 2023 Jul 19;6:752. doi: 10.1038/s42003-023-05076-7 (PMC10356814; doi:10.1038/s42003-023-05076-7)
Supplement: Supplementary file 7 — Supplementary Data 4 [file 42003_2023_5076_MOESM7_ESM.zip › 6VXX_A_segment/candidates/6VXX_A_site1-metrics-merged-enriched_eval_report.html]

 

# Structural Comparison Report for 6VXX\_A\_site1 - segments (total: 52)

---

1

- **Protein name:** Spike glycoprotein
- **Organism:** Bat coronavirus RaTG13
- **Uniprot Accession Number:** A0A6B9WHD3
- **Protein sequence length:** 1269 aa
- **1D identity (%):** 97.41
- **1D identity (%) [Gaps excluded]:** 97.71
- **1D identity - Alignment Gaps:** 4
- **Common reported functions (%):** 0.0
- **Common reported locations (%):** 50.0
- **Common reported processes (%):** 50.0

- **PDB ID:** 6ZGF
- **Chain:** B
- **Crystallized protein length:** 1060 aa
- **Resolution:** 3.1 Å
- **Alinged residues range:** 38-53, 195-229, 274-438, 514-614, 646-778, 857-861, 953-972
- **Aligned to segment part (indices):** 2, 4, 1, 6, 0, 3, 5
- **Alinged residues range of reference:** 38-53, 195-229, 274-438, 514-614, 646-782, 861-865, 957-976
- **b-phipsi:** 0.004367
- **w-rdist:** 0.509383
- **t-alpha:** 0.009605
- **Chemical similarity (Tanimoto Index) (%):** 100.0
- **1D identity (%) [PDB]:** 85.91
- **1D identity (%) [Gaps excluded][PDB]:** 94.08
- **1D identity - Alignment Gaps [PDB]:** 93
- **2D identity (%) [PDB]:** 68.41
- **2D identity (%) [Gaps excluded][PDB]:** 87.14
- **2D identity - Alignment Gaps [PDB]:** 247
- **3D similarity (TM-Score) (%) [PDB]:** 88.88

- **Gene name:** S
- **RefSeq ID:** N/A
- **Sequence length:** N/A
- **5-UTR|CDS|3-UTR identity (%):** N/A | N/A | N/A
- **5-UTR|CDS|3-UTR identity (%) [Gaps excluded]:** N/A | N/A | N/A
- **5-UTR|CDS|3-UTR identity [Alignment Gaps]:** N/A | N/A | N/A

**Uniprot Description:**  
  
Spike protein S1: attaches the virion to the cell membrane by interacting with host receptor, initiating the infection.  
  
Homotrimer; each monomer consists of a S1 and a S2 subunit. The resulting peplomers protrude from the virus surface as spikes.  
  
**Gene Ontology Information:**

Molecular Function  
  
N/A

Location

- host cell plasma membrane
- integral component of membrane
- viral envelope
- virion membrane

Biological process

- endocytosis involved in viral entry into host cell
- fusion of virus membrane with host endosome membrane
- fusion of virus membrane with host plasma membrane
- pathogenesis
- receptor-mediated virion attachment to host cell

---

2

- **Protein name:** Capsid protein VP1
- **Organism:** Norwalk virus (strain GI/Human/United States/Norwalk/1968)
- **Uniprot Accession Number:** Q83884
- **Protein sequence length:** 530 aa
- **1D identity (%):** 8.86
- **1D identity (%) [Gaps excluded]:** 29.64
- **1D identity - Alignment Gaps:** 973
- **Common reported functions (%):** 50.0
- **Common reported locations (%):** 0.0
- **Common reported processes (%):** 0.0

- **PDB ID:** 6OUT
- **Chain:** C
- **Crystallized protein length:** 491 aa
- **Resolution:** 2.6 Å
- **Alinged residues range:** 459-461, 80-84, 457-461, 369-374, 369-379, 44-46
- **Aligned to segment part (indices):** 2, 4, 1, 6, 0, 5
- **Alinged residues range of reference:** 48-50, 226-230, 275-279, 594-599, 719-732, 970-972
- **b-phipsi:** 0.002341
- **w-rdist:** 3.509484
- **t-alpha:** 0.002114
- **Chemical similarity (Tanimoto Index) (%):** 83.9
- **1D identity (%) [PDB]:** 0.07
- **1D identity (%) [Gaps excluded][PDB]:** 100.0
- **1D identity - Alignment Gaps [PDB]:** 1472
- **2D identity (%) [PDB]:** 24.83
- **2D identity (%) [Gaps excluded][PDB]:** 86.06
- **2D identity - Alignment Gaps [PDB]:** 814
- **3D similarity (TM-Score) (%) [PDB]:** 14.53

- **Gene name:** ORF2
- **RefSeq ID:** NC\_001959
- **Genomic sequence length:** 7654
- **5-UTR|CDS|3-UTR identity (%):** 1.51 | 27.74 | 17.28
- **5-UTR|CDS|3-UTR identity (%) [Gaps excluded]:** 100.0 | 81.03 | 80.77
- **5-UTR|CDS|3-UTR identity [Alignment Gaps]:** 261 | 2653 | 191

**Uniprot Description:**  
  
Capsid protein self assembles to form an icosahedral capsid with a T=3 symmetry, about 38 nm in diameter, and consisting of 180 capsid proteins. A smaller form of capsid with a diameter of 23 nm might be capsid proteins assembled as icosahedron with T=1 symmetry. The capsid encapsulate the genomic RNA and VP2 proteins. Attaches virion to target cells by binding histo-blood group antigens present on gastroduodenal epithelial cells.  
  
Homodimerizes, then multimerizes. Binds to histo-blood group antigens at surface of target cells.  
  
**Gene Ontology Information:**

Molecular Function

- identical protein binding

Location

- host cell cytoplasm
- T=3 icosahedral viral capsid

Biological process  
  
N/A

---

3

- **Protein name:** Spike glycoprotein
- **Organism:** Severe acute respiratory syndrome coronavirus
- **Uniprot Accession Number:** P59594
- **Protein sequence length:** 1255 aa
- **1D identity (%):** 76.35
- **1D identity (%) [Gaps excluded]:** 77.94
- **1D identity - Alignment Gaps:** 26
- **Common reported functions (%):** 100.0
- **Common reported locations (%):** 62.5
- **Common reported processes (%):** 90.0

- **PDB ID:** 6CRX
- **Chain:** C
- **Crystallized protein length:** 1069 aa
- **Resolution:** 3.9 Å
- **Alinged residues range:** 42-57, 254-260, 261-389, 513-600, 634-768, 844-847, 939-965
- **Aligned to segment part (indices):** 2, 4, 1, 6, 0, 3, 5
- **Alinged residues range of reference:** 38-53, 203-209, 274-402, 527-614, 648-786, 862-865, 957-983
- **b-phipsi:** 0.007036
- **w-rdist:** 1.197943
- **t-alpha:** 0.020496
- **Chemical similarity (Tanimoto Index) (%):** 94.56
- **1D identity (%) [PDB]:** 65.83
- **1D identity (%) [Gaps excluded][PDB]:** 78.3
- **1D identity - Alignment Gaps [PDB]:** 178
- **2D identity (%) [PDB]:** 65.12
- **2D identity (%) [Gaps excluded][PDB]:** 84.79
- **2D identity - Alignment Gaps [PDB]:** 270
- **3D similarity (TM-Score) (%) [PDB]:** 80.36

- **Gene name:** S
- **RefSeq ID:** NC\_004718
- **Genomic sequence length:** 29751
- **5-UTR|CDS|3-UTR identity (%):** 88.52 | 73.15 | 22.38
- **5-UTR|CDS|3-UTR identity (%) [Gaps excluded]:** 92.28 | 78.79 | 98.18
- **5-UTR|CDS|3-UTR identity [Alignment Gaps]:** 11 | 282 | 745

**Uniprot Description:**  
  
Spike glycoprotein
May down-regulate host tetherin (BST2) by lysosomal degradation, thereby counteracting its antiviral activity.  
  
Homotrimer; each monomer consists of a S1 and a S2 subunit. The resulting peplomers protrude from the virus surface as spikes (By similarity). Binds to human and palm civet ACE2 and human CLEC4M/DC-SIGNR. Interacts with the accessory proteins 3a and 7a.  
  
**Gene Ontology Information:**

Molecular Function

- host cell surface receptor binding
- identical protein binding

Location

- host cell endoplasmic reticulum-Golgi intermediate compartment membrane
- host cell plasma membrane
- integral component of membrane
- viral envelope
- virion membrane

Biological process

- endocytosis involved in viral entry into host cell
- fusion of virus membrane with host endosome membrane
- fusion of virus membrane with host plasma membrane
- pathogenesis
- receptor-mediated virion attachment to host cell
- suppression by virus of host tetherin activity
- suppression by virus of host type I interferon-mediated signaling pathway
- viral protein processing
- viral translation

---

4

- **Protein name:** Tail knob protein gp9
- **Organism:** Bacillus phage phi29
- **Uniprot Accession Number:** P04331
- **Protein sequence length:** 599 aa
- **1D identity (%):** 9.24
- **1D identity (%) [Gaps excluded]:** 28.85
- **1D identity - Alignment Gaps:** 964
- **Common reported functions (%):** 0.0
- **Common reported locations (%):** 0.0
- **Common reported processes (%):** 0.0

- **PDB ID:** 5FB4
- **Chain:** A
- **Crystallized protein length:** 511 aa
- **Resolution:** 2.04 Å
- **Alinged residues range:** 311-314, 209-213, 126-130, 511-514, 567-573, 570-573
- **Aligned to segment part (indices):** 2, 4, 1, 6, 0, 5
- **Alinged residues range of reference:** 49-52, 204-208, 399-403, 595-598, 775-781, 977-980
- **b-phipsi:** 0.001817
- **w-rdist:** 2.853018
- **t-alpha:** 0.036145
- **Chemical similarity (Tanimoto Index) (%):** 83.52
- **1D identity (%) [PDB]:** 0.0
- **1D identity (%) [Gaps excluded][PDB]:** 0.0
- **1D identity - Alignment Gaps [PDB]:** 1495
- **2D identity (%) [PDB]:** 27.07
- **2D identity (%) [Gaps excluded][PDB]:** 88.57
- **2D identity - Alignment Gaps [PDB]:** 795
- **3D similarity (TM-Score) (%) [PDB]:** 13.52

- **Gene name:** 9
- **RefSeq ID:** NC\_011048
- **Genomic sequence length:** 19282
- **5-UTR|CDS|3-UTR identity (%):** N/A | 30.88 | N/A
- **5-UTR|CDS|3-UTR identity (%) [Gaps excluded]:** N/A | 81.04 | N/A
- **5-UTR|CDS|3-UTR identity [Alignment Gaps]:** N/A | 2520 | N/A

**Uniprot Description:**  
  
Distal (knob) tail protein that plugs the end of the tube before DNA ejection and forms a channel perforating the host membrane during ejection.  
  
Homohexamer; forms a hexameric tube structure with six flexible hydrophobic loops.  
  
**Gene Ontology Information:**

Molecular Function  
  
N/A

Location

- virus tail
- virus tail, tube

Biological process

- pore-mediated entry of viral genome into host cell
- viral genome ejection through host cell envelope, short tail mechanism

---

5

- **Protein name:** Capsid protein VP1
- **Organism:** Porcine parvovirus (strain NADL-2)
- **Uniprot Accession Number:** P18546
- **Protein sequence length:** 729 aa
- **1D identity (%):** 9.62
- **1D identity (%) [Gaps excluded]:** 26.26
- **1D identity - Alignment Gaps:** 928
- **Common reported functions (%):** 0.0
- **Common reported locations (%):** 0.0
- **Common reported processes (%):** 0.0

- **PDB ID:** 1K3V
- **Chain:** A
- **Crystallized protein length:** 542 aa
- **Resolution:** 3.5 Å
- **Alinged residues range:** 163-167, 519-524, 152-156, 228-235, 519-527, 413-415
- **Aligned to segment part (indices):** 2, 4, 1, 6, 0, 5
- **Alinged residues range of reference:** 49-53, 204-209, 311-315, 528-535, 715-724, 969-971
- **b-phipsi:** 0.004913
- **w-rdist:** 4.359013
- **t-alpha:** 0.011765
- **Chemical similarity (Tanimoto Index) (%):** 83.35
- **1D identity (%) [PDB]:** 0.07
- **1D identity (%) [Gaps excluded][PDB]:** 50.0
- **1D identity - Alignment Gaps [PDB]:** 1521
- **2D identity (%) [PDB]:** 25.54
- **2D identity (%) [Gaps excluded][PDB]:** 83.24
- **2D identity - Alignment Gaps [PDB]:** 809
- **3D similarity (TM-Score) (%) [PDB]:** 13.59

- **Gene name:** N/A
- **RefSeq ID:** NC\_001718
- **Genomic sequence length:** 5075
- **5-UTR|CDS|3-UTR identity (%):** N/A | 31.64 | N/A
- **5-UTR|CDS|3-UTR identity (%) [Gaps excluded]:** N/A | 80.45 | N/A
- **5-UTR|CDS|3-UTR identity [Alignment Gaps]:** N/A | 2422 | N/A

**Uniprot Description:**  
  
Capsid protein self-assembles to form an icosahedral capsid with a T=1 symmetry, about 22 nm in diameter, and consisting of 60 copies of two size variants of the capsid proteins, VP1 and VP2, which differ by the presence of an N-terminal extension in the minor protein VP1. The capsid encapsulates the genomic ssDNA. Capsid proteins are responsible for the attachment to host cell receptors. This attachment induces virion internalization predominantly through clathrin-dependent endocytosis. Binding to the host receptors also induces capsid rearrangements leading to surface exposure of VP1 N-terminus, specifically its phospholipase A2-like region and putative nuclear localization signal(s). VP1 N-terminus might serve as a lipolytic enzyme to breach the endosomal membrane during entry into host cell and might contribute to virus transport to the nucleus (By similarity).  
  
**Gene Ontology Information:**

Molecular Function

- metal ion binding
- structural molecule activity

Location

- host cell nucleus
- T=1 icosahedral viral capsid

Biological process

- clathrin-dependent endocytosis of virus by host cell
- microtubule-dependent intracellular transport of viral material towards nucleus
- permeabilization of host organelle membrane involved in viral entry into host cell
- viral entry via permeabilization of inner membrane
- viral penetration into host nucleus
- virion attachment to host cell

---

6

- **Protein name:** Matrix remodeling-associated protein 8
- **Organism:** Mus musculus
- **Uniprot Accession Number:** Q9DBV4
- **Protein sequence length:** 442 aa
- **1D identity (%):** 3.78
- **1D identity (%) [Gaps excluded]:** 24.03
- **1D identity - Alignment Gaps:** 1249
- **Common reported functions (%):** 0.0
- **Common reported locations (%):** 12.5
- **Common reported processes (%):** 0.0

- **PDB ID:** 6NK6
- **Chain:** N
- **Crystallized protein length:** 261 aa
- **Resolution:** 4.06 Å
- **Alinged residues range:** 269-272, 268-272, 36-39, 267-273
- **Aligned to segment part (indices):** 2, 4, 1, 0
- **Alinged residues range of reference:** 50-53, 203-207, 312-315, 721-727
- **b-phipsi:** 0.003706
- **w-rdist:** 1.913864
- **t-alpha:** 0.319386
- **Chemical similarity (Tanimoto Index) (%):** 99.55
- **1D identity (%) [PDB]:** 0.08
- **1D identity (%) [Gaps excluded][PDB]:** 100.0
- **1D identity - Alignment Gaps [PDB]:** 1242
- **2D identity (%) [PDB]:** 15.83
- **2D identity (%) [Gaps excluded][PDB]:** 88.36
- **2D identity - Alignment Gaps [PDB]:** 866
- **3D similarity (TM-Score) (%) [PDB]:** 15.47

- **Gene name:** Mxra8
- **RefSeq ID:** N/A
- **Sequence length:** N/A
- **5-UTR|CDS|3-UTR identity (%):** N/A | N/A | N/A
- **5-UTR|CDS|3-UTR identity (%) [Gaps excluded]:** N/A | N/A | N/A
- **5-UTR|CDS|3-UTR identity [Alignment Gaps]:** N/A | N/A | N/A

**Uniprot Description:**  
  
Transmembrane protein which can modulate activity of various signaling pathways, probably via binding to integrin ITGAV:ITGB3 (PubMed:18366072, PubMed:22492581, PubMed:29702220). Mediates heterophilic cell-cell interactions in vitro (PubMed:18366072). Inhibits osteoclastogenesis downstream of TNFSF11/RANKL and CSF1, where it may function by attenuating signaling via integrin ITGB3 and MAP kinase p38 (PubMed:22492581). Plays a role in cartilage formation where it promotes proliferation and maturation of growth plate chondrocytes (PubMed:29702220). Stimulates formation of primary cilia in chondrocytes (PubMed:29702220). Enhances expression of genes involved in the hedgehog signaling pathway in chondrocytes, including the hedgehog signaling molecule IHH; may also promote signaling via the PTHLH/PTHrP pathway (PubMed:29702220). Plays a role in angiogenesis where it suppresses migration of endothelial cells and also promotes their apoptosis (By similarity). Inhibits VEGF-induced activation of AKT and p38 MAP kinase in endothelial cells (By similarity). Also inhibits VTN (vitronectin)-mediated integrin ITGAV:ITGB3 signaling and activation of PTK2/FAK (By similarity). May play a role in the maturation and maintenance of the blood-brain barrier (PubMed:14603461).  
  
Homodimer in cis (PubMed:18366072). Does not appear to form trans-homodimers (PubMed:18366072). Interacts with ITGB3; the interaction inhibits ITGAV:ITGB3 heterodimer formation (PubMed:22492581).  
  
**Gene Ontology Information:**

Molecular Function  
  
N/A

Location

- bicellular tight junction
- cell surface
- ciliary membrane
- cytoplasm
- integral component of membrane
- nucleus

Biological process

- cell adhesion
- establishment of glial blood-brain barrier

---

7

- **Protein name:** Gag-Pol polyprotein
- **Organism:** Human immunodeficiency virus type 1 group M subtype B (isolate HXB2)
- **Uniprot Accession Number:** P04585
- **Protein sequence length:** 1435 aa
- **1D identity (%):** 12.98
- **1D identity (%) [Gaps excluded]:** 28.67
- **1D identity - Alignment Gaps:** 1020
- **Common reported functions (%):** 50.0
- **Common reported locations (%):** 25.0
- **Common reported processes (%):** 10.0

- **PDB ID:** 1LW2
- **Chain:** B
- **Crystallized protein length:** 406 aa
- **Resolution:** 3.0 Å
- **Alinged residues range:** 349-353, 338-352, 412-415, 334-341, 254-259, 32-36
- **Aligned to segment part (indices):** 2, 4, 1, 6, 0, 5
- **Alinged residues range of reference:** 48-52, 200-209, 447-450, 544-551, 772-777, 960-964
- **b-phipsi:** 0.020324
- **w-rdist:** 3.058551
- **t-alpha:** 0.028261
- **Chemical similarity (Tanimoto Index) (%):** 83.58
- **1D identity (%) [PDB]:** 0.0
- **1D identity (%) [Gaps excluded][PDB]:** 0.0
- **1D identity - Alignment Gaps [PDB]:** 1392
- **2D identity (%) [PDB]:** 18.36
- **2D identity (%) [Gaps excluded][PDB]:** 86.83
- **2D identity - Alignment Gaps [PDB]:** 906
- **3D similarity (TM-Score) (%) [PDB]:** 12.83

- **Gene name:** gag-pol
- **RefSeq ID:** NC\_001802
- **Genomic sequence length:** 9181
- **5-UTR|CDS|3-UTR identity (%):** 23.6 | 40.46 | 23.63
- **5-UTR|CDS|3-UTR identity (%) [Gaps excluded]:** 75.9 | 77.79 | 80.65
- **5-UTR|CDS|3-UTR identity [Alignment Gaps]:** 184 | 2566 | 374

**Uniprot Description:**  
  
Gag-Pol polyprotein
Mediates, with Gag polyprotein, the essential events in virion assembly, including binding the plasma membrane, making the protein-protein interactions necessary to create spherical particles, recruiting the viral Env proteins, and packaging the genomic RNA via direct interactions with the RNA packaging sequence (Psi). Gag-Pol polyprotein may regulate its own translation, by the binding genomic RNA in the 5'-UTR. At low concentration, the polyprotein would promote translation, whereas at high concentration, the polyprotein would encapsidate genomic RNA and then shut off translation.  
  
Matrix protein p17
Homotrimer; further assembles as hexamers of trimers (PubMed:19327811). Interacts with gp41 (via C-terminus) (By similarity). Interacts with host CALM1; this interaction induces a conformational change in the Matrix protein, triggering exposure of the myristate group (PubMed:24500712). Interacts with host AP3D1; this interaction allows the polyprotein trafficking to multivesicular bodies during virus assembly (By similarity). Part of the pre-integration complex (PIC) which is composed of viral genome, matrix protein, Vpr and integrase (By similarity).  
  
**Gene Ontology Information:**

Molecular Function

- aspartic-type endopeptidase activity
- DNA binding
- DNA-directed DNA polymerase activity
- exoribonuclease H activity
- identical protein binding
- lipid binding
- RNA binding
- RNA-directed DNA polymerase activity
- RNA-DNA hybrid ribonuclease activity
- structural molecule activity
- zinc ion binding

Location

- host cell nucleus
- host cell plasma membrane
- host multivesicular body
- viral nucleocapsid
- virion membrane

Biological process

- DNA integration
- DNA recombination
- entry into host
- establishment of integrated proviral latency
- fusion of virus membrane with host plasma membrane
- induction by virus of host cysteine-type endopeptidase activity involved in apoptotic process
- RNA-dependent DNA biosynthetic process
- suppression by virus of host gene expression
- uncoating of virus
- viral genome integration into host DNA
- viral genome packaging
- viral life cycle
- viral penetration into host nucleus
- virion assembly

---

8

- **Protein name:** Hemagglutinin
- **Organism:** Influenza A virus (A/environment/Hong Kong/437-6/99 (H5N1))
- **Uniprot Accession Number:** Q9EA62
- **Protein sequence length:** 568 aa
- **1D identity (%):** 8.63
- **1D identity (%) [Gaps excluded]:** 25.76
- **1D identity - Alignment Gaps:** 917
- **Common reported functions (%):** 50.0
- **Common reported locations (%):** 50.0
- **Common reported processes (%):** 20.0

- **PDB ID:** 3S11
- **Chain:** C
- **Crystallized protein length:** 321 aa
- **Resolution:** 2.5 Å
- **Alinged residues range:** 274-277, 274-279, 258-261, 242-246, 13-17, 108-114
- **Aligned to segment part (indices):** 2, 4, 1, 6, 0, 5
- **Alinged residues range of reference:** 50-53, 204-209, 431-434, 538-542, 720-724, 977-983
- **b-phipsi:** 0.001109
- **w-rdist:** 3.612615
- **t-alpha:** 0.299451
- **Chemical similarity (Tanimoto Index) (%):** N/A
- **1D identity (%) [PDB]:** 0.0
- **1D identity (%) [Gaps excluded][PDB]:** 0.0
- **1D identity - Alignment Gaps [PDB]:** 1296
- **2D identity (%) [PDB]:** 19.43
- **2D identity (%) [Gaps excluded][PDB]:** 82.93
- **2D identity - Alignment Gaps [PDB]:** 804
- **3D similarity (TM-Score) (%) [PDB]:** 13.38

- **Gene name:** HA
- **RefSeq ID:** N/A
- **Sequence length:** N/A
- **5-UTR|CDS|3-UTR identity (%):** N/A | N/A | N/A
- **5-UTR|CDS|3-UTR identity (%) [Gaps excluded]:** N/A | N/A | N/A
- **5-UTR|CDS|3-UTR identity [Alignment Gaps]:** N/A | N/A | N/A

**Uniprot Description:**  
  
Binds to sialic acid-containing receptors on the cell surface, bringing about the attachment of the virus particle to the cell. This attachment induces virion internalization either through clathrin-dependent endocytosis or through clathrin- and caveolin-independent pathway. Plays a major role in the determination of host range restriction and virulence. Class I viral fusion protein. Responsible for penetration of the virus into the cell cytoplasm by mediating the fusion of the membrane of the endocytosed virus particle with the endosomal membrane. Low pH in endosomes induces an irreversible conformational change in HA2, releasing the fusion hydrophobic peptide. Several trimers are required to form a competent fusion pore.  
  
Homotrimer of disulfide-linked HA1-HA2.  
  
**Gene Ontology Information:**

Molecular Function

- host cell surface receptor binding

Location

- host cell plasma membrane
- integral component of membrane
- viral envelope
- virion membrane

Biological process

- clathrin-dependent endocytosis of virus by host cell
- fusion of virus membrane with host endosome membrane
- fusion of virus membrane with host plasma membrane
- viral budding from plasma membrane
- virion attachment to host cell

---

9

- **Protein name:** Major capsid protein L1
- **Organism:** Human papillomavirus type 18
- **Uniprot Accession Number:** Q5G244
- **Protein sequence length:** 535 aa
- **1D identity (%):** 7.85
- **1D identity (%) [Gaps excluded]:** 30.62
- **1D identity - Alignment Gaps:** 1070
- **Common reported functions (%):** 0.0
- **Common reported locations (%):** 0.0
- **Common reported processes (%):** 10.0

- **PDB ID:** 5W1X
- **Chain:** G
- **Crystallized protein length:** 422 aa
- **Resolution:** 3.37 Å
- **Alinged residues range:** 363-367, 362-365, 165-170, 27-36, 32-38, 468-472
- **Aligned to segment part (indices):** 2, 4, 1, 6, 0, 5
- **Alinged residues range of reference:** 49-53, 206-209, 395-400, 545-554, 711-719, 960-964
- **b-phipsi:** 0.001212
- **w-rdist:** 4.354399
- **t-alpha:** 0.200508
- **Chemical similarity (Tanimoto Index) (%):** N/A
- **1D identity (%) [PDB]:** 0.07
- **1D identity (%) [Gaps excluded][PDB]:** 50.0
- **1D identity - Alignment Gaps [PDB]:** 1402
- **2D identity (%) [PDB]:** 25.23
- **2D identity (%) [Gaps excluded][PDB]:** 85.62
- **2D identity - Alignment Gaps [PDB]:** 766
- **3D similarity (TM-Score) (%) [PDB]:** 14.58

- **Gene name:** L1
- **RefSeq ID:** N/A
- **Sequence length:** N/A
- **5-UTR|CDS|3-UTR identity (%):** N/A | N/A | N/A
- **5-UTR|CDS|3-UTR identity (%) [Gaps excluded]:** N/A | N/A | N/A
- **5-UTR|CDS|3-UTR identity [Alignment Gaps]:** N/A | N/A | N/A

**Uniprot Description:**  
  
Forms an icosahedral capsid with a T=7 symmetry and a 50 nm diameter. The capsid is composed of 72 pentamers linked to each other by disulfide bonds and associated with L2 proteins. Binds to heparan sulfate proteoglycans on cell surface of basal layer keratinocytes to provide initial virion attachment. This binding mediates a conformational change in the virus capsid that facilitates efficient infection. The virion enters the host cell via endocytosis. During virus trafficking, L1 protein dissociates from the viral DNA and the genomic DNA is released to the host nucleus. The virion assembly takes place within the cell nucleus. Encapsulates the genomic DNA together with protein L2.  
  
Self-assembles into homopentamers. The capsid has an icosahedral symmetry and consists of 72 capsomers, with each capsomer being a pentamer of L1. Interacts with the minor capsid protein L2; this interaction is necessary for viral genome encapsidation.  
  
**Gene Ontology Information:**

Molecular Function

- structural molecule activity

Location

- host cell nucleus
- T=7 icosahedral viral capsid

Biological process

- endocytosis involved in viral entry into host cell
- virion attachment to host cell

---

10

- **Protein name:** Morphogenesis protein 1
- **Organism:** Bacillus phage phi29
- **Uniprot Accession Number:** P15132
- **Protein sequence length:** 365 aa
- **1D identity (%):** 7.56
- **1D identity (%) [Gaps excluded]:** 30.18
- **1D identity - Alignment Gaps:** 982
- **Common reported functions (%):** 0.0
- **Common reported locations (%):** 0.0
- **Common reported processes (%):** 0.0

- **PDB ID:** 3CSQ
- **Chain:** C
- **Crystallized protein length:** 324 aa
- **Resolution:** 1.8 Å
- **Alinged residues range:** 292-294, 281-294, 179-183, 318-325, 92-96, 22-25
- **Aligned to segment part (indices):** 2, 4, 1, 6, 0, 5
- **Alinged residues range of reference:** 50-52, 201-206, 436-440, 597-606, 761-765, 959-962
- **b-phipsi:** 0.013268
- **w-rdist:** 2.130156
- **t-alpha:** 0.223803
- **Chemical similarity (Tanimoto Index) (%):** 79.8
- **1D identity (%) [PDB]:** 0.0
- **1D identity (%) [Gaps excluded][PDB]:** 0.0
- **1D identity - Alignment Gaps [PDB]:** 1309
- **2D identity (%) [PDB]:** 14.54
- **2D identity (%) [Gaps excluded][PDB]:** 90.61
- **2D identity - Alignment Gaps [PDB]:** 947
- **3D similarity (TM-Score) (%) [PDB]:** 13.18

- **Gene name:** 13
- **RefSeq ID:** NC\_011048
- **Genomic sequence length:** 19282
- **5-UTR|CDS|3-UTR identity (%):** N/A | 18.76 | N/A
- **5-UTR|CDS|3-UTR identity (%) [Gaps excluded]:** N/A | 78.23 | N/A
- **5-UTR|CDS|3-UTR identity [Alignment Gaps]:** N/A | 3015 | N/A

**Uniprot Description:**  
  
May serve as a plug to restrain the highly pressurized packaged genome and thus would be the first virion protein to contact the host cell wall, degrading the peptidoglycan layer and thereby facilitating viral genome entry into the host bacteria. Acts probably as a multifunctional enzyme that degrades N-acetylglucosamine polymers (in vitro) and cleaves the peptide cross-links of the host cell wall. Essential for the tail assembly.  
  
**Gene Ontology Information:**

Molecular Function

- hydrolase activity, acting on glycosyl bonds
- metal ion binding
- metallopeptidase activity

Location

- virus tail, tip

Biological process

- cell wall organization
- cytolysis
- defense response to bacterium
- disruption by virus of host cell wall peptidoglycan during virus entry
- viral genome ejection through host cell envelope, short tail mechanism
- virus tail fiber assembly

---

11

- **Protein name:** Putative capsid protein V20
- **Organism:** Sputnik virophage
- **Uniprot Accession Number:** B4YNG0
- **Protein sequence length:** 595 aa
- **1D identity (%):** 6.98
- **1D identity (%) [Gaps excluded]:** 32.04
- **1D identity - Alignment Gaps:** 1200
- **Common reported functions (%):** 0.0
- **Common reported locations (%):** 0.0
- **Common reported processes (%):** 0.0

- **PDB ID:** 3J26
- **Chain:** M
- **Crystallized protein length:** 508 aa
- **Resolution:** 3.5 Å
- **Alinged residues range:** 465-469, 70-76, 465-469, 293-302, 458-473, 487-490
- **Aligned to segment part (indices):** 2, 4, 1, 6, 0, 5
- **Alinged residues range of reference:** 47-51, 202-208, 312-316, 546-557, 716-728, 978-981
- **b-phipsi:** 0.001207
- **w-rdist:** 6.509736
- **t-alpha:** 0.116883
- **Chemical similarity (Tanimoto Index) (%):** N/A
- **1D identity (%) [PDB]:** 0.07
- **1D identity (%) [Gaps excluded][PDB]:** 50.0
- **1D identity - Alignment Gaps [PDB]:** 1487
- **2D identity (%) [PDB]:** 26.18
- **2D identity (%) [Gaps excluded][PDB]:** 85.67
- **2D identity - Alignment Gaps [PDB]:** 793
- **3D similarity (TM-Score) (%) [PDB]:** 13.74

- **Gene name:** ORF20
- **RefSeq ID:** NC\_011132
- **Genomic sequence length:** 18343
- **5-UTR|CDS|3-UTR identity (%):** N/A | 32.62 | N/A
- **5-UTR|CDS|3-UTR identity (%) [Gaps excluded]:** N/A | 80.84 | N/A
- **5-UTR|CDS|3-UTR identity [Alignment Gaps]:** N/A | 2384 | N/A

**Uniprot Description:**  
  
May self assemble to form an icosahedral capsid. Most abundant protein in the virion.  
  
**Gene Ontology Information:**

Molecular Function  
  
N/A

Location

- viral capsid

Biological process  
  
N/A

---

12

- **Protein name:** Tail spike protein
- **Organism:** Enterobacteria phage HK620
- **Uniprot Accession Number:** Q9AYY6
- **Protein sequence length:** 710 aa
- **1D identity (%):** 10.6
- **1D identity (%) [Gaps excluded]:** 27.06
- **1D identity - Alignment Gaps:** 867
- **Common reported functions (%):** 0.0
- **Common reported locations (%):** 0.0
- **Common reported processes (%):** 0.0

- **PDB ID:** 2X85
- **Chain:** A
- **Crystallized protein length:** 597 aa
- **Resolution:** 1.5 Å
- **Alinged residues range:** 689-691, 634-637, 654-660, 341-345, 686-691, 144-146
- **Aligned to segment part (indices):** 2, 4, 1, 6, 0, 5
- **Alinged residues range of reference:** 50-52, 227-230, 371-377, 593-597, 720-725, 979-981
- **b-phipsi:** 0.004855
- **w-rdist:** 3.881277
- **t-alpha:** 0.060538
- **Chemical similarity (Tanimoto Index) (%):** 80.3
- **1D identity (%) [PDB]:** 0.0
- **1D identity (%) [Gaps excluded][PDB]:** 0.0
- **1D identity - Alignment Gaps [PDB]:** 1580
- **2D identity (%) [PDB]:** 27.17
- **2D identity (%) [Gaps excluded][PDB]:** 82.61
- **2D identity - Alignment Gaps [PDB]:** 798
- **3D similarity (TM-Score) (%) [PDB]:** 16.93

- **Gene name:** 9
- **RefSeq ID:** NC\_002730
- **Genomic sequence length:** 38297
- **5-UTR|CDS|3-UTR identity (%):** N/A | 35.1 | N/A
- **5-UTR|CDS|3-UTR identity (%) [Gaps excluded]:** N/A | 79.68 | N/A
- **5-UTR|CDS|3-UTR identity [Alignment Gaps]:** N/A | 2313 | N/A

**Uniprot Description:**  
  
N/A  
  
**Gene Ontology Information:**

Molecular Function  
  
N/A

Location

- virus tail, fiber

Biological process

- disruption of host cell envelope during viral entry
- virion attachment to host cell

---

13

- **Protein name:** Terminase, large subunit
- **Organism:** Enterobacteria phage T4
- **Uniprot Accession Number:** P17312
- **Protein sequence length:** 610 aa
- **1D identity (%):** 9.69
- **1D identity (%) [Gaps excluded]:** 31.03
- **1D identity - Alignment Gaps:** 987
- **Common reported functions (%):** 0.0
- **Common reported locations (%):** 0.0
- **Common reported processes (%):** 0.0

- **PDB ID:** 3CPE
- **Chain:** A
- **Crystallized protein length:** 553 aa
- **Resolution:** 2.8 Å
- **Alinged residues range:** 428-431, 423-431, 42-46, 537-541, 106-112, 503-508
- **Aligned to segment part (indices):** 2, 4, 1, 6, 0, 5
- **Alinged residues range of reference:** 47-50, 200-208, 420-424, 603-607, 771-777, 959-964
- **b-phipsi:** 0.023836
- **w-rdist:** 3.865522
- **t-alpha:** 0.028261
- **Chemical similarity (Tanimoto Index) (%):** 83.66
- **1D identity (%) [PDB]:** 0.07
- **1D identity (%) [Gaps excluded][PDB]:** 50.0
- **1D identity - Alignment Gaps [PDB]:** 1532
- **2D identity (%) [PDB]:** 20.88
- **2D identity (%) [Gaps excluded][PDB]:** 91.26
- **2D identity - Alignment Gaps [PDB]:** 964
- **3D similarity (TM-Score) (%) [PDB]:** 17.95

- **Gene name:** 17
- **RefSeq ID:** NC\_000866
- **Genomic sequence length:** 168903
- **5-UTR|CDS|3-UTR identity (%):** N/A | 26.54 | N/A
- **5-UTR|CDS|3-UTR identity (%) [Gaps excluded]:** N/A | 79.63 | N/A
- **5-UTR|CDS|3-UTR identity [Alignment Gaps]:** N/A | 2670 | N/A

**Uniprot Description:**  
  
Isoform Terminase large subunit
The terminase large subunit acts as an ATP driven molecular motor necessary for viral DNA translocation into empty capsids and as an endonuclease that cuts the viral genome to initiate and to end a packaging reaction (PubMed:11846554, PubMed:10967092, PubMed:12466275). The terminase lies at a unique vertex of the procapsid and is composed of two subunits, a small terminase subunit involved in viral DNA recognition (packaging sequence), and a large terminase subunit possessing endonucleolytic and ATPase activities (PubMed:12466275). Both terminase subunits heterooligomerize and are docked on the portal protein to form the packaging machine. The terminase large subunit exhibits endonuclease activity and cleaves the viral genome concatemer once the capsid is full (headful packaging) (PubMed:8063105, PubMed:10967092, PubMed:12466275). Once the capsid is packaged with the DNA, the terminase complex is substituted by the tail.  
  
Isoform Terminase large subunit
Interacts with the terminase small subunit; the active complex is composed of a pentamer of terminase large subunits and a dodecamer of terminase small subunits (PubMed:12466275, PubMed:17386269). Interacts with the portal protein (PubMed:10366503, PubMed:22345478). Interacts with the RNA polymerase sigma factor gp55 (By similarity) (PubMed:12051907).  
  
**Gene Ontology Information:**

Molecular Function

- ATP binding
- ATPase activity
- deoxyribonuclease activity
- endonuclease activity
- metal ion binding
- nuclease activity

Location

- viral terminase, large subunit

Biological process

- DNA packaging
- viral DNA genome packaging
- viral genome packaging
- viral procapsid maturation

---

14

- **Protein name:** RNA-directed RNA polymerase
- **Organism:** Pseudomonas phage phi6
- **Uniprot Accession Number:** P11124
- **Protein sequence length:** 665 aa
- **1D identity (%):** 9.98
- **1D identity (%) [Gaps excluded]:** 27.57
- **1D identity - Alignment Gaps:** 908
- **Common reported functions (%):** 0.0
- **Common reported locations (%):** 0.0
- **Common reported processes (%):** 0.0

- **PDB ID:** 1HI8
- **Chain:** A
- **Crystallized protein length:** 639 aa
- **Resolution:** 2.5 Å
- **Alinged residues range:** 38-40, 38-40, 625-632, 200-203, 309-313, 305-310
- **Aligned to segment part (indices):** 2, 4, 1, 6, 0, 5
- **Alinged residues range of reference:** 49-51, 207-209, 416-423, 596-599, 773-777, 974-979
- **b-phipsi:** 0.03513
- **w-rdist:** 6.32481
- **t-alpha:** 0.003181
- **Chemical similarity (Tanimoto Index) (%):** 82.07
- **1D identity (%) [PDB]:** 0.0
- **1D identity (%) [Gaps excluded][PDB]:** 0.0
- **1D identity - Alignment Gaps [PDB]:** 1647
- **2D identity (%) [PDB]:** 25.29
- **2D identity (%) [Gaps excluded][PDB]:** 88.52
- **2D identity - Alignment Gaps [PDB]:** 915
- **3D similarity (TM-Score) (%) [PDB]:** 17.61

- **Gene name:** P2
- **RefSeq ID:** NC\_003715
- **Genomic sequence length:** 6374
- **5-UTR|CDS|3-UTR identity (%):** N/A | 31.76 | N/A
- **5-UTR|CDS|3-UTR identity (%) [Gaps excluded]:** N/A | 77.29 | N/A
- **5-UTR|CDS|3-UTR identity [Alignment Gaps]:** N/A | 2430 | N/A

**Uniprot Description:**  
  
Rna-dependent RNA polymerase part of the packaging complex that packages the viral RNA segments, replicate them into a double-stranded form and transcribe them.  
  
Part of the packaging complex composed of RDRP, P4 and P7. Interacts with P7 (Probable).  
  
**Gene Ontology Information:**

Molecular Function

- metal ion binding
- nucleotide binding
- RNA binding
- RNA uridylyltransferase activity
- RNA-directed 5'-3' RNA polymerase activity

Location

- virion

Biological process

- transcription, DNA-templated
- viral RNA genome replication

---

15

- **Protein name:** Rhodopsin
- **Organism:** Homo sapiens
- **Uniprot Accession Number:** P08100
- **Protein sequence length:** 348 aa
- **1D identity (%):** 5.95
- **1D identity (%) [Gaps excluded]:** 33.74
- **1D identity - Alignment Gaps:** 1135
- **Common reported functions (%):** 0.0
- **Common reported locations (%):** 0.0
- **Common reported processes (%):** 0.0

- **PDB ID:** 5W0P
- **Chain:** D
- **Crystallized protein length:** 773 aa
- **Resolution:** 3.01 Å
- **Alinged residues range:** 2324-2329, 2236-2244, 2344-2351, 186-195, 2323-2330, 124-129
- **Aligned to segment part (indices):** 2, 4, 1, 6, 0, 5
- **Alinged residues range of reference:** 47-52, 195-207, 430-437, 573-583, 719-726, 958-963
- **b-phipsi:** 0.023433
- **w-rdist:** 6.732277
- **t-alpha:** 0.002114
- **Chemical similarity (Tanimoto Index) (%):** 94.16
- **1D identity (%) [PDB]:** 0.0
- **1D identity (%) [Gaps excluded][PDB]:** 0.0
- **1D identity - Alignment Gaps [PDB]:** 1673
- **2D identity (%) [PDB]:** N/A
- **2D identity (%) [Gaps excluded][PDB]:** N/A
- **2D identity - Alignment Gaps [PDB]:** N/A
- **3D similarity (TM-Score) (%) [PDB]:** 19.47

- **Gene name:** RHO
- **RefSeq ID:** NM\_000539
- **Transcript sequence length:** 2768
- **5-UTR|CDS|3-UTR identity (%):** 23.91 | 18.15 | 8.54
- **5-UTR|CDS|3-UTR identity (%) [Gaps excluded]:** 78.57 | 78.56 | 79.01
- **5-UTR|CDS|3-UTR identity [Alignment Gaps]:** 192 | 3041 | 1493

**Uniprot Description:**  
  
Photoreceptor required for image-forming vision at low light intensity (PubMed:8107847, PubMed:7846071). Required for photoreceptor cell viability after birth (PubMed:2215617, PubMed:12566452). Light-induced isomerization of the chromophore 11-cis-retinal to all-trans-retinal triggers a conformational change that activates signaling via G-proteins (PubMed:8107847, PubMed:28524165, PubMed:26200343, PubMed:28753425). Subsequent receptor phosphorylation mediates displacement of the bound G-protein alpha subunit by the arrestin SAG and terminates signaling (PubMed:28524165, PubMed:26200343).  
  
Homodimer (By similarity). May form a complex composed of RHO, GRK1 and RCVRN in a Ca(2+)-dependent manner; RCVRN prevents the interaction between GRK1 and RHO (By similarity). Interacts with GRK1 (PubMed:28524165). Interacts (phosphorylated form) with SAG (PubMed:28524165, PubMed:26200343, PubMed:28753425). Interacts with GNAT1 (PubMed:26200343). Interacts with GNAT3. SAG and G-proteins compete for a common binding site (PubMed:26200343). Interacts with PRCD; the interaction promotes PRCD stability (By similarity).  
  
**Gene Ontology Information:**

Molecular Function

- G protein-coupled receptor binding
- opsin binding
- phosphoprotein binding
- spectrin binding

Location

- membrane
- photoreceptor inner segment
- photoreceptor outer segment

Biological process

- G protein-coupled receptor internalization
- signal transduction

---

16

- **Protein name:** Integrin alpha-V
- **Organism:** Homo sapiens
- **Uniprot Accession Number:** P06756
- **Protein sequence length:** 1048 aa
- **1D identity (%):** 14.16
- **1D identity (%) [Gaps excluded]:** 27.5
- **1D identity - Alignment Gaps:** 743
- **Common reported functions (%):** 0.0
- **Common reported locations (%):** 0.0
- **Common reported processes (%):** 10.0

- **PDB ID:** 5NER
- **Chain:** A
- **Crystallized protein length:** 589 aa
- **Resolution:** 11.5 Å
- **Alinged residues range:** 327-330, 522-526, 407-411, 432-435, 522-528, 208-210, 385-388
- **Aligned to segment part (indices):** 2, 4, 1, 6, 0, 3, 5
- **Alinged residues range of reference:** 49-52, 205-209, 396-400, 609-612, 715-721, 861-863, 972-975
- **b-phipsi:** 0.019719
- **w-rdist:** 2.208929
- **t-alpha:** 0.23822
- **Chemical similarity (Tanimoto Index) (%):** N/A
- **1D identity (%) [PDB]:** 0.38
- **1D identity (%) [Gaps excluded][PDB]:** 85.71
- **1D identity - Alignment Gaps [PDB]:** 1561
- **2D identity (%) [PDB]:** 30.55
- **2D identity (%) [Gaps excluded][PDB]:** 85.96
- **2D identity - Alignment Gaps [PDB]:** 749
- **3D similarity (TM-Score) (%) [PDB]:** 9.09

- **Gene name:** ITGAV
- **RefSeq ID:** NM\_002210
- **Transcript sequence length:** 7039
- **5-UTR|CDS|3-UTR identity (%):** 19.91 | 41.96 | 4.69
- **5-UTR|CDS|3-UTR identity (%) [Gaps excluded]:** 78.38 | 78.18 | 79.81
- **5-UTR|CDS|3-UTR identity [Alignment Gaps]:** 326 | 2101 | 3412

**Uniprot Description:**  
  
The alpha-V (ITGAV) integrins are receptors for vitronectin, cytotactin, fibronectin, fibrinogen, laminin, matrix metalloproteinase-2, osteopontin, osteomodulin, prothrombin, thrombospondin and vWF. They recognize the sequence R-G-D in a wide array of ligands. ITGAV:ITGB3 binds to fractalkine (CX3CL1) and may act as its coreceptor in CX3CR1-dependent fractalkine signaling (PubMed:23125415). ITGAV:ITGB3 binds to NRG1 (via EGF domain) and this binding is essential for NRG1-ERBB signaling (PubMed:20682778). ITGAV:ITGB3 binds to FGF1 and this binding is essential for FGF1 signaling (PubMed:18441324). ITGAV:ITGB3 binds to FGF2 and this binding is essential for FGF2 signaling (PubMed:28302677). ITGAV:ITGB3 binds to IGF1 and this binding is essential for IGF1 signaling (PubMed:19578119). ITGAV:ITGB3 binds to IGF2 and this binding is essential for IGF2 signaling (PubMed:28873464). ITGAV:ITGB3 binds to IL1B and this binding is essential for IL1B signaling (PubMed:29030430). ITGAV:ITGB3 binds to PLA2G2A via a site (site 2) which is distinct from the classical ligand-binding site (site 1) and this induces integrin conformational changes and enhanced ligand binding to site 1 (PubMed:18635536, PubMed:25398877). ITGAV:ITGB3 and ITGAV:ITGB6 act as a receptor for fibrillin-1 (FBN1) and mediate R-G-D-dependent cell adhesion to FBN1 (PubMed:12807887, PubMed:17158881). Integrin alpha-V/beta-6 or alpha-V/beta-8 (ITGAV:ITGB6 or ITGAV:ITGB8) mediates R-G-D-dependent release of transforming growth factor beta-1 (TGF-beta-1) from regulatory Latency-associated peptide (LAP), thereby playing a key role in TGF-beta-1 activation (PubMed:15184403, PubMed:22278742, PubMed:28117447). ITGAV:ITGB3 act as a receptor for CD40LG (PubMed:31331973).  
  
Heterodimer of an alpha and a beta subunit. The alpha subunit is composed of a heavy and a light chain linked by a disulfide bond. Alpha-V (ITGAV) associates with either beta-1 (ITGB1), beta-3 (ITGB3), beta-5 (ITGB5), beta-6 (ITGB6) or beta-8 (ITGB8). Interacts with CIB1 (PubMed:24011356). Interacts with RAB25 (PubMed:17925226). Integrins ITGAV:ITGB3 and ITGAV:ITGB5 interact with FBLN5 (via N-terminus) (By similarity). ITGAV:ITGB3 and ITGAV:ITGB5 interact with CCN3 (PubMed:12695522). ITGAV:ITGB3 interacts with ADGRA2 (PubMed:16982628). ITGAV:ITGB3 interacts with FGF2; it is likely that FGF2 can simultaneously bind ITGAV:ITGB3 and FGF receptors (PubMed:28302677). ITGAV:ITGB3 interacts with IL1B (PubMed:29030430). ITGAV:ITGB3 is found in a ternary complex with CX3CR1 and CX3CL1 (PubMed:23125415). ITGAV:ITGB3 is found in a ternary complex with NRG1 and ERBB3 (PubMed:20682778). ITGAV:ITGB3 is found in a ternary complex with FGF1 and FGFR1 (PubMed:18441324). ITGAV:ITGB3 is found in a ternary complex with IGF1 and IGF1R (PubMed:19578119). ITGAV:ITGB3 interacts with IGF2 (PubMed:28873464). ITGAV:ITGB3 and ITGAV:ITGB6 interact with FBN1 (PubMed:12807887, PubMed:17158881). ITGAV:ITGB3 interacts with CD9, CD81 and CD151 (via second extracellular domain) (PubMed:27993971). ITGAV:ITGB6 interacts with TGFB1 (PubMed:22278742, PubMed:28117447). ITGAV:ITGB3 interacts with PTN (PubMed:19141530). Forms a complex with PTPRZ1 and PTN that stimulates endothelial cell migration through ITGB3 'Tyr-773' phosphorylation (PubMed:19141530).  
  
**Gene Ontology Information:**

Molecular Function

- coreceptor activity
- extracellular matrix binding
- extracellular matrix protein binding
- fibronectin binding
- integrin binding
- metal ion binding
- opsonin binding
- protease binding
- protein kinase C binding
- transforming growth factor beta binding
- virus receptor activity
- voltage-gated calcium channel activity

Location

- alphav-beta3 integrin-HMGB1 complex
- alphav-beta3 integrin-IGF-1-IGF1R complex
- alphav-beta3 integrin-PKCalpha complex
- cell surface
- cytosol
- external side of plasma membrane
- extracellular exosome
- filopodium membrane
- focal adhesion
- integral component of plasma membrane
- integrin alphav-beta3 complex
- integrin alphav-beta5 complex
- integrin alphav-beta6 complex
- integrin alphav-beta8 complex
- integrin complex
- lamellipodium membrane
- membrane
- microvillus membrane
- phagocytic vesicle
- plasma membrane
- ruffle membrane
- specific granule membrane

Biological process

- angiogenesis
- antigen processing and presentation of exogenous peptide antigen via MHC class I, TAP-dependent
- apolipoprotein A-I-mediated signaling pathway
- apoptotic cell clearance
- calcium ion transmembrane transport
- cell adhesion
- cell adhesion mediated by integrin
- cell-matrix adhesion
- cell-substrate adhesion
- endodermal cell differentiation
- entry into host cell by a symbiont-containing vacuole
- ERK1 and ERK2 cascade
- extracellular matrix organization
- extrinsic apoptotic signaling pathway in absence of ligand
- heterotypic cell-cell adhesion
- integrin-mediated signaling pathway
- leukocyte migration
- negative chemotaxis
- negative regulation of entry of bacterium into host cell
- negative regulation of extrinsic apoptotic signaling pathway
- negative regulation of lipid storage
- negative regulation of lipid transport
- negative regulation of lipoprotein metabolic process
- negative regulation of low-density lipoprotein receptor activity
- negative regulation of macrophage derived foam cell differentiation
- neutrophil degranulation
- positive regulation of cell adhesion
- positive regulation of cell migration
- positive regulation of cell population proliferation
- positive regulation of cytosolic calcium ion concentration
- positive regulation of osteoblast proliferation
- regulation of phagocytosis
- regulation of transforming growth factor beta activation
- substrate adhesion-dependent cell spreading
- transforming growth factor beta production
- vascular endothelial growth factor receptor signaling pathway
- vasculogenesis
- viral entry into host cell

---

17

- **Protein name:** DNA-directed DNA polymerase
- **Organism:** Escherichia phage RB69
- **Uniprot Accession Number:** Q38087
- **Protein sequence length:** 903 aa
- **1D identity (%):** 12.76
- **1D identity (%) [Gaps excluded]:** 27.66
- **1D identity - Alignment Gaps:** 802
- **Common reported functions (%):** 0.0
- **Common reported locations (%):** 0.0
- **Common reported processes (%):** 0.0

- **PDB ID:** 2P5O
- **Chain:** C
- **Crystallized protein length:** 829 aa
- **Resolution:** 2.8 Å
- **Alinged residues range:** 426-431, 706-713, 867-871, 118-121, 591-609, 605-610
- **Aligned to segment part (indices):** 2, 4, 1, 6, 0, 5
- **Alinged residues range of reference:** 38-43, 198-204, 364-368, 600-603, 764-782, 977-982
- **b-phipsi:** 0.047618
- **w-rdist:** 3.729797
- **t-alpha:** 0.013742
- **Chemical similarity (Tanimoto Index) (%):** 84.58
- **1D identity (%) [PDB]:** 0.0
- **1D identity (%) [Gaps excluded][PDB]:** 0.0
- **1D identity - Alignment Gaps [PDB]:** 1834
- **2D identity (%) [PDB]:** 33.13
- **2D identity (%) [Gaps excluded][PDB]:** 88.4
- **2D identity - Alignment Gaps [PDB]:** 834
- **3D similarity (TM-Score) (%) [PDB]:** 20.49

- **Gene name:** 43
- **RefSeq ID:** NC\_004928
- **Genomic sequence length:** 167560
- **5-UTR|CDS|3-UTR identity (%):** N/A | 40.03 | N/A
- **5-UTR|CDS|3-UTR identity (%) [Gaps excluded]:** N/A | 78.33 | N/A
- **5-UTR|CDS|3-UTR identity [Alignment Gaps]:** N/A | 2114 | N/A

**Uniprot Description:**  
  
Replicates the viral genomic DNA. This polymerase possesses two enzymatic activities: DNA synthesis (polymerase) and an exonucleolytic activity that degrades single-stranded DNA in the 3'- to 5'-direction for proofreading purpose.  
  
Part of the replicase complex that includes the DNA polymerase, the polymerase clamp, the clamp loader complex, the single-stranded DNA binding protein, and the primase/helicase (By similarity). Interacts with the polymerase clamp; this interaction constitutes the polymerase holoenzyme (PubMed:10535734).  
  
**Gene Ontology Information:**

Molecular Function

- 3'-5' exonuclease activity
- DNA binding
- DNA-directed DNA polymerase activity
- metal ion binding
- nucleotide binding

Location  
  
N/A

Biological process

- bidirectional double-stranded viral DNA replication
- DNA replication

---

18

- **Protein name:** Fiber protein
- **Organism:** Human adenovirus C serotype 2
- **Uniprot Accession Number:** P03275
- **Protein sequence length:** 582 aa
- **1D identity (%):** 8.83
- **1D identity (%) [Gaps excluded]:** 28.47
- **1D identity - Alignment Gaps:** 977
- **Common reported functions (%):** 0.0
- **Common reported locations (%):** 0.0
- **Common reported processes (%):** 10.0

- **PDB ID:** 1QIU
- **Chain:** E
- **Crystallized protein length:** 264 aa
- **Resolution:** 2.4 Å
- **Alinged residues range:** 442-444, 441-444, 366-371, 419-427, 440-457, 346-349
- **Aligned to segment part (indices):** 2, 4, 1, 6, 0, 5
- **Alinged residues range of reference:** 50-52, 203-206, 278-283, 541-554, 713-722, 968-971
- **b-phipsi:** 0.013133
- **w-rdist:** 1.842737
- **t-alpha:** 0.895792
- **Chemical similarity (Tanimoto Index) (%):** 83.4
- **1D identity (%) [PDB]:** 0.08
- **1D identity (%) [Gaps excluded][PDB]:** 50.0
- **1D identity - Alignment Gaps [PDB]:** 1243
- **2D identity (%) [PDB]:** 14.57
- **2D identity (%) [Gaps excluded][PDB]:** 84.7
- **2D identity - Alignment Gaps [PDB]:** 881
- **3D similarity (TM-Score) (%) [PDB]:** 10.5

- **Gene name:** L5
- **RefSeq ID:** NC\_001405
- **Genomic sequence length:** 35937
- **5-UTR|CDS|3-UTR identity (%):** N/A | 28.53 | N/A
- **5-UTR|CDS|3-UTR identity (%) [Gaps excluded]:** N/A | 79.36 | N/A
- **5-UTR|CDS|3-UTR identity [Alignment Gaps]:** N/A | 2625 | N/A

**Uniprot Description:**  
  
Forms spikes that protrude from each vertex of the icosahedral capsid. Interacts with host coxsackievirus and adenovirus receptor CXADR located at the cell tight junctions to provide virion initial attachment to target cell. The fiber protein binds to CXADR with a higher affinity than CXADR binds to itself, thereby blocking the cell-cell adhesion function of CXADR dimers and leading to local disruption of the tight junction. Fiber protein present on neo-synthesized particles may thus disrupt the junctional integrity in order to facilitate further neighboring cells infection. Fiber proteins are shed during virus entry, when virus is still at the cell surface. Fiber shedding is dependent on viral CXADR drifting motion and subsequent binding to immobile integrins. Heparan sulfate might also play a role in virus binding.  
  
Homotrimer; arranged in a triple beta-spiral. Interacts with host receptor CXADR. Interacts (via N-terminal tail region) with pentons (Probable).  
  
**Gene Ontology Information:**

Molecular Function  
  
N/A

Location

- host cell nucleus
- viral capsid

Biological process

- adhesion receptor-mediated virion attachment to host cell
- cell adhesion
- viral entry into host cell

---

19

- **Protein name:** Capsid protein alpha
- **Organism:** Orsay virus
- **Uniprot Accession Number:** E9KNV5
- **Protein sequence length:** 391 aa
- **1D identity (%):** 7.64
- **1D identity (%) [Gaps excluded]:** 29.53
- **1D identity - Alignment Gaps:** 980
- **Common reported functions (%):** 0.0
- **Common reported locations (%):** 0.0
- **Common reported processes (%):** 0.0

- **PDB ID:** 4NWV
- **Chain:** A
- **Crystallized protein length:** 355 aa
- **Resolution:** 3.25 Å
- **Alinged residues range:** 50-54, 202-207, 291-297, 280-285, 291-298, 378-380
- **Aligned to segment part (indices):** 2, 4, 1, 6, 0, 5
- **Alinged residues range of reference:** 48-52, 224-229, 312-318, 593-598, 666-673, 960-962
- **b-phipsi:** 0.00109
- **w-rdist:** 6.586554
- **t-alpha:** 0.339943
- **Chemical similarity (Tanimoto Index) (%):** 84.54
- **1D identity (%) [PDB]:** 0.07
- **1D identity (%) [Gaps excluded][PDB]:** 100.0
- **1D identity - Alignment Gaps [PDB]:** 1336
- **2D identity (%) [PDB]:** 23.41
- **2D identity (%) [Gaps excluded][PDB]:** 92.59
- **2D identity - Alignment Gaps [PDB]:** 798
- **3D similarity (TM-Score) (%) [PDB]:** 12.45

- **Gene name:** N/A
- **RefSeq ID:** NC\_028098
- **Genomic sequence length:** 2574
- **5-UTR|CDS|3-UTR identity (%):** 22.99 | 19.18 | 38.04
- **5-UTR|CDS|3-UTR identity (%) [Gaps excluded]:** 79.75 | 77.76 | 73.94
- **5-UTR|CDS|3-UTR identity [Alignment Gaps]:** 195 | 3020 | 134

**Uniprot Description:**  
  
N/A  
  
**Gene Ontology Information:**

Molecular Function

- metal ion binding

Location  
  
N/A

Biological process  
  
N/A

---

20

- **Protein name:** Endonuclease I
- **Organism:** Escherichia phage T7
- **Uniprot Accession Number:** P00641
- **Protein sequence length:** 149 aa
- **1D identity (%):** 3.09
- **1D identity (%) [Gaps excluded]:** 31.75
- **1D identity - Alignment Gaps:** 1170
- **Common reported functions (%):** 0.0
- **Common reported locations (%):** 0.0
- **Common reported processes (%):** 0.0

- **PDB ID:** 1M0I
- **Chain:** C
- **Crystallized protein length:** 129 aa
- **Resolution:** 2.55 Å
- **Alinged residues range:** 48-50, 40-52, 43-47, 40-67, 20-26, 25-29
- **Aligned to segment part (indices):** 2, 4, 1, 6, 0, 5
- **Alinged residues range of reference:** 51-53, 202-209, 325-329, 586-613, 774-780, 957-961
- **b-phipsi:** 0.00536
- **w-rdist:** 1.840851
- **t-alpha:** 1.94704
- **Chemical similarity (Tanimoto Index) (%):** 81.67
- **1D identity (%) [PDB]:** 0.09
- **1D identity (%) [Gaps excluded][PDB]:** 100.0
- **1D identity - Alignment Gaps [PDB]:** 1110
- **2D identity (%) [PDB]:** 8.46
- **2D identity (%) [Gaps excluded][PDB]:** 89.58
- **2D identity - Alignment Gaps [PDB]:** 920
- **3D similarity (TM-Score) (%) [PDB]:** 6.68

- **Gene name:** 3
- **RefSeq ID:** NC\_001604
- **Genomic sequence length:** 39937
- **5-UTR|CDS|3-UTR identity (%):** N/A | 7.75 | N/A
- **5-UTR|CDS|3-UTR identity (%) [Gaps excluded]:** N/A | 80.11 | N/A
- **5-UTR|CDS|3-UTR identity [Alignment Gaps]:** N/A | 3518 | N/A

**Uniprot Description:**  
  
Junction-resolving enzyme that selectively binds and cleaves four-way (Holliday) DNA junctions present after viral genomic replication. These intermediates are created during DNA repair, processing of stalled replication forks and homologous genetic recombination. Introduces two nicks on the two non-crossing strands, at 5' sides of the junction. Participates also together with gp6 in the degradation of host chromosome to provide nucleotides for phage DNA synthesis.  
  
Homodimer.  
  
**Gene Ontology Information:**

Molecular Function

- crossover junction endodeoxyribonuclease activity
- deoxyribonuclease IV (phage-T4-induced) activity
- DNA binding
- double-stranded DNA endodeoxyribonuclease activity

Location  
  
N/A

Biological process

- degradation of host chromosome by virus
- DNA integration
- suppression by virus of host gene expression

---

21

- **Protein name:** Major head protein
- **Organism:** Staphylococcus phage P68
- **Uniprot Accession Number:** Q859I3
- **Protein sequence length:** 408 aa
- **1D identity (%):** 6.59
- **1D identity (%) [Gaps excluded]:** 26.89
- **1D identity - Alignment Gaps:** 1019
- **Common reported functions (%):** 0.0
- **Common reported locations (%):** 0.0
- **Common reported processes (%):** 0.0

- **PDB ID:** 6IAW
- **Chain:** B
- **Crystallized protein length:** 386 aa
- **Resolution:** 3.8 Å
- **Alinged residues range:** 86-88, 371-379, 372-377, 34-41, 150-162, 20-24
- **Aligned to segment part (indices):** 2, 4, 1, 6, 0, 5
- **Alinged residues range of reference:** 51-53, 202-209, 312-317, 521-532, 764-779, 975-979
- **b-phipsi:** 0.019861
- **w-rdist:** 4.823558
- **t-alpha:** 0.035011
- **Chemical similarity (Tanimoto Index) (%):** N/A
- **1D identity (%) [PDB]:** 0.0
- **1D identity (%) [Gaps excluded][PDB]:** 0.0
- **1D identity - Alignment Gaps [PDB]:** 1369
- **2D identity (%) [PDB]:** 20.14
- **2D identity (%) [Gaps excluded][PDB]:** 91.5
- **2D identity - Alignment Gaps [PDB]:** 875
- **3D similarity (TM-Score) (%) [PDB]:** 14.51

- **Gene name:** N/A
- **RefSeq ID:** NC\_004679
- **Genomic sequence length:** 18227
- **5-UTR|CDS|3-UTR identity (%):** N/A | 23.32 | N/A
- **5-UTR|CDS|3-UTR identity (%) [Gaps excluded]:** N/A | 80.96 | N/A
- **5-UTR|CDS|3-UTR identity [Alignment Gaps]:** N/A | 2791 | N/A

**Uniprot Description:**  
  
N/A  
  
**Gene Ontology Information:**

Molecular Function  
  
N/A

Location  
  
N/A

Biological process  
  
N/A

---

22

- **Protein name:** Interferon-induced helicase C domain-containing protein 1
- **Organism:** Mus musculus
- **Uniprot Accession Number:** Q8R5F7
- **Protein sequence length:** 1025 aa
- **1D identity (%):** 6.88
- **1D identity (%) [Gaps excluded]:** 26.98
- **1D identity - Alignment Gaps:** 1364
- **Common reported functions (%):** 50.0
- **Common reported locations (%):** 0.0
- **Common reported processes (%):** 0.0

- **PDB ID:** 6H66
- **Chain:** A
- **Crystallized protein length:** 667 aa
- **Resolution:** 4.16 Å
- **Alinged residues range:** 916-919, 978-980, 731-736, 909-917, 625-637, 685-690
- **Aligned to segment part (indices):** 2, 4, 1, 6, 0, 5
- **Alinged residues range of reference:** 38-41, 228-230, 365-370, 519-527, 761-777, 957-962
- **b-phipsi:** 0.077265
- **w-rdist:** 4.118178
- **t-alpha:** 0.005313
- **Chemical similarity (Tanimoto Index) (%):** 83.08
- **1D identity (%) [PDB]:** 0.3
- **1D identity (%) [Gaps excluded][PDB]:** 71.43
- **1D identity - Alignment Gaps [PDB]:** 1643
- **2D identity (%) [PDB]:** 23.66
- **2D identity (%) [Gaps excluded][PDB]:** 89.34
- **2D identity - Alignment Gaps [PDB]:** 963
- **3D similarity (TM-Score) (%) [PDB]:** 17.73

- **Gene name:** Ifih1
- **RefSeq ID:** N/A
- **Sequence length:** N/A
- **5-UTR|CDS|3-UTR identity (%):** N/A | N/A | N/A
- **5-UTR|CDS|3-UTR identity (%) [Gaps excluded]:** N/A | N/A | N/A
- **5-UTR|CDS|3-UTR identity [Alignment Gaps]:** N/A | N/A | N/A

**Uniprot Description:**  
  
Innate immune receptor which acts as a cytoplasmic sensor of viral nucleic acids and plays a major role in sensing viral infection and in the activation of a cascade of antiviral responses including the induction of type I interferons and proinflammatory cytokines. Its ligands include mRNA lacking 2'-O-methylation at their 5' cap and long-dsRNA (>1 kb in length). Upon ligand binding it associates with mitochondria antiviral signaling protein (MAVS/IPS1) which activates the IKK-related kinases: TBK1 and IKBKE which phosphorylate interferon regulatory factors: IRF3 and IRF7 which in turn activate transcription of antiviral immunological genes, including interferons (IFNs); IFN-alpha and IFN-beta. Responsible for detecting the Picornaviridae family members such as encephalomyocarditis virus (EMCV), mengo encephalomyocarditis virus (ENMG), and theiler's murine encephalomyelitis virus (TMEV). Can also detect other viruses such as dengue virus (DENV), west Nile virus (WNV), and reovirus. Also involved in antiviral signaling in response to viruses containing a dsDNA genome, such as vaccinia virus. Plays an important role in amplifying innate immune signaling through recognition of RNA metabolites that are produced during virus infection by ribonuclease L (RNase L). May play an important role in enhancing natural killer cell function and may be involved in growth inhibition and apoptosis in several tumor cell lines.  
  
Monomer in the absence of ligands and homodimerizes in the presence of dsRNA ligands. Can assemble into helical or linear polymeric filaments on long dsRNA. Interacts with MAVS/IPS1. Interacts with PCBP2. Interacts with NLRC5. Interacts with PIAS2-beta. Interacts with DDX60. Interacts with ANKRD17. Interacts with IKBKE. Interacts (via the CARD domains) with TKFC, the interaction is inhibited by viral infection (By similarity). Interacts with ATG5 and ATG12, either as ATG5 and ATG12 monomers or as ATG12-ATG5 conjugates (By similarity). Interacts with ZCCHC3; leading to activate IFIH1/MDA5 (By similarity). Interacts with RNF123 (By similarity). Interacts with DDX3X (By similarity).  
  
**Gene Ontology Information:**

Molecular Function

- ATP binding
- DNA binding
- double-stranded RNA binding
- hydrolase activity
- identical protein binding
- ribonucleoprotein complex binding
- RNA helicase activity
- single-stranded RNA binding
- zinc ion binding

Location

- cytoplasm
- nucleus

Biological process

- cellular response to exogenous dsRNA
- defense response to virus
- innate immune response
- MDA-5 signaling pathway
- positive regulation of interferon-alpha production
- positive regulation of interferon-beta production
- positive regulation of interleukin-6 production
- positive regulation of response to cytokine stimulus
- positive regulation of tumor necrosis factor production
- protein sumoylation
- response to virus

---

23

- **Protein name:** Tail spike protein
- **Organism:** Escherichia phage K1F
- **Uniprot Accession Number:** Q04830
- **Protein sequence length:** 1064 aa
- **1D identity (%):** 8.96
- **1D identity (%) [Gaps excluded]:** 29.76
- **1D identity - Alignment Gaps:** 1255
- **Common reported functions (%):** 50.0
- **Common reported locations (%):** 0.0
- **Common reported processes (%):** 0.0

- **PDB ID:** 3GW6
- **Chain:** E
- **Crystallized protein length:** 257 aa
- **Resolution:** 2.6 Å
- **Alinged residues range:** 1014-1017, 1029-1033, 993-999, 1013-1022, 1014-1023, 958-962
- **Aligned to segment part (indices):** 2, 4, 1, 6, 0, 5
- **Alinged residues range of reference:** 50-53, 205-209, 431-437, 547-554, 715-726, 976-980
- **b-phipsi:** 0.002581
- **w-rdist:** 2.45059
- **t-alpha:** 1.884146
- **Chemical similarity (Tanimoto Index) (%):** 83.21
- **1D identity (%) [PDB]:** 0.0
- **1D identity (%) [Gaps excluded][PDB]:** 0.0
- **1D identity - Alignment Gaps [PDB]:** 1242
- **2D identity (%) [PDB]:** 14.79
- **2D identity (%) [Gaps excluded][PDB]:** 90.8
- **2D identity - Alignment Gaps [PDB]:** 894
- **3D similarity (TM-Score) (%) [PDB]:** 11.59

- **Gene name:** N/A
- **RefSeq ID:** NC\_007456
- **Genomic sequence length:** 39704
- **5-UTR|CDS|3-UTR identity (%):** N/A | 41.66 | N/A
- **5-UTR|CDS|3-UTR identity (%) [Gaps excluded]:** N/A | 78.64 | N/A
- **5-UTR|CDS|3-UTR identity [Alignment Gaps]:** N/A | 2157 | N/A

**Uniprot Description:**  
  
Tail spike protein
Responsible for initial absorption of the phage to the host bacterium. Degrades the alpha-2,8-linked polysialic acid K1 capsule by cleaving within the polymer chain of polysialic acid.  
  
Tail spike protein
Homotrimer (PubMed:3546309, PubMed:20124697). Interacts with sialic acid (PubMed:15608653, PubMed:20096705).  
  
**Gene Ontology Information:**

Molecular Function

- endo-alpha-(2,8)-sialidase activity
- identical protein binding

Location

- virus tail, fiber

Biological process

- adhesion receptor-mediated virion attachment to host cell
- disruption of host cell glycocalyx during viral entry
- entry into host
- metabolic process
- virion attachment to host cell

---

24

- **Protein name:** Complement decay-accelerating factor
- **Organism:** Homo sapiens
- **Uniprot Accession Number:** P08174
- **Protein sequence length:** 381 aa
- **1D identity (%):** 5.09
- **1D identity (%) [Gaps excluded]:** 30.0
- **1D identity - Alignment Gaps:** 1174
- **Common reported functions (%):** 0.0
- **Common reported locations (%):** 0.0
- **Common reported processes (%):** 0.0

- **PDB ID:** 6ILK
- **Chain:** E
- **Crystallized protein length:** 192 aa
- **Resolution:** 3.0 Å
- **Alinged residues range:** 251-253, 110-112, 230-238, 232-234, 232-238
- **Aligned to segment part (indices):** 2, 4, 1, 6, 0
- **Alinged residues range of reference:** 43-45, 228-230, 309-317, 594-596, 719-725
- **b-phipsi:** 0.016628
- **w-rdist:** 1.890417
- **t-alpha:** 1.324324
- **Chemical similarity (Tanimoto Index) (%):** 86.07
- **1D identity (%) [PDB]:** 0.0
- **1D identity (%) [Gaps excluded][PDB]:** 0.0
- **1D identity - Alignment Gaps [PDB]:** 1175
- **2D identity (%) [PDB]:** 12.77
- **2D identity (%) [Gaps excluded][PDB]:** 82.8
- **2D identity - Alignment Gaps [PDB]:** 861
- **3D similarity (TM-Score) (%) [PDB]:** 9.41

- **Gene name:** CD55
- **RefSeq ID:** NM\_001300904
- **Transcript sequence length:** 2683
- **5-UTR|CDS|3-UTR identity (%):** 18.06 | 19.18 | 10.21
- **5-UTR|CDS|3-UTR identity (%) [Gaps excluded]:** 80.0 | 78.35 | 79.47
- **5-UTR|CDS|3-UTR identity [Alignment Gaps]:** 223 | 3019 | 1289

**Uniprot Description:**  
  
This protein recognizes C4b and C3b fragments that condense with cell-surface hydroxyl or amino groups when nascent C4b and C3b are locally generated during C4 and c3 activation. Interaction of daf with cell-associated C4b and C3b polypeptides interferes with their ability to catalyze the conversion of C2 and factor B to enzymatically active C2a and Bb and thereby prevents the formation of C4b2a and C3bBb, the amplification convertases of the complement cascade (PubMed:7525274). Inhibits complement activation by destabilizing and preventing the formation of C3 and C5 convertases, which prevents complement damage (PubMed:28657829).  
  
Monomer (major form) and non-disulfide-linked, covalent homodimer (minor form). Interacts with ADGRE5 (PubMed:11297558).  
  
**Gene Ontology Information:**

Molecular Function

- lipid binding
- virus receptor activity

Location

- anchored component of membrane
- cell surface
- endoplasmic reticulum-Golgi intermediate compartment membrane
- extracellular exosome
- extracellular region
- ficolin-1-rich granule membrane
- Golgi membrane
- membrane raft
- plasma membrane
- secretory granule membrane
- transport vesicle

Biological process

- complement activation, classical pathway
- endoplasmic reticulum to Golgi vesicle-mediated transport
- innate immune response
- negative regulation of complement activation
- neutrophil degranulation
- positive regulation of CD4-positive, alpha-beta T cell activation
- positive regulation of CD4-positive, alpha-beta T cell proliferation
- positive regulation of cytosolic calcium ion concentration
- positive regulation of T cell cytokine production
- regulation of complement activation
- regulation of complement-dependent cytotoxicity
- regulation of lipopolysaccharide-mediated signaling pathway
- respiratory burst

---

25

- **Protein name:** DNA primase/helicase
- **Organism:** Escherichia phage T7
- **Uniprot Accession Number:** P03692
- **Protein sequence length:** 566 aa
- **1D identity (%):** 11.03
- **1D identity (%) [Gaps excluded]:** 30.53
- **1D identity - Alignment Gaps:** 863
- **Common reported functions (%):** 50.0
- **Common reported locations (%):** 0.0
- **Common reported processes (%):** 0.0

- **PDB ID:** 5IKN
- **Chain:** D
- **Crystallized protein length:** 485 aa
- **Resolution:** 4.8 Å
- **Alinged residues range:** 542-545, 532-545, 496-500, 120-125, 525-532, 325-329
- **Aligned to segment part (indices):** 2, 4, 1, 6, 0, 5
- **Alinged residues range of reference:** 48-51, 200-209, 323-327, 573-578, 689-696, 957-961
- **b-phipsi:** 0.040393
- **w-rdist:** 3.442844
- **t-alpha:** 0.059351
- **Chemical similarity (Tanimoto Index) (%):** 83.13
- **1D identity (%) [PDB]:** 0.0
- **1D identity (%) [Gaps excluded][PDB]:** 0.0
- **1D identity - Alignment Gaps [PDB]:** 1468
- **2D identity (%) [PDB]:** 21.27
- **2D identity (%) [Gaps excluded][PDB]:** 89.05
- **2D identity - Alignment Gaps [PDB]:** 902
- **3D similarity (TM-Score) (%) [PDB]:** 16.1

- **Gene name:** 4
- **RefSeq ID:** NC\_001604
- **Genomic sequence length:** 39937
- **5-UTR|CDS|3-UTR identity (%):** N/A | 29.08 | N/A
- **5-UTR|CDS|3-UTR identity (%) [Gaps excluded]:** N/A | 78.0 | N/A
- **5-UTR|CDS|3-UTR identity [Alignment Gaps]:** N/A | 2523 | N/A

**Uniprot Description:**  
  
Synthesizes short RNA primers for DNA replication. Unwinds the DNA at the replication forks and generates single-stranded DNA for both leading and lagging strand synthesis. The primase synthesizes short RNA primers on the lagging strand that the polymerase elongates using dNTPs.  
  
Homohexamer. Present in a mixture of heptamers and hexamers in the absence of DNA, and assembles onto ssDNA as a hexamer. Interacts with the DNA polymerase gp5; this interaction is essential to initiate leading-strand DNA synthesis. Interacts with single-stranded DNA-binding protein gp2.5.  
  
**Gene Ontology Information:**

Molecular Function

- ATP binding
- DNA helicase activity
- DNA primase activity
- identical protein binding
- zinc ion binding

Location  
  
N/A

Biological process  
  
N/A

---

26

- **Protein name:** Maltose/maltodextrin-binding periplasmic protein
- **Organism:** Escherichia coli (strain K12)
- **Uniprot Accession Number:** P0AEX9
- **Protein sequence length:** 396 aa
- **1D identity (%):** 5.61
- **1D identity (%) [Gaps excluded]:** 30.27
- **1D identity - Alignment Gaps:** 1147
- **Common reported functions (%):** 0.0
- **Common reported locations (%):** 0.0
- **Common reported processes (%):** 0.0

- **PDB ID:** 4GIZ
- **Chain:** A
- **Crystallized protein length:** 382 aa
- **Resolution:** 2.55 Å
- **Alinged residues range:** 17-19, 176-180, 241-246, 243-246, 293-310, 195-201
- **Aligned to segment part (indices):** 2, 4, 1, 6, 0, 5
- **Alinged residues range of reference:** 38-40, 224-228, 308-313, 593-596, 771-780, 958-964
- **b-phipsi:** 0.040555
- **w-rdist:** 6.656103
- **t-alpha:** 0.006383
- **Chemical similarity (Tanimoto Index) (%):** 84.77
- **1D identity (%) [PDB]:** 0.0
- **1D identity (%) [Gaps excluded][PDB]:** 0.0
- **1D identity - Alignment Gaps [PDB]:** 1365
- **2D identity (%) [PDB]:** 16.81
- **2D identity (%) [Gaps excluded][PDB]:** 91.94
- **2D identity - Alignment Gaps [PDB]:** 943
- **3D similarity (TM-Score) (%) [PDB]:** 10.35

- **Gene name:** malE
- **RefSeq ID:** N/A
- **Sequence length:** N/A
- **5-UTR|CDS|3-UTR identity (%):** N/A | N/A | N/A
- **5-UTR|CDS|3-UTR identity (%) [Gaps excluded]:** N/A | N/A | N/A
- **5-UTR|CDS|3-UTR identity [Alignment Gaps]:** N/A | N/A | N/A

**Uniprot Description:**  
  
Part of the ABC transporter complex MalEFGK involved in maltose/maltodextrin import. Binds maltose and higher maltodextrins such as maltotriose.  
  
The complex is composed of two ATP-binding proteins (MalK), two transmembrane proteins (MalG and MalF) and a solute-binding protein (MalE).  
  
**Gene Ontology Information:**

Molecular Function

- carbohydrate transmembrane transporter activity
- maltose binding

Location

- ATP-binding cassette (ABC) transporter complex
- ATP-binding cassette (ABC) transporter complex, substrate-binding subunit-containing
- outer membrane-bounded periplasmic space
- periplasmic space

Biological process

- carbohydrate transport
- cell chemotaxis
- cellular response to DNA damage stimulus
- detection of maltose stimulus
- maltodextrin transport
- maltose transport

---

27

- **Protein name:** Matrix protein VP40
- **Organism:** Zaire ebolavirus (strain Mayinga-76)
- **Uniprot Accession Number:** Q05128
- **Protein sequence length:** 326 aa
- **1D identity (%):** 6.31
- **1D identity (%) [Gaps excluded]:** 27.42
- **1D identity - Alignment Gaps:** 1001
- **Common reported functions (%):** 50.0
- **Common reported locations (%):** 25.0
- **Common reported processes (%):** 0.0

- **PDB ID:** 7JZJ
- **Chain:** D
- **Crystallized protein length:** 246 aa
- **Resolution:** 2.46 Å
- **Alinged residues range:** 74-76, 304-308, 304-307, 234-237, 90-95, 265-269
- **Aligned to segment part (indices):** 2, 4, 1, 6, 0, 5
- **Alinged residues range of reference:** 47-49, 200-204, 399-402, 605-608, 662-667, 960-964
- **b-phipsi:** 0.000759
- **w-rdist:** 9.015852
- **t-alpha:** 0.138387
- **Chemical similarity (Tanimoto Index) (%):** 85.77
- **1D identity (%) [PDB]:** 0.08
- **1D identity (%) [Gaps excluded][PDB]:** 50.0
- **1D identity - Alignment Gaps [PDB]:** 1229
- **2D identity (%) [PDB]:** 16.36
- **2D identity (%) [Gaps excluded][PDB]:** 84.5
- **2D identity - Alignment Gaps [PDB]:** 833
- **3D similarity (TM-Score) (%) [PDB]:** 9.47

- **Gene name:** VP40
- **RefSeq ID:** NC\_002549
- **Genomic sequence length:** 18959
- **5-UTR|CDS|3-UTR identity (%):** 15.79 | 17.43 | 19.86
- **5-UTR|CDS|3-UTR identity (%) [Gaps excluded]:** 77.78 | 79.12 | 74.35
- **5-UTR|CDS|3-UTR identity [Alignment Gaps]:** 212 | 3069 | 524

**Uniprot Description:**  
  
Plays an essential role virus particle assembly and budding (PubMed:16719918). Acts by interacting with viral ribonucleocapsid and host members of the ESCRT (endosomal sorting complex required for transport) system such as host VPS4, PDCD6IP/ALIX, NEDD4 or TGS101 (PubMed:15892969, PubMed:16719918, PubMed:23637409, PubMed:25786915, PubMed:26753796, PubMed:27489272). May play a role in immune cell dysfunction by being packaged into exosomes that can decrease the viability of recipient cells (via RNAi suppression and exosome-bystander apoptosis) (PubMed:27872619).  
  
Homodimer (PubMed:23953110). Homohexamer (PubMed:23953110, PubMed:11118208). Homooctamer (PubMed:12919741). Exists as a dimer until it reorganizes at the plasma membrane into a hexameric form using phosphatidylinositol 4,5-bisphosphate (PI(4,5)P2) (PubMed:23953110, PubMed:25159197, PubMed:26753796, PubMed:29950600). Hexamers are critical for budding (PubMed:23953110). Octamers function in genome replication and RNA binding (PubMed:12919741). Interacts with host TSG101 (PubMed:12559917). As a homohexamer, interacts with the WW domain 3 of host NEDD4 (PubMed:11095724, PubMed:12559917). Interacts with the nucleoprotein/NP (PubMed:17229682, PubMed:21987757). Interacts (via YPx(n)L/I motif) with host PDCD6IP/ALIX; this interaction supports efficient egress of viral particles (PubMed:25786915). Interacts with VP35 (PubMed:16698994). Interacts with host ITCH; this interaction is required for efficient egress (PubMed:27489272).  
  
**Gene Ontology Information:**

Molecular Function

- identical protein binding
- RNA binding
- structural constituent of virion

Location

- extracellular region
- host cell endomembrane system
- host cell late endosome membrane
- host cell plasma membrane
- integral to membrane of host cell
- membrane raft
- virion membrane

Biological process

- intracellular transport of virus
- mitigation of host immune response by virus
- suppression of host defenses by symbiont
- viral budding
- viral budding from plasma membrane
- viral budding via host ESCRT complex

---

28

- **Protein name:** Genome polyprotein
- **Organism:** Yellow fever virus (strain 17D vaccine)
- **Uniprot Accession Number:** P03314
- **Protein sequence length:** 3411 aa
- **1D identity (%):** 7.05
- **1D identity (%) [Gaps excluded]:** 28.12
- **1D identity - Alignment Gaps:** 2806
- **Common reported functions (%):** 0.0
- **Common reported locations (%):** 37.5
- **Common reported processes (%):** 20.0

- **PDB ID:** 6IW2
- **Chain:** P
- **Crystallized protein length:** 387 aa
- **Resolution:** 2.9 Å
- **Alinged residues range:** 307-312, 308-311, 111-116, 63-67, 307-313, 254-256
- **Aligned to segment part (indices):** 2, 4, 1, 6, 0, 5
- **Alinged residues range of reference:** 47-52, 206-209, 431-436, 609-613, 670-676, 959-961
- **b-phipsi:** 0.001208
- **w-rdist:** 6.641087
- **t-alpha:** 0.457627
- **Chemical similarity (Tanimoto Index) (%):** 85.37
- **1D identity (%) [PDB]:** 0.0
- **1D identity (%) [Gaps excluded][PDB]:** 0.0
- **1D identity - Alignment Gaps [PDB]:** 1371
- **2D identity (%) [PDB]:** 24.79
- **2D identity (%) [Gaps excluded][PDB]:** 89.26
- **2D identity - Alignment Gaps [PDB]:** 775
- **3D similarity (TM-Score) (%) [PDB]:** 15.62

- **Gene name:** N/A
- **RefSeq ID:** NC\_002031
- **Genomic sequence length:** 10862
- **5-UTR|CDS|3-UTR identity (%):** N/A | 23.34 | N/A
- **5-UTR|CDS|3-UTR identity (%) [Gaps excluded]:** N/A | 79.01 | N/A
- **5-UTR|CDS|3-UTR identity [Alignment Gaps]:** N/A | 7646 | N/A

**Uniprot Description:**  
  
Capsid protein C
Plays a role in virus budding by binding to the cell membrane and gathering the viral RNA into a nucleocapsid that forms the core of a mature virus particle. During virus entry, may induce genome penetration into the host cytoplasm after hemifusion induced by the surface proteins. Can migrate to the cell nucleus where it modulates host functions.  
  
Capsid protein C
Homodimer (PubMed:12768036). Interacts (via N-terminus) with host EXOC1 (via C-terminus); this interaction results in EXOC1 degradation through the proteasome degradation pathway (By similarity).  
  
**Gene Ontology Information:**

Molecular Function

- ATP binding
- double-stranded RNA binding
- GTP binding
- metal ion binding
- mRNA (guanine-N7-)-methyltransferase activity
- mRNA (nucleoside-2'-O-)-methyltransferase activity
- nucleoside-triphosphatase activity
- protein dimerization activity
- RNA helicase activity
- RNA-directed 5'-3' RNA polymerase activity
- serine-type endopeptidase activity
- structural molecule activity

Location

- extracellular region
- host cell endoplasmic reticulum membrane
- host cell nucleus
- host cell perinuclear region of cytoplasm
- integral component of membrane
- viral capsid
- viral envelope
- virion membrane

Biological process

- clathrin-dependent endocytosis of virus by host cell
- fusion of virus membrane with host endosome membrane
- induction by virus of host autophagy
- negative regulation of RNA interference
- suppression by virus of host STAT2 activity
- suppression by virus of host type I interferon-mediated signaling pathway
- viral budding from endoplasmic reticulum membrane
- viral RNA genome replication
- virion attachment to host cell

---

29

- **Protein name:** Major inner protein P1
- **Organism:** Pseudomonas phage phi6
- **Uniprot Accession Number:** P11126
- **Protein sequence length:** 769 aa
- **1D identity (%):** 8.42
- **1D identity (%) [Gaps excluded]:** 29.71
- **1D identity - Alignment Gaps:** 1140
- **Common reported functions (%):** 50.0
- **Common reported locations (%):** 0.0
- **Common reported processes (%):** 0.0

- **PDB ID:** 5FJ7
- **Chain:** A
- **Crystallized protein length:** 761 aa
- **Resolution:** 7.9 Å
- **Alinged residues range:** 541-543, 374-377, 251-255, 373-376, 635-644, 156-159
- **Aligned to segment part (indices):** 2, 4, 1, 6, 0, 5
- **Alinged residues range of reference:** 44-46, 208-211, 365-369, 592-595, 766-775, 979-982
- **b-phipsi:** 0.068853
- **w-rdist:** 3.547811
- **t-alpha:** 0.04185
- **Chemical similarity (Tanimoto Index) (%):** N/A
- **1D identity (%) [PDB]:** 0.0
- **1D identity (%) [Gaps excluded][PDB]:** 0.0
- **1D identity - Alignment Gaps [PDB]:** 1744
- **2D identity (%) [PDB]:** 27.23
- **2D identity (%) [Gaps excluded][PDB]:** 91.5
- **2D identity - Alignment Gaps [PDB]:** 944
- **3D similarity (TM-Score) (%) [PDB]:** 18.23

- **Gene name:** P1
- **RefSeq ID:** NC\_003715
- **Genomic sequence length:** 6374
- **5-UTR|CDS|3-UTR identity (%):** N/A | 34.85 | N/A
- **5-UTR|CDS|3-UTR identity (%) [Gaps excluded]:** N/A | 76.33 | N/A
- **5-UTR|CDS|3-UTR identity [Alignment Gaps]:** N/A | 2288 | N/A

**Uniprot Description:**  
  
P1 is the major inner capsid (core) protein of the polyhedral procapsid, which is responsible for genomic replication and transcription. Forms a dodecahedral shell from 60 asymmetric dimers. Binds to RNA and may be involved in genomic packaging.  
  
Homodimer. Associates with the polymerase complex.  
  
**Gene Ontology Information:**

Molecular Function

- identical protein binding
- RNA binding

Location

- T=2 icosahedral viral capsid
- viral inner capsid
- viral nucleocapsid
- virion

Biological process  
  
N/A

---

30

- **Protein name:** Genome polyprotein
- **Organism:** Dengue virus type 4 (strain Dominica/814669/1981)
- **Uniprot Accession Number:** P09866
- **Protein sequence length:** 3387 aa
- **1D identity (%):** 7.09
- **1D identity (%) [Gaps excluded]:** 26.69
- **1D identity - Alignment Gaps:** 2704
- **Common reported functions (%):** 0.0
- **Common reported locations (%):** 37.5
- **Common reported processes (%):** 20.0

- **PDB ID:** 3UAJ
- **Chain:** B
- **Crystallized protein length:** 371 aa
- **Resolution:** 3.23 Å
- **Alinged residues range:** 38-42, 122-138, 315-322, 196-206, 113-127, 259-262
- **Aligned to segment part (indices):** 2, 4, 1, 6, 0, 5
- **Alinged residues range of reference:** 49-53, 200-209, 426-433, 538-548, 714-728, 958-961
- **b-phipsi:** 0.01908
- **w-rdist:** 1.957328
- **t-alpha:** 1.116331
- **Chemical similarity (Tanimoto Index) (%):** 99.74
- **1D identity (%) [PDB]:** 0.0
- **1D identity (%) [Gaps excluded][PDB]:** 0.0
- **1D identity - Alignment Gaps [PDB]:** 1357
- **2D identity (%) [PDB]:** 23.62
- **2D identity (%) [Gaps excluded][PDB]:** 88.46
- **2D identity - Alignment Gaps [PDB]:** 785
- **3D similarity (TM-Score) (%) [PDB]:** 11.52

- **Gene name:** N/A
- **RefSeq ID:** NC\_002640
- **Genomic sequence length:** 10649
- **5-UTR|CDS|3-UTR identity (%):** 21.48 | 24.77 | 26.58
- **5-UTR|CDS|3-UTR identity (%) [Gaps excluded]:** 74.39 | 80.32 | 79.22
- **5-UTR|CDS|3-UTR identity [Alignment Gaps]:** 202 | 7392 | 305

**Uniprot Description:**  
  
Capsid protein C
Plays a role in virus budding by binding to the cell membrane and gathering the viral RNA into a nucleocapsid that forms the core of a mature virus particle. During virus entry, may induce genome penetration into the host cytoplasm after hemifusion induced by the surface proteins. Can migrate to the cell nucleus where it modulates host functions. Overcomes the anti-viral effects of host EXOC1 by sequestering and degrading the latter through the proteasome degradation pathway.  
  
Capsid protein C
Homodimer. Interacts (via N-terminus) with host EXOC1 (via C-terminus); this interaction results in EXOC1 degradation through the proteasome degradation pathway.  
  
**Gene Ontology Information:**

Molecular Function

- ATP binding
- double-stranded RNA binding
- exogenous protein binding
- ion channel activity
- metal ion binding
- mRNA (guanine-N7-)-methyltransferase activity
- mRNA (nucleoside-2'-O-)-methyltransferase activity
- nucleoside-triphosphatase activity
- protein dimerization activity
- RNA helicase activity
- RNA-directed 5'-3' RNA polymerase activity
- serine-type endopeptidase activity
- structural molecule activity

Location

- extracellular region
- host cell endoplasmic reticulum membrane
- host cell mitochondrion
- host cell nucleus
- host cell perinuclear region of cytoplasm
- integral component of membrane
- integral to membrane of host cell
- viral capsid
- viral envelope
- virion membrane

Biological process

- clathrin-dependent endocytosis of virus by host cell
- fusion of virus membrane with host endosome membrane
- induction by virus of host autophagy
- pore formation by virus in membrane of host cell
- protein complex oligomerization
- suppression by virus of host MAVS activity
- suppression by virus of host STAT2 activity
- suppression by virus of host transcription
- suppression by virus of host TYK2 activity
- suppression by virus of host type I interferon-mediated signaling pathway
- viral RNA genome replication
- virion attachment to host cell

---

31

- **Protein name:** p81
- **Organism:** Providence virus (isolate Helicoverpa zea/United States/vFLM1/-)
- **Uniprot Accession Number:** Q80IX5
- **Protein sequence length:** 754 aa
- **1D identity (%):** N/A
- **1D identity (%) [Gaps excluded]:** N/A
- **1D identity - Alignment Gaps:** N/A
- **Common reported functions (%):** N/A
- **Common reported locations (%):** N/A
- **Common reported processes (%):** N/A

- **PDB ID:** 2QQP
- **Chain:** A
- **Crystallized protein length:** 484 aa
- **Resolution:** 3.8 Å
- **Alinged residues range:** 505-510, 511-516, 509-513, 196-205, 505-517, 174-178
- **Aligned to segment part (indices):** 2, 4, 1, 6, 0, 5
- **Alinged residues range of reference:** 47-52, 204-209, 313-317, 542-551, 712-725, 960-964
- **b-phipsi:** 0.009628
- **w-rdist:** 7.009897
- **t-alpha:** 0.058166
- **Chemical similarity (Tanimoto Index) (%):** N/A
- **1D identity (%) [PDB]:** N/A
- **1D identity (%) [Gaps excluded][PDB]:** N/A
- **1D identity - Alignment Gaps [PDB]:** N/A
- **2D identity (%) [PDB]:** N/A
- **2D identity (%) [Gaps excluded][PDB]:** N/A
- **2D identity - Alignment Gaps [PDB]:** N/A
- **3D similarity (TM-Score) (%) [PDB]:** N/A

- **Gene name:** N/A
- **RefSeq ID:** N/A
- **Sequence length:** N/A
- **5-UTR|CDS|3-UTR identity (%):** N/A | N/A | N/A
- **5-UTR|CDS|3-UTR identity (%) [Gaps excluded]:** N/A | N/A | N/A
- **5-UTR|CDS|3-UTR identity [Alignment Gaps]:** N/A | N/A | N/A

**Uniprot Description:**  
  
N/A  
  
**Gene Ontology Information:**

Molecular Function

- identical protein binding

Location  
  
N/A

Biological process  
  
N/A

---

32

- **Protein name:** Replicative DNA helicase
- **Organism:** Escherichia coli (strain K12)
- **Uniprot Accession Number:** P0ACB0
- **Protein sequence length:** 471 aa
- **1D identity (%):** 8.47
- **1D identity (%) [Gaps excluded]:** 26.54
- **1D identity - Alignment Gaps:** 900
- **Common reported functions (%):** 50.0
- **Common reported locations (%):** 0.0
- **Common reported processes (%):** 0.0

- **PDB ID:** 6BBM
- **Chain:** B
- **Crystallized protein length:** 447 aa
- **Resolution:** 4.1 Å
- **Alinged residues range:** 178-181, 431-436, 435-440, 455-458, 48-52, 184-186
- **Aligned to segment part (indices):** 2, 4, 1, 6, 0, 5
- **Alinged residues range of reference:** 38-41, 198-203, 432-437, 602-605, 778-782, 979-981
- **b-phipsi:** 0.075508
- **w-rdist:** 4.159158
- **t-alpha:** 0.031712
- **Chemical similarity (Tanimoto Index) (%):** 81.98
- **1D identity (%) [PDB]:** 0.07
- **1D identity (%) [Gaps excluded][PDB]:** 100.0
- **1D identity - Alignment Gaps [PDB]:** 1428
- **2D identity (%) [PDB]:** 18.74
- **2D identity (%) [Gaps excluded][PDB]:** 92.92
- **2D identity - Alignment Gaps [PDB]:** 950
- **3D similarity (TM-Score) (%) [PDB]:** 16.96

- **Gene name:** dnaB
- **RefSeq ID:** N/A
- **Sequence length:** N/A
- **5-UTR|CDS|3-UTR identity (%):** N/A | N/A | N/A
- **5-UTR|CDS|3-UTR identity (%) [Gaps excluded]:** N/A | N/A | N/A
- **5-UTR|CDS|3-UTR identity [Alignment Gaps]:** N/A | N/A | N/A

**Uniprot Description:**  
  
Participates in initiation and elongation during chromosome replication; it exhibits DNA-dependent ATPase activity and contains distinct active sites for ATP binding, DNA binding, and interaction with DnaC protein, primase, and other prepriming proteins.  
  
Homohexamer.  
  
**Gene Ontology Information:**

Molecular Function

- ATP binding
- DNA binding
- DNA helicase activity
- helicase activity
- identical protein binding

Location

- cytosol
- primosome complex

Biological process

- DNA replication
- DNA replication, synthesis of RNA primer
- DNA unwinding involved in DNA replication
- response to ionizing radiation

---

33

- **Protein name:** Calcium uniporter protein, mitochondrial
- **Organism:** Homo sapiens
- **Uniprot Accession Number:** Q8NE86
- **Protein sequence length:** 351 aa
- **1D identity (%):** 4.98
- **1D identity (%) [Gaps excluded]:** 26.25
- **1D identity - Alignment Gaps:** 1106
- **Common reported functions (%):** 50.0
- **Common reported locations (%):** 0.0
- **Common reported processes (%):** 0.0

- **PDB ID:** 4XSJ
- **Chain:** A
- **Crystallized protein length:** 253 aa
- **Resolution:** 1.8 Å
- **Alinged residues range:** 15-19, 1157-1159, 1154-1160, 1075-1079, 60-67, 105-111
- **Aligned to segment part (indices):** 2, 4, 1, 6, 0, 5
- **Alinged residues range of reference:** 48-52, 204-206, 427-433, 595-599, 775-782, 957-963
- **b-phipsi:** 0.054048
- **w-rdist:** 7.656617
- **t-alpha:** 0.004246
- **Chemical similarity (Tanimoto Index) (%):** 82.95
- **1D identity (%) [PDB]:** 0.0
- **1D identity (%) [Gaps excluded][PDB]:** 0.0
- **1D identity - Alignment Gaps [PDB]:** 1237
- **2D identity (%) [PDB]:** 16.11
- **2D identity (%) [Gaps excluded][PDB]:** 89.89
- **2D identity - Alignment Gaps [PDB]:** 861
- **3D similarity (TM-Score) (%) [PDB]:** 10.21

- **Gene name:** MCU
- **RefSeq ID:** NM\_138357
- **Transcript sequence length:** 2937
- **5-UTR|CDS|3-UTR identity (%):** 2.64 | 18.42 | 7.67
- **5-UTR|CDS|3-UTR identity (%) [Gaps excluded]:** 77.78 | 79.15 | 74.11
- **5-UTR|CDS|3-UTR identity [Alignment Gaps]:** 256 | 3036 | 1707

**Uniprot Description:**  
  
Mitochondrial inner membrane calcium uniporter that mediates calcium uptake into mitochondria (PubMed:21685888, PubMed:21685886, PubMed:23101630, PubMed:22904319, PubMed:23178883, PubMed:22829870, PubMed:22822213, PubMed:24332854, PubMed:23755363, PubMed:26341627). Constitutes the pore-forming and calcium-conducting subunit of the uniporter complex (uniplex) (PubMed:23755363). Activity is regulated by MICU1 and MICU2. At low Ca(2+) levels MCU activity is down-regulated by MICU1 and MICU2; at higher Ca(2+) levels MICU1 increases MCU activity (PubMed:24560927, PubMed:26903221). Mitochondrial calcium homeostasis plays key roles in cellular physiology and regulates cell bioenergetics, cytoplasmic calcium signals and activation of cell death pathways. Involved in buffering the amplitude of systolic calcium rises in cardiomyocytes (PubMed:22822213). While dispensable for baseline homeostatic cardiac function, acts as a key regulator of short-term mitochondrial calcium loading underlying a 'fight-or-flight' response during acute stress: acts by mediating a rapid increase of mitochondrial calcium in pacemaker cells (PubMed:25603276). participates in mitochondrial permeability transition during ischemia-reperfusion injury (By similarity). Regulates glucose-dependent insulin secretion in pancreatic beta-cells by regulating mitochondrial calcium uptake (PubMed:22904319, PubMed:22829870). Mitochondrial calcium uptake in skeletal muscle cells is involved in muscle size in adults (By similarity). Regulates synaptic vesicle endocytosis kinetics in central nerve terminal (By similarity). Involved in antigen processing and presentation (By similarity).  
  
Component of the uniplex complex, composed of MCU, MCUB, MICU1, MICU2 and EMRE/SMDT1 (PubMed:24231807). Homooligomer (PubMed:21685886, PubMed:26341627). Forms a pentamer (By similarity). Heterooligomer with CCDC109B/MCUB; this inhibits channel activity (By similarity). Interacts with MICU1; MICU1 acts as an essential regulator for MCU (PubMed:21685886, PubMed:23101630, PubMed:23178883, PubMed:24332854, PubMed:26341627). Interacts with MCUR1 (PubMed:23178883, PubMed:26341627, PubMed:27184846, PubMed:26976564). Interacts with CCDC90B (PubMed:27184846). Interactions with MICU1 and MCUR1 are mutually exclusive (PubMed:23178883). Interacts with MICU2 (PubMed:26341627). Interacts with SLC25A23 (PubMed:24430870).  
  
**Gene Ontology Information:**

Molecular Function

- calcium channel activity
- identical protein binding
- uniporter activity

Location

- calcium channel complex
- integral component of mitochondrial inner membrane
- mitochondrial inner membrane
- mitochondrion
- uniplex complex

Biological process

- actin filament reorganization
- calcium import into the mitochondrion
- calcium-mediated signaling
- glucose homeostasis
- mitochondrial calcium ion homeostasis
- mitochondrial calcium ion transmembrane transport
- positive regulation of insulin secretion
- positive regulation of mitochondrial calcium ion concentration
- positive regulation of mitochondrial fission
- positive regulation of neutrophil chemotaxis
- protein complex oligomerization

---

34

- **Protein name:** Signal transducer and activator of transcription 2
- **Organism:** Homo sapiens
- **Uniprot Accession Number:** P52630
- **Protein sequence length:** 851 aa
- **1D identity (%):** 12.4
- **1D identity (%) [Gaps excluded]:** 28.24
- **1D identity - Alignment Gaps:** 828
- **Common reported functions (%):** 50.0
- **Common reported locations (%):** 0.0
- **Common reported processes (%):** 0.0

- **PDB ID:** 6WCZ
- **Chain:** A
- **Crystallized protein length:** 350 aa
- **Resolution:** 4.0 Å
- **Alinged residues range:** 436-438, 434-438, 392-395, 479-483, 163-173, 224-229
- **Aligned to segment part (indices):** 2, 4, 1, 6, 0, 5
- **Alinged residues range of reference:** 49-51, 205-209, 420-423, 533-537, 772-782, 959-964
- **b-phipsi:** 0.052671
- **w-rdist:** 2.277779
- **t-alpha:** 0.273217
- **Chemical similarity (Tanimoto Index) (%):** 83.13
- **1D identity (%) [PDB]:** 0.07
- **1D identity (%) [Gaps excluded][PDB]:** 100.0
- **1D identity - Alignment Gaps [PDB]:** 1334
- **2D identity (%) [PDB]:** 17.98
- **2D identity (%) [Gaps excluded][PDB]:** 92.2
- **2D identity - Alignment Gaps [PDB]:** 900
- **3D similarity (TM-Score) (%) [PDB]:** 11.81

- **Gene name:** STAT2
- **RefSeq ID:** NM\_198332
- **Transcript sequence length:** 4393
- **5-UTR|CDS|3-UTR identity (%):** 14.98 | 37.06 | 7.24
- **5-UTR|CDS|3-UTR identity (%) [Gaps excluded]:** 72.73 | 78.25 | 79.29
- **5-UTR|CDS|3-UTR identity [Alignment Gaps]:** 212 | 2274 | 1683

**Uniprot Description:**  
  
Signal transducer and activator of transcription that mediates signaling by type I IFNs (IFN-alpha and IFN-beta). Following type I IFN binding to cell surface receptors, Jak kinases (TYK2 and JAK1) are activated, leading to tyrosine phosphorylation of STAT1 and STAT2. The phosphorylated STATs dimerize, associate with IRF9/ISGF3G to form a complex termed ISGF3 transcription factor, that enters the nucleus. ISGF3 binds to the IFN stimulated response element (ISRE) to activate the transcription of interferon stimulated genes, which drive the cell in an antiviral state (PubMed:9020188, PubMed:23391734). Acts as a regulator of mitochondrial fission by modulating the phosphorylation of DNM1L at 'Ser-616' and 'Ser-637' which activate and inactivate the GTPase activity of DNM1L respectively (PubMed:26122121).  
  
Heterodimer with STAT1 upon IFN-alpha/beta induced phosphorylation (By similarity). The heterodimer STAT1:STAT2 forms the interferon-stimulated gene factor 3 complex (ISGF3) with IRF9; interacts with IRF9 in the cytoplasm (By similarity). Interacts with CRSP2 and CRSP6 (PubMed:12509459). Can form a homodimer upon IFN-alpha induced phosphorylation (PubMed:9020188). Interacts with IFNAR1; the interaction requires the phosphorylation of IFNAR1 at 'Tyr-466' (PubMed:9121453). Interacts with IFNAR2 (PubMed:9121453). Interacts with ARL2BP (By similarity). Interacts with E3 ubiquitin ligase DCST1; the interaction results in STAT2 ubiquitin-mediated proteasomal degradation (PubMed:27782195).  
  
**Gene Ontology Information:**

Molecular Function

- DNA-binding transcription factor activity, RNA polymerase II-specific
- identical protein binding
- RNA polymerase II cis-regulatory region sequence-specific DNA binding
- ubiquitin-like protein ligase binding

Location

- chromatin
- cytosol
- nucleoplasm
- plasma membrane

Biological process

- cytokine-mediated signaling pathway
- defense response
- defense response to virus
- negative regulation of type I interferon-mediated signaling pathway
- receptor signaling pathway via JAK-STAT
- regulation of cell population proliferation
- regulation of mitochondrial fission
- regulation of protein phosphorylation
- regulation of transcription by RNA polymerase II
- response to peptide hormone
- type I interferon signaling pathway
- viral process

---

35

- **Protein name:** Genome polyprotein
- **Organism:** Human hepatitis A virus genotype IB (isolate HM175)
- **Uniprot Accession Number:** P08617
- **Protein sequence length:** 2227 aa
- **1D identity (%):** 9.99
- **1D identity (%) [Gaps excluded]:** 28.99
- **1D identity - Alignment Gaps:** 1706
- **Common reported functions (%):** 0.0
- **Common reported locations (%):** 12.5
- **Common reported processes (%):** 10.0

- **PDB ID:** 5WTH
- **Chain:** A
- **Crystallized protein length:** 260 aa
- **Resolution:** 4.2 Å
- **Alinged residues range:** 106-108, 223-226, 115-121, 226-231, 107-112, 25-27
- **Aligned to segment part (indices):** 2, 4, 1, 6, 0, 5
- **Alinged residues range of reference:** 49-51, 198-201, 380-386, 592-597, 716-721, 973-975
- **b-phipsi:** 0.016645
- **w-rdist:** 2.475554
- **t-alpha:** 1.034409
- **Chemical similarity (Tanimoto Index) (%):** 83.58
- **1D identity (%) [PDB]:** 0.16
- **1D identity (%) [Gaps excluded][PDB]:** 66.67
- **1D identity - Alignment Gaps [PDB]:** 1238
- **2D identity (%) [PDB]:** 16.41
- **2D identity (%) [Gaps excluded][PDB]:** 87.76
- **2D identity - Alignment Gaps [PDB]:** 852
- **3D similarity (TM-Score) (%) [PDB]:** 9.58

- **Gene name:** N/A
- **RefSeq ID:** NC\_001489
- **Genomic sequence length:** 7478
- **5-UTR|CDS|3-UTR identity (%):** N/A | 37.4 | N/A
- **5-UTR|CDS|3-UTR identity (%) [Gaps excluded]:** N/A | 79.22 | N/A
- **5-UTR|CDS|3-UTR identity [Alignment Gaps]:** N/A | 3768 | N/A

**Uniprot Description:**  
  
Capsid protein VP1
Capsid proteins VP1, VP2, and VP3 form a closed capsid enclosing the viral positive strand RNA genome (PubMed:25327248, PubMed:28074040). All these proteins contain a beta-sheet structure called beta-barrel jelly roll (PubMed:25327248). Together they form an icosahedral capsid (T=3) composed of 60 copies of each VP1, VP2, and VP3, with a diameter of approximately 300 Angstroms (PubMed:25327248). VP1 is situated at the 12 fivefold axes, whereas VP2 and VP3 are located at the quasi-sixfold axes (PubMed:25327248). The naked capsid interacts with the host receptor HAVCR1 to provide virion attachment to and probably entry into the target cell (PubMed:9658108, PubMed:11134285, PubMed:29437974).  
  
Protein 2B
Homodimer. Homomultimer; probably interacts with membranes in a multimeric form (PubMed:26515753). Seems to assemble into amyloid-like fibers (PubMed:25589659).  
  
**Gene Ontology Information:**

Molecular Function

- ATP binding
- cysteine-type endopeptidase activity
- ion channel activity
- nucleoside-triphosphatase activity
- RNA binding
- RNA helicase activity
- RNA-directed 5'-3' RNA polymerase activity
- structural molecule activity

Location

- host cell cytoplasmic vesicle membrane
- host cell mitochondrial outer membrane
- host multivesicular body
- icosahedral viral capsid
- integral component of membrane
- integral to membrane of host cell
- T=pseudo3 icosahedral viral capsid

Biological process

- negative regulation of toll-like receptor 3 signaling pathway
- pore formation by virus in membrane of host cell
- protein complex oligomerization
- RNA-protein covalent cross-linking
- suppression by virus of host MAVS activity
- transcription, DNA-templated
- viral entry into host cell
- viral RNA genome replication
- virion assembly
- virion attachment to host cell

---

36

- **Protein name:** Nucleoprotein
- **Organism:** Influenza A virus (strain A/Puerto Rico/8/1934 H1N1)
- **Uniprot Accession Number:** P03466
- **Protein sequence length:** 498 aa
- **1D identity (%):** 7.89
- **1D identity (%) [Gaps excluded]:** 29.26
- **1D identity - Alignment Gaps:** 1019
- **Common reported functions (%):** 50.0
- **Common reported locations (%):** 0.0
- **Common reported processes (%):** 10.0

- **PDB ID:** 2WFS
- **Chain:** I
- **Crystallized protein length:** 426 aa
- **Resolution:** 12.0 Å
- **Alinged residues range:** 375-377, 375-381, 139-142, 94-97, 141-154, 224-228
- **Aligned to segment part (indices):** 2, 4, 1, 6, 0, 5
- **Alinged residues range of reference:** 49-51, 206-212, 297-300, 593-596, 761-767, 957-961
- **b-phipsi:** 0.06715
- **w-rdist:** 7.175718
- **t-alpha:** 0.008529
- **Chemical similarity (Tanimoto Index) (%):** 30.08
- **1D identity (%) [PDB]:** 0.0
- **1D identity (%) [Gaps excluded][PDB]:** 0.0
- **1D identity - Alignment Gaps [PDB]:** 1413
- **2D identity (%) [PDB]:** 19.06
- **2D identity (%) [Gaps excluded][PDB]:** 94.12
- **2D identity - Alignment Gaps [PDB]:** 937
- **3D similarity (TM-Score) (%) [PDB]:** 13.57

- **Gene name:** NP
- **RefSeq ID:** NC\_002019
- **Genomic sequence length:** 1565
- **5-UTR|CDS|3-UTR identity (%):** N/A | 26.03 | N/A
- **5-UTR|CDS|3-UTR identity (%) [Gaps excluded]:** N/A | 77.68 | N/A
- **5-UTR|CDS|3-UTR identity [Alignment Gaps]:** N/A | 2649 | N/A

**Uniprot Description:**  
  
Encapsidates the negative strand viral RNA, protecting it from nucleases. The encapsidated genomic RNA is termed the ribonucleoprotein (RNP) and serves as template for transcription and replication. The RNP needs to be localized in the host nucleus to start an infectious cycle, but is too large to diffuse through the nuclear pore complex. NP comprises at least 2 nuclear localization signals that are responsible for the active RNP import into the nucleus through cellular importin alpha/beta pathway. Later in the infection, nclear export of RNPs are mediated through viral proteins NEP interacting with M1 which binds nucleoproteins. It is possible that nucleoprotein binds directly host exportin-1/XPO1 and plays an active role in RNPs nuclear export. M1 interaction with RNP seems to hide nucleoprotein's nuclear localization signals. Soon after a virion infects a new cell, M1 dissociates from the RNP under acidification of the virion driven by M2 protein. Dissociation of M1 from RNP unmasks nucleoprotein's nuclear localization signals, targeting the RNP to the nucleus.  
  
Homomultimerizes to form the nucleocapsid. May bind host exportin-1/XPO1. Binds to viral genomic RNA. Protein-RNA contacts are mediated by a combination of electrostatic interactions between positively charged residues and the phosphate backbone and planar interactions between aromatic side chains and bases.  
  
**Gene Ontology Information:**

Molecular Function

- identical protein binding
- RNA binding
- structural molecule activity

Location

- extracellular region
- helical viral capsid
- host cell nucleus
- viral nucleocapsid

Biological process

- fusion of virus membrane with host plasma membrane
- intracellular transport of virus
- receptor-mediated endocytosis of virus by host cell
- uncoating of virus
- viral budding from plasma membrane
- viral genome maturation
- viral genome packaging
- viral penetration into host nucleus
- viral transcription
- virion assembly
- virion attachment to host cell

---

37

- **Protein name:** Endolysin
- **Organism:** Enterobacteria phage T4
- **Uniprot Accession Number:** P00720
- **Protein sequence length:** 164 aa
- **1D identity (%):** 3.27
- **1D identity (%) [Gaps excluded]:** 27.81
- **1D identity - Alignment Gaps:** 1135
- **Common reported functions (%):** 0.0
- **Common reported locations (%):** 0.0
- **Common reported processes (%):** 0.0

- **PDB ID:** 5T04
- **Chain:** A
- **Crystallized protein length:** 458 aa
- **Resolution:** 3.3 Å
- **Alinged residues range:** 1015-1019, 226-228, 1109-1115, 1025-1028, 1060-1067, 1105-1111
- **Aligned to segment part (indices):** 2, 4, 1, 6, 0, 5
- **Alinged residues range of reference:** 48-52, 208-210, 365-371, 593-596, 775-782, 957-963
- **b-phipsi:** 0.203435
- **w-rdist:** 1.694124
- **t-alpha:** 0.259654
- **Chemical similarity (Tanimoto Index) (%):** 85.61
- **1D identity (%) [PDB]:** 0.07
- **1D identity (%) [Gaps excluded][PDB]:** 50.0
- **1D identity - Alignment Gaps [PDB]:** 1440
- **2D identity (%) [PDB]:** 18.52
- **2D identity (%) [Gaps excluded][PDB]:** 90.61
- **2D identity - Alignment Gaps [PDB]:** 954
- **3D similarity (TM-Score) (%) [PDB]:** 15.83

- **Gene name:** E
- **RefSeq ID:** NC\_000866
- **Genomic sequence length:** 168903
- **5-UTR|CDS|3-UTR identity (%):** N/A | 9.19 | N/A
- **5-UTR|CDS|3-UTR identity (%) [Gaps excluded]:** N/A | 80.18 | N/A
- **5-UTR|CDS|3-UTR identity [Alignment Gaps]:** N/A | 3429 | N/A

**Uniprot Description:**  
  
Endolysin with lysozyme activity that degrades host peptidoglycans and participates with the holin and spanin proteins in the sequential events which lead to the programmed host cell lysis releasing the mature viral particles. Once the holin has permeabilized the host cell membrane, the endolysin can reach the periplasm and break down the peptidoglycan layer.  
  
**Gene Ontology Information:**

Molecular Function

- lysozyme activity

Location

- host cell cytoplasm

Biological process

- cell wall macromolecule catabolic process
- cytolysis
- defense response to bacterium
- peptidoglycan catabolic process
- viral release from host cell by cytolysis

---

38

- **Protein name:** Polyhedrin
- **Organism:** Autographa californica nuclear polyhedrosis virus
- **Uniprot Accession Number:** P04871
- **Protein sequence length:** 245 aa
- **1D identity (%):** 3.76
- **1D identity (%) [Gaps excluded]:** 26.6
- **1D identity - Alignment Gaps:** 1142
- **Common reported functions (%):** 50.0
- **Common reported locations (%):** 0.0
- **Common reported processes (%):** 0.0

- **PDB ID:** 2WUX
- **Chain:** A
- **Crystallized protein length:** 213 aa
- **Resolution:** 1.84 Å
- **Alinged residues range:** 19-23, 20-23, 14-22, 206-209, 208-221, 24-27
- **Aligned to segment part (indices):** 2, 4, 1, 6, 0, 5
- **Alinged residues range of reference:** 49-53, 204-207, 311-317, 596-599, 720-727, 977-980
- **b-phipsi:** 0.001773
- **w-rdist:** 8.739336
- **t-alpha:** 0.256308
- **Chemical similarity (Tanimoto Index) (%):** 82.78
- **1D identity (%) [PDB]:** 0.17
- **1D identity (%) [Gaps excluded][PDB]:** 100.0
- **1D identity - Alignment Gaps [PDB]:** 1194
- **2D identity (%) [PDB]:** 14.42
- **2D identity (%) [Gaps excluded][PDB]:** 90.3
- **2D identity - Alignment Gaps [PDB]:** 868
- **3D similarity (TM-Score) (%) [PDB]:** 10.01

- **Gene name:** PH
- **RefSeq ID:** NC\_001623
- **Genomic sequence length:** 133894
- **5-UTR|CDS|3-UTR identity (%):** N/A | 11.75 | N/A
- **5-UTR|CDS|3-UTR identity (%) [Gaps excluded]:** N/A | 78.32 | N/A
- **5-UTR|CDS|3-UTR identity [Alignment Gaps]:** N/A | 3370 | N/A

**Uniprot Description:**  
  
Major component of the virus occlusion bodies which are large proteinaceous structures termed polyhedra. These structures serve as the protective package for the virus particles outside the infected host and allow natural transmission of virus between insect hosts, assisting persistence in the environment. Forms the paracrystalline lattice of polyhedra and interacts with enveloped virions as well as other accessory molecules and structures to form a mature viral occlusion body.  
  
**Gene Ontology Information:**

Molecular Function

- identical protein binding
- structural molecule activity

Location

- host cell nuclear matrix
- viral occlusion body

Biological process  
  
N/A

---

39

- **Protein name:** Hemagglutinin
- **Organism:** Influenza A virus (strain A/Puerto Rico/8/1934 H1N1)
- **Uniprot Accession Number:** P03452
- **Protein sequence length:** 565 aa
- **1D identity (%):** 8.8
- **1D identity (%) [Gaps excluded]:** 23.03
- **1D identity - Alignment Gaps:** 822
- **Common reported functions (%):** 50.0
- **Common reported locations (%):** 50.0
- **Common reported processes (%):** 30.0

- **PDB ID:** 1RVX
- **Chain:** B
- **Crystallized protein length:** 160 aa
- **Resolution:** 2.2 Å
- **Alinged residues range:** 527-532, 654-660, 654-658, 614-622, 609-616
- **Aligned to segment part (indices):** 4, 1, 6, 0, 5
- **Alinged residues range of reference:** 195-200, 420-426, 603-607, 773-781, 957-964
- **b-phipsi:** 0.097333
- **w-rdist:** 1.055326
- **t-alpha:** 0.508772
- **Chemical similarity (Tanimoto Index) (%):** 89.49
- **1D identity (%) [PDB]:** 0.0
- **1D identity (%) [Gaps excluded][PDB]:** 0.0
- **1D identity - Alignment Gaps [PDB]:** 1143
- **2D identity (%) [PDB]:** 12.46
- **2D identity (%) [Gaps excluded][PDB]:** 95.45
- **2D identity - Alignment Gaps [PDB]:** 879
- **3D similarity (TM-Score) (%) [PDB]:** 10.86

- **Gene name:** HA
- **RefSeq ID:** NC\_002017
- **Genomic sequence length:** 1778
- **5-UTR|CDS|3-UTR identity (%):** N/A | 29.7 | N/A
- **5-UTR|CDS|3-UTR identity (%) [Gaps excluded]:** N/A | 80.17 | N/A
- **5-UTR|CDS|3-UTR identity [Alignment Gaps]:** N/A | 2537 | N/A

**Uniprot Description:**  
  
Binds to sialic acid-containing receptors on the cell surface, bringing about the attachment of the virus particle to the cell. This attachment induces virion internalization either through clathrin-dependent endocytosis or through clathrin- and caveolin-independent pathway. Plays a major role in the determination of host range restriction and virulence. Class I viral fusion protein. Responsible for penetration of the virus into the cell cytoplasm by mediating the fusion of the membrane of the endocytosed virus particle with the endosomal membrane. Low pH in endosomes induces an irreversible conformational change in HA2, releasing the fusion hydrophobic peptide. Several trimers are required to form a competent fusion pore.  
  
Homotrimer of disulfide-linked HA1-HA2. Interacts with human CACNA1C (PubMed:29779930).  
  
**Gene Ontology Information:**

Molecular Function

- host cell surface receptor binding

Location

- extracellular region
- host cell plasma membrane
- integral component of membrane
- viral envelope
- virion membrane

Biological process

- clathrin-dependent endocytosis of virus by host cell
- fusion of virus membrane with host endosome membrane
- fusion of virus membrane with host plasma membrane
- intracellular transport of viral protein in host cell
- receptor-mediated endocytosis of virus by host cell
- regulation of immune response
- uncoating of virus
- viral budding from plasma membrane
- viral genome packaging
- viral protein processing
- virion assembly
- virion attachment to host cell

---

40

- **Protein name:** Envelope glycoprotein H
- **Organism:** Human herpesvirus 8
- **Uniprot Accession Number:** Q98142
- **Protein sequence length:** 730 aa
- **1D identity (%):** 12.33
- **1D identity (%) [Gaps excluded]:** 27.76
- **1D identity - Alignment Gaps:** 771
- **Common reported functions (%):** 0.0
- **Common reported locations (%):** 50.0
- **Common reported processes (%):** 10.0

- **PDB ID:** 7CZF
- **Chain:** E
- **Crystallized protein length:** 642 aa
- **Resolution:** 3.2 Å
- **Alinged residues range:** 643-645, 46-48, 687-693, 576-579, 639-644, 542-545
- **Aligned to segment part (indices):** 2, 4, 1, 6, 0, 5
- **Alinged residues range of reference:** 50-52, 228-230, 281-287, 596-599, 691-696, 957-960
- **b-phipsi:** 0.055117
- **w-rdist:** 4.456155
- **t-alpha:** 0.055804
- **Chemical similarity (Tanimoto Index) (%):** N/A
- **1D identity (%) [PDB]:** 0.06
- **1D identity (%) [Gaps excluded][PDB]:** 100.0
- **1D identity - Alignment Gaps [PDB]:** 1624
- **2D identity (%) [PDB]:** 32.06
- **2D identity (%) [Gaps excluded][PDB]:** 91.47
- **2D identity - Alignment Gaps [PDB]:** 782
- **3D similarity (TM-Score) (%) [PDB]:** 9.51

- **Gene name:** ORF22
- **RefSeq ID:** NC\_009333
- **Genomic sequence length:** 137969
- **5-UTR|CDS|3-UTR identity (%):** N/A | 33.92 | N/A
- **5-UTR|CDS|3-UTR identity (%) [Gaps excluded]:** N/A | 78.56 | N/A
- **5-UTR|CDS|3-UTR identity [Alignment Gaps]:** N/A | 2387 | N/A

**Uniprot Description:**  
  
The heterodimer glycoprotein H-glycoprotein L is required for the fusion of viral and plasma membranes leading to virus entry into the host cell. Following initial binding to host receptor, membrane fusion is mediated by the fusion machinery composed of gB and the heterodimer gH/gL. May also be involved in the fusion between the virion envelope and the outer nuclear membrane during virion morphogenesis.  
  
Interacts with glycoprotein L (gL); this interaction is necessary for the correct processing and cell surface expression of gH. The heterodimer gH/gL seems to interact with gB trimers during fusion.  
  
**Gene Ontology Information:**

Molecular Function  
  
N/A

Location

- host cell endosome membrane
- host cell plasma membrane
- integral component of membrane
- viral envelope
- virion membrane

Biological process

- fusion of virus membrane with host plasma membrane

---

41

- **Protein name:** Major capsid protein P2
- **Organism:** Pseudoalteromonas phage PM2
- **Uniprot Accession Number:** P15794
- **Protein sequence length:** 269 aa
- **1D identity (%):** 2.15
- **1D identity (%) [Gaps excluded]:** 30.1
- **1D identity - Alignment Gaps:** 1336
- **Common reported functions (%):** 0.0
- **Common reported locations (%):** 0.0
- **Common reported processes (%):** 0.0

- **PDB ID:** 2VVF
- **Chain:** E
- **Crystallized protein length:** 269 aa
- **Resolution:** 2.5 Å
- **Alinged residues range:** 56-60, 188-192, 155-160, 184-191, 200-205, 207-209
- **Aligned to segment part (indices):** 2, 4, 1, 6, 0, 5
- **Alinged residues range of reference:** 48-52, 196-200, 309-314, 538-545, 773-778, 977-979
- **b-phipsi:** 0.010605
- **w-rdist:** 9.746253
- **t-alpha:** 0.049945
- **Chemical similarity (Tanimoto Index) (%):** 82.13
- **1D identity (%) [PDB]:** 0.0
- **1D identity (%) [Gaps excluded][PDB]:** 0.0
- **1D identity - Alignment Gaps [PDB]:** 1252
- **2D identity (%) [PDB]:** 20.37
- **2D identity (%) [Gaps excluded][PDB]:** 87.71
- **2D identity - Alignment Gaps [PDB]:** 780
- **3D similarity (TM-Score) (%) [PDB]:** 9.31

- **Gene name:** II
- **RefSeq ID:** NC\_000867
- **Genomic sequence length:** 10079
- **5-UTR|CDS|3-UTR identity (%):** N/A | 15.15 | N/A
- **5-UTR|CDS|3-UTR identity (%) [Gaps excluded]:** N/A | 80.74 | N/A
- **5-UTR|CDS|3-UTR identity [Alignment Gaps]:** N/A | 3168 | N/A

**Uniprot Description:**  
  
Major capsid protein.  
  
Homotrimer.  
  
**Gene Ontology Information:**

Molecular Function  
  
N/A

Location

- viral capsid, major subunit
- virion

Biological process  
  
N/A

---

42

- **Protein name:** 40S ribosomal protein S11
- **Organism:** Homo sapiens
- **Uniprot Accession Number:** P62280
- **Protein sequence length:** 158 aa
- **1D identity (%):** 3.68
- **1D identity (%) [Gaps excluded]:** 30.72
- **1D identity - Alignment Gaps:** 1125
- **Common reported functions (%):** 0.0
- **Common reported locations (%):** 0.0
- **Common reported processes (%):** 0.0

- **PDB ID:** 5FLX
- **Chain:** L
- **Crystallized protein length:** 153 aa
- **Resolution:** 3.9 Å
- **Alinged residues range:** 142-144, 91-93, 127-130, 74-81, 124-129, 14-16
- **Aligned to segment part (indices):** 2, 4, 1, 6, 0, 5
- **Alinged residues range of reference:** 47-49, 224-226, 311-314, 549-556, 662-667, 974-976
- **b-phipsi:** 0.00152
- **w-rdist:** 8.000573
- **t-alpha:** 0.72
- **Chemical similarity (Tanimoto Index) (%):** N/A
- **1D identity (%) [PDB]:** 0.0
- **1D identity (%) [Gaps excluded][PDB]:** 0.0
- **1D identity - Alignment Gaps [PDB]:** 1136
- **2D identity (%) [PDB]:** 10.38
- **2D identity (%) [Gaps excluded][PDB]:** 84.68
- **2D identity - Alignment Gaps [PDB]:** 888
- **3D similarity (TM-Score) (%) [PDB]:** 8.86

- **Gene name:** RPS11
- **RefSeq ID:** NM\_001015
- **Transcript sequence length:** 68689
- **5-UTR|CDS|3-UTR identity (%):** 30.75 | 3.61 | 0.24
- **5-UTR|CDS|3-UTR identity (%) [Gaps excluded]:** 75.51 | 77.35 | 84.77
- **5-UTR|CDS|3-UTR identity [Alignment Gaps]:** 214 | 3694 | 68047

**Uniprot Description:**  
  
N/A  
  
**Gene Ontology Information:**

Molecular Function

- RNA binding
- rRNA binding
- structural constituent of ribosome

Location

- cytoplasm
- cytosol
- cytosolic ribosome
- cytosolic small ribosomal subunit
- extracellular exosome
- focal adhesion
- membrane
- nucleolus
- nucleoplasm
- ribosome

Biological process

- cytoplasmic translation
- nuclear-transcribed mRNA catabolic process, nonsense-mediated decay
- SRP-dependent cotranslational protein targeting to membrane
- translation
- translational initiation
- viral transcription

---

43

- **Protein name:** Plexin-C1
- **Organism:** Homo sapiens
- **Uniprot Accession Number:** O60486
- **Protein sequence length:** 1568 aa
- **1D identity (%):** 15.09
- **1D identity (%) [Gaps excluded]:** 28.43
- **1D identity - Alignment Gaps:** 871
- **Common reported functions (%):** 0.0
- **Common reported locations (%):** 0.0
- **Common reported processes (%):** 0.0

- **PDB ID:** 3NVN
- **Chain:** B
- **Crystallized protein length:** 474 aa
- **Resolution:** 2.26 Å
- **Alinged residues range:** 250-252, 414-418, 404-421, 162-167, 378-383, 463-466
- **Aligned to segment part (indices):** 2, 4, 1, 6, 0, 5
- **Alinged residues range of reference:** 50-52, 202-206, 425-440, 594-599, 721-726, 979-982
- **b-phipsi:** 0.001666
- **w-rdist:** 8.716052
- **t-alpha:** 0.455385
- **Chemical similarity (Tanimoto Index) (%):** N/A
- **1D identity (%) [PDB]:** 0.07
- **1D identity (%) [Gaps excluded][PDB]:** 50.0
- **1D identity - Alignment Gaps [PDB]:** 1453
- **2D identity (%) [PDB]:** 27.02
- **2D identity (%) [Gaps excluded][PDB]:** 84.18
- **2D identity - Alignment Gaps [PDB]:** 749
- **3D similarity (TM-Score) (%) [PDB]:** 13.25

- **Gene name:** PLXNC1
- **RefSeq ID:** NM\_005761
- **Transcript sequence length:** 7492
- **5-UTR|CDS|3-UTR identity (%):** 34.68 | 36.91 | 6.99
- **5-UTR|CDS|3-UTR identity (%) [Gaps excluded]:** 72.77 | 78.45 | 77.42
- **5-UTR|CDS|3-UTR identity [Alignment Gaps]:** 234 | 3071 | 2185

**Uniprot Description:**  
  
Receptor for SEMA7A, for smallpox semaphorin A39R, vaccinia virus semaphorin A39R and for herpesvirus Sema protein. Binding of semaphorins triggers cellular responses leading to the rearrangement of the cytoskeleton and to secretion of IL6 and IL8 (By similarity).  
  
Monomer. Homodimer. Interacts with SEMA7A.  
  
**Gene Ontology Information:**

Molecular Function

- semaphorin receptor activity
- signaling receptor binding

Location

- integral component of plasma membrane
- membrane
- plasma membrane
- semaphorin receptor complex

Biological process

- cell adhesion
- negative regulation of cell adhesion
- positive regulation of axonogenesis
- regulation of cell migration
- regulation of cell shape
- regulation of GTPase activity
- semaphorin-plexin signaling pathway involved in axon guidance

---

44

- **Protein name:** Angiotensin-converting enzyme 2
- **Organism:** Homo sapiens
- **Uniprot Accession Number:** Q9BYF1
- **Protein sequence length:** 805 aa
- **1D identity (%):** 13.33
- **1D identity (%) [Gaps excluded]:** 30.09
- **1D identity - Alignment Gaps:** 802
- **Common reported functions (%):** 50.0
- **Common reported locations (%):** 37.5
- **Common reported processes (%):** 20.0

- **PDB ID:** 3D0H
- **Chain:** B
- **Crystallized protein length:** 597 aa
- **Resolution:** 3.1 Å
- **Alinged residues range:** 137-141, 350-352, 463-471, 335-345, 545-572, 570-573
- **Aligned to segment part (indices):** 2, 4, 1, 6, 0, 5
- **Alinged residues range of reference:** 49-53, 228-230, 418-428, 529-536, 744-774, 977-980
- **b-phipsi:** 0.111886
- **w-rdist:** 7.427861
- **t-alpha:** 0.005313
- **Chemical similarity (Tanimoto Index) (%):** 92.25
- **1D identity (%) [PDB]:** 0.06
- **1D identity (%) [Gaps excluded][PDB]:** 100.0
- **1D identity - Alignment Gaps [PDB]:** 1578
- **2D identity (%) [PDB]:** 15.8
- **2D identity (%) [Gaps excluded][PDB]:** 91.81
- **2D identity - Alignment Gaps [PDB]:** 1116
- **3D similarity (TM-Score) (%) [PDB]:** 15.68

- **Gene name:** ACE2
- **RefSeq ID:** NM\_021804
- **Transcript sequence length:** 3596
- **5-UTR|CDS|3-UTR identity (%):** 38.79 | 37.72 | 17.59
- **5-UTR|CDS|3-UTR identity (%) [Gaps excluded]:** 76.56 | 77.61 | 77.83
- **5-UTR|CDS|3-UTR identity [Alignment Gaps]:** 187 | 2158 | 695

**Uniprot Description:**  
  
Essential counter-regulatory carboxypeptidase of the renin-angiotensin hormone system that is a critical regulator of blood volume, systemic vascular resistance, and thus cardiovascular homeostasis (PubMed:27217402). Converts angiotensin I to angiotensin 1-9, a nine-amino acid peptide with anti-hypertrophic effects in cardiomyocytes, and angiotensin II to angiotensin 1-7, which then acts as a beneficial vasodilator and anti-proliferation agent, counterbalancing the actions of the vasoconstrictor angiotensin II (PubMed:10969042, PubMed:10924499, PubMed:11815627, PubMed:19021774, PubMed:14504186). Also removes the C-terminal residue from three other vasoactive peptides, neurotensin, kinetensin, and des-Arg bradykinin, but is not active on bradykinin (PubMed:10969042, PubMed:11815627). Also cleaves other biological peptides, such as apelins (apelin-13, [Pyr1]apelin-13, apelin-17, apelin-36), casomorphins (beta-casomorphin-7, neocasomorphin) and dynorphin A with high efficiency (PubMed:11815627, PubMed:27217402, PubMed:28293165). In addition, ACE2 C-terminus is homologous to collectrin and is responsible for the trafficking of the neutral amino acid transporter SL6A19 to the plasma membrane of gut epithelial cells via direct interaction, regulating its expression on the cell surface and its catalytic activity (PubMed:18424768, PubMed:19185582).  
  
Homodimer (PubMed:32132184). Interacts with the catalytically active form of TMPRSS2 (PubMed:21068237). Interacts with SLC6A19; this interaction is essential for expression and function of SLC6A19 in intestine (By similarity). Interacts with ITGA5:ITGB1 (PubMed:15276642, PubMed:33102950).  
  
**Gene Ontology Information:**

Molecular Function

- carboxypeptidase activity
- endopeptidase activity
- identical protein binding
- metallocarboxypeptidase activity
- metallopeptidase activity
- peptidyl-dipeptidase activity
- virus receptor activity
- zinc ion binding

Location

- apical plasma membrane
- brush border membrane
- cell surface
- cilium
- endocytic vesicle membrane
- endoplasmic reticulum lumen
- extracellular exosome
- extracellular region
- extracellular space
- integral component of membrane
- membrane raft
- plasma membrane

Biological process

- angiotensin maturation
- angiotensin-mediated drinking behavior
- blood vessel diameter maintenance
- negative regulation of signaling receptor activity
- positive regulation of amino acid transport
- positive regulation of cardiac muscle contraction
- positive regulation of gap junction assembly
- positive regulation of L-proline import across plasma membrane
- positive regulation of reactive oxygen species metabolic process
- proteolysis
- receptor-mediated virion attachment to host cell
- regulation of cardiac conduction
- regulation of cell population proliferation
- regulation of cytokine production
- regulation of inflammatory response
- regulation of systemic arterial blood pressure by renin-angiotensin
- regulation of transmembrane transporter activity
- regulation of vasoconstriction
- tryptophan transport
- viral entry into host cell

---

45

- **Protein name:** Cyclin-dependent kinase 9
- **Organism:** Homo sapiens
- **Uniprot Accession Number:** P50750
- **Protein sequence length:** 372 aa
- **1D identity (%):** 5.24
- **1D identity (%) [Gaps excluded]:** 29.08
- **1D identity - Alignment Gaps:** 1143
- **Common reported functions (%):** 0.0
- **Common reported locations (%):** 0.0
- **Common reported processes (%):** 0.0

- **PDB ID:** 4OGR
- **Chain:** E
- **Crystallized protein length:** 310 aa
- **Resolution:** 3.0 Å
- **Alinged residues range:** 19-21, 81-83, 285-289, 40-43, 234-237, 288-293
- **Aligned to segment part (indices):** 2, 4, 1, 6, 0, 5
- **Alinged residues range of reference:** 51-53, 226-228, 364-368, 603-606, 774-777, 958-964
- **b-phipsi:** 0.026235
- **w-rdist:** 8.132072
- **t-alpha:** 0.037281
- **Chemical similarity (Tanimoto Index) (%):** 85.84
- **1D identity (%) [PDB]:** 0.0
- **1D identity (%) [Gaps excluded][PDB]:** 0.0
- **1D identity - Alignment Gaps [PDB]:** 1296
- **2D identity (%) [PDB]:** 18.07
- **2D identity (%) [Gaps excluded][PDB]:** 89.86
- **2D identity - Alignment Gaps [PDB]:** 862
- **3D similarity (TM-Score) (%) [PDB]:** 10.53

- **Gene name:** CDK9
- **RefSeq ID:** NM\_001261
- **Transcript sequence length:** 338605
- **5-UTR|CDS|3-UTR identity (%):** 22.7 | 17.89 | 0.05
- **5-UTR|CDS|3-UTR identity (%) [Gaps excluded]:** 65.31 | 80.42 | 83.33
- **5-UTR|CDS|3-UTR identity [Alignment Gaps]:** 184 | 3143 | 337180

**Uniprot Description:**  
  
Protein kinase involved in the regulation of transcription (PubMed:10574912, PubMed:10757782, PubMed:11145967, PubMed:11575923, PubMed:11809800, PubMed:11884399, PubMed:14701750, PubMed:16109376, PubMed:16109377, PubMed:20930849, PubMed:28426094). Member of the cyclin-dependent kinase pair (CDK9/cyclin-T) complex, also called positive transcription elongation factor b (P-TEFb), which facilitates the transition from abortive to productive elongation by phosphorylating the CTD (C-terminal domain) of the large subunit of RNA polymerase II (RNAP II) POLR2A, SUPT5H and RDBP (PubMed:10574912, PubMed:10757782, PubMed:11145967, PubMed:11575923, PubMed:11809800, PubMed:11884399, PubMed:14701750, PubMed:16109376, PubMed:16109377, PubMed:20930849, PubMed:28426094). This complex is inactive when in the 7SK snRNP complex form (PubMed:10574912, PubMed:10757782, PubMed:11145967, PubMed:11575923, PubMed:11809800, PubMed:11884399, PubMed:14701750, PubMed:16109376, PubMed:16109377, PubMed:20930849, PubMed:28426094). Phosphorylates EP300, MYOD1, RPB1/POLR2A and AR and the negative elongation factors DSIF and NELF (PubMed:9857195, PubMed:10912001, PubMed:11112772, PubMed:12037670, PubMed:20081228, PubMed:20980437, PubMed:21127351). Regulates cytokine inducible transcription networks by facilitating promoter recognition of target transcription factors (e.g. TNF-inducible RELA/p65 activation and IL-6-inducible STAT3 signaling) (PubMed:17956865, PubMed:18362169). Promotes RNA synthesis in genetic programs for cell growth, differentiation and viral pathogenesis (PubMed:10393184, PubMed:11112772). P-TEFb is also involved in cotranscriptional histone modification, mRNA processing and mRNA export (PubMed:15564463, PubMed:19575011, PubMed:19844166). Modulates a complex network of chromatin modifications including histone H2B monoubiquitination (H2Bub1), H3 lysine 4 trimethylation (H3K4me3) and H3K36me3; integrates phosphorylation during transcription with chromatin modifications to control co-transcriptional histone mRNA processing (PubMed:15564463, PubMed:19575011, PubMed:19844166). The CDK9/cyclin-K complex has also a kinase activity towards CTD of RNAP II and can substitute for CDK9/cyclin-T P-TEFb in vitro (PubMed:21127351). Replication stress response protein; the CDK9/cyclin-K complex is required for genome integrity maintenance, by promoting cell cycle recovery from replication arrest and limiting single-stranded DNA amount in response to replication stress, thus reducing the breakdown of stalled replication forks and avoiding DNA damage (PubMed:20493174). In addition, probable function in DNA repair of isoform 2 via interaction with KU70/XRCC6 (PubMed:20493174). Promotes cardiac myocyte enlargement (PubMed:20081228). RPB1/POLR2A phosphorylation on 'Ser-2' in CTD activates transcription (PubMed:21127351). AR phosphorylation modulates AR transcription factor promoter selectivity and cell growth. DSIF and NELF phosphorylation promotes transcription by inhibiting their negative effect (PubMed:9857195, PubMed:10912001, PubMed:11112772). The phosphorylation of MYOD1 enhances its transcriptional activity and thus promotes muscle differentiation (PubMed:12037670).  
  
Component of the super elongation complex (SEC), at least composed of EAF1, EAF2, CDK9, MLLT3/AF9, AFF (AFF1 or AFF4), the P-TEFb complex and ELL (ELL, ELL2 or ELL3). Associates with CCNT1/cyclin-T1, CCNT2/cyclin-T2 (isoform A and isoform B) or CCNK/cyclin-K to form active P-TEFb. P-TEFb forms a complex with AFF4/AF5Q31 and is part of the super elongation complex (SEC). Component of a complex which is composed of at least 5 members: HTATSF1/Tat-SF1, P-TEFb complex, RNA pol II, SUPT5H and NCL/nucleolin. Associates with UBR5 and forms a transcription regulatory complex composed of CDK9, RNAP II, UBR5 and TFIIS/TCEA1 that can stimulate target gene transcription (e.g. gamma fibrinogen/FGG) by recruiting their promoters. Component of the 7SK snRNP inactive complex which is composed of at least 8 members: P-TEFb (composed of CDK9 and CCNT1/cyclin-T1), HEXIM1, HEXIM2, LARP7, BCDIN3, SART3 proteins and 7SK and U6 snRNAs. This inactive 7SK snRNP complex can also interact with NCOR1 and HDAC3, probably to regulate CDK9 acetylation. Release of P-TEFb from P-TEFb/7SK snRNP complex requires both PP2B to transduce calcium Ca(2+) signaling in response to stimuli (e.g. UV or hexamethylene bisacetamide (HMBA)) and PPP1CA to dephosphorylate Thr-186. This released P-TEFb remains inactive in the pre-initiation complex with BRD4 until new Thr-186 phosphorylation occurs after the synthesis of a short RNA (PubMed:10393184, PubMed:10574912, PubMed:12037670, PubMed:11884399, PubMed:12065898, PubMed:12718890, PubMed:15965233, PubMed:16109376, PubMed:17452463, PubMed:17643375, PubMed:18249148, PubMed:18483222, PubMed:18566585, PubMed:20159561, PubMed:20471948, PubMed:21127351, PubMed:21779453, PubMed:22195968, PubMed:9491887). Interacts with BRD4; to target chromatin binding (PubMed:16109376, PubMed:16109377, PubMed:18483222). Interacts with JMJD6 (PubMed:24360279). Interacts with activated nuclear STAT3 and RELA/p65 (PubMed:17956865, PubMed:18362169). Binds to AR and MYOD1 (PubMed:12037670, PubMed:20980437). Forms a complex composed of CDK9, CCNT1/cyclin-T1, EP300 and GATA4 that stimulates hypertrophy in cardiomyocytes (PubMed:20081228). The large PER complex involved in the repression of transcriptional termination is composed of at least PER2, CDK9, DDX5, DHX9, NCBP1 and POLR2A (By similarity). Interacts with HSF1 (PubMed:27189267). Interacts with TBX21 (By similarity). Isoform 3: binds to KU70/XRCC6 (PubMed:20535204). Interacts with WDR43 (By similarity).  
  
**Gene Ontology Information:**

Molecular Function

- 7SK snRNA binding
- ATP binding
- chromatin binding
- cyclin-dependent protein serine/threonine kinase activity
- DNA binding
- protein kinase activity
- protein kinase binding
- protein serine kinase activity
- protein serine/threonine kinase activity
- protein serine/threonine kinase activity
- RNA polymerase II cis-regulatory region sequence-specific DNA binding
- RNA polymerase II CTD heptapeptide repeat kinase activity
- transcription coactivator binding

Location

- cyclin/CDK positive transcription elongation factor complex
- cytoplasmic ribonucleoprotein granule
- mediator complex
- membrane
- nucleoplasm
- nucleus
- P-TEFb complex
- PML body
- transcription elongation factor complex

Biological process

- cell population proliferation
- cellular response to cytokine stimulus
- DNA repair
- negative regulation of cell cycle arrest
- negative regulation of mRNA polyadenylation
- phosphorylation of RNA polymerase II C-terminal domain
- positive regulation of cardiac muscle hypertrophy
- positive regulation of histone H2B ubiquitination
- positive regulation of histone phosphorylation
- positive regulation of mRNA 3'-UTR binding
- positive regulation of transcription by RNA polymerase II
- positive regulation of viral transcription
- protein phosphorylation
- regulation of DNA repair
- regulation of histone modification
- regulation of muscle cell differentiation
- replication fork processing
- response to drug
- snRNA transcription by RNA polymerase II
- transcription by RNA polymerase II
- transcription elongation from RNA polymerase II promoter
- transcription initiation from RNA polymerase II promoter

---

46

- **Protein name:** HLA class II histocompatibility antigen, DRB1 beta chain
- **Organism:** Homo sapiens
- **Uniprot Accession Number:** P01911
- **Protein sequence length:** 266 aa
- **1D identity (%):** 3.75
- **1D identity (%) [Gaps excluded]:** 28.49
- **1D identity - Alignment Gaps:** 1181
- **Common reported functions (%):** 0.0
- **Common reported locations (%):** 12.5
- **Common reported processes (%):** 0.0

- **PDB ID:** 2IPK
- **Chain:** B
- **Crystallized protein length:** 190 aa
- **Resolution:** 2.3 Å
- **Alinged residues range:** 161-163, 156-161, 155-159, 142-146, 99-103, 68-70
- **Aligned to segment part (indices):** 2, 4, 1, 6, 0, 5
- **Alinged residues range of reference:** 48-50, 224-229, 397-401, 597-601, 720-724, 977-979
- **b-phipsi:** 0.001354
- **w-rdist:** 8.30698
- **t-alpha:** 0.979079
- **Chemical similarity (Tanimoto Index) (%):** 85.59
- **1D identity (%) [PDB]:** 0.09
- **1D identity (%) [Gaps excluded][PDB]:** 100.0
- **1D identity - Alignment Gaps [PDB]:** 1171
- **2D identity (%) [PDB]:** 14.38
- **2D identity (%) [Gaps excluded][PDB]:** 87.88
- **2D identity - Alignment Gaps [PDB]:** 843
- **3D similarity (TM-Score) (%) [PDB]:** 7.99

- **Gene name:** HLA-DRB1
- **RefSeq ID:** N/A
- **Sequence length:** N/A
- **5-UTR|CDS|3-UTR identity (%):** N/A | N/A | N/A
- **5-UTR|CDS|3-UTR identity (%) [Gaps excluded]:** N/A | N/A | N/A
- **5-UTR|CDS|3-UTR identity [Alignment Gaps]:** N/A | N/A | N/A

**Uniprot Description:**  
  
A beta chain of antigen-presenting major histocompatibility complex class II (MHCII) molecule. In complex with the alpha chain HLA-DRA, displays antigenic peptides on professional antigen presenting cells (APCs) for recognition by alpha-beta T cell receptor (TCR) on HLA-DRB1-restricted CD4-positive T cells. This guides antigen-specific T-helper effector functions, both antibody-mediated immune response and macrophage activation, to ultimately eliminate the infectious agents and transformed cells (PubMed:29884618, PubMed:22327072, PubMed:27591323, PubMed:8642306, PubMed:15265931, PubMed:31495665, PubMed:16148104). Typically presents extracellular peptide antigens of 10 to 30 amino acids that arise from proteolysis of endocytosed antigens in lysosomes (PubMed:8145819). In the tumor microenvironment, presents antigenic peptides that are primarily generated in tumor-resident APCs likely via phagocytosis of apoptotic tumor cells or macropinocytosis of secreted tumor proteins (PubMed:31495665). Presents peptides derived from intracellular proteins that are trapped in autolysosomes after macroautophagy, a mechanism especially relevant for T cell selection in the thymus and central immune tolerance (PubMed:17182262, PubMed:23783831). The selection of the immunodominant epitopes follows two processing modes: 'bind first, cut/trim later' for pathogen-derived antigenic peptides and 'cut first, bind later' for autoantigens/self-peptides (PubMed:25413013). The anchor residue at position 1 of the peptide N-terminus, usually a large hydrophobic residue, is essential for high affinity interaction with MHCII molecules (PubMed:8145819).  
  
Heterotrimer that consists of an alpha chain HLA-DRA, a beta chain HLA-DRB1 and a peptide (peptide-MHCII) (PubMed:7477400, PubMed:9354468, PubMed:9782128, PubMed:31619516, PubMed:32668259). Newly synthesized alpha and beta chains forms a heterodimer (MHCII) that associates with the CD74/invariant chain (Ii) in the endoplasmic reticulum (ER). Ii is a trimer composed of three subunits and each subunit interacts with one MHCII dimer, blocking the peptide-binding cleft. As a result, MHCII molecules can not bind peptides present in the ER (PubMed:7479981). The complex of MHCII and CD74/Ii is transported in vesicles from ER to Golgi to lysosomes, where it encounters antigenic peptides generated via proteolysis of endocytosed antigens. MHCII dimers are dissociated from CD74/Ii by the combined action of proteolysis and HLA-DM (PubMed:25413013). Lysosomal enzymes such as cathepsin, degrade CD74/Ii leaving a 24 amino acid remnant called class II-associated Ii or CLIP. Interacts (via the peptide binding cleft) with CLIP; this interaction inhibits antigen peptide binding before entry in the endosomal compartment (PubMed:7477400, PubMed:9075930). The displacement of CLIP and replacement by a high affinity peptide in lysosomes is performed by HLA-DM heterodimer. HLA-DM catalyzes CLIP dissociation from MHCII, stabilizes empty MHCII and mediates the selection of high affinity peptides (PubMed:23260142, PubMed:11070170, PubMed:9075930). Interacts with HLA-DM heterodimer; this interaction is direct (PubMed:25413013). Interacts with TCR (via CDR3) (PubMed:29884618). Interacts (via beta-2 domain) with CD4 coreceptor (via Ig-like V-type domain); this interaction is of exceptionally low affinity yet necessary for optimal recognition of antigenic peptides (PubMed:21900604, PubMed:27114505).  
  
**Gene Ontology Information:**

Molecular Function

- CD4 receptor binding
- MHC class II protein complex binding
- MHC class II receptor activity
- peptide antigen binding
- polysaccharide binding
- structural constituent of cytoskeleton
- T cell receptor binding

Location

- cell surface
- clathrin-coated endocytic vesicle membrane
- endocytic vesicle membrane
- ER to Golgi transport vesicle membrane
- external side of plasma membrane
- extracellular exosome
- extracellular space
- Golgi membrane
- immunological synapse
- integral component of lumenal side of endoplasmic reticulum membrane
- integral component of plasma membrane
- intermediate filament
- late endosome membrane
- lysosomal membrane
- membrane
- MHC class II protein complex
- plasma membrane
- trans-Golgi network membrane
- transport vesicle membrane

Biological process

- antigen processing and presentation of endogenous peptide antigen via MHC class II
- antigen processing and presentation of exogenous peptide antigen via MHC class II
- detection of bacterium
- epidermis development
- humoral immune response
- immune response
- inflammatory response to antigenic stimulus
- interferon-gamma-mediated signaling pathway
- macrophage differentiation
- myeloid dendritic cell antigen processing and presentation
- negative regulation of inflammatory response to antigenic stimulus
- negative regulation of interferon-gamma production
- negative regulation of T cell proliferation
- peptide antigen assembly with MHC class II protein complex
- positive regulation of CD4-positive, alpha-beta T cell activation
- positive regulation of CD4-positive, CD25-positive, alpha-beta regulatory T cell differentiation
- positive regulation of ERK1 and ERK2 cascade
- positive regulation of I-kappaB kinase/NF-kappaB signaling
- positive regulation of insulin secretion involved in cellular response to glucose stimulus
- positive regulation of kinase activity
- positive regulation of MAPK cascade
- positive regulation of memory T cell differentiation
- positive regulation of monocyte differentiation
- positive regulation of protein phosphorylation
- positive regulation of T cell mediated cytotoxicity
- positive regulation of T cell mediated immune response to tumor cell
- positive regulation of transcription, DNA-templated
- positive regulation of viral entry into host cell
- protein tetramerization
- regulation of interleukin-10 production
- regulation of interleukin-4 production
- regulation of T-helper cell differentiation
- signal transduction
- T cell receptor signaling pathway
- T-helper 1 type immune response

---

47

- **Protein name:** Envelope glycoprotein gp160
- **Organism:** Human immunodeficiency virus type 1 group M subtype B (isolate HXB2)
- **Uniprot Accession Number:** P04578
- **Protein sequence length:** 856 aa
- **1D identity (%):** 12.69
- **1D identity (%) [Gaps excluded]:** 26.64
- **1D identity - Alignment Gaps:** 755
- **Common reported functions (%):** 50.0
- **Common reported locations (%):** 50.0
- **Common reported processes (%):** 30.0

- **PDB ID:** 5C0R
- **Chain:** A
- **Crystallized protein length:** 270 aa
- **Resolution:** 3.19 Å
- **Alinged residues range:** 71-74, 71-74, 236-240, 236-240, 11-26, 191-198
- **Aligned to segment part (indices):** 2, 4, 1, 6, 0, 5
- **Alinged residues range of reference:** 50-53, 204-207, 420-424, 603-607, 658-666, 957-964
- **b-phipsi:** 0.088199
- **w-rdist:** 2.381815
- **t-alpha:** 0.379009
- **Chemical similarity (Tanimoto Index) (%):** 95.06
- **1D identity (%) [PDB]:** 0.08
- **1D identity (%) [Gaps excluded][PDB]:** 50.0
- **1D identity - Alignment Gaps [PDB]:** 1252
- **2D identity (%) [PDB]:** 21.05
- **2D identity (%) [Gaps excluded][PDB]:** 93.91
- **2D identity - Alignment Gaps [PDB]:** 796
- **3D similarity (TM-Score) (%) [PDB]:** 15.52

- **Gene name:** env
- **RefSeq ID:** NC\_001802
- **Genomic sequence length:** 9181
- **5-UTR|CDS|3-UTR identity (%):** 23.6 | 39.16 | 23.63
- **5-UTR|CDS|3-UTR identity (%) [Gaps excluded]:** 75.9 | 79.04 | 80.65
- **5-UTR|CDS|3-UTR identity [Alignment Gaps]:** 184 | 2157 | 374

**Uniprot Description:**  
  
Envelope glycoprotein gp160
Oligomerizes in the host endoplasmic reticulum into predominantly trimers. In a second time, gp160 transits in the host Golgi, where glycosylation is completed. The precursor is then proteolytically cleaved in the trans-Golgi and thereby activated by cellular furin or furin-like proteases to produce gp120 and gp41.  
  
Surface protein gp120
The mature envelope protein (Env) consists of a homotrimer of non-covalently associated gp120-gp41 heterodimers. The resulting complex protrudes from the virus surface as a spike. There seems to be as few as 10 spikes on the average virion. Interacts with host CD4, CCR5 and CXCR4. Gp120 also interacts with the C-type lectins CD209/DC-SIGN and CLEC4M/DC-SIGNR (collectively referred to as DC-SIGN(R)). Gp120 and gp41 interact with GalCer. Gp120 interacts with host ITGA4/ITGB7 complex; on CD4+ T-cells, this interaction results in rapid activation of integrin ITGAL/LFA-1, which facilitates efficient cell-to-cell spreading of HIV-1. Gp120 interacts with cell-associated heparan sulfate; this interaction increases virus infectivity on permissive cells and may be involved in infection of CD4- cells.  
  
**Gene Ontology Information:**

Molecular Function

- identical protein binding
- structural molecule activity

Location

- host cell endosome membrane
- host cell plasma membrane
- integral component of membrane
- viral envelope
- virion
- virion membrane

Biological process

- actin filament reorganization
- clathrin-dependent endocytosis of virus by host cell
- entry into host
- fusion of virus membrane with host endosome membrane
- fusion of virus membrane with host plasma membrane
- mitigation of host immune response by virus
- positive regulation of establishment of T cell polarity
- positive regulation of plasma membrane raft polarization
- positive regulation of receptor clustering
- stimulatory C-type lectin receptor signaling pathway
- viral life cycle
- viral protein processing
- virion assembly
- virion attachment to host cell

---

48

- **Protein name:** Spike glycoprotein
- **Organism:** Human coronavirus 229E
- **Uniprot Accession Number:** P15423
- **Protein sequence length:** 1173 aa
- **1D identity (%):** 26.53
- **1D identity (%) [Gaps excluded]:** 36.78
- **1D identity - Alignment Gaps:** 396
- **Common reported functions (%):** 0.0
- **Common reported locations (%):** 50.0
- **Common reported processes (%):** 50.0

- **PDB ID:** 6U7H
- **Chain:** B
- **Crystallized protein length:** 965 aa
- **Resolution:** 3.1 Å
- **Alinged residues range:** 951-956, 584-589, 68-72, 455-479, 584-612, 854-860
- **Aligned to segment part (indices):** 2, 4, 1, 6, 0, 5
- **Alinged residues range of reference:** 47-52, 203-208, 324-328, 593-606, 714-742, 969-975
- **b-phipsi:** 0.020483
- **w-rdist:** 8.841248
- **t-alpha:** 0.044397
- **Chemical similarity (Tanimoto Index) (%):** 94.45
- **1D identity (%) [PDB]:** 0.05
- **1D identity (%) [Gaps excluded][PDB]:** 50.0
- **1D identity - Alignment Gaps [PDB]:** 1947
- **2D identity (%) [PDB]:** 53.78
- **2D identity (%) [Gaps excluded][PDB]:** 89.36
- **2D identity - Alignment Gaps [PDB]:** 485
- **3D similarity (TM-Score) (%) [PDB]:** 62.2

- **Gene name:** S
- **RefSeq ID:** NC\_002645
- **Genomic sequence length:** 27317
- **5-UTR|CDS|3-UTR identity (%):** 46.55 | 46.24 | 32.46
- **5-UTR|CDS|3-UTR identity (%) [Gaps excluded]:** 77.51 | 79.68 | 84.38
- **5-UTR|CDS|3-UTR identity [Alignment Gaps]:** 139 | 1950 | 307

**Uniprot Description:**  
  
S1 region attaches the virion to the cell membrane by interacting with host ANPEP/aminopeptidase N, initiating the infection. Binding to the receptor probably induces conformational changes in the S glycoprotein unmasking the fusion peptide of S2 region and activating membranes fusion. S2 region belongs to the class I viral fusion protein. Under the current model, the protein has at least 3 conformational states: pre-fusion native state, pre-hairpin intermediate state, and post-fusion hairpin state. During viral and target cell membrane fusion, the coiled coil regions (heptad repeats) regions assume a trimer-of-hairpins structure, positioning the fusion peptide in close proximity to the C-terminal region of the ectodomain. The formation of this structure appears to drive apposition and subsequent fusion of viral and target cell membranes.  
  
Homotrimer. During virus morphogenesis, found in a complex with M and HE proteins. Interacts with host ANPEP.  
  
**Gene Ontology Information:**

Molecular Function  
  
N/A

Location

- host cell endoplasmic reticulum-Golgi intermediate compartment membrane
- integral component of membrane
- viral envelope
- virion membrane

Biological process

- endocytosis involved in viral entry into host cell
- fusion of virus membrane with host endosome membrane
- fusion of virus membrane with host plasma membrane
- pathogenesis
- receptor-mediated virion attachment to host cell

---

49

- **Protein name:** T7 RNA polymerase
- **Organism:** Escherichia phage T7
- **Uniprot Accession Number:** P00573
- **Protein sequence length:** 883 aa
- **1D identity (%):** 14.33
- **1D identity (%) [Gaps excluded]:** 26.32
- **1D identity - Alignment Gaps:** 636
- **Common reported functions (%):** 0.0
- **Common reported locations (%):** 0.0
- **Common reported processes (%):** 0.0

- **PDB ID:** 1H38
- **Chain:** B
- **Crystallized protein length:** 857 aa
- **Resolution:** 2.9 Å
- **Alinged residues range:** 606-608, 711-715, 598-610, 601-604, 481-490, 694-699
- **Aligned to segment part (indices):** 2, 4, 1, 6, 0, 5
- **Alinged residues range of reference:** 51-53, 195-199, 279-290, 603-606, 771-782, 957-962
- **b-phipsi:** 0.090734
- **w-rdist:** 1.983871
- **t-alpha:** 0.865878
- **Chemical similarity (Tanimoto Index) (%):** 83.86
- **1D identity (%) [PDB]:** 0.05
- **1D identity (%) [Gaps excluded][PDB]:** 100.0
- **1D identity - Alignment Gaps [PDB]:** 1840
- **2D identity (%) [PDB]:** 25.38
- **2D identity (%) [Gaps excluded][PDB]:** 89.22
- **2D identity - Alignment Gaps [PDB]:** 1026
- **3D similarity (TM-Score) (%) [PDB]:** 18.07

- **Gene name:** 1
- **RefSeq ID:** NC\_001604
- **Genomic sequence length:** 39937
- **5-UTR|CDS|3-UTR identity (%):** N/A | 38.21 | N/A
- **5-UTR|CDS|3-UTR identity (%) [Gaps excluded]:** N/A | 78.09 | N/A
- **5-UTR|CDS|3-UTR identity [Alignment Gaps]:** N/A | 2220 | N/A

**Uniprot Description:**  
  
Highly processive DNA-dependent RNA polymerase that catalyzes the transcription of class II and class III viral genes. Recognizes a specific promoter sequence and enters first into an 'abortive phase' where very short transcripts are synthesized and released before proceeding to the processive transcription of long RNA chains. Unwinds the double-stranded DNA to expose the coding strand for templating. Participates in the initiation of viral DNA replication presumably by making primers accessible to the DNA polymerase, thus facilitating the DNA opening. Plays also a role in viral DNA packaging, probably by pausing the transcription at the right end of concatemer junction to allow packaging complex recruitment and beginning of the packaging process.  
  
Monomer. Interacts with T7 lysozyme; this interaction inhibits transcriptional function of T7 RNA polymerase.  
  
**Gene Ontology Information:**

Molecular Function

- DNA binding
- DNA-directed 5'-3' RNA polymerase activity

Location  
  
N/A

Biological process

- DNA-templated viral transcription
- transcription, DNA-templated

---

50

- **Protein name:** DNA-directed DNA polymerase
- **Organism:** Escherichia phage T7
- **Uniprot Accession Number:** P00581
- **Protein sequence length:** 704 aa
- **1D identity (%):** 13.18
- **1D identity (%) [Gaps excluded]:** 28.43
- **1D identity - Alignment Gaps:** 725
- **Common reported functions (%):** 0.0
- **Common reported locations (%):** 0.0
- **Common reported processes (%):** 0.0

- **PDB ID:** 6N9W
- **Chain:** H
- **Crystallized protein length:** 645 aa
- **Resolution:** 4.0 Å
- **Alinged residues range:** 575-578, 575-577, 360-365, 151-154, 382-389, 609-615
- **Aligned to segment part (indices):** 2, 4, 1, 6, 0, 5
- **Alinged residues range of reference:** 49-52, 207-209, 305-310, 527-530, 771-778, 958-964
- **b-phipsi:** 0.093822
- **w-rdist:** 5.758308
- **t-alpha:** 0.04878
- **Chemical similarity (Tanimoto Index) (%):** N/A
- **1D identity (%) [PDB]:** 0.0
- **1D identity (%) [Gaps excluded][PDB]:** 0.0
- **1D identity - Alignment Gaps [PDB]:** 1630
- **2D identity (%) [PDB]:** 28.24
- **2D identity (%) [Gaps excluded][PDB]:** 88.58
- **2D identity - Alignment Gaps [PDB]:** 842
- **3D similarity (TM-Score) (%) [PDB]:** 16.16

- **Gene name:** 5
- **RefSeq ID:** NC\_001604
- **Genomic sequence length:** 39937
- **5-UTR|CDS|3-UTR identity (%):** N/A | 34.48 | N/A
- **5-UTR|CDS|3-UTR identity (%) [Gaps excluded]:** N/A | 77.56 | N/A
- **5-UTR|CDS|3-UTR identity [Alignment Gaps]:** N/A | 2283 | N/A

**Uniprot Description:**  
  
Replicates viral genomic DNA. This polymerase possesses two enzymatic activities: DNA synthesis (polymerase) and an exonucleolytic activity that degrades single-stranded DNA in the 3'-5' direction (By similarity). Non-processive DNA polymerase that achieves processivity by binding to host thioredoxin (TrxA). This interaction increases the rate of dNTP incorporation to yield a processivity of approximately 800 nucleotides (nt) per binding event. Interacts with DNA helicase gp4 to coordinate nucleotide polymerization with unwinding of the DNA. The leading strand is synthesized continuously while synthesis of the lagging strand requires the synthesis of oligoribonucleotides by the primase domain of gp4.  
  
Composed of two subunits. One is encoded by the phage and the other is encoded by the host thioredoxin. Interacts with helicase/primase gp4; this interaction is essential for the coordination of DNA unwinding and nucleotide polymerization on duplex DNA. Interacts with the ssDNA-binding protein gp2.5.  
  
**Gene Ontology Information:**

Molecular Function

- 3'-5' exonuclease activity
- DNA binding
- DNA-directed DNA polymerase activity
- exodeoxyribonuclease activity
- metal ion binding
- nucleotide binding

Location  
  
N/A

Biological process

- DNA synthesis involved in DNA replication
- DNA-dependent DNA replication
- viral DNA genome replication

---

51

- **Protein name:** 40S ribosomal protein S17
- **Organism:** Homo sapiens
- **Uniprot Accession Number:** P08708
- **Protein sequence length:** 135 aa
- **1D identity (%):** 2.22
- **1D identity (%) [Gaps excluded]:** 28.16
- **1D identity - Alignment Gaps:** 1202
- **Common reported functions (%):** 0.0
- **Common reported locations (%):** 0.0
- **Common reported processes (%):** 0.0

- **PDB ID:** 6ZN5
- **Chain:** S
- **Crystallized protein length:** 132 aa
- **Resolution:** 3.2 Å
- **Alinged residues range:** 115-118, 123-126, 43-46, 117-119, 74-79, 104-107
- **Aligned to segment part (indices):** 2, 4, 1, 6, 0, 5
- **Alinged residues range of reference:** 46-49, 217-220, 438-441, 585-587, 774-779, 957-960
- **b-phipsi:** 0.111953
- **w-rdist:** 1.127689
- **t-alpha:** 1.515957
- **Chemical similarity (Tanimoto Index) (%):** 72.97
- **1D identity (%) [PDB]:** 0.0
- **1D identity (%) [Gaps excluded][PDB]:** 0.0
- **1D identity - Alignment Gaps [PDB]:** 1115
- **2D identity (%) [PDB]:** 7.45
- **2D identity (%) [Gaps excluded][PDB]:** 95.06
- **2D identity - Alignment Gaps [PDB]:** 953
- **3D similarity (TM-Score) (%) [PDB]:** 8.06

- **Gene name:** RPS17
- **RefSeq ID:** NM\_001021
- **Transcript sequence length:** 488
- **5-UTR|CDS|3-UTR identity (%):** 7.49 | 7.23 | 14.58
- **5-UTR|CDS|3-UTR identity (%) [Gaps excluded]:** 74.07 | 78.87 | 87.5
- **5-UTR|CDS|3-UTR identity [Alignment Gaps]:** 240 | 3520 | 200

**Uniprot Description:**  
  
N/A  
  
**Gene Ontology Information:**

Molecular Function

- RNA binding
- structural constituent of ribosome

Location

- cytosol
- cytosolic ribosome
- cytosolic small ribosomal subunit
- focal adhesion
- membrane
- nucleoplasm
- ribosome

Biological process

- cytoplasmic translation
- erythrocyte homeostasis
- nuclear-transcribed mRNA catabolic process, nonsense-mediated decay
- ribosomal small subunit biogenesis
- rRNA processing
- SRP-dependent cotranslational protein targeting to membrane
- translation
- translational initiation
- viral transcription

---

52

- **Protein name:** Tyrosine--tRNA ligase, mitochondrial
- **Organism:** Neurospora crassa (strain ATCC 24698 / 74-OR23-1A / CBS 708.71 / DSM 1257 / FGSC 987)
- **Uniprot Accession Number:** P12063
- **Protein sequence length:** 669 aa
- **1D identity (%):** 10.11
- **1D identity (%) [Gaps excluded]:** 27.8
- **1D identity - Alignment Gaps:** 906
- **Common reported functions (%):** 50.0
- **Common reported locations (%):** 0.0
- **Common reported processes (%):** 0.0

- **PDB ID:** 2RKJ
- **Chain:** M
- **Crystallized protein length:** 367 aa
- **Resolution:** 4.5 Å
- **Alinged residues range:** 194-197, 266-269, 398-403, 99-102, 41-53, 398-401
- **Aligned to segment part (indices):** 2, 4, 1, 6, 0, 5
- **Alinged residues range of reference:** 44-47, 201-204, 295-300, 594-597, 768-779, 961-964
- **b-phipsi:** 0.082153
- **w-rdist:** 7.629833
- **t-alpha:** 0.035011
- **Chemical similarity (Tanimoto Index) (%):** 83.45
- **1D identity (%) [PDB]:** 0.0
- **1D identity (%) [Gaps excluded][PDB]:** 0.0
- **1D identity - Alignment Gaps [PDB]:** 1351
- **2D identity (%) [PDB]:** 18.4
- **2D identity (%) [Gaps excluded][PDB]:** 91.59
- **2D identity - Alignment Gaps [PDB]:** 899
- **3D similarity (TM-Score) (%) [PDB]:** 11.54

- **Gene name:** cyt-18
- **RefSeq ID:** N/A
- **Sequence length:** N/A
- **5-UTR|CDS|3-UTR identity (%):** N/A | N/A | N/A
- **5-UTR|CDS|3-UTR identity (%) [Gaps excluded]:** N/A | N/A | N/A
- **5-UTR|CDS|3-UTR identity [Alignment Gaps]:** N/A | N/A | N/A

**Uniprot Description:**  
  
Catalyzes the attachment of tyrosine to tRNA(Tyr) in a two-step reaction: tyrosine is first activated by ATP to form Tyr-AMP and then transferred to the acceptor end of tRNA(Tyr). Has both an aminoacyl-tRNA synthetase activity and is involved in the splicing of group I introns. It acts in intron splicing by stabilizing the catalytically active structure of the intron.  
  
**Gene Ontology Information:**

Molecular Function

- ATP binding
- identical protein binding
- RNA binding
- tyrosine-tRNA ligase activity

Location

- cytosol
- mitochondrial matrix
- mitochondrion

Biological process

- Group I intron splicing
- mRNA processing
- positive regulation of RNA splicing
- RNA folding
- tRNA aminoacylation
- tyrosyl-tRNA aminoacylation

---
