# Supplementary material for: Identifying and profiling structural similarities between Spike of SARS-CoV-2 and other viral or host proteins with Machaon
Source: Commun Biol. 2023 Jul 19;6:752. doi: 10.1038/s42003-023-05076-7 (PMC10356814; doi:10.1038/s42003-023-05076-7)
Supplement: Supplementary file 7 — Supplementary Data 4 [file 42003_2023_5076_MOESM7_ESM.zip › 6VXX_A_segment/candidates/6VXX_A_site2-metrics-merged-enriched_eval_report.html]

 

# Structural Comparison Report for 6VXX\_A\_site2 - segments (total: 57)

---

1

- **Protein name:** Spike glycoprotein
- **Organism:** Severe acute respiratory syndrome coronavirus
- **Uniprot Accession Number:** P59594
- **Protein sequence length:** 1255 aa
- **1D identity (%):** 76.35
- **1D identity (%) [Gaps excluded]:** 77.94
- **1D identity - Alignment Gaps:** 26
- **Common reported functions (%):** 100.0
- **Common reported locations (%):** 62.5
- **Common reported processes (%):** 90.0

- **PDB ID:** 5XLR
- **Chain:** C
- **Crystallized protein length:** 1022 aa
- **Resolution:** 3.8 Å
- **Alinged residues range:** 596-598, 751-768, 863-865, 929-936, 994-1046
- **Aligned to segment part (indices):** 4, 1, 2, 3, 0
- **Alinged residues range of reference:** 726-728, 769-786, 889-891, 947-954, 1012-1064
- **b-phipsi:** 0.005575
- **w-rdist:** 0.103236
- **t-alpha:** 0.013817
- **Chemical similarity (Tanimoto Index) (%):** 86.07
- **1D identity (%) [PDB]:** 69.22
- **1D identity (%) [Gaps excluded][PDB]:** 78.56
- **1D identity - Alignment Gaps [PDB]:** 127
- **2D identity (%) [PDB]:** 59.62
- **2D identity (%) [Gaps excluded][PDB]:** 81.94
- **2D identity - Alignment Gaps [PDB]:** 317
- **3D similarity (TM-Score) (%) [PDB]:** 93.79

- **Gene name:** S
- **RefSeq ID:** NC\_004718
- **Genomic sequence length:** 29751
- **5-UTR|CDS|3-UTR identity (%):** 88.52 | 73.15 | 22.38
- **5-UTR|CDS|3-UTR identity (%) [Gaps excluded]:** 92.28 | 78.79 | 98.18
- **5-UTR|CDS|3-UTR identity [Alignment Gaps]:** 11 | 282 | 745

**Uniprot Description:**  
  
Spike glycoprotein
May down-regulate host tetherin (BST2) by lysosomal degradation, thereby counteracting its antiviral activity.  
  
Homotrimer; each monomer consists of a S1 and a S2 subunit. The resulting peplomers protrude from the virus surface as spikes (By similarity). Binds to human and palm civet ACE2 and human CLEC4M/DC-SIGNR. Interacts with the accessory proteins 3a and 7a.  
  
**Gene Ontology Information:**

Molecular Function

- host cell surface receptor binding
- identical protein binding

Location

- host cell endoplasmic reticulum-Golgi intermediate compartment membrane
- host cell plasma membrane
- integral component of membrane
- viral envelope
- virion membrane

Biological process

- endocytosis involved in viral entry into host cell
- fusion of virus membrane with host endosome membrane
- fusion of virus membrane with host plasma membrane
- pathogenesis
- receptor-mediated virion attachment to host cell
- suppression by virus of host tetherin activity
- suppression by virus of host type I interferon-mediated signaling pathway
- viral protein processing
- viral translation

---

2

- **Protein name:** Adenosine deaminase
- **Organism:** Bos taurus
- **Uniprot Accession Number:** P56658
- **Protein sequence length:** 363 aa
- **1D identity (%):** 6.34
- **1D identity (%) [Gaps excluded]:** 28.72
- **1D identity - Alignment Gaps:** 1044
- **Common reported functions (%):** 0.0
- **Common reported locations (%):** 0.0
- **Common reported processes (%):** 0.0

- **PDB ID:** 2BGN
- **Chain:** F
- **Crystallized protein length:** 352 aa
- **Resolution:** 3.15 Å
- **Alinged residues range:** 147-150, 338-342, 200-202, 222-226, 197-203
- **Aligned to segment part (indices):** 4, 1, 2, 3, 0
- **Alinged residues range of reference:** 725-728, 773-777, 888-890, 950-954, 1017-1023
- **b-phipsi:** 0.005721
- **w-rdist:** 0.317999
- **t-alpha:** 0.169323
- **Chemical similarity (Tanimoto Index) (%):** N/A
- **1D identity (%) [PDB]:** 0.0
- **1D identity (%) [Gaps excluded][PDB]:** 0.0
- **1D identity - Alignment Gaps [PDB]:** 1335
- **2D identity (%) [PDB]:** 16.61
- **2D identity (%) [Gaps excluded][PDB]:** 92.61
- **2D identity - Alignment Gaps [PDB]:** 929
- **3D similarity (TM-Score) (%) [PDB]:** 18.11

- **Gene name:** ADA
- **RefSeq ID:** N/A
- **Sequence length:** N/A
- **5-UTR|CDS|3-UTR identity (%):** N/A | N/A | N/A
- **5-UTR|CDS|3-UTR identity (%) [Gaps excluded]:** N/A | N/A | N/A
- **5-UTR|CDS|3-UTR identity [Alignment Gaps]:** N/A | N/A | N/A

**Uniprot Description:**  
  
Catalyzes the hydrolytic deamination of adenosine and 2-deoxyadenosine (By similarity). Plays an important role in purine metabolism and in adenosine homeostasis (By similarity). Modulates signaling by extracellular adenosine, and so contributes indirectly to cellular signaling events (By similarity). Acts as a positive regulator of T-cell coactivation, by binding DPP4 (By similarity). Its interaction with DPP4 regulates lymphocyte-epithelial cell adhesion (By similarity). Enhances dendritic cell immunogenicity by affecting dendritic cell costimulatory molecule expression and cytokines and chemokines secretion (PubMed:23240012). Enhances CD4+ T-cell differentiation and proliferation (By similarity). Acts as a positive modulator of adenosine receptors ADORA1 and ADORA2A, by enhancing their ligand affinity via conformational change (By similarity). Stimulates plasminogen activation (By similarity). Plays a role in male fertility (By similarity). Plays a protective role in early postimplantation embryonic development (By similarity).  
  
Interacts with DPP4 (via extracellular domain). Interacts with PLG (via Kringle 4 domain); the interaction stimulates PLG activation when in complex with DPP4.  
  
**Gene Ontology Information:**

Molecular Function

- adenosine deaminase activity
- zinc ion binding

Location

- cell junction
- cytoplasmic vesicle lumen
- cytosol
- external side of plasma membrane
- lysosome

Biological process

- adenosine catabolic process
- cell adhesion
- hypoxanthine salvage
- inosine biosynthetic process
- negative regulation of adenosine receptor signaling pathway
- nucleotide metabolic process
- purine ribonucleoside monophosphate biosynthetic process
- T cell activation

---

3

- **Protein name:** Endolysin
- **Organism:** Escherichia phage lambda
- **Uniprot Accession Number:** P03706
- **Protein sequence length:** 158 aa
- **1D identity (%):** 2.71
- **1D identity (%) [Gaps excluded]:** 34.29
- **1D identity - Alignment Gaps:** 1221
- **Common reported functions (%):** 0.0
- **Common reported locations (%):** 0.0
- **Common reported processes (%):** 0.0

- **PDB ID:** 1D9U
- **Chain:** B
- **Crystallized protein length:** 154 aa
- **Resolution:** 2.6 Å
- **Alinged residues range:** 94-101, 96-101, 43-45
- **Aligned to segment part (indices):** 1, 3, 0
- **Alinged residues range of reference:** 770-777, 948-953, 1035-1037
- **b-phipsi:** 0.003693
- **w-rdist:** 0.571
- **t-alpha:** 0.278867
- **Chemical similarity (Tanimoto Index) (%):** 88.81
- **1D identity (%) [PDB]:** 0.0
- **1D identity (%) [Gaps excluded][PDB]:** 0.0
- **1D identity - Alignment Gaps [PDB]:** 1137
- **2D identity (%) [PDB]:** 8.27
- **2D identity (%) [Gaps excluded][PDB]:** 88.66
- **2D identity - Alignment Gaps [PDB]:** 943
- **3D similarity (TM-Score) (%) [PDB]:** 7.62

- **Gene name:** R
- **RefSeq ID:** NC\_001416
- **Genomic sequence length:** 48502
- **5-UTR|CDS|3-UTR identity (%):** N/A | 8.2 | N/A
- **5-UTR|CDS|3-UTR identity (%) [Gaps excluded]:** N/A | 75.71 | N/A
- **5-UTR|CDS|3-UTR identity [Alignment Gaps]:** N/A | 3459 | N/A

**Uniprot Description:**  
  
Endolysin with transglycosylase activity that degrades host peptidoglycans and participates with the holin and spanin proteins in the sequential events which lead to the programmed host cell lysis releasing the mature viral particles. Once the holin has permeabilized the host cell membrane, the endolysin can reach the periplasm and break down the peptidoglycan layer.  
  
Monomer.  
  
**Gene Ontology Information:**

Molecular Function

- lyase activity
- lysozyme activity
- lytic transglycosylase activity

Location

- host cell cytoplasm

Biological process

- cell wall macromolecule catabolic process
- cytolysis
- defense response to bacterium
- peptidoglycan catabolic process
- viral release from host cell by cytolysis

---

4

- **Protein name:** Glutaredoxin
- **Organism:** Enterobacteria phage T4
- **Uniprot Accession Number:** P00276
- **Protein sequence length:** 87 aa
- **1D identity (%):** 1.94
- **1D identity (%) [Gaps excluded]:** 34.25
- **1D identity - Alignment Gaps:** 1214
- **Common reported functions (%):** 0.0
- **Common reported locations (%):** 0.0
- **Common reported processes (%):** 0.0

- **PDB ID:** 1AAZ
- **Chain:** B
- **Crystallized protein length:** 87 aa
- **Resolution:** 2.0 Å
- **Alinged residues range:** 3-6, 46-49, 46-50, 49-51
- **Aligned to segment part (indices):** 4, 1, 3, 0
- **Alinged residues range of reference:** 725-728, 779-782, 949-953, 1022-1024
- **b-phipsi:** 0.00629
- **w-rdist:** 0.595767
- **t-alpha:** 0.137597
- **Chemical similarity (Tanimoto Index) (%):** 74.67
- **1D identity (%) [PDB]:** 0.0
- **1D identity (%) [Gaps excluded][PDB]:** 0.0
- **1D identity - Alignment Gaps [PDB]:** 1070
- **2D identity (%) [PDB]:** 5.37
- **2D identity (%) [Gaps excluded][PDB]:** 84.38
- **2D identity - Alignment Gaps [PDB]:** 942
- **3D similarity (TM-Score) (%) [PDB]:** 6.0

- **Gene name:** NRDC
- **RefSeq ID:** NC\_000866
- **Genomic sequence length:** 168903
- **5-UTR|CDS|3-UTR identity (%):** N/A | 5.2 | N/A
- **5-UTR|CDS|3-UTR identity (%) [Gaps excluded]:** N/A | 82.3 | N/A
- **5-UTR|CDS|3-UTR identity [Alignment Gaps]:** N/A | 3600 | N/A

**Uniprot Description:**  
  
Serves as a reducing agent for the phage-induced ribonucleotide reductase, but not for the bacterial ones. This specificity may be the result of sequence differences around the redox-active disulfide bond. The oxidized form accepts electrons from bacterial glutathione and will, in turn, reduce other small disulfides. Can also be reduced by NADPH and by bacterial thioredoxin reductase.  
  
**Gene Ontology Information:**

Molecular Function

- electron transfer activity
- protein disulfide oxidoreductase activity

Location  
  
N/A

Biological process

- deoxyribonucleotide biosynthetic process

---

5

- **Protein name:** Spike protein
- **Organism:** Porcine deltacoronavirus
- **Uniprot Accession Number:** A0A140ESF1
- **Protein sequence length:** 1160 aa
- **1D identity (%):** 26.87
- **1D identity (%) [Gaps excluded]:** 35.93
- **1D identity - Alignment Gaps:** 351
- **Common reported functions (%):** 0.0
- **Common reported locations (%):** 37.5
- **Common reported processes (%):** 50.0

- **PDB ID:** 6BFU
- **Chain:** C
- **Crystallized protein length:** 964 aa
- **Resolution:** 3.5 Å
- **Alinged residues range:** 965-967, 812-818, 754-756, 631-639, 884-933
- **Aligned to segment part (indices):** 4, 1, 2, 3, 0
- **Alinged residues range of reference:** 726-728, 770-776, 888-890, 947-953, 1015-1064
- **b-phipsi:** 0.008601
- **w-rdist:** 0.600101
- **t-alpha:** 0.171657
- **Chemical similarity (Tanimoto Index) (%):** 94.45
- **1D identity (%) [PDB]:** 0.0
- **1D identity (%) [Gaps excluded][PDB]:** 0.0
- **1D identity - Alignment Gaps [PDB]:** 1948
- **2D identity (%) [PDB]:** 55.23
- **2D identity (%) [Gaps excluded][PDB]:** 89.38
- **2D identity - Alignment Gaps [PDB]:** 460
- **3D similarity (TM-Score) (%) [PDB]:** 65.22

- **Gene name:** N/A
- **RefSeq ID:** N/A
- **Sequence length:** N/A
- **5-UTR|CDS|3-UTR identity (%):** N/A | N/A | N/A
- **5-UTR|CDS|3-UTR identity (%) [Gaps excluded]:** N/A | N/A | N/A
- **5-UTR|CDS|3-UTR identity [Alignment Gaps]:** N/A | N/A | N/A

**Uniprot Description:**  
  
N/A  
  
**Gene Ontology Information:**

Molecular Function  
  
N/A

Location

- host cell membrane
- integral component of membrane
- viral envelope
- virion membrane

Biological process

- endocytosis involved in viral entry into host cell
- fusion of virus membrane with host endosome membrane
- fusion of virus membrane with host plasma membrane
- pathogenesis
- receptor-mediated virion attachment to host cell

---

6

- **Protein name:** Late protein H7
- **Organism:** Vaccinia virus (strain Western Reserve)
- **Uniprot Accession Number:** P08586
- **Protein sequence length:** 146 aa
- **1D identity (%):** 1.87
- **1D identity (%) [Gaps excluded]:** 31.25
- **1D identity - Alignment Gaps:** 1259
- **Common reported functions (%):** 0.0
- **Common reported locations (%):** 12.5
- **Common reported processes (%):** 0.0

- **PDB ID:** 4W60
- **Chain:** B
- **Crystallized protein length:** 116 aa
- **Resolution:** 2.7 Å
- **Alinged residues range:** 40-42, 89-101, 91-95, 10-12
- **Aligned to segment part (indices):** 4, 1, 3, 0
- **Alinged residues range of reference:** 726-728, 769-781, 950-954, 1024-1026
- **b-phipsi:** 0.001148
- **w-rdist:** 2.215001
- **t-alpha:** 0.133205
- **Chemical similarity (Tanimoto Index) (%):** 73.1
- **1D identity (%) [PDB]:** 0.18
- **1D identity (%) [Gaps excluded][PDB]:** 50.0
- **1D identity - Alignment Gaps [PDB]:** 1091
- **2D identity (%) [PDB]:** 7.79
- **2D identity (%) [Gaps excluded][PDB]:** 92.94
- **2D identity - Alignment Gaps [PDB]:** 929
- **3D similarity (TM-Score) (%) [PDB]:** 6.42

- **Gene name:** VACWR105
- **RefSeq ID:** NC\_006998
- **Genomic sequence length:** 194711
- **5-UTR|CDS|3-UTR identity (%):** N/A | 7.84 | N/A
- **5-UTR|CDS|3-UTR identity (%) [Gaps excluded]:** N/A | 73.48 | N/A
- **5-UTR|CDS|3-UTR identity [Alignment Gaps]:** N/A | 3441 | N/A

**Uniprot Description:**  
  
Contributes to the formation of crescents and immature virions (IV). Interacts with phosphatidylinositol-3-phosphate (PI3P) and phosphatidylinositol-4-phosphate (PI4P) lipids in order to form virion membranes.  
  
**Gene Ontology Information:**

Molecular Function  
  
N/A

Location

- host cell cytoplasm
- integral component of membrane

Biological process  
  
N/A

---

7

- **Protein name:** Major capsid protein
- **Organism:** Escherichia phage T5
- **Uniprot Accession Number:** Q6QGD8
- **Protein sequence length:** 458 aa
- **1D identity (%):** 7.05
- **1D identity (%) [Gaps excluded]:** 24.8
- **1D identity - Alignment Gaps:** 965
- **Common reported functions (%):** 0.0
- **Common reported locations (%):** 0.0
- **Common reported processes (%):** 0.0

- **PDB ID:** 6OMA
- **Chain:** E
- **Crystallized protein length:** 299 aa
- **Resolution:** 7.2 Å
- **Alinged residues range:** 281-285, 286-289, 283-285, 250-254
- **Aligned to segment part (indices):** 1, 2, 3, 0
- **Alinged residues range of reference:** 770-774, 888-891, 952-954, 1052-1056
- **b-phipsi:** 0.033957
- **w-rdist:** 0.28988
- **t-alpha:** 0.460199
- **Chemical similarity (Tanimoto Index) (%):** 81.91
- **1D identity (%) [PDB]:** 0.08
- **1D identity (%) [Gaps excluded][PDB]:** 50.0
- **1D identity - Alignment Gaps [PDB]:** 1278
- **2D identity (%) [PDB]:** 19.29
- **2D identity (%) [Gaps excluded][PDB]:** 85.96
- **2D identity - Alignment Gaps [PDB]:** 812
- **3D similarity (TM-Score) (%) [PDB]:** 10.47

- **Gene name:** D20
- **RefSeq ID:** NC\_005859
- **Genomic sequence length:** 121750
- **5-UTR|CDS|3-UTR identity (%):** N/A | 23.02 | N/A
- **5-UTR|CDS|3-UTR identity (%) [Gaps excluded]:** N/A | 79.52 | N/A
- **5-UTR|CDS|3-UTR identity [Alignment Gaps]:** N/A | 2865 | N/A

**Uniprot Description:**  
  
Major capsid protein that self-associates to form 120 hexamers and 11 pentamers, building the T=13 icosahedral capsid which about 860 Angstroms in diameter. Responsible for its self-assembly into a procapsid. The phage does not need to encode a separate scaffolfing protein because its capsid protein contains the delta domain that carries that function. The capsid gains its final stability through the reorganization of the subunits that takes place upon expansion. DNA encapsidation through the portal triggers capsid expansion and the binding of the decoration protein to the capsid exterior.  
  
Interacts with the decoration protein; each hexon binds a single copy of the decoration protein. Interacts with the portal protein (Probable).  
  
**Gene Ontology Information:**

Molecular Function  
  
N/A

Location

- T=13 icosahedral viral capsid
- viral capsid
- viral scaffold

Biological process  
  
N/A

---

8

- **Protein name:** N-acetylmuramoyl-L-alanine amidase
- **Organism:** Bacillus phage Gamma
- **Uniprot Accession Number:** Q8LTE6
- **Protein sequence length:** 233 aa
- **1D identity (%):** 3.73
- **1D identity (%) [Gaps excluded]:** 30.3
- **1D identity - Alignment Gaps:** 1176
- **Common reported functions (%):** 0.0
- **Common reported locations (%):** 0.0
- **Common reported processes (%):** 0.0

- **PDB ID:** 2L47
- **Chain:** A
- **Crystallized protein length:** 3300 aa
- **Resolution:** -1.0 Å
- **Alinged residues range:** 148-156, 116-118, 152-156, 22-24
- **Aligned to segment part (indices):** 1, 2, 3, 0
- **Alinged residues range of reference:** 769-777, 888-890, 950-954, 1036-1038
- **b-phipsi:** 0.001452
- **w-rdist:** 2.54895
- **t-alpha:** 0.078365
- **Chemical similarity (Tanimoto Index) (%):** 83.79
- **1D identity (%) [PDB]:** 0.35
- **1D identity (%) [Gaps excluded][PDB]:** 66.67
- **1D identity - Alignment Gaps [PDB]:** 1136
- **2D identity (%) [PDB]:** 10.14
- **2D identity (%) [Gaps excluded][PDB]:** 85.25
- **2D identity - Alignment Gaps [PDB]:** 904
- **3D similarity (TM-Score) (%) [PDB]:** 7.61

- **Gene name:** PlyG
- **RefSeq ID:** NC\_007458
- **Genomic sequence length:** 37253
- **5-UTR|CDS|3-UTR identity (%):** N/A | 12.22 | N/A
- **5-UTR|CDS|3-UTR identity (%) [Gaps excluded]:** N/A | 79.3 | N/A
- **5-UTR|CDS|3-UTR identity [Alignment Gaps]:** N/A | 3316 | N/A

**Uniprot Description:**  
  
N/A  
  
**Gene Ontology Information:**

Molecular Function

- N-acetylmuramoyl-L-alanine amidase activity

Location  
  
N/A

Biological process

- cytolysis
- defense response to bacterium
- viral release from host cell by cytolysis

---

9

- **Protein name:** Holliday junction resolvase
- **Organism:** Fowlpox virus (strain NVSL)
- **Uniprot Accession Number:** Q9J546
- **Protein sequence length:** 156 aa
- **1D identity (%):** 2.72
- **1D identity (%) [Gaps excluded]:** 24.65
- **1D identity - Alignment Gaps:** 1145
- **Common reported functions (%):** 0.0
- **Common reported locations (%):** 0.0
- **Common reported processes (%):** 0.0

- **PDB ID:** 6P7A
- **Chain:** B
- **Crystallized protein length:** 144 aa
- **Resolution:** 3.08 Å
- **Alinged residues range:** 108-134, 121-126, 14-33
- **Aligned to segment part (indices):** 1, 3, 0
- **Alinged residues range of reference:** 770-782, 949-954, 1050-1064
- **b-phipsi:** 0.001068
- **w-rdist:** 5.292229
- **t-alpha:** 0.013629
- **Chemical similarity (Tanimoto Index) (%):** 77.7
- **1D identity (%) [PDB]:** 0.0
- **1D identity (%) [Gaps excluded][PDB]:** 0.0
- **1D identity - Alignment Gaps [PDB]:** 1129
- **2D identity (%) [PDB]:** 11.45
- **2D identity (%) [Gaps excluded][PDB]:** 92.0
- **2D identity - Alignment Gaps [PDB]:** 879
- **3D similarity (TM-Score) (%) [PDB]:** 7.75

- **Gene name:** FPV187
- **RefSeq ID:** NC\_002188
- **Genomic sequence length:** 288539
- **5-UTR|CDS|3-UTR identity (%):** N/A | 9.35 | N/A
- **5-UTR|CDS|3-UTR identity (%) [Gaps excluded]:** N/A | 81.26 | N/A
- **5-UTR|CDS|3-UTR identity [Alignment Gaps]:** N/A | 3407 | N/A

**Uniprot Description:**  
  
Nuclease that specifically cleaves and resolves four-way DNA Holliday junctions into linear duplex products.  
  
**Gene Ontology Information:**

Molecular Function

- crossover junction endodeoxyribonuclease activity
- four-way junction DNA binding
- magnesium ion binding

Location  
  
N/A

Biological process

- DNA recombination
- DNA repair

---

10

- **Protein name:** 40S ribosomal protein S19
- **Organism:** Homo sapiens
- **Uniprot Accession Number:** P39019
- **Protein sequence length:** 145 aa
- **1D identity (%):** 3.06
- **1D identity (%) [Gaps excluded]:** 27.46
- **1D identity - Alignment Gaps:** 1134
- **Common reported functions (%):** 50.0
- **Common reported locations (%):** 0.0
- **Common reported processes (%):** 0.0

- **PDB ID:** 6ZMT
- **Chain:** U
- **Crystallized protein length:** 144 aa
- **Resolution:** 3.0 Å
- **Alinged residues range:** 126-139, 130-134, 15-20
- **Aligned to segment part (indices):** 1, 3, 0
- **Alinged residues range of reference:** 777-782, 947-951, 1018-1026
- **b-phipsi:** 0.004102
- **w-rdist:** 4.845039
- **t-alpha:** 0.003419
- **Chemical similarity (Tanimoto Index) (%):** 81.85
- **1D identity (%) [PDB]:** 0.0
- **1D identity (%) [Gaps excluded][PDB]:** 0.0
- **1D identity - Alignment Gaps [PDB]:** 1127
- **2D identity (%) [PDB]:** 7.88
- **2D identity (%) [Gaps excluded][PDB]:** 95.35
- **2D identity - Alignment Gaps [PDB]:** 955
- **3D similarity (TM-Score) (%) [PDB]:** 7.21

- **Gene name:** RPS19
- **RefSeq ID:** N/A
- **Sequence length:** N/A
- **5-UTR|CDS|3-UTR identity (%):** N/A | N/A | N/A
- **5-UTR|CDS|3-UTR identity (%) [Gaps excluded]:** N/A | N/A | N/A
- **5-UTR|CDS|3-UTR identity [Alignment Gaps]:** N/A | N/A | N/A

**Uniprot Description:**  
  
Required for pre-rRNA processing and maturation of 40S ribosomal subunits.  
  
Interacts with RPS19BP1.  
  
**Gene Ontology Information:**

Molecular Function

- fibroblast growth factor binding
- identical protein binding
- protein kinase binding
- RNA binding
- structural constituent of ribosome

Location

- cytoplasm
- cytosol
- cytosolic ribosome
- cytosolic small ribosomal subunit
- extracellular exosome
- focal adhesion
- membrane
- nucleolus
- nucleoplasm
- postsynaptic density
- ribosome

Biological process

- antimicrobial humoral immune response mediated by antimicrobial peptide
- cytoplasmic translation
- defense response to Gram-negative bacterium
- erythrocyte differentiation
- killing of cells of other organism
- maturation of SSU-rRNA
- maturation of SSU-rRNA from tricistronic rRNA transcript (SSU-rRNA, 5.8S rRNA, LSU-rRNA)
- monocyte chemotaxis
- negative regulation of respiratory burst involved in inflammatory response
- nuclear-transcribed mRNA catabolic process, nonsense-mediated decay
- nucleolus organization
- positive regulation of cellular component movement
- positive regulation of respiratory burst involved in inflammatory response
- response to extracellular stimulus
- ribosomal small subunit assembly
- ribosomal small subunit biogenesis
- rRNA processing
- SRP-dependent cotranslational protein targeting to membrane
- translation
- translational initiation
- viral transcription

---

11

- **Protein name:** Nucleoprotein
- **Organism:** Equine arteritis virus (strain Bucyrus)
- **Uniprot Accession Number:** P19810
- **Protein sequence length:** 110 aa
- **1D identity (%):** 2.04
- **1D identity (%) [Gaps excluded]:** 24.07
- **1D identity - Alignment Gaps:** 1167
- **Common reported functions (%):** 0.0
- **Common reported locations (%):** 0.0
- **Common reported processes (%):** 0.0

- **PDB ID:** 2I9F
- **Chain:** A
- **Crystallized protein length:** 58 aa
- **Resolution:** 2.0 Å
- **Alinged residues range:** 98-100, 98-100, 61-65
- **Aligned to segment part (indices):** 1, 3, 0
- **Alinged residues range of reference:** 779-781, 949-951, 1017-1021
- **b-phipsi:** 0.012319
- **w-rdist:** 0.177486
- **t-alpha:** 0.706395
- **Chemical similarity (Tanimoto Index) (%):** 82.06
- **1D identity (%) [PDB]:** 0.1
- **1D identity (%) [Gaps excluded][PDB]:** 100.0
- **1D identity - Alignment Gaps [PDB]:** 1041
- **2D identity (%) [PDB]:** 2.66
- **2D identity (%) [Gaps excluded][PDB]:** 93.1
- **2D identity - Alignment Gaps [PDB]:** 985
- **3D similarity (TM-Score) (%) [PDB]:** 4.19

- **Gene name:** N
- **RefSeq ID:** NC\_002532
- **Genomic sequence length:** 12704
- **5-UTR|CDS|3-UTR identity (%):** N/A | 5.16 | N/A
- **5-UTR|CDS|3-UTR identity (%) [Gaps excluded]:** N/A | 77.91 | N/A
- **5-UTR|CDS|3-UTR identity [Alignment Gaps]:** N/A | 3639 | N/A

**Uniprot Description:**  
  
N/A  
  
**Gene Ontology Information:**

Molecular Function

- RNA binding

Location

- viral nucleocapsid

Biological process  
  
N/A

---

12

- **Protein name:** Maltose/maltodextrin-binding periplasmic protein
- **Organism:** Escherichia coli (strain K12)
- **Uniprot Accession Number:** P0AEX9
- **Protein sequence length:** 396 aa
- **1D identity (%):** 5.61
- **1D identity (%) [Gaps excluded]:** 30.27
- **1D identity - Alignment Gaps:** 1147
- **Common reported functions (%):** 0.0
- **Common reported locations (%):** 0.0
- **Common reported processes (%):** 0.0

- **PDB ID:** 6TZC
- **Chain:** A
- **Crystallized protein length:** 367 aa
- **Resolution:** 2.41 Å
- **Alinged residues range:** 287-289, 318-335, 362-364, 316-322, 343-350
- **Aligned to segment part (indices):** 4, 1, 2, 3, 0
- **Alinged residues range of reference:** 726-728, 771-780, 888-890, 948-954, 1018-1025
- **b-phipsi:** 0.055561
- **w-rdist:** 0.406618
- **t-alpha:** 0.287281
- **Chemical similarity (Tanimoto Index) (%):** 84.56
- **1D identity (%) [PDB]:** 0.3
- **1D identity (%) [Gaps excluded][PDB]:** 80.0
- **1D identity - Alignment Gaps [PDB]:** 1340
- **2D identity (%) [PDB]:** 16.28
- **2D identity (%) [Gaps excluded][PDB]:** 93.03
- **2D identity - Alignment Gaps [PDB]:** 948
- **3D similarity (TM-Score) (%) [PDB]:** 10.57

- **Gene name:** malE
- **RefSeq ID:** N/A
- **Sequence length:** N/A
- **5-UTR|CDS|3-UTR identity (%):** N/A | N/A | N/A
- **5-UTR|CDS|3-UTR identity (%) [Gaps excluded]:** N/A | N/A | N/A
- **5-UTR|CDS|3-UTR identity [Alignment Gaps]:** N/A | N/A | N/A

**Uniprot Description:**  
  
Part of the ABC transporter complex MalEFGK involved in maltose/maltodextrin import. Binds maltose and higher maltodextrins such as maltotriose.  
  
The complex is composed of two ATP-binding proteins (MalK), two transmembrane proteins (MalG and MalF) and a solute-binding protein (MalE).  
  
**Gene Ontology Information:**

Molecular Function

- carbohydrate transmembrane transporter activity
- maltose binding

Location

- ATP-binding cassette (ABC) transporter complex
- ATP-binding cassette (ABC) transporter complex, substrate-binding subunit-containing
- outer membrane-bounded periplasmic space
- periplasmic space

Biological process

- carbohydrate transport
- cell chemotaxis
- cellular response to DNA damage stimulus
- detection of maltose stimulus
- maltodextrin transport
- maltose transport

---

13

- **Protein name:** Polyprotein P1234
- **Organism:** Getah virus
- **Uniprot Accession Number:** Q5Y389
- **Protein sequence length:** 2467 aa
- **1D identity (%):** 10.13
- **1D identity (%) [Gaps excluded]:** 26.5
- **1D identity - Alignment Gaps:** 1672
- **Common reported functions (%):** 0.0
- **Common reported locations (%):** 12.5
- **Common reported processes (%):** 0.0

- **PDB ID:** 6R0T
- **Chain:** B
- **Crystallized protein length:** 161 aa
- **Resolution:** 1.85 Å
- **Alinged residues range:** 146-150, 129-131, 147-154, 84-87
- **Aligned to segment part (indices):** 1, 2, 3, 0
- **Alinged residues range of reference:** 776-780, 888-890, 947-954, 1023-1026
- **b-phipsi:** 0.006887
- **w-rdist:** 1.048141
- **t-alpha:** 0.019097
- **Chemical similarity (Tanimoto Index) (%):** 84.64
- **1D identity (%) [PDB]:** 0.0
- **1D identity (%) [Gaps excluded][PDB]:** 0.0
- **1D identity - Alignment Gaps [PDB]:** 1143
- **2D identity (%) [PDB]:** 10.17
- **2D identity (%) [Gaps excluded][PDB]:** 86.67
- **2D identity - Alignment Gaps [PDB]:** 903
- **3D similarity (TM-Score) (%) [PDB]:** 8.46

- **Gene name:** N/A
- **RefSeq ID:** NC\_006558
- **Genomic sequence length:** 11597
- **5-UTR|CDS|3-UTR identity (%):** N/A | 31.55 | N/A
- **5-UTR|CDS|3-UTR identity (%) [Gaps excluded]:** N/A | 77.9 | N/A
- **5-UTR|CDS|3-UTR identity [Alignment Gaps]:** N/A | 4754 | N/A

**Uniprot Description:**  
  
Polyprotein P1234
Inactive precursor of the viral replicase, which is activated by cleavages carried out by the viral protease nsP2.  
  
mRNA-capping enzyme nsP1
Interacts with non-structural protein 3 (By similarity). Interacts with RNA-directed RNA polymerase nsP4 (By similarity). Interacts with protease nsP2 (By similarity). interacts with itself (By similarity).  
  
**Gene Ontology Information:**

Molecular Function

- ATP binding
- cysteine-type peptidase activity
- GTP binding
- metal ion binding
- mRNA methyltransferase activity
- nucleoside-triphosphatase activity
- polynucleotide 5'-phosphatase activity
- polynucleotide adenylyltransferase activity
- RNA binding
- RNA helicase activity
- RNA-directed 5'-3' RNA polymerase activity

Location

- host cell cytoplasmic vesicle membrane
- host cell filopodium
- host cell nucleus
- host cell plasma membrane
- membrane

Biological process

- 7-methylguanosine mRNA capping
- suppression by virus of host RNA polymerase II activity
- transcription, DNA-templated
- viral RNA genome replication

---

14

- **Protein name:** Elongation factor Ts
- **Organism:** Escherichia coli (strain K12)
- **Uniprot Accession Number:** P0A6P1
- **Protein sequence length:** 283 aa
- **1D identity (%):** N/A
- **1D identity (%) [Gaps excluded]:** N/A
- **1D identity - Alignment Gaps:** N/A
- **Common reported functions (%):** N/A
- **Common reported locations (%):** N/A
- **Common reported processes (%):** N/A

- **PDB ID:** 3AGP
- **Chain:** A
- **Crystallized protein length:** 1202 aa
- **Resolution:** 2.8 Å
- **Alinged residues range:** 1056-1058, 192-202, 477-479, 189-193, 87-95
- **Aligned to segment part (indices):** 4, 1, 2, 3, 0
- **Alinged residues range of reference:** 726-728, 775-782, 888-890, 950-954, 1020-1028
- **b-phipsi:** 0.002257
- **w-rdist:** 9.448322
- **t-alpha:** 0.015571
- **Chemical similarity (Tanimoto Index) (%):** N/A
- **1D identity (%) [PDB]:** N/A
- **1D identity (%) [Gaps excluded][PDB]:** N/A
- **1D identity - Alignment Gaps [PDB]:** N/A
- **2D identity (%) [PDB]:** N/A
- **2D identity (%) [Gaps excluded][PDB]:** N/A
- **2D identity - Alignment Gaps [PDB]:** N/A
- **3D similarity (TM-Score) (%) [PDB]:** N/A

- **Gene name:** tsf
- **RefSeq ID:** N/A
- **Sequence length:** N/A
- **5-UTR|CDS|3-UTR identity (%):** N/A | N/A | N/A
- **5-UTR|CDS|3-UTR identity (%) [Gaps excluded]:** N/A | N/A | N/A
- **5-UTR|CDS|3-UTR identity [Alignment Gaps]:** N/A | N/A | N/A

**Uniprot Description:**  
  
Associates with the EF-Tu.GDP complex and induces the exchange of GDP to GTP. It remains bound to the aminoacyl-tRNA.EF-Tu.GTP complex up to the GTP hydrolysis stage on the ribosome.  
  
Heterotetramer composed of two EF-Ts.EF-Tu dimer complexes.  
  
**Gene Ontology Information:**

Molecular Function

- guanyl-nucleotide exchange factor activity
- translation elongation factor activity
- zinc ion binding

Location

- cytoplasm
- cytosol
- membrane

Biological process

- translational elongation

---

15

- **Protein name:** Antitoxin phd
- **Organism:** Escherichia phage P1
- **Uniprot Accession Number:** Q06253
- **Protein sequence length:** 73 aa
- **1D identity (%):** 1.57
- **1D identity (%) [Gaps excluded]:** 27.4
- **1D identity - Alignment Gaps:** 1200
- **Common reported functions (%):** 0.0
- **Common reported locations (%):** 0.0
- **Common reported processes (%):** 0.0

- **PDB ID:** 3KH2
- **Chain:** G
- **Crystallized protein length:** 71 aa
- **Resolution:** 2.71 Å
- **Alinged residues range:** 45-51, 42-44, 13-19, 42-48
- **Aligned to segment part (indices):** 1, 2, 3, 0
- **Alinged residues range of reference:** 776-782, 888-890, 948-954, 1020-1028
- **b-phipsi:** 0.004777
- **w-rdist:** 0.34746
- **t-alpha:** 0.963211
- **Chemical similarity (Tanimoto Index) (%):** 64.49
- **1D identity (%) [PDB]:** 0.09
- **1D identity (%) [Gaps excluded][PDB]:** 50.0
- **1D identity - Alignment Gaps [PDB]:** 1051
- **2D identity (%) [PDB]:** 5.42
- **2D identity (%) [Gaps excluded][PDB]:** 93.1
- **2D identity - Alignment Gaps [PDB]:** 939
- **3D similarity (TM-Score) (%) [PDB]:** 4.2

- **Gene name:** phd
- **RefSeq ID:** NC\_005856
- **Genomic sequence length:** 94800
- **5-UTR|CDS|3-UTR identity (%):** N/A | 4.25 | N/A
- **5-UTR|CDS|3-UTR identity (%) [Gaps excluded]:** N/A | 79.13 | N/A
- **5-UTR|CDS|3-UTR identity [Alignment Gaps]:** N/A | 3632 | N/A

**Uniprot Description:**  
  
Antitoxin component of a type II toxin-antitoxin (TA) system (PubMed:18398006, PubMed:24141193, PubMed:18757857). A labile antitoxin that binds to cognate doc toxin and neutralizes its ability to phosphorylate host EF-Tu. Does not reverse phosphorylation. Bacteriophage P1 lysogenizes bacteria as a low-copy number plasmid; phd and doc proteins function in unison to stabilize plasmid number by inducing a lethal response to P1 plasmid prophage loss (PubMed:8411153).  
  
Homodimer. Interacts with cognate toxin doc, the exact ratio of doc:phd varies from 1:1 to 1:3. Interaction with doc prevents both kinase activity and dephosphorylation of EF-Tu.  
  
**Gene Ontology Information:**

Molecular Function

- protein homodimerization activity
- sequence-specific DNA binding

Location

- protein-DNA complex

Biological process  
  
N/A

---

16

- **Protein name:** Deoxycytidylate 5-hydroxymethyltransferase
- **Organism:** Enterobacteria phage T4
- **Uniprot Accession Number:** P08773
- **Protein sequence length:** 246 aa
- **1D identity (%):** 3.74
- **1D identity (%) [Gaps excluded]:** 27.47
- **1D identity - Alignment Gaps:** 1155
- **Common reported functions (%):** 0.0
- **Common reported locations (%):** 0.0
- **Common reported processes (%):** 0.0

- **PDB ID:** 1B5E
- **Chain:** A
- **Crystallized protein length:** 241 aa
- **Resolution:** 1.6 Å
- **Alinged residues range:** 211-214, 80-85, 174-176, 187-193, 174-183
- **Aligned to segment part (indices):** 4, 1, 2, 3, 0
- **Alinged residues range of reference:** 725-728, 776-781, 888-890, 948-954, 1020-1028
- **b-phipsi:** 0.003527
- **w-rdist:** 4.660712
- **t-alpha:** 0.026224
- **Chemical similarity (Tanimoto Index) (%):** 85.83
- **1D identity (%) [PDB]:** 0.08
- **1D identity (%) [Gaps excluded][PDB]:** 50.0
- **1D identity - Alignment Gaps [PDB]:** 1220
- **2D identity (%) [PDB]:** 11.46
- **2D identity (%) [Gaps excluded][PDB]:** 87.32
- **2D identity - Alignment Gaps [PDB]:** 940
- **3D similarity (TM-Score) (%) [PDB]:** 8.92

- **Gene name:** 42
- **RefSeq ID:** NC\_000866
- **Genomic sequence length:** 168903
- **5-UTR|CDS|3-UTR identity (%):** N/A | 13.2 | N/A
- **5-UTR|CDS|3-UTR identity (%) [Gaps excluded]:** N/A | 78.78 | N/A
- **5-UTR|CDS|3-UTR identity [Alignment Gaps]:** N/A | 3253 | N/A

**Uniprot Description:**  
  
N/A  
  
**Gene Ontology Information:**

Molecular Function

- deoxycytidylate 5-hydroxymethyltransferase activity

Location  
  
N/A

Biological process  
  
N/A

---

17

- **Protein name:** 40S ribosomal protein S16
- **Organism:** Homo sapiens
- **Uniprot Accession Number:** P62249
- **Protein sequence length:** 146 aa
- **1D identity (%):** 2.15
- **1D identity (%) [Gaps excluded]:** 23.93
- **1D identity - Alignment Gaps:** 1185
- **Common reported functions (%):** 0.0
- **Common reported locations (%):** 0.0
- **Common reported processes (%):** 0.0

- **PDB ID:** 5OA3
- **Chain:** Q
- **Crystallized protein length:** 139 aa
- **Resolution:** 4.3 Å
- **Alinged residues range:** 11-14, 105-111, 105-110, 79-83
- **Aligned to segment part (indices):** 4, 1, 3, 0
- **Alinged residues range of reference:** 725-728, 775-781, 949-954, 1016-1020
- **b-phipsi:** 0.005642
- **w-rdist:** 1.967887
- **t-alpha:** 0.063406
- **Chemical similarity (Tanimoto Index) (%):** 73.04
- **1D identity (%) [PDB]:** 0.09
- **1D identity (%) [Gaps excluded][PDB]:** 50.0
- **1D identity - Alignment Gaps [PDB]:** 1118
- **2D identity (%) [PDB]:** 9.04
- **2D identity (%) [Gaps excluded][PDB]:** 88.46
- **2D identity - Alignment Gaps [PDB]:** 914
- **3D similarity (TM-Score) (%) [PDB]:** 7.41

- **Gene name:** RPS16
- **RefSeq ID:** NM\_001020
- **Transcript sequence length:** 23531
- **5-UTR|CDS|3-UTR identity (%):** 19.7 | 35.41 | 0.87
- **5-UTR|CDS|3-UTR identity (%) [Gaps excluded]:** 79.33 | 78.56 | 86.19
- **5-UTR|CDS|3-UTR identity [Alignment Gaps]:** 454 | 2291 | 20622

**Uniprot Description:**  
  
N/A  
  
**Gene Ontology Information:**

Molecular Function

- RNA binding
- structural constituent of ribosome

Location

- cytosol
- cytosolic ribosome
- cytosolic small ribosomal subunit
- extracellular exosome
- focal adhesion
- membrane
- nucleoplasm
- small ribosomal subunit

Biological process

- cytoplasmic translation
- maturation of SSU-rRNA from tricistronic rRNA transcript (SSU-rRNA, 5.8S rRNA, LSU-rRNA)
- nuclear-transcribed mRNA catabolic process, nonsense-mediated decay
- ribosomal small subunit biogenesis
- rRNA processing
- SRP-dependent cotranslational protein targeting to membrane
- translation
- translational initiation
- viral transcription

---

18

- **Protein name:** Polymerase acidic protein
- **Organism:** Influenza A virus (strain A/Goose/Guangdong/1/1996 H5N1 genotype Gs/Gd)
- **Uniprot Accession Number:** Q9Q0U9
- **Protein sequence length:** 716 aa
- **1D identity (%):** 10.66
- **1D identity (%) [Gaps excluded]:** 28.97
- **1D identity - Alignment Gaps:** 919
- **Common reported functions (%):** 0.0
- **Common reported locations (%):** 0.0
- **Common reported processes (%):** 0.0

- **PDB ID:** 3HW4
- **Chain:** A
- **Crystallized protein length:** 178 aa
- **Resolution:** 1.9 Å
- **Alinged residues range:** 146-149, 133-138, 134-138, 17-23
- **Aligned to segment part (indices):** 4, 1, 3, 0
- **Alinged residues range of reference:** 725-728, 776-781, 947-951, 1022-1028
- **b-phipsi:** 0.004451
- **w-rdist:** 3.745575
- **t-alpha:** 0.022648
- **Chemical similarity (Tanimoto Index) (%):** N/A
- **1D identity (%) [PDB]:** 0.0
- **1D identity (%) [Gaps excluded][PDB]:** 0.0
- **1D identity - Alignment Gaps [PDB]:** 1158
- **2D identity (%) [PDB]:** 9.74
- **2D identity (%) [Gaps excluded][PDB]:** 91.89
- **2D identity - Alignment Gaps [PDB]:** 936
- **3D similarity (TM-Score) (%) [PDB]:** 7.74

- **Gene name:** PA
- **RefSeq ID:** NC\_007359
- **Genomic sequence length:** 2233
- **5-UTR|CDS|3-UTR identity (%):** N/A | 36.09 | N/A
- **5-UTR|CDS|3-UTR identity (%) [Gaps excluded]:** N/A | 78.52 | N/A
- **5-UTR|CDS|3-UTR identity [Alignment Gaps]:** N/A | 2211 | N/A

**Uniprot Description:**  
  
Plays an essential role in viral RNA transcription and replication by forming the heterotrimeric polymerase complex together with PB1 and PB2 subunits. The complex transcribes viral mRNAs by using a unique mechanism called cap-snatching. It consists in the hijacking and cleavage of host capped pre-mRNAs. These short capped RNAs are then used as primers for viral mRNAs. The PB2 subunit is responsible for the binding of the 5' cap of cellular pre-mRNAs which are subsequently cleaved after 10-13 nucleotides by the PA subunit that carries the endonuclease activity.  
  
Influenza RNA polymerase is composed of three subunits: PB1, PB2 and PA. Interacts (via C-terminus) with PB1 (via N-terminus).  
  
**Gene Ontology Information:**

Molecular Function

- endonuclease activity
- metal ion binding
- RNA binding

Location

- host cell cytoplasm
- host cell nucleus

Biological process

- cap snatching
- suppression by virus of host RNA polymerase II activity
- transcription, DNA-templated
- viral RNA genome replication

---

19

- **Protein name:** HLA class I histocompatibility antigen, B alpha chain
- **Organism:** Homo sapiens
- **Uniprot Accession Number:** P01889
- **Protein sequence length:** 362 aa
- **1D identity (%):** 5.07
- **1D identity (%) [Gaps excluded]:** 25.27
- **1D identity - Alignment Gaps:** 1089
- **Common reported functions (%):** 0.0
- **Common reported locations (%):** 0.0
- **Common reported processes (%):** 0.0

- **PDB ID:** 1A1N
- **Chain:** A
- **Crystallized protein length:** 276 aa
- **Resolution:** 2.0 Å
- **Alinged residues range:** 173-178, 172-174, 112-126
- **Aligned to segment part (indices):** 1, 3, 0
- **Alinged residues range of reference:** 773-778, 948-950, 1047-1063
- **b-phipsi:** 0.00169
- **w-rdist:** 3.561903
- **t-alpha:** 0.207819
- **Chemical similarity (Tanimoto Index) (%):** 86.01
- **1D identity (%) [PDB]:** 0.08
- **1D identity (%) [Gaps excluded][PDB]:** 50.0
- **1D identity - Alignment Gaps [PDB]:** 1255
- **2D identity (%) [PDB]:** 17.86
- **2D identity (%) [Gaps excluded][PDB]:** 88.21
- **2D identity - Alignment Gaps [PDB]:** 835
- **3D similarity (TM-Score) (%) [PDB]:** 10.24

- **Gene name:** HLA-B
- **RefSeq ID:** NM\_005514
- **Transcript sequence length:** 1536
- **5-UTR|CDS|3-UTR identity (%):** 6.79 | 16.87 | 33.71
- **5-UTR|CDS|3-UTR identity (%) [Gaps excluded]:** 85.71 | 75.47 | 72.95
- **5-UTR|CDS|3-UTR identity [Alignment Gaps]:** 244 | 3117 | 241

**Uniprot Description:**  
  
Antigen-presenting major histocompatibility complex class I (MHCI) molecule. In complex with B2M/beta 2 microglobulin displays primarily viral and tumor-derived peptides on antigen-presenting cells for recognition by alpha-beta T cell receptor (TCR) on HLA-B-restricted CD8-positive T cells, guiding antigen-specific T cell immune response to eliminate infected or transformed cells (PubMed:25808313, PubMed:29531227, PubMed:9620674, PubMed:23209413). May also present self-peptides derived from the signal sequence of secreted or membrane proteins, although T cells specific for these peptides are usually inactivated to prevent autoreactivity (PubMed:7743181, PubMed:18991276). Both the peptide and the MHC molecule are recognized by TCR, the peptide is responsible for the fine specificity of antigen recognition and MHC residues account for the MHC restriction of T cells (PubMed:29531227, PubMed:9620674, PubMed:24600035). Typically presents intracellular peptide antigens of 8 to 13 amino acids that arise from cytosolic proteolysis via constitutive proteasome and IFNG-induced immunoproteasome (PubMed:23209413). Can bind different peptides containing allele-specific binding motifs, which are mainly defined by anchor residues at position 2 and 9 (PubMed:25808313, PubMed:29531227).  
  
Heterotrimer that consists of an alpha chain HLA-B, a beta chain B2M and a peptide (peptide-HLA-B-B2M) (PubMed:25808313, PubMed:29531227, PubMed:15657948, PubMed:17057332, PubMed:22020283, PubMed:24600035). Early in biogenesis, HLA-B-B2M dimer interacts with the components of the peptide-loading complex composed of TAPBP, TAP1-TAP2, TAPBPL, PDIA3/ERP57 and CALR (PubMed:9036970, PubMed:9620674, PubMed:26439010, PubMed:26416272). Interacts with TAP1-TAP2 transporter via TAPBP; this interaction is obligatory for the loading of peptide epitopes delivered to the ER by TAP1-TAP2 transporter (PubMed:9036970, PubMed:9620674). Interacts with TAPBPL; TAPBPL binds peptide-free HLA-B-B2M complexes or those loaded with low affinity peptides, likely facilitating peptide exchange for higher affinity peptides (PubMed:26439010). Only optimally assembled peptide-HLA-B-B2M trimer translocates to the surface of antigen-presenting cells, where it interacts with TCR and CD8 coreceptor on the surface of T cells. HLA-B (via polymorphic alpha-1 and alpha-2 domains) interacts with antigen-specific TCR (via CDR1, CDR2 and CDR3 domains) (PubMed:29531227, PubMed:24600035). One HLA-B molecule (mainly via nonpolymorphic alpha-3 domain) interacts with one CD8A homodimer (via CDR-like loop); this interaction insures peptide-HLA-B-B2M recognition by CD8-positive T cells only (PubMed:29531227). Allele B\*57:01 interacts (via Bw4 motif) with KIR3DL1 (via Ig-like C2-type domain); this interaction may interfere with peptide binding (PubMed:22020283, PubMed:25480565). Allele B\*46:01 interacts with KIR2DL3 (PubMed:28514659).  
  
**Gene Ontology Information:**

Molecular Function

- chaperone binding
- peptide antigen binding
- signaling receptor binding
- TAP binding

Location

- cell surface
- early endosome membrane
- endoplasmic reticulum
- ER to Golgi transport vesicle membrane
- extracellular exosome
- Golgi apparatus
- Golgi membrane
- integral component of lumenal side of endoplasmic reticulum membrane
- integral component of plasma membrane
- membrane
- MHC class I protein complex
- phagocytic vesicle membrane
- plasma membrane
- recycling endosome membrane
- secretory granule membrane

Biological process

- adaptive immune response
- antigen processing and presentation of endogenous peptide antigen via MHC class I via ER pathway, TAP-independent
- antigen processing and presentation of exogenous peptide antigen via MHC class I, TAP-dependent
- antigen processing and presentation of exogenous peptide antigen via MHC class I, TAP-independent
- antigen processing and presentation of peptide antigen via MHC class I
- defense response
- detection of bacterium
- immune response
- interferon-gamma-mediated signaling pathway
- neutrophil degranulation
- positive regulation of T cell mediated cytotoxicity
- protection from natural killer cell mediated cytotoxicity
- regulation of dendritic cell differentiation
- regulation of immune response
- regulation of interleukin-12 production
- regulation of interleukin-6 production
- regulation of T cell anergy
- type I interferon signaling pathway
- viral process

---

20

- **Protein name:** E3 ubiquitin-protein ligase RBX1
- **Organism:** Homo sapiens
- **Uniprot Accession Number:** P62877
- **Protein sequence length:** 108 aa
- **1D identity (%):** 2.14
- **1D identity (%) [Gaps excluded]:** 38.89
- **1D identity - Alignment Gaps:** 1237
- **Common reported functions (%):** 0.0
- **Common reported locations (%):** 0.0
- **Common reported processes (%):** 0.0

- **PDB ID:** 2HYE
- **Chain:** D
- **Crystallized protein length:** 90 aa
- **Resolution:** 3.1 Å
- **Alinged residues range:** 82-84, 82-84, 30-32
- **Aligned to segment part (indices):** 1, 3, 0
- **Alinged residues range of reference:** 779-781, 949-951, 1061-1063
- **b-phipsi:** 0.031811
- **w-rdist:** 0.644094
- **t-alpha:** 0.340183
- **Chemical similarity (Tanimoto Index) (%):** N/A
- **1D identity (%) [PDB]:** 0.0
- **1D identity (%) [Gaps excluded][PDB]:** 0.0
- **1D identity - Alignment Gaps [PDB]:** 1073
- **2D identity (%) [PDB]:** 6.59
- **2D identity (%) [Gaps excluded][PDB]:** 91.67
- **2D identity - Alignment Gaps [PDB]:** 929
- **3D similarity (TM-Score) (%) [PDB]:** 20.63

- **Gene name:** RBX1
- **RefSeq ID:** NM\_014248
- **Transcript sequence length:** 1169
- **5-UTR|CDS|3-UTR identity (%):** 5.66 | 5.85 | 18.44
- **5-UTR|CDS|3-UTR identity (%) [Gaps excluded]:** 71.43 | 79.3 | 76.47
- **5-UTR|CDS|3-UTR identity [Alignment Gaps]:** 244 | 3579 | 642

**Uniprot Description:**  
  
E3 ubiquitin ligase component of multiple cullin-RING-based E3 ubiquitin-protein ligase (CRLs) complexes which mediate the ubiquitination and subsequent proteasomal degradation of target proteins, including proteins involved in cell cycle progression, signal transduction, transcription and transcription-coupled nucleotide excision repair (PubMed:10230407, PubMed:10579999, PubMed:15983046, PubMed:16678110, PubMed:19112177, PubMed:19679664, PubMed:23455478, PubMed:27565346, PubMed:29769719, PubMed:11961546, PubMed:22748924). CRLs complexes and ARIH1 collaborate in tandem to mediate ubiquitination of target proteins, ARIH1 mediating addition of the first ubiquitin on CRLs targets (PubMed:27565346). The functional specificity of the E3 ubiquitin-protein ligase complexes depends on the variable substrate recognition components. As a component of the CSA complex promotes the ubiquitination of ERCC6 resulting in proteasomal degradation. Recruits the E2 ubiquitin-conjugating enzyme CDC34 to the complex and brings it into close proximity to the substrate. Probably also stimulates CDC34 autoubiquitination. May be required for histone H3 and histone H4 ubiquitination in response to ultraviolet and for subsequent DNA repair. Promotes the neddylation of CUL1, CUL2, CUL4 and CUL4 via its interaction with UBE2M. Involved in the ubiquitination of KEAP1, ENC1 and KLHL41. In concert with ATF2 and CUL3, promotes degradation of KAT5 thereby attenuating its ability to acetylate and activate ATM.  
  
Part of a SCF complex consisting of CUL1, RBX1, SKP1 and SKP2 (PubMed:11961546). Part of a SCF-like complex consisting of CUL7, RBX1, SKP1 and FBXW8. Part of CBC(VHL) complexes with elongin BC complex (ELOB and ELOC), CUL2 or CUL5 and VHL. Part of the CSA complex (DCX(ERCC8) complex), a DCX E3 ubiquitin-protein ligase complex containing ERCC8, RBX1, DDB1 and CUL4A; the CSA complex interacts with RNA polymerase II; upon UV irradiation it interacts with the COP9 signalosome and preferentially with the hyperphosphorylated form of RNA polymerase II. Part of multisubunit E3 ubiquitin ligase complexes with elongin BC complex (ELOB and ELOC), CUL2 and MED8; elongin BC complex (ELOB and ELOC), CUL5 and MUF1. Part of multisubunit complexes with elongin BC complex (ELOB and ELOC), elongin A/ELOA or SOCS1 or WSB1 and CUL5. Interacts directly with CUL1 and probably also with CUL2, CUL3, CUL4A, CUL4B, CUL5 and CUL7. Interacts with CDC34 (PubMed:22748924). Interacts with GLMN. GLMN competes for the binding site of the E2 ubiquitin-conjugating enzyme CDC34 and disrupts CDC34 binding (PubMed:22748924). Interacts with COPS6. Component of the DCX DET1-COP1 ubiquitin ligase complex at least composed of RBX1, DET1, DDB1, CUL4A and COP1. Part of an E3 ligase complex composed of RBX1, DDB1, DDB2 and CUL4A or CUL4B. Interacts with UBE2M. Part of a SCF complex consisting of CUL1, FBXO3, RBX1 and SKP1; this complex interacts with PML via FBXO3. Component of the SCF(Cyclin F) complex consisting of CUL1, RBX1, SKP1 and CCNF. Identified in a SCF (SKP1-CUL1-F-box protein) E3 ubiquitin ligase complex together with HINT1 and CDC34. Component of multiple BCR (BTB-CUL3-RBX1) E3 ubiquitin-protein ligase complexes formed of CUL3, RBX1 and a variable BTB domain-containing protein. Part of the BCR(ENC1) complex containing ENC1. Part of the BCR(GAN) complex containing GAN. Part of the BCR(KLHL41) complex containing KLHL41. Part of the BCR(KEAP1) complex containing KEAP1. Interacts with SESN1 and SESN2 (PubMed:23274085). Interacts with NOTCH2 (PubMed:29149593). Component of the BCR(KLHL22) E3 ubiquitin ligase complex, at least composed of CUL3, KLHL22 and RBX1 (PubMed:23455478). Interacts with DCUN1D1, DCUN1D2, DCUN1D3, DCUN1D4 and DCUN1D5 (PubMed:26906416, PubMed:24192928, PubMed:25349211). Component of a BCR3 (BTB-CUL3-RBX1) E3 ubiquitin ligase complex, also named Cul3-RING ubiquitin ligase complex CUL3(KBTBD6/7), composed of CUL3, RBX1, KBTBD6 and KBTBD7 (PubMed:25684205).  
  
**Gene Ontology Information:**

Molecular Function

- cullin family protein binding
- NEDD8 ligase activity
- protein-containing complex binding
- transcription factor binding
- ubiquitin protein ligase activity
- ubiquitin protein ligase binding
- ubiquitin-ubiquitin ligase activity
- zinc ion binding

Location

- Cul2-RING ubiquitin ligase complex
- Cul3-RING ubiquitin ligase complex
- Cul4A-RING E3 ubiquitin ligase complex
- Cul4B-RING E3 ubiquitin ligase complex
- Cul5-RING ubiquitin ligase complex
- Cul7-RING ubiquitin ligase complex
- cullin-RING ubiquitin ligase complex
- cytosol
- nucleoplasm
- nucleus
- SCF ubiquitin ligase complex
- VCB complex

Biological process

- DNA damage response, detection of DNA damage
- global genome nucleotide-excision repair
- interleukin-1-mediated signaling pathway
- MAPK cascade
- negative regulation of canonical Wnt signaling pathway
- negative regulation of G2/M transition of mitotic cell cycle
- nucleotide-excision repair, DNA damage recognition
- nucleotide-excision repair, DNA duplex unwinding
- nucleotide-excision repair, DNA incision
- nucleotide-excision repair, DNA incision, 3'-to lesion
- nucleotide-excision repair, DNA incision, 5'-to lesion
- nucleotide-excision repair, preincision complex assembly
- nucleotide-excision repair, preincision complex stabilization
- positive regulation of proteasomal ubiquitin-dependent protein catabolic process
- positive regulation of protein autoubiquitination
- post-translational protein modification
- proteasome-mediated ubiquitin-dependent protein catabolic process
- protein K48-linked ubiquitination
- protein monoubiquitination
- protein neddylation
- protein polyubiquitination
- protein ubiquitination
- regulation of transcription from RNA polymerase II promoter in response to hypoxia
- SCF complex assembly
- SCF-dependent proteasomal ubiquitin-dependent protein catabolic process
- transcription-coupled nucleotide-excision repair
- ubiquitin-dependent protein catabolic process
- viral process
- Wnt signaling pathway

---

21

- **Protein name:** 40S ribosomal protein S15
- **Organism:** Oryctolagus cuniculus
- **Uniprot Accession Number:** G1U0Q2
- **Protein sequence length:** 145 aa
- **1D identity (%):** 2.13
- **1D identity (%) [Gaps excluded]:** 26.67
- **1D identity - Alignment Gaps:** 1208
- **Common reported functions (%):** 0.0
- **Common reported locations (%):** 0.0
- **Common reported processes (%):** 0.0

- **PDB ID:** 4D61
- **Chain:** Q
- **Crystallized protein length:** 139 aa
- **Resolution:** 9.0 Å
- **Alinged residues range:** 11-14, 105-111, 105-110, 79-83
- **Aligned to segment part (indices):** 4, 1, 3, 0
- **Alinged residues range of reference:** 725-728, 775-781, 949-954, 1016-1020
- **b-phipsi:** 0.004758
- **w-rdist:** 2.101255
- **t-alpha:** 0.212947
- **Chemical similarity (Tanimoto Index) (%):** N/A
- **1D identity (%) [PDB]:** 0.09
- **1D identity (%) [Gaps excluded][PDB]:** 50.0
- **1D identity - Alignment Gaps [PDB]:** 1118
- **2D identity (%) [PDB]:** 9.03
- **2D identity (%) [Gaps excluded][PDB]:** 89.32
- **2D identity - Alignment Gaps [PDB]:** 916
- **3D similarity (TM-Score) (%) [PDB]:** 7.87

- **Gene name:** RPS15
- **RefSeq ID:** N/A
- **Sequence length:** N/A
- **5-UTR|CDS|3-UTR identity (%):** N/A | N/A | N/A
- **5-UTR|CDS|3-UTR identity (%) [Gaps excluded]:** N/A | N/A | N/A
- **5-UTR|CDS|3-UTR identity [Alignment Gaps]:** N/A | N/A | N/A

**Uniprot Description:**  
  
N/A  
  
**Gene Ontology Information:**

Molecular Function  
  
N/A

Location  
  
N/A

Biological process  
  
N/A

---

22

- **Protein name:** Cyclin homolog
- **Organism:** Saimiriine herpesvirus 2 (strain 11)
- **Uniprot Accession Number:** Q01043
- **Protein sequence length:** 254 aa
- **1D identity (%):** 4.46
- **1D identity (%) [Gaps excluded]:** 25.44
- **1D identity - Alignment Gaps:** 1071
- **Common reported functions (%):** 0.0
- **Common reported locations (%):** 0.0
- **Common reported processes (%):** 0.0

- **PDB ID:** 2EUF
- **Chain:** A
- **Crystallized protein length:** 244 aa
- **Resolution:** 3.0 Å
- **Alinged residues range:** 133-138, 129-136, 58-63
- **Aligned to segment part (indices):** 1, 3, 0
- **Alinged residues range of reference:** 772-777, 947-954, 1021-1026
- **b-phipsi:** 0.027056
- **w-rdist:** 1.248561
- **t-alpha:** 0.008591
- **Chemical similarity (Tanimoto Index) (%):** 83.44
- **1D identity (%) [PDB]:** 0.24
- **1D identity (%) [Gaps excluded][PDB]:** 100.0
- **1D identity - Alignment Gaps [PDB]:** 1222
- **2D identity (%) [PDB]:** 16.79
- **2D identity (%) [Gaps excluded][PDB]:** 94.09
- **2D identity - Alignment Gaps [PDB]:** 856
- **3D similarity (TM-Score) (%) [PDB]:** 10.5

- **Gene name:** 72
- **RefSeq ID:** NC\_001350
- **Genomic sequence length:** 112930
- **5-UTR|CDS|3-UTR identity (%):** N/A | 13.76 | N/A
- **5-UTR|CDS|3-UTR identity (%) [Gaps excluded]:** N/A | 82.72 | N/A
- **5-UTR|CDS|3-UTR identity [Alignment Gaps]:** N/A | 3279 | N/A

**Uniprot Description:**  
  
May be highly relevant to the process of cellular transformation and rapid T-cell proliferation effected by HVS during latent infections of T-cells in susceptible hosts.  
  
**Gene Ontology Information:**

Molecular Function  
  
N/A

Location  
  
N/A

Biological process

- cell cycle
- cell division
- modulation by virus of host cell cycle

---

23

- **Protein name:** ADP-ribosylation factor 1
- **Organism:** Homo sapiens
- **Uniprot Accession Number:** P84077
- **Protein sequence length:** 181 aa
- **1D identity (%):** 3.01
- **1D identity (%) [Gaps excluded]:** 32.26
- **1D identity - Alignment Gaps:** 1206
- **Common reported functions (%):** 0.0
- **Common reported locations (%):** 0.0
- **Common reported processes (%):** 0.0

- **PDB ID:** 6CRI
- **Chain:** H
- **Crystallized protein length:** 163 aa
- **Resolution:** 6.8 Å
- **Alinged residues range:** 52-55, 138-142, 108-110, 138-142, 136-143
- **Aligned to segment part (indices):** 4, 1, 2, 3, 0
- **Alinged residues range of reference:** 725-728, 776-780, 888-890, 950-954, 1015-1024
- **b-phipsi:** 0.022056
- **w-rdist:** 3.662731
- **t-alpha:** 0.001704
- **Chemical similarity (Tanimoto Index) (%):** 83.08
- **1D identity (%) [PDB]:** 0.0
- **1D identity (%) [Gaps excluded][PDB]:** 0.0
- **1D identity - Alignment Gaps [PDB]:** 1146
- **2D identity (%) [PDB]:** 9.23
- **2D identity (%) [Gaps excluded][PDB]:** 90.57
- **2D identity - Alignment Gaps [PDB]:** 934
- **3D similarity (TM-Score) (%) [PDB]:** 18.24

- **Gene name:** ARF1
- **RefSeq ID:** NM\_001658
- **Transcript sequence length:** 1840
- **5-UTR|CDS|3-UTR identity (%):** 23.27 | 9.58 | 11.0
- **5-UTR|CDS|3-UTR identity (%) [Gaps excluded]:** 75.29 | 77.02 | 75.27
- **5-UTR|CDS|3-UTR identity [Alignment Gaps]:** 190 | 3402 | 1064

**Uniprot Description:**  
  
GTP-binding protein involved in protein trafficking among different compartments. Modulates vesicle budding and uncoating within the Golgi complex. Deactivation induces the redistribution of the entire Golgi complex to the endoplasmic reticulum, suggesting a crucial role in protein trafficking. In its GTP-bound form, its triggers the association with coat proteins with the Golgi membrane. The hydrolysis of ARF1-bound GTP, which is mediated by ARFGAPs proteins, is required for dissociation of coat proteins from Golgi membranes and vesicles. The GTP-bound form interacts with PICK1 to limit PICK1-mediated inhibition of Arp2/3 complex activity; the function is linked to AMPA receptor (AMPAR) trafficking, regulation of synaptic plasicity of excitatory synapses and spine shrinkage during long-term depression (LTD).  
  
Interacts (when activated) with GGA1, GGA2 and GGA3; the interaction is required for proper subcellular location of GGA1, GGA2 and GGA3 (PubMed:11950392, PubMed:28868155). Interacts with ARHGAP21, ASAP2, HERC1, PRKCABP, PIP5K1B, TMED2, PSCD2, TMED10 and GRIA2 (PubMed:10022920, PubMed:17347647, PubMed:23889934, PubMed:8861955). Interacts with ARFGAP1, which hydrolyzes GTP and thus, regulates its function (PubMed:10102276). Interacts with PI4KB in the Golgi complex (PubMed:17555535). Interacts with NCS1/FREQ in the Golgi and at the plasma membrane (PubMed:17555535). Interacts with PLEKHA3 (PubMed:21454700). Interacts with PLEKHA8; the interaction, together with phosphatidylinositol 4-phosphate binding, is required for FAPP2-mediated glucosylceramide transfer activity (PubMed:15107860). Interacts (activated) with PICK1 (via PDZ domain); the interaction blocks Arp2/3 complex inhibition (PubMed:23889934). Interacts with IQSEC1 (PubMed:24058294). Interacts with C9orf72 (By similarity) (PubMed:24058294).  
  
**Gene Ontology Information:**

Molecular Function

- GDP binding
- GTP binding
- magnesium ion binding
- phospholipase D activator activity
- protein domain specific binding
- RNA binding

Location

- cell leading edge
- COPI-coated vesicle
- cytosol
- extracellular exosome
- focal adhesion
- glutamatergic synapse
- Golgi membrane
- late endosome
- neuron projection
- perinuclear region of cytoplasm
- peroxisomal membrane
- plasma membrane
- postsynaptic density
- protein-containing complex
- sarcomere
- trans-Golgi network

Biological process

- actin filament organization
- antigen processing and presentation of exogenous peptide antigen via MHC class II
- cellular copper ion homeostasis
- cellular response to virus
- dendritic spine organization
- Golgi to transport vesicle transport
- interleukin-12-mediated signaling pathway
- intracellular protein transport
- long-term synaptic depression
- lysosomal membrane organization
- mitotic cleavage furrow ingression
- phosphatidylinositol biosynthetic process
- positive regulation of calcium ion-dependent exocytosis
- positive regulation of dendritic spine development
- positive regulation of endocytosis
- positive regulation of ER to Golgi vesicle-mediated transport
- positive regulation of late endosome to lysosome transport
- positive regulation of protein secretion
- positive regulation of sodium ion transmembrane transport
- post-Golgi vesicle-mediated transport
- postsynaptic actin cytoskeleton organization
- regulation of Arp2/3 complex-mediated actin nucleation
- regulation of defense response to virus by virus
- regulation of phospholipid metabolic process
- regulation of receptor internalization
- synaptic vesicle budding
- very-low-density lipoprotein particle assembly
- vesicle-mediated transport

---

24

- **Protein name:** Polynucleotide kinase
- **Organism:** Enterobacteria phage T4
- **Uniprot Accession Number:** P06855
- **Protein sequence length:** 301 aa
- **1D identity (%):** 4.33
- **1D identity (%) [Gaps excluded]:** 27.96
- **1D identity - Alignment Gaps:** 1152
- **Common reported functions (%):** 0.0
- **Common reported locations (%):** 0.0
- **Common reported processes (%):** 0.0

- **PDB ID:** 5UJ0
- **Chain:** A
- **Crystallized protein length:** 142 aa
- **Resolution:** 2.3 Å
- **Alinged residues range:** 257-260, 199-201, 254-259, 262-266
- **Aligned to segment part (indices):** 1, 2, 3, 0
- **Alinged residues range of reference:** 772-775, 888-890, 949-954, 1029-1033
- **b-phipsi:** 0.00523
- **w-rdist:** 1.605588
- **t-alpha:** 0.243644
- **Chemical similarity (Tanimoto Index) (%):** N/A
- **1D identity (%) [PDB]:** 0.09
- **1D identity (%) [Gaps excluded][PDB]:** 100.0
- **1D identity - Alignment Gaps [PDB]:** 1125
- **2D identity (%) [PDB]:** 8.03
- **2D identity (%) [Gaps excluded][PDB]:** 88.3
- **2D identity - Alignment Gaps [PDB]:** 939
- **3D similarity (TM-Score) (%) [PDB]:** 7.84

- **Gene name:** pseT
- **RefSeq ID:** NC\_000866
- **Genomic sequence length:** 168903
- **5-UTR|CDS|3-UTR identity (%):** N/A | 16.79 | N/A
- **5-UTR|CDS|3-UTR identity (%) [Gaps excluded]:** N/A | 79.9 | N/A
- **5-UTR|CDS|3-UTR identity [Alignment Gaps]:** N/A | 3086 | N/A

**Uniprot Description:**  
  
Acts as a 5'-hydroxyl kinase, a 3'-phosphatase and a 2',3'-cyclic phosphodiesterase. Catalyzes the transfer of the terminal phosphate of ATP to the 5'-hydroxyl termini of ribo- and deoxyribonucleotides. In the presence of ADP the enzyme also catalyzes an exchange reaction. In the exchange reaction, an excess ADP causes the enzyme to transfer the 5' terminal phosphate from phosphorylated DNA to ADP (PubMed:5323016, PubMed:199248). Involved in countering a host defense mechanism which activates T4-induced anticodon nuclease and shuts off viral translation. The polynucleotide kinase modifies the ends of nicked tRNA generated by the antiviral response of the host bacteria and facilitates repair by T4 RNA ligase (PubMed:2444436).  
  
Homotetramer.  
  
**Gene Ontology Information:**

Molecular Function

- ATP binding
- ATP-dependent polydeoxyribonucleotide 5'-hydroxyl-kinase activity
- deoxynucleotide 3'-phosphatase activity

Location  
  
N/A

Biological process

- DNA repair
- viral process

---

25

- **Protein name:** Late genes activator p4
- **Organism:** Bacillus phage phi29
- **Uniprot Accession Number:** P03682
- **Protein sequence length:** 125 aa
- **1D identity (%):** 2.26
- **1D identity (%) [Gaps excluded]:** 25.89
- **1D identity - Alignment Gaps:** 1174
- **Common reported functions (%):** 0.0
- **Common reported locations (%):** 0.0
- **Common reported processes (%):** 0.0

- **PDB ID:** 2FIP
- **Chain:** C
- **Crystallized protein length:** 115 aa
- **Resolution:** 2.0 Å
- **Alinged residues range:** 18-20, 47-55, 47-53, 66-73
- **Aligned to segment part (indices):** 4, 1, 3, 0
- **Alinged residues range of reference:** 726-728, 773-781, 947-953, 1016-1023
- **b-phipsi:** 0.076776
- **w-rdist:** 0.543869
- **t-alpha:** 0.394299
- **Chemical similarity (Tanimoto Index) (%):** 81.96
- **1D identity (%) [PDB]:** 0.0
- **1D identity (%) [Gaps excluded][PDB]:** 0.0
- **1D identity - Alignment Gaps [PDB]:** 1098
- **2D identity (%) [PDB]:** 7.59
- **2D identity (%) [Gaps excluded][PDB]:** 91.67
- **2D identity - Alignment Gaps [PDB]:** 930
- **3D similarity (TM-Score) (%) [PDB]:** 6.65

- **Gene name:** 4
- **RefSeq ID:** NC\_011048
- **Genomic sequence length:** 19282
- **5-UTR|CDS|3-UTR identity (%):** N/A | 7.26 | N/A
- **5-UTR|CDS|3-UTR identity (%) [Gaps excluded]:** N/A | 77.72 | N/A
- **5-UTR|CDS|3-UTR identity [Alignment Gaps]:** N/A | 3482 | N/A

**Uniprot Description:**  
  
Mediates, together with protein p6, the early to late transcriptional switch by stabilizing the binding of host RNA polymerase (RNAP) to the late A3 promoter. Activates transcription from the late A3 promoter and represses the main early promoters A2b and A2c by modifying the topology of the sequences encompassing early promoters A2c and A2b and late promoter A3 in a hairpin. Proteins p6 and p4 bind cooperatively to an approximately 200 bp DNA region located between the late A3 and the early A2c promoters. Binding of p4 molecules induces the reorganization of the binding of protein p6, giving rise to the nucleoprotein complex responsible for the switch from early to late transcription.  
  
Interacts with host RNA polymerase (via C-terminus) (PubMed:8617213, PubMed:8799127, PubMed:9784366). Interacts with DNA; binds to the A2b, A2c and A3 promoters (PubMed:8617213). Interacts (via C-terminus) with the histone-like protein p6 (PubMed:12426390).  
  
**Gene Ontology Information:**

Molecular Function

- DNA binding
- DNA-directed 5'-3' RNA polymerase activity
- sigma factor activity

Location  
  
N/A

Biological process

- regulation of transcription, DNA-templated

---

26

- **Protein name:** Endonuclease V
- **Organism:** Enterobacteria phage T4
- **Uniprot Accession Number:** P04418
- **Protein sequence length:** 138 aa
- **1D identity (%):** 2.38
- **1D identity (%) [Gaps excluded]:** 29.25
- **1D identity - Alignment Gaps:** 1199
- **Common reported functions (%):** 0.0
- **Common reported locations (%):** 0.0
- **Common reported processes (%):** 0.0

- **PDB ID:** 1ENK
- **Chain:** A
- **Crystallized protein length:** 137 aa
- **Resolution:** 2.0 Å
- **Alinged residues range:** 110-121, 28-30, 118-122, 16-19
- **Aligned to segment part (indices):** 1, 2, 3, 0
- **Alinged residues range of reference:** 769-777, 888-890, 948-952, 1023-1026
- **b-phipsi:** 0.022409
- **w-rdist:** 4.095302
- **t-alpha:** 0.0
- **Chemical similarity (Tanimoto Index) (%):** 83.1
- **1D identity (%) [PDB]:** 0.09
- **1D identity (%) [Gaps excluded][PDB]:** 100.0
- **1D identity - Alignment Gaps [PDB]:** 1118
- **2D identity (%) [PDB]:** 9.92
- **2D identity (%) [Gaps excluded][PDB]:** 81.15
- **2D identity - Alignment Gaps [PDB]:** 876
- **3D similarity (TM-Score) (%) [PDB]:** 7.16

- **Gene name:** N/A
- **RefSeq ID:** NC\_000866
- **Genomic sequence length:** 168903
- **5-UTR|CDS|3-UTR identity (%):** N/A | 7.04 | N/A
- **5-UTR|CDS|3-UTR identity (%) [Gaps excluded]:** N/A | 83.08 | N/A
- **5-UTR|CDS|3-UTR identity [Alignment Gaps]:** N/A | 3577 | N/A

**Uniprot Description:**  
  
Participates in the repair of UV-damaged DNA by excising pyrimidine dimers that are the major UV-lesions (PubMed:6254991). DNA glycosylase activity hydrolyzes the glycosylic bond of the 5' pyrimidine of the dimer (PubMed:6254991). This leaves apurinic/apyrimidic (AP) sites in the DNA. These AP sites are removed by the AP lyase activity which cleaves the intrapyrimidine phosphodiester bond (PubMed:6254991). Catalysis proceeds via a protonated imine covalent intermediate between the alpha-amino group of the N-terminal threonine residue and the C1' of the deoxyribose sugar of the 5' pyrimidine at the dimer site (PubMed:8347626) (PubMed:16916523).  
  
Monomer.  
  
**Gene Ontology Information:**

Molecular Function

- class I DNA-(apurinic or apyrimidinic site) endonuclease activity
- deoxyribodipyrimidine endonucleosidase activity
- DNA-(apurinic or apyrimidinic site) endonuclease activity
- endonuclease activity
- pyrimidine dimer DNA N-glycosylase activity

Location  
  
N/A

Biological process

- DNA repair

---

27

- **Protein name:** Major capsid protein
- **Organism:** Salmonella phage epsilon15
- **Uniprot Accession Number:** Q858G8
- **Protein sequence length:** 335 aa
- **1D identity (%):** 6.07
- **1D identity (%) [Gaps excluded]:** 29.67
- **1D identity - Alignment Gaps:** 1062
- **Common reported functions (%):** 0.0
- **Common reported locations (%):** 0.0
- **Common reported processes (%):** 0.0

- **PDB ID:** 3J40
- **Chain:** E
- **Crystallized protein length:** 335 aa
- **Resolution:** 4.5 Å
- **Alinged residues range:** 117-126, 123-125, 90-92
- **Aligned to segment part (indices):** 1, 3, 0
- **Alinged residues range of reference:** 775-782, 949-951, 1047-1049
- **b-phipsi:** 0.015228
- **w-rdist:** 0.256379
- **t-alpha:** 1.111511
- **Chemical similarity (Tanimoto Index) (%):** 83.1
- **1D identity (%) [PDB]:** 0.08
- **1D identity (%) [Gaps excluded][PDB]:** 100.0
- **1D identity - Alignment Gaps [PDB]:** 1316
- **2D identity (%) [PDB]:** 19.44
- **2D identity (%) [Gaps excluded][PDB]:** 88.24
- **2D identity - Alignment Gaps [PDB]:** 842
- **3D similarity (TM-Score) (%) [PDB]:** 10.24

- **Gene name:** N/A
- **RefSeq ID:** NC\_004775
- **Genomic sequence length:** 39672
- **5-UTR|CDS|3-UTR identity (%):** N/A | 17.54 | N/A
- **5-UTR|CDS|3-UTR identity (%) [Gaps excluded]:** N/A | 78.75 | N/A
- **5-UTR|CDS|3-UTR identity [Alignment Gaps]:** N/A | 3070 | N/A

**Uniprot Description:**  
  
N/A  
  
**Gene Ontology Information:**

Molecular Function  
  
N/A

Location

- viral capsid

Biological process  
  
N/A

---

28

- **Protein name:** DNA primase/helicase
- **Organism:** Escherichia phage T7
- **Uniprot Accession Number:** P03692
- **Protein sequence length:** 566 aa
- **1D identity (%):** 11.03
- **1D identity (%) [Gaps excluded]:** 30.53
- **1D identity - Alignment Gaps:** 863
- **Common reported functions (%):** 50.0
- **Common reported locations (%):** 0.0
- **Common reported processes (%):** 0.0

- **PDB ID:** 6N9U
- **Chain:** F
- **Crystallized protein length:** 196 aa
- **Resolution:** 3.7 Å
- **Alinged residues range:** 215-218, 214-218, 105-109
- **Aligned to segment part (indices):** 1, 3, 0
- **Alinged residues range of reference:** 771-774, 950-954, 1052-1056
- **b-phipsi:** 0.032248
- **w-rdist:** 3.414416
- **t-alpha:** 0.003419
- **Chemical similarity (Tanimoto Index) (%):** 85.18
- **1D identity (%) [PDB]:** 0.0
- **1D identity (%) [Gaps excluded][PDB]:** 0.0
- **1D identity - Alignment Gaps [PDB]:** 1179
- **2D identity (%) [PDB]:** 12.98
- **2D identity (%) [Gaps excluded][PDB]:** 81.48
- **2D identity - Alignment Gaps [PDB]:** 855
- **3D similarity (TM-Score) (%) [PDB]:** 7.89

- **Gene name:** 4
- **RefSeq ID:** NC\_001604
- **Genomic sequence length:** 39937
- **5-UTR|CDS|3-UTR identity (%):** N/A | 29.08 | N/A
- **5-UTR|CDS|3-UTR identity (%) [Gaps excluded]:** N/A | 78.0 | N/A
- **5-UTR|CDS|3-UTR identity [Alignment Gaps]:** N/A | 2523 | N/A

**Uniprot Description:**  
  
Synthesizes short RNA primers for DNA replication. Unwinds the DNA at the replication forks and generates single-stranded DNA for both leading and lagging strand synthesis. The primase synthesizes short RNA primers on the lagging strand that the polymerase elongates using dNTPs.  
  
Homohexamer. Present in a mixture of heptamers and hexamers in the absence of DNA, and assembles onto ssDNA as a hexamer. Interacts with the DNA polymerase gp5; this interaction is essential to initiate leading-strand DNA synthesis. Interacts with single-stranded DNA-binding protein gp2.5.  
  
**Gene Ontology Information:**

Molecular Function

- ATP binding
- DNA helicase activity
- DNA primase activity
- identical protein binding
- zinc ion binding

Location  
  
N/A

Biological process  
  
N/A

---

29

- **Protein name:** Tyrosine-protein phosphatase non-receptor type 4
- **Organism:** Homo sapiens
- **Uniprot Accession Number:** P29074
- **Protein sequence length:** 926 aa
- **1D identity (%):** 1.76
- **1D identity (%) [Gaps excluded]:** 23.84
- **1D identity - Alignment Gaps:** 1897
- **Common reported functions (%):** 0.0
- **Common reported locations (%):** 0.0
- **Common reported processes (%):** 0.0

- **PDB ID:** 3NFK
- **Chain:** A
- **Crystallized protein length:** 92 aa
- **Resolution:** 1.43 Å
- **Alinged residues range:** 531-534, 580-582, 580-583, 552-555
- **Aligned to segment part (indices):** 4, 1, 3, 0
- **Alinged residues range of reference:** 725-728, 779-781, 949-952, 1021-1024
- **b-phipsi:** 0.095203
- **w-rdist:** 0.612557
- **t-alpha:** 0.220374
- **Chemical similarity (Tanimoto Index) (%):** 73.23
- **1D identity (%) [PDB]:** 0.09
- **1D identity (%) [Gaps excluded][PDB]:** 50.0
- **1D identity - Alignment Gaps [PDB]:** 1071
- **2D identity (%) [PDB]:** 7.37
- **2D identity (%) [Gaps excluded][PDB]:** 85.88
- **2D identity - Alignment Gaps [PDB]:** 905
- **3D similarity (TM-Score) (%) [PDB]:** 5.22

- **Gene name:** PTPN4
- **RefSeq ID:** N/A
- **Sequence length:** N/A
- **5-UTR|CDS|3-UTR identity (%):** N/A | N/A | N/A
- **5-UTR|CDS|3-UTR identity (%) [Gaps excluded]:** N/A | N/A | N/A
- **5-UTR|CDS|3-UTR identity [Alignment Gaps]:** N/A | N/A | N/A

**Uniprot Description:**  
  
Phosphatase that plays a role in immunity, learning, synaptic plasticity or cell homeostasis (PubMed:25825441, PubMed:27246854). Regulates neuronal cell homeostasis by protecting neurons against apoptosis (PubMed:20086240). Negatively regulates TLR4-induced interferon beta production by dephosphorylating adapter TICAM2 and inhibiting subsequent TRAM-TRIF interaction (PubMed:25825441). Dephosphorylates also the immunoreceptor tyrosine-based activation motifs/ITAMs of the TCR zeta subunit and thereby negatively regulates TCR-mediated signaling pathway (By similarity). May act at junctions between the membrane and the cytoskeleton.  
  
Interacts with MAPK12 (via C-terminus); this interaction abolishes PTPN4 catalytic autoinhibition and thus activates the phosphatase activity.  
  
**Gene Ontology Information:**

Molecular Function

- cytoskeletal protein binding
- non-membrane spanning protein tyrosine phosphatase activity
- protein tyrosine phosphatase activity

Location

- cytoplasm
- cytoplasmic side of plasma membrane
- cytoskeleton
- cytosol
- nucleoplasm

Biological process

- cellular response to cytokine stimulus
- protein dephosphorylation

---

30

- **Protein name:** Uncharacterized protein
- **Organism:** Oryctolagus cuniculus
- **Uniprot Accession Number:** G1T1F0
- **Protein sequence length:** 151 aa
- **1D identity (%):** 3.18
- **1D identity (%) [Gaps excluded]:** 30.83
- **1D identity - Alignment Gaps:** 1158
- **Common reported functions (%):** 0.0
- **Common reported locations (%):** 0.0
- **Common reported processes (%):** 0.0

- **PDB ID:** 6P4G
- **Chain:** P
- **Crystallized protein length:** 136 aa
- **Resolution:** 3.1 Å
- **Alinged residues range:** 79-82, 79-83, 35-41
- **Aligned to segment part (indices):** 1, 3, 0
- **Alinged residues range of reference:** 779-782, 949-953, 1050-1056
- **b-phipsi:** 0.021276
- **w-rdist:** 0.798373
- **t-alpha:** 0.445813
- **Chemical similarity (Tanimoto Index) (%):** 73.46
- **1D identity (%) [PDB]:** 0.09
- **1D identity (%) [Gaps excluded][PDB]:** 100.0
- **1D identity - Alignment Gaps [PDB]:** 1117
- **2D identity (%) [PDB]:** 8.88
- **2D identity (%) [Gaps excluded][PDB]:** 85.71
- **2D identity - Alignment Gaps [PDB]:** 909
- **3D similarity (TM-Score) (%) [PDB]:** 6.05

- **Gene name:** N/A
- **RefSeq ID:** N/A
- **Sequence length:** N/A
- **5-UTR|CDS|3-UTR identity (%):** N/A | N/A | N/A
- **5-UTR|CDS|3-UTR identity (%) [Gaps excluded]:** N/A | N/A | N/A
- **5-UTR|CDS|3-UTR identity [Alignment Gaps]:** N/A | N/A | N/A

**Uniprot Description:**  
  
N/A  
  
**Gene Ontology Information:**

Molecular Function

- structural constituent of ribosome

Location

- ribosome

Biological process

- translation

---

31

- **Protein name:** Phosphoprotein
- **Organism:** Rabies virus (strain Pasteur vaccins / PV)
- **Uniprot Accession Number:** P06747
- **Protein sequence length:** 297 aa
- **1D identity (%):** 2.23
- **1D identity (%) [Gaps excluded]:** 23.36
- **1D identity - Alignment Gaps:** 1296
- **Common reported functions (%):** 0.0
- **Common reported locations (%):** 0.0
- **Common reported processes (%):** 20.0

- **PDB ID:** 3OA1
- **Chain:** A
- **Crystallized protein length:** 102 aa
- **Resolution:** 2.2 Å
- **Alinged residues range:** 265-282, 267-269, 234-240, 274-276
- **Aligned to segment part (indices):** 1, 2, 3, 0
- **Alinged residues range of reference:** 769-780, 888-890, 948-954, 1022-1024
- **b-phipsi:** 0.057689
- **w-rdist:** 0.460416
- **t-alpha:** 0.55291
- **Chemical similarity (Tanimoto Index) (%):** 82.89
- **1D identity (%) [PDB]:** 0.18
- **1D identity (%) [Gaps excluded][PDB]:** 66.67
- **1D identity - Alignment Gaps [PDB]:** 1080
- **2D identity (%) [PDB]:** 6.73
- **2D identity (%) [Gaps excluded][PDB]:** 90.67
- **2D identity - Alignment Gaps [PDB]:** 936
- **3D similarity (TM-Score) (%) [PDB]:** 6.09

- **Gene name:** P
- **RefSeq ID:** NC\_001542
- **Genomic sequence length:** 11932
- **5-UTR|CDS|3-UTR identity (%):** N/A | 16.37 | N/A
- **5-UTR|CDS|3-UTR identity (%) [Gaps excluded]:** N/A | 78.0 | N/A
- **5-UTR|CDS|3-UTR identity [Alignment Gaps]:** N/A | 3080 | N/A

**Uniprot Description:**  
  
Non catalytic polymerase cofactor and regulatory protein that plays a role in viral transcription and replication. Stabilizes the RNA polymerase L to the N-RNA template and binds the soluble protein N, preventing it from encapsidating non-genomic RNA. Also inhibits host IFN-alpha and IFN-beta signaling by binding and retaining phosphorylated STAT1 in the cytoplasm or by inhibiting the DNA binding of STAT1 in the nucleus. Might be involved, through interaction with host dynein, in intracellular microtubule-dependent virus transport of incoming virus from the synapse toward the cell body (By similarity).  
  
Phosphoprotein
Homotrimer when phosphorylated. This trimer is stabilized by binding to the L protein. Binds soluble protein N, and ribonucleocapsid. Interacts with host DYNLL1 and DYNLL2; this interaction may play a role in intracellular microtubule-dependent virus transport of incoming virus. Interacts with host STAT1, STAT2 and PML.  
  
**Gene Ontology Information:**

Molecular Function

- RNA-directed 5'-3' RNA polymerase activity

Location

- host cell cytoplasm
- host cell nucleus
- virion

Biological process

- microtubule-dependent intracellular transport of viral material towards nucleus
- suppression by virus of host STAT1 activity
- suppression by virus of host STAT2 activity
- suppression by virus of host type I interferon-mediated signaling pathway
- viral entry into host cell
- viral transcription

---

32

- **Protein name:** Core protein
- **Organism:** Meaban virus
- **Uniprot Accession Number:** A0EKU1
- **Protein sequence length:** 3421 aa
- **1D identity (%):** 7.62
- **1D identity (%) [Gaps excluded]:** 28.46
- **1D identity - Alignment Gaps:** 2712
- **Common reported functions (%):** 0.0
- **Common reported locations (%):** 25.0
- **Common reported processes (%):** 20.0

- **PDB ID:** 2OXT
- **Chain:** A
- **Crystallized protein length:** 265 aa
- **Resolution:** 2.9 Å
- **Alinged residues range:** 170-173, 64-66, 169-171, 60-64
- **Aligned to segment part (indices):** 1, 2, 3, 0
- **Alinged residues range of reference:** 776-779, 888-890, 948-950, 1021-1025
- **b-phipsi:** 0.034935
- **w-rdist:** 3.506868
- **t-alpha:** 0.005137
- **Chemical similarity (Tanimoto Index) (%):** 84.21
- **1D identity (%) [PDB]:** 0.0
- **1D identity (%) [Gaps excluded][PDB]:** 0.0
- **1D identity - Alignment Gaps [PDB]:** 1248
- **2D identity (%) [PDB]:** 10.9
- **2D identity (%) [Gaps excluded][PDB]:** 94.57
- **2D identity - Alignment Gaps [PDB]:** 990
- **3D similarity (TM-Score) (%) [PDB]:** 9.75

- **Gene name:** N/A
- **RefSeq ID:** NC\_033721
- **Genomic sequence length:** 10266
- **5-UTR|CDS|3-UTR identity (%):** N/A | 23.14 | N/A
- **5-UTR|CDS|3-UTR identity (%) [Gaps excluded]:** N/A | 80.22 | N/A
- **5-UTR|CDS|3-UTR identity [Alignment Gaps]:** N/A | 7780 | N/A

**Uniprot Description:**  
  
Functions as a signal peptide for NS4B and is required for the interferon antagonism activity of the latter.  
  
**Gene Ontology Information:**

Molecular Function

- ATP binding
- double-stranded RNA binding
- metal ion binding
- mRNA (guanine-N7-)-methyltransferase activity
- mRNA (nucleoside-2'-O-)-methyltransferase activity
- protein dimerization activity
- RNA helicase activity
- RNA-directed 5'-3' RNA polymerase activity
- serine-type endopeptidase activity

Location

- extracellular region
- host cell endoplasmic reticulum membrane
- host cell nucleus
- integral component of membrane
- viral capsid
- virion membrane

Biological process

- fusion of virus membrane with host endosome membrane
- induction by virus of host autophagy
- suppression by virus of host STAT2 activity
- suppression by virus of host type I interferon-mediated signaling pathway
- viral RNA genome replication
- virion attachment to host cell

---

33

- **Protein name:** HLA class II histocompatibility antigen, DRB1 beta chain
- **Organism:** Homo sapiens
- **Uniprot Accession Number:** P01911
- **Protein sequence length:** 266 aa
- **1D identity (%):** 3.75
- **1D identity (%) [Gaps excluded]:** 28.49
- **1D identity - Alignment Gaps:** 1181
- **Common reported functions (%):** 0.0
- **Common reported locations (%):** 12.5
- **Common reported processes (%):** 0.0

- **PDB ID:** 6CPO
- **Chain:** E
- **Crystallized protein length:** 190 aa
- **Resolution:** 2.4 Å
- **Alinged residues range:** 128-131, 65-67, 71-73, 65-70, 21-28
- **Aligned to segment part (indices):** 4, 1, 2, 3, 0
- **Alinged residues range of reference:** 725-728, 779-781, 888-890, 949-954, 1057-1064
- **b-phipsi:** 0.035431
- **w-rdist:** 0.419218
- **t-alpha:** 0.677143
- **Chemical similarity (Tanimoto Index) (%):** 85.48
- **1D identity (%) [PDB]:** 0.09
- **1D identity (%) [Gaps excluded][PDB]:** 100.0
- **1D identity - Alignment Gaps [PDB]:** 1171
- **2D identity (%) [PDB]:** 14.12
- **2D identity (%) [Gaps excluded][PDB]:** 89.38
- **2D identity - Alignment Gaps [PDB]:** 853
- **3D similarity (TM-Score) (%) [PDB]:** 7.96

- **Gene name:** HLA-DRB1
- **RefSeq ID:** N/A
- **Sequence length:** N/A
- **5-UTR|CDS|3-UTR identity (%):** N/A | N/A | N/A
- **5-UTR|CDS|3-UTR identity (%) [Gaps excluded]:** N/A | N/A | N/A
- **5-UTR|CDS|3-UTR identity [Alignment Gaps]:** N/A | N/A | N/A

**Uniprot Description:**  
  
A beta chain of antigen-presenting major histocompatibility complex class II (MHCII) molecule. In complex with the alpha chain HLA-DRA, displays antigenic peptides on professional antigen presenting cells (APCs) for recognition by alpha-beta T cell receptor (TCR) on HLA-DRB1-restricted CD4-positive T cells. This guides antigen-specific T-helper effector functions, both antibody-mediated immune response and macrophage activation, to ultimately eliminate the infectious agents and transformed cells (PubMed:29884618, PubMed:22327072, PubMed:27591323, PubMed:8642306, PubMed:15265931, PubMed:31495665, PubMed:16148104). Typically presents extracellular peptide antigens of 10 to 30 amino acids that arise from proteolysis of endocytosed antigens in lysosomes (PubMed:8145819). In the tumor microenvironment, presents antigenic peptides that are primarily generated in tumor-resident APCs likely via phagocytosis of apoptotic tumor cells or macropinocytosis of secreted tumor proteins (PubMed:31495665). Presents peptides derived from intracellular proteins that are trapped in autolysosomes after macroautophagy, a mechanism especially relevant for T cell selection in the thymus and central immune tolerance (PubMed:17182262, PubMed:23783831). The selection of the immunodominant epitopes follows two processing modes: 'bind first, cut/trim later' for pathogen-derived antigenic peptides and 'cut first, bind later' for autoantigens/self-peptides (PubMed:25413013). The anchor residue at position 1 of the peptide N-terminus, usually a large hydrophobic residue, is essential for high affinity interaction with MHCII molecules (PubMed:8145819).  
  
Heterotrimer that consists of an alpha chain HLA-DRA, a beta chain HLA-DRB1 and a peptide (peptide-MHCII) (PubMed:7477400, PubMed:9354468, PubMed:9782128, PubMed:31619516, PubMed:32668259). Newly synthesized alpha and beta chains forms a heterodimer (MHCII) that associates with the CD74/invariant chain (Ii) in the endoplasmic reticulum (ER). Ii is a trimer composed of three subunits and each subunit interacts with one MHCII dimer, blocking the peptide-binding cleft. As a result, MHCII molecules can not bind peptides present in the ER (PubMed:7479981). The complex of MHCII and CD74/Ii is transported in vesicles from ER to Golgi to lysosomes, where it encounters antigenic peptides generated via proteolysis of endocytosed antigens. MHCII dimers are dissociated from CD74/Ii by the combined action of proteolysis and HLA-DM (PubMed:25413013). Lysosomal enzymes such as cathepsin, degrade CD74/Ii leaving a 24 amino acid remnant called class II-associated Ii or CLIP. Interacts (via the peptide binding cleft) with CLIP; this interaction inhibits antigen peptide binding before entry in the endosomal compartment (PubMed:7477400, PubMed:9075930). The displacement of CLIP and replacement by a high affinity peptide in lysosomes is performed by HLA-DM heterodimer. HLA-DM catalyzes CLIP dissociation from MHCII, stabilizes empty MHCII and mediates the selection of high affinity peptides (PubMed:23260142, PubMed:11070170, PubMed:9075930). Interacts with HLA-DM heterodimer; this interaction is direct (PubMed:25413013). Interacts with TCR (via CDR3) (PubMed:29884618). Interacts (via beta-2 domain) with CD4 coreceptor (via Ig-like V-type domain); this interaction is of exceptionally low affinity yet necessary for optimal recognition of antigenic peptides (PubMed:21900604, PubMed:27114505).  
  
**Gene Ontology Information:**

Molecular Function

- CD4 receptor binding
- MHC class II protein complex binding
- MHC class II receptor activity
- peptide antigen binding
- polysaccharide binding
- structural constituent of cytoskeleton
- T cell receptor binding

Location

- cell surface
- clathrin-coated endocytic vesicle membrane
- endocytic vesicle membrane
- ER to Golgi transport vesicle membrane
- external side of plasma membrane
- extracellular exosome
- extracellular space
- Golgi membrane
- immunological synapse
- integral component of lumenal side of endoplasmic reticulum membrane
- integral component of plasma membrane
- intermediate filament
- late endosome membrane
- lysosomal membrane
- membrane
- MHC class II protein complex
- plasma membrane
- trans-Golgi network membrane
- transport vesicle membrane

Biological process

- antigen processing and presentation of endogenous peptide antigen via MHC class II
- antigen processing and presentation of exogenous peptide antigen via MHC class II
- detection of bacterium
- epidermis development
- humoral immune response
- immune response
- inflammatory response to antigenic stimulus
- interferon-gamma-mediated signaling pathway
- macrophage differentiation
- myeloid dendritic cell antigen processing and presentation
- negative regulation of inflammatory response to antigenic stimulus
- negative regulation of interferon-gamma production
- negative regulation of T cell proliferation
- peptide antigen assembly with MHC class II protein complex
- positive regulation of CD4-positive, alpha-beta T cell activation
- positive regulation of CD4-positive, CD25-positive, alpha-beta regulatory T cell differentiation
- positive regulation of ERK1 and ERK2 cascade
- positive regulation of I-kappaB kinase/NF-kappaB signaling
- positive regulation of insulin secretion involved in cellular response to glucose stimulus
- positive regulation of kinase activity
- positive regulation of MAPK cascade
- positive regulation of memory T cell differentiation
- positive regulation of monocyte differentiation
- positive regulation of protein phosphorylation
- positive regulation of T cell mediated cytotoxicity
- positive regulation of T cell mediated immune response to tumor cell
- positive regulation of transcription, DNA-templated
- positive regulation of viral entry into host cell
- protein tetramerization
- regulation of interleukin-10 production
- regulation of interleukin-4 production
- regulation of T-helper cell differentiation
- signal transduction
- T cell receptor signaling pathway
- T-helper 1 type immune response

---

34

- **Protein name:** Lysozyme
- **Organism:** Streptococcus phage Cp-1
- **Uniprot Accession Number:** P15057
- **Protein sequence length:** 339 aa
- **1D identity (%):** 4.05
- **1D identity (%) [Gaps excluded]:** 32.22
- **1D identity - Alignment Gaps:** 1252
- **Common reported functions (%):** 0.0
- **Common reported locations (%):** 0.0
- **Common reported processes (%):** 0.0

- **PDB ID:** 2J8G
- **Chain:** A
- **Crystallized protein length:** 338 aa
- **Resolution:** 1.69 Å
- **Alinged residues range:** 130-140, 138-140, 110-117
- **Aligned to segment part (indices):** 1, 3, 0
- **Alinged residues range of reference:** 771-781, 949-951, 1014-1023
- **b-phipsi:** 0.000495
- **w-rdist:** 0.797463
- **t-alpha:** 1.240458
- **Chemical similarity (Tanimoto Index) (%):** 88.86
- **1D identity (%) [PDB]:** 0.08
- **1D identity (%) [Gaps excluded][PDB]:** 100.0
- **1D identity - Alignment Gaps [PDB]:** 1319
- **2D identity (%) [PDB]:** 19.28
- **2D identity (%) [Gaps excluded][PDB]:** 90.52
- **2D identity - Alignment Gaps [PDB]:** 857
- **3D similarity (TM-Score) (%) [PDB]:** 14.13

- **Gene name:** CPL1
- **RefSeq ID:** NC\_001825
- **Genomic sequence length:** 19343
- **5-UTR|CDS|3-UTR identity (%):** N/A | 15.9 | N/A
- **5-UTR|CDS|3-UTR identity (%) [Gaps excluded]:** N/A | 82.06 | N/A
- **5-UTR|CDS|3-UTR identity [Alignment Gaps]:** N/A | 3270 | N/A

**Uniprot Description:**  
  
Responsible for the separation of the host daughter cells at the end of cell division and participates in the liberation of progeny bacteriophage into the medium. Strictly depends on the presence of choline-containing cell walls for activity.  
  
**Gene Ontology Information:**

Molecular Function

- lysozyme activity

Location  
  
N/A

Biological process

- cell wall macromolecule catabolic process
- cytolysis
- defense response to bacterium
- peptidoglycan catabolic process
- viral release from host cell by cytolysis

---

35

- **Protein name:** Non-structural protein S
- **Organism:** Rift valley fever virus (strain ZH-548 M12)
- **Uniprot Accession Number:** P21698
- **Protein sequence length:** 265 aa
- **1D identity (%):** 4.72
- **1D identity (%) [Gaps excluded]:** 31.19
- **1D identity - Alignment Gaps:** 1134
- **Common reported functions (%):** 0.0
- **Common reported locations (%):** 0.0
- **Common reported processes (%):** 10.0

- **PDB ID:** 5OOO
- **Chain:** A
- **Crystallized protein length:** 166 aa
- **Resolution:** 2.2 Å
- **Alinged residues range:** 139-142, 106-109, 135-146
- **Aligned to segment part (indices):** 1, 3, 0
- **Alinged residues range of reference:** 779-782, 947-950, 1017-1026
- **b-phipsi:** 0.035081
- **w-rdist:** 0.378873
- **t-alpha:** 0.736686
- **Chemical similarity (Tanimoto Index) (%):** 83.19
- **1D identity (%) [PDB]:** 0.0
- **1D identity (%) [Gaps excluded][PDB]:** 0.0
- **1D identity - Alignment Gaps [PDB]:** 1149
- **2D identity (%) [PDB]:** 12.41
- **2D identity (%) [Gaps excluded][PDB]:** 94.03
- **2D identity - Alignment Gaps [PDB]:** 881
- **3D similarity (TM-Score) (%) [PDB]:** 7.98

- **Gene name:** NSS
- **RefSeq ID:** NC\_014395
- **Genomic sequence length:** 1690
- **5-UTR|CDS|3-UTR identity (%):** N/A | 13.81 | N/A
- **5-UTR|CDS|3-UTR identity (%) [Gaps excluded]:** N/A | 78.81 | N/A
- **5-UTR|CDS|3-UTR identity [Alignment Gaps]:** N/A | 3242 | N/A

**Uniprot Description:**  
  
Plays a role in the escape of host innate immune response by promoting the degradation of host EIF2AK2/PKR and inhibiting host transcription. Cytoplasmic NSs interacts with host FBXW11 to degrade PKR whereas nuclear pool binds to host FBXO3 to target TFIIH subunit GTF2H1 for proteasomal degradation (PubMed:19197350, PubMed:19211744, PubMed:21543505, PubMed:23063407, PubMed:26837067). Forms filaments in the nucleus that may sequester NSs binding partners, causing cell cycle arrest (PubMed:28915104, PubMed:10233964).  
  
Multimerizes; forms 0.5-1 mm thick proteinaceous filaments in the nucleus (PubMed:10233964, PubMed:28915104). Interacts with host FBXW11; this interaction is important for PKR degradation (PubMed:21543505). Interacts with host FBXO3; this interaction is important for GT2H1 degradation (PubMed:26837067).  
  
**Gene Ontology Information:**

Molecular Function  
  
N/A

Location

- host cell cytoplasm
- host cell nucleus

Biological process

- negative stranded viral RNA replication
- suppression by virus of host gene expression
- suppression by virus of host PKR activity
- suppression by virus of host transcription
- suppression by virus of host transcription initiation from RNA polymerase II promoter
- suppression by virus of host type I interferon production
- suppression by virus of host type I interferon-mediated signaling pathway

---

36

- **Protein name:** Histone H2A type 1
- **Organism:** Xenopus laevis
- **Uniprot Accession Number:** P06897
- **Protein sequence length:** 130 aa
- **1D identity (%):** 2.0
- **1D identity (%) [Gaps excluded]:** 25.74
- **1D identity - Alignment Gaps:** 1201
- **Common reported functions (%):** 0.0
- **Common reported locations (%):** 0.0
- **Common reported processes (%):** 0.0

- **PDB ID:** 5F99
- **Chain:** G
- **Crystallized protein length:** 121 aa
- **Resolution:** 2.63 Å
- **Alinged residues range:** 86-95, 92-95, 47-53
- **Aligned to segment part (indices):** 1, 3, 0
- **Alinged residues range of reference:** 771-780, 947-950, 1020-1026
- **b-phipsi:** 0.082506
- **w-rdist:** 0.557095
- **t-alpha:** 0.486076
- **Chemical similarity (Tanimoto Index) (%):** 68.88
- **1D identity (%) [PDB]:** 0.0
- **1D identity (%) [Gaps excluded][PDB]:** 0.0
- **1D identity - Alignment Gaps [PDB]:** 1104
- **2D identity (%) [PDB]:** 7.89
- **2D identity (%) [Gaps excluded][PDB]:** 88.89
- **2D identity - Alignment Gaps [PDB]:** 924
- **3D similarity (TM-Score) (%) [PDB]:** 7.95

- **Gene name:** N/A
- **RefSeq ID:** N/A
- **Sequence length:** N/A
- **5-UTR|CDS|3-UTR identity (%):** N/A | N/A | N/A
- **5-UTR|CDS|3-UTR identity (%) [Gaps excluded]:** N/A | N/A | N/A
- **5-UTR|CDS|3-UTR identity [Alignment Gaps]:** N/A | N/A | N/A

**Uniprot Description:**  
  
Core component of nucleosome. Nucleosomes wrap and compact DNA into chromatin, limiting DNA accessibility to the cellular machineries which require DNA as a template. Histones thereby play a central role in transcription regulation, DNA repair, DNA replication and chromosomal stability. DNA accessibility is regulated via a complex set of post-translational modifications of histones, also called histone code, and nucleosome remodeling.  
  
The nucleosome is a histone octamer containing two molecules each of H2A, H2B, H3 and H4 assembled in one H3-H4 heterotetramer and two H2A-H2B heterodimers. The octamer wraps approximately 147 bp of DNA.  
  
**Gene Ontology Information:**

Molecular Function

- DNA binding
- protein heterodimerization activity

Location

- nucleosome
- nucleus

Biological process  
  
N/A

---

37

- **Protein name:** Middle transcription regulatory protein motA
- **Organism:** Enterobacteria phage T4
- **Uniprot Accession Number:** P22915
- **Protein sequence length:** 211 aa
- **1D identity (%):** 4.56
- **1D identity (%) [Gaps excluded]:** 31.05
- **1D identity - Alignment Gaps:** 1104
- **Common reported functions (%):** 0.0
- **Common reported locations (%):** 0.0
- **Common reported processes (%):** 0.0

- **PDB ID:** 1BJA
- **Chain:** A
- **Crystallized protein length:** 95 aa
- **Resolution:** 2.19 Å
- **Alinged residues range:** 74-78, 74-81, 34-38
- **Aligned to segment part (indices):** 1, 3, 0
- **Alinged residues range of reference:** 777-781, 947-954, 1015-1019
- **b-phipsi:** 0.020991
- **w-rdist:** 0.812936
- **t-alpha:** 0.493639
- **Chemical similarity (Tanimoto Index) (%):** 69.26
- **1D identity (%) [PDB]:** 0.09
- **1D identity (%) [Gaps excluded][PDB]:** 100.0
- **1D identity - Alignment Gaps [PDB]:** 1076
- **2D identity (%) [PDB]:** 7.09
- **2D identity (%) [Gaps excluded][PDB]:** 93.42
- **2D identity - Alignment Gaps [PDB]:** 926
- **3D similarity (TM-Score) (%) [PDB]:** 5.77

- **Gene name:** motA
- **RefSeq ID:** NC\_000866
- **Genomic sequence length:** 168903
- **5-UTR|CDS|3-UTR identity (%):** N/A | 11.47 | N/A
- **5-UTR|CDS|3-UTR identity (%) [Gaps excluded]:** N/A | 81.01 | N/A
- **5-UTR|CDS|3-UTR identity [Alignment Gaps]:** N/A | 3352 | N/A

**Uniprot Description:**  
  
Required for the transcriptional activation of middle promoters. Middle promoters are characterized by the presence of the conserved sequence [AT]3TGCTTNA (MotA box). MotA binds directly to MotA boxes.  
  
**Gene Ontology Information:**

Molecular Function

- DNA binding

Location  
  
N/A

Biological process  
  
N/A

---

38

- **Protein name:** Non-structural protein 1
- **Organism:** Influenza A virus (strain A/Puerto Rico/8/1934 H1N1)
- **Uniprot Accession Number:** P03496
- **Protein sequence length:** 230 aa
- **1D identity (%):** 3.89
- **1D identity (%) [Gaps excluded]:** 26.56
- **1D identity - Alignment Gaps:** 1119
- **Common reported functions (%):** 50.0
- **Common reported locations (%):** 0.0
- **Common reported processes (%):** 10.0

- **PDB ID:** 3O9T
- **Chain:** B
- **Crystallized protein length:** 121 aa
- **Resolution:** 2.2 Å
- **Alinged residues range:** 159-162, 172-176, 172-176, 128-131
- **Aligned to segment part (indices):** 4, 1, 3, 0
- **Alinged residues range of reference:** 725-728, 776-780, 950-954, 1061-1064
- **b-phipsi:** 0.130831
- **w-rdist:** 0.475694
- **t-alpha:** 0.508997
- **Chemical similarity (Tanimoto Index) (%):** 82.94
- **1D identity (%) [PDB]:** 0.09
- **1D identity (%) [Gaps excluded][PDB]:** 100.0
- **1D identity - Alignment Gaps [PDB]:** 1102
- **2D identity (%) [PDB]:** 9.81
- **2D identity (%) [Gaps excluded][PDB]:** 93.33
- **2D identity - Alignment Gaps [PDB]:** 894
- **3D similarity (TM-Score) (%) [PDB]:** 6.66

- **Gene name:** NS
- **RefSeq ID:** NC\_002020
- **Genomic sequence length:** 890
- **5-UTR|CDS|3-UTR identity (%):** N/A | 12.44 | N/A
- **5-UTR|CDS|3-UTR identity (%) [Gaps excluded]:** N/A | 81.3 | N/A
- **5-UTR|CDS|3-UTR identity [Alignment Gaps]:** N/A | 3317 | N/A

**Uniprot Description:**  
  
Prevents the establishment of the cellular antiviral state by inhibiting TRIM25-mediated DDX58 ubiquitination, which normally triggers the antiviral transduction signal that leads to the activation of type I IFN genes by transcription factors IRF3 and IRF7. Prevents human EIF2AK2/PKR activation, either by binding double-strand RNA, or by interacting directly with EIF2AK2/PKR. This function may be important at the very beginning of the infection, when NS1 is mainly present in the cytoplasm. Also binds poly(A) and U6 snRNA.  
  
Homodimer. Interacts with host TRIM25 (via coiled coil); this interaction specifically inhibits TRIM25 multimerization and TRIM25-mediated DDX58 CARD ubiquitination. Interacts with human EIF2AK2/PKR, CPSF4, IVNS1ABP and PABPN1.  
  
**Gene Ontology Information:**

Molecular Function

- identical protein binding
- RNA binding

Location

- host cell cytoplasm
- host cell nucleus

Biological process

- mitigation of host immune response by virus
- modification by virus of host mRNA processing
- modulation by virus of host cellular process
- necroptotic process
- suppression by virus of host mRNA processing
- suppression by virus of host PKR activity
- suppression by virus of host RIG-I activity
- suppression by virus of host type I interferon-mediated signaling pathway

---

39

- **Protein name:** Histone H2A type 1-B/E
- **Organism:** Homo sapiens
- **Uniprot Accession Number:** P04908
- **Protein sequence length:** 130 aa
- **1D identity (%):** 1.98
- **1D identity (%) [Gaps excluded]:** 28.26
- **1D identity - Alignment Gaps:** 1219
- **Common reported functions (%):** 0.0
- **Common reported locations (%):** 0.0
- **Common reported processes (%):** 0.0

- **PDB ID:** 5GTC
- **Chain:** G
- **Crystallized protein length:** 107 aa
- **Resolution:** 2.7 Å
- **Alinged residues range:** 91-95, 92-95, 47-53
- **Aligned to segment part (indices):** 1, 3, 0
- **Alinged residues range of reference:** 776-780, 947-950, 1020-1026
- **b-phipsi:** 0.175277
- **w-rdist:** 0.558818
- **t-alpha:** 0.482323
- **Chemical similarity (Tanimoto Index) (%):** 68.88
- **1D identity (%) [PDB]:** 0.0
- **1D identity (%) [Gaps excluded][PDB]:** 0.0
- **1D identity - Alignment Gaps [PDB]:** 1090
- **2D identity (%) [PDB]:** 6.35
- **2D identity (%) [Gaps excluded][PDB]:** 98.48
- **2D identity - Alignment Gaps [PDB]:** 958
- **3D similarity (TM-Score) (%) [PDB]:** 7.11

- **Gene name:** H2AC4
- **RefSeq ID:** NM\_021052
- **Transcript sequence length:** 517
- **5-UTR|CDS|3-UTR identity (%):** 12.32 | 6.34 | 16.94
- **5-UTR|CDS|3-UTR identity (%) [Gaps excluded]:** 79.07 | 73.87 | 71.93
- **5-UTR|CDS|3-UTR identity [Alignment Gaps]:** 233 | 3549 | 185

**Uniprot Description:**  
  
Core component of nucleosome. Nucleosomes wrap and compact DNA into chromatin, limiting DNA accessibility to the cellular machineries which require DNA as a template. Histones thereby play a central role in transcription regulation, DNA repair, DNA replication and chromosomal stability. DNA accessibility is regulated via a complex set of post-translational modifications of histones, also called histone code, and nucleosome remodeling.  
  
The nucleosome is a histone octamer containing two molecules each of H2A, H2B, H3 and H4 assembled in one H3-H4 heterotetramer and two H2A-H2B heterodimers. The octamer wraps approximately 147 bp of DNA.  
  
**Gene Ontology Information:**

Molecular Function

- DNA binding
- protein heterodimerization activity

Location

- extracellular exosome
- nucleosome
- nucleus

Biological process

- chromatin silencing
- negative regulation of cell population proliferation

---

40

- **Protein name:** Histone H2A type 1
- **Organism:** Homo sapiens
- **Uniprot Accession Number:** P0C0S8
- **Protein sequence length:** 130 aa
- **1D identity (%):** 1.98
- **1D identity (%) [Gaps excluded]:** 28.26
- **1D identity - Alignment Gaps:** 1219
- **Common reported functions (%):** 0.0
- **Common reported locations (%):** 0.0
- **Common reported processes (%):** 0.0

- **PDB ID:** 6RNY
- **Chain:** G
- **Crystallized protein length:** 110 aa
- **Resolution:** 3.9 Å
- **Alinged residues range:** 91-95, 92-95, 47-53
- **Aligned to segment part (indices):** 1, 3, 0
- **Alinged residues range of reference:** 776-780, 947-950, 1020-1026
- **b-phipsi:** 0.069784
- **w-rdist:** 0.732092
- **t-alpha:** 0.482323
- **Chemical similarity (Tanimoto Index) (%):** N/A
- **1D identity (%) [PDB]:** 0.0
- **1D identity (%) [Gaps excluded][PDB]:** 0.0
- **1D identity - Alignment Gaps [PDB]:** 1093
- **2D identity (%) [PDB]:** 5.21
- **2D identity (%) [Gaps excluded][PDB]:** 96.43
- **2D identity - Alignment Gaps [PDB]:** 981
- **3D similarity (TM-Score) (%) [PDB]:** 6.99

- **Gene name:** H2AC11
- **RefSeq ID:** NM\_021064
- **Transcript sequence length:** 493
- **5-UTR|CDS|3-UTR identity (%):** 7.41 | 6.37 | 18.85
- **5-UTR|CDS|3-UTR identity (%) [Gaps excluded]:** 83.33 | 77.74 | 82.14
- **5-UTR|CDS|3-UTR identity [Alignment Gaps]:** 246 | 3577 | 188

**Uniprot Description:**  
  
Core component of nucleosome. Nucleosomes wrap and compact DNA into chromatin, limiting DNA accessibility to the cellular machineries which require DNA as a template. Histones thereby play a central role in transcription regulation, DNA repair, DNA replication and chromosomal stability. DNA accessibility is regulated via a complex set of post-translational modifications of histones, also called histone code, and nucleosome remodeling.  
  
The nucleosome is a histone octamer containing two molecules each of H2A, H2B, H3 and H4 assembled in one H3-H4 heterotetramer and two H2A-H2B heterodimers. The octamer wraps approximately 147 bp of DNA.  
  
**Gene Ontology Information:**

Molecular Function

- DNA binding
- enzyme binding
- protein heterodimerization activity

Location

- extracellular exosome
- nucleosome
- nucleus

Biological process

- chromatin silencing

---

41

- **Protein name:** Uracil-DNA glycosylase
- **Organism:** Vaccinia virus (strain Ankara)
- **Uniprot Accession Number:** Q91UM2
- **Protein sequence length:** 218 aa
- **1D identity (%):** 3.96
- **1D identity (%) [Gaps excluded]:** 29.38
- **1D identity - Alignment Gaps:** 1137
- **Common reported functions (%):** 0.0
- **Common reported locations (%):** 0.0
- **Common reported processes (%):** 0.0

- **PDB ID:** 2OWQ
- **Chain:** B
- **Crystallized protein length:** 224 aa
- **Resolution:** 2.4 Å
- **Alinged residues range:** 154-156, 190-197, 196-199, 193-197
- **Aligned to segment part (indices):** 4, 1, 3, 0
- **Alinged residues range of reference:** 726-728, 774-781, 950-953, 1014-1018
- **b-phipsi:** 0.072247
- **w-rdist:** 0.606415
- **t-alpha:** 0.608219
- **Chemical similarity (Tanimoto Index) (%):** 83.55
- **1D identity (%) [PDB]:** 0.08
- **1D identity (%) [Gaps excluded][PDB]:** 50.0
- **1D identity - Alignment Gaps [PDB]:** 1196
- **2D identity (%) [PDB]:** 11.21
- **2D identity (%) [Gaps excluded][PDB]:** 86.23
- **2D identity - Alignment Gaps [PDB]:** 924
- **3D similarity (TM-Score) (%) [PDB]:** 7.95

- **Gene name:** UNG
- **RefSeq ID:** NC\_006998
- **Genomic sequence length:** 194711
- **5-UTR|CDS|3-UTR identity (%):** N/A | 11.15 | N/A
- **5-UTR|CDS|3-UTR identity (%) [Gaps excluded]:** N/A | 77.9 | N/A
- **5-UTR|CDS|3-UTR identity [Alignment Gaps]:** N/A | 3357 | N/A

**Uniprot Description:**  
  
Excises uracil residues from the DNA which can arise as a result of misincorporation of dUMP residues by DNA polymerase or due to deamination of cytosine. Also part of a heterodimeric processivity factor which potentiates the DNA polymerase activity. Binds to DNA.  
  
Homodimer. Interacts with protein A20. Component of the Uracil-DNA glycosylase(UDG)-A20-polymerase complex; A20 and UDG form a heterodimeric processivity factor that associates with E9 to form the processive polymerase holoenzyme (By similarity).  
  
**Gene Ontology Information:**

Molecular Function

- DNA binding
- hydrolase activity, hydrolyzing N-glycosyl compounds

Location  
  
N/A

Biological process

- DNA repair

---

42

- **Protein name:** Fibritin
- **Organism:** Enterobacteria phage T4
- **Uniprot Accession Number:** P10104
- **Protein sequence length:** 487 aa
- **1D identity (%):** 7.64
- **1D identity (%) [Gaps excluded]:** 26.13
- **1D identity - Alignment Gaps:** 964
- **Common reported functions (%):** 0.0
- **Common reported locations (%):** 0.0
- **Common reported processes (%):** 0.0

- **PDB ID:** 6JX7
- **Chain:** A
- **Crystallized protein length:** 1245 aa
- **Resolution:** 3.31 Å
- **Alinged residues range:** 312-314, 1076-1080, 1115-1117, 1315-1326, 1189-1232
- **Aligned to segment part (indices):** 4, 1, 2, 3, 0
- **Alinged residues range of reference:** 726-728, 773-777, 888-890, 949-954, 1016-1059
- **b-phipsi:** 0.030904
- **w-rdist:** 0.722975
- **t-alpha:** 0.686782
- **Chemical similarity (Tanimoto Index) (%):** 94.27
- **1D identity (%) [PDB]:** 0.0
- **1D identity (%) [Gaps excluded][PDB]:** 0.0
- **1D identity - Alignment Gaps [PDB]:** 2230
- **2D identity (%) [PDB]:** 48.51
- **2D identity (%) [Gaps excluded][PDB]:** 88.24
- **2D identity - Alignment Gaps [PDB]:** 648
- **3D similarity (TM-Score) (%) [PDB]:** 62.85

- **Gene name:** wac
- **RefSeq ID:** NC\_000866
- **Genomic sequence length:** 168903
- **5-UTR|CDS|3-UTR identity (%):** N/A | 27.1 | N/A
- **5-UTR|CDS|3-UTR identity (%) [Gaps excluded]:** N/A | 80.92 | N/A
- **5-UTR|CDS|3-UTR identity [Alignment Gaps]:** N/A | 2634 | N/A

**Uniprot Description:**  
  
Chaperone involved in tail fiber assembly and retraction. Acts as a chaperone helping to attach the long tail fibers to the virus during the assembly process. During phage assembly, twelve fibritin molecules attach to the phage neck via gp13: six molecules forming the collar and six molecules forming the whiskers.  
  
Homotrimer (PubMed:9261070, PubMed:15033360, PubMed:19361528). Interacts (via N-terminal domain) with neck protein gp13; this interaction allows attachment of the fibrous collar and wiskers (PubMed:23434847).  
  
**Gene Ontology Information:**

Molecular Function  
  
N/A

Location

- virion

Biological process  
  
N/A

---

43

- **Protein name:** Translation repressor protein
- **Organism:** Enterobacteria phage T4
- **Uniprot Accession Number:** P69702
- **Protein sequence length:** 122 aa
- **1D identity (%):** 2.38
- **1D identity (%) [Gaps excluded]:** 32.63
- **1D identity - Alignment Gaps:** 1205
- **Common reported functions (%):** 0.0
- **Common reported locations (%):** 0.0
- **Common reported processes (%):** 0.0

- **PDB ID:** 1REG
- **Chain:** X
- **Crystallized protein length:** 122 aa
- **Resolution:** 1.9 Å
- **Alinged residues range:** 31-33, 65-68, 65-69, 46-48
- **Aligned to segment part (indices):** 4, 1, 3, 0
- **Alinged residues range of reference:** 726-728, 774-777, 947-951, 1059-1061
- **b-phipsi:** 0.044521
- **w-rdist:** 0.670839
- **t-alpha:** 0.639665
- **Chemical similarity (Tanimoto Index) (%):** 82.68
- **1D identity (%) [PDB]:** 0.27
- **1D identity (%) [Gaps excluded][PDB]:** 75.0
- **1D identity - Alignment Gaps [PDB]:** 1097
- **2D identity (%) [PDB]:** 6.47
- **2D identity (%) [Gaps excluded][PDB]:** 97.1
- **2D identity - Alignment Gaps [PDB]:** 967
- **3D similarity (TM-Score) (%) [PDB]:** 5.44

- **Gene name:** regA
- **RefSeq ID:** NC\_000866
- **Genomic sequence length:** 168903
- **5-UTR|CDS|3-UTR identity (%):** N/A | 6.96 | N/A
- **5-UTR|CDS|3-UTR identity (%) [Gaps excluded]:** N/A | 82.26 | N/A
- **5-UTR|CDS|3-UTR identity [Alignment Gaps]:** N/A | 3537 | N/A

**Uniprot Description:**  
  
Controls the translation of a number of proteins (such as regA itself, rIIB and at least 35 others) by binding to their mRNA.  
  
**Gene Ontology Information:**

Molecular Function

- RNA binding

Location  
  
N/A

Biological process

- regulation of translation

---

44

- **Protein name:** Uncharacterized protein
- **Organism:** Pseudomonas phage LUZ7
- **Uniprot Accession Number:** C8ZKB3
- **Protein sequence length:** 99 aa
- **1D identity (%):** 1.78
- **1D identity (%) [Gaps excluded]:** 29.11
- **1D identity - Alignment Gaps:** 1214
- **Common reported functions (%):** 0.0
- **Common reported locations (%):** 0.0
- **Common reported processes (%):** 0.0

- **PDB ID:** 6QLC
- **Chain:** A
- **Crystallized protein length:** 82 aa
- **Resolution:** 2.2 Å
- **Alinged residues range:** 34-36, 70-72, 52-55, 70-75
- **Aligned to segment part (indices):** 1, 2, 3, 0
- **Alinged residues range of reference:** 786-788, 888-890, 947-950, 1020-1025
- **b-phipsi:** 0.004842
- **w-rdist:** 0.976369
- **t-alpha:** 0.817337
- **Chemical similarity (Tanimoto Index) (%):** 73.17
- **1D identity (%) [PDB]:** 0.0
- **1D identity (%) [Gaps excluded][PDB]:** 0.0
- **1D identity - Alignment Gaps [PDB]:** 1065
- **2D identity (%) [PDB]:** 5.77
- **2D identity (%) [Gaps excluded][PDB]:** 98.31
- **2D identity - Alignment Gaps [PDB]:** 947
- **3D similarity (TM-Score) (%) [PDB]:** 4.56

- **Gene name:** N/A
- **RefSeq ID:** NC\_013691
- **Genomic sequence length:** 74901
- **5-UTR|CDS|3-UTR identity (%):** N/A | 5.15 | N/A
- **5-UTR|CDS|3-UTR identity (%) [Gaps excluded]:** N/A | 77.13 | N/A
- **5-UTR|CDS|3-UTR identity [Alignment Gaps]:** N/A | 3606 | N/A

**Uniprot Description:**  
  
N/A  
  
**Gene Ontology Information:**

Molecular Function  
  
N/A

Location  
  
N/A

Biological process  
  
N/A

---

45

- **Protein name:** Vesicle-trafficking protein SEC22b
- **Organism:** Homo sapiens
- **Uniprot Accession Number:** O75396
- **Protein sequence length:** 215 aa
- **1D identity (%):** 4.26
- **1D identity (%) [Gaps excluded]:** 27.78
- **1D identity - Alignment Gaps:** 1092
- **Common reported functions (%):** 0.0
- **Common reported locations (%):** 25.0
- **Common reported processes (%):** 0.0

- **PDB ID:** 3EGD
- **Chain:** C
- **Crystallized protein length:** 137 aa
- **Resolution:** 2.7 Å
- **Alinged residues range:** 118-124, 119-121, 82-85
- **Aligned to segment part (indices):** 1, 3, 0
- **Alinged residues range of reference:** 771-777, 952-954, 1023-1026
- **b-phipsi:** 0.128141
- **w-rdist:** 0.65172
- **t-alpha:** 0.532637
- **Chemical similarity (Tanimoto Index) (%):** 73.71
- **1D identity (%) [PDB]:** 0.0
- **1D identity (%) [Gaps excluded][PDB]:** 0.0
- **1D identity - Alignment Gaps [PDB]:** 1122
- **2D identity (%) [PDB]:** 8.05
- **2D identity (%) [Gaps excluded][PDB]:** 91.21
- **2D identity - Alignment Gaps [PDB]:** 940
- **3D similarity (TM-Score) (%) [PDB]:** 8.04

- **Gene name:** SEC22B
- **RefSeq ID:** NM\_004892
- **Transcript sequence length:** 6927
- **5-UTR|CDS|3-UTR identity (%):** 29.82 | 11.98 | 2.84
- **5-UTR|CDS|3-UTR identity (%) [Gaps excluded]:** 71.43 | 80.21 | 82.16
- **5-UTR|CDS|3-UTR identity [Alignment Gaps]:** 166 | 3308 | 5943

**Uniprot Description:**  
  
SNARE involved in targeting and fusion of ER-derived transport vesicles with the Golgi complex as well as Golgi-derived retrograde transport vesicles with the ER.  
  
Interacts with STX17 (By similarity). Component of two distinct SNARE complexes consisting of STX5, GOSR2/BOS1, BET1 and SEC22B or STX18, USE1L, BNIP1/SEC20L and SEC22B. YKT6 can probably replace SEC22B in either complex. Interacts with the COPII Sec23/24 complex composed of SEC23A and SEC24A; recruits SEC22B into COPII-coated vesicles to allow its transport from the endoplasmic reticulum to the Golgi (PubMed:17499046, PubMed:18843296).  
  
**Gene Ontology Information:**

Molecular Function

- SNAP receptor activity
- syntaxin binding

Location

- COPI-coated vesicle
- endoplasmic reticulum membrane
- endoplasmic reticulum-Golgi intermediate compartment
- endoplasmic reticulum-Golgi intermediate compartment membrane
- ER to Golgi transport vesicle membrane
- Golgi membrane
- integral component of membrane
- melanosome
- phagocytic vesicle membrane
- SNARE complex
- synaptic vesicle
- transport vesicle

Biological process

- antigen processing and presentation of exogenous peptide antigen via MHC class I, TAP-dependent
- COPII vesicle coating
- endoplasmic reticulum to Golgi vesicle-mediated transport
- negative regulation of autophagosome assembly
- positive regulation of protein catabolic process
- protein transport
- retrograde vesicle-mediated transport, Golgi to endoplasmic reticulum
- vesicle fusion with endoplasmic reticulum-Golgi intermediate compartment (ERGIC) membrane
- vesicle fusion with Golgi apparatus

---

46

- **Protein name:** Poly-gamma-glutamate hydrolase
- **Organism:** Bacillus phage phiNIT1
- **Uniprot Accession Number:** Q852V1
- **Protein sequence length:** 208 aa
- **1D identity (%):** 3.54
- **1D identity (%) [Gaps excluded]:** 25.41
- **1D identity - Alignment Gaps:** 1119
- **Common reported functions (%):** 0.0
- **Common reported locations (%):** 0.0
- **Common reported processes (%):** 0.0

- **PDB ID:** 3A9L
- **Chain:** A
- **Crystallized protein length:** 207 aa
- **Resolution:** 1.9 Å
- **Alinged residues range:** 198-206, 197-201, 169-173
- **Aligned to segment part (indices):** 1, 3, 0
- **Alinged residues range of reference:** 771-778, 950-954, 1016-1020
- **b-phipsi:** 0.034362
- **w-rdist:** 0.755253
- **t-alpha:** 0.736686
- **Chemical similarity (Tanimoto Index) (%):** 78.13
- **1D identity (%) [PDB]:** 0.0
- **1D identity (%) [Gaps excluded][PDB]:** 0.0
- **1D identity - Alignment Gaps [PDB]:** 1190
- **2D identity (%) [PDB]:** 11.61
- **2D identity (%) [Gaps excluded][PDB]:** 87.77
- **2D identity - Alignment Gaps [PDB]:** 912
- **3D similarity (TM-Score) (%) [PDB]:** 9.03

- **Gene name:** pghP
- **RefSeq ID:** NC\_021856
- **Genomic sequence length:** 155631
- **5-UTR|CDS|3-UTR identity (%):** N/A | 12.08 | N/A
- **5-UTR|CDS|3-UTR identity (%) [Gaps excluded]:** N/A | 80.24 | N/A
- **5-UTR|CDS|3-UTR identity [Alignment Gaps]:** N/A | 3285 | N/A

**Uniprot Description:**  
  
N/A  
  
**Gene Ontology Information:**

Molecular Function

- hydrolase activity
- metal ion binding

Location  
  
N/A

Biological process  
  
N/A

---

47

- **Protein name:** Terminase, large subunit
- **Organism:** Bacillus phage SPP1
- **Uniprot Accession Number:** P54308
- **Protein sequence length:** 422 aa
- **1D identity (%):** 5.67
- **1D identity (%) [Gaps excluded]:** 32.8
- **1D identity - Alignment Gaps:** 1195
- **Common reported functions (%):** 0.0
- **Common reported locations (%):** 0.0
- **Common reported processes (%):** 0.0

- **PDB ID:** 2WC9
- **Chain:** A
- **Crystallized protein length:** 176 aa
- **Resolution:** 2.5 Å
- **Alinged residues range:** 351-354, 350-352, 318-320
- **Aligned to segment part (indices):** 1, 3, 0
- **Alinged residues range of reference:** 776-779, 952-954, 1060-1062
- **b-phipsi:** 0.010441
- **w-rdist:** 0.843295
- **t-alpha:** 0.912052
- **Chemical similarity (Tanimoto Index) (%):** 79.75
- **1D identity (%) [PDB]:** 0.0
- **1D identity (%) [Gaps excluded][PDB]:** 0.0
- **1D identity - Alignment Gaps [PDB]:** 1159
- **2D identity (%) [PDB]:** 9.52
- **2D identity (%) [Gaps excluded][PDB]:** 91.74
- **2D identity - Alignment Gaps [PDB]:** 941
- **3D similarity (TM-Score) (%) [PDB]:** 7.98

- **Gene name:** 2
- **RefSeq ID:** NC\_004166
- **Genomic sequence length:** 44010
- **5-UTR|CDS|3-UTR identity (%):** N/A | 23.12 | N/A
- **5-UTR|CDS|3-UTR identity (%) [Gaps excluded]:** N/A | 79.22 | N/A
- **5-UTR|CDS|3-UTR identity [Alignment Gaps]:** N/A | 2791 | N/A

**Uniprot Description:**  
  
The terminase large subunit acts as an ATP driven molecular motor necessary for viral DNA translocation into empty capsids and as an endonuclease that cuts the viral genome to initiate and to end a packaging reaction (PubMed:10930407). The terminase lies at a unique vertex of the procapsid and is composed of two subunits, a small terminase subunit involved in viral DNA recognition (packaging sequence), and a large terminase subunit possessing endonucleolytic and ATPase activities (PubMed:23419885). Both terminase subunits heterooligomerize and are docked on the portal protein to form the packaging machine (PubMed:23419885). The terminase large subunit exhibits endonuclease activity and cleaves the viral genome concatemer once the capsid is full (headful packaging) (Probable) (PubMed:10930407). Once the capsid is packaged with the DNA, the terminase complex is substituted by the adapter (gp15) and the stopper protein (gp16) that form the connector (Probable).  
  
Monomer. Interacts with the terminase small subunit; the active complex is probably composed of two decameric ring-shaped terminase small subunit and two monomeric terminase large subunit (PubMed:12697751). Interacts with the portal protein (PubMed:23118480, PubMed:12697751).  
  
**Gene Ontology Information:**

Molecular Function

- ATP binding
- ATPase activity
- endonuclease activity
- metal ion binding
- nuclease activity

Location

- viral terminase, large subunit

Biological process

- DNA packaging
- viral DNA genome packaging

---

48

- **Protein name:** Protein L1
- **Organism:** Vaccinia virus (strain Western Reserve)
- **Uniprot Accession Number:** P07612
- **Protein sequence length:** 250 aa
- **1D identity (%):** 3.53
- **1D identity (%) [Gaps excluded]:** 29.09
- **1D identity - Alignment Gaps:** 1193
- **Common reported functions (%):** 0.0
- **Common reported locations (%):** 37.5
- **Common reported processes (%):** 10.0

- **PDB ID:** 2I9L
- **Chain:** L
- **Crystallized protein length:** 173 aa
- **Resolution:** 3.1 Å
- **Alinged residues range:** 105-113, 91-93, 107-114, 61-74
- **Aligned to segment part (indices):** 1, 2, 3, 0
- **Alinged residues range of reference:** 772-780, 888-890, 947-954, 1020-1028
- **b-phipsi:** 0.087438
- **w-rdist:** 0.859412
- **t-alpha:** 0.424757
- **Chemical similarity (Tanimoto Index) (%):** 75.85
- **1D identity (%) [PDB]:** 0.09
- **1D identity (%) [Gaps excluded][PDB]:** 50.0
- **1D identity - Alignment Gaps [PDB]:** 1152
- **2D identity (%) [PDB]:** 10.34
- **2D identity (%) [Gaps excluded][PDB]:** 88.43
- **2D identity - Alignment Gaps [PDB]:** 914
- **3D similarity (TM-Score) (%) [PDB]:** 9.04

- **Gene name:** VACWR088
- **RefSeq ID:** NC\_006998
- **Genomic sequence length:** 194711
- **5-UTR|CDS|3-UTR identity (%):** N/A | 13.77 | N/A
- **5-UTR|CDS|3-UTR identity (%) [Gaps excluded]:** N/A | 79.67 | N/A
- **5-UTR|CDS|3-UTR identity [Alignment Gaps]:** N/A | 3227 | N/A

**Uniprot Description:**  
  
Envelope protein which probably plays a role in virus entry into the host cell. Is probably involved in the virus attachment to the host cell surface and associates with the entry/fusion complex (EFC). Needed for fusion and penetration of the virus core into host cell.  
  
Interacts with G4; this interaction involves formation of a transient disulfide-bonded intermediate, allowing disulfide bond transfer.  
  
**Gene Ontology Information:**

Molecular Function  
  
N/A

Location

- integral component of membrane
- viral envelope
- virion membrane

Biological process

- viral entry into host cell
- virion attachment to host cell

---

49

- **Protein name:** Neck appendage protein
- **Organism:** Bacillus phage GA-1
- **Uniprot Accession Number:** Q9FZW3
- **Protein sequence length:** 740 aa
- **1D identity (%):** 7.58
- **1D identity (%) [Gaps excluded]:** 27.91
- **1D identity - Alignment Gaps:** 1153
- **Common reported functions (%):** 50.0
- **Common reported locations (%):** 0.0
- **Common reported processes (%):** 0.0

- **PDB ID:** 3GUD
- **Chain:** A
- **Crystallized protein length:** 119 aa
- **Resolution:** 2.2 Å
- **Alinged residues range:** 715-718, 652-675, 669-673, 645-647
- **Aligned to segment part (indices):** 4, 1, 3, 0
- **Alinged residues range of reference:** 725-728, 771-782, 949-953, 1053-1055
- **b-phipsi:** 0.068979
- **w-rdist:** 0.673331
- **t-alpha:** 0.875399
- **Chemical similarity (Tanimoto Index) (%):** 82.63
- **1D identity (%) [PDB]:** 0.0
- **1D identity (%) [Gaps excluded][PDB]:** 0.0
- **1D identity - Alignment Gaps [PDB]:** 1102
- **2D identity (%) [PDB]:** 6.64
- **2D identity (%) [Gaps excluded][PDB]:** 87.18
- **2D identity - Alignment Gaps [PDB]:** 946
- **3D similarity (TM-Score) (%) [PDB]:** 7.71

- **Gene name:** gene 12
- **RefSeq ID:** NC\_002649
- **Genomic sequence length:** 21129
- **5-UTR|CDS|3-UTR identity (%):** N/A | 37.71 | N/A
- **5-UTR|CDS|3-UTR identity (%) [Gaps excluded]:** N/A | 79.97 | N/A
- **5-UTR|CDS|3-UTR identity [Alignment Gaps]:** N/A | 2171 | N/A

**Uniprot Description:**  
  
N/A  
  
**Gene Ontology Information:**

Molecular Function

- identical protein binding

Location

- virus tail, fiber

Biological process  
  
N/A

---

50

- **Protein name:** PDZ and LIM domain protein 2
- **Organism:** Homo sapiens
- **Uniprot Accession Number:** Q96JY6
- **Protein sequence length:** 352 aa
- **1D identity (%):** 4.97
- **1D identity (%) [Gaps excluded]:** 26.46
- **1D identity - Alignment Gaps:** 1111
- **Common reported functions (%):** 0.0
- **Common reported locations (%):** 0.0
- **Common reported processes (%):** 0.0

- **PDB ID:** 3PDV
- **Chain:** A
- **Crystallized protein length:** 89 aa
- **Resolution:** 2.2 Å
- **Alinged residues range:** 62-68, 63-65, 37-40, 24-28
- **Aligned to segment part (indices):** 1, 2, 3, 0
- **Alinged residues range of reference:** 773-779, 888-890, 947-950, 1058-1062
- **b-phipsi:** 0.101983
- **w-rdist:** 0.798889
- **t-alpha:** 0.56117
- **Chemical similarity (Tanimoto Index) (%):** 79.77
- **1D identity (%) [PDB]:** 0.0
- **1D identity (%) [Gaps excluded][PDB]:** 0.0
- **1D identity - Alignment Gaps [PDB]:** 1072
- **2D identity (%) [PDB]:** 5.98
- **2D identity (%) [Gaps excluded][PDB]:** 86.96
- **2D identity - Alignment Gaps [PDB]:** 934
- **3D similarity (TM-Score) (%) [PDB]:** 5.75

- **Gene name:** PDLIM2
- **RefSeq ID:** NM\_198042
- **Transcript sequence length:** 1459
- **5-UTR|CDS|3-UTR identity (%):** 27.82 | 14.23 | 22.32
- **5-UTR|CDS|3-UTR identity (%) [Gaps excluded]:** 71.17 | 74.87 | 77.64
- **5-UTR|CDS|3-UTR identity [Alignment Gaps]:** 173 | 3171 | 399

**Uniprot Description:**  
  
Probable adapter protein located at the actin cytoskeleton that promotes cell attachment. Necessary for the migratory capacity of epithelial cells. Overexpression enhances cell adhesion to collagen and fibronectin and suppresses anchorage independent growth. May contribute to tumor cell migratory capacity.  
  
Interacts with alpha-actinins ACTN1 and ACTN4, FLNA and MYH9.  
  
**Gene Ontology Information:**

Molecular Function

- actin binding
- metal ion binding
- muscle alpha-actinin binding

Location

- adherens junction
- filamentous actin
- nucleus
- stress fiber
- Z disc

Biological process

- actin cytoskeleton organization
- heart development
- muscle structure development

---

51

- **Protein name:** N/A
- **Organism:** N/A
- **Uniprot Accession Number:** F5HCP3
- **Protein sequence length:** N/A
- **1D identity (%):** N/A
- **1D identity (%) [Gaps excluded]:** N/A
- **1D identity - Alignment Gaps:** N/A
- **Common reported functions (%):** 0.0
- **Common reported locations (%):** 25.0
- **Common reported processes (%):** 10.0

- **PDB ID:** 5VOD
- **Chain:** D
- **Crystallized protein length:** 164 aa
- **Resolution:** 5.9 Å
- **Alinged residues range:** 141-145, 147-149, 108-111, 144-150
- **Aligned to segment part (indices):** 1, 2, 3, 0
- **Alinged residues range of reference:** 772-776, 888-890, 949-952, 1016-1023
- **b-phipsi:** 0.257087
- **w-rdist:** 0.712832
- **t-alpha:** 0.677143
- **Chemical similarity (Tanimoto Index) (%):** 94.73
- **1D identity (%) [PDB]:** 0.09
- **1D identity (%) [Gaps excluded][PDB]:** 50.0
- **1D identity - Alignment Gaps [PDB]:** 1143
- **2D identity (%) [PDB]:** 12.33
- **2D identity (%) [Gaps excluded][PDB]:** 87.94
- **2D identity - Alignment Gaps [PDB]:** 865
- **3D similarity (TM-Score) (%) [PDB]:** 8.21

- **Gene name:** N/A
- **RefSeq ID:** N/A
- **Sequence length:** N/A
- **5-UTR|CDS|3-UTR identity (%):** N/A | N/A | N/A
- **5-UTR|CDS|3-UTR identity (%) [Gaps excluded]:** N/A | N/A | N/A
- **5-UTR|CDS|3-UTR identity [Alignment Gaps]:** N/A | N/A | N/A

**Uniprot Description:**  
  
N/A  
  
**Gene Ontology Information:**

Molecular Function  
  
N/A

Location

- viral envelope
- virion membrane

Biological process

- entry receptor-mediated virion attachment to host cell
- viral entry into host cell
- viral exocytosis
- viral life cycle
- viral process
- viral transcription

---

52

- **Protein name:** Endolysin
- **Organism:** Enterobacteria phage T4
- **Uniprot Accession Number:** P00720
- **Protein sequence length:** 164 aa
- **1D identity (%):** 3.27
- **1D identity (%) [Gaps excluded]:** 27.81
- **1D identity - Alignment Gaps:** 1135
- **Common reported functions (%):** 0.0
- **Common reported locations (%):** 0.0
- **Common reported processes (%):** 0.0

- **PDB ID:** 3JR6
- **Chain:** C
- **Crystallized protein length:** 159 aa
- **Resolution:** 3.0 Å
- **Alinged residues range:** 66-73, 110-112, 74-78, 102-111
- **Aligned to segment part (indices):** 1, 2, 3, 0
- **Alinged residues range of reference:** 775-782, 888-890, 949-953, 1014-1023
- **b-phipsi:** 0.404281
- **w-rdist:** 0.715585
- **t-alpha:** 0.621547
- **Chemical similarity (Tanimoto Index) (%):** N/A
- **1D identity (%) [PDB]:** 0.26
- **1D identity (%) [Gaps excluded][PDB]:** 60.0
- **1D identity - Alignment Gaps [PDB]:** 1133
- **2D identity (%) [PDB]:** 12.56
- **2D identity (%) [Gaps excluded][PDB]:** 84.46
- **2D identity - Alignment Gaps [PDB]:** 847
- **3D similarity (TM-Score) (%) [PDB]:** 8.28

- **Gene name:** E
- **RefSeq ID:** NC\_000866
- **Genomic sequence length:** 168903
- **5-UTR|CDS|3-UTR identity (%):** N/A | 9.19 | N/A
- **5-UTR|CDS|3-UTR identity (%) [Gaps excluded]:** N/A | 80.18 | N/A
- **5-UTR|CDS|3-UTR identity [Alignment Gaps]:** N/A | 3429 | N/A

**Uniprot Description:**  
  
Endolysin with lysozyme activity that degrades host peptidoglycans and participates with the holin and spanin proteins in the sequential events which lead to the programmed host cell lysis releasing the mature viral particles. Once the holin has permeabilized the host cell membrane, the endolysin can reach the periplasm and break down the peptidoglycan layer.  
  
**Gene Ontology Information:**

Molecular Function

- lysozyme activity

Location

- host cell cytoplasm

Biological process

- cell wall macromolecule catabolic process
- cytolysis
- defense response to bacterium
- peptidoglycan catabolic process
- viral release from host cell by cytolysis

---

53

- **Protein name:** Uncharacterized protein ORF140
- **Organism:** Acidianus filamentous virus 1 (isolate United States/Yellowstone)
- **Uniprot Accession Number:** Q70LC6
- **Protein sequence length:** 140 aa
- **1D identity (%):** 2.34
- **1D identity (%) [Gaps excluded]:** 34.07
- **1D identity - Alignment Gaps:** 1231
- **Common reported functions (%):** 0.0
- **Common reported locations (%):** 0.0
- **Common reported processes (%):** 0.0

- **PDB ID:** 5W7G
- **Chain:** Q
- **Crystallized protein length:** 131 aa
- **Resolution:** 4.5 Å
- **Alinged residues range:** 77-94, 86-91, 50-55
- **Aligned to segment part (indices):** 1, 3, 0
- **Alinged residues range of reference:** 769-778, 949-954, 1023-1028
- **b-phipsi:** 0.308069
- **w-rdist:** 0.844281
- **t-alpha:** 0.478589
- **Chemical similarity (Tanimoto Index) (%):** 81.92
- **1D identity (%) [PDB]:** 0.0
- **1D identity (%) [Gaps excluded][PDB]:** 0.0
- **1D identity - Alignment Gaps [PDB]:** 1114
- **2D identity (%) [PDB]:** 11.26
- **2D identity (%) [Gaps excluded][PDB]:** 94.12
- **2D identity - Alignment Gaps [PDB]:** 876
- **3D similarity (TM-Score) (%) [PDB]:** 6.64

- **Gene name:** ORF140
- **RefSeq ID:** NC\_005830
- **Genomic sequence length:** 20869
- **5-UTR|CDS|3-UTR identity (%):** N/A | 8.16 | N/A
- **5-UTR|CDS|3-UTR identity (%) [Gaps excluded]:** N/A | 81.82 | N/A
- **5-UTR|CDS|3-UTR identity [Alignment Gaps]:** N/A | 3475 | N/A

**Uniprot Description:**  
  
N/A  
  
**Gene Ontology Information:**

Molecular Function  
  
N/A

Location  
  
N/A

Biological process  
  
N/A

---

54

- **Protein name:** Gag-Pol polyprotein
- **Organism:** Human immunodeficiency virus type 1 group M subtype B (isolate HXB2)
- **Uniprot Accession Number:** P04585
- **Protein sequence length:** 1435 aa
- **1D identity (%):** 12.98
- **1D identity (%) [Gaps excluded]:** 28.67
- **1D identity - Alignment Gaps:** 1020
- **Common reported functions (%):** 50.0
- **Common reported locations (%):** 25.0
- **Common reported processes (%):** 10.0

- **PDB ID:** 4U7Q
- **Chain:** A
- **Crystallized protein length:** 99 aa
- **Resolution:** 1.7 Å
- **Alinged residues range:** 92-95, 90-93, 52-55
- **Aligned to segment part (indices):** 1, 3, 0
- **Alinged residues range of reference:** 780-783, 948-951, 1051-1054
- **b-phipsi:** 0.146486
- **w-rdist:** 0.843276
- **t-alpha:** 0.58221
- **Chemical similarity (Tanimoto Index) (%):** 82.66
- **1D identity (%) [PDB]:** 0.0
- **1D identity (%) [Gaps excluded][PDB]:** 0.0
- **1D identity - Alignment Gaps [PDB]:** 1082
- **2D identity (%) [PDB]:** 8.17
- **2D identity (%) [Gaps excluded][PDB]:** 90.0
- **2D identity - Alignment Gaps [PDB]:** 902
- **3D similarity (TM-Score) (%) [PDB]:** 4.78

- **Gene name:** gag-pol
- **RefSeq ID:** NC\_001802
- **Genomic sequence length:** 9181
- **5-UTR|CDS|3-UTR identity (%):** 23.6 | 40.46 | 23.63
- **5-UTR|CDS|3-UTR identity (%) [Gaps excluded]:** 75.9 | 77.79 | 80.65
- **5-UTR|CDS|3-UTR identity [Alignment Gaps]:** 184 | 2566 | 374

**Uniprot Description:**  
  
Gag-Pol polyprotein
Mediates, with Gag polyprotein, the essential events in virion assembly, including binding the plasma membrane, making the protein-protein interactions necessary to create spherical particles, recruiting the viral Env proteins, and packaging the genomic RNA via direct interactions with the RNA packaging sequence (Psi). Gag-Pol polyprotein may regulate its own translation, by the binding genomic RNA in the 5'-UTR. At low concentration, the polyprotein would promote translation, whereas at high concentration, the polyprotein would encapsidate genomic RNA and then shut off translation.  
  
Matrix protein p17
Homotrimer; further assembles as hexamers of trimers (PubMed:19327811). Interacts with gp41 (via C-terminus) (By similarity). Interacts with host CALM1; this interaction induces a conformational change in the Matrix protein, triggering exposure of the myristate group (PubMed:24500712). Interacts with host AP3D1; this interaction allows the polyprotein trafficking to multivesicular bodies during virus assembly (By similarity). Part of the pre-integration complex (PIC) which is composed of viral genome, matrix protein, Vpr and integrase (By similarity).  
  
**Gene Ontology Information:**

Molecular Function

- aspartic-type endopeptidase activity
- DNA binding
- DNA-directed DNA polymerase activity
- exoribonuclease H activity
- identical protein binding
- lipid binding
- RNA binding
- RNA-directed DNA polymerase activity
- RNA-DNA hybrid ribonuclease activity
- structural molecule activity
- zinc ion binding

Location

- host cell nucleus
- host cell plasma membrane
- host multivesicular body
- viral nucleocapsid
- virion membrane

Biological process

- DNA integration
- DNA recombination
- entry into host
- establishment of integrated proviral latency
- fusion of virus membrane with host plasma membrane
- induction by virus of host cysteine-type endopeptidase activity involved in apoptotic process
- RNA-dependent DNA biosynthetic process
- suppression by virus of host gene expression
- uncoating of virus
- viral genome integration into host DNA
- viral genome packaging
- viral life cycle
- viral penetration into host nucleus
- virion assembly

---

55

- **Protein name:** 60S ribosomal protein L41
- **Organism:** Homo sapiens
- **Uniprot Accession Number:** P62945
- **Protein sequence length:** 25 aa
- **1D identity (%):** 0.31
- **1D identity (%) [Gaps excluded]:** 16.0
- **1D identity - Alignment Gaps:** 1248
- **Common reported functions (%):** 0.0
- **Common reported locations (%):** 0.0
- **Common reported processes (%):** 0.0

- **PDB ID:** 6ZLW
- **Chain:** H
- **Crystallized protein length:** 186 aa
- **Resolution:** 2.6 Å
- **Alinged residues range:** 124-127, 125-127, 92-94
- **Aligned to segment part (indices):** 1, 3, 0
- **Alinged residues range of reference:** 771-774, 952-954, 1060-1062
- **b-phipsi:** 0.189063
- **w-rdist:** 0.764264
- **t-alpha:** 0.912052
- **Chemical similarity (Tanimoto Index) (%):** 71.31
- **1D identity (%) [PDB]:** 0.0
- **1D identity (%) [Gaps excluded][PDB]:** 0.0
- **1D identity - Alignment Gaps [PDB]:** 1169
- **2D identity (%) [PDB]:** 9.03
- **2D identity (%) [Gaps excluded][PDB]:** 90.57
- **2D identity - Alignment Gaps [PDB]:** 957
- **3D similarity (TM-Score) (%) [PDB]:** 9.07

- **Gene name:** RPL41
- **RefSeq ID:** NM\_021104
- **Transcript sequence length:** 561
- **5-UTR|CDS|3-UTR identity (%):** 10.57 | 1.44 | 27.57
- **5-UTR|CDS|3-UTR identity (%) [Gaps excluded]:** 70.0 | 76.39 | 72.04
- **5-UTR|CDS|3-UTR identity [Alignment Gaps]:** 225 | 3756 | 300

**Uniprot Description:**  
  
Interacts with the beta subunit of protein kinase CKII and stimulates phosphorylation of DNA topoisomerase II alpha by CKII.  
  
**Gene Ontology Information:**

Molecular Function

- mRNA 3'-UTR binding
- mRNA 5'-UTR binding
- RNA binding
- structural constituent of ribosome

Location

- cytosol
- cytosolic large ribosomal subunit
- endoplasmic reticulum
- polysomal ribosome

Biological process

- cytoplasmic translation
- nuclear-transcribed mRNA catabolic process, nonsense-mediated decay
- rRNA processing
- SRP-dependent cotranslational protein targeting to membrane
- translation
- translational initiation
- viral transcription

---

56

- **Protein name:** SAR-endolysin
- **Organism:** Escherichia phage P1
- **Uniprot Accession Number:** Q37875
- **Protein sequence length:** 185 aa
- **1D identity (%):** 3.23
- **1D identity (%) [Gaps excluded]:** 26.42
- **1D identity - Alignment Gaps:** 1140
- **Common reported functions (%):** 0.0
- **Common reported locations (%):** 25.0
- **Common reported processes (%):** 0.0

- **PDB ID:** 1XJU
- **Chain:** A
- **Crystallized protein length:** 156 aa
- **Resolution:** 1.07 Å
- **Alinged residues range:** 74-83, 106-108, 83-94, 103-107
- **Aligned to segment part (indices):** 1, 2, 3, 0
- **Alinged residues range of reference:** 774-780, 888-890, 949-954, 1023-1027
- **b-phipsi:** 0.166237
- **w-rdist:** 0.768207
- **t-alpha:** 0.950166
- **Chemical similarity (Tanimoto Index) (%):** 85.57
- **1D identity (%) [PDB]:** 0.0
- **1D identity (%) [Gaps excluded][PDB]:** 0.0
- **1D identity - Alignment Gaps [PDB]:** 1140
- **2D identity (%) [PDB]:** 9.3
- **2D identity (%) [Gaps excluded][PDB]:** 88.89
- **2D identity - Alignment Gaps [PDB]:** 924
- **3D similarity (TM-Score) (%) [PDB]:** 7.5

- **Gene name:** 17
- **RefSeq ID:** NC\_005856
- **Genomic sequence length:** 94800
- **5-UTR|CDS|3-UTR identity (%):** N/A | 8.91 | N/A
- **5-UTR|CDS|3-UTR identity (%) [Gaps excluded]:** N/A | 75.05 | N/A
- **5-UTR|CDS|3-UTR identity [Alignment Gaps]:** N/A | 3450 | N/A

**Uniprot Description:**  
  
Signal-arrest-release (SAR) endolysin with lysozyme activity that degrades host peptidoglycans and participates with the pinholin and spanin proteins in the sequential events which lead to programmed host cell lysis releasing the mature viral particles. Once the pinholin has permeabilized the host cell membrane, the SAR-endolysin is released into the periplasm where it breaks down the peptidoglycan layer.  
  
**Gene Ontology Information:**

Molecular Function

- lysozyme activity

Location

- host cell plasma membrane
- integral component of membrane

Biological process

- cell wall macromolecule catabolic process
- cytolysis
- defense response to bacterium
- peptidoglycan catabolic process

---

57

- **Protein name:** Genome polyprotein
- **Organism:** Poliovirus type 1 (strain Mahoney)
- **Uniprot Accession Number:** P03300
- **Protein sequence length:** 2209 aa
- **1D identity (%):** 10.76
- **1D identity (%) [Gaps excluded]:** 26.36
- **1D identity - Alignment Gaps:** 1464
- **Common reported functions (%):** 0.0
- **Common reported locations (%):** 0.0
- **Common reported processes (%):** 10.0

- **PDB ID:** 6PSZ
- **Chain:** 1
- **Crystallized protein length:** 193 aa
- **Resolution:** 3.2 Å
- **Alinged residues range:** 156-159, 143-159
- **Aligned to segment part (indices):** 4, 0
- **Alinged residues range of reference:** 725-728, 1044-1051
- **b-phipsi:** 0.192478
- **w-rdist:** 0.735549
- **t-alpha:** 1.795238
- **Chemical similarity (Tanimoto Index) (%):** 83.13
- **1D identity (%) [PDB]:** 0.17
- **1D identity (%) [Gaps excluded][PDB]:** 50.0
- **1D identity - Alignment Gaps [PDB]:** 1169
- **2D identity (%) [PDB]:** 13.15
- **2D identity (%) [Gaps excluded][PDB]:** 90.0
- **2D identity - Alignment Gaps [PDB]:** 877
- **3D similarity (TM-Score) (%) [PDB]:** 8.38

- **Gene name:** N/A
- **RefSeq ID:** NC\_002058
- **Genomic sequence length:** 7440
- **5-UTR|CDS|3-UTR identity (%):** 22.22 | 36.52 | N/A
- **5-UTR|CDS|3-UTR identity (%) [Gaps excluded]:** 77.68 | 79.95 | N/A
- **5-UTR|CDS|3-UTR identity [Alignment Gaps]:** 559 | 3898 | N/A

**Uniprot Description:**  
  
Capsid protein VP1
Forms an icosahedral capsid of pseudo T=3 symmetry with capsid proteins VP2 and VP3 (PubMed:2994218). The capsid is 300 Angstroms in diameter, composed of 60 copies of each capsid protein and enclosing the viral positive strand RNA genome (PubMed:2994218). Capsid protein VP1 mainly forms the vertices of the capsid (PubMed:23365424). Capsid protein VP1 interacts with host cell receptor PVR to provide virion attachment to target host epithelial cells (PubMed:25631086). This attachment induces virion internalization predominantly through clathrin- and caveolin-independent endocytosis in Hela cells and through caveolin-mediated endocytosis in brain microvascular endothelial cells (PubMed:17717529, PubMed:18191571, PubMed:17622193). Tyrosine kinases are probably involved in the entry process (PubMed:17717529). Virus binding to PVR induces increased junctional permeability and rearrangement of junctional proteins (PubMed:17717529). Modulation of endothelial tight junctions, as well as cytolytic infection of endothelial cells themselves, may result in loss of endothelial integrity which may help the virus to reach the CNS (PubMed:17717529). After binding to its receptor, the capsid undergoes conformational changes (PubMed:25631086). Capsid protein VP1 N-terminus (that contains an amphipathic alpha-helix) and capsid protein VP4 are externalized (PubMed:25631086). Together, they shape a pore in the host membrane through which viral genome is translocated to host cell cytoplasm (PubMed:25631086).  
  
Capsid protein VP0
Interacts with capsid protein VP1 and capsid protein VP3 to form heterotrimeric protomers.  
  
**Gene Ontology Information:**

Molecular Function

- ATP binding
- cysteine-type endopeptidase activity
- ion channel activity
- metal ion binding
- nucleoside-triphosphatase activity
- RNA binding
- RNA helicase activity
- RNA-directed 5'-3' RNA polymerase activity
- structural molecule activity

Location

- host cell cytoplasmic vesicle membrane
- host cell nucleus
- integral to membrane of host cell
- membrane
- T=pseudo3 icosahedral viral capsid
- viral capsid

Biological process

- endocytosis involved in viral entry into host cell
- induction by virus of host autophagy
- pore formation by virus in membrane of host cell
- pore-mediated entry of viral genome into host cell
- positive stranded viral RNA replication
- protein complex oligomerization
- RNA-protein covalent cross-linking
- suppression by virus of host MAVS activity
- suppression by virus of host MDA-5 activity
- suppression by virus of host mRNA export from nucleus
- suppression by virus of host RIG-I activity
- suppression by virus of host translation initiation factor activity
- transcription, DNA-templated
- viral RNA genome replication
- virion assembly
- virion attachment to host cell

---
