# Supplementary material for: Identifying and profiling structural similarities between Spike of SARS-CoV-2 and other viral or host proteins with Machaon
Source: Commun Biol. 2023 Jul 19;6:752. doi: 10.1038/s42003-023-05076-7 (PMC10356814; doi:10.1038/s42003-023-05076-7)
Supplement: Supplementary file 7 — Supplementary Data 4 [file 42003_2023_5076_MOESM7_ESM.zip › 6VXX_A_segment/candidates/6VXX_A_site3-metrics-merged-enriched_eval_report.html]

 

# Structural Comparison Report for 6VXX\_A\_site3 - segments (total: 22)

---

1

- **Protein name:** Replicase polyprotein 1ab
- **Organism:** Human coronavirus NL63
- **Uniprot Accession Number:** P0C6X5
- **Protein sequence length:** 6729 aa
- **1D identity (%):** 3.65
- **1D identity (%) [Gaps excluded]:** 28.52
- **1D identity - Alignment Gaps:** 6186
- **Common reported functions (%):** 0.0
- **Common reported locations (%):** 12.5
- **Common reported processes (%):** 10.0

- **PDB ID:** 6FV2
- **Chain:** A
- **Crystallized protein length:** 301 aa
- **Resolution:** 2.95 Å
- **Alinged residues range:** 120-126, 163-165, 190-193, 164-166
- **Aligned to segment part (indices):** 2, 1, 4, 3
- **Alinged residues range of reference:** 101-107, 195-197, 232-235, 354-356
- **b-phipsi:** 0.018367
- **w-rdist:** 0.899082
- **t-alpha:** 0.010417
- **Chemical similarity (Tanimoto Index) (%):** 84.72
- **1D identity (%) [PDB]:** 0.08
- **1D identity (%) [Gaps excluded][PDB]:** 100.0
- **1D identity - Alignment Gaps [PDB]:** 1282
- **2D identity (%) [PDB]:** 19.65
- **2D identity (%) [Gaps excluded][PDB]:** 82.93
- **2D identity - Alignment Gaps [PDB]:** 792
- **3D similarity (TM-Score) (%) [PDB]:** 11.97

- **Gene name:** rep
- **RefSeq ID:** NC\_005831
- **Genomic sequence length:** 27553
- **5-UTR|CDS|3-UTR identity (%):** 46.29 | 13.21 | 37.18
- **5-UTR|CDS|3-UTR identity (%) [Gaps excluded]:** 80.6 | 80.78 | 81.99
- **5-UTR|CDS|3-UTR identity [Alignment Gaps]:** 149 | 17260 | 194

**Uniprot Description:**  
  
The replicase polyprotein of coronaviruses is a multifunctional protein: it contains the activities necessary for the transcription of negative stranded RNA, leader RNA, subgenomic mRNAs and progeny virion RNA as well as proteinases responsible for the cleavage of the polyprotein into functional products.  
  
3CL-PRO exists as monomer and homodimer. Eight copies of nsp7 and eight copies of nsp8 assemble to form a heterohexadecamer. Nsp9 is a dimer. Nsp10 forms a dodecamer (By similarity).  
  
**Gene Ontology Information:**

Molecular Function

- ATP binding
- cysteine-type endopeptidase activity
- DNA helicase activity
- endonuclease activity
- exoribonuclease activity
- methyltransferase activity
- RNA binding
- RNA helicase activity
- RNA-directed 5'-3' RNA polymerase activity
- thiol-dependent ubiquitin-specific protease activity
- zinc ion binding

Location

- host cell endoplasmic reticulum-Golgi intermediate compartment
- host cell membrane
- host cell perinuclear region of cytoplasm
- integral component of membrane

Biological process

- induction by virus of host autophagy
- methylation
- modulation by virus of host protein ubiquitination
- suppression by virus of host IRF3 activity
- transcription, DNA-templated
- viral protein processing
- viral RNA genome replication

---

2

- **Protein name:** Spike glycoprotein
- **Organism:** Severe acute respiratory syndrome coronavirus
- **Uniprot Accession Number:** P59594
- **Protein sequence length:** 1255 aa
- **1D identity (%):** 76.35
- **1D identity (%) [Gaps excluded]:** 77.94
- **1D identity - Alignment Gaps:** 26
- **Common reported functions (%):** 100.0
- **Common reported locations (%):** 62.5
- **Common reported processes (%):** 90.0

- **PDB ID:** 5X58
- **Chain:** C
- **Crystallized protein length:** 1052 aa
- **Resolution:** 3.2 Å
- **Alinged residues range:** 83-125, 188-192, 222-229, 340-343, 450-455
- **Aligned to segment part (indices):** 2, 1, 4, 3, 0
- **Alinged residues range of reference:** 86-128, 195-199, 229-236, 353-356, 463-468
- **b-phipsi:** 0.011666
- **w-rdist:** 0.229189
- **t-alpha:** 0.059896
- **Chemical similarity (Tanimoto Index) (%):** 100.0
- **1D identity (%) [PDB]:** 67.43
- **1D identity (%) [Gaps excluded][PDB]:** 77.99
- **1D identity - Alignment Gaps [PDB]:** 148
- **2D identity (%) [PDB]:** 55.92
- **2D identity (%) [Gaps excluded][PDB]:** 82.73
- **2D identity - Alignment Gaps [PDB]:** 394
- **3D similarity (TM-Score) (%) [PDB]:** 94.55

- **Gene name:** S
- **RefSeq ID:** NC\_004718
- **Genomic sequence length:** 29751
- **5-UTR|CDS|3-UTR identity (%):** 88.52 | 73.15 | 22.38
- **5-UTR|CDS|3-UTR identity (%) [Gaps excluded]:** 92.28 | 78.79 | 98.18
- **5-UTR|CDS|3-UTR identity [Alignment Gaps]:** 11 | 282 | 745

**Uniprot Description:**  
  
Spike glycoprotein
May down-regulate host tetherin (BST2) by lysosomal degradation, thereby counteracting its antiviral activity.  
  
Homotrimer; each monomer consists of a S1 and a S2 subunit. The resulting peplomers protrude from the virus surface as spikes (By similarity). Binds to human and palm civet ACE2 and human CLEC4M/DC-SIGNR. Interacts with the accessory proteins 3a and 7a.  
  
**Gene Ontology Information:**

Molecular Function

- host cell surface receptor binding
- identical protein binding

Location

- host cell endoplasmic reticulum-Golgi intermediate compartment membrane
- host cell plasma membrane
- integral component of membrane
- viral envelope
- virion membrane

Biological process

- endocytosis involved in viral entry into host cell
- fusion of virus membrane with host endosome membrane
- fusion of virus membrane with host plasma membrane
- pathogenesis
- receptor-mediated virion attachment to host cell
- suppression by virus of host tetherin activity
- suppression by virus of host type I interferon-mediated signaling pathway
- viral protein processing
- viral translation

---

3

- **Protein name:** Replicase polyprotein 1a
- **Organism:** Human coronavirus NL63
- **Uniprot Accession Number:** P0C6U6
- **Protein sequence length:** 4060 aa
- **1D identity (%):** 7.08
- **1D identity (%) [Gaps excluded]:** 27.9
- **1D identity - Alignment Gaps:** 3175
- **Common reported functions (%):** 0.0
- **Common reported locations (%):** 12.5
- **Common reported processes (%):** 10.0

- **PDB ID:** 3TLO
- **Chain:** B
- **Crystallized protein length:** 302 aa
- **Resolution:** 1.6 Å
- **Alinged residues range:** 120-126, 163-165, 190-193, 164-166, 106-109
- **Aligned to segment part (indices):** 2, 1, 4, 3, 0
- **Alinged residues range of reference:** 101-107, 195-197, 232-235, 354-356, 462-465
- **b-phipsi:** 0.010706
- **w-rdist:** 0.981256
- **t-alpha:** 0.021277
- **Chemical similarity (Tanimoto Index) (%):** 83.51
- **1D identity (%) [PDB]:** 0.08
- **1D identity (%) [Gaps excluded][PDB]:** 100.0
- **1D identity - Alignment Gaps [PDB]:** 1283
- **2D identity (%) [PDB]:** 20.5
- **2D identity (%) [Gaps excluded][PDB]:** 86.59
- **2D identity - Alignment Gaps [PDB]:** 793
- **3D similarity (TM-Score) (%) [PDB]:** 12.37

- **Gene name:** 1a
- **RefSeq ID:** N/A
- **Sequence length:** N/A
- **5-UTR|CDS|3-UTR identity (%):** N/A | N/A | N/A
- **5-UTR|CDS|3-UTR identity (%) [Gaps excluded]:** N/A | N/A | N/A
- **5-UTR|CDS|3-UTR identity [Alignment Gaps]:** N/A | N/A | N/A

**Uniprot Description:**  
  
The papain-like proteinase 1 (PLP1) and papain-like proteinase 2 (PLP2) are responsible for the cleavages located at the N-terminus of the replicase polyprotein. In addition, PLP2 possesses a deubiquitinating/deISGylating activity and processes both 'Lys-48'- and 'Lys-63'-linked polyubiquitin chains from cellular substrates. PLP2 also antagonizes innate immune induction of type I interferon by blocking the nuclear translocation of host IRF-3.  
  
3CL-PRO exists as monomer and homodimer. Eight copies of nsp7 and eight copies of nsp8 assemble to form a heterohexadecamer. Nsp9 is a dimer. Nsp10 forms a dodecamer (By similarity).  
  
**Gene Ontology Information:**

Molecular Function

- cysteine-type endopeptidase activity
- RNA binding
- thiol-dependent ubiquitin-specific protease activity
- transferase activity
- zinc ion binding

Location

- host cell membrane
- host cell perinuclear region of cytoplasm
- integral component of membrane

Biological process

- induction by virus of host autophagy
- modulation by virus of host protein ubiquitination
- suppression by virus of host IRF3 activity
- viral genome replication
- viral protein processing

---

4

- **Protein name:** DNA-directed RNA polymerase subunit beta
- **Organism:** Thermus thermophilus (strain HB8 / ATCC 27634 / DSM 579)
- **Uniprot Accession Number:** Q8RQE9
- **Protein sequence length:** 1119 aa
- **1D identity (%):** 13.58
- **1D identity (%) [Gaps excluded]:** 26.58
- **1D identity - Alignment Gaps:** 774
- **Common reported functions (%):** 0.0
- **Common reported locations (%):** 0.0
- **Common reported processes (%):** 0.0

- **PDB ID:** 3WOF
- **Chain:** M
- **Crystallized protein length:** 128 aa
- **Resolution:** 3.3 Å
- **Alinged residues range:** 823-828, 748-751, 723-726, 704-707, 750-753
- **Aligned to segment part (indices):** 2, 1, 4, 3, 0
- **Alinged residues range of reference:** 101-106, 196-199, 232-235, 354-357, 462-465
- **b-phipsi:** 0.0138
- **w-rdist:** 1.153384
- **t-alpha:** 0.044271
- **Chemical similarity (Tanimoto Index) (%):** 67.8
- **1D identity (%) [PDB]:** 0.0
- **1D identity (%) [Gaps excluded][PDB]:** 0.0
- **1D identity - Alignment Gaps [PDB]:** 1111
- **2D identity (%) [PDB]:** 8.82
- **2D identity (%) [Gaps excluded][PDB]:** 77.88
- **2D identity - Alignment Gaps [PDB]:** 885
- **3D similarity (TM-Score) (%) [PDB]:** 6.37

- **Gene name:** rpoB
- **RefSeq ID:** N/A
- **Sequence length:** N/A
- **5-UTR|CDS|3-UTR identity (%):** N/A | N/A | N/A
- **5-UTR|CDS|3-UTR identity (%) [Gaps excluded]:** N/A | N/A | N/A
- **5-UTR|CDS|3-UTR identity [Alignment Gaps]:** N/A | N/A | N/A

**Uniprot Description:**  
  
DNA-dependent RNA polymerase catalyzes the transcription of DNA into RNA using the four ribonucleoside triphosphates as substrates.  
  
The RNAP catalytic core consists of 2 alpha, 1 beta, 1 beta' and 1 omega subunit. When a sigma factor is associated with the core the holoenzyme is formed, which can initiate transcription.  
  
**Gene Ontology Information:**

Molecular Function

- DNA binding
- DNA-directed 5'-3' RNA polymerase activity
- ribonucleoside binding

Location  
  
N/A

Biological process

- transcription, DNA-templated

---

5

- **Protein name:** Structural polyprotein
- **Organism:** Semliki forest virus
- **Uniprot Accession Number:** P03315
- **Protein sequence length:** 1253 aa
- **1D identity (%):** 8.19
- **1D identity (%) [Gaps excluded]:** 28.8
- **1D identity - Alignment Gaps:** 1408
- **Common reported functions (%):** 0.0
- **Common reported locations (%):** 50.0
- **Common reported processes (%):** 10.0

- **PDB ID:** 2V33
- **Chain:** A
- **Crystallized protein length:** 91 aa
- **Resolution:** 1.55 Å
- **Alinged residues range:** 302-305, 345-348, 347-349
- **Aligned to segment part (indices):** 2, 1, 0
- **Alinged residues range of reference:** 104-107, 196-199, 462-464
- **b-phipsi:** 0.067099
- **w-rdist:** 0.600988
- **t-alpha:** 0.010417
- **Chemical similarity (Tanimoto Index) (%):** 74.41
- **1D identity (%) [PDB]:** 0.0
- **1D identity (%) [Gaps excluded][PDB]:** 0.0
- **1D identity - Alignment Gaps [PDB]:** 1074
- **2D identity (%) [PDB]:** 6.7
- **2D identity (%) [Gaps excluded][PDB]:** 90.54
- **2D identity - Alignment Gaps [PDB]:** 926
- **3D similarity (TM-Score) (%) [PDB]:** 6.14

- **Gene name:** N/A
- **RefSeq ID:** NC\_003215
- **Genomic sequence length:** 11442
- **5-UTR|CDS|3-UTR identity (%):** N/A | 40.5 | N/A
- **5-UTR|CDS|3-UTR identity (%) [Gaps excluded]:** N/A | 76.59 | N/A
- **5-UTR|CDS|3-UTR identity [Alignment Gaps]:** N/A | 2338 | N/A

**Uniprot Description:**  
  
Capsid protein
Forms an icosahedral capsid with a T=4 symmetry composed of 240 copies of the capsid protein surrounded by a lipid membrane through which penetrate 80 spikes composed of trimers of E1-E2 heterodimers (By similarity). The capsid protein binds to the viral RNA genome at a site adjacent to a ribosome binding site for viral genome translation following genome release (By similarity). Possesses a protease activity that results in its autocatalytic cleavage from the nascent structural protein (PubMed:3553612, PubMed:9642067). Following its self-cleavage, the capsid protein transiently associates with ribosomes, and within several minutes the protein binds to viral RNA and rapidly assembles into icosahedric core particles (PubMed:516447). The resulting nucleocapsid eventually associates with the cytoplasmic domain of the spike glycoprotein E2 at the cell membrane, leading to budding and formation of mature virions (By similarity). In case of infection, new virions attach to target cells and after clathrin-mediated endocytosis their membrane fuses with the host endosomal membrane (PubMed:15954801). This leads to the release of the nucleocapsid into the cytoplasm, followed by an uncoating event necessary for the genomic RNA to become accessible (PubMed:1433506). The uncoating might be triggered by the interaction of capsid proteins with ribosomes (PubMed:1433506). Binding of ribosomes would release the genomic RNA since the same region is genomic RNA-binding and ribosome-binding (PubMed:1433506).  
  
Capsid protein
Homodimer (By similarity). Homomultimer (Probable). Interacts with host karyopherin KPNA4; this interaction allows the nuclear import of the viral capsid protein (By similarity). Interacts with spike glycoprotein E2 (By similarity).  
  
**Gene Ontology Information:**

Molecular Function

- RNA binding
- serine-type endopeptidase activity
- structural molecule activity

Location

- host cell endosome
- host cell nucleus
- host cell plasma membrane
- integral component of membrane
- T=4 icosahedral viral capsid
- viral envelope
- virion membrane

Biological process

- clathrin-dependent endocytosis of virus by host cell
- fusion of virus membrane with host endosome membrane
- virion assembly
- virion attachment to host cell

---

6

- **Protein name:** DNA polymerase processivity factor
- **Organism:** Human herpesvirus 1 (strain 17)
- **Uniprot Accession Number:** P10226
- **Protein sequence length:** 488 aa
- **1D identity (%):** 7.68
- **1D identity (%) [Gaps excluded]:** 29.08
- **1D identity - Alignment Gaps:** 1025
- **Common reported functions (%):** 0.0
- **Common reported locations (%):** 0.0
- **Common reported processes (%):** 0.0

- **PDB ID:** 1DML
- **Chain:** G
- **Crystallized protein length:** 275 aa
- **Resolution:** 2.7 Å
- **Alinged residues range:** 57-77, 75-77, 28-30
- **Aligned to segment part (indices):** 2, 1, 0
- **Alinged residues range of reference:** 116-127, 195-197, 489-491
- **b-phipsi:** 0.055802
- **w-rdist:** 1.424332
- **t-alpha:** 0.002604
- **Chemical similarity (Tanimoto Index) (%):** 83.69
- **1D identity (%) [PDB]:** 0.0
- **1D identity (%) [Gaps excluded][PDB]:** 0.0
- **1D identity - Alignment Gaps [PDB]:** 1259
- **2D identity (%) [PDB]:** 16.76
- **2D identity (%) [Gaps excluded][PDB]:** 90.36
- **2D identity - Alignment Gaps [PDB]:** 865
- **3D similarity (TM-Score) (%) [PDB]:** 9.96

- **Gene name:** UL42
- **RefSeq ID:** NC\_001806
- **Genomic sequence length:** 152222
- **5-UTR|CDS|3-UTR identity (%):** N/A | 21.7 | N/A
- **5-UTR|CDS|3-UTR identity (%) [Gaps excluded]:** N/A | 74.58 | N/A
- **5-UTR|CDS|3-UTR identity [Alignment Gaps]:** N/A | 2905 | N/A

**Uniprot Description:**  
  
Plays an essential role in viral DNA replication by acting as the polymerase accessory subunit. Associates with the viral polymerase to increase its processivity and forms high-affinity direct interactions with DNA. Facilitates the origin-binding protein UL9 loading onto DNA thus increasing its ability to assemble into a functional complex capable of unwinding duplex DNA.  
  
Interacts with the DNA polymerase catalytic subunit UL30. Interacts with the origin-binding protein.  
  
**Gene Ontology Information:**

Molecular Function

- DNA binding
- DNA polymerase processivity factor activity

Location

- DNA polymerase complex
- host cell nucleus

Biological process

- bidirectional double-stranded viral DNA replication
- DNA replication

---

7

- **Protein name:** DNA adenine methylase
- **Organism:** Enterobacteria phage T4
- **Uniprot Accession Number:** P04392
- **Protein sequence length:** 259 aa
- **1D identity (%):** 4.09
- **1D identity (%) [Gaps excluded]:** 25.47
- **1D identity - Alignment Gaps:** 1108
- **Common reported functions (%):** 0.0
- **Common reported locations (%):** 0.0
- **Common reported processes (%):** 16890300.0

- **PDB ID:** 1YFJ
- **Chain:** D
- **Crystallized protein length:** 254 aa
- **Resolution:** 2.69 Å
- **Alinged residues range:** 212-218, 214-217, 160-162, 231-233
- **Aligned to segment part (indices):** 2, 1, 4, 3
- **Alinged residues range of reference:** 119-125, 196-199, 233-235, 354-356
- **b-phipsi:** 0.023704
- **w-rdist:** 0.949094
- **t-alpha:** 0.106771
- **Chemical similarity (Tanimoto Index) (%):** N/A
- **1D identity (%) [PDB]:** 0.41
- **1D identity (%) [Gaps excluded][PDB]:** 71.43
- **1D identity - Alignment Gaps [PDB]:** 1224
- **2D identity (%) [PDB]:** 13.41
- **2D identity (%) [Gaps excluded][PDB]:** 92.36
- **2D identity - Alignment Gaps [PDB]:** 924
- **3D similarity (TM-Score) (%) [PDB]:** N/A

- **Gene name:** DAM
- **RefSeq ID:** N/A
- **Sequence length:** NC\_000866
- **5-UTR|CDS|3-UTR identity (%):** N/A | 14.88 | N/A
- **5-UTR|CDS|3-UTR identity (%) [Gaps excluded]:** N/A | 81.43 | N/A
- **5-UTR|CDS|3-UTR identity [Alignment Gaps]:** N/A | 3180 | N/A

**Uniprot Description:**  
  
Methyltransferase that methylates adenine residues in the dsDNA sequence GATC. May prevent degradation of viral DNA by the host restriction-modification antiviral defense system.  
  
Monomer.  
  
**Gene Ontology Information:**

Molecular Function

- DNA-methyltransferase activity
- nucleic acid binding
- site-specific DNA-methyltransferase (adenine-specific) activity

Location  
  
N/A

Biological process

- DNA replication
- methylation
- restriction-modification system evasion by virus

---

8

- **Protein name:** Major capsid protein VP1
- **Organism:** B-lymphotropic polyomavirus
- **Uniprot Accession Number:** P04010
- **Protein sequence length:** 368 aa
- **1D identity (%):** 6.55
- **1D identity (%) [Gaps excluded]:** 27.88
- **1D identity - Alignment Gaps:** 1017
- **Common reported functions (%):** 0.0
- **Common reported locations (%):** 0.0
- **Common reported processes (%):** 10.0

- **PDB ID:** 4MBZ
- **Chain:** B
- **Crystallized protein length:** 274 aa
- **Resolution:** 1.75 Å
- **Alinged residues range:** 293-297, 238-241, 292-294
- **Aligned to segment part (indices):** 2, 4, 3
- **Alinged residues range of reference:** 125-129, 230-233, 355-357
- **b-phipsi:** 0.035367
- **w-rdist:** 7.204435
- **t-alpha:** 0.005208
- **Chemical similarity (Tanimoto Index) (%):** 87.6
- **1D identity (%) [PDB]:** 0.08
- **1D identity (%) [Gaps excluded][PDB]:** 100.0
- **1D identity - Alignment Gaps [PDB]:** 1256
- **2D identity (%) [PDB]:** 17.03
- **2D identity (%) [Gaps excluded][PDB]:** 89.55
- **2D identity - Alignment Gaps [PDB]:** 856
- **3D similarity (TM-Score) (%) [PDB]:** 10.59

- **Gene name:** N/A
- **RefSeq ID:** NC\_004763
- **Genomic sequence length:** 5270
- **5-UTR|CDS|3-UTR identity (%):** N/A | 20.89 | N/A
- **5-UTR|CDS|3-UTR identity (%) [Gaps excluded]:** N/A | 80.67 | N/A
- **5-UTR|CDS|3-UTR identity [Alignment Gaps]:** N/A | 2901 | N/A

**Uniprot Description:**  
  
Forms an icosahedral capsid with a T=7 symmetry and a 40 nm diameter. The capsid is composed of 72 pentamers linked to each other by disulfide bonds and associated with VP2 or VP3 proteins. Interacts with a N-linked glycoprotein containing sialic acids on the cell surface to provide virion attachment to target cell. Once attached, the virion is internalized by endocytosis and traffics to the endoplasmic reticulum. Inside the endoplasmic reticulum, the protein folding machinery isomerizes VP1 interpentamer disulfide bonds, thereby triggering initial uncoating. Next, the virion uses the endoplasmic reticulum-associated degradation machinery to probably translocate in the cytosol before reaching the nucleus. Nuclear entry of the viral DNA involves the selective exposure and importin recognition of VP2/Vp3 nuclear localization signal. In late phase of infection, neo-synthesized VP1 encapsulates replicated genomic DNA in the nucleus, and participates in rearranging nucleosomes around the viral DNA.  
  
Homomultimer; disulfide-linked. The virus capsid is composed of 72 icosahedral units, each one composed of five disulfide-linked copies of VP1. Interacts with minor capsid proteins VP2 and VP3.  
  
**Gene Ontology Information:**

Molecular Function

- structural molecule activity

Location

- host cell nucleus
- T=7 icosahedral viral capsid

Biological process

- endocytosis involved in viral entry into host cell
- virion attachment to host cell

---

9

- **Protein name:** Nucleoprotein
- **Organism:** Severe acute respiratory syndrome coronavirus
- **Uniprot Accession Number:** P59595
- **Protein sequence length:** 422 aa
- **1D identity (%):** 6.48
- **1D identity (%) [Gaps excluded]:** 27.64
- **1D identity - Alignment Gaps:** 1051
- **Common reported functions (%):** 50.0
- **Common reported locations (%):** 0.0
- **Common reported processes (%):** 20.0

- **PDB ID:** 1SSK
- **Chain:** A
- **Crystallized protein length:** 158 aa
- **Resolution:** -1.0 Å
- **Alinged residues range:** 84-90, 51-53, 83-85, 121-124
- **Aligned to segment part (indices):** 2, 4, 3, 0
- **Alinged residues range of reference:** 89-95, 232-234, 353-355, 465-468
- **b-phipsi:** 0.026971
- **w-rdist:** 3.663681
- **t-alpha:** 0.029491
- **Chemical similarity (Tanimoto Index) (%):** 82.36
- **1D identity (%) [PDB]:** 0.0
- **1D identity (%) [Gaps excluded][PDB]:** 0.0
- **1D identity - Alignment Gaps [PDB]:** 1141
- **2D identity (%) [PDB]:** 10.99
- **2D identity (%) [Gaps excluded][PDB]:** 84.73
- **2D identity - Alignment Gaps [PDB]:** 879
- **3D similarity (TM-Score) (%) [PDB]:** 6.71

- **Gene name:** N
- **RefSeq ID:** NC\_004718
- **Genomic sequence length:** 29751
- **5-UTR|CDS|3-UTR identity (%):** 88.52 | 4.33 | 22.38
- **5-UTR|CDS|3-UTR identity (%) [Gaps excluded]:** 92.28 | 82.18 | 98.18
- **5-UTR|CDS|3-UTR identity [Alignment Gaps]:** 11 | 3631 | 745

**Uniprot Description:**  
  
Packages the positive strand viral genome RNA into a helical ribonucleocapsid (RNP) and plays a fundamental role during virion assembly through its interactions with the viral genome and membrane protein M. Plays an important role in enhancing the efficiency of subgenomic viral RNA transcription as well as viral replication (PubMed:17210170). May modulate transforming growth factor-beta signaling by binding host SMAD3 (PubMed:18055455).  
  
Homooligomer. Both monomeric and oligomeric forms interact with RNA. Interacts with protein M (PubMed:15351485). Interacts with protein E (PubMed:24766657). May bind to host HNRNPA1 (Probable). Interacts with NSP3; this interaction serves to tether the genome to the newly translated replicase-transcriptase complex at a very early stage of infection (By similarity). May interact with host SMAD3 (Probable). Interacts with host PPIA/CYPA (PubMed:15688292).  
  
**Gene Ontology Information:**

Molecular Function

- identical protein binding
- RNA binding

Location

- host cell endoplasmic reticulum-Golgi intermediate compartment
- host cell Golgi apparatus
- host cell perinuclear region of cytoplasm
- viral capsid
- viral nucleocapsid

Biological process

- viral protein processing
- viral translation

---

10

- **Protein name:** Dyslexia-associated protein KIAA0319-like protein
- **Organism:** Homo sapiens
- **Uniprot Accession Number:** Q8IZA0
- **Protein sequence length:** 1049 aa
- **1D identity (%):** 15.94
- **1D identity (%) [Gaps excluded]:** 30.45
- **1D identity - Alignment Gaps:** 726
- **Common reported functions (%):** 0.0
- **Common reported locations (%):** 12.5
- **Common reported processes (%):** 0.0

- **PDB ID:** 6JCQ
- **Chain:** R
- **Crystallized protein length:** 91 aa
- **Resolution:** 3.3 Å
- **Alinged residues range:** 477-481, 417-419, 455-457, 446-448, 453-456
- **Aligned to segment part (indices):** 2, 1, 4, 3, 0
- **Alinged residues range of reference:** 117-121, 195-197, 234-236, 354-356, 465-468
- **b-phipsi:** 0.129343
- **w-rdist:** 2.356343
- **t-alpha:** 0.005236
- **Chemical similarity (Tanimoto Index) (%):** 79.68
- **1D identity (%) [PDB]:** 0.0
- **1D identity (%) [Gaps excluded][PDB]:** 0.0
- **1D identity - Alignment Gaps [PDB]:** 1074
- **2D identity (%) [PDB]:** 6.95
- **2D identity (%) [Gaps excluded][PDB]:** 85.19
- **2D identity - Alignment Gaps [PDB]:** 912
- **3D similarity (TM-Score) (%) [PDB]:** 5.55

- **Gene name:** KIAA0319L
- **RefSeq ID:** N/A
- **Sequence length:** N/A
- **5-UTR|CDS|3-UTR identity (%):** N/A | N/A | N/A
- **5-UTR|CDS|3-UTR identity (%) [Gaps excluded]:** N/A | N/A | N/A
- **5-UTR|CDS|3-UTR identity [Alignment Gaps]:** N/A | N/A | N/A

**Uniprot Description:**  
  
Possible role in axon guidance through interaction with RTN4R.  
  
Interacts with RTN4R.  
  
**Gene Ontology Information:**

Molecular Function  
  
N/A

Location

- cytoplasmic vesicle
- Golgi apparatus
- Golgi membrane
- integral component of membrane
- nucleolus
- plasma membrane

Biological process

- viral process

---

11

- **Protein name:** Attachment protein G3P
- **Organism:** Enterobacteria phage fd
- **Uniprot Accession Number:** P03661
- **Protein sequence length:** 424 aa
- **1D identity (%):** 6.39
- **1D identity (%) [Gaps excluded]:** 27.59
- **1D identity - Alignment Gaps:** 1059
- **Common reported functions (%):** 0.0
- **Common reported locations (%):** 12.5
- **Common reported processes (%):** 10.0

- **PDB ID:** 3DGS
- **Chain:** B
- **Crystallized protein length:** 186 aa
- **Resolution:** 1.9 Å
- **Alinged residues range:** 95-99, 143-146, 100-104, 142-144
- **Aligned to segment part (indices):** 2, 1, 4, 3
- **Alinged residues range of reference:** 103-107, 196-199, 230-234, 355-357
- **b-phipsi:** 0.035645
- **w-rdist:** 3.077439
- **t-alpha:** 0.037838
- **Chemical similarity (Tanimoto Index) (%):** 82.3
- **1D identity (%) [PDB]:** 0.09
- **1D identity (%) [Gaps excluded][PDB]:** 100.0
- **1D identity - Alignment Gaps [PDB]:** 1168
- **2D identity (%) [PDB]:** 12.08
- **2D identity (%) [Gaps excluded][PDB]:** 92.59
- **2D identity - Alignment Gaps [PDB]:** 900
- **3D similarity (TM-Score) (%) [PDB]:** 7.34

- **Gene name:** III
- **RefSeq ID:** NC\_025824
- **Genomic sequence length:** 6408
- **5-UTR|CDS|3-UTR identity (%):** N/A | 22.2 | N/A
- **5-UTR|CDS|3-UTR identity (%) [Gaps excluded]:** N/A | 80.11 | N/A
- **5-UTR|CDS|3-UTR identity [Alignment Gaps]:** N/A | 2885 | N/A

**Uniprot Description:**  
  
Plays essential roles both in the penetration of the viral genome into the bacterial host via pilus retraction and in the extrusion process. During the initial step of infection, G3P mediates adsorption of the phage to its primary receptor, the tip of host F-pilus. Subsequent interaction with the host entry receptor tolA induces penetration of the viral DNA into the host cytoplasm. In the extrusion process, G3P mediates the release of the membrane-anchored virion from the cell via its C-terminal domain.  
  
Interacts with G6P; this interaction is required for proper integration of G3P and G6P into the virion. Interacts with G8P (By similarity). Interacts with host tolA.  
  
**Gene Ontology Information:**

Molecular Function  
  
N/A

Location

- host cell membrane
- integral component of membrane
- viral capsid

Biological process

- adhesion receptor-mediated virion attachment to host cell
- entry receptor-mediated virion attachment to host cell
- pathogenesis
- viral entry into host cell via pilus basal pore
- viral extrusion
- virion attachment to host cell pilus

---

12

- **Protein name:** Aminopeptidase N
- **Organism:** Homo sapiens
- **Uniprot Accession Number:** P15144
- **Protein sequence length:** 967 aa
- **1D identity (%):** 11.89
- **1D identity (%) [Gaps excluded]:** 26.59
- **1D identity - Alignment Gaps:** 856
- **Common reported functions (%):** 0.0
- **Common reported locations (%):** 25.0
- **Common reported processes (%):** 0.0

- **PDB ID:** 6U7G
- **Chain:** B
- **Crystallized protein length:** 899 aa
- **Resolution:** 2.35 Å
- **Alinged residues range:** 172-181, 154-158, 573-575, 174-177, 689-691
- **Aligned to segment part (indices):** 2, 1, 4, 3, 0
- **Alinged residues range of reference:** 87-96, 195-199, 234-236, 353-356, 465-467
- **b-phipsi:** 0.045457
- **w-rdist:** 0.496154
- **t-alpha:** 0.246753
- **Chemical similarity (Tanimoto Index) (%):** 88.61
- **1D identity (%) [PDB]:** 0.05
- **1D identity (%) [Gaps excluded][PDB]:** 50.0
- **1D identity - Alignment Gaps [PDB]:** 1879
- **2D identity (%) [PDB]:** 35.43
- **2D identity (%) [Gaps excluded][PDB]:** 89.51
- **2D identity - Alignment Gaps [PDB]:** 815
- **3D similarity (TM-Score) (%) [PDB]:** 21.43

- **Gene name:** ANPEP
- **RefSeq ID:** N/A
- **Sequence length:** N/A
- **5-UTR|CDS|3-UTR identity (%):** N/A | N/A | N/A
- **5-UTR|CDS|3-UTR identity (%) [Gaps excluded]:** N/A | N/A | N/A
- **5-UTR|CDS|3-UTR identity [Alignment Gaps]:** N/A | N/A | N/A

**Uniprot Description:**  
  
Broad specificity aminopeptidase which plays a role in the final digestion of peptides generated from hydrolysis of proteins by gastric and pancreatic proteases. Also involved in the processing of various peptides including peptide hormones, such as angiotensin III and IV, neuropeptides, and chemokines. May also be involved the cleavage of peptides bound to major histocompatibility complex class II molecules of antigen presenting cells. May have a role in angiogenesis and promote cholesterol crystallization. May have a role in amino acid transport by acting as binding partner of amino acid transporter SLC6A19 and regulating its activity (By similarity).  
  
Homodimer. Interacts with SLC6A19 (By similarity).  
  
**Gene Ontology Information:**

Molecular Function

- aminopeptidase activity
- metalloaminopeptidase activity
- metallopeptidase activity
- peptide binding
- signaling receptor activity
- virus receptor activity
- zinc ion binding

Location

- cytoplasm
- endoplasmic reticulum-Golgi intermediate compartment
- extracellular exosome
- extracellular space
- integral component of membrane
- lysosomal membrane
- plasma membrane
- secretory granule membrane

Biological process

- angiogenesis
- cell differentiation
- neutrophil degranulation
- peptide catabolic process
- proteolysis
- regulation of blood pressure
- signal transduction

---

13

- **Protein name:** Genome polyprotein
- **Organism:** Zika virus
- **Uniprot Accession Number:** Q32ZE1
- **Protein sequence length:** 3419 aa
- **1D identity (%):** 4.15
- **1D identity (%) [Gaps excluded]:** 24.96
- **1D identity - Alignment Gaps:** 3354
- **Common reported functions (%):** 50.0
- **Common reported locations (%):** 37.5
- **Common reported processes (%):** 20.0

- **PDB ID:** 5YOD
- **Chain:** B
- **Crystallized protein length:** 152 aa
- **Resolution:** 1.9 Å
- **Alinged residues range:** 23-27, 107-109, 98-101, 108-110
- **Aligned to segment part (indices):** 2, 1, 4, 3
- **Alinged residues range of reference:** 103-107, 195-197, 229-232, 354-356
- **b-phipsi:** 0.004448
- **w-rdist:** 2.419129
- **t-alpha:** 0.200521
- **Chemical similarity (Tanimoto Index) (%):** 85.16
- **1D identity (%) [PDB]:** 0.0
- **1D identity (%) [Gaps excluded][PDB]:** 0.0
- **1D identity - Alignment Gaps [PDB]:** 1136
- **2D identity (%) [PDB]:** 12.88
- **2D identity (%) [Gaps excluded][PDB]:** 90.14
- **2D identity - Alignment Gaps [PDB]:** 852
- **3D similarity (TM-Score) (%) [PDB]:** 6.45

- **Gene name:** N/A
- **RefSeq ID:** NC\_012532
- **Genomic sequence length:** 10794
- **5-UTR|CDS|3-UTR identity (%):** 21.75 | 23.21 | 32.03
- **5-UTR|CDS|3-UTR identity (%) [Gaps excluded]:** 72.09 | 78.24 | 74.24
- **5-UTR|CDS|3-UTR identity [Alignment Gaps]:** 199 | 7638 | 261

**Uniprot Description:**  
  
Capsid protein C
Plays a role in virus budding by binding to the host cell membrane and packages the viral RNA into a nucleocapsid that forms the core of the mature virus particle. During virus entry, may induce genome penetration into the host cytoplasm after hemifusion induced by the surface proteins. Can migrate to the cell nucleus where it modulates host functions.  
  
Capsid protein C
Homodimer.  
  
**Gene Ontology Information:**

Molecular Function

- ATP binding
- double-stranded RNA binding
- GTP binding
- identical protein binding
- metal ion binding
- mRNA (guanine-N7-)-methyltransferase activity
- mRNA (nucleoside-2'-O-)-methyltransferase activity
- nucleoside-triphosphatase activity
- protein dimerization activity
- RNA helicase activity
- RNA-directed 5'-3' RNA polymerase activity
- serine-type endopeptidase activity
- structural molecule activity

Location

- extracellular region
- host cell endoplasmic reticulum membrane
- host cell nucleus
- host cell perinuclear region of cytoplasm
- integral component of membrane
- viral capsid
- viral envelope
- virion membrane

Biological process

- clathrin-dependent endocytosis of virus by host cell
- fusion of virus membrane with host endosome membrane
- induction by virus of host autophagy
- suppression by virus of host STAT1 activity
- suppression by virus of host STAT2 activity
- suppression by virus of host TBK1 activity
- suppression by virus of host toll-like receptor signaling pathway
- suppression by virus of host TYK2 activity
- suppression by virus of host type I interferon-mediated signaling pathway
- viral RNA genome replication
- virion attachment to host cell

---

14

- **Protein name:** Secreted protein BARF1
- **Organism:** Epstein-Barr virus (strain B95-8)
- **Uniprot Accession Number:** P03228
- **Protein sequence length:** 221 aa
- **1D identity (%):** 2.79
- **1D identity (%) [Gaps excluded]:** 29.01
- **1D identity - Alignment Gaps:** 1232
- **Common reported functions (%):** 50.0
- **Common reported locations (%):** 0.0
- **Common reported processes (%):** 0.0

- **PDB ID:** 4ADF
- **Chain:** Q
- **Crystallized protein length:** 185 aa
- **Resolution:** 4.4 Å
- **Alinged residues range:** 198-205, 204-207, 130-133
- **Aligned to segment part (indices):** 2, 1, 3
- **Alinged residues range of reference:** 89-96, 195-198, 354-357
- **b-phipsi:** 0.054478
- **w-rdist:** 2.133273
- **t-alpha:** 0.065104
- **Chemical similarity (Tanimoto Index) (%):** 94.14
- **1D identity (%) [PDB]:** 0.0
- **1D identity (%) [Gaps excluded][PDB]:** 0.0
- **1D identity - Alignment Gaps [PDB]:** 1169
- **2D identity (%) [PDB]:** 13.75
- **2D identity (%) [Gaps excluded][PDB]:** 92.72
- **2D identity - Alignment Gaps [PDB]:** 867
- **3D similarity (TM-Score) (%) [PDB]:** 10.25

- **Gene name:** BARF1
- **RefSeq ID:** NC\_007605
- **Genomic sequence length:** 171823
- **5-UTR|CDS|3-UTR identity (%):** N/A | 10.16 | N/A
- **5-UTR|CDS|3-UTR identity (%) [Gaps excluded]:** N/A | 79.06 | N/A
- **5-UTR|CDS|3-UTR identity [Alignment Gaps]:** N/A | 3466 | N/A

**Uniprot Description:**  
  
Plays diverse functions in immunomodulation and oncogenicity, maybe by acting as a functional receptor for human CSF1. May inhibit interferon secretion from mononuclear cells. Exhibits oncogenic activity in vitro.  
  
Homohexamer. Interacts with human CSF1.  
  
**Gene Ontology Information:**

Molecular Function

- identical protein binding

Location

- extracellular region

Biological process  
  
N/A

---

15

- **Protein name:** T-cell surface glycoprotein CD4
- **Organism:** Homo sapiens
- **Uniprot Accession Number:** P01730
- **Protein sequence length:** 458 aa
- **1D identity (%):** 6.23
- **1D identity (%) [Gaps excluded]:** 27.59
- **1D identity - Alignment Gaps:** 1093
- **Common reported functions (%):** 50.0
- **Common reported locations (%):** 12.5
- **Common reported processes (%):** 10.0

- **PDB ID:** 6CM3
- **Chain:** I
- **Crystallized protein length:** 97 aa
- **Resolution:** 3.54 Å
- **Alinged residues range:** 2-4, 91-93, 23-25, 92-94, 30-33
- **Aligned to segment part (indices):** 2, 1, 4, 3, 0
- **Alinged residues range of reference:** 125-127, 195-197, 232-234, 354-356, 462-465
- **b-phipsi:** 0.051488
- **w-rdist:** 0.863527
- **t-alpha:** 0.458333
- **Chemical similarity (Tanimoto Index) (%):** N/A
- **1D identity (%) [PDB]:** 0.0
- **1D identity (%) [Gaps excluded][PDB]:** 0.0
- **1D identity - Alignment Gaps [PDB]:** 1080
- **2D identity (%) [PDB]:** 6.26
- **2D identity (%) [Gaps excluded][PDB]:** 85.14
- **2D identity - Alignment Gaps [PDB]:** 932
- **3D similarity (TM-Score) (%) [PDB]:** 6.98

- **Gene name:** CD4
- **RefSeq ID:** NM\_000616
- **Transcript sequence length:** 3049
- **5-UTR|CDS|3-UTR identity (%):** 36.71 | 23.32 | 8.36
- **5-UTR|CDS|3-UTR identity (%) [Gaps excluded]:** 76.82 | 77.54 | 76.65
- **5-UTR|CDS|3-UTR identity [Alignment Gaps]:** 165 | 2795 | 1365

**Uniprot Description:**  
  
Integral membrane glycoprotein that plays an essential role in the immune response and serves multiple functions in responses against both external and internal offenses. In T-cells, functions primarily as a coreceptor for MHC class II molecule:peptide complex. The antigens presented by class II peptides are derived from extracellular proteins while class I peptides are derived from cytosolic proteins. Interacts simultaneously with the T-cell receptor (TCR) and the MHC class II presented by antigen presenting cells (APCs). In turn, recruits the Src kinase LCK to the vicinity of the TCR-CD3 complex. LCK then initiates different intracellular signaling pathways by phosphorylating various substrates ultimately leading to lymphokine production, motility, adhesion and activation of T-helper cells. In other cells such as macrophages or NK cells, plays a role in differentiation/activation, cytokine expression and cell migration in a TCR/LCK-independent pathway. Participates in the development of T-helper cells in the thymus and triggers the differentiation of monocytes into functional mature macrophages.  
  
Forms disulfide-linked homodimers at the cell surface. Interacts with LCK (PubMed:16888650). Interacts with PTK2/FAK1 (PubMed:18078954). Binds to P4HB/PDI. Interacts with IL16; this interaction induces a CD4-dependent signaling in lymphocytes (PubMed:1673145). Interacts (via Ig-like V-type domain) with MHCII alpha chain (via alpha-2 domain) and beta chain (via beta-2 domain); this interaction increases the affinity of TCR for peptide-MHCII. CD4 oligomerization via Ig-like C2-type 2 and 3 domains appears to be required for stable binding to MHCII and adhesion between T cells and APCs (PubMed:27114505, PubMed:21900604, PubMed:7604010).  
  
**Gene Ontology Information:**

Molecular Function

- coreceptor activity
- enzyme binding
- extracellular matrix structural constituent
- identical protein binding
- immunoglobulin binding
- interleukin-16 binding
- interleukin-16 receptor activity
- MHC class II protein binding
- MHC class II protein complex binding
- protein homodimerization activity
- protein kinase binding
- protein tyrosine kinase binding
- signaling receptor activity
- transmembrane signaling receptor activity
- virus receptor activity
- zinc ion binding

Location

- clathrin-coated endocytic vesicle membrane
- early endosome
- endoplasmic reticulum lumen
- endoplasmic reticulum membrane
- external side of plasma membrane
- integral component of plasma membrane
- membrane raft
- plasma membrane
- T cell receptor complex

Biological process

- adaptive immune response
- cell adhesion
- cell surface receptor signaling pathway
- cellular response to granulocyte macrophage colony-stimulating factor stimulus
- cytokine-mediated signaling pathway
- defense response to Gram-negative bacterium
- entry into host
- enzyme linked receptor protein signaling pathway
- fusion of virus membrane with host plasma membrane
- helper T cell enhancement of adaptive immune response
- immune response
- induction by virus of host cell-cell fusion
- interleukin-15-mediated signaling pathway
- macrophage differentiation
- maintenance of protein location in cell
- membrane organization
- positive regulation of calcium ion transport into cytosol
- positive regulation of calcium-mediated signaling
- positive regulation of ERK1 and ERK2 cascade
- positive regulation of I-kappaB kinase/NF-kappaB signaling
- positive regulation of interleukin-2 production
- positive regulation of kinase activity
- positive regulation of MAPK cascade
- positive regulation of monocyte differentiation
- positive regulation of peptidyl-tyrosine phosphorylation
- positive regulation of protein kinase activity
- positive regulation of protein phosphorylation
- positive regulation of T cell proliferation
- positive regulation of transcription, DNA-templated
- positive regulation of viral entry into host cell
- regulation of calcium ion transport
- regulation of defense response to virus by virus
- regulation of T cell activation
- response to estradiol
- response to vitamin D
- signal transduction
- T cell activation
- T cell differentiation
- T cell receptor signaling pathway
- T cell selection
- transmembrane receptor protein tyrosine kinase signaling pathway

---

16

- **Protein name:** Gag-Pol polyprotein
- **Organism:** Human immunodeficiency virus type 1 group M subtype B (isolate HXB2)
- **Uniprot Accession Number:** P04585
- **Protein sequence length:** 1435 aa
- **1D identity (%):** 12.98
- **1D identity (%) [Gaps excluded]:** 28.67
- **1D identity - Alignment Gaps:** 1020
- **Common reported functions (%):** 50.0
- **Common reported locations (%):** 25.0
- **Common reported processes (%):** 10.0

- **PDB ID:** 6OR7
- **Chain:** A
- **Crystallized protein length:** 545 aa
- **Resolution:** 2.53 Å
- **Alinged residues range:** 329-333, 331-335, 274-276
- **Aligned to segment part (indices):** 2, 1, 4
- **Alinged residues range of reference:** 120-124, 195-199, 233-235
- **b-phipsi:** 0.158455
- **w-rdist:** 6.265318
- **t-alpha:** 0.007874
- **Chemical similarity (Tanimoto Index) (%):** 79.05
- **1D identity (%) [PDB]:** 0.0
- **1D identity (%) [Gaps excluded][PDB]:** 0.0
- **1D identity - Alignment Gaps [PDB]:** 1529
- **2D identity (%) [PDB]:** 20.96
- **2D identity (%) [Gaps excluded][PDB]:** 93.91
- **2D identity - Alignment Gaps [PDB]:** 971
- **3D similarity (TM-Score) (%) [PDB]:** 16.93

- **Gene name:** gag-pol
- **RefSeq ID:** NC\_001802
- **Genomic sequence length:** 9181
- **5-UTR|CDS|3-UTR identity (%):** 23.6 | 40.46 | 23.63
- **5-UTR|CDS|3-UTR identity (%) [Gaps excluded]:** 75.9 | 77.79 | 80.65
- **5-UTR|CDS|3-UTR identity [Alignment Gaps]:** 184 | 2566 | 374

**Uniprot Description:**  
  
Gag-Pol polyprotein
Mediates, with Gag polyprotein, the essential events in virion assembly, including binding the plasma membrane, making the protein-protein interactions necessary to create spherical particles, recruiting the viral Env proteins, and packaging the genomic RNA via direct interactions with the RNA packaging sequence (Psi). Gag-Pol polyprotein may regulate its own translation, by the binding genomic RNA in the 5'-UTR. At low concentration, the polyprotein would promote translation, whereas at high concentration, the polyprotein would encapsidate genomic RNA and then shut off translation.  
  
Matrix protein p17
Homotrimer; further assembles as hexamers of trimers (PubMed:19327811). Interacts with gp41 (via C-terminus) (By similarity). Interacts with host CALM1; this interaction induces a conformational change in the Matrix protein, triggering exposure of the myristate group (PubMed:24500712). Interacts with host AP3D1; this interaction allows the polyprotein trafficking to multivesicular bodies during virus assembly (By similarity). Part of the pre-integration complex (PIC) which is composed of viral genome, matrix protein, Vpr and integrase (By similarity).  
  
**Gene Ontology Information:**

Molecular Function

- aspartic-type endopeptidase activity
- DNA binding
- DNA-directed DNA polymerase activity
- exoribonuclease H activity
- identical protein binding
- lipid binding
- RNA binding
- RNA-directed DNA polymerase activity
- RNA-DNA hybrid ribonuclease activity
- structural molecule activity
- zinc ion binding

Location

- host cell nucleus
- host cell plasma membrane
- host multivesicular body
- viral nucleocapsid
- virion membrane

Biological process

- DNA integration
- DNA recombination
- entry into host
- establishment of integrated proviral latency
- fusion of virus membrane with host plasma membrane
- induction by virus of host cysteine-type endopeptidase activity involved in apoptotic process
- RNA-dependent DNA biosynthetic process
- suppression by virus of host gene expression
- uncoating of virus
- viral genome integration into host DNA
- viral genome packaging
- viral life cycle
- viral penetration into host nucleus
- virion assembly

---

17

- **Protein name:** Membrane-associated protein VP24
- **Organism:** Lake Victoria marburgvirus (strain Musoke-80)
- **Uniprot Accession Number:** P35256
- **Protein sequence length:** 253 aa
- **1D identity (%):** 4.15
- **1D identity (%) [Gaps excluded]:** 27.5
- **1D identity - Alignment Gaps:** 1126
- **Common reported functions (%):** 0.0
- **Common reported locations (%):** 25.0
- **Common reported processes (%):** 0.0

- **PDB ID:** 4OR8
- **Chain:** A
- **Crystallized protein length:** 225 aa
- **Resolution:** 2.65 Å
- **Alinged residues range:** 187-190, 186-188, 134-137, 180-182
- **Aligned to segment part (indices):** 2, 1, 4, 3
- **Alinged residues range of reference:** 125-128, 195-197, 233-236, 354-356
- **b-phipsi:** 0.133626
- **w-rdist:** 6.407594
- **t-alpha:** 0.015873
- **Chemical similarity (Tanimoto Index) (%):** 81.85
- **1D identity (%) [PDB]:** 0.0
- **1D identity (%) [Gaps excluded][PDB]:** 0.0
- **1D identity - Alignment Gaps [PDB]:** 1207
- **2D identity (%) [PDB]:** 17.29
- **2D identity (%) [Gaps excluded][PDB]:** 89.74
- **2D identity - Alignment Gaps [PDB]:** 817
- **3D similarity (TM-Score) (%) [PDB]:** 9.99

- **Gene name:** VP24
- **RefSeq ID:** NC\_001608
- **Genomic sequence length:** 19111
- **5-UTR|CDS|3-UTR identity (%):** N/A | 14.0 | N/A
- **5-UTR|CDS|3-UTR identity (%) [Gaps excluded]:** N/A | 80.92 | N/A
- **5-UTR|CDS|3-UTR identity [Alignment Gaps]:** N/A | 3232 | N/A

**Uniprot Description:**  
  
May act as a minor matrix protein that plays a role in assembly of viral nucleocapsid and virion budding (PubMed:16227263). Unlike Ebola VP24, mVP24 has no measurable impact of host dendritic cell function (PubMed:26962215).  
  
Monomer or homotetramer (Potential). Interacts with nucleoprotein.  
  
**Gene Ontology Information:**

Molecular Function

- structural constituent of virion

Location

- host cell endomembrane system
- host cell plasma membrane
- viral nucleocapsid
- virion membrane

Biological process

- viral process

---

18

- **Protein name:** Desmoglein-2
- **Organism:** Homo sapiens
- **Uniprot Accession Number:** Q14126
- **Protein sequence length:** 1118 aa
- **1D identity (%):** 14.46
- **1D identity (%) [Gaps excluded]:** 26.95
- **1D identity - Alignment Gaps:** 721
- **Common reported functions (%):** 0.0
- **Common reported locations (%):** 12.5
- **Common reported processes (%):** 0.0

- **PDB ID:** 6QNU
- **Chain:** E
- **Crystallized protein length:** 187 aa
- **Resolution:** 3.8 Å
- **Alinged residues range:** 117-119, 277-280, 239-242, 238-241
- **Aligned to segment part (indices):** 2, 1, 4, 0
- **Alinged residues range of reference:** 103-105, 196-199, 233-236, 465-468
- **b-phipsi:** 0.050926
- **w-rdist:** 8.4404
- **t-alpha:** 0.070312
- **Chemical similarity (Tanimoto Index) (%):** 82.24
- **1D identity (%) [PDB]:** 0.0
- **1D identity (%) [Gaps excluded][PDB]:** 0.0
- **1D identity - Alignment Gaps [PDB]:** 1175
- **2D identity (%) [PDB]:** 15.11
- **2D identity (%) [Gaps excluded][PDB]:** 82.42
- **2D identity - Alignment Gaps [PDB]:** 811
- **3D similarity (TM-Score) (%) [PDB]:** 8.52

- **Gene name:** DSG2
- **RefSeq ID:** NM\_001943
- **Transcript sequence length:** 5697
- **5-UTR|CDS|3-UTR identity (%):** 15.09 | 42.54 | 7.25
- **5-UTR|CDS|3-UTR identity (%) [Gaps excluded]:** 78.18 | 77.22 | 81.77
- **5-UTR|CDS|3-UTR identity [Alignment Gaps]:** 230 | 2079 | 2088

**Uniprot Description:**  
  
Component of intercellular desmosome junctions. Involved in the interaction of plaque proteins and intermediate filaments mediating cell-cell adhesion.  
  
**Gene Ontology Information:**

Molecular Function

- calcium ion binding
- cell adhesion molecule binding
- cell adhesive protein binding involved in bundle of His cell-Purkinje myocyte communication

Location

- apical plasma membrane
- cell junction
- cell surface
- cell-cell junction
- cornified envelope
- desmosome
- extracellular exosome
- integral component of membrane
- intercalated disc
- intracellular membrane-bounded organelle
- lateral plasma membrane
- plasma membrane

Biological process

- bundle of His cell-Purkinje myocyte adhesion involved in cell communication
- cell adhesion
- cell-cell adhesion
- cornification
- desmosome organization
- homophilic cell adhesion via plasma membrane adhesion molecules
- keratinization
- maternal process involved in female pregnancy
- Purkinje myocyte development
- regulation of heart rate by cardiac conduction
- regulation of ventricular cardiac muscle cell action potential
- response to progesterone

---

19

- **Protein name:** Putative P4-specific DNA primase
- **Organism:** Enterobacteria phage P4
- **Uniprot Accession Number:** P10277
- **Protein sequence length:** 777 aa
- **1D identity (%):** 10.94
- **1D identity (%) [Gaps excluded]:** 27.85
- **1D identity - Alignment Gaps:** 894
- **Common reported functions (%):** 0.0
- **Common reported locations (%):** 0.0
- **Common reported processes (%):** 0.0

- **PDB ID:** 1KA8
- **Chain:** E
- **Crystallized protein length:** 100 aa
- **Resolution:** 2.95 Å
- **Alinged residues range:** 16-18, 50-53, 51-53
- **Aligned to segment part (indices):** 2, 4, 0
- **Alinged residues range of reference:** 112-114, 232-235, 466-468
- **b-phipsi:** 0.187309
- **w-rdist:** 1.702512
- **t-alpha:** 0.139466
- **Chemical similarity (Tanimoto Index) (%):** 81.6
- **1D identity (%) [PDB]:** 0.09
- **1D identity (%) [Gaps excluded][PDB]:** 50.0
- **1D identity - Alignment Gaps [PDB]:** 1079
- **2D identity (%) [PDB]:** 7.09
- **2D identity (%) [Gaps excluded][PDB]:** 87.65
- **2D identity - Alignment Gaps [PDB]:** 921
- **3D similarity (TM-Score) (%) [PDB]:** 6.32

- **Gene name:** Alpha
- **RefSeq ID:** NC\_001609
- **Genomic sequence length:** 11624
- **5-UTR|CDS|3-UTR identity (%):** N/A | 33.11 | N/A
- **5-UTR|CDS|3-UTR identity (%) [Gaps excluded]:** N/A | 76.41 | N/A
- **5-UTR|CDS|3-UTR identity [Alignment Gaps]:** N/A | 2434 | N/A

**Uniprot Description:**  
  
This protein acts as a DNA primase generating di- to pentaribonucleotides; the predominant product being the dimer pppApG. It complexes specifically to the P4 origin of replication (ori) and its cis replication region (crr). It also acts as a DNA helicase.  
  
Homohexamer.  
  
**Gene Ontology Information:**

Molecular Function

- ATP binding
- DNA binding
- DNA helicase activity
- DNA primase activity
- hydrolase activity
- zinc ion binding

Location  
  
N/A

Biological process  
  
N/A

---

20

- **Protein name:** Deoxycytidylate 5-hydroxymethyltransferase
- **Organism:** Enterobacteria phage T4
- **Uniprot Accession Number:** P08773
- **Protein sequence length:** 246 aa
- **1D identity (%):** 3.74
- **1D identity (%) [Gaps excluded]:** 27.47
- **1D identity - Alignment Gaps:** 1155
- **Common reported functions (%):** 0.0
- **Common reported locations (%):** 0.0
- **Common reported processes (%):** 0.0

- **PDB ID:** 1B5E
- **Chain:** A
- **Crystallized protein length:** 241 aa
- **Resolution:** 1.6 Å
- **Alinged residues range:** 162-166, 235-237, 233-236
- **Aligned to segment part (indices):** 2, 4, 0
- **Alinged residues range of reference:** 125-129, 234-236, 465-468
- **b-phipsi:** 0.216963
- **w-rdist:** 5.287658
- **t-alpha:** 0.132812
- **Chemical similarity (Tanimoto Index) (%):** 85.83
- **1D identity (%) [PDB]:** 0.08
- **1D identity (%) [Gaps excluded][PDB]:** 50.0
- **1D identity - Alignment Gaps [PDB]:** 1220
- **2D identity (%) [PDB]:** 11.46
- **2D identity (%) [Gaps excluded][PDB]:** 87.32
- **2D identity - Alignment Gaps [PDB]:** 940
- **3D similarity (TM-Score) (%) [PDB]:** 8.92

- **Gene name:** 42
- **RefSeq ID:** NC\_000866
- **Genomic sequence length:** 168903
- **5-UTR|CDS|3-UTR identity (%):** N/A | 13.2 | N/A
- **5-UTR|CDS|3-UTR identity (%) [Gaps excluded]:** N/A | 78.78 | N/A
- **5-UTR|CDS|3-UTR identity [Alignment Gaps]:** N/A | 3253 | N/A

**Uniprot Description:**  
  
N/A  
  
**Gene Ontology Information:**

Molecular Function

- deoxycytidylate 5-hydroxymethyltransferase activity

Location  
  
N/A

Biological process  
  
N/A

---

21

- **Protein name:** H-2 class I histocompatibility antigen, D-B alpha chain
- **Organism:** Mus musculus
- **Uniprot Accession Number:** P01899
- **Protein sequence length:** 362 aa
- **1D identity (%):** 6.68
- **1D identity (%) [Gaps excluded]:** 26.2
- **1D identity - Alignment Gaps:** 971
- **Common reported functions (%):** 0.0
- **Common reported locations (%):** 0.0
- **Common reported processes (%):** 0.0

- **PDB ID:** 5SWZ
- **Chain:** P
- **Crystallized protein length:** 238 aa
- **Resolution:** 2.65 Å
- **Alinged residues range:** 121-125, 188-190, 186-189
- **Aligned to segment part (indices):** 2, 4, 0
- **Alinged residues range of reference:** 102-106, 234-236, 465-468
- **b-phipsi:** 0.23625
- **w-rdist:** 8.301742
- **t-alpha:** 0.072917
- **Chemical similarity (Tanimoto Index) (%):** 83.13
- **1D identity (%) [PDB]:** 0.0
- **1D identity (%) [Gaps excluded][PDB]:** 0.0
- **1D identity - Alignment Gaps [PDB]:** 1224
- **2D identity (%) [PDB]:** 14.06
- **2D identity (%) [Gaps excluded][PDB]:** 90.85
- **2D identity - Alignment Gaps [PDB]:** 896
- **3D similarity (TM-Score) (%) [PDB]:** 8.89

- **Gene name:** H2-D1
- **RefSeq ID:** N/A
- **Sequence length:** N/A
- **5-UTR|CDS|3-UTR identity (%):** N/A | N/A | N/A
- **5-UTR|CDS|3-UTR identity (%) [Gaps excluded]:** N/A | N/A | N/A
- **5-UTR|CDS|3-UTR identity [Alignment Gaps]:** N/A | N/A | N/A

**Uniprot Description:**  
  
Involved in the presentation of foreign antigens to the immune system.  
  
Heterodimer of an alpha chain and a beta chain (beta-2-microglobulin). Interacts with murid herpesvirus 4 protein K3 (mK3).  
  
**Gene Ontology Information:**

Molecular Function

- beta-2-microglobulin binding
- CD8 receptor binding
- peptide antigen binding
- peptide binding
- protein-containing complex binding
- signaling receptor binding
- T cell receptor binding
- TAP binding
- TAP complex binding

Location

- cell surface
- endoplasmic reticulum
- endoplasmic reticulum exit site
- external side of plasma membrane
- extracellular space
- Golgi apparatus
- Golgi medial cisterna
- integral component of lumenal side of endoplasmic reticulum membrane
- MHC class I peptide loading complex
- MHC class I protein complex
- phagocytic vesicle membrane
- plasma membrane

Biological process

- antigen processing and presentation of endogenous peptide antigen via MHC class I via ER pathway, TAP-dependent
- antigen processing and presentation of endogenous peptide antigen via MHC class Ib
- immune response
- negative regulation of neuron projection development
- positive regulation of T cell mediated cytotoxicity

---

22

- **Protein name:** Angiotensin-converting enzyme 2
- **Organism:** Homo sapiens
- **Uniprot Accession Number:** Q9BYF1
- **Protein sequence length:** 805 aa
- **1D identity (%):** 13.33
- **1D identity (%) [Gaps excluded]:** 30.09
- **1D identity - Alignment Gaps:** 802
- **Common reported functions (%):** 50.0
- **Common reported locations (%):** 37.5
- **Common reported processes (%):** 20.0

- **PDB ID:** 3D0G
- **Chain:** B
- **Crystallized protein length:** 597 aa
- **Resolution:** 2.8 Å
- **Alinged residues range:** 132-134, 133-138, 53-55, 130-133
- **Aligned to segment part (indices):** 2, 1, 4, 3
- **Alinged residues range of reference:** 120-122, 195-200, 234-236, 353-356
- **b-phipsi:** 0.418814
- **w-rdist:** 9.231837
- **t-alpha:** 0.111979
- **Chemical similarity (Tanimoto Index) (%):** 89.7
- **1D identity (%) [PDB]:** 0.06
- **1D identity (%) [Gaps excluded][PDB]:** 100.0
- **1D identity - Alignment Gaps [PDB]:** 1578
- **2D identity (%) [PDB]:** 17.27
- **2D identity (%) [Gaps excluded][PDB]:** 92.74
- **2D identity - Alignment Gaps [PDB]:** 1084
- **3D similarity (TM-Score) (%) [PDB]:** 15.5

- **Gene name:** ACE2
- **RefSeq ID:** NM\_021804
- **Transcript sequence length:** 3596
- **5-UTR|CDS|3-UTR identity (%):** 38.79 | 37.72 | 17.59
- **5-UTR|CDS|3-UTR identity (%) [Gaps excluded]:** 76.56 | 77.61 | 77.83
- **5-UTR|CDS|3-UTR identity [Alignment Gaps]:** 187 | 2158 | 695

**Uniprot Description:**  
  
Essential counter-regulatory carboxypeptidase of the renin-angiotensin hormone system that is a critical regulator of blood volume, systemic vascular resistance, and thus cardiovascular homeostasis (PubMed:27217402). Converts angiotensin I to angiotensin 1-9, a nine-amino acid peptide with anti-hypertrophic effects in cardiomyocytes, and angiotensin II to angiotensin 1-7, which then acts as a beneficial vasodilator and anti-proliferation agent, counterbalancing the actions of the vasoconstrictor angiotensin II (PubMed:10969042, PubMed:10924499, PubMed:11815627, PubMed:19021774, PubMed:14504186). Also removes the C-terminal residue from three other vasoactive peptides, neurotensin, kinetensin, and des-Arg bradykinin, but is not active on bradykinin (PubMed:10969042, PubMed:11815627). Also cleaves other biological peptides, such as apelins (apelin-13, [Pyr1]apelin-13, apelin-17, apelin-36), casomorphins (beta-casomorphin-7, neocasomorphin) and dynorphin A with high efficiency (PubMed:11815627, PubMed:27217402, PubMed:28293165). In addition, ACE2 C-terminus is homologous to collectrin and is responsible for the trafficking of the neutral amino acid transporter SL6A19 to the plasma membrane of gut epithelial cells via direct interaction, regulating its expression on the cell surface and its catalytic activity (PubMed:18424768, PubMed:19185582).  
  
Homodimer (PubMed:32132184). Interacts with the catalytically active form of TMPRSS2 (PubMed:21068237). Interacts with SLC6A19; this interaction is essential for expression and function of SLC6A19 in intestine (By similarity). Interacts with ITGA5:ITGB1 (PubMed:15276642, PubMed:33102950).  
  
**Gene Ontology Information:**

Molecular Function

- carboxypeptidase activity
- endopeptidase activity
- identical protein binding
- metallocarboxypeptidase activity
- metallopeptidase activity
- peptidyl-dipeptidase activity
- virus receptor activity
- zinc ion binding

Location

- apical plasma membrane
- brush border membrane
- cell surface
- cilium
- endocytic vesicle membrane
- endoplasmic reticulum lumen
- extracellular exosome
- extracellular region
- extracellular space
- integral component of membrane
- membrane raft
- plasma membrane

Biological process

- angiotensin maturation
- angiotensin-mediated drinking behavior
- blood vessel diameter maintenance
- negative regulation of signaling receptor activity
- positive regulation of amino acid transport
- positive regulation of cardiac muscle contraction
- positive regulation of gap junction assembly
- positive regulation of L-proline import across plasma membrane
- positive regulation of reactive oxygen species metabolic process
- proteolysis
- receptor-mediated virion attachment to host cell
- regulation of cardiac conduction
- regulation of cell population proliferation
- regulation of cytokine production
- regulation of inflammatory response
- regulation of systemic arterial blood pressure by renin-angiotensin
- regulation of transmembrane transporter activity
- regulation of vasoconstriction
- tryptophan transport
- viral entry into host cell

---
