# Supplementary material for: Identifying and profiling structural similarities between Spike of SARS-CoV-2 and other viral or host proteins with Machaon
Source: Commun Biol. 2023 Jul 19;6:752. doi: 10.1038/s42003-023-05076-7 (PMC10356814; doi:10.1038/s42003-023-05076-7)
Supplement: Supplementary file 7 — Supplementary Data 4 [file 42003_2023_5076_MOESM7_ESM.zip › 6VXX_A_segment/candidates/6VXX_A_site4-metrics-merged-enriched_eval_report.html]

 

# Structural Comparison Report for 6VXX\_A\_site4 - segments (total: 32)

---

1

- **Protein name:** Fiber protein 2
- **Organism:** Human adenovirus F serotype 41
- **Uniprot Accession Number:** P16883
- **Protein sequence length:** 387 aa
- **1D identity (%):** 6.65
- **1D identity (%) [Gaps excluded]:** 27.73
- **1D identity - Alignment Gaps:** 1018
- **Common reported functions (%):** 0.0
- **Common reported locations (%):** 0.0
- **Common reported processes (%):** 10.0

- **PDB ID:** 2BZU
- **Chain:** A
- **Crystallized protein length:** 159 aa
- **Resolution:** 1.5 Å
- **Alinged residues range:** 363-365, 363-366, 264-271
- **Aligned to segment part (indices):** 4, 0, 3
- **Alinged residues range of reference:** 200-202, 396-399, 546-553
- **b-phipsi:** 0.002439
- **w-rdist:** 0.869501
- **t-alpha:** 0.008591
- **Chemical similarity (Tanimoto Index) (%):** 83.39
- **1D identity (%) [PDB]:** 0.09
- **1D identity (%) [Gaps excluded][PDB]:** 100.0
- **1D identity - Alignment Gaps [PDB]:** 1140
- **2D identity (%) [PDB]:** 11.98
- **2D identity (%) [Gaps excluded][PDB]:** 91.67
- **2D identity - Alignment Gaps [PDB]:** 878
- **3D similarity (TM-Score) (%) [PDB]:** 8.01

- **Gene name:** N/A
- **RefSeq ID:** N/A
- **Sequence length:** N/A
- **5-UTR|CDS|3-UTR identity (%):** N/A | N/A | N/A
- **5-UTR|CDS|3-UTR identity (%) [Gaps excluded]:** N/A | N/A | N/A
- **5-UTR|CDS|3-UTR identity [Alignment Gaps]:** N/A | N/A | N/A

**Uniprot Description:**  
  
Forms spikes that protrude from each vertex of the icosahedral capsid. Interacts with host receptor CXCAR to provide virion initial attachment to target cell. Fiber proteins are shed during virus entry, when virus is still at the cell surface (By similarity).  
  
Homotrimer. Interacts with host receptor CXCAR. Interacts (via N-terminal tail region) with pentons (By similarity).  
  
**Gene Ontology Information:**

Molecular Function  
  
N/A

Location

- host cell nucleus
- viral capsid

Biological process

- adhesion receptor-mediated virion attachment to host cell
- cell adhesion
- viral entry into host cell

---

2

- **Protein name:** Coat protein
- **Organism:** Turnip yellow mosaic virus
- **Uniprot Accession Number:** P03608
- **Protein sequence length:** 189 aa
- **1D identity (%):** 3.39
- **1D identity (%) [Gaps excluded]:** 26.83
- **1D identity - Alignment Gaps:** 1134
- **Common reported functions (%):** 0.0
- **Common reported locations (%):** 0.0
- **Common reported processes (%):** 0.0

- **PDB ID:** 1W39
- **Chain:** C
- **Crystallized protein length:** 189 aa
- **Resolution:** 3.75 Å
- **Alinged residues range:** 178-180, 32-39, 177-180
- **Aligned to segment part (indices):** 4, 0, 3
- **Alinged residues range of reference:** 200-202, 394-401, 513-516
- **b-phipsi:** 0.002617
- **w-rdist:** 0.873455
- **t-alpha:** 0.006814
- **Chemical similarity (Tanimoto Index) (%):** 84.17
- **1D identity (%) [PDB]:** 0.0
- **1D identity (%) [Gaps excluded][PDB]:** 0.0
- **1D identity - Alignment Gaps [PDB]:** 1172
- **2D identity (%) [PDB]:** 13.16
- **2D identity (%) [Gaps excluded][PDB]:** 87.01
- **2D identity - Alignment Gaps [PDB]:** 864
- **3D similarity (TM-Score) (%) [PDB]:** 9.54

- **Gene name:** N/A
- **RefSeq ID:** NC\_004063
- **Genomic sequence length:** 6318
- **5-UTR|CDS|3-UTR identity (%):** N/A | 9.11 | N/A
- **5-UTR|CDS|3-UTR identity (%) [Gaps excluded]:** N/A | 77.16 | N/A
- **5-UTR|CDS|3-UTR identity [Alignment Gaps]:** N/A | 3464 | N/A

**Uniprot Description:**  
  
N/A  
  
The virus coat is composed of 180 copies of the coat protein arranged in an icosahedral shell.  
  
**Gene Ontology Information:**

Molecular Function

- structural molecule activity

Location

- T=3 icosahedral viral capsid

Biological process  
  
N/A

---

3

- **Protein name:** Genome polyprotein
- **Organism:** Yellow fever virus (strain 17D vaccine)
- **Uniprot Accession Number:** P03314
- **Protein sequence length:** 3411 aa
- **1D identity (%):** 7.05
- **1D identity (%) [Gaps excluded]:** 28.12
- **1D identity - Alignment Gaps:** 2806
- **Common reported functions (%):** 0.0
- **Common reported locations (%):** 37.5
- **Common reported processes (%):** 20.0

- **PDB ID:** 3EVD
- **Chain:** A
- **Crystallized protein length:** 261 aa
- **Resolution:** 1.5 Å
- **Alinged residues range:** 125-127, 124-127, 215-220, 19-25
- **Aligned to segment part (indices):** 4, 0, 3, 1
- **Alinged residues range of reference:** 200-202, 395-398, 561-566, 984-990
- **b-phipsi:** 0.024354
- **w-rdist:** 0.587146
- **t-alpha:** 0.005111
- **Chemical similarity (Tanimoto Index) (%):** 83.36
- **1D identity (%) [PDB]:** 0.0
- **1D identity (%) [Gaps excluded][PDB]:** 0.0
- **1D identity - Alignment Gaps [PDB]:** 1244
- **2D identity (%) [PDB]:** 11.93
- **2D identity (%) [Gaps excluded][PDB]:** 95.65
- **2D identity - Alignment Gaps [PDB]:** 968
- **3D similarity (TM-Score) (%) [PDB]:** 9.22

- **Gene name:** N/A
- **RefSeq ID:** NC\_002031
- **Genomic sequence length:** 10862
- **5-UTR|CDS|3-UTR identity (%):** N/A | 23.34 | N/A
- **5-UTR|CDS|3-UTR identity (%) [Gaps excluded]:** N/A | 79.01 | N/A
- **5-UTR|CDS|3-UTR identity [Alignment Gaps]:** N/A | 7646 | N/A

**Uniprot Description:**  
  
Capsid protein C
Plays a role in virus budding by binding to the cell membrane and gathering the viral RNA into a nucleocapsid that forms the core of a mature virus particle. During virus entry, may induce genome penetration into the host cytoplasm after hemifusion induced by the surface proteins. Can migrate to the cell nucleus where it modulates host functions.  
  
Capsid protein C
Homodimer (PubMed:12768036). Interacts (via N-terminus) with host EXOC1 (via C-terminus); this interaction results in EXOC1 degradation through the proteasome degradation pathway (By similarity).  
  
**Gene Ontology Information:**

Molecular Function

- ATP binding
- double-stranded RNA binding
- GTP binding
- metal ion binding
- mRNA (guanine-N7-)-methyltransferase activity
- mRNA (nucleoside-2'-O-)-methyltransferase activity
- nucleoside-triphosphatase activity
- protein dimerization activity
- RNA helicase activity
- RNA-directed 5'-3' RNA polymerase activity
- serine-type endopeptidase activity
- structural molecule activity

Location

- extracellular region
- host cell endoplasmic reticulum membrane
- host cell nucleus
- host cell perinuclear region of cytoplasm
- integral component of membrane
- viral capsid
- viral envelope
- virion membrane

Biological process

- clathrin-dependent endocytosis of virus by host cell
- fusion of virus membrane with host endosome membrane
- induction by virus of host autophagy
- negative regulation of RNA interference
- suppression by virus of host STAT2 activity
- suppression by virus of host type I interferon-mediated signaling pathway
- viral budding from endoplasmic reticulum membrane
- viral RNA genome replication
- virion attachment to host cell

---

4

- **Protein name:** Spike glycoprotein
- **Organism:** Severe acute respiratory syndrome coronavirus
- **Uniprot Accession Number:** P59594
- **Protein sequence length:** 1255 aa
- **1D identity (%):** 76.35
- **1D identity (%) [Gaps excluded]:** 77.94
- **1D identity - Alignment Gaps:** 26
- **Common reported functions (%):** 100.0
- **Common reported locations (%):** 62.5
- **Common reported processes (%):** 90.0

- **PDB ID:** 5X5B
- **Chain:** C
- **Crystallized protein length:** 1053 aa
- **Resolution:** 3.7 Å
- **Alinged residues range:** 383-385, 342-429, 492-557, 955-963
- **Aligned to segment part (indices):** 4, 0, 3, 1
- **Alinged residues range of reference:** 200-202, 355-442, 506-571, 973-981
- **b-phipsi:** 0.005646
- **w-rdist:** 0.705769
- **t-alpha:** 0.046346
- **Chemical similarity (Tanimoto Index) (%):** 86.07
- **1D identity (%) [PDB]:** 67.43
- **1D identity (%) [Gaps excluded][PDB]:** 77.91
- **1D identity - Alignment Gaps [PDB]:** 147
- **2D identity (%) [PDB]:** 55.06
- **2D identity (%) [Gaps excluded][PDB]:** 83.03
- **2D identity - Alignment Gaps [PDB]:** 413
- **3D similarity (TM-Score) (%) [PDB]:** 94.77

- **Gene name:** S
- **RefSeq ID:** NC\_004718
- **Genomic sequence length:** 29751
- **5-UTR|CDS|3-UTR identity (%):** 88.52 | 73.15 | 22.38
- **5-UTR|CDS|3-UTR identity (%) [Gaps excluded]:** 92.28 | 78.79 | 98.18
- **5-UTR|CDS|3-UTR identity [Alignment Gaps]:** 11 | 282 | 745

**Uniprot Description:**  
  
Spike glycoprotein
May down-regulate host tetherin (BST2) by lysosomal degradation, thereby counteracting its antiviral activity.  
  
Homotrimer; each monomer consists of a S1 and a S2 subunit. The resulting peplomers protrude from the virus surface as spikes (By similarity). Binds to human and palm civet ACE2 and human CLEC4M/DC-SIGNR. Interacts with the accessory proteins 3a and 7a.  
  
**Gene Ontology Information:**

Molecular Function

- host cell surface receptor binding
- identical protein binding

Location

- host cell endoplasmic reticulum-Golgi intermediate compartment membrane
- host cell plasma membrane
- integral component of membrane
- viral envelope
- virion membrane

Biological process

- endocytosis involved in viral entry into host cell
- fusion of virus membrane with host endosome membrane
- fusion of virus membrane with host plasma membrane
- pathogenesis
- receptor-mediated virion attachment to host cell
- suppression by virus of host tetherin activity
- suppression by virus of host type I interferon-mediated signaling pathway
- viral protein processing
- viral translation

---

5

- **Protein name:** H-2 class I histocompatibility antigen, D-B alpha chain
- **Organism:** Mus musculus
- **Uniprot Accession Number:** P01899
- **Protein sequence length:** 362 aa
- **1D identity (%):** 6.68
- **1D identity (%) [Gaps excluded]:** 26.2
- **1D identity - Alignment Gaps:** 971
- **Common reported functions (%):** 0.0
- **Common reported locations (%):** 0.0
- **Common reported processes (%):** 0.0

- **PDB ID:** 5SWS
- **Chain:** A
- **Crystallized protein length:** 273 aa
- **Resolution:** 2.86 Å
- **Alinged residues range:** 100-102, 7-12, 100-103, 64-70
- **Aligned to segment part (indices):** 4, 0, 3, 1
- **Alinged residues range of reference:** 200-202, 396-401, 514-517, 986-992
- **b-phipsi:** 0.009871
- **w-rdist:** 0.75155
- **t-alpha:** 0.028021
- **Chemical similarity (Tanimoto Index) (%):** 85.56
- **1D identity (%) [PDB]:** 0.0
- **1D identity (%) [Gaps excluded][PDB]:** 0.0
- **1D identity - Alignment Gaps [PDB]:** 1258
- **2D identity (%) [PDB]:** 16.98
- **2D identity (%) [Gaps excluded][PDB]:** 87.75
- **2D identity - Alignment Gaps [PDB]:** 850
- **3D similarity (TM-Score) (%) [PDB]:** 10.83

- **Gene name:** H2-D1
- **RefSeq ID:** N/A
- **Sequence length:** N/A
- **5-UTR|CDS|3-UTR identity (%):** N/A | N/A | N/A
- **5-UTR|CDS|3-UTR identity (%) [Gaps excluded]:** N/A | N/A | N/A
- **5-UTR|CDS|3-UTR identity [Alignment Gaps]:** N/A | N/A | N/A

**Uniprot Description:**  
  
Involved in the presentation of foreign antigens to the immune system.  
  
Heterodimer of an alpha chain and a beta chain (beta-2-microglobulin). Interacts with murid herpesvirus 4 protein K3 (mK3).  
  
**Gene Ontology Information:**

Molecular Function

- beta-2-microglobulin binding
- CD8 receptor binding
- peptide antigen binding
- peptide binding
- protein-containing complex binding
- signaling receptor binding
- T cell receptor binding
- TAP binding
- TAP complex binding

Location

- cell surface
- endoplasmic reticulum
- endoplasmic reticulum exit site
- external side of plasma membrane
- extracellular space
- Golgi apparatus
- Golgi medial cisterna
- integral component of lumenal side of endoplasmic reticulum membrane
- MHC class I peptide loading complex
- MHC class I protein complex
- phagocytic vesicle membrane
- plasma membrane

Biological process

- antigen processing and presentation of endogenous peptide antigen via MHC class I via ER pathway, TAP-dependent
- antigen processing and presentation of endogenous peptide antigen via MHC class Ib
- immune response
- negative regulation of neuron projection development
- positive regulation of T cell mediated cytotoxicity

---

6

- **Protein name:** Capsid protein
- **Organism:** Faba bean necrotic stunt virus
- **Uniprot Accession Number:** C7DLN3
- **Protein sequence length:** 172 aa
- **1D identity (%):** 2.81
- **1D identity (%) [Gaps excluded]:** 28.68
- **1D identity - Alignment Gaps:** 1187
- **Common reported functions (%):** 0.0
- **Common reported locations (%):** 0.0
- **Common reported processes (%):** 0.0

- **PDB ID:** 6S44
- **Chain:** A
- **Crystallized protein length:** 146 aa
- **Resolution:** 3.19 Å
- **Alinged residues range:** 133-135, 133-138, 31-35, 91-93
- **Aligned to segment part (indices):** 4, 0, 3, 1
- **Alinged residues range of reference:** 200-202, 396-401, 508-512, 973-975
- **b-phipsi:** 0.016495
- **w-rdist:** 0.723803
- **t-alpha:** 0.027257
- **Chemical similarity (Tanimoto Index) (%):** 83.13
- **1D identity (%) [PDB]:** 0.0
- **1D identity (%) [Gaps excluded][PDB]:** 0.0
- **1D identity - Alignment Gaps [PDB]:** 1129
- **2D identity (%) [PDB]:** 11.31
- **2D identity (%) [Gaps excluded][PDB]:** 94.21
- **2D identity - Alignment Gaps [PDB]:** 887
- **3D similarity (TM-Score) (%) [PDB]:** 8.75

- **Gene name:** N/A
- **RefSeq ID:** NC\_013095
- **Genomic sequence length:** 992
- **5-UTR|CDS|3-UTR identity (%):** N/A | 9.89 | N/A
- **5-UTR|CDS|3-UTR identity (%) [Gaps excluded]:** N/A | 82.01 | N/A
- **5-UTR|CDS|3-UTR identity [Alignment Gaps]:** N/A | 3407 | N/A

**Uniprot Description:**  
  
N/A  
  
**Gene Ontology Information:**

Molecular Function  
  
N/A

Location  
  
N/A

Biological process  
  
N/A

---

7

- **Protein name:** H-2 class I histocompatibility antigen, K-B alpha chain
- **Organism:** Mus musculus
- **Uniprot Accession Number:** P01901
- **Protein sequence length:** 369 aa
- **1D identity (%):** 6.17
- **1D identity (%) [Gaps excluded]:** 30.0
- **1D identity - Alignment Gaps:** 1082
- **Common reported functions (%):** 0.0
- **Common reported locations (%):** 0.0
- **Common reported processes (%):** 0.0

- **PDB ID:** 1N59
- **Chain:** A
- **Crystallized protein length:** 276 aa
- **Resolution:** 2.95 Å
- **Alinged residues range:** 100-102, 7-12, 97-102, 64-70
- **Aligned to segment part (indices):** 4, 0, 3, 1
- **Alinged residues range of reference:** 200-202, 396-401, 511-516, 986-992
- **b-phipsi:** 0.021538
- **w-rdist:** 0.873559
- **t-alpha:** 0.003407
- **Chemical similarity (Tanimoto Index) (%):** 85.5
- **1D identity (%) [PDB]:** 0.0
- **1D identity (%) [Gaps excluded][PDB]:** 0.0
- **1D identity - Alignment Gaps [PDB]:** 1259
- **2D identity (%) [PDB]:** 18.27
- **2D identity (%) [Gaps excluded][PDB]:** 86.76
- **2D identity - Alignment Gaps [PDB]:** 821
- **3D similarity (TM-Score) (%) [PDB]:** 10.02

- **Gene name:** H2-K1
- **RefSeq ID:** N/A
- **Sequence length:** N/A
- **5-UTR|CDS|3-UTR identity (%):** N/A | N/A | N/A
- **5-UTR|CDS|3-UTR identity (%) [Gaps excluded]:** N/A | N/A | N/A
- **5-UTR|CDS|3-UTR identity [Alignment Gaps]:** N/A | N/A | N/A

**Uniprot Description:**  
  
Involved in the presentation of foreign antigens to the immune system.  
  
Heterodimer of an alpha chain and a beta chain (beta-2-microglobulin).  
  
**Gene Ontology Information:**

Molecular Function

- beta-2-microglobulin binding
- CD8 receptor binding
- peptide antigen binding
- peptide binding
- protein-containing complex binding
- signaling receptor binding
- T cell receptor binding
- TAP binding
- TAP complex binding

Location

- cell surface
- endoplasmic reticulum
- endoplasmic reticulum exit site
- external side of plasma membrane
- extracellular space
- Golgi apparatus
- Golgi medial cisterna
- integral component of lumenal side of endoplasmic reticulum membrane
- MHC class I peptide loading complex
- MHC class I protein complex
- phagocytic vesicle membrane
- plasma membrane

Biological process

- antigen processing and presentation of endogenous peptide antigen via MHC class I via ER pathway, TAP-dependent
- antigen processing and presentation of endogenous peptide antigen via MHC class Ib
- antigen processing and presentation of exogenous peptide antigen via MHC class I
- defense response to bacterium
- immune response
- inner ear development
- negative regulation of neuron projection development
- positive regulation of T cell mediated cytotoxicity

---

8

- **Protein name:** DNA primase/helicase
- **Organism:** Escherichia phage T7
- **Uniprot Accession Number:** P03692
- **Protein sequence length:** 566 aa
- **1D identity (%):** 11.03
- **1D identity (%) [Gaps excluded]:** 30.53
- **1D identity - Alignment Gaps:** 863
- **Common reported functions (%):** 50.0
- **Common reported locations (%):** 0.0
- **Common reported processes (%):** 0.0

- **PDB ID:** 6N7V
- **Chain:** E
- **Crystallized protein length:** 259 aa
- **Resolution:** 3.8 Å
- **Alinged residues range:** 532-534, 529-537, 451-454
- **Aligned to segment part (indices):** 4, 0, 1
- **Alinged residues range of reference:** 200-202, 434-440, 987-990
- **b-phipsi:** 0.008822
- **w-rdist:** 0.595764
- **t-alpha:** 0.142023
- **Chemical similarity (Tanimoto Index) (%):** 83.2
- **1D identity (%) [PDB]:** 0.08
- **1D identity (%) [Gaps excluded][PDB]:** 50.0
- **1D identity - Alignment Gaps [PDB]:** 1242
- **2D identity (%) [PDB]:** 15.2
- **2D identity (%) [Gaps excluded][PDB]:** 86.1
- **2D identity - Alignment Gaps [PDB]:** 872
- **3D similarity (TM-Score) (%) [PDB]:** 10.27

- **Gene name:** 4
- **RefSeq ID:** NC\_001604
- **Genomic sequence length:** 39937
- **5-UTR|CDS|3-UTR identity (%):** N/A | 29.08 | N/A
- **5-UTR|CDS|3-UTR identity (%) [Gaps excluded]:** N/A | 78.0 | N/A
- **5-UTR|CDS|3-UTR identity [Alignment Gaps]:** N/A | 2523 | N/A

**Uniprot Description:**  
  
Synthesizes short RNA primers for DNA replication. Unwinds the DNA at the replication forks and generates single-stranded DNA for both leading and lagging strand synthesis. The primase synthesizes short RNA primers on the lagging strand that the polymerase elongates using dNTPs.  
  
Homohexamer. Present in a mixture of heptamers and hexamers in the absence of DNA, and assembles onto ssDNA as a hexamer. Interacts with the DNA polymerase gp5; this interaction is essential to initiate leading-strand DNA synthesis. Interacts with single-stranded DNA-binding protein gp2.5.  
  
**Gene Ontology Information:**

Molecular Function

- ATP binding
- DNA helicase activity
- DNA primase activity
- identical protein binding
- zinc ion binding

Location  
  
N/A

Biological process  
  
N/A

---

9

- **Protein name:** Matrix protein VP40
- **Organism:** Zaire ebolavirus (strain Mayinga-76)
- **Uniprot Accession Number:** Q05128
- **Protein sequence length:** 326 aa
- **1D identity (%):** 6.31
- **1D identity (%) [Gaps excluded]:** 27.42
- **1D identity - Alignment Gaps:** 1001
- **Common reported functions (%):** 50.0
- **Common reported locations (%):** 25.0
- **Common reported processes (%):** 0.0

- **PDB ID:** 7JZT
- **Chain:** C
- **Crystallized protein length:** 233 aa
- **Resolution:** 3.77 Å
- **Alinged residues range:** 173-175, 301-304, 300-303, 236-238
- **Aligned to segment part (indices):** 4, 0, 3, 1
- **Alinged residues range of reference:** 200-202, 423-426, 530-533, 990-992
- **b-phipsi:** 0.000914
- **w-rdist:** 0.851054
- **t-alpha:** 0.189097
- **Chemical similarity (Tanimoto Index) (%):** 82.3
- **1D identity (%) [PDB]:** 0.08
- **1D identity (%) [Gaps excluded][PDB]:** 50.0
- **1D identity - Alignment Gaps [PDB]:** 1216
- **2D identity (%) [PDB]:** 13.62
- **2D identity (%) [Gaps excluded][PDB]:** 88.34
- **2D identity - Alignment Gaps [PDB]:** 894
- **3D similarity (TM-Score) (%) [PDB]:** 9.11

- **Gene name:** VP40
- **RefSeq ID:** NC\_002549
- **Genomic sequence length:** 18959
- **5-UTR|CDS|3-UTR identity (%):** 15.79 | 17.43 | 19.86
- **5-UTR|CDS|3-UTR identity (%) [Gaps excluded]:** 77.78 | 79.12 | 74.35
- **5-UTR|CDS|3-UTR identity [Alignment Gaps]:** 212 | 3069 | 524

**Uniprot Description:**  
  
Plays an essential role virus particle assembly and budding (PubMed:16719918). Acts by interacting with viral ribonucleocapsid and host members of the ESCRT (endosomal sorting complex required for transport) system such as host VPS4, PDCD6IP/ALIX, NEDD4 or TGS101 (PubMed:15892969, PubMed:16719918, PubMed:23637409, PubMed:25786915, PubMed:26753796, PubMed:27489272). May play a role in immune cell dysfunction by being packaged into exosomes that can decrease the viability of recipient cells (via RNAi suppression and exosome-bystander apoptosis) (PubMed:27872619).  
  
Homodimer (PubMed:23953110). Homohexamer (PubMed:23953110, PubMed:11118208). Homooctamer (PubMed:12919741). Exists as a dimer until it reorganizes at the plasma membrane into a hexameric form using phosphatidylinositol 4,5-bisphosphate (PI(4,5)P2) (PubMed:23953110, PubMed:25159197, PubMed:26753796, PubMed:29950600). Hexamers are critical for budding (PubMed:23953110). Octamers function in genome replication and RNA binding (PubMed:12919741). Interacts with host TSG101 (PubMed:12559917). As a homohexamer, interacts with the WW domain 3 of host NEDD4 (PubMed:11095724, PubMed:12559917). Interacts with the nucleoprotein/NP (PubMed:17229682, PubMed:21987757). Interacts (via YPx(n)L/I motif) with host PDCD6IP/ALIX; this interaction supports efficient egress of viral particles (PubMed:25786915). Interacts with VP35 (PubMed:16698994). Interacts with host ITCH; this interaction is required for efficient egress (PubMed:27489272).  
  
**Gene Ontology Information:**

Molecular Function

- identical protein binding
- RNA binding
- structural constituent of virion

Location

- extracellular region
- host cell endomembrane system
- host cell late endosome membrane
- host cell plasma membrane
- integral to membrane of host cell
- membrane raft
- virion membrane

Biological process

- intracellular transport of virus
- mitigation of host immune response by virus
- suppression of host defenses by symbiont
- viral budding
- viral budding from plasma membrane
- viral budding via host ESCRT complex

---

10

- **Protein name:** Ran-specific GTPase-activating protein 1
- **Organism:** Saccharomyces cerevisiae (strain ATCC 204508 / S288c)
- **Uniprot Accession Number:** P41920
- **Protein sequence length:** 201 aa
- **1D identity (%):** 3.31
- **1D identity (%) [Gaps excluded]:** 24.57
- **1D identity - Alignment Gaps:** 1124
- **Common reported functions (%):** 0.0
- **Common reported locations (%):** 0.0
- **Common reported processes (%):** 0.0

- **PDB ID:** 6A3B
- **Chain:** B
- **Crystallized protein length:** 129 aa
- **Resolution:** 2.51 Å
- **Alinged residues range:** 161-163, 157-162, 85-88, 186-190
- **Aligned to segment part (indices):** 4, 0, 3, 1
- **Alinged residues range of reference:** 200-202, 395-400, 551-554, 988-992
- **b-phipsi:** 0.003787
- **w-rdist:** 2.357049
- **t-alpha:** 0.0
- **Chemical similarity (Tanimoto Index) (%):** 82.56
- **1D identity (%) [PDB]:** 0.0
- **1D identity (%) [Gaps excluded][PDB]:** 0.0
- **1D identity - Alignment Gaps [PDB]:** 1113
- **2D identity (%) [PDB]:** 10.07
- **2D identity (%) [Gaps excluded][PDB]:** 91.82
- **2D identity - Alignment Gaps [PDB]:** 893
- **3D similarity (TM-Score) (%) [PDB]:** 6.75

- **Gene name:** YRB1
- **RefSeq ID:** N/A
- **Sequence length:** N/A
- **5-UTR|CDS|3-UTR identity (%):** N/A | N/A | N/A
- **5-UTR|CDS|3-UTR identity (%) [Gaps excluded]:** N/A | N/A | N/A
- **5-UTR|CDS|3-UTR identity [Alignment Gaps]:** N/A | N/A | N/A

**Uniprot Description:**  
  
Important for the export of protein containing nuclear export signal (NES) out of the nucleus. Stimulates the GTPase activity of GSP1 and GSP2.  
  
Interacts with GSP1 and PRP20.  
  
**Gene Ontology Information:**

Molecular Function

- GTPase activator activity
- small GTPase binding

Location

- cytoplasm
- nuclear pore
- nucleus

Biological process

- G1/S transition of mitotic cell cycle
- positive regulation of GTPase activity
- protein import into nucleus
- RNA export from nucleus
- ubiquitin-dependent protein catabolic process

---

11

- **Protein name:** RNA silencing suppressor p19
- **Organism:** Carnation Italian ringspot virus
- **Uniprot Accession Number:** Q66104
- **Protein sequence length:** 172 aa
- **1D identity (%):** 4.52
- **1D identity (%) [Gaps excluded]:** 35.8
- **1D identity - Alignment Gaps:** 1121
- **Common reported functions (%):** 0.0
- **Common reported locations (%):** 0.0
- **Common reported processes (%):** 0.0

- **PDB ID:** 6BJG
- **Chain:** B
- **Crystallized protein length:** 146 aa
- **Resolution:** 2.29 Å
- **Alinged residues range:** 109-111, 109-114, 33-35, 83-88
- **Aligned to segment part (indices):** 4, 0, 3, 1
- **Alinged residues range of reference:** 200-202, 396-401, 555-557, 977-982
- **b-phipsi:** 0.016057
- **w-rdist:** 1.002828
- **t-alpha:** 0.011925
- **Chemical similarity (Tanimoto Index) (%):** 82.94
- **1D identity (%) [PDB]:** 0.0
- **1D identity (%) [Gaps excluded][PDB]:** 0.0
- **1D identity - Alignment Gaps [PDB]:** 1130
- **2D identity (%) [PDB]:** 8.55
- **2D identity (%) [Gaps excluded][PDB]:** 87.13
- **2D identity - Alignment Gaps [PDB]:** 928
- **3D similarity (TM-Score) (%) [PDB]:** 7.18

- **Gene name:** ORF4
- **RefSeq ID:** NC\_003500
- **Genomic sequence length:** 4763
- **5-UTR|CDS|3-UTR identity (%):** N/A | 9.2 | N/A
- **5-UTR|CDS|3-UTR identity (%) [Gaps excluded]:** N/A | 79.38 | N/A
- **5-UTR|CDS|3-UTR identity [Alignment Gaps]:** N/A | 3439 | N/A

**Uniprot Description:**  
  
Acts as a suppressor of RNA-mediated gene silencing, also known as post-transcriptional gene silencing (PTGS), a mechanism of plant viral defense that limits the accumulation of viral RNAs. Binds to short interfering RNAs (siRNAs) with high affinity. Acts as a molecular caliper to specifically select siRNAs based on the length of the duplex region of the RNA.  
  
Homodimer.  
  
**Gene Ontology Information:**

Molecular Function

- RNA binding

Location

- virion

Biological process  
  
N/A

---

12

- **Protein name:** Epstein-Barr nuclear antigen 1
- **Organism:** Epstein-Barr virus (strain B95-8)
- **Uniprot Accession Number:** P03211
- **Protein sequence length:** 641 aa
- **1D identity (%):** 0.0
- **1D identity (%) [Gaps excluded]:** 0.0
- **1D identity - Alignment Gaps:** 1914
- **Common reported functions (%):** 0.0
- **Common reported locations (%):** 0.0
- **Common reported processes (%):** 0.0

- **PDB ID:** 6NPP
- **Chain:** B
- **Crystallized protein length:** 134 aa
- **Resolution:** 1.35 Å
- **Alinged residues range:** 599-601, 558-561, 594-601, 476-479
- **Aligned to segment part (indices):** 4, 0, 3, 1
- **Alinged residues range of reference:** 200-202, 433-436, 509-516, 987-990
- **b-phipsi:** 0.023935
- **w-rdist:** 0.517847
- **t-alpha:** 0.102215
- **Chemical similarity (Tanimoto Index) (%):** 83.99
- **1D identity (%) [PDB]:** 0.09
- **1D identity (%) [Gaps excluded][PDB]:** 100.0
- **1D identity - Alignment Gaps [PDB]:** 1115
- **2D identity (%) [PDB]:** 5.79
- **2D identity (%) [Gaps excluded][PDB]:** 96.83
- **2D identity - Alignment Gaps [PDB]:** 991
- **3D similarity (TM-Score) (%) [PDB]:** 6.51

- **Gene name:** EBNA1
- **RefSeq ID:** NC\_007605
- **Genomic sequence length:** 171823
- **5-UTR|CDS|3-UTR identity (%):** N/A | N/A | N/A
- **5-UTR|CDS|3-UTR identity (%) [Gaps excluded]:** N/A | N/A | N/A
- **5-UTR|CDS|3-UTR identity [Alignment Gaps]:** N/A | N/A | N/A

**Uniprot Description:**  
  
Plays an essential role in replication and partitioning of viral genomic DNA during latent viral infection. During this phase, the circular double-stranded viral DNA undergoes replication once per cell cycle and is efficiently partitioned to the daughter cells. EBNA1 activates the initiation of viral DNA replication through binding to specific sites in the viral latent origin of replication, oriP. Additionally, it governs the segregation of viral episomes by mediating their attachment to host cell metaphase chromosomes. Also activates the transcription of several viral latency genes. Finally, it can counteract the stabilization of host p53/TP53 by host USP7, thereby decreasing apoptosis and increasing host cell survival.  
  
Interacts with human USP7. Interacts with human EBP2; this interaction is important for the stable segregation of EBV episomes during cell division but not for the replication of the episomes. Interacts with BGLF4; this interaction facilitates the switch from latent to lytic DNA replication by down-regulating EBNA1 replication function. Interacts with human PAX5; this interaction promotes EBNA1-dependent transcription.  
  
**Gene Ontology Information:**

Molecular Function

- DNA binding
- DNA-binding transcription factor activity

Location

- host cell nucleus

Biological process

- positive regulation of transcription, DNA-templated
- regulation of DNA replication
- suppression by virus of host adaptive immune response
- suppression by virus of host antigen processing and presentation
- suppression by virus of host NF-kappaB transcription factor activity
- viral latency

---

13

- **Protein name:** L-alanyl-D-glutamate peptidase
- **Organism:** Listeria phage A500
- **Uniprot Accession Number:** Q37979
- **Protein sequence length:** 289 aa
- **1D identity (%):** 2.45
- **1D identity (%) [Gaps excluded]:** 26.72
- **1D identity - Alignment Gaps:** 1300
- **Common reported functions (%):** 0.0
- **Common reported locations (%):** 0.0
- **Common reported processes (%):** 0.0

- **PDB ID:** 2VO9
- **Chain:** A
- **Crystallized protein length:** 149 aa
- **Resolution:** 1.8 Å
- **Alinged residues range:** 132-135, 18-22, 133-135, 11-15
- **Aligned to segment part (indices):** 4, 0, 3, 1
- **Alinged residues range of reference:** 199-202, 411-415, 540-542, 988-992
- **b-phipsi:** 0.029424
- **w-rdist:** 0.998082
- **t-alpha:** 0.003419
- **Chemical similarity (Tanimoto Index) (%):** 80.77
- **1D identity (%) [PDB]:** 0.09
- **1D identity (%) [Gaps excluded][PDB]:** 100.0
- **1D identity - Alignment Gaps [PDB]:** 1129
- **2D identity (%) [PDB]:** 6.0
- **2D identity (%) [Gaps excluded][PDB]:** 98.46
- **2D identity - Alignment Gaps [PDB]:** 1001
- **3D similarity (TM-Score) (%) [PDB]:** 7.35

- **Gene name:** ply
- **RefSeq ID:** NC\_009810
- **Genomic sequence length:** 38867
- **5-UTR|CDS|3-UTR identity (%):** N/A | 15.38 | N/A
- **5-UTR|CDS|3-UTR identity (%) [Gaps excluded]:** N/A | 80.59 | N/A
- **5-UTR|CDS|3-UTR identity [Alignment Gaps]:** N/A | 3188 | N/A

**Uniprot Description:**  
  
Cell wall lytic enzyme. Hydrolyzes the link between L-alanine and D-glutamate residues in certain bacterial cell-wall glycopeptides.  
  
**Gene Ontology Information:**

Molecular Function

- peptidase activity

Location

- extracellular region

Biological process

- cell wall organization

---

14

- **Protein name:** NTF2-related export protein 1
- **Organism:** Homo sapiens
- **Uniprot Accession Number:** Q9UKK6
- **Protein sequence length:** 140 aa
- **1D identity (%):** 3.04
- **1D identity (%) [Gaps excluded]:** 30.47
- **1D identity - Alignment Gaps:** 1157
- **Common reported functions (%):** 0.0
- **Common reported locations (%):** 0.0
- **Common reported processes (%):** 0.0

- **PDB ID:** 6E5U
- **Chain:** B
- **Crystallized protein length:** 138 aa
- **Resolution:** 3.8 Å
- **Alinged residues range:** 116-118, 128-131, 114-118, 25-29
- **Aligned to segment part (indices):** 4, 0, 3, 1
- **Alinged residues range of reference:** 200-202, 434-437, 512-516, 986-990
- **b-phipsi:** 0.004807
- **w-rdist:** 1.172909
- **t-alpha:** 0.030664
- **Chemical similarity (Tanimoto Index) (%):** N/A
- **1D identity (%) [PDB]:** 0.09
- **1D identity (%) [Gaps excluded][PDB]:** 100.0
- **1D identity - Alignment Gaps [PDB]:** 1119
- **2D identity (%) [PDB]:** 9.32
- **2D identity (%) [Gaps excluded][PDB]:** 93.14
- **2D identity - Alignment Gaps [PDB]:** 917
- **3D similarity (TM-Score) (%) [PDB]:** 12.28

- **Gene name:** NXT1
- **RefSeq ID:** N/A
- **Sequence length:** N/A
- **5-UTR|CDS|3-UTR identity (%):** N/A | N/A | N/A
- **5-UTR|CDS|3-UTR identity (%) [Gaps excluded]:** N/A | N/A | N/A
- **5-UTR|CDS|3-UTR identity [Alignment Gaps]:** N/A | N/A | N/A

**Uniprot Description:**  
  
Stimulator of protein export for NES-containing proteins (PubMed:10567585). Also plays a role in the nuclear export of U1 snRNA, tRNA, and mRNA (PubMed:10848583). The NXF1-NXT1 heterodimer is involved in the export of HSP70 mRNA in conjunction with ALYREF/THOC4 and THOC5 (PubMed:19165146, PubMed:11259602).  
  
Heterodimer with NXF1 (PubMed:11583626). Forms a complex with RANGAP1, RANBP2/NUP358 and NXF1 (PubMed:14729961). Interacts (via NTF2 domain) with NXF1 (PubMed:11583626). Stabilizes the NTF2 domain of NXF1 by heterodimerization (PubMed:11583626). The formation of NXF1-NXT1 heterodimers is required for the NXF1-mediated nuclear mRNA export (PubMed:11583626). Preferentially binds Ran-GTP (PubMed:10567585). Associates with NXF2, NXF3 and NXF5. Does not bind nucleoporins (NPC) directly, its association to NPC is mediated by NXF1 (PubMed:11583626).  
  
**Gene Ontology Information:**

Molecular Function

- small GTPase binding

Location

- cytoplasm
- cytosol
- nuclear pore
- nuclear pore central transport channel
- nuclear speck
- nucleoplasm

Biological process

- mRNA export from nucleus
- nucleocytoplasmic transport
- protein import into nucleus

---

15

- **Protein name:** Epstein-Barr nuclear antigen 1
- **Organism:** Epstein-Barr virus (strain B95-8)
- **Uniprot Accession Number:** P03211
- **Protein sequence length:** 641 aa
- **1D identity (%):** 0.0
- **1D identity (%) [Gaps excluded]:** 0.0
- **1D identity - Alignment Gaps:** 1914
- **Common reported functions (%):** 0.0
- **Common reported locations (%):** 0.0
- **Common reported processes (%):** 0.0

- **PDB ID:** 5WMF
- **Chain:** C
- **Crystallized protein length:** 138 aa
- **Resolution:** 1.9 Å
- **Alinged residues range:** 599-601, 498-506, 594-601, 572-574
- **Aligned to segment part (indices):** 4, 0, 3, 1
- **Alinged residues range of reference:** 200-202, 426-436, 509-516, 989-991
- **b-phipsi:** 0.05213
- **w-rdist:** 0.667109
- **t-alpha:** 0.011925
- **Chemical similarity (Tanimoto Index) (%):** 83.51
- **1D identity (%) [PDB]:** 0.0
- **1D identity (%) [Gaps excluded][PDB]:** 0.0
- **1D identity - Alignment Gaps [PDB]:** 1121
- **2D identity (%) [PDB]:** 8.35
- **2D identity (%) [Gaps excluded][PDB]:** 94.51
- **2D identity - Alignment Gaps [PDB]:** 939
- **3D similarity (TM-Score) (%) [PDB]:** 6.61

- **Gene name:** EBNA1
- **RefSeq ID:** NC\_007605
- **Genomic sequence length:** 171823
- **5-UTR|CDS|3-UTR identity (%):** N/A | N/A | N/A
- **5-UTR|CDS|3-UTR identity (%) [Gaps excluded]:** N/A | N/A | N/A
- **5-UTR|CDS|3-UTR identity [Alignment Gaps]:** N/A | N/A | N/A

**Uniprot Description:**  
  
Plays an essential role in replication and partitioning of viral genomic DNA during latent viral infection. During this phase, the circular double-stranded viral DNA undergoes replication once per cell cycle and is efficiently partitioned to the daughter cells. EBNA1 activates the initiation of viral DNA replication through binding to specific sites in the viral latent origin of replication, oriP. Additionally, it governs the segregation of viral episomes by mediating their attachment to host cell metaphase chromosomes. Also activates the transcription of several viral latency genes. Finally, it can counteract the stabilization of host p53/TP53 by host USP7, thereby decreasing apoptosis and increasing host cell survival.  
  
Interacts with human USP7. Interacts with human EBP2; this interaction is important for the stable segregation of EBV episomes during cell division but not for the replication of the episomes. Interacts with BGLF4; this interaction facilitates the switch from latent to lytic DNA replication by down-regulating EBNA1 replication function. Interacts with human PAX5; this interaction promotes EBNA1-dependent transcription.  
  
**Gene Ontology Information:**

Molecular Function

- DNA binding
- DNA-binding transcription factor activity

Location

- host cell nucleus

Biological process

- positive regulation of transcription, DNA-templated
- regulation of DNA replication
- suppression by virus of host adaptive immune response
- suppression by virus of host antigen processing and presentation
- suppression by virus of host NF-kappaB transcription factor activity
- viral latency

---

16

- **Protein name:** Structural polyprotein
- **Organism:** Barmah forest virus
- **Uniprot Accession Number:** P89946
- **Protein sequence length:** 1239 aa
- **1D identity (%):** 15.55
- **1D identity (%) [Gaps excluded]:** 26.18
- **1D identity - Alignment Gaps:** 640
- **Common reported functions (%):** 0.0
- **Common reported locations (%):** 37.5
- **Common reported processes (%):** 10.0

- **PDB ID:** 2YEW
- **Chain:** A
- **Crystallized protein length:** 171 aa
- **Resolution:** 5.0 Å
- **Alinged residues range:** 223-225, 223-227, 219-225, 138-143
- **Aligned to segment part (indices):** 4, 0, 3, 1
- **Alinged residues range of reference:** 200-202, 376-380, 511-516, 985-990
- **b-phipsi:** 0.026362
- **w-rdist:** 0.702732
- **t-alpha:** 0.051971
- **Chemical similarity (Tanimoto Index) (%):** 82.98
- **1D identity (%) [PDB]:** 0.0
- **1D identity (%) [Gaps excluded][PDB]:** 0.0
- **1D identity - Alignment Gaps [PDB]:** 1154
- **2D identity (%) [PDB]:** 13.33
- **2D identity (%) [Gaps excluded][PDB]:** 89.93
- **2D identity - Alignment Gaps [PDB]:** 856
- **3D similarity (TM-Score) (%) [PDB]:** 6.93

- **Gene name:** N/A
- **RefSeq ID:** NC\_001786
- **Genomic sequence length:** 11488
- **5-UTR|CDS|3-UTR identity (%):** N/A | 40.54 | N/A
- **5-UTR|CDS|3-UTR identity (%) [Gaps excluded]:** N/A | 77.89 | N/A
- **5-UTR|CDS|3-UTR identity [Alignment Gaps]:** N/A | 2378 | N/A

**Uniprot Description:**  
  
Capsid protein
Forms an icosahedral capsid with a T=4 symmetry composed of 240 copies of the capsid protein surrounded by a lipid membrane through which penetrate 80 spikes composed of trimers of E1-E2 heterodimers (By similarity). The capsid protein binds to the viral RNA genome at a site adjacent to a ribosome binding site for viral genome translation following genome release (By similarity). Possesses a protease activity that results in its autocatalytic cleavage from the nascent structural protein (By similarity). Following its self-cleavage, the capsid protein transiently associates with ribosomes, and within several minutes the protein binds to viral RNA and rapidly assembles into icosahedric core particles (By similarity). The resulting nucleocapsid eventually associates with the cytoplasmic domain of the spike glycoprotein E2 at the cell membrane, leading to budding and formation of mature virions (By similarity). In case of infection, new virions attach to target cells and after clathrin-mediated endocytosis their membrane fuses with the host endosomal membrane (By similarity). This leads to the release of the nucleocapsid into the cytoplasm, followed by an uncoating event necessary for the genomic RNA to become accessible (By similarity). The uncoating might be triggered by the interaction of capsid proteins with ribosomes (By similarity). Binding of ribosomes would release the genomic RNA since the same region is genomic RNA-binding and ribosome-binding (By similarity).  
  
Capsid protein
Homodimer (By similarity). Homomultimer (Probable). Interacts with host karyopherin KPNA4; this interaction allows the nuclear import of the viral capsid protein (By similarity).  
  
**Gene Ontology Information:**

Molecular Function

- RNA binding
- serine-type endopeptidase activity
- structural molecule activity

Location

- host cell cytoplasm
- host cell nucleus
- host cell plasma membrane
- integral component of membrane
- T=4 icosahedral viral capsid
- virion membrane

Biological process

- fusion of virus membrane with host endosome membrane
- virion attachment to host cell

---

17

- **Protein name:** Tail tube terminator protein
- **Organism:** Escherichia phage lambda
- **Uniprot Accession Number:** P03732
- **Protein sequence length:** 131 aa
- **1D identity (%):** 2.58
- **1D identity (%) [Gaps excluded]:** 26.83
- **1D identity - Alignment Gaps:** 1158
- **Common reported functions (%):** 0.0
- **Common reported locations (%):** 0.0
- **Common reported processes (%):** 0.0

- **PDB ID:** 3FZ2
- **Chain:** H
- **Crystallized protein length:** 128 aa
- **Resolution:** 2.7 Å
- **Alinged residues range:** 123-125, 51-59, 121-127, 17-19
- **Aligned to segment part (indices):** 4, 0, 3, 1
- **Alinged residues range of reference:** 200-202, 436-444, 547-553, 977-979
- **b-phipsi:** 0.030346
- **w-rdist:** 0.612108
- **t-alpha:** 0.071168
- **Chemical similarity (Tanimoto Index) (%):** 81.73
- **1D identity (%) [PDB]:** 0.09
- **1D identity (%) [Gaps excluded][PDB]:** 100.0
- **1D identity - Alignment Gaps [PDB]:** 1113
- **2D identity (%) [PDB]:** 8.64
- **2D identity (%) [Gaps excluded][PDB]:** 90.72
- **2D identity - Alignment Gaps [PDB]:** 921
- **3D similarity (TM-Score) (%) [PDB]:** 6.34

- **Gene name:** U
- **RefSeq ID:** NC\_001416
- **Genomic sequence length:** 48502
- **5-UTR|CDS|3-UTR identity (%):** N/A | 6.41 | N/A
- **5-UTR|CDS|3-UTR identity (%) [Gaps excluded]:** N/A | 82.57 | N/A
- **5-UTR|CDS|3-UTR identity [Alignment Gaps]:** N/A | 3610 | N/A

**Uniprot Description:**  
  
Plays an essential role in tail assembly by capping the rapidly polymerizing tail once it has reached its requisite length and serving as the interaction surface for the completion protein.  
  
Homohexamer. May bind to major tail protein V, and /or tape measure protein.  
  
**Gene Ontology Information:**

Molecular Function  
  
N/A

Location

- host cell cytoplasm
- virus tail

Biological process

- viral genome ejection through host cell envelope, long flexible tail mechanism
- viral tail assembly

---

18

- **Protein name:** HLA class I histocompatibility antigen, alpha chain E
- **Organism:** Homo sapiens
- **Uniprot Accession Number:** P13747
- **Protein sequence length:** 358 aa
- **1D identity (%):** 4.64
- **1D identity (%) [Gaps excluded]:** 25.4
- **1D identity - Alignment Gaps:** 1127
- **Common reported functions (%):** 0.0
- **Common reported locations (%):** 0.0
- **Common reported processes (%):** 0.0

- **PDB ID:** 5W1V
- **Chain:** A
- **Crystallized protein length:** 272 aa
- **Resolution:** 3.31 Å
- **Alinged residues range:** 100-102, 97-101, 24-28, 59-73
- **Aligned to segment part (indices):** 4, 0, 3, 1
- **Alinged residues range of reference:** 200-202, 396-400, 547-551, 986-991
- **b-phipsi:** 0.018243
- **w-rdist:** 0.788708
- **t-alpha:** 0.137597
- **Chemical similarity (Tanimoto Index) (%):** N/A
- **1D identity (%) [PDB]:** 0.08
- **1D identity (%) [Gaps excluded][PDB]:** 100.0
- **1D identity - Alignment Gaps [PDB]:** 1254
- **2D identity (%) [PDB]:** 17.53
- **2D identity (%) [Gaps excluded][PDB]:** 86.32
- **2D identity - Alignment Gaps [PDB]:** 832
- **3D similarity (TM-Score) (%) [PDB]:** 9.82

- **Gene name:** HLA-E
- **RefSeq ID:** NM\_005516
- **Transcript sequence length:** 2548
- **5-UTR|CDS|3-UTR identity (%):** 7.55 | 17.46 | 9.86
- **5-UTR|CDS|3-UTR identity (%) [Gaps excluded]:** 86.96 | 75.11 | 84.09
- **5-UTR|CDS|3-UTR identity [Alignment Gaps]:** 242 | 3051 | 1325

**Uniprot Description:**  
  
Non-classical major histocompatibility class Ib molecule involved in immune self-nonself discrimination. In complex with B2M/beta-2-microglobulin binds nonamer self-peptides derived from the signal sequence of classical MHC class Ia molecules (VL9 peptides) (PubMed:9754572, PubMed:18083576, PubMed:18339401). Peptide-bound HLA-E-B2M heterotrimeric complex primarily functions as a ligand for natural killer (NK) cell inhibitory receptor KLRD1-KLRC1, enabling NK cells to monitor the expression of other MHC class I molecules in healthy cells and to tolerate self (PubMed:9754572, PubMed:9486650, PubMed:17179229, PubMed:18083576). Upon cellular stress, preferentially binds signal sequence-derived peptides from stress-induced chaperones and is no longer recognized by NK cell inhibitory receptor KLRD1-KLRC1, resulting in impaired protection from NK cells (PubMed:12461076). Binds signal sequence-derived peptides from non-classical MHC class Ib HLA-G molecules and acts as a ligand for NK cell activating receptor KLRD1-KLRC2, likely playing a role in the generation and effector functions of adaptive NK cells and in maternal-fetal tolerance during pregnancy (PubMed:9754572, PubMed:30134159). Besides self-peptides, can also bind and present pathogen-derived peptides conformationally similar to VL9 peptides to alpha-beta T cell receptor (TCR) on unconventional CD8+ cytotoxic T cells, ultimately triggering antimicrobial immune response (PubMed:16474394, PubMed:30087334).  
  
Forms a heterotrimer with B2M and a self- or a pathogen-derived peptide (peptide-bound HLA-E-B2M) (PubMed:18339401, PubMed:30087334). Similarly to MHC class Ia assembly, HLA-E-B2M heterodimer interacts with components of the antigen processing machinery TAPBP and TAP1-TAP2 complex; this interaction is required for peptide loading and translocation to the cell surface (PubMed:9427624). Interacts with CALCR; this interaction is required for appropriate folding (PubMed:9427624). The optimum binding peptide is a nonamer (VL9) that is primarily derived from amino-acid residues 3-11 of the signal sequences of most HLA-A, -B, -C and -G molecules (PubMed:9754572, PubMed:18083576, PubMed:9660937, PubMed:18339401). The VL9 peptide anchors to five main sites in the peptide-binding groove of HLA-E (PubMed:18339401). Peptide-bound HLA-E-B2M complex interacts with KLRD1-KLRC1 receptor on NK cells (PubMed:9486650, PubMed:18083576). Binds with lower affinity to activating KLRD1-KLRC2 (PubMed:18083576, PubMed:23335510). The common subunit KLRC1 plays a prominent role in directly interacting with HLA-E (PubMed:18083576). Peptide-bound HLA-E-B2M interacts with the alpha-beta TCR on unconventional CD8+ T cells (PubMed:16474394). Peptide-free HLA-E interacts with HLA-F-B2M complex; this interaction may regulate the intracellular trafficking and the stability of peptide-free MHC class I open conformers (OCs).  
  
**Gene Ontology Information:**

Molecular Function

- beta-2-microglobulin binding
- MHC class I protein binding
- natural killer cell lectin-like receptor binding
- peptide antigen binding
- signaling receptor binding
- T cell receptor binding

Location

- cell surface
- early endosome membrane
- ER to Golgi transport vesicle membrane
- external side of plasma membrane
- extracellular exosome
- extracellular space
- Golgi membrane
- integral component of lumenal side of endoplasmic reticulum membrane
- MHC class I protein complex
- MHC class Ib protein complex
- phagocytic vesicle membrane
- plasma membrane
- recycling endosome membrane

Biological process

- adaptive immune response
- antibacterial humoral response
- antigen processing and presentation of endogenous peptide antigen via MHC class Ib
- antigen processing and presentation of exogenous peptide antigen via MHC class I, TAP-dependent
- antigen processing and presentation of exogenous peptide antigen via MHC class I, TAP-independent
- antigen processing and presentation of exogenous peptide antigen via MHC class Ib
- antigen processing and presentation of peptide antigen via MHC class I
- CD8-positive, alpha-beta T cell activation
- defense response to Gram-positive bacterium
- innate immune response
- interferon-gamma-mediated signaling pathway
- natural killer cell tolerance induction
- negative regulation of natural killer cell mediated cytotoxicity
- positive regulation of antibody-dependent cellular cytotoxicity
- positive regulation of CD8-positive, alpha-beta T cell activation
- positive regulation of CD8-positive, alpha-beta T cell proliferation
- positive regulation of immunoglobulin production
- positive regulation of interleukin-13 production
- positive regulation of interleukin-4 production
- positive regulation of natural killer cell cytokine production
- positive regulation of natural killer cell mediated cytotoxicity
- positive regulation of natural killer cell mediated immunity
- positive regulation of natural killer cell proliferation
- positive regulation of T cell mediated cytotoxicity
- positive regulation of TRAIL production
- positive regulation of tumor necrosis factor production
- protection from natural killer cell mediated cytotoxicity
- regulation of immune response
- regulation of natural killer cell mediated immunity
- type I interferon signaling pathway
- viral process

---

19

- **Protein name:** Genome polyprotein
- **Organism:** Poliovirus type 1 (strain Mahoney)
- **Uniprot Accession Number:** P03300
- **Protein sequence length:** 2209 aa
- **1D identity (%):** 10.76
- **1D identity (%) [Gaps excluded]:** 26.36
- **1D identity - Alignment Gaps:** 1464
- **Common reported functions (%):** 0.0
- **Common reported locations (%):** 0.0
- **Common reported processes (%):** 10.0

- **PDB ID:** 1L1N
- **Chain:** A
- **Crystallized protein length:** 180 aa
- **Resolution:** 2.1 Å
- **Alinged residues range:** 159-161, 158-161, 58-61, 90-92
- **Aligned to segment part (indices):** 4, 0, 3, 1
- **Alinged residues range of reference:** 200-202, 395-398, 540-543, 973-975
- **b-phipsi:** 0.01562
- **w-rdist:** 1.19211
- **t-alpha:** 0.038938
- **Chemical similarity (Tanimoto Index) (%):** 73.35
- **1D identity (%) [PDB]:** 0.0
- **1D identity (%) [Gaps excluded][PDB]:** 0.0
- **1D identity - Alignment Gaps [PDB]:** 1163
- **2D identity (%) [PDB]:** 15.12
- **2D identity (%) [Gaps excluded][PDB]:** 92.07
- **2D identity - Alignment Gaps [PDB]:** 835
- **3D similarity (TM-Score) (%) [PDB]:** 7.46

- **Gene name:** N/A
- **RefSeq ID:** NC\_002058
- **Genomic sequence length:** 7440
- **5-UTR|CDS|3-UTR identity (%):** 22.22 | 36.52 | N/A
- **5-UTR|CDS|3-UTR identity (%) [Gaps excluded]:** 77.68 | 79.95 | N/A
- **5-UTR|CDS|3-UTR identity [Alignment Gaps]:** 559 | 3898 | N/A

**Uniprot Description:**  
  
Capsid protein VP1
Forms an icosahedral capsid of pseudo T=3 symmetry with capsid proteins VP2 and VP3 (PubMed:2994218). The capsid is 300 Angstroms in diameter, composed of 60 copies of each capsid protein and enclosing the viral positive strand RNA genome (PubMed:2994218). Capsid protein VP1 mainly forms the vertices of the capsid (PubMed:23365424). Capsid protein VP1 interacts with host cell receptor PVR to provide virion attachment to target host epithelial cells (PubMed:25631086). This attachment induces virion internalization predominantly through clathrin- and caveolin-independent endocytosis in Hela cells and through caveolin-mediated endocytosis in brain microvascular endothelial cells (PubMed:17717529, PubMed:18191571, PubMed:17622193). Tyrosine kinases are probably involved in the entry process (PubMed:17717529). Virus binding to PVR induces increased junctional permeability and rearrangement of junctional proteins (PubMed:17717529). Modulation of endothelial tight junctions, as well as cytolytic infection of endothelial cells themselves, may result in loss of endothelial integrity which may help the virus to reach the CNS (PubMed:17717529). After binding to its receptor, the capsid undergoes conformational changes (PubMed:25631086). Capsid protein VP1 N-terminus (that contains an amphipathic alpha-helix) and capsid protein VP4 are externalized (PubMed:25631086). Together, they shape a pore in the host membrane through which viral genome is translocated to host cell cytoplasm (PubMed:25631086).  
  
Capsid protein VP0
Interacts with capsid protein VP1 and capsid protein VP3 to form heterotrimeric protomers.  
  
**Gene Ontology Information:**

Molecular Function

- ATP binding
- cysteine-type endopeptidase activity
- ion channel activity
- metal ion binding
- nucleoside-triphosphatase activity
- RNA binding
- RNA helicase activity
- RNA-directed 5'-3' RNA polymerase activity
- structural molecule activity

Location

- host cell cytoplasmic vesicle membrane
- host cell nucleus
- integral to membrane of host cell
- membrane
- T=pseudo3 icosahedral viral capsid
- viral capsid

Biological process

- endocytosis involved in viral entry into host cell
- induction by virus of host autophagy
- pore formation by virus in membrane of host cell
- pore-mediated entry of viral genome into host cell
- positive stranded viral RNA replication
- protein complex oligomerization
- RNA-protein covalent cross-linking
- suppression by virus of host MAVS activity
- suppression by virus of host MDA-5 activity
- suppression by virus of host mRNA export from nucleus
- suppression by virus of host RIG-I activity
- suppression by virus of host translation initiation factor activity
- transcription, DNA-templated
- viral RNA genome replication
- virion assembly
- virion attachment to host cell

---

20

- **Protein name:** Main hemagglutinin component type C
- **Organism:** Clostridium botulinum C phage
- **Uniprot Accession Number:** P0DPR0
- **Protein sequence length:** 286 aa
- **1D identity (%):** 5.52
- **1D identity (%) [Gaps excluded]:** 30.93
- **1D identity - Alignment Gaps:** 1087
- **Common reported functions (%):** 0.0
- **Common reported locations (%):** 0.0
- **Common reported processes (%):** 10.0

- **PDB ID:** 1QXM
- **Chain:** B
- **Crystallized protein length:** 283 aa
- **Resolution:** 1.7 Å
- **Alinged residues range:** 237-252, 241-247, 144-146
- **Aligned to segment part (indices):** 0, 3, 1
- **Alinged residues range of reference:** 427-450, 548-554, 987-989
- **b-phipsi:** 0.011574
- **w-rdist:** 2.347235
- **t-alpha:** 0.013817
- **Chemical similarity (Tanimoto Index) (%):** 83.23
- **1D identity (%) [PDB]:** 0.0
- **1D identity (%) [Gaps excluded][PDB]:** 0.0
- **1D identity - Alignment Gaps [PDB]:** 1266
- **2D identity (%) [PDB]:** 20.61
- **2D identity (%) [Gaps excluded][PDB]:** 85.02
- **2D identity - Alignment Gaps [PDB]:** 772
- **3D similarity (TM-Score) (%) [PDB]:** 9.94

- **Gene name:** HA-33
- **RefSeq ID:** NC\_007581
- **Genomic sequence length:** 185683
- **5-UTR|CDS|3-UTR identity (%):** N/A | 16.49 | N/A
- **5-UTR|CDS|3-UTR identity (%) [Gaps excluded]:** N/A | 79.7 | N/A
- **5-UTR|CDS|3-UTR identity [Alignment Gaps]:** N/A | 3077 | N/A

**Uniprot Description:**  
  
Agglutinates human erythrocytes (PubMed:2205574). The hemagglutinin (HA) component of the progenitor toxin protects the structural integrity of botulinum neurotoxin; may increase internalization of the neurotoxin into the bloodstream of the host (PubMed:9421908). The hemagglutinin (HA) component is involved in binding to the upper small intestine through interactions with glycolipids and glycoproteins containing sialic acid moieties (Probable). Binds galactose or oligosaccharides with galactose at their non-reducing end (PubMed:14663070). Binds eukaryotic host mucins; binding is inhibited by N-acetyl-beta-neuraminic acid, N-acetyl-D-galactosamine, galactose, and methyl N-acetyl-beta-neuraminic acid (PubMed:18178224). Binds N-acetyl-beta-neuraminic acid, N-acetyl-D-galactosamine and galactose (but not glucose) via 2 sites (PubMed:18178224, PubMed:21640703).  
  
Botulinum toxins are produced as progenitor toxins of large molecular sizes of 12S (M toxin) and 16S (L toxin). M toxin consists of a non-toxic, non-hemagglutinin component (NTNHA) and the neurotoxin (Probable). L toxin consists of the M toxin and the 3 subcomponents of hemagglutinin (HA) (PubMed:7802661). HA is composed of subcomponents of 70, 33, and 17 kDa (PubMed:7802661). The 70 kDa subcomponent undergoes proteolytic processing and is split into HA-55 (also called HA-53 and HA3b) and HA-22-23 (also called HA3a) (PubMed:7802661). The stoichiometry of the whole complex has been modeled as one BoNT/C, one NTNHA, three HA-70, six HA-33 and three HA-17 (By similarity).  
  
**Gene Ontology Information:**

Molecular Function

- carbohydrate binding

Location

- extracellular region

Biological process

- pathogenesis

---

21

- **Protein name:** Envelope glycoprotein gp160
- **Organism:** Human immunodeficiency virus type 1 group M subtype B (isolate HXB2)
- **Uniprot Accession Number:** P04578
- **Protein sequence length:** 856 aa
- **1D identity (%):** 12.69
- **1D identity (%) [Gaps excluded]:** 26.64
- **1D identity - Alignment Gaps:** 755
- **Common reported functions (%):** 50.0
- **Common reported locations (%):** 50.0
- **Common reported processes (%):** 30.0

- **PDB ID:** 4JPJ
- **Chain:** C
- **Crystallized protein length:** 157 aa
- **Resolution:** 2.5 Å
- **Alinged residues range:** 148-150, 148-153, 20-25, 116-119
- **Aligned to segment part (indices):** 4, 0, 3, 1
- **Alinged residues range of reference:** 200-202, 376-381, 548-553, 977-980
- **b-phipsi:** 0.022226
- **w-rdist:** 1.029594
- **t-alpha:** 0.069847
- **Chemical similarity (Tanimoto Index) (%):** 98.15
- **1D identity (%) [PDB]:** 0.09
- **1D identity (%) [Gaps excluded][PDB]:** 50.0
- **1D identity - Alignment Gaps [PDB]:** 1138
- **2D identity (%) [PDB]:** 11.51
- **2D identity (%) [Gaps excluded][PDB]:** 86.57
- **2D identity - Alignment Gaps [PDB]:** 874
- **3D similarity (TM-Score) (%) [PDB]:** 8.63

- **Gene name:** env
- **RefSeq ID:** NC\_001802
- **Genomic sequence length:** 9181
- **5-UTR|CDS|3-UTR identity (%):** 23.6 | 39.16 | 23.63
- **5-UTR|CDS|3-UTR identity (%) [Gaps excluded]:** 75.9 | 79.04 | 80.65
- **5-UTR|CDS|3-UTR identity [Alignment Gaps]:** 184 | 2157 | 374

**Uniprot Description:**  
  
Envelope glycoprotein gp160
Oligomerizes in the host endoplasmic reticulum into predominantly trimers. In a second time, gp160 transits in the host Golgi, where glycosylation is completed. The precursor is then proteolytically cleaved in the trans-Golgi and thereby activated by cellular furin or furin-like proteases to produce gp120 and gp41.  
  
Surface protein gp120
The mature envelope protein (Env) consists of a homotrimer of non-covalently associated gp120-gp41 heterodimers. The resulting complex protrudes from the virus surface as a spike. There seems to be as few as 10 spikes on the average virion. Interacts with host CD4, CCR5 and CXCR4. Gp120 also interacts with the C-type lectins CD209/DC-SIGN and CLEC4M/DC-SIGNR (collectively referred to as DC-SIGN(R)). Gp120 and gp41 interact with GalCer. Gp120 interacts with host ITGA4/ITGB7 complex; on CD4+ T-cells, this interaction results in rapid activation of integrin ITGAL/LFA-1, which facilitates efficient cell-to-cell spreading of HIV-1. Gp120 interacts with cell-associated heparan sulfate; this interaction increases virus infectivity on permissive cells and may be involved in infection of CD4- cells.  
  
**Gene Ontology Information:**

Molecular Function

- identical protein binding
- structural molecule activity

Location

- host cell endosome membrane
- host cell plasma membrane
- integral component of membrane
- viral envelope
- virion
- virion membrane

Biological process

- actin filament reorganization
- clathrin-dependent endocytosis of virus by host cell
- entry into host
- fusion of virus membrane with host endosome membrane
- fusion of virus membrane with host plasma membrane
- mitigation of host immune response by virus
- positive regulation of establishment of T cell polarity
- positive regulation of plasma membrane raft polarization
- positive regulation of receptor clustering
- stimulatory C-type lectin receptor signaling pathway
- viral life cycle
- viral protein processing
- virion assembly
- virion attachment to host cell

---

22

- **Protein name:** Matrix protein
- **Organism:** Borna disease virus 1
- **Uniprot Accession Number:** P0C794
- **Protein sequence length:** 142 aa
- **1D identity (%):** 2.41
- **1D identity (%) [Gaps excluded]:** 37.21
- **1D identity - Alignment Gaps:** 1243
- **Common reported functions (%):** 50.0
- **Common reported locations (%):** 12.5
- **Common reported processes (%):** 0.0

- **PDB ID:** 3F1J
- **Chain:** A
- **Crystallized protein length:** 138 aa
- **Resolution:** 2.65 Å
- **Alinged residues range:** 76-78, 74-78, 4-7, 134-136
- **Aligned to segment part (indices):** 4, 0, 3, 1
- **Alinged residues range of reference:** 200-202, 394-398, 528-531, 986-988
- **b-phipsi:** 0.005899
- **w-rdist:** 1.138861
- **t-alpha:** 0.276087
- **Chemical similarity (Tanimoto Index) (%):** 84.47
- **1D identity (%) [PDB]:** 0.09
- **1D identity (%) [Gaps excluded][PDB]:** 100.0
- **1D identity - Alignment Gaps [PDB]:** 1121
- **2D identity (%) [PDB]:** 10.23
- **2D identity (%) [Gaps excluded][PDB]:** 88.79
- **2D identity - Alignment Gaps [PDB]:** 891
- **3D similarity (TM-Score) (%) [PDB]:** 6.85

- **Gene name:** M
- **RefSeq ID:** NC\_001607
- **Genomic sequence length:** 8910
- **5-UTR|CDS|3-UTR identity (%):** N/A | 7.91 | N/A
- **5-UTR|CDS|3-UTR identity (%) [Gaps excluded]:** N/A | 77.61 | N/A
- **5-UTR|CDS|3-UTR identity [Alignment Gaps]:** N/A | 3465 | N/A

**Uniprot Description:**  
  
Plays a crucial role in virion assembly and budding.  
  
Homooligomer (By similarity). Homotetramer (PubMed:19237566). Interacts with phosphoprotein P (By similarity). Binds to ssRNA (PubMed:19237566).  
  
**Gene Ontology Information:**

Molecular Function

- identical protein binding
- structural constituent of virion

Location

- host cell cytoplasm
- host cell plasma membrane
- membrane
- virion

Biological process  
  
N/A

---

23

- **Protein name:** Spike protein
- **Organism:** Escherichia phage P2
- **Uniprot Accession Number:** P31340
- **Protein sequence length:** 211 aa
- **1D identity (%):** 3.84
- **1D identity (%) [Gaps excluded]:** 32.69
- **1D identity - Alignment Gaps:** 1172
- **Common reported functions (%):** 0.0
- **Common reported locations (%):** 0.0
- **Common reported processes (%):** 0.0

- **PDB ID:** 3QR8
- **Chain:** A
- **Crystallized protein length:** 154 aa
- **Resolution:** 2.03 Å
- **Alinged residues range:** 115-118, 21-29, 110-122
- **Aligned to segment part (indices):** 4, 0, 3
- **Alinged residues range of reference:** 198-201, 431-439, 539-549
- **b-phipsi:** 0.004837
- **w-rdist:** 1.199238
- **t-alpha:** 0.202869
- **Chemical similarity (Tanimoto Index) (%):** 84.1
- **1D identity (%) [PDB]:** 0.0
- **1D identity (%) [Gaps excluded][PDB]:** 0.0
- **1D identity - Alignment Gaps [PDB]:** 1140
- **2D identity (%) [PDB]:** 9.25
- **2D identity (%) [Gaps excluded][PDB]:** 84.07
- **2D identity - Alignment Gaps [PDB]:** 914
- **3D similarity (TM-Score) (%) [PDB]:** 6.93

- **Gene name:** V
- **RefSeq ID:** NC\_001895
- **Genomic sequence length:** 33593
- **5-UTR|CDS|3-UTR identity (%):** N/A | 11.12 | N/A
- **5-UTR|CDS|3-UTR identity (%) [Gaps excluded]:** N/A | 78.2 | N/A
- **5-UTR|CDS|3-UTR identity [Alignment Gaps]:** N/A | 3348 | N/A

**Uniprot Description:**  
  
Forms the small spikes on the baseplate that plug the end of the tube before DNA ejection and form a channel perforating the host membrane during ejection. Involved in baseplate assembly.  
  
homotrimer.  
  
**Gene Ontology Information:**

Molecular Function

- metal ion binding

Location

- virus tail, baseplate

Biological process

- entry receptor-mediated virion attachment to host cell
- pore-mediated entry of viral genome into host cell
- viral tail assembly

---

24

- **Protein name:** Bcl-2
- **Organism:** Human herpesvirus 8
- **Uniprot Accession Number:** Q76RI8
- **Protein sequence length:** 175 aa
- **1D identity (%):** 3.48
- **1D identity (%) [Gaps excluded]:** 29.03
- **1D identity - Alignment Gaps:** 1138
- **Common reported functions (%):** 0.0
- **Common reported locations (%):** 12.5
- **Common reported processes (%):** 0.0

- **PDB ID:** 1K3K
- **Chain:** A
- **Crystallized protein length:** 146 aa
- **Resolution:** -1.0 Å
- **Alinged residues range:** 82-92, 2-6, 83-87
- **Aligned to segment part (indices):** 0, 3, 1
- **Alinged residues range of reference:** 363-369, 556-560, 985-989
- **b-phipsi:** 0.174935
- **w-rdist:** 0.851146
- **t-alpha:** 0.103918
- **Chemical similarity (Tanimoto Index) (%):** 83.32
- **1D identity (%) [PDB]:** 0.27
- **1D identity (%) [Gaps excluded][PDB]:** 100.0
- **1D identity - Alignment Gaps [PDB]:** 1123
- **2D identity (%) [PDB]:** 11.37
- **2D identity (%) [Gaps excluded][PDB]:** 90.48
- **2D identity - Alignment Gaps [PDB]:** 877
- **3D similarity (TM-Score) (%) [PDB]:** 7.17

- **Gene name:** ORF16
- **RefSeq ID:** NC\_009333
- **Genomic sequence length:** 137969
- **5-UTR|CDS|3-UTR identity (%):** N/A | 9.25 | N/A
- **5-UTR|CDS|3-UTR identity (%) [Gaps excluded]:** N/A | 78.6 | N/A
- **5-UTR|CDS|3-UTR identity [Alignment Gaps]:** N/A | 3434 | N/A

**Uniprot Description:**  
  
N/A  
  
**Gene Ontology Information:**

Molecular Function  
  
N/A

Location

- integral component of membrane

Biological process

- negative regulation by symbiont of host apoptotic process

---

25

- **Protein name:** RecBCD enzyme subunit RecD
- **Organism:** Escherichia coli (strain K12)
- **Uniprot Accession Number:** P04993
- **Protein sequence length:** 608 aa
- **1D identity (%):** 10.15
- **1D identity (%) [Gaps excluded]:** 27.15
- **1D identity - Alignment Gaps:** 857
- **Common reported functions (%):** 0.0
- **Common reported locations (%):** 0.0
- **Common reported processes (%):** 0.0

- **PDB ID:** 5MBV
- **Chain:** D
- **Crystallized protein length:** 593 aa
- **Resolution:** 3.8 Å
- **Alinged residues range:** 287-294, 291-296, 179-183
- **Aligned to segment part (indices):** 0, 3, 1
- **Alinged residues range of reference:** 426-435, 509-514, 986-990
- **b-phipsi:** 0.115822
- **w-rdist:** 1.004479
- **t-alpha:** 0.056218
- **Chemical similarity (Tanimoto Index) (%):** 83.45
- **1D identity (%) [PDB]:** 0.0
- **1D identity (%) [Gaps excluded][PDB]:** 0.0
- **1D identity - Alignment Gaps [PDB]:** 1578
- **2D identity (%) [PDB]:** 21.46
- **2D identity (%) [Gaps excluded][PDB]:** 86.03
- **2D identity - Alignment Gaps [PDB]:** 948
- **3D similarity (TM-Score) (%) [PDB]:** 5.27

- **Gene name:** recD
- **RefSeq ID:** N/A
- **Sequence length:** N/A
- **5-UTR|CDS|3-UTR identity (%):** N/A | N/A | N/A
- **5-UTR|CDS|3-UTR identity (%) [Gaps excluded]:** N/A | N/A | N/A
- **5-UTR|CDS|3-UTR identity [Alignment Gaps]:** N/A | N/A | N/A

**Uniprot Description:**  
  
A helicase/nuclease that prepares dsDNA breaks (DSB) for recombinational DNA repair. Binds to DSBs and unwinds DNA via a rapid (>1 kb/second) and highly processive (>30 kb) ATP-dependent bidirectional helicase. Unwinds dsDNA until it encounters a Chi (crossover hotspot instigator, 5'-GCTGGTGG-3') sequence from the 3' direction. Cuts ssDNA a few nucleotides 3' to Chi site, by nicking one strand or switching the strand degraded (depending on the reaction conditions). The properties and activities of the enzyme are changed at Chi. The Chi-altered holoenzyme produces a long 3'-ssDNA overhang which facilitates RecA-binding to the ssDNA for homologous DNA recombination and repair. In the holoenzyme this subunit contributes ssDNA-dependent ATPase and fast 5'-3' helicase activity. When added to pre-assembled RecBC greatly stimulates nuclease activity and augments holoenzyme processivity. Negatively regulates the RecA-loading ability of RecBCD.  
  
Heterotrimer of RecB, RecC and RecD. All subunits contribute to DNA-binding.  
  
**Gene Ontology Information:**

Molecular Function

- 5'-3' DNA helicase activity
- ATP binding
- DNA binding
- exodeoxyribonuclease V activity
- helicase activity
- single-stranded DNA helicase activity

Location

- exodeoxyribonuclease V complex

Biological process

- cellular response to DNA damage stimulus
- DNA recombination
- double-strand break repair via homologous recombination

---

26

- **Protein name:** Tail completion protein gp17
- **Organism:** Bacillus phage SPP1
- **Uniprot Accession Number:** O48448
- **Protein sequence length:** 134 aa
- **1D identity (%):** 2.63
- **1D identity (%) [Gaps excluded]:** 29.57
- **1D identity - Alignment Gaps:** 1177
- **Common reported functions (%):** 0.0
- **Common reported locations (%):** 0.0
- **Common reported processes (%):** 0.0

- **PDB ID:** 5A20
- **Chain:** G
- **Crystallized protein length:** 133 aa
- **Resolution:** 7.6 Å
- **Alinged residues range:** 65-67, 64-67, 62-68, 12-16
- **Aligned to segment part (indices):** 4, 0, 3, 1
- **Alinged residues range of reference:** 200-202, 395-398, 537-543, 988-992
- **b-phipsi:** 0.022441
- **w-rdist:** 1.104536
- **t-alpha:** 0.55291
- **Chemical similarity (Tanimoto Index) (%):** N/A
- **1D identity (%) [PDB]:** 0.0
- **1D identity (%) [Gaps excluded][PDB]:** 0.0
- **1D identity - Alignment Gaps [PDB]:** 1116
- **2D identity (%) [PDB]:** 6.77
- **2D identity (%) [Gaps excluded][PDB]:** 85.37
- **2D identity - Alignment Gaps [PDB]:** 952
- **3D similarity (TM-Score) (%) [PDB]:** 13.39

- **Gene name:** N/A
- **RefSeq ID:** NC\_004166
- **Genomic sequence length:** 44010
- **5-UTR|CDS|3-UTR identity (%):** N/A | 7.39 | N/A
- **5-UTR|CDS|3-UTR identity (%) [Gaps excluded]:** N/A | 77.03 | N/A
- **5-UTR|CDS|3-UTR identity [Alignment Gaps]:** N/A | 3487 | N/A

**Uniprot Description:**  
  
Tail completion protein that caps the tail and interacts with the connector gp16, thereby attaching the tail to the capsid.  
  
Homohexamer (PubMed:25991862, PubMed:22072538). Interacts with gp16 connector protein (PubMed:22072538, PubMed:25991862).  
  
**Gene Ontology Information:**

Molecular Function  
  
N/A

Location

- virus tail

Biological process

- virus tail fiber assembly

---

27

- **Protein name:** Putative poly(A) polymerase catalytic subunit
- **Organism:** Acanthamoeba polyphaga mimivirus
- **Uniprot Accession Number:** Q5UQS6
- **Protein sequence length:** 584 aa
- **1D identity (%):** 9.59
- **1D identity (%) [Gaps excluded]:** 28.3
- **1D identity - Alignment Gaps:** 917
- **Common reported functions (%):** 0.0
- **Common reported locations (%):** 0.0
- **Common reported processes (%):** 0.0

- **PDB ID:** 4WSE
- **Chain:** B
- **Crystallized protein length:** 495 aa
- **Resolution:** 2.84 Å
- **Alinged residues range:** 141-143, 370-374, 130-143, 33-37
- **Aligned to segment part (indices):** 4, 0, 3, 1
- **Alinged residues range of reference:** 200-202, 447-451, 506-516, 988-992
- **b-phipsi:** 0.042328
- **w-rdist:** 1.025224
- **t-alpha:** 0.374707
- **Chemical similarity (Tanimoto Index) (%):** 85.75
- **1D identity (%) [PDB]:** 0.0
- **1D identity (%) [Gaps excluded][PDB]:** 0.0
- **1D identity - Alignment Gaps [PDB]:** 1480
- **2D identity (%) [PDB]:** 18.95
- **2D identity (%) [Gaps excluded][PDB]:** 90.62
- **2D identity - Alignment Gaps [PDB]:** 968
- **3D similarity (TM-Score) (%) [PDB]:** 14.83

- **Gene name:** MIMI\_R341
- **RefSeq ID:** NC\_014649
- **Genomic sequence length:** 1181549
- **5-UTR|CDS|3-UTR identity (%):** 8.15 | 32.86 | N/A
- **5-UTR|CDS|3-UTR identity (%) [Gaps excluded]:** 84.62 | 80.41 | N/A
- **5-UTR|CDS|3-UTR identity [Alignment Gaps]:** 244 | 2341 | N/A

**Uniprot Description:**  
  
Polymerase that creates the 3'-poly(A) tail of mRNA's.  
  
**Gene Ontology Information:**

Molecular Function

- ATP binding
- polynucleotide adenylyltransferase activity

Location

- virion

Biological process

- mRNA processing

---

28

- **Protein name:** mRNA-capping enzyme
- **Organism:** Paramecium bursaria Chlorella virus 1
- **Uniprot Accession Number:** Q84424
- **Protein sequence length:** 330 aa
- **1D identity (%):** 4.32
- **1D identity (%) [Gaps excluded]:** 32.11
- **1D identity - Alignment Gaps:** 1223
- **Common reported functions (%):** 0.0
- **Common reported locations (%):** 0.0
- **Common reported processes (%):** 0.0

- **PDB ID:** 1CKM
- **Chain:** B
- **Crystallized protein length:** 317 aa
- **Resolution:** 2.5 Å
- **Alinged residues range:** 286-290, 214-219, 280-294, 313-315
- **Aligned to segment part (indices):** 4, 0, 3, 1
- **Alinged residues range of reference:** 198-202, 431-436, 539-553, 990-992
- **b-phipsi:** 0.042691
- **w-rdist:** 1.055957
- **t-alpha:** 0.251599
- **Chemical similarity (Tanimoto Index) (%):** 84.63
- **1D identity (%) [PDB]:** 0.0
- **1D identity (%) [Gaps excluded][PDB]:** 0.0
- **1D identity - Alignment Gaps [PDB]:** 1300
- **2D identity (%) [PDB]:** 20.09
- **2D identity (%) [Gaps excluded][PDB]:** 88.75
- **2D identity - Alignment Gaps [PDB]:** 820
- **3D similarity (TM-Score) (%) [PDB]:** 12.48

- **Gene name:** A103R
- **RefSeq ID:** NC\_000852
- **Genomic sequence length:** 330611
- **5-UTR|CDS|3-UTR identity (%):** N/A | 16.37 | N/A
- **5-UTR|CDS|3-UTR identity (%) [Gaps excluded]:** N/A | 78.96 | N/A
- **5-UTR|CDS|3-UTR identity [Alignment Gaps]:** N/A | 3161 | N/A

**Uniprot Description:**  
  
mRNA capping. Transfers a GMP cap onto the end of mRNA that terminates with a 5'-diphosphate tail.  
  
Monomer.  
  
**Gene Ontology Information:**

Molecular Function

- GTP binding
- mRNA guanylyltransferase activity

Location  
  
N/A

Biological process

- 7-methylguanosine mRNA capping

---

29

- **Protein name:** NTPase P4
- **Organism:** Pseudomonas phage phi12
- **Uniprot Accession Number:** Q94M05
- **Protein sequence length:** 331 aa
- **1D identity (%):** 5.93
- **1D identity (%) [Gaps excluded]:** 29.04
- **1D identity - Alignment Gaps:** 1060
- **Common reported functions (%):** 0.0
- **Common reported locations (%):** 0.0
- **Common reported processes (%):** 0.0

- **PDB ID:** 4BLS
- **Chain:** B
- **Crystallized protein length:** 303 aa
- **Resolution:** 2.6 Å
- **Alinged residues range:** 88-92, 119-127, 236-239, 236-242
- **Aligned to segment part (indices):** 4, 0, 3, 1
- **Alinged residues range of reference:** 198-202, 394-402, 526-529, 974-980
- **b-phipsi:** 0.03003
- **w-rdist:** 1.266004
- **t-alpha:** 0.124521
- **Chemical similarity (Tanimoto Index) (%):** 83.9
- **1D identity (%) [PDB]:** 0.0
- **1D identity (%) [Gaps excluded][PDB]:** 0.0
- **1D identity - Alignment Gaps [PDB]:** 1288
- **2D identity (%) [PDB]:** 16.16
- **2D identity (%) [Gaps excluded][PDB]:** 88.44
- **2D identity - Alignment Gaps [PDB]:** 890
- **3D similarity (TM-Score) (%) [PDB]:** 11.1

- **Gene name:** 4
- **RefSeq ID:** NC\_004173
- **Genomic sequence length:** 6751
- **5-UTR|CDS|3-UTR identity (%):** N/A | 16.45 | N/A
- **5-UTR|CDS|3-UTR identity (%) [Gaps excluded]:** N/A | 77.58 | N/A
- **5-UTR|CDS|3-UTR identity [Alignment Gaps]:** N/A | 3132 | N/A

**Uniprot Description:**  
  
N/A  
  
**Gene Ontology Information:**

Molecular Function

- ATP binding

Location  
  
N/A

Biological process

- viral genome packaging

---

30

- **Protein name:** 40S ribosomal protein S27
- **Organism:** Oryctolagus cuniculus
- **Uniprot Accession Number:** G1TZ76
- **Protein sequence length:** 84 aa
- **1D identity (%):** 1.56
- **1D identity (%) [Gaps excluded]:** 27.03
- **1D identity - Alignment Gaps:** 1209
- **Common reported functions (%):** 0.0
- **Common reported locations (%):** 0.0
- **Common reported processes (%):** 0.0

- **PDB ID:** 7JQB
- **Chain:** E
- **Crystallized protein length:** 228 aa
- **Resolution:** 2.7 Å
- **Alinged residues range:** 133-135, 130-135, 213-218, 18-22
- **Aligned to segment part (indices):** 4, 0, 3, 1
- **Alinged residues range of reference:** 200-202, 373-378, 555-560, 988-992
- **b-phipsi:** 0.043969
- **w-rdist:** 1.174421
- **t-alpha:** 0.478589
- **Chemical similarity (Tanimoto Index) (%):** 83.32
- **1D identity (%) [PDB]:** 0.0
- **1D identity (%) [Gaps excluded][PDB]:** 0.0
- **1D identity - Alignment Gaps [PDB]:** 1211
- **2D identity (%) [PDB]:** 12.25
- **2D identity (%) [Gaps excluded][PDB]:** 86.67
- **2D identity - Alignment Gaps [PDB]:** 911
- **3D similarity (TM-Score) (%) [PDB]:** 10.18

- **Gene name:** N/A
- **RefSeq ID:** N/A
- **Sequence length:** N/A
- **5-UTR|CDS|3-UTR identity (%):** N/A | N/A | N/A
- **5-UTR|CDS|3-UTR identity (%) [Gaps excluded]:** N/A | N/A | N/A
- **5-UTR|CDS|3-UTR identity [Alignment Gaps]:** N/A | N/A | N/A

**Uniprot Description:**  
  
N/A  
  
**Gene Ontology Information:**

Molecular Function  
  
N/A

Location  
  
N/A

Biological process  
  
N/A

---

31

- **Protein name:** Nucleoprotein
- **Organism:** Severe acute respiratory syndrome coronavirus
- **Uniprot Accession Number:** P59595
- **Protein sequence length:** 422 aa
- **1D identity (%):** 6.48
- **1D identity (%) [Gaps excluded]:** 27.64
- **1D identity - Alignment Gaps:** 1051
- **Common reported functions (%):** 50.0
- **Common reported locations (%):** 0.0
- **Common reported processes (%):** 20.0

- **PDB ID:** 2CJR
- **Chain:** B
- **Crystallized protein length:** 113 aa
- **Resolution:** 2.5 Å
- **Alinged residues range:** 322-324, 333-338, 321-331, 270-274
- **Aligned to segment part (indices):** 4, 0, 3, 1
- **Alinged residues range of reference:** 200-202, 396-401, 539-549, 985-989
- **b-phipsi:** 0.047474
- **w-rdist:** 1.226853
- **t-alpha:** 0.411058
- **Chemical similarity (Tanimoto Index) (%):** 82.45
- **1D identity (%) [PDB]:** 0.0
- **1D identity (%) [Gaps excluded][PDB]:** 0.0
- **1D identity - Alignment Gaps [PDB]:** 1096
- **2D identity (%) [PDB]:** 8.49
- **2D identity (%) [Gaps excluded][PDB]:** 89.47
- **2D identity - Alignment Gaps [PDB]:** 906
- **3D similarity (TM-Score) (%) [PDB]:** 5.75

- **Gene name:** N
- **RefSeq ID:** NC\_004718
- **Genomic sequence length:** 29751
- **5-UTR|CDS|3-UTR identity (%):** 88.52 | 4.33 | 22.38
- **5-UTR|CDS|3-UTR identity (%) [Gaps excluded]:** 92.28 | 82.18 | 98.18
- **5-UTR|CDS|3-UTR identity [Alignment Gaps]:** 11 | 3631 | 745

**Uniprot Description:**  
  
Packages the positive strand viral genome RNA into a helical ribonucleocapsid (RNP) and plays a fundamental role during virion assembly through its interactions with the viral genome and membrane protein M. Plays an important role in enhancing the efficiency of subgenomic viral RNA transcription as well as viral replication (PubMed:17210170). May modulate transforming growth factor-beta signaling by binding host SMAD3 (PubMed:18055455).  
  
Homooligomer. Both monomeric and oligomeric forms interact with RNA. Interacts with protein M (PubMed:15351485). Interacts with protein E (PubMed:24766657). May bind to host HNRNPA1 (Probable). Interacts with NSP3; this interaction serves to tether the genome to the newly translated replicase-transcriptase complex at a very early stage of infection (By similarity). May interact with host SMAD3 (Probable). Interacts with host PPIA/CYPA (PubMed:15688292).  
  
**Gene Ontology Information:**

Molecular Function

- identical protein binding
- RNA binding

Location

- host cell endoplasmic reticulum-Golgi intermediate compartment
- host cell Golgi apparatus
- host cell perinuclear region of cytoplasm
- viral capsid
- viral nucleocapsid

Biological process

- viral protein processing
- viral translation

---

32

- **Protein name:** Pre-glycoprotein polyprotein GP complex
- **Organism:** Lassa virus (strain Mouse/Sierra Leone/Josiah/1976)
- **Uniprot Accession Number:** P08669
- **Protein sequence length:** 491 aa
- **1D identity (%):** 1.42
- **1D identity (%) [Gaps excluded]:** 30.38
- **1D identity - Alignment Gaps:** 1606
- **Common reported functions (%):** 0.0
- **Common reported locations (%):** 50.0
- **Common reported processes (%):** 10.0

- **PDB ID:** 5VK2
- **Chain:** a
- **Crystallized protein length:** 155 aa
- **Resolution:** 3.2 Å
- **Alinged residues range:** 371-373, 368-372, 369-371, 317-321
- **Aligned to segment part (indices):** 4, 0, 3, 1
- **Alinged residues range of reference:** 200-202, 448-452, 548-550, 988-992
- **b-phipsi:** 0.107051
- **w-rdist:** 1.20605
- **t-alpha:** 0.501279
- **Chemical similarity (Tanimoto Index) (%):** 94.32
- **1D identity (%) [PDB]:** 0.0
- **1D identity (%) [Gaps excluded][PDB]:** 0.0
- **1D identity - Alignment Gaps [PDB]:** 1139
- **2D identity (%) [PDB]:** 9.14
- **2D identity (%) [Gaps excluded][PDB]:** 85.45
- **2D identity - Alignment Gaps [PDB]:** 919
- **3D similarity (TM-Score) (%) [PDB]:** 10.27

- **Gene name:** GPC
- **RefSeq ID:** NC\_004296
- **Genomic sequence length:** 3402
- **5-UTR|CDS|3-UTR identity (%):** N/A | 25.44 | N/A
- **5-UTR|CDS|3-UTR identity (%) [Gaps excluded]:** N/A | 81.11 | N/A
- **5-UTR|CDS|3-UTR identity [Alignment Gaps]:** N/A | 2768 | N/A

**Uniprot Description:**  
  
Glycoprotein G1
interacts with the host receptor (By similarity). Mediates virus attachment to host receptor alpha-dystroglycan DAG1. This attachment induces virion internalization predominantly through clathrin- and caveolin-independent endocytosis (PubMed:11967329).  
  
Glycoprotein G1
homotetramer; disulfide-linked (By similarity). Interacts with host DAG1 (PubMed:11967329).  
  
**Gene Ontology Information:**

Molecular Function

- metal ion binding

Location

- host cell endoplasmic reticulum membrane
- host cell Golgi membrane
- host cell plasma membrane
- integral component of membrane
- viral envelope
- virion membrane

Biological process

- fusion of virus membrane with host endosome membrane
- receptor-mediated endocytosis of virus by host cell
- virion attachment to host cell

---
