# Supplementary figures and images for: Identifying and profiling structural similarities between Spike of SARS-CoV-2 and other viral or host proteins with Machaon (part 2 of 2)
Source: Commun Biol. 2023 Jul 19;6:752. doi: 10.1038/s42003-023-05076-7 (PMC10356814; doi:10.1038/s42003-023-05076-7)

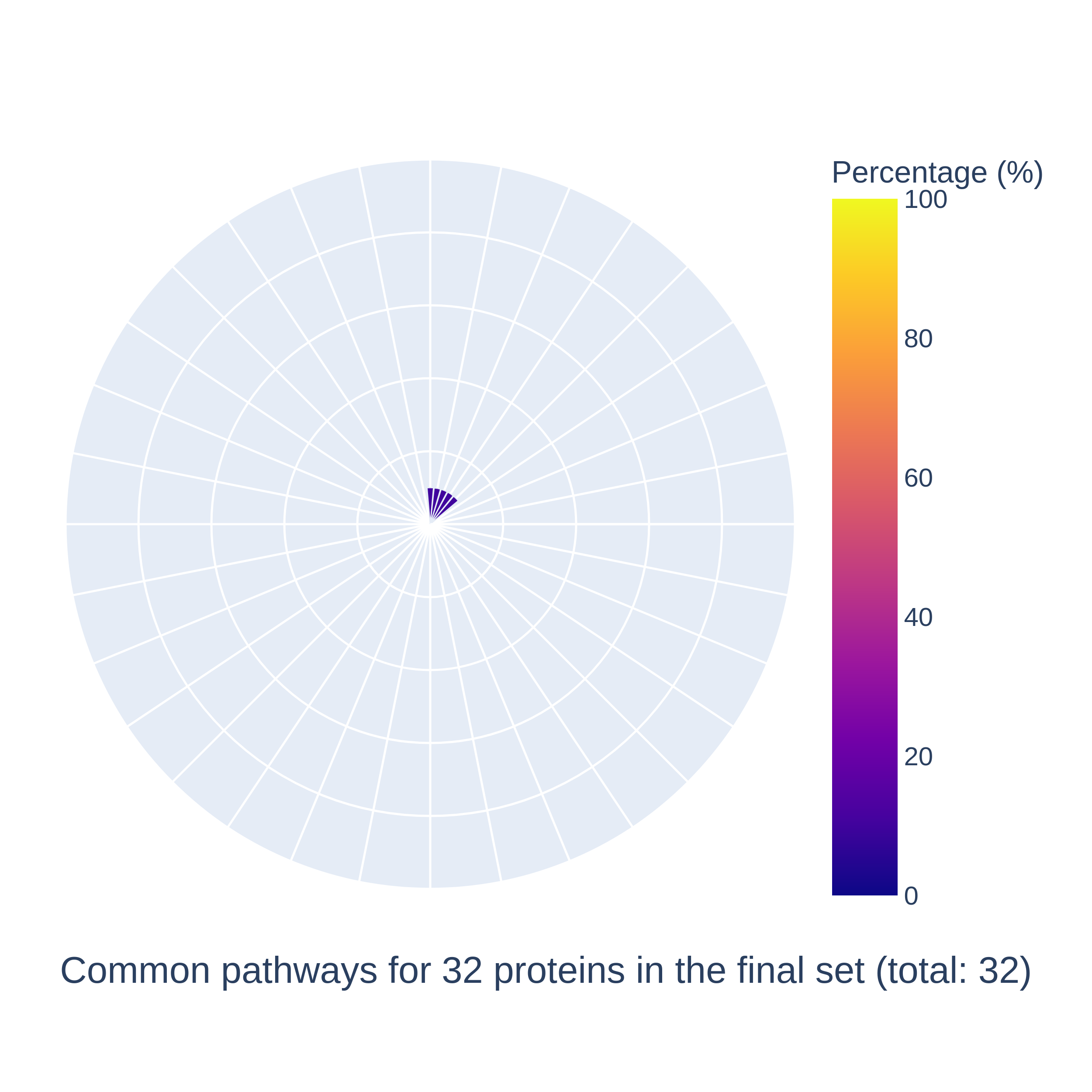

Supplement: Supplementary file 7 — Supplementary Data 4 [file 42003_2023_5076_MOESM7_ESM.zip › 6VXX_A_domain/plots/6VXX_A_BetaCoV-S1-NTD_biologicalProcessSim.png]

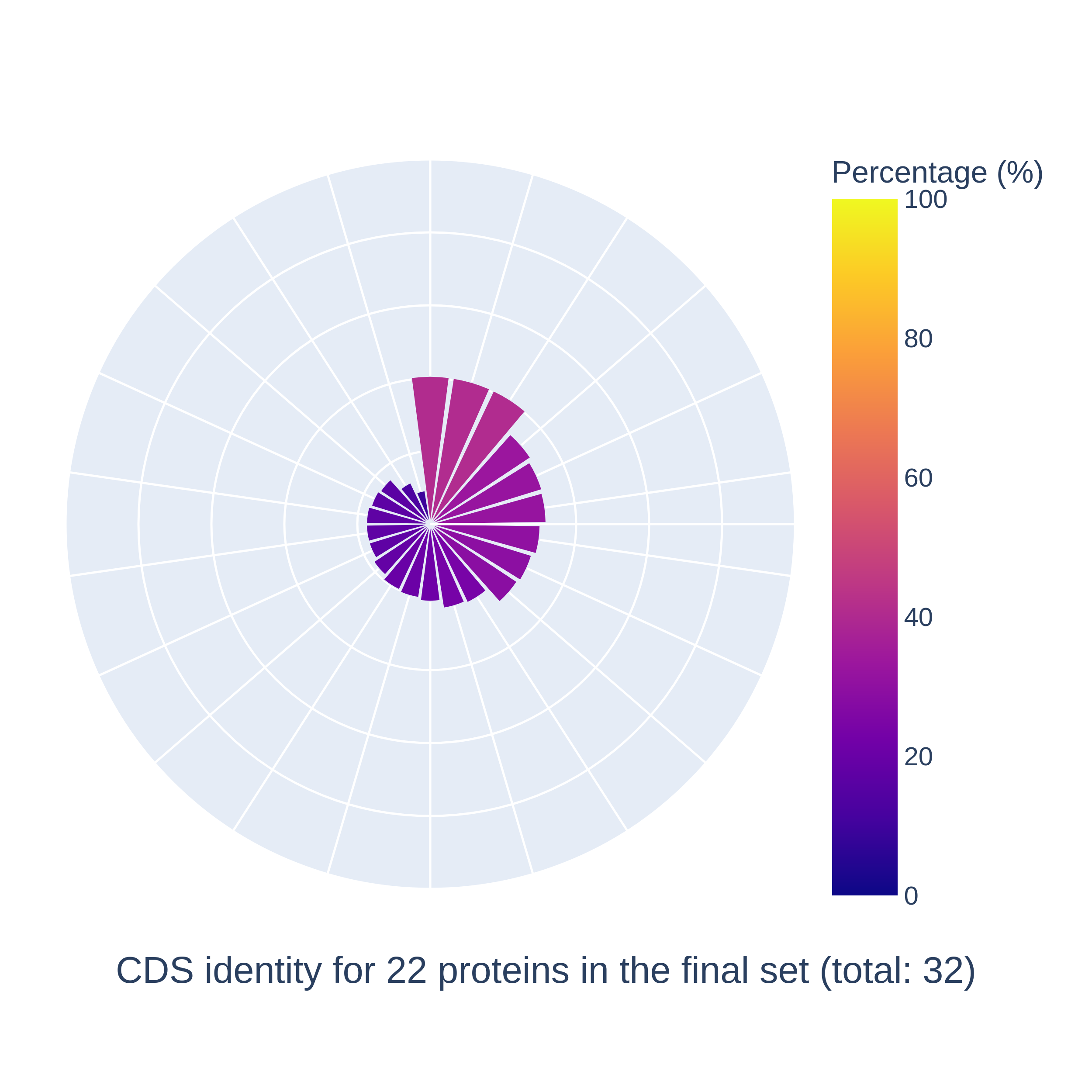

Supplement: Supplementary file 7 — Supplementary Data 4 [file 42003_2023_5076_MOESM7_ESM.zip › 6VXX_A_domain/plots/6VXX_A_BetaCoV-S1-NTD_CDS-identity.png]

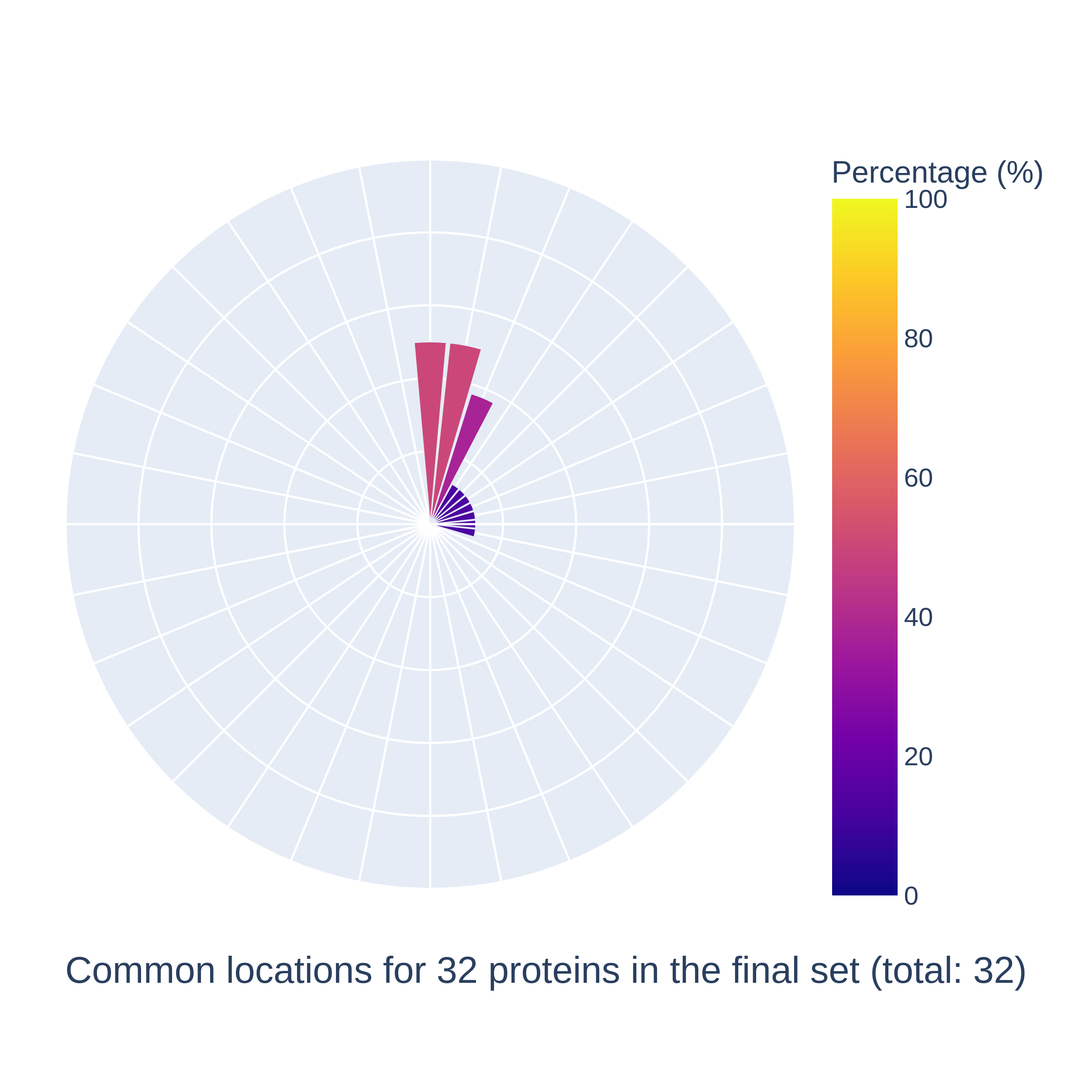

Supplement: Supplementary file 7 — Supplementary Data 4 [file 42003_2023_5076_MOESM7_ESM.zip › 6VXX_A_domain/plots/6VXX_A_BetaCoV-S1-NTD_cellularComponentSim.png]

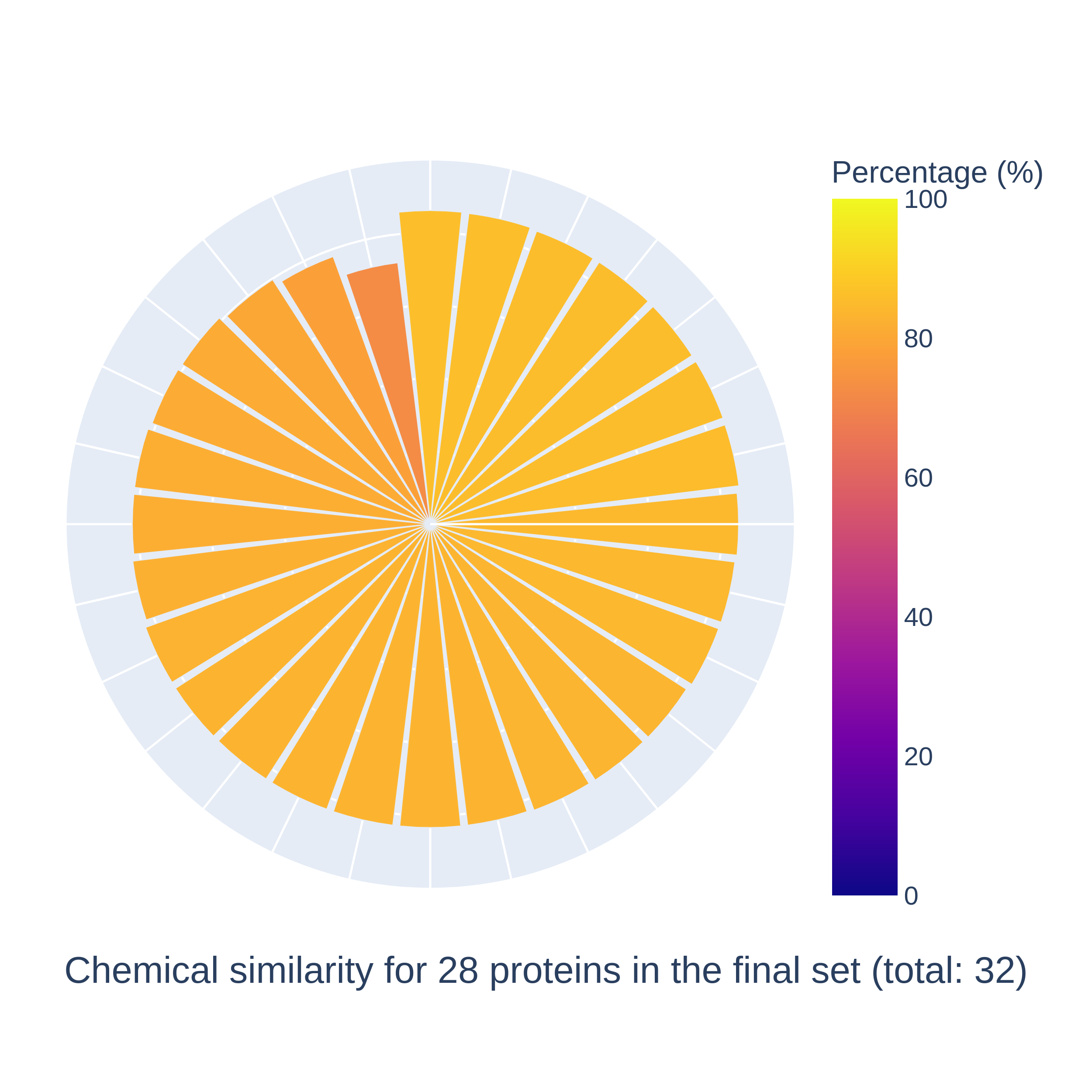

Supplement: Supplementary file 7 — Supplementary Data 4 [file 42003_2023_5076_MOESM7_ESM.zip › 6VXX_A_domain/plots/6VXX_A_BetaCoV-S1-NTD_chemSim.png]

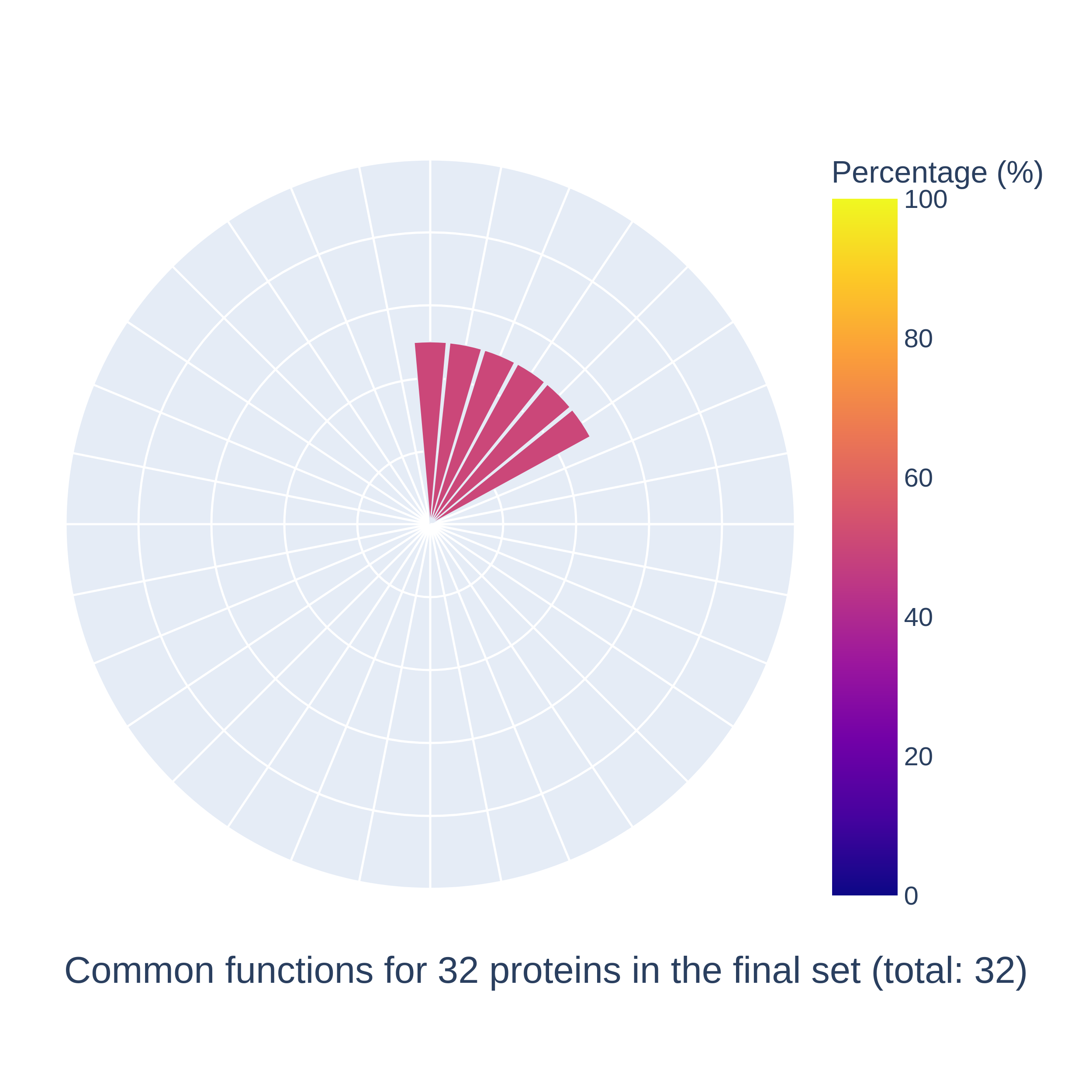

Supplement: Supplementary file 7 — Supplementary Data 4 [file 42003_2023_5076_MOESM7_ESM.zip › 6VXX_A_domain/plots/6VXX_A_BetaCoV-S1-NTD_molecularFunctionSim.png]

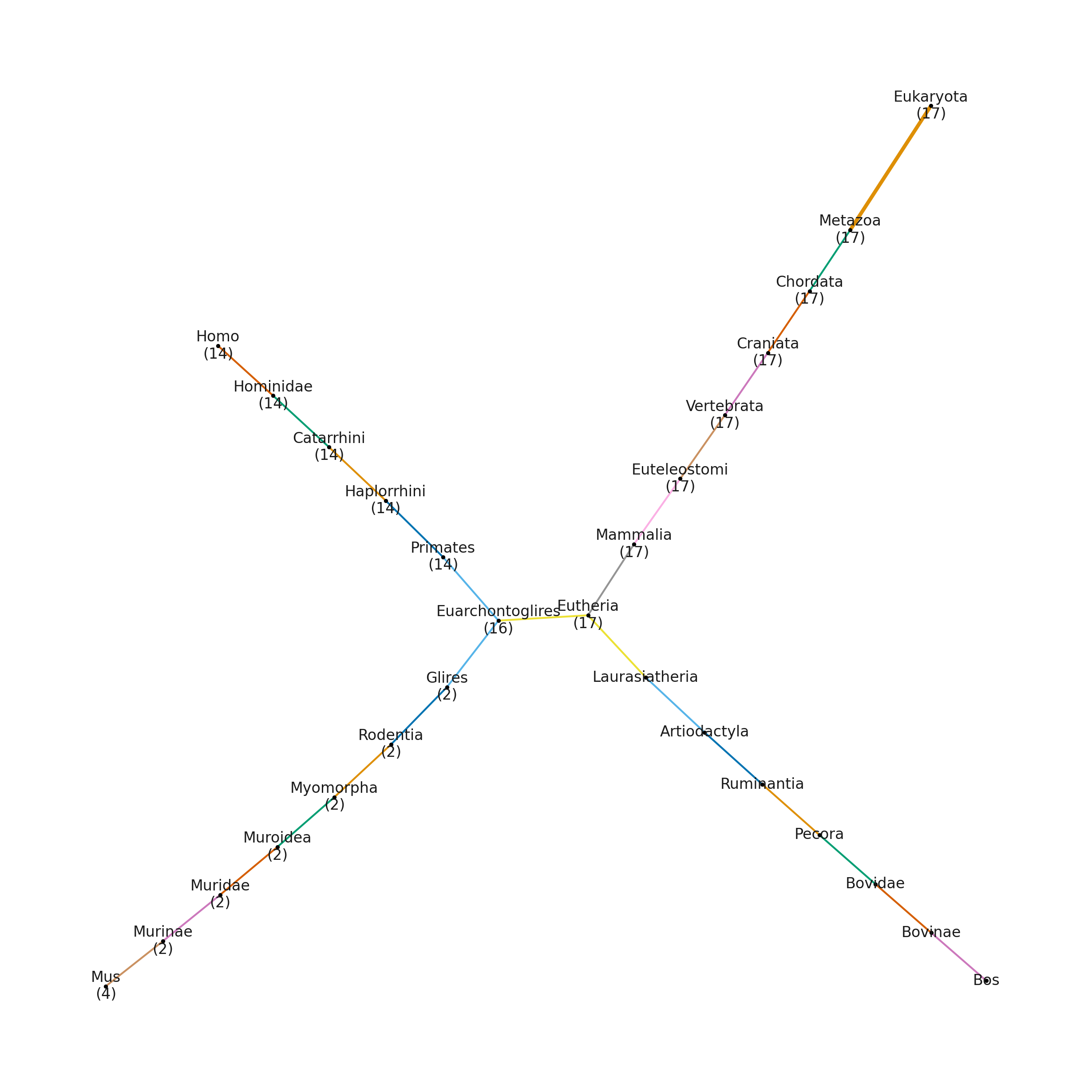

Supplement: Supplementary file 7 — Supplementary Data 4 [file 42003_2023_5076_MOESM7_ESM.zip › 6VXX_A_domain/plots/6VXX_A_RBD-Eukaryota-tree.png]

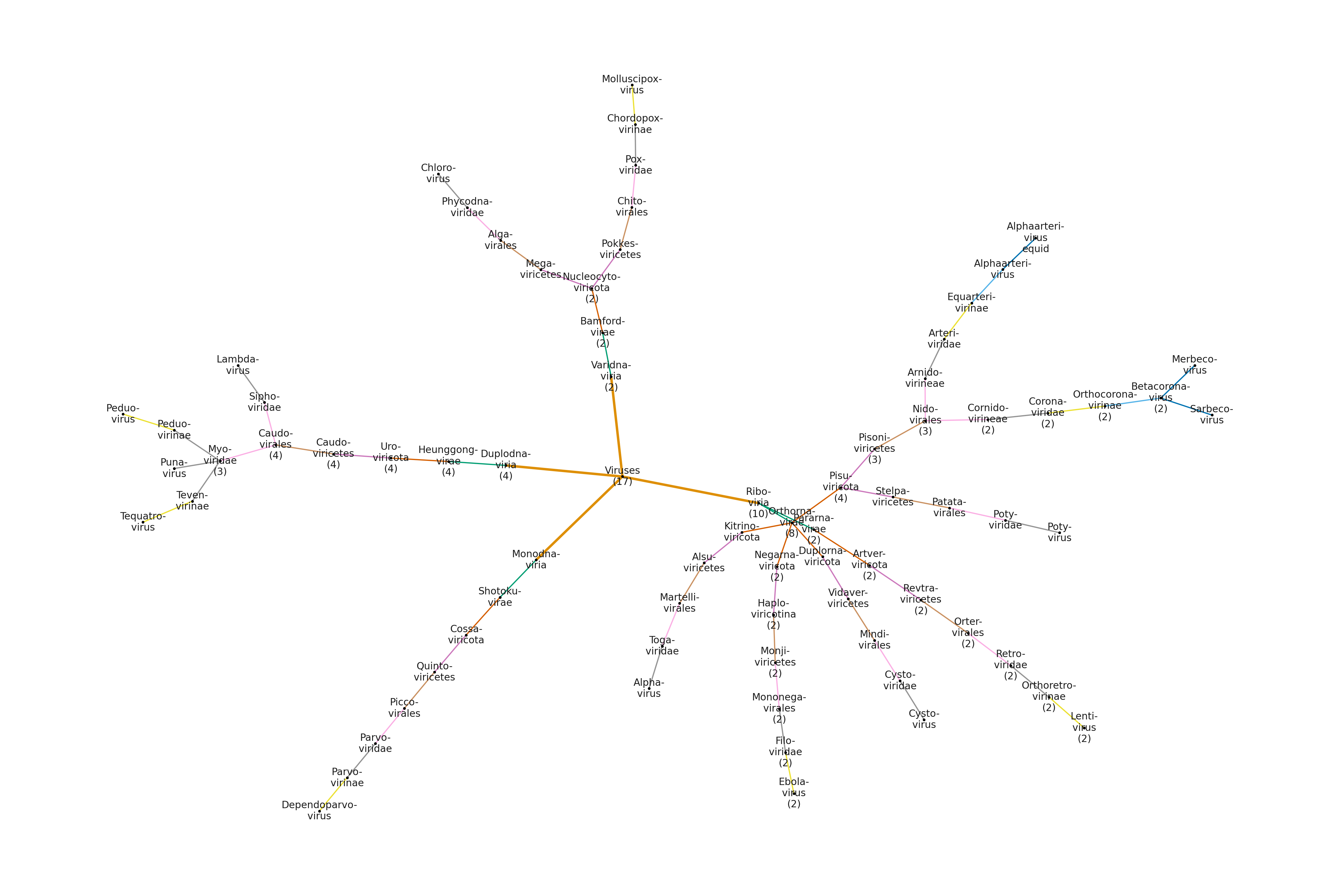

Supplement: Supplementary file 7 — Supplementary Data 4 [file 42003_2023_5076_MOESM7_ESM.zip › 6VXX_A_domain/plots/6VXX_A_RBD-Viruses-tree.png]

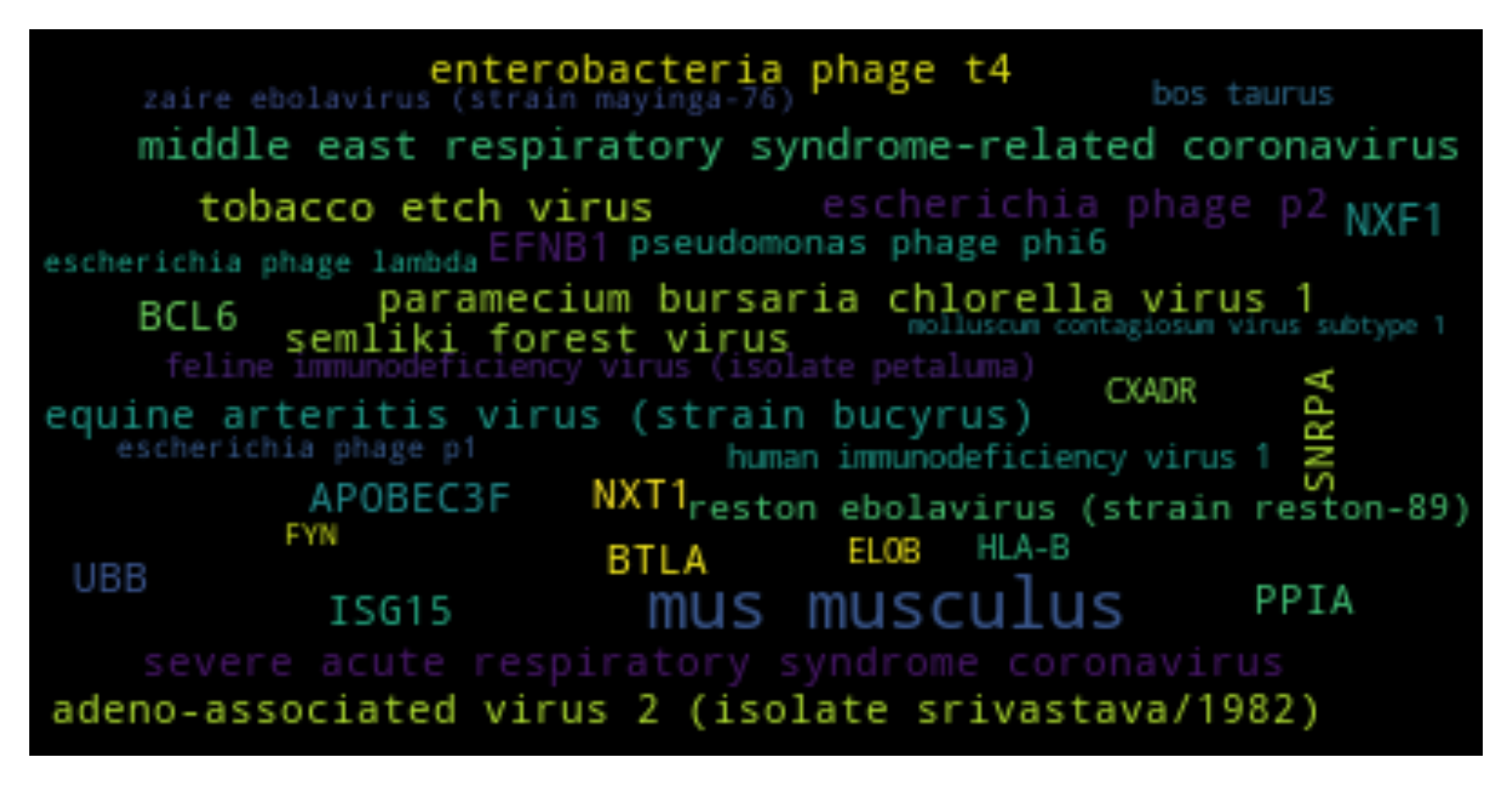

Supplement: Supplementary file 7 — Supplementary Data 4 [file 42003_2023_5076_MOESM7_ESM.zip › 6VXX_A_domain/plots/6VXX_A_RBD-wordcloud.png]

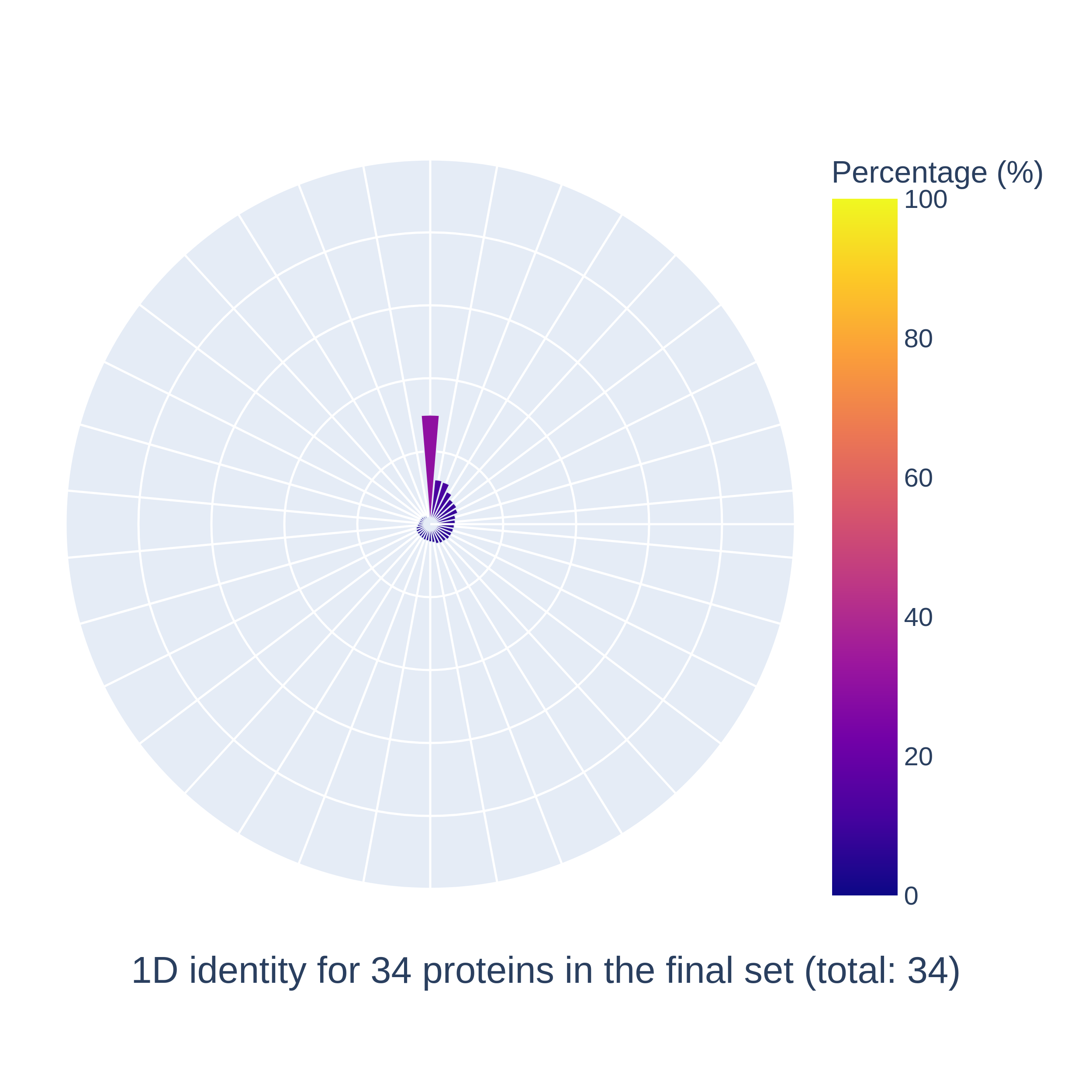

Supplement: Supplementary file 7 — Supplementary Data 4 [file 42003_2023_5076_MOESM7_ESM.zip › 6VXX_A_domain/plots/6VXX_A_RBD_1D-identity.png]

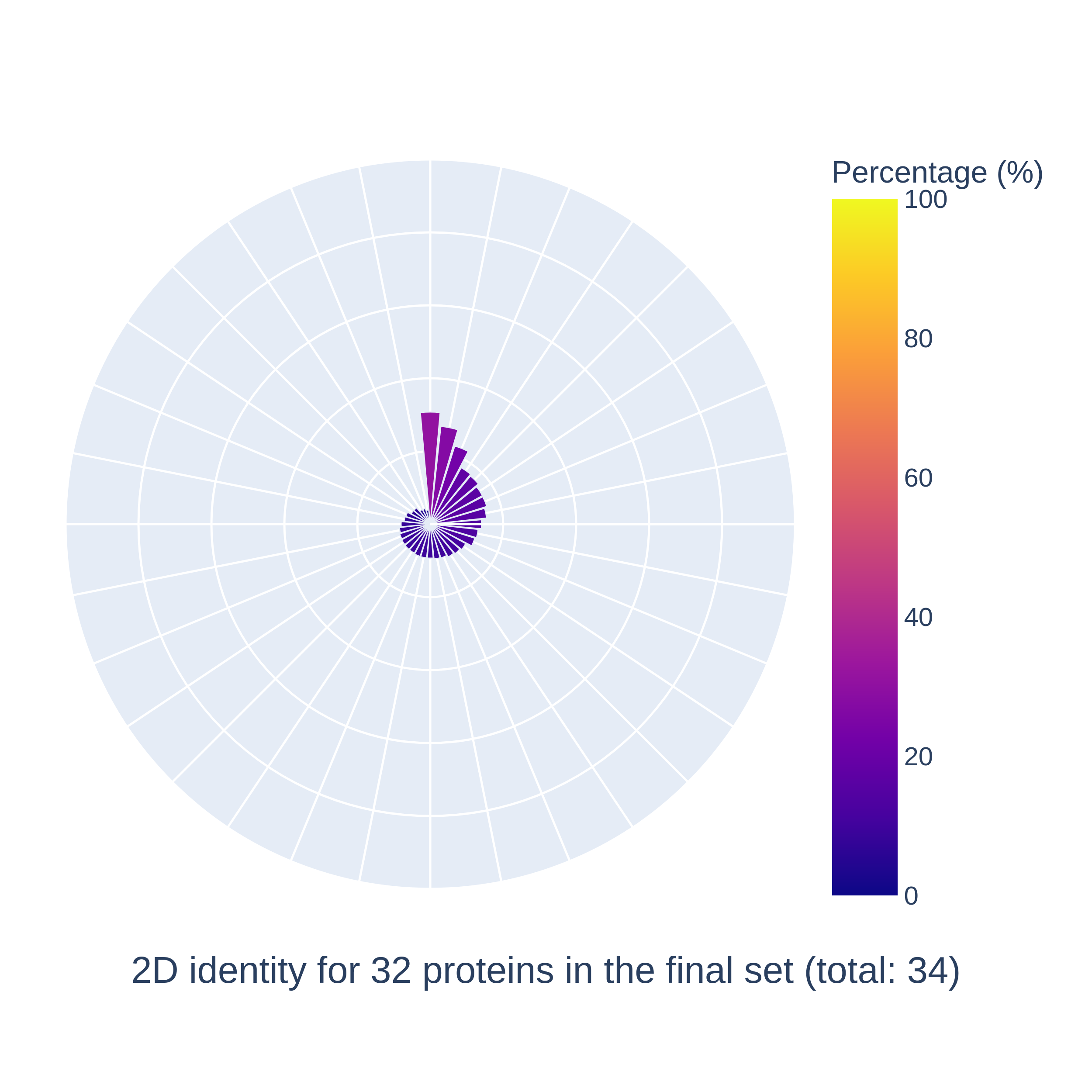

Supplement: Supplementary file 7 — Supplementary Data 4 [file 42003_2023_5076_MOESM7_ESM.zip › 6VXX_A_domain/plots/6VXX_A_RBD_2D-identity.png]

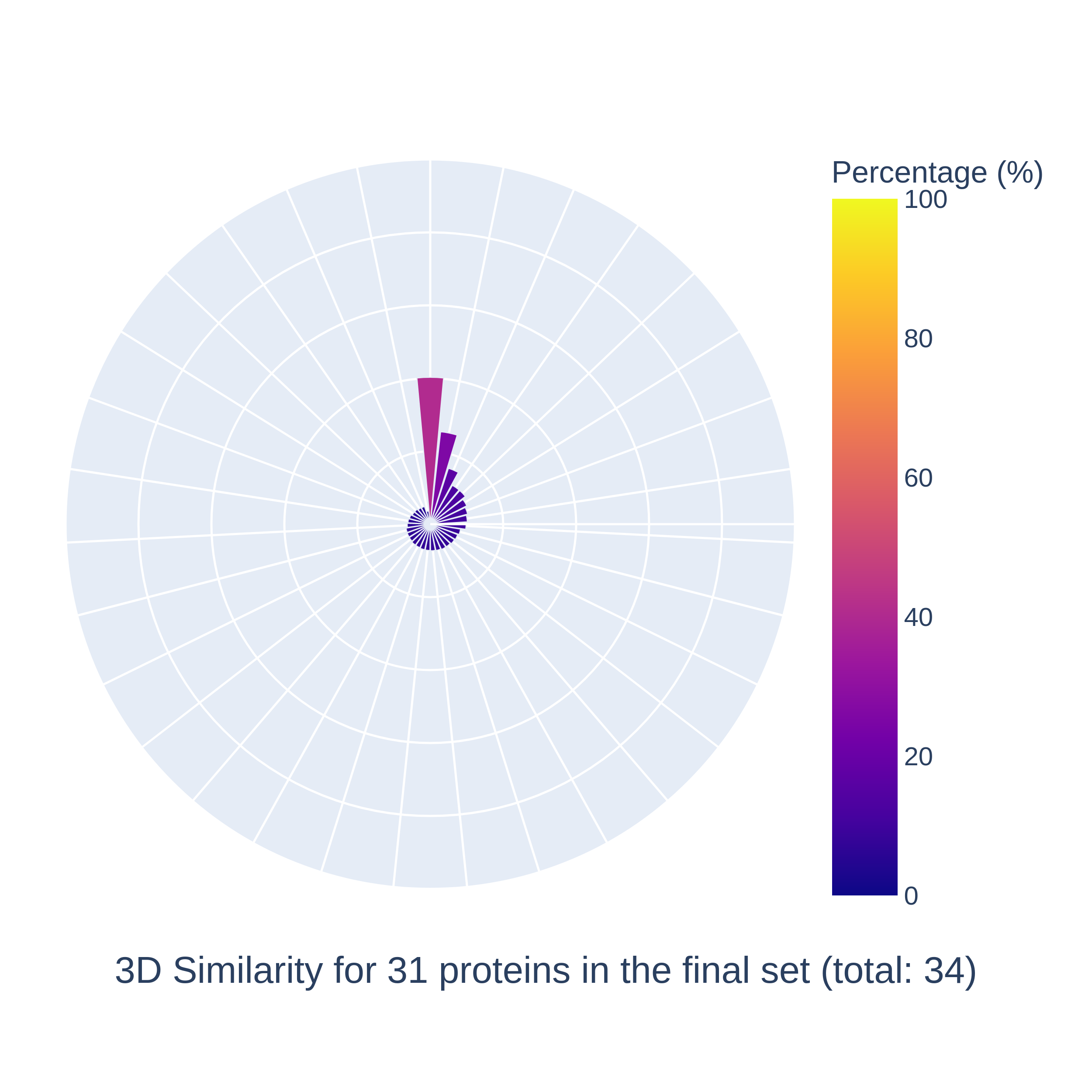

Supplement: Supplementary file 7 — Supplementary Data 4 [file 42003_2023_5076_MOESM7_ESM.zip › 6VXX_A_domain/plots/6VXX_A_RBD_3D-score.png]

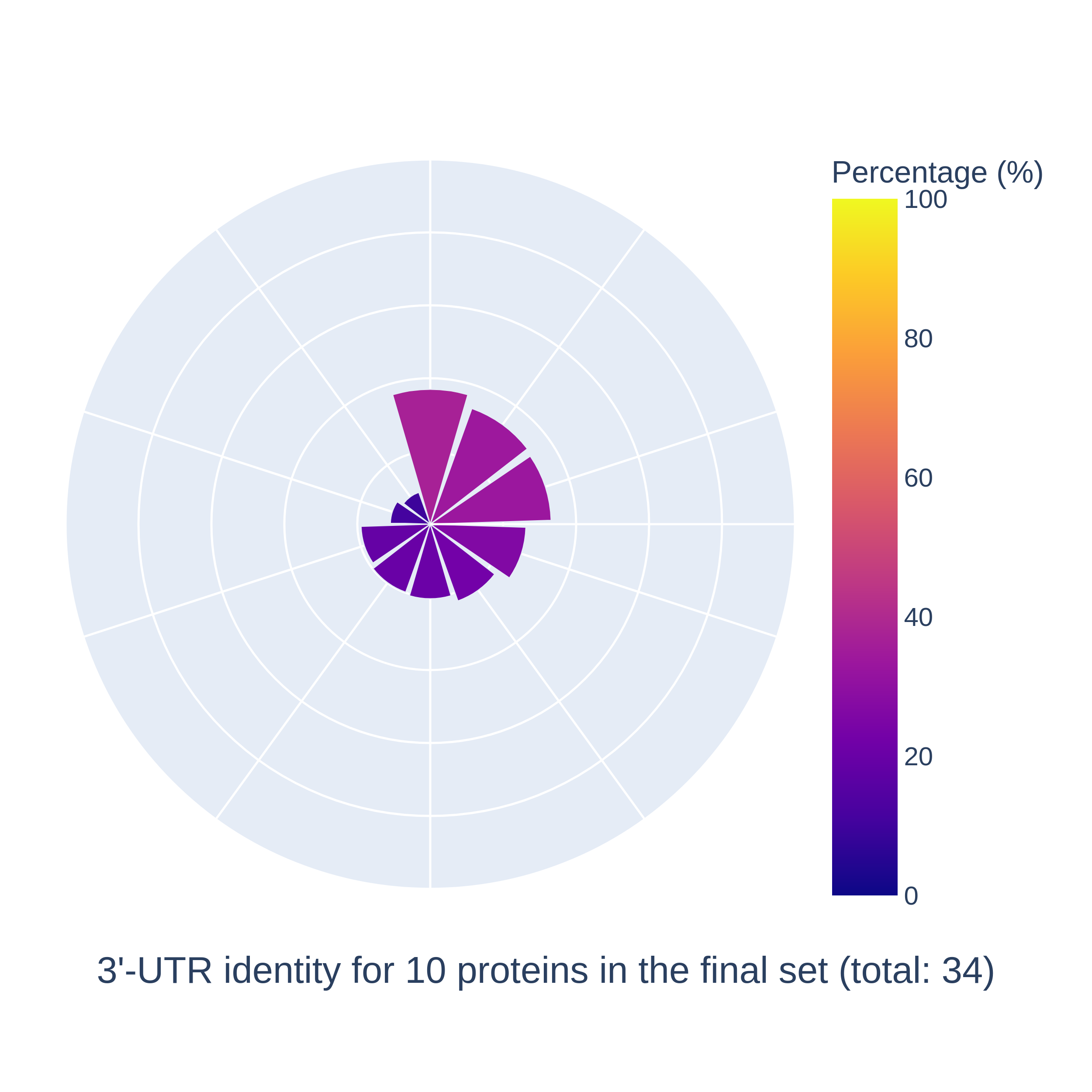

Supplement: Supplementary file 7 — Supplementary Data 4 [file 42003_2023_5076_MOESM7_ESM.zip › 6VXX_A_domain/plots/6VXX_A_RBD_3UTR-identity.png]

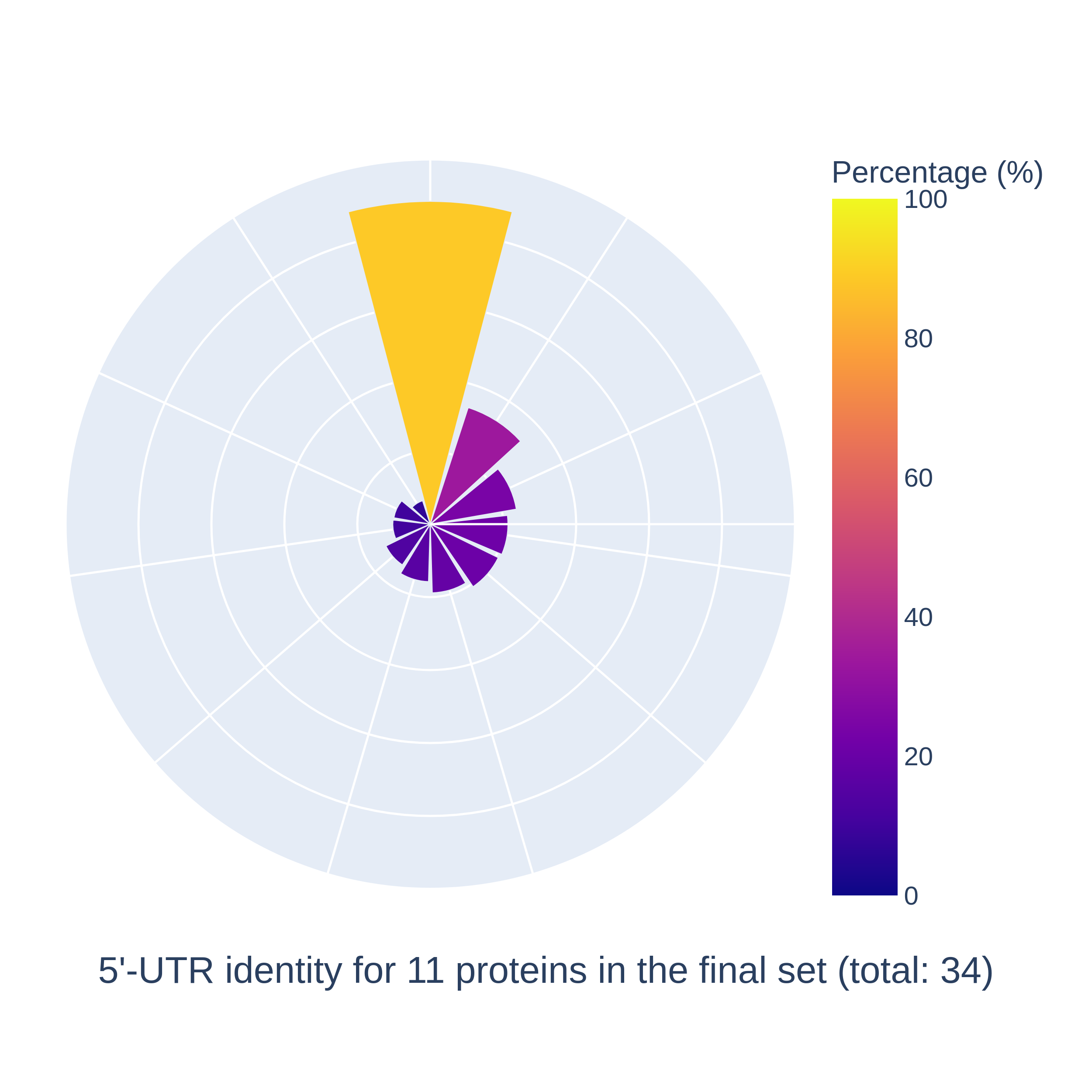

Supplement: Supplementary file 7 — Supplementary Data 4 [file 42003_2023_5076_MOESM7_ESM.zip › 6VXX_A_domain/plots/6VXX_A_RBD_5UTR-identity.png]

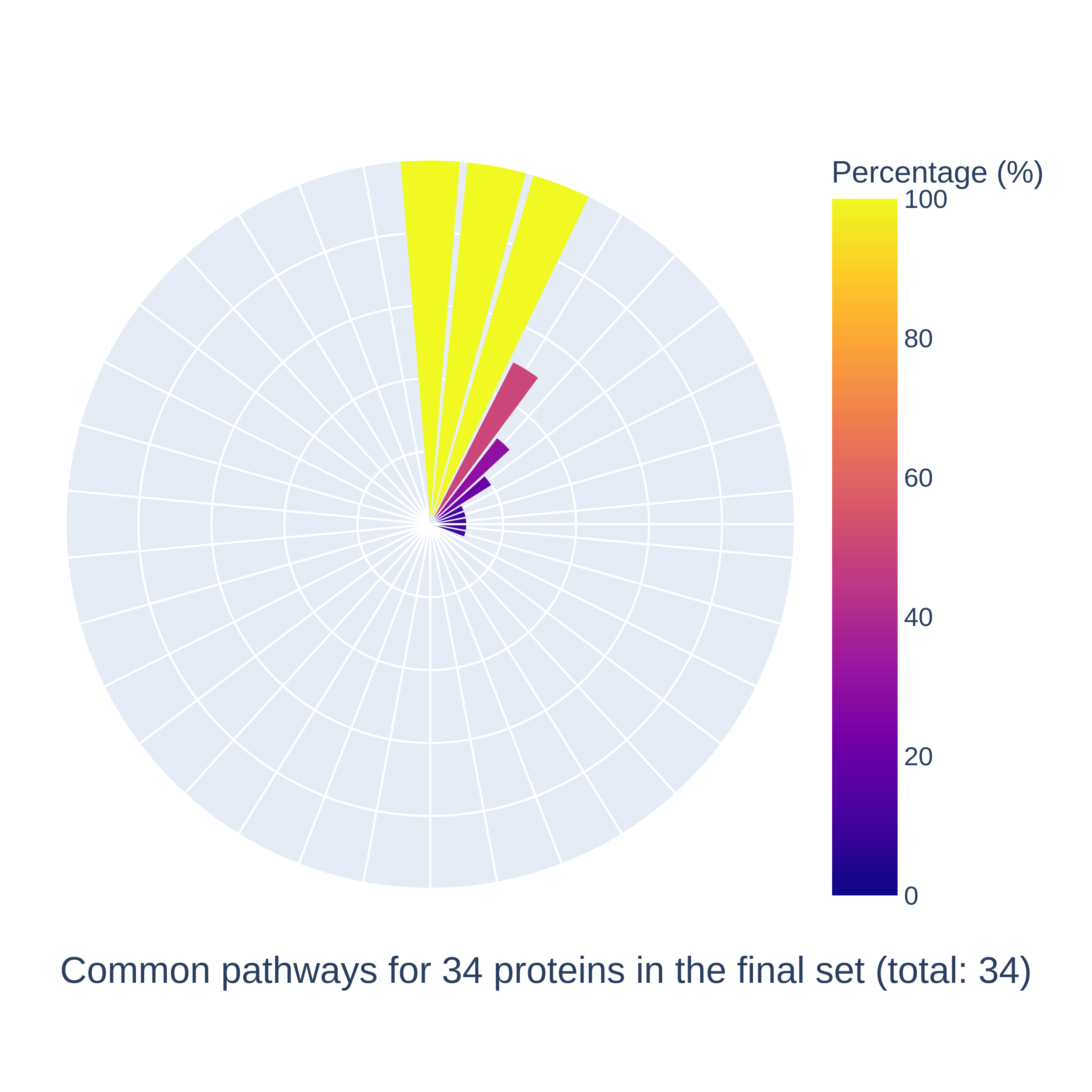

Supplement: Supplementary file 7 — Supplementary Data 4 [file 42003_2023_5076_MOESM7_ESM.zip › 6VXX_A_domain/plots/6VXX_A_RBD_biologicalProcessSim.png]

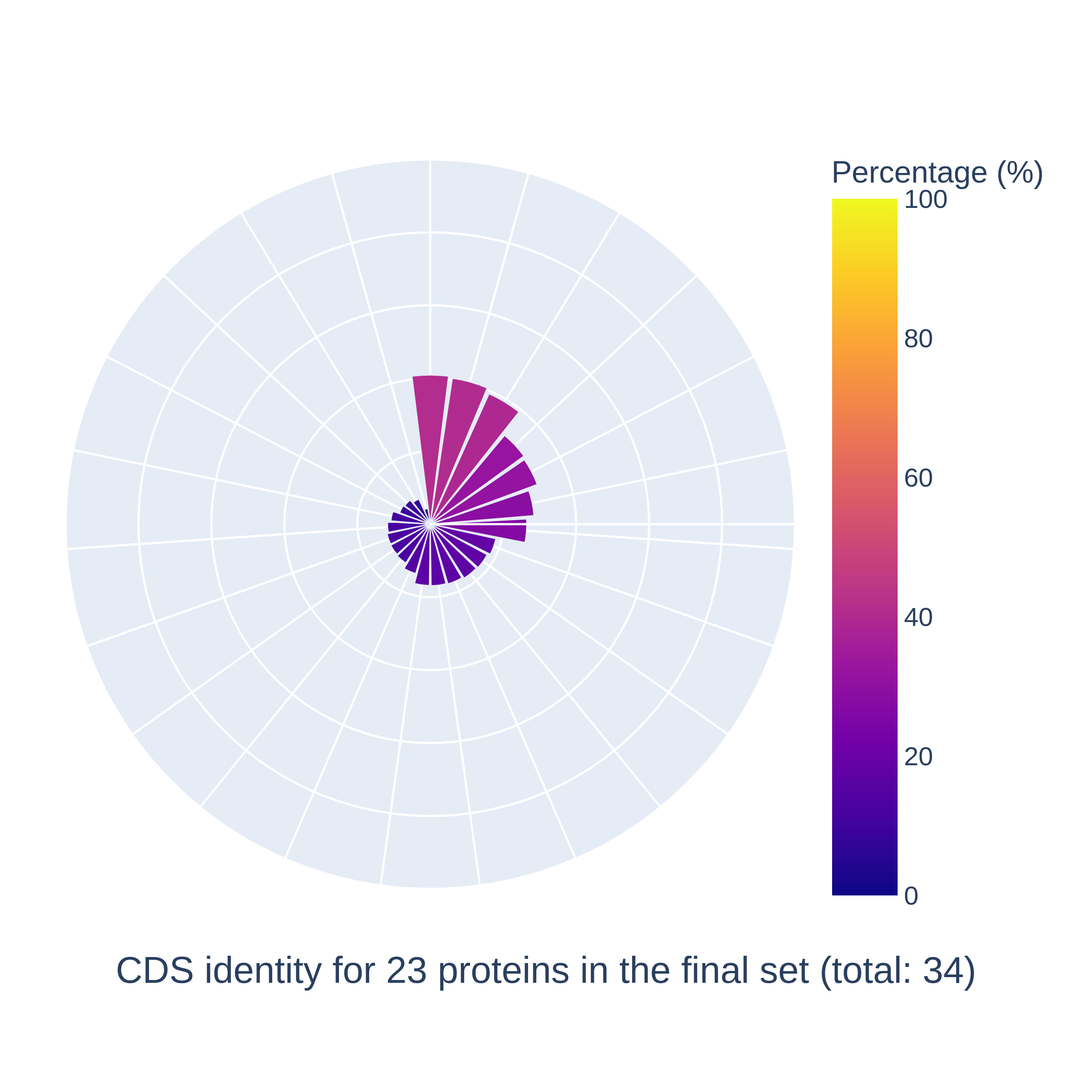

Supplement: Supplementary file 7 — Supplementary Data 4 [file 42003_2023_5076_MOESM7_ESM.zip › 6VXX_A_domain/plots/6VXX_A_RBD_CDS-identity.png]

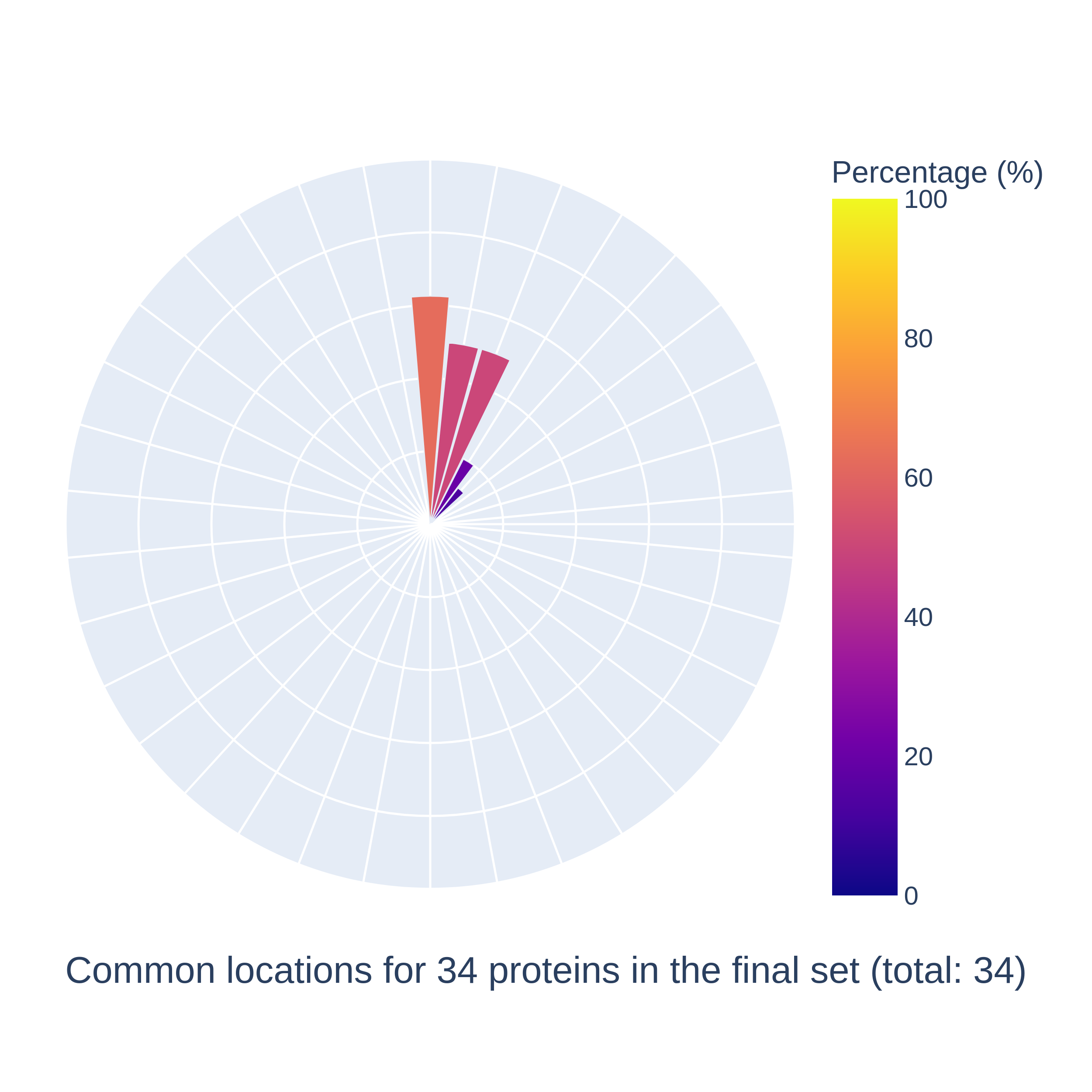

Supplement: Supplementary file 7 — Supplementary Data 4 [file 42003_2023_5076_MOESM7_ESM.zip › 6VXX_A_domain/plots/6VXX_A_RBD_cellularComponentSim.png]

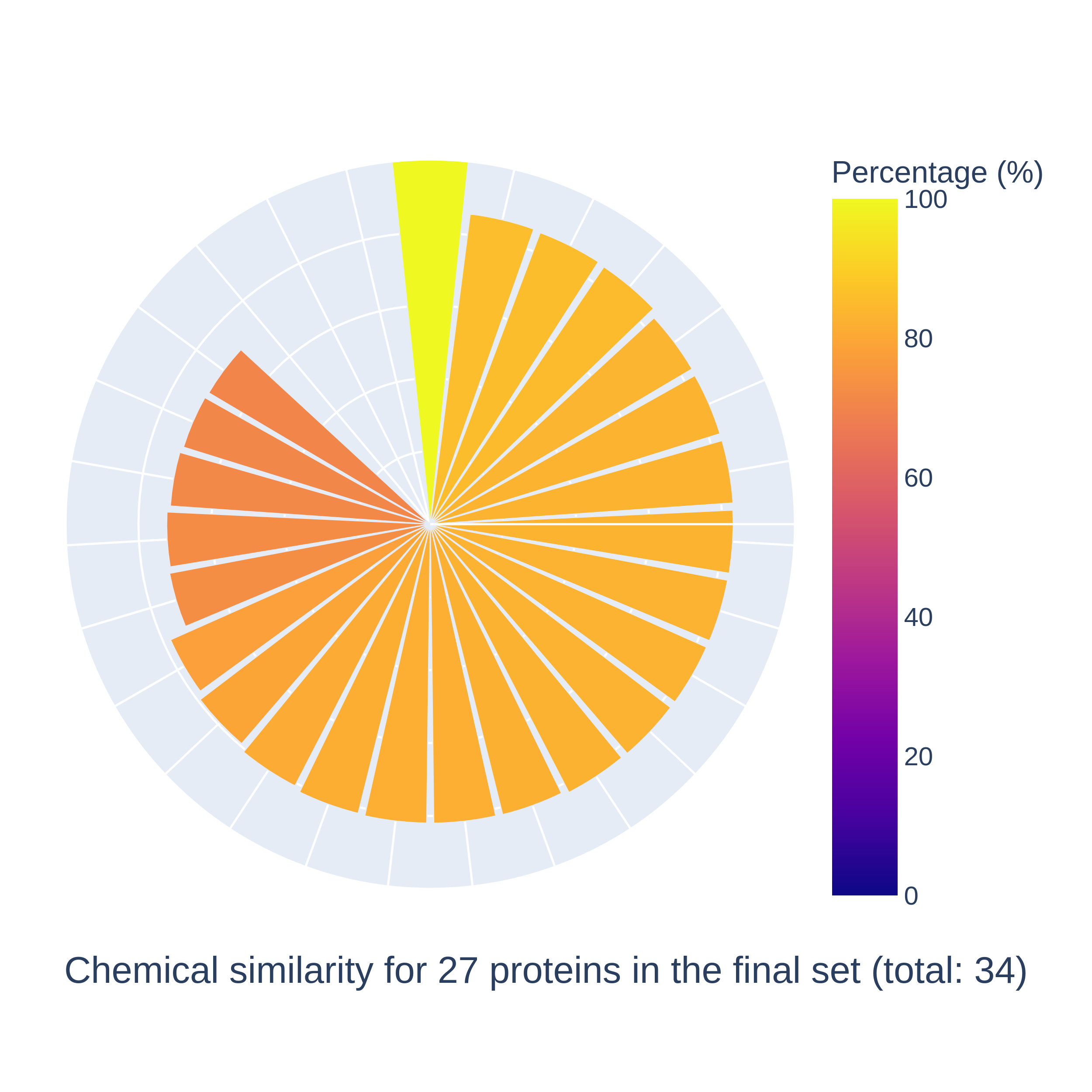

Supplement: Supplementary file 7 — Supplementary Data 4 [file 42003_2023_5076_MOESM7_ESM.zip › 6VXX_A_domain/plots/6VXX_A_RBD_chemSim.png]

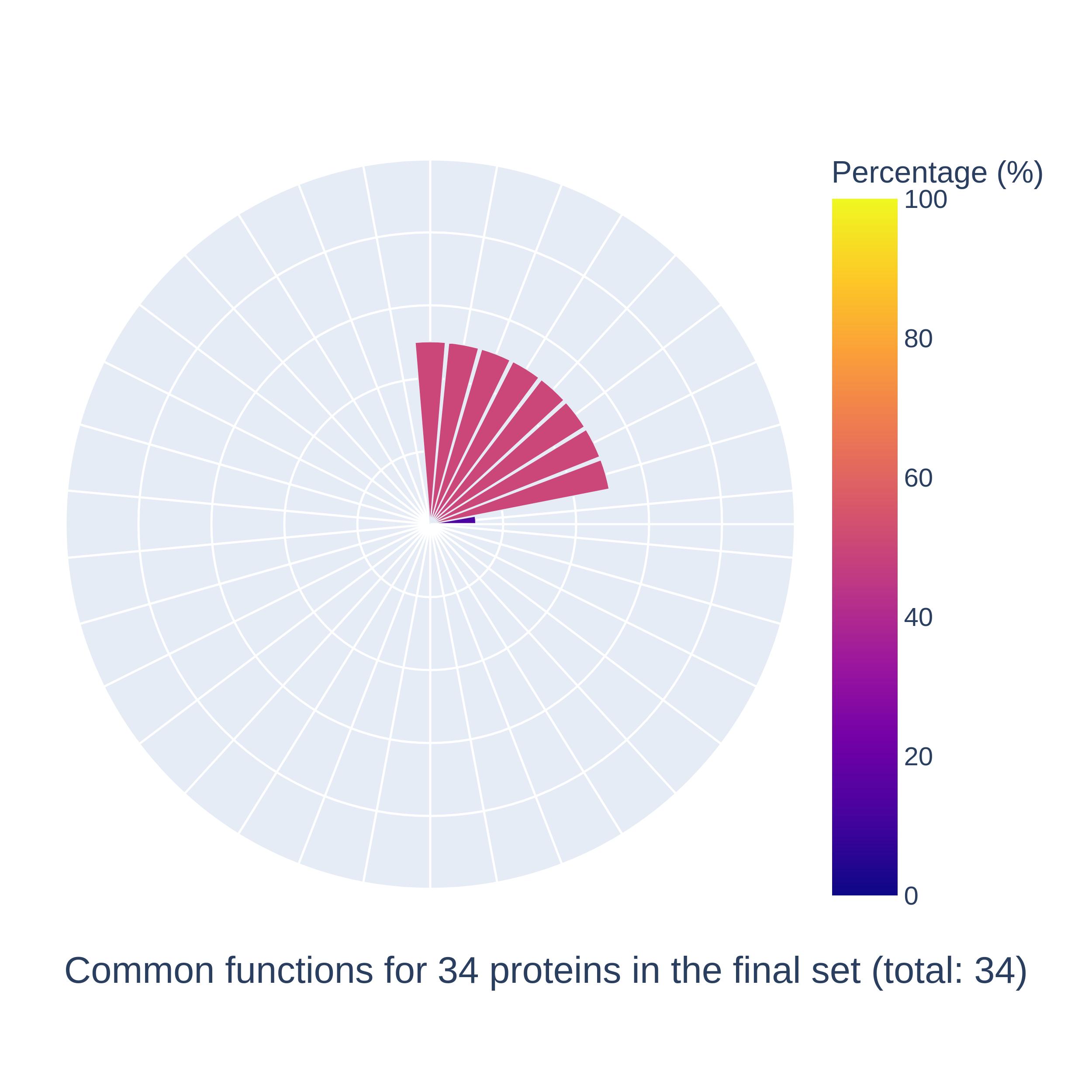

Supplement: Supplementary file 7 — Supplementary Data 4 [file 42003_2023_5076_MOESM7_ESM.zip › 6VXX_A_domain/plots/6VXX_A_RBD_molecularFunctionSim.png]

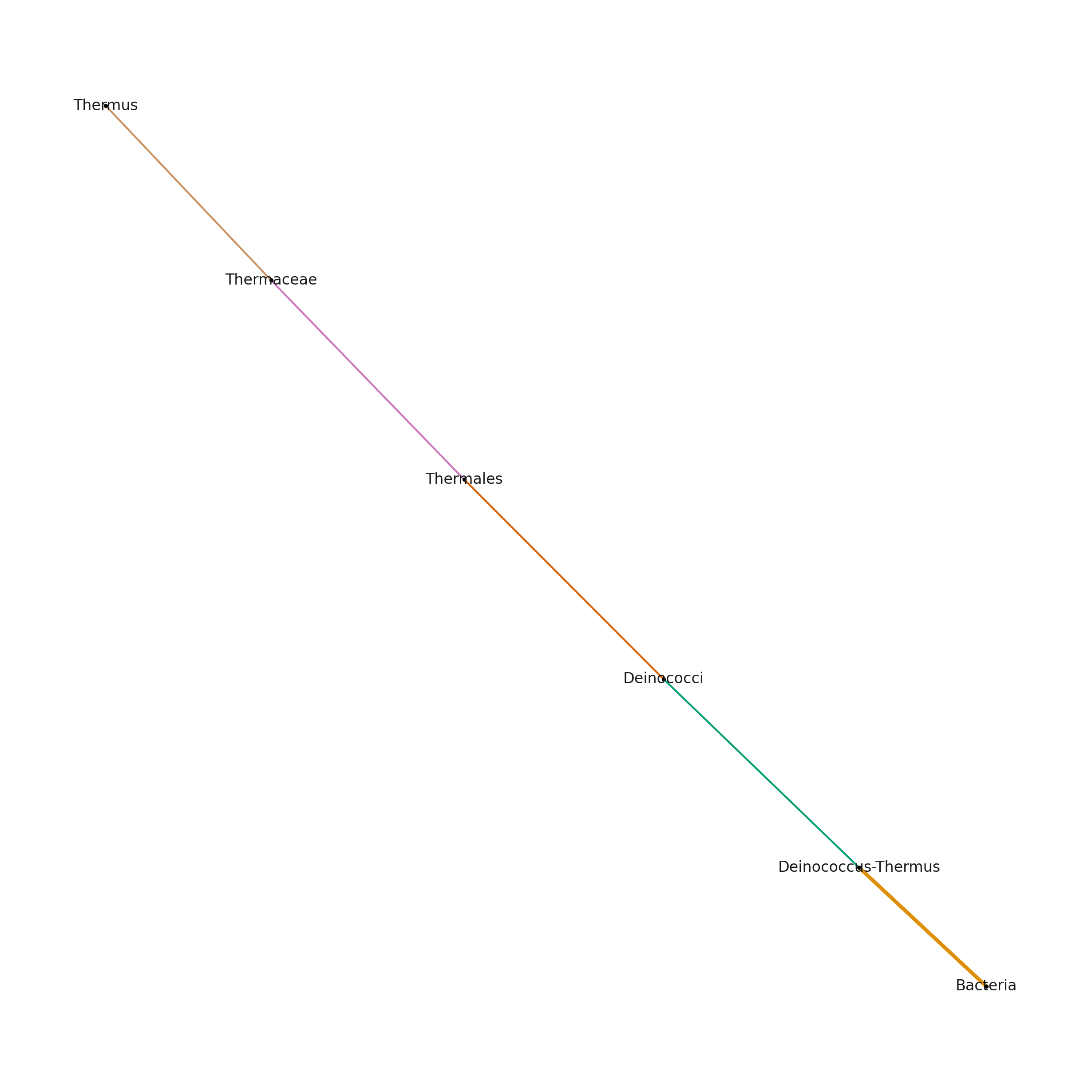

Supplement: Supplementary file 7 — Supplementary Data 4 [file 42003_2023_5076_MOESM7_ESM.zip › 6VXX_A_segment/plots/6VXX_A_site0-metrics-Bacteria-tree.png]

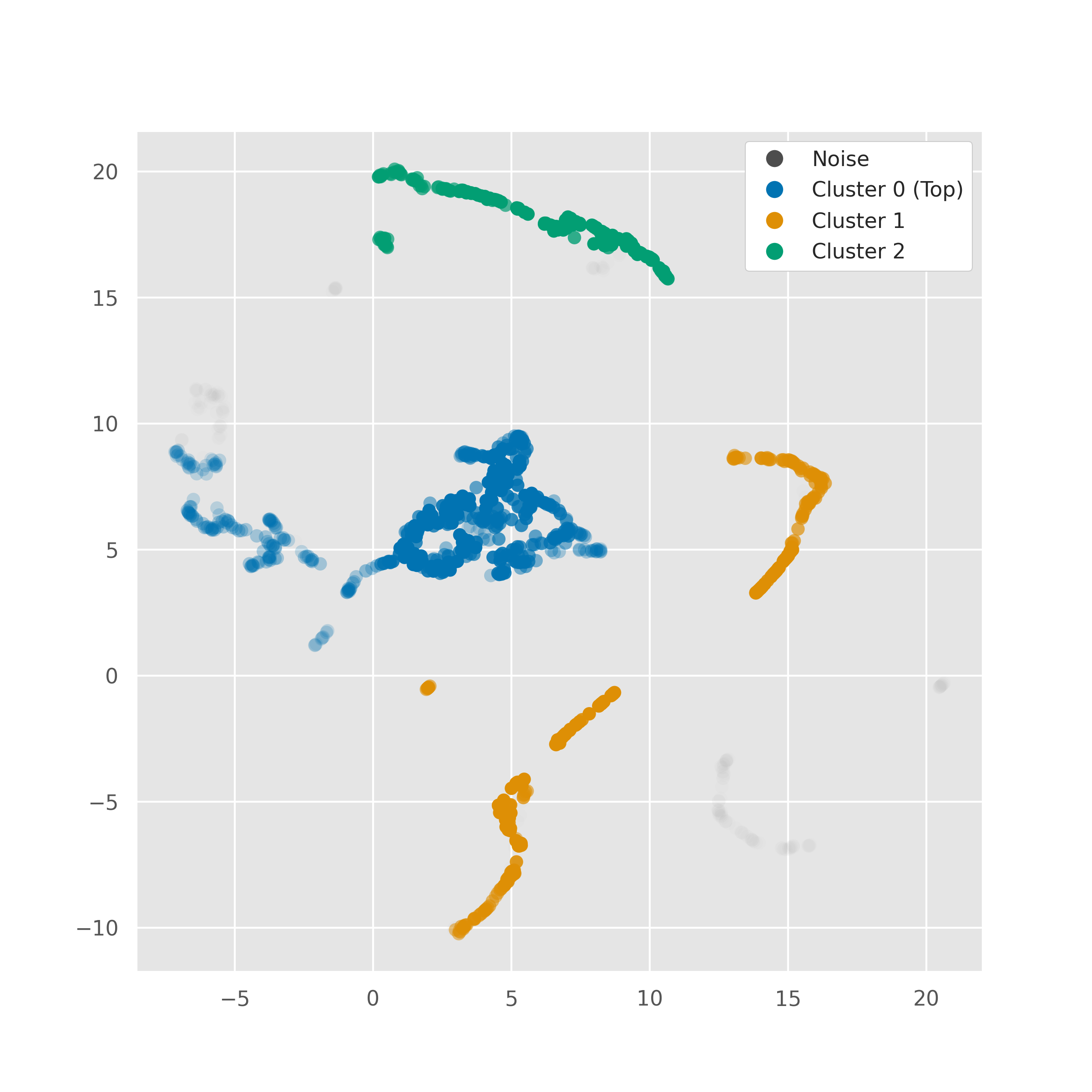

Supplement: Supplementary file 7 — Supplementary Data 4 [file 42003_2023_5076_MOESM7_ESM.zip › 6VXX_A_segment/plots/6VXX_A_site0-metrics-clusters-.png]

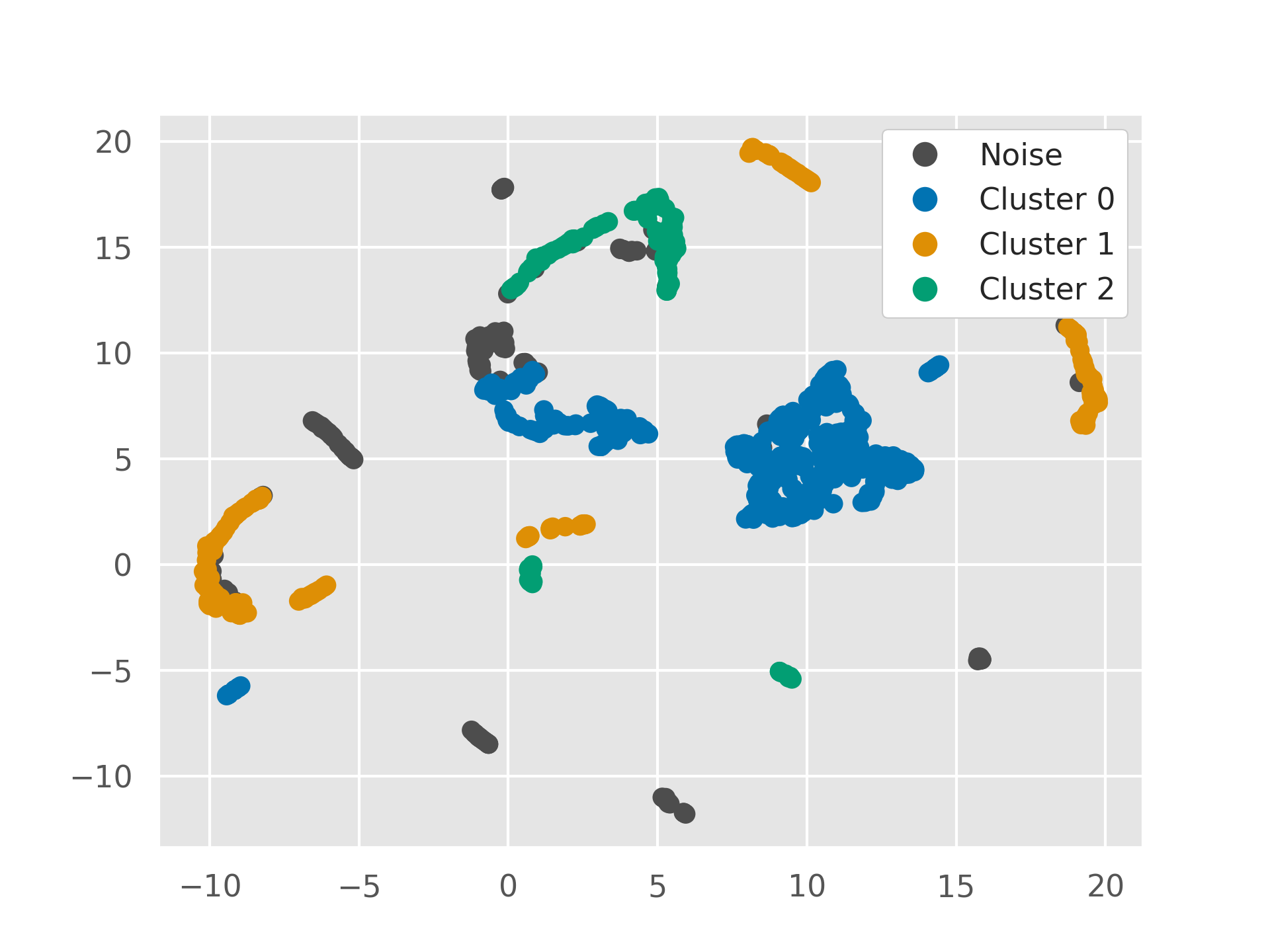

Supplement: Supplementary file 7 — Supplementary Data 4 [file 42003_2023_5076_MOESM7_ESM.zip › 6VXX_A_segment/plots/6VXX_A_site0-metrics-clusters-initial.png]

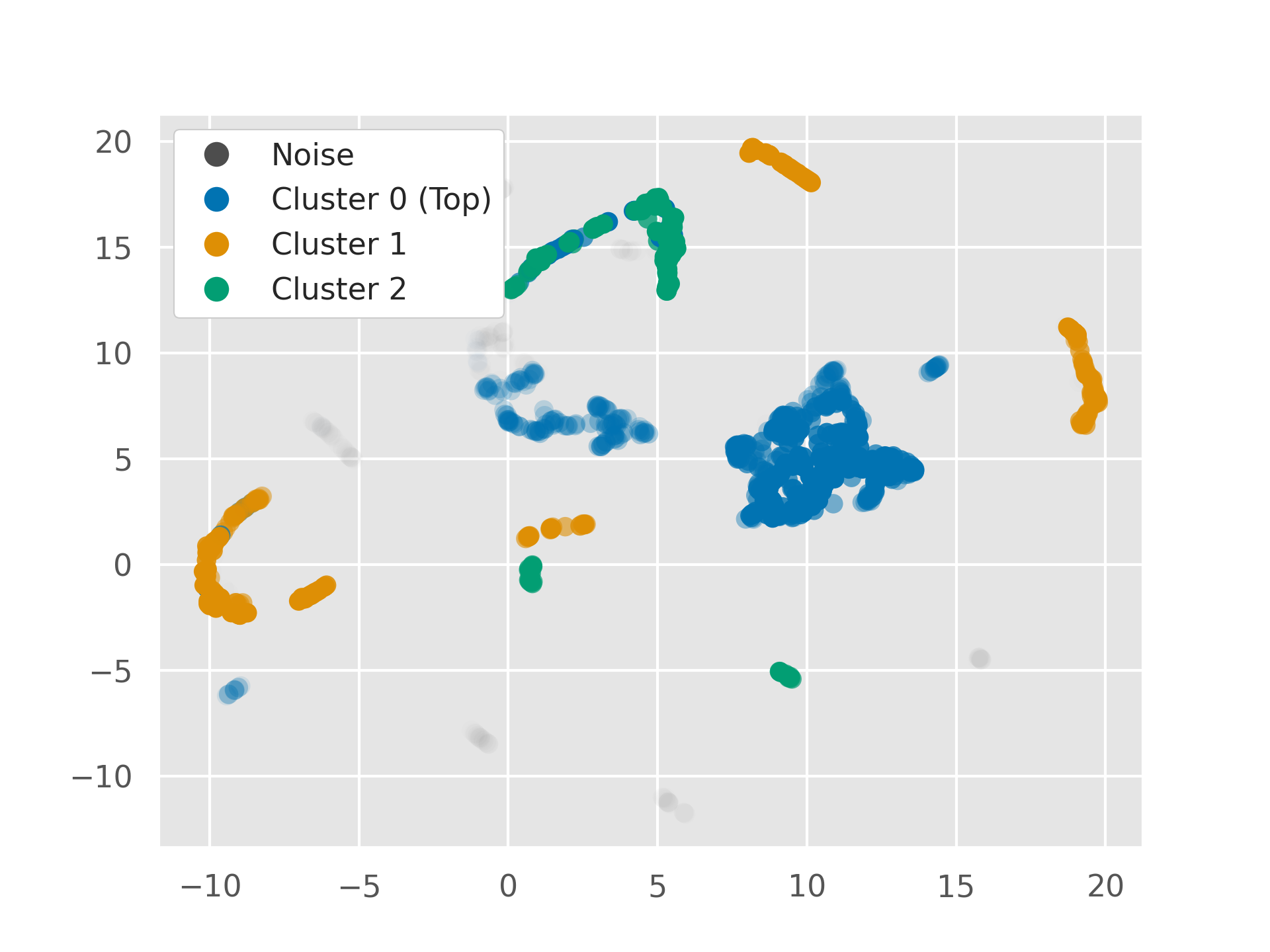

Supplement: Supplementary file 7 — Supplementary Data 4 [file 42003_2023_5076_MOESM7_ESM.zip › 6VXX_A_segment/plots/6VXX_A_site0-metrics-clusters.png]

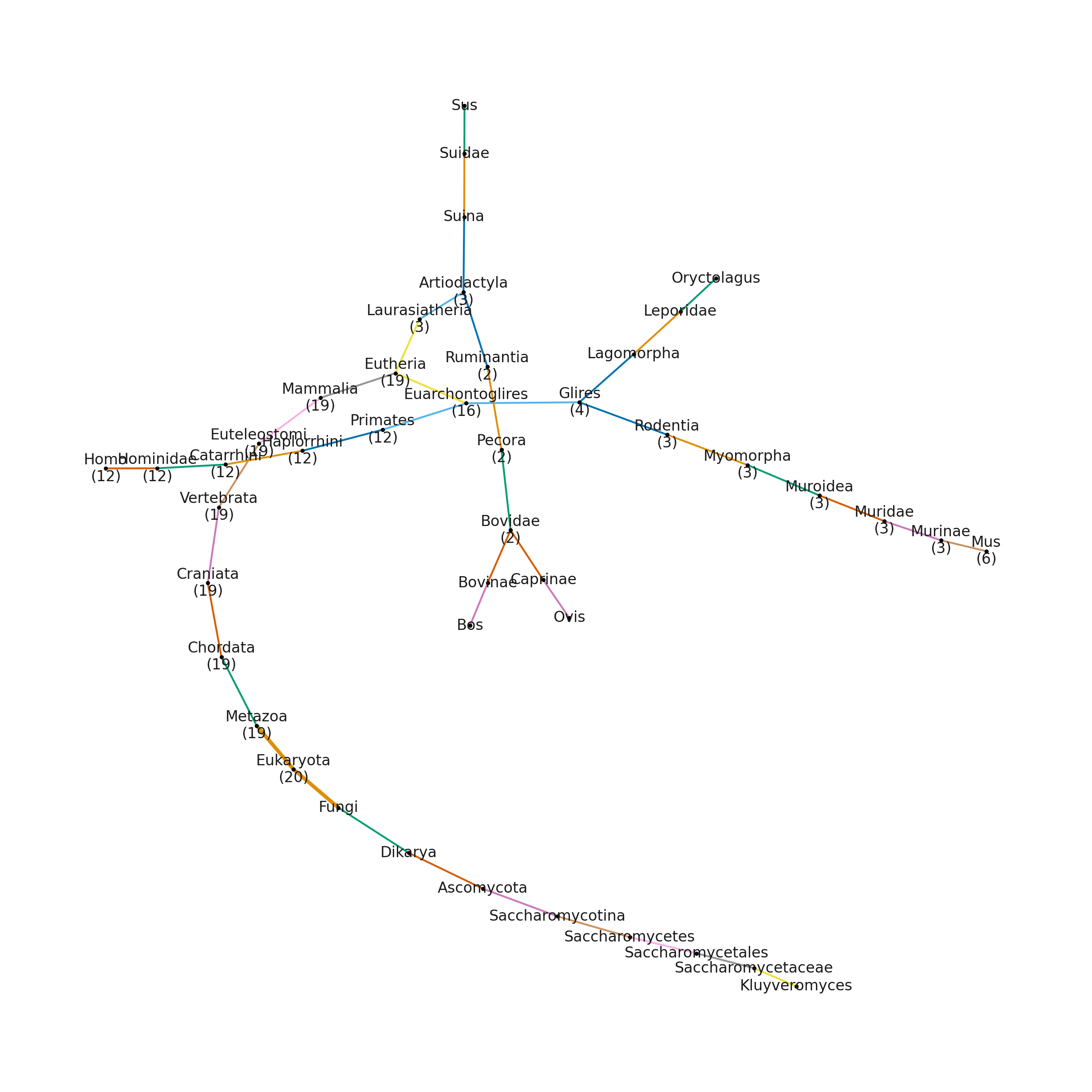

Supplement: Supplementary file 7 — Supplementary Data 4 [file 42003_2023_5076_MOESM7_ESM.zip › 6VXX_A_segment/plots/6VXX_A_site0-metrics-Eukaryota-tree.png]

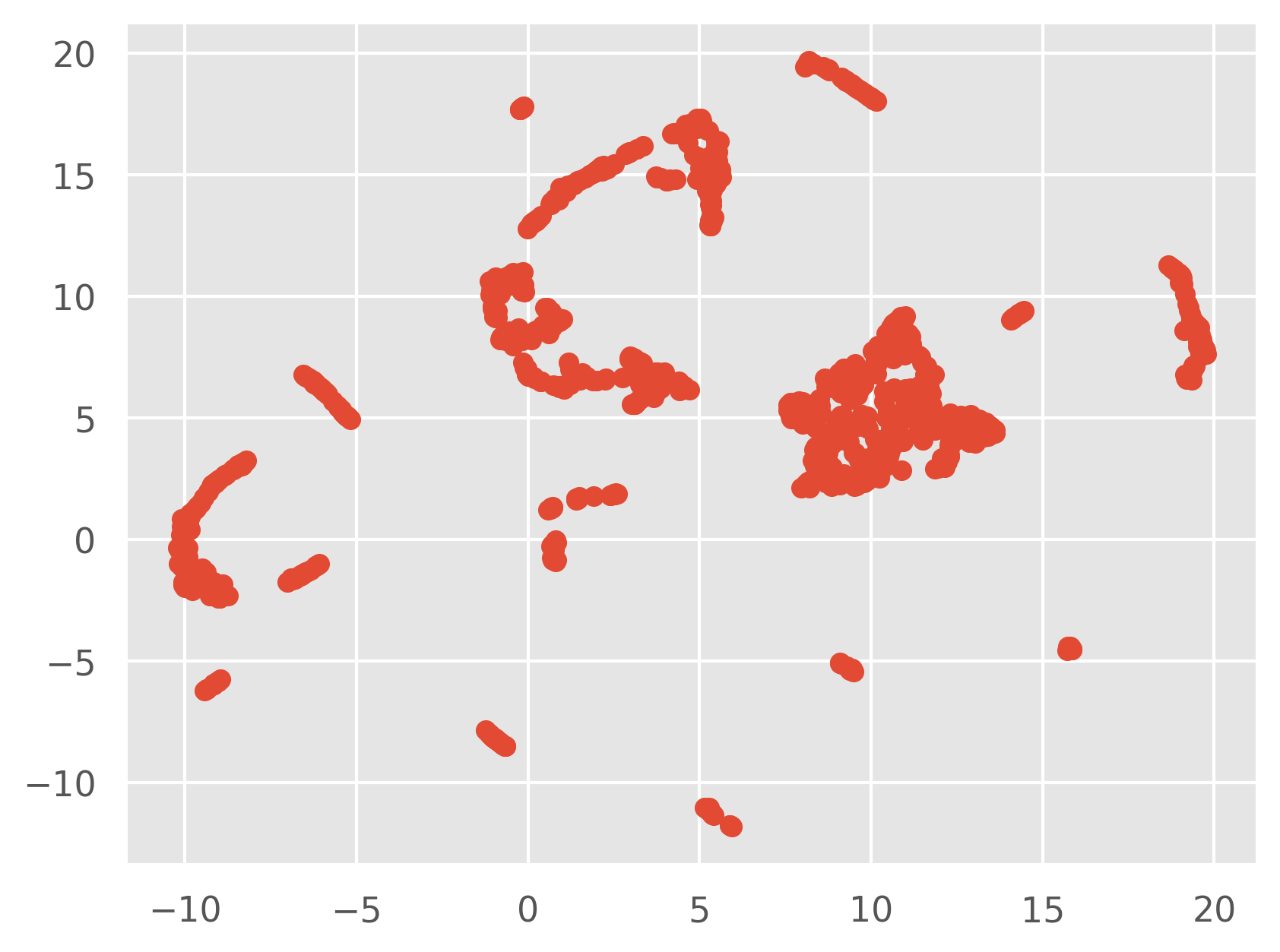

Supplement: Supplementary file 7 — Supplementary Data 4 [file 42003_2023_5076_MOESM7_ESM.zip › 6VXX_A_segment/plots/6VXX_A_site0-metrics-UMAP-.png]

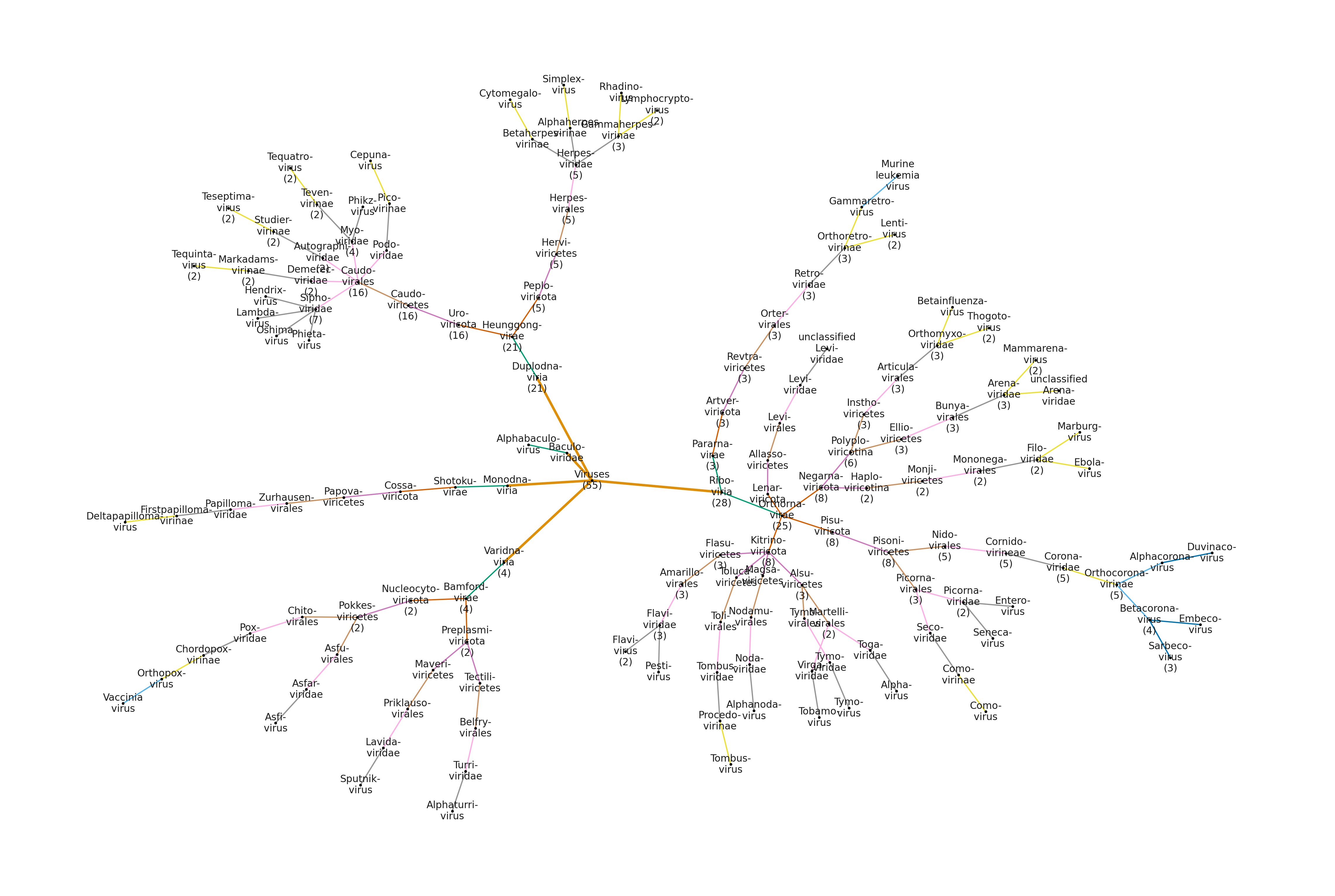

Supplement: Supplementary file 7 — Supplementary Data 4 [file 42003_2023_5076_MOESM7_ESM.zip › 6VXX_A_segment/plots/6VXX_A_site0-metrics-Viruses-tree.png]

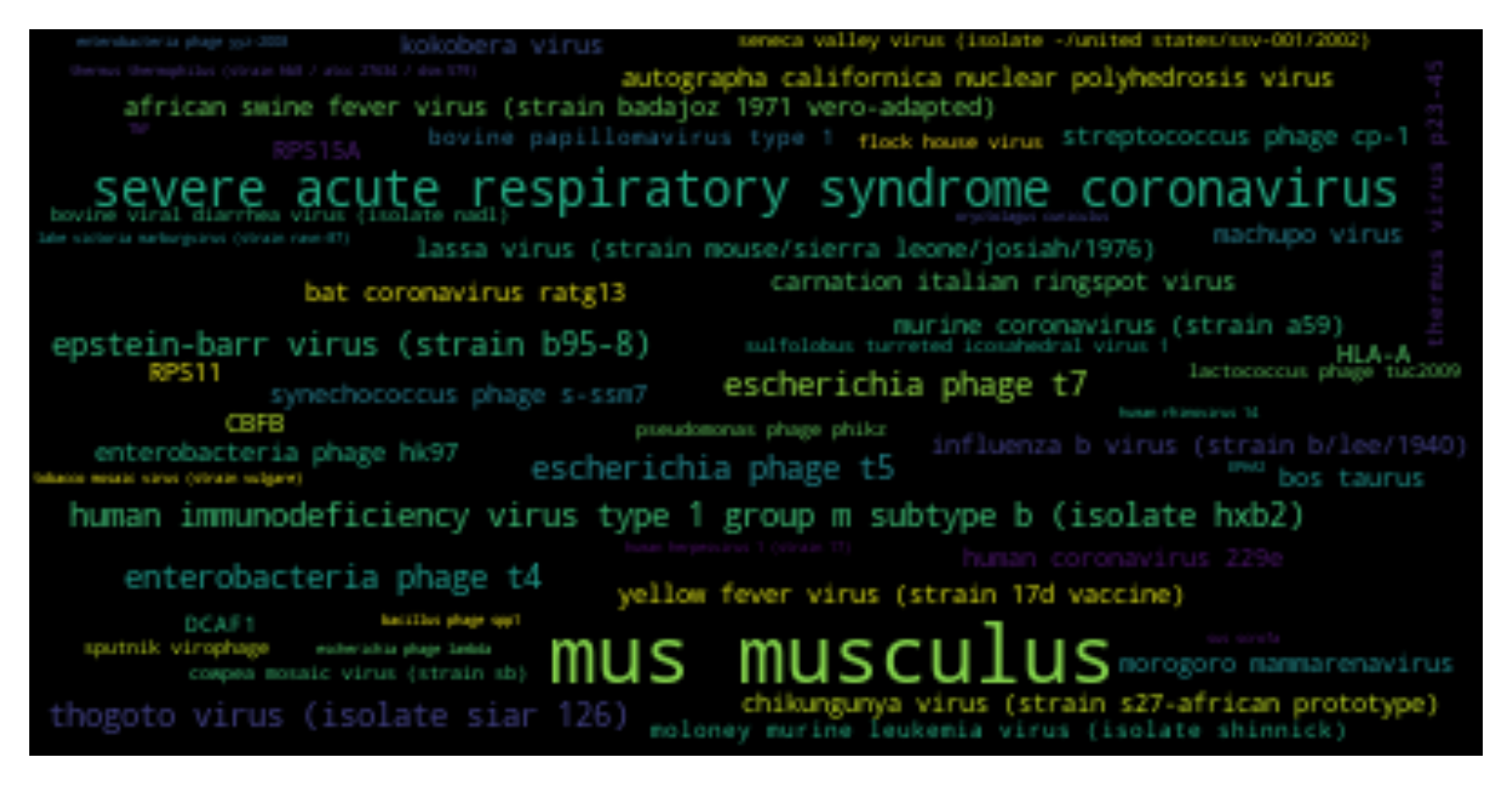

Supplement: Supplementary file 7 — Supplementary Data 4 [file 42003_2023_5076_MOESM7_ESM.zip › 6VXX_A_segment/plots/6VXX_A_site0-metrics-wordcloud.png]

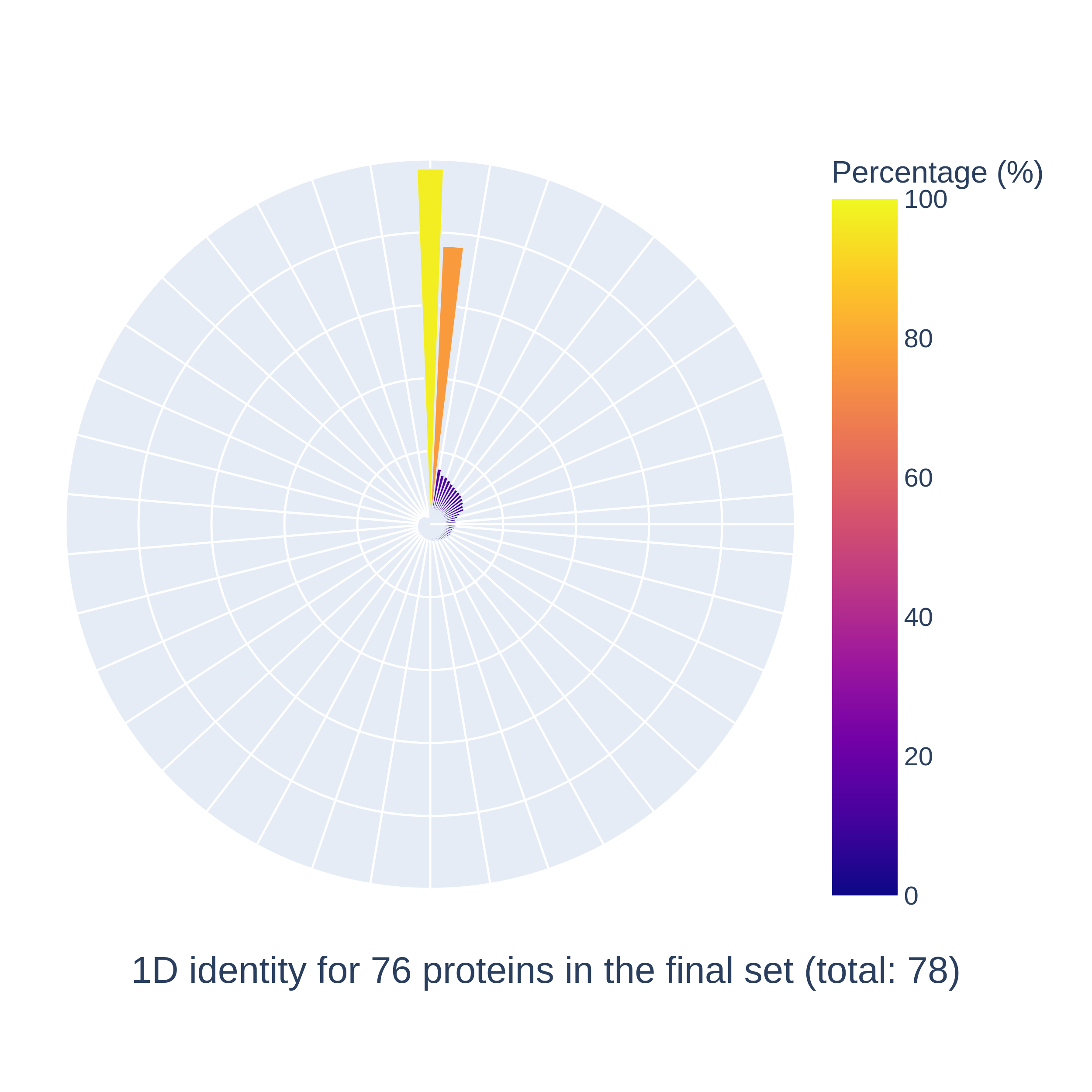

Supplement: Supplementary file 7 — Supplementary Data 4 [file 42003_2023_5076_MOESM7_ESM.zip › 6VXX_A_segment/plots/6VXX_A_site0-metrics_1D-identity.png]

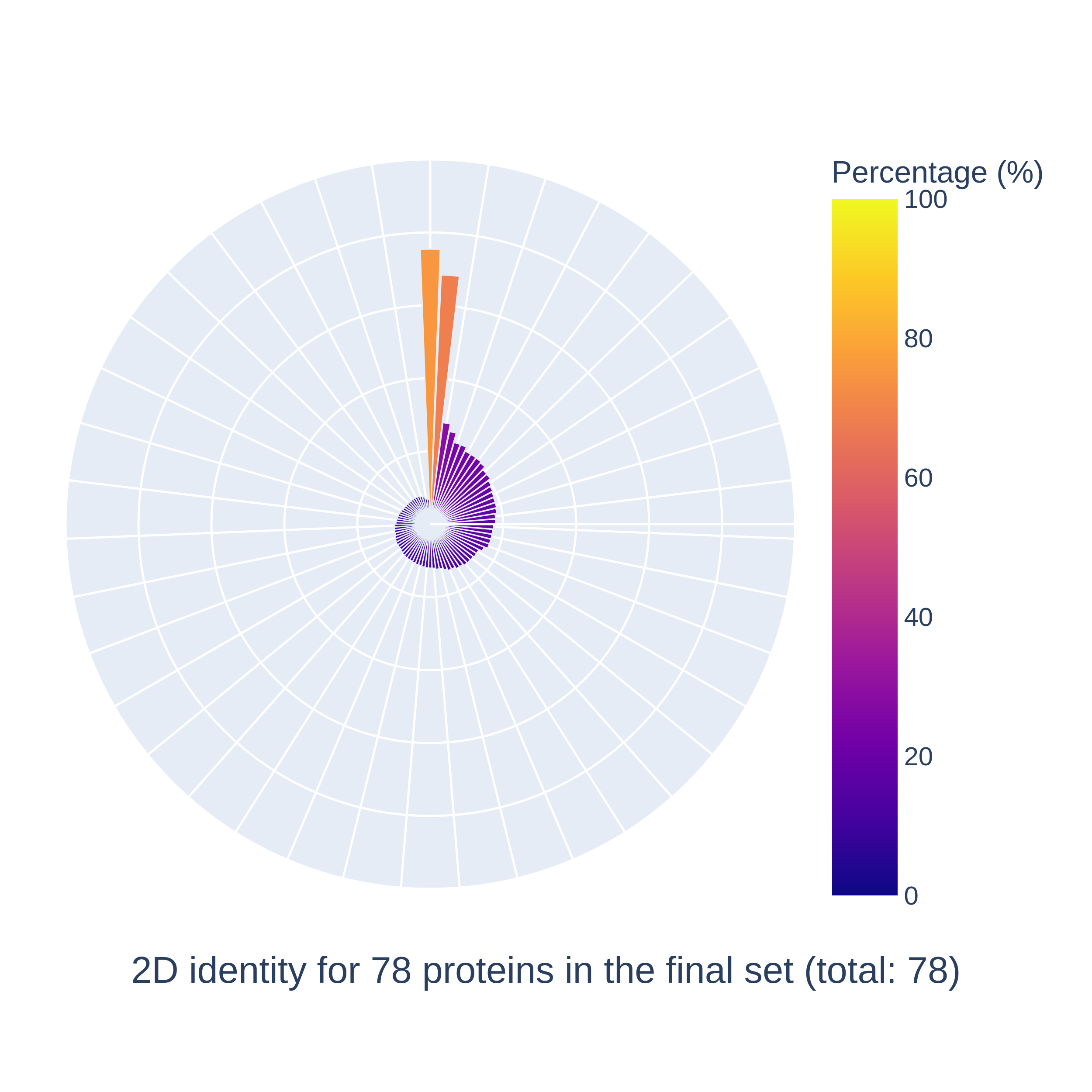

Supplement: Supplementary file 7 — Supplementary Data 4 [file 42003_2023_5076_MOESM7_ESM.zip › 6VXX_A_segment/plots/6VXX_A_site0-metrics_2D-identity.png]

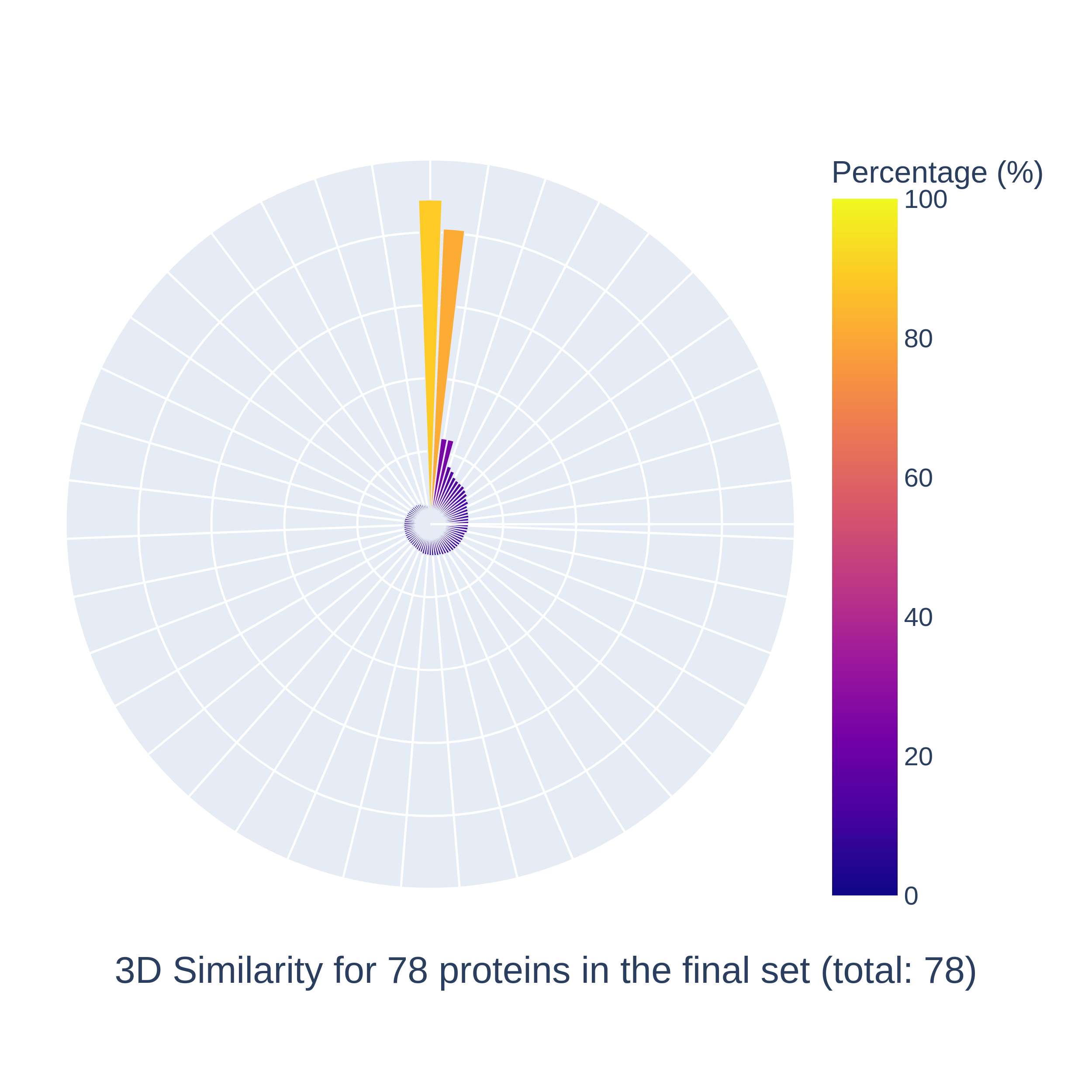

Supplement: Supplementary file 7 — Supplementary Data 4 [file 42003_2023_5076_MOESM7_ESM.zip › 6VXX_A_segment/plots/6VXX_A_site0-metrics_3D-score.png]

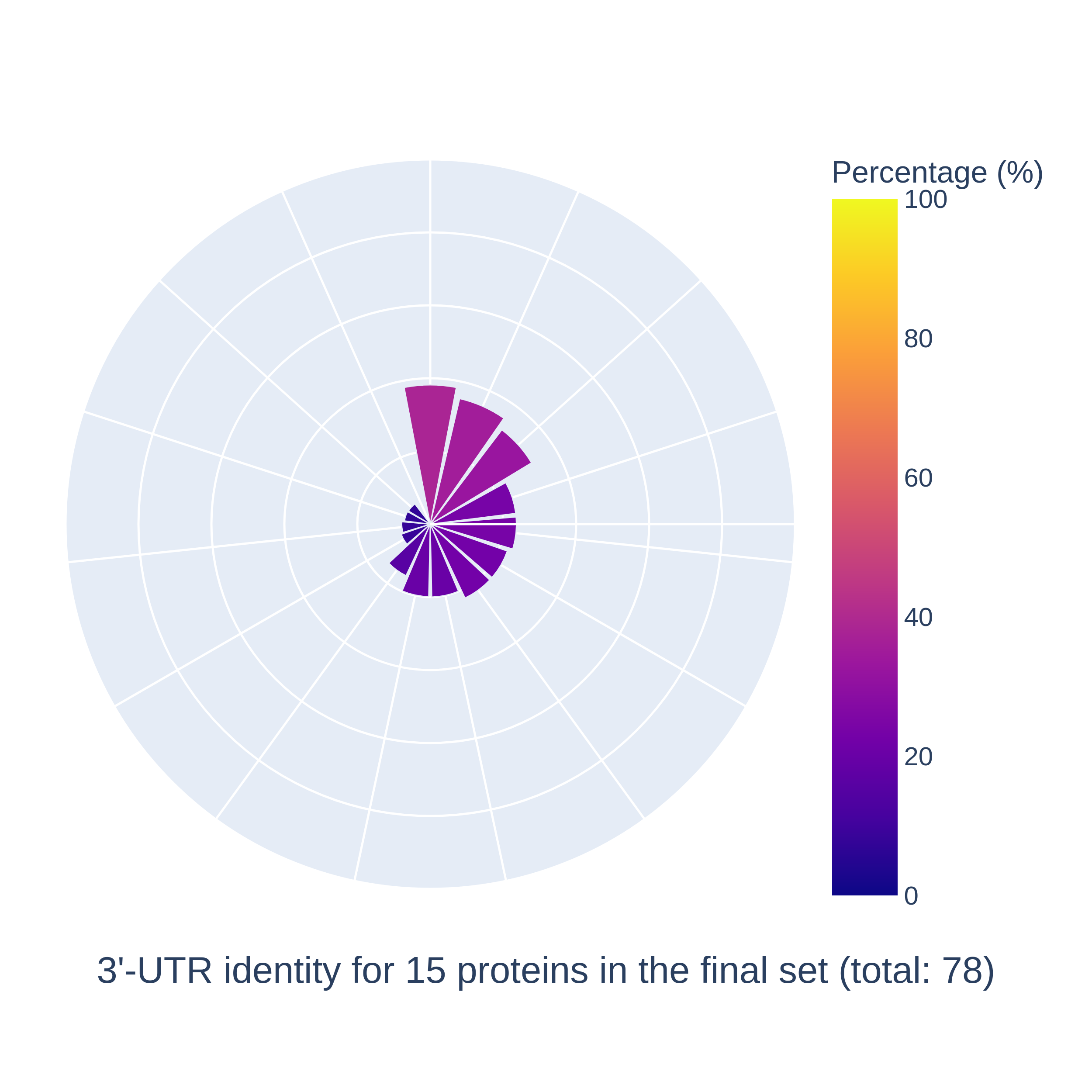

Supplement: Supplementary file 7 — Supplementary Data 4 [file 42003_2023_5076_MOESM7_ESM.zip › 6VXX_A_segment/plots/6VXX_A_site0-metrics_3UTR-identity.png]

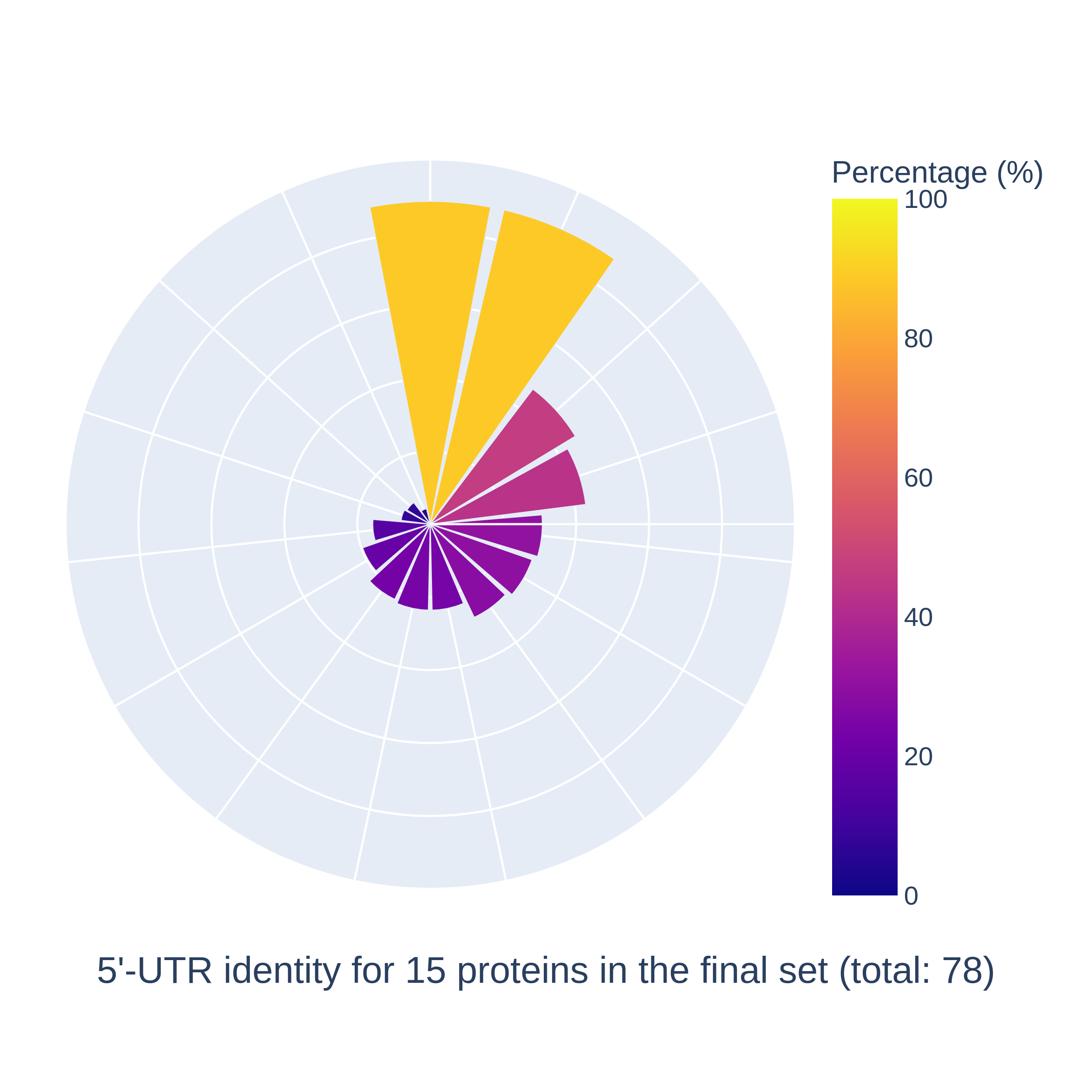

Supplement: Supplementary file 7 — Supplementary Data 4 [file 42003_2023_5076_MOESM7_ESM.zip › 6VXX_A_segment/plots/6VXX_A_site0-metrics_5UTR-identity.png]

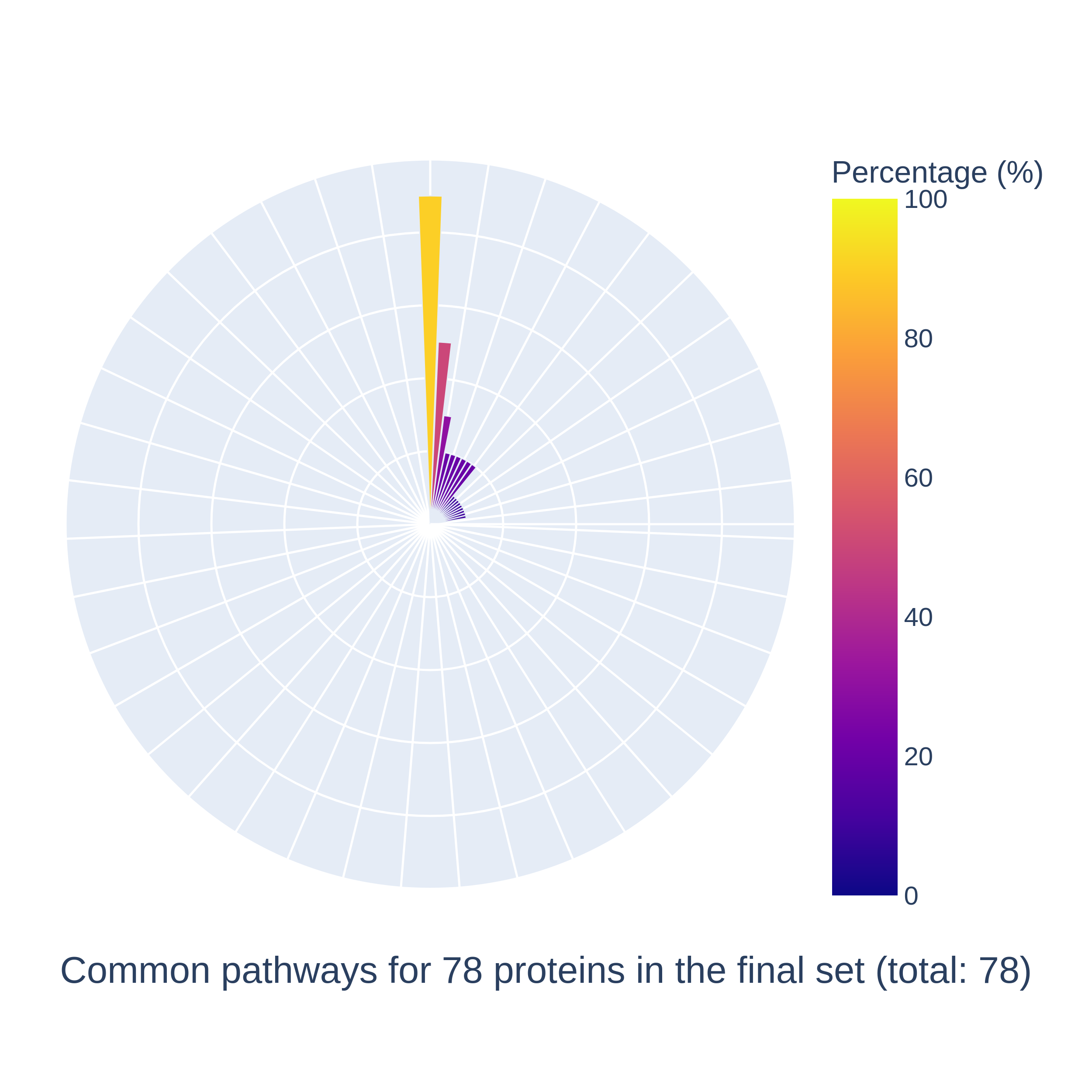

Supplement: Supplementary file 7 — Supplementary Data 4 [file 42003_2023_5076_MOESM7_ESM.zip › 6VXX_A_segment/plots/6VXX_A_site0-metrics_biologicalProcessSim.png]

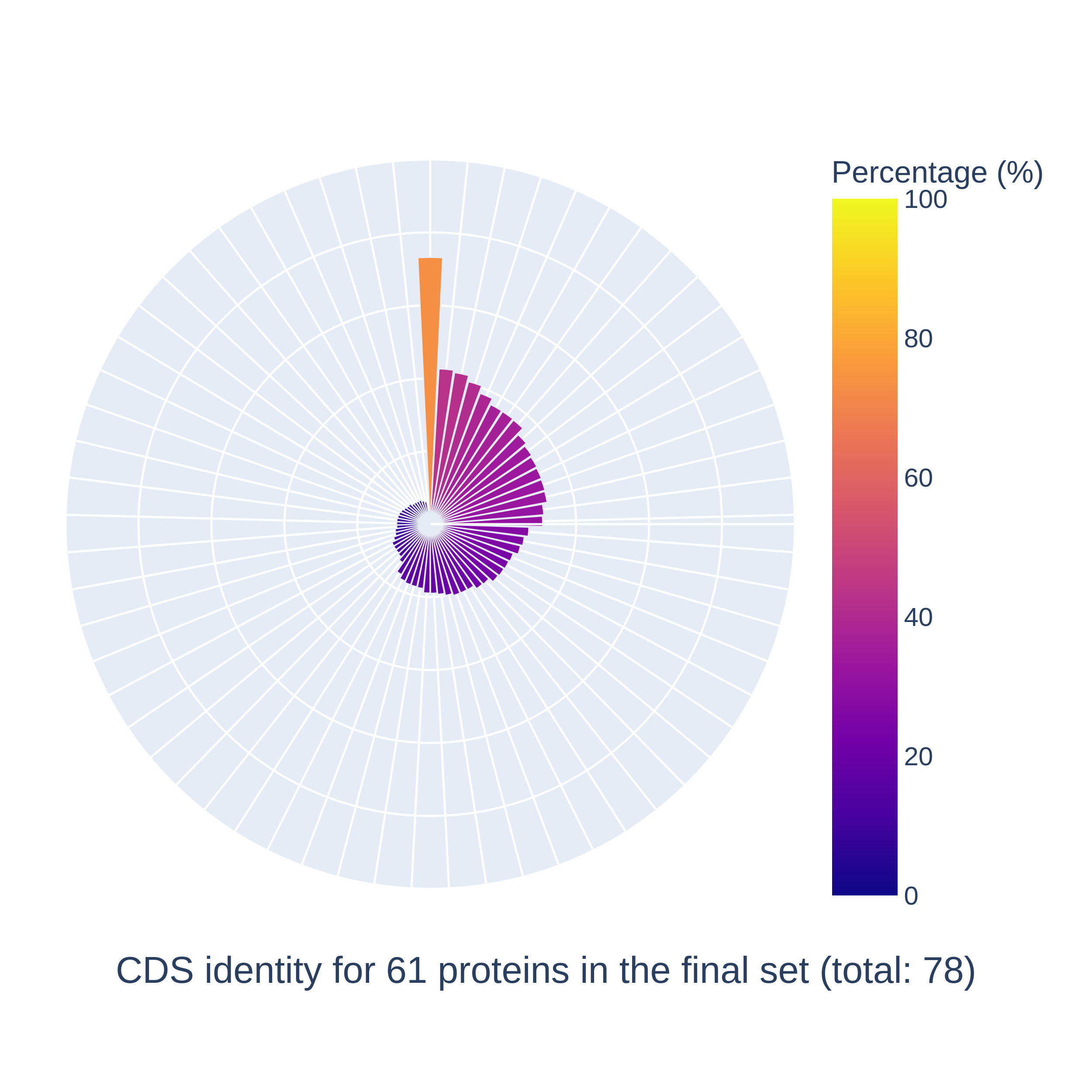

Supplement: Supplementary file 7 — Supplementary Data 4 [file 42003_2023_5076_MOESM7_ESM.zip › 6VXX_A_segment/plots/6VXX_A_site0-metrics_CDS-identity.png]

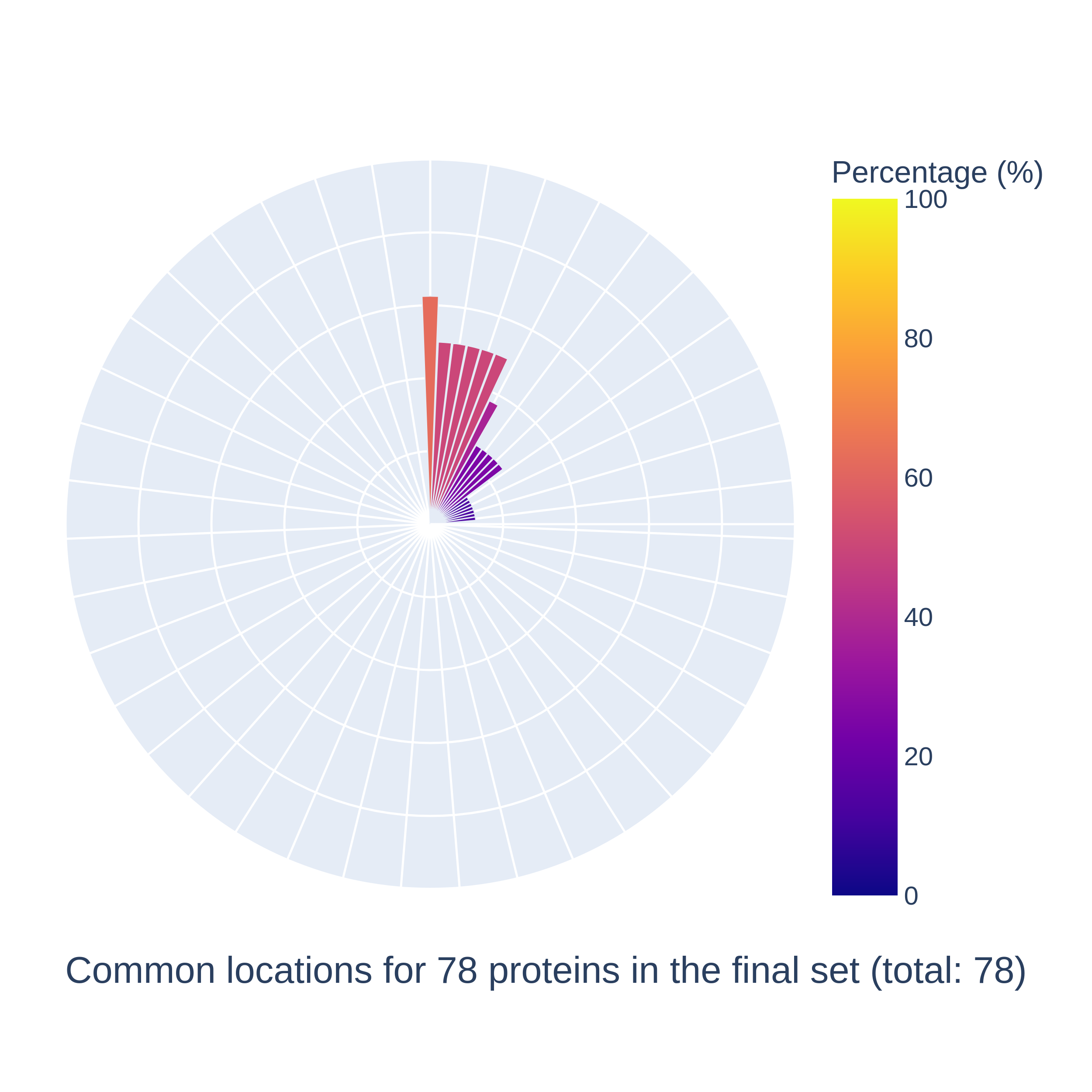

Supplement: Supplementary file 7 — Supplementary Data 4 [file 42003_2023_5076_MOESM7_ESM.zip › 6VXX_A_segment/plots/6VXX_A_site0-metrics_cellularComponentSim.png]

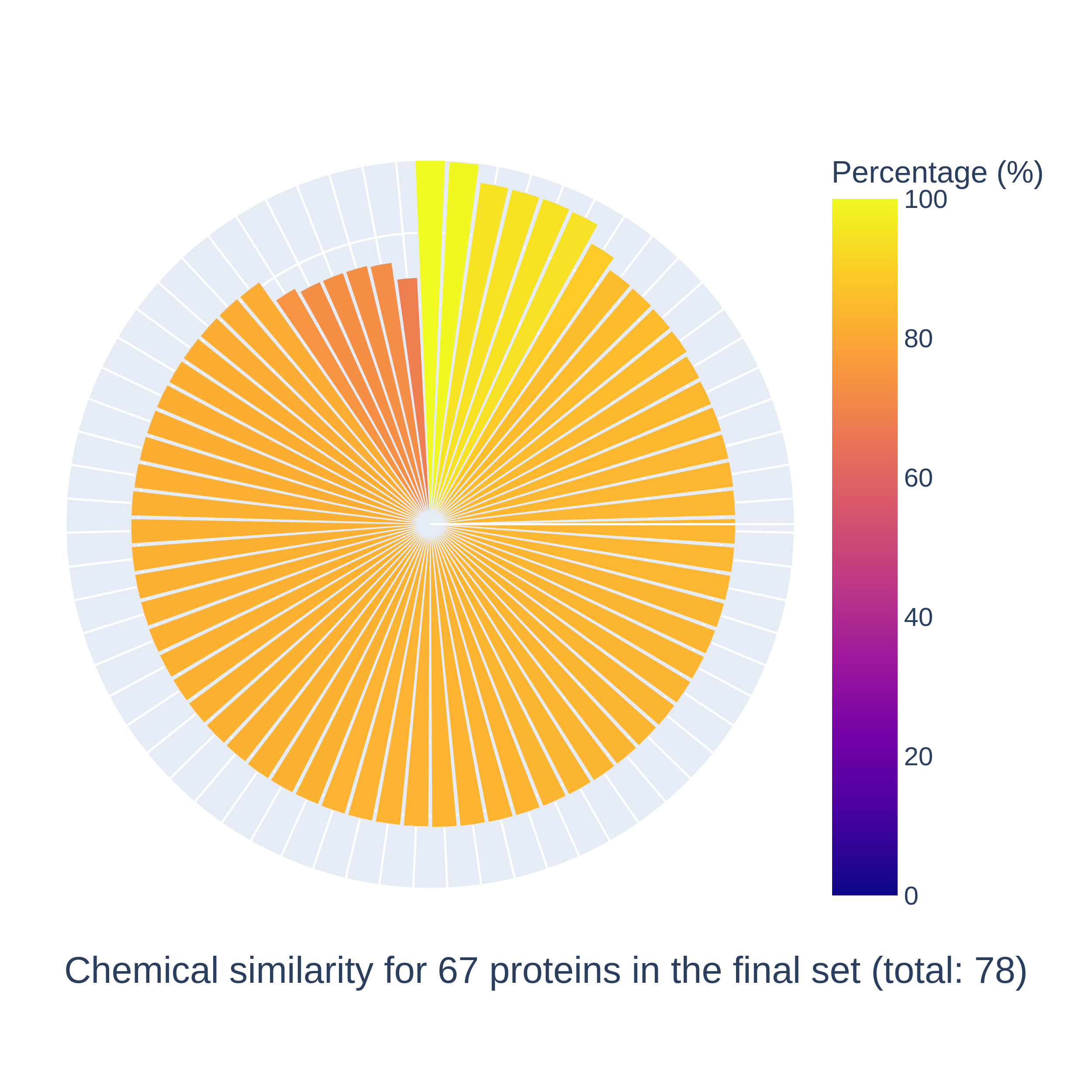

Supplement: Supplementary file 7 — Supplementary Data 4 [file 42003_2023_5076_MOESM7_ESM.zip › 6VXX_A_segment/plots/6VXX_A_site0-metrics_chemSim.png]

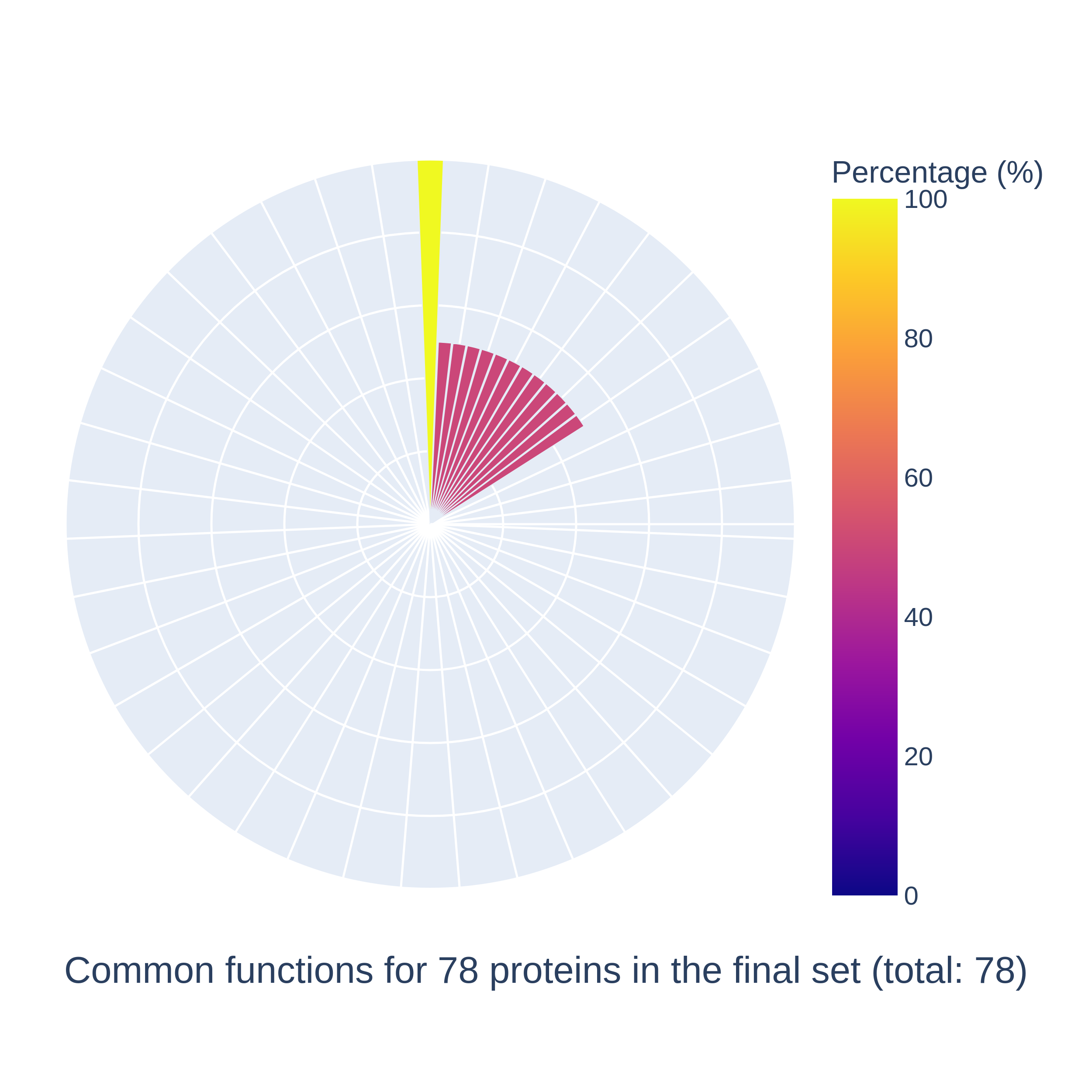

Supplement: Supplementary file 7 — Supplementary Data 4 [file 42003_2023_5076_MOESM7_ESM.zip › 6VXX_A_segment/plots/6VXX_A_site0-metrics_molecularFunctionSim.png]

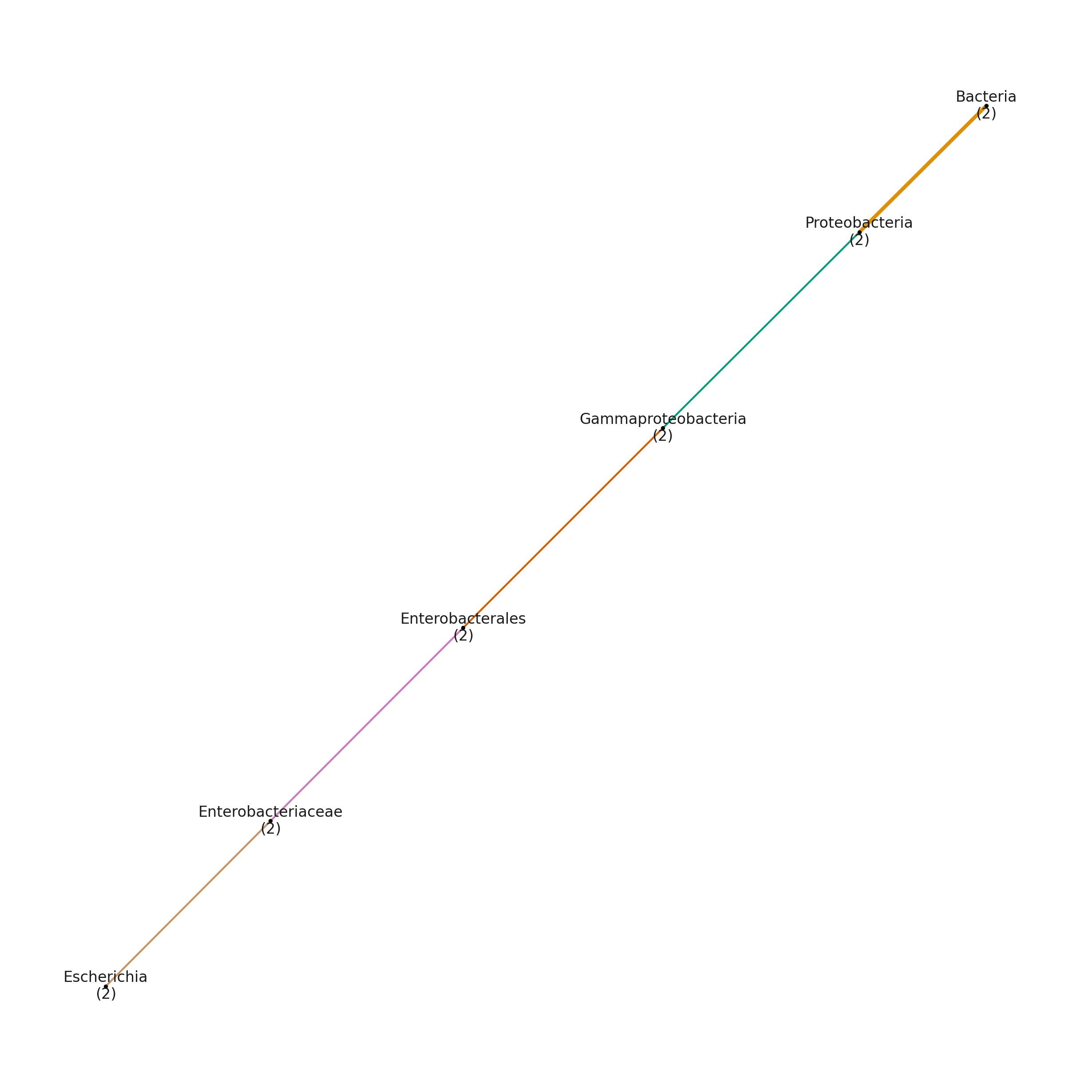

Supplement: Supplementary file 7 — Supplementary Data 4 [file 42003_2023_5076_MOESM7_ESM.zip › 6VXX_A_segment/plots/6VXX_A_site1-metrics-Bacteria-tree.png]

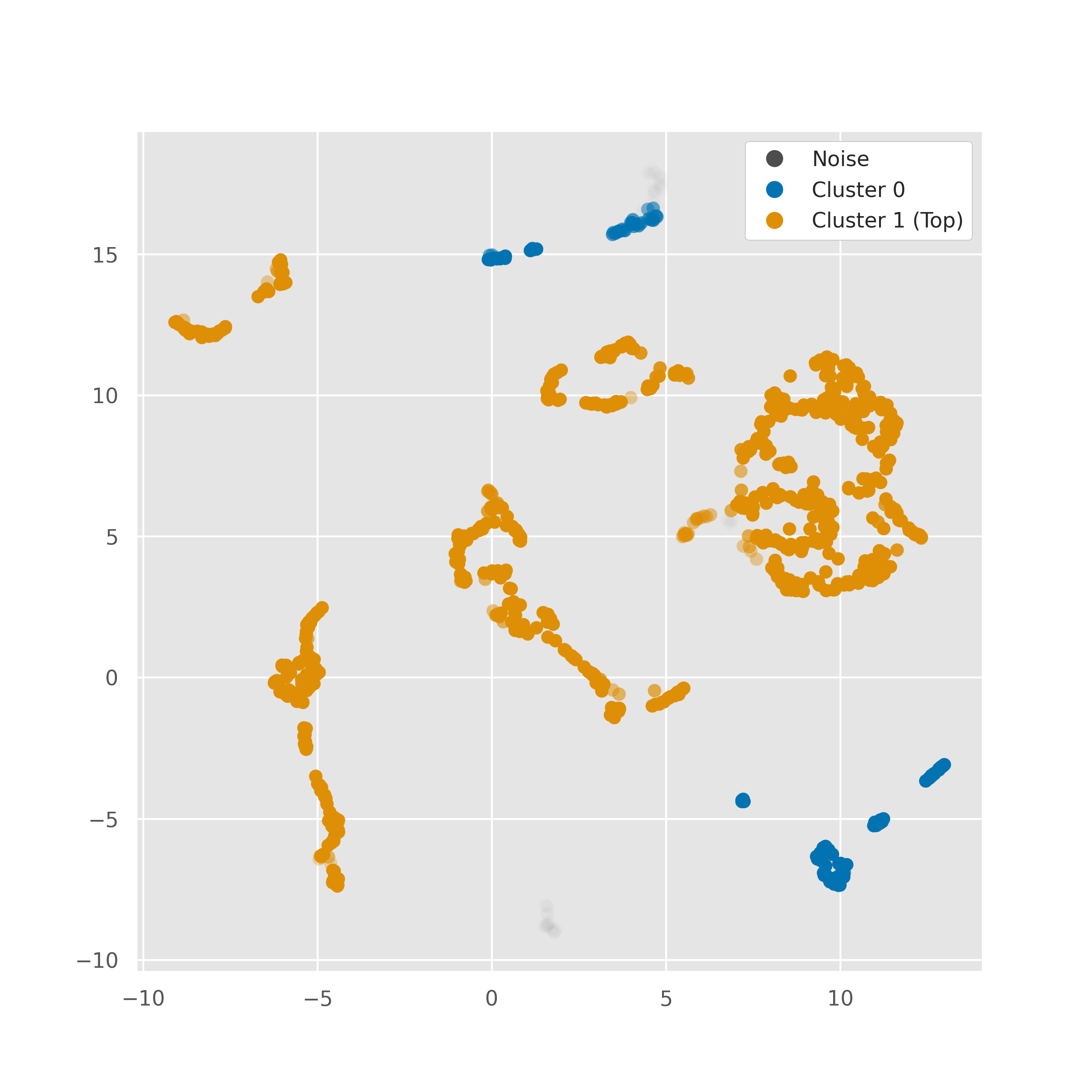

Supplement: Supplementary file 7 — Supplementary Data 4 [file 42003_2023_5076_MOESM7_ESM.zip › 6VXX_A_segment/plots/6VXX_A_site1-metrics-clusters-.png]

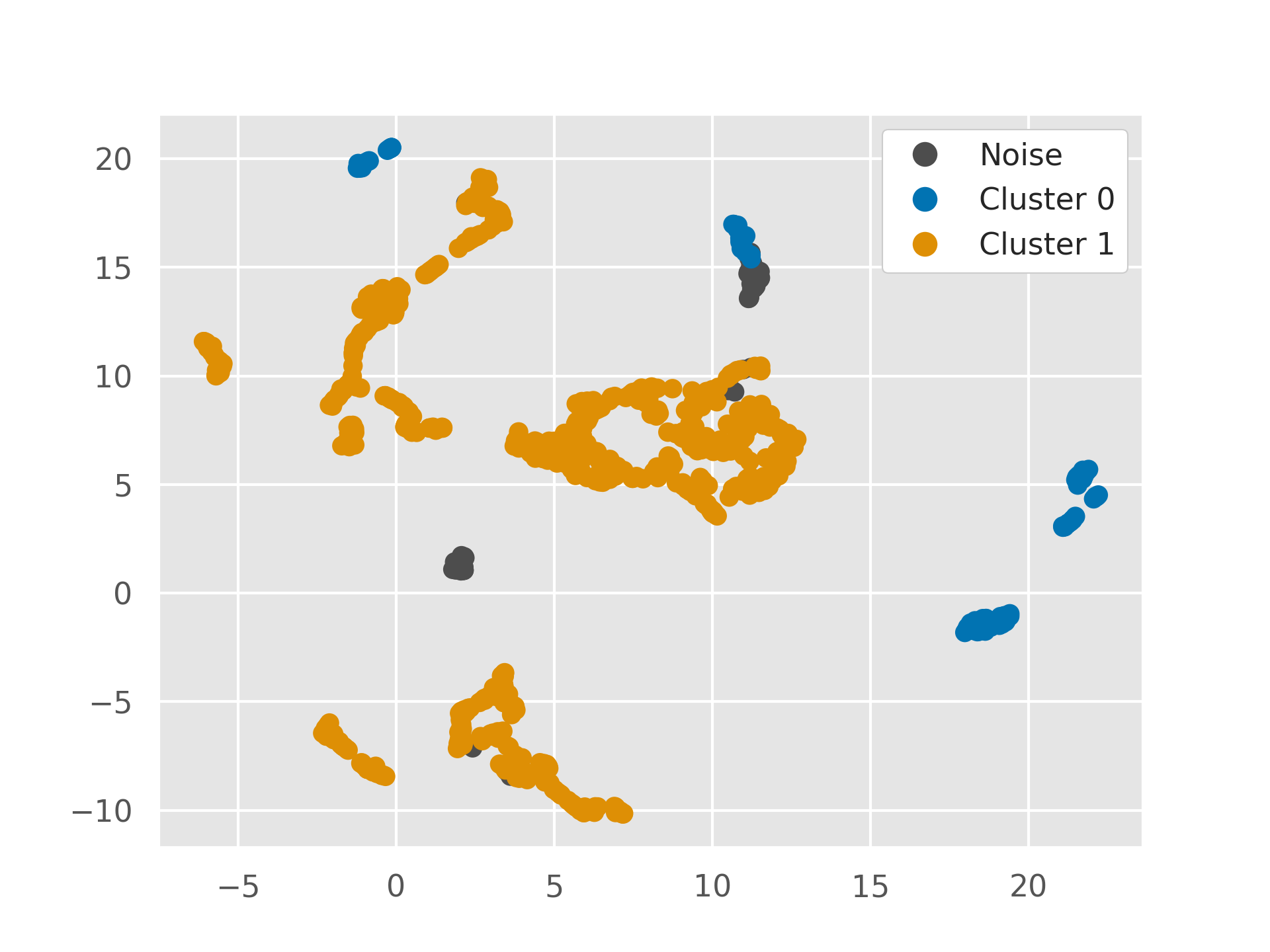

Supplement: Supplementary file 7 — Supplementary Data 4 [file 42003_2023_5076_MOESM7_ESM.zip › 6VXX_A_segment/plots/6VXX_A_site1-metrics-clusters-initial.png]

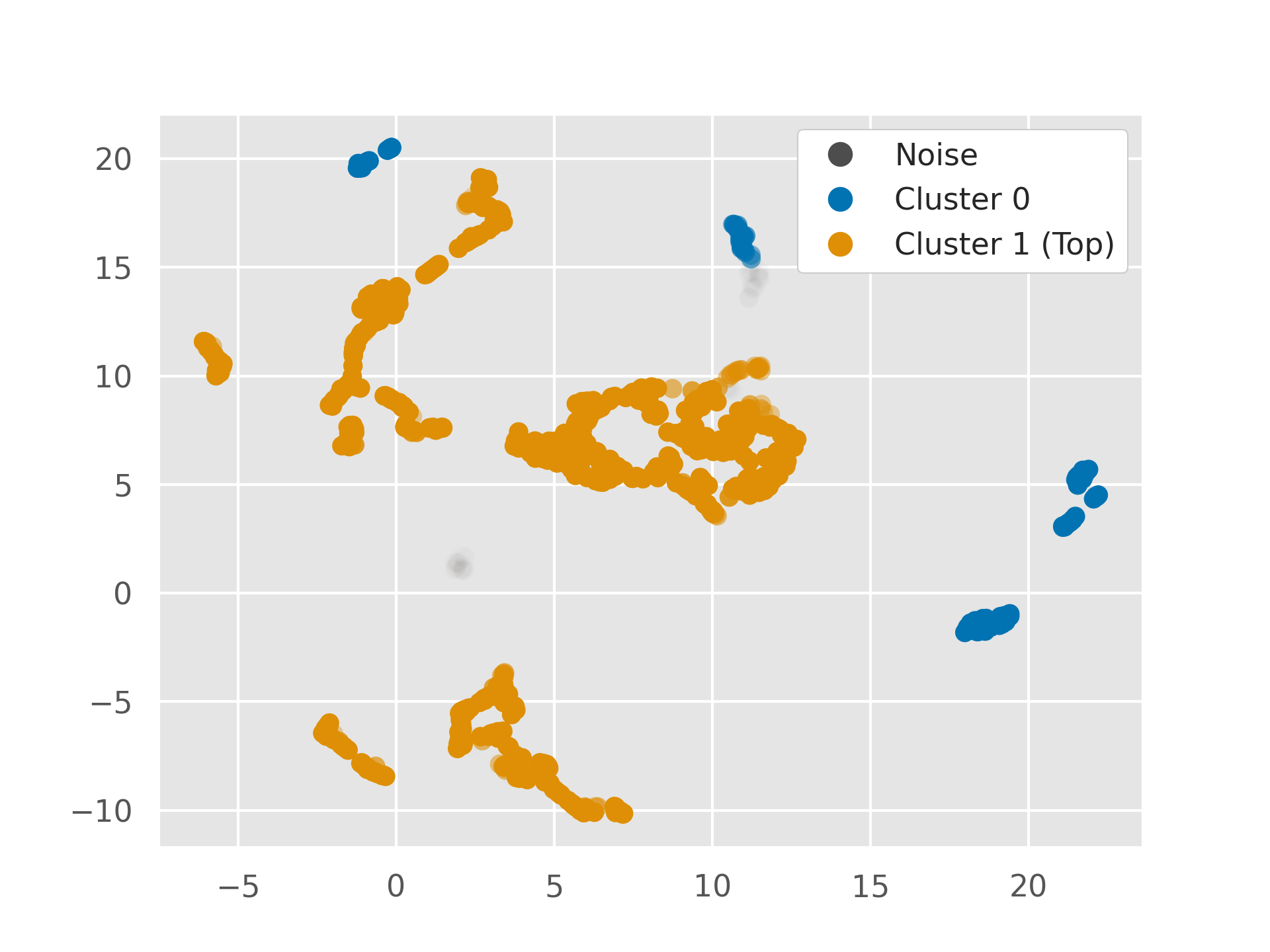

Supplement: Supplementary file 7 — Supplementary Data 4 [file 42003_2023_5076_MOESM7_ESM.zip › 6VXX_A_segment/plots/6VXX_A_site1-metrics-clusters.png]

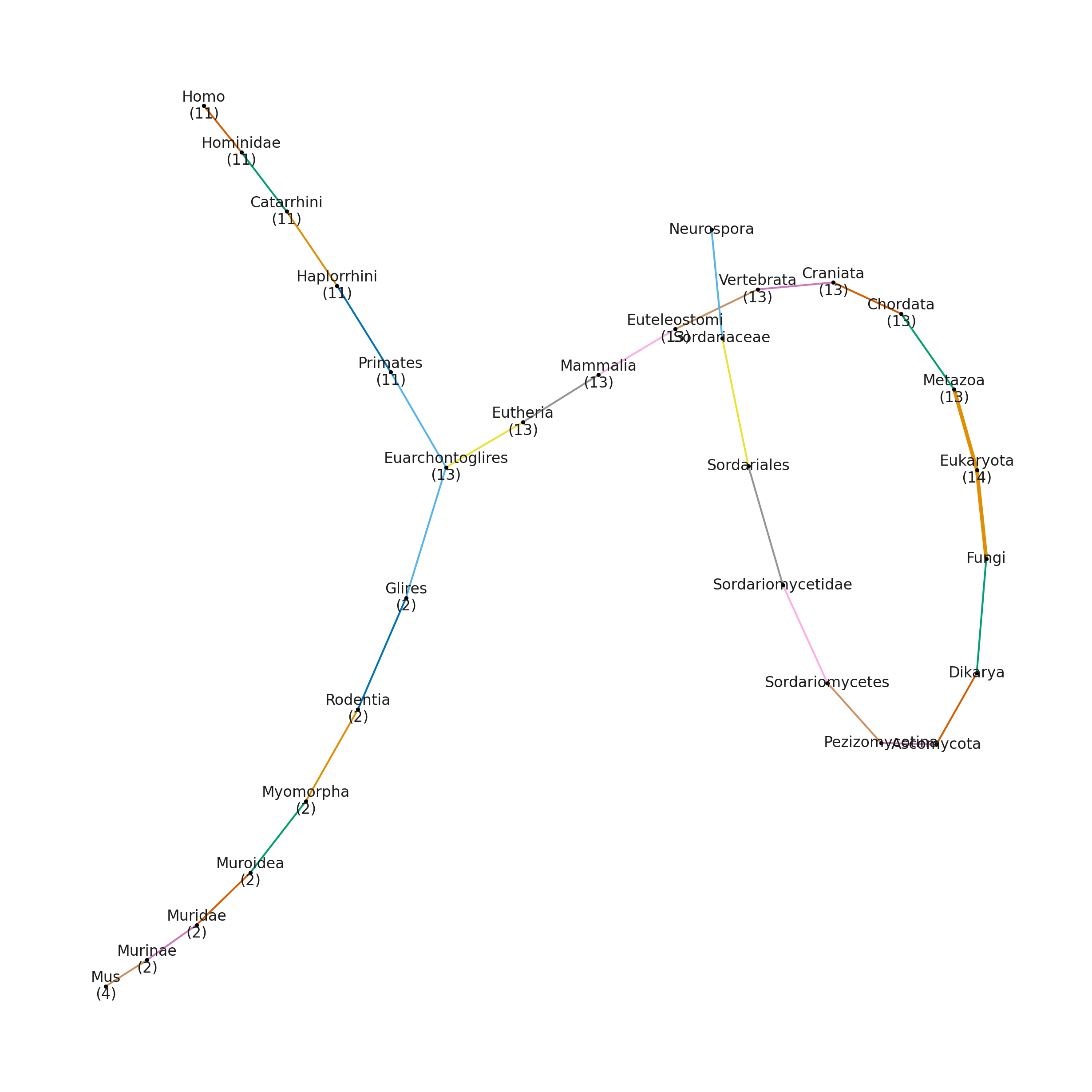

Supplement: Supplementary file 7 — Supplementary Data 4 [file 42003_2023_5076_MOESM7_ESM.zip › 6VXX_A_segment/plots/6VXX_A_site1-metrics-Eukaryota-tree.png]

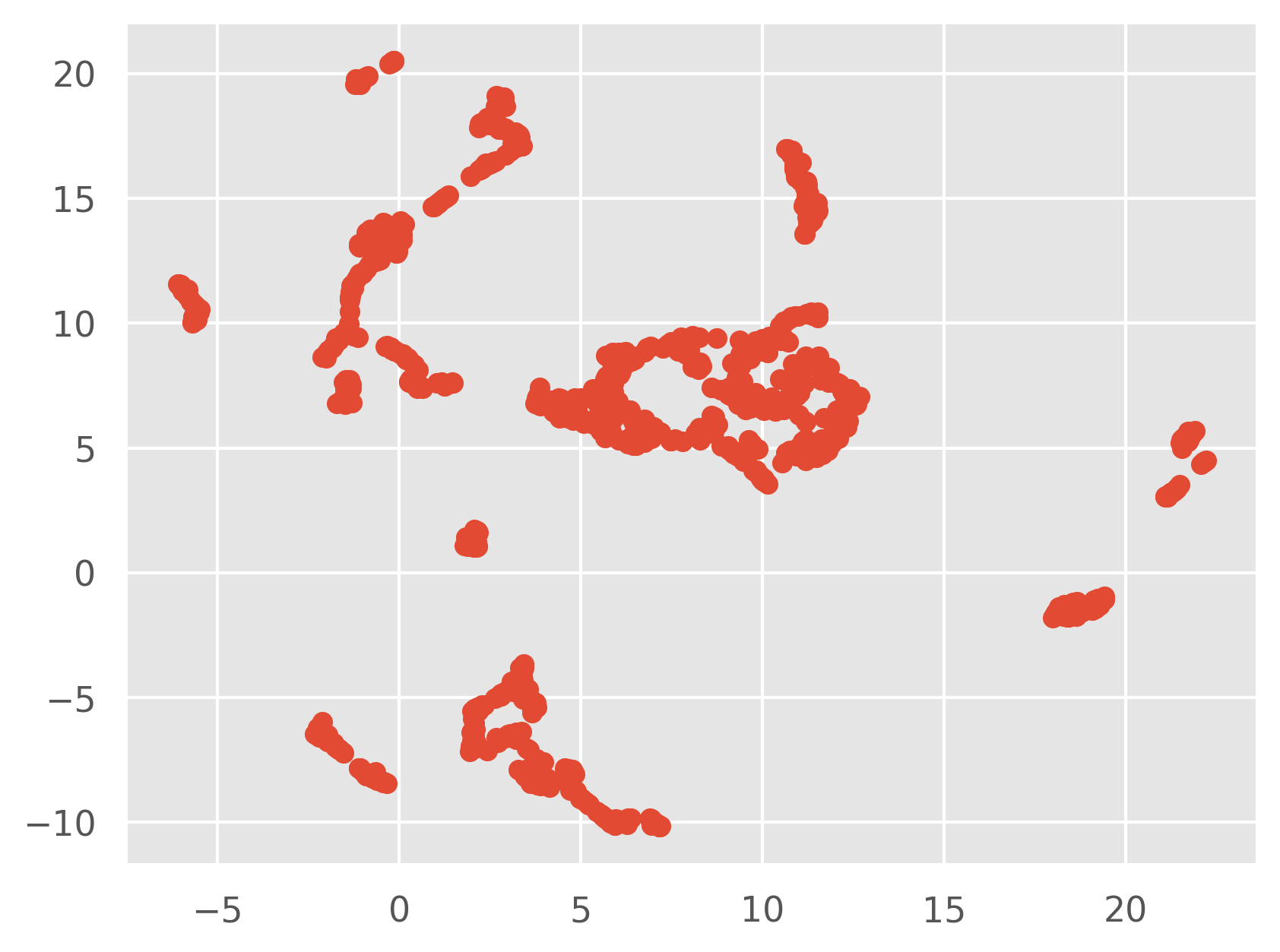

Supplement: Supplementary file 7 — Supplementary Data 4 [file 42003_2023_5076_MOESM7_ESM.zip › 6VXX_A_segment/plots/6VXX_A_site1-metrics-UMAP-.png]

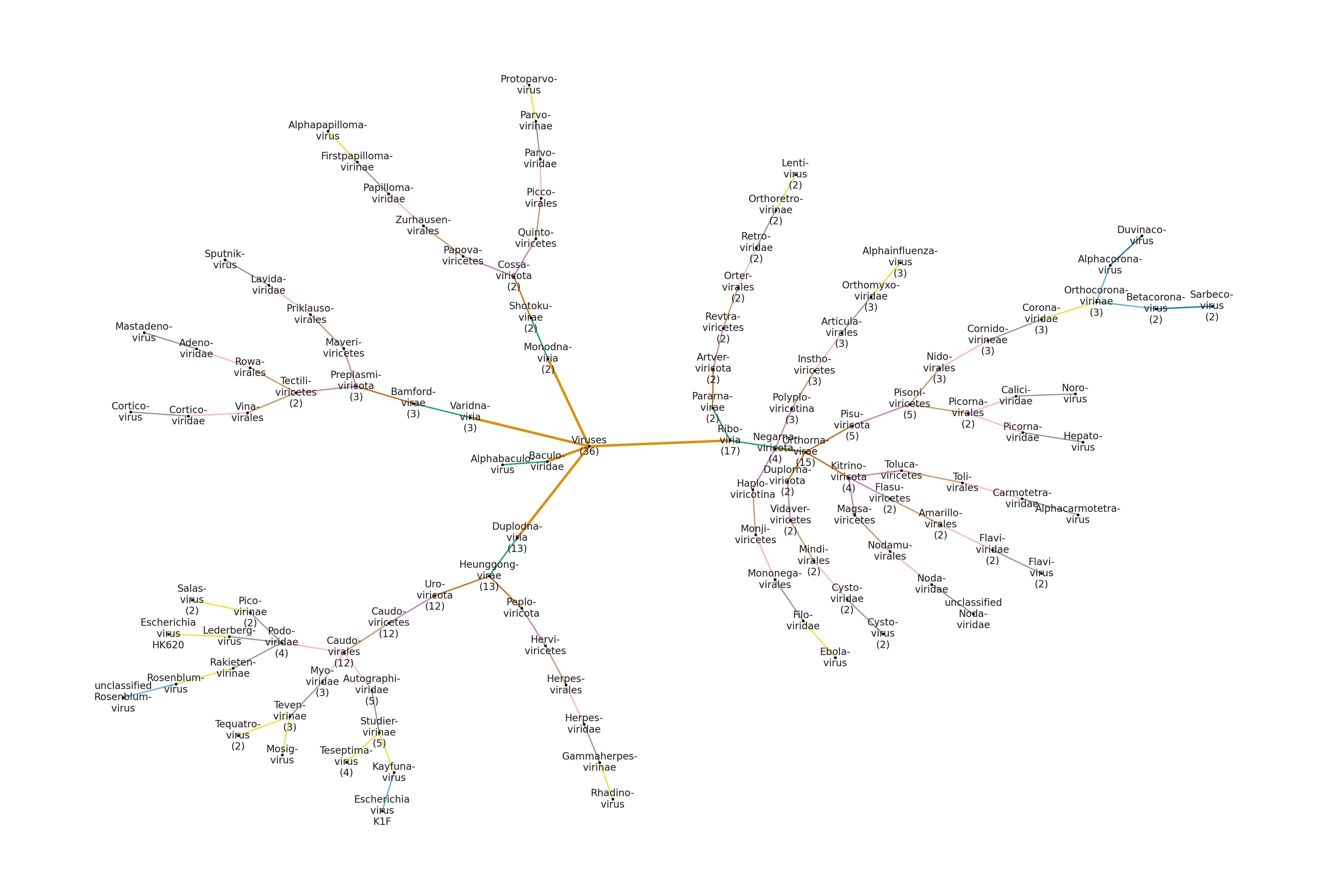

Supplement: Supplementary file 7 — Supplementary Data 4 [file 42003_2023_5076_MOESM7_ESM.zip › 6VXX_A_segment/plots/6VXX_A_site1-metrics-Viruses-tree.png]

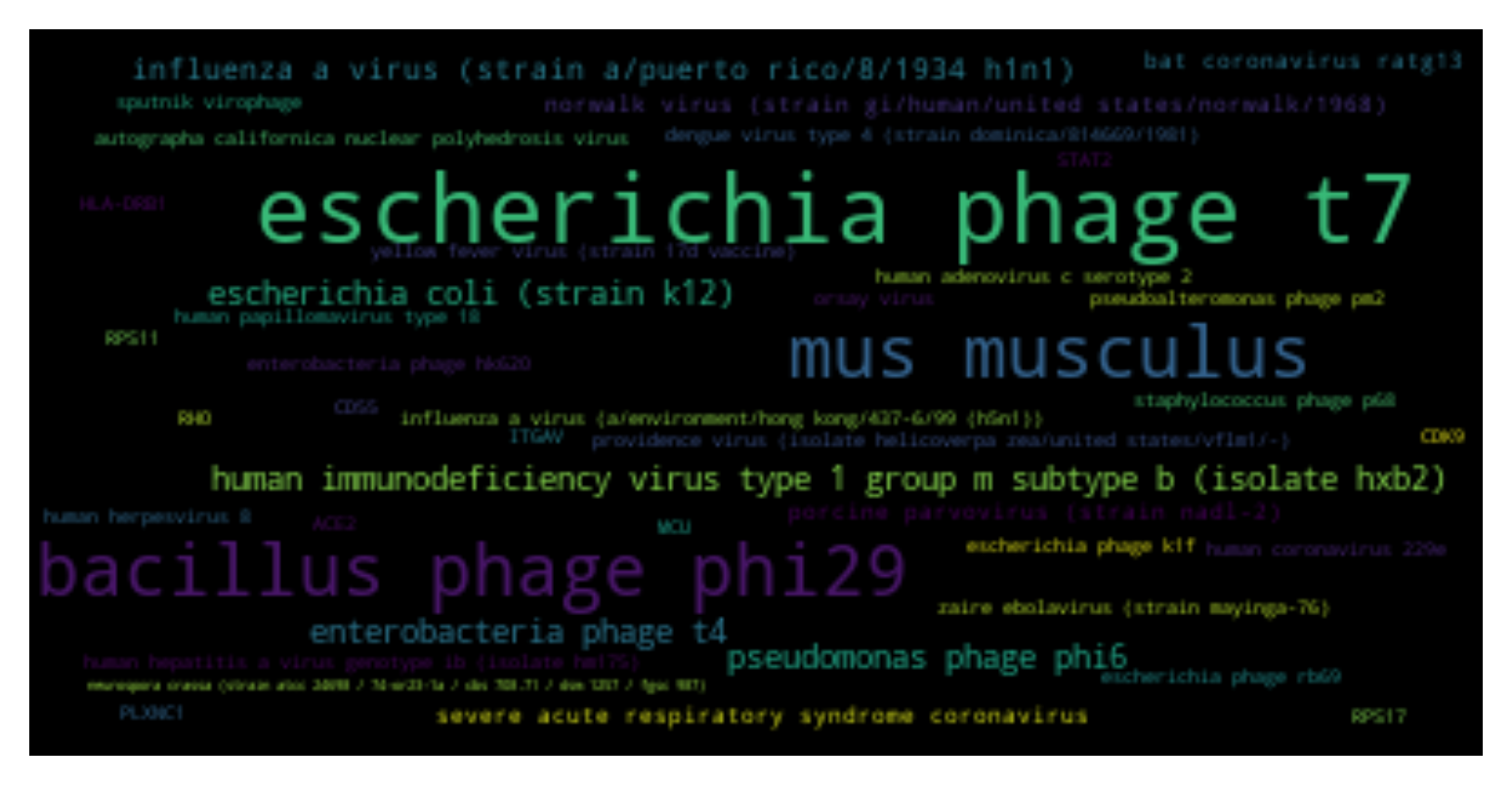

Supplement: Supplementary file 7 — Supplementary Data 4 [file 42003_2023_5076_MOESM7_ESM.zip › 6VXX_A_segment/plots/6VXX_A_site1-metrics-wordcloud.png]

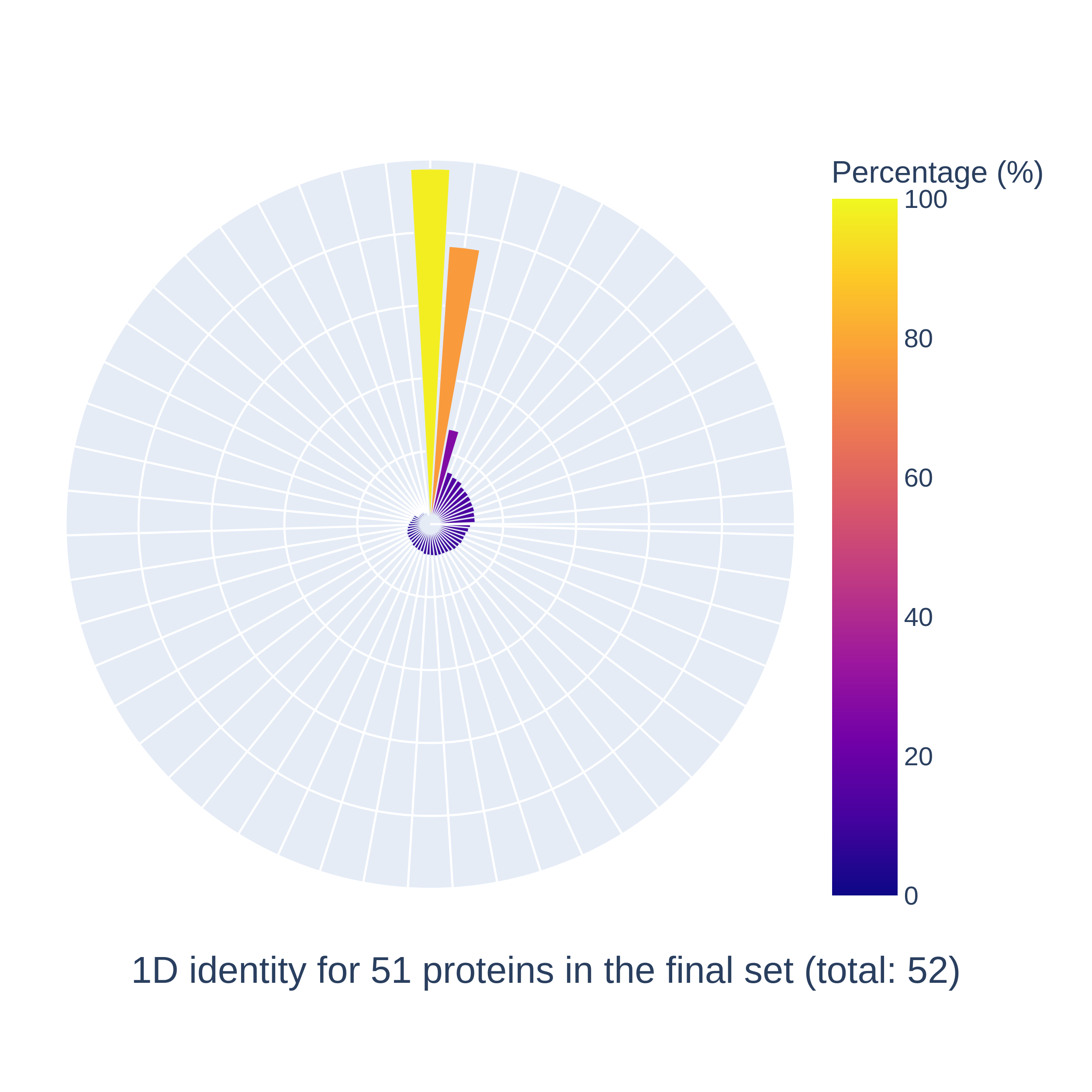

Supplement: Supplementary file 7 — Supplementary Data 4 [file 42003_2023_5076_MOESM7_ESM.zip › 6VXX_A_segment/plots/6VXX_A_site1-metrics_1D-identity.png]

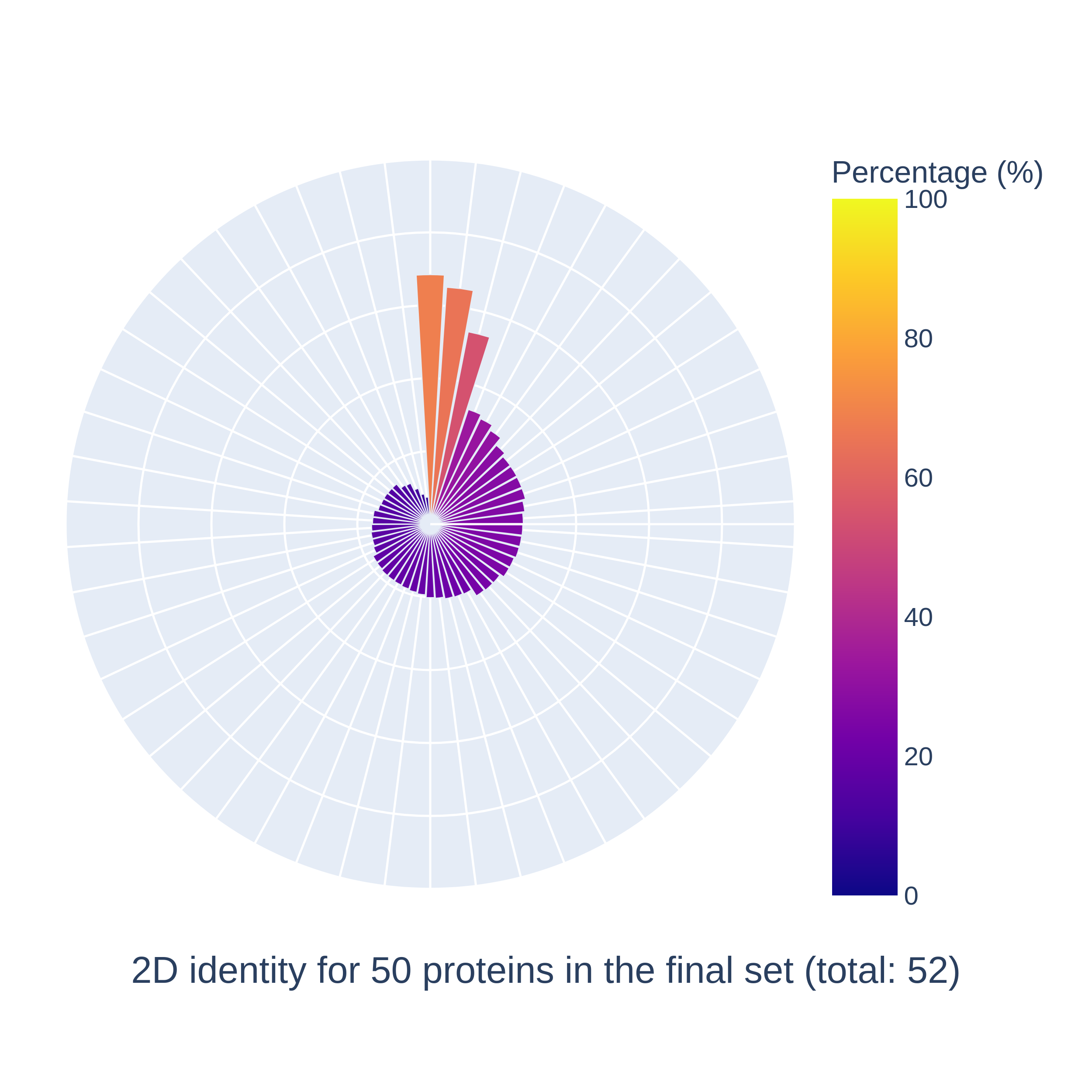

Supplement: Supplementary file 7 — Supplementary Data 4 [file 42003_2023_5076_MOESM7_ESM.zip › 6VXX_A_segment/plots/6VXX_A_site1-metrics_2D-identity.png]

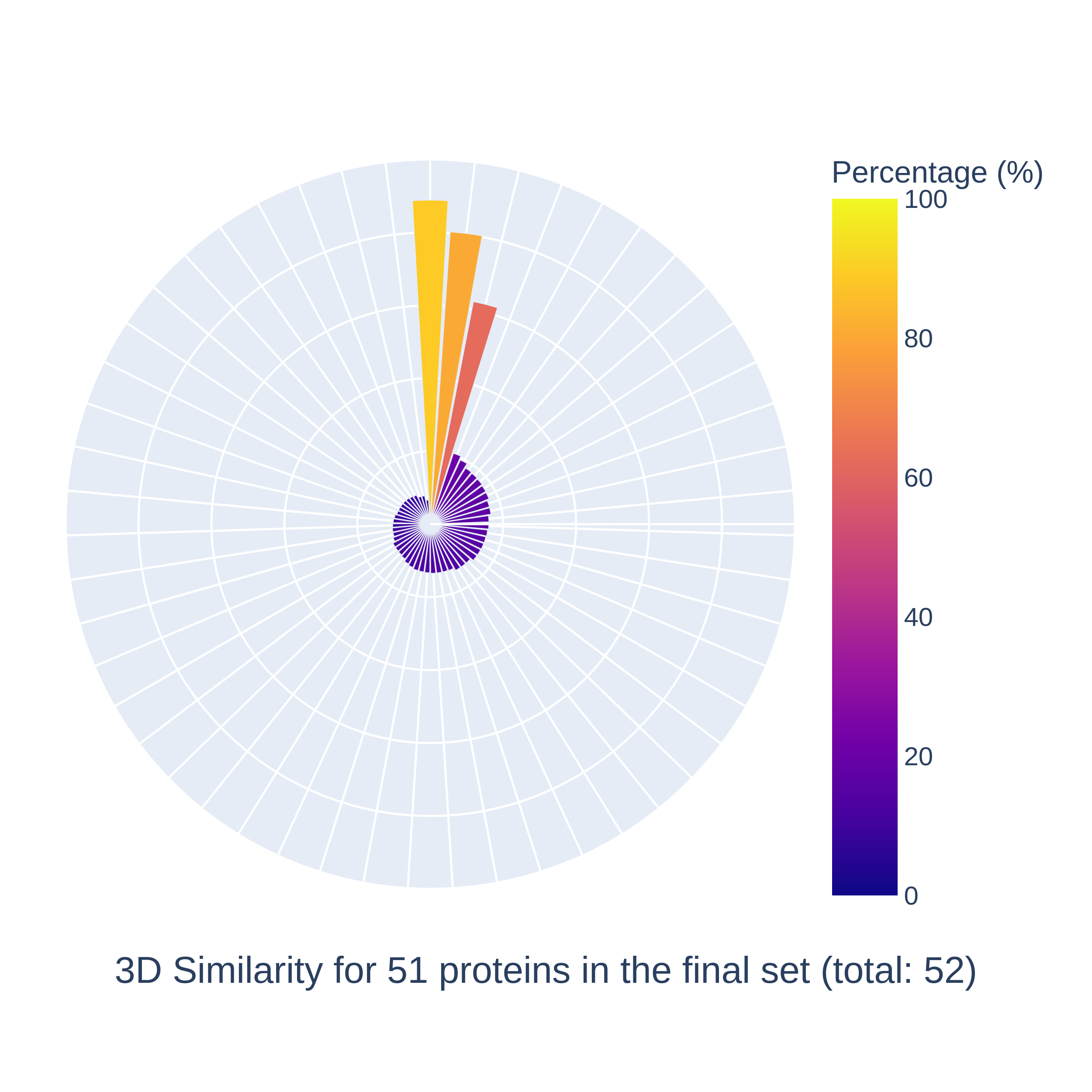

Supplement: Supplementary file 7 — Supplementary Data 4 [file 42003_2023_5076_MOESM7_ESM.zip › 6VXX_A_segment/plots/6VXX_A_site1-metrics_3D-score.png]

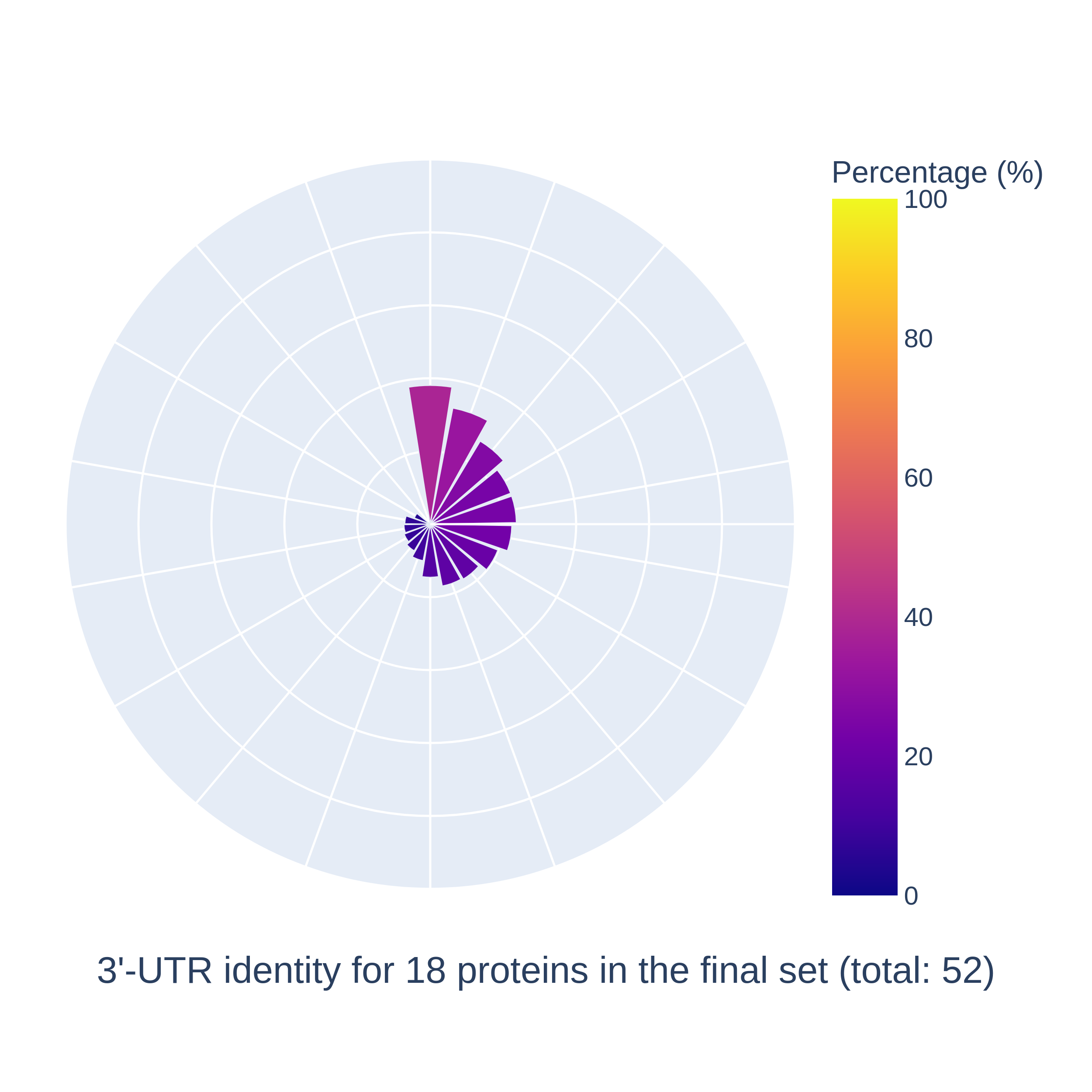

Supplement: Supplementary file 7 — Supplementary Data 4 [file 42003_2023_5076_MOESM7_ESM.zip › 6VXX_A_segment/plots/6VXX_A_site1-metrics_3UTR-identity.png]

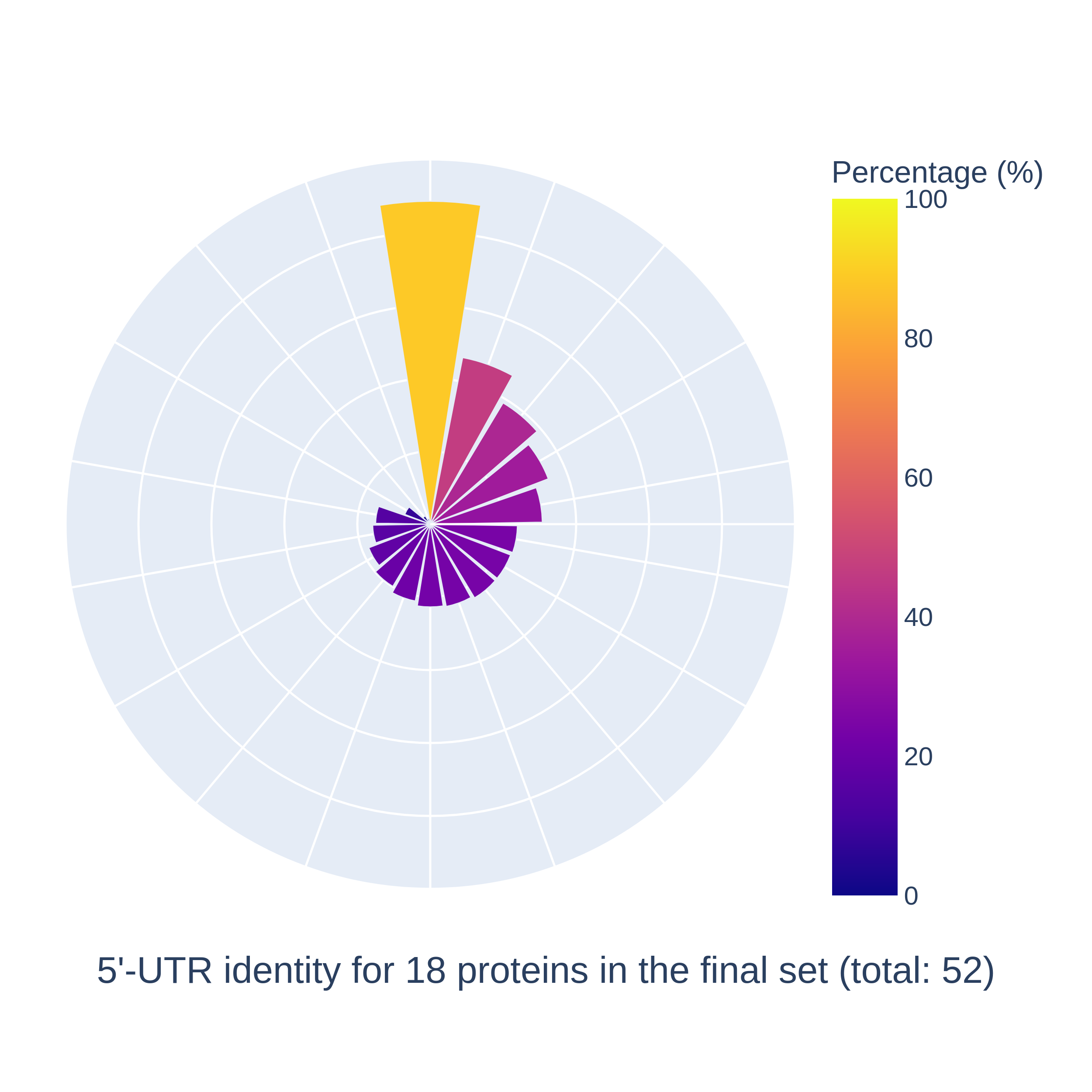

Supplement: Supplementary file 7 — Supplementary Data 4 [file 42003_2023_5076_MOESM7_ESM.zip › 6VXX_A_segment/plots/6VXX_A_site1-metrics_5UTR-identity.png]

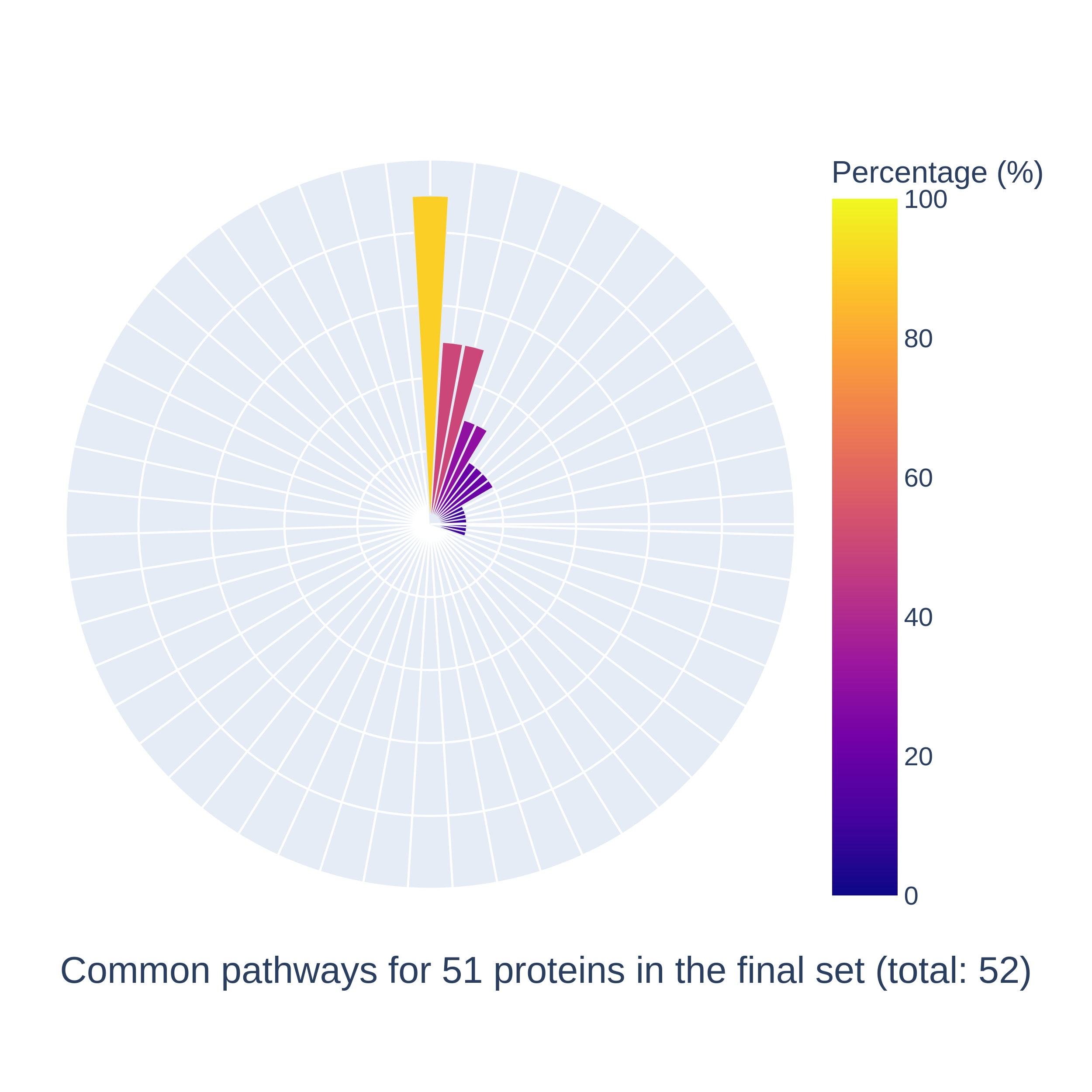

Supplement: Supplementary file 7 — Supplementary Data 4 [file 42003_2023_5076_MOESM7_ESM.zip › 6VXX_A_segment/plots/6VXX_A_site1-metrics_biologicalProcessSim.png]

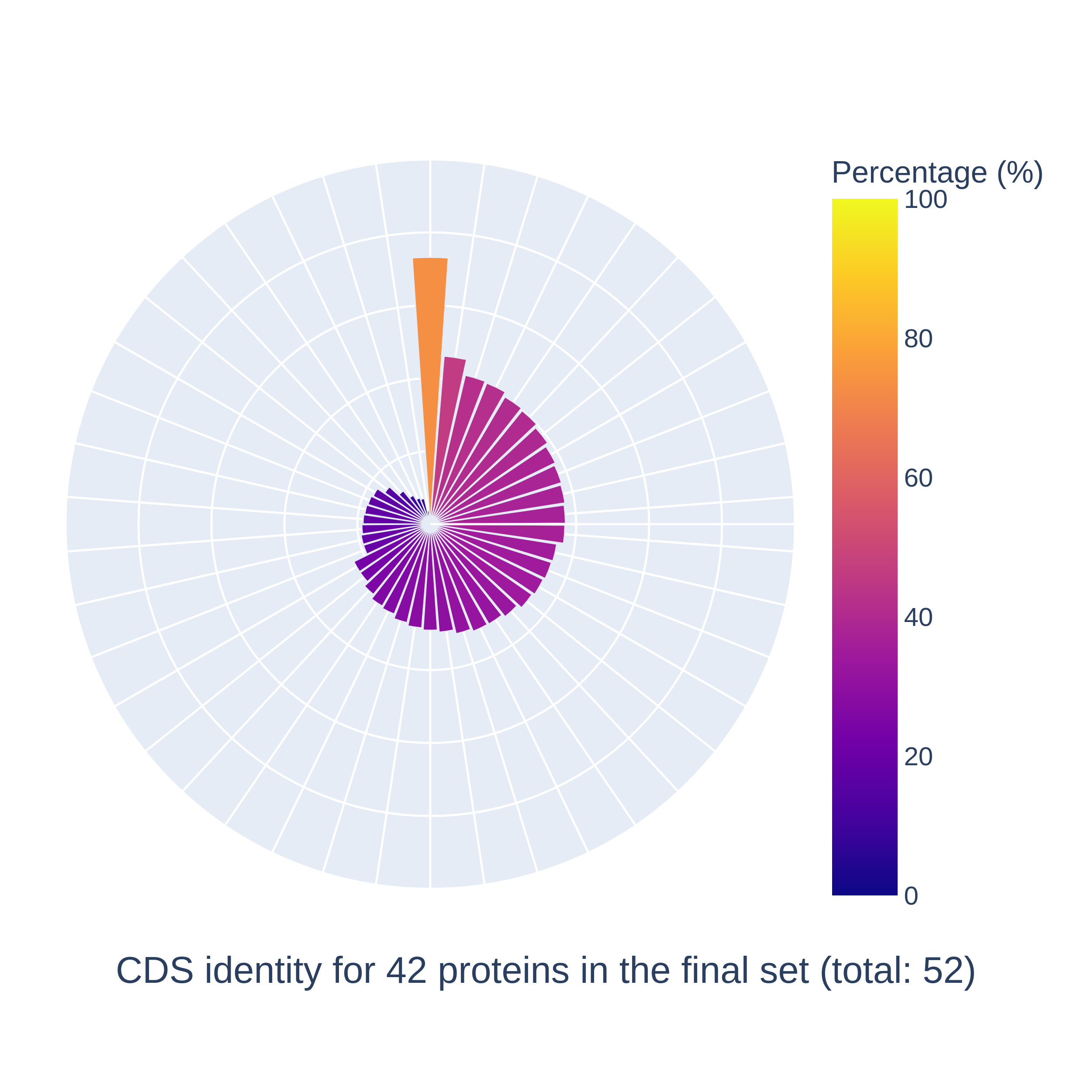

Supplement: Supplementary file 7 — Supplementary Data 4 [file 42003_2023_5076_MOESM7_ESM.zip › 6VXX_A_segment/plots/6VXX_A_site1-metrics_CDS-identity.png]

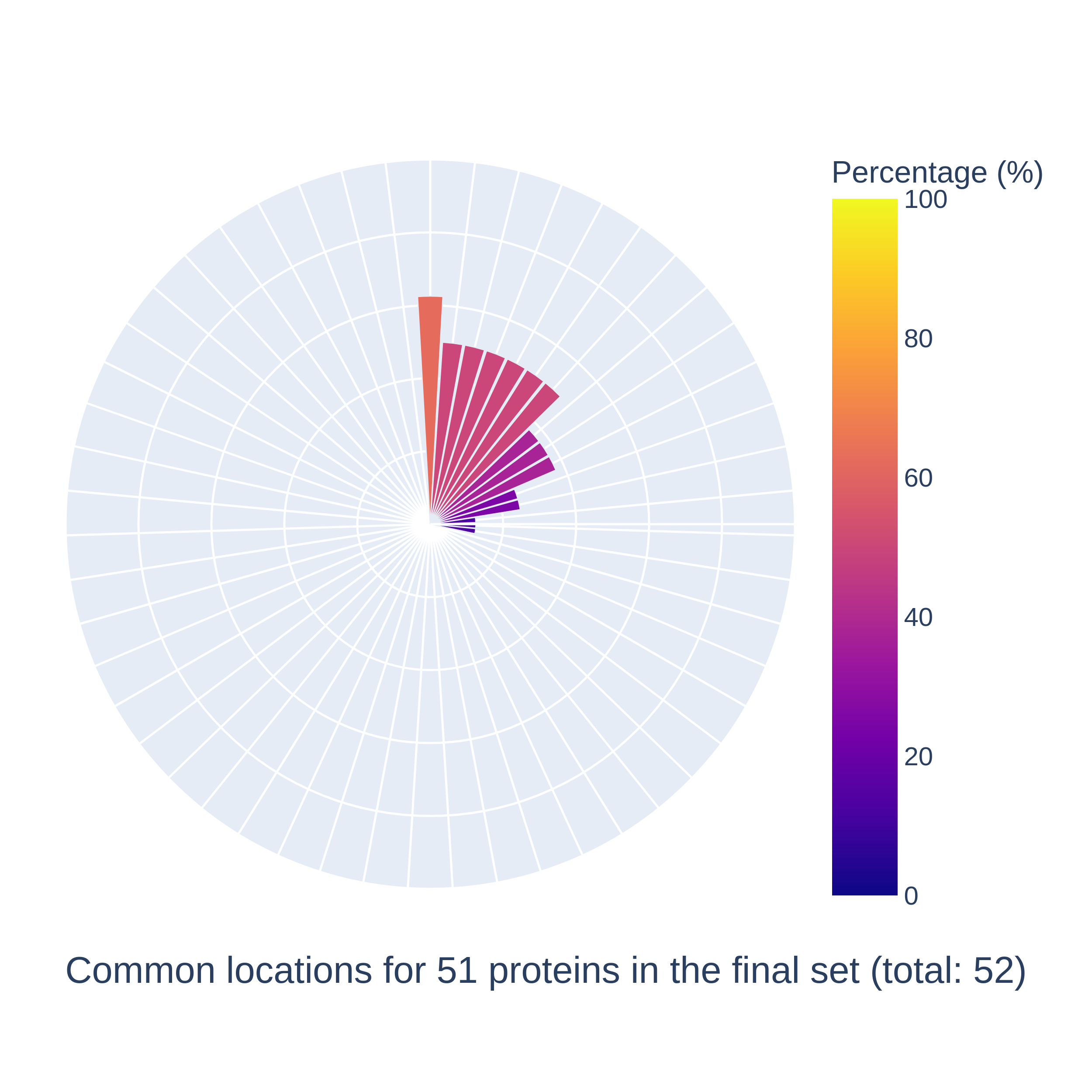

Supplement: Supplementary file 7 — Supplementary Data 4 [file 42003_2023_5076_MOESM7_ESM.zip › 6VXX_A_segment/plots/6VXX_A_site1-metrics_cellularComponentSim.png]

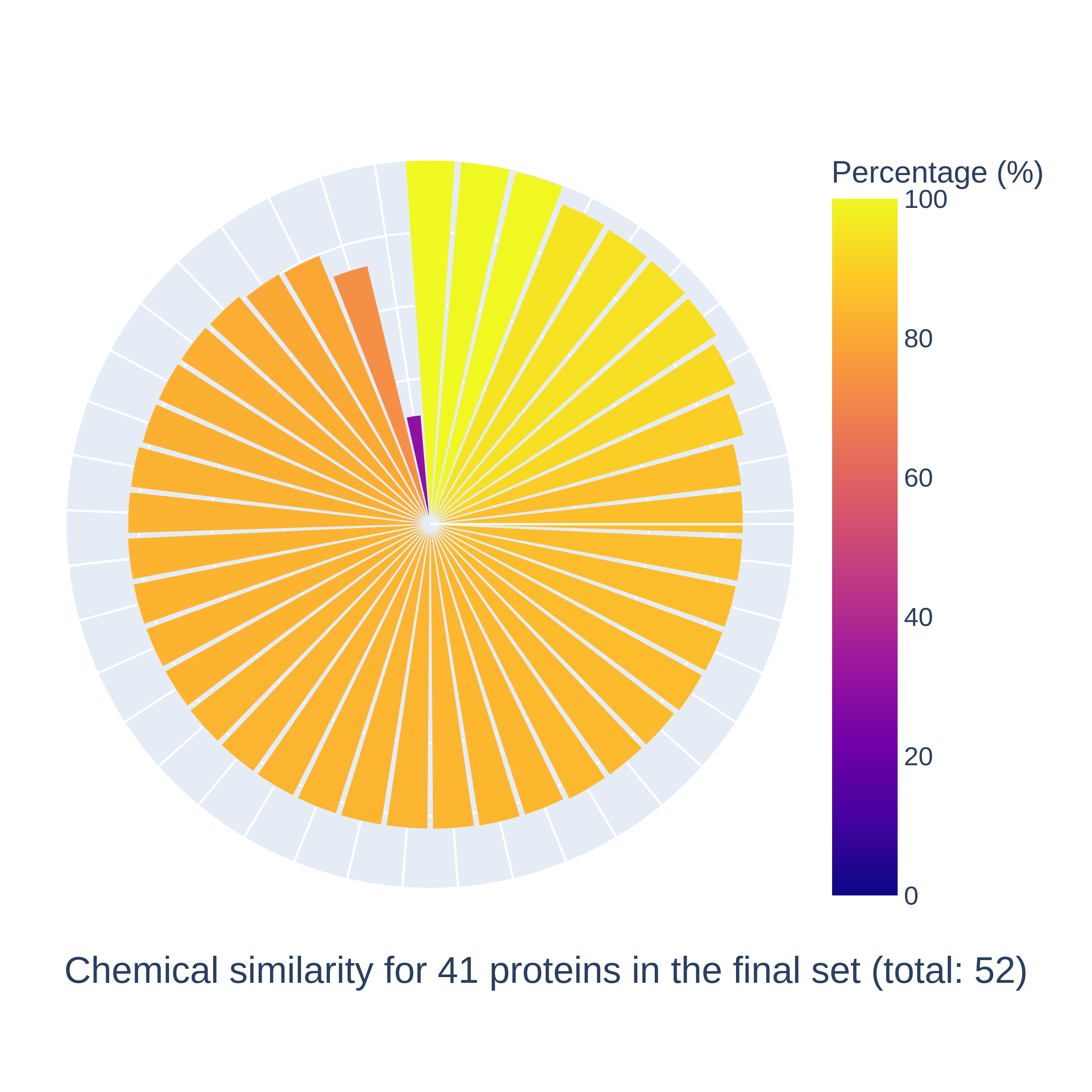

Supplement: Supplementary file 7 — Supplementary Data 4 [file 42003_2023_5076_MOESM7_ESM.zip › 6VXX_A_segment/plots/6VXX_A_site1-metrics_chemSim.png]

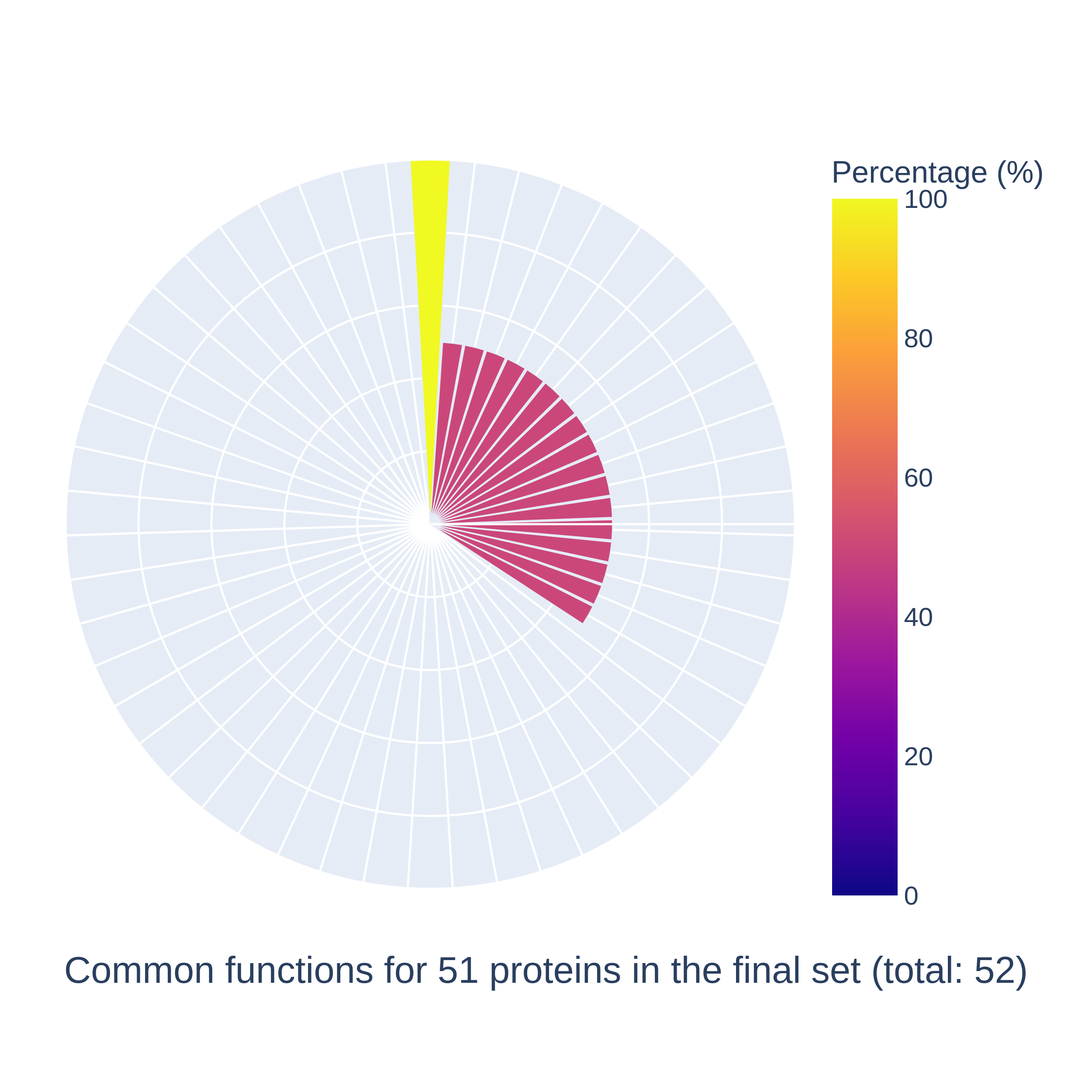

Supplement: Supplementary file 7 — Supplementary Data 4 [file 42003_2023_5076_MOESM7_ESM.zip › 6VXX_A_segment/plots/6VXX_A_site1-metrics_molecularFunctionSim.png]

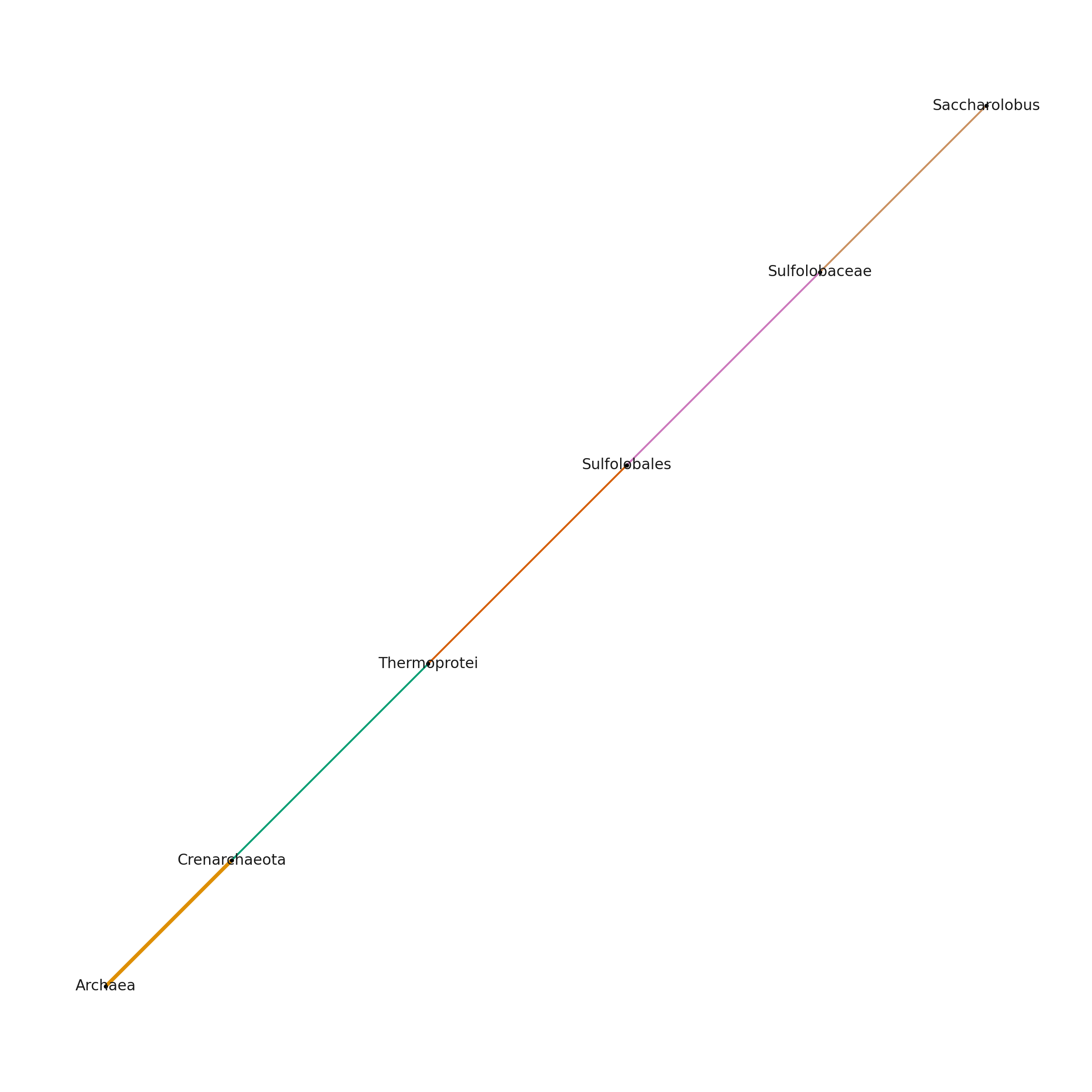

Supplement: Supplementary file 7 — Supplementary Data 4 [file 42003_2023_5076_MOESM7_ESM.zip › 6VXX_A_segment/plots/6VXX_A_site2-metrics-Archaea-tree.png]

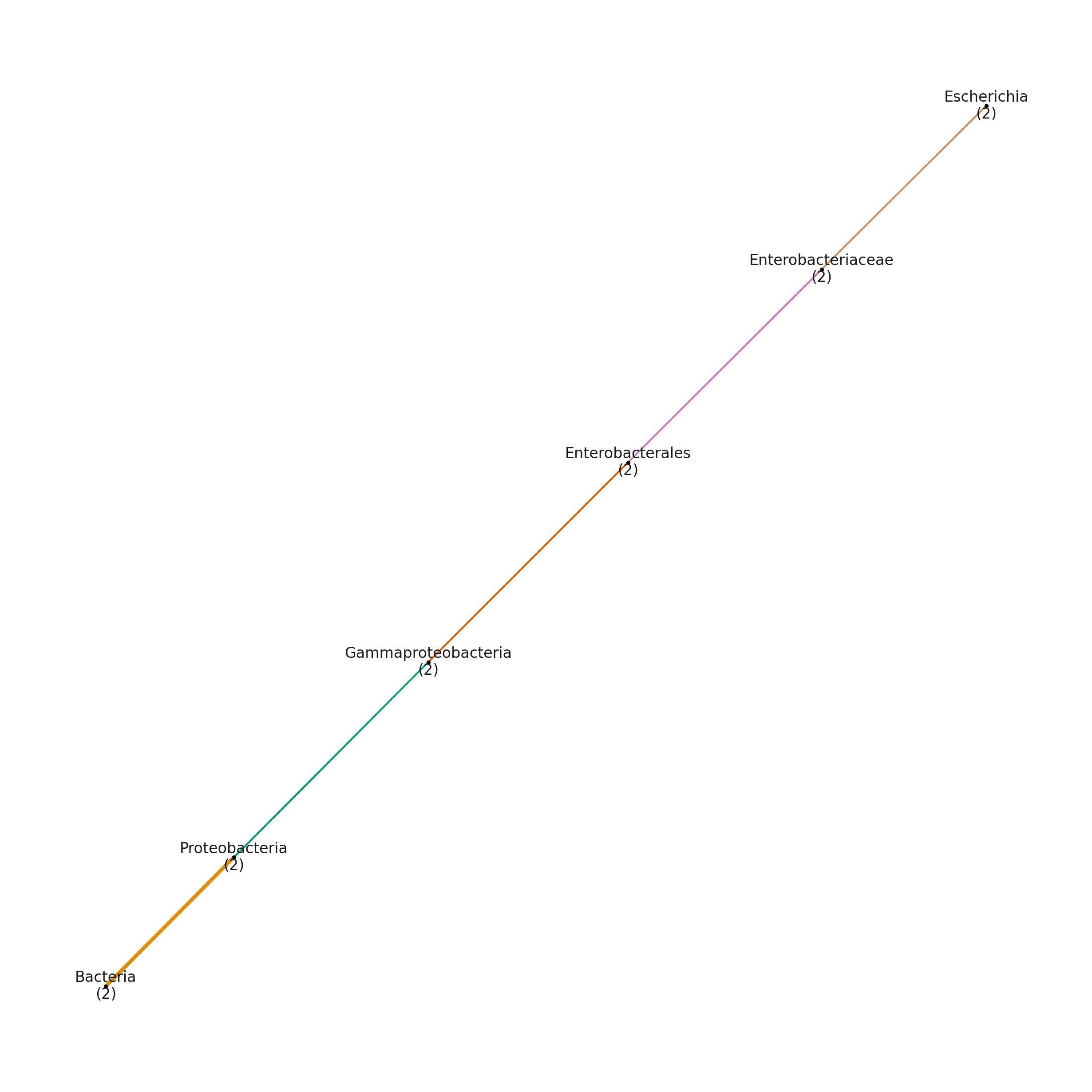

Supplement: Supplementary file 7 — Supplementary Data 4 [file 42003_2023_5076_MOESM7_ESM.zip › 6VXX_A_segment/plots/6VXX_A_site2-metrics-Bacteria-tree.png]

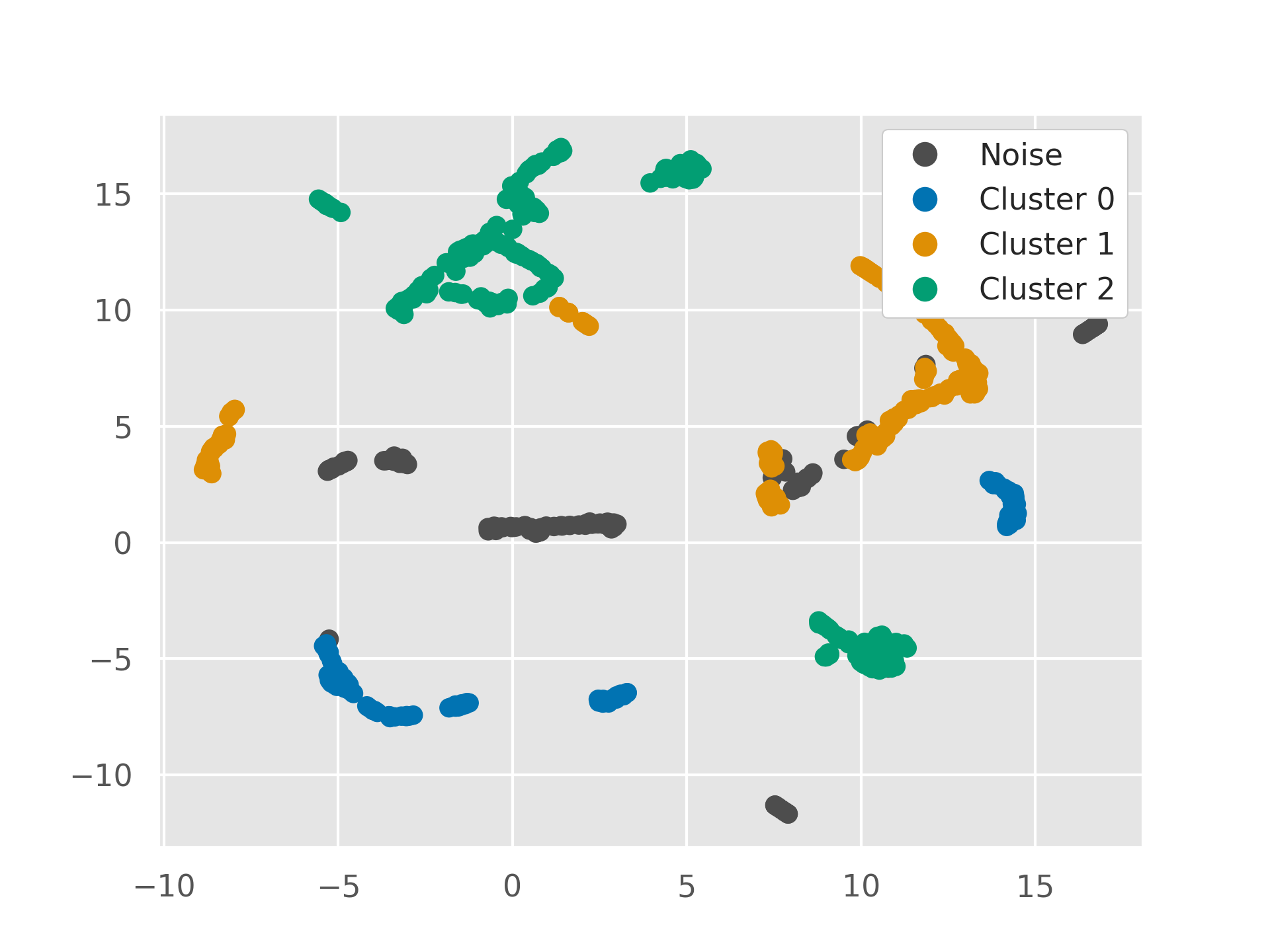

Supplement: Supplementary file 7 — Supplementary Data 4 [file 42003_2023_5076_MOESM7_ESM.zip › 6VXX_A_segment/plots/6VXX_A_site2-metrics-clusters-initial.png]

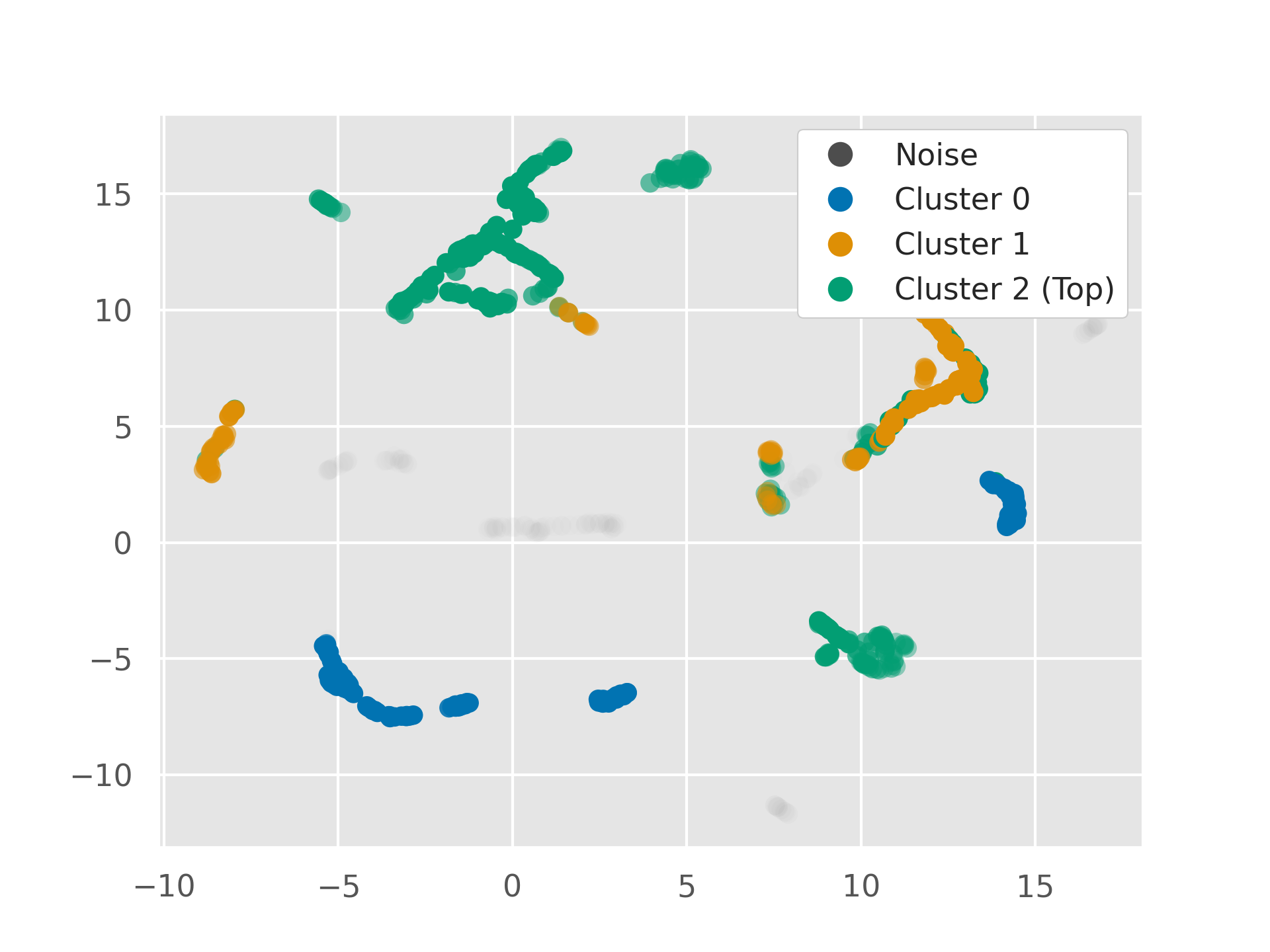

Supplement: Supplementary file 7 — Supplementary Data 4 [file 42003_2023_5076_MOESM7_ESM.zip › 6VXX_A_segment/plots/6VXX_A_site2-metrics-clusters.png]

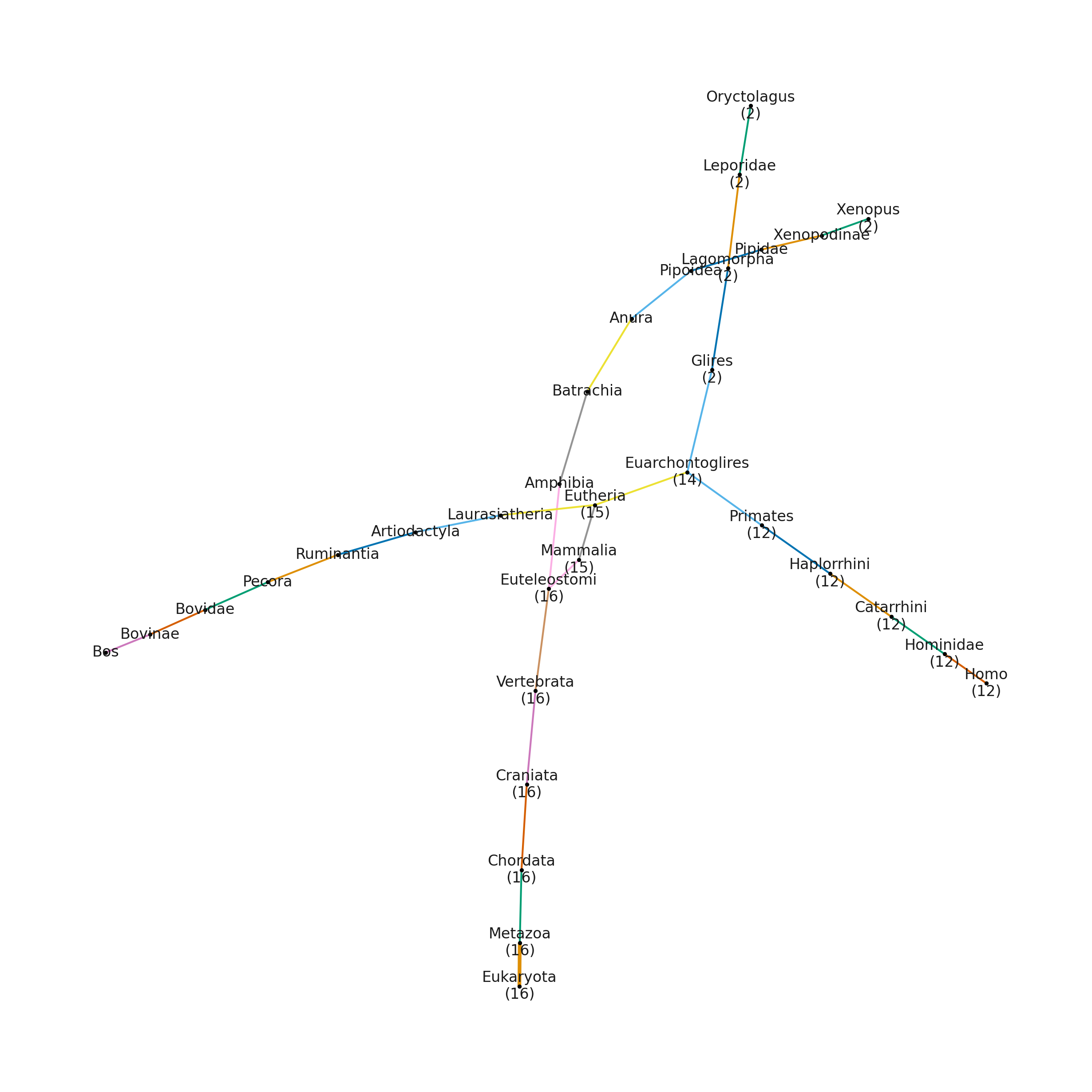

Supplement: Supplementary file 7 — Supplementary Data 4 [file 42003_2023_5076_MOESM7_ESM.zip › 6VXX_A_segment/plots/6VXX_A_site2-metrics-Eukaryota-tree.png]

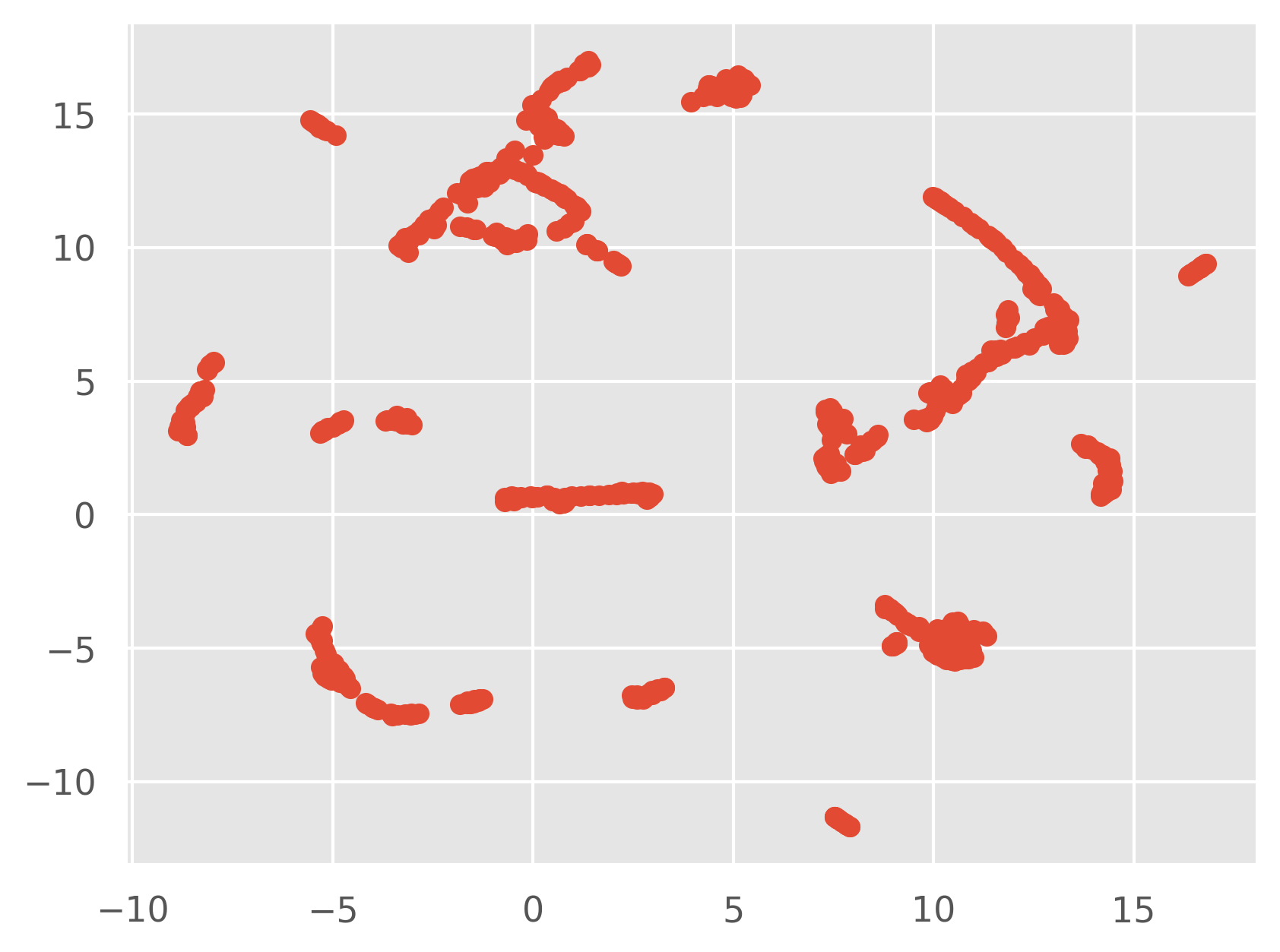

Supplement: Supplementary file 7 — Supplementary Data 4 [file 42003_2023_5076_MOESM7_ESM.zip › 6VXX_A_segment/plots/6VXX_A_site2-metrics-UMAP-.png]

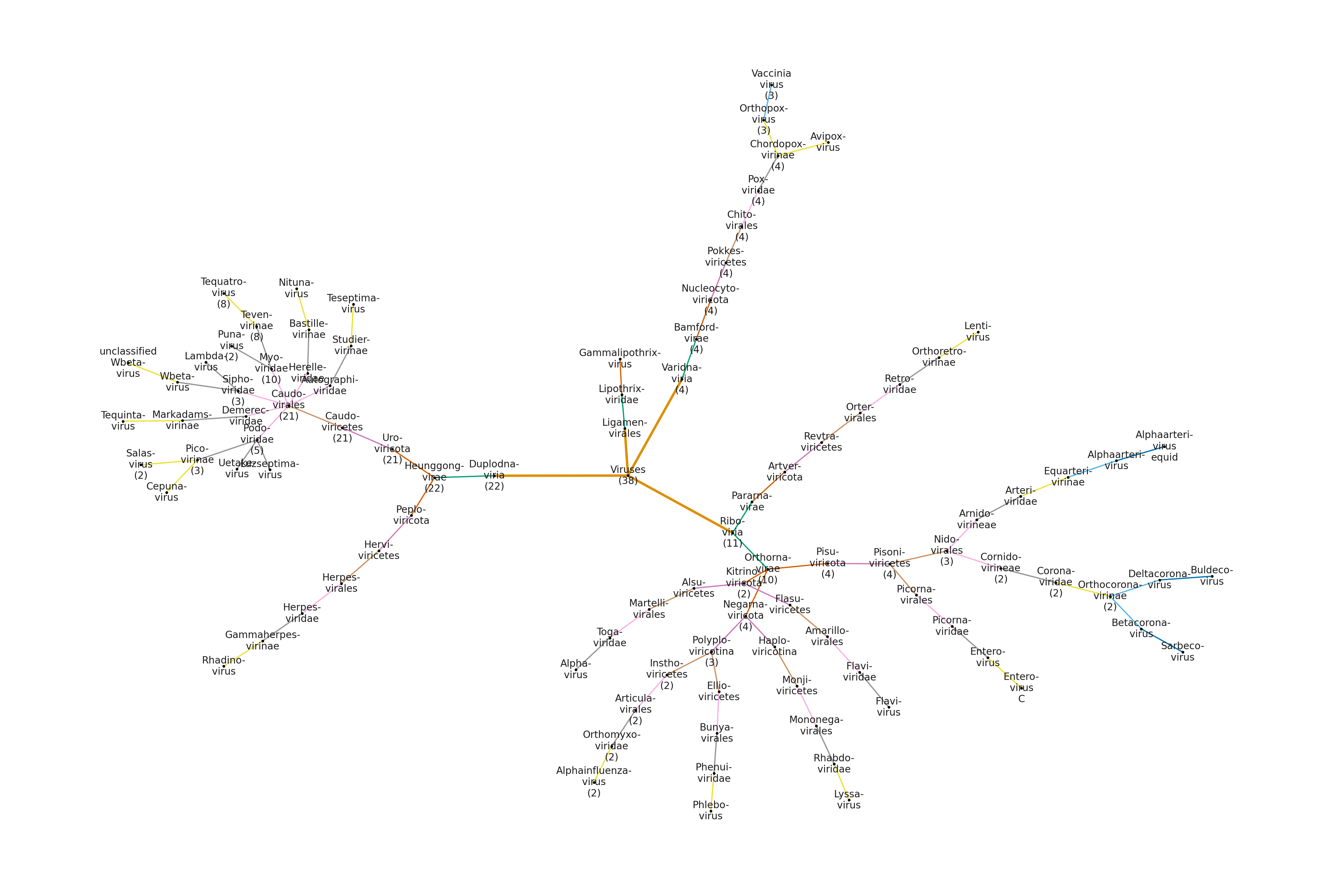

Supplement: Supplementary file 7 — Supplementary Data 4 [file 42003_2023_5076_MOESM7_ESM.zip › 6VXX_A_segment/plots/6VXX_A_site2-metrics-Viruses-tree.png]

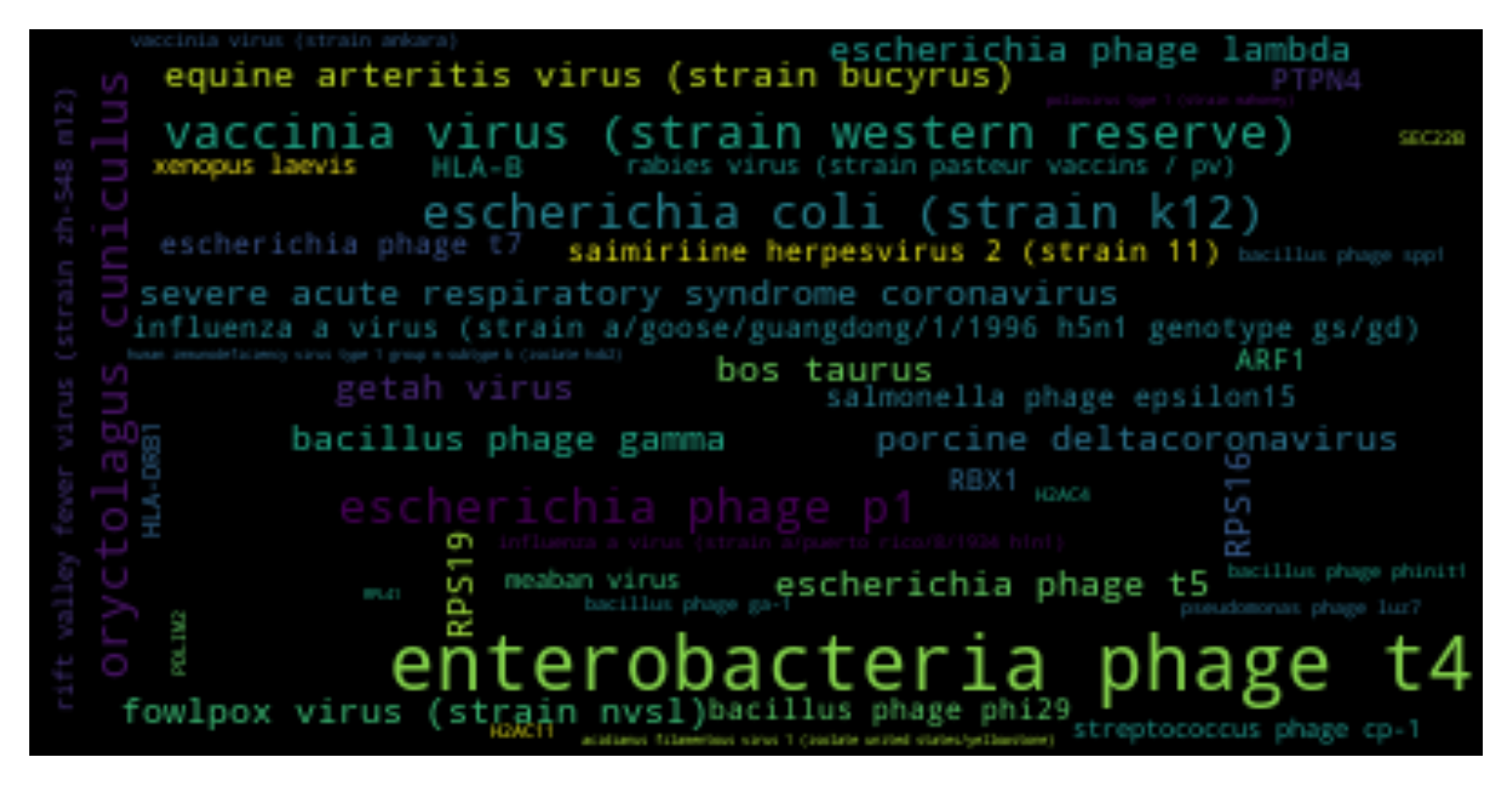

Supplement: Supplementary file 7 — Supplementary Data 4 [file 42003_2023_5076_MOESM7_ESM.zip › 6VXX_A_segment/plots/6VXX_A_site2-metrics-wordcloud.png]

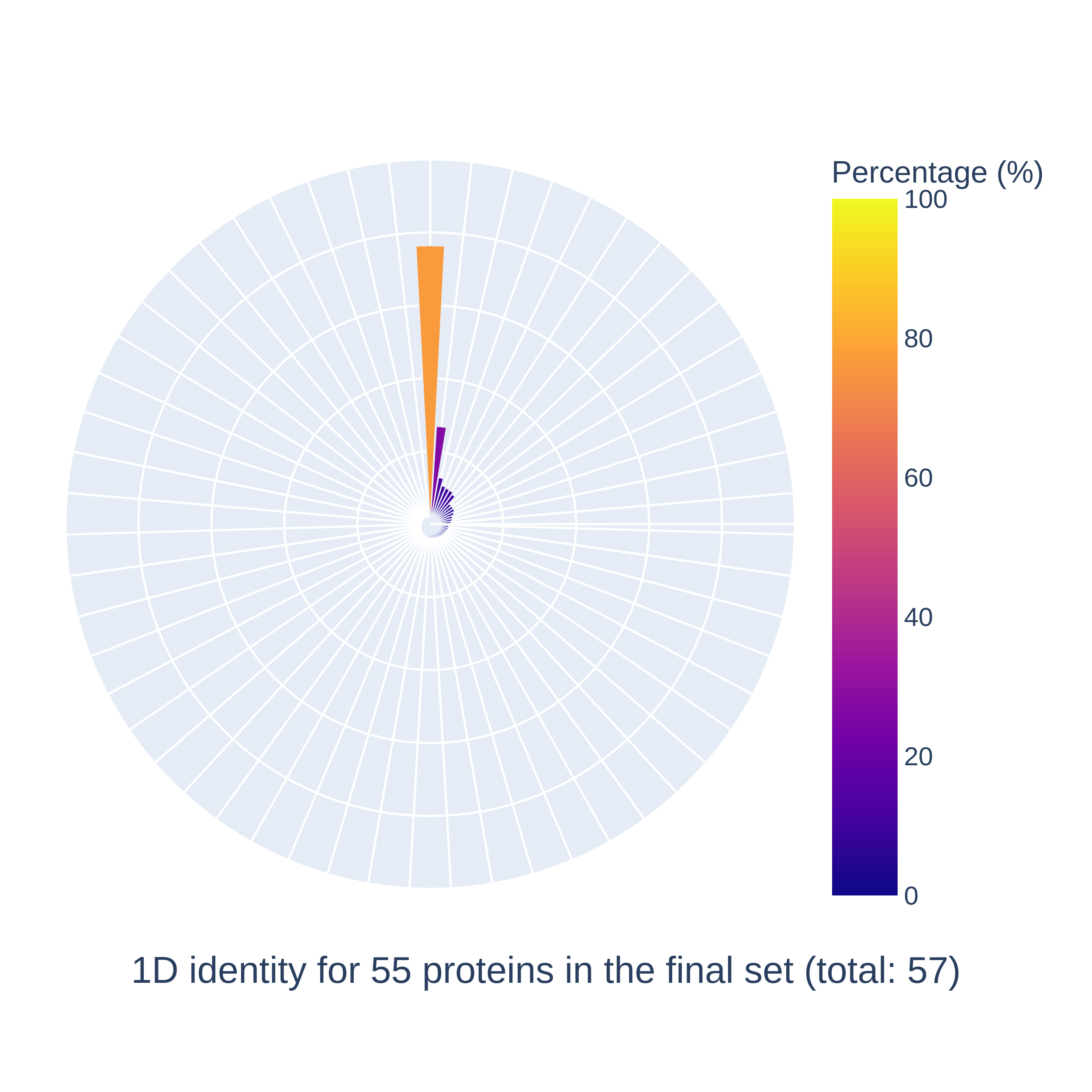

Supplement: Supplementary file 7 — Supplementary Data 4 [file 42003_2023_5076_MOESM7_ESM.zip › 6VXX_A_segment/plots/6VXX_A_site2-metrics_1D-identity.png]

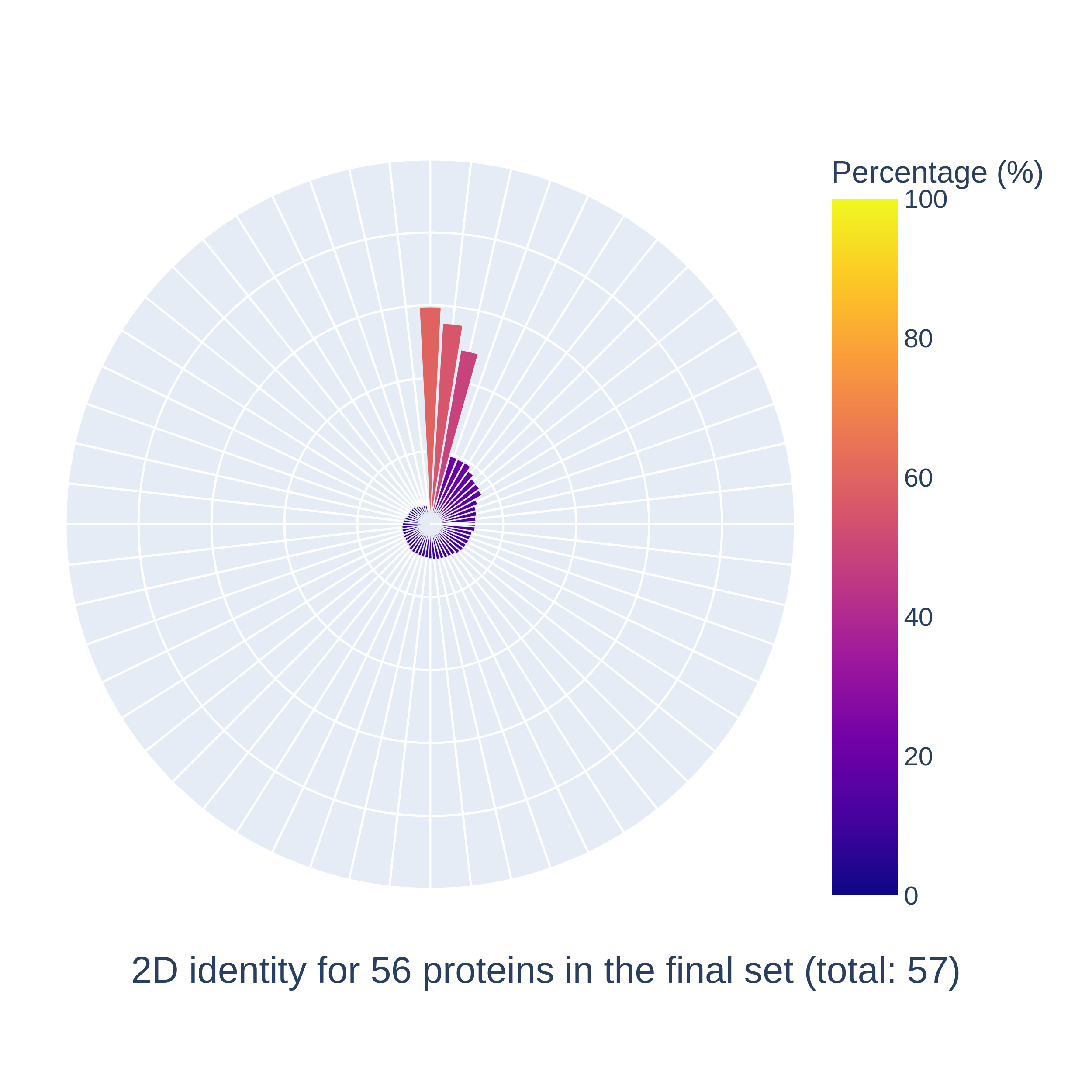

Supplement: Supplementary file 7 — Supplementary Data 4 [file 42003_2023_5076_MOESM7_ESM.zip › 6VXX_A_segment/plots/6VXX_A_site2-metrics_2D-identity.png]

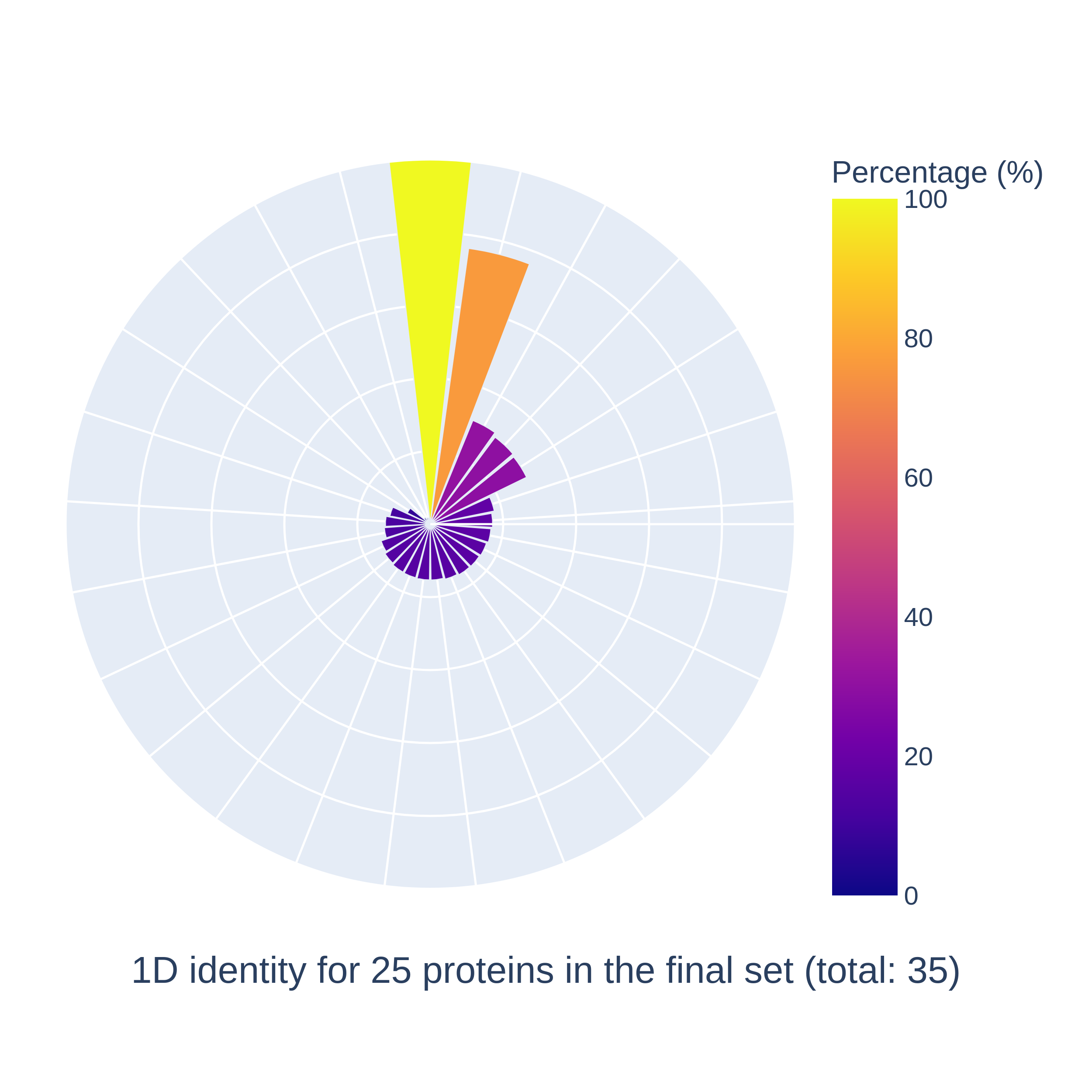

Supplement: Supplementary file 8 — Supplementary Data 5 [file 42003_2023_5076_MOESM8_ESM.zip › 6VXX_A_whole_human_exp_dataset/plots/6VXX_A_1D-identity.png]

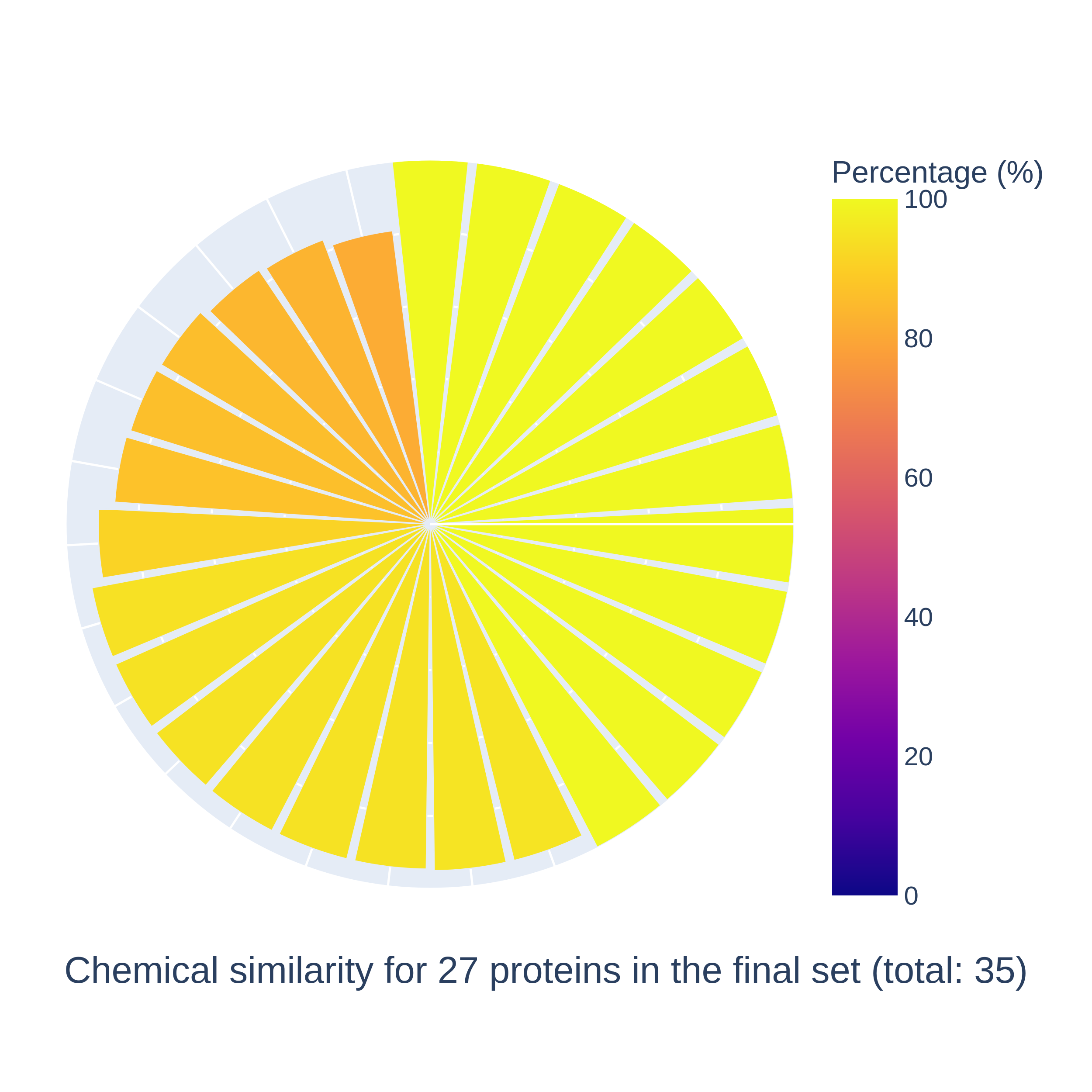

Supplement: Supplementary file 8 — Supplementary Data 5 [file 42003_2023_5076_MOESM8_ESM.zip › 6VXX_A_whole_human_exp_dataset/plots/6VXX_A_chemSim.png]

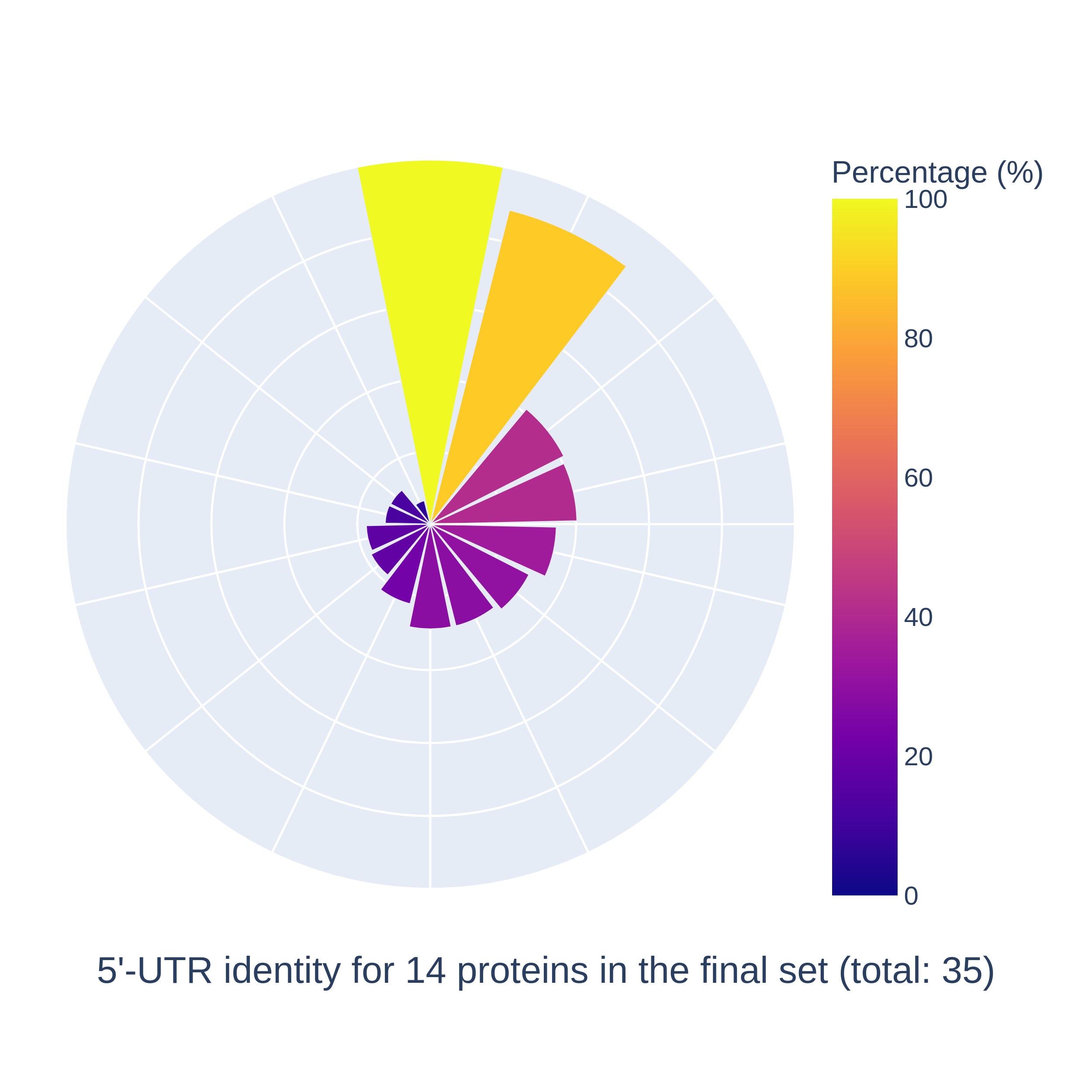

Supplement: Supplementary file 8 — Supplementary Data 5 [file 42003_2023_5076_MOESM8_ESM.zip › 6VXX_A_whole_human_exp_dataset/plots/6VXX_A_5UTR-identity.png]

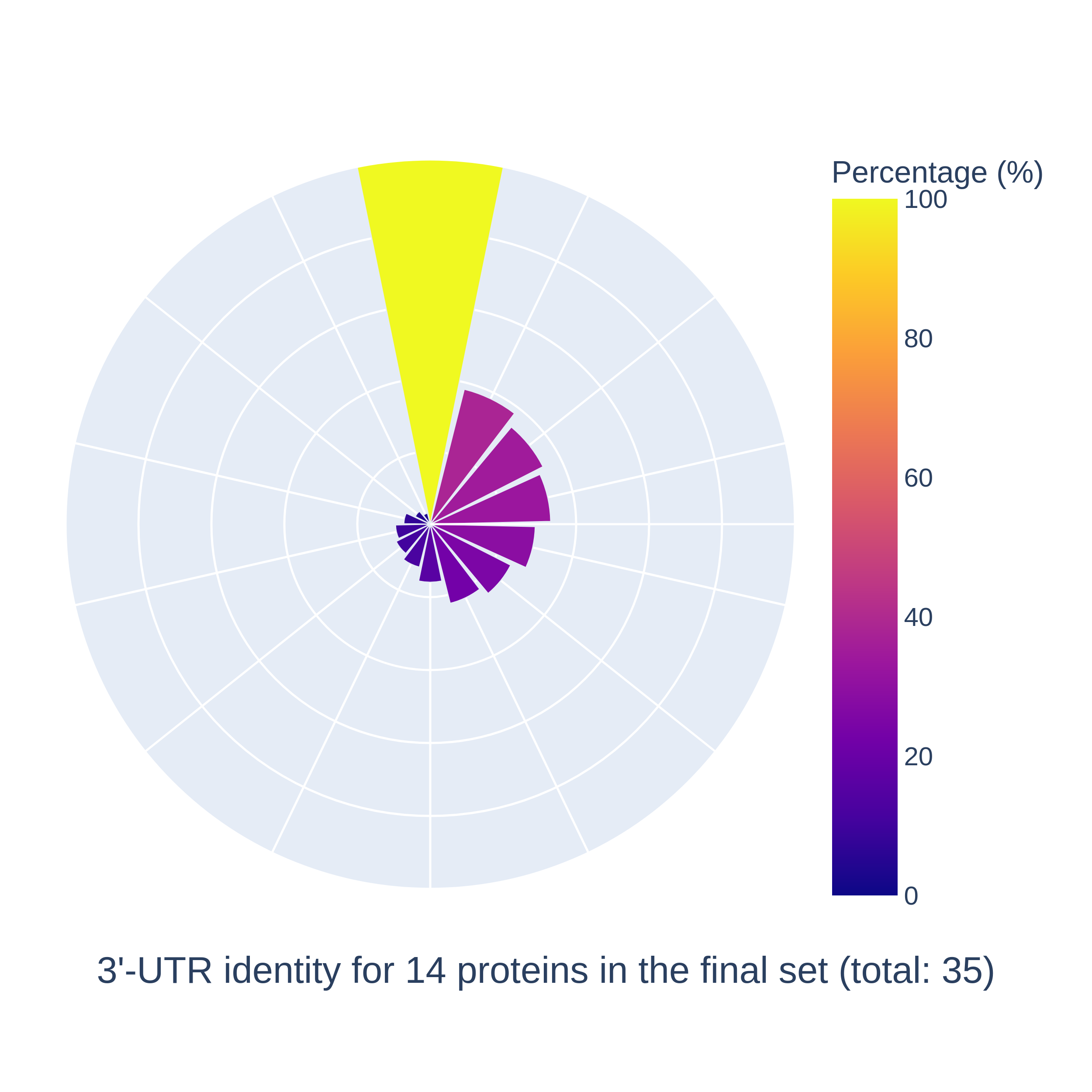

Supplement: Supplementary file 8 — Supplementary Data 5 [file 42003_2023_5076_MOESM8_ESM.zip › 6VXX_A_whole_human_exp_dataset/plots/6VXX_A_3UTR-identity.png]

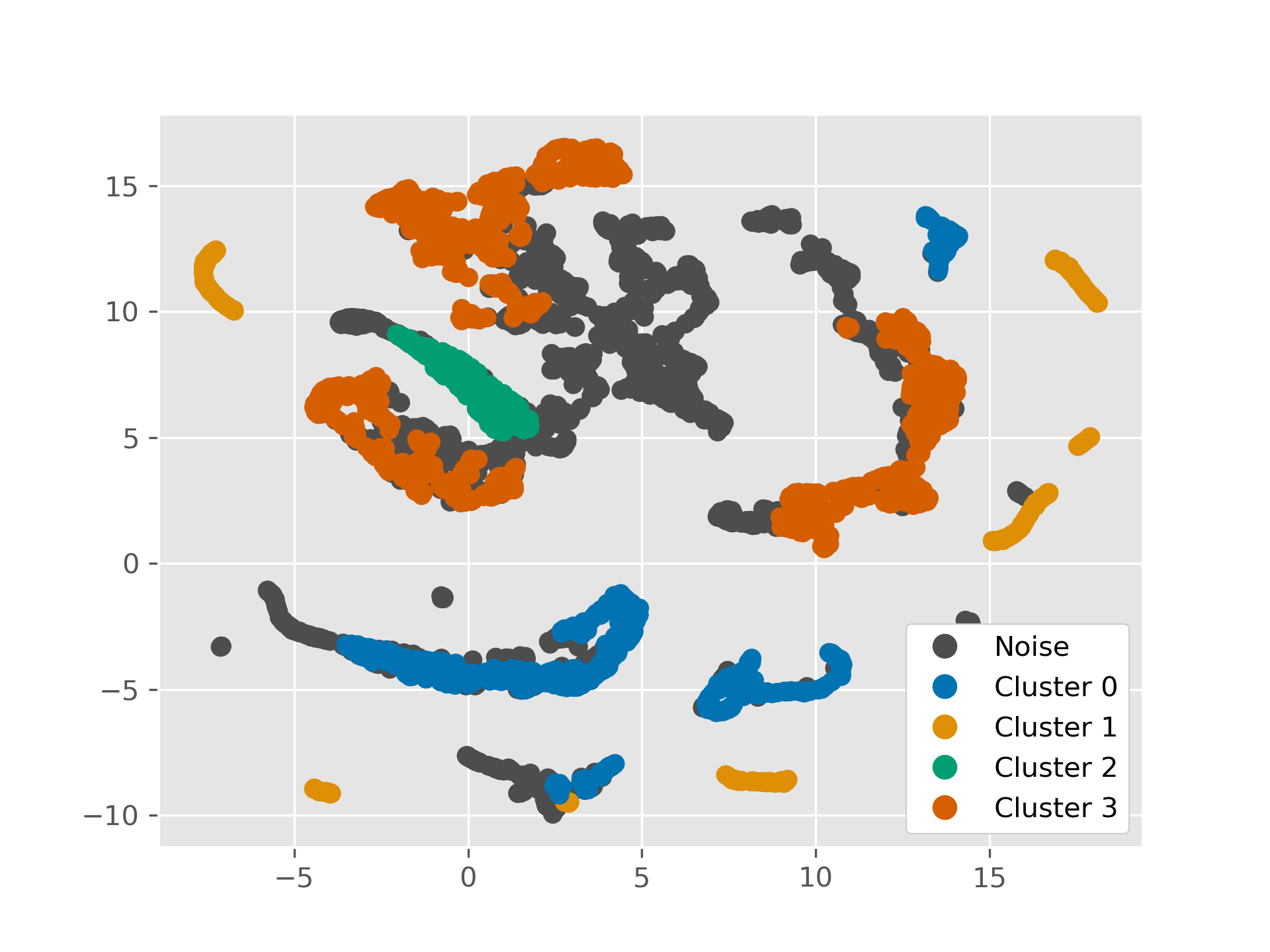

Supplement: Supplementary file 8 — Supplementary Data 5 [file 42003_2023_5076_MOESM8_ESM.zip › 6VXX_A_whole_human_exp_dataset/plots/6VXX_A-clusters-initial.png]

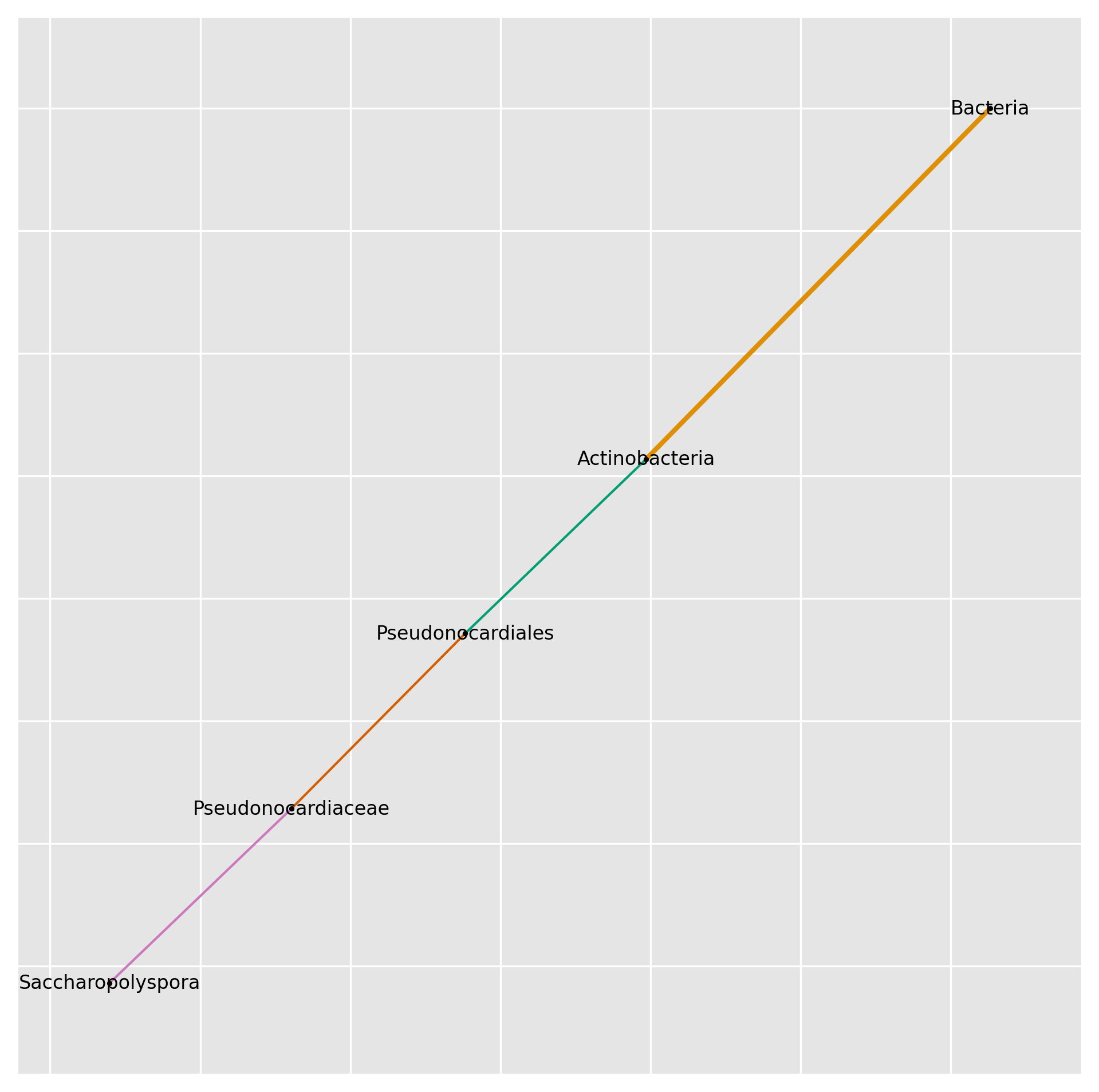

Supplement: Supplementary file 8 — Supplementary Data 5 [file 42003_2023_5076_MOESM8_ESM.zip › 6VXX_A_whole_human_exp_dataset/plots/6VXX_A-Bacteria-tree.png]

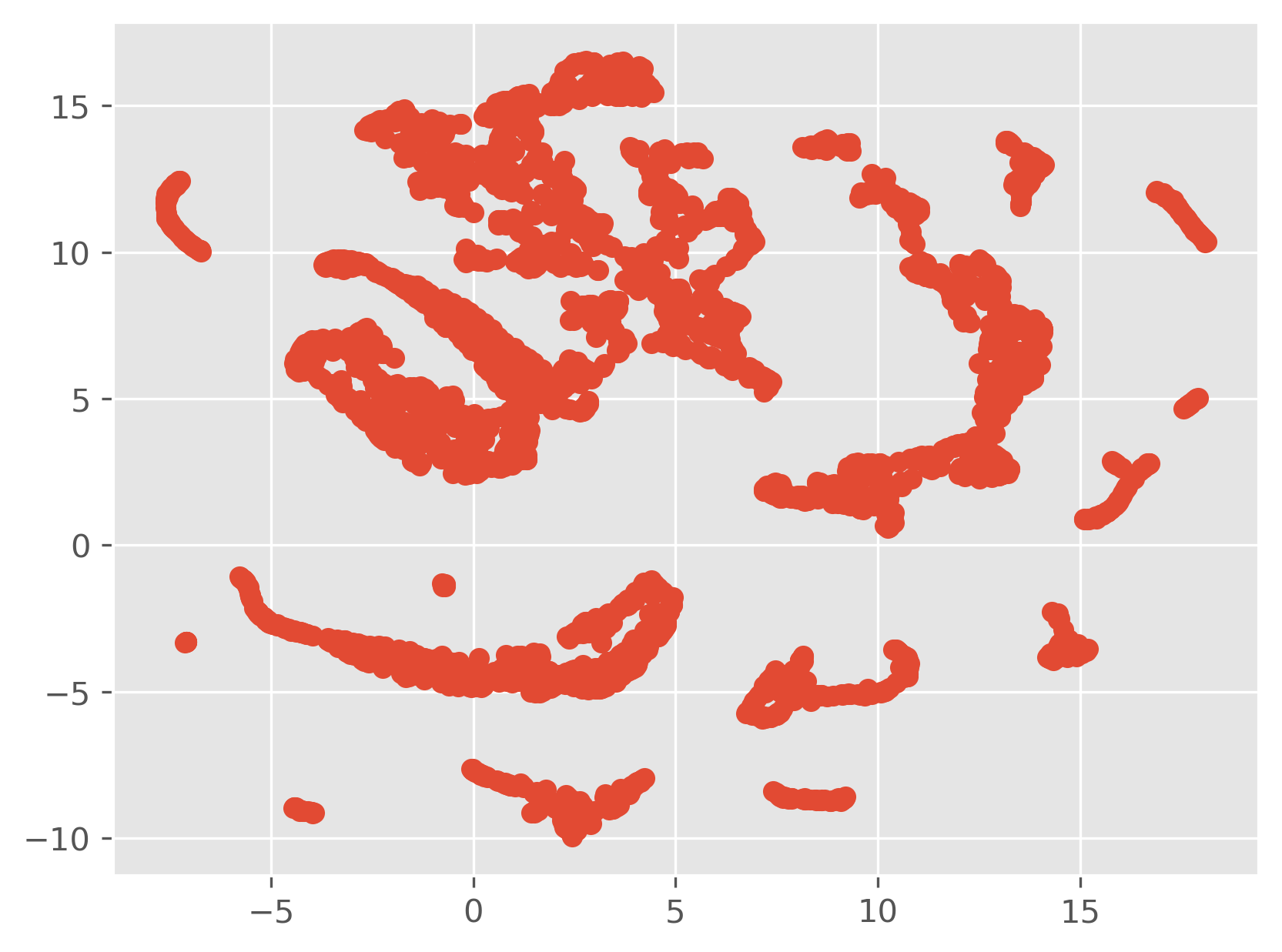

Supplement: Supplementary file 8 — Supplementary Data 5 [file 42003_2023_5076_MOESM8_ESM.zip › 6VXX_A_whole_human_exp_dataset/plots/6VXX_A-UMAP.png]

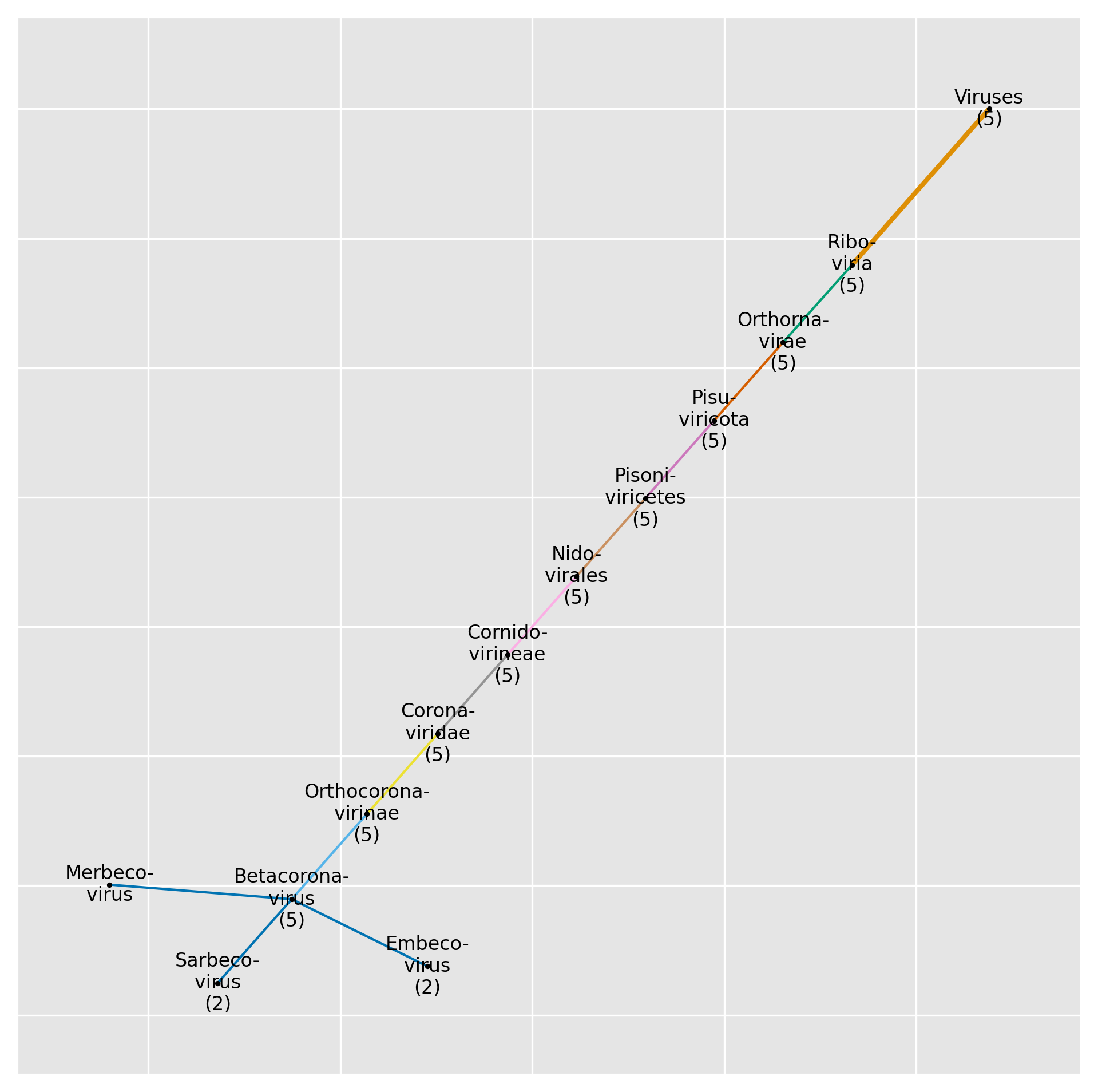

Supplement: Supplementary file 8 — Supplementary Data 5 [file 42003_2023_5076_MOESM8_ESM.zip › 6VXX_A_whole_human_exp_dataset/plots/6VXX_A-Viruses-tree.png]

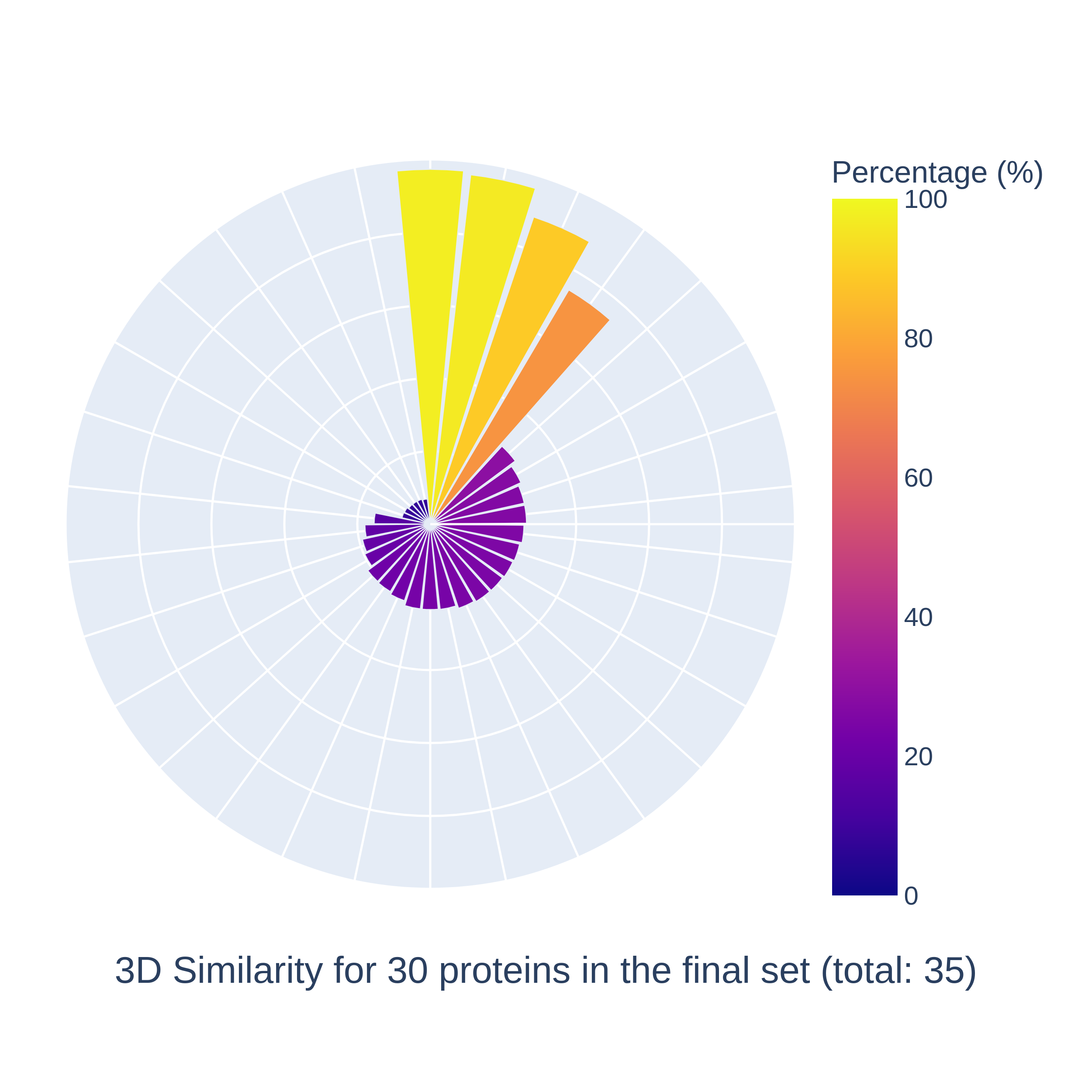

Supplement: Supplementary file 8 — Supplementary Data 5 [file 42003_2023_5076_MOESM8_ESM.zip › 6VXX_A_whole_human_exp_dataset/plots/6VXX_A_3D-score.png]

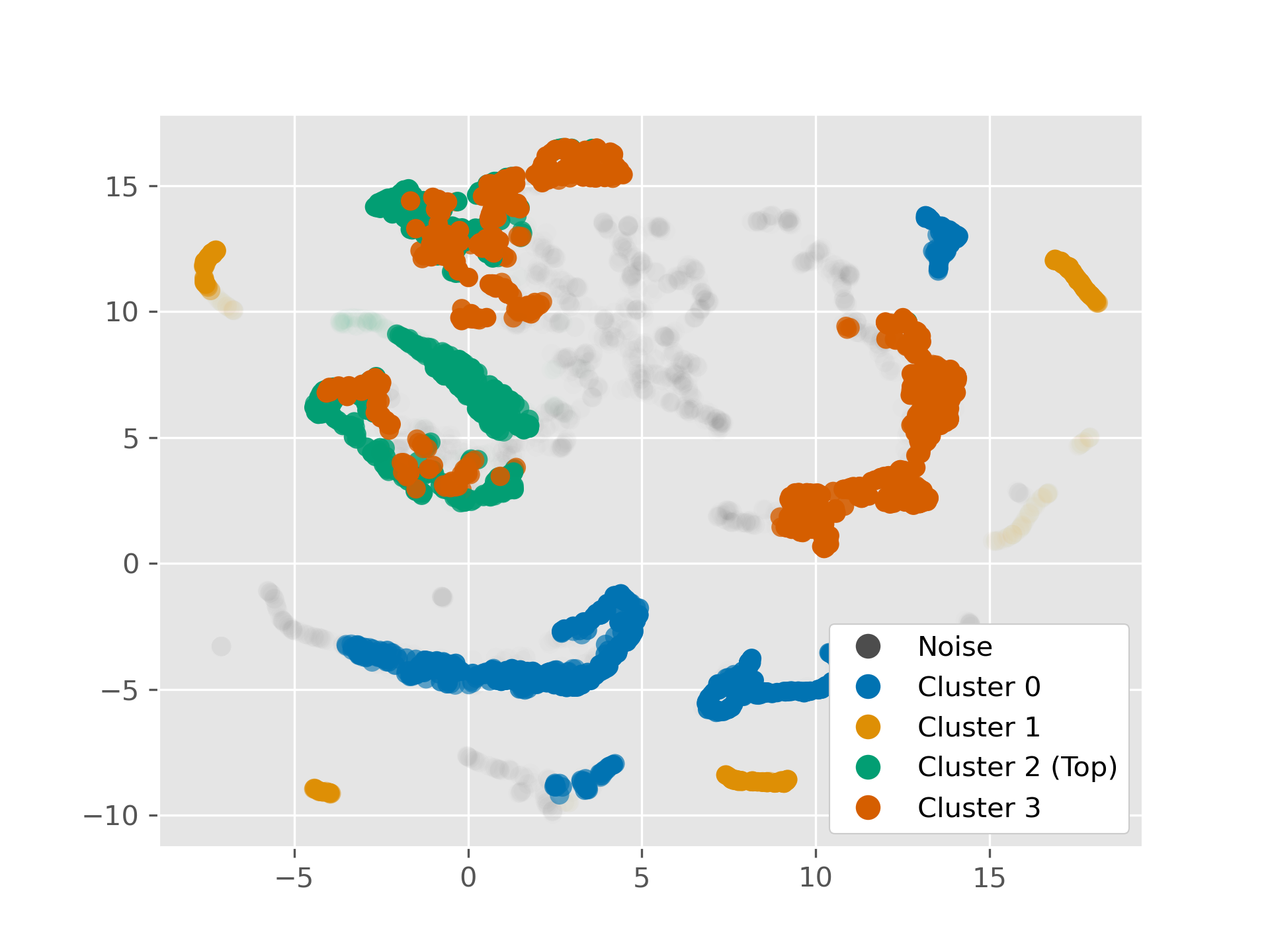

Supplement: Supplementary file 8 — Supplementary Data 5 [file 42003_2023_5076_MOESM8_ESM.zip › 6VXX_A_whole_human_exp_dataset/plots/6VXX_A-clusters.png]

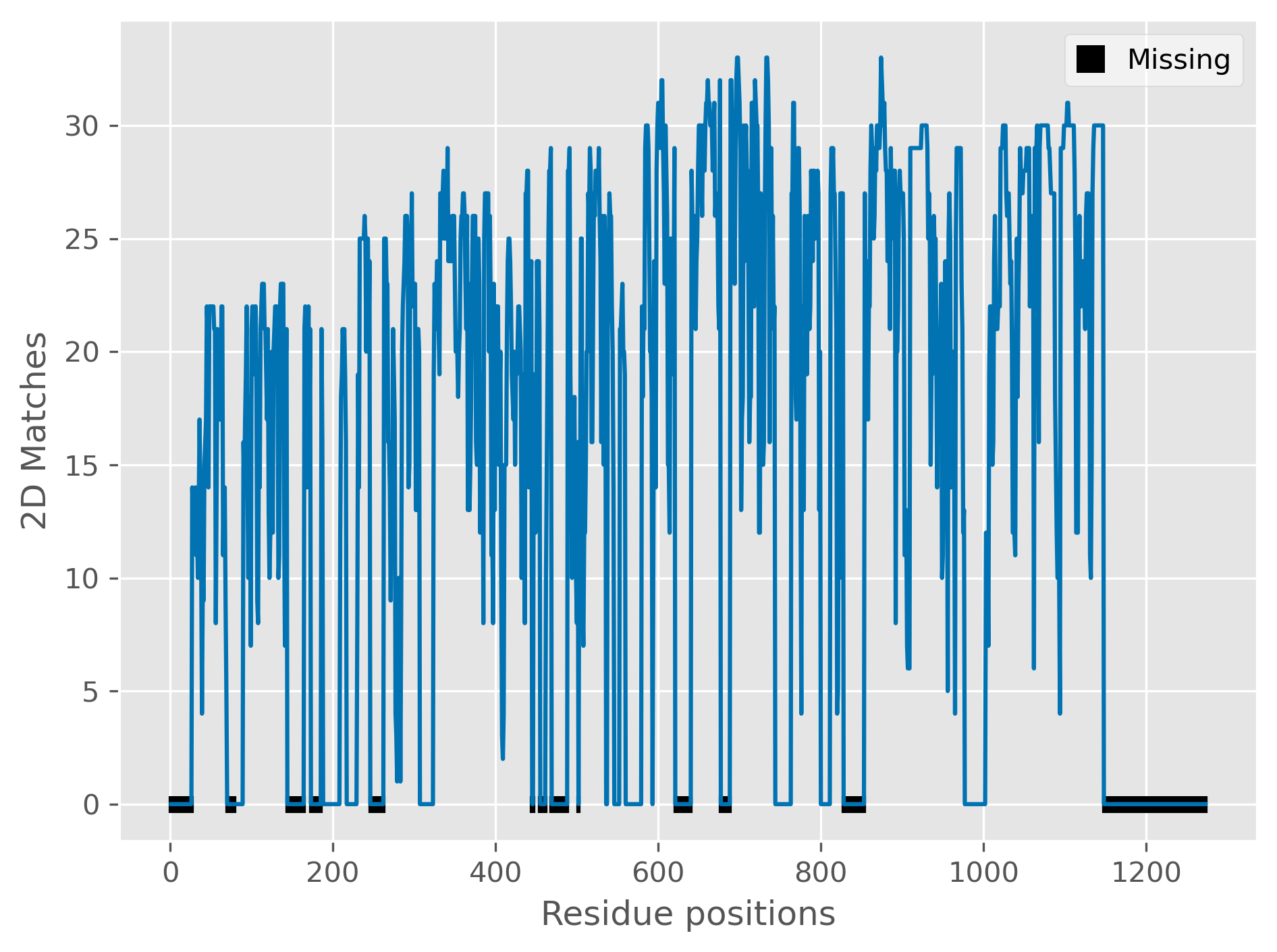

Supplement: Supplementary file 8 — Supplementary Data 5 [file 42003_2023_5076_MOESM8_ESM.zip › 6VXX_A_whole_human_exp_dataset/plots/6VXX_A-2Dmatches.png]

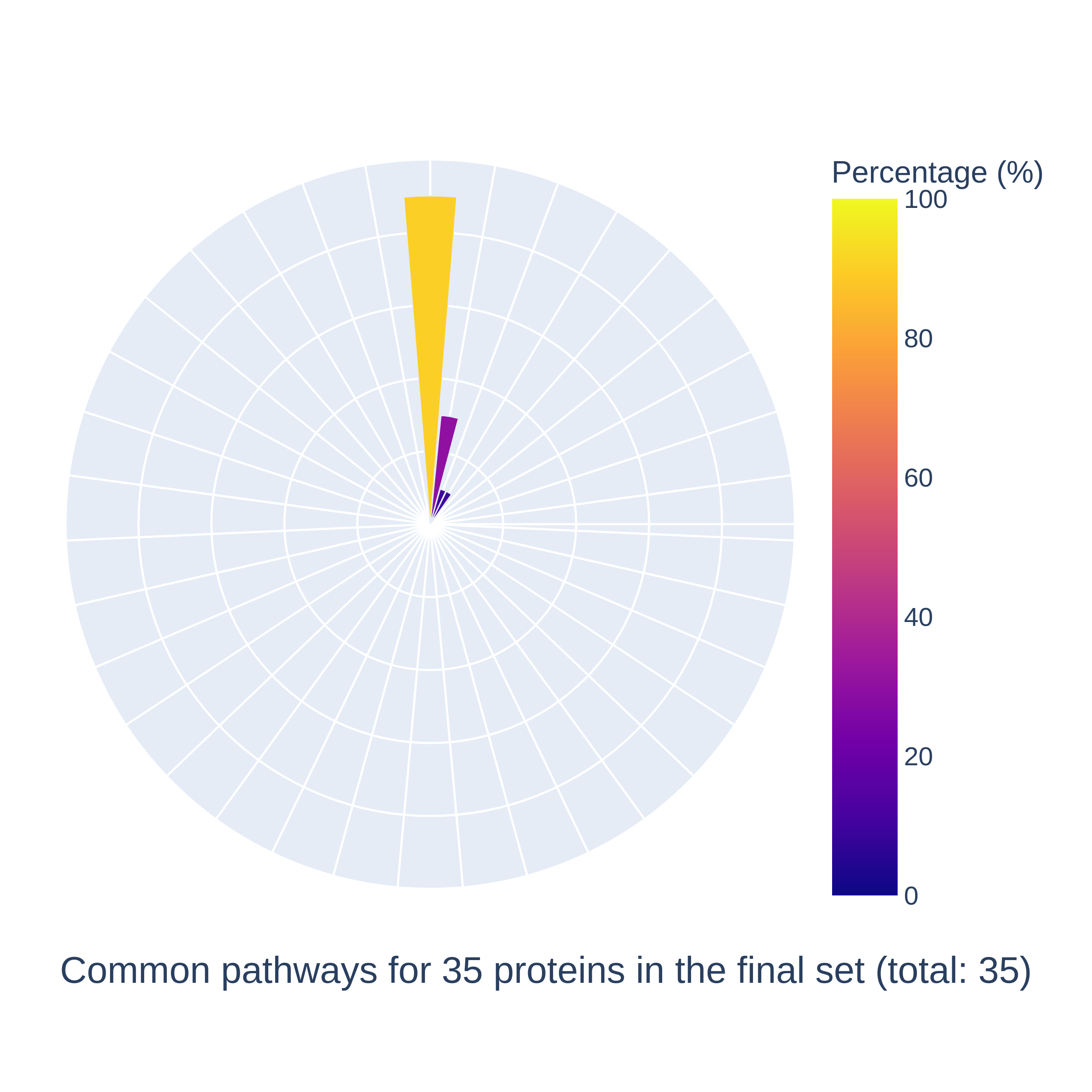

Supplement: Supplementary file 8 — Supplementary Data 5 [file 42003_2023_5076_MOESM8_ESM.zip › 6VXX_A_whole_human_exp_dataset/plots/6VXX_A_biologicalProcessSim.png]

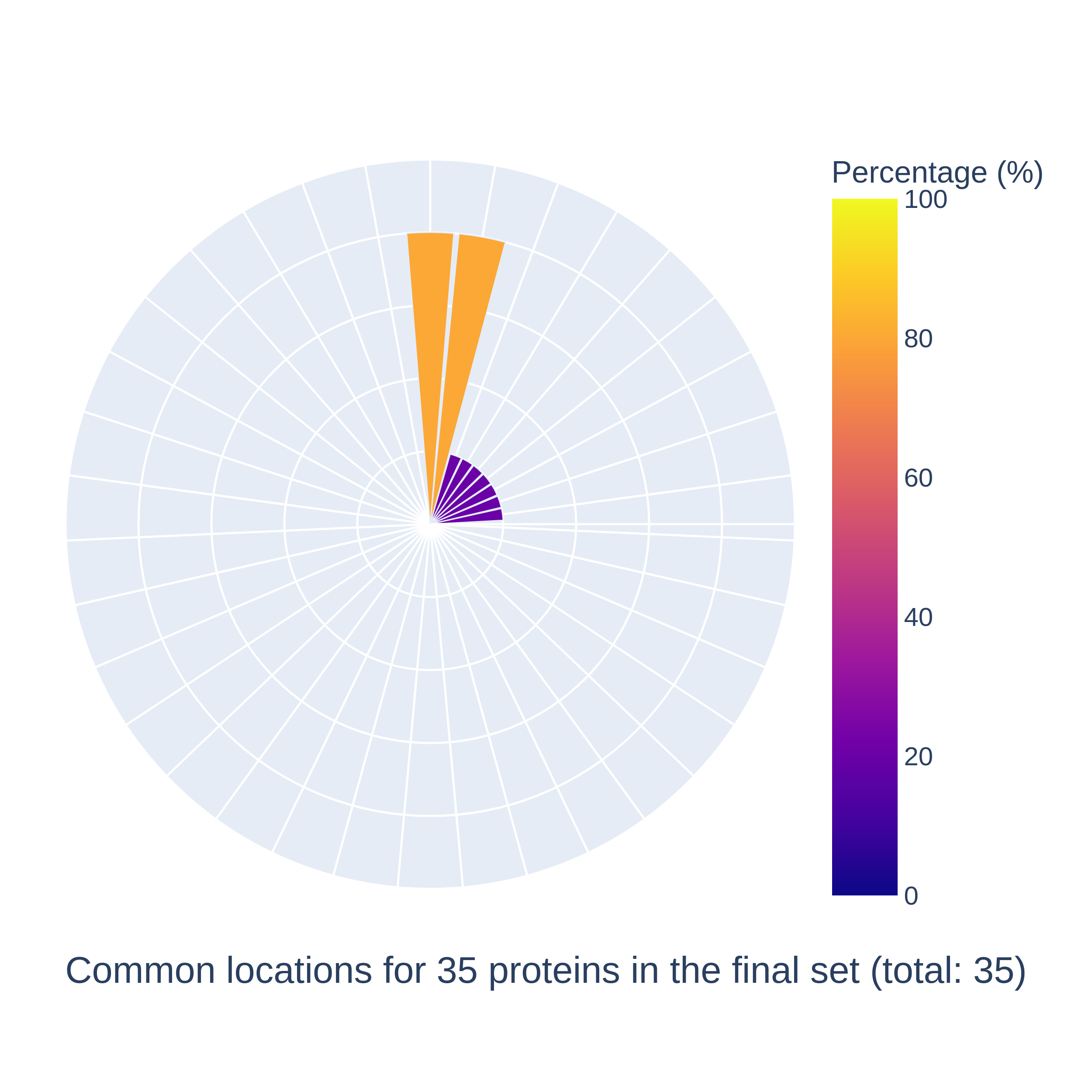

Supplement: Supplementary file 8 — Supplementary Data 5 [file 42003_2023_5076_MOESM8_ESM.zip › 6VXX_A_whole_human_exp_dataset/plots/6VXX_A_cellularComponentSim.png]

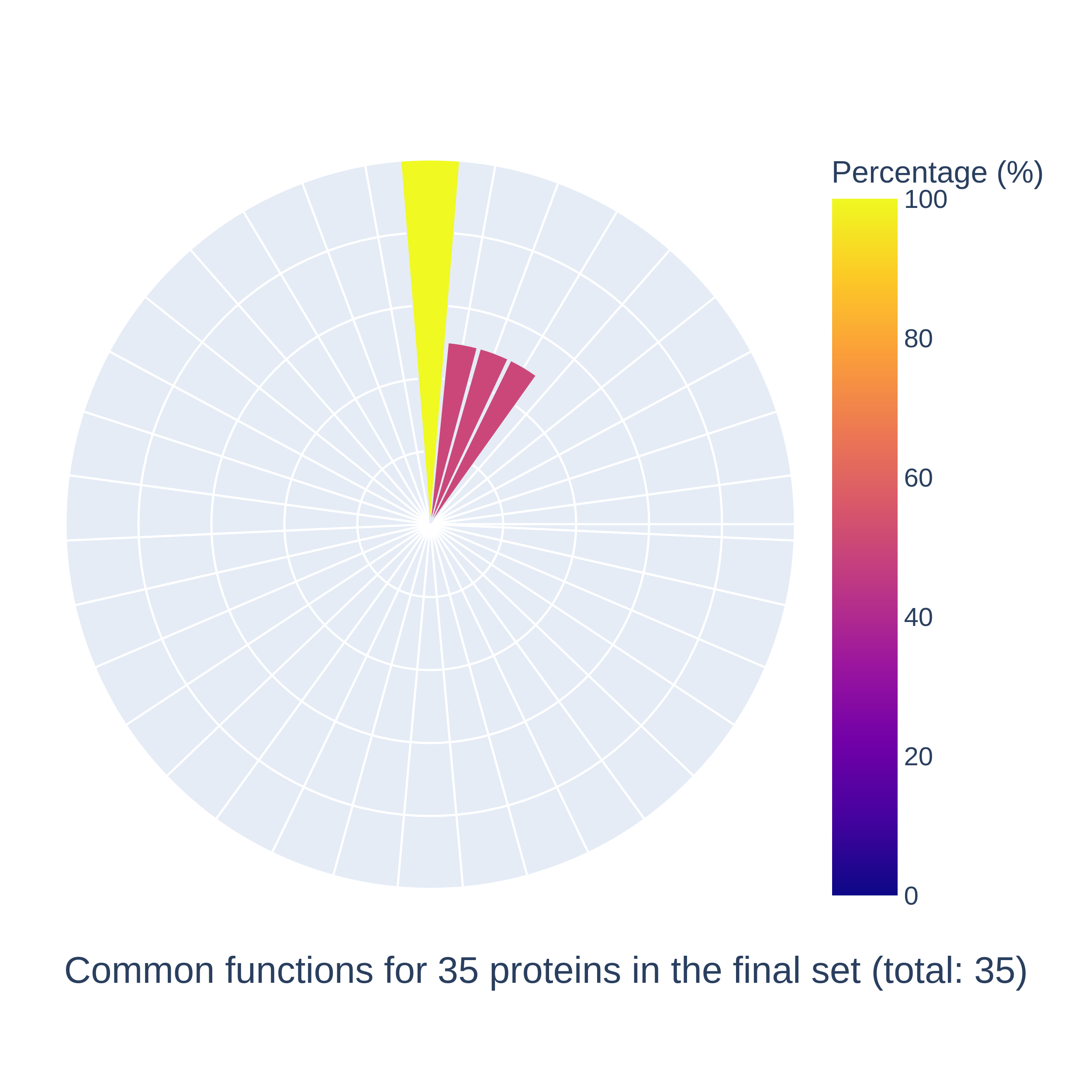

Supplement: Supplementary file 8 — Supplementary Data 5 [file 42003_2023_5076_MOESM8_ESM.zip › 6VXX_A_whole_human_exp_dataset/plots/6VXX_A_molecularFunctionSim.png]

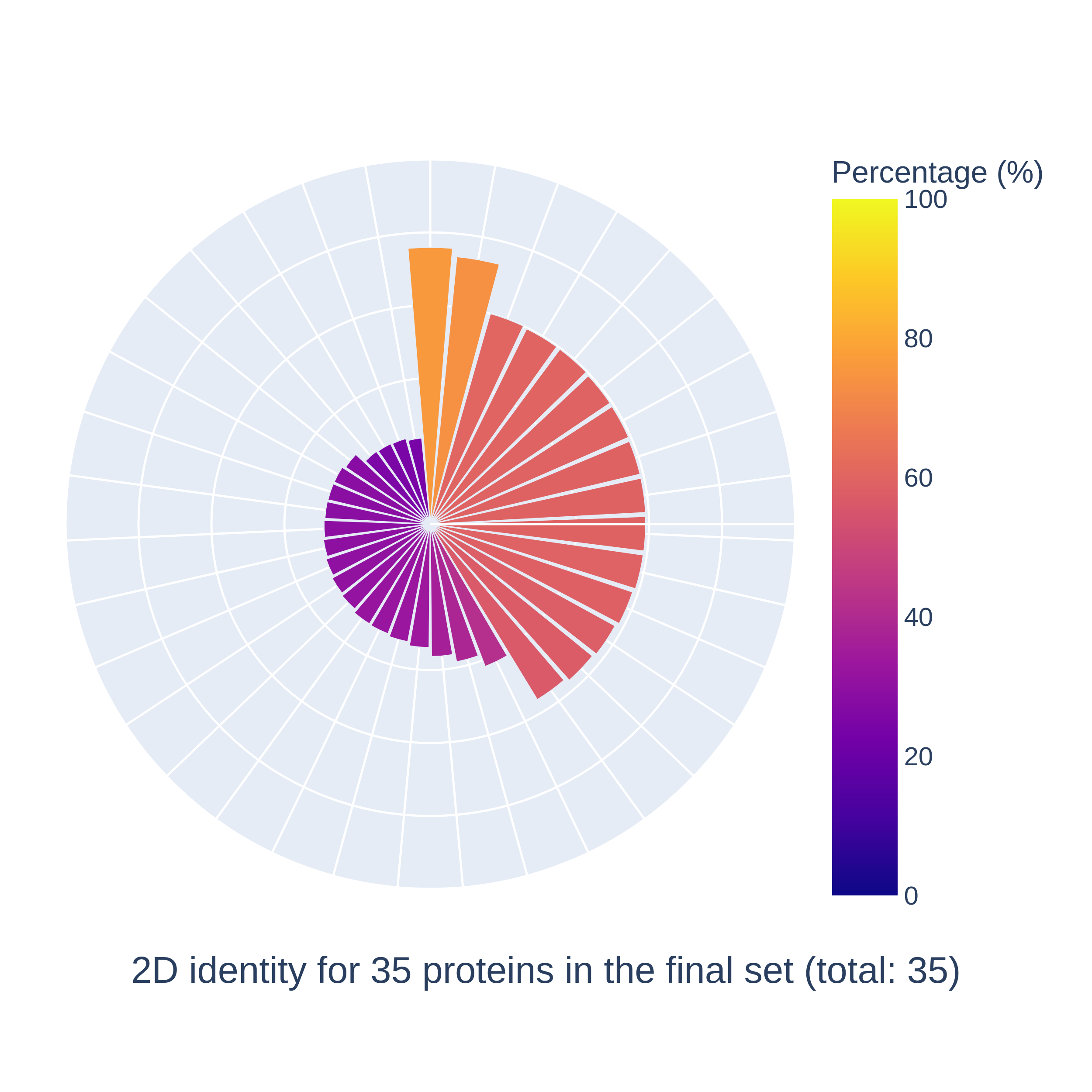

Supplement: Supplementary file 8 — Supplementary Data 5 [file 42003_2023_5076_MOESM8_ESM.zip › 6VXX_A_whole_human_exp_dataset/plots/6VXX_A_2D-identity.png]

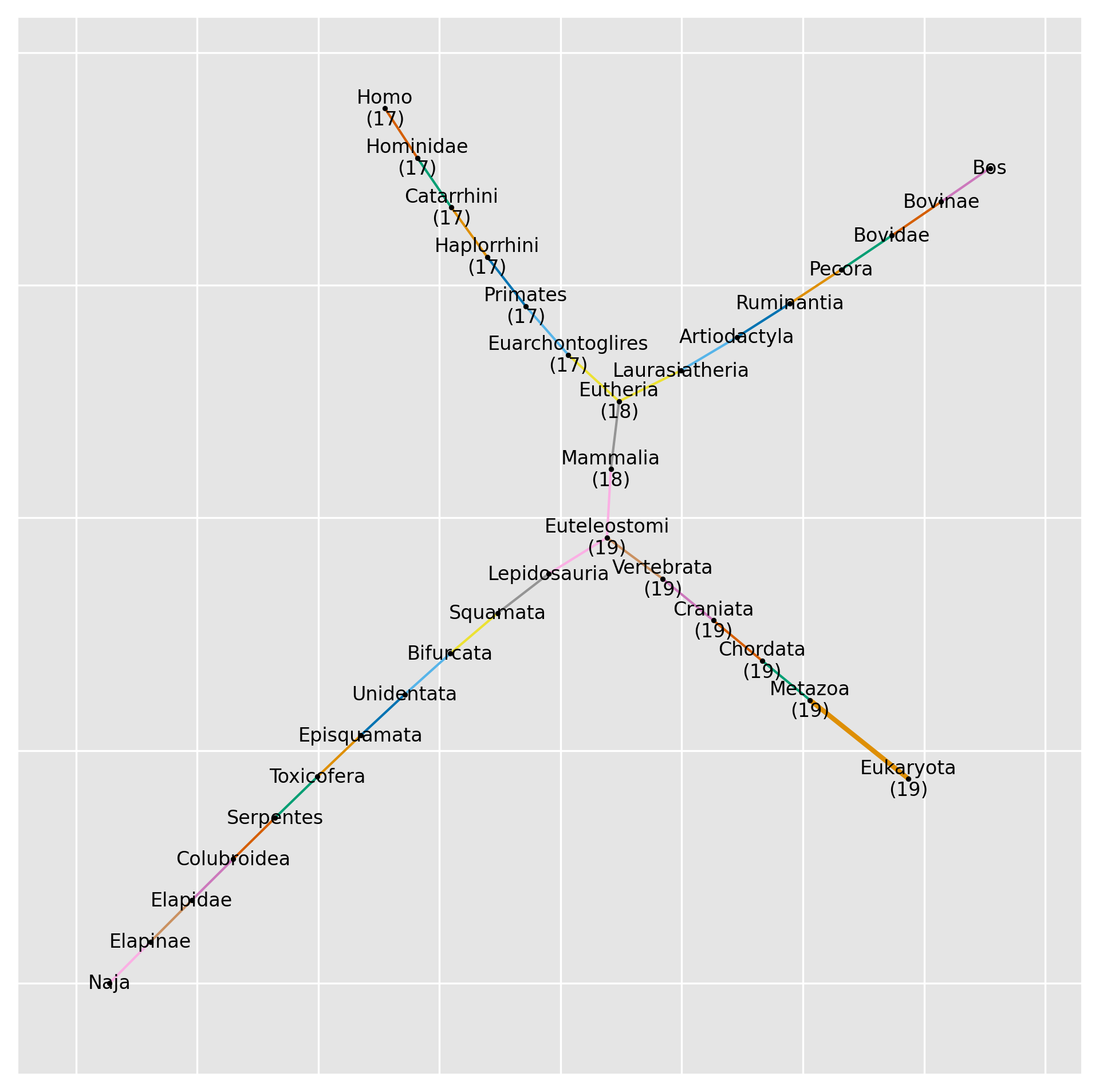

Supplement: Supplementary file 8 — Supplementary Data 5 [file 42003_2023_5076_MOESM8_ESM.zip › 6VXX_A_whole_human_exp_dataset/plots/6VXX_A-Eukaryota-tree.png]

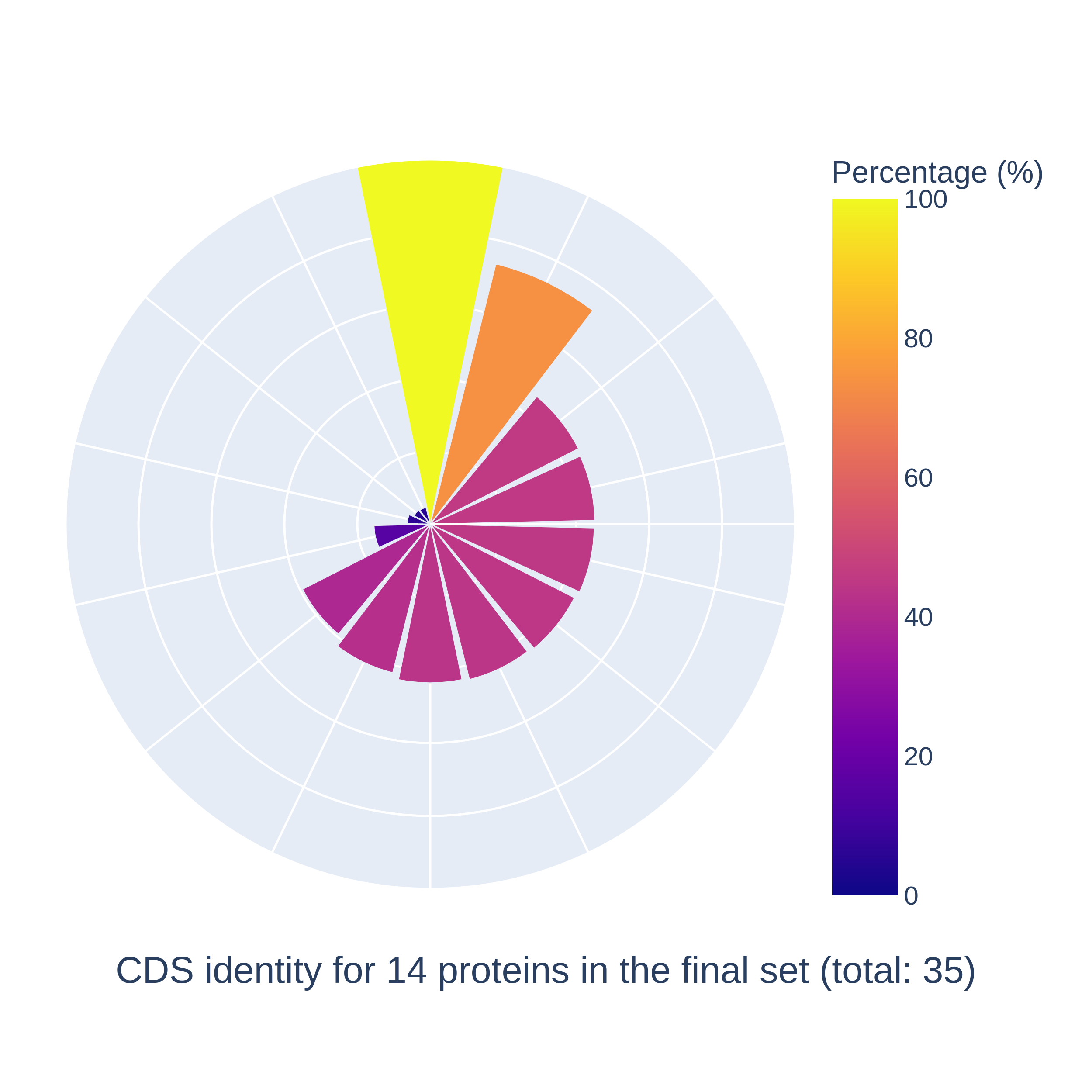

Supplement: Supplementary file 8 — Supplementary Data 5 [file 42003_2023_5076_MOESM8_ESM.zip › 6VXX_A_whole_human_exp_dataset/plots/6VXX_A_CDS-identity.png]

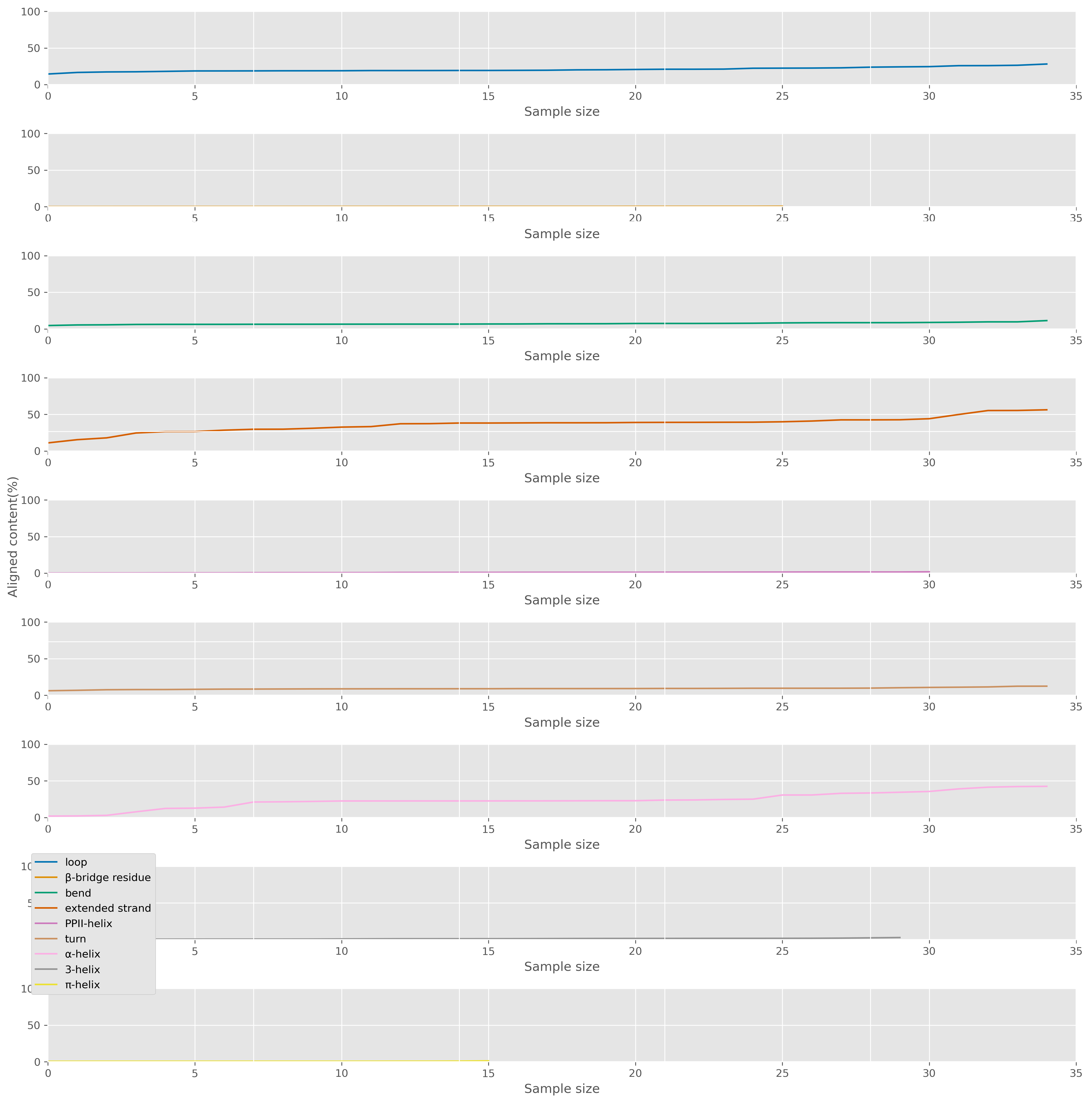

Supplement: Supplementary file 8 — Supplementary Data 5 [file 42003_2023_5076_MOESM8_ESM.zip › 6VXX_A_whole_human_exp_dataset/plots/6VXX_A-2Dfold_coverage.png]

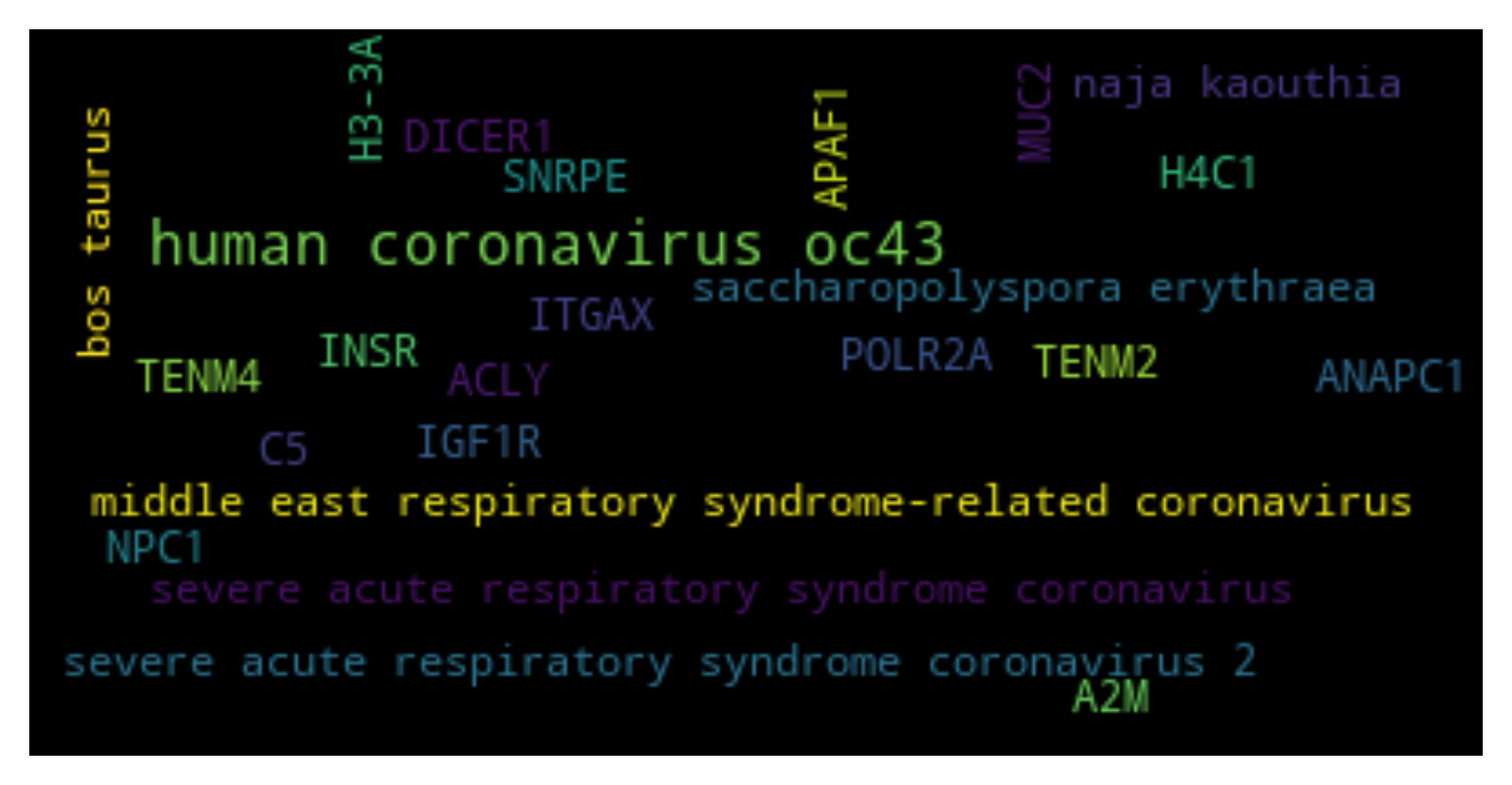

Supplement: Supplementary file 8 — Supplementary Data 5 [file 42003_2023_5076_MOESM8_ESM.zip › 6VXX_A_whole_human_exp_dataset/plots/6VXX_A-wordcloud.png]

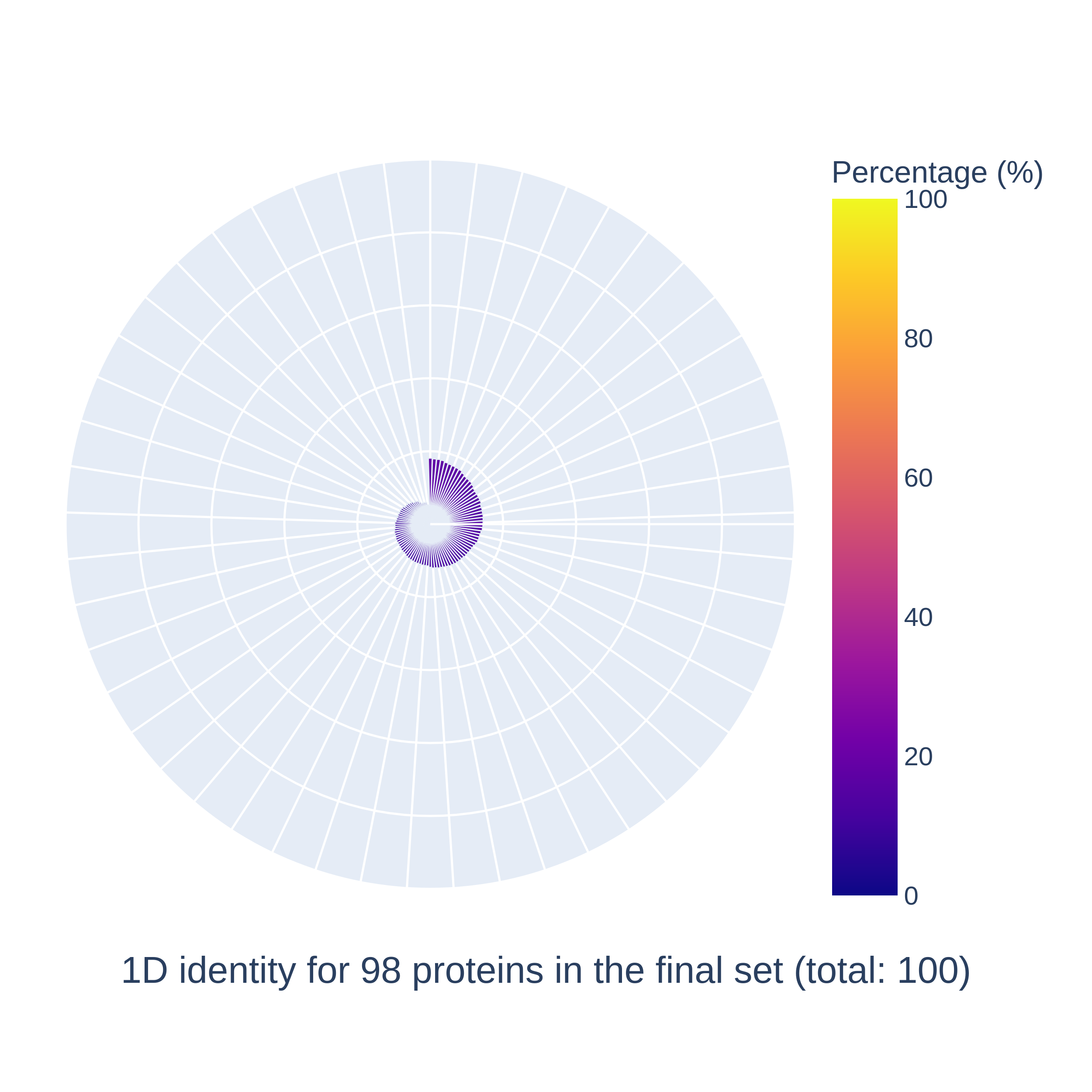

Supplement: Supplementary file 9 — Supplementary Data 6 [file 42003_2023_5076_MOESM9_ESM.zip › 6VXX_A_whole_alphafold4_dataset/plots/6VXX_A_1D-identity.png]

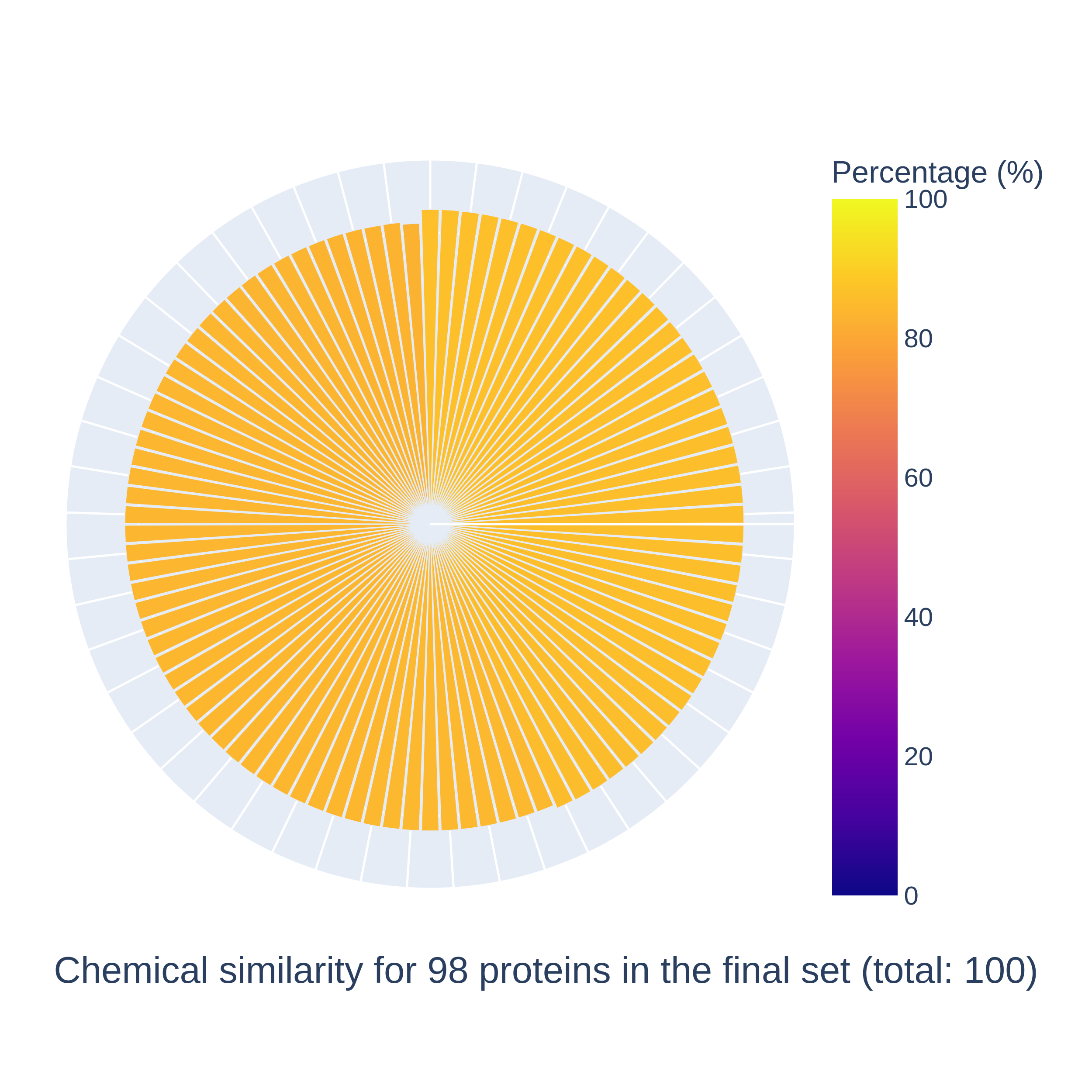

Supplement: Supplementary file 9 — Supplementary Data 6 [file 42003_2023_5076_MOESM9_ESM.zip › 6VXX_A_whole_alphafold4_dataset/plots/6VXX_A_chemSim.png]

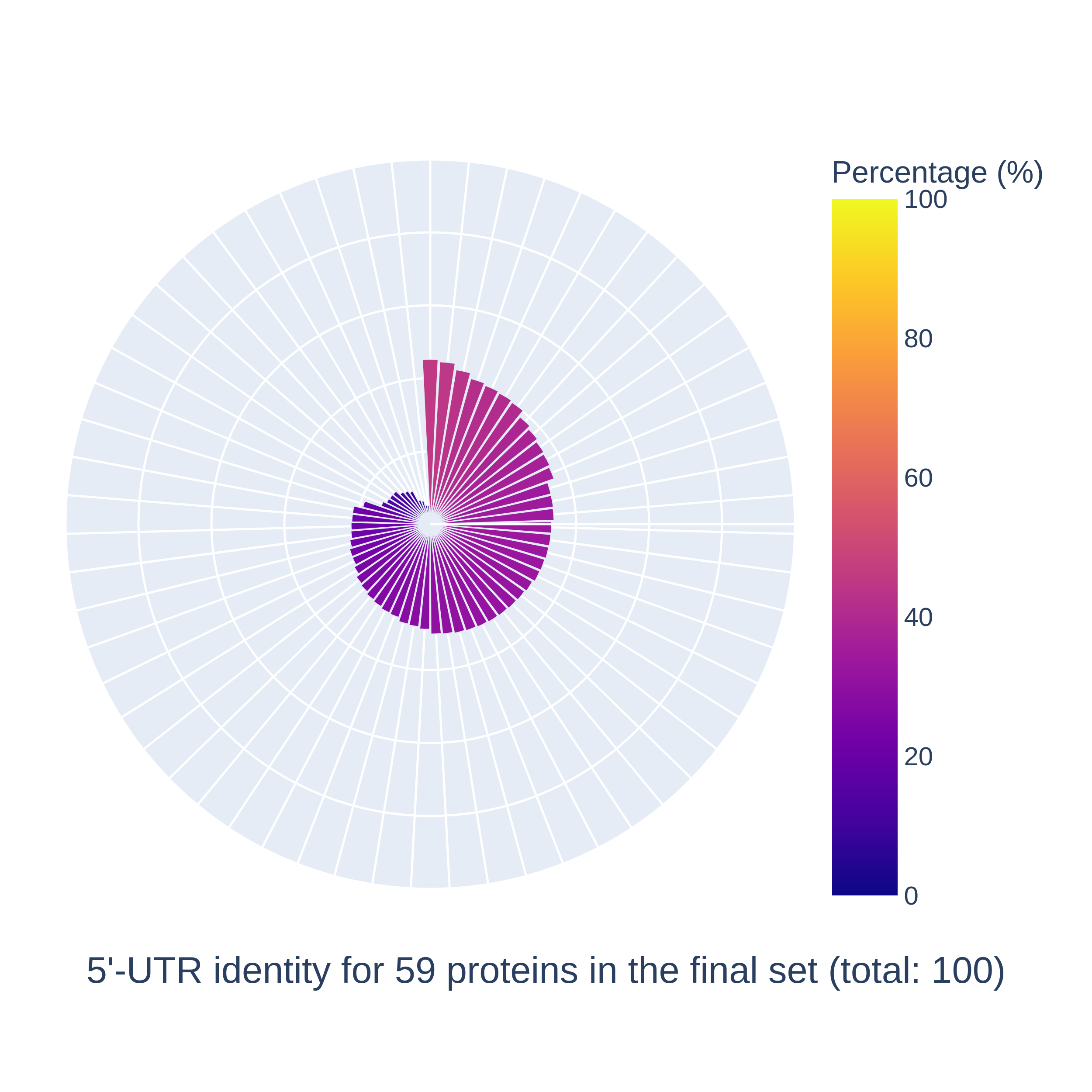

Supplement: Supplementary file 9 — Supplementary Data 6 [file 42003_2023_5076_MOESM9_ESM.zip › 6VXX_A_whole_alphafold4_dataset/plots/6VXX_A_5UTR-identity.png]

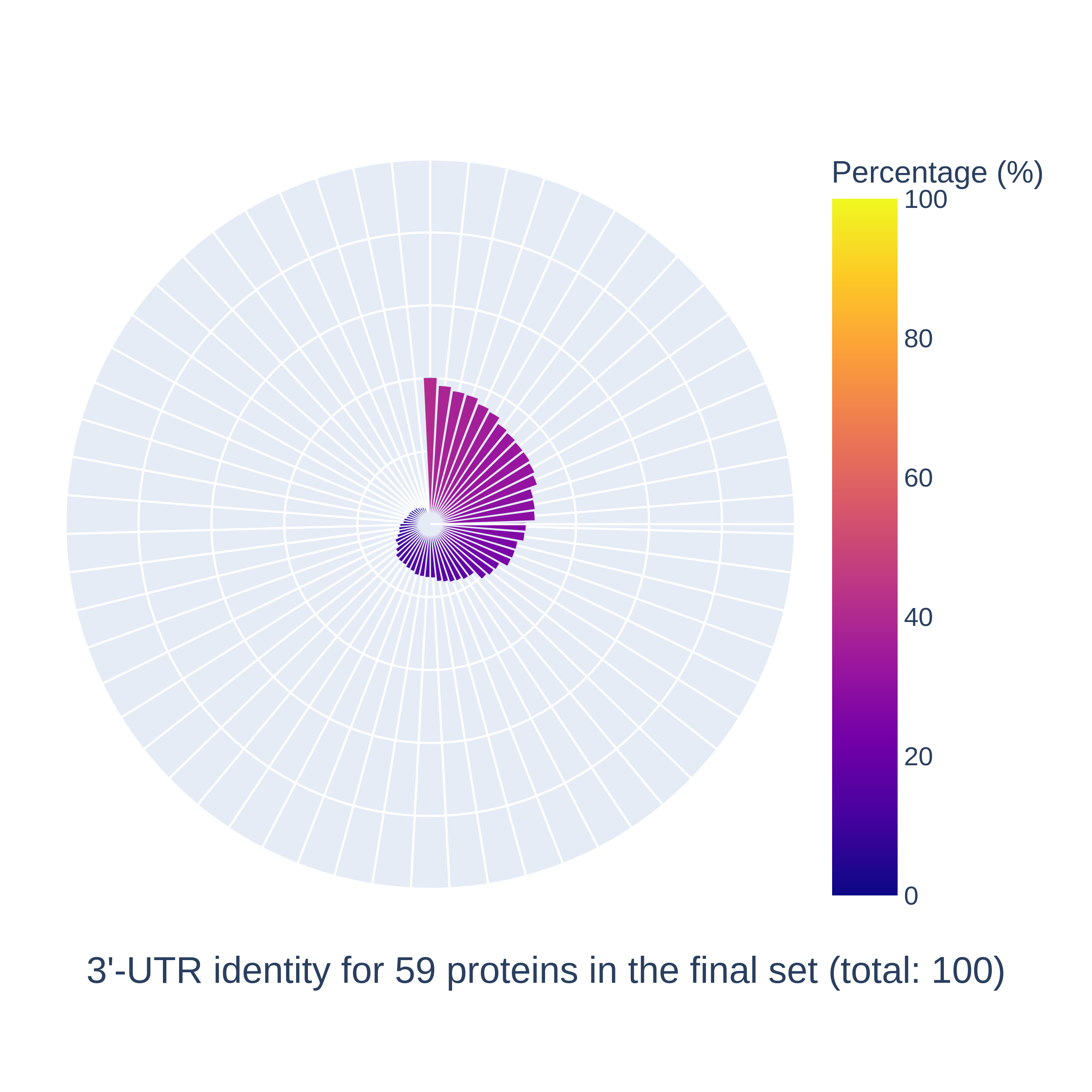

Supplement: Supplementary file 9 — Supplementary Data 6 [file 42003_2023_5076_MOESM9_ESM.zip › 6VXX_A_whole_alphafold4_dataset/plots/6VXX_A_3UTR-identity.png]

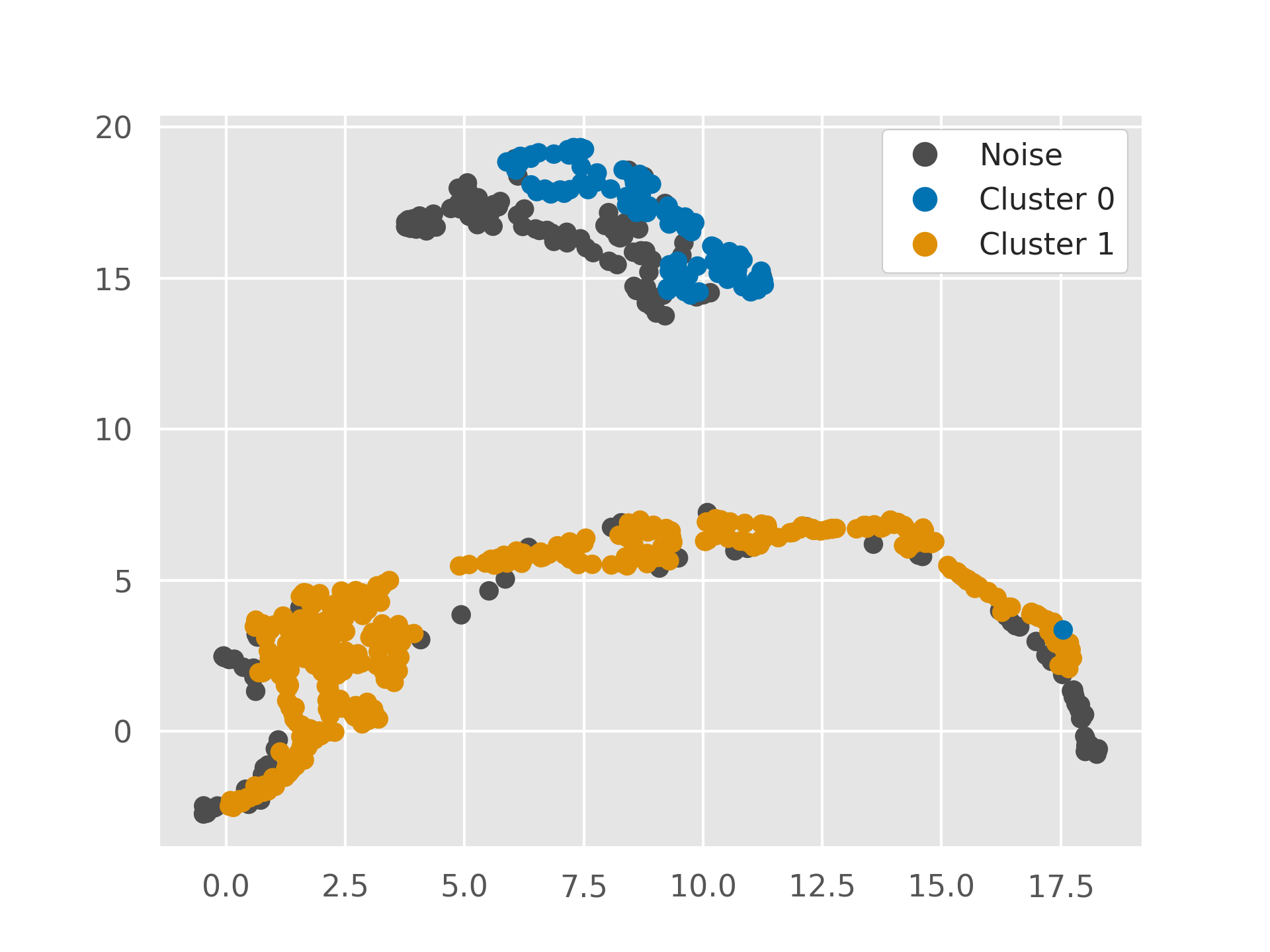

Supplement: Supplementary file 9 — Supplementary Data 6 [file 42003_2023_5076_MOESM9_ESM.zip › 6VXX_A_whole_alphafold4_dataset/plots/6VXX_A-clusters-initial.png]

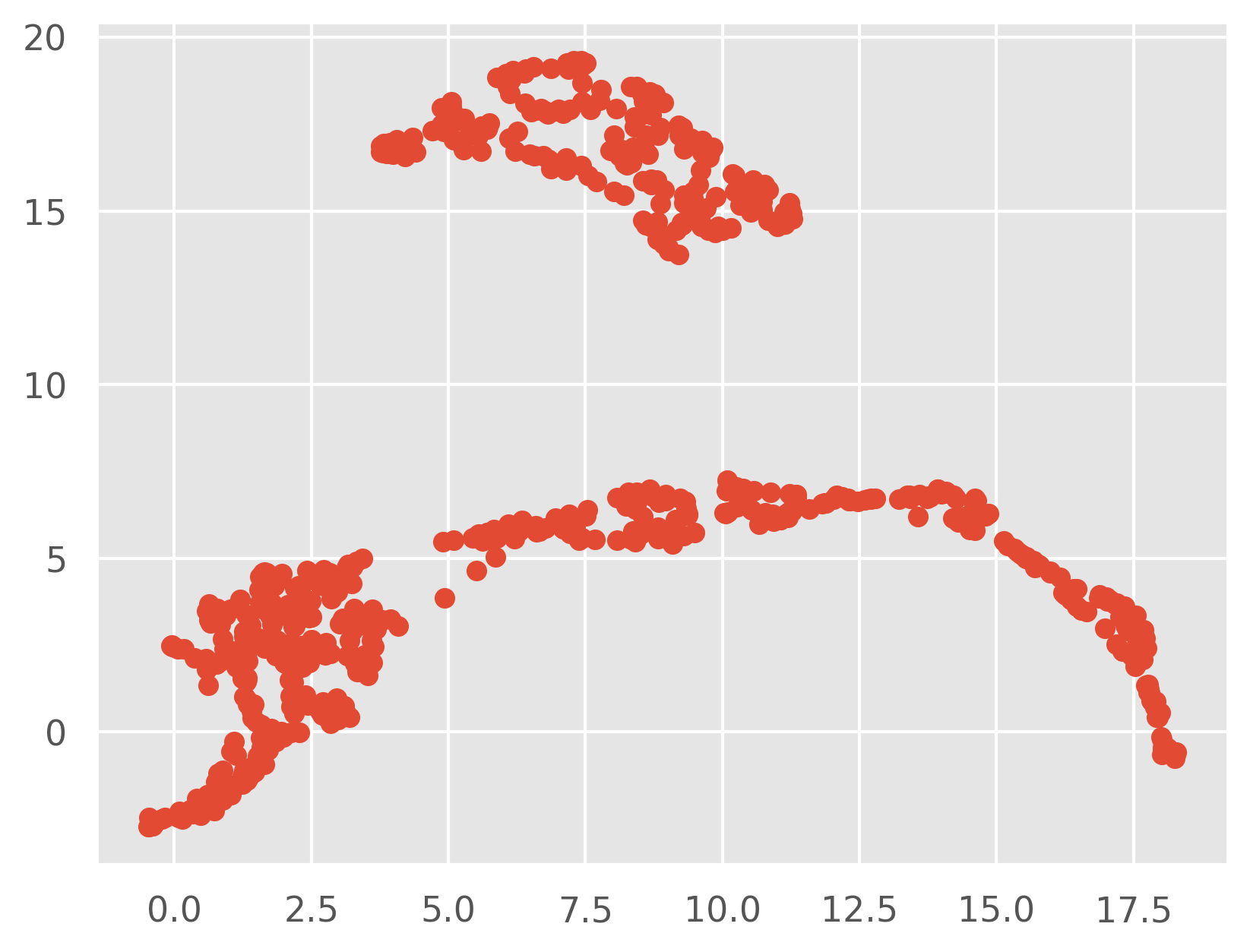

Supplement: Supplementary file 9 — Supplementary Data 6 [file 42003_2023_5076_MOESM9_ESM.zip › 6VXX_A_whole_alphafold4_dataset/plots/6VXX_A-UMAP.png]

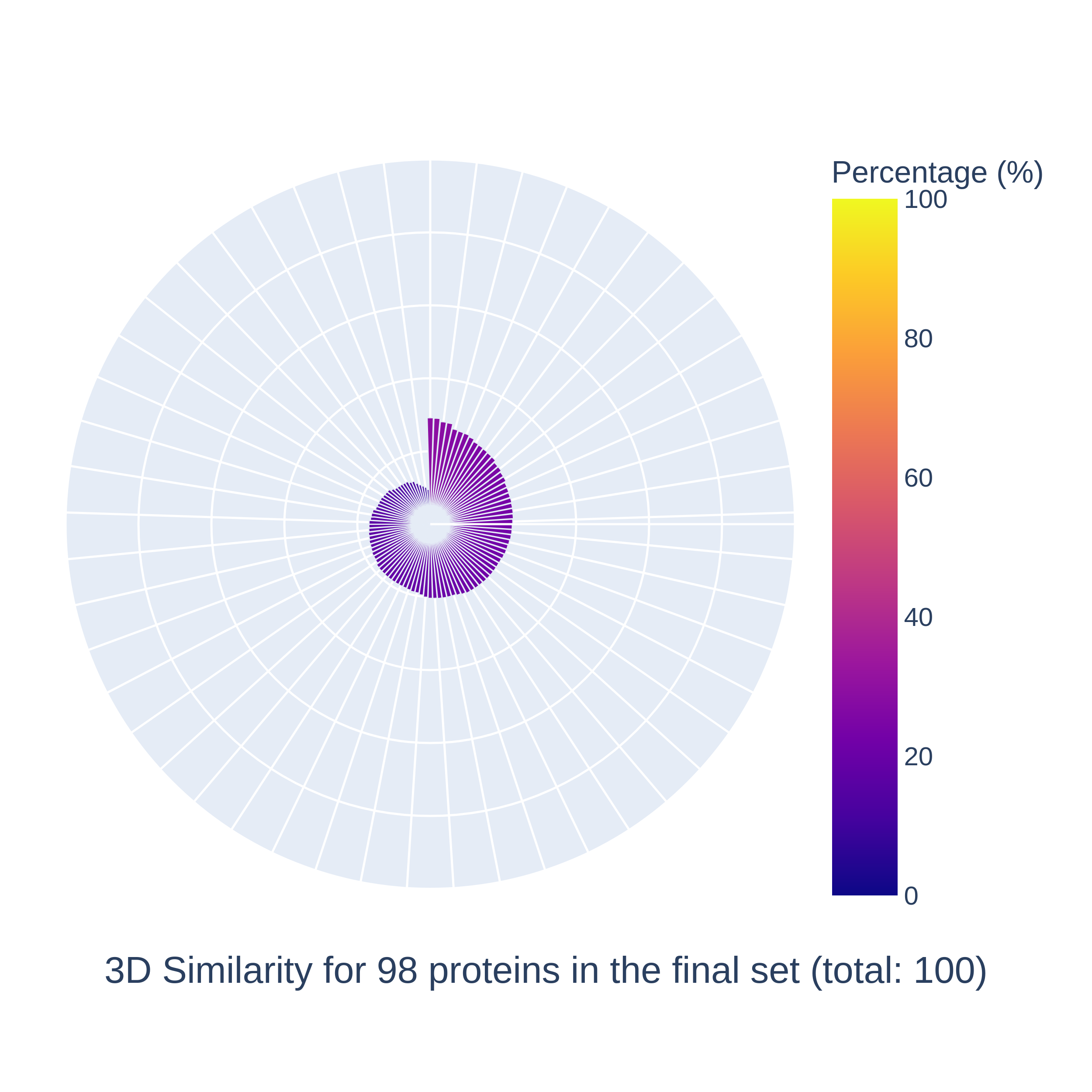

Supplement: Supplementary file 9 — Supplementary Data 6 [file 42003_2023_5076_MOESM9_ESM.zip › 6VXX_A_whole_alphafold4_dataset/plots/6VXX_A_3D-score.png]

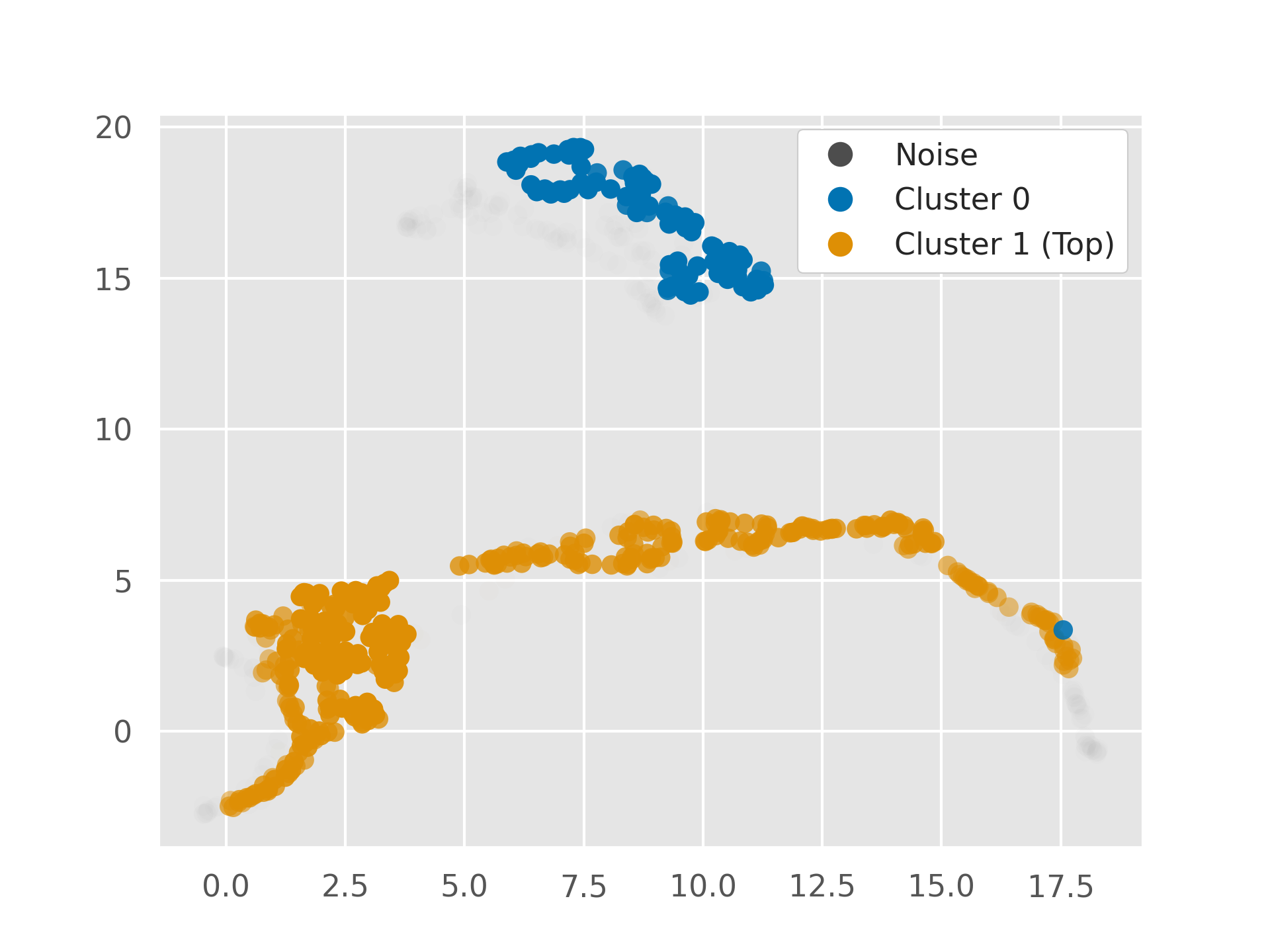

Supplement: Supplementary file 9 — Supplementary Data 6 [file 42003_2023_5076_MOESM9_ESM.zip › 6VXX_A_whole_alphafold4_dataset/plots/6VXX_A-clusters.png]

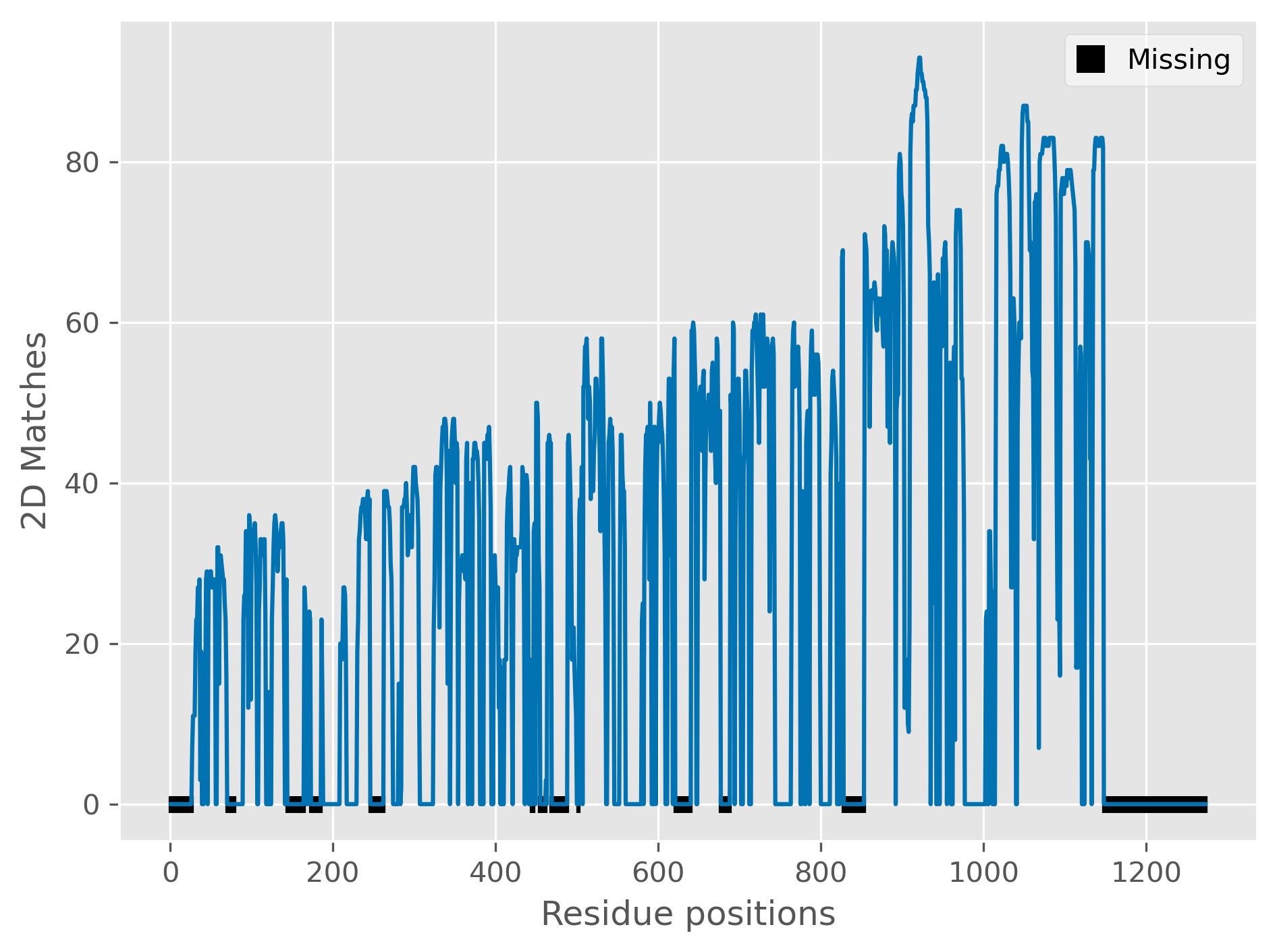

Supplement: Supplementary file 9 — Supplementary Data 6 [file 42003_2023_5076_MOESM9_ESM.zip › 6VXX_A_whole_alphafold4_dataset/plots/6VXX_A-2Dmatches.png]

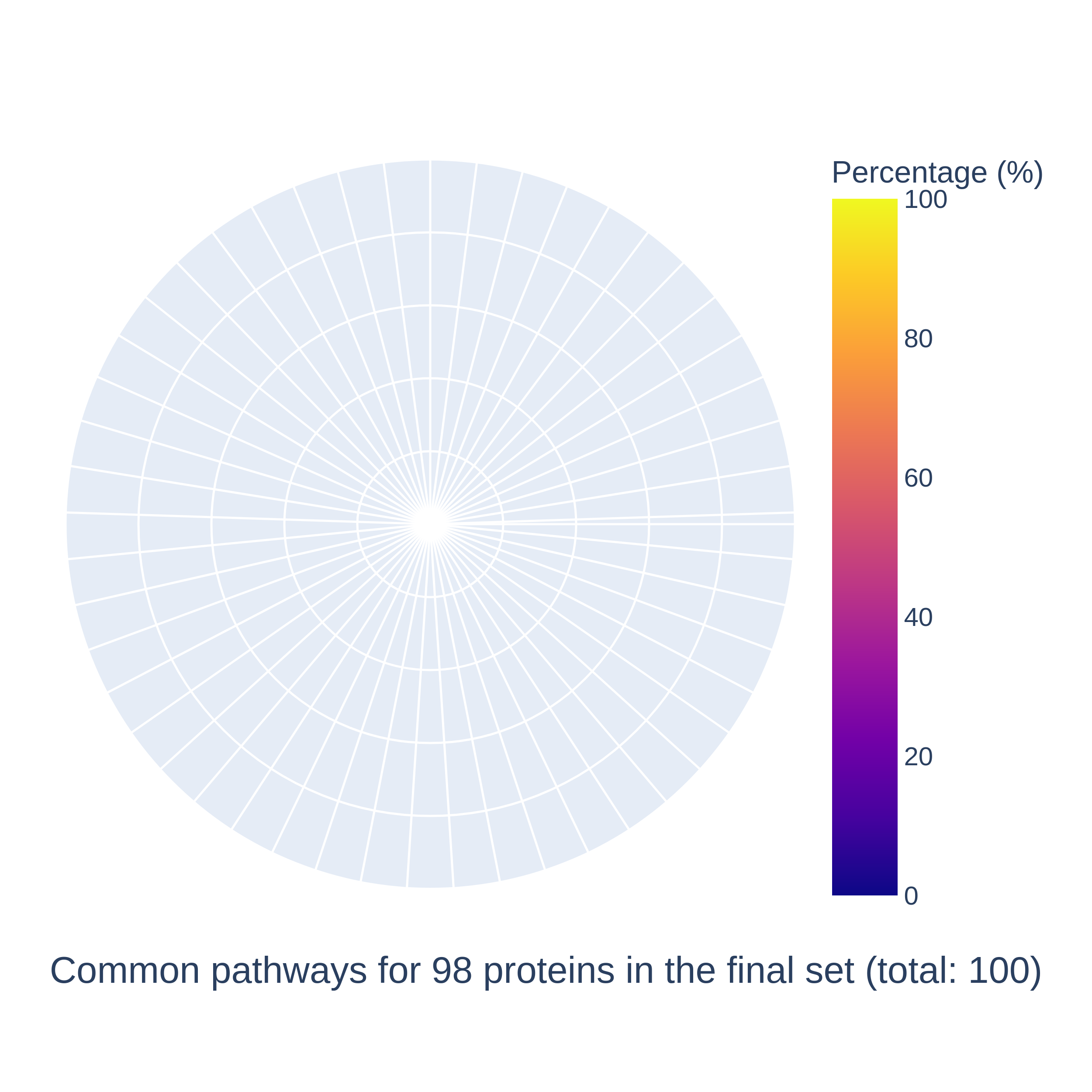

Supplement: Supplementary file 9 — Supplementary Data 6 [file 42003_2023_5076_MOESM9_ESM.zip › 6VXX_A_whole_alphafold4_dataset/plots/6VXX_A_biologicalProcessSim.png]

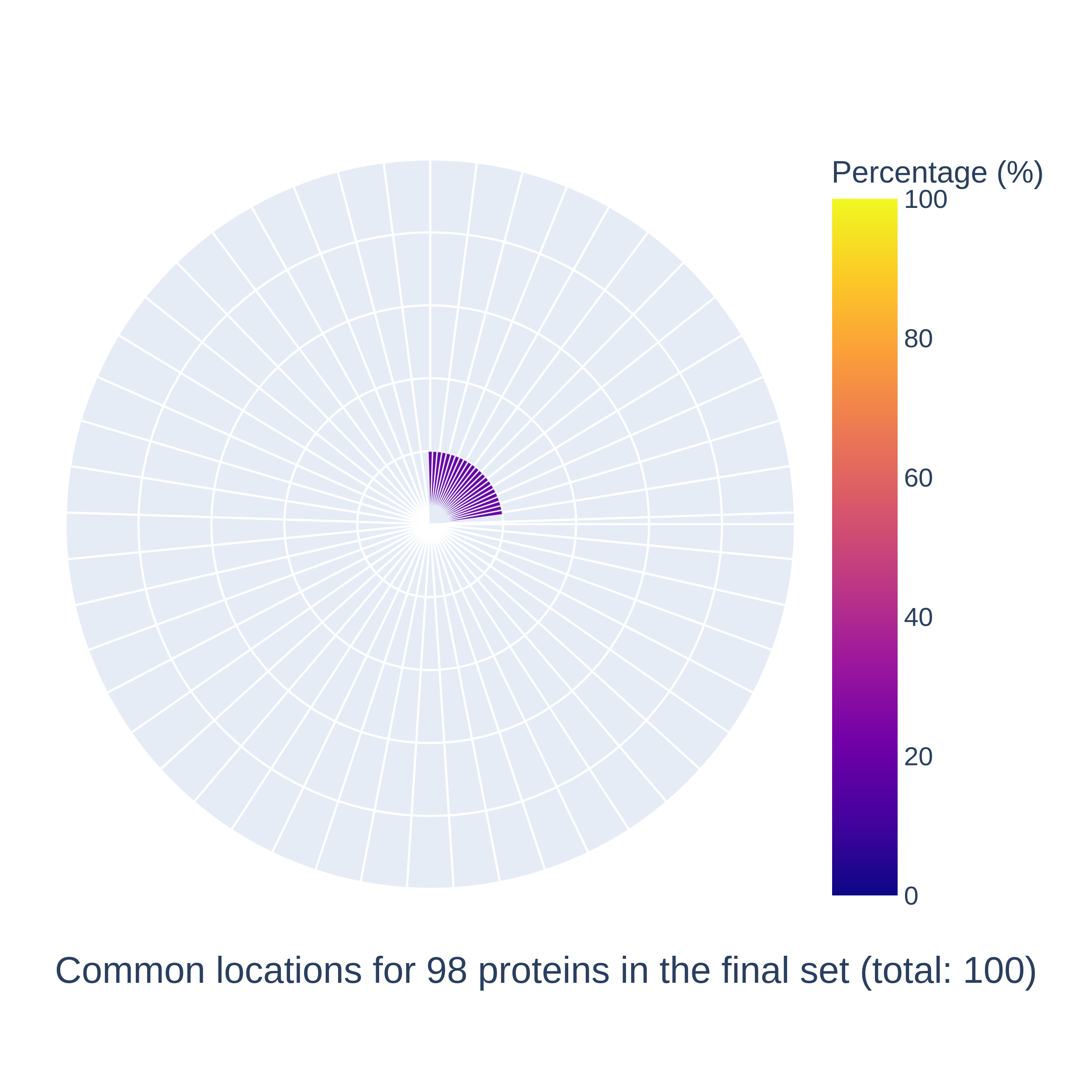

Supplement: Supplementary file 9 — Supplementary Data 6 [file 42003_2023_5076_MOESM9_ESM.zip › 6VXX_A_whole_alphafold4_dataset/plots/6VXX_A_cellularComponentSim.png]

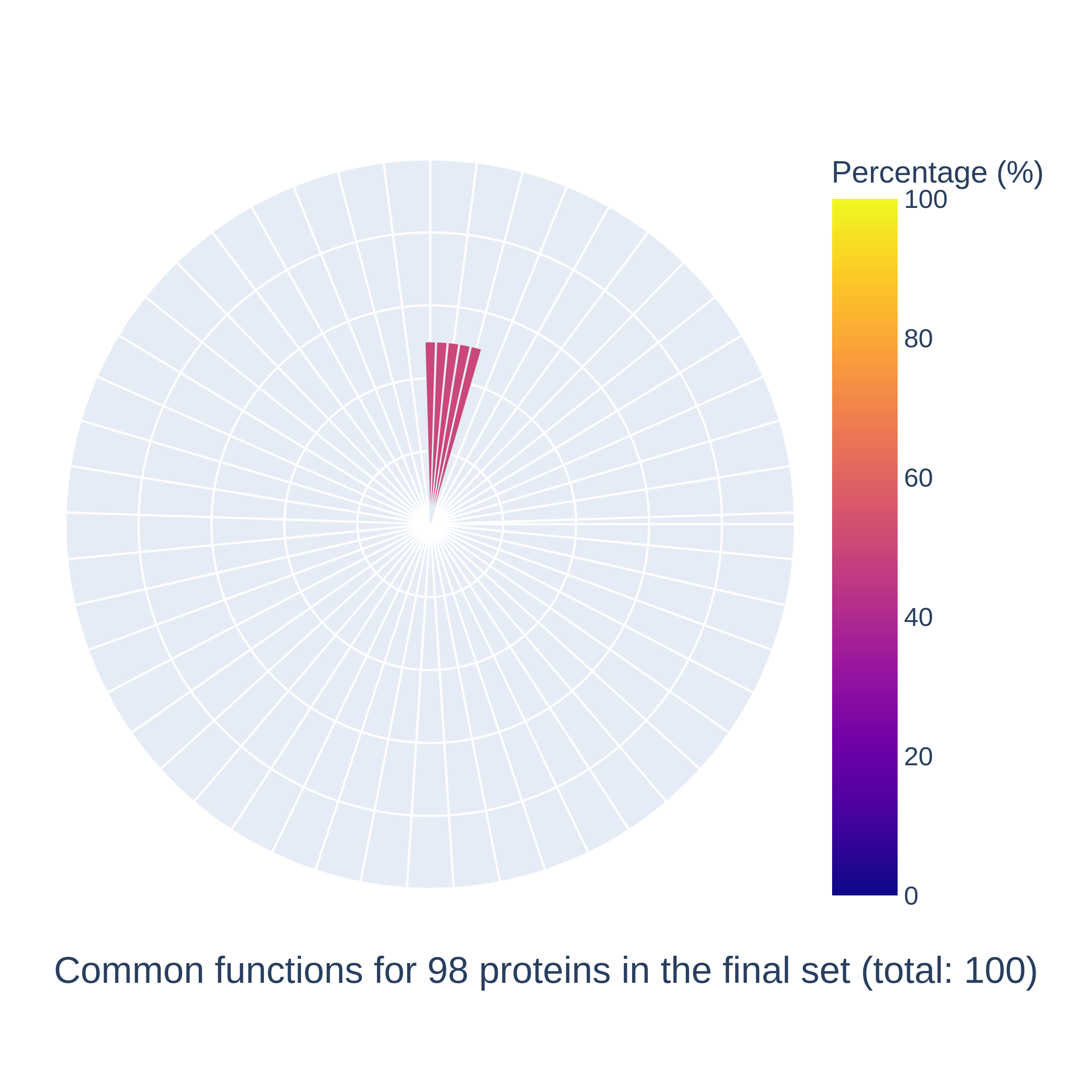

Supplement: Supplementary file 9 — Supplementary Data 6 [file 42003_2023_5076_MOESM9_ESM.zip › 6VXX_A_whole_alphafold4_dataset/plots/6VXX_A_molecularFunctionSim.png]

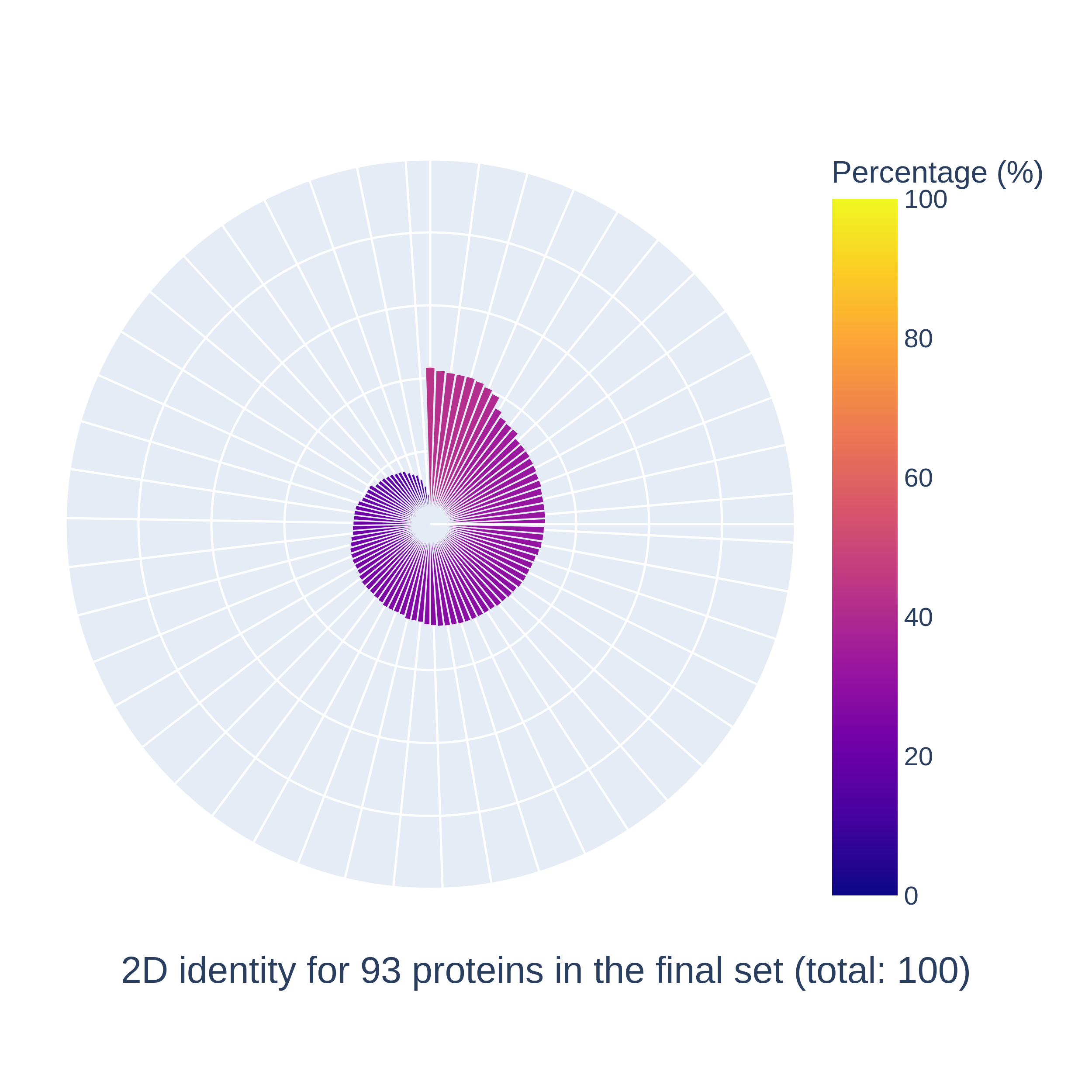

Supplement: Supplementary file 9 — Supplementary Data 6 [file 42003_2023_5076_MOESM9_ESM.zip › 6VXX_A_whole_alphafold4_dataset/plots/6VXX_A_2D-identity.png]

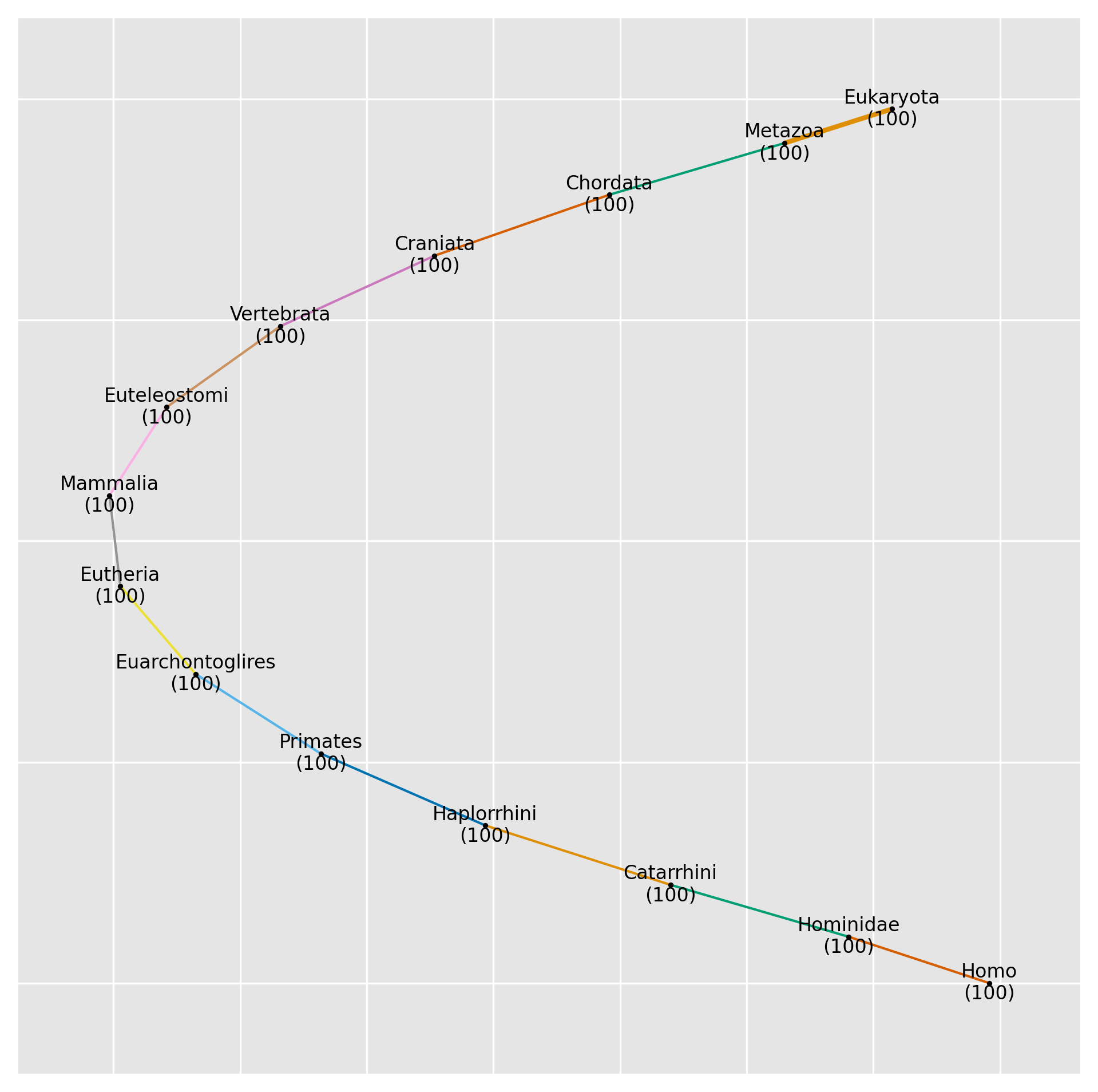

Supplement: Supplementary file 9 — Supplementary Data 6 [file 42003_2023_5076_MOESM9_ESM.zip › 6VXX_A_whole_alphafold4_dataset/plots/6VXX_A-Eukaryota-tree.png]

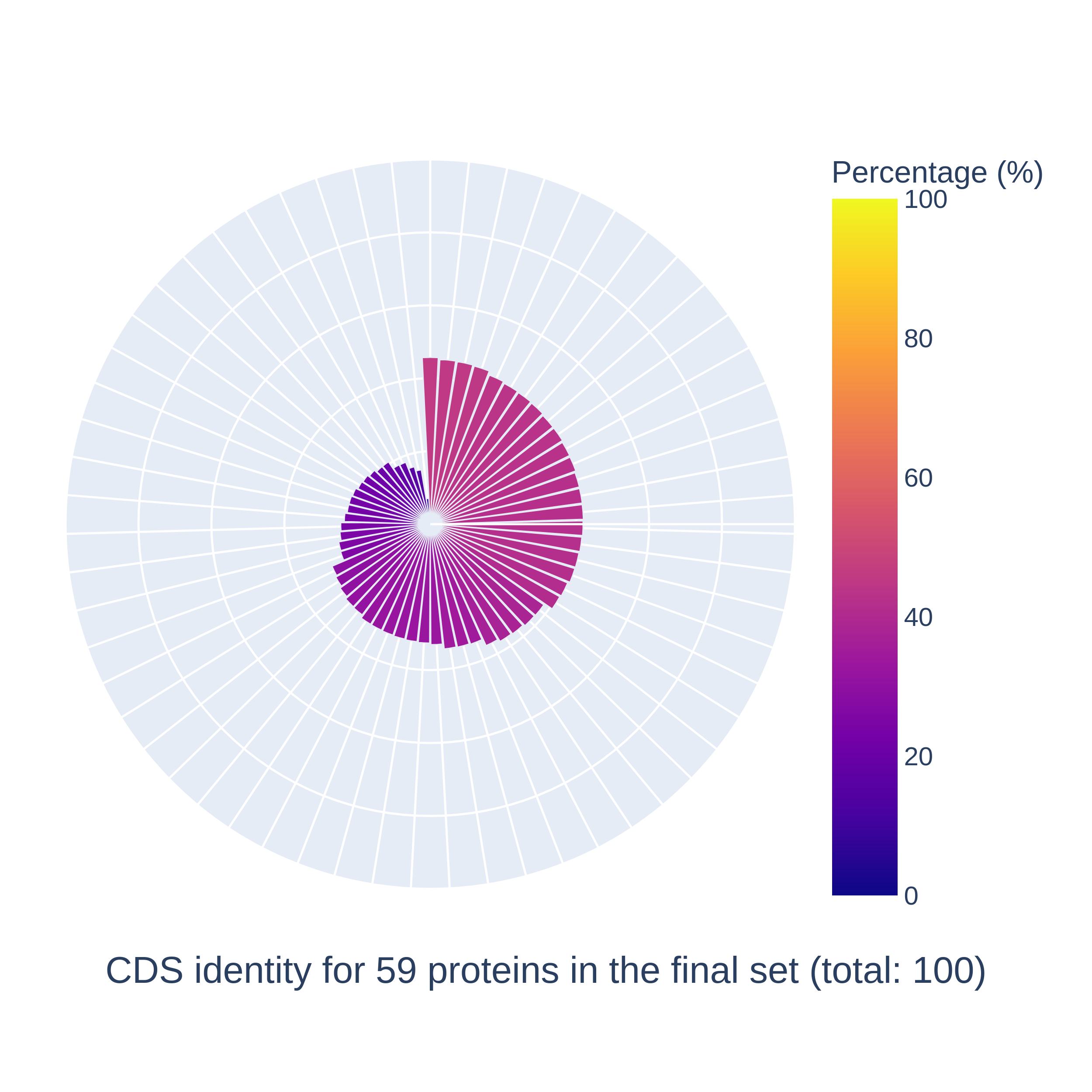

Supplement: Supplementary file 9 — Supplementary Data 6 [file 42003_2023_5076_MOESM9_ESM.zip › 6VXX_A_whole_alphafold4_dataset/plots/6VXX_A_CDS-identity.png]

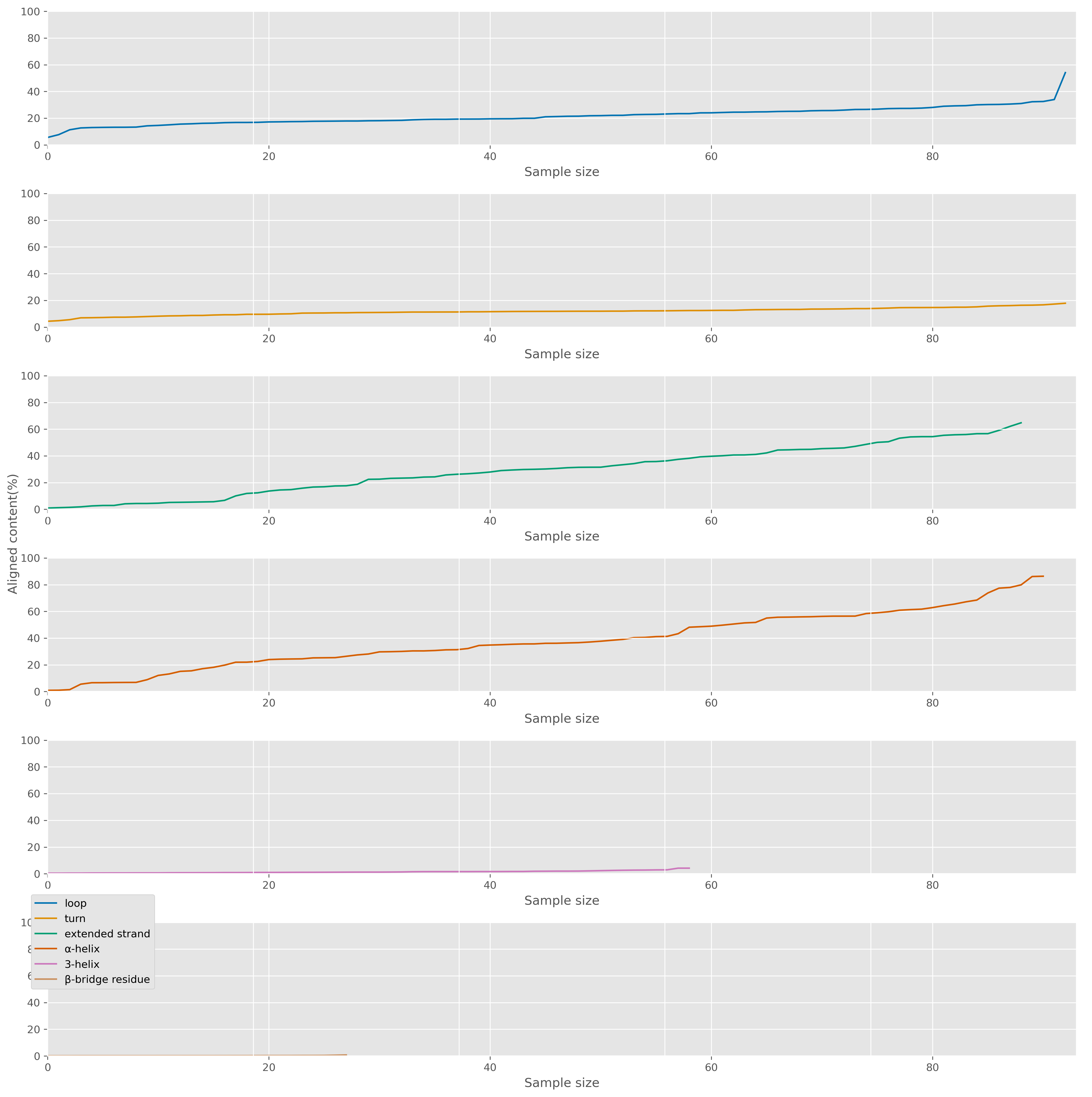

Supplement: Supplementary file 9 — Supplementary Data 6 [file 42003_2023_5076_MOESM9_ESM.zip › 6VXX_A_whole_alphafold4_dataset/plots/6VXX_A-2Dfold_coverage.png]

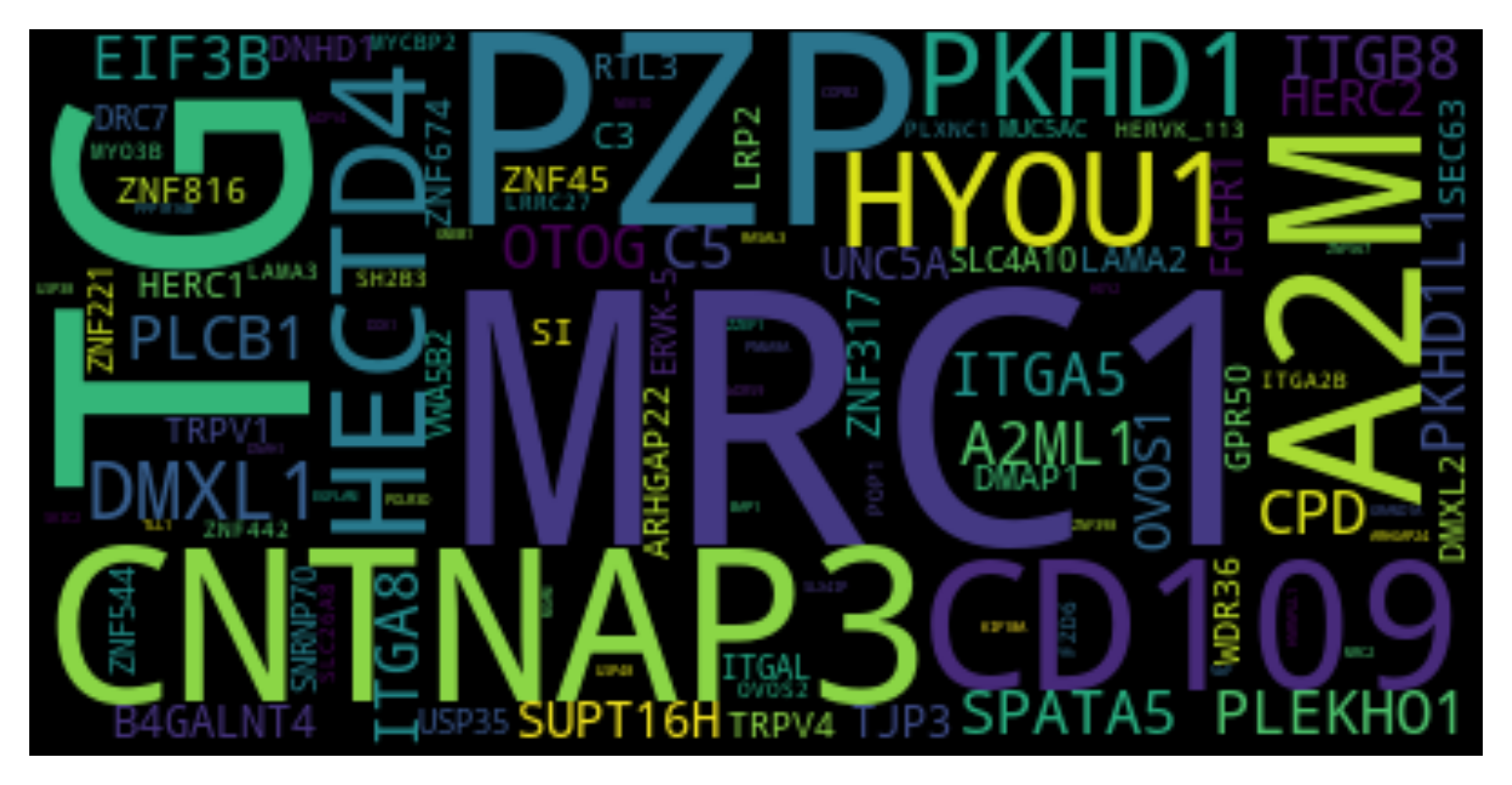

Supplement: Supplementary file 9 — Supplementary Data 6 [file 42003_2023_5076_MOESM9_ESM.zip › 6VXX_A_whole_alphafold4_dataset/plots/6VXX_A-wordcloud.png]
