# Supplementary material for: Identifying and profiling structural similarities between Spike of SARS-CoV-2 and other viral or host proteins with Machaon
Source: Commun Biol. 2023 Jul 19;6:752. doi: 10.1038/s42003-023-05076-7 (PMC10356814; doi:10.1038/s42003-023-05076-7)
Supplement: Supplementary file 8 — Supplementary Data 5 [file 42003_2023_5076_MOESM8_ESM.zip › 6VXX_A_whole_human_exp_dataset/candidates/6VXX_A-merged-enriched_eval_report.html]

 

# Structural Comparison Report for 6VXX\_A - whole structures (total: 35)

---

1

- **Protein name:** Spike glycoprotein
- **Organism:** Middle East respiratory syndrome-related coronavirus
- **Uniprot Accession Number:** A0A140AYW5
- **Protein sequence length:** 1353 aa
- **1D identity (%):** 30.76
- **1D identity (%) [Gaps excluded]:** 35.18
- **1D identity - Alignment Gaps:** 176
- **1D aligned content (<aminoacid>:%):** {'M': 0.46, 'V': 6.96, 'F': 7.66, 'L': 10.21, 'S': 6.03, 'C': 7.19, 'P': 5.8, 'G': 9.74, 'Y': 5.1, 'Q': 3.48, 'A': 6.5, 'H': 1.16, 'T': 4.64, 'K': 3.25, 'I': 4.64, 'R': 3.25, 'D': 4.64, 'N': 5.1, 'W': 1.16, 'E': 3.02}
- **Common reported functions (%):** 0.0
- **Common reported locations (%):** 80.0
- **Common reported processes (%):** 30.0

- **PDB ID:** 6NB4
- **Chain:** C
- **Crystallized protein length:** 959 aa
- **Resolution:** 3.6 Å
- **b-phipsi:** 0.001068
- **w-rdist:** 0.420809
- **t-alpha:** 0.001654
- **Chemical similarity (Tanimoto Index) (%):** 94.56
- **1D identity (%) [PDB]:** 13.01
- **1D identity (%) [Gaps excluded][PDB]:** 58.15
- **1D identity - Alignment Gaps [PDB]:** 1235
- **1D aligned content [PDB] (<aminoacid>:%):** {'T': 5.31, 'S': 6.76, 'N': 6.76, 'V': 6.28, 'A': 11.59, 'Q': 6.28, 'D': 3.86, 'G': 9.18, 'I': 7.73, 'E': 2.9, 'Y': 3.38, 'M': 0.97, 'L': 10.14, 'P': 4.35, 'F': 5.8, 'K': 3.86, 'R': 3.38, 'W': 0.48, 'H': 0.97}
- **2D identity (%) [PDB]:** 56.37
- **2D identity (%) [Gaps excluded][PDB]:** 84.7
- **2D identity - Alignment Gaps [PDB]:** 391
- **2D aligned content [PDB] (<2D-fold>:%):** {'.': 18.06, 'B': 0.46, 'S': 6.53, 'E': 38.24, 'P': 1.21, 'T': 9.71, 'H': 24.58, 'G': 0.46, 'I': 0.76}
- **3D similarity (TM-Score) (%) [PDB]:** 74.62

- **Gene name:** S
- **Entrez ID:** N/A
- **RefSeq ID:** N/A
- **Sequence length:** N/A
- **5-UTR|CDS|3-UTR identity (%):** N/A | N/A | N/A
- **5-UTR|CDS|3-UTR identity (%) [Gaps excluded]:** N/A | N/A | N/A
- **5-UTR|CDS|3-UTR identity [Alignment Gaps]:** N/A | N/A | N/A
- **5-UTR aligned content (<base>:%):** N/A
- **CDS aligned content (<base>:%):** N/A
- **3-UTR aligned content (<base>:%):** N/A

**Uniprot Description:**  
  
 Spike protein S1: attaches the virion to the cell membrane by interacting with host receptor, initiating the infection.   
  
Homotrimer; each monomer consists of a S1 and a S2 subunit. The resulting peplomers protrude from the virus surface as spikes.   
  
 **Gene Ontology Information:**

Molecular Function   
  
N/A

Location

- host cell endoplasmic reticulum-Golgi intermediate compartment membrane
- host cell plasma membrane
- integral component of membrane
- viral envelope
- virion membrane

Biological process

- endocytosis involved in viral entry into host cell
- fusion of virus membrane with host endosome membrane
- fusion of virus membrane with host plasma membrane
- pathogenesis
- receptor-mediated virion attachment to host cell

---

2

- **Protein name:** Spike glycoprotein
- **Organism:** Severe acute respiratory syndrome coronavirus
- **Uniprot Accession Number:** P59594
- **Protein sequence length:** 1255 aa
- **1D identity (%):** 76.35
- **1D identity (%) [Gaps excluded]:** 77.94
- **1D identity - Alignment Gaps:** 26
- **1D aligned content (<aminoacid>:%):** {'M': 1.13, 'F': 7.08, 'L': 8.41, 'S': 6.26, 'T': 7.28, 'P': 5.13, 'Y': 4.72, 'R': 3.08, 'G': 7.18, 'V': 7.28, 'D': 5.33, 'Q': 4.41, 'N': 6.77, 'H': 0.92, 'I': 5.95, 'A': 6.26, 'E': 3.38, 'K': 4.51, 'W': 1.03, 'C': 3.9}
- **Common reported functions (%):** 100.0
- **Common reported locations (%):** 80.0
- **Common reported processes (%):** 90.0

- **PDB ID:** 6NB6
- **Chain:** A
- **Crystallized protein length:** 1052 aa
- **Resolution:** 4.2 Å
- **b-phipsi:** 0.001058
- **w-rdist:** 0.5121
- **t-alpha:** 0.002481
- **Chemical similarity (Tanimoto Index) (%):** 94.56
- **1D identity (%) [PDB]:** 70.31
- **1D identity (%) [Gaps excluded][PDB]:** 77.55
- **1D identity - Alignment Gaps [PDB]:** 100
- **1D aligned content [PDB] (<aminoacid>:%):** {'S': 6.24, 'R': 3.32, 'G': 7.44, 'V': 8.1, 'Y': 5.05, 'P': 5.84, 'D': 5.05, 'F': 7.97, 'L': 8.1, 'T': 7.97, 'Q': 5.05, 'N': 7.7, 'H': 0.8, 'I': 5.98, 'A': 6.51, 'E': 3.19, 'K': 3.98, 'W': 0.66, 'M': 1.06}
- **2D identity (%) [PDB]:** 73.72
- **2D identity (%) [Gaps excluded][PDB]:** 85.41
- **2D identity - Alignment Gaps [PDB]:** 150
- **2D aligned content [PDB] (<2D-fold>:%):** {'.': 21.16, 'E': 37.25, 'T': 8.66, 'S': 7.43, 'H': 21.91, 'P': 0.99, 'B': 0.5, 'G': 1.49, 'I': 0.62}
- **3D similarity (TM-Score) (%) [PDB]:** 96.46

- **Gene name:** S
- **Entrez ID:** 1489668
- **RefSeq ID:** NC\_004718
- **Genomic sequence length:** 29751
- **5-UTR|CDS|3-UTR identity (%):** 88.81 | 73.63 | 22.38
- **5-UTR|CDS|3-UTR identity (%) [Gaps excluded]:** 91.19 | 78.35 | 98.18
- **5-UTR|CDS|3-UTR identity [Alignment Gaps]:** 7 | 236 | 745
- **5-UTR aligned content (<base>:%):** {'A': 25.21, 'T': 28.99, 'G': 21.85, 'C': 23.95}
- **CDS aligned content (<base>:%):** {'A': 28.81, 'T': 34.05, 'G': 18.92, 'C': 18.22}
- **3-UTR aligned content (<base>:%):** {'C': 15.74, 'A': 38.89, 'T': 24.07, 'G': 21.3}

**Uniprot Description:**  
  
 Spike glycoprotein
May down-regulate host tetherin (BST2) by lysosomal degradation, thereby counteracting its antiviral activity.   
  
Homotrimer; each monomer consists of a S1 and a S2 subunit. The resulting peplomers protrude from the virus surface as spikes (By similarity). Binds to human and palm civet ACE2 and human CLEC4M/DC-SIGNR. Interacts with the accessory proteins 3a and 7a.   
  
 **Gene Ontology Information:**

Molecular Function

- host cell surface receptor binding
- identical protein binding

Location

- host cell endoplasmic reticulum-Golgi intermediate compartment membrane
- host cell plasma membrane
- membrane
- viral envelope
- virion membrane

Biological process

- endocytosis involved in viral entry into host cell
- fusion of virus membrane with host endosome membrane
- fusion of virus membrane with host plasma membrane
- membrane fusion
- positive regulation of viral entry into host cell
- receptor-mediated endocytosis of virus by host cell
- receptor-mediated virion attachment to host cell
- suppression by virus of host tetherin activity
- suppression by virus of host type I interferon-mediated signaling pathway
- viral entry into host cell

---

3

- **Protein name:** Spike glycoprotein
- **Organism:** Severe acute respiratory syndrome coronavirus 2
- **Uniprot Accession Number:** P0DTC2
- **Protein sequence length:** 1273 aa
- **1D identity (%):** 100.0
- **1D identity (%) [Gaps excluded]:** 100.0
- **1D identity - Alignment Gaps:** N/A
- **1D aligned content (<aminoacid>:%):** {'M': 1.1, 'F': 6.05, 'V': 7.62, 'L': 8.48, 'P': 4.56, 'S': 7.78, 'Q': 4.87, 'C': 3.14, 'N': 6.91, 'T': 7.62, 'R': 3.3, 'A': 6.21, 'Y': 4.24, 'G': 6.44, 'D': 4.87, 'K': 4.79, 'H': 1.34, 'W': 0.94, 'I': 5.97, 'E': 3.77}
- **Common reported functions (%):** 0.0
- **Common reported locations (%):** 0.0
- **Common reported processes (%):** 0.0

- **PDB ID:** 7WLZ
- **Chain:** A
- **Crystallized protein length:** 1028 aa
- **Resolution:** 2.98 Å
- **b-phipsi:** 0.000581
- **w-rdist:** 0.241686
- **t-alpha:** 0.015113
- **Chemical similarity (Tanimoto Index) (%):** 100.0
- **1D identity (%) [PDB]:** 90.09
- **1D identity (%) [Gaps excluded][PDB]:** 95.51
- **1D identity - Alignment Gaps [PDB]:** 59
- **1D aligned content [PDB] (<aminoacid>:%):** {'A': 6.41, 'Y': 4.27, 'T': 8.33, 'N': 7.05, 'S': 7.37, 'F': 6.94, 'R': 3.21, 'G': 6.09, 'V': 8.12, 'P': 5.02, 'D': 4.91, 'K': 4.7, 'L': 8.55, 'H': 1.39, 'Q': 5.02, 'W': 0.64, 'E': 3.85, 'I': 6.52, 'C': 0.32, 'B': 0.21, 'M': 0.85, 'J': 0.21}
- **2D identity (%) [PDB]:** 75.9
- **2D identity (%) [Gaps excluded][PDB]:** 83.56
- **2D identity - Alignment Gaps [PDB]:** 97
- **2D aligned content [PDB] (<2D-fold>:%):** {'.': 22.91, 'E': 33.37, 'S': 9.09, 'T': 8.47, 'H': 22.67, 'P': 1.0, 'B': 0.5, 'G': 1.37, 'I': 0.62}
- **3D similarity (TM-Score) (%) [PDB]:** 97.3

- **Gene name:** S
- **Entrez ID:** N/A
- **RefSeq ID:** NC\_045512
- **Genomic sequence length:** 29903
- **5-UTR|CDS|3-UTR identity (%):** 100.0 | 100.0 | 100.0
- **5-UTR|CDS|3-UTR identity (%) [Gaps excluded]:** 100.0 | 100.0 | 100.0
- **5-UTR|CDS|3-UTR identity [Alignment Gaps]:** N/A | N/A | N/A
- **5-UTR aligned content (<base>:%):** {'A': 25.28, 'T': 30.19, 'G': 20.75, 'C': 23.77}
- **CDS aligned content (<base>:%):** {'A': 29.43, 'T': 33.25, 'G': 18.39, 'C': 18.92}
- **3-UTR aligned content (<base>:%):** {'C': 15.72, 'A': 40.61, 'T': 23.14, 'G': 20.52}

**Uniprot Description:**  
  
 Spike protein S1
attaches the virion to the cell membrane by interacting with host receptor, initiating the infection. Binding to human ACE2 receptor and internalization of the virus into the endosomes of the host cell induces conformational changes in the Spike glycoprotein (PubMed:32142651, PubMed:32075877, PubMed:32155444). Binding to host NRP1 and NRP2 via C-terminal polybasic sequence enhances virion entry into host cell (PubMed:33082294, PubMed:33082293). This interaction may explain virus tropism of human olfactory epithelium cells, which express high level of NRP1 and NRP2 but low level of ACE2 (PubMed:33082293). The stalk domain of S contains three hinges, giving the head unexpected orientational freedom (PubMed:32817270). Uses human TMPRSS2 for priming in human lung cells which is an essential step for viral entry (PubMed:32142651). Can be alternatively processed by host furin (PubMed:32362314). Proteolysis by cathepsin CTSL may unmask the fusion peptide of S2 and activate membranes fusion within endosomes.   
  
Spike glycoprotein
Homotrimer; each monomer consists of a S1 and a S2 subunit (PubMed:32075877, PubMed:32155444, PubMed:32245784). The resulting peplomers protrude from the virus surface as spikes (PubMed:32979942). Interacts with ORF3a protein and ORF7a protein (By similarity) (PubMed:32075877, PubMed:32155444, PubMed:32245784, PubMed:32979942). There are an average of 26 +/-15 spike trimers at the surface of virion particles (PubMed:32979942).   
  
 **Gene Ontology Information:**

Molecular Function   
  
N/A

Location   
  
N/A

Biological process   
  
N/A

---

4

- **Protein name:** Complement C5
- **Organism:** Homo sapiens
- **Uniprot Accession Number:** P01031
- **Protein sequence length:** 1676 aa
- **1D identity (%):** 17.13
- **1D identity (%) [Gaps excluded]:** 24.24
- **1D identity - Alignment Gaps:** 507
- **1D aligned content (<aminoacid>:%):** {'M': 0.34, 'F': 6.42, 'L': 13.18, 'S': 7.77, 'N': 7.09, 'Y': 4.05, 'T': 8.45, 'P': 5.74, 'D': 5.07, 'K': 7.77, 'H': 1.01, 'V': 6.76, 'G': 6.76, 'I': 4.73, 'A': 3.38, 'E': 3.72, 'R': 2.36, 'C': 3.04, 'Q': 2.36}
- **Common reported functions (%):** 0.0
- **Common reported locations (%):** 0.0
- **Common reported processes (%):** 0.0

- **PDB ID:** 5I5K
- **Chain:** A
- **Crystallized protein length:** 1632 aa
- **Resolution:** 4.2 Å
- **b-phipsi:** 0.004354
- **w-rdist:** 0.342824
- **t-alpha:** 0.007444
- **Chemical similarity (Tanimoto Index) (%):** N/A
- **1D identity (%) [PDB]:** 4.76
- **1D identity (%) [Gaps excluded][PDB]:** 64.8
- **1D identity - Alignment Gaps [PDB]:** 2260
- **1D aligned content [PDB] (<aminoacid>:%):** {'Q': 5.17, 'Y': 1.72, 'K': 6.9, 'P': 3.45, 'F': 5.17, 'G': 8.62, 'N': 6.03, 'S': 8.62, 'L': 15.52, 'D': 3.45, 'R': 2.59, 'E': 4.31, 'V': 5.17, 'T': 6.03, 'I': 5.17, 'A': 11.21, 'M': 0.86}
- **2D identity (%) [PDB]:** 38.42
- **2D identity (%) [Gaps excluded][PDB]:** 87.94
- **2D identity - Alignment Gaps [PDB]:** 1026
- **2D aligned content [PDB] (<2D-fold>:%):** {'.': 17.29, 'E': 42.71, 'T': 6.86, 'S': 7.43, 'P': 0.43, 'H': 25.0, 'B': 0.29}
- **3D similarity (TM-Score) (%) [PDB]:** 29.05

- **Gene name:** C5
- **Entrez ID:** 727
- **RefSeq ID:** NM\_001735
- **Transcript sequence length:** 5464
- **5-UTR|CDS|3-UTR identity (%):** 6.67 | 45.0 | 38.15
- **5-UTR|CDS|3-UTR identity (%) [Gaps excluded]:** 75.0 | 75.8 | 76.3
- **5-UTR|CDS|3-UTR identity [Alignment Gaps]:** 246 | 2257 | 211
- **5-UTR aligned content (<base>:%):** {'A': 27.78, 'T': 22.22, 'C': 44.44, 'G': 5.56}
- **CDS aligned content (<base>:%):** {'A': 31.68, 'T': 33.88, 'G': 17.64, 'C': 16.8}
- **3-UTR aligned content (<base>:%):** {'A': 47.2, 'T': 19.25, 'C': 15.53, 'G': 18.01}

**Uniprot Description:**  
  
 Activation of C5 by a C5 convertase initiates the spontaneous assembly of the late complement components, C5-C9, into the membrane attack complex. C5b has a transient binding site for C6. The C5b-C6 complex is the foundation upon which the lytic complex is assembled.   
  
C5 precursor is first processed by the removal of 4 basic residues, forming two chains, beta and alpha, linked by a disulfide bond. C5 convertase activates C5 by cleaving the alpha chain, releasing C5a anaphylatoxin and generating C5b (beta chain + alpha' chain). The C5a anaphylatoxin interacts with C5AR1. Interacts with tick complement inhibitor.   
  
 **Gene Ontology Information:**

Molecular Function

- chemokine activity
- endopeptidase inhibitor activity
- signaling receptor binding

Location

- extracellular exosome
- extracellular region
- extracellular space
- membrane attack complex

Biological process

- cell surface receptor signaling pathway
- chemotaxis
- complement activation
- complement activation, alternative pathway
- complement activation, classical pathway
- cytolysis
- G protein-coupled receptor signaling pathway
- in utero embryonic development
- inflammatory response
- negative regulation of macrophage chemotaxis
- positive regulation of angiogenesis
- positive regulation of chemokine production
- positive regulation of immune response
- positive regulation of vascular endothelial growth factor production

---

5

- **Protein name:** Apoptotic protease-activating factor 1
- **Organism:** Homo sapiens
- **Uniprot Accession Number:** O14727
- **Protein sequence length:** 1248 aa
- **1D identity (%):** 14.26
- **1D identity (%) [Gaps excluded]:** 19.59
- **1D identity - Alignment Gaps:** 397
- **1D aligned content (<aminoacid>:%):** {'C': 3.37, 'L': 10.58, 'R': 4.33, 'S': 12.5, 'K': 4.33, 'V': 6.73, 'A': 6.25, 'G': 10.58, 'D': 7.21, 'T': 6.73, 'N': 5.29, 'W': 0.96, 'F': 5.29, 'P': 3.85, 'E': 1.92, 'I': 4.33, 'Y': 0.96, 'Q': 2.88, 'H': 1.92}
- **Common reported functions (%):** 50.0
- **Common reported locations (%):** 0.0
- **Common reported processes (%):** 0.0

- **PDB ID:** 5JUY
- **Chain:** E
- **Crystallized protein length:** 1234 aa
- **Resolution:** 4.1 Å
- **b-phipsi:** 0.006124
- **w-rdist:** 0.494608
- **t-alpha:** 0.001657
- **Chemical similarity (Tanimoto Index) (%):** 81.1
- **1D identity (%) [PDB]:** 2.72
- **1D identity (%) [Gaps excluded][PDB]:** 69.88
- **1D identity - Alignment Gaps [PDB]:** 2053
- **1D aligned content [PDB] (<aminoacid>:%):** {'A': 5.17, 'I': 6.9, 'G': 6.9, 'K': 5.17, 'Q': 10.34, 'D': 6.9, 'S': 13.79, 'L': 20.69, 'T': 6.9, 'V': 8.62, 'F': 1.72, 'R': 1.72, 'E': 3.45, 'Y': 1.72}
- **2D identity (%) [PDB]:** 23.76
- **2D identity (%) [Gaps excluded][PDB]:** 89.87
- **2D identity - Alignment Gaps [PDB]:** 1291
- **2D aligned content [PDB] (<2D-fold>:%):** {'T': 10.79, 'S': 9.59, '.': 17.51, 'H': 42.21, 'P': 1.2, 'E': 17.99, 'G': 0.72}
- **3D similarity (TM-Score) (%) [PDB]:** 24.92

- **Gene name:** APAF1
- **Entrez ID:** 317
- **RefSeq ID:** NM\_181869
- **Transcript sequence length:** 4556
- **5-UTR|CDS|3-UTR identity (%):** 28.78 | 15.45 | 7.24
- **5-UTR|CDS|3-UTR identity (%) [Gaps excluded]:** 74.68 | 73.66 | 94.27
- **5-UTR|CDS|3-UTR identity [Alignment Gaps]:** 378 | 3161 | 2727
- **5-UTR aligned content (<base>:%):** {'A': 21.47, 'G': 25.99, 'T': 24.86, 'C': 27.68}
- **CDS aligned content (<base>:%):** {'T': 30.26, 'G': 21.04, 'C': 16.34, 'A': 32.36}
- **3-UTR aligned content (<base>:%):** {'A': 40.65, 'T': 22.9, 'C': 15.42, 'G': 21.03}

**Uniprot Description:**  
  
 Oligomeric Apaf-1 mediates the cytochrome c-dependent autocatalytic activation of pro-caspase-9 (Apaf-3), leading to the activation of caspase-3 and apoptosis. This activation requires ATP. Isoform 6 is less effective in inducing apoptosis.   
  
Monomer. Oligomerizes to a heptameric ring, known as the apoptosome, upon binding of cytochrome c and dATP. Oligomeric Apaf-1 and pro-caspase-9 bind to each other via their respective NH2-terminal CARD domains and consecutively mature caspase-9 is released from the complex. Pro-caspase-3 is recruited into the Apaf-1-pro-caspase-9 complex via interaction with pro-caspase-9. Interacts with APIP. Interacts (via CARD and NACHT domains) with NAIP/BIRC1 (via NACHT domain). Interacts with CIAO2A (PubMed:25716227).   
  
 **Gene Ontology Information:**

Molecular Function

- ADP binding
- ATP binding
- cysteine-type endopeptidase activator activity involved in apoptotic process
- heat shock protein binding
- identical protein binding
- nucleotide binding

Location

- apoptosome
- cytosol
- extracellular exosome
- extracellular region
- ficolin-1-rich granule lumen
- nucleus
- protein-containing complex
- secretory granule lumen

Biological process

- activation of cysteine-type endopeptidase activity
- activation of cysteine-type endopeptidase activity involved in apoptotic process
- activation of cysteine-type endopeptidase activity involved in apoptotic process by cytochrome c
- aging
- apoptotic process
- cardiac muscle cell apoptotic process
- cell differentiation
- cellular response to transforming growth factor beta stimulus
- forebrain development
- intrinsic apoptotic signaling pathway
- intrinsic apoptotic signaling pathway in response to endoplasmic reticulum stress
- kidney development
- nervous system development
- neural tube closure
- neuron apoptotic process
- positive regulation of apoptotic process
- positive regulation of apoptotic signaling pathway
- regulation of apoptotic DNA fragmentation
- regulation of apoptotic process
- response to G1 DNA damage checkpoint signaling
- response to hypoxia
- response to nutrient

---

6

- **Protein name:** Insulin-like growth factor 1 receptor
- **Organism:** Homo sapiens
- **Uniprot Accession Number:** P08069
- **Protein sequence length:** 1367 aa
- **1D identity (%):** 16.69
- **1D identity (%) [Gaps excluded]:** 23.81
- **1D identity - Alignment Gaps:** 464
- **1D aligned content (<aminoacid>:%):** {'S': 7.34, 'L': 8.49, 'F': 6.18, 'A': 6.18, 'T': 5.41, 'G': 7.72, 'P': 6.95, 'N': 9.27, 'D': 5.41, 'E': 4.63, 'I': 4.63, 'K': 4.63, 'Y': 5.41, 'R': 3.09, 'C': 2.7, 'V': 6.56, 'Q': 2.32, 'W': 1.16, 'M': 1.54, 'H': 0.39}
- **Common reported functions (%):** 50.0
- **Common reported locations (%):** 20.0
- **Common reported processes (%):** 0.0

- **PDB ID:** 5U8R
- **Chain:** A
- **Crystallized protein length:** 801 aa
- **Resolution:** 3.0 Å
- **b-phipsi:** 0.004542
- **w-rdist:** 0.559004
- **t-alpha:** 0.004988
- **Chemical similarity (Tanimoto Index) (%):** 95.01
- **1D identity (%) [PDB]:** 2.07
- **1D identity (%) [Gaps excluded][PDB]:** 75.0
- **1D identity - Alignment Gaps [PDB]:** 1693
- **1D aligned content [PDB] (<aminoacid>:%):** {'L': 13.89, 'K': 5.56, 'E': 8.33, 'N': 8.33, 'T': 8.33, 'I': 11.11, 'D': 5.56, 'A': 2.78, 'V': 8.33, 'P': 5.56, 'S': 5.56, 'F': 8.33, 'G': 2.78, 'Y': 2.78, 'R': 2.78}
- **2D identity (%) [PDB]:** 28.78
- **2D identity (%) [Gaps excluded][PDB]:** 80.3
- **2D identity - Alignment Gaps [PDB]:** 845
- **2D aligned content [PDB] (<2D-fold>:%):** {'E': 55.41, '.': 26.39, 'S': 6.07, 'T': 7.92, 'H': 2.11, 'G': 0.79, 'B': 0.26, 'P': 1.06}
- **3D similarity (TM-Score) (%) [PDB]:** 17.93

- **Gene name:** IGF1R
- **Entrez ID:** 3480
- **RefSeq ID:** NM\_001291858
- **Transcript sequence length:** 12232
- **5-UTR|CDS|3-UTR identity (%):** 18.21 | 43.96 | 3.12
- **5-UTR|CDS|3-UTR identity (%) [Gaps excluded]:** 77.82 | 73.38 | 96.93
- **5-UTR|CDS|3-UTR identity [Alignment Gaps]:** 812 | 1987 | 6861
- **5-UTR aligned content (<base>:%):** {'A': 20.21, 'G': 25.91, 'T': 25.39, 'C': 28.5}
- **CDS aligned content (<base>:%):** {'A': 28.56, 'T': 26.68, 'G': 21.9, 'C': 22.87}
- **3-UTR aligned content (<base>:%):** {'A': 41.63, 'T': 22.17, 'C': 15.38, 'G': 20.81}

**Uniprot Description:**  
  
 Receptor tyrosine kinase which mediates actions of insulin-like growth factor 1 (IGF1). Binds IGF1 with high affinity and IGF2 and insulin (INS) with a lower affinity. The activated IGF1R is involved in cell growth and survival control. IGF1R is crucial for tumor transformation and survival of malignant cell. Ligand binding activates the receptor kinase, leading to receptor autophosphorylation, and tyrosines phosphorylation of multiple substrates, that function as signaling adapter proteins including, the insulin-receptor substrates (IRS1/2), Shc and 14-3-3 proteins. Phosphorylation of IRSs proteins lead to the activation of two main signaling pathways: the PI3K-AKT/PKB pathway and the Ras-MAPK pathway. The result of activating the MAPK pathway is increased cellular proliferation, whereas activating the PI3K pathway inhibits apoptosis and stimulates protein synthesis. Phosphorylated IRS1 can activate the 85 kDa regulatory subunit of PI3K (PIK3R1), leading to activation of several downstream substrates, including protein AKT/PKB. AKT phosphorylation, in turn, enhances protein synthesis through mTOR activation and triggers the antiapoptotic effects of IGFIR through phosphorylation and inactivation of BAD. In parallel to PI3K-driven signaling, recruitment of Grb2/SOS by phosphorylated IRS1 or Shc leads to recruitment of Ras and activation of the ras-MAPK pathway. In addition to these two main signaling pathways IGF1R signals also through the Janus kinase/signal transducer and activator of transcription pathway (JAK/STAT). Phosphorylation of JAK proteins can lead to phosphorylation/activation of signal transducers and activators of transcription (STAT) proteins. In particular activation of STAT3, may be essential for the transforming activity of IGF1R. The JAK/STAT pathway activates gene transcription and may be responsible for the transforming activity. JNK kinases can also be activated by the IGF1R. IGF1 exerts inhibiting activities on JNK activation via phosphorylation and inhibition of MAP3K5/ASK1, which is able to directly associate with the IGF1R.   
  
Tetramer of 2 alpha and 2 beta chains linked by disulfide bonds. The alpha chains contribute to the formation of the ligand-binding domain, while the beta chain carries the kinase domain. Interacts with PIK3R1 and with the PTB/PID domains of IRS1 and SHC1 in vitro when autophosphorylated on tyrosine residues. Forms a hybrid receptor with INSR, the hybrid is a tetramer consisting of 1 alpha chain and 1 beta chain of INSR and 1 alpha chain and 1 beta chain of IGF1R. Interacts with ARRB1 and ARRB2. Interacts with GRB10. Interacts with RACK1. Interacts with SOCS1, SOCS2 and SOCS3. Interacts with 14-3-3 proteins. Interacts with NMD2. Interacts with MAP3K5. Interacts with STAT3. Found in a ternary complex with IGF1 and ITGAV:ITGB3 or ITGA6:ITGB4 (PubMed:19578119, PubMed:22351760). Interacts (nascent precursor form) with ZFAND2B (PubMed:26692333).   
  
 **Gene Ontology Information:**

Molecular Function

- ATP binding
- G-protein alpha-subunit binding
- identical protein binding
- insulin binding
- insulin-activated receptor activity
- insulin receptor binding
- insulin receptor substrate binding
- insulin-like growth factor binding
- insulin-like growth factor I binding
- insulin-like growth factor-activated receptor activity
- phosphatidylinositol 3-kinase binding
- protein transporter activity
- protein tyrosine kinase activity
- structural molecule activity
- transmembrane receptor protein tyrosine kinase activity

Location

- alphav-beta3 integrin-IGF-1-IGF1R complex
- axon
- caveola
- cytoplasm
- insulin receptor complex
- intracellular membrane-bounded organelle
- membrane
- neuronal cell body
- nucleus
- plasma membrane
- protein kinase complex
- receptor complex
- T-tubule

Biological process

- aging
- amyloid-beta clearance
- axonogenesis
- cardiac atrium development
- cellular response to aldosterone
- cellular response to amyloid-beta
- cellular response to angiotensin
- cellular response to dexamethasone stimulus
- cellular response to estradiol stimulus
- cellular response to glucose stimulus
- cellular response to insulin-like growth factor stimulus
- cellular response to mechanical stimulus
- cellular response to progesterone stimulus
- cellular response to testosterone stimulus
- cellular response to transforming growth factor beta stimulus
- cellular response to zinc ion starvation
- cellular senescence
- cerebellum development
- dendritic spine maintenance
- establishment of cell polarity
- estrous cycle
- glucose homeostasis
- hippocampus development
- immune response
- insulin receptor signaling pathway
- insulin-like growth factor receptor signaling pathway
- negative regulation of apoptotic process
- negative regulation of cholangiocyte apoptotic process
- negative regulation of hepatocyte apoptotic process
- negative regulation of MAPK cascade
- negative regulation of muscle cell apoptotic process
- peptidyl-tyrosine autophosphorylation
- phosphatidylinositol 3-kinase signaling
- phosphatidylinositol-mediated signaling
- positive regulation of axon regeneration
- positive regulation of cell migration
- positive regulation of cell population proliferation
- positive regulation of cold-induced thermogenesis
- positive regulation of cytokinesis
- positive regulation of DNA metabolic process
- positive regulation of kinase activity
- positive regulation of MAPK cascade
- positive regulation of osteoblast proliferation
- positive regulation of phosphatidylinositol 3-kinase signaling
- positive regulation of protein kinase B signaling
- positive regulation of protein-containing complex disassembly
- positive regulation of smooth muscle cell proliferation
- positive regulation of steroid hormone biosynthetic process
- protein autophosphorylation
- regulation of JNK cascade
- response to ethanol
- response to L-glutamate
- response to nicotine
- response to vitamin E
- signal transduction
- transcytosis
- transmembrane receptor protein tyrosine kinase signaling pathway

---

7

- **Protein name:** Endoribonuclease Dicer
- **Organism:** Homo sapiens
- **Uniprot Accession Number:** Q9UPY3
- **Protein sequence length:** 1922 aa
- **1D identity (%):** 15.64
- **1D identity (%) [Gaps excluded]:** 24.7
- **1D identity - Alignment Gaps:** 717
- **1D aligned content (<aminoacid>:%):** {'L': 12.42, 'P': 8.5, 'S': 8.17, 'T': 4.9, 'Y': 5.88, 'K': 7.19, 'H': 0.98, 'F': 4.9, 'I': 2.61, 'V': 4.9, 'N': 5.88, 'D': 7.19, 'G': 8.17, 'A': 3.92, 'C': 3.59, 'E': 4.25, 'Q': 2.29, 'R': 3.59, 'W': 0.65}
- **Common reported functions (%):** 0.0
- **Common reported locations (%):** 0.0
- **Common reported processes (%):** 0.0

- **PDB ID:** 5ZAM
- **Chain:** A
- **Crystallized protein length:** 1314 aa
- **Resolution:** 5.7 Å
- **b-phipsi:** 0.04952
- **w-rdist:** 0.432582
- **t-alpha:** 0.001654
- **Chemical similarity (Tanimoto Index) (%):** 86.07
- **1D identity (%) [PDB]:** 2.1
- **1D identity (%) [Gaps excluded][PDB]:** 69.12
- **1D identity - Alignment Gaps [PDB]:** 2169
- **1D aligned content [PDB] (<aminoacid>:%):** {'K': 6.38, 'Q': 4.26, 'S': 17.02, 'L': 23.4, 'T': 4.26, 'A': 4.26, 'G': 4.26, 'V': 4.26, 'N': 6.38, 'F': 2.13, 'I': 8.51, 'R': 4.26, 'D': 4.26, 'P': 4.26, 'E': 2.13}
- **2D identity (%) [PDB]:** 24.85
- **2D identity (%) [Gaps excluded][PDB]:** 86.58
- **2D identity - Alignment Gaps [PDB]:** 1277
- **2D aligned content [PDB] (<2D-fold>:%):** {'.': 23.82, 'H': 41.35, 'T': 11.46, 'S': 6.74, 'E': 15.51, 'G': 0.67, 'P': 0.45}
- **3D similarity (TM-Score) (%) [PDB]:** 26.41

- **Gene name:** DICER1
- **Entrez ID:** 23405
- **RefSeq ID:** N/A
- **Sequence length:** N/A
- **5-UTR|CDS|3-UTR identity (%):** N/A | N/A | N/A
- **5-UTR|CDS|3-UTR identity (%) [Gaps excluded]:** N/A | N/A | N/A
- **5-UTR|CDS|3-UTR identity [Alignment Gaps]:** N/A | N/A | N/A
- **5-UTR aligned content (<base>:%):** N/A
- **CDS aligned content (<base>:%):** N/A
- **3-UTR aligned content (<base>:%):** N/A

**Uniprot Description:**  
  
 Double-stranded RNA (dsRNA) endoribonuclease playing a central role in short dsRNA-mediated post-transcriptional gene silencing. Cleaves naturally occurring long dsRNAs and short hairpin pre-microRNAs (miRNA) into fragments of twenty-one to twenty-three nucleotides with 3' overhang of two nucleotides, producing respectively short interfering RNAs (siRNA) and mature microRNAs. SiRNAs and miRNAs serve as guide to direct the RNA-induced silencing complex (RISC) to complementary RNAs to degrade them or prevent their translation. Gene silencing mediated by siRNAs, also called RNA interference, controls the elimination of transcripts from mobile and repetitive DNA elements of the genome but also the degradation of exogenous RNA of viral origin for instance. The miRNA pathway on the other side is a mean to specifically regulate the expression of target genes.   
  
Component of the RISC loading complex (RLC), or micro-RNA (miRNA) loading complex (miRLC), which is composed of DICER1, AGO2 and TARBP2; DICER1 and TARBP2 are required to process precursor miRNAs (pre-miRNAs) to mature miRNAs and then load them onto AGO2. Note that the trimeric RLC/miRLC is also referred to as RISC. Interacts with DHX9, AGO1, PIWIL1 and PRKRA. Associates with the 60S ribosome. Interacts with BCDIN3D. Interacts with AGO2, TARBP2, EIF6, MOV10 and RPL7A (60S ribosome subunit); they form a large RNA-induced silencing complex (RISC) (PubMed:17507929). Interacts (via Dicer dsRNA-binding fold domain) with ALOX5 (via PLAT domain); this interaction enhances arachidonate 5-lipoxygenase activity and modifies the miRNA precursor processing activity of DICER1 (PubMed:19022417).   
  
 **Gene Ontology Information:**

Molecular Function

- ATP binding
- deoxyribonuclease I activity
- DNA binding
- double-stranded RNA binding
- endoribonuclease activity
- helicase activity
- metal ion binding
- pre-miRNA binding
- protein domain specific binding
- ribonuclease III activity
- RNA binding
- siRNA binding

Location

- cytoplasm
- cytosol
- extracellular exosome
- nucleus
- perinuclear region of cytoplasm
- RISC complex
- RISC-loading complex

Biological process

- apoptotic DNA fragmentation
- global gene silencing by mRNA cleavage
- miRNA metabolic process
- production of miRNAs involved in gene silencing by miRNA
- negative regulation of gene expression
- negative regulation of Schwann cell proliferation
- negative regulation of transcription by RNA polymerase II
- negative regulation of tumor necrosis factor production
- nerve development
- neuron projection morphogenesis
- NIK/NF-kappaB signaling
- peripheral nervous system myelin formation
- positive regulation of myelination
- positive regulation of Schwann cell differentiation
- pre-miRNA processing
- small RNA loading onto RISC
- RNA phosphodiester bond hydrolysis
- RNA phosphodiester bond hydrolysis, endonucleolytic
- production of siRNA involved in RNA interference
- tRNA catabolic process

---

8

- **Protein name:** N/A
- **Organism:** N/A
- **Uniprot Accession Number:** N/A
- **Protein sequence length:** N/A
- **1D identity (%):** N/A
- **1D identity (%) [Gaps excluded]:** N/A
- **1D identity - Alignment Gaps:** N/A
- **1D aligned content (<aminoacid>:%):** N/A
- **Common reported functions (%):** 0.0
- **Common reported locations (%):** 0.0
- **Common reported processes (%):** 0.0

- **PDB ID:** 7SBW
- **Chain:** A
- **Crystallized protein length:** 1185 aa
- **Resolution:** 3.2 Å
- **b-phipsi:** 0.000357
- **w-rdist:** 0.800046
- **t-alpha:** 0.008341
- **Chemical similarity (Tanimoto Index) (%):** 99.68
- **1D identity (%) [PDB]:** 7.94
- **1D identity (%) [Gaps excluded][PDB]:** 58.91
- **1D identity - Alignment Gaps [PDB]:** 1657
- **1D aligned content [PDB] (<aminoacid>:%):** {'V': 7.24, 'F': 5.92, 'A': 10.53, 'K': 5.92, 'Y': 3.29, 'T': 4.61, 'P': 7.89, 'G': 5.92, 'N': 5.92, 'D': 2.63, 'S': 7.89, 'R': 3.95, 'I': 5.26, 'E': 2.63, 'L': 12.5, 'W': 0.66, 'Q': 5.92, 'H': 1.32}
- **2D identity (%) [PDB]:** 59.02
- **2D identity (%) [Gaps excluded][PDB]:** 86.39
- **2D identity - Alignment Gaps [PDB]:** 409
- **2D aligned content [PDB] (<2D-fold>:%):** {'.': 19.29, 'E': 38.58, 'T': 9.19, 'B': 0.39, 'S': 6.69, 'P': 1.44, 'H': 22.57, 'G': 1.18, 'I': 0.66}
- **3D similarity (TM-Score) (%) [PDB]:** 6.97

- **Gene name:** N/A
- **Entrez ID:** N/A
- **RefSeq ID:** N/A
- **Sequence length:** N/A
- **5-UTR|CDS|3-UTR identity (%):** N/A | N/A | N/A
- **5-UTR|CDS|3-UTR identity (%) [Gaps excluded]:** N/A | N/A | N/A
- **5-UTR|CDS|3-UTR identity [Alignment Gaps]:** N/A | N/A | N/A
- **5-UTR aligned content (<base>:%):** N/A
- **CDS aligned content (<base>:%):** N/A
- **3-UTR aligned content (<base>:%):** N/A

**Uniprot Description:**  
  
 N/A N/A   
  
 **Gene Ontology Information:**

Molecular Function   
  
N/A

Location   
  
N/A

Biological process   
  
N/A

---

9

- **Protein name:** Spike glycoprotein
- **Organism:** Human coronavirus OC43
- **Uniprot Accession Number:** Q696P8
- **Protein sequence length:** 1353 aa
- **1D identity (%):** 29.39
- **1D identity (%) [Gaps excluded]:** 35.46
- **1D identity - Alignment Gaps:** 246
- **1D aligned content (<aminoacid>:%):** {'F': 6.4, 'L': 11.14, 'P': 5.21, 'S': 7.58, 'V': 6.16, 'T': 4.98, 'Y': 4.5, 'N': 6.87, 'G': 8.53, 'I': 4.27, 'K': 4.74, 'D': 4.98, 'Q': 3.55, 'C': 8.53, 'H': 0.71, 'A': 4.03, 'R': 3.55, 'E': 2.84, 'W': 1.42}
- **Common reported functions (%):** 0.0
- **Common reported locations (%):** 0.0
- **Common reported processes (%):** 0.0

- **PDB ID:** 7PNQ
- **Chain:** B
- **Crystallized protein length:** 1175 aa
- **Resolution:** 3.7 Å
- **b-phipsi:** 0.001283
- **w-rdist:** 0.734038
- **t-alpha:** 0.006617
- **Chemical similarity (Tanimoto Index) (%):** 100.0
- **1D identity (%) [PDB]:** 11.97
- **1D identity (%) [Gaps excluded][PDB]:** 56.91
- **1D identity - Alignment Gaps [PDB]:** 1412
- **1D aligned content [PDB] (<aminoacid>:%):** {'V': 8.41, 'S': 10.28, 'I': 7.48, 'T': 5.14, 'G': 7.48, 'N': 7.01, 'L': 11.21, 'Y': 3.74, 'D': 2.8, 'E': 3.74, 'F': 5.14, 'R': 3.27, 'A': 8.41, 'H': 2.34, 'P': 3.74, 'K': 3.74, 'M': 0.47, 'Q': 5.14, 'W': 0.47}
- **2D identity (%) [PDB]:** 59.06
- **2D identity (%) [Gaps excluded][PDB]:** 85.52
- **2D identity - Alignment Gaps [PDB]:** 396
- **2D aligned content [PDB] (<2D-fold>:%):** {'.': 19.31, 'E': 39.95, 'T': 8.2, 'B': 0.4, 'S': 7.01, 'P': 1.06, 'H': 22.88, 'G': 0.53, 'I': 0.66}
- **3D similarity (TM-Score) (%) [PDB]:** 7.28

- **Gene name:** S
- **Entrez ID:** N/A
- **RefSeq ID:** N/A
- **Sequence length:** N/A
- **5-UTR|CDS|3-UTR identity (%):** N/A | N/A | N/A
- **5-UTR|CDS|3-UTR identity (%) [Gaps excluded]:** N/A | N/A | N/A
- **5-UTR|CDS|3-UTR identity [Alignment Gaps]:** N/A | N/A | N/A
- **5-UTR aligned content (<base>:%):** N/A
- **CDS aligned content (<base>:%):** N/A
- **3-UTR aligned content (<base>:%):** N/A

**Uniprot Description:**  
  
 Spike protein S1: attaches the virion to the cell membrane by interacting with host receptor, initiating the infection.   
  
Homotrimer; each monomer consists of a S1 and a S2 subunit. The resulting peplomers protrude from the virus surface as spikes.   
  
 **Gene Ontology Information:**

Molecular Function   
  
N/A

Location   
  
N/A

Biological process   
  
N/A

---

10

- **Protein name:** Teneurin-2
- **Organism:** Homo sapiens
- **Uniprot Accession Number:** Q9NT68
- **Protein sequence length:** 2774 aa
- **1D identity (%):** 12.64
- **1D identity (%) [Gaps excluded]:** 28.92
- **1D identity - Alignment Gaps:** 1585
- **1D aligned content (<aminoacid>:%):** {'L': 8.15, 'T': 8.99, 'R': 3.09, 'Y': 5.9, 'S': 8.15, 'V': 6.18, 'H': 1.69, 'D': 6.74, 'F': 4.21, 'N': 7.87, 'A': 5.62, 'I': 5.34, 'G': 11.8, 'C': 2.53, 'P': 5.06, 'K': 3.93, 'Q': 1.69, 'W': 0.84, 'E': 2.25}
- **Common reported functions (%):** 0.0
- **Common reported locations (%):** 20.0
- **Common reported processes (%):** 0.0

- **PDB ID:** 6VHH
- **Chain:** A
- **Crystallized protein length:** 1723 aa
- **Resolution:** 2.97 Å
- **b-phipsi:** 0.003698
- **w-rdist:** 0.703188
- **t-alpha:** 0.006617
- **Chemical similarity (Tanimoto Index) (%):** 99.87
- **1D identity (%) [PDB]:** 3.58
- **1D identity (%) [Gaps excluded][PDB]:** 66.19
- **1D identity - Alignment Gaps [PDB]:** 2430
- **1D aligned content [PDB] (<aminoacid>:%):** {'V': 4.35, 'P': 3.26, 'L': 15.22, 'T': 5.43, 'E': 4.35, 'I': 6.52, 'A': 10.87, 'Y': 2.17, 'S': 10.87, 'G': 8.7, 'F': 2.17, 'M': 1.09, 'R': 3.26, 'N': 9.78, 'K': 4.35, 'Q': 5.43, 'D': 2.17}
- **2D identity (%) [PDB]:** 28.94
- **2D identity (%) [Gaps excluded][PDB]:** 87.52
- **2D identity - Alignment Gaps [PDB]:** 1362
- **2D aligned content [PDB] (<2D-fold>:%):** {'.': 22.58, 'E': 44.14, 'T': 9.68, 'B': 0.34, 'S': 8.49, 'G': 2.04, 'H': 12.73}
- **3D similarity (TM-Score) (%) [PDB]:** 24.69

- **Gene name:** TENM2
- **Entrez ID:** 57451
- **RefSeq ID:** N/A
- **Sequence length:** N/A
- **5-UTR|CDS|3-UTR identity (%):** N/A | N/A | N/A
- **5-UTR|CDS|3-UTR identity (%) [Gaps excluded]:** N/A | N/A | N/A
- **5-UTR|CDS|3-UTR identity [Alignment Gaps]:** N/A | N/A | N/A
- **5-UTR aligned content (<base>:%):** N/A
- **CDS aligned content (<base>:%):** N/A
- **3-UTR aligned content (<base>:%):** N/A

**Uniprot Description:**  
  
 Involved in neural development, regulating the establishment of proper connectivity within the nervous system. Promotes the formation of filopodia and enlarged growth cone in neuronal cells. Induces homophilic cell-cell adhesion (By similarity). May function as a cellular signal transducer.   
  
Homodimer; disulfide-linked (Probable). Heterodimer with either TENM1 or TENM3. May also form heterodimer with TENM4 (By similarity). Isoform 2 (C-terminal globular domain) interacts with ADGRL1 isoform 2.   
  
 **Gene Ontology Information:**

Molecular Function

- calcium ion binding
- cell adhesion molecule binding
- protein heterodimerization activity
- protein homodimerization activity
- signaling receptor binding

Location

- cell junction
- cell-cell junction
- dendrite
- dendritic spine
- endoplasmic reticulum
- filopodium
- Golgi apparatus
- growth cone
- neuron projection
- nucleus
- plasma membrane
- PML body
- postsynaptic membrane
- synapse

Biological process

- axon guidance
- calcium-mediated signaling using intracellular calcium source
- cell-cell adhesion
- heterophilic cell-cell adhesion via plasma membrane cell adhesion molecules
- negative regulation of transcription by RNA polymerase II
- neuron development
- positive regulation of filopodium assembly
- retrograde trans-synaptic signaling by trans-synaptic protein complex
- signal transduction

---

11

- **Protein name:** Cobra venom factor
- **Organism:** Naja kaouthia
- **Uniprot Accession Number:** Q91132
- **Protein sequence length:** 1642 aa
- **1D identity (%):** 16.07
- **1D identity (%) [Gaps excluded]:** 26.83
- **1D identity - Alignment Gaps:** 731
- **1D aligned content (<aminoacid>:%):** {'M': 0.34, 'L': 10.92, 'V': 7.17, 'T': 8.53, 'Q': 2.39, 'A': 8.19, 'S': 4.1, 'P': 5.12, 'K': 6.14, 'F': 7.51, 'G': 10.24, 'R': 3.07, 'N': 7.17, 'Y': 5.46, 'D': 4.44, 'E': 3.41, 'H': 1.02, 'I': 2.73, 'C': 2.05}
- **Common reported functions (%):** 0.0
- **Common reported locations (%):** 0.0
- **Common reported processes (%):** 0.0

- **PDB ID:** 3PRX
- **Chain:** D
- **Crystallized protein length:** 1215 aa
- **Resolution:** 4.3 Å
- **b-phipsi:** 0.007832
- **w-rdist:** 0.694785
- **t-alpha:** 0.002488
- **Chemical similarity (Tanimoto Index) (%):** 95.02
- **1D identity (%) [PDB]:** 4.29
- **1D identity (%) [Gaps excluded][PDB]:** 70.08
- **1D identity - Alignment Gaps [PDB]:** 1949
- **1D aligned content [PDB] (<aminoacid>:%):** {'A': 5.62, 'L': 7.87, 'S': 8.99, 'K': 7.87, 'T': 8.99, 'F': 7.87, 'E': 4.49, 'G': 4.49, 'I': 5.62, 'Y': 6.74, 'Q': 2.25, 'N': 8.99, 'R': 3.37, 'P': 5.62, 'V': 6.74, 'D': 4.49}
- **2D identity (%) [PDB]:** 32.8
- **2D identity (%) [Gaps excluded][PDB]:** 82.46
- **2D identity - Alignment Gaps [PDB]:** 949
- **2D aligned content [PDB] (<2D-fold>:%):** {'.': 25.92, 'E': 49.9, 'T': 9.67, 'S': 6.19, 'H': 7.74, 'G': 0.58}
- **3D similarity (TM-Score) (%) [PDB]:** 21.35

- **Gene name:** N/A
- **Entrez ID:** N/A
- **RefSeq ID:** N/A
- **Sequence length:** N/A
- **5-UTR|CDS|3-UTR identity (%):** N/A | N/A | N/A
- **5-UTR|CDS|3-UTR identity (%) [Gaps excluded]:** N/A | N/A | N/A
- **5-UTR|CDS|3-UTR identity [Alignment Gaps]:** N/A | N/A | N/A
- **5-UTR aligned content (<base>:%):** N/A
- **CDS aligned content (<base>:%):** N/A
- **3-UTR aligned content (<base>:%):** N/A

**Uniprot Description:**  
  
 Complement-activating protein in cobra venom. It is a structural and functional analog of complement component C3b, the activated form of C3. It binds factor B (CFB), which is subsequently cleaved by factor D (CFD) to form the bimolecular complex CVF/Bb. CVF/Bb is a C3/C5 convertase that cleaves both complement components C3 and C5. Structurally, it resembles the C3b degradation product C3c, which is not able to form a C3/C5 convertase. Unlike C3b/Bb, CVF/Bb is a stable complex and completely resistant to the actions of complement regulatory factors H (CFH) and I (CFI). Therefore, CVF continuously activates complement resulting in the depletion of complement activity.   
  
Heterotrimer of alpha, beta and gamma chains; disulfide-linked. Is active with factor B in the presence of factor D.   
  
 **Gene Ontology Information:**

Molecular Function

- endopeptidase inhibitor activity
- metal ion binding
- toxin activity

Location

- extracellular space

Biological process

- complement activation
- inflammatory response

---

12

- **Protein name:** Histone H3.3
- **Organism:** Homo sapiens
- **Uniprot Accession Number:** P84243
- **Protein sequence length:** 136 aa
- **1D identity (%):** 2.25
- **1D identity (%) [Gaps excluded]:** 24.58
- **1D identity - Alignment Gaps:** 1173
- **1D aligned content (<aminoacid>:%):** {'A': 24.14, 'T': 10.34, 'G': 13.79, 'V': 6.9, 'Y': 3.45, 'I': 6.9, 'S': 6.9, 'K': 3.45, 'L': 6.9, 'Q': 10.34, 'R': 6.9}
- **Common reported functions (%):** 0.0
- **Common reported locations (%):** 0.0
- **Common reported processes (%):** 0.0

- **PDB ID:** 6J4Z
- **Chain:** A
- **Crystallized protein length:** 1414 aa
- **Resolution:** 4.1 Å
- **b-phipsi:** 0.021727
- **w-rdist:** 0.592766
- **t-alpha:** 0.004963
- **Chemical similarity (Tanimoto Index) (%):** N/A
- **1D identity (%) [PDB]:** 4.48
- **1D identity (%) [Gaps excluded][PDB]:** 68.71
- **1D identity - Alignment Gaps [PDB]:** 2108
- **1D aligned content [PDB] (<aminoacid>:%):** {'Q': 12.87, 'M': 2.97, 'A': 11.88, 'R': 3.96, 'F': 4.95, 'N': 2.97, 'G': 8.91, 'T': 5.94, 'V': 5.94, 'L': 13.86, 'K': 3.96, 'I': 2.97, 'S': 10.89, 'D': 1.98, 'P': 1.98, 'E': 1.98, 'Y': 1.98}
- **2D identity (%) [PDB]:** 30.74
- **2D identity (%) [Gaps excluded][PDB]:** 87.22
- **2D identity - Alignment Gaps [PDB]:** 1150
- **2D aligned content [PDB] (<2D-fold>:%):** {'.': 18.86, 'S': 6.23, 'P': 0.18, 'E': 26.56, 'T': 12.45, 'H': 34.43, 'G': 1.28}
- **3D similarity (TM-Score) (%) [PDB]:** 24.34

- **Gene name:** H3-3A
- **Entrez ID:** 3020; 3021
- **RefSeq ID:** NM\_005324
- **Transcript sequence length:** 2705
- **5-UTR|CDS|3-UTR identity (%):** 22.55 | 6.33 | 9.52
- **5-UTR|CDS|3-UTR identity (%) [Gaps excluded]:** 75.0 | 70.49 | 90.75
- **5-UTR|CDS|3-UTR identity [Alignment Gaps]:** 214 | 3535 | 1936
- **5-UTR aligned content (<base>:%):** {'G': 34.78, 'A': 14.49, 'C': 23.19, 'T': 27.54}
- **CDS aligned content (<base>:%):** {'A': 27.64, 'T': 21.95, 'G': 24.8, 'C': 25.61}
- **3-UTR aligned content (<base>:%):** {'C': 15.53, 'A': 41.26, 'T': 23.3, 'G': 19.9}

**Uniprot Description:**  
  
 Variant histone H3 which replaces conventional H3 in a wide range of nucleosomes in active genes. Constitutes the predominant form of histone H3 in non-dividing cells and is incorporated into chromatin independently of DNA synthesis. Deposited at sites of nucleosomal displacement throughout transcribed genes, suggesting that it represents an epigenetic imprint of transcriptionally active chromatin. Nucleosomes wrap and compact DNA into chromatin, limiting DNA accessibility to the cellular machineries which require DNA as a template. Histones thereby play a central role in transcription regulation, DNA repair, DNA replication and chromosomal stability. DNA accessibility is regulated via a complex set of post-translational modifications of histones, also called histone code, and nucleosome remodeling.   
  
The nucleosome is a histone octamer containing two molecules each of H2A, H2B, H3 and H4 assembled in one H3-H4 heterotetramer and two H2A-H2B heterodimers. The octamer wraps approximately 147 bp of DNA. Interacts with HIRA, a chaperone required for its incorporation into nucleosomes. Interacts with ZMYND11; when trimethylated at 'Lys-36' (H3.3K36me3).   
  
 **Gene Ontology Information:**

Molecular Function

- nucleosomal DNA binding
- protein heterodimerization activity
- RNA polymerase II cis-regulatory region sequence-specific DNA binding
- RNA polymerase II core promoter sequence-specific DNA binding
- structural constituent of chromatin

Location

- chromosome, telomeric region
- extracellular exosome
- extracellular region
- nucleoplasm
- nucleosome
- nucleus
- protein-containing complex

Biological process

- nucleosome assembly
- positive regulation of cell growth
- telomere organization

---

13

- **Protein name:** Spike glycoprotein
- **Organism:** Human coronavirus OC43
- **Uniprot Accession Number:** A0A0B4N7I3
- **Protein sequence length:** 1362 aa
- **1D identity (%):** 29.76
- **1D identity (%) [Gaps excluded]:** 36.13
- **1D identity - Alignment Gaps:** 255
- **1D aligned content (<aminoacid>:%):** {'F': 6.28, 'L': 11.86, 'P': 5.58, 'S': 7.67, 'V': 5.81, 'T': 5.12, 'Y': 4.42, 'N': 6.28, 'G': 8.84, 'R': 3.72, 'I': 4.42, 'K': 4.65, 'D': 4.88, 'Q': 3.49, 'C': 8.14, 'H': 0.7, 'A': 3.72, 'E': 2.79, 'W': 1.4, 'M': 0.23}
- **Common reported functions (%):** 0.0
- **Common reported locations (%):** 0.0
- **Common reported processes (%):** 0.0

- **PDB ID:** 7SB4
- **Chain:** A
- **Crystallized protein length:** 1176 aa
- **Resolution:** 4.7 Å
- **b-phipsi:** 0.001081
- **w-rdist:** 0.782989
- **t-alpha:** 0.0075
- **Chemical similarity (Tanimoto Index) (%):** 99.68
- **1D identity (%) [PDB]:** 8.15
- **1D identity (%) [Gaps excluded][PDB]:** 59.16
- **1D identity - Alignment Gaps [PDB]:** 1640
- **1D aligned content [PDB] (<aminoacid>:%):** {'V': 7.1, 'F': 6.45, 'A': 10.32, 'K': 5.81, 'Y': 3.23, 'T': 4.52, 'P': 6.45, 'I': 6.45, 'D': 3.23, 'N': 5.81, 'S': 8.39, 'L': 12.9, 'R': 3.87, 'E': 2.58, 'G': 5.16, 'W': 0.65, 'Q': 5.81, 'H': 1.29}
- **2D identity (%) [PDB]:** 57.86
- **2D identity (%) [Gaps excluded][PDB]:** 83.62
- **2D identity - Alignment Gaps [PDB]:** 394
- **2D aligned content [PDB] (<2D-fold>:%):** {'.': 19.46, 'S': 6.35, 'E': 38.24, 'P': 1.22, 'T': 8.78, 'H': 23.78, 'B': 0.27, 'G': 1.22, 'I': 0.68}
- **3D similarity (TM-Score) (%) [PDB]:** 88.88

- **Gene name:** S
- **Entrez ID:** N/A
- **RefSeq ID:** N/A
- **Sequence length:** N/A
- **5-UTR|CDS|3-UTR identity (%):** N/A | N/A | N/A
- **5-UTR|CDS|3-UTR identity (%) [Gaps excluded]:** N/A | N/A | N/A
- **5-UTR|CDS|3-UTR identity [Alignment Gaps]:** N/A | N/A | N/A
- **5-UTR aligned content (<base>:%):** N/A
- **CDS aligned content (<base>:%):** N/A
- **3-UTR aligned content (<base>:%):** N/A

**Uniprot Description:**  
  
 Spike protein S1: attaches the virion to the cell membrane by interacting with host receptor, initiating the infection.   
  
Homotrimer; each monomer consists of a S1 and a S2 subunit. The resulting peplomers protrude from the virus surface as spikes.   
  
 **Gene Ontology Information:**

Molecular Function   
  
N/A

Location   
  
N/A

Biological process   
  
N/A

---

14

- **Protein name:** N/A
- **Organism:** N/A
- **Uniprot Accession Number:** N/A
- **Protein sequence length:** N/A
- **1D identity (%):** N/A
- **1D identity (%) [Gaps excluded]:** N/A
- **1D identity - Alignment Gaps:** N/A
- **1D aligned content (<aminoacid>:%):** N/A
- **Common reported functions (%):** 0.0
- **Common reported locations (%):** 0.0
- **Common reported processes (%):** 0.0

- **PDB ID:** 6NB3
- **Chain:** B
- **Crystallized protein length:** 958 aa
- **Resolution:** 3.5 Å
- **b-phipsi:** 0.001176
- **w-rdist:** 0.454125
- **t-alpha:** 0.07754
- **Chemical similarity (Tanimoto Index) (%):** 94.45
- **1D identity (%) [PDB]:** 13.09
- **1D identity (%) [Gaps excluded][PDB]:** 58.26
- **1D identity - Alignment Gaps [PDB]:** 1232
- **1D aligned content [PDB] (<aminoacid>:%):** {'T': 5.29, 'S': 6.73, 'N': 6.73, 'V': 6.73, 'A': 11.54, 'Q': 6.25, 'D': 4.33, 'G': 9.13, 'I': 7.69, 'H': 1.44, 'Y': 2.88, 'E': 2.4, 'M': 0.96, 'L': 10.1, 'P': 4.33, 'F': 5.77, 'K': 3.85, 'R': 3.37, 'W': 0.48}
- **2D identity (%) [PDB]:** 57.33
- **2D identity (%) [Gaps excluded][PDB]:** 85.88
- **2D identity - Alignment Gaps [PDB]:** 388
- **2D aligned content [PDB] (<2D-fold>:%):** {'.': 16.59, 'E': 40.96, 'T': 9.87, 'B': 0.6, 'S': 5.38, 'P': 1.35, 'H': 23.92, 'G': 0.6, 'I': 0.75}
- **3D similarity (TM-Score) (%) [PDB]:** 7.36

- **Gene name:** N/A
- **Entrez ID:** N/A
- **RefSeq ID:** N/A
- **Sequence length:** N/A
- **5-UTR|CDS|3-UTR identity (%):** N/A | N/A | N/A
- **5-UTR|CDS|3-UTR identity (%) [Gaps excluded]:** N/A | N/A | N/A
- **5-UTR|CDS|3-UTR identity [Alignment Gaps]:** N/A | N/A | N/A
- **5-UTR aligned content (<base>:%):** N/A
- **CDS aligned content (<base>:%):** N/A
- **3-UTR aligned content (<base>:%):** N/A

**Uniprot Description:**  
  
 N/A N/A   
  
 **Gene Ontology Information:**

Molecular Function   
  
N/A

Location   
  
N/A

Biological process   
  
N/A

---

15

- **Protein name:** N/A
- **Organism:** N/A
- **Uniprot Accession Number:** N/A
- **Protein sequence length:** N/A
- **1D identity (%):** N/A
- **1D identity (%) [Gaps excluded]:** N/A
- **1D identity - Alignment Gaps:** N/A
- **1D aligned content (<aminoacid>:%):** N/A
- **Common reported functions (%):** 0.0
- **Common reported locations (%):** 0.0
- **Common reported processes (%):** 0.0

- **PDB ID:** 7SBX
- **Chain:** J
- **Crystallized protein length:** 1176 aa
- **Resolution:** 3.0 Å
- **b-phipsi:** 0.001044
- **w-rdist:** 0.784647
- **t-alpha:** 0.010025
- **Chemical similarity (Tanimoto Index) (%):** 99.68
- **1D identity (%) [PDB]:** 8.15
- **1D identity (%) [Gaps excluded][PDB]:** 59.16
- **1D identity - Alignment Gaps [PDB]:** 1641
- **1D aligned content [PDB] (<aminoacid>:%):** {'V': 7.1, 'F': 6.45, 'A': 10.32, 'K': 5.81, 'Y': 3.23, 'T': 4.52, 'P': 6.45, 'I': 6.45, 'D': 3.23, 'N': 5.81, 'S': 8.39, 'L': 12.9, 'R': 3.87, 'E': 2.58, 'G': 5.16, 'W': 0.65, 'Q': 5.81, 'H': 1.29}
- **2D identity (%) [PDB]:** 59.73
- **2D identity (%) [Gaps excluded][PDB]:** 84.6
- **2D identity - Alignment Gaps [PDB]:** 373
- **2D aligned content [PDB] (<2D-fold>:%):** {'.': 18.73, 'E': 39.31, 'B': 0.4, 'S': 6.2, 'P': 1.58, 'T': 9.5, 'H': 22.56, 'G': 1.72}
- **3D similarity (TM-Score) (%) [PDB]:** N/A

- **Gene name:** N/A
- **Entrez ID:** N/A
- **RefSeq ID:** N/A
- **Sequence length:** N/A
- **5-UTR|CDS|3-UTR identity (%):** N/A | N/A | N/A
- **5-UTR|CDS|3-UTR identity (%) [Gaps excluded]:** N/A | N/A | N/A
- **5-UTR|CDS|3-UTR identity [Alignment Gaps]:** N/A | N/A | N/A
- **5-UTR aligned content (<base>:%):** N/A
- **CDS aligned content (<base>:%):** N/A
- **3-UTR aligned content (<base>:%):** N/A

**Uniprot Description:**  
  
 N/A N/A   
  
 **Gene Ontology Information:**

Molecular Function   
  
N/A

Location   
  
N/A

Biological process   
  
N/A

---

16

- **Protein name:** Teneurin-4
- **Organism:** Homo sapiens
- **Uniprot Accession Number:** Q6N022
- **Protein sequence length:** 2769 aa
- **1D identity (%):** 12.31
- **1D identity (%) [Gaps excluded]:** 27.59
- **1D identity - Alignment Gaps:** 1548
- **1D aligned content (<aminoacid>:%):** {'M': 0.29, 'F': 4.36, 'L': 11.05, 'P': 7.85, 'S': 7.27, 'C': 2.91, 'N': 7.27, 'T': 8.43, 'R': 3.49, 'A': 4.07, 'G': 11.63, 'D': 6.98, 'H': 1.74, 'V': 5.23, 'Y': 4.65, 'W': 1.16, 'K': 2.91, 'I': 4.07, 'E': 2.62, 'Q': 2.03}
- **Common reported functions (%):** 0.0
- **Common reported locations (%):** 20.0
- **Common reported processes (%):** 0.0

- **PDB ID:** 7BAN
- **Chain:** A
- **Crystallized protein length:** 1904 aa
- **Resolution:** 2.7 Å
- **b-phipsi:** 0.004115
- **w-rdist:** 0.407259
- **t-alpha:** 0.052109
- **Chemical similarity (Tanimoto Index) (%):** 86.7
- **1D identity (%) [PDB]:** 3.18
- **1D identity (%) [Gaps excluded][PDB]:** 68.75
- **1D identity - Alignment Gaps [PDB]:** 2635
- **1D aligned content [PDB] (<aminoacid>:%):** {'A': 11.36, 'G': 4.55, 'L': 14.77, 'Q': 7.95, 'I': 6.82, 'P': 3.41, 'F': 3.41, 'R': 4.55, 'N': 6.82, 'V': 7.95, 'E': 3.41, 'K': 2.27, 'S': 12.5, 'D': 4.55, 'T': 5.68}
- **2D identity (%) [PDB]:** 29.16
- **2D identity (%) [Gaps excluded][PDB]:** 87.29
- **2D identity - Alignment Gaps [PDB]:** 1443
- **2D aligned content [PDB] (<2D-fold>:%):** {'.': 22.47, 'T': 12.5, 'B': 0.32, 'S': 8.54, 'E': 42.56, 'P': 0.32, 'G': 0.95, 'H': 12.34}
- **3D similarity (TM-Score) (%) [PDB]:** 26.42

- **Gene name:** TENM4
- **Entrez ID:** 26011
- **RefSeq ID:** N/A
- **Sequence length:** N/A
- **5-UTR|CDS|3-UTR identity (%):** N/A | N/A | N/A
- **5-UTR|CDS|3-UTR identity (%) [Gaps excluded]:** N/A | N/A | N/A
- **5-UTR|CDS|3-UTR identity [Alignment Gaps]:** N/A | N/A | N/A
- **5-UTR aligned content (<base>:%):** N/A
- **CDS aligned content (<base>:%):** N/A
- **3-UTR aligned content (<base>:%):** N/A

**Uniprot Description:**  
  
 Involved in neural development, regulating the establishment of proper connectivity within the nervous system. Plays a role in the establishment of the anterior-posterior axis during gastrulation. Regulates the differentiation and cellular process formation of oligodendrocytes and myelination of small-diameter axons in the central nervous system (CNS) (PubMed:26188006). Promotes activation of focal adhesion kinase. May function as a cellular signal transducer (By similarity).   
  
Homodimer; disulfide-linked (Probable). May also form heterodimer with either TENM1 or TENM2 or TENM3 (By similarity).   
  
 **Gene Ontology Information:**

Molecular Function

- cell adhesion molecule binding
- protein heterodimerization activity
- protein homodimerization activity

Location

- cytoplasm
- neuron projection
- nucleus
- plasma membrane

Biological process

- cardiac cell fate specification
- cardiac muscle cell proliferation
- central nervous system myelin formation
- gastrulation with mouth forming second
- heterophilic cell-cell adhesion via plasma membrane cell adhesion molecules
- neuron development
- positive regulation of gastrulation
- positive regulation of myelination
- positive regulation of oligodendrocyte differentiation
- regulation of myelination
- signal transduction

---

17

- **Protein name:** Anaphase-promoting complex subunit 1
- **Organism:** Homo sapiens
- **Uniprot Accession Number:** Q9H1A4
- **Protein sequence length:** 1944 aa
- **1D identity (%):** 15.98
- **1D identity (%) [Gaps excluded]:** 26.57
- **1D identity - Alignment Gaps:** 801
- **1D aligned content (<aminoacid>:%):** {'M': 0.93, 'F': 5.92, 'P': 8.41, 'C': 3.74, 'N': 5.61, 'L': 13.71, 'R': 3.43, 'Q': 3.12, 'A': 4.67, 'G': 9.35, 'S': 6.85, 'W': 0.93, 'V': 6.85, 'D': 5.3, 'K': 1.87, 'T': 6.23, 'I': 4.05, 'Y': 3.74, 'H': 1.56, 'E': 3.74}
- **Common reported functions (%):** 0.0
- **Common reported locations (%):** 0.0
- **Common reported processes (%):** 0.0

- **PDB ID:** 5LCW
- **Chain:** A
- **Crystallized protein length:** 1441 aa
- **Resolution:** 4.0 Å
- **b-phipsi:** 0.028634
- **w-rdist:** 0.619121
- **t-alpha:** 0.003309
- **Chemical similarity (Tanimoto Index) (%):** N/A
- **1D identity (%) [PDB]:** 4.01
- **1D identity (%) [Gaps excluded][PDB]:** 74.4
- **1D identity - Alignment Gaps [PDB]:** 2195
- **1D aligned content [PDB] (<aminoacid>:%):** {'I': 4.3, 'A': 12.9, 'N': 4.3, 'S': 13.98, 'G': 3.23, 'K': 2.15, 'Q': 8.6, 'D': 4.3, 'L': 16.13, 'V': 9.68, 'T': 4.3, 'R': 3.23, 'P': 3.23, 'E': 3.23, 'M': 1.08, 'F': 2.15, 'H': 2.15, 'Y': 1.08}
- **2D identity (%) [PDB]:** 31.15
- **2D identity (%) [Gaps excluded][PDB]:** 84.78
- **2D identity - Alignment Gaps [PDB]:** 1131
- **2D aligned content [PDB] (<2D-fold>:%):** {'E': 32.68, '.': 19.21, 'S': 7.54, 'T': 9.16, 'P': 0.72, 'H': 30.7}
- **3D similarity (TM-Score) (%) [PDB]:** 23.54

- **Gene name:** ANAPC1
- **Entrez ID:** 64682
- **RefSeq ID:** NM\_022662
- **Transcript sequence length:** 7762
- **5-UTR|CDS|3-UTR identity (%):** 41.12 | 42.04 | 12.15
- **5-UTR|CDS|3-UTR identity (%) [Gaps excluded]:** 68.47 | 78.0 | 88.94
- **5-UTR|CDS|3-UTR identity [Alignment Gaps]:** 135 | 2893 | 1428
- **5-UTR aligned content (<base>:%):** {'C': 32.37, 'A': 23.02, 'G': 22.3, 'T': 22.3}
- **CDS aligned content (<base>:%):** {'A': 29.68, 'T': 33.62, 'G': 18.04, 'C': 18.65}
- **3-UTR aligned content (<base>:%):** {'C': 14.93, 'A': 41.79, 'T': 23.38, 'G': 19.9}

**Uniprot Description:**  
  
 Component of the anaphase promoting complex/cyclosome (APC/C), a cell cycle-regulated E3 ubiquitin ligase that controls progression through mitosis and the G1 phase of the cell cycle. The APC/C complex acts by mediating ubiquitination and subsequent degradation of target proteins: it mainly mediates the formation of 'Lys-11'-linked polyubiquitin chains and, to a lower extent, the formation of 'Lys-48'- and 'Lys-63'-linked polyubiquitin chains.   
  
The mammalian APC/C is composed at least of 14 distinct subunits ANAPC1, ANAPC2, CDC27/APC3, ANAPC4, ANAPC5, CDC16/APC6, ANAPC7, CDC23/APC8, ANAPC10, ANAPC11, CDC26/APC12, ANAPC13, ANAPC15 and ANAPC16 that assemble into a complex of at least 19 chains with a combined molecular mass of around 1.2 MDa; APC/C interacts with FZR1 and FBXO5.   
  
 **Gene Ontology Information:**

Molecular Function

- molecular adaptor activity

Location

- anaphase-promoting complex
- cytosol
- nucleoplasm

Biological process

- anaphase-promoting complex-dependent catabolic process
- cell division
- metaphase/anaphase transition of mitotic cell cycle
- protein K11-linked ubiquitination
- regulation of meiotic cell cycle
- regulation of mitotic cell cycle

---

18

- **Protein name:** N/A
- **Organism:** N/A
- **Uniprot Accession Number:** N/A
- **Protein sequence length:** N/A
- **1D identity (%):** N/A
- **1D identity (%) [Gaps excluded]:** N/A
- **1D identity - Alignment Gaps:** N/A
- **1D aligned content (<aminoacid>:%):** N/A
- **Common reported functions (%):** 0.0
- **Common reported locations (%):** 0.0
- **Common reported processes (%):** 0.0

- **PDB ID:** 6NB3
- **Chain:** C
- **Crystallized protein length:** 1169 aa
- **Resolution:** 3.5 Å
- **b-phipsi:** 0.00083
- **w-rdist:** 0.638474
- **t-alpha:** 0.173697
- **Chemical similarity (Tanimoto Index) (%):** 94.45
- **1D identity (%) [PDB]:** 11.74
- **1D identity (%) [Gaps excluded][PDB]:** 57.07
- **1D identity - Alignment Gaps [PDB]:** 1420
- **1D aligned content [PDB] (<aminoacid>:%):** {'T': 5.71, 'S': 7.62, 'N': 7.14, 'V': 6.19, 'A': 10.95, 'Q': 5.71, 'D': 3.81, 'G': 9.05, 'R': 3.81, 'I': 7.62, 'Y': 3.33, 'E': 2.38, 'H': 1.43, 'M': 0.95, 'L': 10.0, 'P': 4.29, 'F': 5.71, 'K': 3.81, 'W': 0.48}
- **2D identity (%) [PDB]:** 58.56
- **2D identity (%) [Gaps excluded][PDB]:** 84.58
- **2D identity - Alignment Gaps [PDB]:** 392
- **2D aligned content [PDB] (<2D-fold>:%):** {'.': 19.17, 'B': 0.4, 'S': 6.97, 'E': 37.4, 'P': 1.88, 'T': 9.25, 'H': 22.65, 'G': 1.61, 'I': 0.67}
- **3D similarity (TM-Score) (%) [PDB]:** 7.79

- **Gene name:** N/A
- **Entrez ID:** N/A
- **RefSeq ID:** N/A
- **Sequence length:** N/A
- **5-UTR|CDS|3-UTR identity (%):** N/A | N/A | N/A
- **5-UTR|CDS|3-UTR identity (%) [Gaps excluded]:** N/A | N/A | N/A
- **5-UTR|CDS|3-UTR identity [Alignment Gaps]:** N/A | N/A | N/A
- **5-UTR aligned content (<base>:%):** N/A
- **CDS aligned content (<base>:%):** N/A
- **3-UTR aligned content (<base>:%):** N/A

**Uniprot Description:**  
  
 N/A N/A   
  
 **Gene Ontology Information:**

Molecular Function   
  
N/A

Location   
  
N/A

Biological process   
  
N/A

---

19

- **Protein name:** Histone H4
- **Organism:** Homo sapiens
- **Uniprot Accession Number:** P62805
- **Protein sequence length:** 103 aa
- **1D identity (%):** 1.86
- **1D identity (%) [Gaps excluded]:** 27.59
- **1D identity - Alignment Gaps:** 1202
- **1D aligned content (<aminoacid>:%):** {'G': 25.0, 'A': 12.5, 'P': 4.17, 'R': 4.17, 'Y': 4.17, 'E': 4.17, 'F': 8.33, 'I': 4.17, 'D': 8.33, 'T': 8.33, 'K': 4.17, 'V': 8.33, 'L': 4.17}
- **Common reported functions (%):** 0.0
- **Common reported locations (%):** 0.0
- **Common reported processes (%):** 0.0

- **PDB ID:** 6J4W
- **Chain:** B
- **Crystallized protein length:** 1157 aa
- **Resolution:** 7.9 Å
- **b-phipsi:** 0.004411
- **w-rdist:** 0.931124
- **t-alpha:** 0.001654
- **Chemical similarity (Tanimoto Index) (%):** N/A
- **1D identity (%) [PDB]:** 3.36
- **1D identity (%) [Gaps excluded][PDB]:** 73.4
- **1D identity - Alignment Gaps [PDB]:** 1957
- **1D aligned content [PDB] (<aminoacid>:%):** {'S': 14.49, 'A': 14.49, 'I': 8.7, 'G': 4.35, 'K': 4.35, 'Q': 7.25, 'D': 4.35, 'L': 15.94, 'T': 8.7, 'N': 5.8, 'V': 2.9, 'F': 1.45, 'P': 1.45, 'R': 4.35, 'E': 1.45}
- **2D identity (%) [PDB]:** 24.43
- **2D identity (%) [Gaps excluded][PDB]:** 93.05
- **2D identity - Alignment Gaps [PDB]:** 1253
- **2D aligned content [PDB] (<2D-fold>:%):** {'.': 14.46, 'E': 31.08, 'T': 9.4, 'S': 4.58, 'P': 0.24, 'H': 39.04, 'G': 0.72, 'B': 0.48}
- **3D similarity (TM-Score) (%) [PDB]:** 23.39

- **Gene name:** H4C1
- **Entrez ID:** 121504; 554313; 8294; 8359; 8360; 8361; 8362; 8363; 8364; 8365; 8366; 8367; 8368; 8370
- **RefSeq ID:** NM\_175054
- **Transcript sequence length:** 412
- **5-UTR|CDS|3-UTR identity (%):** 12.18 | 4.77 | 10.48
- **5-UTR|CDS|3-UTR identity (%) [Gaps excluded]:** 80.49 | 71.98 | 76.47
- **5-UTR|CDS|3-UTR identity [Alignment Gaps]:** 230 | 3620 | 214
- **5-UTR aligned content (<base>:%):** {'A': 18.18, 'C': 24.24, 'G': 33.33, 'T': 24.24}
- **CDS aligned content (<base>:%):** {'A': 26.49, 'G': 28.65, 'T': 22.16, 'C': 22.7}
- **3-UTR aligned content (<base>:%):** {'G': 19.23, 'C': 23.08, 'T': 30.77, 'A': 26.92}

**Uniprot Description:**  
  
 Core component of nucleosome. Nucleosomes wrap and compact DNA into chromatin, limiting DNA accessibility to the cellular machineries which require DNA as a template. Histones thereby play a central role in transcription regulation, DNA repair, DNA replication and chromosomal stability. DNA accessibility is regulated via a complex set of post-translational modifications of histones, also called histone code, and nucleosome remodeling.   
  
The nucleosome is a histone octamer containing two molecules each of H2A, H2B, H3 and H4 assembled in one H3-H4 heterotetramer and two H2A-H2B heterodimers. The octamer wraps approximately 147 bp of DNA.   
  
 **Gene Ontology Information:**

Molecular Function

- DNA binding
- protein domain specific binding
- protein heterodimerization activity
- RNA binding
- structural constituent of chromatin

Location

- CENP-A containing nucleosome
- chromosome, telomeric region
- extracellular exosome
- extracellular region
- membrane
- nucleoplasm
- nucleosome
- nucleus
- protein-containing complex

Biological process

- negative regulation of megakaryocyte differentiation
- nucleosome assembly
- protein localization to CENP-A containing chromatin
- telomere organization

---

20

- **Protein name:** N/A
- **Organism:** N/A
- **Uniprot Accession Number:** N/A
- **Protein sequence length:** N/A
- **1D identity (%):** N/A
- **1D identity (%) [Gaps excluded]:** N/A
- **1D identity - Alignment Gaps:** N/A
- **1D aligned content (<aminoacid>:%):** N/A
- **Common reported functions (%):** 0.0
- **Common reported locations (%):** 0.0
- **Common reported processes (%):** 0.0

- **PDB ID:** 7SB3
- **Chain:** J
- **Crystallized protein length:** 1183 aa
- **Resolution:** 3.3 Å
- **b-phipsi:** 0.001356
- **w-rdist:** 0.813745
- **t-alpha:** 0.005824
- **Chemical similarity (Tanimoto Index) (%):** 99.68
- **1D identity (%) [PDB]:** 8.12
- **1D identity (%) [Gaps excluded][PDB]:** 59.16
- **1D identity - Alignment Gaps [PDB]:** 1647
- **1D aligned content [PDB] (<aminoacid>:%):** {'V': 7.1, 'F': 6.45, 'A': 10.32, 'K': 5.81, 'Y': 3.23, 'T': 4.52, 'P': 6.45, 'I': 6.45, 'D': 3.23, 'N': 5.81, 'S': 8.39, 'L': 12.9, 'R': 3.87, 'E': 2.58, 'G': 5.16, 'W': 0.65, 'Q': 5.81, 'H': 1.29}
- **2D identity (%) [PDB]:** 59.15
- **2D identity (%) [Gaps excluded][PDB]:** 83.06
- **2D identity - Alignment Gaps [PDB]:** 365
- **2D aligned content [PDB] (<2D-fold>:%):** {'.': 20.13, 'S': 7.07, 'E': 38.4, 'P': 1.2, 'T': 8.53, 'B': 0.27, 'H': 22.53, 'G': 1.2, 'I': 0.67}
- **3D similarity (TM-Score) (%) [PDB]:** N/A

- **Gene name:** N/A
- **Entrez ID:** N/A
- **RefSeq ID:** N/A
- **Sequence length:** N/A
- **5-UTR|CDS|3-UTR identity (%):** N/A | N/A | N/A
- **5-UTR|CDS|3-UTR identity (%) [Gaps excluded]:** N/A | N/A | N/A
- **5-UTR|CDS|3-UTR identity [Alignment Gaps]:** N/A | N/A | N/A
- **5-UTR aligned content (<base>:%):** N/A
- **CDS aligned content (<base>:%):** N/A
- **3-UTR aligned content (<base>:%):** N/A

**Uniprot Description:**  
  
 N/A N/A   
  
 **Gene Ontology Information:**

Molecular Function   
  
N/A

Location   
  
N/A

Biological process   
  
N/A

---

21

- **Protein name:** DNA-directed RNA polymerase II subunit RPB1
- **Organism:** Homo sapiens
- **Uniprot Accession Number:** P24928
- **Protein sequence length:** 1970 aa
- **1D identity (%):** 15.32
- **1D identity (%) [Gaps excluded]:** 25.64
- **1D identity - Alignment Gaps:** 817
- **1D aligned content (<aminoacid>:%):** {'P': 7.72, 'S': 8.36, 'C': 0.96, 'R': 4.18, 'Q': 2.57, 'G': 9.97, 'Y': 4.82, 'V': 7.72, 'T': 7.4, 'L': 8.36, 'F': 4.5, 'H': 0.96, 'N': 7.4, 'D': 6.43, 'A': 6.75, 'E': 3.86, 'K': 4.18, 'W': 0.64, 'M': 1.29, 'I': 1.93}
- **Common reported functions (%):** 0.0
- **Common reported locations (%):** 0.0
- **Common reported processes (%):** 0.0

- **PDB ID:** 5IYC
- **Chain:** A
- **Crystallized protein length:** 1454 aa
- **Resolution:** 3.9 Å
- **b-phipsi:** 0.028341
- **w-rdist:** 0.582144
- **t-alpha:** 0.008271
- **Chemical similarity (Tanimoto Index) (%):** N/A
- **1D identity (%) [PDB]:** 3.32
- **1D identity (%) [Gaps excluded][PDB]:** 66.38
- **1D identity - Alignment Gaps [PDB]:** 2206
- **1D aligned content [PDB] (<aminoacid>:%):** {'S': 6.49, 'A': 10.39, 'L': 15.58, 'G': 7.79, 'D': 3.9, 'V': 6.49, 'Q': 9.09, 'N': 6.49, 'T': 3.9, 'K': 5.19, 'F': 3.9, 'I': 3.9, 'P': 2.6, 'E': 5.19, 'R': 5.19, 'M': 2.6, 'H': 1.3}
- **2D identity (%) [PDB]:** 32.03
- **2D identity (%) [Gaps excluded][PDB]:** 83.31
- **2D identity - Alignment Gaps [PDB]:** 1084
- **2D aligned content [PDB] (<2D-fold>:%):** {'.': 22.34, 'E': 24.65, 'S': 8.16, 'T': 9.22, 'B': 0.35, 'H': 32.98, 'P': 0.71, 'G': 0.71, 'I': 0.89}
- **3D similarity (TM-Score) (%) [PDB]:** 25.7

- **Gene name:** POLR2A
- **Entrez ID:** 5430
- **RefSeq ID:** NM\_000937
- **Transcript sequence length:** 6749
- **5-UTR|CDS|3-UTR identity (%):** 34.6 | 39.3 | 33.04
- **5-UTR|CDS|3-UTR identity (%) [Gaps excluded]:** 71.76 | 73.95 | 73.79
- **5-UTR|CDS|3-UTR identity [Alignment Gaps]:** 232 | 2979 | 254
- **5-UTR aligned content (<base>:%):** {'G': 19.35, 'T': 29.03, 'A': 18.71, 'C': 32.9}
- **CDS aligned content (<base>:%):** {'A': 29.46, 'T': 28.66, 'G': 19.5, 'C': 22.38}
- **3-UTR aligned content (<base>:%):** {'C': 17.76, 'A': 34.87, 'G': 25.0, 'T': 22.37}

**Uniprot Description:**  
  
 DNA-dependent RNA polymerase catalyzes the transcription of DNA into RNA using the four ribonucleoside triphosphates as substrates. Largest and catalytic component of RNA polymerase II which synthesizes mRNA precursors and many functional non-coding RNAs. Forms the polymerase active center together with the second largest subunit. Pol II is the central component of the basal RNA polymerase II transcription machinery. It is composed of mobile elements that move relative to each other. RPB1 is part of the core element with the central large cleft, the clamp element that moves to open and close the cleft and the jaws that are thought to grab the incoming DNA template. At the start of transcription, a single-stranded DNA template strand of the promoter is positioned within the central active site cleft of Pol II. A bridging helix emanates from RPB1 and crosses the cleft near the catalytic site and is thought to promote translocation of Pol II by acting as a ratchet that moves the RNA-DNA hybrid through the active site by switching from straight to bent conformations at each step of nucleotide addition. During transcription elongation, Pol II moves on the template as the transcript elongates. Elongation is influenced by the phosphorylation status of the C-terminal domain (CTD) of Pol II largest subunit (RPB1), which serves as a platform for assembly of factors that regulate transcription initiation, elongation, termination and mRNA processing. Regulation of gene expression levels depends on the balance between methylation and acetylation levels of tha CTD-lysines (By similarity). Initiation or early elongation steps of transcription of growth-factors-induced immediate early genes are regulated by the acetylation status of the CTD (PubMed:24207025). Methylation and dimethylation have a repressive effect on target genes expression (By similarity).   
  
Component of the RNA polymerase II (Pol II) complex consisting of 12 subunits. Component of a complex which is at least composed of HTATSF1/Tat-SF1, the P-TEFb complex components CDK9 and CCNT1, RNA polymerase II, SUPT5H, and NCL/nucleolin. The large PER complex involved in the repression of transcriptional termination is composed of at least PER2, CDK9, DDX5, DHX9, NCBP1 and POLR2A (active). Interacts (via the C-terminal domain (CTD)) with U2AF2; recruits PRPF19 and the Prp19 complex to the pre-mRNA and may couple transcription to pre-mRNA splicing. Interacts (via the C-terminal domain (CTD)) with SMN1/SMN2; recruits SMN1/SMN2 to RNA Pol II elongation complexes. Interacts via the phosphorylated C-terminal domain with WDR82 and with SETD1A and SETD1B only in the presence of WDR82. When phosphorylated at 'Ser-5', interacts with MEN1; the unphosphorylated form, or phosphorylated at 'Ser-2' does not interact. When phosphorylated at 'Ser-2', interacts with SUPT6H (via SH2 domain). Interacts with RECQL5 and TCEA1; binding of RECQL5 prevents TCEA1 binding. The phosphorylated C-terminal domain interacts with FNBP3 and SYNCRIP. Interacts with ATF7IP. Interacts with DDX5. Interacts with WWP2. Interacts with SETX. Interacts (phosphorylated) with PIH1D1. Interacts (via the C-terminal domain (CTD)) with TDRD3. Interacts with PRMT5. Interacts with XRN2. Interacts with SAFB/SAFB1. Interacts with CCNL1. Interacts with CCNL2, MYO1C, PAF1 and SFRS19. Interacts (via C-terminus) with CMTR1, CTDSP1 and SCAF8. Interacts (via the C-terminal domain (CTD)) with CCNT2 (PubMed:15563843). Interacts with FUS. Interacts with MCM3AP isoform GANP (PubMed:23652018). Interacts with kinase SRPK2; the interaction occurs during the co-transcriptional formation of inappropriate R-loops (PubMed:28076779).   
  
 **Gene Ontology Information:**

Molecular Function

- DNA binding
- DNA-directed 5'-3' RNA polymerase activity
- kinase binding
- metal ion binding
- promoter-specific chromatin binding
- protein C-terminus binding
- RNA binding
- RNA-directed 5'-3' RNA polymerase activity
- ubiquitin protein ligase binding

Location

- chromosome
- cytoplasm
- nucleoplasm
- nucleus
- RNA polymerase II, core complex

Biological process

- DNA-templated transcription, termination
- positive regulation of RNA splicing
- regulation of transcription, DNA-templated
- transcription by RNA polymerase II

---

22

- **Protein name:** Integrin alpha-X
- **Organism:** Homo sapiens
- **Uniprot Accession Number:** P20702
- **Protein sequence length:** 1163 aa
- **1D identity (%):** 15.24
- **1D identity (%) [Gaps excluded]:** 22.92
- **1D identity - Alignment Gaps:** 490
- **1D aligned content (<aminoacid>:%):** {'R': 4.04, 'F': 6.73, 'A': 5.38, 'L': 12.56, 'G': 10.76, 'N': 3.14, 'D': 4.93, 'S': 10.76, 'W': 1.79, 'Y': 4.48, 'P': 7.62, 'T': 5.83, 'C': 1.79, 'E': 4.04, 'V': 6.73, 'I': 4.04, 'K': 2.24, 'Q': 3.14}
- **Common reported functions (%):** 0.0
- **Common reported locations (%):** 20.0
- **Common reported processes (%):** 0.0

- **PDB ID:** 4NEN
- **Chain:** A
- **Crystallized protein length:** 1063 aa
- **Resolution:** 2.9 Å
- **b-phipsi:** 0.010617
- **w-rdist:** 0.355692
- **t-alpha:** 0.032451
- **Chemical similarity (Tanimoto Index) (%):** 85.71
- **1D identity (%) [PDB]:** 3.1
- **1D identity (%) [Gaps excluded][PDB]:** 78.21
- **1D identity - Alignment Gaps [PDB]:** 1892
- **1D aligned content [PDB] (<aminoacid>:%):** {'F': 1.64, 'N': 4.92, 'S': 13.11, 'A': 9.84, 'G': 4.92, 'K': 3.28, 'I': 6.56, 'Q': 9.84, 'D': 6.56, 'T': 4.92, 'L': 16.39, 'V': 9.84, 'R': 3.28, 'P': 1.64, 'E': 3.28}
- **2D identity (%) [PDB]:** 28.0
- **2D identity (%) [Gaps excluded][PDB]:** 85.54
- **2D identity - Alignment Gaps [PDB]:** 1038
- **2D aligned content [PDB] (<2D-fold>:%):** {'.': 24.54, 'B': 0.46, 'S': 8.8, 'E': 55.32, 'T': 7.87, 'P': 1.16, 'H': 1.85}
- **3D similarity (TM-Score) (%) [PDB]:** 22.16

- **Gene name:** ITGAX
- **Entrez ID:** 3687
- **RefSeq ID:** NM\_001286375
- **Transcript sequence length:** 4092
- **5-UTR|CDS|3-UTR identity (%):** 17.54 | 43.52 | 34.71
- **5-UTR|CDS|3-UTR identity (%) [Gaps excluded]:** 84.75 | 72.98 | 79.73
- **5-UTR|CDS|3-UTR identity [Alignment Gaps]:** 226 | 1854 | 288
- **5-UTR aligned content (<base>:%):** {'A': 18.0, 'C': 34.0, 'T': 30.0, 'G': 18.0}
- **CDS aligned content (<base>:%):** {'T': 27.06, 'G': 23.11, 'C': 24.16, 'A': 25.66}
- **3-UTR aligned content (<base>:%):** {'A': 40.11, 'T': 21.47, 'C': 15.82, 'G': 22.6}

**Uniprot Description:**  
  
 Integrin alpha-X/beta-2 is a receptor for fibrinogen. It recognizes the sequence G-P-R in fibrinogen. It mediates cell-cell interaction during inflammatory responses. It is especially important in monocyte adhesion and chemotaxis.   
  
Heterodimer of an alpha and a beta subunit. Alpha-X associates with beta-2.   
  
 **Gene Ontology Information:**

Molecular Function

- integrin binding
- metal ion binding
- receptor tyrosine kinase binding
- signaling receptor activity

Location

- cell surface
- external side of plasma membrane
- ficolin-1-rich granule membrane
- integrin alphaX-beta2 complex
- integrin complex
- membrane
- plasma membrane
- secretory granule membrane
- tertiary granule membrane

Biological process

- animal organ morphogenesis
- cell adhesion
- cell adhesion mediated by integrin
- cell-cell adhesion
- cell-matrix adhesion
- defense response to virus
- heterotypic cell-cell adhesion
- integrin-mediated signaling pathway
- positive regulation of angiogenesis
- positive regulation of cell migration
- positive regulation of cell population proliferation
- positive regulation of endothelial tube morphogenesis
- positive regulation of gene expression
- positive regulation of myelination

---

23

- **Protein name:** Alpha-2-macroglobulin
- **Organism:** Homo sapiens
- **Uniprot Accession Number:** P01023
- **Protein sequence length:** 1474 aa
- **1D identity (%):** 17.94
- **1D identity (%) [Gaps excluded]:** 24.61
- **1D identity - Alignment Gaps:** 431
- **1D aligned content (<aminoacid>:%):** {'M': 1.05, 'L': 12.28, 'V': 9.82, 'P': 8.42, 'S': 6.67, 'T': 8.42, 'G': 8.42, 'Y': 4.56, 'F': 6.32, 'H': 0.7, 'N': 5.96, 'K': 4.56, 'E': 4.56, 'Q': 2.46, 'R': 3.16, 'D': 2.46, 'A': 5.96, 'I': 2.81, 'C': 1.4}
- **Common reported functions (%):** 0.0
- **Common reported locations (%):** 0.0
- **Common reported processes (%):** 0.0

- **PDB ID:** 4ACQ
- **Chain:** C
- **Crystallized protein length:** 1402 aa
- **Resolution:** 4.3 Å
- **b-phipsi:** 0.000249
- **w-rdist:** 0.755588
- **t-alpha:** 0.227461
- **Chemical similarity (Tanimoto Index) (%):** 94.39
- **1D identity (%) [PDB]:** 4.65
- **1D identity (%) [Gaps excluded][PDB]:** 68.42
- **1D identity - Alignment Gaps [PDB]:** 2083
- **1D aligned content [PDB] (<aminoacid>:%):** {'G': 8.65, 'K': 4.81, 'Q': 7.69, 'D': 3.85, 'V': 11.54, 'A': 6.73, 'L': 9.62, 'N': 4.81, 'S': 7.69, 'F': 4.81, 'P': 3.85, 'E': 4.81, 'I': 5.77, 'T': 6.73, 'Y': 3.85, 'R': 1.92, 'H': 1.92, 'M': 0.96}
- **2D identity (%) [PDB]:** 41.82
- **2D identity (%) [Gaps excluded][PDB]:** 86.15
- **2D identity - Alignment Gaps [PDB]:** 827
- **2D aligned content [PDB] (<2D-fold>:%):** {'.': 20.98, 'S': 6.55, 'E': 42.56, 'T': 6.25, 'P': 0.45, 'G': 0.45, 'H': 22.77}
- **3D similarity (TM-Score) (%) [PDB]:** 25.12

- **Gene name:** A2M
- **Entrez ID:** N/A
- **RefSeq ID:** NM\_001347425
- **Transcript sequence length:** 4450
- **5-UTR|CDS|3-UTR identity (%):** 40.25 | 45.18 | 28.81
- **5-UTR|CDS|3-UTR identity (%) [Gaps excluded]:** 71.56 | 75.14 | 69.31
- **5-UTR|CDS|3-UTR identity [Alignment Gaps]:** 175 | 1941 | 142
- **5-UTR aligned content (<base>:%):** {'A': 21.12, 'G': 20.5, 'T': 31.68, 'C': 26.71}
- **CDS aligned content (<base>:%):** {'A': 30.05, 'C': 21.36, 'T': 28.91, 'G': 19.68}
- **3-UTR aligned content (<base>:%):** {'A': 27.14, 'G': 28.57, 'C': 14.29, 'T': 30.0}

**Uniprot Description:**  
  
 Is able to inhibit all four classes of proteinases by a unique 'trapping' mechanism. This protein has a peptide stretch, called the 'bait region' which contains specific cleavage sites for different proteinases. When a proteinase cleaves the bait region, a conformational change is induced in the protein which traps the proteinase. The entrapped enzyme remains active against low molecular weight substrates (activity against high molecular weight substrates is greatly reduced). Following cleavage in the bait region, a thioester bond is hydrolyzed and mediates the covalent binding of the protein to the proteinase.   
  
Homotetramer; disulfide-linked.   
  
 **Gene Ontology Information:**

Molecular Function   
  
N/A

Location   
  
N/A

Biological process   
  
N/A

---

24

- **Protein name:** N/A
- **Organism:** N/A
- **Uniprot Accession Number:** N/A
- **Protein sequence length:** N/A
- **1D identity (%):** N/A
- **1D identity (%) [Gaps excluded]:** N/A
- **1D identity - Alignment Gaps:** N/A
- **1D aligned content (<aminoacid>:%):** N/A
- **Common reported functions (%):** 0.0
- **Common reported locations (%):** 0.0
- **Common reported processes (%):** 0.0

- **PDB ID:** 7SBY
- **Chain:** A
- **Crystallized protein length:** 1176 aa
- **Resolution:** 3.0 Å
- **b-phipsi:** 0.000984
- **w-rdist:** 0.800724
- **t-alpha:** 0.014061
- **Chemical similarity (Tanimoto Index) (%):** 99.68
- **1D identity (%) [PDB]:** 7.97
- **1D identity (%) [Gaps excluded][PDB]:** 58.91
- **1D identity - Alignment Gaps [PDB]:** 1648
- **1D aligned content [PDB] (<aminoacid>:%):** {'V': 7.24, 'F': 5.92, 'A': 10.53, 'K': 5.26, 'Y': 3.29, 'T': 4.61, 'P': 8.55, 'G': 5.92, 'N': 5.92, 'D': 2.63, 'S': 7.89, 'R': 3.95, 'I': 5.26, 'E': 2.63, 'L': 12.5, 'W': 0.66, 'Q': 5.92, 'H': 1.32}
- **2D identity (%) [PDB]:** 59.39
- **2D identity (%) [Gaps excluded][PDB]:** 84.85
- **2D identity - Alignment Gaps [PDB]:** 382
- **2D aligned content [PDB] (<2D-fold>:%):** {'.': 18.65, 'E': 39.15, 'T': 8.99, 'B': 0.4, 'S': 6.48, 'P': 1.59, 'H': 22.88, 'G': 1.19, 'I': 0.66}
- **3D similarity (TM-Score) (%) [PDB]:** 7.94

- **Gene name:** N/A
- **Entrez ID:** N/A
- **RefSeq ID:** N/A
- **Sequence length:** N/A
- **5-UTR|CDS|3-UTR identity (%):** N/A | N/A | N/A
- **5-UTR|CDS|3-UTR identity (%) [Gaps excluded]:** N/A | N/A | N/A
- **5-UTR|CDS|3-UTR identity [Alignment Gaps]:** N/A | N/A | N/A
- **5-UTR aligned content (<base>:%):** N/A
- **CDS aligned content (<base>:%):** N/A
- **3-UTR aligned content (<base>:%):** N/A

**Uniprot Description:**  
  
 N/A N/A   
  
 **Gene Ontology Information:**

Molecular Function   
  
N/A

Location   
  
N/A

Biological process   
  
N/A

---

25

- **Protein name:** N/A
- **Organism:** N/A
- **Uniprot Accession Number:** N/A
- **Protein sequence length:** N/A
- **1D identity (%):** N/A
- **1D identity (%) [Gaps excluded]:** N/A
- **1D identity - Alignment Gaps:** N/A
- **1D aligned content (<aminoacid>:%):** N/A
- **Common reported functions (%):** 0.0
- **Common reported locations (%):** 0.0
- **Common reported processes (%):** 0.0

- **PDB ID:** 7SBV
- **Chain:** J
- **Crystallized protein length:** 1176 aa
- **Resolution:** 3.1 Å
- **b-phipsi:** 0.000852
- **w-rdist:** 0.807956
- **t-alpha:** 0.013411
- **Chemical similarity (Tanimoto Index) (%):** 100.0
- **1D identity (%) [PDB]:** 8.2
- **1D identity (%) [Gaps excluded][PDB]:** 59.54
- **1D identity - Alignment Gaps [PDB]:** 1640
- **1D aligned content [PDB] (<aminoacid>:%):** {'V': 7.05, 'F': 6.41, 'A': 10.26, 'K': 5.77, 'Y': 3.21, 'T': 4.49, 'P': 7.69, 'I': 6.41, 'D': 2.56, 'N': 5.77, 'S': 8.33, 'L': 12.82, 'R': 3.85, 'E': 2.56, 'G': 5.13, 'W': 0.64, 'Q': 5.77, 'H': 1.28}
- **2D identity (%) [PDB]:** 59.14
- **2D identity (%) [Gaps excluded][PDB]:** 82.12
- **2D identity - Alignment Gaps [PDB]:** 352
- **2D aligned content [PDB] (<2D-fold>:%):** {'.': 19.62, 'E': 38.58, 'B': 0.4, 'S': 7.39, 'P': 1.48, 'T': 9.01, 'H': 21.1, 'G': 1.75, 'I': 0.67}
- **3D similarity (TM-Score) (%) [PDB]:** N/A

- **Gene name:** N/A
- **Entrez ID:** N/A
- **RefSeq ID:** N/A
- **Sequence length:** N/A
- **5-UTR|CDS|3-UTR identity (%):** N/A | N/A | N/A
- **5-UTR|CDS|3-UTR identity (%) [Gaps excluded]:** N/A | N/A | N/A
- **5-UTR|CDS|3-UTR identity [Alignment Gaps]:** N/A | N/A | N/A
- **5-UTR aligned content (<base>:%):** N/A
- **CDS aligned content (<base>:%):** N/A
- **3-UTR aligned content (<base>:%):** N/A

**Uniprot Description:**  
  
 N/A N/A   
  
 **Gene Ontology Information:**

Molecular Function   
  
N/A

Location   
  
N/A

Biological process   
  
N/A

---

26

- **Protein name:** Small nuclear ribonucleoprotein E
- **Organism:** Homo sapiens
- **Uniprot Accession Number:** P62304
- **Protein sequence length:** 92 aa
- **1D identity (%):** 1.71
- **1D identity (%) [Gaps excluded]:** 28.95
- **1D identity - Alignment Gaps:** 1213
- **1D aligned content (<aminoacid>:%):** {'M': 4.55, 'A': 9.09, 'Y': 4.55, 'R': 4.55, 'G': 4.55, 'V': 18.18, 'Q': 13.64, 'L': 9.09, 'I': 9.09, 'S': 9.09, 'K': 4.55, 'N': 9.09}
- **Common reported functions (%):** 0.0
- **Common reported locations (%):** 0.0
- **Common reported processes (%):** 0.0

- **PDB ID:** 5O9Z
- **Chain:** C
- **Crystallized protein length:** 1693 aa
- **Resolution:** 4.5 Å
- **b-phipsi:** 0.032294
- **w-rdist:** 0.271928
- **t-alpha:** 0.015967
- **Chemical similarity (Tanimoto Index) (%):** N/A
- **1D identity (%) [PDB]:** 3.41
- **1D identity (%) [Gaps excluded][PDB]:** 69.05
- **1D identity - Alignment Gaps [PDB]:** 2424
- **1D aligned content [PDB] (<aminoacid>:%):** {'G': 6.9, 'L': 10.34, 'P': 4.6, 'K': 6.9, 'Y': 2.3, 'N': 8.05, 'T': 12.64, 'I': 5.75, 'A': 4.6, 'D': 4.6, 'S': 6.9, 'F': 6.9, 'V': 6.9, 'E': 4.6, 'Q': 2.3, 'C': 1.15, 'R': 4.6}
- **2D identity (%) [PDB]:** 30.15
- **2D identity (%) [Gaps excluded][PDB]:** 87.46
- **2D identity - Alignment Gaps [PDB]:** 1304
- **2D aligned content [PDB] (<2D-fold>:%):** {'.': 19.17, 'E': 29.83, 'S': 8.5, 'T': 9.67, 'H': 30.67, 'G': 1.67, 'P': 0.33, 'B': 0.17}
- **3D similarity (TM-Score) (%) [PDB]:** 24.66

- **Gene name:** SNRPE
- **Entrez ID:** 6635
- **RefSeq ID:** NM\_003094
- **Transcript sequence length:** 1560
- **5-UTR|CDS|3-UTR identity (%):** 12.37 | 5.0 | 15.93
- **5-UTR|CDS|3-UTR identity (%) [Gaps excluded]:** 81.4 | 73.0 | 86.67
- **5-UTR|CDS|3-UTR identity [Alignment Gaps]:** 240 | 3575 | 999
- **5-UTR aligned content (<base>:%):** {'T': 31.43, 'C': 25.71, 'G': 34.29, 'A': 8.57}
- **CDS aligned content (<base>:%):** {'T': 30.21, 'G': 19.79, 'C': 15.1, 'A': 34.9}
- **3-UTR aligned content (<base>:%):** {'A': 41.03, 'T': 24.62, 'C': 14.36, 'G': 20.0}

**Uniprot Description:**  
  
 Plays role in pre-mRNA splicing as core component of the SMN-Sm complex that mediates spliceosomal snRNP assembly and as component of the spliceosomal U1, U2, U4 and U5 small nuclear ribonucleoproteins (snRNPs), the building blocks of the spliceosome (PubMed:11991638, PubMed:18984161, PubMed:23246290, PubMed:19325628, PubMed:23333303, PubMed:25555158, PubMed:26912367, PubMed:28502770, PubMed:28781166, PubMed:28076346). Component of both the pre-catalytic spliceosome B complex and activated spliceosome C complexes (PubMed:11991638, PubMed:28502770, PubMed:28781166, PubMed:28076346). Is also a component of the minor U12 spliceosome (PubMed:15146077). As part of the U7 snRNP it is involved in histone 3'-end processing (PubMed:12975319). May indirectly play a role in hair development (PubMed:23246290).   
  
Core component of the spliceosomal U1, U2, U4 and U5 small nuclear ribonucleoproteins (snRNPs), the building blocks of the spliceosome (PubMed:11991638, PubMed:23246290, PubMed:19325628, PubMed:21516107, PubMed:25555158, PubMed:26912367, PubMed:28502770, PubMed:28781166, PubMed:28076346). Most spliceosomal snRNPs contain a common set of Sm proteins, SNRPB, SNRPD1, SNRPD2, SNRPD3, SNRPE, SNRPF and SNRPG that assemble in a heptameric protein ring on the Sm site of the small nuclear RNA to form the core snRNP (PubMed:19325628, PubMed:21516107, PubMed:25555158, PubMed:26912367, PubMed:28502770, PubMed:28781166, PubMed:28076346). Component of the U1 snRNP (PubMed:19325628, PubMed:25555158). The U1 snRNP is composed of the U1 snRNA and the 7 core Sm proteins SNRPB, SNRPD1, SNRPD2, SNRPD3, SNRPE, SNRPF and SNRPG, and at least three U1 snRNP-specific proteins SNRNP70/U1-70K, SNRPA/U1-A and SNRPC/U1-C (PubMed:19325628, PubMed:25555158). Component of the U4/U6-U5 tri-snRNP complex composed of the U4, U6 and U5 snRNAs and at least PRPF3, PRPF4, PRPF6, PRPF8, PRPF31, SNRNP200, TXNL4A, SNRNP40, SNRPB, SNRPD1, SNRPD2, SNRPD3, SNRPE, SNRPF, SNRPG, DDX23, CD2BP2, PPIH, SNU13, EFTUD2, SART1 and USP39, plus LSM2, LSM3, LSM4, LSM5, LSM6, LSM7 and LSM8 (PubMed:26912367). Component of the U7 snRNP complex, or U7 Sm protein core complex, that is composed of the U7 snRNA and at least LSM10, LSM11, SNRPB, SNRPD3, SNRPE, SNRPF and SNRPG; the complex does not contain SNRPD1 and SNRPD2 (PubMed:11574479). Component of the U11/U12 snRNPs that are part of the U12-type spliceosome (PubMed:15146077). Part of the SMN-Sm complex that contains SMN1, GEMIN2/SIP1, DDX20/GEMIN3, GEMIN4, GEMIN5, GEMIN6, GEMIN7, GEMIN8, STRAP/UNRIP and the Sm proteins SNRPB, SNRPD1, SNRPD2, SNRPD3, SNRPE, SNRPF and SNRPG; catalyzes core snRNPs assembly (PubMed:18984161). Forms a 6S pICln-Sm complex composed of CLNS1A/pICln, SNRPD1, SNRPD2, SNRPE, SNRPF and SNRPG; ring-like structure where CLNS1A/pICln mimics additional Sm proteins and which is unable to assemble into the core snRNP (PubMed:18984161, PubMed:23333303).   
  
 **Gene Ontology Information:**

Molecular Function

- RNA binding

Location

- catalytic step 2 spliceosome
- cytosol
- methylosome
- nucleoplasm
- nucleus
- pICln-Sm protein complex
- precatalytic spliceosome
- small nuclear ribonucleoprotein complex
- SMN-Sm protein complex
- spliceosomal complex
- telomerase holoenzyme complex
- U1 snRNP
- U12-type spliceosomal complex
- U2 snRNP
- U2-type catalytic step 2 spliceosome
- U2-type precatalytic spliceosome
- U4 snRNP
- U4/U6 x U5 tri-snRNP complex
- U5 snRNP
- U7 snRNP

Biological process

- 7-methylguanosine cap hypermethylation
- mRNA splicing, via spliceosome
- spliceosomal complex assembly
- spliceosomal snRNP assembly
- U2-type prespliceosome assembly

---

27

- **Protein name:** NPC intracellular cholesterol transporter 1
- **Organism:** Homo sapiens
- **Uniprot Accession Number:** O15118
- **Protein sequence length:** 1278 aa
- **1D identity (%):** 15.16
- **1D identity (%) [Gaps excluded]:** 24.51
- **1D identity - Alignment Gaps:** 601
- **1D aligned content (<aminoacid>:%):** {'R': 2.09, 'L': 12.55, 'P': 7.11, 'F': 7.95, 'W': 1.26, 'G': 10.88, 'K': 3.35, 'Y': 3.77, 'E': 2.93, 'T': 5.02, 'D': 7.95, 'N': 6.28, 'C': 4.6, 'Q': 1.67, 'V': 5.44, 'S': 7.11, 'I': 3.77, 'A': 5.86, 'H': 0.42}
- **Common reported functions (%):** 0.0
- **Common reported locations (%):** 20.0
- **Common reported processes (%):** 10.0

- **PDB ID:** 6W5S
- **Chain:** A
- **Crystallized protein length:** 1194 aa
- **Resolution:** 3.0 Å
- **b-phipsi:** 0.048481
- **w-rdist:** 0.554039
- **t-alpha:** 0.009182
- **Chemical similarity (Tanimoto Index) (%):** N/A
- **1D identity (%) [PDB]:** 3.54
- **1D identity (%) [Gaps excluded][PDB]:** 61.86
- **1D identity - Alignment Gaps [PDB]:** 1943
- **1D aligned content [PDB] (<aminoacid>:%):** {'S': 8.22, 'A': 8.22, 'L': 17.81, 'K': 6.85, 'D': 4.11, 'V': 9.59, 'Q': 8.22, 'N': 5.48, 'T': 1.37, 'F': 4.11, 'G': 8.22, 'I': 4.11, 'R': 5.48, 'E': 2.74, 'Y': 1.37, 'H': 2.74, 'P': 1.37}
- **2D identity (%) [PDB]:** 24.63
- **2D identity (%) [Gaps excluded][PDB]:** 83.13
- **2D identity - Alignment Gaps [PDB]:** 1183
- **2D aligned content [PDB] (<2D-fold>:%):** {'.': 25.85, 'E': 11.11, 'T': 9.18, 'S': 7.73, 'H': 42.51, 'G': 2.42, 'I': 1.21}
- **3D similarity (TM-Score) (%) [PDB]:** 27.36

- **Gene name:** NPC1
- **Entrez ID:** 4864
- **RefSeq ID:** NM\_000271
- **Transcript sequence length:** 4760
- **5-UTR|CDS|3-UTR identity (%):** 30.41 | 45.63 | 24.77
- **5-UTR|CDS|3-UTR identity (%) [Gaps excluded]:** 68.18 | 74.19 | 85.59
- **5-UTR|CDS|3-UTR identity [Alignment Gaps]:** 164 | 1825 | 545
- **5-UTR aligned content (<base>:%):** {'C': 38.89, 'T': 16.67, 'G': 27.78, 'A': 16.67}
- **CDS aligned content (<base>:%):** {'A': 26.16, 'T': 33.18, 'G': 19.69, 'C': 20.98}
- **3-UTR aligned content (<base>:%):** {'C': 14.21, 'T': 23.68, 'A': 41.58, 'G': 20.53}

**Uniprot Description:**  
  
 Intracellular cholesterol transporter which acts in concert with NPC2 and plays an important role in the egress of cholesterol from the endosomal/lysosomal compartment (PubMed:9211849, PubMed:9927649, PubMed:10821832, PubMed:18772377, PubMed:27238017, PubMed:12554680). Unesterified cholesterol that has been released from LDLs in the lumen of the late endosomes/lysosomes is transferred by NPC2 to the cholesterol-binding pocket in the N-terminal domain of NPC1 (PubMed:9211849, PubMed:9927649, PubMed:18772377, PubMed:19563754, PubMed:27238017, PubMed:28784760). Cholesterol binds to NPC1 with the hydroxyl group buried in the binding pocket (PubMed:19563754). Binds oxysterol with higher affinity than cholesterol. May play a role in vesicular trafficking in glia, a process that may be crucial for maintaining the structural and functional integrity of nerve terminals (Probable).   
  
Interacts (via the second lumenal domain) with NPC2 (PubMed:18772377, PubMed:27238017, PubMed:27551080). Interacts with TMEM97 (PubMed:19583955). Interacts with TIM1 (PubMed:25855742).   
  
 **Gene Ontology Information:**

Molecular Function

- cholesterol binding
- signaling receptor activity
- sterol binding
- sterol transporter activity
- transmembrane signaling receptor activity
- virus receptor activity

Location

- endoplasmic reticulum
- extracellular exosome
- extracellular region
- Golgi apparatus
- late endosome membrane
- lysosomal membrane
- lysosome
- membrane
- membrane raft
- nuclear envelope
- perinuclear region of cytoplasm
- plasma membrane

Biological process

- adult walking behavior
- autophagy
- bile acid metabolic process
- cellular response to low-density lipoprotein particle stimulus
- cellular response to steroid hormone stimulus
- cholesterol efflux
- cholesterol homeostasis
- cholesterol metabolic process
- cholesterol transport
- endocytosis
- establishment of protein localization to membrane
- gene expression
- intestinal cholesterol absorption
- intracellular cholesterol transport
- lysosomal transport
- macroautophagy
- membrane raft organization
- negative regulation of cell death
- negative regulation of macroautophagy
- negative regulation of TORC1 signaling
- protein glycosylation
- response to cadmium ion
- response to xenobiotic stimulus
- sterol transport
- viral entry into host cell

---

28

- **Protein name:** 6-deoxyerythronolide-B synthase EryA1, modules 1 and 2
- **Organism:** Saccharopolyspora erythraea
- **Uniprot Accession Number:** Q03131
- **Protein sequence length:** 3491 aa
- **1D identity (%):** 11.29
- **1D identity (%) [Gaps excluded]:** 31.25
- **1D identity - Alignment Gaps:** 2236
- **1D aligned content (<aminoacid>:%):** {'M': 0.76, 'F': 5.57, 'V': 9.87, 'P': 9.11, 'L': 11.9, 'R': 5.32, 'G': 14.18, 'H': 2.03, 'S': 4.81, 'Q': 1.77, 'D': 5.82, 'T': 5.82, 'W': 1.01, 'N': 2.28, 'A': 10.38, 'I': 2.03, 'C': 1.27, 'E': 3.8, 'Y': 2.03, 'K': 0.25}
- **Common reported functions (%):** 0.0
- **Common reported locations (%):** 0.0
- **Common reported processes (%):** 0.0

- **PDB ID:** 7M7F
- **Chain:** A
- **Crystallized protein length:** 1390 aa
- **Resolution:** 3.2 Å
- **b-phipsi:** 0.020257
- **w-rdist:** 0.633972
- **t-alpha:** 0.010025
- **Chemical similarity (Tanimoto Index) (%):** N/A
- **1D identity (%) [PDB]:** 1.94
- **1D identity (%) [Gaps excluded][PDB]:** 76.27
- **1D identity - Alignment Gaps [PDB]:** 2255
- **1D aligned content [PDB] (<aminoacid>:%):** {'D': 11.11, 'S': 17.78, 'L': 15.56, 'T': 4.44, 'A': 11.11, 'G': 4.44, 'Q': 4.44, 'V': 11.11, 'K': 2.22, 'F': 2.22, 'R': 4.44, 'P': 4.44, 'E': 4.44, 'I': 2.22}
- **2D identity (%) [PDB]:** 29.61
- **2D identity (%) [Gaps excluded][PDB]:** 86.16
- **2D identity - Alignment Gaps [PDB]:** 1159
- **2D aligned content [PDB] (<2D-fold>:%):** {'.': 28.11, 'S': 8.41, 'E': 28.49, 'T': 11.09, 'P': 0.76, 'H': 21.41, 'G': 1.72}
- **3D similarity (TM-Score) (%) [PDB]:** 19.75

- **Gene name:** eryA
- **Entrez ID:** N/A
- **RefSeq ID:** N/A
- **Sequence length:** N/A
- **5-UTR|CDS|3-UTR identity (%):** N/A | N/A | N/A
- **5-UTR|CDS|3-UTR identity (%) [Gaps excluded]:** N/A | N/A | N/A
- **5-UTR|CDS|3-UTR identity [Alignment Gaps]:** N/A | N/A | N/A
- **5-UTR aligned content (<base>:%):** N/A
- **CDS aligned content (<base>:%):** N/A
- **3-UTR aligned content (<base>:%):** N/A

**Uniprot Description:**  
  
 Involved in the biosynthesis of antibiotic erythromycin via the biosynthesis of its aglycone precursor, 6-deoxyerythronolide B (6-dEB).   
  
Homodimer (PubMed:16564177). Erythronolide synthase is composed of EryAI, EryAII and EryAIII multimodular (2 modules) polypeptides each coding for a functional synthase subunit which participates in 2 of the six FAS-like elongation steps required for formation of the polyketide. Module 1, 2, 3, 4, 5, and 6 participating in biosynthesis steps 1, 2, 3, 4, 5, and 6, respectively.   
  
 **Gene Ontology Information:**

Molecular Function

- 3-oxoacyl-[acyl-carrier-protein] synthase activity
- erythronolide synthase activity
- phosphopantetheine binding

Location   
  
N/A

Biological process

- fatty acid biosynthetic process
- macrolide biosynthetic process

---

29

- **Protein name:** DNA-directed RNA polymerase subunit
- **Organism:** Bos taurus
- **Uniprot Accession Number:** G3MZY8
- **Protein sequence length:** 1970 aa
- **1D identity (%):** 15.31
- **1D identity (%) [Gaps excluded]:** 25.66
- **1D identity - Alignment Gaps:** 819
- **1D aligned content (<aminoacid>:%):** {'P': 7.72, 'S': 8.36, 'C': 0.96, 'R': 4.18, 'Q': 2.57, 'G': 9.97, 'Y': 4.82, 'V': 7.72, 'T': 7.4, 'L': 8.36, 'F': 4.5, 'H': 0.96, 'N': 7.4, 'D': 6.75, 'A': 6.75, 'E': 3.54, 'K': 4.18, 'W': 0.64, 'M': 1.29, 'I': 1.93}
- **Common reported functions (%):** 0.0
- **Common reported locations (%):** 0.0
- **Common reported processes (%):** 0.0

- **PDB ID:** 5OIK
- **Chain:** A
- **Crystallized protein length:** 1421 aa
- **Resolution:** 3.7 Å
- **b-phipsi:** 0.025317
- **w-rdist:** 0.609123
- **t-alpha:** 0.01087
- **Chemical similarity (Tanimoto Index) (%):** 83.31
- **1D identity (%) [PDB]:** 3.26
- **1D identity (%) [Gaps excluded][PDB]:** 68.81
- **1D identity - Alignment Gaps [PDB]:** 2190
- **1D aligned content [PDB] (<aminoacid>:%):** {'S': 8.0, 'A': 10.67, 'L': 16.0, 'G': 8.0, 'D': 4.0, 'V': 6.67, 'Q': 6.67, 'N': 6.67, 'T': 4.0, 'K': 5.33, 'F': 4.0, 'I': 4.0, 'P': 2.67, 'E': 5.33, 'R': 5.33, 'H': 1.33, 'M': 1.33}
- **2D identity (%) [PDB]:** 32.21
- **2D identity (%) [Gaps excluded][PDB]:** 84.77
- **2D identity - Alignment Gaps [PDB]:** 1082
- **2D aligned content [PDB] (<2D-fold>:%):** {'.': 20.28, 'E': 26.51, 'S': 9.61, 'T': 8.9, 'H': 33.45, 'G': 1.07, 'B': 0.18}
- **3D similarity (TM-Score) (%) [PDB]:** 23.42

- **Gene name:** POLR2A
- **Entrez ID:** N/A
- **RefSeq ID:** N/A
- **Sequence length:** N/A
- **5-UTR|CDS|3-UTR identity (%):** N/A | N/A | N/A
- **5-UTR|CDS|3-UTR identity (%) [Gaps excluded]:** N/A | N/A | N/A
- **5-UTR|CDS|3-UTR identity [Alignment Gaps]:** N/A | N/A | N/A
- **5-UTR aligned content (<base>:%):** N/A
- **CDS aligned content (<base>:%):** N/A
- **3-UTR aligned content (<base>:%):** N/A

**Uniprot Description:**  
  
 DNA-dependent RNA polymerase catalyzes the transcription of DNA into RNA using the four ribonucleoside triphosphates as substrates. N/A   
  
 **Gene Ontology Information:**

Molecular Function   
  
N/A

Location   
  
N/A

Biological process   
  
N/A

---

30

- **Protein name:** N/A
- **Organism:** N/A
- **Uniprot Accession Number:** N/A
- **Protein sequence length:** N/A
- **1D identity (%):** N/A
- **1D identity (%) [Gaps excluded]:** N/A
- **1D identity - Alignment Gaps:** N/A
- **1D aligned content (<aminoacid>:%):** N/A
- **Common reported functions (%):** 0.0
- **Common reported locations (%):** 0.0
- **Common reported processes (%):** 0.0

- **PDB ID:** 7SBW
- **Chain:** B
- **Crystallized protein length:** 1196 aa
- **Resolution:** 3.2 Å
- **b-phipsi:** 0.000828
- **w-rdist:** 0.814068
- **t-alpha:** 0.076923
- **Chemical similarity (Tanimoto Index) (%):** 99.68
- **1D identity (%) [PDB]:** 7.92
- **1D identity (%) [Gaps excluded][PDB]:** 57.79
- **1D identity - Alignment Gaps [PDB]:** 1656
- **1D aligned content [PDB] (<aminoacid>:%):** {'V': 7.24, 'F': 6.58, 'A': 10.53, 'K': 5.26, 'Q': 6.58, 'Y': 3.29, 'T': 4.61, 'P': 6.58, 'G': 6.58, 'N': 5.92, 'S': 7.89, 'L': 13.16, 'R': 3.95, 'I': 5.26, 'E': 2.63, 'D': 1.97, 'W': 0.66, 'H': 1.32}
- **2D identity (%) [PDB]:** 59.47
- **2D identity (%) [Gaps excluded][PDB]:** 85.68
- **2D identity - Alignment Gaps [PDB]:** 394
- **2D aligned content [PDB] (<2D-fold>:%):** {'.': 18.67, 'E': 38.64, 'T': 9.4, 'B': 0.39, 'S': 6.53, 'P': 1.31, 'H': 22.58, 'G': 1.83, 'I': 0.65}
- **3D similarity (TM-Score) (%) [PDB]:** 7.23

- **Gene name:** N/A
- **Entrez ID:** N/A
- **RefSeq ID:** N/A
- **Sequence length:** N/A
- **5-UTR|CDS|3-UTR identity (%):** N/A | N/A | N/A
- **5-UTR|CDS|3-UTR identity (%) [Gaps excluded]:** N/A | N/A | N/A
- **5-UTR|CDS|3-UTR identity [Alignment Gaps]:** N/A | N/A | N/A
- **5-UTR aligned content (<base>:%):** N/A
- **CDS aligned content (<base>:%):** N/A
- **3-UTR aligned content (<base>:%):** N/A

**Uniprot Description:**  
  
 N/A N/A   
  
 **Gene Ontology Information:**

Molecular Function   
  
N/A

Location   
  
N/A

Biological process   
  
N/A

---

31

- **Protein name:** Mucin-2
- **Organism:** Homo sapiens
- **Uniprot Accession Number:** Q02817
- **Protein sequence length:** 5289 aa
- **1D identity (%):** 7.02
- **1D identity (%) [Gaps excluded]:** 29.91
- **1D identity - Alignment Gaps:** 4068
- **1D aligned content (<aminoacid>:%):** {'L': 7.24, 'P': 8.58, 'C': 6.7, 'T': 15.01, 'R': 1.61, 'F': 4.56, 'G': 10.46, 'Y': 4.29, 'V': 7.24, 'S': 9.12, 'D': 4.56, 'W': 0.8, 'H': 1.34, 'N': 2.95, 'E': 2.95, 'K': 2.95, 'Q': 2.41, 'I': 4.29, 'A': 2.14, 'M': 0.8}
- **Common reported functions (%):** 0.0
- **Common reported locations (%):** 20.0
- **Common reported processes (%):** 0.0

- **PDB ID:** 7POV
- **Chain:** A
- **Crystallized protein length:** 708 aa
- **Resolution:** 3.8 Å
- **b-phipsi:** 0.005231
- **w-rdist:** 0.733795
- **t-alpha:** 0.025641
- **Chemical similarity (Tanimoto Index) (%):** 91.04
- **1D identity (%) [PDB]:** 2.71
- **1D identity (%) [Gaps excluded][PDB]:** 62.86
- **1D identity - Alignment Gaps [PDB]:** 1553
- **1D aligned content [PDB] (<aminoacid>:%):** {'N': 4.55, 'D': 9.09, 'Y': 9.09, 'K': 6.82, 'S': 2.27, 'P': 9.09, 'L': 18.18, 'V': 6.82, 'G': 9.09, 'A': 6.82, 'E': 4.55, 'I': 2.27, 'T': 4.55, 'F': 2.27, 'Q': 2.27, 'R': 2.27}
- **2D identity (%) [PDB]:** 36.3
- **2D identity (%) [Gaps excluded][PDB]:** 80.76
- **2D identity - Alignment Gaps [PDB]:** 643
- **2D aligned content [PDB] (<2D-fold>:%):** {'E': 39.39, 'T': 10.38, '.': 20.99, 'S': 11.32, 'H': 14.15, 'P': 0.71, 'G': 2.83, 'B': 0.24}
- **3D similarity (TM-Score) (%) [PDB]:** 19.06

- **Gene name:** MUC2
- **Entrez ID:** N/A
- **RefSeq ID:** N/A
- **Sequence length:** N/A
- **5-UTR|CDS|3-UTR identity (%):** N/A | N/A | N/A
- **5-UTR|CDS|3-UTR identity (%) [Gaps excluded]:** N/A | N/A | N/A
- **5-UTR|CDS|3-UTR identity [Alignment Gaps]:** N/A | N/A | N/A
- **5-UTR aligned content (<base>:%):** N/A
- **CDS aligned content (<base>:%):** N/A
- **3-UTR aligned content (<base>:%):** N/A

**Uniprot Description:**  
  
 Coats the epithelia of the intestines and other mucus membrane-containing organs to provide a protective, lubricating barrier against particles and infectious agents at mucosal surfaces (PubMed:17058067, PubMed:19432394, PubMed:33031746). Major constituent of the colon mucus, which is mainly formed by large polymeric networks of MUC2 secreted by goblet cells that cover the exposed surfaces of intestine (PubMed:19432394, PubMed:33031746). MUC2 networks form hydrogels that guard the underlying epithelium from pathogens and other hazardous matter entering from the outside world, while permitting nutrient absorption and gas exchange (PubMed:33031746, PubMed:36206754). Acts as a divalent copper chaperone that protects intestinal cells from copper toxicity and facilitates nutritional copper unptake into cells (PubMed:36206754). Binds both Cu(2+) and its reduced form, Cu(1+), at two juxtaposed binding sites: Cu(2+), once reduced to Cu(1+) by vitamin C (ascorbate) or other dietary antioxidants, transits to the other binding site (PubMed:36206754). MUC2-bound Cu(1+) is protected from oxidation in aerobic environments, and can be released for nutritional delivery to cells (PubMed:36206754). Mucin gels store antimicrobial molecules that participate in innate immunity (PubMed:33031746). Mucin glycoproteins also house and feed the microbiome, lubricate tissue surfaces, and may facilitate the removal of contaminants and waste products from the body (PubMed:33031746). Goblet cells synthesize two forms of MUC2 mucin that differ in branched chain O-glycosylation and the site of production in the colon: a (1) 'thick' mucus that wraps the microbiota to form fecal pellets is produced in the proximal, ascending colon (By similarity). 'Thick' mucus transits along the descending colon and is lubricated by a (2) 'thin' MUC2 mucus produced in the distal colon which adheres to the 'thick' mucus (By similarity).   
  
Homomultimer; disulfide-linked (PubMed:12374796, PubMed:31310764, PubMed:33031746, PubMed:35377815). The N- and C-terminus mediate their assembly into higher order structures to form filaments (PubMed:33031746, PubMed:35377815). The CTCK domains of two polypeptides associate in the endoplasmic reticulum to generate intermolecularly disulfide-bonded dimers (By similarity). These dimers progress to the Golgi apparatus, which is a more acidic environment than the endoplasmic reticulum (PubMed:33031746). Under acidic conditions, the N-termini form non-covalent intermolecular interactions that juxtapose assemblies of the third VWD domain (VWD3) from different CTCK-linked dimers (PubMed:33031746). The VWD3 assemblies then become disulfide bonded to one another to produce long, disulfide-linked polymers that remain highly compact until secretion (PubMed:33031746). Interacts with FCGBP (PubMed:19432394). Interacts with AGR2; disulfide-linked (PubMed:19359471).   
  
 **Gene Ontology Information:**

Molecular Function

- cupric ion binding
- cuprous ion binding

Location

- collagen-containing extracellular matrix
- extracellular matrix
- extracellular space
- Golgi lumen
- inner mucus layer
- outer mucus layer
- plasma membrane

Biological process

- detoxification of copper ion
- host-mediated regulation of intestinal microbiota composition
- maintenance of gastrointestinal epithelium

---

32

- **Protein name:** ATP-citrate synthase
- **Organism:** Homo sapiens
- **Uniprot Accession Number:** P53396
- **Protein sequence length:** 1101 aa
- **1D identity (%):** 16.29
- **1D identity (%) [Gaps excluded]:** 23.91
- **1D identity - Alignment Gaps:** 450
- **1D aligned content (<aminoacid>:%):** {'S': 4.78, 'G': 10.87, 'K': 4.78, 'I': 5.65, 'F': 5.65, 'Y': 3.91, 'T': 9.13, 'P': 7.83, 'L': 10.0, 'D': 6.96, 'V': 8.7, 'N': 3.48, 'A': 6.52, 'E': 2.17, 'C': 2.17, 'W': 0.87, 'R': 2.61, 'Q': 3.48, 'M': 0.43}
- **Common reported functions (%):** 0.0
- **Common reported locations (%):** 0.0
- **Common reported processes (%):** 0.0

- **PDB ID:** 6UIA
- **Chain:** B
- **Crystallized protein length:** 1021 aa
- **Resolution:** 4.3 Å
- **b-phipsi:** 0.020798
- **w-rdist:** 0.74421
- **t-alpha:** 0.01087
- **Chemical similarity (Tanimoto Index) (%):** 84.05
- **1D identity (%) [PDB]:** 3.8
- **1D identity (%) [Gaps excluded][PDB]:** 63.72
- **1D identity - Alignment Gaps [PDB]:** 1780
- **1D aligned content [PDB] (<aminoacid>:%):** {'A': 8.33, 'E': 5.56, 'K': 4.17, 'S': 2.78, 'N': 6.94, 'L': 9.72, 'V': 9.72, 'T': 8.33, 'G': 12.5, 'F': 5.56, 'P': 4.17, 'R': 4.17, 'I': 6.94, 'D': 5.56, 'Q': 2.78, 'Y': 1.39, 'H': 1.39}
- **2D identity (%) [PDB]:** 33.85
- **2D identity (%) [Gaps excluded][PDB]:** 84.03
- **2D identity - Alignment Gaps [PDB]:** 854
- **2D aligned content [PDB] (<2D-fold>:%):** {'.': 20.66, 'S': 5.58, 'E': 29.75, 'T': 7.64, 'H': 35.54, 'P': 0.62, 'B': 0.21}
- **3D similarity (TM-Score) (%) [PDB]:** 21.49

- **Gene name:** ACLY
- **Entrez ID:** 47
- **RefSeq ID:** N/A
- **Sequence length:** N/A
- **5-UTR|CDS|3-UTR identity (%):** N/A | N/A | N/A
- **5-UTR|CDS|3-UTR identity (%) [Gaps excluded]:** N/A | N/A | N/A
- **5-UTR|CDS|3-UTR identity [Alignment Gaps]:** N/A | N/A | N/A
- **5-UTR aligned content (<base>:%):** N/A
- **CDS aligned content (<base>:%):** N/A
- **3-UTR aligned content (<base>:%):** N/A

**Uniprot Description:**  
  
 Catalyzes the cleavage of citrate into oxaloacetate and acetyl-CoA, the latter serving as common substrate for de novo cholesterol and fatty acid synthesis.   
  
Homotetramer.   
  
 **Gene Ontology Information:**

Molecular Function

- ATP binding
- ATP citrate synthase activity
- metal ion binding

Location

- azurophil granule lumen
- cytosol
- extracellular exosome
- extracellular region
- ficolin-1-rich granule lumen
- membrane
- nucleoplasm

Biological process

- acetyl-CoA biosynthetic process
- cholesterol biosynthetic process
- citrate metabolic process
- coenzyme A metabolic process
- fatty acid biosynthetic process
- lipid biosynthetic process
- oxaloacetate metabolic process
- tricarboxylic acid cycle

---

33

- **Protein name:** N/A
- **Organism:** N/A
- **Uniprot Accession Number:** N/A
- **Protein sequence length:** N/A
- **1D identity (%):** N/A
- **1D identity (%) [Gaps excluded]:** N/A
- **1D identity - Alignment Gaps:** N/A
- **1D aligned content (<aminoacid>:%):** N/A
- **Common reported functions (%):** 0.0
- **Common reported locations (%):** 0.0
- **Common reported processes (%):** 0.0

- **PDB ID:** 7SBW
- **Chain:** J
- **Crystallized protein length:** 1191 aa
- **Resolution:** 3.2 Å
- **b-phipsi:** 0.001188
- **w-rdist:** 0.802092
- **t-alpha:** 0.028061
- **Chemical similarity (Tanimoto Index) (%):** 100.0
- **1D identity (%) [PDB]:** 8.09
- **1D identity (%) [Gaps excluded][PDB]:** 59.16
- **1D identity - Alignment Gaps [PDB]:** 1654
- **1D aligned content [PDB] (<aminoacid>:%):** {'V': 7.1, 'F': 6.45, 'A': 10.32, 'K': 5.81, 'Y': 3.23, 'T': 4.52, 'P': 6.45, 'I': 6.45, 'D': 3.23, 'N': 5.81, 'S': 8.39, 'L': 12.9, 'R': 3.87, 'E': 2.58, 'G': 5.16, 'W': 0.65, 'Q': 5.81, 'H': 1.29}
- **2D identity (%) [PDB]:** 59.77
- **2D identity (%) [Gaps excluded][PDB]:** 86.0
- **2D identity - Alignment Gaps [PDB]:** 392
- **2D aligned content [PDB] (<2D-fold>:%):** {'.': 18.88, 'E': 39.19, 'T': 8.85, 'B': 0.39, 'S': 6.38, 'P': 1.56, 'H': 22.53, 'G': 1.56, 'I': 0.65}
- **3D similarity (TM-Score) (%) [PDB]:** N/A

- **Gene name:** N/A
- **Entrez ID:** N/A
- **RefSeq ID:** N/A
- **Sequence length:** N/A
- **5-UTR|CDS|3-UTR identity (%):** N/A | N/A | N/A
- **5-UTR|CDS|3-UTR identity (%) [Gaps excluded]:** N/A | N/A | N/A
- **5-UTR|CDS|3-UTR identity [Alignment Gaps]:** N/A | N/A | N/A
- **5-UTR aligned content (<base>:%):** N/A
- **CDS aligned content (<base>:%):** N/A
- **3-UTR aligned content (<base>:%):** N/A

**Uniprot Description:**  
  
 N/A N/A   
  
 **Gene Ontology Information:**

Molecular Function   
  
N/A

Location   
  
N/A

Biological process   
  
N/A

---

34

- **Protein name:** Insulin receptor
- **Organism:** Homo sapiens
- **Uniprot Accession Number:** P06213
- **Protein sequence length:** 1382 aa
- **1D identity (%):** 14.85
- **1D identity (%) [Gaps excluded]:** 22.62
- **1D identity - Alignment Gaps:** 551
- **1D aligned content (<aminoacid>:%):** {'G': 14.29, 'L': 10.08, 'A': 5.04, 'N': 7.56, 'S': 7.98, 'E': 5.04, 'F': 6.72, 'P': 5.88, 'R': 3.78, 'K': 3.78, 'I': 3.36, 'Y': 2.52, 'T': 8.4, 'C': 3.78, 'Q': 3.36, 'V': 4.2, 'W': 0.84, 'D': 3.36}
- **Common reported functions (%):** 50.0
- **Common reported locations (%):** 20.0
- **Common reported processes (%):** 10.0

- **PDB ID:** 4ZXB
- **Chain:** E
- **Crystallized protein length:** 800 aa
- **Resolution:** 3.3 Å
- **b-phipsi:** 0.004987
- **w-rdist:** 0.764615
- **t-alpha:** 0.020253
- **Chemical similarity (Tanimoto Index) (%):** 94.56
- **1D identity (%) [PDB]:** 1.6
- **1D identity (%) [Gaps excluded][PDB]:** 82.35
- **1D identity - Alignment Gaps [PDB]:** 1721
- **1D aligned content [PDB] (<aminoacid>:%):** {'L': 7.14, 'E': 3.57, 'I': 10.71, 'D': 7.14, 'P': 3.57, 'F': 7.14, 'S': 10.71, 'G': 10.71, 'V': 10.71, 'T': 10.71, 'N': 7.14, 'Q': 3.57, 'A': 3.57, 'Y': 3.57}
- **2D identity (%) [PDB]:** 28.83
- **2D identity (%) [Gaps excluded][PDB]:** 80.68
- **2D identity - Alignment Gaps [PDB]:** 847
- **2D aligned content [PDB] (<2D-fold>:%):** {'.': 24.21, 'S': 6.32, 'E': 56.32, 'T': 8.95, 'P': 1.32, 'H': 2.89}
- **3D similarity (TM-Score) (%) [PDB]:** 15.4

- **Gene name:** INSR
- **Entrez ID:** 3643
- **RefSeq ID:** NM\_001079817
- **Transcript sequence length:** 9427
- **5-UTR|CDS|3-UTR identity (%):** 28.73 | 44.4 | 4.63
- **5-UTR|CDS|3-UTR identity (%) [Gaps excluded]:** 69.26 | 72.41 | 96.94
- **5-UTR|CDS|3-UTR identity [Alignment Gaps]:** 326 | 1903 | 4562
- **5-UTR aligned content (<base>:%):** {'A': 14.37, 'G': 26.25, 'T': 23.75, 'C': 35.62}
- **CDS aligned content (<base>:%):** {'A': 27.2, 'T': 28.34, 'G': 22.25, 'C': 22.21}
- **3-UTR aligned content (<base>:%):** {'C': 14.86, 'A': 40.09, 'T': 23.87, 'G': 21.17}

**Uniprot Description:**  
  
 Receptor tyrosine kinase which mediates the pleiotropic actions of insulin. Binding of insulin leads to phosphorylation of several intracellular substrates, including, insulin receptor substrates (IRS1, 2, 3, 4), SHC, GAB1, CBL and other signaling intermediates. Each of these phosphorylated proteins serve as docking proteins for other signaling proteins that contain Src-homology-2 domains (SH2 domain) that specifically recognize different phosphotyrosine residues, including the p85 regulatory subunit of PI3K and SHP2. Phosphorylation of IRSs proteins lead to the activation of two main signaling pathways: the PI3K-AKT/PKB pathway, which is responsible for most of the metabolic actions of insulin, and the Ras-MAPK pathway, which regulates expression of some genes and cooperates with the PI3K pathway to control cell growth and differentiation. Binding of the SH2 domains of PI3K to phosphotyrosines on IRS1 leads to the activation of PI3K and the generation of phosphatidylinositol-(3, 4, 5)-triphosphate (PIP3), a lipid second messenger, which activates several PIP3-dependent serine/threonine kinases, such as PDPK1 and subsequently AKT/PKB. The net effect of this pathway is to produce a translocation of the glucose transporter SLC2A4/GLUT4 from cytoplasmic vesicles to the cell membrane to facilitate glucose transport. Moreover, upon insulin stimulation, activated AKT/PKB is responsible for: anti-apoptotic effect of insulin by inducing phosphorylation of BAD; regulates the expression of gluconeogenic and lipogenic enzymes by controlling the activity of the winged helix or forkhead (FOX) class of transcription factors. Another pathway regulated by PI3K-AKT/PKB activation is mTORC1 signaling pathway which regulates cell growth and metabolism and integrates signals from insulin. AKT mediates insulin-stimulated protein synthesis by phosphorylating TSC2 thereby activating mTORC1 pathway. The Ras/RAF/MAP2K/MAPK pathway is mainly involved in mediating cell growth, survival and cellular differentiation of insulin. Phosphorylated IRS1 recruits GRB2/SOS complex, which triggers the activation of the Ras/RAF/MAP2K/MAPK pathway. In addition to binding insulin, the insulin receptor can bind insulin-like growth factors (IGFI and IGFII). Isoform Short has a higher affinity for IGFII binding. When present in a hybrid receptor with IGF1R, binds IGF1. PubMed:12138094 shows that hybrid receptors composed of IGF1R and INSR isoform Long are activated with a high affinity by IGF1, with low affinity by IGF2 and not significantly activated by insulin, and that hybrid receptors composed of IGF1R and INSR isoform Short are activated by IGF1, IGF2 and insulin. In contrast, PubMed:16831875 shows that hybrid receptors composed of IGF1R and INSR isoform Long and hybrid receptors composed of IGF1R and INSR isoform Short have similar binding characteristics, both bind IGF1 and have a low affinity for insulin. In adipocytes, inhibits lipolysis (By similarity).   
  
Tetramer of 2 alpha and 2 beta chains linked by disulfide bonds. The alpha chains carry the insulin-binding regions, while the beta chains carry the kinase domain. Forms a hybrid receptor with IGF1R, the hybrid is a tetramer consisting of 1 alpha chain and 1 beta chain of INSR and 1 alpha chain and 1 beta chain of IGF1R. Interacts with SORBS1 but dissociates from it following insulin stimulation. Binds SH2B2. Activated form of INSR interacts (via Tyr-999) with the PTB/PID domains of IRS1 and SHC1. The sequences surrounding the phosphorylated NPXY motif contribute differentially to either IRS1 or SHC1 recognition. Interacts (via tyrosines in the C-terminus) with IRS2 (via PTB domain and 591-786 AA); the 591-786 would be the primary anchor of IRS2 to INSR while the PTB domain would have a stabilizing action on the interaction with INSR. Interacts with the SH2 domains of the 85 kDa regulatory subunit of PI3K (PIK3R1) in vitro, when autophosphorylated on tyrosine residues. Interacts with SOCS7. Interacts (via the phosphorylated Tyr-999), with SOCS3. Interacts (via the phosphorylated Tyr-1185, Tyr-1189, Tyr-1190) with SOCS1. Interacts with CAV2 (tyrosine-phosphorylated form); the interaction is increased with 'Tyr-27'phosphorylation of CAV2 (By similarity). Interacts with ARRB2 (By similarity). Interacts with GRB10; this interaction blocks the association between IRS1/IRS2 and INSR, significantly reduces insulin-stimulated tyrosine phosphorylation of IRS1 and IRS2 and thus decreases insulin signaling. Interacts with GRB7. Interacts with PDPK1. Interacts (via Tyr-1190) with GRB14 (via BPS domain); this interaction protects the tyrosines in the activation loop from dephosphorylation, but promotes dephosphorylation of Tyr-999, this results in decreased interaction with, and phosphorylation of, IRS1. Interacts (via subunit alpha) with ENPP1 (via 485-599 AA); this interaction blocks autophosphorylation. Interacts with PTPRE; this interaction is dependent of Tyr-1185, Tyr-1189 and Tyr-1190 of the INSR. Interacts with STAT5B (via SH2 domain). Interacts with PTPRF. Interacts with ATIC; ATIC together with PRKAA2/AMPK2 and HACD3/PTPLAD1 is proposed to be part of a signaling netwok regulating INSR autophosphorylation and endocytosis (By similarity). Interacts with the cone snail venom insulin Con-Ins G1 (PubMed:27617429). Interacts with the insulin receptor SORL1; this interaction strongly increases its surface exposure, hence strengthens insulin signal reception (PubMed:27322061). Interacts (tyrosine phosphorylated) with CCDC88A/GIV (via SH2-like region); binding requires autophosphorylation of the INSR C-terminal region (PubMed:25187647). Interacts with GNAI3; the interaction is probably mediated by CCDC88A/GIV (PubMed:25187647). Interacts with LMBRD1 (By similarity).   
  
 **Gene Ontology Information:**

Molecular Function

- 1-phosphatidylinositol-3-kinase regulator activity
- amyloid-beta binding
- ATP binding
- cargo receptor activity
- GTP binding
- identical protein binding
- insulin binding
- insulin-activated receptor activity
- insulin receptor substrate binding
- insulin-like growth factor I binding
- insulin-like growth factor II binding
- insulin-like growth factor receptor binding
- phosphatidylinositol 3-kinase binding
- protein domain specific binding
- protein serine/threonine kinase activator activity
- protein tyrosine kinase activator activity
- protein tyrosine kinase activity
- protein-containing complex binding
- PTB domain binding
- structural molecule activity
- transmembrane receptor protein tyrosine kinase activity

Location

- axon
- caveola
- dendrite membrane
- endosome membrane
- external side of plasma membrane
- extracellular exosome
- insulin receptor complex
- late endosome
- lysosome
- membrane
- neuronal cell body membrane
- plasma membrane
- receptor complex

Biological process

- activation of protein kinase activity
- activation of protein kinase B activity
- adrenal gland development
- amyloid-beta clearance
- carbohydrate metabolic process
- cellular response to growth factor stimulus
- cellular response to insulin stimulus
- dendritic spine maintenance
- epidermis development
- exocrine pancreas development
- G protein-coupled receptor signaling pathway
- glucose homeostasis
- heart morphogenesis
- insulin receptor signaling pathway
- learning
- male gonad development
- male sex determination
- memory
- neuron projection maintenance
- peptidyl-tyrosine autophosphorylation
- peptidyl-tyrosine phosphorylation
- positive regulation of cell migration
- positive regulation of cell population proliferation
- positive regulation of developmental growth
- positive regulation of transcription, DNA-templated
- positive regulation of glucose import
- positive regulation of glycogen biosynthetic process
- positive regulation of glycolytic process
- positive regulation of kinase activity
- positive regulation of MAP kinase activity
- positive regulation of MAPK cascade
- positive regulation of meiotic cell cycle
- positive regulation of mitotic nuclear division
- positive regulation of nitric oxide biosynthetic process
- positive regulation of phosphatidylinositol 3-kinase signaling
- positive regulation of protein kinase B signaling
- positive regulation of protein phosphorylation
- positive regulation of protein-containing complex disassembly
- positive regulation of receptor internalization
- positive regulation of respiratory burst
- protein autophosphorylation
- protein phosphorylation
- receptor internalization
- receptor-mediated endocytosis
- regulation of transcription, DNA-templated
- regulation of embryonic development
- regulation of female gonad development
- transmembrane receptor protein tyrosine kinase signaling pathway
- transport across blood-brain barrier
- viral entry into host cell

---

35

- **Protein name:** N/A
- **Organism:** N/A
- **Uniprot Accession Number:** N/A
- **Protein sequence length:** N/A
- **1D identity (%):** N/A
- **1D identity (%) [Gaps excluded]:** N/A
- **1D identity - Alignment Gaps:** N/A
- **1D aligned content (<aminoacid>:%):** N/A
- **Common reported functions (%):** 0.0
- **Common reported locations (%):** 0.0
- **Common reported processes (%):** 0.0

- **PDB ID:** 7SBY
- **Chain:** J
- **Crystallized protein length:** 1171 aa
- **Resolution:** 3.0 Å
- **b-phipsi:** 0.001588
- **w-rdist:** 0.792224
- **t-alpha:** 0.029813
- **Chemical similarity (Tanimoto Index) (%):** 100.0
- **1D identity (%) [PDB]:** 8.21
- **1D identity (%) [Gaps excluded][PDB]:** 59.54
- **1D identity - Alignment Gaps [PDB]:** 1637
- **1D aligned content [PDB] (<aminoacid>:%):** {'V': 7.05, 'F': 6.41, 'A': 10.26, 'K': 5.77, 'Y': 3.21, 'T': 4.49, 'P': 7.05, 'I': 6.41, 'D': 3.21, 'N': 5.77, 'S': 8.33, 'L': 12.82, 'R': 3.85, 'E': 2.56, 'G': 5.13, 'W': 0.64, 'Q': 5.77, 'H': 1.28}
- **2D identity (%) [PDB]:** 60.06
- **2D identity (%) [Gaps excluded][PDB]:** 85.12
- **2D identity - Alignment Gaps [PDB]:** 373
- **2D aligned content [PDB] (<2D-fold>:%):** {'.': 18.92, 'E': 39.03, 'S': 6.44, 'P': 1.58, 'T': 8.94, 'B': 0.26, 'H': 22.6, 'G': 1.58, 'I': 0.66}
- **3D similarity (TM-Score) (%) [PDB]:** N/A

- **Gene name:** N/A
- **Entrez ID:** N/A
- **RefSeq ID:** N/A
- **Sequence length:** N/A
- **5-UTR|CDS|3-UTR identity (%):** N/A | N/A | N/A
- **5-UTR|CDS|3-UTR identity (%) [Gaps excluded]:** N/A | N/A | N/A
- **5-UTR|CDS|3-UTR identity [Alignment Gaps]:** N/A | N/A | N/A
- **5-UTR aligned content (<base>:%):** N/A
- **CDS aligned content (<base>:%):** N/A
- **3-UTR aligned content (<base>:%):** N/A

**Uniprot Description:**  
  
 N/A N/A   
  
 **Gene Ontology Information:**

Molecular Function   
  
N/A

Location   
  
N/A

Biological process   
  
N/A

---
