# Supplementary material for: Identifying and profiling structural similarities between Spike of SARS-CoV-2 and other viral or host proteins with Machaon
Source: Commun Biol. 2023 Jul 19;6:752. doi: 10.1038/s42003-023-05076-7 (PMC10356814; doi:10.1038/s42003-023-05076-7)
Supplement: Supplementary file 8 — Supplementary Data 5 [file 42003_2023_5076_MOESM8_ESM.zip › 6VXX_A_whole_human_exp_dataset/candidates/6VXX_A-merged-notenriched_report.html]

 

# Structural Comparison Report for 6VXX\_A - whole structures (total: 586)

---

1

- **PDB ID:** 7JV6 | **Chain:** B
- **b-phipsi:** 1.9828060446155213e-05
- **w-rdist:** 0.1619863176794701
- **t-alpha:** 0.0134114913151814

---

---

2

- **PDB ID:** 7ND8 | **Chain:** C
- **b-phipsi:** 0.0002089660885967
- **w-rdist:** 0.3207916877348661
- **t-alpha:** 0.0049877163597098

---

---

3

- **PDB ID:** 7L2E | **Chain:** A
- **b-phipsi:** 0.0004687328818433
- **w-rdist:** 0.2828015349891228
- **t-alpha:** 0.0016569498401739

---

---

4

- **PDB ID:** 7K8Z | **Chain:** A
- **b-phipsi:** 0.0005902213632381
- **w-rdist:** 0.1436010258764374
- **t-alpha:** 0.0024878881015579

---

---

5

- **PDB ID:** 6WPS | **Chain:** B
- **b-phipsi:** 0.0004107374272846
- **w-rdist:** 0.2874778239915183
- **t-alpha:** 0.0041355535019986

---

---

6

- **PDB ID:** 6VXX | **Chain:** B
- **b-phipsi:** 5.894373919306382e-09
- **w-rdist:** 7.617386263618201e-05
- **t-alpha:** 0.0254452761871732

---

---

7

- **PDB ID:** 7M6E | **Chain:** E
- **b-phipsi:** 0.0004587074219777
- **w-rdist:** 0.3066311555315696
- **t-alpha:** 0.0033085120789013

---

---

8

- **PDB ID:** 7LS9 | **Chain:** C
- **b-phipsi:** 0.0004931557336508
- **w-rdist:** 0.2738241680185225
- **t-alpha:** 0.0058238157721357

---

---

9

- **PDB ID:** 7M6G | **Chain:** B
- **b-phipsi:** 0.0007999114174453
- **w-rdist:** 0.208287152360664
- **t-alpha:** 0.0008271775551371

---

---

10

- **PDB ID:** 7E7D | **Chain:** C
- **b-phipsi:** 0.00018824637014
- **w-rdist:** 0.3412584883583441
- **t-alpha:** 0.010752376359526

---

---

11

- **PDB ID:** 7K43 | **Chain:** A
- **b-phipsi:** 0.0002449305946686
- **w-rdist:** 0.3597386342446795
- **t-alpha:** 0.0074438168005386

---

---

12

- **PDB ID:** 7NDB | **Chain:** C
- **b-phipsi:** 0.000700188306123
- **w-rdist:** 0.2558497714596573
- **t-alpha:** 0.0041527907791232

---

---

13

- **PDB ID:** 7JV4 | **Chain:** C
- **b-phipsi:** 0.0002894637019574
- **w-rdist:** 0.1135149754813402
- **t-alpha:** 0.0228428607189075

---

---

14

- **PDB ID:** 7KS9 | **Chain:** C
- **b-phipsi:** 0.0001192786928561
- **w-rdist:** 0.3990506186176142
- **t-alpha:** 0.0108695004677259

---

---

15

- **PDB ID:** 7K43 | **Chain:** E
- **b-phipsi:** 0.0002448353823325
- **w-rdist:** 0.3597379628907657
- **t-alpha:** 0.0115797655430185

---

---

16

- **PDB ID:** 6WPT | **Chain:** A
- **b-phipsi:** 0.0001907112906
- **w-rdist:** 0.3365624349288467
- **t-alpha:** 0.017676751760596

---

---

17

- **PDB ID:** 7R8O | **Chain:** A
- **b-phipsi:** 0.0001123722627207
- **w-rdist:** 0.3523138564716368
- **t-alpha:** 0.0190237705806923

---

---

18

- **PDB ID:** 7KNB | **Chain:** A
- **b-phipsi:** 0.0004452962942296
- **w-rdist:** 0.3749379654064083
- **t-alpha:** 0.0066610907906692

---

---

19

- **PDB ID:** 7Q9I | **Chain:** A
- **b-phipsi:** 0.0001054739761345
- **w-rdist:** 0.5211813559787952
- **t-alpha:** 0.0049877163597098

---

---

20

- **PDB ID:** 6WPS | **Chain:** E
- **b-phipsi:** 0.0004101899653639
- **w-rdist:** 0.2874749563492629
- **t-alpha:** 0.017369624679081

---

---

21

- **PDB ID:** 7ND8 | **Chain:** A
- **b-phipsi:** 3.60737070527693e-05
- **w-rdist:** 0.3713102441789194
- **t-alpha:** 0.0228428607189075

---

---

22

- **PDB ID:** 7Q9G | **Chain:** C
- **b-phipsi:** 0.000242648253847
- **w-rdist:** 0.4895986980570274
- **t-alpha:** 0.0049627974633414

---

---

23

- **PDB ID:** 7ND7 | **Chain:** B
- **b-phipsi:** 0.0002824681076567
- **w-rdist:** 0.3681674282196736
- **t-alpha:** 0.0165423471063261

---

---

24

- **PDB ID:** 7S0C | **Chain:** B
- **b-phipsi:** 7.411468150874427e-05
- **w-rdist:** 0.524745966844762
- **t-alpha:** 0.0066610907906692

---

---

25

- **PDB ID:** 7WLZ | **Chain:** A
- **b-phipsi:** 0.0005810653681752
- **w-rdist:** 0.2416858267449977
- **t-alpha:** 0.0151134131271917

---

---

26

- **PDB ID:** 7K8T | **Chain:** A
- **b-phipsi:** 0.0004510995768821
- **w-rdist:** 0.3845682369247561
- **t-alpha:** 0.0091819121329137

---

---

27

- **PDB ID:** 7LY2 | **Chain:** J
- **b-phipsi:** 0.0001862129708138
- **w-rdist:** 0.4821381889272673
- **t-alpha:** 0.0083406172007201

---

---

28

- **PDB ID:** 7KS9 | **Chain:** A
- **b-phipsi:** 0.0004104577390169
- **w-rdist:** 0.378726512251663
- **t-alpha:** 0.012562807806465

---

---

29

- **PDB ID:** 7L2E | **Chain:** B
- **b-phipsi:** 0.0004401332718291
- **w-rdist:** 0.2826707542392753
- **t-alpha:** 0.0237089470211593

---

---

30

- **PDB ID:** 7RW2 | **Chain:** C
- **b-phipsi:** 0.0007143805576073
- **w-rdist:** 0.3121610988793534
- **t-alpha:** 0.0100253538396266

---

---

31

- **PDB ID:** 7M6E | **Chain:** A
- **b-phipsi:** 0.000527104422087
- **w-rdist:** 0.3068347246289138
- **t-alpha:** 0.017676751760596

---

---

32

- **PDB ID:** 7K8W | **Chain:** A
- **b-phipsi:** 0.0014582952101854
- **w-rdist:** 0.2592120652671683
- **t-alpha:** 0.0016569498401739

---

---

33

- **PDB ID:** 7K8X | **Chain:** A
- **b-phipsi:** 0.0005208874707231
- **w-rdist:** 0.4459059432893972
- **t-alpha:** 0.0049877163597098

---

---

34

- **PDB ID:** 7K8Z | **Chain:** C
- **b-phipsi:** 0.0008298856317738
- **w-rdist:** 0.0880111459451454
- **t-alpha:** 0.0181966044744523

---

---

35

- **PDB ID:** 7Q0A | **Chain:** A
- **b-phipsi:** 0.0008860507097144
- **w-rdist:** 0.329273791461733
- **t-alpha:** 0.0083406172007201

---

---

36

- **PDB ID:** 7ND3 | **Chain:** C
- **b-phipsi:** 0.0005476616949015
- **w-rdist:** 0.4173818830320326
- **t-alpha:** 0.0083406172007201

---

---

37

- **PDB ID:** 7K8V | **Chain:** C
- **b-phipsi:** 0.001996083434591
- **w-rdist:** 0.1872050171687379
- **t-alpha:** 0.0024878881015579

---

---

38

- **PDB ID:** 7LXY | **Chain:** B
- **b-phipsi:** 0.0001565174590749
- **w-rdist:** 0.4313745645787316
- **t-alpha:** 0.0231594461372635

---

---

39

- **PDB ID:** 7K8S | **Chain:** A
- **b-phipsi:** 0.0005765318507878
- **w-rdist:** 0.424802954294296
- **t-alpha:** 0.0075000236060698

---

---

40

- **PDB ID:** 7NDA | **Chain:** A
- **b-phipsi:** 0.0005397081053465
- **w-rdist:** 0.325792983659764
- **t-alpha:** 0.0237089470211593

---

---

41

- **PDB ID:** 7S6L | **Chain:** B
- **b-phipsi:** 0.0005404817067831
- **w-rdist:** 0.181445718712477
- **t-alpha:** 0.0298127438669044

---

---

42

- **PDB ID:** 7M6G | **Chain:** A
- **b-phipsi:** 0.0001644638466818
- **w-rdist:** 0.4082270499131745
- **t-alpha:** 0.026468223205744

---

---

43

- **PDB ID:** 7KJ3 | **Chain:** C
- **b-phipsi:** 0.0001351762221393
- **w-rdist:** 0.0948536174734534
- **t-alpha:** 0.0545905224299128

---

---

44

- **PDB ID:** 7R8O | **Chain:** E
- **b-phipsi:** 8.128392331868429e-05
- **w-rdist:** 0.3504062127900773
- **t-alpha:** 0.0388749924124196

---

---

45

- **PDB ID:** 7LS9 | **Chain:** A
- **b-phipsi:** 0.0006478008030638
- **w-rdist:** 0.2741722728601115
- **t-alpha:** 0.0263160129948751

---

---

46

- **PDB ID:** 7MW2 | **Chain:** A
- **b-phipsi:** 0.0010945034935917
- **w-rdist:** 0.3955537134349211
- **t-alpha:** 0.0033085120789013

---

---

47

- **PDB ID:** 7RA8 | **Chain:** B
- **b-phipsi:** 3.1581708843822325e-05
- **w-rdist:** 0.6289722139200175
- **t-alpha:** 0.010752376359526

---

---

48

- **PDB ID:** 7LXZ | **Chain:** B
- **b-phipsi:** 7.379895139722556e-05
- **w-rdist:** 0.5279736606168327
- **t-alpha:** 0.0202533741750159

---

---

49

- **PDB ID:** 6NB4 | **Chain:** C
- **b-phipsi:** 0.0010681136584744
- **w-rdist:** 0.4208094940046027
- **t-alpha:** 0.0016540840807526

---

---

50

- **PDB ID:** 7LXY | **Chain:** J
- **b-phipsi:** 0.000156547451807
- **w-rdist:** 0.4313728344117633
- **t-alpha:** 0.0280611759977658

---

---

51

- **PDB ID:** 6VXX | **Chain:** C
- **b-phipsi:** 2.204205180302208e-08
- **w-rdist:** 7.653592189866652e-05
- **t-alpha:** 0.0689655430268945

---

---

52

- **PDB ID:** 7ND5 | **Chain:** C
- **b-phipsi:** 0.0005592086458954
- **w-rdist:** 0.4188260081058901
- **t-alpha:** 0.0140611636219507

---

---

53

- **PDB ID:** 7ND3 | **Chain:** A
- **b-phipsi:** 0.0008825631460148
- **w-rdist:** 0.3457551197908364
- **t-alpha:** 0.0142618534263445

---

---

54

- **PDB ID:** 7M6E | **Chain:** B
- **b-phipsi:** 0.0005230889143817
- **w-rdist:** 0.3067534970758635
- **t-alpha:** 0.0289493438129448

---

---

55

- **PDB ID:** 7S6L | **Chain:** C
- **b-phipsi:** 0.00166592525167
- **w-rdist:** 0.2125624533348116
- **t-alpha:** 0.0091819121329137

---

---

56

- **PDB ID:** 7KXK | **Chain:** C
- **b-phipsi:** 0.0009131843187079
- **w-rdist:** 0.2659455485028394
- **t-alpha:** 0.0215054061986588

---

---

57

- **PDB ID:** 7Q9M | **Chain:** B
- **b-phipsi:** 0.0006416313459877
- **w-rdist:** 0.370286640792962
- **t-alpha:** 0.0190237705806923

---

---

58

- **PDB ID:** 7R8O | **Chain:** B
- **b-phipsi:** 0.0001026932461644
- **w-rdist:** 0.3509000988326368
- **t-alpha:** 0.0446646519250406

---

---

59

- **PDB ID:** 7KMK | **Chain:** C
- **b-phipsi:** 0.0006857469950691
- **w-rdist:** 0.2887092946692354
- **t-alpha:** 0.026468223205744

---

---

60

- **PDB ID:** 7JV6 | **Chain:** A
- **b-phipsi:** 1.977862813553842e-05
- **w-rdist:** 0.161989966168585
- **t-alpha:** 0.0680214444040752

---

---

61

- **PDB ID:** 7K4N | **Chain:** E
- **b-phipsi:** 0.0001644581135374
- **w-rdist:** 0.7860326818988504
- **t-alpha:** 0.0016540840807526

---

---

62

- **PDB ID:** 7K8S | **Chain:** C
- **b-phipsi:** 0.0005088002786227
- **w-rdist:** 0.4530615281970078
- **t-alpha:** 0.0140611636219507

---

---

63

- **PDB ID:** 7M6H | **Chain:** B
- **b-phipsi:** 0.0008039492154596
- **w-rdist:** 0.2623331859884831
- **t-alpha:** 0.0271879789729934

---

---

64

- **PDB ID:** 7MW2 | **Chain:** B
- **b-phipsi:** 0.0006876520627291
- **w-rdist:** 0.3940233187895655
- **t-alpha:** 0.0185340926063368

---

---

65

- **PDB ID:** 7L2D | **Chain:** C
- **b-phipsi:** 0.0016654199630195
- **w-rdist:** 0.2969143337519753
- **t-alpha:** 0.0091819121329137

---

---

66

- **PDB ID:** 7R8N | **Chain:** E
- **b-phipsi:** 0.0009418371687485
- **w-rdist:** 0.3468302739989374
- **t-alpha:** 0.0165423471063261

---

---

67

- **PDB ID:** 7L02 | **Chain:** B
- **b-phipsi:** 6.5094149286774415e-06
- **w-rdist:** 0.0009575192775314
- **t-alpha:** 0.0813954771139466

---

---

68

- **PDB ID:** 7L09 | **Chain:** B
- **b-phipsi:** 6.543515863664246e-06
- **w-rdist:** 0.0009518696956254
- **t-alpha:** 0.082363784576418

---

---

69

- **PDB ID:** 7KNE | **Chain:** C
- **b-phipsi:** 0.0010274684252118
- **w-rdist:** 0.3583579456633973
- **t-alpha:** 0.014888357714047

---

---

70

- **PDB ID:** 7E7B | **Chain:** C
- **b-phipsi:** 0.0003357988906735
- **w-rdist:** 0.528468485799406
- **t-alpha:** 0.0190237705806923

---

---

71

- **PDB ID:** 7K43 | **Chain:** B
- **b-phipsi:** 0.0002451225616081
- **w-rdist:** 0.3597373316601878
- **t-alpha:** 0.0446646519250406

---

---

72

- **PDB ID:** 7L2D | **Chain:** A
- **b-phipsi:** 0.000853814113006
- **w-rdist:** 0.3462066696079995
- **t-alpha:** 0.0237089470211593

---

---

73

- **PDB ID:** 7Q6E | **Chain:** A
- **b-phipsi:** 0.0003742840252866
- **w-rdist:** 0.3740790121329381
- **t-alpha:** 0.0351026483066376

---

---

74

- **PDB ID:** 7Q9I | **Chain:** B
- **b-phipsi:** 0.0002693045526576
- **w-rdist:** 0.7934594465378608
- **t-alpha:** 0.0008271775551371

---

---

75

- **PDB ID:** 7BYR | **Chain:** C
- **b-phipsi:** 0.0044872081212143
- **w-rdist:** 0.1457062152770529
- **t-alpha:** 0.0058238157721357

---

---

76

- **PDB ID:** 6WPT | **Chain:** C
- **b-phipsi:** 0.0001032780266557
- **w-rdist:** 0.2351546496421769
- **t-alpha:** 0.0708593084227287

---

---

77

- **PDB ID:** 7M6I | **Chain:** C
- **b-phipsi:** 0.0016776932930549
- **w-rdist:** 0.3102152628772477
- **t-alpha:** 0.0108695004677259

---

---

78

- **PDB ID:** 7WLY | **Chain:** A
- **b-phipsi:** 0.0011829912983277
- **w-rdist:** 0.427003333422655
- **t-alpha:** 0.0058238157721357

---

---

79

- **PDB ID:** 7L06 | **Chain:** C
- **b-phipsi:** 3.078770769148547e-07
- **w-rdist:** 0.0007676045396418
- **t-alpha:** 0.1224152168765049

---

---

80

- **PDB ID:** 6NB6 | **Chain:** A
- **b-phipsi:** 0.0010584179234524
- **w-rdist:** 0.5120995532788646
- **t-alpha:** 0.0024811957952362

---

---

81

- **PDB ID:** 7N8H | **Chain:** K
- **b-phipsi:** 0.0003973726273375
- **w-rdist:** 0.4454994797196821
- **t-alpha:** 0.0280611759977658

---

---

82

- **PDB ID:** 7KJ2 | **Chain:** C
- **b-phipsi:** 0.0008017616143762
- **w-rdist:** 0.0701014838811584
- **t-alpha:** 0.0397021010306442

---

---

83

- **PDB ID:** 7L02 | **Chain:** C
- **b-phipsi:** 2.766161158607103e-07
- **w-rdist:** 0.0010986050468293
- **t-alpha:** 0.1613834803230558

---

---

84

- **PDB ID:** 7L09 | **Chain:** C
- **b-phipsi:** 2.693811224331148e-07
- **w-rdist:** 0.0011022231101016
- **t-alpha:** 0.1613834803230558

---

---

85

- **PDB ID:** 7KJ2 | **Chain:** A
- **b-phipsi:** 0.0002023633610506
- **w-rdist:** 0.078040551930263
- **t-alpha:** 0.077540150779898

---

---

86

- **PDB ID:** 7LS9 | **Chain:** B
- **b-phipsi:** 0.0009529878005222
- **w-rdist:** 0.274347639663284
- **t-alpha:** 0.0280611759977658

---

---

87

- **PDB ID:** 7Q9P | **Chain:** C
- **b-phipsi:** 0.0007128156491745
- **w-rdist:** 0.3608557191895086
- **t-alpha:** 0.0271879789729934

---

---

88

- **PDB ID:** 7L2D | **Chain:** B
- **b-phipsi:** 0.0008611593101101
- **w-rdist:** 0.2968369267479988
- **t-alpha:** 0.0298127438669044

---

---

89

- **PDB ID:** 6WPS | **Chain:** A
- **b-phipsi:** 0.0004105064002951
- **w-rdist:** 0.2874745355703215
- **t-alpha:** 0.0513043822350767

---

---

90

- **PDB ID:** 7JV6 | **Chain:** E
- **b-phipsi:** 1.9862282340721987e-05
- **w-rdist:** 0.1619903112556173
- **t-alpha:** 0.1000913049362774

---

---

91

- **PDB ID:** 7K4N | **Chain:** B
- **b-phipsi:** 0.0001645367358476
- **w-rdist:** 0.7860340133266828
- **t-alpha:** 0.0066610907906692

---

---

92

- **PDB ID:** 7ND8 | **Chain:** B
- **b-phipsi:** 0.0001571394277919
- **w-rdist:** 0.3340526369212543
- **t-alpha:** 0.0623900411658351

---

---

93

- **PDB ID:** 7ND7 | **Chain:** C
- **b-phipsi:** 0.0001963356938378
- **w-rdist:** 0.3704033958510991
- **t-alpha:** 0.0540540988034132

---

---

94

- **PDB ID:** 7CAC | **Chain:** A
- **b-phipsi:** 0.0018540111686423
- **w-rdist:** 0.3993865257150472
- **t-alpha:** 0.0066169305252397

---

---

95

- **PDB ID:** 7KL9 | **Chain:** B
- **b-phipsi:** 0.0084581971181665
- **w-rdist:** 0.2702614294113635
- **t-alpha:** 0.0033085120789013

---

---

96

- **PDB ID:** 7E7D | **Chain:** B
- **b-phipsi:** 0.0003278725963079
- **w-rdist:** 0.3443901086820769
- **t-alpha:** 0.0554175614600527

---

---

97

- **PDB ID:** 7SO9 | **Chain:** K
- **b-phipsi:** 0.0002414924515102
- **w-rdist:** 0.5671606495895675
- **t-alpha:** 0.026468223205744

---

---

98

- **PDB ID:** 7M6I | **Chain:** A
- **b-phipsi:** 0.000803623532548
- **w-rdist:** 0.2873909609837611
- **t-alpha:** 0.0363936588404032

---

---

99

- **PDB ID:** 7S0C | **Chain:** A
- **b-phipsi:** 0.0023227477585591
- **w-rdist:** 0.3023280149888626
- **t-alpha:** 0.0140611636219507

---

---

100

- **PDB ID:** 7ND5 | **Chain:** A
- **b-phipsi:** 0.0008851607700634
- **w-rdist:** 0.3292765964699219
- **t-alpha:** 0.0306906295197915

---

---

101

- **PDB ID:** 7SN3 | **Chain:** A
- **b-phipsi:** 0.0018242520042948
- **w-rdist:** 0.3579241379899047
- **t-alpha:** 0.012562807806465

---

---

102

- **PDB ID:** 7Q0A | **Chain:** C
- **b-phipsi:** 0.0005592297809898
- **w-rdist:** 0.418823765065609
- **t-alpha:** 0.0298127438669044

---

---

103

- **PDB ID:** 7ND6 | **Chain:** C
- **b-phipsi:** 0.0005595328346514
- **w-rdist:** 0.4188258454238287
- **t-alpha:** 0.030603642719128

---

---

104

- **PDB ID:** 7KXJ | **Chain:** C
- **b-phipsi:** 0.0012131549884823
- **w-rdist:** 0.4027443989674221
- **t-alpha:** 0.0142618534263445

---

---

105

- **PDB ID:** 7THT | **Chain:** C
- **b-phipsi:** 0.0005247886724678
- **w-rdist:** 0.2323446129345116
- **t-alpha:** 0.0570720736347449

---

---

106

- **PDB ID:** 7L2E | **Chain:** C
- **b-phipsi:** 0.0004585238199694
- **w-rdist:** 0.2832143772023781
- **t-alpha:** 0.0577426744115672

---

---

107

- **PDB ID:** 7ENF | **Chain:** C
- **b-phipsi:** 0.0017146175799969
- **w-rdist:** 0.2744791809003384
- **t-alpha:** 0.0245762552329136

---

---

108

- **PDB ID:** 7JV4 | **Chain:** A
- **b-phipsi:** 0.0003465786903133
- **w-rdist:** 0.1726675123160092
- **t-alpha:** 0.0794645485926308

---

---

109

- **PDB ID:** 7L56 | **Chain:** B
- **b-phipsi:** 0.0027952908078968
- **w-rdist:** 0.3837472408911725
- **t-alpha:** 0.0066610907906692

---

---

110

- **PDB ID:** 7NY5 | **Chain:** E
- **b-phipsi:** 0.0004087460688524
- **w-rdist:** 0.813591050619514
- **t-alpha:** 0.0008281320187666

---

---

111

- **PDB ID:** 7R8M | **Chain:** B
- **b-phipsi:** 0.0039805546619842
- **w-rdist:** 0.1862937491852379
- **t-alpha:** 0.0157152579062525

---

---

112

- **PDB ID:** 7ND9 | **Chain:** A
- **b-phipsi:** 0.000996824953155
- **w-rdist:** 0.2005589666034246
- **t-alpha:** 0.0377684208359867

---

---

113

- **PDB ID:** 7LXZ | **Chain:** K
- **b-phipsi:** 6.514935649202147e-05
- **w-rdist:** 0.5279739924753565
- **t-alpha:** 0.0422415493952179

---

---

114

- **PDB ID:** 6XEY | **Chain:** A
- **b-phipsi:** 0.0013397990378154
- **w-rdist:** 0.485591367819386
- **t-alpha:** 0.0066610907906692

---

---

115

- **PDB ID:** 5I5K | **Chain:** A
- **b-phipsi:** 0.0043536099130699
- **w-rdist:** 0.342823563543469
- **t-alpha:** 0.0074438168005386

---

---

116

- **PDB ID:** 7S0D | **Chain:** C
- **b-phipsi:** 0.0009383697359432
- **w-rdist:** 0.1921592325688822
- **t-alpha:** 0.0430106603185429

---

---

117

- **PDB ID:** 7RW2 | **Chain:** A
- **b-phipsi:** 0.0007214910967154
- **w-rdist:** 0.3113319037756883
- **t-alpha:** 0.0446646519250406

---

---

118

- **PDB ID:** 7CHH | **Chain:** B
- **b-phipsi:** 0.0014323681508637
- **w-rdist:** 0.3606001934071889
- **t-alpha:** 0.0215054061986588

---

---

119

- **PDB ID:** 7P40 | **Chain:** E
- **b-phipsi:** 0.0004167707876055
- **w-rdist:** 0.8101882344403887
- **t-alpha:** 0.0024878881015579

---

---

120

- **PDB ID:** 7KJ4 | **Chain:** A
- **b-phipsi:** 0.0005521493162268
- **w-rdist:** 0.7769271040664747
- **t-alpha:** 0.0041527907791232

---

---

121

- **PDB ID:** 7S6L | **Chain:** A
- **b-phipsi:** 0.0001945111571395
- **w-rdist:** 0.820559994710997
- **t-alpha:** 0.0066610907906692

---

---

122

- **PDB ID:** 7KNH | **Chain:** C
- **b-phipsi:** 0.0038670727767929
- **w-rdist:** 0.351911283348928
- **t-alpha:** 0.0091819121329137

---

---

123

- **PDB ID:** 7RW2 | **Chain:** B
- **b-phipsi:** 0.0007389233415224
- **w-rdist:** 0.3119631975790221
- **t-alpha:** 0.0458478938296924

---

---

124

- **PDB ID:** 7V2A | **Chain:** C
- **b-phipsi:** 0.0043607750936307
- **w-rdist:** 0.2865351776723294
- **t-alpha:** 0.0124068665100041

---

---

125

- **PDB ID:** 7Q6E | **Chain:** B
- **b-phipsi:** 0.0004302786529525
- **w-rdist:** 0.4357611742589435
- **t-alpha:** 0.040447508308947

---

---

126

- **PDB ID:** 6NB6 | **Chain:** C
- **b-phipsi:** 0.0008173237482404
- **w-rdist:** 0.7352956446779483
- **t-alpha:** 0.0024811957952362

---

---

127

- **PDB ID:** 7TAT | **Chain:** A
- **b-phipsi:** 0.0020781207906571
- **w-rdist:** 0.3735840605839423
- **t-alpha:** 0.014888357714047

---

---

128

- **PDB ID:** 7CHH | **Chain:** C
- **b-phipsi:** 0.0005682419588058
- **w-rdist:** 0.4427193074651996
- **t-alpha:** 0.0333332138395487

---

---

129

- **PDB ID:** 7NDA | **Chain:** B
- **b-phipsi:** 0.0003011321574895
- **w-rdist:** 0.2848686529408433
- **t-alpha:** 0.0911551233030605

---

---

130

- **PDB ID:** 7Q9M | **Chain:** C
- **b-phipsi:** 0.0006101849672181
- **w-rdist:** 0.3615906606496673
- **t-alpha:** 0.0467534245579859

---

---

131

- **PDB ID:** 7LD1 | **Chain:** C
- **b-phipsi:** 0.0010076270573324
- **w-rdist:** 0.5470690634396651
- **t-alpha:** 0.010752376359526

---

---

132

- **PDB ID:** 7SBW | **Chain:** A
- **b-phipsi:** 0.0003568226726397
- **w-rdist:** 0.8000464722842542
- **t-alpha:** 0.0083406172007201

---

---

133

- **PDB ID:** 7JWB | **Chain:** A
- **b-phipsi:** 0.0001249612555758
- **w-rdist:** 0.5277843400227087
- **t-alpha:** 0.0467534245579859

---

---

134

- **PDB ID:** 7S6K | **Chain:** A
- **b-phipsi:** 0.0003746630868982
- **w-rdist:** 0.6513283372488442
- **t-alpha:** 0.0245762552329136

---

---

135

- **PDB ID:** 7N8H | **Chain:** F
- **b-phipsi:** 0.0003971164755464
- **w-rdist:** 0.4454998111876727
- **t-alpha:** 0.0463192707105852

---

---

136

- **PDB ID:** 7K90 | **Chain:** B
- **b-phipsi:** 0.0020975032942123
- **w-rdist:** 0.3314801917699287
- **t-alpha:** 0.0239867377672513

---

---

137

- **PDB ID:** 7TM0 | **Chain:** A
- **b-phipsi:** 0.0003075742631325
- **w-rdist:** 0.5582380396145925
- **t-alpha:** 0.0359899834238583

---

---

138

- **PDB ID:** 7LJR | **Chain:** B
- **b-phipsi:** 0.000932412479556
- **w-rdist:** 0.2618635926866953
- **t-alpha:** 0.0494792275614968

---

---

139

- **PDB ID:** 7M6H | **Chain:** A
- **b-phipsi:** 0.0023535702002393
- **w-rdist:** 0.2784560139621441
- **t-alpha:** 0.0263160129948751

---

---

140

- **PDB ID:** 7ND6 | **Chain:** A
- **b-phipsi:** 0.0008858848517644
- **w-rdist:** 0.3292707999747721
- **t-alpha:** 0.0458478938296924

---

---

141

- **PDB ID:** 7LSS | **Chain:** B
- **b-phipsi:** 0.0022575049361005
- **w-rdist:** 0.2976700964961644
- **t-alpha:** 0.0263160129948751

---

---

142

- **PDB ID:** 7M6G | **Chain:** C
- **b-phipsi:** 0.0015290645784621
- **w-rdist:** 0.22710807844925
- **t-alpha:** 0.0368785736255408

---

---

143

- **PDB ID:** 7LY2 | **Chain:** B
- **b-phipsi:** 0.0001863156451751
- **w-rdist:** 0.4821386112277945
- **t-alpha:** 0.0545905224299128

---

---

144

- **PDB ID:** 7S0D | **Chain:** B
- **b-phipsi:** 0.0011265109951953
- **w-rdist:** 0.2623524627092954
- **t-alpha:** 0.0440416709650406

---

---

145

- **PDB ID:** 7Q9F | **Chain:** C
- **b-phipsi:** 0.0013890229569915
- **w-rdist:** 0.4517448673264408
- **t-alpha:** 0.0142618534263445

---

---

146

- **PDB ID:** 7NDC | **Chain:** C
- **b-phipsi:** 0.0003043033892372
- **w-rdist:** 0.5998235367786995
- **t-alpha:** 0.0333332138395487

---

---

147

- **PDB ID:** 7R8N | **Chain:** B
- **b-phipsi:** 0.0008003510586586
- **w-rdist:** 0.3445909581781683
- **t-alpha:** 0.048568838795896

---

---

148

- **PDB ID:** 7NDA | **Chain:** C
- **b-phipsi:** 0.0004022263910436
- **w-rdist:** 0.2980278276635238
- **t-alpha:** 0.0852785698448104

---

---

149

- **PDB ID:** 7E7B | **Chain:** B
- **b-phipsi:** 0.000342110221502
- **w-rdist:** 0.5301495549012387
- **t-alpha:** 0.0380480467994648

---

---

150

- **PDB ID:** 7K8V | **Chain:** A
- **b-phipsi:** 0.0017837108782501
- **w-rdist:** 0.1858537331951237
- **t-alpha:** 0.0359899834238583

---

---

151

- **PDB ID:** 7V2A | **Chain:** A
- **b-phipsi:** 0.0044483097379111
- **w-rdist:** 0.286500147317179
- **t-alpha:** 0.0185340926063368

---

---

152

- **PDB ID:** 7XOD | **Chain:** C
- **b-phipsi:** 0.0008880915543032
- **w-rdist:** 0.4941425770431713
- **t-alpha:** 0.0237089470211593

---

---

153

- **PDB ID:** 7ND9 | **Chain:** C
- **b-phipsi:** 0.0009274188466022
- **w-rdist:** 0.237089687478418
- **t-alpha:** 0.0545905224299128

---

---

154

- **PDB ID:** 7LAB | **Chain:** C
- **b-phipsi:** 0.0004689403611927
- **w-rdist:** 0.0811830776120461
- **t-alpha:** 0.164598802026713

---

---

155

- **PDB ID:** 7E7D | **Chain:** A
- **b-phipsi:** 0.0003032929589663
- **w-rdist:** 0.3446354810847326
- **t-alpha:** 0.0918114230686562

---

---

156

- **PDB ID:** 7K8Z | **Chain:** B
- **b-phipsi:** 0.0010307996608251
- **w-rdist:** 0.5094246561883289
- **t-alpha:** 0.0193931278866674

---

---

157

- **PDB ID:** 7ND6 | **Chain:** B
- **b-phipsi:** 0.000825783285585
- **w-rdist:** 0.7820749439177468
- **t-alpha:** 0.0033195156791741

---

---

158

- **PDB ID:** 7LQW | **Chain:** B
- **b-phipsi:** 0.0033477305859989
- **w-rdist:** 0.3340151081381352
- **t-alpha:** 0.0223323354003182

---

---

159

- **PDB ID:** 7L09 | **Chain:** A
- **b-phipsi:** 0.0005151851034698
- **w-rdist:** 0.0344858892138911
- **t-alpha:** 0.2018198347105269

---

---

160

- **PDB ID:** 7WS9 | **Chain:** B
- **b-phipsi:** 0.0005901061767009
- **w-rdist:** 0.613247029149771
- **t-alpha:** 0.0245762552329136

---

---

161

- **PDB ID:** 7LAB | **Chain:** B
- **b-phipsi:** 0.0004793794480094
- **w-rdist:** 0.1075905807426241
- **t-alpha:** 0.2125720663506898

---

---

162

- **PDB ID:** 4A5W | **Chain:** A
- **b-phipsi:** 0.0018938062087036
- **w-rdist:** 0.5565785962631171
- **t-alpha:** 0.0058238157721357

---

---

163

- **PDB ID:** 7FJN | **Chain:** C
- **b-phipsi:** 0.0018188050509104
- **w-rdist:** 0.4363806758510484
- **t-alpha:** 0.0159666566861913

---

---

164

- **PDB ID:** 7Q9K | **Chain:** A
- **b-phipsi:** 0.0003300256463409
- **w-rdist:** 0.409533844256617
- **t-alpha:** 0.064260485408695

---

---

165

- **PDB ID:** 7K8X | **Chain:** B
- **b-phipsi:** 0.0001879543526488
- **w-rdist:** 0.4569281194779326
- **t-alpha:** 0.061457317504612

---

---

166

- **PDB ID:** 7L06 | **Chain:** B
- **b-phipsi:** 0.0005397602551655
- **w-rdist:** 0.0345306561132186
- **t-alpha:** 0.162117484981902

---

---

167

- **PDB ID:** 6ACK | **Chain:** A
- **b-phipsi:** 0.0130043464453334
- **w-rdist:** 0.3378569375689125
- **t-alpha:** 0.0075000236060698

---

---

168

- **PDB ID:** 7WPA | **Chain:** B
- **b-phipsi:** 0.0030878994461518
- **w-rdist:** 0.3856649805938991
- **t-alpha:** 0.0168211022886111

---

---

169

- **PDB ID:** 7KMK | **Chain:** B
- **b-phipsi:** 0.0009277953861147
- **w-rdist:** 0.74406770287384
- **t-alpha:** 0.0049877163597098

---

---

170

- **PDB ID:** 7L02 | **Chain:** A
- **b-phipsi:** 0.0005379648111835
- **w-rdist:** 0.0345297479965273
- **t-alpha:** 0.2043009301723122

---

---

171

- **PDB ID:** 7ND7 | **Chain:** A
- **b-phipsi:** 0.0001950924957195
- **w-rdist:** 0.3693035705083682
- **t-alpha:** 0.1091741848281244

---

---

172

- **PDB ID:** 7R8N | **Chain:** A
- **b-phipsi:** 0.0011198566666698
- **w-rdist:** 0.3454670446223923
- **t-alpha:** 0.0388749924124196

---

---

173

- **PDB ID:** 7THT | **Chain:** S
- **b-phipsi:** 0.00088063508835
- **w-rdist:** 0.2954198272253244
- **t-alpha:** 0.0568180623319403

---

---

174

- **PDB ID:** 7ND3 | **Chain:** B
- **b-phipsi:** 0.0008203117026062
- **w-rdist:** 0.7908491199615817
- **t-alpha:** 0.0041355535019986

---

---

175

- **PDB ID:** 7L06 | **Chain:** A
- **b-phipsi:** 0.000545453354926
- **w-rdist:** 0.0350728304601037
- **t-alpha:** 0.2167080260661611

---

---

176

- **PDB ID:** 7MW4 | **Chain:** A
- **b-phipsi:** 0.0011656350680449
- **w-rdist:** 0.3880254381974524
- **t-alpha:** 0.0322581402352546

---

---

177

- **PDB ID:** 7N8H | **Chain:** A
- **b-phipsi:** 0.0003975063836057
- **w-rdist:** 0.4455008269509126
- **t-alpha:** 0.0568180623319403

---

---

178

- **PDB ID:** 7LXY | **Chain:** A
- **b-phipsi:** 0.0001564091843139
- **w-rdist:** 0.4313741717670274
- **t-alpha:** 0.0813954771139466

---

---

179

- **PDB ID:** 7SN3 | **Chain:** B
- **b-phipsi:** 0.0010196479549424
- **w-rdist:** 0.3282654902519882
- **t-alpha:** 0.048568838795896

---

---

180

- **PDB ID:** 7L2F | **Chain:** A
- **b-phipsi:** 0.0006216894988464
- **w-rdist:** 0.3617116601430972
- **t-alpha:** 0.0605264241229863

---

---

181

- **PDB ID:** 7S6K | **Chain:** E
- **b-phipsi:** 0.0003746172964203
- **w-rdist:** 0.6513297422718466
- **t-alpha:** 0.031431051558405

---

---

182

- **PDB ID:** 7L2F | **Chain:** B
- **b-phipsi:** 0.001437934431343
- **w-rdist:** 0.3659815197657959
- **t-alpha:** 0.0322581402352546

---

---

183

- **PDB ID:** 7Q9I | **Chain:** C
- **b-phipsi:** 0.0005059207004252
- **w-rdist:** 0.5101154984040475
- **t-alpha:** 0.0422415493952179

---

---

184

- **PDB ID:** 7ND9 | **Chain:** B
- **b-phipsi:** 0.0018336938633656
- **w-rdist:** 0.6878439039334289
- **t-alpha:** 0.0016540840807526

---

---

185

- **PDB ID:** 7T9K | **Chain:** B
- **b-phipsi:** 0.0009075152758287
- **w-rdist:** 0.7987061037665798
- **t-alpha:** 0.0024878881015579

---

---

186

- **PDB ID:** 7LD1 | **Chain:** B
- **b-phipsi:** 0.0012992348941424
- **w-rdist:** 0.7586902285341182
- **t-alpha:** 0.0

---

---

187

- **PDB ID:** 7LJR | **Chain:** A
- **b-phipsi:** 0.0015581024848517
- **w-rdist:** 0.1882134229339011
- **t-alpha:** 0.0496278660020452

---

---

188

- **PDB ID:** 7ND5 | **Chain:** B
- **b-phipsi:** 0.0007646924789491
- **w-rdist:** 0.7819843157032086
- **t-alpha:** 0.0091819121329137

---

---

189

- **PDB ID:** 7ENF | **Chain:** A
- **b-phipsi:** 0.0025270553283335
- **w-rdist:** 0.6212701954822236
- **t-alpha:** 0.0033085120789013

---

---

190

- **PDB ID:** 7SOB | **Chain:** K
- **b-phipsi:** 0.0001146107054616
- **w-rdist:** 0.5677479760813198
- **t-alpha:** 0.0586691050610685

---

---

191

- **PDB ID:** 7M6F | **Chain:** B
- **b-phipsi:** 0.0001552749454505
- **w-rdist:** 0.8114066058094632
- **t-alpha:** 0.0254452761871732

---

---

192

- **PDB ID:** 7Q6E | **Chain:** C
- **b-phipsi:** 0.0010519402323078
- **w-rdist:** 0.3794339901698493
- **t-alpha:** 0.0413438942267598

---

---

193

- **PDB ID:** 7KNB | **Chain:** C
- **b-phipsi:** 0.002337322490555
- **w-rdist:** 0.3894285715330959
- **t-alpha:** 0.0254452761871732

---

---

194

- **PDB ID:** 7ENF | **Chain:** B
- **b-phipsi:** 0.0011769253940498
- **w-rdist:** 0.4622479025862954
- **t-alpha:** 0.025640883005656

---

---

195

- **PDB ID:** 5JUY | **Chain:** E
- **b-phipsi:** 0.0061240281814654
- **w-rdist:** 0.4946075514383613
- **t-alpha:** 0.0016569498401739

---

---

196

- **PDB ID:** 7S6K | **Chain:** B
- **b-phipsi:** 0.000374915776365
- **w-rdist:** 0.651328364411099
- **t-alpha:** 0.0351026483066376

---

---

197

- **PDB ID:** 7RA8 | **Chain:** A
- **b-phipsi:** 3.157541911509033e-05
- **w-rdist:** 0.6289724885383943
- **t-alpha:** 0.0558952654697204

---

---

198

- **PDB ID:** 7NDD | **Chain:** A
- **b-phipsi:** 0.0002903588100522
- **w-rdist:** 0.6032214136655423
- **t-alpha:** 0.0467534245579859

---

---

199

- **PDB ID:** 7NYC | **Chain:** A
- **b-phipsi:** 0.001950278422244
- **w-rdist:** 0.5463281470253923
- **t-alpha:** 0.0108695004677259

---

---

200

- **PDB ID:** 7Q0A | **Chain:** B
- **b-phipsi:** 0.0009621615960986
- **w-rdist:** 0.7811600882215823
- **t-alpha:** 0.0058238157721357

---

---

201

- **PDB ID:** 7LQW | **Chain:** A
- **b-phipsi:** 0.0040423652325125
- **w-rdist:** 0.3695138400003818
- **t-alpha:** 0.0223323354003182

---

---

202

- **PDB ID:** 3CU7 | **Chain:** A
- **b-phipsi:** 0.0034461458606175
- **w-rdist:** 0.5549983686365654
- **t-alpha:** 0.0058238157721357

---

---

203

- **PDB ID:** 7Q9K | **Chain:** C
- **b-phipsi:** 0.0004348345130669
- **w-rdist:** 0.6396305444483872
- **t-alpha:** 0.0342175526592682

---

---

204

- **PDB ID:** 7NDC | **Chain:** A
- **b-phipsi:** 0.0003044402047965
- **w-rdist:** 0.5998246266002312
- **t-alpha:** 0.0476602388888385

---

---

205

- **PDB ID:** 7NDD | **Chain:** C
- **b-phipsi:** 0.0003309594557297
- **w-rdist:** 0.5796865847863779
- **t-alpha:** 0.0503909076104127

---

---

206

- **PDB ID:** 7KS9 | **Chain:** B
- **b-phipsi:** 0.000851339148377
- **w-rdist:** 0.7804142315178263
- **t-alpha:** 0.0091819121329137

---

---

207

- **PDB ID:** 7RQ6 | **Chain:** A
- **b-phipsi:** 0.0030579210509764
- **w-rdist:** 0.675070001669796
- **t-alpha:** 0.0

---

---

208

- **PDB ID:** 5O9Z | **Chain:** C
- **b-phipsi:** 0.0322939701084823
- **w-rdist:** 0.271928303360898
- **t-alpha:** 0.0159666566861913

---

---

209

- **PDB ID:** 5ZAM | **Chain:** A
- **b-phipsi:** 0.0495203465321244
- **w-rdist:** 0.4325816348002631
- **t-alpha:** 0.0016540840807526

---

---

210

- **PDB ID:** 7NDB | **Chain:** A
- **b-phipsi:** 0.0010106327271116
- **w-rdist:** 0.1755938092730642
- **t-alpha:** 0.0727594979737589

---

---

211

- **PDB ID:** 7LAA | **Chain:** A
- **b-phipsi:** 0.0004576140615239
- **w-rdist:** 0.3437656603312317
- **t-alpha:** 0.1968569880104891

---

---

212

- **PDB ID:** 7JWB | **Chain:** C
- **b-phipsi:** 0.0007455313648382
- **w-rdist:** 0.6453518861219625
- **t-alpha:** 0.0254452761871732

---

---

213

- **PDB ID:** 7K8S | **Chain:** B
- **b-phipsi:** 0.0003096672915354
- **w-rdist:** 0.4807385000211982
- **t-alpha:** 0.0661703178086101

---

---

214

- **PDB ID:** 7NDD | **Chain:** B
- **b-phipsi:** 0.0004990684443266
- **w-rdist:** 0.8164851736913556
- **t-alpha:** 0.0132341605941959

---

---

215

- **PDB ID:** 7LQV | **Chain:** A
- **b-phipsi:** 0.0023468107689072
- **w-rdist:** 0.4296148374213478
- **t-alpha:** 0.0237089470211593

---

---

216

- **PDB ID:** 7XID | **Chain:** A
- **b-phipsi:** 0.0090241827868642
- **w-rdist:** 0.4236003915172087
- **t-alpha:** 0.0082713823222237

---

---

217

- **PDB ID:** 7NDC | **Chain:** B
- **b-phipsi:** 0.0003049562659115
- **w-rdist:** 0.599827868129446
- **t-alpha:** 0.0512818239323704

---

---

218

- **PDB ID:** 7PNQ | **Chain:** B
- **b-phipsi:** 0.0012826474895852
- **w-rdist:** 0.7340383525969747
- **t-alpha:** 0.0066169305252397

---

---

219

- **PDB ID:** 7MW2 | **Chain:** C
- **b-phipsi:** 0.0011220647123632
- **w-rdist:** 0.3952266533981221
- **t-alpha:** 0.0438375773921617

---

---

220

- **PDB ID:** 7K8W | **Chain:** B
- **b-phipsi:** 0.0009922241434504
- **w-rdist:** 0.516428607963215
- **t-alpha:** 0.0298127438669044

---

---

221

- **PDB ID:** 7L2F | **Chain:** C
- **b-phipsi:** 0.0010370502999066
- **w-rdist:** 0.3665048338699797
- **t-alpha:** 0.0529363797789721

---

---

222

- **PDB ID:** 7WHK | **Chain:** B
- **b-phipsi:** 0.0058506409365323
- **w-rdist:** 0.4801938468520391
- **t-alpha:** 0.0066610907906692

---

---

223

- **PDB ID:** 5U8R | **Chain:** A
- **b-phipsi:** 0.0045415605452242
- **w-rdist:** 0.5590043023356055
- **t-alpha:** 0.0049877163597098

---

---

224

- **PDB ID:** 7BYR | **Chain:** B
- **b-phipsi:** 0.0048968495843386
- **w-rdist:** 0.4529203640139637
- **t-alpha:** 0.0099256639124456

---

---

225

- **PDB ID:** 7LY2 | **Chain:** A
- **b-phipsi:** 0.0001863639137292
- **w-rdist:** 0.4821377543092749
- **t-alpha:** 0.0872302669785827

---

---

226

- **PDB ID:** 7T9K | **Chain:** C
- **b-phipsi:** 0.0009206990518567
- **w-rdist:** 0.7156236975186292
- **t-alpha:** 0.017676751760596

---

---

227

- **PDB ID:** 7K90 | **Chain:** C
- **b-phipsi:** 0.0021159778856105
- **w-rdist:** 0.3314095994625535
- **t-alpha:** 0.0413438942267598

---

---

228

- **PDB ID:** 7DZY | **Chain:** A
- **b-phipsi:** 0.0019555721156535
- **w-rdist:** 0.3984820259333862
- **t-alpha:** 0.031431051558405

---

---

229

- **PDB ID:** 7SG4 | **Chain:** C
- **b-phipsi:** 0.000777090924891
- **w-rdist:** 0.7962380063828033
- **t-alpha:** 0.0115797655430185

---

---

230

- **PDB ID:** 7LXZ | **Chain:** A
- **b-phipsi:** 6.517516843655485e-05
- **w-rdist:** 0.527973376560118
- **t-alpha:** 0.0893298667141726

---

---

231

- **PDB ID:** 7TM0 | **Chain:** C
- **b-phipsi:** 0.0004358059332673
- **w-rdist:** 0.4989940385559143
- **t-alpha:** 0.061457317504612

---

---

232

- **PDB ID:** 7SG4 | **Chain:** A
- **b-phipsi:** 0.0010977090475169
- **w-rdist:** 0.7383551234531761
- **t-alpha:** 0.010752376359526

---

---

233

- **PDB ID:** 7A94 | **Chain:** C
- **b-phipsi:** 0.0007878441995019
- **w-rdist:** 0.7315529172535815
- **t-alpha:** 0.0223323354003182

---

---

234

- **PDB ID:** 7SB4 | **Chain:** A
- **b-phipsi:** 0.0010809601911423
- **w-rdist:** 0.782989091703955
- **t-alpha:** 0.0075000236060698

---

---

235

- **PDB ID:** 7X08 | **Chain:** B
- **b-phipsi:** 0.0034260393283667
- **w-rdist:** 0.6300534570141314
- **t-alpha:** 0.0049877163597098

---

---

236

- **PDB ID:** 7AKJ | **Chain:** B
- **b-phipsi:** 0.0025839432990215
- **w-rdist:** 0.7433563677330135
- **t-alpha:** 0.0008271775551371

---

---

237

- **PDB ID:** 7SN3 | **Chain:** C
- **b-phipsi:** 0.0014288061541138
- **w-rdist:** 0.1605415129832136
- **t-alpha:** 0.0689655430268945

---

---

238

- **PDB ID:** 7Q9F | **Chain:** B
- **b-phipsi:** 0.002253224402425
- **w-rdist:** 0.4507056860446694
- **t-alpha:** 0.0248137209778811

---

---

239

- **PDB ID:** 7CAC | **Chain:** C
- **b-phipsi:** 0.0019520476234013
- **w-rdist:** 0.3999692848985617
- **t-alpha:** 0.0333332138395487

---

---

240

- **PDB ID:** 7PO5 | **Chain:** C
- **b-phipsi:** 0.0020389735375285
- **w-rdist:** 0.7301792354501602
- **t-alpha:** 0.0057897637424946

---

---

241

- **PDB ID:** 6H04 | **Chain:** A
- **b-phipsi:** 0.0011245816882857
- **w-rdist:** 0.6249932508465268
- **t-alpha:** 0.0231594461372635

---

---

242

- **PDB ID:** 7SG4 | **Chain:** B
- **b-phipsi:** 0.0008722154204139
- **w-rdist:** 0.7532776282643857
- **t-alpha:** 0.017369624679081

---

---

243

- **PDB ID:** 7LJR | **Chain:** C
- **b-phipsi:** 0.0006609803227328
- **w-rdist:** 0.3464055372928779
- **t-alpha:** 0.110192776017427

---

---

244

- **PDB ID:** 7E7B | **Chain:** A
- **b-phipsi:** 0.0003622236493983
- **w-rdist:** 0.5298197630507787
- **t-alpha:** 0.065342980668609

---

---

245

- **PDB ID:** 6ACJ | **Chain:** A
- **b-phipsi:** 0.0155564957937322
- **w-rdist:** 0.3603630029519704
- **t-alpha:** 0.0168211022886111

---

---

246

- **PDB ID:** 3KLS | **Chain:** A
- **b-phipsi:** 0.0103839129722398
- **w-rdist:** 0.5679065557451368
- **t-alpha:** 0.0016540840807526

---

---

247

- **PDB ID:** 7SBX | **Chain:** J
- **b-phipsi:** 0.0010435075766128
- **w-rdist:** 0.7846469153070441
- **t-alpha:** 0.0100253538396266

---

---

248

- **PDB ID:** 7XO7 | **Chain:** B
- **b-phipsi:** 0.0062177786436271
- **w-rdist:** 0.4932519909990672
- **t-alpha:** 0.0083406172007201

---

---

249

- **PDB ID:** 7WWL | **Chain:** C
- **b-phipsi:** 0.0115761379710354
- **w-rdist:** 0.5824686261409108
- **t-alpha:** 0.0008281320187666

---

---

250

- **PDB ID:** 7Q9M | **Chain:** A
- **b-phipsi:** 0.000590555830319
- **w-rdist:** 0.7652776834740259
- **t-alpha:** 0.0263160129948751

---

---

251

- **PDB ID:** 7ZR7 | **Chain:** B
- **b-phipsi:** 0.0001635552910537
- **w-rdist:** 0.8496618588188362
- **t-alpha:** 0.0289360965836333

---

---

252

- **PDB ID:** 7LCN | **Chain:** C
- **b-phipsi:** 0.0009299466779039
- **w-rdist:** 0.1704831508058507
- **t-alpha:** 0.2928034136839046

---

---

253

- **PDB ID:** 7ZRC | **Chain:** B
- **b-phipsi:** 0.0002924760793724
- **w-rdist:** 0.8153786552345419
- **t-alpha:** 0.0281224550452081

---

---

254

- **PDB ID:** 7SOB | **Chain:** D
- **b-phipsi:** 0.00011457682019
- **w-rdist:** 0.5677472729643853
- **t-alpha:** 0.0951087693785319

---

---

255

- **PDB ID:** 7K8W | **Chain:** G
- **b-phipsi:** 0.0018376783916273
- **w-rdist:** 0.2291002690624512
- **t-alpha:** 0.0628617784103293

---

---

256

- **PDB ID:** 7ZRC | **Chain:** A
- **b-phipsi:** 0.0006296521163452
- **w-rdist:** 0.5052847229317776
- **t-alpha:** 0.0568180623319403

---

---

257

- **PDB ID:** 7MJM | **Chain:** B
- **b-phipsi:** 0.0003569944424858
- **w-rdist:** 0.8441188108188247
- **t-alpha:** 0.0245762552329136

---

---

258

- **PDB ID:** 7SO9 | **Chain:** A
- **b-phipsi:** 0.0002414996662395
- **w-rdist:** 0.5671598590163828
- **t-alpha:** 0.077540150779898

---

---

259

- **PDB ID:** 7KJ3 | **Chain:** A
- **b-phipsi:** 0.0003284366064897
- **w-rdist:** 0.7879653907103781
- **t-alpha:** 0.0351026483066376

---

---

260

- **PDB ID:** 7RA8 | **Chain:** E
- **b-phipsi:** 3.1484737223938105e-05
- **w-rdist:** 0.6289718688319816
- **t-alpha:** 0.0804290662984963

---

---

261

- **PDB ID:** 7SOB | **Chain:** A
- **b-phipsi:** 0.0001146150854999
- **w-rdist:** 0.5677471594379921
- **t-alpha:** 0.1000913049362774

---

---

262

- **PDB ID:** 7SBV | **Chain:** J
- **b-phipsi:** 0.000851908963643
- **w-rdist:** 0.8079562448419207
- **t-alpha:** 0.0134114913151814

---

---

263

- **PDB ID:** 7LCN | **Chain:** K
- **b-phipsi:** 0.0009548643395683
- **w-rdist:** 0.2025766633312697
- **t-alpha:** 0.2216704632306791

---

---

264

- **PDB ID:** 6WPT | **Chain:** B
- **b-phipsi:** 0.000536422438245
- **w-rdist:** 0.531929722575563
- **t-alpha:** 0.0586691050610685

---

---

265

- **PDB ID:** 7L56 | **Chain:** A
- **b-phipsi:** 0.0018349839949964
- **w-rdist:** 0.368032548127912
- **t-alpha:** 0.0476602388888385

---

---

266

- **PDB ID:** 7UAP | **Chain:** B
- **b-phipsi:** 0.0026326148019996
- **w-rdist:** 0.508564746515996
- **t-alpha:** 0.0237089470211593

---

---

267

- **PDB ID:** 7SC1 | **Chain:** C
- **b-phipsi:** 0.0004038090577232
- **w-rdist:** 0.7806227728954057
- **t-alpha:** 0.0351026483066376

---

---

268

- **PDB ID:** 7K8V | **Chain:** B
- **b-phipsi:** 0.0065832457979237
- **w-rdist:** 0.6109840957551297
- **t-alpha:** 0.0033085120789013

---

---

269

- **PDB ID:** 7KNE | **Chain:** A
- **b-phipsi:** 0.001513164378298
- **w-rdist:** 0.3522791247450941
- **t-alpha:** 0.0568180623319403

---

---

270

- **PDB ID:** 7LAB | **Chain:** A
- **b-phipsi:** 0.0011158759572199
- **w-rdist:** 0.071450937172113
- **t-alpha:** 0.249792625866539

---

---

271

- **PDB ID:** 7KMS | **Chain:** B
- **b-phipsi:** 0.0027180928553405
- **w-rdist:** 0.7899408780955622
- **t-alpha:** 0.0

---

---

272

- **PDB ID:** 7S6J | **Chain:** B
- **b-phipsi:** 0.0016105590028427
- **w-rdist:** 0.6530444461874293
- **t-alpha:** 0.0181966044744523

---

---

273

- **PDB ID:** 7XOD | **Chain:** A
- **b-phipsi:** 0.0014326895178612
- **w-rdist:** 0.8282084979443688
- **t-alpha:** 0.0016569498401739

---

---

274

- **PDB ID:** 7S0C | **Chain:** C
- **b-phipsi:** 0.0004895826888065
- **w-rdist:** 0.525134561983987
- **t-alpha:** 0.0689655430268945

---

---

275

- **PDB ID:** 7KMZ | **Chain:** C
- **b-phipsi:** 0.0025600807658267
- **w-rdist:** 0.3642798245669403
- **t-alpha:** 0.0449441429984014

---

---

276

- **PDB ID:** 6VHH | **Chain:** A
- **b-phipsi:** 0.0036982175665957
- **w-rdist:** 0.7031884419638592
- **t-alpha:** 0.0066169305252397

---

---

277

- **PDB ID:** 7K8T | **Chain:** C
- **b-phipsi:** 0.0017466281310355
- **w-rdist:** 0.4165538394028335
- **t-alpha:** 0.0446646519250406

---

---

278

- **PDB ID:** 7SBY | **Chain:** A
- **b-phipsi:** 0.0009838887159526
- **w-rdist:** 0.8007236721074187
- **t-alpha:** 0.0140611636219507

---

---

279

- **PDB ID:** 7SO9 | **Chain:** F
- **b-phipsi:** 0.0002417691106547
- **w-rdist:** 0.5671597138912067
- **t-alpha:** 0.0990908911987924

---

---

280

- **PDB ID:** 7VXD | **Chain:** D
- **b-phipsi:** 0.0040457062489501
- **w-rdist:** 0.6356668668169436
- **t-alpha:** 0.0090981841206985

---

---

281

- **PDB ID:** 7AKD | **Chain:** C
- **b-phipsi:** 0.0015549282472621
- **w-rdist:** 0.7134189578449177
- **t-alpha:** 0.017676751760596

---

---

282

- **PDB ID:** 7SB3 | **Chain:** J
- **b-phipsi:** 0.0013556166698899
- **w-rdist:** 0.8137450658236032
- **t-alpha:** 0.0058238157721357

---

---

283

- **PDB ID:** 7LCN | **Chain:** A
- **b-phipsi:** 0.0011382662593467
- **w-rdist:** 0.1936857728727078
- **t-alpha:** 0.2034737123256018

---

---

284

- **PDB ID:** 7WS3 | **Chain:** A
- **b-phipsi:** 0.0016196074579673
- **w-rdist:** 0.659573240361729
- **t-alpha:** 0.0202533741750159

---

---

285

- **PDB ID:** 5ZAK | **Chain:** A
- **b-phipsi:** 0.0507121928877957
- **w-rdist:** 0.4572947942258734
- **t-alpha:** 0.0083406172007201

---

---

286

- **PDB ID:** 7MJM | **Chain:** C
- **b-phipsi:** 0.0006055924270852
- **w-rdist:** 0.5003132601103539
- **t-alpha:** 0.068651600081727

---

---

287

- **PDB ID:** 7SB5 | **Chain:** C
- **b-phipsi:** 0.0006791584117517
- **w-rdist:** 0.7934178428841161
- **t-alpha:** 0.026468223205744

---

---

288

- **PDB ID:** 7PO5 | **Chain:** B
- **b-phipsi:** 0.001932159429124
- **w-rdist:** 0.730000846345173
- **t-alpha:** 0.012562807806465

---

---

289

- **PDB ID:** 7Q9P | **Chain:** B
- **b-phipsi:** 0.0005039964478544
- **w-rdist:** 0.785787638180402
- **t-alpha:** 0.0351026483066376

---

---

290

- **PDB ID:** 7ZRC | **Chain:** C
- **b-phipsi:** 0.0003386795254971
- **w-rdist:** 0.8042235533493188
- **t-alpha:** 0.0368785736255408

---

---

291

- **PDB ID:** 7Q9F | **Chain:** A
- **b-phipsi:** 0.0011833916394481
- **w-rdist:** 0.4317683484035796
- **t-alpha:** 0.0540540988034132

---

---

292

- **PDB ID:** 7K90 | **Chain:** A
- **b-phipsi:** 0.0016380392586157
- **w-rdist:** 0.3316624490989872
- **t-alpha:** 0.0670786884214349

---

---

293

- **PDB ID:** 7WS0 | **Chain:** C
- **b-phipsi:** 0.0022575883720505
- **w-rdist:** 0.672260278001079
- **t-alpha:** 0.0151134131271917

---

---

294

- **PDB ID:** 7TCA | **Chain:** A
- **b-phipsi:** 0.0064910629567018
- **w-rdist:** 0.6662789735505674
- **t-alpha:** 0.0033195156791741

---

---

295

- **PDB ID:** 3PRX | **Chain:** D
- **b-phipsi:** 0.0078315611501881
- **w-rdist:** 0.6947849653543595
- **t-alpha:** 0.0024878881015579

---

---

296

- **PDB ID:** 7RQ6 | **Chain:** B
- **b-phipsi:** 0.0038339358431015
- **w-rdist:** 0.676249601070644
- **t-alpha:** 0.0099256639124456

---

---

297

- **PDB ID:** 5WVE | **Chain:** A
- **b-phipsi:** 0.0047020073104983
- **w-rdist:** 0.7304656519468761
- **t-alpha:** 0.0049627974633414

---

---

298

- **PDB ID:** 7KNH | **Chain:** B
- **b-phipsi:** 0.0016321734066124
- **w-rdist:** 0.7992318236418727
- **t-alpha:** 0.0083406172007201

---

---

299

- **PDB ID:** 7SY5 | **Chain:** C
- **b-phipsi:** 0.0007014470234367
- **w-rdist:** 0.8400531073500802
- **t-alpha:** 0.0202533741750159

---

---

300

- **PDB ID:** 6J4Z | **Chain:** A
- **b-phipsi:** 0.0217266503234563
- **w-rdist:** 0.5927664648113062
- **t-alpha:** 0.0049627974633414

---

---

301

- **PDB ID:** 7SB3 | **Chain:** A
- **b-phipsi:** 0.0012920777501945
- **w-rdist:** 0.8093065065836899
- **t-alpha:** 0.0100253538396266

---

---

302

- **PDB ID:** 6ACG | **Chain:** B
- **b-phipsi:** 0.0155569754397894
- **w-rdist:** 0.4070116866811867
- **t-alpha:** 0.0237089470211593

---

---

303

- **PDB ID:** 7DF4 | **Chain:** D
- **b-phipsi:** 0.0007973630791698
- **w-rdist:** 0.4209574562692408
- **t-alpha:** 0.0921410228992365

---

---

304

- **PDB ID:** 6J50 | **Chain:** A
- **b-phipsi:** 0.0217261945692708
- **w-rdist:** 0.5927664497794718
- **t-alpha:** 0.0058238157721357

---

---

305

- **PDB ID:** 5LCW | **Chain:** A
- **b-phipsi:** 0.0286335997351462
- **w-rdist:** 0.61912142810699
- **t-alpha:** 0.0033085120789013

---

---

306

- **PDB ID:** 4NEN | **Chain:** A
- **b-phipsi:** 0.0106169117458048
- **w-rdist:** 0.3556922281277379
- **t-alpha:** 0.0324511081324321

---

---

307

- **PDB ID:** 7V2A | **Chain:** B
- **b-phipsi:** 0.0041481474036391
- **w-rdist:** 0.286710422220452
- **t-alpha:** 0.0568180623319403

---

---

308

- **PDB ID:** 7L3N | **Chain:** B
- **b-phipsi:** 0.0017439124641234
- **w-rdist:** 0.3507722352155074
- **t-alpha:** 0.0699114965509424

---

---

309

- **PDB ID:** 6IR9 | **Chain:** A
- **b-phipsi:** 0.0218292421837478
- **w-rdist:** 0.5943633512675053
- **t-alpha:** 0.0066169305252397

---

---

310

- **PDB ID:** 7WE9 | **Chain:** B
- **b-phipsi:** 0.004587438038353
- **w-rdist:** 0.7881710507486843
- **t-alpha:** 0.0016569498401739

---

---

311

- **PDB ID:** 7WS9 | **Chain:** C
- **b-phipsi:** 0.0003761562461548
- **w-rdist:** 0.5873041038514227
- **t-alpha:** 0.0992554669556216

---

---

312

- **PDB ID:** 6J4X | **Chain:** A
- **b-phipsi:** 0.0220216026025118
- **w-rdist:** 0.5949340720706642
- **t-alpha:** 0.0066169305252397

---

---

313

- **PDB ID:** 5G05 | **Chain:** A
- **b-phipsi:** 0.0290431204626599
- **w-rdist:** 0.4766033977114394
- **t-alpha:** 0.0140611636219507

---

---

314

- **PDB ID:** 7WS8 | **Chain:** C
- **b-phipsi:** 0.0004586514767224
- **w-rdist:** 0.606584441632073
- **t-alpha:** 0.0752689332757208

---

---

315

- **PDB ID:** 7L56 | **Chain:** C
- **b-phipsi:** 0.0012439699161155
- **w-rdist:** 0.3783790748732981
- **t-alpha:** 0.0785014061978188

---

---

316

- **PDB ID:** 7KNI | **Chain:** B
- **b-phipsi:** 0.0022172569386434
- **w-rdist:** 0.8202937360207689
- **t-alpha:** 0.0049627974633414

---

---

317

- **PDB ID:** 7JVC | **Chain:** E
- **b-phipsi:** 0.0006355817826631
- **w-rdist:** 0.8402043231794112
- **t-alpha:** 0.0263160129948751

---

---

318

- **PDB ID:** 7SBX | **Chain:** A
- **b-phipsi:** 0.0003285712690522
- **w-rdist:** 0.7921191256507002
- **t-alpha:** 0.0529363797789721

---

---

319

- **PDB ID:** 7WS4 | **Chain:** C
- **b-phipsi:** 0.0004939786948312
- **w-rdist:** 0.6084132084120686
- **t-alpha:** 0.0744417083525081

---

---

320

- **PDB ID:** 7WEA | **Chain:** A
- **b-phipsi:** 0.0034485348934519
- **w-rdist:** 0.8182015571118683
- **t-alpha:** 0.0008281320187666

---

---

321

- **PDB ID:** 7WS5 | **Chain:** A
- **b-phipsi:** 0.0008001509219435
- **w-rdist:** 0.6268182988943122
- **t-alpha:** 0.0537631265310847

---

---

322

- **PDB ID:** 7CHH | **Chain:** A
- **b-phipsi:** 0.0045599828306466
- **w-rdist:** 0.8058810984767688
- **t-alpha:** 0.0

---

---

323

- **PDB ID:** 7NTC | **Chain:** B
- **b-phipsi:** 0.001578968895956
- **w-rdist:** 0.7542901004445867
- **t-alpha:** 0.0202533741750159

---

---

324

- **PDB ID:** 6W5S | **Chain:** A
- **b-phipsi:** 0.0484808942296313
- **w-rdist:** 0.5540390276198479
- **t-alpha:** 0.0091819121329137

---

---

325

- **PDB ID:** 5IYC | **Chain:** A
- **b-phipsi:** 0.0283410105968308
- **w-rdist:** 0.5821441377559046
- **t-alpha:** 0.0082713823222237

---

---

326

- **PDB ID:** 7PO5 | **Chain:** A
- **b-phipsi:** 0.0020069346583898
- **w-rdist:** 0.7301850276466126
- **t-alpha:** 0.0211148333140693

---

---

327

- **PDB ID:** 6ACG | **Chain:** A
- **b-phipsi:** 0.0156018690720308
- **w-rdist:** 0.3933047896059525
- **t-alpha:** 0.0289360965836333

---

---

328

- **PDB ID:** 7K8U | **Chain:** A
- **b-phipsi:** 0.0031514396621121
- **w-rdist:** 0.4174214429414954
- **t-alpha:** 0.0467534245579859

---

---

329

- **PDB ID:** 7KML | **Chain:** B
- **b-phipsi:** 0.0006971175110181
- **w-rdist:** 0.7532935045444393
- **t-alpha:** 0.0413438942267598

---

---

330

- **PDB ID:** 7MJM | **Chain:** A
- **b-phipsi:** 0.0007258807336468
- **w-rdist:** 0.8440099957964458
- **t-alpha:** 0.0248137209778811

---

---

331

- **PDB ID:** 5JUY | **Chain:** G
- **b-phipsi:** 0.0061265652587843
- **w-rdist:** 0.5876870314258739
- **t-alpha:** 0.0159666566861913

---

---

332

- **PDB ID:** 7TEX | **Chain:** B
- **b-phipsi:** 0.0013562266096764
- **w-rdist:** 0.8430994082291043
- **t-alpha:** 0.0099256639124456

---

---

333

- **PDB ID:** 6NB7 | **Chain:** B
- **b-phipsi:** 0.0011063835213892
- **w-rdist:** 0.7925903417788942
- **t-alpha:** 0.0239867377672513

---

---

334

- **PDB ID:** 7SY3 | **Chain:** C
- **b-phipsi:** 0.0005322848009318
- **w-rdist:** 0.6959150091536306
- **t-alpha:** 0.0605264241229863

---

---

335

- **PDB ID:** 7WPA | **Chain:** C
- **b-phipsi:** 0.0033389353007801
- **w-rdist:** 0.4621779696095175
- **t-alpha:** 0.0363936588404032

---

---

336

- **PDB ID:** 7V81 | **Chain:** A
- **b-phipsi:** 0.0017271902374434
- **w-rdist:** 0.923111136511747
- **t-alpha:** 0.0049627974633414

---

---

337

- **PDB ID:** 7WS5 | **Chain:** C
- **b-phipsi:** 0.0009604194010563
- **w-rdist:** 0.5974402225877014
- **t-alpha:** 0.0521092782942007

---

---

338

- **PDB ID:** 7EJ4 | **Chain:** B
- **b-phipsi:** 0.0086185410706506
- **w-rdist:** 0.7286674532680553
- **t-alpha:** 0.0058238157721357

---

---

339

- **PDB ID:** 7N5H | **Chain:** C
- **b-phipsi:** 0.0011823015244348
- **w-rdist:** 0.8693583849641366
- **t-alpha:** 0.010752376359526

---

---

340

- **PDB ID:** 7L58 | **Chain:** C
- **b-phipsi:** 0.0013822593119171
- **w-rdist:** 0.5836288932113778
- **t-alpha:** 0.0422415493952179

---

---

341

- **PDB ID:** 5U8Q | **Chain:** A
- **b-phipsi:** 0.0030536407199845
- **w-rdist:** 0.550037060069288
- **t-alpha:** 0.0315702473264509

---

---

342

- **PDB ID:** 7SB3 | **Chain:** B
- **b-phipsi:** 0.0003237682401312
- **w-rdist:** 0.8116309545862574
- **t-alpha:** 0.0512818239323704

---

---

343

- **PDB ID:** 7KML | **Chain:** A
- **b-phipsi:** 0.00069731840186
- **w-rdist:** 0.7532943816757811
- **t-alpha:** 0.0440416709650406

---

---

344

- **PDB ID:** 7KJ4 | **Chain:** C
- **b-phipsi:** 0.0002234313528465
- **w-rdist:** 0.8227062818019872
- **t-alpha:** 0.0522196547392181

---

---

345

- **PDB ID:** 7UAR | **Chain:** A
- **b-phipsi:** 0.0009523967682424
- **w-rdist:** 0.7313070405823859
- **t-alpha:** 0.0363936588404032

---

---

346

- **PDB ID:** 7UAP | **Chain:** C
- **b-phipsi:** 0.002247342509171
- **w-rdist:** 0.5225050325836617
- **t-alpha:** 0.0386600222545225

---

---

347

- **PDB ID:** 7X08 | **Chain:** C
- **b-phipsi:** 0.0032917643630846
- **w-rdist:** 0.6302354075805052
- **t-alpha:** 0.0248137209778811

---

---

348

- **PDB ID:** 7K8U | **Chain:** C
- **b-phipsi:** 0.0034028894398891
- **w-rdist:** 0.8183432090628691
- **t-alpha:** 0.0033195156791741

---

---

349

- **PDB ID:** 7P40 | **Chain:** G
- **b-phipsi:** 0.0004173699636353
- **w-rdist:** 0.8101899594353605
- **t-alpha:** 0.0449441429984014

---

---

350

- **PDB ID:** 7D0D | **Chain:** C
- **b-phipsi:** 0.0058979598071329
- **w-rdist:** 0.4489975991060595
- **t-alpha:** 0.031431051558405

---

---

351

- **PDB ID:** 7MW4 | **Chain:** C
- **b-phipsi:** 0.0009148226660979
- **w-rdist:** 0.8019771952142155
- **t-alpha:** 0.0280611759977658

---

---

352

- **PDB ID:** 7BYR | **Chain:** A
- **b-phipsi:** 0.0053953513579617
- **w-rdist:** 0.5635985749796124
- **t-alpha:** 0.0239867377672513

---

---

353

- **PDB ID:** 7L3N | **Chain:** C
- **b-phipsi:** 0.001341610857772
- **w-rdist:** 0.4122158953815467
- **t-alpha:** 0.074666646402848

---

---

354

- **PDB ID:** 7ND4 | **Chain:** A
- **b-phipsi:** 0.0002973137327293
- **w-rdist:** 0.8029159057843139
- **t-alpha:** 0.0568180623319403

---

---

355

- **PDB ID:** 7C2L | **Chain:** B
- **b-phipsi:** 0.012956405718406
- **w-rdist:** 0.5873051920762471
- **t-alpha:** 0.0134114913151814

---

---

356

- **PDB ID:** 7FEM | **Chain:** B
- **b-phipsi:** 0.0053282976805149
- **w-rdist:** 0.5360927505699365
- **t-alpha:** 0.025640883005656

---

---

357

- **PDB ID:** 7DF4 | **Chain:** C
- **b-phipsi:** 0.0012493703753858
- **w-rdist:** 0.4254040240718462
- **t-alpha:** 0.0737123927562979

---

---

358

- **PDB ID:** 7R8M | **Chain:** A
- **b-phipsi:** 0.0048950148540133
- **w-rdist:** 0.3223243926048389
- **t-alpha:** 0.0577426744115672

---

---

359

- **PDB ID:** 7WE7 | **Chain:** D
- **b-phipsi:** 0.0013004712115676
- **w-rdist:** 0.6762403588089632
- **t-alpha:** 0.034739440546361

---

---

360

- **PDB ID:** 6XEY | **Chain:** C
- **b-phipsi:** 0.0011535693899704
- **w-rdist:** 0.4889612725589763
- **t-alpha:** 0.063324598494771

---

---

361

- **PDB ID:** 6J51 | **Chain:** A
- **b-phipsi:** 0.0220202554111515
- **w-rdist:** 0.5949337691688521
- **t-alpha:** 0.010752376359526

---

---

362

- **PDB ID:** 7PNQ | **Chain:** C
- **b-phipsi:** 0.0013591655412594
- **w-rdist:** 0.7341959218308949
- **t-alpha:** 0.0289360965836333

---

---

363

- **PDB ID:** 6XEY | **Chain:** B
- **b-phipsi:** 0.0026122474857622
- **w-rdist:** 0.4796991183685825
- **t-alpha:** 0.0449441429984014

---

---

364

- **PDB ID:** 7WS0 | **Chain:** B
- **b-phipsi:** 0.0020579061495377
- **w-rdist:** 0.6955069546694652
- **t-alpha:** 0.0271879789729934

---

---

365

- **PDB ID:** 7WS3 | **Chain:** C
- **b-phipsi:** 0.0022690350084432
- **w-rdist:** 0.6800617687867546
- **t-alpha:** 0.026468223205744

---

---

366

- **PDB ID:** 5IYB | **Chain:** A
- **b-phipsi:** 0.0276326389106319
- **w-rdist:** 0.5828243067602806
- **t-alpha:** 0.0117157998763879

---

---

367

- **PDB ID:** 7Q9J | **Chain:** B
- **b-phipsi:** 0.0005109028328765
- **w-rdist:** 0.826283501899028
- **t-alpha:** 0.0386600222545225

---

---

368

- **PDB ID:** 7XOD | **Chain:** B
- **b-phipsi:** 0.0012050151038659
- **w-rdist:** 0.8169342172721769
- **t-alpha:** 0.0198511223599899

---

---

369

- **PDB ID:** 6J4Y | **Chain:** A
- **b-phipsi:** 0.0220227044566875
- **w-rdist:** 0.594934192428932
- **t-alpha:** 0.0115797655430185

---

---

370

- **PDB ID:** 7D03 | **Chain:** C
- **b-phipsi:** 0.0143373703927551
- **w-rdist:** 0.4237904762850173
- **t-alpha:** 0.030603642719128

---

---

371

- **PDB ID:** 7AKD | **Chain:** B
- **b-phipsi:** 0.0035197575575318
- **w-rdist:** 0.7235861026341405
- **t-alpha:** 0.0202533741750159

---

---

372

- **PDB ID:** 6NB4 | **Chain:** B
- **b-phipsi:** 0.0011116972422532
- **w-rdist:** 0.440746412472577
- **t-alpha:** 0.0882089880243695

---

---

373

- **PDB ID:** 7PNQ | **Chain:** A
- **b-phipsi:** 0.0013588607032256
- **w-rdist:** 0.7339962957064784
- **t-alpha:** 0.0306906295197915

---

---

374

- **PDB ID:** 7WS8 | **Chain:** B
- **b-phipsi:** 0.0002871230117938
- **w-rdist:** 0.8688342718120265
- **t-alpha:** 0.0503909076104127

---

---

375

- **PDB ID:** 7EH5 | **Chain:** A
- **b-phipsi:** 0.0037545407236643
- **w-rdist:** 0.9548936224727524
- **t-alpha:** 0.0008271775551371

---

---

376

- **PDB ID:** 7M6F | **Chain:** A
- **b-phipsi:** 0.0002203890324435
- **w-rdist:** 0.7862878064699517
- **t-alpha:** 0.074666646402848

---

---

377

- **PDB ID:** 7T9K | **Chain:** A
- **b-phipsi:** 0.0012107640430557
- **w-rdist:** 0.814577206217162
- **t-alpha:** 0.0215054061986588

---

---

378

- **PDB ID:** 7M7F | **Chain:** A
- **b-phipsi:** 0.020256751773186
- **w-rdist:** 0.6339723708532407
- **t-alpha:** 0.0100253538396266

---

---

379

- **PDB ID:** 7KL9 | **Chain:** A
- **b-phipsi:** 0.0031910109605142
- **w-rdist:** 0.7833997789930598
- **t-alpha:** 0.0140611636219507

---

---

380

- **PDB ID:** 7BAN | **Chain:** A
- **b-phipsi:** 0.0041146014703666
- **w-rdist:** 0.4072591382759139
- **t-alpha:** 0.0521092782942007

---

---

381

- **PDB ID:** 7VXK | **Chain:** D
- **b-phipsi:** 0.0040202742744927
- **w-rdist:** 0.7048288013630457
- **t-alpha:** 0.0202533741750159

---

---

382

- **PDB ID:** 7WUH | **Chain:** A
- **b-phipsi:** 0.0099290754401155
- **w-rdist:** 0.493673254157163
- **t-alpha:** 0.026468223205744

---

---

383

- **PDB ID:** 7BAN | **Chain:** B
- **b-phipsi:** 0.0041147022995569
- **w-rdist:** 0.4072595981828747
- **t-alpha:** 0.0521092782942007

---

---

384

- **PDB ID:** 5OIK | **Chain:** A
- **b-phipsi:** 0.0253166929998967
- **w-rdist:** 0.6091230909454769
- **t-alpha:** 0.0108695004677259

---

---

385

- **PDB ID:** 7UAR | **Chain:** B
- **b-phipsi:** 0.001294621256052
- **w-rdist:** 0.5500181229328409
- **t-alpha:** 0.0568180623319403

---

---

386

- **PDB ID:** 7SY5 | **Chain:** B
- **b-phipsi:** 0.0007139803887129
- **w-rdist:** 0.8191881480260323
- **t-alpha:** 0.0330849069147651

---

---

387

- **PDB ID:** 7C2L | **Chain:** C
- **b-phipsi:** 0.0119805173109969
- **w-rdist:** 0.5975081585430668
- **t-alpha:** 0.0168211022886111

---

---

388

- **PDB ID:** 7TAT | **Chain:** B
- **b-phipsi:** 0.0002386959199408
- **w-rdist:** 0.8190659210628615
- **t-alpha:** 0.0586691050610685

---

---

389

- **PDB ID:** 7UAP | **Chain:** A
- **b-phipsi:** 0.0010450741956633
- **w-rdist:** 0.4434758699852937
- **t-alpha:** 0.1031022210331831

---

---

390

- **PDB ID:** 3K71 | **Chain:** G
- **b-phipsi:** 0.01190740962006
- **w-rdist:** 0.3504060347069406
- **t-alpha:** 0.048568838795896

---

---

391

- **PDB ID:** 7WEB | **Chain:** B
- **b-phipsi:** 0.0024317063697934
- **w-rdist:** 0.980425009664938
- **t-alpha:** 0.0057897637424946

---

---

392

- **PDB ID:** 7KXK | **Chain:** A
- **b-phipsi:** 0.0007616646449712
- **w-rdist:** 0.6670156095692805
- **t-alpha:** 0.063324598494771

---

---

393

- **PDB ID:** 7SY3 | **Chain:** A
- **b-phipsi:** 0.0008168260588415
- **w-rdist:** 0.6987104129236693
- **t-alpha:** 0.0568180623319403

---

---

394

- **PDB ID:** 7V81 | **Chain:** C
- **b-phipsi:** 0.0023324138298741
- **w-rdist:** 0.6326810444543992
- **t-alpha:** 0.0324511081324321

---

---

395

- **PDB ID:** 7WK4 | **Chain:** C
- **b-phipsi:** 0.0041018071061073
- **w-rdist:** 0.5636649546543291
- **t-alpha:** 0.0315702473264509

---

---

396

- **PDB ID:** 7M6F | **Chain:** E
- **b-phipsi:** 0.0001619478790559
- **w-rdist:** 0.8325919515474592
- **t-alpha:** 0.064260485408695

---

---

397

- **PDB ID:** 7BAO | **Chain:** A
- **b-phipsi:** 0.00404509876368
- **w-rdist:** 0.4098499074024881
- **t-alpha:** 0.0545905224299128

---

---

398

- **PDB ID:** 6NB3 | **Chain:** B
- **b-phipsi:** 0.0011757074771285
- **w-rdist:** 0.4541247102609561
- **t-alpha:** 0.077540150779898

---

---

399

- **PDB ID:** 6NB4 | **Chain:** A
- **b-phipsi:** 0.00060502613717
- **w-rdist:** 0.6370523026647665
- **t-alpha:** 0.0810588103777436

---

---

400

- **PDB ID:** 7CAI | **Chain:** B
- **b-phipsi:** 0.0124943724695565
- **w-rdist:** 0.7919535799156074
- **t-alpha:** 0.0024811957952362

---

---

401

- **PDB ID:** 7D0B | **Chain:** B
- **b-phipsi:** 0.0097211016622645
- **w-rdist:** 0.4501082414531167
- **t-alpha:** 0.0322581402352546

---

---

402

- **PDB ID:** 6A5P | **Chain:** A
- **b-phipsi:** 0.0285503006216445
- **w-rdist:** 0.614364116636062
- **t-alpha:** 0.0117157998763879

---

---

403

- **PDB ID:** 3KM9 | **Chain:** A
- **b-phipsi:** 0.0101652204082933
- **w-rdist:** 0.7468937757410988
- **t-alpha:** 0.0074438168005386

---

---

404

- **PDB ID:** 7XO7 | **Chain:** C
- **b-phipsi:** 0.0030759520292627
- **w-rdist:** 0.8099431140320534
- **t-alpha:** 0.0108695004677259

---

---

405

- **PDB ID:** 7MW3 | **Chain:** A
- **b-phipsi:** 0.0025326169303091
- **w-rdist:** 0.4166272592268742
- **t-alpha:** 0.0661703178086101

---

---

406

- **PDB ID:** 7SBV | **Chain:** A
- **b-phipsi:** 0.0011119117699576
- **w-rdist:** 0.7975180806339643
- **t-alpha:** 0.030603642719128

---

---

407

- **PDB ID:** 7DZX | **Chain:** B
- **b-phipsi:** 0.0018804252729479
- **w-rdist:** 0.7351385880710134
- **t-alpha:** 0.0281224550452081

---

---

408

- **PDB ID:** 7Q9K | **Chain:** B
- **b-phipsi:** 0.0002107570259638
- **w-rdist:** 0.7645841675498319
- **t-alpha:** 0.1142860673801178

---

---

409

- **PDB ID:** 7NY5 | **Chain:** G
- **b-phipsi:** 0.0004085337319456
- **w-rdist:** 0.8135909342759005
- **t-alpha:** 0.0568180623319403

---

---

410

- **PDB ID:** 7V26 | **Chain:** E
- **b-phipsi:** 0.0056115971366435
- **w-rdist:** 0.8481896128371896
- **t-alpha:** 0.0016540840807526

---

---

411

- **PDB ID:** 7WUH | **Chain:** C
- **b-phipsi:** 0.0095287262324859
- **w-rdist:** 0.407089324955905
- **t-alpha:** 0.0440416709650406

---

---

412

- **PDB ID:** 7MW6 | **Chain:** C
- **b-phipsi:** 0.0042003971578079
- **w-rdist:** 0.8533574790059373
- **t-alpha:** 0.0041355535019986

---

---

413

- **PDB ID:** 6J4W | **Chain:** B
- **b-phipsi:** 0.0044111376826172
- **w-rdist:** 0.9311244331801792
- **t-alpha:** 0.0016540840807526

---

---

414

- **PDB ID:** 7SBW | **Chain:** J
- **b-phipsi:** 0.0011876565524695
- **w-rdist:** 0.8020921893252427
- **t-alpha:** 0.0280611759977658

---

---

415

- **PDB ID:** 7WEA | **Chain:** C
- **b-phipsi:** 0.0028033914267517
- **w-rdist:** 0.7867833150211504
- **t-alpha:** 0.0190237705806923

---

---

416

- **PDB ID:** 7TEX | **Chain:** C
- **b-phipsi:** 0.0008672054412991
- **w-rdist:** 0.713443080636319
- **t-alpha:** 0.0568180623319403

---

---

417

- **PDB ID:** 7E9O | **Chain:** A
- **b-phipsi:** 0.0043188883549258
- **w-rdist:** 0.8798865485545018
- **t-alpha:** 0.0024878881015579

---

---

418

- **PDB ID:** 7K4N | **Chain:** A
- **b-phipsi:** 0.000164835095512
- **w-rdist:** 0.7860325967141983
- **t-alpha:** 0.1204821019187498

---

---

419

- **PDB ID:** 6XCM | **Chain:** B
- **b-phipsi:** 0.0110871776038338
- **w-rdist:** 0.8080520363244265
- **t-alpha:** 0.0024811957952362

---

---

420

- **PDB ID:** 7WWM | **Chain:** C
- **b-phipsi:** 0.0118846417130853
- **w-rdist:** 0.6428758692420069
- **t-alpha:** 0.0165423471063261

---

---

421

- **PDB ID:** 7BAM | **Chain:** B
- **b-phipsi:** 0.0038887874415782
- **w-rdist:** 0.39787456558494
- **t-alpha:** 0.0636887102856156

---

---

422

- **PDB ID:** 7DX7 | **Chain:** B
- **b-phipsi:** 0.0123810221143336
- **w-rdist:** 0.4105830308842044
- **t-alpha:** 0.0405293719948107

---

---

423

- **PDB ID:** 7A94 | **Chain:** B
- **b-phipsi:** 0.0007768969686321
- **w-rdist:** 0.7278373448923997
- **t-alpha:** 0.0620344765721561

---

---

424

- **PDB ID:** 7DX6 | **Chain:** B
- **b-phipsi:** 0.0117860694619073
- **w-rdist:** 0.3898420499919486
- **t-alpha:** 0.0463192707105852

---

---

425

- **PDB ID:** 7WHI | **Chain:** B
- **b-phipsi:** 0.0056621618844509
- **w-rdist:** 0.486818905388652
- **t-alpha:** 0.0368785736255408

---

---

426

- **PDB ID:** 7BAM | **Chain:** A
- **b-phipsi:** 0.0038892560039243
- **w-rdist:** 0.3978755563949835
- **t-alpha:** 0.0636887102856156

---

---

427

- **PDB ID:** 4UI9 | **Chain:** A
- **b-phipsi:** 0.0266009479501654
- **w-rdist:** 0.6248975391730938
- **t-alpha:** 0.014888357714047

---

---

428

- **PDB ID:** 7NDB | **Chain:** B
- **b-phipsi:** 0.0017208616554227
- **w-rdist:** 0.6947407676689329
- **t-alpha:** 0.0372207702648281

---

---

429

- **PDB ID:** 7KXJ | **Chain:** B
- **b-phipsi:** 0.0017535091212637
- **w-rdist:** 0.637907218551881
- **t-alpha:** 0.0422415493952179

---

---

430

- **PDB ID:** 7DX8 | **Chain:** B
- **b-phipsi:** 0.0135065930334537
- **w-rdist:** 0.3927353471440474
- **t-alpha:** 0.0446646519250406

---

---

431

- **PDB ID:** 7S6J | **Chain:** A
- **b-phipsi:** 0.001607798045465
- **w-rdist:** 0.653043374166291
- **t-alpha:** 0.0440416709650406

---

---

432

- **PDB ID:** 7MW5 | **Chain:** B
- **b-phipsi:** 0.0021323843608289
- **w-rdist:** 0.905695415556632
- **t-alpha:** 0.0100253538396266

---

---

433

- **PDB ID:** 7MXP | **Chain:** C
- **b-phipsi:** 0.0069950954275395
- **w-rdist:** 0.5098065915490928
- **t-alpha:** 0.0322581402352546

---

---

434

- **PDB ID:** 7K8T | **Chain:** B
- **b-phipsi:** 0.0006757040876107
- **w-rdist:** 0.8603450604305969
- **t-alpha:** 0.0368785736255408

---

---

435

- **PDB ID:** 7S0D | **Chain:** A
- **b-phipsi:** 0.0012142996790984
- **w-rdist:** 0.5901946350466512
- **t-alpha:** 0.0586691050610685

---

---

436

- **PDB ID:** 7TM0 | **Chain:** B
- **b-phipsi:** 0.0003895732543276
- **w-rdist:** 0.827593068057757
- **t-alpha:** 0.0568180623319403

---

---

437

- **PDB ID:** 7Q9G | **Chain:** B
- **b-phipsi:** 0.0002163553221414
- **w-rdist:** 0.8143311612046156
- **t-alpha:** 0.0727594979737589

---

---

438

- **PDB ID:** 7D0B | **Chain:** C
- **b-phipsi:** 0.0143357884105572
- **w-rdist:** 0.423788838862722
- **t-alpha:** 0.0372207702648281

---

---

439

- **PDB ID:** 7WS1 | **Chain:** A
- **b-phipsi:** 0.0018486704536062
- **w-rdist:** 0.6554451453354577
- **t-alpha:** 0.0380480467994648

---

---

440

- **PDB ID:** 7WHJ | **Chain:** C
- **b-phipsi:** 0.006411816955766
- **w-rdist:** 0.3833794528143308
- **t-alpha:** 0.0554175614600527

---

---

441

- **PDB ID:** 6NB3 | **Chain:** A
- **b-phipsi:** 0.0006957189503602
- **w-rdist:** 0.6315556043096923
- **t-alpha:** 0.0885027242012517

---

---

442

- **PDB ID:** 7V89 | **Chain:** B
- **b-phipsi:** 0.0037859895216612
- **w-rdist:** 0.9165374385978016
- **t-alpha:** 0.0049877163597098

---

---

443

- **PDB ID:** 7WS4 | **Chain:** A
- **b-phipsi:** 0.0006689180024831
- **w-rdist:** 0.6338286765937702
- **t-alpha:** 0.0959466802142319

---

---

444

- **PDB ID:** 7R40 | **Chain:** C
- **b-phipsi:** 0.0029969802024011
- **w-rdist:** 0.912989409663478
- **t-alpha:** 0.0075000236060698

---

---

445

- **PDB ID:** 7TEX | **Chain:** A
- **b-phipsi:** 0.0009896103732664
- **w-rdist:** 0.7091982191651041
- **t-alpha:** 0.0549737774297427

---

---

446

- **PDB ID:** 7KXJ | **Chain:** A
- **b-phipsi:** 0.0016386166821975
- **w-rdist:** 0.6732221567767278
- **t-alpha:** 0.0422415493952179

---

---

447

- **PDB ID:** 7LQV | **Chain:** B
- **b-phipsi:** 0.0030584543462386
- **w-rdist:** 0.8088785431074005
- **t-alpha:** 0.0151134131271917

---

---

448

- **PDB ID:** 7ND4 | **Chain:** B
- **b-phipsi:** 0.0001836300204167
- **w-rdist:** 0.8029158117681218
- **t-alpha:** 0.0980929580625606

---

---

449

- **PDB ID:** 7SB5 | **Chain:** B
- **b-phipsi:** 0.0009664787817455
- **w-rdist:** 0.7938435601643103
- **t-alpha:** 0.0380480467994648

---

---

450

- **PDB ID:** 7MW5 | **Chain:** A
- **b-phipsi:** 0.0088933555648844
- **w-rdist:** 0.5336748112285927
- **t-alpha:** 0.030603642719128

---

---

451

- **PDB ID:** 3K6S | **Chain:** A
- **b-phipsi:** 0.0126926443416385
- **w-rdist:** 0.3869260126330454
- **t-alpha:** 0.048568838795896

---

---

452

- **PDB ID:** 7E9N | **Chain:** A
- **b-phipsi:** 0.0051993354595994
- **w-rdist:** 0.4379835323786063
- **t-alpha:** 0.0503909076104127

---

---

453

- **PDB ID:** 7MXP | **Chain:** A
- **b-phipsi:** 0.005373797216276
- **w-rdist:** 0.503408137147477
- **t-alpha:** 0.0380480467994648

---

---

454

- **PDB ID:** 5WVE | **Chain:** E
- **b-phipsi:** 0.0046926010737459
- **w-rdist:** 0.7306265340654237
- **t-alpha:** 0.0219779944667735

---

---

455

- **PDB ID:** 7SB4 | **Chain:** C
- **b-phipsi:** 0.0011272109369546
- **w-rdist:** 0.7824968312676372
- **t-alpha:** 0.0372207702648281

---

---

456

- **PDB ID:** 7CAI | **Chain:** C
- **b-phipsi:** 0.0089602770352638
- **w-rdist:** 0.4179055354973293
- **t-alpha:** 0.0476602388888385

---

---

457

- **PDB ID:** 7S6J | **Chain:** E
- **b-phipsi:** 0.0015178773724476
- **w-rdist:** 0.6530447239089718
- **t-alpha:** 0.0476602388888385

---

---

458

- **PDB ID:** 7M6I | **Chain:** B
- **b-phipsi:** 0.0008936809101751
- **w-rdist:** 0.7898576485672202
- **t-alpha:** 0.0449441429984014

---

---

459

- **PDB ID:** 7MW3 | **Chain:** B
- **b-phipsi:** 0.0014161237053048
- **w-rdist:** 0.4592710984052985
- **t-alpha:** 0.0843050695607743

---

---

460

- **PDB ID:** 4ACQ | **Chain:** C
- **b-phipsi:** 0.0002488680008504
- **w-rdist:** 0.7555875646242053
- **t-alpha:** 0.2274605867519343

---

---

461

- **PDB ID:** 6ACK | **Chain:** B
- **b-phipsi:** 0.0102271373405693
- **w-rdist:** 0.3541041619302249
- **t-alpha:** 0.0577426744115672

---

---

462

- **PDB ID:** 3JBT | **Chain:** E
- **b-phipsi:** 0.0048330144889271
- **w-rdist:** 0.7306126213773477
- **t-alpha:** 0.0223323354003182

---

---

463

- **PDB ID:** 7TF0 | **Chain:** C
- **b-phipsi:** 0.0008281821471019
- **w-rdist:** 0.7177581981479197
- **t-alpha:** 0.064260485408695

---

---

464

- **PDB ID:** 7W98 | **Chain:** B
- **b-phipsi:** 0.0016038022271962
- **w-rdist:** 0.6218218369797647
- **t-alpha:** 0.0531362262599823

---

---

465

- **PDB ID:** 7MW6 | **Chain:** B
- **b-phipsi:** 0.0037434499142044
- **w-rdist:** 0.8696021603414246
- **t-alpha:** 0.0075000236060698

---

---

466

- **PDB ID:** 7ND4 | **Chain:** C
- **b-phipsi:** 0.0002972573567924
- **w-rdist:** 0.80291337040968
- **t-alpha:** 0.082363784576418

---

---

467

- **PDB ID:** 7SBY | **Chain:** J
- **b-phipsi:** 0.001587614467577
- **w-rdist:** 0.7922235343698085
- **t-alpha:** 0.0298127438669044

---

---

468

- **PDB ID:** 7SB5 | **Chain:** A
- **b-phipsi:** 0.001810323806679
- **w-rdist:** 0.797465913217172
- **t-alpha:** 0.026468223205744

---

---

469

- **PDB ID:** 7XO8 | **Chain:** B
- **b-phipsi:** 0.0039951289905225
- **w-rdist:** 0.8579012779632552
- **t-alpha:** 0.0075000236060698

---

---

470

- **PDB ID:** 7DX5 | **Chain:** B
- **b-phipsi:** 0.012455396287743
- **w-rdist:** 0.4003499390646138
- **t-alpha:** 0.0479735119257889

---

---

471

- **PDB ID:** 6NB7 | **Chain:** A
- **b-phipsi:** 0.0010185967442941
- **w-rdist:** 0.8523367620310096
- **t-alpha:** 0.0289360965836333

---

---

472

- **PDB ID:** 7Q9J | **Chain:** C
- **b-phipsi:** 0.0009017155363365
- **w-rdist:** 0.8338075199147428
- **t-alpha:** 0.0342175526592682

---

---

473

- **PDB ID:** 7V81 | **Chain:** B
- **b-phipsi:** 0.0026931309831791
- **w-rdist:** 0.9078622058519258
- **t-alpha:** 0.0100253538396266

---

---

474

- **PDB ID:** 7UAR | **Chain:** C
- **b-phipsi:** 0.0009499907056125
- **w-rdist:** 0.5524980624618321
- **t-alpha:** 0.1058724543546798

---

---

475

- **PDB ID:** 7L58 | **Chain:** A
- **b-phipsi:** 0.000887799155034
- **w-rdist:** 0.5603487946214933
- **t-alpha:** 0.13096385708245

---

---

476

- **PDB ID:** 7RKV | **Chain:** A
- **b-phipsi:** 0.0001851377583857
- **w-rdist:** 0.878652359327603
- **t-alpha:** 0.0699114965509424

---

---

477

- **PDB ID:** 7V83 | **Chain:** A
- **b-phipsi:** 0.0032660661718341
- **w-rdist:** 0.904344355205408
- **t-alpha:** 0.0099256639124456

---

---

478

- **PDB ID:** 7SY3 | **Chain:** B
- **b-phipsi:** 0.0009138809949462
- **w-rdist:** 0.8231107467420987
- **t-alpha:** 0.0363936588404032

---

---

479

- **PDB ID:** 7AKD | **Chain:** A
- **b-phipsi:** 0.0015103519628931
- **w-rdist:** 0.8697491202164229
- **t-alpha:** 0.0228428607189075

---

---

480

- **PDB ID:** 7DZX | **Chain:** C
- **b-phipsi:** 0.0026855331082183
- **w-rdist:** 0.4081671228019028
- **t-alpha:** 0.0868487681283765

---

---

481

- **PDB ID:** 7M7I | **Chain:** A
- **b-phipsi:** 0.0184070726488898
- **w-rdist:** 0.6358364783692314
- **t-alpha:** 0.0211148333140693

---

---

482

- **PDB ID:** 7MXP | **Chain:** B
- **b-phipsi:** 0.0053781333833355
- **w-rdist:** 0.6037155438782883
- **t-alpha:** 0.0322581402352546

---

---

483

- **PDB ID:** 7EPX | **Chain:** C
- **b-phipsi:** 0.016449073982592
- **w-rdist:** 0.3984767840019033
- **t-alpha:** 0.0504550203401277

---

---

484

- **PDB ID:** 7WHK | **Chain:** A
- **b-phipsi:** 0.0060795524178582
- **w-rdist:** 0.8716702672716345
- **t-alpha:** 0.0041527907791232

---

---

485

- **PDB ID:** 4ZXB | **Chain:** E
- **b-phipsi:** 0.0049866264164505
- **w-rdist:** 0.764614935891152
- **t-alpha:** 0.0202533741750159

---

---

486

- **PDB ID:** 7M7H | **Chain:** B
- **b-phipsi:** 0.0181665301006623
- **w-rdist:** 0.4612901100596998
- **t-alpha:** 0.0363936588404032

---

---

487

- **PDB ID:** 6UIA | **Chain:** B
- **b-phipsi:** 0.0207975290678138
- **w-rdist:** 0.7442104292634261
- **t-alpha:** 0.0108695004677259

---

---

488

- **PDB ID:** 6NB6 | **Chain:** B
- **b-phipsi:** 0.0009391109610644
- **w-rdist:** 0.8558760408618676
- **t-alpha:** 0.0339123357766464

---

---

489

- **PDB ID:** 7DX7 | **Chain:** C
- **b-phipsi:** 0.0129422247992768
- **w-rdist:** 0.3904579710564737
- **t-alpha:** 0.0562447454201915

---

---

490

- **PDB ID:** 7WS0 | **Chain:** A
- **b-phipsi:** 0.0016160139888317
- **w-rdist:** 0.6543422951611374
- **t-alpha:** 0.0537631265310847

---

---

491

- **PDB ID:** 7R40 | **Chain:** A
- **b-phipsi:** 0.0029976735195008
- **w-rdist:** 0.9129881034036652
- **t-alpha:** 0.0117157998763879

---

---

492

- **PDB ID:** 7TAT | **Chain:** C
- **b-phipsi:** 0.0007057125858607
- **w-rdist:** 0.7257268441594995
- **t-alpha:** 0.0862534621133408

---

---

493

- **PDB ID:** 7WUH | **Chain:** E
- **b-phipsi:** 0.0112341717689602
- **w-rdist:** 0.468676426163407
- **t-alpha:** 0.0413438942267598

---

---

494

- **PDB ID:** 7ZR9 | **Chain:** B
- **b-phipsi:** 0.0004471599499605
- **w-rdist:** 0.8858256446716182
- **t-alpha:** 0.0568180623319403

---

---

495

- **PDB ID:** 4ACQ | **Chain:** A
- **b-phipsi:** 0.0005742907982655
- **w-rdist:** 0.7684129118023593
- **t-alpha:** 0.0802312519388588

---

---

496

- **PDB ID:** 7JV4 | **Chain:** B
- **b-phipsi:** 0.0006827806823806
- **w-rdist:** 0.6838260763770272
- **t-alpha:** 0.1215212383717661

---

---

497

- **PDB ID:** 7L57 | **Chain:** B
- **b-phipsi:** 0.0022126736275323
- **w-rdist:** 0.5103612779741795
- **t-alpha:** 0.0680214444040752

---

---

498

- **PDB ID:** 7TF0 | **Chain:** A
- **b-phipsi:** 0.0008338159106248
- **w-rdist:** 0.7113852503368505
- **t-alpha:** 0.0765807788243952

---

---

499

- **PDB ID:** 7LQV | **Chain:** C
- **b-phipsi:** 0.0026646604395475
- **w-rdist:** 0.5214247814129181
- **t-alpha:** 0.0628617784103293

---

---

500

- **PDB ID:** 7K8Y | **Chain:** B
- **b-phipsi:** 0.0027021139585352
- **w-rdist:** 0.4627007014723959
- **t-alpha:** 0.0689655430268945

---

---

501

- **PDB ID:** 7SC1 | **Chain:** B
- **b-phipsi:** 0.0007939998964206
- **w-rdist:** 0.7802005582452913
- **t-alpha:** 0.064260485408695

---

---

502

- **PDB ID:** 7ZR9 | **Chain:** C
- **b-phipsi:** 0.0006793181840736
- **w-rdist:** 0.8487558268754595
- **t-alpha:** 0.0503909076104127

---

---

503

- **PDB ID:** 7E9O | **Chain:** C
- **b-phipsi:** 0.0032936896483736
- **w-rdist:** 0.8591805601954157
- **t-alpha:** 0.0142618534263445

---

---

504

- **PDB ID:** 7WPA | **Chain:** A
- **b-phipsi:** 0.0033393120858696
- **w-rdist:** 0.7941921183397561
- **t-alpha:** 0.0248137209778811

---

---

505

- **PDB ID:** 5ES4 | **Chain:** A
- **b-phipsi:** 0.008282216487682
- **w-rdist:** 0.4068221129567034
- **t-alpha:** 0.061457317504612

---

---

506

- **PDB ID:** 4ACQ | **Chain:** D
- **b-phipsi:** 0.0005043299463999
- **w-rdist:** 0.7498041134318689
- **t-alpha:** 0.1571546982894849

---

---

507

- **PDB ID:** 7POV | **Chain:** A
- **b-phipsi:** 0.005230871249445
- **w-rdist:** 0.7337945705304884
- **t-alpha:** 0.025640883005656

---

---

508

- **PDB ID:** 7TF0 | **Chain:** B
- **b-phipsi:** 0.001281559981639
- **w-rdist:** 0.8128112795160993
- **t-alpha:** 0.034739440546361

---

---

509

- **PDB ID:** 7A95 | **Chain:** B
- **b-phipsi:** 0.0102567219066792
- **w-rdist:** 0.7450378199353986
- **t-alpha:** 0.0181966044744523

---

---

510

- **PDB ID:** 7L58 | **Chain:** B
- **b-phipsi:** 0.0007091451900534
- **w-rdist:** 0.8250138397344102
- **t-alpha:** 0.0531362262599823

---

---

511

- **PDB ID:** 7D0C | **Chain:** B
- **b-phipsi:** 0.0058471003320042
- **w-rdist:** 0.448968570565292
- **t-alpha:** 0.0570720736347449

---

---

512

- **PDB ID:** 7K8U | **Chain:** B
- **b-phipsi:** 0.0029946657568897
- **w-rdist:** 0.8396048629038503
- **t-alpha:** 0.0202533741750159

---

---

513

- **PDB ID:** 5JUY | **Chain:** C
- **b-phipsi:** 0.0041504646321524
- **w-rdist:** 0.7017263463706258
- **t-alpha:** 0.0324511081324321

---

---

514

- **PDB ID:** 3JBT | **Chain:** A
- **b-phipsi:** 0.0048327770802249
- **w-rdist:** 0.7306132711375296
- **t-alpha:** 0.0280611759977658

---

---

515

- **PDB ID:** 7LD1 | **Chain:** A
- **b-phipsi:** 0.0013238466381551
- **w-rdist:** 0.7230610496266399
- **t-alpha:** 0.0568180623319403

---

---

516

- **PDB ID:** 7DX5 | **Chain:** C
- **b-phipsi:** 0.014919119095537
- **w-rdist:** 0.3702094554682211
- **t-alpha:** 0.0636887102856156

---

---

517

- **PDB ID:** 7CAC | **Chain:** B
- **b-phipsi:** 0.0033343434581452
- **w-rdist:** 0.7069606661573737
- **t-alpha:** 0.0377684208359867

---

---

518

- **PDB ID:** 6ACG | **Chain:** C
- **b-phipsi:** 0.0210288266651665
- **w-rdist:** 0.6057078197241843
- **t-alpha:** 0.0272952510798727

---

---

519

- **PDB ID:** 7KNI | **Chain:** A
- **b-phipsi:** 0.0021825747892635
- **w-rdist:** 0.8002634425394113
- **t-alpha:** 0.030603642719128

---

---

520

- **PDB ID:** 4E0S | **Chain:** A
- **b-phipsi:** 0.0011376292164053
- **w-rdist:** 0.5788217794593749
- **t-alpha:** 0.1050453970057188

---

---

521

- **PDB ID:** 7M7H | **Chain:** A
- **b-phipsi:** 0.0178943587791847
- **w-rdist:** 0.6353269890138571
- **t-alpha:** 0.0263160129948751

---

---

522

- **PDB ID:** 3PVM | **Chain:** B
- **b-phipsi:** 0.007961008450577
- **w-rdist:** 0.6882625365482106
- **t-alpha:** 0.0272952510798727

---

---

523

- **PDB ID:** 7KQB | **Chain:** C
- **b-phipsi:** 0.0007250016669324
- **w-rdist:** 0.6576582376608145
- **t-alpha:** 0.2588911390182236

---

---

524

- **PDB ID:** 6NB3 | **Chain:** C
- **b-phipsi:** 0.0008300268521656
- **w-rdist:** 0.6384735243324219
- **t-alpha:** 0.1736970446997137

---

---

525

- **PDB ID:** 7KMZ | **Chain:** B
- **b-phipsi:** 0.0022215957620415
- **w-rdist:** 0.8190651050733717
- **t-alpha:** 0.0263160129948751

---

---

526

- **PDB ID:** 7E5S | **Chain:** A
- **b-phipsi:** 0.0050292770300552
- **w-rdist:** 0.491705569312524
- **t-alpha:** 0.0558952654697204

---

---

527

- **PDB ID:** 7WEC | **Chain:** C
- **b-phipsi:** 0.0038746991643638
- **w-rdist:** 0.8022216168691627
- **t-alpha:** 0.0239867377672513

---

---

528

- **PDB ID:** 7R8M | **Chain:** C
- **b-phipsi:** 0.0099484905310264
- **w-rdist:** 0.4193728437066629
- **t-alpha:** 0.0577426744115672

---

---

529

- **PDB ID:** 7KML | **Chain:** C
- **b-phipsi:** 0.0006971216401612
- **w-rdist:** 0.7532931358105671
- **t-alpha:** 0.0862534621133408

---

---

530

- **PDB ID:** 7THT | **Chain:** V
- **b-phipsi:** 0.0006953727503366
- **w-rdist:** 0.7689338162584425
- **t-alpha:** 0.0813954771139466

---

---

531

- **PDB ID:** 7WLY | **Chain:** C
- **b-phipsi:** 0.0039280088552704
- **w-rdist:** 0.7533876020023365
- **t-alpha:** 0.0297764065873182

---

---

532

- **PDB ID:** 7V26 | **Chain:** A
- **b-phipsi:** 0.0080788879514133
- **w-rdist:** 0.5744558063919945
- **t-alpha:** 0.0386600222545225

---

---

533

- **PDB ID:** 7FAF | **Chain:** B
- **b-phipsi:** 0.0094280189146671
- **w-rdist:** 0.5798136397010316
- **t-alpha:** 0.0372207702648281

---

---

534

- **PDB ID:** 7MM0 | **Chain:** B
- **b-phipsi:** 0.0034646725132573
- **w-rdist:** 0.7366279556944226
- **t-alpha:** 0.0333332138395487

---

---

535

- **PDB ID:** 7SBV | **Chain:** B
- **b-phipsi:** 0.0005342624205977
- **w-rdist:** 0.8118243894490408
- **t-alpha:** 0.0744417083525081

---

---

536

- **PDB ID:** 7PNM | **Chain:** A
- **b-phipsi:** 0.0037248013769846
- **w-rdist:** 0.7521025176557863
- **t-alpha:** 0.0315702473264509

---

---

537

- **PDB ID:** 7XO8 | **Chain:** C
- **b-phipsi:** 0.0041492022888393
- **w-rdist:** 0.8080470615835957
- **t-alpha:** 0.0228428607189075

---

---

538

- **PDB ID:** 7D03 | **Chain:** B
- **b-phipsi:** 0.0097224921479539
- **w-rdist:** 0.4501085480404914
- **t-alpha:** 0.0545905224299128

---

---

539

- **PDB ID:** 7RKV | **Chain:** B
- **b-phipsi:** 0.0002325289108696
- **w-rdist:** 0.8785423674660211
- **t-alpha:** 0.0961018998709848

---

---

540

- **PDB ID:** 3J2T | **Chain:** E
- **b-phipsi:** 0.0048589204700805
- **w-rdist:** 0.7443992895341115
- **t-alpha:** 0.0280611759977658

---

---

541

- **PDB ID:** 4ACQ | **Chain:** B
- **b-phipsi:** 0.000630903585371
- **w-rdist:** 0.7765455626942382
- **t-alpha:** 0.0967742494764718

---

---

542

- **PDB ID:** 7WE7 | **Chain:** E
- **b-phipsi:** 0.0009942340803178
- **w-rdist:** 0.6966520836506274
- **t-alpha:** 0.0785770064706012

---

---

543

- **PDB ID:** 7JWB | **Chain:** B
- **b-phipsi:** 0.0002224656325932
- **w-rdist:** 0.8654996140237977
- **t-alpha:** 0.1051191692070554

---

---

544

- **PDB ID:** 7KNH | **Chain:** A
- **b-phipsi:** 0.0011196194418958
- **w-rdist:** 0.7969033339198196
- **t-alpha:** 0.0504550203401277

---

---

545

- **PDB ID:** 7TB8 | **Chain:** B
- **b-phipsi:** 0.0050245736324737
- **w-rdist:** 0.7290619630621518
- **t-alpha:** 0.0306906295197915

---

---

546

- **PDB ID:** 3JBT | **Chain:** I
- **b-phipsi:** 0.0048332647946769
- **w-rdist:** 0.7306132201436053
- **t-alpha:** 0.0306906295197915

---

---

547

- **PDB ID:** 5WVE | **Chain:** K
- **b-phipsi:** 0.0046941083355925
- **w-rdist:** 0.7304663320142508
- **t-alpha:** 0.0315702473264509

---

---

548

- **PDB ID:** 5JUY | **Chain:** B
- **b-phipsi:** 0.0061506419667634
- **w-rdist:** 0.5162536048172361
- **t-alpha:** 0.0513043822350767

---

---

549

- **PDB ID:** 7WEC | **Chain:** A
- **b-phipsi:** 0.0042331533242792
- **w-rdist:** 0.7976661983684689
- **t-alpha:** 0.0248137209778811

---

---

550

- **PDB ID:** 7W98 | **Chain:** A
- **b-phipsi:** 0.0024880630301931
- **w-rdist:** 0.6001873245295668
- **t-alpha:** 0.061457317504612

---

---

551

- **PDB ID:** 7AKJ | **Chain:** C
- **b-phipsi:** 0.0025838585756011
- **w-rdist:** 0.7433570057179287
- **t-alpha:** 0.0413438942267598

---

---

552

- **PDB ID:** 7PNM | **Chain:** B
- **b-phipsi:** 0.0036378610359136
- **w-rdist:** 0.7518765356026259
- **t-alpha:** 0.0342175526592682

---

---

553

- **PDB ID:** 7CZP | **Chain:** B
- **b-phipsi:** 0.0058975310868758
- **w-rdist:** 0.4490001785657403
- **t-alpha:** 0.0636887102856156

---

---

554

- **PDB ID:** 7CZR | **Chain:** B
- **b-phipsi:** 0.0058959424283738
- **w-rdist:** 0.4489983089005258
- **t-alpha:** 0.0645157779328724

---

---

555

- **PDB ID:** 7POV | **Chain:** B
- **b-phipsi:** 0.006667391783875
- **w-rdist:** 0.7362068701296696
- **t-alpha:** 0.026468223205744

---

---

556

- **PDB ID:** 7CWL | **Chain:** A
- **b-phipsi:** 0.0040914289733768
- **w-rdist:** 0.5029294795895258
- **t-alpha:** 0.066137781369111

---

---

557

- **PDB ID:** 7PG3 | **Chain:** B
- **b-phipsi:** 0.0085085455918121
- **w-rdist:** 0.5786565970070455
- **t-alpha:** 0.0440416709650406

---

---

558

- **PDB ID:** 7P40 | **Chain:** A
- **b-phipsi:** 0.0004174357879605
- **w-rdist:** 0.8101879873562813
- **t-alpha:** 0.117375261057294

---

---

559

- **PDB ID:** 7NY5 | **Chain:** A
- **b-phipsi:** 0.00040855831768
- **w-rdist:** 0.8135922046288736
- **t-alpha:** 0.1142860673801178

---

---

560

- **PDB ID:** 6ZDH | **Chain:** C
- **b-phipsi:** 0.0003715564877093
- **w-rdist:** 0.9005636786173536
- **t-alpha:** 0.0802312519388588

---

---

561

- **PDB ID:** 7KJ3 | **Chain:** B
- **b-phipsi:** 0.0005029407237353
- **w-rdist:** 0.8032546315580287
- **t-alpha:** 0.107142972791848

---

---

562

- **PDB ID:** 7KMS | **Chain:** C
- **b-phipsi:** 0.0033656150281352
- **w-rdist:** 0.8159813562311804
- **t-alpha:** 0.025640883005656

---

---

563

- **PDB ID:** 7D00 | **Chain:** B
- **b-phipsi:** 0.0058952997783608
- **w-rdist:** 0.4490001912610362
- **t-alpha:** 0.0661703178086101

---

---

564

- **PDB ID:** 7CZY | **Chain:** B
- **b-phipsi:** 0.0058947342161087
- **w-rdist:** 0.4490004923555815
- **t-alpha:** 0.0661703178086101

---

---

565

- **PDB ID:** 7Q9J | **Chain:** A
- **b-phipsi:** 0.0005463592397765
- **w-rdist:** 0.8292406521721943
- **t-alpha:** 0.0718084710189059

---

---

566

- **PDB ID:** 7CZW | **Chain:** B
- **b-phipsi:** 0.0058960998110746
- **w-rdist:** 0.4490004086731003
- **t-alpha:** 0.0661703178086101

---

---

567

- **PDB ID:** 7Q9G | **Chain:** A
- **b-phipsi:** 0.0007834906910942
- **w-rdist:** 0.8123775618743198
- **t-alpha:** 0.0651982118967349

---

---

568

- **PDB ID:** 7CZT | **Chain:** B
- **b-phipsi:** 0.0058972155873353
- **w-rdist:** 0.4489997691125835
- **t-alpha:** 0.0678243742037618

---

---

569

- **PDB ID:** 5WVE | **Chain:** I
- **b-phipsi:** 0.0046940087766907
- **w-rdist:** 0.7304714351967067
- **t-alpha:** 0.0351026483066376

---

---

570

- **PDB ID:** 7WS5 | **Chain:** B
- **b-phipsi:** 0.0003626108843711
- **w-rdist:** 0.8790546991700752
- **t-alpha:** 0.0885027242012517

---

---

571

- **PDB ID:** 7JVC | **Chain:** A
- **b-phipsi:** 0.0006352295387241
- **w-rdist:** 0.8402020718112826
- **t-alpha:** 0.0680214444040752

---

---

572

- **PDB ID:** 3PRX | **Chain:** A
- **b-phipsi:** 0.0094882631898496
- **w-rdist:** 0.4397641989179787
- **t-alpha:** 0.0669972798838651

---

---

573

- **PDB ID:** 7KJ4 | **Chain:** B
- **b-phipsi:** 0.0005111469141501
- **w-rdist:** 0.8083284089843802
- **t-alpha:** 0.1405659229116656

---

---

574

- **PDB ID:** 7K8Y | **Chain:** E
- **b-phipsi:** 0.0006165774677416
- **w-rdist:** 0.8317011179754852
- **t-alpha:** 0.0727594979737589

---

---

575

- **PDB ID:** 7SC1 | **Chain:** A
- **b-phipsi:** 0.000845195785297
- **w-rdist:** 0.7800726490922211
- **t-alpha:** 0.0862534621133408

---

---

576

- **PDB ID:** 7FG3 | **Chain:** A
- **b-phipsi:** 0.0194742796003532
- **w-rdist:** 0.437267873772372
- **t-alpha:** 0.0595968523857786

---

---

577

- **PDB ID:** 7CZQ | **Chain:** B
- **b-phipsi:** 0.0058987240155496
- **w-rdist:** 0.4490010259637587
- **t-alpha:** 0.0711330386749458

---

---

578

- **PDB ID:** 7CZU | **Chain:** B
- **b-phipsi:** 0.0058985308061422
- **w-rdist:** 0.4489981818058689
- **t-alpha:** 0.0744417083525081

---

---

579

- **PDB ID:** 7VX9 | **Chain:** B
- **b-phipsi:** 0.0011372587157745
- **w-rdist:** 0.6456807521310471
- **t-alpha:** 0.1654259730530252

---

---

580

- **PDB ID:** 7ZR9 | **Chain:** A
- **b-phipsi:** 0.0006656014911856
- **w-rdist:** 0.8607146065176177
- **t-alpha:** 0.0689655430268945

---

---

581

- **PDB ID:** 7KMK | **Chain:** A
- **b-phipsi:** 0.0011381415016017
- **w-rdist:** 0.7460887191815514
- **t-alpha:** 0.0756227739283255

---

---

582

- **PDB ID:** 7DF4 | **Chain:** B
- **b-phipsi:** 0.0009782344306117
- **w-rdist:** 0.7706753851260664
- **t-alpha:** 0.0872302669785827

---

---

583

- **PDB ID:** 3PRX | **Chain:** C
- **b-phipsi:** 0.0095252449298418
- **w-rdist:** 0.4532222761846343
- **t-alpha:** 0.0736146078897646

---

---

584

- **PDB ID:** 7SBW | **Chain:** B
- **b-phipsi:** 0.0008276256828067
- **w-rdist:** 0.8140683189319132
- **t-alpha:** 0.076922726695275

---

---

585

- **PDB ID:** 7WS4 | **Chain:** B
- **b-phipsi:** 0.0006506706362387
- **w-rdist:** 0.8364518076190152
- **t-alpha:** 0.0951197353930601

---

---

586

- **PDB ID:** 7CYP | **Chain:** C
- **b-phipsi:** 0.0167397025873237
- **w-rdist:** 0.4512527015860115
- **t-alpha:** 0.0708593084227287

---

---
