# Supplementary material for: Identifying and profiling structural similarities between Spike of SARS-CoV-2 and other viral or host proteins with Machaon
Source: Commun Biol. 2023 Jul 19;6:752. doi: 10.1038/s42003-023-05076-7 (PMC10356814; doi:10.1038/s42003-023-05076-7)
Supplement: Supplementary file 8 — Supplementary Data 5 [file 42003_2023_5076_MOESM8_ESM.zip › 6VXX_A_whole_human_exp_dataset/candidates/6VXX_A-merged-h-enriched_report.html]

 

# Structural Comparison Report for 6VXX\_A - whole structures (total: 16)

---

0

- **PDB ID:** 5I5K | **Chain:** A
- **b-phipsi:** 0.0043536099130699
- **w-rdist:** 0.342823563543469
- **t-alpha:** 0.0074438168005386

---

---

1

- **PDB ID:** 5JUY | **Chain:** E
- **b-phipsi:** 0.0061240281814654
- **w-rdist:** 0.4946075514383613
- **t-alpha:** 0.0016569498401739

---

---

2

- **PDB ID:** 5U8R | **Chain:** A
- **b-phipsi:** 0.0045415605452242
- **w-rdist:** 0.5590043023356055
- **t-alpha:** 0.0049877163597098

---

---

3

- **PDB ID:** 5ZAM | **Chain:** A
- **b-phipsi:** 0.0495203465321244
- **w-rdist:** 0.4325816348002631
- **t-alpha:** 0.0016540840807526

---

---

4

- **PDB ID:** 6VHH | **Chain:** A
- **b-phipsi:** 0.0036982175665957
- **w-rdist:** 0.7031884419638592
- **t-alpha:** 0.0066169305252397

---

---

5

- **PDB ID:** 6J4Z | **Chain:** A
- **b-phipsi:** 0.0217266503234563
- **w-rdist:** 0.5927664648113062
- **t-alpha:** 0.0049627974633414

---

---

6

- **PDB ID:** 7BAN | **Chain:** A
- **b-phipsi:** 0.0041146014703666
- **w-rdist:** 0.4072591382759139
- **t-alpha:** 0.0521092782942007

---

---

7

- **PDB ID:** 5LCW | **Chain:** A
- **b-phipsi:** 0.0286335997351462
- **w-rdist:** 0.61912142810699
- **t-alpha:** 0.0033085120789013

---

---

8

- **PDB ID:** 6J4W | **Chain:** B
- **b-phipsi:** 0.0044111376826172
- **w-rdist:** 0.9311244331801792
- **t-alpha:** 0.0016540840807526

---

---

9

- **PDB ID:** 5IYC | **Chain:** A
- **b-phipsi:** 0.0283410105968308
- **w-rdist:** 0.5821441377559046
- **t-alpha:** 0.0082713823222237

---

---

10

- **PDB ID:** 4NEN | **Chain:** A
- **b-phipsi:** 0.0106169117458048
- **w-rdist:** 0.3556922281277379
- **t-alpha:** 0.0324511081324321

---

---

11

- **PDB ID:** 5O9Z | **Chain:** C
- **b-phipsi:** 0.0322939701084823
- **w-rdist:** 0.271928303360898
- **t-alpha:** 0.0159666566861913

---

---

12

- **PDB ID:** 6W5S | **Chain:** A
- **b-phipsi:** 0.0484808942296313
- **w-rdist:** 0.5540390276198479
- **t-alpha:** 0.0091819121329137

---

---

13

- **PDB ID:** 7POV | **Chain:** A
- **b-phipsi:** 0.005230871249445
- **w-rdist:** 0.7337945705304884
- **t-alpha:** 0.025640883005656

---

---

14

- **PDB ID:** 6UIA | **Chain:** B
- **b-phipsi:** 0.0207975290678138
- **w-rdist:** 0.7442104292634261
- **t-alpha:** 0.0108695004677259

---

---

15

- **PDB ID:** 4ZXB | **Chain:** E
- **b-phipsi:** 0.0049866264164505
- **w-rdist:** 0.764614935891152
- **t-alpha:** 0.0202533741750159

---

---
