# Supplementary material for: Identifying and profiling structural similarities between Spike of SARS-CoV-2 and other viral or host proteins with Machaon
Source: Commun Biol. 2023 Jul 19;6:752. doi: 10.1038/s42003-023-05076-7 (PMC10356814; doi:10.1038/s42003-023-05076-7)
Supplement: Supplementary file 9 — Supplementary Data 6 [file 42003_2023_5076_MOESM9_ESM.zip › 6VXX_A_whole_alphafold4_dataset/candidates/6VXX_A-merged-enriched_eval_report.html]

 

# Structural Comparison Report for 6VXX\_A - whole structures (total: 100)

---

1

- **Protein name:** Macrophage mannose receptor 1
- **Organism:** Homo sapiens
- **Uniprot Accession Number:** P22897
- **Protein sequence length:** 1456 aa
- **1D identity (%):** 16.0
- **1D identity (%) [Gaps excluded]:** 26.18
- **1D identity - Alignment Gaps:** 659
- **1D aligned content (<aminoacid>:%):** {'M': 0.37, 'V': 5.9, 'F': 4.8, 'P': 8.12, 'L': 8.86, 'C': 4.06, 'A': 4.8, 'N': 8.86, 'K': 3.32, 'D': 6.64, 'G': 9.23, 'T': 7.38, 'W': 1.85, 'E': 5.17, 'Y': 4.06, 'S': 8.49, 'R': 1.85, 'Q': 1.85, 'I': 4.06, 'H': 0.37}
- **Common reported functions (%):** 0.0
- **Common reported locations (%):** 20.0
- **Common reported processes (%):** 0.0

- **AF ID:** P22897
- **Chain:** A
- **Protein length:** 1456 aa
- **Resolution:** N/A
- **b-phipsi:** 0.0074
- **w-rdist:** 0.231848
- **t-alpha:** 0.0075
- **Chemical similarity (Tanimoto Index) (%):** 86.09
- **1D identity (%) [PDB]:** 4.13
- **1D identity (%) [Gaps excluded][PDB]:** 68.84
- **1D identity - Alignment Gaps [PDB]:** 2163
- **1D aligned content [PDB] (<aminoacid>:%):** {'S': 12.63, 'Q': 6.32, 'I': 5.26, 'L': 12.63, 'P': 2.11, 'K': 5.26, 'F': 4.21, 'E': 2.11, 'D': 4.21, 'N': 10.53, 'G': 7.37, 'T': 8.42, 'A': 11.58, 'Y': 3.16, 'R': 1.05, 'V': 3.16}
- **2D identity (%) [PDB]:** 31.3
- **2D identity (%) [Gaps excluded][PDB]:** 85.6
- **2D identity - Alignment Gaps [PDB]:** 1133
- **2D aligned content [PDB] (<2D-fold>:%):** {'.': 24.51, 'T': 11.45, 'E': 39.71, 'H': 22.54, 'G': 1.61, 'B': 0.18}
- **3D similarity (TM-Score) (%) [PDB]:** 24.38

- **Gene name:** MRC1
- **Entrez ID:** 4360
- **RefSeq ID:** NM\_002438
- **Transcript sequence length:** 5188
- **5-UTR|CDS|3-UTR identity (%):** 26.79 | 45.7 | 26.32
- **5-UTR|CDS|3-UTR identity (%) [Gaps excluded]:** 72.82 | 74.35 | 82.22
- **5-UTR|CDS|3-UTR identity [Alignment Gaps]:** 177 | 1955 | 478
- **5-UTR aligned content (<base>:%):** {'C': 26.67, 'A': 20.0, 'G': 25.33, 'T': 28.0}
- **CDS aligned content (<base>:%):** {'A': 31.61, 'T': 31.57, 'G': 19.28, 'C': 17.55}
- **3-UTR aligned content (<base>:%):** {'C': 14.59, 'A': 42.16, 'T': 23.78, 'G': 19.46}

**Uniprot Description:**  
  
 Mediates the endocytosis of glycoproteins by macrophages. Binds both sulfated and non-sulfated polysaccharide chains.   
  
(Microbial infection) Interacts with Dengue virus.   
  
 **Gene Ontology Information:**

Molecular Function

- cargo receptor activity
- mannose binding
- signaling receptor activity
- transmembrane signaling receptor activity
- virus receptor activity

Location

- cell surface
- endosome membrane
- plasma membrane

Biological process

- cellular response to interleukin-4
- cellular response to lipopolysaccharide
- cellular response to interferon-gamma
- receptor-mediated endocytosis

---

2

- **Protein name:** Thyroglobulin
- **Organism:** Homo sapiens
- **Uniprot Accession Number:** P01266
- **Protein sequence length:** 2768 aa
- **1D identity (%):** 13.05
- **1D identity (%) [Gaps excluded]:** 28.57
- **1D identity - Alignment Gaps:** 1507
- **1D aligned content (<aminoacid>:%):** {'M': 0.28, 'L': 11.6, 'S': 9.12, 'C': 4.7, 'N': 4.42, 'P': 5.8, 'T': 7.46, 'D': 5.25, 'V': 6.35, 'Q': 5.52, 'W': 1.1, 'G': 11.05, 'F': 6.91, 'Y': 2.76, 'A': 6.35, 'H': 0.83, 'K': 2.21, 'E': 3.59, 'I': 2.49, 'R': 2.21}
- **Common reported functions (%):** 0.0
- **Common reported locations (%):** 0.0
- **Common reported processes (%):** 0.0

- **AF ID:** P01266
- **Chain:** A
- **Protein length:** 1368 aa
- **Resolution:** N/A
- **b-phipsi:** 0.006037
- **w-rdist:** 0.258865
- **t-alpha:** 0.019393
- **Chemical similarity (Tanimoto Index) (%):** 86.24
- **1D identity (%) [PDB]:** 3.29
- **1D identity (%) [Gaps excluded][PDB]:** 74.0
- **1D identity - Alignment Gaps [PDB]:** 2151
- **1D aligned content [PDB] (<aminoacid>:%):** {'I': 8.11, 'Q': 9.46, 'D': 4.05, 'S': 13.51, 'L': 16.22, 'A': 14.86, 'G': 4.05, 'K': 5.41, 'V': 4.05, 'N': 6.76, 'F': 1.35, 'R': 5.41, 'P': 2.7, 'E': 2.7, 'T': 1.35}
- **2D identity (%) [PDB]:** 36.53
- **2D identity (%) [Gaps excluded][PDB]:** 87.05
- **2D identity - Alignment Gaps [PDB]:** 961
- **2D aligned content [PDB] (<2D-fold>:%):** {'E': 40.66, 'T': 10.58, '.': 19.5, 'H': 28.1, 'G': 1.16}
- **3D similarity (TM-Score) (%) [PDB]:** 23.14

- **Gene name:** TG
- **Entrez ID:** 280706
- **RefSeq ID:** N/A
- **Sequence length:** N/A
- **5-UTR|CDS|3-UTR identity (%):** N/A | N/A | N/A
- **5-UTR|CDS|3-UTR identity (%) [Gaps excluded]:** N/A | N/A | N/A
- **5-UTR|CDS|3-UTR identity [Alignment Gaps]:** N/A | N/A | N/A
- **5-UTR aligned content (<base>:%):** N/A
- **CDS aligned content (<base>:%):** N/A
- **3-UTR aligned content (<base>:%):** N/A

**Uniprot Description:**  
  
 Acts as a substrate for the production of iodinated thyroid hormones thyroxine (T4) and triiodothyronine (T3) (PubMed:32025030, PubMed:17532758). The synthesis of T3 and T4 involves iodination of selected tyrosine residues of TG/thyroglobulin followed by their oxidative coupling in the thyroid follicle lumen (PubMed:32025030). Following TG re-internalization and lysosomal-mediated proteolysis, T3 and T4 are released from the polypeptide backbone leading to their secretion into the bloodstream (PubMed:32025030). One dimer produces 7 thyroid hormone molecules (PubMed:32025030).   
  
Monomer (PubMed:32025030). Homodimer (via ChEL region); occurs in the endoplasmic reticulum and is required for export to the Golgi apparatus (PubMed:32025030). Homooligomer; disulfide-linked; stored in this form in the thyroid follicle lumen (PubMed:8626858).   
  
 **Gene Ontology Information:**

Molecular Function

- histone binding
- hormone activity

Location

- extracellular space

Biological process

- hormone biosynthetic process
- thyroid hormone generation

---

3

- **Protein name:** Pregnancy zone protein
- **Organism:** Homo sapiens
- **Uniprot Accession Number:** P20742
- **Protein sequence length:** 1482 aa
- **1D identity (%):** 18.08
- **1D identity (%) [Gaps excluded]:** 24.57
- **1D identity - Alignment Gaps:** 419
- **1D aligned content (<aminoacid>:%):** {'M': 1.05, 'L': 9.06, 'V': 10.45, 'S': 7.32, 'T': 8.36, 'Q': 5.92, 'P': 6.27, 'G': 10.1, 'F': 5.57, 'H': 0.7, 'E': 3.48, 'N': 8.01, 'I': 5.92, 'Y': 1.74, 'K': 4.18, 'R': 2.09, 'A': 5.57, 'C': 2.44, 'D': 1.39, 'W': 0.35}
- **Common reported functions (%):** 0.0
- **Common reported locations (%):** 0.0
- **Common reported processes (%):** 0.0

- **AF ID:** P20742
- **Chain:** A
- **Protein length:** 1482 aa
- **Resolution:** N/A
- **b-phipsi:** 0.006927
- **w-rdist:** 0.302836
- **t-alpha:** 0.07754
- **Chemical similarity (Tanimoto Index) (%):** 86.07
- **1D identity (%) [PDB]:** 5.13
- **1D identity (%) [Gaps excluded][PDB]:** 62.9
- **1D identity - Alignment Gaps [PDB]:** 2093
- **1D aligned content [PDB] (<aminoacid>:%):** {'L': 14.53, 'Q': 6.84, 'Y': 1.71, 'S': 11.97, 'F': 5.98, 'T': 4.27, 'N': 8.55, 'R': 0.85, 'I': 9.4, 'A': 6.84, 'V': 5.98, 'E': 4.27, 'D': 1.71, 'K': 5.98, 'P': 3.42, 'G': 5.13, 'W': 0.85, 'M': 1.71}
- **2D identity (%) [PDB]:** 40.56
- **2D identity (%) [Gaps excluded][PDB]:** 85.05
- **2D identity - Alignment Gaps [PDB]:** 873
- **2D aligned content [PDB] (<2D-fold>:%):** {'.': 18.76, 'T': 11.96, 'E': 44.9, 'H': 24.37}
- **3D similarity (TM-Score) (%) [PDB]:** 22.42

- **Gene name:** PZP
- **Entrez ID:** N/A
- **RefSeq ID:** N/A
- **Sequence length:** N/A
- **5-UTR|CDS|3-UTR identity (%):** N/A | N/A | N/A
- **5-UTR|CDS|3-UTR identity (%) [Gaps excluded]:** N/A | N/A | N/A
- **5-UTR|CDS|3-UTR identity [Alignment Gaps]:** N/A | N/A | N/A
- **5-UTR aligned content (<base>:%):** N/A
- **CDS aligned content (<base>:%):** N/A
- **3-UTR aligned content (<base>:%):** N/A

**Uniprot Description:**  
  
 Is able to inhibit all four classes of proteinases by a unique 'trapping' mechanism. This protein has a peptide stretch, called the 'bait region' which contains specific cleavage sites for different proteinases. When a proteinase cleaves the bait region, a conformational change is induced in the protein which traps the proteinase. The entrapped enzyme remains active against low molecular weight substrates (activity against high molecular weight substrates is greatly reduced). Following cleavage in the bait region a thioester bond is hydrolyzed and mediates the covalent binding of the protein to the proteinase.   
  
Homotetramer, which consists of two pairs of disulfide-linked chains.   
  
 **Gene Ontology Information:**

Molecular Function

- endopeptidase inhibitor activity
- protease binding
- serine-type endopeptidase inhibitor activity

Location

- blood microparticle
- extracellular exosome
- extracellular region

Biological process

- female pregnancy
- negative regulation of peptidase activity

---

4

- **Protein name:** Contactin-associated protein-like 3
- **Organism:** Homo sapiens
- **Uniprot Accession Number:** Q9BZ76
- **Protein sequence length:** 1288 aa
- **1D identity (%):** 16.92
- **1D identity (%) [Gaps excluded]:** 24.19
- **1D identity - Alignment Gaps:** 453
- **1D aligned content (<aminoacid>:%):** {'M': 1.18, 'L': 10.98, 'P': 5.49, 'V': 5.88, 'R': 2.75, 'S': 10.2, 'N': 5.1, 'W': 1.18, 'A': 4.71, 'G': 13.73, 'F': 7.06, 'D': 5.88, 'I': 4.31, 'T': 5.1, 'E': 4.31, 'Y': 3.92, 'K': 2.75, 'C': 3.53, 'H': 0.39, 'Q': 1.57}
- **Common reported functions (%):** 0.0
- **Common reported locations (%):** 0.0
- **Common reported processes (%):** 0.0

- **AF ID:** Q9BZ76
- **Chain:** A
- **Protein length:** 1288 aa
- **Resolution:** N/A
- **b-phipsi:** 0.012899
- **w-rdist:** 0.263064
- **t-alpha:** 0.00332
- **Chemical similarity (Tanimoto Index) (%):** 85.89
- **1D identity (%) [PDB]:** 2.61
- **1D identity (%) [Gaps excluded][PDB]:** 68.67
- **1D identity - Alignment Gaps [PDB]:** 2105
- **1D aligned content [PDB] (<aminoacid>:%):** {'P': 7.02, 'T': 10.53, 'E': 5.26, 'S': 12.28, 'I': 3.51, 'F': 8.77, 'N': 10.53, 'L': 5.26, 'V': 8.77, 'A': 8.77, 'W': 1.75, 'R': 3.51, 'C': 1.75, 'D': 7.02, 'K': 1.75, 'G': 3.51}
- **2D identity (%) [PDB]:** 31.66
- **2D identity (%) [Gaps excluded][PDB]:** 83.92
- **2D identity - Alignment Gaps [PDB]:** 1027
- **2D aligned content [PDB] (<2D-fold>:%):** {'.': 26.05, 'E': 48.66, 'T': 13.03, 'B': 0.19, 'H': 12.07}
- **3D similarity (TM-Score) (%) [PDB]:** 23.15

- **Gene name:** CNTNAP3
- **Entrez ID:** 728577
- **RefSeq ID:** NM\_033655
- **Transcript sequence length:** 13150
- **5-UTR|CDS|3-UTR identity (%):** 27.01 | 41.9 | 2.45
- **5-UTR|CDS|3-UTR identity (%) [Gaps excluded]:** 78.72 | 73.61 | 98.25
- **5-UTR|CDS|3-UTR identity [Alignment Gaps]:** 180 | 2111 | 8951
- **5-UTR aligned content (<base>:%):** {'G': 40.54, 'C': 32.43, 'A': 16.22, 'T': 10.81}
- **CDS aligned content (<base>:%):** {'T': 29.96, 'G': 20.56, 'A': 27.62, 'C': 21.87}
- **3-UTR aligned content (<base>:%):** {'C': 15.11, 'A': 41.33, 'T': 23.56, 'G': 20.0}

**Uniprot Description:**  
  
 N/A N/A   
  
 **Gene Ontology Information:**

Molecular Function   
  
N/A

Location

- membrane

Biological process

- cell adhesion

---

5

- **Protein name:** Alpha-2-macroglobulin
- **Organism:** Homo sapiens
- **Uniprot Accession Number:** P01023
- **Protein sequence length:** 1474 aa
- **1D identity (%):** 17.94
- **1D identity (%) [Gaps excluded]:** 24.61
- **1D identity - Alignment Gaps:** 431
- **1D aligned content (<aminoacid>:%):** {'M': 1.05, 'L': 12.28, 'V': 9.82, 'P': 8.42, 'S': 6.67, 'T': 8.42, 'G': 8.42, 'Y': 4.56, 'F': 6.32, 'H': 0.7, 'N': 5.96, 'K': 4.56, 'E': 4.56, 'Q': 2.46, 'R': 3.16, 'D': 2.46, 'A': 5.96, 'I': 2.81, 'C': 1.4}
- **Common reported functions (%):** 0.0
- **Common reported locations (%):** 0.0
- **Common reported processes (%):** 0.0

- **AF ID:** P01023
- **Chain:** A
- **Protein length:** 1474 aa
- **Resolution:** N/A
- **b-phipsi:** 0.008979
- **w-rdist:** 0.288876
- **t-alpha:** 0.099091
- **Chemical similarity (Tanimoto Index) (%):** 86.03
- **1D identity (%) [PDB]:** 3.98
- **1D identity (%) [Gaps excluded][PDB]:** 63.89
- **1D identity - Alignment Gaps [PDB]:** 2169
- **1D aligned content [PDB] (<aminoacid>:%):** {'K': 6.52, 'N': 8.7, 'T': 7.61, 'Q': 10.87, 'E': 4.35, 'V': 3.26, 'F': 5.43, 'I': 6.52, 'Y': 2.17, 'P': 6.52, 'D': 3.26, 'G': 5.43, 'S': 8.7, 'L': 8.7, 'M': 2.17, 'A': 8.7, 'W': 1.09}
- **2D identity (%) [PDB]:** 41.77
- **2D identity (%) [Gaps excluded][PDB]:** 83.84
- **2D identity - Alignment Gaps [PDB]:** 823
- **2D aligned content [PDB] (<2D-fold>:%):** {'.': 17.66, 'E': 44.82, 'T': 12.12, 'B': 0.29, 'G': 0.88, 'H': 24.23}
- **3D similarity (TM-Score) (%) [PDB]:** 20.82

- **Gene name:** A2M
- **Entrez ID:** 513856
- **RefSeq ID:** NM\_001347425
- **Transcript sequence length:** 4450
- **5-UTR|CDS|3-UTR identity (%):** 40.25 | 45.18 | 28.81
- **5-UTR|CDS|3-UTR identity (%) [Gaps excluded]:** 71.56 | 75.14 | 69.31
- **5-UTR|CDS|3-UTR identity [Alignment Gaps]:** 175 | 1941 | 142
- **5-UTR aligned content (<base>:%):** {'A': 21.12, 'G': 20.5, 'T': 31.68, 'C': 26.71}
- **CDS aligned content (<base>:%):** {'A': 30.05, 'C': 21.36, 'T': 28.91, 'G': 19.68}
- **3-UTR aligned content (<base>:%):** {'A': 27.14, 'G': 28.57, 'C': 14.29, 'T': 30.0}

**Uniprot Description:**  
  
 Is able to inhibit all four classes of proteinases by a unique 'trapping' mechanism. This protein has a peptide stretch, called the 'bait region' which contains specific cleavage sites for different proteinases. When a proteinase cleaves the bait region, a conformational change is induced in the protein which traps the proteinase. The entrapped enzyme remains active against low molecular weight substrates (activity against high molecular weight substrates is greatly reduced). Following cleavage in the bait region, a thioester bond is hydrolyzed and mediates the covalent binding of the protein to the proteinase.   
  
Homotetramer; disulfide-linked.   
  
 **Gene Ontology Information:**

Molecular Function

- endopeptidase inhibitor activity
- protease binding
- serine-type endopeptidase inhibitor activity

Location

- extracellular space

Biological process

- negative regulation of peptidase activity

---

6

- **Protein name:** CD109 antigen
- **Organism:** Homo sapiens
- **Uniprot Accession Number:** Q6YHK3
- **Protein sequence length:** 1445 aa
- **1D identity (%):** 16.51
- **1D identity (%) [Gaps excluded]:** 23.17
- **1D identity - Alignment Gaps:** 456
- **1D aligned content (<aminoacid>:%):** {'P': 6.49, 'L': 9.16, 'G': 7.25, 'F': 5.73, 'T': 8.78, 'I': 4.96, 'A': 4.2, 'N': 7.25, 'V': 7.63, 'E': 3.82, 'Y': 6.49, 'S': 8.78, 'K': 5.34, 'D': 6.87, 'Q': 2.29, 'C': 2.67, 'W': 0.38, 'H': 0.76, 'R': 1.15}
- **Common reported functions (%):** 0.0
- **Common reported locations (%):** 20.0
- **Common reported processes (%):** 0.0

- **AF ID:** Q6YHK3
- **Chain:** A
- **Protein length:** 1445 aa
- **Resolution:** N/A
- **b-phipsi:** 0.011177
- **w-rdist:** 0.289346
- **t-alpha:** 0.105119
- **Chemical similarity (Tanimoto Index) (%):** 86.19
- **1D identity (%) [PDB]:** 3.88
- **1D identity (%) [Gaps excluded][PDB]:** 65.93
- **1D identity - Alignment Gaps [PDB]:** 2158
- **1D aligned content [PDB] (<aminoacid>:%):** {'P': 4.49, 'L': 13.48, 'T': 8.99, 'D': 4.49, 'E': 3.37, 'I': 4.49, 'A': 10.11, 'Y': 2.25, 'S': 11.24, 'G': 5.62, 'W': 1.12, 'F': 4.49, 'Q': 8.99, 'R': 3.37, 'N': 5.62, 'V': 4.49, 'K': 3.37}
- **2D identity (%) [PDB]:** 41.77
- **2D identity (%) [Gaps excluded][PDB]:** 85.0
- **2D identity - Alignment Gaps [PDB]:** 828
- **2D aligned content [PDB] (<2D-fold>:%):** {'.': 18.24, 'T': 11.32, 'E': 44.56, 'H': 25.29, 'B': 0.15, 'G': 0.44}
- **3D similarity (TM-Score) (%) [PDB]:** 25.43

- **Gene name:** CD109
- **Entrez ID:** 135228
- **RefSeq ID:** N/A
- **Sequence length:** N/A
- **5-UTR|CDS|3-UTR identity (%):** N/A | N/A | N/A
- **5-UTR|CDS|3-UTR identity (%) [Gaps excluded]:** N/A | N/A | N/A
- **5-UTR|CDS|3-UTR identity [Alignment Gaps]:** N/A | N/A | N/A
- **5-UTR aligned content (<base>:%):** N/A
- **CDS aligned content (<base>:%):** N/A
- **3-UTR aligned content (<base>:%):** N/A

**Uniprot Description:**  
  
 Modulates negatively TGFB1 signaling in keratinocytes.   
  
Heterodimer; disulfide-linked. Interacts with TGFB1 and TGFBR1. Forms a heteromeric complex with TGFBR1, TGFBR2 and TGFBR3 in a ligand-independent manner.   
  
 **Gene Ontology Information:**

Molecular Function

- serine-type endopeptidase inhibitor activity
- transforming growth factor beta binding

Location

- cell surface
- cytosol
- extracellular region
- extracellular space
- plasma membrane
- platelet alpha granule membrane

Biological process

- hair follicle development
- keratinocyte proliferation
- negative regulation of keratinocyte proliferation
- negative regulation of peptidase activity
- negative regulation of protein phosphorylation
- negative regulation of stem cell proliferation
- negative regulation of transforming growth factor beta receptor signaling pathway
- negative regulation of wound healing
- osteoclast fusion
- protein phosphorylation
- regulation of keratinocyte differentiation
- stem cell proliferation

---

7

- **Protein name:** Hypoxia up-regulated protein 1
- **Organism:** Homo sapiens
- **Uniprot Accession Number:** Q9Y4L1
- **Protein sequence length:** 999 aa
- **1D identity (%):** 12.81
- **1D identity (%) [Gaps excluded]:** 20.16
- **1D identity - Alignment Gaps:** 506
- **1D aligned content (<aminoacid>:%):** {'R': 4.49, 'L': 10.11, 'D': 6.18, 'G': 10.67, 'V': 7.87, 'P': 7.3, 'K': 7.3, 'N': 3.93, 'E': 4.49, 'A': 7.3, 'T': 6.18, 'F': 6.18, 'Q': 4.49, 'S': 6.18, 'Y': 2.81, 'I': 3.37, 'C': 0.56, 'H': 0.56}
- **Common reported functions (%):** 0.0
- **Common reported locations (%):** 0.0
- **Common reported processes (%):** 0.0

- **AF ID:** Q9Y4L1
- **Chain:** A
- **Protein length:** 999 aa
- **Resolution:** N/A
- **b-phipsi:** 0.012004
- **w-rdist:** 0.292817
- **t-alpha:** 0.147229
- **Chemical similarity (Tanimoto Index) (%):** 83.43
- **1D identity (%) [PDB]:** 1.86
- **1D identity (%) [Gaps excluded][PDB]:** 70.59
- **1D identity - Alignment Gaps [PDB]:** 1880
- **1D aligned content [PDB] (<aminoacid>:%):** {'K': 16.67, 'T': 5.56, 'P': 13.89, 'I': 2.78, 'D': 8.33, 'G': 8.33, 'F': 11.11, 'N': 5.56, 'S': 5.56, 'Q': 2.78, 'L': 16.67, 'V': 2.78}
- **2D identity (%) [PDB]:** 32.3
- **2D identity (%) [Gaps excluded][PDB]:** 88.49
- **2D identity - Alignment Gaps [PDB]:** 922
- **2D aligned content [PDB] (<2D-fold>:%):** {'T': 9.59, '.': 11.3, 'E': 40.72, 'H': 38.38}
- **3D similarity (TM-Score) (%) [PDB]:** 20.34

- **Gene name:** HYOU1
- **Entrez ID:** 100689308
- **RefSeq ID:** N/A
- **Sequence length:** N/A
- **5-UTR|CDS|3-UTR identity (%):** N/A | N/A | N/A
- **5-UTR|CDS|3-UTR identity (%) [Gaps excluded]:** N/A | N/A | N/A
- **5-UTR|CDS|3-UTR identity [Alignment Gaps]:** N/A | N/A | N/A
- **5-UTR aligned content (<base>:%):** N/A
- **CDS aligned content (<base>:%):** N/A
- **3-UTR aligned content (<base>:%):** N/A

**Uniprot Description:**  
  
 Has a pivotal role in cytoprotective cellular mechanisms triggered by oxygen deprivation. May play a role as a molecular chaperone and participate in protein folding.   
  
Part of a large chaperone multiprotein complex comprising DNAJB11, HSP90B1, HSPA5, HYOU, PDIA2, PDIA4, PDIA6, PPIB, SDF2L1, UGGT1 and very small amounts of ERP29, but not, or at very low levels, CALR nor CANX.   
  
 **Gene Ontology Information:**

Molecular Function

- ATP binding
- ATP-dependent protein folding chaperone

Location

- endoplasmic reticulum lumen

Biological process   
  
N/A

---

8

- **Protein name:** Probable E3 ubiquitin-protein ligase HECTD4
- **Organism:** Homo sapiens
- **Uniprot Accession Number:** Q9Y4D8
- **Protein sequence length:** 3996 aa
- **1D identity (%):** 10.08
- **1D identity (%) [Gaps excluded]:** 32.04
- **1D identity - Alignment Gaps:** 2747
- **1D aligned content (<aminoacid>:%):** {'M': 0.5, 'V': 5.2, 'F': 5.45, 'L': 11.88, 'S': 7.67, 'N': 6.68, 'T': 6.93, 'P': 8.17, 'Y': 3.22, 'R': 2.48, 'D': 6.44, 'H': 1.49, 'I': 1.98, 'G': 8.66, 'K': 4.95, 'E': 4.7, 'Q': 2.23, 'A': 5.69, 'C': 4.21, 'W': 1.49}
- **Common reported functions (%):** 0.0
- **Common reported locations (%):** 0.0
- **Common reported processes (%):** 0.0

- **AF ID:** Q9Y4D8
- **Chain:** A
- **Protein length:** 1400 aa
- **Resolution:** N/A
- **b-phipsi:** 0.016173
- **w-rdist:** 0.279131
- **t-alpha:** 0.093128
- **Chemical similarity (Tanimoto Index) (%):** 84.09
- **1D identity (%) [PDB]:** 3.67
- **1D identity (%) [Gaps excluded][PDB]:** 69.75
- **1D identity - Alignment Gaps [PDB]:** 2145
- **1D aligned content [PDB] (<aminoacid>:%):** {'L': 14.46, 'V': 7.23, 'S': 9.64, 'T': 9.64, 'K': 8.43, 'D': 6.02, 'I': 7.23, 'M': 1.2, 'Y': 1.2, 'G': 6.02, 'E': 3.61, 'Q': 6.02, 'F': 3.61, 'N': 3.61, 'R': 1.2, 'A': 4.82, 'P': 6.02}
- **2D identity (%) [PDB]:** 31.62
- **2D identity (%) [Gaps excluded][PDB]:** 83.77
- **2D identity - Alignment Gaps [PDB]:** 1077
- **2D aligned content [PDB] (<2D-fold>:%):** {'.': 24.68, 'E': 29.8, 'T': 14.63, 'H': 30.71, 'B': 0.18}
- **3D similarity (TM-Score) (%) [PDB]:** 23.28

- **Gene name:** HECTD4
- **Entrez ID:** N/A
- **RefSeq ID:** N/A
- **Sequence length:** N/A
- **5-UTR|CDS|3-UTR identity (%):** N/A | N/A | N/A
- **5-UTR|CDS|3-UTR identity (%) [Gaps excluded]:** N/A | N/A | N/A
- **5-UTR|CDS|3-UTR identity [Alignment Gaps]:** N/A | N/A | N/A
- **5-UTR aligned content (<base>:%):** N/A
- **CDS aligned content (<base>:%):** N/A
- **3-UTR aligned content (<base>:%):** N/A

**Uniprot Description:**  
  
 E3 ubiquitin-protein ligase which accepts ubiquitin from an E2 ubiquitin-conjugating enzyme in the form of a thioester and then directly transfers the ubiquitin to targeted substrates. N/A   
  
 **Gene Ontology Information:**

Molecular Function

- ubiquitin-protein transferase activity

Location

- membrane

Biological process

- glucose homeostasis
- glucose metabolic process

---

9

- **Protein name:** Fibrocystin
- **Organism:** Homo sapiens
- **Uniprot Accession Number:** P08F94
- **Protein sequence length:** 4074 aa
- **1D identity (%):** 11.28
- **1D identity (%) [Gaps excluded]:** 36.62
- **1D identity - Alignment Gaps:** 2829
- **1D aligned content (<aminoacid>:%):** {'M': 0.43, 'L': 10.85, 'V': 6.51, 'P': 7.38, 'S': 6.72, 'Q': 3.04, 'C': 4.56, 'T': 7.38, 'R': 3.9, 'A': 4.77, 'F': 7.16, 'Y': 3.9, 'D': 3.9, 'K': 1.74, 'H': 1.08, 'N': 4.77, 'W': 1.3, 'G': 12.15, 'E': 2.6, 'I': 5.86}
- **Common reported functions (%):** 0.0
- **Common reported locations (%):** 0.0
- **Common reported processes (%):** 0.0

- **AF ID:** P08F94
- **Chain:** A
- **Protein length:** 1400 aa
- **Resolution:** N/A
- **b-phipsi:** 0.019974
- **w-rdist:** 0.266973
- **t-alpha:** 0.060526
- **Chemical similarity (Tanimoto Index) (%):** 85.56
- **1D identity (%) [PDB]:** 3.2
- **1D identity (%) [Gaps excluded][PDB]:** 73.0
- **1D identity - Alignment Gaps [PDB]:** 2183
- **1D aligned content [PDB] (<aminoacid>:%):** {'L': 15.07, 'Q': 10.96, 'F': 1.37, 'N': 4.11, 'S': 13.7, 'A': 10.96, 'I': 9.59, 'T': 8.22, 'G': 2.74, 'V': 5.48, 'D': 2.74, 'R': 5.48, 'P': 1.37, 'E': 5.48, 'Y': 1.37, 'M': 1.37}
- **2D identity (%) [PDB]:** 31.88
- **2D identity (%) [Gaps excluded][PDB]:** 83.59
- **2D identity - Alignment Gaps [PDB]:** 1067
- **2D aligned content [PDB] (<2D-fold>:%):** {'E': 56.0, '.': 26.55, 'T': 14.91, 'G': 1.64, 'H': 0.91}
- **3D similarity (TM-Score) (%) [PDB]:** 22.22

- **Gene name:** PKHD1
- **Entrez ID:** 474934
- **RefSeq ID:** N/A
- **Sequence length:** N/A
- **5-UTR|CDS|3-UTR identity (%):** N/A | N/A | N/A
- **5-UTR|CDS|3-UTR identity (%) [Gaps excluded]:** N/A | N/A | N/A
- **5-UTR|CDS|3-UTR identity [Alignment Gaps]:** N/A | N/A | N/A
- **5-UTR aligned content (<base>:%):** N/A
- **CDS aligned content (<base>:%):** N/A
- **3-UTR aligned content (<base>:%):** N/A

**Uniprot Description:**  
  
 Promotes ciliogenesis in renal epithelial cells and therefore participates in the tubules formation and/ or ensures the maintenance of the architecture of the lumen of the kidney (By similarity). Has an impact on cellular symmetry by ensuring correct bipolar cell division through the regulation of centrosome duplication and mitotic spindle assembly and by maintaining oriented cell division (OCD) during tubular elongation through planar cell polarity (PCP) pathway (PubMed:20554582). During epithelial cell morphogenesis regulates also cell-cell and cell-matrix adhesion and participates in cell motility (By similarity). Promotes cell-cell contact through the positive regulation of PTK2 kinase activity leading to either positive regulation of epithelial cell proliferation through the HRAS/RAF1 pathways, or negative regulation of apoptosis through the PDK1/AKT1 pathway (By similarity). May act in collecting-duct and biliary differentiation (PubMed:11919560). May participate in the regulation of the cholangiocytes proliferation and the CCN2 production in an CXCL8-dependent manner (PubMed:30898581).   
  
Interacts with CAMLG (PubMed:16243292). Interacts with PKD2 (PubMed:18235088). Interacts (via CST) with ARF4; this interaction allows an efficient PKHD1 trafficking to the cilium. Interacts (via CST) with RAB8A; this interaction controls trafficking through the endomembrane systeme and the cilium. Interacts (via CST) with TULP3; this interaction allows PKHD1 trafficking to the cilium (By similarity).   
  
 **Gene Ontology Information:**

Molecular Function   
  
N/A

Location

- apical plasma membrane
- chromosome, centromeric region
- cilium
- endoplasmic reticulum
- extracellular exosome
- Golgi apparatus
- nucleus
- spindle

Biological process

- branching morphogenesis of an epithelial tube
- cell-cell junction organization
- epithelial cell morphogenesis
- establishment of centrosome localization
- establishment of mitotic spindle orientation
- negative regulation of epithelial cell apoptotic process
- positive regulation of epithelial cell proliferation
- regulation of cell adhesion
- regulation of cell-cell adhesion
- regulation of cell-matrix adhesion
- regulation of cholangiocyte proliferation
- regulation of establishment of planar polarity

---

10

- **Protein name:** DmX-like protein 1
- **Organism:** Homo sapiens
- **Uniprot Accession Number:** Q9Y485
- **Protein sequence length:** 3027 aa
- **1D identity (%):** 12.77
- **1D identity (%) [Gaps excluded]:** 31.33
- **1D identity - Alignment Gaps:** 1810
- **1D aligned content (<aminoacid>:%):** {'M': 0.26, 'V': 6.67, 'L': 10.77, 'C': 4.36, 'R': 2.56, 'Q': 3.85, 'P': 6.92, 'Y': 4.36, 'N': 5.38, 'S': 8.72, 'F': 6.67, 'H': 1.79, 'T': 5.9, 'D': 5.38, 'G': 7.18, 'A': 5.38, 'K': 4.36, 'W': 2.05, 'E': 3.85, 'I': 3.59}
- **Common reported functions (%):** 0.0
- **Common reported locations (%):** 0.0
- **Common reported processes (%):** 0.0

- **AF ID:** Q9Y485
- **Chain:** A
- **Protein length:** 1400 aa
- **Resolution:** N/A
- **b-phipsi:** 0.009244
- **w-rdist:** 0.318083
- **t-alpha:** 0.028061
- **Chemical similarity (Tanimoto Index) (%):** 84.05
- **1D identity (%) [PDB]:** 2.29
- **1D identity (%) [Gaps excluded][PDB]:** 76.81
- **1D identity - Alignment Gaps [PDB]:** 2245
- **1D aligned content [PDB] (<aminoacid>:%):** {'T': 5.66, 'Q': 9.43, 'N': 9.43, 'V': 5.66, 'L': 16.98, 'Y': 1.89, 'E': 1.89, 'K': 7.55, 'I': 5.66, 'S': 16.98, 'G': 5.66, 'D': 5.66, 'A': 5.66, 'F': 1.89}
- **2D identity (%) [PDB]:** 33.18
- **2D identity (%) [Gaps excluded][PDB]:** 84.12
- **2D identity - Alignment Gaps [PDB]:** 1035
- **2D aligned content [PDB] (<2D-fold>:%):** {'.': 19.58, 'E': 36.33, 'T': 11.46, 'H': 31.22, 'B': 0.18, 'G': 1.23}
- **3D similarity (TM-Score) (%) [PDB]:** 25.01

- **Gene name:** DMXL1
- **Entrez ID:** 1657
- **RefSeq ID:** N/A
- **Sequence length:** N/A
- **5-UTR|CDS|3-UTR identity (%):** N/A | N/A | N/A
- **5-UTR|CDS|3-UTR identity (%) [Gaps excluded]:** N/A | N/A | N/A
- **5-UTR|CDS|3-UTR identity [Alignment Gaps]:** N/A | N/A | N/A
- **5-UTR aligned content (<base>:%):** N/A
- **CDS aligned content (<base>:%):** N/A
- **3-UTR aligned content (<base>:%):** N/A

**Uniprot Description:**  
  
 N/A N/A   
  
 **Gene Ontology Information:**

Molecular Function   
  
N/A

Location

- RAVE complex

Biological process

- vacuolar acidification

---

11

- **Protein name:** Pleckstrin homology domain-containing family O member 1
- **Organism:** Homo sapiens
- **Uniprot Accession Number:** Q53GL0
- **Protein sequence length:** 409 aa
- **1D identity (%):** 6.1
- **1D identity (%) [Gaps excluded]:** 21.62
- **1D identity - Alignment Gaps:** 942
- **1D aligned content (<aminoacid>:%):** {'K': 6.25, 'N': 5.0, 'G': 7.5, 'E': 5.0, 'V': 2.5, 'D': 6.25, 'C': 1.25, 'S': 12.5, 'A': 6.25, 'Q': 8.75, 'W': 2.5, 'L': 13.75, 'Y': 2.5, 'P': 10.0, 'R': 2.5, 'T': 3.75, 'F': 1.25, 'I': 2.5}
- **Common reported functions (%):** 0.0
- **Common reported locations (%):** 0.0
- **Common reported processes (%):** 0.0

- **AF ID:** Q53GL0
- **Chain:** A
- **Protein length:** 409 aa
- **Resolution:** N/A
- **b-phipsi:** 0.021737
- **w-rdist:** 0.280628
- **t-alpha:** 0.092141
- **Chemical similarity (Tanimoto Index) (%):** 83.6
- **1D identity (%) [PDB]:** 2.22
- **1D identity (%) [Gaps excluded][PDB]:** 73.17
- **1D identity - Alignment Gaps [PDB]:** 1310
- **1D aligned content [PDB] (<aminoacid>:%):** {'N': 10.0, 'Q': 13.33, 'A': 6.67, 'L': 26.67, 'T': 6.67, 'V': 6.67, 'S': 13.33, 'R': 6.67, 'P': 3.33, 'E': 6.67}
- **2D identity (%) [PDB]:** 22.55
- **2D identity (%) [Gaps excluded][PDB]:** 89.96
- **2D identity - Alignment Gaps [PDB]:** 834
- **2D aligned content [PDB] (<2D-fold>:%):** {'.': 19.92, 'T': 9.96, 'E': 18.73, 'H': 51.39}
- **3D similarity (TM-Score) (%) [PDB]:** 11.77

- **Gene name:** PLEKHO1
- **Entrez ID:** 51177
- **RefSeq ID:** N/A
- **Sequence length:** N/A
- **5-UTR|CDS|3-UTR identity (%):** N/A | N/A | N/A
- **5-UTR|CDS|3-UTR identity (%) [Gaps excluded]:** N/A | N/A | N/A
- **5-UTR|CDS|3-UTR identity [Alignment Gaps]:** N/A | N/A | N/A
- **5-UTR aligned content (<base>:%):** N/A
- **CDS aligned content (<base>:%):** N/A
- **3-UTR aligned content (<base>:%):** N/A

**Uniprot Description:**  
  
 Plays a role in the regulation of the actin cytoskeleton through its interactions with actin capping protein (CP). May function to target CK2 to the plasma membrane thereby serving as an adapter to facilitate the phosphorylation of CP by protein kinase 2 (CK2). Appears to target ATM to the plasma membrane. Appears to also inhibit tumor cell growth by inhibiting AKT-mediated cell-survival. Also implicated in PI3K-regulated muscle differentiation, the regulation of AP-1 activity (plasma membrane bound AP-1 regulator that translocates to the nucleus) and the promotion of apoptosis induced by tumor necrosis factor TNF. When bound to PKB, it inhibits it probably by decreasing PKB level of phosphorylation.   
  
Heterodimer or homodimer. Interacts with CK2 and actin capping subunits (capping protein CP-alpha and CP-beta). CKIP1 and CK2 together inhibit the activity of actin capping protein at the barbed ends of actin filaments. Interacts with ATM, IFP35, JUN, JUND, NMI and PI3K. Interacts with AKT1, AKT2 and AKT3 (each isozyme of PKB), PtdIns(3,5)P2, PtdIns(4,5)P2 and PtdIns(3,4,5)P2.   
  
 **Gene Ontology Information:**

Molecular Function   
  
N/A

Location

- cytoplasm
- muscle cell projection membrane
- nucleus
- ruffle membrane

Biological process

- lamellipodium morphogenesis
- myoblast fusion
- myoblast migration
- regulation of cell shape
- regulation of myoblast fusion

---

12

- **Protein name:** Integrin beta-8
- **Organism:** Homo sapiens
- **Uniprot Accession Number:** P26012
- **Protein sequence length:** 769 aa
- **1D identity (%):** 10.04
- **1D identity (%) [Gaps excluded]:** 23.14
- **1D identity - Alignment Gaps:** 806
- **1D aligned content (<aminoacid>:%):** {'G': 11.19, 'L': 4.9, 'T': 6.29, 'N': 9.09, 'S': 10.49, 'R': 4.9, 'C': 5.59, 'V': 6.29, 'F': 5.59, 'K': 8.39, 'I': 4.2, 'P': 4.9, 'A': 4.2, 'Y': 3.5, 'E': 2.8, 'Q': 2.1, 'D': 2.8, 'W': 0.7, 'H': 2.1}
- **Common reported functions (%):** 0.0
- **Common reported locations (%):** 20.0
- **Common reported processes (%):** 0.0

- **AF ID:** P26012
- **Chain:** A
- **Protein length:** 769 aa
- **Resolution:** N/A
- **b-phipsi:** 0.013755
- **w-rdist:** 0.315204
- **t-alpha:** 0.207792
- **Chemical similarity (Tanimoto Index) (%):** 85.7
- **1D identity (%) [PDB]:** 1.81
- **1D identity (%) [Gaps excluded][PDB]:** 81.58
- **1D identity - Alignment Gaps [PDB]:** 1676
- **1D aligned content [PDB] (<aminoacid>:%):** {'L': 16.13, 'V': 6.45, 'S': 19.35, 'N': 6.45, 'A': 6.45, 'D': 9.68, 'R': 9.68, 'P': 6.45, 'E': 6.45, 'I': 6.45, 'G': 3.23, 'Q': 3.23}
- **2D identity (%) [PDB]:** 25.86
- **2D identity (%) [Gaps excluded][PDB]:** 81.52
- **2D identity - Alignment Gaps [PDB]:** 908
- **2D aligned content [PDB] (<2D-fold>:%):** {'T': 15.7, '.': 26.74, 'E': 40.12, 'H': 15.12, 'G': 2.33}
- **3D similarity (TM-Score) (%) [PDB]:** 18.56

- **Gene name:** ITGB8
- **Entrez ID:** 3696
- **RefSeq ID:** N/A
- **Sequence length:** N/A
- **5-UTR|CDS|3-UTR identity (%):** N/A | N/A | N/A
- **5-UTR|CDS|3-UTR identity (%) [Gaps excluded]:** N/A | N/A | N/A
- **5-UTR|CDS|3-UTR identity [Alignment Gaps]:** N/A | N/A | N/A
- **5-UTR aligned content (<base>:%):** N/A
- **CDS aligned content (<base>:%):** N/A
- **3-UTR aligned content (<base>:%):** N/A

**Uniprot Description:**  
  
 Integrin alpha-V:beta-8 (ITGAV:ITGB8) is a receptor for fibronectin (PubMed:1918072). It recognizes the sequence R-G-D in its ligands (PubMed:1918072). Integrin alpha-V:beta-6 (ITGAV:ITGB6) mediates R-G-D-dependent release of transforming growth factor beta-1 (TGF-beta-1) from regulatory Latency-associated peptide (LAP), thereby playing a key role in TGF-beta-1 activation on the surface of activated regulatory T-cells (Tregs) (Probable). Required during vasculogenesis (By similarity).   
  
Heterodimer of an alpha and a beta subunit (PubMed:1918072). Beta-8 (ITGB8) associates with alpha-V (ITGAV) to form ITGAV:ITGB8 (PubMed:1918072, PubMed:22278742). ITGAV:ITGB8 interacts with TGFB1 (PubMed:22278742).   
  
 **Gene Ontology Information:**

Molecular Function

- extracellular matrix protein binding
- integrin binding

Location

- cell surface
- extracellular exosome
- focal adhesion
- integrin alphav-beta8 complex
- integrin complex
- plasma membrane

Biological process

- cartilage development
- cell adhesion
- cell adhesion mediated by integrin
- cell migration
- cell-matrix adhesion
- ganglioside metabolic process
- hard palate development
- immune response
- integrin-mediated signaling pathway
- Langerhans cell differentiation
- negative regulation of gene expression
- placenta blood vessel development
- positive regulation of angiogenesis
- positive regulation of gene expression
- regulation of transforming growth factor beta activation
- response to virus
- transforming growth factor beta receptor signaling pathway
- vasculogenesis

---

13

- **Protein name:** Alpha-2-macroglobulin-like protein 1
- **Organism:** Homo sapiens
- **Uniprot Accession Number:** A8K2U0
- **Protein sequence length:** 1454 aa
- **1D identity (%):** 17.91
- **1D identity (%) [Gaps excluded]:** 25.09
- **1D identity - Alignment Gaps:** 455
- **1D aligned content (<aminoacid>:%):** {'M': 0.7, 'L': 12.28, 'P': 9.82, 'V': 6.67, 'R': 1.4, 'Y': 4.21, 'F': 4.91, 'T': 5.61, 'D': 4.91, 'K': 4.56, 'H': 0.7, 'I': 4.21, 'S': 9.12, 'G': 11.23, 'N': 5.26, 'W': 0.35, 'C': 2.46, 'A': 4.91, 'E': 2.81, 'Q': 3.86}
- **Common reported functions (%):** 0.0
- **Common reported locations (%):** 0.0
- **Common reported processes (%):** 0.0

- **AF ID:** A8K2U0
- **Chain:** A
- **Protein length:** 1454 aa
- **Resolution:** N/A
- **b-phipsi:** 0.010719
- **w-rdist:** 0.31946
- **t-alpha:** 0.050455
- **Chemical similarity (Tanimoto Index) (%):** 86.07
- **1D identity (%) [PDB]:** 3.27
- **1D identity (%) [Gaps excluded][PDB]:** 65.52
- **1D identity - Alignment Gaps [PDB]:** 2205
- **1D aligned content [PDB] (<aminoacid>:%):** {'M': 1.32, 'A': 11.84, 'Q': 10.53, 'Y': 2.63, 'T': 6.58, 'S': 10.53, 'L': 13.16, 'G': 11.84, 'F': 3.95, 'P': 2.63, 'R': 1.32, 'N': 6.58, 'V': 3.95, 'E': 2.63, 'K': 3.95, 'I': 2.63, 'D': 3.95}
- **2D identity (%) [PDB]:** 41.29
- **2D identity (%) [Gaps excluded][PDB]:** 85.28
- **2D identity - Alignment Gaps [PDB]:** 847
- **2D aligned content [PDB] (<2D-fold>:%):** {'E': 45.43, '.': 19.03, 'T': 10.91, 'H': 24.48, 'B': 0.15}
- **3D similarity (TM-Score) (%) [PDB]:** 26.86

- **Gene name:** A2ML1
- **Entrez ID:** 144568
- **RefSeq ID:** N/A
- **Sequence length:** N/A
- **5-UTR|CDS|3-UTR identity (%):** N/A | N/A | N/A
- **5-UTR|CDS|3-UTR identity (%) [Gaps excluded]:** N/A | N/A | N/A
- **5-UTR|CDS|3-UTR identity [Alignment Gaps]:** N/A | N/A | N/A
- **5-UTR aligned content (<base>:%):** N/A
- **CDS aligned content (<base>:%):** N/A
- **3-UTR aligned content (<base>:%):** N/A

**Uniprot Description:**  
  
 Is able to inhibit all four classes of proteinases by a unique 'trapping' mechanism. This protein has a peptide stretch, called the 'bait region' which contains specific cleavage sites for different proteinases. When a proteinase cleaves the bait region, a conformational change is induced in the protein which traps the proteinase. The entrapped enzyme remains active against low molecular weight substrates (activity against high molecular weight substrates is greatly reduced). Following cleavage in the bait region a thioester bond is hydrolyzed and mediates the covalent binding of the protein to the proteinase (By similarity). Displays inhibitory activity against chymotrypsin, papain, thermolysin, subtilisin A and, to a lesser extent, elastase but not trypsin. May play an important role during desquamation by inhibiting extracellular proteases.   
  
Monomer.   
  
 **Gene Ontology Information:**

Molecular Function

- endopeptidase inhibitor activity
- peptidase inhibitor activity
- protease binding
- serine-type endopeptidase inhibitor activity

Location

- extracellular exosome
- extracellular space

Biological process

- negative regulation of peptidase activity
- regulation of endopeptidase activity

---

14

- **Protein name:** 1-phosphatidylinositol 4,5-bisphosphate phosphodiesterase beta-1
- **Organism:** Homo sapiens
- **Uniprot Accession Number:** Q9NQ66
- **Protein sequence length:** 1216 aa
- **1D identity (%):** 15.18
- **1D identity (%) [Gaps excluded]:** 24.14
- **1D identity - Alignment Gaps:** 567
- **1D aligned content (<aminoacid>:%):** {'H': 0.43, 'A': 3.88, 'S': 5.6, 'G': 7.76, 'T': 4.31, 'K': 6.03, 'D': 8.62, 'P': 9.48, 'F': 7.33, 'L': 10.34, 'V': 4.31, 'N': 6.9, 'Y': 5.6, 'R': 2.59, 'E': 6.03, 'Q': 3.88, 'I': 5.6, 'C': 1.29}
- **Common reported functions (%):** 50.0
- **Common reported locations (%):** 0.0
- **Common reported processes (%):** 0.0

- **AF ID:** Q9NQ66
- **Chain:** A
- **Protein length:** 1216 aa
- **Resolution:** N/A
- **b-phipsi:** 0.021894
- **w-rdist:** 0.29334
- **t-alpha:** 0.069479
- **Chemical similarity (Tanimoto Index) (%):** 84.09
- **1D identity (%) [PDB]:** 2.95
- **1D identity (%) [Gaps excluded][PDB]:** 64.58
- **1D identity - Alignment Gaps [PDB]:** 2007
- **1D aligned content [PDB] (<aminoacid>:%):** {'G': 4.84, 'Q': 4.84, 'P': 9.68, 'T': 8.06, 'N': 8.06, 'V': 14.52, 'Y': 1.61, 'R': 1.61, 'L': 11.29, 'S': 4.84, 'F': 8.06, 'E': 3.23, 'K': 6.45, 'D': 4.84, 'I': 4.84, 'A': 3.23}
- **2D identity (%) [PDB]:** 30.76
- **2D identity (%) [Gaps excluded][PDB]:** 83.31
- **2D identity - Alignment Gaps [PDB]:** 1013
- **2D aligned content [PDB] (<2D-fold>:%):** {'T': 11.54, '.': 22.87, 'E': 29.96, 'H': 35.63}
- **3D similarity (TM-Score) (%) [PDB]:** 26.62

- **Gene name:** PLCB1
- **Entrez ID:** 23236
- **RefSeq ID:** NM\_182734
- **Transcript sequence length:** 7206
- **5-UTR|CDS|3-UTR identity (%):** 31.03 | 45.25 | 6.52
- **5-UTR|CDS|3-UTR identity (%) [Gaps excluded]:** 77.01 | 73.78 | 94.3
- **5-UTR|CDS|3-UTR identity [Alignment Gaps]:** 277 | 1760 | 3071
- **5-UTR aligned content (<base>:%):** {'A': 14.58, 'C': 33.33, 'T': 23.61, 'G': 28.47}
- **CDS aligned content (<base>:%):** {'A': 33.79, 'T': 28.06, 'C': 18.54, 'G': 19.61}
- **3-UTR aligned content (<base>:%):** {'C': 15.81, 'A': 39.53, 'T': 24.65, 'G': 20.0}

**Uniprot Description:**  
  
 Catalyzes the hydrolysis of 1-phosphatidylinositol 4,5-bisphosphate into diacylglycerol (DAG) and inositol 1,4,5-trisphosphate (IP3) and mediates intracellular signaling downstream of G protein-coupled receptors (PubMed:9188725). Regulates the function of the endothelial barrier.   
  
Interacts with DGKQ.   
  
 **Gene Ontology Information:**

Molecular Function

- calcium channel regulator activity
- calcium ion binding
- calmodulin binding
- channel activator activity
- enzyme binding
- GTPase activator activity
- identical protein binding
- ion channel regulator activity involved in G protein-coupled receptor signaling pathway
- lamin binding
- phosphatidylinositol phospholipase C activity
- phosphatidylinositol-4,5-bisphosphate binding
- phospholipase C activity
- protein serine/threonine kinase activator activity

Location

- chromatin
- cytoplasm
- cytosol
- extracellular exosome
- GABA-ergic synapse
- glutamatergic synapse
- nuclear membrane
- nuclear speck
- nucleus
- postsynaptic cytosol
- protein-containing complex

Biological process

- activation of meiosis involved in egg activation
- cellular response to fluoride
- cellular response to glyceraldehyde
- cellular response to ionomycin
- cellular response to vasopressin
- cerebral cortex development
- fat cell differentiation
- G protein-coupled acetylcholine receptor signaling pathway
- G protein-coupled receptor signaling pathway
- G2/M transition of mitotic cell cycle
- glutamate receptor signaling pathway
- inositol trisphosphate metabolic process
- insulin-like growth factor receptor signaling pathway
- interleukin-1-mediated signaling pathway
- interleukin-12-mediated signaling pathway
- interleukin-15-mediated signaling pathway
- ion channel modulating, G protein-coupled receptor signaling pathway
- learning
- ligand-gated ion channel signaling pathway
- memory
- negative regulation of transcription, DNA-templated
- negative regulation of monocyte extravasation
- phosphatidylinositol catabolic process
- phosphatidylinositol metabolic process
- phosphatidylinositol-mediated signaling
- phospholipase C-activating G protein-coupled acetylcholine receptor signaling pathway
- positive regulation of acrosome reaction
- positive regulation of CD24 production
- positive regulation of developmental growth
- positive regulation of transcription, DNA-templated
- positive regulation of embryonic development
- positive regulation of G1/S transition of mitotic cell cycle
- positive regulation of insulin secretion
- positive regulation of interleukin-12 production
- positive regulation of JNK cascade
- positive regulation of myoblast differentiation
- postsynaptic modulation of chemical synaptic transmission
- regulation of establishment of endothelial barrier
- regulation of fertilization
- regulation of G protein-coupled receptor signaling pathway
- regulation of retrograde trans-synaptic signaling by endocanabinoid
- signal transduction

---

15

- **Protein name:** Ribosome biogenesis protein SPATA5
- **Organism:** Homo sapiens
- **Uniprot Accession Number:** Q8NB90
- **Protein sequence length:** 893 aa
- **1D identity (%):** 11.05
- **1D identity (%) [Gaps excluded]:** 22.28
- **1D identity - Alignment Gaps:** 730
- **1D aligned content (<aminoacid>:%):** {'R': 3.75, 'L': 15.62, 'E': 4.38, 'N': 8.12, 'G': 13.12, 'A': 8.75, 'F': 5.62, 'I': 5.0, 'Y': 2.5, 'V': 3.75, 'Q': 3.75, 'P': 6.88, 'D': 2.5, 'T': 5.62, 'C': 3.12, 'K': 3.12, 'S': 4.38}
- **Common reported functions (%):** 0.0
- **Common reported locations (%):** 0.0
- **Common reported processes (%):** 0.0

- **AF ID:** Q8NB90
- **Chain:** A
- **Protein length:** 893 aa
- **Resolution:** N/A
- **b-phipsi:** 0.027798
- **w-rdist:** 0.24262
- **t-alpha:** 0.03866
- **Chemical similarity (Tanimoto Index) (%):** 83.55
- **1D identity (%) [PDB]:** 3.9
- **1D identity (%) [Gaps excluded][PDB]:** 65.09
- **1D identity - Alignment Gaps [PDB]:** 1664
- **1D aligned content [PDB] (<aminoacid>:%):** {'N': 4.35, 'G': 11.59, 'L': 17.39, 'T': 10.14, 'V': 4.35, 'P': 2.9, 'D': 2.9, 'M': 1.45, 'I': 8.7, 'A': 14.49, 'Q': 10.14, 'S': 5.8, 'F': 1.45, 'R': 1.45, 'K': 2.9}
- **2D identity (%) [PDB]:** 31.23
- **2D identity (%) [Gaps excluded][PDB]:** 86.9
- **2D identity - Alignment Gaps [PDB]:** 884
- **2D aligned content [PDB] (<2D-fold>:%):** {'.': 27.15, 'E': 24.13, 'T': 7.19, 'H': 41.3, 'B': 0.23}
- **3D similarity (TM-Score) (%) [PDB]:** 17.83

- **Gene name:** SPATA5
- **Entrez ID:** 166378
- **RefSeq ID:** N/A
- **Sequence length:** N/A
- **5-UTR|CDS|3-UTR identity (%):** N/A | N/A | N/A
- **5-UTR|CDS|3-UTR identity (%) [Gaps excluded]:** N/A | N/A | N/A
- **5-UTR|CDS|3-UTR identity [Alignment Gaps]:** N/A | N/A | N/A
- **5-UTR aligned content (<base>:%):** N/A
- **CDS aligned content (<base>:%):** N/A
- **3-UTR aligned content (<base>:%):** N/A

**Uniprot Description:**  
  
 ATP-dependent chaperone, which plays an essential role in the cytoplasmic maturation steps of pre-60S ribosomal particles by promoting the release of shuttling protein RSL24D1/RLP24 from the pre-ribosomal particles (PubMed:35354024). Acts together with SPATA5L1, C1orf109 and CINP (PubMed:35354024). May be involved in morphological and functional mitochondrial transformations during spermatogenesis (By similarity).   
  
Associates with pre-60S ribosomal particles (PubMed:35354024). Interacts with C1orf109 (PubMed:35354024).   
  
 **Gene Ontology Information:**

Molecular Function

- ATP binding
- ATPase activity
- preribosome binding

Location

- cytoplasm
- mitochondrion
- spindle

Biological process

- brain development
- cell differentiation
- ribosomal large subunit biogenesis
- spermatogenesis

---

16

- **Protein name:** Otogelin
- **Organism:** Homo sapiens
- **Uniprot Accession Number:** Q6ZRI0
- **Protein sequence length:** 2925 aa
- **1D identity (%):** 13.26
- **1D identity (%) [Gaps excluded]:** 31.03
- **1D identity - Alignment Gaps:** 1684
- **1D aligned content (<aminoacid>:%):** {'M': 0.26, 'V': 7.95, 'L': 8.46, 'P': 9.23, 'A': 8.46, 'T': 9.49, 'D': 6.67, 'S': 8.46, 'H': 1.54, 'F': 5.13, 'R': 3.33, 'N': 2.82, 'Y': 3.33, 'E': 3.33, 'I': 2.05, 'W': 1.03, 'G': 9.49, 'K': 1.54, 'Q': 2.31, 'C': 5.13}
- **Common reported functions (%):** 0.0
- **Common reported locations (%):** 0.0
- **Common reported processes (%):** 0.0

- **AF ID:** Q6ZRI0
- **Chain:** A
- **Protein length:** 1400 aa
- **Resolution:** N/A
- **b-phipsi:** 0.008113
- **w-rdist:** 0.341949
- **t-alpha:** 0.068652
- **Chemical similarity (Tanimoto Index) (%):** 86.24
- **1D identity (%) [PDB]:** 3.31
- **1D identity (%) [Gaps excluded][PDB]:** 65.79
- **1D identity - Alignment Gaps [PDB]:** 2155
- **1D aligned content [PDB] (<aminoacid>:%):** {'A': 8.0, 'S': 9.33, 'L': 20.0, 'G': 5.33, 'K': 4.0, 'D': 5.33, 'V': 10.67, 'N': 4.0, 'Q': 6.67, 'T': 6.67, 'F': 2.67, 'R': 5.33, 'P': 2.67, 'E': 4.0, 'I': 1.33, 'Y': 2.67, 'H': 1.33}
- **2D identity (%) [PDB]:** 30.14
- **2D identity (%) [Gaps excluded][PDB]:** 86.08
- **2D identity - Alignment Gaps [PDB]:** 1147
- **2D aligned content [PDB] (<2D-fold>:%):** {'.': 19.55, 'E': 39.29, 'T': 16.35, 'H': 21.99, 'G': 2.63, 'B': 0.19}
- **3D similarity (TM-Score) (%) [PDB]:** 21.48

- **Gene name:** OTOG
- **Entrez ID:** 340990
- **RefSeq ID:** N/A
- **Sequence length:** N/A
- **5-UTR|CDS|3-UTR identity (%):** N/A | N/A | N/A
- **5-UTR|CDS|3-UTR identity (%) [Gaps excluded]:** N/A | N/A | N/A
- **5-UTR|CDS|3-UTR identity [Alignment Gaps]:** N/A | N/A | N/A
- **5-UTR aligned content (<base>:%):** N/A
- **CDS aligned content (<base>:%):** N/A
- **3-UTR aligned content (<base>:%):** N/A

**Uniprot Description:**  
  
 Glycoprotein specific to acellular membranes of the inner ear. May be required for the anchoring of the otoconial membranes and cupulae to the underlying neuroepithelia in the vestibule. May be involved in the organization and/or stabilization of the fibrillar network that compose the tectorial membrane in the cochlea. May play a role in mechanotransduction processes (By similarity). N/A   
  
 **Gene Ontology Information:**

Molecular Function

- alpha-L-arabinofuranosidase activity

Location

- apical plasma membrane
- extracellular matrix
- extracellular space

Biological process

- L-arabinose metabolic process

---

17

- **Protein name:** Complement C5
- **Organism:** Homo sapiens
- **Uniprot Accession Number:** P01031
- **Protein sequence length:** 1676 aa
- **1D identity (%):** 17.13
- **1D identity (%) [Gaps excluded]:** 24.24
- **1D identity - Alignment Gaps:** 507
- **1D aligned content (<aminoacid>:%):** {'M': 0.34, 'F': 6.42, 'L': 13.18, 'S': 7.77, 'N': 7.09, 'Y': 4.05, 'T': 8.45, 'P': 5.74, 'D': 5.07, 'K': 7.77, 'H': 1.01, 'V': 6.76, 'G': 6.76, 'I': 4.73, 'A': 3.38, 'E': 3.72, 'R': 2.36, 'C': 3.04, 'Q': 2.36}
- **Common reported functions (%):** 0.0
- **Common reported locations (%):** 0.0
- **Common reported processes (%):** 0.0

- **AF ID:** P01031
- **Chain:** A
- **Protein length:** 1676 aa
- **Resolution:** N/A
- **b-phipsi:** 0.00765
- **w-rdist:** 0.343695
- **t-alpha:** 0.082364
- **Chemical similarity (Tanimoto Index) (%):** 85.36
- **1D identity (%) [PDB]:** 4.45
- **1D identity (%) [Gaps excluded][PDB]:** 66.47
- **1D identity - Alignment Gaps [PDB]:** 2325
- **1D aligned content [PDB] (<aminoacid>:%):** {'G': 8.11, 'F': 4.5, 'S': 9.91, 'I': 7.21, 'L': 16.22, 'P': 3.6, 'D': 3.6, 'K': 5.41, 'R': 2.7, 'E': 4.5, 'N': 5.41, 'V': 5.41, 'T': 6.31, 'A': 10.81, 'Y': 0.9, 'Q': 4.5, 'M': 0.9}
- **2D identity (%) [PDB]:** N/A
- **2D identity (%) [Gaps excluded][PDB]:** N/A
- **2D identity - Alignment Gaps [PDB]:** N/A
- **2D aligned content [PDB] (<2D-fold>:%):** N/A
- **3D similarity (TM-Score) (%) [PDB]:** 28.27

- **Gene name:** C5
- **Entrez ID:** 727
- **RefSeq ID:** NM\_001735
- **Transcript sequence length:** 5464
- **5-UTR|CDS|3-UTR identity (%):** 6.67 | 45.0 | 38.15
- **5-UTR|CDS|3-UTR identity (%) [Gaps excluded]:** 75.0 | 75.8 | 76.3
- **5-UTR|CDS|3-UTR identity [Alignment Gaps]:** 246 | 2257 | 211
- **5-UTR aligned content (<base>:%):** {'A': 27.78, 'T': 22.22, 'C': 44.44, 'G': 5.56}
- **CDS aligned content (<base>:%):** {'A': 31.68, 'T': 33.88, 'G': 17.64, 'C': 16.8}
- **3-UTR aligned content (<base>:%):** {'A': 47.2, 'T': 19.25, 'C': 15.53, 'G': 18.01}

**Uniprot Description:**  
  
 Activation of C5 by a C5 convertase initiates the spontaneous assembly of the late complement components, C5-C9, into the membrane attack complex. C5b has a transient binding site for C6. The C5b-C6 complex is the foundation upon which the lytic complex is assembled.   
  
C5 precursor is first processed by the removal of 4 basic residues, forming two chains, beta and alpha, linked by a disulfide bond. C5 convertase activates C5 by cleaving the alpha chain, releasing C5a anaphylatoxin and generating C5b (beta chain + alpha' chain). The C5a anaphylatoxin interacts with C5AR1. Interacts with tick complement inhibitor.   
  
 **Gene Ontology Information:**

Molecular Function

- chemokine activity
- endopeptidase inhibitor activity
- signaling receptor binding

Location

- extracellular exosome
- extracellular region
- extracellular space
- membrane attack complex

Biological process

- cell surface receptor signaling pathway
- chemotaxis
- complement activation
- complement activation, alternative pathway
- complement activation, classical pathway
- cytolysis
- G protein-coupled receptor signaling pathway
- in utero embryonic development
- inflammatory response
- negative regulation of macrophage chemotaxis
- positive regulation of angiogenesis
- positive regulation of chemokine production
- positive regulation of immune response
- positive regulation of vascular endothelial growth factor production

---

18

- **Protein name:** Carboxypeptidase D
- **Organism:** Homo sapiens
- **Uniprot Accession Number:** O75976
- **Protein sequence length:** 1380 aa
- **1D identity (%):** N/A
- **1D identity (%) [Gaps excluded]:** N/A
- **1D identity - Alignment Gaps:** N/A
- **1D aligned content (<aminoacid>:%):** N/A
- **Common reported functions (%):** N/A
- **Common reported locations (%):** N/A
- **Common reported processes (%):** N/A

- **AF ID:** O75976
- **Chain:** A
- **Protein length:** 1380 aa
- **Resolution:** N/A
- **b-phipsi:** 0.025759
- **w-rdist:** 0.293856
- **t-alpha:** 0.093466
- **Chemical similarity (Tanimoto Index) (%):** N/A
- **1D identity (%) [PDB]:** N/A
- **1D identity (%) [Gaps excluded][PDB]:** N/A
- **1D identity - Alignment Gaps [PDB]:** N/A
- **1D aligned content [PDB] (<aminoacid>:%):** N/A
- **2D identity (%) [PDB]:** N/A
- **2D identity (%) [Gaps excluded][PDB]:** N/A
- **2D identity - Alignment Gaps [PDB]:** N/A
- **2D aligned content [PDB] (<2D-fold>:%):** N/A
- **3D similarity (TM-Score) (%) [PDB]:** N/A

- **Gene name:** CPD
- **Entrez ID:** 1362
- **RefSeq ID:** N/A
- **Sequence length:** N/A
- **5-UTR|CDS|3-UTR identity (%):** N/A | N/A | N/A
- **5-UTR|CDS|3-UTR identity (%) [Gaps excluded]:** N/A | N/A | N/A
- **5-UTR|CDS|3-UTR identity [Alignment Gaps]:** N/A | N/A | N/A
- **5-UTR aligned content (<base>:%):** N/A
- **CDS aligned content (<base>:%):** N/A
- **3-UTR aligned content (<base>:%):** N/A

**Uniprot Description:**  
  
 N/A N/A   
  
 **Gene Ontology Information:**

Molecular Function

- metallocarboxypeptidase activity
- serine-type carboxypeptidase activity
- zinc ion binding

Location

- extracellular exosome
- extracellular space
- membrane
- plasma membrane

Biological process

- peptide metabolic process
- protein processing

---

19

- **Protein name:** Eukaryotic translation initiation factor 3 subunit B
- **Organism:** Homo sapiens
- **Uniprot Accession Number:** P55884
- **Protein sequence length:** 814 aa
- **1D identity (%):** 10.26
- **1D identity (%) [Gaps excluded]:** 21.05
- **1D identity - Alignment Gaps:** 719
- **1D aligned content (<aminoacid>:%):** {'E': 3.47, 'N': 4.17, 'A': 6.94, 'P': 7.64, 'G': 10.42, 'T': 7.64, 'I': 5.56, 'V': 9.03, 'S': 6.94, 'L': 5.56, 'R': 2.08, 'D': 6.25, 'F': 8.33, 'K': 4.17, 'Y': 2.78, 'Q': 4.86, 'C': 2.08, 'H': 0.69, 'W': 1.39}
- **Common reported functions (%):** 0.0
- **Common reported locations (%):** 0.0
- **Common reported processes (%):** 0.0

- **AF ID:** P55884
- **Chain:** A
- **Protein length:** 814 aa
- **Resolution:** N/A
- **b-phipsi:** 0.021235
- **w-rdist:** 0.31729
- **t-alpha:** 0.08354
- **Chemical similarity (Tanimoto Index) (%):** 83.65
- **1D identity (%) [PDB]:** 2.42
- **1D identity (%) [Gaps excluded][PDB]:** 68.85
- **1D identity - Alignment Gaps [PDB]:** 1675
- **1D aligned content [PDB] (<aminoacid>:%):** {'R': 4.76, 'A': 4.76, 'L': 4.76, 'G': 7.14, 'I': 7.14, 'V': 7.14, 'K': 14.29, 'N': 4.76, 'T': 7.14, 'F': 11.9, 'Q': 7.14, 'Y': 2.38, 'P': 4.76, 'D': 4.76, 'S': 4.76, 'E': 2.38}
- **2D identity (%) [PDB]:** 28.72
- **2D identity (%) [Gaps excluded][PDB]:** 85.21
- **2D identity - Alignment Gaps [PDB]:** 891
- **2D aligned content [PDB] (<2D-fold>:%):** {'T': 13.47, '.': 14.25, 'E': 54.4, 'G': 0.78, 'H': 17.1}
- **3D similarity (TM-Score) (%) [PDB]:** 17.1

- **Gene name:** EIF3B
- **Entrez ID:** 8662
- **RefSeq ID:** N/A
- **Sequence length:** N/A
- **5-UTR|CDS|3-UTR identity (%):** N/A | N/A | N/A
- **5-UTR|CDS|3-UTR identity (%) [Gaps excluded]:** N/A | N/A | N/A
- **5-UTR|CDS|3-UTR identity [Alignment Gaps]:** N/A | N/A | N/A
- **5-UTR aligned content (<base>:%):** N/A
- **CDS aligned content (<base>:%):** N/A
- **3-UTR aligned content (<base>:%):** N/A

**Uniprot Description:**  
  
 RNA-binding component of the eukaryotic translation initiation factor 3 (eIF-3) complex, which is required for several steps in the initiation of protein synthesis (PubMed:9388245, PubMed:17581632, PubMed:25849773, PubMed:27462815). The eIF-3 complex associates with the 40S ribosome and facilitates the recruitment of eIF-1, eIF-1A, eIF-2:GTP:methionyl-tRNAi and eIF-5 to form the 43S pre-initiation complex (43S PIC). The eIF-3 complex stimulates mRNA recruitment to the 43S PIC and scanning of the mRNA for AUG recognition. The eIF-3 complex is also required for disassembly and recycling of post-termination ribosomal complexes and subsequently prevents premature joining of the 40S and 60S ribosomal subunits prior to initiation (PubMed:9388245, PubMed:17581632). The eIF-3 complex specifically targets and initiates translation of a subset of mRNAs involved in cell proliferation, including cell cycling, differentiation and apoptosis, and uses different modes of RNA stem-loop binding to exert either translational activation or repression (PubMed:25849773).   
  
Component of the eukaryotic translation initiation factor 3 (eIF-3) complex, which is composed of 13 subunits: EIF3A, EIF3B, EIF3C, EIF3D, EIF3E, EIF3F, EIF3G, EIF3H, EIF3I, EIF3J, EIF3K, EIF3L and EIF3M. The eIF-3 complex appears to include 3 stable modules: module A is composed of EIF3A, EIF3B, EIF3G and EIF3I; module B is composed of EIF3F, EIF3H, and EIF3M; and module C is composed of EIF3C, EIF3D, EIF3E, EIF3K and EIF3L. EIF3C of module C binds EIF3B of module A and EIF3H of module B, thereby linking the three modules. EIF3J is a labile subunit that binds to the eIF-3 complex via EIF3B. The eIF-3 complex interacts with RPS6KB1 under conditions of nutrient depletion. Mitogenic stimulation leads to binding and activation of a complex composed of MTOR and RPTOR, leading to phosphorylation and release of RPS6KB1 and binding of EIF4B to eIF-3. Also interacts with UPF2 and HNRPD. Interacts with METTL3 (PubMed:27117702). Interacts with DDX3X (PubMed:18628297).   
  
 **Gene Ontology Information:**

Molecular Function

- molecular adaptor activity
- RNA binding
- translation initiation factor activity
- translation initiation factor binding

Location

- cytoplasmic stress granule
- cytosol
- eukaryotic 43S preinitiation complex
- eukaryotic 48S preinitiation complex
- eukaryotic translation initiation factor 3 complex
- eukaryotic translation initiation factor 3 complex, eIF3m
- extracellular exosome
- synapse

Biological process

- formation of cytoplasmic translation initiation complex
- IRES-dependent viral translational initiation
- regulation of translational initiation
- translational initiation
- viral translational termination-reinitiation

---

20

- **Protein name:** Fibrocystin-L
- **Organism:** Homo sapiens
- **Uniprot Accession Number:** Q86WI1
- **Protein sequence length:** 4243 aa
- **1D identity (%):** 10.28
- **1D identity (%) [Gaps excluded]:** 34.87
- **1D identity - Alignment Gaps:** 3004
- **1D aligned content (<aminoacid>:%):** {'M': 0.46, 'L': 8.68, 'P': 5.71, 'V': 5.94, 'N': 7.53, 'T': 7.99, 'R': 2.28, 'F': 6.62, 'G': 12.1, 'S': 7.53, 'H': 0.91, 'Q': 3.2, 'D': 5.71, 'W': 0.91, 'A': 3.65, 'Y': 4.57, 'I': 4.34, 'K': 4.11, 'E': 4.34, 'C': 3.42}
- **Common reported functions (%):** 0.0
- **Common reported locations (%):** 0.0
- **Common reported processes (%):** 0.0

- **AF ID:** Q86WI1
- **Chain:** A
- **Protein length:** 1400 aa
- **Resolution:** N/A
- **b-phipsi:** 0.015159
- **w-rdist:** 0.330552
- **t-alpha:** 0.11326
- **Chemical similarity (Tanimoto Index) (%):** 86.18
- **1D identity (%) [PDB]:** 3.11
- **1D identity (%) [Gaps excluded][PDB]:** 72.45
- **1D identity - Alignment Gaps [PDB]:** 2187
- **1D aligned content [PDB] (<aminoacid>:%):** {'G': 11.27, 'T': 4.23, 'S': 11.27, 'W': 1.41, 'F': 5.63, 'A': 11.27, 'L': 14.08, 'Q': 9.86, 'I': 7.04, 'P': 1.41, 'R': 1.41, 'V': 7.04, 'Y': 1.41, 'N': 7.04, 'D': 2.82, 'K': 2.82}
- **2D identity (%) [PDB]:** 31.69
- **2D identity (%) [Gaps excluded][PDB]:** 86.12
- **2D identity - Alignment Gaps [PDB]:** 1101
- **2D aligned content [PDB] (<2D-fold>:%):** {'.': 25.72, 'E': 53.26, 'T': 10.87, 'B': 0.18, 'G': 1.09, 'H': 8.88}
- **3D similarity (TM-Score) (%) [PDB]:** 26.69

- **Gene name:** PKHD1L1
- **Entrez ID:** 93035
- **RefSeq ID:** NM\_177531
- **Transcript sequence length:** 19969
- **5-UTR|CDS|3-UTR identity (%):** 23.94 | 24.85 | 3.12
- **5-UTR|CDS|3-UTR identity (%) [Gaps excluded]:** 67.33 | 84.99 | 97.37
- **5-UTR|CDS|3-UTR identity [Alignment Gaps]:** 183 | 9064 | 6890
- **5-UTR aligned content (<base>:%):** {'G': 20.59, 'T': 16.18, 'A': 26.47, 'C': 36.76}
- **CDS aligned content (<base>:%):** {'A': 29.85, 'T': 33.8, 'G': 18.32, 'C': 18.03}
- **3-UTR aligned content (<base>:%):** {'A': 40.54, 'T': 23.87, 'C': 14.86, 'G': 20.72}

**Uniprot Description:**  
  
 N/A N/A   
  
 **Gene Ontology Information:**

Molecular Function

- signaling receptor activity

Location

- cytosol
- extracellular space
- membrane
- stereocilium coat
- stereocilium tip

Biological process

- immune response
- sensory perception of sound

---

21

- **Protein name:** Integrin alpha-5
- **Organism:** Homo sapiens
- **Uniprot Accession Number:** P08648
- **Protein sequence length:** 1049 aa
- **1D identity (%):** 14.08
- **1D identity (%) [Gaps excluded]:** 23.67
- **1D identity - Alignment Gaps:** 590
- **1D aligned content (<aminoacid>:%):** {'G': 14.15, 'E': 1.95, 'P': 6.34, 'L': 10.73, 'R': 3.9, 'A': 7.8, 'V': 6.34, 'F': 5.85, 'T': 4.88, 'D': 3.41, 'S': 7.32, 'C': 3.41, 'Y': 4.88, 'W': 0.98, 'K': 2.93, 'Q': 4.39, 'I': 4.39, 'N': 4.88, 'H': 1.46}
- **Common reported functions (%):** 0.0
- **Common reported locations (%):** 20.0
- **Common reported processes (%):** 0.0

- **AF ID:** P08648
- **Chain:** A
- **Protein length:** 1049 aa
- **Resolution:** N/A
- **b-phipsi:** 0.024743
- **w-rdist:** 0.313122
- **t-alpha:** 0.054054
- **Chemical similarity (Tanimoto Index) (%):** 85.86
- **1D identity (%) [PDB]:** 3.86
- **1D identity (%) [Gaps excluded][PDB]:** 63.25
- **1D identity - Alignment Gaps [PDB]:** 1798
- **1D aligned content [PDB] (<aminoacid>:%):** {'P': 6.76, 'L': 16.22, 'F': 9.46, 'N': 8.11, 'D': 4.05, 'G': 6.76, 'V': 4.05, 'Y': 2.7, 'A': 6.76, 'S': 6.76, 'T': 4.05, 'E': 4.05, 'K': 5.41, 'I': 8.11, 'R': 4.05, 'Q': 2.7}
- **2D identity (%) [PDB]:** 30.31
- **2D identity (%) [Gaps excluded][PDB]:** 85.02
- **2D identity - Alignment Gaps [PDB]:** 964
- **2D aligned content [PDB] (<2D-fold>:%):** {'.': 25.11, 'T': 13.22, 'E': 54.41, 'G': 0.66, 'H': 6.61}
- **3D similarity (TM-Score) (%) [PDB]:** 21.91

- **Gene name:** ITGA5
- **Entrez ID:** 3678
- **RefSeq ID:** NM\_002205
- **Transcript sequence length:** 4250
- **5-UTR|CDS|3-UTR identity (%):** 11.64 | 40.7 | 17.94
- **5-UTR|CDS|3-UTR identity (%) [Gaps excluded]:** 82.93 | 71.81 | 83.04
- **5-UTR|CDS|3-UTR identity [Alignment Gaps]:** 251 | 1928 | 813
- **5-UTR aligned content (<base>:%):** {'A': 17.65, 'T': 26.47, 'C': 20.59, 'G': 35.29}
- **CDS aligned content (<base>:%):** {'T': 26.56, 'C': 24.68, 'A': 25.23, 'G': 23.52}
- **3-UTR aligned content (<base>:%):** {'C': 17.2, 'A': 39.78, 'T': 20.43, 'G': 22.58}

**Uniprot Description:**  
  
 Integrin alpha-5/beta-1 (ITGA5:ITGB1) is a receptor for fibronectin and fibrinogen. It recognizes the sequence R-G-D in its ligands. ITGA5:ITGB1 binds to PLA2G2A via a site (site 2) which is distinct from the classical ligand-binding site (site 1) and this induces integrin conformational changes and enhanced ligand binding to site 1 (PubMed:18635536, PubMed:25398877). ITGA5:ITGB1 acts as a receptor for fibrillin-1 (FBN1) and mediates R-G-D-dependent cell adhesion to FBN1 (PubMed:12807887, PubMed:17158881). ITGA5:ITGB1 is a receptor for IL1B and binding is essential for IL1B signaling (PubMed:29030430). ITGA5:ITGB3 is a receptor for soluble CD40LG and is required for CD40/CD40LG signaling (PubMed:31331973).   
  
Heterodimer of an alpha and a beta subunit. The alpha subunit is composed of a heavy and a light chain linked by a disulfide bond. Alpha-5 associates with beta-1. Interacts with HPS5 and NISCH. Interacts with RAB21 and COMP. Interacts with CIB1. ITGA5:ITGB1 interacts with CCN3. ITGA5:ITGB1 interacts with FBN1 (PubMed:12807887, PubMed:17158881). ITGA5:ITGB1 interacts with IL1B (PubMed:29030430). ITGA5:ITGB1 interacts with ACE2 (PubMed:33102950). Interacts with ANGPT2 (PubMed:32908006).   
  
 **Gene Ontology Information:**

Molecular Function

- epidermal growth factor receptor binding
- integrin binding
- metal ion binding
- platelet-derived growth factor receptor binding
- vascular endothelial growth factor receptor 2 binding
- virus receptor activity

Location

- alphav-beta3 integrin-vitronectin complex
- cell surface
- cytoplasmic vesicle
- endoplasmic reticulum
- external side of plasma membrane
- focal adhesion
- glutamatergic synapse
- Golgi apparatus
- integrin alpha5-beta1 complex
- integrin complex
- plasma membrane
- postsynaptic membrane
- ruffle

Biological process

- angiogenesis
- CD40 signaling pathway
- cell adhesion
- cell adhesion mediated by integrin
- cell-cell adhesion
- cell-cell adhesion mediated by integrin
- cell-matrix adhesion
- cell-substrate adhesion
- cell-substrate junction assembly
- endodermal cell differentiation
- female pregnancy
- heterophilic cell-cell adhesion via plasma membrane cell adhesion molecules
- heterotypic cell-cell adhesion
- integrin-mediated signaling pathway
- leukocyte cell-cell adhesion
- memory
- negative regulation of anoikis
- positive regulation of cell migration
- positive regulation of cell-substrate adhesion
- positive regulation of peptidyl-tyrosine phosphorylation
- positive regulation of sprouting angiogenesis
- positive regulation of vascular endothelial growth factor receptor signaling pathway
- wound healing, spreading of epidermal cells

---

22

- **Protein name:** Integrin alpha-8
- **Organism:** Homo sapiens
- **Uniprot Accession Number:** P53708
- **Protein sequence length:** 1063 aa
- **1D identity (%):** 12.34
- **1D identity (%) [Gaps excluded]:** 21.45
- **1D identity - Alignment Gaps:** 630
- **1D aligned content (<aminoacid>:%):** {'P': 9.29, 'G': 10.93, 'S': 4.92, 'A': 5.46, 'L': 9.29, 'C': 2.73, 'K': 5.46, 'T': 7.65, 'Y': 4.37, 'V': 5.46, 'I': 4.37, 'F': 3.28, 'N': 6.56, 'R': 4.37, 'D': 7.65, 'Q': 3.28, 'W': 1.64, 'E': 1.64, 'M': 0.55, 'H': 1.09}
- **Common reported functions (%):** 0.0
- **Common reported locations (%):** 0.0
- **Common reported processes (%):** 0.0

- **AF ID:** P53708
- **Chain:** A
- **Protein length:** 1063 aa
- **Resolution:** N/A
- **b-phipsi:** 0.02527
- **w-rdist:** 0.316318
- **t-alpha:** 0.082364
- **Chemical similarity (Tanimoto Index) (%):** 86.03
- **1D identity (%) [PDB]:** 1.8
- **1D identity (%) [Gaps excluded][PDB]:** 70.59
- **1D identity - Alignment Gaps [PDB]:** 1944
- **1D aligned content [PDB] (<aminoacid>:%):** {'S': 16.67, 'A': 11.11, 'L': 16.67, 'G': 5.56, 'Q': 8.33, 'D': 5.56, 'V': 11.11, 'N': 5.56, 'T': 2.78, 'K': 2.78, 'F': 2.78, 'R': 2.78, 'P': 5.56, 'E': 2.78}
- **2D identity (%) [PDB]:** 30.01
- **2D identity (%) [Gaps excluded][PDB]:** 83.7
- **2D identity - Alignment Gaps [PDB]:** 966
- **2D aligned content [PDB] (<2D-fold>:%):** {'.': 25.0, 'T': 13.5, 'E': 54.2, 'G': 0.66, 'H': 6.64}
- **3D similarity (TM-Score) (%) [PDB]:** 22.05

- **Gene name:** ITGA8
- **Entrez ID:** 396225
- **RefSeq ID:** NM\_003638
- **Transcript sequence length:** 6547
- **5-UTR|CDS|3-UTR identity (%):** 34.15 | 41.48 | 6.74
- **5-UTR|CDS|3-UTR identity (%) [Gaps excluded]:** 73.48 | 73.74 | 94.32
- **5-UTR|CDS|3-UTR identity [Alignment Gaps]:** 152 | 1964 | 2975
- **5-UTR aligned content (<base>:%):** {'A': 16.49, 'C': 34.02, 'G': 37.11, 'T': 12.37}
- **CDS aligned content (<base>:%):** {'A': 30.45, 'T': 30.02, 'G': 19.76, 'C': 19.76}
- **3-UTR aligned content (<base>:%):** {'C': 15.74, 'A': 41.67, 'T': 22.69, 'G': 19.91}

**Uniprot Description:**  
  
 Integrin alpha-8/beta-1 functions in the genesis of kidney and probably of other organs by regulating the recruitment of mesenchymal cells into epithelial structures. It recognizes the sequence R-G-D in a wide array of ligands including TNC, FN1, SPP1 TGFB1, TGFB3 and VTN. NPNT is probably its functional ligand in kidney genesis. Neuronal receptor for TNC it mediates cell-cell interactions and regulates neurite outgrowth of sensory and motor neurons.   
  
Heterodimer of an alpha and a beta subunit. The alpha subunit is composed of a heavy and a light chain linked by a disulfide bond. Alpha-8 associates with beta-1.   
  
 **Gene Ontology Information:**

Molecular Function

- integrin binding
- metal ion binding

Location

- external side of plasma membrane
- integrin complex

Biological process

- cell adhesion mediated by integrin
- cell differentiation
- cell-cell adhesion
- cell-matrix adhesion
- integrin-mediated signaling pathway
- nervous system development

---

23

- **Protein name:** FACT complex subunit SPT16
- **Organism:** Homo sapiens
- **Uniprot Accession Number:** Q9Y5B9
- **Protein sequence length:** 1047 aa
- **1D identity (%):** 14.67
- **1D identity (%) [Gaps excluded]:** 24.54
- **1D identity - Alignment Gaps:** 584
- **1D aligned content (<aminoacid>:%):** {'R': 3.76, 'Y': 4.23, 'S': 4.69, 'W': 0.94, 'G': 9.86, 'T': 3.76, 'L': 8.45, 'I': 7.51, 'D': 7.51, 'K': 7.51, 'N': 6.57, 'P': 4.23, 'F': 4.69, 'A': 6.1, 'C': 1.88, 'V': 6.57, 'E': 8.45, 'H': 0.47, 'Q': 2.35, 'M': 0.47}
- **Common reported functions (%):** 0.0
- **Common reported locations (%):** 0.0
- **Common reported processes (%):** 0.0

- **AF ID:** Q9Y5B9
- **Chain:** A
- **Protein length:** 1047 aa
- **Resolution:** N/A
- **b-phipsi:** 0.007772
- **w-rdist:** 0.361745
- **t-alpha:** 0.119444
- **Chemical similarity (Tanimoto Index) (%):** 84.14
- **1D identity (%) [PDB]:** 3.25
- **1D identity (%) [Gaps excluded][PDB]:** 68.48
- **1D identity - Alignment Gaps [PDB]:** 1846
- **1D aligned content [PDB] (<aminoacid>:%):** {'M': 3.17, 'T': 7.94, 'K': 11.11, 'S': 9.52, 'V': 4.76, 'D': 7.94, 'I': 9.52, 'N': 7.94, 'L': 9.52, 'Q': 3.17, 'Y': 3.17, 'F': 7.94, 'R': 1.59, 'G': 4.76, 'A': 1.59, 'E': 3.17, 'P': 3.17}
- **2D identity (%) [PDB]:** 25.77
- **2D identity (%) [Gaps excluded][PDB]:** 86.48
- **2D identity - Alignment Gaps [PDB]:** 1098
- **2D aligned content [PDB] (<2D-fold>:%):** {'.': 18.36, 'E': 31.51, 'T': 14.89, 'H': 34.49, 'G': 0.74}
- **3D similarity (TM-Score) (%) [PDB]:** 18.62

- **Gene name:** SUPT16H
- **Entrez ID:** 11198
- **RefSeq ID:** NM\_007192
- **Transcript sequence length:** 4433
- **5-UTR|CDS|3-UTR identity (%):** 19.08 | 42.92 | 15.84
- **5-UTR|CDS|3-UTR identity (%) [Gaps excluded]:** 72.97 | 73.55 | 86.82
- **5-UTR|CDS|3-UTR identity [Alignment Gaps]:** 209 | 1832 | 986
- **5-UTR aligned content (<base>:%):** {'G': 42.59, 'A': 14.81, 'T': 18.52, 'C': 24.07}
- **CDS aligned content (<base>:%):** {'A': 34.11, 'T': 28.23, 'G': 21.19, 'C': 16.47}
- **3-UTR aligned content (<base>:%):** {'C': 16.23, 'A': 39.27, 'T': 24.61, 'G': 19.9}

**Uniprot Description:**  
  
 Component of the FACT complex, a general chromatin factor that acts to reorganize nucleosomes. The FACT complex is involved in multiple processes that require DNA as a template such as mRNA elongation, DNA replication and DNA repair. During transcription elongation the FACT complex acts as a histone chaperone that both destabilizes and restores nucleosomal structure. It facilitates the passage of RNA polymerase II and transcription by promoting the dissociation of one histone H2A-H2B dimer from the nucleosome, then subsequently promotes the reestablishment of the nucleosome following the passage of RNA polymerase II. The FACT complex is probably also involved in phosphorylation of 'Ser-392' of p53/TP53 via its association with CK2 (casein kinase II).   
  
Interacts with MYOG (via C-terminal region) (By similarity). Component of the FACT complex, a stable heterodimer of SSRP1 and SUPT16H (PubMed:10421373). Also a component of a CK2-SPT16-SSRP1 complex which forms following UV irradiation, composed of SSRP1, SUPT16H, CSNK2A1, CSNK2A2 and CSNK2B (PubMed:11239457, PubMed:12393879). Interacts with NEK9 (PubMed:14660563). Binds to histone H2A-H2B (PubMed:10421373). Identified in a centromere complex containing histones H2A, H2B and H4, and at least CENPA, CENPB, CENPC, CENPT, CENPN, HJURP, SUPT16H, SSRP1 and RSF1 (PubMed:27499292). Interacts with GTF2E2 (PubMed:10792464).   
  
 **Gene Ontology Information:**

Molecular Function

- nucleosome binding
- RNA binding

Location

- FACT complex
- nucleoplasm
- nucleus

Biological process

- DNA repair
- DNA replication
- nucleosome assembly
- nucleosome disassembly
- positive regulation of DNA-templated transcription, elongation
- transcription by RNA polymerase II
- transcription elongation from RNA polymerase II promoter

---

24

- **Protein name:** E3 ubiquitin-protein ligase HERC2
- **Organism:** Homo sapiens
- **Uniprot Accession Number:** O95714
- **Protein sequence length:** 4834 aa
- **1D identity (%):** 9.09
- **1D identity (%) [Gaps excluded]:** 35.06
- **1D identity - Alignment Gaps:** 3591
- **1D aligned content (<aminoacid>:%):** {'P': 7.03, 'S': 7.48, 'C': 4.76, 'Q': 2.72, 'L': 12.93, 'F': 3.17, 'R': 2.72, 'D': 7.48, 'T': 6.8, 'W': 1.81, 'V': 5.44, 'G': 11.56, 'N': 3.85, 'A': 7.48, 'E': 3.17, 'K': 3.17, 'I': 3.63, 'Y': 2.95, 'H': 0.91, 'M': 0.91}
- **Common reported functions (%):** 0.0
- **Common reported locations (%):** 0.0
- **Common reported processes (%):** 0.0

- **AF ID:** O95714
- **Chain:** A
- **Protein length:** 1400 aa
- **Resolution:** N/A
- **b-phipsi:** 0.008759
- **w-rdist:** 0.362094
- **t-alpha:** 0.064516
- **Chemical similarity (Tanimoto Index) (%):** 83.61
- **1D identity (%) [PDB]:** 3.72
- **1D identity (%) [Gaps excluded][PDB]:** 68.29
- **1D identity - Alignment Gaps [PDB]:** 2137
- **1D aligned content [PDB] (<aminoacid>:%):** {'K': 3.57, 'N': 3.57, 'G': 8.33, 'L': 17.86, 'T': 8.33, 'V': 5.95, 'P': 3.57, 'D': 2.38, 'E': 2.38, 'A': 15.48, 'Q': 9.52, 'S': 11.9, 'W': 1.19, 'I': 3.57, 'M': 2.38}
- **2D identity (%) [PDB]:** 32.28
- **2D identity (%) [Gaps excluded][PDB]:** 88.11
- **2D identity - Alignment Gaps [PDB]:** 1105
- **2D aligned content [PDB] (<2D-fold>:%):** {'.': 21.49, 'E': 33.39, 'T': 13.85, 'G': 1.24, 'H': 29.84, 'B': 0.18}
- **3D similarity (TM-Score) (%) [PDB]:** 21.64

- **Gene name:** HERC2
- **Entrez ID:** N/A
- **RefSeq ID:** NM\_004667
- **Transcript sequence length:** 15364
- **5-UTR|CDS|3-UTR identity (%):** 21.86 | 21.79 | 24.49
- **5-UTR|CDS|3-UTR identity (%) [Gaps excluded]:** 75.56 | 84.34 | 81.0
- **5-UTR|CDS|3-UTR identity [Alignment Gaps]:** 221 | 10803 | 510
- **5-UTR aligned content (<base>:%):** {'G': 39.71, 'C': 30.88, 'A': 17.65, 'T': 11.76}
- **CDS aligned content (<base>:%):** {'A': 28.74, 'T': 32.21, 'G': 19.89, 'C': 19.16}
- **3-UTR aligned content (<base>:%):** {'A': 42.46, 'T': 23.46, 'C': 13.97, 'G': 20.11}

**Uniprot Description:**  
  
 E3 ubiquitin-protein ligase that regulates ubiquitin-dependent retention of repair proteins on damaged chromosomes. Recruited to sites of DNA damage in response to ionizing radiation (IR) and facilitates the assembly of UBE2N and RNF8 promoting DNA damage-induced formation of 'Lys-63'-linked ubiquitin chains. Acts as a mediator of binding specificity between UBE2N and RNF8. Involved in the maintenance of RNF168 levels. E3 ubiquitin-protein ligase that promotes the ubiquitination and proteasomal degradation of XPA which influences the circadian oscillation of DNA excision repair activity. By controlling the steady-state expression of the IGF1R receptor, indirectly regulates the insulin-like growth factor receptor signaling pathway (PubMed:26692333).   
  
Interacts (when phosphorylated at Thr-4827 and sumoylated) with RNF8 (via FHA domain); this interaction increases after ionizing radiation (IR) treatment. Interacts with XPA. Interacts with NEURL4. Via its interaction with NEURL4, may indirectly interact with CCP110 and CEP97.   
  
 **Gene Ontology Information:**

Molecular Function

- magnesium ion binding
- manganese ion binding
- phosphopentomutase activity

Location

- cytoplasm

Biological process

- 5-phosphoribose 1-diphosphate biosynthetic process
- cellular metabolic compound salvage
- deoxyribonucleotide catabolic process

---

25

- **Protein name:** Ovostatin homolog 1
- **Organism:** Homo sapiens
- **Uniprot Accession Number:** Q6IE37
- **Protein sequence length:** 1185 aa
- **1D identity (%):** 13.55
- **1D identity (%) [Gaps excluded]:** 25.32
- **1D identity - Alignment Gaps:** 744
- **1D aligned content (<aminoacid>:%):** {'C': 4.15, 'V': 6.45, 'Y': 4.15, 'S': 10.6, 'D': 5.53, 'A': 4.15, 'F': 8.29, 'Q': 5.07, 'I': 5.99, 'K': 2.76, 'N': 7.83, 'G': 7.37, 'R': 2.76, 'P': 5.99, 'T': 5.53, 'E': 4.15, 'L': 8.29, 'H': 0.46, 'M': 0.46}
- **Common reported functions (%):** 0.0
- **Common reported locations (%):** 0.0
- **Common reported processes (%):** 0.0

- **AF ID:** Q6IE37
- **Chain:** A
- **Protein length:** 1185 aa
- **Resolution:** N/A
- **b-phipsi:** 0.009583
- **w-rdist:** 0.358868
- **t-alpha:** 0.053136
- **Chemical similarity (Tanimoto Index) (%):** 86.27
- **1D identity (%) [PDB]:** 2.48
- **1D identity (%) [Gaps excluded][PDB]:** 72.22
- **1D identity - Alignment Gaps [PDB]:** 2024
- **1D aligned content [PDB] (<aminoacid>:%):** {'Y': 5.77, 'T': 13.46, 'S': 19.23, 'L': 11.54, 'A': 3.85, 'E': 7.69, 'N': 7.69, 'V': 3.85, 'I': 7.69, 'P': 3.85, 'F': 1.92, 'K': 1.92, 'G': 3.85, 'D': 1.92, 'Q': 3.85, 'R': 1.92}
- **2D identity (%) [PDB]:** 43.06
- **2D identity (%) [Gaps excluded][PDB]:** 81.58
- **2D identity - Alignment Gaps [PDB]:** 670
- **2D aligned content [PDB] (<2D-fold>:%):** {'.': 17.18, 'E': 45.66, 'T': 11.78, 'B': 0.16, 'H': 25.2}
- **3D similarity (TM-Score) (%) [PDB]:** 24.42

- **Gene name:** OVOS1
- **Entrez ID:** N/A
- **RefSeq ID:** N/A
- **Sequence length:** N/A
- **5-UTR|CDS|3-UTR identity (%):** N/A | N/A | N/A
- **5-UTR|CDS|3-UTR identity (%) [Gaps excluded]:** N/A | N/A | N/A
- **5-UTR|CDS|3-UTR identity [Alignment Gaps]:** N/A | N/A | N/A
- **5-UTR aligned content (<base>:%):** N/A
- **CDS aligned content (<base>:%):** N/A
- **3-UTR aligned content (<base>:%):** N/A

**Uniprot Description:**  
  
 Is able to inhibit all four classes of proteinases by a unique 'trapping' mechanism.   
  
Homotetramer.   
  
 **Gene Ontology Information:**

Molecular Function

- serine-type endopeptidase inhibitor activity

Location

- extracellular space

Biological process

- negative regulation of peptidase activity

---

26

- **Protein name:** N-acetyl-beta-glucosaminyl-glycoprotein 4-beta-N-acetylgalactosaminyltransferase 1
- **Organism:** Homo sapiens
- **Uniprot Accession Number:** Q76KP1
- **Protein sequence length:** 1039 aa
- **1D identity (%):** 13.89
- **1D identity (%) [Gaps excluded]:** 23.54
- **1D identity - Alignment Gaps:** 596
- **1D aligned content (<aminoacid>:%):** {'P': 10.89, 'V': 6.93, 'L': 10.89, 'R': 5.45, 'Y': 3.96, 'G': 12.87, 'T': 5.45, 'Q': 5.94, 'E': 5.94, 'D': 6.44, 'F': 5.94, 'C': 1.49, 'A': 8.42, 'K': 1.49, 'S': 4.46, 'N': 1.98, 'H': 1.49}
- **Common reported functions (%):** 0.0
- **Common reported locations (%):** 0.0
- **Common reported processes (%):** 0.0

- **AF ID:** Q76KP1
- **Chain:** A
- **Protein length:** 1039 aa
- **Resolution:** N/A
- **b-phipsi:** 0.031264
- **w-rdist:** 0.294876
- **t-alpha:** 0.067824
- **Chemical similarity (Tanimoto Index) (%):** 85.86
- **1D identity (%) [PDB]:** 3.11
- **1D identity (%) [Gaps excluded][PDB]:** 64.52
- **1D identity - Alignment Gaps [PDB]:** 1836
- **1D aligned content [PDB] (<aminoacid>:%):** {'T': 6.67, 'Y': 5.0, 'V': 10.0, 'L': 8.33, 'R': 3.33, 'A': 15.0, 'E': 6.67, 'K': 5.0, 'S': 5.0, 'G': 6.67, 'M': 1.67, 'P': 6.67, 'Q': 5.0, 'F': 6.67, 'H': 3.33, 'D': 1.67, 'N': 3.33}
- **2D identity (%) [PDB]:** 23.58
- **2D identity (%) [Gaps excluded][PDB]:** 85.78
- **2D identity - Alignment Gaps [PDB]:** 1150
- **2D aligned content [PDB] (<2D-fold>:%):** {'.': 33.96, 'T': 14.71, 'E': 34.22, 'G': 1.6, 'H': 15.51}
- **3D similarity (TM-Score) (%) [PDB]:** 18.89

- **Gene name:** B4GALNT4
- **Entrez ID:** 338707
- **RefSeq ID:** NM\_178537
- **Transcript sequence length:** 3750
- **5-UTR|CDS|3-UTR identity (%):** 30.33 | 38.07 | 34.95
- **5-UTR|CDS|3-UTR identity (%) [Gaps excluded]:** 65.19 | 69.67 | 71.43
- **5-UTR|CDS|3-UTR identity [Alignment Gaps]:** 208 | 2036 | 190
- **5-UTR aligned content (<base>:%):** {'G': 39.83, 'A': 14.41, 'T': 12.71, 'C': 33.05}
- **CDS aligned content (<base>:%):** {'A': 23.05, 'T': 22.76, 'C': 26.21, 'G': 27.97}
- **3-UTR aligned content (<base>:%):** {'C': 20.77, 'T': 23.85, 'A': 26.15, 'G': 29.23}

**Uniprot Description:**  
  
 Transfers N-acetylgalactosamine (GalNAc) from UDP-GalNAc to N-acetylglucosamine-beta-benzyl with a beta-1,4-linkage to form N,N'-diacetyllactosediamine, GalNAc-beta-1,4-GlcNAc structures in N-linked glycans and probably O-linked glycans. N/A   
  
 **Gene Ontology Information:**

Molecular Function

- acetylgalactosaminyltransferase activity
- N-acetyl-beta-glucosaminyl-glycoprotein 4-beta-N-acetylgalactosaminyltransferase activity

Location

- Golgi cisterna membrane

Biological process   
  
N/A

---

27

- **Protein name:** Netrin receptor UNC5A
- **Organism:** Homo sapiens
- **Uniprot Accession Number:** Q6ZN44
- **Protein sequence length:** 842 aa
- **1D identity (%):** 11.17
- **1D identity (%) [Gaps excluded]:** 21.7
- **1D identity - Alignment Gaps:** 677
- **1D aligned content (<aminoacid>:%):** {'P': 8.33, 'S': 11.54, 'G': 10.26, 'N': 2.56, 'V': 5.77, 'E': 3.85, 'I': 5.13, 'K': 6.41, 'L': 10.26, 'A': 5.13, 'T': 6.41, 'C': 5.13, 'H': 1.28, 'M': 0.64, 'F': 3.85, 'Y': 6.41, 'Q': 1.92, 'D': 3.21, 'R': 1.92}
- **Common reported functions (%):** 0.0
- **Common reported locations (%):** 20.0
- **Common reported processes (%):** 0.0

- **AF ID:** Q6ZN44
- **Chain:** A
- **Protein length:** 842 aa
- **Resolution:** N/A
- **b-phipsi:** 0.025506
- **w-rdist:** 0.326237
- **t-alpha:** 0.09512
- **Chemical similarity (Tanimoto Index) (%):** 85.89
- **1D identity (%) [PDB]:** 2.97
- **1D identity (%) [Gaps excluded][PDB]:** 70.27
- **1D identity - Alignment Gaps [PDB]:** 1677
- **1D aligned content [PDB] (<aminoacid>:%):** {'M': 1.92, 'A': 11.54, 'R': 1.92, 'N': 7.69, 'G': 5.77, 'V': 7.69, 'Q': 7.69, 'L': 15.38, 'E': 5.77, 'K': 5.77, 'I': 7.69, 'F': 3.85, 'S': 11.54, 'D': 1.92, 'P': 3.85}
- **2D identity (%) [PDB]:** 29.41
- **2D identity (%) [Gaps excluded][PDB]:** 83.58
- **2D identity - Alignment Gaps [PDB]:** 875
- **2D aligned content [PDB] (<2D-fold>:%):** {'.': 17.88, 'E': 50.13, 'T': 13.85, 'H': 18.14}
- **3D similarity (TM-Score) (%) [PDB]:** 20.55

- **Gene name:** UNC5A
- **Entrez ID:** 90249
- **RefSeq ID:** NM\_133369
- **Transcript sequence length:** 3733
- **5-UTR|CDS|3-UTR identity (%):** 33.22 | 34.42 | 17.75
- **5-UTR|CDS|3-UTR identity (%) [Gaps excluded]:** 60.49 | 72.62 | 81.9
- **5-UTR|CDS|3-UTR identity [Alignment Gaps]:** 133 | 2267 | 799
- **5-UTR aligned content (<base>:%):** {'G': 33.67, 'A': 12.24, 'C': 42.86, 'T': 11.22}
- **CDS aligned content (<base>:%):** {'A': 24.14, 'T': 24.21, 'G': 24.07, 'C': 27.58}
- **3-UTR aligned content (<base>:%):** {'C': 18.78, 'A': 34.81, 'T': 22.65, 'G': 23.76}

**Uniprot Description:**  
  
 Receptor for netrin required for axon guidance. Functions in the netrin signaling pathway and promotes neurite outgrowth in response to NTN1. Mediates axon repulsion of neuronal growth cones in the developing nervous system in response to netrin. Axon repulsion in growth cones may be mediated by its association with DCC that may trigger signaling for repulsion. It also acts as a dependence receptor required for apoptosis induction when not associated with netrin ligand.   
  
Homodimer and homooligomer. Interacts with the cytoplasmic part of DCC. Interacts with MAGED1. Interacts with PRKCABP, possibly mediating some interaction with PKC (By similarity). Interacts (via extracellular domain) with FLRT2 (via extracellular domain) (PubMed:25374360). Interacts (via extracellular domain) with FLRT3 (via extracellular domain) (By similarity).   
  
 **Gene Ontology Information:**

Molecular Function

- netrin receptor activity

Location

- membrane raft
- neuron projection membrane
- neuronal cell body membrane
- plasma membrane

Biological process

- anterior/posterior axon guidance
- apoptotic process
- axon guidance
- netrin-activated signaling pathway
- neuron projection development

---

28

- **Protein name:** Tight junction protein ZO-3
- **Organism:** Homo sapiens
- **Uniprot Accession Number:** O95049
- **Protein sequence length:** 919 aa
- **1D identity (%):** 12.65
- **1D identity (%) [Gaps excluded]:** 21.97
- **1D identity - Alignment Gaps:** 590
- **1D aligned content (<aminoacid>:%):** {'L': 7.95, 'E': 5.68, 'S': 13.07, 'G': 13.64, 'R': 6.82, 'P': 9.09, 'V': 4.55, 'D': 6.82, 'N': 2.27, 'T': 3.41, 'K': 3.98, 'A': 7.39, 'F': 3.41, 'Q': 5.68, 'I': 3.98, 'H': 0.57, 'Y': 1.7}
- **Common reported functions (%):** 0.0
- **Common reported locations (%):** 20.0
- **Common reported processes (%):** 0.0

- **AF ID:** O95049
- **Chain:** A
- **Protein length:** 919 aa
- **Resolution:** N/A
- **b-phipsi:** 0.03069
- **w-rdist:** 0.309852
- **t-alpha:** 0.241522
- **Chemical similarity (Tanimoto Index) (%):** 83.77
- **1D identity (%) [PDB]:** 3.21
- **1D identity (%) [Gaps excluded][PDB]:** 62.37
- **1D identity - Alignment Gaps [PDB]:** 1716
- **1D aligned content [PDB] (<aminoacid>:%):** {'T': 5.17, 'A': 15.52, 'G': 1.72, 'L': 18.97, 'Q': 12.07, 'V': 6.9, 'N': 5.17, 'K': 1.72, 'S': 10.34, 'D': 3.45, 'I': 5.17, 'P': 3.45, 'E': 3.45, 'R': 6.9}
- **2D identity (%) [PDB]:** 28.21
- **2D identity (%) [Gaps excluded][PDB]:** 84.28
- **2D identity - Alignment Gaps [PDB]:** 948
- **2D aligned content [PDB] (<2D-fold>:%):** {'.': 21.89, 'E': 32.59, 'T': 11.69, 'H': 31.34, 'G': 2.49}
- **3D similarity (TM-Score) (%) [PDB]:** 22.49

- **Gene name:** TJP3
- **Entrez ID:** 403852
- **RefSeq ID:** NM\_001267561
- **Transcript sequence length:** 3072
- **5-UTR|CDS|3-UTR identity (%):** 22.5 | 31.94 | 26.07
- **5-UTR|CDS|3-UTR identity (%) [Gaps excluded]:** 71.29 | 71.79 | 66.34
- **5-UTR|CDS|3-UTR identity [Alignment Gaps]:** 219 | 2539 | 156
- **5-UTR aligned content (<base>:%):** {'A': 18.06, 'C': 33.33, 'T': 18.06, 'G': 30.56}
- **CDS aligned content (<base>:%):** {'A': 26.21, 'T': 22.86, 'G': 26.15, 'C': 24.78}
- **3-UTR aligned content (<base>:%):** {'C': 28.36, 'G': 19.4, 'A': 25.37, 'T': 26.87}

**Uniprot Description:**  
  
 TJP1, TJP2, and TJP3 are closely related scaffolding proteins that link tight junction (TJ) transmembrane proteins such as claudins, junctional adhesion molecules, and occludin to the actin cytoskeleton (PubMed:16129888). The tight junction acts to limit movement of substances through the paracellular space and as a boundary between the compositionally distinct apical and basolateral plasma membrane domains of epithelial and endothelial cells. Binds and recruits PATJ to tight junctions where it connects and stabilizes apical and lateral components of tight junctions (PubMed:16129888). Promotes cell-cycle progression through the sequestration of cyclin D1 (CCND1) at tight junctions during mitosis which prevents CCND1 degradation during M-phase and enables S-phase transition (PubMed:21411630). With TJP1 and TJP2, participates in the junctional retention and stability of the transcription factor DBPA, but is not involved in its shuttling to the nucleus (By similarity). Contrary to TJP2, TJP3 is dispensable for individual viability, embryonic development, epithelial differentiation, and the establishment of TJs, at least in the laboratory environment (By similarity).   
  
Interacts with occludin OCLN, claudins and TPJ1 (By similarity). Interacts with PATJ (By similarity). Interacts with UBN1 (PubMed:20434232). Interacts with FASLG (PubMed:19807924). Interacts with CCND1 (PubMed:21411630).   
  
 **Gene Ontology Information:**

Molecular Function

- cell adhesion molecule binding

Location

- bicellular tight junction
- cell surface
- nucleus
- plasma membrane
- tight junction

Biological process

- cell-cell adhesion
- cell-cell junction organization
- establishment of endothelial intestinal barrier
- positive regulation of blood-brain barrier permeability
- protein localization to cell-cell junction

---

29

- **Protein name:** Zinc finger protein 317
- **Organism:** Homo sapiens
- **Uniprot Accession Number:** Q96PQ6
- **Protein sequence length:** 595 aa
- **1D identity (%):** 8.83
- **1D identity (%) [Gaps excluded]:** 22.18
- **1D identity - Alignment Gaps:** 804
- **1D aligned content (<aminoacid>:%):** {'F': 3.39, 'Q': 8.47, 'D': 6.78, 'V': 7.63, 'L': 8.47, 'C': 4.24, 'S': 5.93, 'G': 13.56, 'T': 8.47, 'P': 3.39, 'W': 0.85, 'E': 3.39, 'N': 5.08, 'R': 3.39, 'A': 6.78, 'M': 0.85, 'K': 4.24, 'Y': 2.54, 'H': 2.54}
- **Common reported functions (%):** 0.0
- **Common reported locations (%):** 0.0
- **Common reported processes (%):** 0.0

- **AF ID:** Q96PQ6
- **Chain:** A
- **Protein length:** 595 aa
- **Resolution:** N/A
- **b-phipsi:** 0.017445
- **w-rdist:** 0.353947
- **t-alpha:** 0.07775
- **Chemical similarity (Tanimoto Index) (%):** 83.94
- **1D identity (%) [PDB]:** 2.44
- **1D identity (%) [Gaps excluded][PDB]:** 62.71
- **1D identity - Alignment Gaps [PDB]:** 1460
- **1D aligned content [PDB] (<aminoacid>:%):** {'A': 13.51, 'L': 16.22, 'F': 5.41, 'Q': 13.51, 'G': 8.11, 'V': 2.7, 'T': 2.7, 'Y': 2.7, 'E': 2.7, 'N': 8.11, 'K': 8.11, 'I': 2.7, 'S': 10.81, 'D': 2.7}
- **2D identity (%) [PDB]:** 21.18
- **2D identity (%) [Gaps excluded][PDB]:** 87.34
- **2D identity - Alignment Gaps [PDB]:** 962
- **2D aligned content [PDB] (<2D-fold>:%):** {'.': 29.37, 'H': 61.34, 'T': 7.43, 'E': 1.86}
- **3D similarity (TM-Score) (%) [PDB]:** 14.84

- **Gene name:** ZNF317
- **Entrez ID:** 57693
- **RefSeq ID:** NM\_020933
- **Transcript sequence length:** 4056
- **5-UTR|CDS|3-UTR identity (%):** 38.38 | 25.69 | 10.58
- **5-UTR|CDS|3-UTR identity (%) [Gaps excluded]:** 77.6 | 75.1 | 93.33
- **5-UTR|CDS|3-UTR identity [Alignment Gaps]:** 187 | 2750 | 1759
- **5-UTR aligned content (<base>:%):** {'A': 19.72, 'T': 28.87, 'G': 21.83, 'C': 29.58}
- **CDS aligned content (<base>:%):** {'A': 30.63, 'T': 23.74, 'G': 21.6, 'C': 24.02}
- **3-UTR aligned content (<base>:%):** {'A': 42.38, 'T': 23.33, 'C': 15.24, 'G': 19.05}

**Uniprot Description:**  
  
 May function as a transcription factor. May play an important role in erythroid maturation and lymphoid proliferation. N/A   
  
 **Gene Ontology Information:**

Molecular Function

- DNA-binding transcription factor activity, RNA polymerase II-specific
- metal ion binding
- RNA polymerase II cis-regulatory region sequence-specific DNA binding

Location

- nucleus

Biological process

- regulation of transcription by RNA polymerase II

---

30

- **Protein name:** Fibroblast growth factor receptor 1
- **Organism:** Homo sapiens
- **Uniprot Accession Number:** P11362
- **Protein sequence length:** 822 aa
- **1D identity (%):** 10.28
- **1D identity (%) [Gaps excluded]:** 22.56
- **1D identity - Alignment Gaps:** 783
- **1D aligned content (<aminoacid>:%):** {'K': 4.05, 'C': 4.05, 'L': 12.84, 'T': 7.43, 'R': 2.7, 'P': 6.76, 'E': 4.05, 'S': 9.46, 'N': 6.08, 'I': 4.05, 'V': 5.41, 'A': 5.41, 'F': 3.38, 'D': 7.43, 'Y': 3.38, 'W': 1.35, 'G': 8.78, 'Q': 3.38}
- **Common reported functions (%):** 50.0
- **Common reported locations (%):** 20.0
- **Common reported processes (%):** 0.0

- **AF ID:** P11362
- **Chain:** A
- **Protein length:** 822 aa
- **Resolution:** N/A
- **b-phipsi:** 0.044756
- **w-rdist:** 0.239767
- **t-alpha:** 0.126748
- **Chemical similarity (Tanimoto Index) (%):** 86.23
- **1D identity (%) [PDB]:** 1.7
- **1D identity (%) [Gaps excluded][PDB]:** 83.33
- **1D identity - Alignment Gaps [PDB]:** 1733
- **1D aligned content [PDB] (<aminoacid>:%):** {'D': 13.33, 'T': 13.33, 'A': 3.33, 'V': 3.33, 'R': 3.33, 'P': 6.67, 'Q': 6.67, 'L': 6.67, 'E': 3.33, 'I': 6.67, 'F': 6.67, 'S': 10.0, 'G': 10.0, 'N': 6.67}
- **2D identity (%) [PDB]:** 28.59
- **2D identity (%) [Gaps excluded][PDB]:** 86.61
- **2D identity - Alignment Gaps [PDB]:** 909
- **2D aligned content [PDB] (<2D-fold>:%):** {'.': 17.27, 'H': 36.6, 'T': 13.66, 'E': 31.44, 'B': 0.26, 'G': 0.77}
- **3D similarity (TM-Score) (%) [PDB]:** 19.16

- **Gene name:** FGFR1
- **Entrez ID:** 2260
- **RefSeq ID:** NM\_023110
- **Transcript sequence length:** 5697
- **5-UTR|CDS|3-UTR identity (%):** 24.93 | 33.03 | 8.33
- **5-UTR|CDS|3-UTR identity (%) [Gaps excluded]:** 78.93 | 72.4 | 90.39
- **5-UTR|CDS|3-UTR identity [Alignment Gaps]:** 524 | 2349 | 2256
- **5-UTR aligned content (<base>:%):** {'A': 20.94, 'G': 26.7, 'T': 21.99, 'C': 30.37}
- **CDS aligned content (<base>:%):** {'A': 28.03, 'T': 24.95, 'G': 22.92, 'C': 24.11}
- **3-UTR aligned content (<base>:%):** {'C': 15.46, 'A': 40.58, 'T': 21.74, 'G': 22.22}

**Uniprot Description:**  
  
 Tyrosine-protein kinase that acts as cell-surface receptor for fibroblast growth factors and plays an essential role in the regulation of embryonic development, cell proliferation, differentiation and migration. Required for normal mesoderm patterning and correct axial organization during embryonic development, normal skeletogenesis and normal development of the gonadotropin-releasing hormone (GnRH) neuronal system. Phosphorylates PLCG1, FRS2, GAB1 and SHB. Ligand binding leads to the activation of several signaling cascades. Activation of PLCG1 leads to the production of the cellular signaling molecules diacylglycerol and inositol 1,4,5-trisphosphate. Phosphorylation of FRS2 triggers recruitment of GRB2, GAB1, PIK3R1 and SOS1, and mediates activation of RAS, MAPK1/ERK2, MAPK3/ERK1 and the MAP kinase signaling pathway, as well as of the AKT1 signaling pathway. Promotes phosphorylation of SHC1, STAT1 and PTPN11/SHP2. In the nucleus, enhances RPS6KA1 and CREB1 activity and contributes to the regulation of transcription. FGFR1 signaling is down-regulated by IL17RD/SEF, and by FGFR1 ubiquitination, internalization and degradation.   
  
Monomer. Homodimer after ligand binding. Interacts predominantly with FGF1 and FGF2, but can also interact with FGF3, FGF4, FGF5, FGF6, FGF8, FGF10, FGF19, FGF21, FGF22 and FGF23 (in vitro) (PubMed:1697263, PubMed:1722683, PubMed:8663044, PubMed:9655399, PubMed:12181353, PubMed:16597617, PubMed:17623664). Ligand specificity is determined by tissue-specific expression of isoforms, and differences in the third Ig-like domain are crucial for ligand specificity. Affinity for fibroblast growth factors (FGFs) is increased by heparan sulfate glycosaminoglycans that function as coreceptors. Likewise, KLB increases the affinity for FGF19, FGF21 and FGF23 (PubMed:19966287). Interacts (phosphorylated on Tyr-766) with PLCG1 (via SH2 domains) (PubMed:1656221, PubMed:1379697, PubMed:21765395). Interacts with FRS2 (PubMed:21765395). Interacts with RPS6KA1 (PubMed:15117958). Interacts (via C-terminus) with NEDD4 (via WW3 domain) (PubMed:21765395). Interacts with KL (By similarity). Interacts with SHB (via SH2 domain) (PubMed:12181353). Interacts with GRB10 (PubMed:10454568). Interacts with ANOS1; this interaction does not interfere with FGF2-binding to FGFR1, but prevents binding of heparin-bound FGF2 (PubMed:19696444). Interacts with SOX2 and SOX3. Interacts with FLRT1, FLRT2 and FLRT3 (By similarity). Found in a ternary complex with FGF1 and ITGAV:ITGB3 (PubMed:20422052, PubMed:18441324).   
  
 **Gene Ontology Information:**

Molecular Function

- ATP binding
- fibroblast growth factor binding
- fibroblast growth factor-activated receptor activity
- heparin binding
- identical protein binding
- protein homodimerization activity
- protein tyrosine kinase activity
- receptor-receptor interaction
- transmembrane receptor protein tyrosine kinase activity

Location

- cytoplasmic vesicle
- cytosol
- extracellular region
- membrane
- nucleus
- plasma membrane
- receptor complex

Biological process

- cell migration
- chordate embryonic development
- epithelial to mesenchymal transition
- fibroblast growth factor receptor signaling pathway
- MAPK cascade
- neuron migration
- peptidyl-tyrosine phosphorylation
- phosphatidylinositol-mediated signaling
- positive regulation of blood vessel endothelial cell migration
- positive regulation of cell differentiation
- positive regulation of cell population proliferation
- positive regulation of endothelial cell chemotaxis to fibroblast growth factor
- positive regulation of kinase activity
- positive regulation of MAP kinase activity
- positive regulation of MAPK cascade
- positive regulation of neuron differentiation
- positive regulation of phosphatidylinositol 3-kinase signaling
- positive regulation of phospholipase activity
- positive regulation of phospholipase C activity
- positive regulation of protein kinase B signaling
- positive regulation of vascular endothelial cell proliferation
- protein autophosphorylation
- protein phosphorylation
- regulation of cell differentiation
- regulation of extrinsic apoptotic signaling pathway in absence of ligand
- skeletal system development
- skeletal system morphogenesis
- transmembrane receptor protein tyrosine kinase signaling pathway

---

31

- **Protein name:** Zinc finger protein 816
- **Organism:** Homo sapiens
- **Uniprot Accession Number:** Q0VGE8
- **Protein sequence length:** 651 aa
- **1D identity (%):** 8.4
- **1D identity (%) [Gaps excluded]:** 21.36
- **1D identity - Alignment Gaps:** 838
- **1D aligned content (<aminoacid>:%):** {'L': 10.34, 'A': 3.45, 'T': 11.21, 'K': 10.34, 'F': 4.31, 'R': 4.31, 'S': 8.62, 'G': 8.62, 'V': 6.03, 'I': 6.03, 'D': 2.59, 'E': 3.45, 'H': 4.31, 'W': 0.86, 'P': 2.59, 'Q': 4.31, 'N': 1.72, 'M': 0.86, 'C': 5.17, 'Y': 0.86}
- **Common reported functions (%):** 0.0
- **Common reported locations (%):** 0.0
- **Common reported processes (%):** 0.0

- **AF ID:** Q0VGE8
- **Chain:** A
- **Protein length:** 651 aa
- **Resolution:** N/A
- **b-phipsi:** 0.020775
- **w-rdist:** 0.352607
- **t-alpha:** 0.117375
- **Chemical similarity (Tanimoto Index) (%):** 83.15
- **1D identity (%) [PDB]:** 1.95
- **1D identity (%) [Gaps excluded][PDB]:** 75.61
- **1D identity - Alignment Gaps [PDB]:** 1552
- **1D aligned content [PDB] (<aminoacid>:%):** {'L': 9.68, 'N': 3.23, 'R': 6.45, 'A': 6.45, 'T': 6.45, 'G': 9.68, 'Q': 6.45, 'E': 3.23, 'V': 3.23, 'K': 12.9, 'P': 6.45, 'I': 6.45, 'D': 3.23, 'F': 9.68, 'S': 6.45}
- **2D identity (%) [PDB]:** 24.8
- **2D identity (%) [Gaps excluded][PDB]:** 85.33
- **2D identity - Alignment Gaps [PDB]:** 898
- **2D aligned content [PDB] (<2D-fold>:%):** {'.': 32.48, 'H': 55.73, 'T': 9.24, 'E': 2.55}
- **3D similarity (TM-Score) (%) [PDB]:** 16.9

- **Gene name:** ZNF816
- **Entrez ID:** 125893
- **RefSeq ID:** NM\_001202457
- **Transcript sequence length:** 2560
- **5-UTR|CDS|3-UTR identity (%):** 33.11 | 31.09 | 37.17
- **5-UTR|CDS|3-UTR identity (%) [Gaps excluded]:** 68.31 | 76.47 | 79.62
- **5-UTR|CDS|3-UTR identity [Alignment Gaps]:** 151 | 2438 | 241
- **5-UTR aligned content (<base>:%):** {'A': 18.56, 'C': 28.87, 'G': 30.93, 'T': 21.65}
- **CDS aligned content (<base>:%):** {'A': 34.77, 'T': 29.44, 'G': 18.79, 'C': 16.99}
- **3-UTR aligned content (<base>:%):** {'C': 14.88, 'A': 47.02, 'T': 22.62, 'G': 15.48}

**Uniprot Description:**  
  
 May be involved in transcriptional regulation. N/A   
  
 **Gene Ontology Information:**

Molecular Function

- DNA-binding transcription factor activity, RNA polymerase II-specific
- metal ion binding
- RNA polymerase II cis-regulatory region sequence-specific DNA binding

Location

- nucleus

Biological process

- regulation of transcription by RNA polymerase II

---

32

- **Protein name:** Probable E3 ubiquitin-protein ligase HERC1
- **Organism:** Homo sapiens
- **Uniprot Accession Number:** Q15751
- **Protein sequence length:** 4861 aa
- **1D identity (%):** 9.06
- **1D identity (%) [Gaps excluded]:** 34.86
- **1D identity - Alignment Gaps:** 3604
- **1D aligned content (<aminoacid>:%):** {'M': 0.68, 'V': 7.03, 'L': 10.66, 'P': 7.03, 'S': 9.07, 'Q': 4.76, 'C': 4.31, 'F': 4.08, 'G': 9.98, 'D': 5.67, 'R': 3.63, 'H': 1.81, 'A': 6.58, 'T': 6.58, 'Y': 1.81, 'K': 2.72, 'I': 3.4, 'N': 4.99, 'W': 1.59, 'E': 3.63}
- **Common reported functions (%):** 0.0
- **Common reported locations (%):** 0.0
- **Common reported processes (%):** 0.0

- **AF ID:** Q15751
- **Chain:** A
- **Protein length:** 1400 aa
- **Resolution:** N/A
- **b-phipsi:** 0.036506
- **w-rdist:** 0.286952
- **t-alpha:** 0.076923
- **Chemical similarity (Tanimoto Index) (%):** 83.84
- **1D identity (%) [PDB]:** 2.98
- **1D identity (%) [Gaps excluded][PDB]:** 69.39
- **1D identity - Alignment Gaps [PDB]:** 2187
- **1D aligned content [PDB] (<aminoacid>:%):** {'S': 17.65, 'L': 14.71, 'A': 11.76, 'K': 4.41, 'Q': 10.29, 'V': 7.35, 'T': 2.94, 'G': 4.41, 'I': 5.88, 'D': 2.94, 'R': 7.35, 'P': 1.47, 'E': 4.41, 'Y': 1.47, 'N': 1.47, 'M': 1.47}
- **2D identity (%) [PDB]:** 28.58
- **2D identity (%) [Gaps excluded][PDB]:** 87.24
- **2D identity - Alignment Gaps [PDB]:** 1207
- **2D aligned content [PDB] (<2D-fold>:%):** {'.': 23.39, 'E': 25.73, 'T': 12.48, 'H': 35.67, 'G': 2.73}
- **3D similarity (TM-Score) (%) [PDB]:** 23.92

- **Gene name:** HERC1
- **Entrez ID:** 8925
- **RefSeq ID:** NM\_003922
- **Transcript sequence length:** 15197
- **5-UTR|CDS|3-UTR identity (%):** 32.87 | 22.07 | 32.44
- **5-UTR|CDS|3-UTR identity (%) [Gaps excluded]:** 76.61 | 85.91 | 77.07
- **5-UTR|CDS|3-UTR identity [Alignment Gaps]:** 165 | 10884 | 282
- **5-UTR aligned content (<base>:%):** {'T': 21.05, 'C': 30.53, 'A': 21.05, 'G': 27.37}
- **CDS aligned content (<base>:%):** {'A': 28.71, 'T': 32.98, 'G': 19.31, 'C': 19.0}
- **3-UTR aligned content (<base>:%):** {'C': 14.56, 'T': 29.11, 'A': 39.24, 'G': 17.09}

**Uniprot Description:**  
  
 Involved in membrane trafficking via some guanine nucleotide exchange factor (GEF) activity and its ability to bind clathrin. Acts as a GEF for Arf and Rab, by exchanging bound GDP for free GTP. Binds phosphatidylinositol 4,5-bisphosphate, which is required for GEF activity. May also act as a E3 ubiquitin-protein ligase which accepts ubiquitin from an E2 ubiquitin-conjugating enzyme in the form of a thioester and then directly transfers the ubiquitin to targeted substrates.   
  
Interacts with TSC2; interaction is inhibited by TSC1. Interacts with PKM, ARF1 and ARF6. Forms a ternary complex with clathrin heavy chain (CLTC) and HSPA1A.   
  
 **Gene Ontology Information:**

Molecular Function

- guanyl-nucleotide exchange factor activity
- ubiquitin-protein transferase activity

Location

- cytoplasm
- cytosol
- Golgi apparatus
- membrane

Biological process

- autophagy
- cerebellar Purkinje cell differentiation
- corpus callosum development
- negative regulation of autophagy
- neuromuscular process controlling balance
- neuron projection development

---

33

- **Protein name:** Transient receptor potential cation channel subfamily V member 1
- **Organism:** Homo sapiens
- **Uniprot Accession Number:** Q8NER1
- **Protein sequence length:** 839 aa
- **1D identity (%):** 12.81
- **1D identity (%) [Gaps excluded]:** 24.65
- **1D identity - Alignment Gaps:** 668
- **1D aligned content (<aminoacid>:%):** {'K': 5.62, 'S': 3.93, 'L': 11.8, 'D': 7.87, 'P': 7.87, 'G': 7.3, 'T': 10.11, 'R': 5.06, 'F': 7.3, 'V': 6.74, 'Q': 5.62, 'E': 3.93, 'Y': 5.06, 'I': 2.25, 'A': 4.49, 'N': 2.25, 'C': 1.69, 'W': 0.56, 'M': 0.56}
- **Common reported functions (%):** 50.0
- **Common reported locations (%):** 20.0
- **Common reported processes (%):** 0.0

- **AF ID:** Q8NER1
- **Chain:** A
- **Protein length:** 839 aa
- **Resolution:** N/A
- **b-phipsi:** 0.045759
- **w-rdist:** 0.232142
- **t-alpha:** 0.179512
- **Chemical similarity (Tanimoto Index) (%):** 84.14
- **1D identity (%) [PDB]:** 2.44
- **1D identity (%) [Gaps excluded][PDB]:** 72.88
- **1D identity - Alignment Gaps [PDB]:** 1704
- **1D aligned content [PDB] (<aminoacid>:%):** {'M': 6.98, 'L': 11.63, 'G': 6.98, 'A': 6.98, 'S': 11.63, 'V': 9.3, 'N': 6.98, 'I': 9.3, 'P': 2.33, 'T': 9.3, 'F': 4.65, 'E': 4.65, 'K': 2.33, 'D': 4.65, 'Q': 2.33}
- **2D identity (%) [PDB]:** 23.31
- **2D identity (%) [Gaps excluded][PDB]:** 83.42
- **2D identity - Alignment Gaps [PDB]:** 1026
- **2D aligned content [PDB] (<2D-fold>:%):** {'.': 28.92, 'T': 9.04, 'E': 12.35, 'H': 49.7}
- **3D similarity (TM-Score) (%) [PDB]:** 18.64

- **Gene name:** TRPV1
- **Entrez ID:** 7442
- **RefSeq ID:** NM\_080706
- **Transcript sequence length:** 4443
- **5-UTR|CDS|3-UTR identity (%):** 31.37 | 34.54 | 14.38
- **5-UTR|CDS|3-UTR identity (%) [Gaps excluded]:** 76.19 | 73.0 | 88.55
- **5-UTR|CDS|3-UTR identity [Alignment Gaps]:** 330 | 2268 | 1171
- **5-UTR aligned content (<base>:%):** {'C': 27.84, 'T': 25.57, 'G': 24.43, 'A': 22.16}
- **CDS aligned content (<base>:%):** {'A': 27.24, 'T': 25.69, 'G': 23.0, 'C': 24.08}
- **3-UTR aligned content (<base>:%):** {'C': 15.42, 'A': 41.79, 'T': 21.39, 'G': 21.39}

**Uniprot Description:**  
  
 Ligand-activated non-selective calcium permeant cation channel involved in detection of noxious chemical and thermal stimuli. Seems to mediate proton influx and may be involved in intracellular acidosis in nociceptive neurons. Involved in mediation of inflammatory pain and hyperalgesia. Sensitized by a phosphatidylinositol second messenger system activated by receptor tyrosine kinases, which involves PKC isozymes and PCL. Activation by vanilloids, like capsaicin, and temperatures higher than 42 degrees Celsius, exhibits a time- and Ca(2+)-dependent outward rectification, followed by a long-lasting refractory state. Mild extracellular acidic pH (6.5) potentiates channel activation by noxious heat and vanilloids, whereas acidic conditions (pH <6) directly activate the channel. Can be activated by endogenous compounds, including 12-hydroperoxytetraenoic acid and bradykinin. Acts as ionotropic endocannabinoid receptor with central neuromodulatory effects. Triggers a form of long-term depression (TRPV1-LTD) mediated by the endocannabinoid anandamine in the hippocampus and nucleus accumbens by affecting AMPA receptors endocytosis.   
  
Interacts with PIRT (By similarity). Homotetramer (By similarity). Interacts with TRPV3 and may also form a heteromeric channel with TRPV3 (PubMed:12077606). Interacts with CALM, PRKCM and CSK. Interacts with PRKCG and NTRK1, probably by forming a trimeric complex (By similarity). Interacts with the Scolopendra mutilans RhTx toxin (By similarity). Interacts with TMEM100 (By similarity). Interacts with PACS2 (PubMed:29656858).   
  
 **Gene Ontology Information:**

Molecular Function

- ATP binding
- calcium channel activity
- calcium-release channel activity
- calmodulin binding
- chloride channel regulator activity
- excitatory extracellular ligand-gated ion channel activity
- extracellular ligand-gated ion channel activity
- identical protein binding
- ion channel activity
- metal ion binding
- phosphatidylinositol binding
- phosphoprotein binding
- temperature-gated ion channel activity
- transmembrane signaling receptor activity
- voltage-gated calcium channel activity

Location

- cytosol
- dendritic spine membrane
- external side of plasma membrane
- membrane
- mitochondrion
- neuronal cell body
- plasma membrane
- postsynaptic membrane

Biological process

- behavioral response to pain
- calcium ion import across plasma membrane
- calcium ion transmembrane transport
- cell surface receptor signaling pathway
- cellular response to acidic pH
- cellular response to alkaloid
- cellular response to ATP
- cellular response to heat
- cellular response to nerve growth factor stimulus
- cellular response to temperature stimulus
- cellular response to tumor necrosis factor
- chemosensory behavior
- detection of chemical stimulus involved in sensory perception of pain
- detection of temperature stimulus involved in sensory perception of pain
- detection of temperature stimulus involved in thermoception
- diet induced thermogenesis
- fever generation
- glutamate secretion
- lipid metabolic process
- microglial cell activation
- negative regulation of establishment of blood-brain barrier
- negative regulation of heart rate
- negative regulation of mitochondrial membrane potential
- negative regulation of systemic arterial blood pressure
- negative regulation of transcription by RNA polymerase II
- peptide secretion
- positive regulation of apoptotic process
- positive regulation of cytosolic calcium ion concentration
- positive regulation of gastric acid secretion
- positive regulation of nitric oxide biosynthetic process
- protein homotetramerization
- response to capsazepine
- response to peptide hormone
- sensory perception of mechanical stimulus
- smooth muscle contraction involved in micturition
- thermoception

---

34

- **Protein name:** Rho GTPase-activating protein 22
- **Organism:** Homo sapiens
- **Uniprot Accession Number:** Q7Z5H3
- **Protein sequence length:** 698 aa
- **1D identity (%):** 11.25
- **1D identity (%) [Gaps excluded]:** 23.51
- **1D identity - Alignment Gaps:** 695
- **1D aligned content (<aminoacid>:%):** {'S': 10.67, 'I': 2.0, 'Q': 5.33, 'A': 6.0, 'V': 3.33, 'G': 10.67, 'P': 7.33, 'R': 5.33, 'K': 6.67, 'N': 4.67, 'L': 12.67, 'F': 2.67, 'D': 3.33, 'W': 0.67, 'H': 1.33, 'Y': 3.33, 'C': 2.0, 'T': 6.0, 'E': 6.0}
- **Common reported functions (%):** 0.0
- **Common reported locations (%):** 0.0
- **Common reported processes (%):** 0.0

- **AF ID:** Q7Z5H3
- **Chain:** A
- **Protein length:** 698 aa
- **Resolution:** N/A
- **b-phipsi:** 0.042497
- **w-rdist:** 0.261288
- **t-alpha:** 0.020253
- **Chemical similarity (Tanimoto Index) (%):** 83.82
- **1D identity (%) [PDB]:** 2.01
- **1D identity (%) [Gaps excluded][PDB]:** 78.57
- **1D identity - Alignment Gaps [PDB]:** 1597
- **1D aligned content [PDB] (<aminoacid>:%):** {'L': 18.18, 'S': 6.06, 'R': 12.12, 'P': 6.06, 'E': 9.09, 'A': 12.12, 'V': 6.06, 'Q': 6.06, 'I': 6.06, 'T': 9.09, 'G': 3.03, 'Y': 3.03, 'K': 3.03}
- **2D identity (%) [PDB]:** 22.08
- **2D identity (%) [Gaps excluded][PDB]:** 88.39
- **2D identity - Alignment Gaps [PDB]:** 1009
- **2D aligned content [PDB] (<2D-fold>:%):** {'.': 21.21, 'T': 7.41, 'E': 14.48, 'H': 55.89, 'G': 1.01}
- **3D similarity (TM-Score) (%) [PDB]:** 17.14

- **Gene name:** ARHGAP22
- **Entrez ID:** 58504
- **RefSeq ID:** N/A
- **Sequence length:** N/A
- **5-UTR|CDS|3-UTR identity (%):** N/A | N/A | N/A
- **5-UTR|CDS|3-UTR identity (%) [Gaps excluded]:** N/A | N/A | N/A
- **5-UTR|CDS|3-UTR identity [Alignment Gaps]:** N/A | N/A | N/A
- **5-UTR aligned content (<base>:%):** N/A
- **CDS aligned content (<base>:%):** N/A
- **3-UTR aligned content (<base>:%):** N/A

**Uniprot Description:**  
  
 Rho GTPase-activating protein involved in the signal transduction pathway that regulates endothelial cell capillary tube formation during angiogenesis. Acts as a GTPase activator for the RAC1 by converting it to an inactive GDP-bound state. Inhibits RAC1-dependent lamellipodia formation. May also play a role in transcription regulation via its interaction with VEZF1, by regulating activity of the endothelin-1 (EDN1) promoter (By similarity).   
  
Interacts with VEZF1.   
  
 **Gene Ontology Information:**

Molecular Function

- GTPase activator activity

Location

- cytosol
- focal adhesion
- glutamatergic synapse
- nucleus

Biological process

- activation of GTPase activity
- angiogenesis
- cell differentiation
- regulation of postsynapse organization
- regulation of small GTPase mediated signal transduction
- signal transduction

---

35

- **Protein name:** Zinc finger protein 45
- **Organism:** Homo sapiens
- **Uniprot Accession Number:** Q02386
- **Protein sequence length:** 682 aa
- **1D identity (%):** 9.91
- **1D identity (%) [Gaps excluded]:** 23.33
- **1D identity - Alignment Gaps:** 789
- **1D aligned content (<aminoacid>:%):** {'S': 6.62, 'T': 7.35, 'F': 7.35, 'V': 5.88, 'L': 5.88, 'D': 2.94, 'K': 5.15, 'R': 5.88, 'N': 4.41, 'P': 3.68, 'G': 15.44, 'Y': 5.88, 'W': 1.47, 'Q': 3.68, 'C': 7.35, 'E': 2.21, 'I': 3.68, 'A': 4.41, 'H': 0.74}
- **Common reported functions (%):** 0.0
- **Common reported locations (%):** 0.0
- **Common reported processes (%):** 0.0

- **AF ID:** Q02386
- **Chain:** A
- **Protein length:** 682 aa
- **Resolution:** N/A
- **b-phipsi:** 0.03214
- **w-rdist:** 0.31691
- **t-alpha:** 0.212638
- **Chemical similarity (Tanimoto Index) (%):** 83.68
- **1D identity (%) [PDB]:** 1.34
- **1D identity (%) [Gaps excluded][PDB]:** 88.0
- **1D identity - Alignment Gaps [PDB]:** 1615
- **1D aligned content [PDB] (<aminoacid>:%):** {'E': 9.09, 'A': 4.55, 'V': 9.09, 'Q': 22.73, 'I': 9.09, 'R': 13.64, 'L': 18.18, 'T': 4.55, 'G': 4.55, 'S': 4.55}
- **2D identity (%) [PDB]:** 19.57
- **2D identity (%) [Gaps excluded][PDB]:** 86.93
- **2D identity - Alignment Gaps [PDB]:** 1053
- **2D aligned content [PDB] (<2D-fold>:%):** {'.': 18.05, 'H': 64.29, 'T': 12.41, 'E': 5.26}
- **3D similarity (TM-Score) (%) [PDB]:** 17.96

- **Gene name:** ZNF45
- **Entrez ID:** 7596
- **RefSeq ID:** N/A
- **Sequence length:** N/A
- **5-UTR|CDS|3-UTR identity (%):** N/A | N/A | N/A
- **5-UTR|CDS|3-UTR identity (%) [Gaps excluded]:** N/A | N/A | N/A
- **5-UTR|CDS|3-UTR identity [Alignment Gaps]:** N/A | N/A | N/A
- **5-UTR aligned content (<base>:%):** N/A
- **CDS aligned content (<base>:%):** N/A
- **3-UTR aligned content (<base>:%):** N/A

**Uniprot Description:**  
  
 May be involved in transcriptional regulation. N/A   
  
 **Gene Ontology Information:**

Molecular Function

- DNA-binding transcription factor activity, RNA polymerase II-specific
- metal ion binding
- RNA polymerase II cis-regulatory region sequence-specific DNA binding

Location

- nucleoplasm
- nucleus

Biological process

- regulation of transcription by RNA polymerase II

---

36

- **Protein name:** DNA methyltransferase 1-associated protein 1
- **Organism:** Homo sapiens
- **Uniprot Accession Number:** Q9NPF5
- **Protein sequence length:** 467 aa
- **1D identity (%):** 6.61
- **1D identity (%) [Gaps excluded]:** 25.0
- **1D identity - Alignment Gaps:** 1012
- **1D aligned content (<aminoacid>:%):** {'A': 13.19, 'V': 8.79, 'I': 5.49, 'P': 9.89, 'D': 5.49, 'S': 4.4, 'K': 6.59, 'E': 4.4, 'L': 14.29, 'F': 4.4, 'G': 5.49, 'Y': 2.2, 'W': 1.1, 'R': 3.3, 'N': 1.1, 'Q': 5.49, 'C': 1.1, 'H': 1.1, 'T': 2.2}
- **Common reported functions (%):** 0.0
- **Common reported locations (%):** 0.0
- **Common reported processes (%):** 0.0

- **AF ID:** Q9NPF5
- **Chain:** A
- **Protein length:** 467 aa
- **Resolution:** N/A
- **b-phipsi:** 0.028788
- **w-rdist:** 0.329958
- **t-alpha:** 0.090172
- **Chemical similarity (Tanimoto Index) (%):** 83.98
- **1D identity (%) [PDB]:** 2.2
- **1D identity (%) [Gaps excluded][PDB]:** 73.81
- **1D identity - Alignment Gaps [PDB]:** 1366
- **1D aligned content [PDB] (<aminoacid>:%):** {'M': 3.23, 'A': 16.13, 'R': 3.23, 'F': 6.45, 'N': 6.45, 'T': 6.45, 'V': 3.23, 'L': 12.9, 'Y': 3.23, 'Q': 6.45, 'K': 9.68, 'I': 6.45, 'S': 9.68, 'G': 6.45}
- **2D identity (%) [PDB]:** 21.46
- **2D identity (%) [Gaps excluded][PDB]:** 93.36
- **2D identity - Alignment Gaps [PDB]:** 908
- **2D aligned content [PDB] (<2D-fold>:%):** {'.': 22.13, 'T': 7.91, 'E': 5.53, 'H': 62.85, 'G': 1.58}
- **3D similarity (TM-Score) (%) [PDB]:** 13.23

- **Gene name:** DMAP1
- **Entrez ID:** 55929
- **RefSeq ID:** N/A
- **Sequence length:** N/A
- **5-UTR|CDS|3-UTR identity (%):** N/A | N/A | N/A
- **5-UTR|CDS|3-UTR identity (%) [Gaps excluded]:** N/A | N/A | N/A
- **5-UTR|CDS|3-UTR identity [Alignment Gaps]:** N/A | N/A | N/A
- **5-UTR aligned content (<base>:%):** N/A
- **CDS aligned content (<base>:%):** N/A
- **3-UTR aligned content (<base>:%):** N/A

**Uniprot Description:**  
  
 Involved in transcription repression and activation. Its interaction with HDAC2 may provide a mechanism for histone deacetylation in heterochromatin following replication of DNA at late firing origins. Can also repress transcription independently of histone deacetylase activity. May specifically potentiate DAXX-mediated repression of glucocorticoid receptor-dependent transcription. Component of the NuA4 histone acetyltransferase (HAT) complex which is involved in transcriptional activation of select genes principally by acetylation of nucleosomal histones H4 and H2A. This modification may both alter nucleosome - DNA interactions and promote interaction of the modified histones with other proteins which positively regulate transcription. This complex may be required for the activation of transcriptional programs associated with oncogene and proto-oncogene mediated growth induction, tumor suppressor mediated growth arrest and replicative senescence, apoptosis, and DNA repair. NuA4 may also play a direct role in DNA repair when recruited to sites of DNA damage. Participates in the nuclear localization of URI1 and increases its transcriptional corepressor activity.   
  
Component of the NuA4 histone acetyltransferase complex which contains the catalytic subunit KAT5/TIP60 and the subunits EP400, TRRAP/PAF400, BRD8/SMAP, EPC1, DMAP1/DNMAP1, RUVBL1/TIP49, RUVBL2, ING3, actin, ACTL6A/BAF53A, MORF4L1/MRG15, MORF4L2/MRGX, MRGBP, YEATS4/GAS41, VPS72/YL1 and MEAF6. Component of a NuA4-related complex which contains EP400, TRRAP/PAF400, SRCAP, BRD8/SMAP, EPC1, DMAP1/DNMAP1, RUVBL1/TIP49, RUVBL2, actin, ACTL6A/BAF53A, VPS72 and YEATS4/GAS41. DMAP1 also forms a complex with DNMT1 and HDAC2. Throughout S phase it interacts directly with the N-terminus of DNMT1, which serves to recruit DMAP1 to replication foci. DMAP1 interacts with ING1, a component of the mSin3A transcription repressor complex, although this interaction is not required for recruitment of ING1 to heterochromatin. Interacts directly with the transcriptional corepressor TSG101. Interacts with the pro-apoptotic protein DAXX. Interacts with URI1.   
  
 **Gene Ontology Information:**

Molecular Function

- RNA polymerase II-specific DNA-binding transcription factor binding
- transcription corepressor activity

Location

- cytoplasm
- cytosol
- NuA4 histone acetyltransferase complex
- nucleoplasm
- nucleosome
- nucleus
- replication fork
- Swr1 complex

Biological process

- chromatin remodeling
- DNA methylation
- DNA repair
- histone acetylation
- histone H2A acetylation
- histone H4 acetylation
- negative regulation of transcription, DNA-templated
- negative regulation of transcription by RNA polymerase II
- positive regulation of transcription, DNA-templated
- positive regulation of double-strand break repair via homologous recombination
- positive regulation of protein import into nucleus
- regulation of apoptotic process
- regulation of cell cycle
- regulation of transcription, DNA-templated
- regulation of double-strand break repair
- response to ethanol

---

37

- **Protein name:** DmX-like protein 2
- **Organism:** Homo sapiens
- **Uniprot Accession Number:** Q8TDJ6
- **Protein sequence length:** 3036 aa
- **1D identity (%):** 12.99
- **1D identity (%) [Gaps excluded]:** 31.94
- **1D identity - Alignment Gaps:** 1817
- **1D aligned content (<aminoacid>:%):** {'M': 0.25, 'V': 7.29, 'L': 9.8, 'C': 4.27, 'P': 7.54, 'A': 6.03, 'Y': 3.27, 'N': 6.03, 'F': 6.78, 'S': 9.05, 'Q': 3.52, 'T': 6.53, 'I': 3.27, 'G': 7.79, 'D': 6.53, 'K': 4.77, 'W': 1.26, 'E': 3.52, 'R': 2.01, 'H': 0.5}
- **Common reported functions (%):** 0.0
- **Common reported locations (%):** 0.0
- **Common reported processes (%):** 0.0

- **AF ID:** Q8TDJ6
- **Chain:** A
- **Protein length:** 1400 aa
- **Resolution:** N/A
- **b-phipsi:** 0.011133
- **w-rdist:** 0.371577
- **t-alpha:** 0.027188
- **Chemical similarity (Tanimoto Index) (%):** 84.08
- **1D identity (%) [PDB]:** 3.11
- **1D identity (%) [Gaps excluded][PDB]:** 71.0
- **1D identity - Alignment Gaps [PDB]:** 2183
- **1D aligned content [PDB] (<aminoacid>:%):** {'L': 15.49, 'N': 2.82, 'D': 5.63, 'S': 8.45, 'R': 4.23, 'P': 2.82, 'A': 11.27, 'E': 5.63, 'I': 2.82, 'T': 7.04, 'G': 4.23, 'V': 4.23, 'Q': 7.04, 'K': 7.04, 'F': 5.63, 'H': 4.23, 'M': 1.41}
- **2D identity (%) [PDB]:** 34.63
- **2D identity (%) [Gaps excluded][PDB]:** 86.36
- **2D identity - Alignment Gaps [PDB]:** 1019
- **2D aligned content [PDB] (<2D-fold>:%):** {'.': 19.35, 'E': 38.2, 'T': 12.22, 'H': 29.71, 'G': 0.51}
- **3D similarity (TM-Score) (%) [PDB]:** 22.48

- **Gene name:** DMXL2
- **Entrez ID:** 23312
- **RefSeq ID:** NM\_015263
- **Transcript sequence length:** 10530
- **5-UTR|CDS|3-UTR identity (%):** 36.91 | 32.1 | 16.3
- **5-UTR|CDS|3-UTR identity (%) [Gaps excluded]:** 67.24 | 80.66 | 86.28
- **5-UTR|CDS|3-UTR identity [Alignment Gaps]:** 143 | 5569 | 970
- **5-UTR aligned content (<base>:%):** {'A': 19.66, 'T': 17.09, 'C': 29.06, 'G': 34.19}
- **CDS aligned content (<base>:%):** {'A': 30.4, 'T': 34.48, 'G': 17.85, 'C': 17.27}
- **3-UTR aligned content (<base>:%):** {'C': 15.9, 'A': 41.03, 'T': 25.13, 'G': 17.95}

**Uniprot Description:**  
  
 May serve as a scaffold protein for MADD and RAB3GA on synaptic vesicles (PubMed:11809763). Plays a role in the brain as a key controller of neuronal and endocrine homeostatic processes (By similarity).   
  
Interacts with MADD and RAB3GAP.   
  
 **Gene Ontology Information:**

Molecular Function

- small GTPase binding

Location

- extracellular space
- neuronal dense core vesicle
- RAVE complex
- synaptic vesicle
- synaptic vesicle membrane

Biological process

- vacuolar acidification

---

38

- **Protein name:** Translocation protein SEC63 homolog
- **Organism:** Homo sapiens
- **Uniprot Accession Number:** Q9UGP8
- **Protein sequence length:** 760 aa
- **1D identity (%):** 9.69
- **1D identity (%) [Gaps excluded]:** 20.12
- **1D identity - Alignment Gaps:** 711
- **1D aligned content (<aminoacid>:%):** {'G': 8.27, 'T': 5.26, 'Y': 7.52, 'L': 12.03, 'F': 8.27, 'W': 1.5, 'N': 4.51, 'R': 2.26, 'K': 7.52, 'P': 4.51, 'I': 4.51, 'A': 5.26, 'V': 7.52, 'E': 5.26, 'Q': 4.51, 'D': 4.51, 'S': 4.51, 'C': 1.5, 'H': 0.75}
- **Common reported functions (%):** 0.0
- **Common reported locations (%):** 0.0
- **Common reported processes (%):** 0.0

- **AF ID:** Q9UGP8
- **Chain:** A
- **Protein length:** 760 aa
- **Resolution:** N/A
- **b-phipsi:** 0.035252
- **w-rdist:** 0.31295
- **t-alpha:** 0.008341
- **Chemical similarity (Tanimoto Index) (%):** 84.16
- **1D identity (%) [PDB]:** 1.52
- **1D identity (%) [Gaps excluded][PDB]:** 78.79
- **1D identity - Alignment Gaps [PDB]:** 1677
- **1D aligned content [PDB] (<aminoacid>:%):** {'A': 11.54, 'S': 3.85, 'F': 3.85, 'V': 7.69, 'I': 11.54, 'R': 7.69, 'G': 7.69, 'D': 15.38, 'E': 3.85, 'P': 3.85, 'T': 3.85, 'K': 7.69, 'N': 3.85, 'Y': 3.85, 'L': 3.85}
- **2D identity (%) [PDB]:** 21.03
- **2D identity (%) [Gaps excluded][PDB]:** 86.76
- **2D identity - Alignment Gaps [PDB]:** 1063
- **2D aligned content [PDB] (<2D-fold>:%):** {'.': 16.27, 'E': 17.63, 'H': 56.27, 'T': 9.83}
- **3D similarity (TM-Score) (%) [PDB]:** 17.37

- **Gene name:** SEC63
- **Entrez ID:** 11231
- **RefSeq ID:** NM\_007214
- **Transcript sequence length:** 6430
- **5-UTR|CDS|3-UTR identity (%):** 31.58 | 37.41 | 5.52
- **5-UTR|CDS|3-UTR identity (%) [Gaps excluded]:** 72.34 | 76.97 | 95.61
- **5-UTR|CDS|3-UTR identity [Alignment Gaps]:** 182 | 2111 | 3721
- **5-UTR aligned content (<base>:%):** {'C': 25.49, 'T': 16.67, 'G': 42.16, 'A': 15.69}
- **CDS aligned content (<base>:%):** {'A': 36.37, 'T': 27.13, 'C': 16.01, 'G': 20.49}
- **3-UTR aligned content (<base>:%):** {'C': 15.14, 'A': 40.83, 'T': 23.85, 'G': 20.18}

**Uniprot Description:**  
  
 Mediates cotranslational and post-translational transport of certain precursor polypeptides across endoplasmic reticulum (ER) (PubMed:22375059, PubMed:29719251). Proposed to play an auxiliary role in recognition of precursors with short and apolar signal peptides. May cooperate with SEC62 and HSPA5/BiP to facilitate targeting of small presecretory proteins into the SEC61 channel-forming translocon complex, triggering channel opening for polypeptide translocation to the ER lumen (PubMed:29719251). Required for efficient PKD1/Polycystin-1 biogenesis and trafficking to the plasma membrane of the primary cilia (By similarity).   
  
The ER translocon complex consists of channel-forming core components SEC61A1, SEC61B and SEC61G and different auxiliary components such as SEC62 and SEC63.   
  
 **Gene Ontology Information:**

Molecular Function

- protein transmembrane transporter activity
- RNA binding
- signaling receptor activity

Location

- endoplasmic reticulum
- membrane
- Sec62/Sec63 complex

Biological process

- liver development
- multicellular organism aging
- nitrogen compound metabolic process
- posttranslational protein targeting to endoplasmic reticulum membrane
- posttranslational protein targeting to membrane, translocation
- protein targeting to membrane
- SRP-dependent cotranslational protein targeting to membrane

---

39

- **Protein name:** Laminin subunit alpha-2
- **Organism:** Homo sapiens
- **Uniprot Accession Number:** P24043
- **Protein sequence length:** 3122 aa
- **1D identity (%):** 12.63
- **1D identity (%) [Gaps excluded]:** 31.73
- **1D identity - Alignment Gaps:** 1893
- **1D aligned content (<aminoacid>:%):** {'M': 0.25, 'V': 4.53, 'L': 10.33, 'P': 8.06, 'Q': 2.27, 'C': 5.29, 'N': 7.3, 'T': 7.3, 'S': 3.53, 'G': 11.34, 'Y': 4.03, 'F': 6.3, 'H': 0.5, 'D': 6.8, 'I': 3.78, 'A': 6.8, 'E': 4.28, 'R': 2.27, 'K': 3.78, 'W': 1.26}
- **Common reported functions (%):** 0.0
- **Common reported locations (%):** 0.0
- **Common reported processes (%):** 0.0

- **AF ID:** P24043
- **Chain:** A
- **Protein length:** 1322 aa
- **Resolution:** N/A
- **b-phipsi:** 0.002912
- **w-rdist:** 0.433094
- **t-alpha:** 0.073615
- **Chemical similarity (Tanimoto Index) (%):** 86.11
- **1D identity (%) [PDB]:** 2.33
- **1D identity (%) [Gaps excluded][PDB]:** 74.29
- **1D identity - Alignment Gaps [PDB]:** 2165
- **1D aligned content [PDB] (<aminoacid>:%):** {'N': 9.62, 'R': 5.77, 'K': 9.62, 'I': 7.69, 'S': 9.62, 'A': 5.77, 'D': 11.54, 'V': 7.69, 'L': 5.77, 'F': 5.77, 'T': 3.85, 'Y': 5.77, 'G': 3.85, 'E': 3.85, 'Q': 1.92, 'P': 1.92}
- **2D identity (%) [PDB]:** 29.11
- **2D identity (%) [Gaps excluded][PDB]:** 84.43
- **2D identity - Alignment Gaps [PDB]:** 1123
- **2D aligned content [PDB] (<2D-fold>:%):** {'T': 16.43, '.': 24.25, 'E': 59.12, 'B': 0.2}
- **3D similarity (TM-Score) (%) [PDB]:** 21.48

- **Gene name:** LAMA2
- **Entrez ID:** 3908
- **RefSeq ID:** NM\_001079823
- **Transcript sequence length:** 9684
- **5-UTR|CDS|3-UTR identity (%):** 23.27 | 30.93 | 40.27
- **5-UTR|CDS|3-UTR identity (%) [Gaps excluded]:** 65.31 | 80.65 | 80.0
- **5-UTR|CDS|3-UTR identity [Alignment Gaps]:** 177 | 5873 | 148
- **5-UTR aligned content (<base>:%):** {'C': 34.38, 'T': 21.88, 'G': 26.56, 'A': 17.19}
- **CDS aligned content (<base>:%):** {'A': 30.41, 'T': 32.62, 'G': 18.7, 'C': 18.26}
- **3-UTR aligned content (<base>:%):** {'T': 30.83, 'A': 45.0, 'G': 11.67, 'C': 12.5}

**Uniprot Description:**  
  
 Binding to cells via a high affinity receptor, laminin is thought to mediate the attachment, migration and organization of cells into tissues during embryonic development by interacting with other extracellular matrix components.   
  
Laminin is a complex glycoprotein, consisting of three different polypeptide chains (alpha, beta, gamma), which are bound to each other by disulfide bonds into a cross-shaped molecule comprising one long and three short arms with globules at each end. Alpha-2 is a subunit of laminin-2 (laminin-211 or merosin), laminin-4 (laminin-221 or S-merosin) and laminin-12 (laminin-213). Interacts with FBLN1, FBLN2 and NID2.   
  
 **Gene Ontology Information:**

Molecular Function

- extracellular matrix structural constituent
- signaling receptor binding
- structural molecule activity

Location

- basement membrane
- collagen-containing extracellular matrix
- extracellular region
- neuromuscular junction
- protein complex involved in cell-matrix adhesion
- sarcolemma
- synaptic cleft

Biological process

- animal organ morphogenesis
- axon guidance
- cell adhesion
- maintenance of blood-brain barrier
- muscle organ development
- positive regulation of cell adhesion
- positive regulation of integrin-mediated signaling pathway
- positive regulation of muscle cell differentiation
- positive regulation of synaptic transmission, cholinergic
- regulation of basement membrane organization
- regulation of cell migration
- regulation of embryonic development
- tissue development

---

40

- **Protein name:** Complement C3
- **Organism:** Homo sapiens
- **Uniprot Accession Number:** P01024
- **Protein sequence length:** 1663 aa
- **1D identity (%):** 16.13
- **1D identity (%) [Gaps excluded]:** 24.81
- **1D identity - Alignment Gaps:** 622
- **1D aligned content (<aminoacid>:%):** {'S': 5.92, 'L': 9.76, 'T': 7.32, 'P': 7.32, 'Y': 3.48, 'R': 3.83, 'V': 7.67, 'D': 5.23, 'N': 3.48, 'F': 5.23, 'G': 13.24, 'K': 4.88, 'I': 3.14, 'E': 5.92, 'Q': 4.53, 'H': 1.05, 'A': 6.62, 'C': 1.39}
- **Common reported functions (%):** 0.0
- **Common reported locations (%):** 0.0
- **Common reported processes (%):** 0.0

- **AF ID:** P01024
- **Chain:** A
- **Protein length:** 1663 aa
- **Resolution:** N/A
- **b-phipsi:** 0.003577
- **w-rdist:** 0.444282
- **t-alpha:** 0.11326
- **Chemical similarity (Tanimoto Index) (%):** 85.55
- **1D identity (%) [PDB]:** 4.45
- **1D identity (%) [Gaps excluded][PDB]:** 72.08
- **1D identity - Alignment Gaps [PDB]:** 2338
- **1D aligned content [PDB] (<aminoacid>:%):** {'G': 7.21, 'S': 10.81, 'V': 9.91, 'T': 13.51, 'P': 2.7, 'N': 7.21, 'Q': 5.41, 'L': 6.31, 'Y': 4.5, 'D': 3.6, 'E': 6.31, 'R': 0.9, 'A': 6.31, 'H': 0.9, 'I': 9.01, 'M': 0.9, 'K': 3.6, 'F': 0.9}
- **2D identity (%) [PDB]:** N/A
- **2D identity (%) [Gaps excluded][PDB]:** N/A
- **2D identity - Alignment Gaps [PDB]:** N/A
- **2D aligned content [PDB] (<2D-fold>:%):** N/A
- **3D similarity (TM-Score) (%) [PDB]:** 25.59

- **Gene name:** C3
- **Entrez ID:** 100135462
- **RefSeq ID:** NM\_000064
- **Transcript sequence length:** 5231
- **5-UTR|CDS|3-UTR identity (%):** 14.34 | 42.01 | 32.03
- **5-UTR|CDS|3-UTR identity (%) [Gaps excluded]:** 72.22 | 73.59 | 71.43
- **5-UTR|CDS|3-UTR identity [Alignment Gaps]:** 218 | 2408 | 155
- **5-UTR aligned content (<base>:%):** {'A': 12.82, 'C': 43.59, 'T': 35.9, 'G': 7.69}
- **CDS aligned content (<base>:%):** {'A': 28.81, 'T': 25.37, 'G': 23.12, 'C': 22.7}
- **3-UTR aligned content (<base>:%):** {'C': 27.78, 'A': 24.44, 'T': 27.78, 'G': 20.0}

**Uniprot Description:**  
  
 C3 plays a central role in the activation of the complement system. Its processing by C3 convertase is the central reaction in both classical and alternative complement pathways. After activation C3b can bind covalently, via its reactive thioester, to cell surface carbohydrates or immune aggregates.   
  
C3 precursor is first processed by the removal of 4 Arg residues, forming two chains, beta and alpha, linked by a disulfide bond. C3 convertase activates C3 by cleaving the alpha chain, releasing C3a anaphylatoxin and generating C3b (beta chain + alpha' chain). Forms the pro-C3-convertase enzyme complex by interacting with Complement factor B Bb fragment (Bb), which is then stabilized by binding CFP, allowing the complex to become active (PubMed:28264884, PubMed:31507604). The interaction with Bb is dependent on Mg2+ (PubMed:31507604). C3b interacts with CR1 (via Sushi 8 and Sushi 9 domains) (PubMed:8175757, PubMed:2972794). C3b interacts with CFH (PubMed:21285368). C3d interacts with CFH (PubMed:21285368, PubMed:21317894). C3dg interacts with CR2 (via the N-terminal Sushi domains 1 and 2). During pregnancy, C3dg exists as a complex (probably a 2:2:2 heterohexamer) with AGT and the proform of PRG2. Interacts with VSIG4. Interacts (both C3a and ASP) with C5AR2; the interaction occurs with higher affinity for ASP, enhancing the phosphorylation and activation of C5AR2, recruitment of ARRB2 to the cell surface and endocytosis of GRP77.   
  
 **Gene Ontology Information:**

Molecular Function

- C5L2 anaphylatoxin chemotactic receptor binding
- endopeptidase inhibitor activity

Location

- extracellular space

Biological process

- complement activation, alternative pathway
- complement activation, classical pathway
- inflammatory response
- positive regulation of G protein-coupled receptor signaling pathway
- positive regulation of glucose transmembrane transport
- positive regulation of lipid storage
- positive regulation of protein phosphorylation
- regulation of triglyceride biosynthetic process

---

41

- **Protein name:** Low-density lipoprotein receptor-related protein 2
- **Organism:** Homo sapiens
- **Uniprot Accession Number:** P98164
- **Protein sequence length:** 4655 aa
- **1D identity (%):** 9.44
- **1D identity (%) [Gaps excluded]:** 35.08
- **1D identity - Alignment Gaps:** 3414
- **1D aligned content (<aminoacid>:%):** {'M': 0.91, 'L': 6.58, 'V': 4.76, 'S': 5.22, 'C': 7.03, 'T': 7.26, 'P': 7.94, 'Y': 5.22, 'F': 6.12, 'R': 3.4, 'G': 10.66, 'H': 1.81, 'Q': 2.49, 'D': 7.71, 'N': 7.71, 'I': 3.85, 'W': 1.36, 'A': 3.17, 'E': 2.95, 'K': 3.85}
- **Common reported functions (%):** 0.0
- **Common reported locations (%):** 20.0
- **Common reported processes (%):** 0.0

- **AF ID:** P98164
- **Chain:** A
- **Protein length:** 1400 aa
- **Resolution:** N/A
- **b-phipsi:** 0.014642
- **w-rdist:** 0.370883
- **t-alpha:** 0.126748
- **Chemical similarity (Tanimoto Index) (%):** 85.89
- **1D identity (%) [PDB]:** 2.61
- **1D identity (%) [Gaps excluded][PDB]:** 68.97
- **1D identity - Alignment Gaps [PDB]:** 2209
- **1D aligned content [PDB] (<aminoacid>:%):** {'S': 13.33, 'T': 10.0, 'A': 18.33, 'L': 13.33, 'G': 5.0, 'Q': 8.33, 'V': 8.33, 'N': 5.0, 'K': 1.67, 'I': 3.33, 'D': 3.33, 'P': 3.33, 'E': 1.67, 'Y': 1.67, 'R': 3.33}
- **2D identity (%) [PDB]:** 28.65
- **2D identity (%) [Gaps excluded][PDB]:** 80.77
- **2D identity - Alignment Gaps [PDB]:** 1135
- **2D aligned content [PDB] (<2D-fold>:%):** {'.': 16.87, 'E': 62.1, 'T': 17.86, 'G': 1.98, 'B': 0.2, 'H': 0.99}
- **3D similarity (TM-Score) (%) [PDB]:** 28.2

- **Gene name:** LRP2
- **Entrez ID:** 4036
- **RefSeq ID:** NM\_004525
- **Transcript sequence length:** 15657
- **5-UTR|CDS|3-UTR identity (%):** 31.25 | 22.68 | 13.04
- **5-UTR|CDS|3-UTR identity (%) [Gaps excluded]:** 80.36 | 84.98 | 89.82
- **5-UTR|CDS|3-UTR identity [Alignment Gaps]:** 176 | 10294 | 1331
- **5-UTR aligned content (<base>:%):** {'A': 17.78, 'C': 27.78, 'T': 17.78, 'G': 36.67}
- **CDS aligned content (<base>:%):** {'A': 29.51, 'T': 32.9, 'G': 18.87, 'C': 18.71}
- **3-UTR aligned content (<base>:%):** {'C': 14.29, 'A': 39.41, 'T': 24.14, 'G': 22.17}

**Uniprot Description:**  
  
 Multiligand endocytic receptor (By similarity). Acts together with CUBN to mediate endocytosis of high-density lipoproteins (By similarity). Mediates receptor-mediated uptake of polybasic drugs such as aprotinin, aminoglycosides and polymyxin B (By similarity). In the kidney, mediates the tubular uptake and clearance of leptin (By similarity). Also mediates transport of leptin across the blood-brain barrier through endocytosis at the choroid plexus epithelium (By similarity). Endocytosis of leptin in neuronal cells is required for hypothalamic leptin signaling and leptin-mediated regulation of feeding and body weight (By similarity). Mediates endocytosis and subsequent lysosomal degradation of CST3 in kidney proximal tubule cells (By similarity). Mediates renal uptake of 25-hydroxyvitamin D3 in complex with the vitamin D3 transporter GC/DBP (By similarity). Mediates renal uptake of metallothionein-bound heavy metals (PubMed:15126248). Together with CUBN, mediates renal reabsorption of myoglobin (By similarity). Mediates renal uptake and subsequent lysosomal degradation of APOM (By similarity). Plays a role in kidney selenium homeostasis by mediating renal endocytosis of selenoprotein SEPP1 (By similarity). Mediates renal uptake of the antiapoptotic protein BIRC5/survivin which may be important for functional integrity of the kidney (PubMed:23825075). Mediates renal uptake of matrix metalloproteinase MMP2 in complex with metalloproteinase inhibitor TIMP1 (By similarity). Mediates endocytosis of Sonic hedgehog protein N-product (ShhN), the active product of SHH (By similarity). Also mediates ShhN transcytosis (By similarity). In the embryonic neuroepithelium, mediates endocytic uptake and degradation of BMP4, is required for correct SHH localization in the ventral neural tube and plays a role in patterning of the ventral telencephalon (By similarity). Required at the onset of neurulation to sequester SHH on the apical surface of neuroepithelial cells of the rostral diencephalon ventral midline and to control PTCH1-dependent uptake and intracellular trafficking of SHH (By similarity). During neurulation, required in neuroepithelial cells for uptake of folate bound to the folate receptor FOLR1 which is necessary for neural tube closure (By similarity). In the adult brain, negatively regulates BMP signaling in the subependymal zone which enables neurogenesis to proceed (By similarity). In astrocytes, mediates endocytosis of ALB which is required for the synthesis of the neurotrophic factor oleic acid (By similarity). Involved in neurite branching (By similarity). During optic nerve development, required for SHH-mediated migration and proliferation of oligodendrocyte precursor cells (By similarity). Mediates endocytic uptake and clearance of SHH in the retinal margin which protects retinal progenitor cells from mitogenic stimuli and keeps them quiescent (By similarity). Plays a role in reproductive organ development by mediating uptake in reproductive tissues of androgen and estrogen bound to the sex hormone binding protein SHBG (By similarity). Mediates endocytosis of angiotensin-2 (By similarity). Also mediates endocytosis of angiotensis 1-7 (By similarity). Binds to the complex composed of beta-amyloid protein 40 and CLU/APOJ and mediates its endocytosis and lysosomal degradation (By similarity). Required for embryonic heart development (By similarity). Required for normal hearing, possibly through interaction with estrogen in the inner ear (By similarity).   
  
Binds plasminogen, extracellular matrix components, plasminogen activator-plasminogen activator inhibitor type I complex, apolipoprotein E-enriched beta-VLDL, lipoprotein lipase, lactoferrin, CLU/clusterin and calcium (PubMed:7768901). Forms a multimeric complex together with LRPAP1 (PubMed:1400426). Interacts (via PxLPxI/L motif) with ANKRA2 (via ankyrin repeats) (By similarity). Interacts with LRP2BP (PubMed:12508107). Interacts (via NPXY motif) with DAB2; the interaction is not affected by tyrosine phosphorylation of the NPXY motif (PubMed:10769163, PubMed:15134832). Interacts with MB (By similarity). Interacts with BMP4 (By similarity). Interacts with the Sonic hedgehog protein N-product which is the active product of SHH (By similarity). Interacts with CST3 in a calcium-dependent manner (PubMed:17462596). Interacts with the vitamin-D binding protein GC/DBP (By similarity). Interacts with sex hormone-binding protein SHBG (PubMed:16143106). Interacts with angiotensin-2 (By similarity). Also interacts with angiotensin 1-7 (By similarity). Interacts with APOM (By similarity). Interacts with selenoprotein SEPP1 (By similarity). Interacts with LEP (By similarity). Interacts with ALB (By similarity). Interacts with the antiapoptotic protein BIRC5/survivin (PubMed:23825075). Interacts with matrix metalloproteinase MMP2 in complex with metalloproteinase inhibitor TIMP1 (By similarity). In neurons, forms a trimeric complex with APP and APPB1/FE65 (By similarity). Interacts with LDLRAP1/ARH; mediates trafficking of LRP2 to the endocytic recycling compartment (By similarity). Does not interact with beta-amyloid protein 40 alone but interacts with the complex composed of beta-amyloid protein 40 and CLU/APOJ (By similarity). Interacts with MDK (By similarity).   
  
 **Gene Ontology Information:**

Molecular Function

- calcium ion binding
- cargo receptor activity
- chaperone binding
- hormone binding
- insulin-like growth factor I binding
- low-density lipoprotein particle receptor activity
- protein transporter activity
- SH3 domain binding

Location

- apical plasma membrane
- axon
- brush border membrane
- clathrin-coated endocytic vesicle membrane
- clathrin-coated pit
- dendrite
- endoplasmic reticulum
- endosome lumen
- external side of plasma membrane
- extracellular exosome
- Golgi apparatus
- lysosomal membrane
- lysosome
- plasma membrane
- receptor complex

Biological process

- amyloid-beta clearance
- aorta development
- cell population proliferation
- cellular response to growth factor stimulus
- cobalamin transport
- coronary artery morphogenesis
- diol metabolic process
- endocytosis
- folate import across plasma membrane
- forebrain development
- kidney development
- lipid metabolic process
- male gonad development
- metal ion transport
- negative regulation of apoptotic process
- negative regulation of BMP signaling pathway
- neural tube closure
- neuron projection arborization
- outflow tract septum morphogenesis
- positive regulation of lysosomal protein catabolic process
- positive regulation of neurogenesis
- positive regulation of oligodendrocyte progenitor proliferation
- positive regulation of protein kinase B signaling
- protein transport
- pulmonary artery morphogenesis
- receptor-mediated endocytosis
- response to leptin
- retinoid metabolic process
- secondary heart field specification
- sensory perception of sound
- transcytosis
- transport across blood-brain barrier
- vagina development
- ventricular compact myocardium morphogenesis
- ventricular septum development
- vitamin D metabolic process

---

42

- **Protein name:** Transient receptor potential cation channel subfamily V member 4
- **Organism:** Homo sapiens
- **Uniprot Accession Number:** Q9HBA0
- **Protein sequence length:** 871 aa
- **1D identity (%):** 12.06
- **1D identity (%) [Gaps excluded]:** 23.96
- **1D identity - Alignment Gaps:** 708
- **1D aligned content (<aminoacid>:%):** {'A': 5.23, 'D': 4.65, 'S': 5.81, 'E': 3.49, 'P': 8.14, 'G': 8.14, 'L': 12.21, 'N': 7.56, 'F': 5.23, 'R': 2.91, 'K': 5.23, 'Y': 4.07, 'V': 6.4, 'T': 9.3, 'I': 4.07, 'Q': 2.91, 'C': 1.16, 'M': 1.16, 'H': 1.16, 'W': 1.16}
- **Common reported functions (%):** 50.0
- **Common reported locations (%):** 20.0
- **Common reported processes (%):** 0.0

- **AF ID:** Q9HBA0
- **Chain:** A
- **Protein length:** 871 aa
- **Resolution:** N/A
- **b-phipsi:** 0.048406
- **w-rdist:** 0.257487
- **t-alpha:** 0.117375
- **Chemical similarity (Tanimoto Index) (%):** 86.19
- **1D identity (%) [PDB]:** 2.46
- **1D identity (%) [Gaps excluded][PDB]:** 68.75
- **1D identity - Alignment Gaps [PDB]:** 1726
- **1D aligned content [PDB] (<aminoacid>:%):** {'V': 6.82, 'L': 22.73, 'P': 6.82, 'Q': 4.55, 'G': 4.55, 'S': 2.27, 'E': 4.55, 'N': 6.82, 'T': 9.09, 'R': 4.55, 'A': 6.82, 'H': 2.27, 'Y': 9.09, 'F': 2.27, 'K': 2.27, 'D': 4.55}
- **2D identity (%) [PDB]:** 25.04
- **2D identity (%) [Gaps excluded][PDB]:** 79.5
- **2D identity - Alignment Gaps [PDB]:** 966
- **2D aligned content [PDB] (<2D-fold>:%):** {'.': 32.29, 'T': 11.61, 'H': 48.16, 'G': 1.98, 'B': 0.57, 'E': 5.38}
- **3D similarity (TM-Score) (%) [PDB]:** 18.74

- **Gene name:** TRPV4
- **Entrez ID:** 59341
- **RefSeq ID:** N/A
- **Sequence length:** N/A
- **5-UTR|CDS|3-UTR identity (%):** N/A | N/A | N/A
- **5-UTR|CDS|3-UTR identity (%) [Gaps excluded]:** N/A | N/A | N/A
- **5-UTR|CDS|3-UTR identity [Alignment Gaps]:** N/A | N/A | N/A
- **5-UTR aligned content (<base>:%):** N/A
- **CDS aligned content (<base>:%):** N/A
- **3-UTR aligned content (<base>:%):** N/A

**Uniprot Description:**  
  
 Non-selective calcium permeant cation channel involved in osmotic sensitivity and mechanosensitivity. Activation by exposure to hypotonicity within the physiological range exhibits an outward rectification (PubMed:18826956, PubMed:18695040, PubMed:29899501). Also activated by heat, low pH, citrate and phorbol esters (PubMed:16293632, PubMed:18826956, PubMed:18695040, PubMed:25256292, PubMed:20037586, PubMed:21964574). Increase of intracellular Ca(2+) potentiates currents. Channel activity seems to be regulated by a calmodulin-dependent mechanism with a negative feedback mechanism (PubMed:12724311, PubMed:18826956). Promotes cell-cell junction formation in skin keratinocytes and plays an important role in the formation and/or maintenance of functional intercellular barriers (By similarity). Acts as a regulator of intracellular Ca(2+) in synoviocytes (PubMed:19759329). Plays an obligatory role as a molecular component in the nonselective cation channel activation induced by 4-alpha-phorbol 12,13-didecanoate and hypotonic stimulation in synoviocytes and also regulates production of IL-8 (PubMed:19759329). Together with PKD2, forms mechano- and thermosensitive channels in cilium (PubMed:18695040). Negatively regulates expression of PPARGC1A, UCP1, oxidative metabolism and respiration in adipocytes (By similarity). Regulates expression of chemokines and cytokines related to pro-inflammatory pathway in adipocytes (By similarity). Together with AQP5, controls regulatory volume decrease in salivary epithelial cells (By similarity). Required for normal development and maintenance of bone and cartilage (PubMed:26249260). In its inactive state, may sequester DDX3X at the plasma membrane. When activated, the interaction between both proteins is affected and DDX3X relocalizes to the nucleus (PubMed:29899501).   
  
Homotetramer (Probable). Self-associates in an isoform-specific manner (PubMed:16293632). Isoform 1 and isoform 5 can oligomerize, but isoform 2, isoform 4 and isoform 6 cannot oligomerize (PubMed:16293632). Interacts with calmodulin (PubMed:12724311). Interacts with Map7 and Src family Tyr protein kinases LYN, SRC, FYN, HCK, LCK and YES (By similarity). Interacts with CTNNB1 (By similarity). The TRPV4 and CTNNB1 complex can interact with CDH1 (By similarity). Interacts with PACSIN1, PACSIN2 and PACSIN3 (via SH3 domain) (By similarity). Part of a complex containing MLC1, AQP4, HEPACAM and ATP1B1 (PubMed:22328087). Interacts with ITPR3 (PubMed:18826956). Interacts with AQP5; the interaction is probably indirect and regulates TRPV4 activation by hypotonicity (By similarity). Interacts with ANO1 (By similarity). Interacts (via C-terminus) with PKD2 (via C-terminus) (PubMed:18695040). Interacts with DDX3X; this interaction is decreased when the channel is activated (PubMed:29899501).   
  
 **Gene Ontology Information:**

Molecular Function

- actin binding
- actin filament binding
- alpha-tubulin binding
- ATP binding
- beta-tubulin binding
- calcium channel activity
- calmodulin binding
- cation channel activity
- identical protein binding
- ion channel activity
- lipid binding
- metal ion binding
- microtubule binding
- osmosensor activity
- protein kinase binding
- protein kinase C binding
- SH2 domain binding
- stretch-activated, monoatomic cation-selective, calcium channel activity

Location

- adherens junction
- apical plasma membrane
- cell surface
- cilium
- cortical actin cytoskeleton
- cytoplasmic microtubule
- cytoplasmic vesicle
- endoplasmic reticulum
- filopodium
- focal adhesion
- growth cone
- lamellipodium
- membrane
- plasma membrane
- ruffle membrane

Biological process

- actin cytoskeleton reorganization
- actin filament organization
- blood vessel endothelial cell delamination
- calcium ion import
- calcium ion import across plasma membrane
- calcium ion import into cytosol
- calcium ion transmembrane transport
- calcium ion transport
- cartilage development involved in endochondral bone morphogenesis
- cell volume homeostasis
- cell-cell junction assembly
- cellular calcium ion homeostasis
- cellular hypotonic response
- cellular hypotonic salinity response
- cellular response to heat
- cellular response to osmotic stress
- cortical microtubule organization
- diet induced thermogenesis
- energy homeostasis
- glucose homeostasis
- hyperosmotic salinity response
- microtubule polymerization
- multicellular organismal water homeostasis
- negative regulation of brown fat cell differentiation
- negative regulation of neuron projection development
- negative regulation of transcription by RNA polymerase II
- osmosensory signaling pathway
- positive regulation of chemokine (C-C motif) ligand 5 production
- positive regulation of chemokine (C-X-C motif) ligand 1 production
- positive regulation of cytosolic calcium ion concentration
- positive regulation of ERK1 and ERK2 cascade
- positive regulation of inflammatory response
- positive regulation of interleukin-6 production
- positive regulation of JNK cascade
- positive regulation of macrophage chemotaxis
- positive regulation of macrophage inflammatory protein 1 alpha production
- positive regulation of microtubule depolymerization
- positive regulation of monocyte chemotactic protein-1 production
- positive regulation of striated muscle contraction
- positive regulation of vascular permeability
- regulation of aerobic respiration
- regulation of response to osmotic stress
- response to insulin
- response to mechanical stimulus
- vasopressin secretion

---

43

- **Protein name:** Zinc finger protein 674
- **Organism:** Homo sapiens
- **Uniprot Accession Number:** Q2M3X9
- **Protein sequence length:** 581 aa
- **1D identity (%):** 9.71
- **1D identity (%) [Gaps excluded]:** 24.57
- **1D identity - Alignment Gaps:** 804
- **1D aligned content (<aminoacid>:%):** {'M': 1.55, 'F': 6.2, 'K': 8.53, 'V': 5.43, 'S': 7.75, 'N': 2.33, 'L': 5.43, 'R': 5.43, 'D': 1.55, 'G': 11.63, 'A': 3.88, 'C': 6.2, 'I': 4.65, 'Y': 4.65, 'Q': 2.33, 'T': 12.4, 'P': 4.65, 'E': 3.1, 'H': 1.55, 'W': 0.78}
- **Common reported functions (%):** 0.0
- **Common reported locations (%):** 0.0
- **Common reported processes (%):** 0.0

- **AF ID:** Q2M3X9
- **Chain:** A
- **Protein length:** 581 aa
- **Resolution:** N/A
- **b-phipsi:** 0.035444
- **w-rdist:** 0.3179
- **t-alpha:** 0.177215
- **Chemical similarity (Tanimoto Index) (%):** 83.58
- **1D identity (%) [PDB]:** 2.11
- **1D identity (%) [Gaps excluded][PDB]:** 71.11
- **1D identity - Alignment Gaps [PDB]:** 1474
- **1D aligned content [PDB] (<aminoacid>:%):** {'M': 3.12, 'A': 6.25, 'Y': 3.12, 'R': 3.12, 'F': 3.12, 'I': 9.38, 'G': 9.38, 'V': 3.12, 'Q': 12.5, 'N': 6.25, 'L': 12.5, 'K': 9.38, 'S': 15.62, 'T': 3.12}
- **2D identity (%) [PDB]:** 20.62
- **2D identity (%) [Gaps excluded][PDB]:** 87.58
- **2D identity - Alignment Gaps [PDB]:** 968
- **2D aligned content [PDB] (<2D-fold>:%):** {'.': 19.16, 'H': 65.52, 'E': 4.6, 'T': 10.73}
- **3D similarity (TM-Score) (%) [PDB]:** 15.26

- **Gene name:** ZNF674
- **Entrez ID:** 641339
- **RefSeq ID:** N/A
- **Sequence length:** N/A
- **5-UTR|CDS|3-UTR identity (%):** N/A | N/A | N/A
- **5-UTR|CDS|3-UTR identity (%) [Gaps excluded]:** N/A | N/A | N/A
- **5-UTR|CDS|3-UTR identity [Alignment Gaps]:** N/A | N/A | N/A
- **5-UTR aligned content (<base>:%):** N/A
- **CDS aligned content (<base>:%):** N/A
- **3-UTR aligned content (<base>:%):** N/A

**Uniprot Description:**  
  
 May be involved in transcriptional regulation. N/A   
  
 **Gene Ontology Information:**

Molecular Function

- DNA-binding transcription factor activity, RNA polymerase II-specific
- DNA-binding transcription repressor activity, RNA polymerase II-specific
- metal ion binding
- RNA polymerase II transcription regulatory region sequence-specific DNA binding

Location

- nucleus

Biological process

- negative regulation of transcription by RNA polymerase II
- regulation of transcription by RNA polymerase II

---

44

- **Protein name:** Retrotransposon Gag-like protein 3
- **Organism:** Homo sapiens
- **Uniprot Accession Number:** Q8N8U3
- **Protein sequence length:** 475 aa
- **1D identity (%):** 6.14
- **1D identity (%) [Gaps excluded]:** 24.71
- **1D identity - Alignment Gaps:** 1052
- **1D aligned content (<aminoacid>:%):** {'A': 11.63, 'Y': 2.33, 'L': 15.12, 'I': 6.98, 'W': 3.49, 'Q': 12.79, 'P': 5.81, 'E': 4.65, 'K': 2.33, 'D': 4.65, 'T': 4.65, 'V': 4.65, 'S': 4.65, 'F': 4.65, 'G': 3.49, 'C': 3.49, 'N': 3.49, 'H': 1.16}
- **Common reported functions (%):** 0.0
- **Common reported locations (%):** 0.0
- **Common reported processes (%):** 0.0

- **AF ID:** Q8N8U3
- **Chain:** A
- **Protein length:** 475 aa
- **Resolution:** N/A
- **b-phipsi:** 0.037535
- **w-rdist:** 0.312344
- **t-alpha:** 0.24
- **Chemical similarity (Tanimoto Index) (%):** 84.13
- **1D identity (%) [PDB]:** 2.65
- **1D identity (%) [Gaps excluded][PDB]:** 61.67
- **1D identity - Alignment Gaps [PDB]:** 1338
- **1D aligned content [PDB] (<aminoacid>:%):** {'A': 10.81, 'L': 13.51, 'Q': 21.62, 'I': 10.81, 'P': 2.7, 'F': 8.11, 'G': 5.41, 'V': 2.7, 'N': 5.41, 'E': 2.7, 'K': 5.41, 'S': 5.41, 'D': 2.7, 'T': 2.7}
- **2D identity (%) [PDB]:** 14.51
- **2D identity (%) [Gaps excluded][PDB]:** 92.89
- **2D identity - Alignment Gaps [PDB]:** 1064
- **2D aligned content [PDB] (<2D-fold>:%):** {'H': 86.34, '.': 7.65, 'G': 1.64, 'T': 4.37}
- **3D similarity (TM-Score) (%) [PDB]:** 12.7

- **Gene name:** RTL3
- **Entrez ID:** 203430
- **RefSeq ID:** NM\_152694
- **Transcript sequence length:** 2649
- **5-UTR|CDS|3-UTR identity (%):** 45.22 | 21.27 | 20.71
- **5-UTR|CDS|3-UTR identity (%) [Gaps excluded]:** 72.22 | 75.06 | 84.58
- **5-UTR|CDS|3-UTR identity [Alignment Gaps]:** 129 | 2932 | 700
- **5-UTR aligned content (<base>:%):** {'A': 22.44, 'T': 33.33, 'C': 26.92, 'G': 17.31}
- **CDS aligned content (<base>:%):** {'A': 31.84, 'T': 23.56, 'G': 20.11, 'C': 24.48}
- **3-UTR aligned content (<base>:%):** {'C': 15.1, 'A': 43.75, 'T': 25.0, 'G': 16.15}

**Uniprot Description:**  
  
 May function as a transcriptional regulator. Plays a role in postnatal myogenesis, may be involved in the regulation of satellite cells self-renewal. N/A   
  
 **Gene Ontology Information:**

Molecular Function

- nucleic acid binding
- zinc ion binding

Location

- nucleus

Biological process   
  
N/A

---

45

- **Protein name:** Dynein heavy chain domain-containing protein 1
- **Organism:** Homo sapiens
- **Uniprot Accession Number:** Q96M86
- **Protein sequence length:** 4753 aa
- **1D identity (%):** 9.24
- **1D identity (%) [Gaps excluded]:** 35.17
- **1D identity - Alignment Gaps:** 3518
- **1D aligned content (<aminoacid>:%):** {'M': 0.23, 'F': 6.58, 'V': 6.8, 'L': 14.51, 'P': 8.39, 'Q': 5.67, 'C': 3.85, 'T': 4.08, 'R': 4.08, 'Y': 2.49, 'D': 5.22, 'K': 3.17, 'H': 2.04, 'S': 5.67, 'W': 2.27, 'G': 9.3, 'N': 3.63, 'E': 4.08, 'A': 5.9, 'I': 2.04}
- **Common reported functions (%):** 0.0
- **Common reported locations (%):** 0.0
- **Common reported processes (%):** 0.0

- **AF ID:** Q96M86
- **Chain:** A
- **Protein length:** 1400 aa
- **Resolution:** N/A
- **b-phipsi:** 0.040139
- **w-rdist:** 0.304007
- **t-alpha:** 0.094118
- **Chemical similarity (Tanimoto Index) (%):** 84.05
- **1D identity (%) [PDB]:** 4.38
- **1D identity (%) [Gaps excluded][PDB]:** 66.22
- **1D identity - Alignment Gaps [PDB]:** 2087
- **1D aligned content [PDB] (<aminoacid>:%):** {'T': 6.12, 'A': 10.2, 'L': 13.27, 'G': 7.14, 'K': 2.04, 'Q': 8.16, 'D': 3.06, 'V': 10.2, 'N': 4.08, 'S': 9.18, 'F': 5.1, 'I': 2.04, 'P': 5.1, 'E': 5.1, 'R': 4.08, 'M': 1.02, 'H': 4.08}
- **2D identity (%) [PDB]:** 26.79
- **2D identity (%) [Gaps excluded][PDB]:** 83.28
- **2D identity - Alignment Gaps [PDB]:** 1223
- **2D aligned content [PDB] (<2D-fold>:%):** {'.': 27.54, 'E': 22.57, 'T': 10.97, 'H': 37.68, 'G': 1.24}
- **3D similarity (TM-Score) (%) [PDB]:** 20.55

- **Gene name:** DNHD1
- **Entrez ID:** 144132
- **RefSeq ID:** NM\_173589
- **Transcript sequence length:** 3226
- **5-UTR|CDS|3-UTR identity (%):** 28.86 | 24.53 | 24.37
- **5-UTR|CDS|3-UTR identity (%) [Gaps excluded]:** 78.43 | 76.11 | 84.47
- **5-UTR|CDS|3-UTR identity [Alignment Gaps]:** 438 | 2878 | 540
- **5-UTR aligned content (<base>:%):** {'A': 21.0, 'T': 31.0, 'G': 21.5, 'C': 26.5}
- **CDS aligned content (<base>:%):** {'A': 25.72, 'T': 28.79, 'G': 21.4, 'C': 24.09}
- **3-UTR aligned content (<base>:%):** {'A': 41.08, 'T': 22.16, 'C': 14.59, 'G': 22.16}

**Uniprot Description:**  
  
 Essential for the normal assembly and function of sperm flagella axonemes. N/A   
  
 **Gene Ontology Information:**

Molecular Function

- ATP binding
- dynein intermediate chain binding
- dynein light intermediate chain binding
- ATP-dependent microtubule motor activity, minus-end-directed

Location

- dynein complex
- extracellular exosome
- inner dynein arm
- sperm flagellum

Biological process

- cilium movement
- flagellated sperm motility
- microtubule-based movement
- sperm flagellum assembly

---

46

- **Protein name:** Melatonin-related receptor
- **Organism:** Homo sapiens
- **Uniprot Accession Number:** Q13585
- **Protein sequence length:** 617 aa
- **1D identity (%):** 8.96
- **1D identity (%) [Gaps excluded]:** 21.78
- **1D identity - Alignment Gaps:** 788
- **1D aligned content (<aminoacid>:%):** {'P': 6.67, 'T': 5.83, 'L': 7.5, 'G': 5.0, 'C': 2.5, 'V': 7.5, 'I': 5.0, 'D': 9.17, 'N': 7.5, 'A': 10.0, 'Y': 4.17, 'S': 8.33, 'F': 6.67, 'M': 2.5, 'E': 3.33, 'K': 2.5, 'R': 1.67, 'W': 0.83, 'Q': 0.83, 'H': 2.5}
- **Common reported functions (%):** 0.0
- **Common reported locations (%):** 20.0
- **Common reported processes (%):** 0.0

- **AF ID:** Q13585
- **Chain:** A
- **Protein length:** 617 aa
- **Resolution:** N/A
- **b-phipsi:** 0.047098
- **w-rdist:** 0.264987
- **t-alpha:** 0.023709
- **Chemical similarity (Tanimoto Index) (%):** 85.89
- **1D identity (%) [PDB]:** 1.79
- **1D identity (%) [Gaps excluded][PDB]:** 71.79
- **1D identity - Alignment Gaps [PDB]:** 1522
- **1D aligned content [PDB] (<aminoacid>:%):** {'V': 10.71, 'D': 7.14, 'L': 17.86, 'I': 14.29, 'G': 7.14, 'N': 10.71, 'T': 7.14, 'A': 14.29, 'Y': 7.14, 'E': 3.57}
- **2D identity (%) [PDB]:** 15.25
- **2D identity (%) [Gaps excluded][PDB]:** 88.14
- **2D identity - Alignment Gaps [PDB]:** 1128
- **2D aligned content [PDB] (<2D-fold>:%):** {'.': 12.98, 'E': 2.88, 'T': 4.81, 'H': 77.4, 'G': 1.92}
- **3D similarity (TM-Score) (%) [PDB]:** 14.97

- **Gene name:** GPR50
- **Entrez ID:** 9248
- **RefSeq ID:** NM\_004224
- **Transcript sequence length:** 2020
- **5-UTR|CDS|3-UTR identity (%):** 30.14 | 25.56 | 7.33
- **5-UTR|CDS|3-UTR identity (%) [Gaps excluded]:** 70.25 | 74.43 | 68.0
- **5-UTR|CDS|3-UTR identity [Alignment Gaps]:** 161 | 2774 | 207
- **5-UTR aligned content (<base>:%):** {'G': 23.53, 'C': 28.24, 'T': 28.24, 'A': 20.0}
- **CDS aligned content (<base>:%):** {'A': 24.72, 'C': 26.2, 'T': 29.63, 'G': 19.44}
- **3-UTR aligned content (<base>:%):** {'A': 23.53, 'T': 17.65, 'C': 29.41, 'G': 29.41}

**Uniprot Description:**  
  
 Does not bind melatonin.   
  
Homodimer, and heterodimer with MTNR1A and MTNR1B.   
  
 **Gene Ontology Information:**

Molecular Function

- G protein-coupled receptor activity
- melatonin receptor activity

Location

- nucleoplasm
- plasma membrane

Biological process

- cell-cell signaling
- G protein-coupled receptor signaling pathway

---

47

- **Protein name:** WD repeat-containing protein 36
- **Organism:** Homo sapiens
- **Uniprot Accession Number:** Q8NI36
- **Protein sequence length:** 951 aa
- **1D identity (%):** 13.82
- **1D identity (%) [Gaps excluded]:** 22.38
- **1D identity - Alignment Gaps:** 526
- **1D aligned content (<aminoacid>:%):** {'L': 12.11, 'A': 3.68, 'V': 9.47, 'C': 3.16, 'P': 4.74, 'K': 5.79, 'S': 10.0, 'R': 3.68, 'T': 6.32, 'F': 5.26, 'Y': 1.58, 'D': 5.79, 'N': 5.79, 'E': 2.63, 'G': 8.95, 'I': 6.84, 'W': 1.05, 'Q': 2.63, 'M': 0.53}
- **Common reported functions (%):** 0.0
- **Common reported locations (%):** 0.0
- **Common reported processes (%):** 0.0

- **AF ID:** Q8NI36
- **Chain:** A
- **Protein length:** 951 aa
- **Resolution:** N/A
- **b-phipsi:** 0.001999
- **w-rdist:** 0.50822
- **t-alpha:** 0.108158
- **Chemical similarity (Tanimoto Index) (%):** 83.63
- **1D identity (%) [PDB]:** 3.24
- **1D identity (%) [Gaps excluded][PDB]:** 72.29
- **1D identity - Alignment Gaps [PDB]:** 1768
- **1D aligned content [PDB] (<aminoacid>:%):** {'M': 3.33, 'I': 8.33, 'A': 13.33, 'Q': 10.0, 'T': 5.0, 'S': 15.0, 'L': 15.0, 'G': 10.0, 'F': 5.0, 'N': 6.67, 'K': 1.67, 'D': 1.67, 'V': 5.0}
- **2D identity (%) [PDB]:** 39.64
- **2D identity (%) [Gaps excluded][PDB]:** 85.62
- **2D identity - Alignment Gaps [PDB]:** 710
- **2D aligned content [PDB] (<2D-fold>:%):** {'.': 16.79, 'E': 47.14, 'T': 12.4, 'G': 1.72, 'H': 21.95}
- **3D similarity (TM-Score) (%) [PDB]:** 18.68

- **Gene name:** WDR36
- **Entrez ID:** 134430
- **RefSeq ID:** NM\_139281
- **Transcript sequence length:** 6416
- **5-UTR|CDS|3-UTR identity (%):** 28.31 | 38.18 | 5.94
- **5-UTR|CDS|3-UTR identity (%) [Gaps excluded]:** 75.49 | 75.94 | 93.89
- **5-UTR|CDS|3-UTR identity [Alignment Gaps]:** 170 | 2154 | 3390
- **5-UTR aligned content (<base>:%):** {'G': 31.17, 'T': 23.38, 'C': 25.97, 'A': 19.48}
- **CDS aligned content (<base>:%):** {'T': 33.86, 'G': 17.23, 'A': 31.26, 'C': 17.65}
- **3-UTR aligned content (<base>:%):** {'A': 40.47, 'T': 24.19, 'C': 14.88, 'G': 20.47}

**Uniprot Description:**  
  
 Involved in the nucleolar processing of SSU 18S rRNA. Involved in T-cell activation and highly coregulated with IL2. N/A   
  
 **Gene Ontology Information:**

Molecular Function

- RNA binding

Location

- nucleolus
- nucleoplasm
- Pwp2p-containing subcomplex of 90S preribosome
- small-subunit processome

Biological process

- response to stimulus
- rRNA processing
- visual perception

---

48

- **Protein name:** Sucrase-isomaltase, intestinal
- **Organism:** Homo sapiens
- **Uniprot Accession Number:** P14410
- **Protein sequence length:** 1827 aa
- **1D identity (%):** 17.03
- **1D identity (%) [Gaps excluded]:** 26.21
- **1D identity - Alignment Gaps:** 658
- **1D aligned content (<aminoacid>:%):** {'M': 0.94, 'F': 5.94, 'V': 4.69, 'L': 8.75, 'T': 8.75, 'P': 6.88, 'A': 6.25, 'R': 2.81, 'S': 6.88, 'W': 1.25, 'H': 1.56, 'N': 6.88, 'D': 7.19, 'I': 6.88, 'G': 9.06, 'Y': 4.69, 'K': 3.75, 'Q': 2.5, 'E': 2.5, 'C': 1.88}
- **Common reported functions (%):** 0.0
- **Common reported locations (%):** 20.0
- **Common reported processes (%):** 0.0

- **AF ID:** P14410
- **Chain:** A
- **Protein length:** 1827 aa
- **Resolution:** N/A
- **b-phipsi:** 0.00156
- **w-rdist:** 0.520609
- **t-alpha:** 0.072787
- **Chemical similarity (Tanimoto Index) (%):** 86.18
- **1D identity (%) [PDB]:** 3.68
- **1D identity (%) [Gaps excluded][PDB]:** 67.59
- **1D identity - Alignment Gaps [PDB]:** 2520
- **1D aligned content [PDB] (<aminoacid>:%):** {'A': 5.1, 'L': 12.24, 'T': 9.18, 'I': 9.18, 'V': 5.1, 'E': 4.08, 'N': 4.08, 'Q': 8.16, 'K': 9.18, 'P': 5.1, 'F': 6.12, 'G': 8.16, 'S': 7.14, 'D': 1.02, 'R': 1.02, 'M': 2.04, 'Y': 3.06}
- **2D identity (%) [PDB]:** N/A
- **2D identity (%) [Gaps excluded][PDB]:** N/A
- **2D identity - Alignment Gaps [PDB]:** N/A
- **2D aligned content [PDB] (<2D-fold>:%):** N/A
- **3D similarity (TM-Score) (%) [PDB]:** 29.08

- **Gene name:** SI
- **Entrez ID:** 6476
- **RefSeq ID:** NM\_001041
- **Transcript sequence length:** 6012
- **5-UTR|CDS|3-UTR identity (%):** 13.36 | 43.58 | 33.33
- **5-UTR|CDS|3-UTR identity (%) [Gaps excluded]:** 71.15 | 76.84 | 79.9
- **5-UTR|CDS|3-UTR identity [Alignment Gaps]:** 225 | 2570 | 285
- **5-UTR aligned content (<base>:%):** {'A': 29.73, 'T': 27.03, 'G': 21.62, 'C': 21.62}
- **CDS aligned content (<base>:%):** {'A': 31.72, 'T': 33.08, 'G': 18.2, 'C': 17.0}
- **3-UTR aligned content (<base>:%):** {'A': 41.72, 'T': 30.06, 'C': 14.11, 'G': 14.11}

**Uniprot Description:**  
  
 Plays an important role in the final stage of carbohydrate digestion. Isomaltase activity is specific for both alpha-1,4- and alpha-1,6-oligosaccharides.   
  
The resulting sucrase and isomaltase subunits stay associated with one another in a complex by non-covalent linkages.   
  
 **Gene Ontology Information:**

Molecular Function

- alpha-1,4-glucosidase activity
- carbohydrate binding
- hydrolase activity, hydrolyzing O-glycosyl compounds
- oligo-1,6-glucosidase activity
- sucrose alpha-glucosidase activity

Location

- apical plasma membrane
- brush border
- extracellular exosome
- Golgi apparatus
- plasma membrane

Biological process

- polysaccharide digestion
- sucrose catabolic process

---

49

- **Protein name:** Sodium-driven chloride bicarbonate exchanger
- **Organism:** Homo sapiens
- **Uniprot Accession Number:** Q6U841
- **Protein sequence length:** 1118 aa
- **1D identity (%):** 14.67
- **1D identity (%) [Gaps excluded]:** 22.14
- **1D identity - Alignment Gaps:** 485
- **1D aligned content (<aminoacid>:%):** {'M': 0.95, 'L': 13.27, 'E': 6.64, 'G': 9.0, 'I': 5.69, 'H': 1.9, 'D': 6.16, 'V': 7.11, 'P': 8.53, 'S': 7.58, 'R': 2.37, 'F': 5.21, 'T': 5.69, 'C': 2.84, 'N': 4.27, 'A': 6.16, 'Q': 2.37, 'K': 2.84, 'Y': 0.95, 'W': 0.47}
- **Common reported functions (%):** 0.0
- **Common reported locations (%):** 20.0
- **Common reported processes (%):** 0.0

- **AF ID:** Q6U841
- **Chain:** A
- **Protein length:** 1118 aa
- **Resolution:** N/A
- **b-phipsi:** 0.027528
- **w-rdist:** 0.346622
- **t-alpha:** 0.044665
- **Chemical similarity (Tanimoto Index) (%):** 85.75
- **1D identity (%) [PDB]:** 2.31
- **1D identity (%) [Gaps excluded][PDB]:** 75.81
- **1D identity - Alignment Gaps [PDB]:** 1977
- **1D aligned content [PDB] (<aminoacid>:%):** {'M': 2.13, 'S': 6.38, 'F': 6.38, 'P': 8.51, 'Q': 8.51, 'A': 8.51, 'V': 12.77, 'L': 2.13, 'H': 4.26, 'T': 10.64, 'E': 4.26, 'K': 4.26, 'N': 6.38, 'G': 6.38, 'W': 2.13, 'I': 4.26, 'D': 2.13}
- **2D identity (%) [PDB]:** 28.56
- **2D identity (%) [Gaps excluded][PDB]:** 84.88
- **2D identity - Alignment Gaps [PDB]:** 1043
- **2D aligned content [PDB] (<2D-fold>:%):** {'.': 30.29, 'T': 10.47, 'E': 15.81, 'H': 40.53, 'G': 2.9}
- **3D similarity (TM-Score) (%) [PDB]:** 21.61

- **Gene name:** SLC4A10
- **Entrez ID:** 57282
- **RefSeq ID:** NM\_022058
- **Transcript sequence length:** 5488
- **5-UTR|CDS|3-UTR identity (%):** 21.53 | 42.9 | 9.57
- **5-UTR|CDS|3-UTR identity (%) [Gaps excluded]:** 77.5 | 75.06 | 89.82
- **5-UTR|CDS|3-UTR identity [Alignment Gaps]:** 208 | 1933 | 1895
- **5-UTR aligned content (<base>:%):** {'A': 29.03, 'G': 25.81, 'C': 25.81, 'T': 19.35}
- **CDS aligned content (<base>:%):** {'T': 32.3, 'A': 30.39, 'G': 19.12, 'C': 18.19}
- **3-UTR aligned content (<base>:%):** {'C': 15.76, 'A': 41.38, 'T': 24.63, 'G': 18.23}

**Uniprot Description:**  
  
 Sodium/bicarbonate cotransporter which plays an important role in regulating intracellular pH (PubMed:18319254). Has been shown to act as a sodium/bicarbonate cotransporter in exchange for intracellular chloride (By similarity). Has also been shown to act as a sodium/biocarbonate cotransporter which does not couple net influx of bicarbonate to net efflux of chloride, with the observed chloride efflux being due to chloride self-exchange (PubMed:18319254). Controls neuronal pH and may contribute to the secretion of cerebrospinal fluid (By similarity). Reduces the excitability of CA1 pyramidal neurons and modulates short-term synaptic plasticity (By similarity). Required in retinal cells to maintain normal pH which is necessary for normal vision (By similarity). In the kidney, likely to mediate bicarbonate reclamation in the apical membrane of the proximal tubules (By similarity). N/A   
  
 **Gene Ontology Information:**

Molecular Function

- sodium,bicarbonate:chloride antiporter activity
- sodium:bicarbonate symporter activity
- inorganic anion exchanger activity
- transmembrane transporter activity

Location

- apical dendrite
- apical plasma membrane
- axon terminus
- basal dendrite
- basolateral plasma membrane
- CA3 pyramidal cell dendrite
- dendrite
- membrane
- neuronal cell body
- perikaryon
- plasma membrane
- postsynapse
- somatodendritic compartment
- synapse

Biological process

- bicarbonate transport
- brain morphogenesis
- chloride transport
- ion homeostasis
- locomotory exploration behavior
- multicellular organism growth
- post-embryonic development
- proton transmembrane transport
- pyramidal neuron development
- regulation of intracellular pH
- regulation of short-term neuronal synaptic plasticity
- response to light stimulus
- transmembrane transport
- visual perception

---

50

- **Protein name:** von Willebrand factor A domain-containing protein 5B2
- **Organism:** Homo sapiens
- **Uniprot Accession Number:** Q8N398
- **Protein sequence length:** 1242 aa
- **1D identity (%):** 15.13
- **1D identity (%) [Gaps excluded]:** 28.0
- **1D identity - Alignment Gaps:** 751
- **1D aligned content (<aminoacid>:%):** {'G': 12.96, 'S': 7.69, 'L': 13.77, 'T': 6.88, 'C': 4.86, 'N': 2.02, 'P': 10.53, 'V': 6.48, 'Y': 2.43, 'F': 4.45, 'A': 8.1, 'E': 2.83, 'R': 4.86, 'D': 4.45, 'Q': 4.05, 'I': 0.81, 'H': 1.21, 'K': 0.81, 'W': 0.4, 'M': 0.4}
- **Common reported functions (%):** 0.0
- **Common reported locations (%):** 0.0
- **Common reported processes (%):** 0.0

- **AF ID:** Q8N398
- **Chain:** A
- **Protein length:** 1242 aa
- **Resolution:** N/A
- **b-phipsi:** 0.030027
- **w-rdist:** 0.340285
- **t-alpha:** 0.210918
- **Chemical similarity (Tanimoto Index) (%):** 84.1
- **1D identity (%) [PDB]:** 1.98
- **1D identity (%) [Gaps excluded][PDB]:** 81.13
- **1D identity - Alignment Gaps [PDB]:** 2119
- **1D aligned content [PDB] (<aminoacid>:%):** {'P': 2.33, 'E': 9.3, 'A': 13.95, 'V': 9.3, 'Q': 11.63, 'R': 9.3, 'L': 13.95, 'T': 6.98, 'G': 4.65, 'S': 9.3, 'I': 4.65, 'K': 4.65}
- **2D identity (%) [PDB]:** 31.08
- **2D identity (%) [Gaps excluded][PDB]:** 81.17
- **2D identity - Alignment Gaps [PDB]:** 993
- **2D aligned content [PDB] (<2D-fold>:%):** {'.': 30.2, 'E': 30.6, 'T': 11.8, 'B': 0.2, 'H': 26.4, 'G': 0.8}
- **3D similarity (TM-Score) (%) [PDB]:** 21.11

- **Gene name:** VWA5B2
- **Entrez ID:** 90113
- **RefSeq ID:** NM\_138345
- **Transcript sequence length:** 4196
- **5-UTR|CDS|3-UTR identity (%):** 40.65 | 42.08 | 31.36
- **5-UTR|CDS|3-UTR identity (%) [Gaps excluded]:** 69.33 | 70.15 | 66.87
- **5-UTR|CDS|3-UTR identity [Alignment Gaps]:** 115 | 1889 | 188
- **5-UTR aligned content (<base>:%):** {'A': 7.08, 'C': 46.9, 'T': 17.7, 'G': 28.32}
- **CDS aligned content (<base>:%):** {'T': 25.73, 'C': 27.14, 'G': 25.33, 'A': 21.8}
- **3-UTR aligned content (<base>:%):** {'A': 25.23, 'G': 27.03, 'T': 23.42, 'C': 24.32}

**Uniprot Description:**  
  
 N/A N/A   
  
 **Gene Ontology Information:**

Molecular Function   
  
N/A

Location   
  
N/A

Biological process   
  
N/A

---

51

- **Protein name:** Ubiquitin carboxyl-terminal hydrolase 35
- **Organism:** Homo sapiens
- **Uniprot Accession Number:** Q9P2H5
- **Protein sequence length:** 1018 aa
- **1D identity (%):** 11.3
- **1D identity (%) [Gaps excluded]:** 21.63
- **1D identity - Alignment Gaps:** 719
- **1D aligned content (<aminoacid>:%):** {'D': 6.47, 'P': 8.24, 'L': 13.53, 'A': 10.0, 'G': 10.59, 'V': 4.71, 'F': 5.29, 'E': 5.88, 'T': 5.88, 'R': 4.12, 'Q': 2.94, 'C': 4.12, 'I': 2.94, 'S': 6.47, 'N': 2.35, 'W': 1.18, 'Y': 1.76, 'H': 1.18, 'K': 2.35}
- **Common reported functions (%):** 0.0
- **Common reported locations (%):** 0.0
- **Common reported processes (%):** 0.0

- **AF ID:** Q9P2H5
- **Chain:** A
- **Protein length:** 1018 aa
- **Resolution:** N/A
- **b-phipsi:** 0.050886
- **w-rdist:** 0.256418
- **t-alpha:** 0.033333
- **Chemical similarity (Tanimoto Index) (%):** 83.61
- **1D identity (%) [PDB]:** 2.55
- **1D identity (%) [Gaps excluded][PDB]:** 62.03
- **1D identity - Alignment Gaps [PDB]:** 1843
- **1D aligned content [PDB] (<aminoacid>:%):** {'D': 8.16, 'K': 8.16, 'E': 2.04, 'V': 8.16, 'F': 10.2, 'A': 6.12, 'Q': 4.08, 'I': 4.08, 'P': 10.2, 'G': 2.04, 'N': 4.08, 'S': 10.2, 'L': 12.24, 'R': 2.04, 'T': 4.08, 'M': 2.04, 'Y': 2.04}
- **2D identity (%) [PDB]:** 18.19
- **2D identity (%) [Gaps excluded][PDB]:** 90.45
- **2D identity - Alignment Gaps [PDB]:** 1331
- **2D aligned content [PDB] (<2D-fold>:%):** {'.': 13.2, 'H': 56.44, 'T': 11.88, 'G': 0.99, 'E': 17.49}
- **3D similarity (TM-Score) (%) [PDB]:** 20.04

- **Gene name:** USP35
- **Entrez ID:** 57558
- **RefSeq ID:** N/A
- **Sequence length:** N/A
- **5-UTR|CDS|3-UTR identity (%):** N/A | N/A | N/A
- **5-UTR|CDS|3-UTR identity (%) [Gaps excluded]:** N/A | N/A | N/A
- **5-UTR|CDS|3-UTR identity [Alignment Gaps]:** N/A | N/A | N/A
- **5-UTR aligned content (<base>:%):** N/A
- **CDS aligned content (<base>:%):** N/A
- **3-UTR aligned content (<base>:%):** N/A

**Uniprot Description:**  
  
 N/A N/A   
  
 **Gene Ontology Information:**

Molecular Function

- thiol-dependent ubiquitin-specific protease activity
- cysteine-type endopeptidase activity

Location

- cytosol
- nucleus

Biological process

- protein deubiquitination
- ubiquitin-dependent protein catabolic process

---

52

- **Protein name:** Integrin alpha-L
- **Organism:** Homo sapiens
- **Uniprot Accession Number:** P20701
- **Protein sequence length:** 1170 aa
- **1D identity (%):** 14.55
- **1D identity (%) [Gaps excluded]:** 22.79
- **1D identity - Alignment Gaps:** 539
- **1D aligned content (<aminoacid>:%):** {'D': 7.37, 'F': 7.83, 'A': 5.07, 'S': 8.29, 'V': 6.45, 'L': 11.98, 'G': 11.52, 'N': 4.61, 'T': 5.99, 'K': 2.76, 'P': 5.07, 'Y': 2.76, 'Q': 5.07, 'I': 4.15, 'R': 3.69, 'W': 0.46, 'E': 3.69, 'H': 0.92, 'C': 2.3}
- **Common reported functions (%):** 0.0
- **Common reported locations (%):** 0.0
- **Common reported processes (%):** 0.0

- **AF ID:** P20701
- **Chain:** A
- **Protein length:** 1170 aa
- **Resolution:** N/A
- **b-phipsi:** 0.007099
- **w-rdist:** 0.471498
- **t-alpha:** 0.006661
- **Chemical similarity (Tanimoto Index) (%):** 86.26
- **1D identity (%) [PDB]:** 2.8
- **1D identity (%) [Gaps excluded][PDB]:** 68.24
- **1D identity - Alignment Gaps [PDB]:** 1983
- **1D aligned content [PDB] (<aminoacid>:%):** {'A': 13.79, 'L': 17.24, 'N': 3.45, 'T': 5.17, 'V': 5.17, 'K': 5.17, 'S': 8.62, 'F': 1.72, 'G': 5.17, 'I': 8.62, 'R': 6.9, 'D': 3.45, 'P': 1.72, 'E': 6.9, 'Q': 3.45, 'Y': 1.72, 'M': 1.72}
- **2D identity (%) [PDB]:** 27.61
- **2D identity (%) [Gaps excluded][PDB]:** 81.47
- **2D identity - Alignment Gaps [PDB]:** 1063
- **2D aligned content [PDB] (<2D-fold>:%):** {'.': 25.68, 'E': 55.41, 'T': 12.16, 'H': 6.76}
- **3D similarity (TM-Score) (%) [PDB]:** 25.26

- **Gene name:** ITGAL
- **Entrez ID:** 281874
- **RefSeq ID:** NM\_002209
- **Transcript sequence length:** 5129
- **5-UTR|CDS|3-UTR identity (%):** 23.19 | 40.97 | 13.07
- **5-UTR|CDS|3-UTR identity (%) [Gaps excluded]:** 75.29 | 73.99 | 87.67
- **5-UTR|CDS|3-UTR identity [Alignment Gaps]:** 191 | 2107 | 1295
- **5-UTR aligned content (<base>:%):** {'A': 15.62, 'T': 26.56, 'C': 28.12, 'G': 29.69}
- **CDS aligned content (<base>:%):** {'A': 26.11, 'T': 28.23, 'G': 23.68, 'C': 21.98}
- **3-UTR aligned content (<base>:%):** {'C': 17.09, 'A': 41.21, 'T': 22.11, 'G': 19.6}

**Uniprot Description:**  
  
 Integrin ITGAL/ITGB2 is a receptor for ICAM1, ICAM2, ICAM3 and ICAM4. Integrin ITGAL/ITGB2 is a receptor for F11R (PubMed:11812992, PubMed:15528364). Integin ITGAL/ITGB2 is a receptor for the secreted form of ubiquitin-like protein ISG15; the interaction is mediated by ITGAL (PubMed:29100055). Involved in a variety of immune phenomena including leukocyte-endothelial cell interaction, cytotoxic T-cell mediated killing, and antibody dependent killing by granulocytes and monocytes. Contributes to natural killer cell cytotoxicity (PubMed:15356110). Involved in leukocyte adhesion and transmigration of leukocytes including T-cells and neutrophils (PubMed:11812992). Required for generation of common lymphoid progenitor cells in bone marrow, indicating a role in lymphopoiesis (By similarity). Integrin ITGAL/ITGB2 in association with ICAM3, contributes to apoptotic neutrophil phagocytosis by macrophages (PubMed:23775590).   
  
Heterodimer of an alpha and a beta subunit (PubMed:12526797). The ITGAL alpha subunit associates with the ITGB2 beta subunit (PubMed:12526797). Interacts with THBD (PubMed:27055590).   
  
 **Gene Ontology Information:**

Molecular Function

- ICAM-3 receptor activity
- integrin binding
- metal ion binding

Location

- external side of plasma membrane
- integrin alphaL-beta2 complex
- integrin complex

Biological process

- cell adhesion mediated by integrin
- cell-cell adhesion
- cell-matrix adhesion
- heterophilic cell-cell adhesion via plasma membrane cell adhesion molecules
- integrin-mediated signaling pathway
- leukocyte cell-cell adhesion
- memory T cell extravasation
- phagocytosis
- receptor clustering
- T cell activation via T cell receptor contact with antigen bound to MHC molecule on antigen presenting cell

---

53

- **Protein name:** U1 small nuclear ribonucleoprotein 70 kDa
- **Organism:** Homo sapiens
- **Uniprot Accession Number:** P08621
- **Protein sequence length:** 437 aa
- **1D identity (%):** 5.27
- **1D identity (%) [Gaps excluded]:** 19.56
- **1D identity - Alignment Gaps:** 984
- **1D aligned content (<aminoacid>:%):** {'Q': 2.82, 'F': 2.82, 'L': 2.82, 'E': 11.27, 'N': 7.04, 'P': 7.04, 'R': 14.08, 'K': 4.23, 'H': 1.41, 'G': 18.31, 'A': 4.23, 'T': 4.23, 'S': 7.04, 'Y': 4.23, 'D': 8.45}
- **Common reported functions (%):** 0.0
- **Common reported locations (%):** 0.0
- **Common reported processes (%):** 0.0

- **AF ID:** P08621
- **Chain:** A
- **Protein length:** 437 aa
- **Resolution:** N/A
- **b-phipsi:** 0.050243
- **w-rdist:** 0.271642
- **t-alpha:** 0.081395
- **Chemical similarity (Tanimoto Index) (%):** 83.27
- **1D identity (%) [PDB]:** 1.0
- **1D identity (%) [Gaps excluded][PDB]:** 77.78
- **1D identity - Alignment Gaps [PDB]:** 1384
- **1D aligned content [PDB] (<aminoacid>:%):** {'Q': 7.14, 'L': 7.14, 'P': 14.29, 'D': 7.14, 'S': 21.43, 'K': 14.29, 'R': 7.14, 'F': 7.14, 'I': 7.14, 'E': 7.14}
- **2D identity (%) [PDB]:** 17.28
- **2D identity (%) [Gaps excluded][PDB]:** 93.24
- **2D identity - Alignment Gaps [PDB]:** 976
- **2D aligned content [PDB] (<2D-fold>:%):** {'.': 19.32, 'T': 12.56, 'H': 67.15, 'E': 0.97}
- **3D similarity (TM-Score) (%) [PDB]:** 13.61

- **Gene name:** SNRNP70
- **Entrez ID:** 535113
- **RefSeq ID:** NM\_003089
- **Transcript sequence length:** 1671
- **5-UTR|CDS|3-UTR identity (%):** 33.99 | 16.34 | 32.84
- **5-UTR|CDS|3-UTR identity (%) [Gaps excluded]:** 67.1 | 69.82 | 74.79
- **5-UTR|CDS|3-UTR identity [Alignment Gaps]:** 151 | 3188 | 152
- **5-UTR aligned content (<base>:%):** {'A': 21.15, 'T': 13.46, 'C': 26.92, 'G': 38.46}
- **CDS aligned content (<base>:%):** {'A': 31.03, 'G': 26.62, 'C': 23.09, 'T': 19.26}
- **3-UTR aligned content (<base>:%):** {'A': 21.35, 'T': 32.58, 'C': 22.47, 'G': 23.6}

**Uniprot Description:**  
  
 Component of the spliceosomal U1 snRNP, which is essential for recognition of the pre-mRNA 5' splice-site and the subsequent assembly of the spliceosome (PubMed:19325628, PubMed:25555158). SNRNP70 binds to the loop I region of U1-snRNA (PubMed:2467746, PubMed:19325628, PubMed:25555158).   
  
Component of the U1 snRNP (PubMed:19325628, PubMed:21113136, PubMed:25555158). The U1 snRNP is composed of the U1 snRNA and the 7 core Sm proteins SNRPB, SNRPD1, SNRPD2, SNRPD3, SNRPE, SNRPF and SNRPG that assemble in a heptameric protein ring on the Sm site of the small nuclear RNA to form the core snRNP, and at least three U1 snRNP-specific proteins SNRNP70/U1-70K, SNRPA/U1-A and SNRPC/U1-C (PubMed:19325628, PubMed:21113136, PubMed:25555158). Interacts with SCNM1 (By similarity). Found in a pre-mRNA splicing complex with SFRS4, SFRS5, SNRNP70, SNRPA1, SRRM1 and SRRM2 (PubMed:9531537). Found in a pre-mRNA exonic splicing enhancer (ESE) complex with SNRNP70, SNRPA1, SRRM1 and TRA2B/SFRS10 (PubMed:10339552). Interacts with dephosphorylated SFRS13A and SFPQ (PubMed:11514619, PubMed:14765198). Interacts with NUDT21/CPSF5, CPSF6, SCAF11, and ZRANB2 (PubMed:14561889, PubMed:11448987, PubMed:9447963). Interacts with GEMIN5 (PubMed:25911097). Interacts with FUS.   
  
 **Gene Ontology Information:**

Molecular Function

- mRNA binding
- RNA binding
- snRNA binding
- U1 snRNA binding

Location

- nuclear speck
- nucleus
- spliceosomal complex
- U1 snRNP
- U2-type prespliceosome

Biological process

- mRNA splicing, via spliceosome
- regulation of RNA splicing

---

54

- **Protein name:** Zinc finger protein 544
- **Organism:** Homo sapiens
- **Uniprot Accession Number:** Q6NX49
- **Protein sequence length:** 715 aa
- **1D identity (%):** 10.59
- **1D identity (%) [Gaps excluded]:** 23.97
- **1D identity - Alignment Gaps:** 770
- **1D aligned content (<aminoacid>:%):** {'P': 5.48, 'V': 6.85, 'F': 2.74, 'W': 0.68, 'L': 10.27, 'D': 2.74, 'E': 6.85, 'G': 10.27, 'K': 4.11, 'S': 9.59, 'C': 10.27, 'N': 4.11, 'R': 2.74, 'Y': 4.79, 'Q': 4.79, 'T': 8.9, 'I': 2.74, 'H': 1.37, 'A': 0.68}
- **Common reported functions (%):** 0.0
- **Common reported locations (%):** 0.0
- **Common reported processes (%):** 0.0

- **AF ID:** Q6NX49
- **Chain:** A
- **Protein length:** 715 aa
- **Resolution:** N/A
- **b-phipsi:** 0.040448
- **w-rdist:** 0.314405
- **t-alpha:** 0.116625
- **Chemical similarity (Tanimoto Index) (%):** 83.92
- **1D identity (%) [PDB]:** 1.44
- **1D identity (%) [Gaps excluded][PDB]:** 75.0
- **1D identity - Alignment Gaps [PDB]:** 1634
- **1D aligned content [PDB] (<aminoacid>:%):** {'G': 8.33, 'K': 8.33, 'Q': 12.5, 'D': 8.33, 'S': 20.83, 'L': 16.67, 'T': 4.17, 'V': 8.33, 'N': 12.5}
- **2D identity (%) [PDB]:** 23.57
- **2D identity (%) [Gaps excluded][PDB]:** 89.8
- **2D identity - Alignment Gaps [PDB]:** 992
- **2D aligned content [PDB] (<2D-fold>:%):** {'.': 26.5, 'H': 51.74, 'T': 16.09, 'E': 5.68}
- **3D similarity (TM-Score) (%) [PDB]:** 15.23

- **Gene name:** ZNF544
- **Entrez ID:** 27300
- **RefSeq ID:** NM\_014480
- **Transcript sequence length:** 3302
- **5-UTR|CDS|3-UTR identity (%):** 41.49 | 32.42 | 21.88
- **5-UTR|CDS|3-UTR identity (%) [Gaps excluded]:** 71.65 | 76.99 | 87.89
- **5-UTR|CDS|3-UTR identity [Alignment Gaps]:** 141 | 2432 | 673
- **5-UTR aligned content (<base>:%):** {'A': 18.71, 'C': 29.5, 'T': 24.46, 'G': 27.34}
- **CDS aligned content (<base>:%):** {'A': 31.57, 'T': 28.63, 'G': 20.12, 'C': 19.68}
- **3-UTR aligned content (<base>:%):** {'A': 42.35, 'C': 15.31, 'T': 23.47, 'G': 18.88}

**Uniprot Description:**  
  
 May be involved in transcriptional regulation. N/A   
  
 **Gene Ontology Information:**

Molecular Function

- DNA-binding transcription activator activity, RNA polymerase II-specific
- metal ion binding
- RNA polymerase II cis-regulatory region sequence-specific DNA binding

Location

- nucleus

Biological process

- regulation of transcription by RNA polymerase II

---

55

- **Protein name:** Endogenous retrovirus group K member 5 Gag polyprotein
- **Organism:** Homo sapiens
- **Uniprot Accession Number:** Q9HDB9
- **Protein sequence length:** 667 aa
- **1D identity (%):** 10.62
- **1D identity (%) [Gaps excluded]:** 25.84
- **1D identity - Alignment Gaps:** 810
- **1D aligned content (<aminoacid>:%):** {'G': 10.27, 'Q': 10.96, 'T': 6.16, 'Y': 2.74, 'F': 1.37, 'K': 7.53, 'N': 7.53, 'L': 9.59, 'R': 4.79, 'I': 5.48, 'E': 3.42, 'A': 8.22, 'P': 7.53, 'S': 4.79, 'C': 1.37, 'V': 4.79, 'M': 2.05, 'D': 1.37}
- **Common reported functions (%):** 0.0
- **Common reported locations (%):** 20.0
- **Common reported processes (%):** 0.0

- **AF ID:** Q9HDB9
- **Chain:** A
- **Protein length:** 667 aa
- **Resolution:** N/A
- **b-phipsi:** 0.036095
- **w-rdist:** 0.320652
- **t-alpha:** 0.005824
- **Chemical similarity (Tanimoto Index) (%):** 84.13
- **1D identity (%) [PDB]:** 2.69
- **1D identity (%) [Gaps excluded][PDB]:** 79.63
- **1D identity - Alignment Gaps [PDB]:** 1542
- **1D aligned content [PDB] (<aminoacid>:%):** {'G': 6.98, 'I': 9.3, 'T': 4.65, 'Q': 16.28, 'N': 18.6, 'V': 2.33, 'Y': 2.33, 'K': 9.3, 'L': 9.3, 'A': 11.63, 'D': 4.65, 'S': 4.65}
- **2D identity (%) [PDB]:** 24.28
- **2D identity (%) [Gaps excluded][PDB]:** 83.11
- **2D identity - Alignment Gaps [PDB]:** 904
- **2D aligned content [PDB] (<2D-fold>:%):** {'H': 56.45, '.': 30.97, 'T': 8.71, 'G': 0.97, 'E': 2.9}
- **3D similarity (TM-Score) (%) [PDB]:** 15.52

- **Gene name:** ERVK-5
- **Entrez ID:** N/A
- **RefSeq ID:** N/A
- **Sequence length:** N/A
- **5-UTR|CDS|3-UTR identity (%):** N/A | N/A | N/A
- **5-UTR|CDS|3-UTR identity (%) [Gaps excluded]:** N/A | N/A | N/A
- **5-UTR|CDS|3-UTR identity [Alignment Gaps]:** N/A | N/A | N/A
- **5-UTR aligned content (<base>:%):** N/A
- **CDS aligned content (<base>:%):** N/A
- **3-UTR aligned content (<base>:%):** N/A

**Uniprot Description:**  
  
 The products of the Gag polyproteins of infectious retroviruses perform highly complex orchestrated tasks during the assembly, budding, maturation, and infection stages of the viral replication cycle. During viral assembly, the proteins form membrane associations and self-associations that ultimately result in budding of an immature virion from the infected cell. Gag precursors also function during viral assembly to selectively bind and package two plus strands of genomic RNA. Endogenous Gag proteins may have kept, lost or modified their original function during evolution. N/A   
  
 **Gene Ontology Information:**

Molecular Function

- nucleic acid binding
- structural molecule activity
- zinc ion binding

Location

- plasma membrane

Biological process

- viral process

---

56

- **Protein name:** Zinc finger protein 221
- **Organism:** Homo sapiens
- **Uniprot Accession Number:** Q9UK13
- **Protein sequence length:** 617 aa
- **1D identity (%):** 9.17
- **1D identity (%) [Gaps excluded]:** 21.79
- **1D identity - Alignment Gaps:** 770
- **1D aligned content (<aminoacid>:%):** {'P': 1.64, 'L': 10.66, 'E': 9.02, 'C': 5.74, 'F': 8.2, 'V': 4.1, 'G': 13.93, 'T': 5.74, 'A': 3.28, 'D': 2.46, 'R': 2.46, 'S': 9.84, 'I': 3.28, 'Q': 7.38, 'K': 6.56, 'N': 2.46, 'Y': 0.82, 'H': 1.64, 'W': 0.82}
- **Common reported functions (%):** 0.0
- **Common reported locations (%):** 0.0
- **Common reported processes (%):** 0.0

- **AF ID:** Q9UK13
- **Chain:** A
- **Protein length:** 617 aa
- **Resolution:** N/A
- **b-phipsi:** 0.037564
- **w-rdist:** 0.318693
- **t-alpha:** 0.116344
- **Chemical similarity (Tanimoto Index) (%):** 83.67
- **1D identity (%) [PDB]:** 1.86
- **1D identity (%) [Gaps excluded][PDB]:** 74.36
- **1D identity - Alignment Gaps [PDB]:** 1522
- **1D aligned content [PDB] (<aminoacid>:%):** {'I': 10.34, 'G': 10.34, 'K': 10.34, 'Q': 13.79, 'L': 13.79, 'S': 13.79, 'A': 13.79, 'N': 6.9, 'T': 3.45, 'F': 3.45}
- **2D identity (%) [PDB]:** 21.11
- **2D identity (%) [Gaps excluded][PDB]:** 85.76
- **2D identity - Alignment Gaps [PDB]:** 968
- **2D aligned content [PDB] (<2D-fold>:%):** {'.': 28.04, 'T': 9.59, 'H': 60.89, 'E': 1.48}
- **3D similarity (TM-Score) (%) [PDB]:** 16.17

- **Gene name:** ZNF221
- **Entrez ID:** 7638
- **RefSeq ID:** N/A
- **Sequence length:** N/A
- **5-UTR|CDS|3-UTR identity (%):** N/A | N/A | N/A
- **5-UTR|CDS|3-UTR identity (%) [Gaps excluded]:** N/A | N/A | N/A
- **5-UTR|CDS|3-UTR identity [Alignment Gaps]:** N/A | N/A | N/A
- **5-UTR aligned content (<base>:%):** N/A
- **CDS aligned content (<base>:%):** N/A
- **3-UTR aligned content (<base>:%):** N/A

**Uniprot Description:**  
  
 May be involved in transcriptional regulation. N/A   
  
 **Gene Ontology Information:**

Molecular Function

- DNA-binding transcription factor activity, RNA polymerase II-specific
- metal ion binding
- RNA polymerase II cis-regulatory region sequence-specific DNA binding

Location

- nucleus

Biological process

- regulation of transcription, DNA-templated

---

57

- **Protein name:** Dynein regulatory complex subunit 7
- **Organism:** Homo sapiens
- **Uniprot Accession Number:** Q8IY82
- **Protein sequence length:** 874 aa
- **1D identity (%):** 11.41
- **1D identity (%) [Gaps excluded]:** 23.1
- **1D identity - Alignment Gaps:** 727
- **1D aligned content (<aminoacid>:%):** {'L': 16.46, 'R': 4.27, 'E': 7.93, 'V': 7.32, 'P': 9.15, 'D': 6.71, 'S': 4.88, 'T': 7.93, 'A': 4.88, 'C': 4.27, 'F': 3.05, 'N': 3.66, 'K': 4.27, 'Y': 4.27, 'G': 3.66, 'H': 0.61, 'I': 4.27, 'Q': 2.44}
- **Common reported functions (%):** 0.0
- **Common reported locations (%):** 0.0
- **Common reported processes (%):** 0.0

- **AF ID:** Q8IY82
- **Chain:** A
- **Protein length:** 874 aa
- **Resolution:** N/A
- **b-phipsi:** 0.045584
- **w-rdist:** 0.307464
- **t-alpha:** 0.156938
- **Chemical similarity (Tanimoto Index) (%):** 83.93
- **1D identity (%) [PDB]:** 2.11
- **1D identity (%) [Gaps excluded][PDB]:** 66.67
- **1D identity - Alignment Gaps [PDB]:** 1743
- **1D aligned content [PDB] (<aminoacid>:%):** {'V': 7.89, 'L': 18.42, 'S': 5.26, 'E': 5.26, 'H': 2.63, 'P': 7.89, 'T': 13.16, 'K': 15.79, 'N': 10.53, 'G': 2.63, 'F': 5.26, 'Q': 5.26}
- **2D identity (%) [PDB]:** 31.73
- **2D identity (%) [Gaps excluded][PDB]:** 84.25
- **2D identity - Alignment Gaps [PDB]:** 841
- **2D aligned content [PDB] (<2D-fold>:%):** {'.': 19.16, 'T': 13.08, 'H': 41.12, 'E': 26.64}
- **3D similarity (TM-Score) (%) [PDB]:** 16.32

- **Gene name:** DRC7
- **Entrez ID:** 84229
- **RefSeq ID:** N/A
- **Sequence length:** N/A
- **5-UTR|CDS|3-UTR identity (%):** N/A | N/A | N/A
- **5-UTR|CDS|3-UTR identity (%) [Gaps excluded]:** N/A | N/A | N/A
- **5-UTR|CDS|3-UTR identity [Alignment Gaps]:** N/A | N/A | N/A
- **5-UTR aligned content (<base>:%):** N/A
- **CDS aligned content (<base>:%):** N/A
- **3-UTR aligned content (<base>:%):** N/A

**Uniprot Description:**  
  
 Component of the nexin-dynein regulatory complex (N-DRC) a key regulator of ciliary/flagellar motility which maintains the alignment and integrity of the distal axoneme and regulates microtubule sliding in motile axonemes (By similarity). Involved in the regulation of flagellar motility (By similarity). Essential for male fertility, sperm head morphogenesis and sperm flagellum formation (By similarity).   
  
Component of the nexin-dynein regulatory complex (N-DRC). Interacts with TCTE1/DRC5 (By similarity). Interacts with DRC3 and GAS8/DRC4 (By similarity).   
  
 **Gene Ontology Information:**

Molecular Function   
  
N/A

Location

- cytoplasm
- cytoskeleton
- motile cilium

Biological process

- cell motility
- flagellated sperm motility
- sperm axoneme assembly
- spermatogenesis

---

58

- **Protein name:** Mucin-5AC
- **Organism:** Homo sapiens
- **Uniprot Accession Number:** P98088
- **Protein sequence length:** 5654 aa
- **1D identity (%):** 7.85
- **1D identity (%) [Gaps excluded]:** 35.4
- **1D identity - Alignment Gaps:** 4413
- **1D aligned content (<aminoacid>:%):** {'M': 0.45, 'V': 6.07, 'L': 7.42, 'P': 9.44, 'S': 7.87, 'T': 11.46, 'A': 4.04, 'Y': 3.15, 'G': 10.56, 'D': 4.04, 'F': 4.49, 'W': 1.12, 'H': 1.57, 'K': 3.15, 'N': 5.39, 'E': 3.37, 'Q': 3.15, 'C': 7.42, 'R': 2.25, 'I': 3.6}
- **Common reported functions (%):** 0.0
- **Common reported locations (%):** 20.0
- **Common reported processes (%):** 0.0

- **AF ID:** P98088
- **Chain:** A
- **Protein length:** 1400 aa
- **Resolution:** N/A
- **b-phipsi:** 0.016171
- **w-rdist:** 0.378914
- **t-alpha:** 0.35153
- **Chemical similarity (Tanimoto Index) (%):** 85.51
- **1D identity (%) [PDB]:** 2.79
- **1D identity (%) [Gaps excluded][PDB]:** 70.33
- **1D identity - Alignment Gaps [PDB]:** 2201
- **1D aligned content [PDB] (<aminoacid>:%):** {'A': 6.25, 'T': 15.62, 'V': 10.94, 'G': 12.5, 'P': 4.69, 'S': 6.25, 'L': 6.25, 'K': 3.12, 'N': 6.25, 'F': 9.38, 'E': 4.69, 'R': 3.12, 'D': 6.25, 'I': 3.12, 'Q': 1.56}
- **2D identity (%) [PDB]:** 28.48
- **2D identity (%) [Gaps excluded][PDB]:** 84.81
- **2D identity - Alignment Gaps [PDB]:** 1185
- **2D aligned content [PDB] (<2D-fold>:%):** {'.': 17.72, 'E': 50.59, 'T': 15.16, 'B': 0.39, 'H': 13.19, 'G': 2.95}
- **3D similarity (TM-Score) (%) [PDB]:** 18.61

- **Gene name:** MUC5AC
- **Entrez ID:** 45050199
- **RefSeq ID:** NM\_001304359
- **Transcript sequence length:** 17448
- **5-UTR|CDS|3-UTR identity (%):** 11.03 | 18.24 | 29.1
- **5-UTR|CDS|3-UTR identity (%) [Gaps excluded]:** 75.0 | 82.58 | 80.23
- **5-UTR|CDS|3-UTR identity [Alignment Gaps]:** 232 | 13265 | 311
- **5-UTR aligned content (<base>:%):** {'T': 20.0, 'C': 40.0, 'G': 26.67, 'A': 13.33}
- **CDS aligned content (<base>:%):** {'A': 28.82, 'T': 30.04, 'G': 19.25, 'C': 21.89}
- **3-UTR aligned content (<base>:%):** {'C': 23.24, 'A': 28.17, 'T': 21.13, 'G': 27.46}

**Uniprot Description:**  
  
 Gel-forming glycoprotein of gastric and respiratory tract epithelia that protects the mucosa from infection and chemical damage by binding to inhaled microorganisms and particles that are subsequently removed by the mucociliary system (PubMed:14535999, PubMed:14718370). Interacts with H.pylori in the gastric epithelium, Barrett's esophagus as well as in gastric metaplasia of the duodenum (GMD) (PubMed:14535999).   
  
Homomultimer; disulfide-linked (PubMed:14718370). The N- and C-terminus mediate their assembly into higher order structures to form filaments (By similarity). The CTCK domains of two polypeptides associate in the endoplasmic reticulum to generate intermolecularly disulfide-bonded dimers (By similarity). These dimers progress to the Golgi apparatus, which is a more acidic environment than the endoplasmic reticulum. Under acidic conditions, the N-termini form non-covalent intermolecular interactions that juxtapose assemblies from different CTCK-linked dimers to produce long, disulfide-linked polymers that remain highly compact until secretion (By similarity).   
  
 **Gene Ontology Information:**

Molecular Function

- Kdo transferase activity

Location

- plasma membrane

Biological process

- lipopolysaccharide core region biosynthetic process

---

59

- **Protein name:** Endogenous retrovirus group K member 113 Pol protein
- **Organism:** Homo sapiens
- **Uniprot Accession Number:** P63132
- **Protein sequence length:** 956 aa
- **1D identity (%):** 15.2
- **1D identity (%) [Gaps excluded]:** 23.57
- **1D identity - Alignment Gaps:** 481
- **1D aligned content (<aminoacid>:%):** {'K': 5.34, 'G': 6.31, 'E': 4.37, 'P': 9.22, 'N': 3.88, 'Q': 4.85, 'L': 11.65, 'A': 6.8, 'H': 0.97, 'S': 6.8, 'W': 1.46, 'I': 6.31, 'D': 5.83, 'C': 4.85, 'F': 5.34, 'T': 6.8, 'R': 2.91, 'V': 4.37, 'Y': 1.94}
- **Common reported functions (%):** 0.0
- **Common reported locations (%):** 0.0
- **Common reported processes (%):** 0.0

- **AF ID:** P63132
- **Chain:** A
- **Protein length:** 956 aa
- **Resolution:** N/A
- **b-phipsi:** 0.009813
- **w-rdist:** 0.451681
- **t-alpha:** 0.00579
- **Chemical similarity (Tanimoto Index) (%):** 86.18
- **1D identity (%) [PDB]:** 2.45
- **1D identity (%) [Gaps excluded][PDB]:** 74.19
- **1D identity - Alignment Gaps [PDB]:** 1815
- **1D aligned content [PDB] (<aminoacid>:%):** {'N': 4.35, 'R': 4.35, 'L': 17.39, 'P': 6.52, 'Q': 4.35, 'G': 8.7, 'F': 6.52, 'S': 2.17, 'D': 4.35, 'I': 6.52, 'T': 8.7, 'A': 8.7, 'Y': 8.7, 'V': 4.35, 'K': 2.17, 'E': 2.17}
- **2D identity (%) [PDB]:** 33.05
- **2D identity (%) [Gaps excluded][PDB]:** 85.4
- **2D identity - Alignment Gaps [PDB]:** 857
- **2D aligned content [PDB] (<2D-fold>:%):** {'.': 23.16, 'E': 27.92, 'T': 11.26, 'H': 36.15, 'G': 1.52}
- **3D similarity (TM-Score) (%) [PDB]:** 18.57

- **Gene name:** HERVK\_113
- **Entrez ID:** N/A
- **RefSeq ID:** N/A
- **Sequence length:** N/A
- **5-UTR|CDS|3-UTR identity (%):** N/A | N/A | N/A
- **5-UTR|CDS|3-UTR identity (%) [Gaps excluded]:** N/A | N/A | N/A
- **5-UTR|CDS|3-UTR identity [Alignment Gaps]:** N/A | N/A | N/A
- **5-UTR aligned content (<base>:%):** N/A
- **CDS aligned content (<base>:%):** N/A
- **3-UTR aligned content (<base>:%):** N/A

**Uniprot Description:**  
  
 Early post-infection, the reverse transcriptase converts the viral RNA genome into double-stranded viral DNA. The RNase H domain of the reverse transcriptase performs two functions. It degrades the RNA template and specifically removes the RNA primer from the RNA/DNA hybrid. Following nuclear import, the integrase catalyzes the insertion of the linear, double-stranded viral DNA into the host cell chromosome. Endogenous Pol proteins may have kept, lost or modified their original function during evolution. N/A   
  
 **Gene Ontology Information:**

Molecular Function

- DNA binding
- RNA-directed DNA polymerase activity
- RNA-DNA hybrid ribonuclease activity
- zinc ion binding

Location   
  
N/A

Biological process

- DNA integration
- DNA recombination
- DNA repair

---

60

- **Protein name:** Leucine-rich repeat-containing protein 27
- **Organism:** Homo sapiens
- **Uniprot Accession Number:** Q9C0I9
- **Protein sequence length:** 530 aa
- **1D identity (%):** 7.16
- **1D identity (%) [Gaps excluded]:** 20.73
- **1D identity - Alignment Gaps:** 877
- **1D aligned content (<aminoacid>:%):** {'G': 10.42, 'Y': 2.08, 'V': 2.08, 'L': 16.67, 'A': 8.33, 'S': 12.5, 'K': 5.21, 'R': 5.21, 'P': 11.46, 'F': 1.04, 'I': 3.12, 'Q': 5.21, 'T': 5.21, 'H': 1.04, 'E': 2.08, 'N': 4.17, 'D': 4.17}
- **Common reported functions (%):** 0.0
- **Common reported locations (%):** 0.0
- **Common reported processes (%):** 0.0

- **AF ID:** Q9C0I9
- **Chain:** A
- **Protein length:** 530 aa
- **Resolution:** N/A
- **b-phipsi:** 0.041982
- **w-rdist:** 0.317385
- **t-alpha:** 0.166989
- **Chemical similarity (Tanimoto Index) (%):** 84.07
- **1D identity (%) [PDB]:** 2.32
- **1D identity (%) [Gaps excluded][PDB]:** 72.34
- **1D identity - Alignment Gaps [PDB]:** 1419
- **1D aligned content [PDB] (<aminoacid>:%):** {'N': 8.82, 'A': 11.76, 'L': 23.53, 'V': 5.88, 'Q': 5.88, 'S': 17.65, 'G': 5.88, 'D': 5.88, 'P': 5.88, 'E': 2.94, 'I': 2.94, 'T': 2.94}
- **2D identity (%) [PDB]:** 24.7
- **2D identity (%) [Gaps excluded][PDB]:** 86.87
- **2D identity - Alignment Gaps [PDB]:** 843
- **2D aligned content [PDB] (<2D-fold>:%):** {'.': 29.21, 'T': 9.62, 'E': 5.15, 'H': 56.01}
- **3D similarity (TM-Score) (%) [PDB]:** 12.56

- **Gene name:** LRRC27
- **Entrez ID:** 80313
- **RefSeq ID:** N/A
- **Sequence length:** N/A
- **5-UTR|CDS|3-UTR identity (%):** N/A | N/A | N/A
- **5-UTR|CDS|3-UTR identity (%) [Gaps excluded]:** N/A | N/A | N/A
- **5-UTR|CDS|3-UTR identity [Alignment Gaps]:** N/A | N/A | N/A
- **5-UTR aligned content (<base>:%):** N/A
- **CDS aligned content (<base>:%):** N/A
- **3-UTR aligned content (<base>:%):** N/A

**Uniprot Description:**  
  
 N/A N/A   
  
 **Gene Ontology Information:**

Molecular Function   
  
N/A

Location   
  
N/A

Biological process   
  
N/A

---

61

- **Protein name:** Frizzled-6
- **Organism:** Homo sapiens
- **Uniprot Accession Number:** O60353
- **Protein sequence length:** 706 aa
- **1D identity (%):** 10.38
- **1D identity (%) [Gaps excluded]:** 24.91
- **1D identity - Alignment Gaps:** 815
- **1D aligned content (<aminoacid>:%):** {'T': 8.28, 'F': 4.83, 'L': 13.79, 'P': 6.21, 'C': 6.21, 'G': 11.72, 'Y': 3.45, 'S': 8.28, 'V': 6.9, 'N': 4.14, 'I': 4.83, 'R': 2.76, 'K': 2.76, 'D': 4.83, 'Q': 4.14, 'E': 2.07, 'A': 3.45, 'H': 0.69, 'M': 0.69}
- **Common reported functions (%):** 0.0
- **Common reported locations (%):** 20.0
- **Common reported processes (%):** 0.0

- **AF ID:** O60353
- **Chain:** A
- **Protein length:** 706 aa
- **Resolution:** N/A
- **b-phipsi:** 0.03515
- **w-rdist:** 0.336532
- **t-alpha:** 0.066138
- **Chemical similarity (Tanimoto Index) (%):** 86.33
- **1D identity (%) [PDB]:** 2.2
- **1D identity (%) [Gaps excluded][PDB]:** 73.47
- **1D identity - Alignment Gaps [PDB]:** 1591
- **1D aligned content [PDB] (<aminoacid>:%):** {'F': 13.89, 'L': 13.89, 'P': 2.78, 'N': 8.33, 'V': 8.33, 'T': 11.11, 'W': 2.78, 'H': 2.78, 'A': 5.56, 'G': 5.56, 'S': 5.56, 'E': 2.78, 'K': 5.56, 'R': 2.78, 'I': 2.78, 'D': 2.78, 'Q': 2.78}
- **2D identity (%) [PDB]:** 21.37
- **2D identity (%) [Gaps excluded][PDB]:** 87.35
- **2D identity - Alignment Gaps [PDB]:** 1025
- **2D aligned content [PDB] (<2D-fold>:%):** {'H': 58.97, '.': 19.31, 'E': 4.14, 'T': 17.24, 'B': 0.34}
- **3D similarity (TM-Score) (%) [PDB]:** 17.85

- **Gene name:** FZD6
- **Entrez ID:** 403610
- **RefSeq ID:** NM\_003506
- **Transcript sequence length:** 3728
- **5-UTR|CDS|3-UTR identity (%):** 44.63 | 34.7 | 14.61
- **5-UTR|CDS|3-UTR identity (%) [Gaps excluded]:** 65.87 | 75.52 | 88.84
- **5-UTR|CDS|3-UTR identity [Alignment Gaps]:** 99 | 2201 | 1138
- **5-UTR aligned content (<base>:%):** {'T': 25.55, 'C': 22.63, 'G': 24.82, 'A': 27.01}
- **CDS aligned content (<base>:%):** {'A': 31.42, 'T': 32.13, 'G': 18.4, 'C': 18.05}
- **3-UTR aligned content (<base>:%):** {'A': 42.21, 'T': 24.62, 'C': 12.06, 'G': 21.11}

**Uniprot Description:**  
  
 Receptor for Wnt proteins. Most of frizzled receptors are coupled to the beta-catenin canonical signaling pathway, which leads to the activation of disheveled proteins, inhibition of GSK-3 kinase, nuclear accumulation of beta-catenin and activation of Wnt target genes. A second signaling pathway involving PKC and calcium fluxes has been seen for some family members, but it is not yet clear if it represents a distinct pathway or if it can be integrated in the canonical pathway, as PKC seems to be required for Wnt-mediated inactivation of GSK-3 kinase. Both pathways seem to involve interactions with G-proteins. May be involved in transduction and intercellular transmission of polarity information during tissue morphogenesis and/or in differentiated tissues. Together with FZD3, is involved in the neural tube closure and plays a role in the regulation of the establishment of planar cell polarity (PCP), particularly in the orientation of asymmetric bundles of stereocilia on the apical faces of a subset of auditory and vestibular sensory cells located in the inner ear (By similarity).   
  
Interacts with LMBR1L.   
  
 **Gene Ontology Information:**

Molecular Function

- G protein-coupled receptor activity
- Wnt-activated receptor activity
- Wnt-protein binding

Location

- apical plasma membrane
- cell surface
- cytoplasmic vesicle membrane
- endoplasmic reticulum membrane
- membrane
- plasma membrane

Biological process

- canonical Wnt signaling pathway
- nervous system development

---

62

- **Protein name:** Integrin alpha-IIb
- **Organism:** Homo sapiens
- **Uniprot Accession Number:** P08514
- **Protein sequence length:** 1039 aa
- **1D identity (%):** 15.73
- **1D identity (%) [Gaps excluded]:** 24.94
- **1D identity - Alignment Gaps:** 524
- **1D aligned content (<aminoacid>:%):** {'P': 8.07, 'L': 11.21, 'N': 3.14, 'Q': 5.38, 'Y': 3.14, 'S': 5.83, 'G': 9.87, 'A': 8.52, 'V': 9.42, 'R': 3.14, 'T': 5.83, 'E': 4.04, 'C': 3.59, 'D': 8.97, 'F': 4.48, 'W': 0.9, 'I': 1.79, 'K': 0.9, 'H': 1.35, 'M': 0.45}
- **Common reported functions (%):** 0.0
- **Common reported locations (%):** 0.0
- **Common reported processes (%):** 0.0

- **AF ID:** P08514
- **Chain:** A
- **Protein length:** 1039 aa
- **Resolution:** N/A
- **b-phipsi:** 0.025941
- **w-rdist:** 0.366776
- **t-alpha:** 0.079465
- **Chemical similarity (Tanimoto Index) (%):** 86.07
- **1D identity (%) [PDB]:** 2.73
- **1D identity (%) [Gaps excluded][PDB]:** 67.95
- **1D identity - Alignment Gaps [PDB]:** 1866
- **1D aligned content [PDB] (<aminoacid>:%):** {'A': 15.09, 'L': 22.64, 'Q': 15.09, 'V': 9.43, 'N': 1.89, 'T': 1.89, 'F': 1.89, 'G': 3.77, 'I': 5.66, 'S': 5.66, 'D': 3.77, 'P': 1.89, 'E': 5.66, 'R': 5.66}
- **2D identity (%) [PDB]:** 29.08
- **2D identity (%) [Gaps excluded][PDB]:** 84.88
- **2D identity - Alignment Gaps [PDB]:** 990
- **2D aligned content [PDB] (<2D-fold>:%):** {'.': 23.97, 'T': 12.56, 'E': 56.62, 'H': 6.85}
- **3D similarity (TM-Score) (%) [PDB]:** 22.95

- **Gene name:** ITGA2B
- **Entrez ID:** N/A
- **RefSeq ID:** N/A
- **Sequence length:** N/A
- **5-UTR|CDS|3-UTR identity (%):** N/A | N/A | N/A
- **5-UTR|CDS|3-UTR identity (%) [Gaps excluded]:** N/A | N/A | N/A
- **5-UTR|CDS|3-UTR identity [Alignment Gaps]:** N/A | N/A | N/A
- **5-UTR aligned content (<base>:%):** N/A
- **CDS aligned content (<base>:%):** N/A
- **3-UTR aligned content (<base>:%):** N/A

**Uniprot Description:**  
  
 Integrin alpha-IIb/beta-3 is a receptor for fibronectin, fibrinogen, plasminogen, prothrombin, thrombospondin and vitronectin. It recognizes the sequence R-G-D in a wide array of ligands. It recognizes the sequence H-H-L-G-G-G-A-K-Q-A-G-D-V in fibrinogen gamma chain. Following activation integrin alpha-IIb/beta-3 brings about platelet/platelet interaction through binding of soluble fibrinogen. This step leads to rapid platelet aggregation which physically plugs ruptured endothelial cell surface.   
  
Heterodimer of an alpha and a beta subunit. The alpha subunit is composed of a heavy and a light chain linked by a disulfide bond. Alpha-IIb associates with beta-3. Directly interacts with RNF181. Interacts (via C-terminus cytoplasmic tail region) with CIB1; the interaction is direct and calcium-dependent. Interacts (via C-terminus cytoplasmic tail region) with CIB2, CIB3 and CIB4; the interactions are stabilized/increased in a calcium and magnesium-dependent manner. ITGA2B:ITGB3 interacts with PPIA/CYPA; the interaction is ROS and PPIase activity-dependent and is increased in the presence of thrombin (By similarity).   
  
 **Gene Ontology Information:**

Molecular Function

- deacetylase activity
- hydrolase activity, acting on carbon-nitrogen (but not peptide) bonds, in linear amides
- metal ion binding

Location   
  
N/A

Biological process

- bacillithiol biosynthetic process

---

63

- **Protein name:** Ovostatin homolog 2
- **Organism:** Homo sapiens
- **Uniprot Accession Number:** Q6IE36
- **Protein sequence length:** 1433 aa
- **1D identity (%):** 17.43
- **1D identity (%) [Gaps excluded]:** 25.02
- **1D identity - Alignment Gaps:** 484
- **1D aligned content (<aminoacid>:%):** {'T': 5.76, 'L': 10.07, 'S': 8.27, 'P': 6.47, 'F': 8.99, 'N': 9.35, 'V': 7.55, 'G': 10.07, 'K': 6.47, 'A': 3.96, 'I': 4.32, 'Q': 4.32, 'D': 3.96, 'Y': 3.6, 'R': 2.16, 'E': 3.24, 'C': 1.08, 'H': 0.36}
- **Common reported functions (%):** 0.0
- **Common reported locations (%):** 0.0
- **Common reported processes (%):** 0.0

- **AF ID:** Q6IE36
- **Chain:** A
- **Protein length:** 1432 aa
- **Resolution:** N/A
- **b-phipsi:** 0.013966
- **w-rdist:** 0.38585
- **t-alpha:** 0.067824
- **Chemical similarity (Tanimoto Index) (%):** 86.05
- **1D identity (%) [PDB]:** 3.2
- **1D identity (%) [Gaps excluded][PDB]:** 70.48
- **1D identity - Alignment Gaps [PDB]:** 2205
- **1D aligned content [PDB] (<aminoacid>:%):** {'T': 9.46, 'E': 4.05, 'S': 10.81, 'V': 10.81, 'F': 8.11, 'N': 13.51, 'I': 2.7, 'L': 4.05, 'P': 4.05, 'A': 8.11, 'R': 4.05, 'W': 1.35, 'K': 4.05, 'D': 4.05, 'Y': 4.05, 'G': 4.05, 'Q': 2.7}
- **2D identity (%) [PDB]:** 42.25
- **2D identity (%) [Gaps excluded][PDB]:** 84.81
- **2D identity - Alignment Gaps [PDB]:** 809
- **2D aligned content [PDB] (<2D-fold>:%):** {'.': 17.47, 'E': 45.96, 'T': 11.89, 'B': 0.29, 'G': 0.44, 'H': 23.94}
- **3D similarity (TM-Score) (%) [PDB]:** 22.85

- **Gene name:** OVOS2
- **Entrez ID:** N/A
- **RefSeq ID:** N/A
- **Sequence length:** N/A
- **5-UTR|CDS|3-UTR identity (%):** N/A | N/A | N/A
- **5-UTR|CDS|3-UTR identity (%) [Gaps excluded]:** N/A | N/A | N/A
- **5-UTR|CDS|3-UTR identity [Alignment Gaps]:** N/A | N/A | N/A
- **5-UTR aligned content (<base>:%):** N/A
- **CDS aligned content (<base>:%):** N/A
- **3-UTR aligned content (<base>:%):** N/A

**Uniprot Description:**  
  
 Is able to inhibit all four classes of proteinases by a unique 'trapping' mechanism.   
  
Homotetramer.   
  
 **Gene Ontology Information:**

Molecular Function

- serine-type endopeptidase inhibitor activity

Location

- extracellular space

Biological process

- negative regulation of peptidase activity

---

64

- **Protein name:** Laminin subunit alpha-3
- **Organism:** Homo sapiens
- **Uniprot Accession Number:** Q16787
- **Protein sequence length:** 3333 aa
- **1D identity (%):** 12.22
- **1D identity (%) [Gaps excluded]:** 32.49
- **1D identity - Alignment Gaps:** 2088
- **1D aligned content (<aminoacid>:%):** {'M': 0.49, 'L': 10.76, 'T': 7.82, 'R': 2.93, 'P': 7.58, 'Y': 5.62, 'N': 7.82, 'G': 11.49, 'H': 1.96, 'Q': 4.16, 'F': 4.4, 'V': 5.13, 'W': 0.98, 'S': 5.38, 'A': 5.13, 'E': 3.42, 'I': 1.71, 'C': 4.4, 'K': 4.16, 'D': 4.65}
- **Common reported functions (%):** 0.0
- **Common reported locations (%):** 0.0
- **Common reported processes (%):** 0.0

- **AF ID:** Q16787
- **Chain:** A
- **Protein length:** 1400 aa
- **Resolution:** N/A
- **b-phipsi:** 0.022457
- **w-rdist:** 0.374013
- **t-alpha:** 0.134822
- **Chemical similarity (Tanimoto Index) (%):** 86.33
- **1D identity (%) [PDB]:** 3.11
- **1D identity (%) [Gaps excluded][PDB]:** 73.2
- **1D identity - Alignment Gaps [PDB]:** 2189
- **1D aligned content [PDB] (<aminoacid>:%):** {'N': 14.08, 'Y': 7.04, 'P': 7.04, 'D': 4.23, 'F': 9.86, 'G': 9.86, 'I': 1.41, 'S': 7.04, 'L': 7.04, 'K': 7.04, 'Q': 2.82, 'T': 4.23, 'V': 9.86, 'E': 4.23, 'H': 1.41, 'A': 2.82}
- **2D identity (%) [PDB]:** 30.0
- **2D identity (%) [Gaps excluded][PDB]:** 82.94
- **2D identity - Alignment Gaps [PDB]:** 1117
- **2D aligned content [PDB] (<2D-fold>:%):** {'.': 22.1, 'E': 55.81, 'T': 14.67, 'H': 5.52, 'G': 1.71, 'B': 0.19}
- **3D similarity (TM-Score) (%) [PDB]:** 21.41

- **Gene name:** LAMA3
- **Entrez ID:** 3909
- **RefSeq ID:** NM\_198129
- **Transcript sequence length:** 10651
- **5-UTR|CDS|3-UTR identity (%):** 36.2 | 29.27 | 36.96
- **5-UTR|CDS|3-UTR identity (%) [Gaps excluded]:** 69.41 | 80.34 | 79.13
- **5-UTR|CDS|3-UTR identity [Alignment Gaps]:** 156 | 6440 | 235
- **5-UTR aligned content (<base>:%):** {'A': 15.25, 'T': 16.95, 'C': 32.2, 'G': 35.59}
- **CDS aligned content (<base>:%):** {'A': 29.5, 'T': 31.46, 'G': 19.55, 'C': 19.49}
- **3-UTR aligned content (<base>:%):** {'C': 14.72, 'A': 47.24, 'G': 12.27, 'T': 25.77}

**Uniprot Description:**  
  
 Binding to cells via a high affinity receptor, laminin is thought to mediate the attachment, migration and organization of cells into tissues during embryonic development by interacting with other extracellular matrix components.   
  
Laminin is a complex glycoprotein, consisting of three different polypeptide chains (alpha, beta, gamma), which are bound to each other by disulfide bonds into a cross-shaped molecule comprising one long and three short arms with globules at each end. Alpha-3 is a subunit of laminin-5 (laminin-332 or epiligrin/kalinin/nicein), laminin-6 (laminin-311 or K-laminin) and laminin-7 (laminin-321 or KS-laminin).   
  
 **Gene Ontology Information:**

Molecular Function

- extracellular matrix structural constituent
- integrin binding
- structural molecule activity

Location

- adherens junction
- basement membrane
- collagen-containing extracellular matrix
- endoplasmic reticulum
- extracellular exosome
- extracellular region
- hemidesmosome
- laminin-3 complex
- laminin-5 complex

Biological process

- animal organ morphogenesis
- axon guidance
- cell migration
- cell-cell adhesion
- endodermal cell differentiation
- epidermis development
- hemidesmosome assembly
- integrin-mediated signaling pathway
- morphogenesis of a polarized epithelium
- regulation of cell adhesion
- regulation of cell migration
- regulation of embryonic development
- tissue development

---

65

- **Protein name:** SH2B adapter protein 3
- **Organism:** Homo sapiens
- **Uniprot Accession Number:** Q9UQQ2
- **Protein sequence length:** 575 aa
- **1D identity (%):** 8.0
- **1D identity (%) [Gaps excluded]:** 24.13
- **1D identity - Alignment Gaps:** 928
- **1D aligned content (<aminoacid>:%):** {'N': 3.6, 'A': 10.81, 'S': 8.11, 'C': 2.7, 'E': 5.41, 'L': 12.61, 'Q': 5.41, 'Y': 1.8, 'F': 8.11, 'P': 9.01, 'K': 4.5, 'G': 7.21, 'R': 2.7, 'W': 0.9, 'V': 5.41, 'I': 2.7, 'D': 4.5, 'T': 1.8, 'H': 2.7}
- **Common reported functions (%):** 0.0
- **Common reported locations (%):** 20.0
- **Common reported processes (%):** 0.0

- **AF ID:** Q9UQQ2
- **Chain:** A
- **Protein length:** 575 aa
- **Resolution:** N/A
- **b-phipsi:** 0.048007
- **w-rdist:** 0.30713
- **t-alpha:** 0.109174
- **Chemical similarity (Tanimoto Index) (%):** 83.61
- **1D identity (%) [PDB]:** 1.11
- **1D identity (%) [Gaps excluded][PDB]:** 77.27
- **1D identity - Alignment Gaps [PDB]:** 1514
- **1D aligned content [PDB] (<aminoacid>:%):** {'N': 5.88, 'V': 11.76, 'R': 5.88, 'D': 5.88, 'L': 23.53, 'P': 17.65, 'Q': 5.88, 'F': 5.88, 'S': 5.88, 'A': 5.88, 'E': 5.88}
- **2D identity (%) [PDB]:** 13.95
- **2D identity (%) [Gaps excluded][PDB]:** 82.67
- **2D identity - Alignment Gaps [PDB]:** 1108
- **2D aligned content [PDB] (<2D-fold>:%):** {'.': 19.89, 'T': 11.29, 'H': 50.54, 'G': 1.61, 'E': 16.67}
- **3D similarity (TM-Score) (%) [PDB]:** 13.51

- **Gene name:** SH2B3
- **Entrez ID:** 10019
- **RefSeq ID:** NM\_005475
- **Transcript sequence length:** 5431
- **5-UTR|CDS|3-UTR identity (%):** 28.09 | 23.58 | 6.53
- **5-UTR|CDS|3-UTR identity (%) [Gaps excluded]:** 74.58 | 69.49 | 95.59
- **5-UTR|CDS|3-UTR identity [Alignment Gaps]:** 293 | 2738 | 3096
- **5-UTR aligned content (<base>:%):** {'A': 15.15, 'C': 40.15, 'T': 18.18, 'G': 26.52}
- **CDS aligned content (<base>:%):** {'A': 21.8, 'G': 25.79, 'C': 29.48, 'T': 22.93}
- **3-UTR aligned content (<base>:%):** {'C': 14.75, 'A': 41.94, 'T': 23.5, 'G': 19.82}

**Uniprot Description:**  
  
 Links T-cell receptor activation signal to phospholipase C-gamma-1, GRB2 and phosphatidylinositol 3-kinase.   
  
Binds to the tyrosine-phosphorylated TCR zeta chain via its SH2 domain.   
  
 **Gene Ontology Information:**

Molecular Function

- protein tyrosine kinase binding
- signaling receptor complex adaptor activity
- stem cell factor receptor binding
- transmembrane receptor protein tyrosine kinase adaptor activity

Location

- cytosol
- plasma membrane

Biological process

- cellular response to chemokine
- cellular response to interleukin-3
- embryonic hemopoiesis
- erythrocyte development
- hematopoietic stem cell differentiation
- intracellular signal transduction
- megakaryocyte development
- monocyte homeostasis
- negative regulation of cell population proliferation
- negative regulation of chemokine-mediated signaling pathway
- negative regulation of Kit signaling pathway
- negative regulation of MAP kinase activity
- negative regulation of platelet aggregation
- negative regulation of protein kinase B signaling
- negative regulation of receptor signaling pathway via JAK-STAT
- negative regulation of response to cytokine stimulus
- negative regulation of tyrosine phosphorylation of STAT protein
- neutrophil homeostasis
- thrombopoietin-mediated signaling pathway

---

66

- **Protein name:** Testis anion transporter 1
- **Organism:** Homo sapiens
- **Uniprot Accession Number:** Q96RN1
- **Protein sequence length:** 970 aa
- **1D identity (%):** 12.22
- **1D identity (%) [Gaps excluded]:** 20.26
- **1D identity - Alignment Gaps:** 555
- **1D aligned content (<aminoacid>:%):** {'L': 9.94, 'R': 2.34, 'S': 12.87, 'A': 4.68, 'T': 8.19, 'F': 7.02, 'N': 7.6, 'I': 4.68, 'V': 5.26, 'C': 4.09, 'Y': 4.09, 'P': 7.6, 'G': 5.85, 'K': 3.51, 'Q': 5.26, 'D': 2.34, 'E': 3.51, 'M': 0.58, 'H': 0.58}
- **Common reported functions (%):** 0.0
- **Common reported locations (%):** 20.0
- **Common reported processes (%):** 0.0

- **AF ID:** Q96RN1
- **Chain:** A
- **Protein length:** 970 aa
- **Resolution:** N/A
- **b-phipsi:** 0.025897
- **w-rdist:** 0.368635
- **t-alpha:** 0.076096
- **Chemical similarity (Tanimoto Index) (%):** 83.78
- **1D identity (%) [PDB]:** 1.73
- **1D identity (%) [Gaps excluded][PDB]:** 73.33
- **1D identity - Alignment Gaps [PDB]:** 1863
- **1D aligned content [PDB] (<aminoacid>:%):** {'A': 12.12, 'Y': 6.06, 'T': 15.15, 'S': 21.21, 'L': 6.06, 'E': 3.03, 'V': 9.09, 'N': 3.03, 'I': 12.12, 'P': 3.03, 'M': 3.03, 'K': 3.03, 'D': 3.03}
- **2D identity (%) [PDB]:** 17.54
- **2D identity (%) [Gaps excluded][PDB]:** 88.82
- **2D identity - Alignment Gaps [PDB]:** 1309
- **2D aligned content [PDB] (<2D-fold>:%):** {'.': 16.78, 'T': 8.39, 'E': 11.89, 'H': 58.39, 'G': 4.2, 'B': 0.35}
- **3D similarity (TM-Score) (%) [PDB]:** 19.55

- **Gene name:** SLC26A8
- **Entrez ID:** 530509
- **RefSeq ID:** N/A
- **Sequence length:** N/A
- **5-UTR|CDS|3-UTR identity (%):** N/A | N/A | N/A
- **5-UTR|CDS|3-UTR identity (%) [Gaps excluded]:** N/A | N/A | N/A
- **5-UTR|CDS|3-UTR identity [Alignment Gaps]:** N/A | N/A | N/A
- **5-UTR aligned content (<base>:%):** N/A
- **CDS aligned content (<base>:%):** N/A
- **3-UTR aligned content (<base>:%):** N/A

**Uniprot Description:**  
  
 Acts as a DIDS-sensitive anion exchanger mediating chloride, sulfate and oxalate transport. May fulfill critical anion exchange functions in male germ line during meiosis and hence may play a role in spermatogenesis. May be involved in a new regulatory pathway linking sulfate transport to RhoGTPase signaling in male germ cells. A critical component of the sperm annulus that is essential for correct sperm tail differentiation and motility and hence male fertility. May form a molecular complex involved in the regulation of chloride and bicarbonate ions fluxes during sperm capacitation.   
  
Interacts with RACGAP1. Interacts with CFTR.   
  
 **Gene Ontology Information:**

Molecular Function

- bicarbonate transmembrane transporter activity
- chloride transmembrane transporter activity
- oxalate transmembrane transporter activity
- sulfate transmembrane transporter activity

Location

- plasma membrane

Biological process

- cell differentiation
- meiotic cell cycle
- spermatogenesis

---

67

- **Protein name:** Zinc finger protein 442
- **Organism:** Homo sapiens
- **Uniprot Accession Number:** Q9H7R0
- **Protein sequence length:** 627 aa
- **1D identity (%):** 8.36
- **1D identity (%) [Gaps excluded]:** 23.35
- **1D identity - Alignment Gaps:** 898
- **1D aligned content (<aminoacid>:%):** {'G': 12.82, 'F': 4.27, 'L': 8.55, 'N': 5.13, 'E': 5.13, 'D': 4.27, 'V': 2.56, 'A': 4.27, 'Q': 3.42, 'S': 8.55, 'R': 4.27, 'I': 2.56, 'T': 5.98, 'C': 10.26, 'K': 7.69, 'P': 4.27, 'Y': 5.98}
- **Common reported functions (%):** 0.0
- **Common reported locations (%):** 0.0
- **Common reported processes (%):** 0.0

- **AF ID:** Q9H7R0
- **Chain:** A
- **Protein length:** 627 aa
- **Resolution:** N/A
- **b-phipsi:** 0.024108
- **w-rdist:** 0.372781
- **t-alpha:** 0.084367
- **Chemical similarity (Tanimoto Index) (%):** 83.3
- **1D identity (%) [PDB]:** 0.94
- **1D identity (%) [Gaps excluded][PDB]:** 78.95
- **1D identity - Alignment Gaps [PDB]:** 1572
- **1D aligned content [PDB] (<aminoacid>:%):** {'I': 13.33, 'L': 13.33, 'P': 20.0, 'D': 13.33, 'S': 20.0, 'K': 6.67, 'R': 6.67, 'F': 6.67}
- **2D identity (%) [PDB]:** 21.09
- **2D identity (%) [Gaps excluded][PDB]:** 88.1
- **2D identity - Alignment Gaps [PDB]:** 988
- **2D aligned content [PDB] (<2D-fold>:%):** {'.': 22.63, 'T': 11.31, 'H': 61.68, 'E': 4.38}
- **3D similarity (TM-Score) (%) [PDB]:** 17.39

- **Gene name:** ZNF442
- **Entrez ID:** 79973
- **RefSeq ID:** N/A
- **Sequence length:** N/A
- **5-UTR|CDS|3-UTR identity (%):** N/A | N/A | N/A
- **5-UTR|CDS|3-UTR identity (%) [Gaps excluded]:** N/A | N/A | N/A
- **5-UTR|CDS|3-UTR identity [Alignment Gaps]:** N/A | N/A | N/A
- **5-UTR aligned content (<base>:%):** N/A
- **CDS aligned content (<base>:%):** N/A
- **3-UTR aligned content (<base>:%):** N/A

**Uniprot Description:**  
  
 May be involved in transcriptional regulation. N/A   
  
 **Gene Ontology Information:**

Molecular Function

- DNA-binding transcription factor activity, RNA polymerase II-specific
- metal ion binding
- RNA polymerase II transcription regulatory region sequence-specific DNA binding

Location

- nucleus

Biological process

- regulation of transcription by RNA polymerase II

---

68

- **Protein name:** Plexin-C1
- **Organism:** Homo sapiens
- **Uniprot Accession Number:** O60486
- **Protein sequence length:** 1568 aa
- **1D identity (%):** 17.77
- **1D identity (%) [Gaps excluded]:** 25.6
- **1D identity - Alignment Gaps:** 513
- **1D aligned content (<aminoacid>:%):** {'R': 2.01, 'P': 5.7, 'A': 4.03, 'Y': 3.02, 'G': 8.72, 'D': 7.72, 'V': 8.05, 'F': 4.36, 'S': 6.71, 'H': 0.67, 'L': 13.09, 'N': 8.72, 'T': 6.38, 'W': 0.34, 'C': 2.68, 'Q': 3.69, 'E': 4.36, 'K': 6.04, 'I': 3.36, 'M': 0.34}
- **Common reported functions (%):** 0.0
- **Common reported locations (%):** 20.0
- **Common reported processes (%):** 0.0

- **AF ID:** O60486
- **Chain:** A
- **Protein length:** 1568 aa
- **Resolution:** N/A
- **b-phipsi:** 0.004977
- **w-rdist:** 0.606515
- **t-alpha:** 0.006661
- **Chemical similarity (Tanimoto Index) (%):** 86.14
- **1D identity (%) [PDB]:** 3.76
- **1D identity (%) [Gaps excluded][PDB]:** 70.0
- **1D identity - Alignment Gaps [PDB]:** 2291
- **1D aligned content [PDB] (<aminoacid>:%):** {'A': 10.99, 'I': 6.59, 'G': 6.59, 'S': 9.89, 'L': 12.09, 'T': 5.49, 'K': 7.69, 'V': 10.99, 'N': 5.49, 'Q': 4.4, 'D': 3.3, 'R': 3.3, 'P': 1.1, 'E': 4.4, 'Y': 1.1, 'M': 1.1, 'F': 2.2, 'H': 3.3}
- **2D identity (%) [PDB]:** N/A
- **2D identity (%) [Gaps excluded][PDB]:** N/A
- **2D identity - Alignment Gaps [PDB]:** N/A
- **2D aligned content [PDB] (<2D-fold>:%):** N/A
- **3D similarity (TM-Score) (%) [PDB]:** 26.23

- **Gene name:** PLXNC1
- **Entrez ID:** 10154
- **RefSeq ID:** NM\_005761
- **Transcript sequence length:** 7492
- **5-UTR|CDS|3-UTR identity (%):** 32.16 | 43.43 | 8.78
- **5-UTR|CDS|3-UTR identity (%) [Gaps excluded]:** 70.87 | 73.8 | 92.51
- **5-UTR|CDS|3-UTR identity [Alignment Gaps]:** 248 | 2209 | 2165
- **5-UTR aligned content (<base>:%):** {'C': 34.93, 'A': 17.81, 'G': 30.82, 'T': 16.44}
- **CDS aligned content (<base>:%):** {'A': 30.62, 'T': 29.07, 'G': 20.93, 'C': 19.38}
- **3-UTR aligned content (<base>:%):** {'C': 15.24, 'A': 40.95, 'T': 23.81, 'G': 20.0}

**Uniprot Description:**  
  
 Receptor for SEMA7A, for smallpox semaphorin A39R, vaccinia virus semaphorin A39R and for herpesvirus Sema protein. Binding of semaphorins triggers cellular responses leading to the rearrangement of the cytoskeleton and to secretion of IL6 and IL8 (By similarity).   
  
Monomer. Homodimer. Interacts with SEMA7A.   
  
 **Gene Ontology Information:**

Molecular Function

- semaphorin receptor activity
- signaling receptor binding

Location

- cerebellar climbing fiber to Purkinje cell synapse
- membrane
- plasma membrane
- semaphorin receptor complex

Biological process

- cell adhesion
- negative regulation of cell adhesion
- positive regulation of axonogenesis
- regulation of cell migration
- regulation of cell shape
- regulation of GTPase activity
- regulation of synapse pruning
- semaphorin-plexin signaling pathway involved in axon guidance

---

69

- **Protein name:** Ribonucleases P/MRP protein subunit POP1
- **Organism:** Homo sapiens
- **Uniprot Accession Number:** Q99575
- **Protein sequence length:** 1024 aa
- **1D identity (%):** 12.97
- **1D identity (%) [Gaps excluded]:** 22.84
- **1D identity - Alignment Gaps:** 633
- **1D aligned content (<aminoacid>:%):** {'N': 7.37, 'K': 6.32, 'P': 8.95, 'T': 7.89, 'V': 6.84, 'S': 7.89, 'G': 11.05, 'A': 5.79, 'L': 10.0, 'D': 4.74, 'E': 2.63, 'I': 5.26, 'Y': 0.53, 'Q': 2.63, 'R': 2.63, 'C': 4.21, 'F': 3.68, 'W': 1.05, 'H': 0.53}
- **Common reported functions (%):** 0.0
- **Common reported locations (%):** 0.0
- **Common reported processes (%):** 0.0

- **AF ID:** Q99575
- **Chain:** A
- **Protein length:** 1024 aa
- **Resolution:** N/A
- **b-phipsi:** 0.013515
- **w-rdist:** 0.47657
- **t-alpha:** 0.002481
- **Chemical similarity (Tanimoto Index) (%):** 83.51
- **1D identity (%) [PDB]:** 2.58
- **1D identity (%) [Gaps excluded][PDB]:** 71.43
- **1D identity - Alignment Gaps [PDB]:** 1867
- **1D aligned content [PDB] (<aminoacid>:%):** {'L': 14.0, 'P': 8.0, 'D': 2.0, 'S': 6.0, 'K': 6.0, 'N': 4.0, 'T': 10.0, 'F': 6.0, 'G': 10.0, 'V': 4.0, 'E': 2.0, 'M': 2.0, 'I': 8.0, 'A': 10.0, 'W': 2.0, 'Q': 2.0, 'Y': 2.0, 'R': 2.0}
- **2D identity (%) [PDB]:** 26.99
- **2D identity (%) [Gaps excluded][PDB]:** 87.53
- **2D identity - Alignment Gaps [PDB]:** 1061
- **2D aligned content [PDB] (<2D-fold>:%):** {'.': 21.01, 'H': 40.34, 'T': 14.01, 'G': 2.17, 'E': 22.46}
- **3D similarity (TM-Score) (%) [PDB]:** 17.57

- **Gene name:** POP1
- **Entrez ID:** 10940
- **RefSeq ID:** NM\_015029
- **Transcript sequence length:** 4667
- **5-UTR|CDS|3-UTR identity (%):** 5.07 | 42.6 | 12.76
- **5-UTR|CDS|3-UTR identity (%) [Gaps excluded]:** 82.35 | 74.36 | 88.5
- **5-UTR|CDS|3-UTR identity [Alignment Gaps]:** 259 | 1873 | 1341
- **5-UTR aligned content (<base>:%):** {'G': 21.43, 'T': 35.71, 'A': 21.43, 'C': 21.43}
- **CDS aligned content (<base>:%):** {'A': 30.94, 'G': 22.11, 'T': 26.45, 'C': 20.5}
- **3-UTR aligned content (<base>:%):** {'A': 42.0, 'T': 24.0, 'C': 14.5, 'G': 19.5}

**Uniprot Description:**  
  
 Component of ribonuclease P, a ribonucleoprotein complex that generates mature tRNA molecules by cleaving their 5'-ends (PubMed:8918471, PubMed:30454648). Also a component of the MRP ribonuclease complex, which cleaves pre-rRNA sequences (PubMed:28115465).   
  
Component of nuclear RNase P and RNase MRP ribonucleoproteins (PubMed:8918471, PubMed:16723659). RNase P consists of a catalytic RNA moiety and 10 different protein chains; POP1, POP4, POP5, POP7, RPP14, RPP21, RPP25, RPP30, RPP38 and RPP40 (PubMed:16723659, PubMed:30454648). Within the RNase P complex, POP1, POP7 and RPP25 form the 'finger' subcomplex, POP5, RPP14, RPP40 and homodimeric RPP30 form the 'palm' subcomplex, and RPP21, POP4 and RPP38 form the 'wrist' subcomplex. All subunits of the RNase P complex interact with the catalytic RNA (PubMed:30454648). Several subunits of RNase P are also part of the RNase MRP complex. RNase MRP consists of a catalytic RNA moiety and about 8 protein subunits; POP1, POP7, RPP25, RPP30, RPP38, RPP40 and possibly also POP4 and POP5 (PubMed:16723659, PubMed:28115465).   
  
 **Gene Ontology Information:**

Molecular Function

- ribonuclease P activity
- ribonuclease P RNA binding
- RNA binding

Location

- extracellular space
- multimeric ribonuclease P complex
- nucleolar ribonuclease P complex
- nucleolus
- nucleoplasm
- ribonuclease MRP complex

Biological process

- RNA phosphodiester bond hydrolysis, endonucleolytic
- tRNA 5'-leader removal
- tRNA catabolic process
- tRNA processing

---

70

- **Protein name:** Myosin-IIIb
- **Organism:** Homo sapiens
- **Uniprot Accession Number:** Q8WXR4
- **Protein sequence length:** 1341 aa
- **1D identity (%):** 15.32
- **1D identity (%) [Gaps excluded]:** 22.92
- **1D identity - Alignment Gaps:** 520
- **1D aligned content (<aminoacid>:%):** {'G': 10.42, 'N': 7.08, 'P': 8.75, 'K': 4.17, 'D': 5.42, 'I': 5.0, 'E': 2.92, 'Q': 3.75, 'F': 5.0, 'Y': 5.42, 'W': 0.83, 'R': 4.58, 'L': 9.58, 'V': 6.67, 'S': 4.58, 'A': 8.75, 'T': 5.0, 'C': 1.67, 'H': 0.42}
- **Common reported functions (%):** 0.0
- **Common reported locations (%):** 0.0
- **Common reported processes (%):** 0.0

- **AF ID:** Q8WXR4
- **Chain:** A
- **Protein length:** 1341 aa
- **Resolution:** N/A
- **b-phipsi:** 0.02407
- **w-rdist:** 0.377979
- **t-alpha:** 0.105872
- **Chemical similarity (Tanimoto Index) (%):** 83.16
- **1D identity (%) [PDB]:** 3.48
- **1D identity (%) [Gaps excluded][PDB]:** 69.37
- **1D identity - Alignment Gaps [PDB]:** 2102
- **1D aligned content [PDB] (<aminoacid>:%):** {'I': 7.79, 'G': 7.79, 'K': 7.79, 'D': 3.9, 'S': 12.99, 'L': 11.69, 'T': 6.49, 'A': 9.09, 'Q': 9.09, 'N': 3.9, 'F': 2.6, 'V': 5.19, 'E': 5.19, 'R': 6.49}
- **2D identity (%) [PDB]:** 26.03
- **2D identity (%) [Gaps excluded][PDB]:** 83.36
- **2D identity - Alignment Gaps [PDB]:** 1218
- **2D aligned content [PDB] (<2D-fold>:%):** {'.': 22.78, 'E': 24.3, 'T': 13.23, 'H': 39.05, 'G': 0.65}
- **3D similarity (TM-Score) (%) [PDB]:** 21.58

- **Gene name:** MYO3B
- **Entrez ID:** 140469
- **RefSeq ID:** N/A
- **Sequence length:** N/A
- **5-UTR|CDS|3-UTR identity (%):** N/A | N/A | N/A
- **5-UTR|CDS|3-UTR identity (%) [Gaps excluded]:** N/A | N/A | N/A
- **5-UTR|CDS|3-UTR identity [Alignment Gaps]:** N/A | N/A | N/A
- **5-UTR aligned content (<base>:%):** N/A
- **CDS aligned content (<base>:%):** N/A
- **3-UTR aligned content (<base>:%):** N/A

**Uniprot Description:**  
  
 Probable actin-based motor with a protein kinase activity. Required for normal cochlear hair bundle development and hearing. Plays an important role in the early steps of cochlear hair bundle morphogenesis. Influences the number and lengths of stereocilia to be produced and limits the growth of microvilli within the forming auditory hair bundles thereby contributing to the architecture of the hair bundle, including its staircase pattern. Involved in the elongation of actin in stereocilia tips by transporting the actin regulatory factor ESPN to the plus ends of actin filaments.   
  
Interacts (via C-terminus) with ESPN. Interacts (via C-terminus) with ESPNL.   
  
 **Gene Ontology Information:**

Molecular Function

- actin binding
- ATP binding
- microfilament motor activity
- protein serine kinase activity
- protein serine/threonine kinase activity

Location

- cytoplasm
- filopodium tip
- myosin complex
- photoreceptor inner segment
- stereocilium tip

Biological process

- cochlea morphogenesis
- peptidyl-serine phosphorylation
- positive regulation of filopodium assembly
- regulation of actin filament length
- response to stimulus
- sensory perception of sound
- visual perception

---

71

- **Protein name:** E3 ubiquitin-protein ligase MYCBP2
- **Organism:** Homo sapiens
- **Uniprot Accession Number:** O75592
- **Protein sequence length:** 4678 aa
- **1D identity (%):** 9.31
- **1D identity (%) [Gaps excluded]:** 34.71
- **1D identity - Alignment Gaps:** 3433
- **1D aligned content (<aminoacid>:%):** {'M': 0.69, 'F': 5.26, 'L': 10.98, 'P': 6.64, 'S': 6.41, 'T': 6.64, 'R': 3.2, 'A': 5.26, 'N': 7.32, 'G': 10.98, 'V': 5.95, 'W': 1.37, 'I': 3.89, 'H': 2.06, 'K': 4.12, 'D': 4.58, 'Y': 2.29, 'Q': 3.2, 'C': 5.95, 'E': 3.2}
- **Common reported functions (%):** 0.0
- **Common reported locations (%):** 20.0
- **Common reported processes (%):** 0.0

- **AF ID:** O75592
- **Chain:** A
- **Protein length:** 1400 aa
- **Resolution:** N/A
- **b-phipsi:** 0.004386
- **w-rdist:** 0.629265
- **t-alpha:** 0.00332
- **Chemical similarity (Tanimoto Index) (%):** 86.13
- **1D identity (%) [PDB]:** 4.3
- **1D identity (%) [Gaps excluded][PDB]:** 64.43
- **1D identity - Alignment Gaps [PDB]:** 2085
- **1D aligned content [PDB] (<aminoacid>:%):** {'E': 4.17, 'Q': 8.33, 'L': 14.58, 'A': 10.42, 'S': 11.46, 'I': 5.21, 'G': 8.33, 'K': 5.21, 'D': 4.17, 'T': 6.25, 'V': 7.29, 'N': 3.12, 'F': 3.12, 'R': 4.17, 'Y': 1.04, 'H': 2.08, 'P': 1.04}
- **2D identity (%) [PDB]:** 33.12
- **2D identity (%) [Gaps excluded][PDB]:** 85.56
- **2D identity - Alignment Gaps [PDB]:** 1053
- **2D aligned content [PDB] (<2D-fold>:%):** {'.': 17.4, 'E': 41.12, 'T': 12.83, 'B': 0.18, 'G': 1.05, 'H': 27.42}
- **3D similarity (TM-Score) (%) [PDB]:** 22.64

- **Gene name:** MYCBP2
- **Entrez ID:** 368439
- **RefSeq ID:** NM\_015057
- **Transcript sequence length:** 15077
- **5-UTR|CDS|3-UTR identity (%):** 36.58 | 22.98 | 24.15
- **5-UTR|CDS|3-UTR identity (%) [Gaps excluded]:** 68.14 | 85.82 | 83.57
- **5-UTR|CDS|3-UTR identity [Alignment Gaps]:** 176 | 10315 | 524
- **5-UTR aligned content (<base>:%):** {'A': 15.83, 'C': 30.94, 'T': 20.14, 'G': 33.09}
- **CDS aligned content (<base>:%):** {'A': 29.94, 'T': 32.93, 'G': 18.63, 'C': 18.5}
- **3-UTR aligned content (<base>:%):** {'A': 37.08, 'T': 25.84, 'C': 14.61, 'G': 22.47}

**Uniprot Description:**  
  
 Atypical E3 ubiquitin-protein ligase which specifically mediates ubiquitination of threonine and serine residues on target proteins, instead of ubiquitinating lysine residues (PubMed:29643511). Shows esterification activity towards both threonine and serine, with a preference for threonine, and acts via two essential catalytic cysteine residues that relay ubiquitin to its substrate via thioester intermediates (PubMed:29643511). Interacts with the E2 enzymes UBE2D1, UBE2D3, UBE2E1 and UBE2L3 (PubMed:18308511, PubMed:29643511). Plays a key role in neural development, probably by mediating ubiquitination of threonine residues on target proteins (Probable). Involved in different processes such as regulation of neurite outgrowth, synaptic growth, synaptogenesis and axon degeneration (By similarity). Required for the formation of major central nervous system axon tracts (By similarity). Required for proper axon growth by regulating axon navigation and axon branching: acts by regulating the subcellular location and stability of MAP3K12/DLK (By similarity). Required for proper localization of retinogeniculate projections but not for eye-specific segregation (By similarity). Regulates axon guidance in the olfactory system (By similarity). Involved in Wallerian axon degeneration, an evolutionarily conserved process that drives the loss of damaged axons: acts by promoting destabilization of NMNAT2, probably via ubiquitination of NMNAT2 (By similarity). Catalyzes ubiquitination of threonine and/or serine residues on NMNAT2, consequences of threonine and/or serine ubiquitination are however unknown (PubMed:29643511). Regulates the internalization of TRPV1 in peripheral sensory neurons (By similarity). Mediates ubiquitination and subsequent proteasomal degradation of TSC2/tuberin (PubMed:18308511, PubMed:27278822). Independently of the E3 ubiquitin-protein ligase activity, also acts as a guanosine exchange factor (GEF) for RAN in neurons of dorsal root ganglia (PubMed:26304119). May function as a facilitator or regulator of transcriptional activation by MYC (PubMed:9689053). Acts in concert with HUWE1 to regulate the circadian clock gene expression by promoting the lithium-induced ubiquination and degradation of NR1D1 (PubMed:20534529).   
  
Interacts with MYC (PubMed:9689053). Interacts with TSC2 (tuberin) when TSC2 is in complex with TSC1 (hamartin) (PubMed:14559897). Interacts with FBXO45 (PubMed:19398581). Interacts with RAE1 (PubMed:22357847). Interacts with CPNE1 (via VWFA domain) and CPNE4 (via VWFA domain) (By similarity). Interacts with (sumoylated) RANGAP1; interaction with sumoylated RANGAP1 inhibits E3 ubiquitin-protein ligase activity and promotes MYCBP2 translocation to the nucleus (PubMed:26304119). Interacts with RAN (PubMed:26304119).Interacts with ATP13A2; the interaction inhibits the ubiquitination of TSC2 by MYCBP2 (PubMed:27278822). Interacts with USP11 (PubMed:29293652).   
  
 **Gene Ontology Information:**

Molecular Function

- guanyl-nucleotide exchange factor activity
- metal ion binding
- small GTPase binding
- ubiquitin protein ligase activity
- ubiquitin-protein transferase activity

Location

- axon
- cytoplasm
- cytoskeleton
- nucleus
- plasma membrane

Biological process

- axon choice point recognition
- axon extension involved in regeneration
- axon guidance
- central nervous system projection neuron axonogenesis
- circadian regulation of gene expression
- developmental pigmentation
- habenula development
- neuromuscular process
- regulation of axon guidance
- regulation of pteridine metabolic process
- regulation of synaptic assembly at neuromuscular junction
- retinal ganglion cell axon guidance

---

72

- **Protein name:** Protein phosphatase 1 regulatory inhibitor subunit 16B
- **Organism:** Homo sapiens
- **Uniprot Accession Number:** Q96T49
- **Protein sequence length:** 567 aa
- **1D identity (%):** 8.27
- **1D identity (%) [Gaps excluded]:** 23.05
- **1D identity - Alignment Gaps:** 868
- **1D aligned content (<aminoacid>:%):** {'D': 5.36, 'T': 10.71, 'L': 10.71, 'Y': 4.46, 'R': 6.25, 'S': 7.14, 'F': 2.68, 'E': 4.46, 'N': 6.25, 'V': 5.36, 'K': 2.68, 'P': 7.14, 'G': 10.71, 'A': 6.25, 'H': 1.79, 'W': 1.79, 'C': 1.79, 'I': 4.46}
- **Common reported functions (%):** 0.0
- **Common reported locations (%):** 20.0
- **Common reported processes (%):** 0.0

- **AF ID:** Q96T49
- **Chain:** A
- **Protein length:** 567 aa
- **Resolution:** N/A
- **b-phipsi:** 0.05171
- **w-rdist:** 0.307462
- **t-alpha:** 0.132022
- **Chemical similarity (Tanimoto Index) (%):** 83.76
- **1D identity (%) [PDB]:** 1.72
- **1D identity (%) [Gaps excluded][PDB]:** 70.27
- **1D identity - Alignment Gaps [PDB]:** 1476
- **1D aligned content [PDB] (<aminoacid>:%):** {'L': 15.38, 'Q': 15.38, 'R': 7.69, 'A': 19.23, 'E': 7.69, 'S': 7.69, 'N': 3.85, 'T': 3.85, 'K': 7.69, 'M': 3.85, 'V': 3.85, 'G': 3.85}
- **2D identity (%) [PDB]:** 16.17
- **2D identity (%) [Gaps excluded][PDB]:** 91.42
- **2D identity - Alignment Gaps [PDB]:** 1084
- **2D aligned content [PDB] (<2D-fold>:%):** {'.': 15.02, 'H': 77.93, 'T': 7.04}
- **3D similarity (TM-Score) (%) [PDB]:** 13.29

- **Gene name:** PPP1R16B
- **Entrez ID:** 282091
- **RefSeq ID:** NM\_015568
- **Transcript sequence length:** 6259
- **5-UTR|CDS|3-UTR identity (%):** 30.84 | 22.94 | 5.03
- **5-UTR|CDS|3-UTR identity (%) [Gaps excluded]:** 70.21 | 71.02 | 95.63
- **5-UTR|CDS|3-UTR identity [Alignment Gaps]:** 180 | 2828 | 4129
- **5-UTR aligned content (<base>:%):** {'A': 15.15, 'C': 36.36, 'T': 14.14, 'G': 34.34}
- **CDS aligned content (<base>:%):** {'A': 28.91, 'T': 22.44, 'G': 24.53, 'C': 24.11}
- **3-UTR aligned content (<base>:%):** {'C': 16.44, 'A': 40.64, 'T': 21.92, 'G': 21.0}

**Uniprot Description:**  
  
 Regulator of protein phosphatase 1 (PP1) that acts as a positive regulator of pulmonary endothelial cell (EC) barrier function (PubMed:18586956). Involved in the regulation of the PI3K/AKT signaling pathway, angiogenesis and endothelial cell proliferation (PubMed:25007873). Regulates angiogenesis and endothelial cell proliferation through the control of ECE1 dephosphorylation, trafficking and activity (By similarity). Protects the endothelial barrier from lipopolysaccharide (LPS)-induced vascular leakage (By similarity). Involved in the regulation of endothelial cell filopodia extension (By similarity). May be a downstream target for TGF-beta1 signaling cascade in endothelial cells (PubMed:16263087, PubMed:18586956). Involved in PKA-mediated moesin dephosphorylation which is important in EC barrier protection against thrombin stimulation (PubMed:18586956). Promotes the interaction of PPP1CA with RPSA/LAMR1 and in turn facilitates the dephosphorylation of RPSA/LAMR1 (PubMed:16263087). Involved in the dephosphorylation of EEF1A1 (PubMed:26497934).   
  
Interacts with PPP1CA, PPP1CB and MSN. Interacts (via its fourth ankyrin repeat) with the mature dimeric form of RPSA/LAMR1 (PubMed:16263087, PubMed:18586956). Interacts with EEF1A1 (PubMed:26497934). Interacts with PTEN (PubMed:25007873). Interacts with ECE1 (By similarity).   
  
 **Gene Ontology Information:**

Molecular Function

- myosin phosphatase regulator activity
- protein phosphatase 1 binding
- protein phosphatase regulator activity

Location

- cell projection
- nuclear speck
- nucleus
- perinuclear region of cytoplasm
- plasma membrane

Biological process

- establishment of endothelial barrier
- negative regulation of peptidyl-serine dephosphorylation
- positive regulation of blood vessel endothelial cell proliferation involved in sprouting angiogenesis
- positive regulation of protein dephosphorylation
- regulation of filopodium assembly
- regulation of phosphatidylinositol 3-kinase signaling
- regulation of protein dephosphorylation
- regulation of sprouting angiogenesis

---

73

- **Protein name:** Pikachurin
- **Organism:** Homo sapiens
- **Uniprot Accession Number:** Q63HQ2
- **Protein sequence length:** 1017 aa
- **1D identity (%):** 14.43
- **1D identity (%) [Gaps excluded]:** 24.5
- **1D identity - Alignment Gaps:** 592
- **1D aligned content (<aminoacid>:%):** {'I': 5.77, 'R': 3.85, 'G': 13.94, 'L': 9.13, 'D': 3.37, 'K': 4.81, 'N': 4.81, 'T': 6.25, 'P': 9.62, 'V': 7.21, 'Y': 3.85, 'S': 7.69, 'E': 2.4, 'A': 3.85, 'F': 4.81, 'C': 5.29, 'Q': 3.37}
- **Common reported functions (%):** 0.0
- **Common reported locations (%):** 0.0
- **Common reported processes (%):** 0.0

- **AF ID:** Q63HQ2
- **Chain:** A
- **Protein length:** 1017 aa
- **Resolution:** N/A
- **b-phipsi:** 0.03051
- **w-rdist:** 0.364101
- **t-alpha:** 0.177215
- **Chemical similarity (Tanimoto Index) (%):** 86.24
- **1D identity (%) [PDB]:** 2.74
- **1D identity (%) [Gaps excluded][PDB]:** 76.81
- **1D identity - Alignment Gaps [PDB]:** 1862
- **1D aligned content [PDB] (<aminoacid>:%):** {'Y': 1.89, 'R': 5.66, 'V': 11.32, 'L': 13.21, 'S': 3.77, 'F': 9.43, 'E': 1.89, 'P': 3.77, 'A': 3.77, 'T': 13.21, 'G': 9.43, 'K': 7.55, 'N': 5.66, 'Q': 3.77, 'D': 3.77, 'I': 1.89}
- **2D identity (%) [PDB]:** 35.13
- **2D identity (%) [Gaps excluded][PDB]:** 80.43
- **2D identity - Alignment Gaps [PDB]:** 784
- **2D aligned content [PDB] (<2D-fold>:%):** {'T': 15.95, '.': 25.15, 'E': 56.65, 'G': 0.82, 'H': 1.43}
- **3D similarity (TM-Score) (%) [PDB]:** 23.01

- **Gene name:** EGFLAM
- **Entrez ID:** 133584
- **RefSeq ID:** NM\_182801
- **Transcript sequence length:** 2217
- **5-UTR|CDS|3-UTR identity (%):** 42.94 | 7.05 | 13.51
- **5-UTR|CDS|3-UTR identity (%) [Gaps excluded]:** 72.96 | 75.0 | 88.6
- **5-UTR|CDS|3-UTR identity [Alignment Gaps]:** 137 | 3545 | 1267
- **5-UTR aligned content (<base>:%):** {'C': 27.97, 'T': 26.57, 'G': 20.98, 'A': 24.48}
- **CDS aligned content (<base>:%):** {'T': 30.43, 'G': 23.91, 'A': 26.81, 'C': 18.84}
- **3-UTR aligned content (<base>:%):** {'C': 15.35, 'A': 40.59, 'T': 23.76, 'G': 20.3}

**Uniprot Description:**  
  
 Involved in both the retinal photoreceptor ribbon synapse formation and physiological functions of visual perception. Necessary for proper bipolar dendritic tip apposition to the photoreceptor ribbon synapse. Promotes matrix assembly and cell adhesiveness (By similarity).   
  
Interacts with DAG1 alpha-dystroglycan.   
  
 **Gene Ontology Information:**

Molecular Function

- calcium ion binding
- glycosaminoglycan binding

Location

- basement membrane
- cell projection
- interstitial matrix
- presynaptic active zone
- synaptic cleft

Biological process

- animal organ morphogenesis
- extracellular matrix organization
- peptide cross-linking via chondroitin 4-sulfate glycosaminoglycan
- positive regulation of cell-substrate adhesion
- tissue development

---

74

- **Protein name:** Serine/threonine-protein kinase Nek10
- **Organism:** Homo sapiens
- **Uniprot Accession Number:** Q6ZWH5
- **Protein sequence length:** 1172 aa
- **1D identity (%):** 16.08
- **1D identity (%) [Gaps excluded]:** 23.15
- **1D identity - Alignment Gaps:** 441
- **1D aligned content (<aminoacid>:%):** {'Q': 2.59, 'T': 9.91, 'Y': 4.31, 'K': 3.88, 'R': 3.02, 'V': 6.9, 'S': 9.48, 'L': 15.09, 'F': 4.31, 'A': 3.88, 'I': 4.74, 'G': 7.33, 'N': 6.47, 'C': 2.16, 'D': 4.31, 'P': 4.74, 'H': 1.72, 'M': 0.86, 'E': 4.31}
- **Common reported functions (%):** 0.0
- **Common reported locations (%):** 0.0
- **Common reported processes (%):** 0.0

- **AF ID:** Q6ZWH5
- **Chain:** A
- **Protein length:** 1172 aa
- **Resolution:** N/A
- **b-phipsi:** 0.04358
- **w-rdist:** 0.324967
- **t-alpha:** 0.059553
- **Chemical similarity (Tanimoto Index) (%):** 84.13
- **1D identity (%) [PDB]:** 4.27
- **1D identity (%) [Gaps excluded][PDB]:** 60.14
- **1D identity - Alignment Gaps [PDB]:** 1869
- **1D aligned content [PDB] (<aminoacid>:%):** {'R': 2.33, 'A': 6.98, 'G': 5.81, 'L': 8.14, 'I': 16.28, 'E': 4.65, 'V': 5.81, 'N': 5.81, 'Y': 2.33, 'D': 2.33, 'H': 1.16, 'S': 13.95, 'T': 6.98, 'P': 6.98, 'K': 4.65, 'Q': 3.49, 'F': 2.33}
- **2D identity (%) [PDB]:** 18.37
- **2D identity (%) [Gaps excluded][PDB]:** 90.38
- **2D identity - Alignment Gaps [PDB]:** 1427
- **2D aligned content [PDB] (<2D-fold>:%):** {'.': 24.01, 'T': 12.16, 'E': 13.68, 'H': 48.94, 'B': 0.3, 'G': 0.91}
- **3D similarity (TM-Score) (%) [PDB]:** 21.42

- **Gene name:** NEK10
- **Entrez ID:** 152110
- **RefSeq ID:** N/A
- **Sequence length:** N/A
- **5-UTR|CDS|3-UTR identity (%):** N/A | N/A | N/A
- **5-UTR|CDS|3-UTR identity (%) [Gaps excluded]:** N/A | N/A | N/A
- **5-UTR|CDS|3-UTR identity [Alignment Gaps]:** N/A | N/A | N/A
- **5-UTR aligned content (<base>:%):** N/A
- **CDS aligned content (<base>:%):** N/A
- **3-UTR aligned content (<base>:%):** N/A

**Uniprot Description:**  
  
 Plays a role in the cellular response to UV irradiation. Mediates G2/M cell cycle arrest, MEK autoactivation and ERK1/2-signaling pathway activation in response to UV irradiation. In ciliated cells of airways, it is involved in the regulation of mucociliary transport (PubMed:31959991).   
  
Interacts with RAF1 and MAP2K1; the interaction is direct with RAF1 and required for ERK1/2-signaling pathway activation in response to UV irradiation.   
  
 **Gene Ontology Information:**

Molecular Function

- ATP binding
- metal ion binding
- protein kinase activity
- protein serine kinase activity
- protein serine/threonine kinase activity

Location

- extracellular region
- protein kinase complex

Biological process

- mucociliary clearance
- positive regulation of MAP kinase activity
- positive regulation of protein autophosphorylation
- protein phosphorylation
- regulation of cell cycle G2/M phase transition
- regulation of ERK1 and ERK2 cascade

---

75

- **Protein name:** Coatomer subunit beta'
- **Organism:** Homo sapiens
- **Uniprot Accession Number:** P35606
- **Protein sequence length:** 906 aa
- **1D identity (%):** 12.78
- **1D identity (%) [Gaps excluded]:** 23.72
- **1D identity - Alignment Gaps:** 653
- **1D aligned content (<aminoacid>:%):** {'I': 4.97, 'R': 4.42, 'S': 7.18, 'P': 4.42, 'T': 8.29, 'N': 7.18, 'V': 9.39, 'W': 1.1, 'A': 7.18, 'F': 2.76, 'K': 4.97, 'D': 7.18, 'G': 8.29, 'Y': 3.87, 'Q': 3.31, 'E': 5.52, 'C': 2.76, 'L': 6.63, 'H': 0.55}
- **Common reported functions (%):** 0.0
- **Common reported locations (%):** 0.0
- **Common reported processes (%):** 0.0

- **AF ID:** P35606
- **Chain:** A
- **Protein length:** 906 aa
- **Resolution:** N/A
- **b-phipsi:** 0.003583
- **w-rdist:** 0.651966
- **t-alpha:** 0.065198
- **Chemical similarity (Tanimoto Index) (%):** 83.78
- **1D identity (%) [PDB]:** 3.91
- **1D identity (%) [Gaps excluded][PDB]:** 71.43
- **1D identity - Alignment Gaps [PDB]:** 1693
- **1D aligned content [PDB] (<aminoacid>:%):** {'L': 17.14, 'Y': 2.86, 'E': 5.71, 'Q': 5.71, 'I': 5.71, 'A': 15.71, 'N': 8.57, 'F': 2.86, 'S': 12.86, 'T': 5.71, 'G': 2.86, 'K': 2.86, 'V': 4.29, 'D': 2.86, 'P': 1.43, 'R': 2.86}
- **2D identity (%) [PDB]:** 41.88
- **2D identity (%) [Gaps excluded][PDB]:** 88.01
- **2D identity - Alignment Gaps [PDB]:** 671
- **2D aligned content [PDB] (<2D-fold>:%):** {'.': 12.69, 'T': 11.75, 'E': 44.4, 'G': 1.12, 'H': 30.04}
- **3D similarity (TM-Score) (%) [PDB]:** 22.09

- **Gene name:** COPB2
- **Entrez ID:** 7807980
- **RefSeq ID:** N/A
- **Sequence length:** N/A
- **5-UTR|CDS|3-UTR identity (%):** N/A | N/A | N/A
- **5-UTR|CDS|3-UTR identity (%) [Gaps excluded]:** N/A | N/A | N/A
- **5-UTR|CDS|3-UTR identity [Alignment Gaps]:** N/A | N/A | N/A
- **5-UTR aligned content (<base>:%):** N/A
- **CDS aligned content (<base>:%):** N/A
- **3-UTR aligned content (<base>:%):** N/A

**Uniprot Description:**  
  
 The coatomer is a cytosolic protein complex that binds to dilysine motifs and reversibly associates with Golgi non-clathrin-coated vesicles, which further mediate biosynthetic protein transport from the ER, via the Golgi up to the trans Golgi network. Coatomer complex is required for budding from Golgi membranes, and is essential for the retrograde Golgi-to-ER transport of dilysine-tagged proteins. In mammals, the coatomer can only be recruited by membranes associated to ADP-ribosylation factors (ARFs), which are small GTP-binding proteins; the complex also influences the Golgi structural integrity, as well as the processing, activity, and endocytic recycling of LDL receptors.   
  
Oligomeric complex that consists of at least the alpha, beta, beta', gamma, delta, epsilon and zeta subunits. Probably interacts with PEX11A. Interacts with SCYL1 (By similarity). Interacts with JAGN1 (PubMed:25129144).   
  
 **Gene Ontology Information:**

Molecular Function

- ATP binding
- carbamoyl-phosphate synthase (glutamine-hydrolyzing) activity
- metal ion binding

Location   
  
N/A

Biological process

- 'de novo' UMP biosynthetic process
- arginine biosynthetic process

---

76

- **Protein name:** Heterogeneous nuclear ribonucleoprotein U-like protein 1
- **Organism:** Homo sapiens
- **Uniprot Accession Number:** Q9BUJ2
- **Protein sequence length:** 856 aa
- **1D identity (%):** 11.15
- **1D identity (%) [Gaps excluded]:** 21.37
- **1D identity - Alignment Gaps:** 669
- **1D aligned content (<aminoacid>:%):** {'K': 3.21, 'N': 8.97, 'R': 5.77, 'L': 3.85, 'D': 5.13, 'A': 1.92, 'E': 5.13, 'G': 12.82, 'H': 0.64, 'S': 6.41, 'P': 11.54, 'M': 0.64, 'Q': 6.41, 'F': 5.13, 'Y': 7.69, 'T': 6.41, 'C': 2.56, 'V': 3.85, 'W': 0.64, 'I': 1.28}
- **Common reported functions (%):** 0.0
- **Common reported locations (%):** 0.0
- **Common reported processes (%):** 0.0

- **AF ID:** Q9BUJ2
- **Chain:** A
- **Protein length:** 856 aa
- **Resolution:** N/A
- **b-phipsi:** 0.062184
- **w-rdist:** 0.256792
- **t-alpha:** 0.169562
- **Chemical similarity (Tanimoto Index) (%):** 83.6
- **1D identity (%) [PDB]:** 3.15
- **1D identity (%) [Gaps excluded][PDB]:** 60.44
- **1D identity - Alignment Gaps [PDB]:** 1657
- **1D aligned content [PDB] (<aminoacid>:%):** {'E': 5.45, 'L': 10.91, 'H': 1.82, 'P': 5.45, 'T': 10.91, 'G': 10.91, 'K': 7.27, 'S': 9.09, 'N': 9.09, 'V': 5.45, 'F': 9.09, 'R': 1.82, 'D': 9.09, 'I': 1.82, 'Q': 1.82}
- **2D identity (%) [PDB]:** 23.74
- **2D identity (%) [Gaps excluded][PDB]:** 82.48
- **2D identity - Alignment Gaps [PDB]:** 1017
- **2D aligned content [PDB] (<2D-fold>:%):** {'.': 24.48, 'T': 13.57, 'H': 34.81, 'E': 26.25, 'G': 0.88}
- **3D similarity (TM-Score) (%) [PDB]:** 16.44

- **Gene name:** HNRNPUL1
- **Entrez ID:** 11100
- **RefSeq ID:** NM\_144732
- **Transcript sequence length:** 3524
- **5-UTR|CDS|3-UTR identity (%):** 7.17 | 32.6 | 14.48
- **5-UTR|CDS|3-UTR identity (%) [Gaps excluded]:** 61.29 | 73.68 | 83.26
- **5-UTR|CDS|3-UTR identity [Alignment Gaps]:** 234 | 2355 | 1021
- **5-UTR aligned content (<base>:%):** {'G': 31.58, 'T': 21.05, 'C': 36.84, 'A': 10.53}
- **CDS aligned content (<base>:%):** {'T': 23.82, 'G': 22.22, 'A': 30.65, 'C': 23.31}
- **3-UTR aligned content (<base>:%):** {'C': 13.41, 'A': 39.66, 'G': 22.91, 'T': 24.02}

**Uniprot Description:**  
  
 Acts as a basic transcriptional regulator. Represses basic transcription driven by several virus and cellular promoters. When associated with BRD7, activates transcription of glucocorticoid-responsive promoter in the absence of ligand-stimulation. Also plays a role in mRNA processing and transport. Binds avidly to poly(G) and poly(C) RNA homopolymers in vitro.   
  
Interacts with the adenovirus type 5 (Ad5) E1B-55 kDa, BRD7, PRMT2, TP53 and NXF1. Associates with histones and BRD7.   
  
 **Gene Ontology Information:**

Molecular Function

- enzyme binding
- RNA binding

Location

- nucleoplasm
- nucleus
- ribonucleoprotein complex
- synapse

Biological process

- response to virus
- RNA processing

---

77

- **Protein name:** Ubiquitin carboxyl-terminal hydrolase 38
- **Organism:** Homo sapiens
- **Uniprot Accession Number:** Q8NB14
- **Protein sequence length:** 1042 aa
- **1D identity (%):** 14.79
- **1D identity (%) [Gaps excluded]:** 22.07
- **1D identity - Alignment Gaps:** 457
- **1D aligned content (<aminoacid>:%):** {'L': 16.59, 'V': 6.83, 'S': 8.78, 'D': 6.34, 'T': 6.83, 'P': 8.78, 'F': 10.24, 'H': 0.49, 'R': 1.95, 'G': 4.88, 'K': 2.93, 'N': 3.9, 'C': 3.41, 'E': 5.37, 'Q': 2.93, 'A': 5.37, 'Y': 1.95, 'I': 2.44}
- **Common reported functions (%):** 0.0
- **Common reported locations (%):** 0.0
- **Common reported processes (%):** 0.0

- **AF ID:** Q8NB14
- **Chain:** A
- **Protein length:** 1042 aa
- **Resolution:** N/A
- **b-phipsi:** 0.03639
- **w-rdist:** 0.342059
- **t-alpha:** 0.114971
- **Chemical similarity (Tanimoto Index) (%):** 83.22
- **1D identity (%) [PDB]:** 2.4
- **1D identity (%) [Gaps excluded][PDB]:** 72.31
- **1D identity - Alignment Gaps [PDB]:** 1895
- **1D aligned content [PDB] (<aminoacid>:%):** {'K': 6.38, 'I': 8.51, 'Q': 6.38, 'D': 8.51, 'S': 19.15, 'T': 4.26, 'A': 10.64, 'L': 14.89, 'V': 2.13, 'N': 6.38, 'F': 2.13, 'R': 4.26, 'P': 4.26, 'G': 2.13}
- **2D identity (%) [PDB]:** 18.01
- **2D identity (%) [Gaps excluded][PDB]:** 86.78
- **2D identity - Alignment Gaps [PDB]:** 1329
- **2D aligned content [PDB] (<2D-fold>:%):** {'.': 14.57, 'H': 55.63, 'T': 11.26, 'G': 1.66, 'E': 16.89}
- **3D similarity (TM-Score) (%) [PDB]:** 21.63

- **Gene name:** USP38
- **Entrez ID:** 84640
- **RefSeq ID:** NM\_032557
- **Transcript sequence length:** 7081
- **5-UTR|CDS|3-UTR identity (%):** 32.73 | 41.53 | 6.29
- **5-UTR|CDS|3-UTR identity (%) [Gaps excluded]:** 72.29 | 75.11 | 94.71
- **5-UTR|CDS|3-UTR identity [Alignment Gaps]:** 301 | 2001 | 3193
- **5-UTR aligned content (<base>:%):** {'A': 18.89, 'T': 25.56, 'G': 27.22, 'C': 28.33}
- **CDS aligned content (<base>:%):** {'A': 29.75, 'T': 32.71, 'G': 19.31, 'C': 18.24}
- **3-UTR aligned content (<base>:%):** {'C': 15.81, 'A': 41.4, 'T': 24.65, 'G': 18.14}

**Uniprot Description:**  
  
 Deubiquitinating enzyme exhibiting a preference towards 'Lys-63'-linked ubiquitin chains. N/A   
  
 **Gene Ontology Information:**

Molecular Function

- thiol-dependent ubiquitin-specific protease activity
- cysteine-type endopeptidase activity

Location

- cytosol
- nucleus

Biological process

- protein deubiquitination
- ubiquitin-dependent protein catabolic process

---

78

- **Protein name:** Ubiquitin carboxyl-terminal hydrolase BAP1
- **Organism:** Homo sapiens
- **Uniprot Accession Number:** Q92560
- **Protein sequence length:** 729 aa
- **1D identity (%):** 10.92
- **1D identity (%) [Gaps excluded]:** 21.95
- **1D identity - Alignment Gaps:** 672
- **1D aligned content (<aminoacid>:%):** {'L': 10.96, 'S': 8.9, 'G': 10.96, 'F': 4.11, 'V': 6.85, 'C': 2.05, 'W': 0.68, 'R': 2.74, 'D': 4.79, 'P': 8.9, 'T': 3.42, 'I': 4.11, 'N': 6.16, 'Q': 4.79, 'E': 2.05, 'Y': 3.42, 'H': 2.05, 'K': 4.11, 'A': 8.9}
- **Common reported functions (%):** 0.0
- **Common reported locations (%):** 0.0
- **Common reported processes (%):** 0.0

- **AF ID:** Q92560
- **Chain:** A
- **Protein length:** 729 aa
- **Resolution:** N/A
- **b-phipsi:** 0.023702
- **w-rdist:** 0.382087
- **t-alpha:** 0.129032
- **Chemical similarity (Tanimoto Index) (%):** 83.81
- **1D identity (%) [PDB]:** 3.05
- **1D identity (%) [Gaps excluded][PDB]:** 68.49
- **1D identity - Alignment Gaps [PDB]:** 1566
- **1D aligned content [PDB] (<aminoacid>:%):** {'N': 6.0, 'T': 8.0, 'L': 20.0, 'V': 6.0, 'Q': 8.0, 'A': 10.0, 'S': 12.0, 'R': 10.0, 'P': 2.0, 'E': 2.0, 'I': 6.0, 'G': 4.0, 'K': 6.0}
- **2D identity (%) [PDB]:** 16.53
- **2D identity (%) [Gaps excluded][PDB]:** 87.5
- **2D identity - Alignment Gaps [PDB]:** 1168
- **2D aligned content [PDB] (<2D-fold>:%):** {'.': 15.55, 'T': 9.24, 'E': 6.72, 'H': 68.49}
- **3D similarity (TM-Score) (%) [PDB]:** 15.35

- **Gene name:** BAP1
- **Entrez ID:** 8314
- **RefSeq ID:** NM\_004656
- **Transcript sequence length:** 3600
- **5-UTR|CDS|3-UTR identity (%):** 21.74 | 29.69 | 14.73
- **5-UTR|CDS|3-UTR identity (%) [Gaps excluded]:** 67.71 | 72.2 | 83.63
- **5-UTR|CDS|3-UTR identity [Alignment Gaps]:** 203 | 2508 | 1057
- **5-UTR aligned content (<base>:%):** {'G': 36.92, 'T': 18.46, 'A': 13.85, 'C': 30.77}
- **CDS aligned content (<base>:%):** {'A': 29.33, 'T': 23.64, 'G': 23.4, 'C': 23.64}
- **3-UTR aligned content (<base>:%):** {'C': 17.46, 'A': 38.1, 'T': 21.16, 'G': 23.28}

**Uniprot Description:**  
  
 Deubiquitinating enzyme that plays a key role in chromatin by mediating deubiquitination of histone H2A and HCFC1 (PubMed:12485996, PubMed:18757409, PubMed:20436459, PubMed:25451922, PubMed:35051358). Catalytic component of the PR-DUB complex, a complex that specifically mediates deubiquitination of histone H2A monoubiquitinated at 'Lys-119' (H2AK119ub1) (PubMed:20436459, PubMed:25451922, PubMed:35051358). Does not deubiquitinate monoubiquitinated histone H2B (PubMed:20436459). Acts as a regulator of cell growth by mediating deubiquitination of HCFC1 N-terminal and C-terminal chains, with some specificity toward 'Lys-48'-linked polyubiquitin chains compared to 'Lys-63'-linked polyubiquitin chains (PubMed:19188440, PubMed:19815555). Deubiquitination of HCFC1 does not lead to increase stability of HCFC1 (PubMed:19188440, PubMed:19815555). Interferes with the BRCA1 and BARD1 heterodimer activity by inhibiting their ability to mediate ubiquitination and autoubiquitination (PubMed:19117993). It however does not mediate deubiquitination of BRCA1 and BARD1 (PubMed:19117993). Able to mediate autodeubiquitination via intramolecular interactions to couteract monoubiquitination at the nuclear localization signal (NLS), thereby protecting it from cytoplasmic sequestration (PubMed:24703950). Acts as a tumor suppressor (PubMed:9528852).   
  
Component of the PR-DUB complex, at least composed of BAP1 and ASXL1 (PubMed:20436459). Interacts with BRCA1 (via the RING finger) (PubMed:19117993, PubMed:9528852). Interacts (via HBM-like motif) with HCFC1 (PubMed:19188440, PubMed:19815555). Interacts (when phosphorylated at Thr-493) with FOXK1 (PubMed:25451922). Interacts (when phosphorylated at Thr-493) with FOXK2; leading to recruit the PR-DUB complex and repress FOXK2 target genes (PubMed:24748658, PubMed:25451922).   
  
 **Gene Ontology Information:**

Molecular Function

- chromatin binding
- chromatin DNA binding
- thiol-dependent ubiquitin-specific protease activity
- peptidase activity

Location

- cytoplasm
- cytosol
- nucleoplasm
- nucleus
- PR-DUB complex

Biological process

- chromatin organization
- common myeloid progenitor cell proliferation
- erythrocyte maturation
- gene expression
- hematopoietic stem cell homeostasis
- in utero embryonic development
- leukocyte proliferation
- macrophage homeostasis
- mitotic cell cycle
- monoubiquitinated histone H2A deubiquitination
- monoubiquitinated protein deubiquitination
- myeloid cell apoptotic process
- negative regulation of cell population proliferation
- negative regulation of transcription, DNA-templated
- neuron cellular homeostasis
- neutrophil differentiation
- nucleate erythrocyte differentiation
- platelet morphogenesis
- positive regulation of protein targeting to mitochondrion
- protein deubiquitination
- protein K48-linked deubiquitination
- protein modification process
- regulation of cell cycle
- regulation of cell growth
- regulation of cytokine production involved in inflammatory response
- regulation of inflammatory response
- response to inorganic substance
- thrombocyte differentiation
- tissue homeostasis
- ubiquitin-dependent protein catabolic process

---

79

- **Protein name:** Hairy/enhancer-of-split related with YRPW motif protein 2
- **Organism:** Homo sapiens
- **Uniprot Accession Number:** Q9UBP5
- **Protein sequence length:** 337 aa
- **1D identity (%):** 4.77
- **1D identity (%) [Gaps excluded]:** 20.06
- **1D identity - Alignment Gaps:** 992
- **1D aligned content (<aminoacid>:%):** {'D': 1.61, 'I': 4.84, 'G': 9.68, 'N': 6.45, 'Y': 3.23, 'T': 8.06, 'V': 8.06, 'S': 14.52, 'R': 3.23, 'L': 4.84, 'E': 3.23, 'Q': 1.61, 'F': 1.61, 'C': 3.23, 'P': 14.52, 'A': 11.29}
- **Common reported functions (%):** 0.0
- **Common reported locations (%):** 0.0
- **Common reported processes (%):** 0.0

- **AF ID:** Q9UBP5
- **Chain:** A
- **Protein length:** 337 aa
- **Resolution:** N/A
- **b-phipsi:** 0.065147
- **w-rdist:** 0.236409
- **t-alpha:** 0.174927
- **Chemical similarity (Tanimoto Index) (%):** 83.35
- **1D identity (%) [PDB]:** 1.63
- **1D identity (%) [Gaps excluded][PDB]:** 72.41
- **1D identity - Alignment Gaps [PDB]:** 1262
- **1D aligned content [PDB] (<aminoacid>:%):** {'M': 4.76, 'S': 19.05, 'E': 4.76, 'G': 14.29, 'Q': 4.76, 'K': 9.52, 'R': 4.76, 'V': 4.76, 'D': 4.76, 'Y': 4.76, 'H': 4.76, 'L': 4.76, 'F': 4.76, 'P': 4.76, 'A': 4.76}
- **2D identity (%) [PDB]:** 12.41
- **2D identity (%) [Gaps excluded][PDB]:** 90.0
- **2D identity - Alignment Gaps [PDB]:** 1000
- **2D aligned content [PDB] (<2D-fold>:%):** {'.': 13.19, 'T': 6.94, 'H': 79.86}
- **3D similarity (TM-Score) (%) [PDB]:** 10.19

- **Gene name:** HEY2
- **Entrez ID:** 493293
- **RefSeq ID:** NM\_012259
- **Transcript sequence length:** 2626
- **5-UTR|CDS|3-UTR identity (%):** 31.77 | 15.07 | 13.73
- **5-UTR|CDS|3-UTR identity (%) [Gaps excluded]:** 66.17 | 73.6 | 89.78
- **5-UTR|CDS|3-UTR identity [Alignment Gaps]:** 144 | 3192 | 1246
- **5-UTR aligned content (<base>:%):** {'G': 37.5, 'A': 9.09, 'C': 38.64, 'T': 14.77}
- **CDS aligned content (<base>:%):** {'A': 29.59, 'T': 23.31, 'G': 21.16, 'C': 25.95}
- **3-UTR aligned content (<base>:%):** {'A': 42.57, 'T': 25.74, 'C': 13.37, 'G': 18.32}

**Uniprot Description:**  
  
 Downstream effector of Notch signaling which may be required for cardiovascular development. Transcriptional repressor which binds preferentially to the canonical E box sequence 5'-CACGTG-3'. Represses transcription by the cardiac transcriptional activators GATA4 and GATA6.   
  
May self-associate (By similarity). Interacts with GATA4, HES1 and HEYL (By similarity). Interacts with HDAC1, NCOR1 and SIN3A (By similarity). Interacts with ARNT and GATA6.   
  
 **Gene Ontology Information:**

Molecular Function

- protein heterodimerization activity
- protein homodimerization activity
- sequence-specific DNA binding

Location

- nucleus

Biological process

- floor plate development
- glomus development
- negative regulation of transcription, DNA-templated
- negative regulation of transcription by RNA polymerase II
- Notch signaling pathway
- pronephros development
- specification of proximal tubule identity

---

80

- **Protein name:** Paraneoplastic antigen-like protein 8A
- **Organism:** Homo sapiens
- **Uniprot Accession Number:** Q86V59
- **Protein sequence length:** 439 aa
- **1D identity (%):** 7.42
- **1D identity (%) [Gaps excluded]:** 23.95
- **1D identity - Alignment Gaps:** 902
- **1D aligned content (<aminoacid>:%):** {'M': 2.06, 'K': 9.28, 'T': 4.12, 'C': 3.09, 'G': 10.31, 'L': 10.31, 'Q': 3.09, 'F': 3.09, 'I': 5.15, 'N': 6.19, 'S': 6.19, 'P': 10.31, 'R': 3.09, 'D': 5.15, 'A': 10.31, 'E': 4.12, 'V': 3.09, 'W': 1.03}
- **Common reported functions (%):** 0.0
- **Common reported locations (%):** 0.0
- **Common reported processes (%):** 0.0

- **AF ID:** Q86V59
- **Chain:** A
- **Protein length:** 439 aa
- **Resolution:** N/A
- **b-phipsi:** 0.054677
- **w-rdist:** 0.309053
- **t-alpha:** 0.027295
- **Chemical similarity (Tanimoto Index) (%):** 83.72
- **1D identity (%) [PDB]:** 2.4
- **1D identity (%) [Gaps excluded][PDB]:** 70.21
- **1D identity - Alignment Gaps [PDB]:** 1328
- **1D aligned content [PDB] (<aminoacid>:%):** {'S': 9.09, 'V': 9.09, 'L': 15.15, 'N': 6.06, 'I': 9.09, 'R': 9.09, 'P': 6.06, 'E': 6.06, 'A': 18.18, 'Q': 3.03, 'D': 3.03, 'T': 3.03, 'K': 3.03}
- **2D identity (%) [PDB]:** 8.25
- **2D identity (%) [Gaps excluded][PDB]:** 95.58
- **2D identity - Alignment Gaps [PDB]:** 1196
- **2D aligned content [PDB] (<2D-fold>:%):** {'.': 5.56, 'H': 86.11, 'G': 2.78, 'T': 5.56}
- **3D similarity (TM-Score) (%) [PDB]:** 10.65

- **Gene name:** PNMA8A
- **Entrez ID:** 532062
- **RefSeq ID:** NM\_018215
- **Transcript sequence length:** 3684
- **5-UTR|CDS|3-UTR identity (%):** 37.57 | 20.57 | 10.53
- **5-UTR|CDS|3-UTR identity (%) [Gaps excluded]:** 73.58 | 73.29 | 95.59
- **5-UTR|CDS|3-UTR identity [Alignment Gaps]:** 185 | 2888 | 1833
- **5-UTR aligned content (<base>:%):** {'T': 26.76, 'C': 29.58, 'A': 18.31, 'G': 25.35}
- **CDS aligned content (<base>:%):** {'A': 30.99, 'T': 20.58, 'G': 26.15, 'C': 22.28}
- **3-UTR aligned content (<base>:%):** {'C': 15.67, 'A': 41.01, 'T': 22.58, 'G': 20.74}

**Uniprot Description:**  
  
 N/A N/A   
  
 **Gene Ontology Information:**

Molecular Function   
  
N/A

Location   
  
N/A

Biological process   
  
N/A

---

81

- **Protein name:** C-type mannose receptor 2
- **Organism:** Homo sapiens
- **Uniprot Accession Number:** Q9UBG0
- **Protein sequence length:** 1479 aa
- **1D identity (%):** 14.35
- **1D identity (%) [Gaps excluded]:** 25.87
- **1D identity - Alignment Gaps:** 788
- **1D aligned content (<aminoacid>:%):** {'M': 0.39, 'V': 2.76, 'F': 6.3, 'L': 9.45, 'Q': 5.51, 'T': 7.48, 'P': 7.09, 'G': 11.42, 'Y': 4.33, 'D': 6.3, 'R': 4.33, 'S': 7.48, 'W': 2.76, 'H': 0.79, 'N': 5.12, 'I': 1.97, 'A': 6.3, 'C': 5.12, 'E': 3.94, 'K': 1.18}
- **Common reported functions (%):** 0.0
- **Common reported locations (%):** 0.0
- **Common reported processes (%):** 0.0

- **AF ID:** Q9UBG0
- **Chain:** A
- **Protein length:** 1479 aa
- **Resolution:** N/A
- **b-phipsi:** 0.008886
- **w-rdist:** 0.638252
- **t-alpha:** 0.004136
- **Chemical similarity (Tanimoto Index) (%):** 86.07
- **1D identity (%) [PDB]:** 2.97
- **1D identity (%) [Gaps excluded][PDB]:** 66.04
- **1D identity - Alignment Gaps [PDB]:** 2250
- **1D aligned content [PDB] (<aminoacid>:%):** {'A': 11.43, 'Q': 8.57, 'L': 15.71, 'N': 2.86, 'T': 7.14, 'S': 11.43, 'G': 5.71, 'D': 4.29, 'P': 5.71, 'E': 5.71, 'V': 7.14, 'I': 2.86, 'R': 2.86, 'M': 2.86, 'Y': 2.86, 'H': 2.86}
- **2D identity (%) [PDB]:** 28.3
- **2D identity (%) [Gaps excluded][PDB]:** 83.9
- **2D identity - Alignment Gaps [PDB]:** 1220
- **2D aligned content [PDB] (<2D-fold>:%):** {'.': 25.53, 'E': 42.23, 'T': 11.9, 'H': 19.77, 'G': 0.58}
- **3D similarity (TM-Score) (%) [PDB]:** 23.42

- **Gene name:** MRC2
- **Entrez ID:** 9902
- **RefSeq ID:** NM\_006039
- **Transcript sequence length:** 5719
- **5-UTR|CDS|3-UTR identity (%):** 21.54 | 41.71 | 16.85
- **5-UTR|CDS|3-UTR identity (%) [Gaps excluded]:** 77.01 | 71.46 | 86.61
- **5-UTR|CDS|3-UTR identity [Alignment Gaps]:** 224 | 2172 | 927
- **5-UTR aligned content (<base>:%):** {'A': 13.43, 'T': 19.4, 'C': 32.84, 'G': 34.33}
- **CDS aligned content (<base>:%):** {'A': 25.51, 'T': 24.59, 'G': 23.94, 'C': 25.97}
- **3-UTR aligned content (<base>:%):** {'C': 17.53, 'A': 38.14, 'T': 22.16, 'G': 22.16}

**Uniprot Description:**  
  
 May play a role as endocytotic lectin receptor displaying calcium-dependent lectin activity. Internalizes glycosylated ligands from the extracellular space for release in an endosomal compartment via clathrin-mediated endocytosis. May be involved in plasminogen activation system controlling the extracellular level of PLAUR/PLAU, and thus may regulate protease activity at the cell surface. May contribute to cellular uptake, remodeling and degradation of extracellular collagen matrices. May play a role during cancer progression as well as in other chronic tissue destructive diseases acting on collagen turnover. May participate in remodeling of extracellular matrix cooperating with the matrix metalloproteinases (MMPs).   
  
Interacts with C-terminal region of type I collagen/COL1A1 (By similarity). Interacts directly with PLAUR/UPAR and PLAU/pro-UPA to form a tri-molecular complex. Interacts with collagen V.   
  
 **Gene Ontology Information:**

Molecular Function

- carbohydrate binding
- collagen binding
- signaling receptor activity

Location

- focal adhesion
- membrane

Biological process

- collagen catabolic process
- endocytosis
- osteoblast differentiation

---

82

- **Protein name:** Docking protein 1
- **Organism:** Homo sapiens
- **Uniprot Accession Number:** Q99704
- **Protein sequence length:** 481 aa
- **1D identity (%):** N/A
- **1D identity (%) [Gaps excluded]:** N/A
- **1D identity - Alignment Gaps:** N/A
- **1D aligned content (<aminoacid>:%):** N/A
- **Common reported functions (%):** N/A
- **Common reported locations (%):** N/A
- **Common reported processes (%):** N/A

- **AF ID:** Q99704
- **Chain:** A
- **Protein length:** 481 aa
- **Resolution:** N/A
- **b-phipsi:** 0.026886
- **w-rdist:** 0.380212
- **t-alpha:** 0.186457
- **Chemical similarity (Tanimoto Index) (%):** N/A
- **1D identity (%) [PDB]:** N/A
- **1D identity (%) [Gaps excluded][PDB]:** N/A
- **1D identity - Alignment Gaps [PDB]:** N/A
- **1D aligned content [PDB] (<aminoacid>:%):** N/A
- **2D identity (%) [PDB]:** N/A
- **2D identity (%) [Gaps excluded][PDB]:** N/A
- **2D identity - Alignment Gaps [PDB]:** N/A
- **2D aligned content [PDB] (<2D-fold>:%):** N/A
- **3D similarity (TM-Score) (%) [PDB]:** N/A

- **Gene name:** DOK1
- **Entrez ID:** N/A
- **RefSeq ID:** N/A
- **Sequence length:** N/A
- **5-UTR|CDS|3-UTR identity (%):** N/A | N/A | N/A
- **5-UTR|CDS|3-UTR identity (%) [Gaps excluded]:** N/A | N/A | N/A
- **5-UTR|CDS|3-UTR identity [Alignment Gaps]:** N/A | N/A | N/A
- **5-UTR aligned content (<base>:%):** N/A
- **CDS aligned content (<base>:%):** N/A
- **3-UTR aligned content (<base>:%):** N/A

**Uniprot Description:**  
  
 DOK proteins are enzymatically inert adaptor or scaffolding proteins. They provide a docking platform for the assembly of multimolecular signaling complexes. DOK1 appears to be a negative regulator of the insulin signaling pathway. Modulates integrin activation by competing with talin for the same binding site on ITGB3.   
  
Interacts with ABL1 (By similarity). Interacts with RasGAP and INPP5D/SHIP1. Interacts directly with phosphorylated ITGB3. Interacts with SRMS (via the SH2 and SH3 domains).   
  
 **Gene Ontology Information:**

Molecular Function

- chorismate synthase activity

Location   
  
N/A

Biological process

- cellular amino acid biosynthetic process
- aromatic amino acid family biosynthetic process
- chorismate biosynthetic process

---

83

- **Protein name:** Tolloid-like protein 1
- **Organism:** Homo sapiens
- **Uniprot Accession Number:** O43897
- **Protein sequence length:** 1013 aa
- **1D identity (%):** 14.49
- **1D identity (%) [Gaps excluded]:** 24.76
- **1D identity - Alignment Gaps:** 598
- **1D aligned content (<aminoacid>:%):** {'G': 11.48, 'L': 7.18, 'S': 5.74, 'W': 2.39, 'Y': 3.35, 'T': 8.61, 'A': 3.83, 'V': 7.18, 'D': 5.26, 'F': 7.18, 'I': 6.22, 'N': 4.78, 'P': 6.22, 'K': 3.83, 'R': 3.83, 'Q': 3.35, 'E': 5.26, 'C': 3.83, 'H': 0.48}
- **Common reported functions (%):** 0.0
- **Common reported locations (%):** 0.0
- **Common reported processes (%):** 0.0

- **AF ID:** O43897
- **Chain:** A
- **Protein length:** 1013 aa
- **Resolution:** N/A
- **b-phipsi:** 0.027191
- **w-rdist:** 0.379875
- **t-alpha:** 0.086253
- **Chemical similarity (Tanimoto Index) (%):** 86.24
- **1D identity (%) [PDB]:** 2.12
- **1D identity (%) [Gaps excluded][PDB]:** 69.49
- **1D identity - Alignment Gaps [PDB]:** 1878
- **1D aligned content [PDB] (<aminoacid>:%):** {'G': 12.2, 'A': 14.63, 'Q': 7.32, 'P': 2.44, 'F': 7.32, 'M': 2.44, 'Y': 4.88, 'R': 2.44, 'I': 7.32, 'V': 2.44, 'T': 4.88, 'L': 9.76, 'E': 2.44, 'N': 7.32, 'S': 4.88, 'K': 4.88, 'D': 2.44}
- **2D identity (%) [PDB]:** 25.29
- **2D identity (%) [Gaps excluded][PDB]:** 84.93
- **2D identity - Alignment Gaps [PDB]:** 1080
- **2D aligned content [PDB] (<2D-fold>:%):** {'.': 23.39, 'E': 64.78, 'T': 10.54, 'G': 1.29}
- **3D similarity (TM-Score) (%) [PDB]:** 21.7

- **Gene name:** TLL1
- **Entrez ID:** 7092
- **RefSeq ID:** NM\_012464
- **Transcript sequence length:** 7291
- **5-UTR|CDS|3-UTR identity (%):** 26.32 | 42.13 | 5.97
- **5-UTR|CDS|3-UTR identity (%) [Gaps excluded]:** 78.63 | 75.48 | 95.11
- **5-UTR|CDS|3-UTR identity [Alignment Gaps]:** 465 | 1946 | 3360
- **5-UTR aligned content (<base>:%):** {'A': 17.39, 'C': 29.89, 'T': 28.26, 'G': 24.46}
- **CDS aligned content (<base>:%):** {'A': 32.27, 'T': 30.39, 'G': 20.47, 'C': 16.86}
- **3-UTR aligned content (<base>:%):** {'C': 15.89, 'A': 41.12, 'T': 23.83, 'G': 19.16}

**Uniprot Description:**  
  
 Protease which processes procollagen C-propeptides, such as chordin, pro-biglycan and pro-lysyl oxidase. Required for the embryonic development. Predominant protease, which in the development, influences dorsal-ventral patterning and skeletogenesis. N/A   
  
 **Gene Ontology Information:**

Molecular Function

- calcium ion binding
- metalloendopeptidase activity
- serine-type endopeptidase activity
- zinc ion binding

Location

- extracellular region
- extracellular space

Biological process

- cell differentiation
- collagen fibril organization
- dorsal/ventral pattern formation
- protein processing
- skeletal system development

---

84

- **Protein name:** Zinc finger protein 567
- **Organism:** Homo sapiens
- **Uniprot Accession Number:** Q8N184
- **Protein sequence length:** 647 aa
- **1D identity (%):** 9.71
- **1D identity (%) [Gaps excluded]:** 24.18
- **1D identity - Alignment Gaps:** 820
- **1D aligned content (<aminoacid>:%):** {'Q': 3.01, 'G': 10.53, 'F': 6.77, 'D': 1.5, 'H': 2.26, 'L': 9.02, 'E': 5.26, 'T': 11.28, 'R': 4.51, 'P': 4.51, 'S': 9.02, 'K': 9.77, 'V': 3.01, 'N': 6.77, 'A': 0.75, 'Y': 3.76, 'I': 2.26, 'C': 6.02}
- **Common reported functions (%):** 0.0
- **Common reported locations (%):** 0.0
- **Common reported processes (%):** 0.0

- **AF ID:** Q8N184
- **Chain:** A
- **Protein length:** 647 aa
- **Resolution:** N/A
- **b-phipsi:** 0.031319
- **w-rdist:** 0.369421
- **t-alpha:** 0.034739
- **Chemical similarity (Tanimoto Index) (%):** 83.94
- **1D identity (%) [PDB]:** 1.5
- **1D identity (%) [Gaps excluded][PDB]:** 77.42
- **1D identity - Alignment Gaps [PDB]:** 1568
- **1D aligned content [PDB] (<aminoacid>:%):** {'N': 12.5, 'V': 16.67, 'L': 8.33, 'Y': 8.33, 'D': 4.17, 'G': 12.5, 'T': 8.33, 'E': 8.33, 'A': 8.33, 'I': 4.17, 'H': 4.17, 'S': 4.17}
- **2D identity (%) [PDB]:** 25.84
- **2D identity (%) [Gaps excluded][PDB]:** 86.17
- **2D identity - Alignment Gaps [PDB]:** 878
- **2D aligned content [PDB] (<2D-fold>:%):** {'.': 30.56, 'H': 56.48, 'T': 11.73, 'E': 1.23}
- **3D similarity (TM-Score) (%) [PDB]:** 16.82

- **Gene name:** ZNF567
- **Entrez ID:** 163081
- **RefSeq ID:** N/A
- **Sequence length:** N/A
- **5-UTR|CDS|3-UTR identity (%):** N/A | N/A | N/A
- **5-UTR|CDS|3-UTR identity (%) [Gaps excluded]:** N/A | N/A | N/A
- **5-UTR|CDS|3-UTR identity [Alignment Gaps]:** N/A | N/A | N/A
- **5-UTR aligned content (<base>:%):** N/A
- **CDS aligned content (<base>:%):** N/A
- **3-UTR aligned content (<base>:%):** N/A

**Uniprot Description:**  
  
 May be involved in transcriptional regulation. N/A   
  
 **Gene Ontology Information:**

Molecular Function

- DNA-binding transcription factor activity
- metal ion binding
- RNA polymerase II cis-regulatory region sequence-specific DNA binding

Location

- nucleus

Biological process

- regulation of transcription by RNA polymerase II

---

85

- **Protein name:** RAS protein activator like-3
- **Organism:** Homo sapiens
- **Uniprot Accession Number:** Q86YV0
- **Protein sequence length:** 1011 aa
- **1D identity (%):** 12.44
- **1D identity (%) [Gaps excluded]:** 21.51
- **1D identity - Alignment Gaps:** 610
- **1D aligned content (<aminoacid>:%):** {'M': 1.67, 'S': 8.89, 'T': 5.56, 'G': 10.56, 'K': 1.67, 'F': 3.89, 'P': 10.56, 'V': 7.22, 'R': 7.78, 'A': 6.11, 'L': 14.44, 'E': 5.56, 'N': 2.22, 'C': 2.78, 'D': 1.67, 'Q': 5.56, 'W': 1.11, 'I': 1.67, 'H': 0.56, 'Y': 0.56}
- **Common reported functions (%):** 50.0
- **Common reported locations (%):** 0.0
- **Common reported processes (%):** 0.0

- **AF ID:** Q86YV0
- **Chain:** A
- **Protein length:** 1011 aa
- **Resolution:** N/A
- **b-phipsi:** 0.021839
- **w-rdist:** 0.479493
- **t-alpha:** 0.006661
- **Chemical similarity (Tanimoto Index) (%):** 84.23
- **1D identity (%) [PDB]:** 2.38
- **1D identity (%) [Gaps excluded][PDB]:** 74.19
- **1D identity - Alignment Gaps [PDB]:** 1870
- **1D aligned content [PDB] (<aminoacid>:%):** {'D': 4.35, 'S': 19.57, 'T': 6.52, 'A': 10.87, 'L': 17.39, 'G': 6.52, 'Q': 13.04, 'V': 8.7, 'F': 2.17, 'I': 4.35, 'R': 6.52}
- **2D identity (%) [PDB]:** 24.66
- **2D identity (%) [Gaps excluded][PDB]:** 84.86
- **2D identity - Alignment Gaps [PDB]:** 1096
- **2D aligned content [PDB] (<2D-fold>:%):** {'G': 4.2, 'T': 11.02, '.': 18.11, 'E': 23.36, 'H': 43.31}
- **3D similarity (TM-Score) (%) [PDB]:** 18.69

- **Gene name:** RASAL3
- **Entrez ID:** 540027
- **RefSeq ID:** NM\_022904
- **Transcript sequence length:** 3266
- **5-UTR|CDS|3-UTR identity (%):** 13.06 | 36.65 | 32.52
- **5-UTR|CDS|3-UTR identity (%) [Gaps excluded]:** 62.5 | 68.69 | 81.58
- **5-UTR|CDS|3-UTR identity [Alignment Gaps]:** 212 | 2086 | 172
- **5-UTR aligned content (<base>:%):** {'G': 28.57, 'C': 34.29, 'A': 17.14, 'T': 20.0}
- **CDS aligned content (<base>:%):** {'A': 24.28, 'G': 26.54, 'C': 27.39, 'T': 21.78}
- **3-UTR aligned content (<base>:%):** {'C': 19.35, 'A': 27.96, 'T': 25.81, 'G': 26.88}

**Uniprot Description:**  
  
 Functions as a Ras GTPase-activating protein. Plays an important role in the expansion and functions of natural killer T (NKT) cells in the liver by negatively regulating RAS activity and the down-stream ERK signaling pathway. N/A   
  
 **Gene Ontology Information:**

Molecular Function

- GTPase activator activity
- identical protein binding

Location

- cell cortex
- cytoplasm
- cytoplasmic side of membrane

Biological process

- negative regulation of Ras protein signal transduction
- positive regulation of NK T cell proliferation
- regulation of GTPase activity

---

86

- **Protein name:** WD repeat- and FYVE domain-containing protein 4
- **Organism:** Homo sapiens
- **Uniprot Accession Number:** Q6ZS81
- **Protein sequence length:** 3184 aa
- **1D identity (%):** 11.47
- **1D identity (%) [Gaps excluded]:** 29.78
- **1D identity - Alignment Gaps:** 1979
- **1D aligned content (<aminoacid>:%):** {'P': 7.32, 'N': 3.52, 'D': 5.42, 'S': 8.94, 'L': 10.84, 'F': 5.42, 'W': 1.9, 'H': 1.9, 'R': 3.25, 'E': 5.15, 'G': 10.3, 'T': 5.42, 'C': 6.78, 'A': 4.88, 'Y': 1.36, 'V': 5.69, 'K': 4.61, 'Q': 3.25, 'I': 3.25, 'M': 0.81}
- **Common reported functions (%):** 0.0
- **Common reported locations (%):** 0.0
- **Common reported processes (%):** 0.0

- **AF ID:** Q6ZS81
- **Chain:** A
- **Protein length:** 1384 aa
- **Resolution:** N/A
- **b-phipsi:** 0.013558
- **w-rdist:** 0.615869
- **t-alpha:** 0.00579
- **Chemical similarity (Tanimoto Index) (%):** 86.08
- **1D identity (%) [PDB]:** 3.14
- **1D identity (%) [Gaps excluded][PDB]:** 65.74
- **1D identity - Alignment Gaps [PDB]:** 2151
- **1D aligned content [PDB] (<aminoacid>:%):** {'F': 2.82, 'S': 15.49, 'A': 11.27, 'I': 7.04, 'K': 5.63, 'Q': 8.45, 'L': 16.9, 'G': 5.63, 'D': 4.23, 'V': 7.04, 'N': 4.23, 'T': 5.63, 'E': 4.23, 'M': 1.41}
- **2D identity (%) [PDB]:** 26.86
- **2D identity (%) [Gaps excluded][PDB]:** 82.76
- **2D identity - Alignment Gaps [PDB]:** 1207
- **2D aligned content [PDB] (<2D-fold>:%):** {'.': 27.29, 'T': 16.67, 'H': 30.42, 'E': 23.54, 'G': 1.88, 'B': 0.21}
- **3D similarity (TM-Score) (%) [PDB]:** 20.54

- **Gene name:** WDFY4
- **Entrez ID:** 57705
- **RefSeq ID:** N/A
- **Sequence length:** N/A
- **5-UTR|CDS|3-UTR identity (%):** N/A | N/A | N/A
- **5-UTR|CDS|3-UTR identity (%) [Gaps excluded]:** N/A | N/A | N/A
- **5-UTR|CDS|3-UTR identity [Alignment Gaps]:** N/A | N/A | N/A
- **5-UTR aligned content (<base>:%):** N/A
- **CDS aligned content (<base>:%):** N/A
- **3-UTR aligned content (<base>:%):** N/A

**Uniprot Description:**  
  
 Plays a critical role in the regulation of cDC1-mediated cross-presentation of viral and tumor antigens in dendritic cells. Mechanistically, acts near the plasma membrane and interacts with endosomal membranes to promote endosomal-to-cytosol antigen trafficking. Also plays a role in B-cell survival through regulation of autophagy.   
  
Interacts with HSP90AB1.   
  
 **Gene Ontology Information:**

Molecular Function   
  
N/A

Location

- early endosome
- endoplasmic reticulum

Biological process

- antigen processing and presentation
- autophagy
- CD8-positive, alpha-beta T cell activation
- cellular response to virus

---

87

- **Protein name:** GATOR complex protein WDR59
- **Organism:** Homo sapiens
- **Uniprot Accession Number:** Q6PJI9
- **Protein sequence length:** 974 aa
- **1D identity (%):** 13.16
- **1D identity (%) [Gaps excluded]:** 22.64
- **1D identity - Alignment Gaps:** 595
- **1D aligned content (<aminoacid>:%):** {'N': 3.21, 'V': 2.67, 'R': 6.95, 'D': 6.95, 'L': 12.83, 'F': 6.42, 'H': 1.6, 'S': 8.56, 'W': 2.14, 'G': 6.42, 'A': 8.02, 'Y': 2.67, 'K': 5.35, 'E': 2.14, 'T': 10.16, 'I': 2.67, 'C': 3.21, 'Q': 1.6, 'P': 6.42}
- **Common reported functions (%):** 0.0
- **Common reported locations (%):** 0.0
- **Common reported processes (%):** 0.0

- **AF ID:** Q6PJI9
- **Chain:** A
- **Protein length:** 974 aa
- **Resolution:** N/A
- **b-phipsi:** 0.007349
- **w-rdist:** 0.700357
- **t-alpha:** 0.002488
- **Chemical similarity (Tanimoto Index) (%):** 83.67
- **1D identity (%) [PDB]:** 3.44
- **1D identity (%) [Gaps excluded][PDB]:** 68.09
- **1D identity - Alignment Gaps [PDB]:** 1769
- **1D aligned content [PDB] (<aminoacid>:%):** {'V': 7.81, 'N': 4.69, 'Q': 6.25, 'A': 7.81, 'L': 17.19, 'T': 6.25, 'S': 12.5, 'G': 6.25, 'D': 4.69, 'I': 6.25, 'P': 1.56, 'E': 3.12, 'R': 6.25, 'Y': 1.56, 'K': 3.12, 'F': 3.12, 'H': 1.56}
- **2D identity (%) [PDB]:** 34.63
- **2D identity (%) [Gaps excluded][PDB]:** 84.68
- **2D identity - Alignment Gaps [PDB]:** 821
- **2D aligned content [PDB] (<2D-fold>:%):** {'.': 17.88, 'E': 37.42, 'T': 11.85, 'H': 32.22, 'G': 0.62}
- **3D similarity (TM-Score) (%) [PDB]:** 19.12

- **Gene name:** WDR59
- **Entrez ID:** 79726
- **RefSeq ID:** NM\_030581
- **Transcript sequence length:** 5878
- **5-UTR|CDS|3-UTR identity (%):** 25.36 | 37.61 | 7.44
- **5-UTR|CDS|3-UTR identity (%) [Gaps excluded]:** 73.68 | 74.22 | 93.39
- **5-UTR|CDS|3-UTR identity [Alignment Gaps]:** 181 | 2209 | 2622
- **5-UTR aligned content (<base>:%):** {'G': 42.86, 'T': 18.57, 'C': 30.0, 'A': 8.57}
- **CDS aligned content (<base>:%):** {'A': 26.6, 'T': 28.86, 'C': 21.5, 'G': 23.04}
- **3-UTR aligned content (<base>:%):** {'A': 40.09, 'T': 22.17, 'C': 16.04, 'G': 21.7}

**Uniprot Description:**  
  
 As a component of the GATOR subcomplex GATOR2, functions within the amino acid-sensing branch of the TORC1 signaling pathway. Indirectly activates mTORC1 and the TORC1 signaling pathway through the inhibition of the GATOR1 subcomplex (PubMed:23723238). It is negatively regulated by the upstream amino acid sensors SESN2 and CASTOR1 (PubMed:25457612, PubMed:27487210).   
  
Interacts with DDB1-CUL4A/B E3 ligase complexes (By similarity). Within the GATOR complex, component of the GATOR2 subcomplex, made of MIOS, SEC13, SEH1L, WDR24 and WDR59. The GATOR complex strongly interacts with RRAGA/RRAGC and RRAGB/RRAGC heterodimers. The GATOR2 complex interacts with CASTOR2 and CASTOR1; the interaction is negatively regulated by arginine (PubMed:26972053). The GATOR2 complex interacts with SESN1, SESN2 and SESN3; the interaction is negatively regulated by amino acids (PubMed:25263562, PubMed:25457612).   
  
 **Gene Ontology Information:**

Molecular Function

- signaling adaptor activity

Location

- GATOR2 complex
- lysosomal membrane
- Seh1-associated complex
- vacuolar membrane

Biological process

- cellular response to amino acid starvation
- negative regulation of TORC1 signaling
- positive regulation of TOR signaling
- positive regulation of TORC1 signaling

---

88

- **Protein name:** Dynein axonemal heavy chain 1
- **Organism:** Homo sapiens
- **Uniprot Accession Number:** Q9P2D7
- **Protein sequence length:** 4265 aa
- **1D identity (%):** 10.07
- **1D identity (%) [Gaps excluded]:** 33.94
- **1D identity - Alignment Gaps:** 3004
- **1D aligned content (<aminoacid>:%):** {'M': 1.16, 'F': 5.58, 'L': 10.47, 'P': 6.74, 'Q': 4.65, 'C': 3.49, 'T': 7.44, 'R': 2.33, 'A': 4.65, 'Y': 5.12, 'G': 7.21, 'K': 4.42, 'V': 6.05, 'H': 1.4, 'W': 1.63, 'N': 6.98, 'D': 4.88, 'S': 6.98, 'I': 4.42, 'E': 4.42}
- **Common reported functions (%):** 0.0
- **Common reported locations (%):** 0.0
- **Common reported processes (%):** 0.0

- **AF ID:** Q9P2D7
- **Chain:** A
- **Protein length:** 1400 aa
- **Resolution:** N/A
- **b-phipsi:** 0.062885
- **w-rdist:** 0.281437
- **t-alpha:** 0.065198
- **Chemical similarity (Tanimoto Index) (%):** 84.16
- **1D identity (%) [PDB]:** 2.94
- **1D identity (%) [Gaps excluded][PDB]:** 64.42
- **1D identity - Alignment Gaps [PDB]:** 2175
- **1D aligned content [PDB] (<aminoacid>:%):** {'V': 11.94, 'F': 11.94, 'N': 5.97, 'G': 8.96, 'L': 10.45, 'T': 8.96, 'E': 5.97, 'S': 7.46, 'K': 1.49, 'R': 2.99, 'A': 7.46, 'D': 2.99, 'P': 4.48, 'I': 4.48, 'Q': 4.48}
- **2D identity (%) [PDB]:** 28.11
- **2D identity (%) [Gaps excluded][PDB]:** 85.42
- **2D identity - Alignment Gaps [PDB]:** 1203
- **2D aligned content [PDB] (<2D-fold>:%):** {'.': 21.43, 'E': 31.15, 'T': 10.71, 'H': 36.11, 'G': 0.6}
- **3D similarity (TM-Score) (%) [PDB]:** 20.5

- **Gene name:** DNAH1
- **Entrez ID:** 25981
- **RefSeq ID:** N/A
- **Sequence length:** N/A
- **5-UTR|CDS|3-UTR identity (%):** N/A | N/A | N/A
- **5-UTR|CDS|3-UTR identity (%) [Gaps excluded]:** N/A | N/A | N/A
- **5-UTR|CDS|3-UTR identity [Alignment Gaps]:** N/A | N/A | N/A
- **5-UTR aligned content (<base>:%):** N/A
- **CDS aligned content (<base>:%):** N/A
- **3-UTR aligned content (<base>:%):** N/A

**Uniprot Description:**  
  
 Force generating protein of cilia required for sperm flagellum motility. Produces force towards the minus ends of microtubules. Dynein has ATPase activity; the force-producing power stroke is thought to occur on release of ADP. Required in spermatozoa for the formation of the inner dynein arms and biogenesis of the axoneme (PubMed:24360805).   
  
Consists of at least two heavy chains and a number of intermediate and light chains.   
  
 **Gene Ontology Information:**

Molecular Function

- ATP binding
- dynein intermediate chain binding
- dynein light intermediate chain binding
- microtubule motor activity
- ATP-dependent microtubule motor activity, minus-end-directed

Location

- axonemal dynein complex
- axoneme
- dynein complex
- extracellular region
- inner dynein arm
- microtubule
- sperm flagellum

Biological process

- cilium movement
- cilium-dependent cell motility
- epithelial cilium movement involved in extracellular fluid movement
- flagellated sperm motility
- inner dynein arm assembly
- microtubule-based movement
- sperm axoneme assembly

---

89

- **Protein name:** Glucocorticoid modulatory element-binding protein 1
- **Organism:** Homo sapiens
- **Uniprot Accession Number:** Q9Y692
- **Protein sequence length:** 573 aa
- **1D identity (%):** 7.36
- **1D identity (%) [Gaps excluded]:** 21.35
- **1D identity - Alignment Gaps:** 900
- **1D aligned content (<aminoacid>:%):** {'N': 4.95, 'P': 6.93, 'V': 9.9, 'T': 5.94, 'E': 4.95, 'I': 4.95, 'G': 11.88, 'S': 3.96, 'A': 5.94, 'D': 8.91, 'Y': 0.99, 'K': 6.93, 'F': 4.95, 'L': 9.9, 'C': 0.99, 'Q': 5.94, 'R': 1.98}
- **Common reported functions (%):** 0.0
- **Common reported locations (%):** 0.0
- **Common reported processes (%):** 0.0

- **AF ID:** Q9Y692
- **Chain:** A
- **Protein length:** 573 aa
- **Resolution:** N/A
- **b-phipsi:** 0.04088
- **w-rdist:** 0.347696
- **t-alpha:** 0.013234
- **Chemical similarity (Tanimoto Index) (%):** 84.16
- **1D identity (%) [PDB]:** 2.12
- **1D identity (%) [Gaps excluded][PDB]:** 72.73
- **1D identity - Alignment Gaps [PDB]:** 1468
- **1D aligned content [PDB] (<aminoacid>:%):** {'M': 3.12, 'A': 6.25, 'R': 3.12, 'G': 12.5, 'I': 12.5, 'V': 6.25, 'Q': 9.38, 'N': 3.12, 'L': 12.5, 'Y': 3.12, 'E': 3.12, 'K': 3.12, 'S': 15.62, 'T': 3.12, 'D': 3.12}
- **2D identity (%) [PDB]:** 19.58
- **2D identity (%) [Gaps excluded][PDB]:** 87.68
- **2D identity - Alignment Gaps [PDB]:** 988
- **2D aligned content [PDB] (<2D-fold>:%):** {'.': 27.31, 'T': 7.63, 'E': 10.04, 'H': 55.02}
- **3D similarity (TM-Score) (%) [PDB]:** 14.08

- **Gene name:** GMEB1
- **Entrez ID:** 540374
- **RefSeq ID:** N/A
- **Sequence length:** N/A
- **5-UTR|CDS|3-UTR identity (%):** N/A | N/A | N/A
- **5-UTR|CDS|3-UTR identity (%) [Gaps excluded]:** N/A | N/A | N/A
- **5-UTR|CDS|3-UTR identity [Alignment Gaps]:** N/A | N/A | N/A
- **5-UTR aligned content (<base>:%):** N/A
- **CDS aligned content (<base>:%):** N/A
- **3-UTR aligned content (<base>:%):** N/A

**Uniprot Description:**  
  
 Trans-acting factor that binds to glucocorticoid modulatory elements (GME) present in the TAT (tyrosine aminotransferase) promoter and increases sensitivity to low concentrations of glucocorticoids. Binds also to the transferrin receptor promoter. Essential auxiliary factor for the replication of parvoviruses.   
  
Homodimer, and heterodimer of GMEB1 and GMEB2. GMEB1 and GMEB2 form the parvovirus initiator complex (PIF). Interacts with the glucocorticoid receptor (NR3C1) and NCOA2/TIF2 (By similarity). May interact with HSP27 and CREB-binding protein (CBP).   
  
 **Gene Ontology Information:**

Molecular Function

- DNA-binding transcription activator activity, RNA polymerase II-specific
- metal ion binding
- RNA polymerase II cis-regulatory region sequence-specific DNA binding

Location

- cytoplasm
- nucleus

Biological process   
  
N/A

---

90

- **Protein name:** Kinesin-like protein KIF18A
- **Organism:** Homo sapiens
- **Uniprot Accession Number:** Q8NI77
- **Protein sequence length:** 898 aa
- **1D identity (%):** 11.41
- **1D identity (%) [Gaps excluded]:** 21.94
- **1D identity - Alignment Gaps:** 685
- **1D aligned content (<aminoacid>:%):** {'C': 4.91, 'P': 7.98, 'T': 7.36, 'G': 5.52, 'V': 5.52, 'L': 11.66, 'F': 6.75, 'H': 1.23, 'N': 9.2, 'K': 7.36, 'D': 4.29, 'S': 6.75, 'I': 7.36, 'R': 4.91, 'E': 3.68, 'Q': 4.29, 'Y': 0.61, 'M': 0.61}
- **Common reported functions (%):** 0.0
- **Common reported locations (%):** 0.0
- **Common reported processes (%):** 0.0

- **AF ID:** Q8NI77
- **Chain:** A
- **Protein length:** 898 aa
- **Resolution:** N/A
- **b-phipsi:** 0.025821
- **w-rdist:** 0.419984
- **t-alpha:** 0.002481
- **Chemical similarity (Tanimoto Index) (%):** 83.81
- **1D identity (%) [PDB]:** 2.3
- **1D identity (%) [Gaps excluded][PDB]:** 77.78
- **1D identity - Alignment Gaps [PDB]:** 1773
- **1D aligned content [PDB] (<aminoacid>:%):** {'G': 4.76, 'K': 7.14, 'I': 7.14, 'D': 7.14, 'L': 19.05, 'S': 16.67, 'T': 4.76, 'A': 9.52, 'Q': 7.14, 'V': 2.38, 'N': 7.14, 'F': 2.38, 'R': 2.38, 'P': 2.38}
- **2D identity (%) [PDB]:** 23.09
- **2D identity (%) [Gaps excluded][PDB]:** 85.5
- **2D identity - Alignment Gaps [PDB]:** 1081
- **2D aligned content [PDB] (<2D-fold>:%):** {'.': 15.79, 'T': 8.48, 'E': 27.19, 'H': 48.54}
- **3D similarity (TM-Score) (%) [PDB]:** 21.62

- **Gene name:** KIF18A
- **Entrez ID:** 81930
- **RefSeq ID:** N/A
- **Sequence length:** N/A
- **5-UTR|CDS|3-UTR identity (%):** N/A | N/A | N/A
- **5-UTR|CDS|3-UTR identity (%) [Gaps excluded]:** N/A | N/A | N/A
- **5-UTR|CDS|3-UTR identity [Alignment Gaps]:** N/A | N/A | N/A
- **5-UTR aligned content (<base>:%):** N/A
- **CDS aligned content (<base>:%):** N/A
- **3-UTR aligned content (<base>:%):** N/A

**Uniprot Description:**  
  
 Microtubule-depolymerizing kinesin which plays a role in chromosome congression by reducing the amplitude of preanaphase oscillations and slowing poleward movement during anaphase, thus suppressing chromosome movements. May stabilize the CENPE-BUB1B complex at the kinetochores during early mitosis and maintains CENPE levels at kinetochores during chromosome congression.   
  
Interacts with CENPE and ESR1.   
  
 **Gene Ontology Information:**

Molecular Function

- actin binding
- ATP binding
- ATPase activity
- microtubule binding
- microtubule motor activity
- microtubule plus-end binding
- ATP-dependent microtubule motor activity, plus-end-directed
- tubulin-dependent ATPase activity

Location

- caveola
- cytoplasm
- cytosol
- kinesin complex
- kinetochore
- kinetochore microtubule
- microtubule
- microtubule cytoskeleton
- microtubule organizing center
- mitotic spindle astral microtubule
- mitotic spindle midzone
- nucleus
- ruffle

Biological process

- cellular response to estradiol stimulus
- male meiotic nuclear division
- microtubule depolymerization
- microtubule-based movement
- mitotic metaphase plate congression
- mitotic sister chromatid segregation
- protein transport
- regulation of microtubule cytoskeleton organization
- seminiferous tubule development

---

91

- **Protein name:** Protein Aster-A
- **Organism:** Homo sapiens
- **Uniprot Accession Number:** Q96CP6
- **Protein sequence length:** 724 aa
- **1D identity (%):** 11.81
- **1D identity (%) [Gaps excluded]:** 22.63
- **1D identity - Alignment Gaps:** 627
- **1D aligned content (<aminoacid>:%):** {'F': 3.23, 'T': 9.68, 'H': 0.65, 'P': 7.74, 'S': 11.61, 'L': 12.26, 'R': 4.52, 'E': 5.16, 'G': 9.68, 'V': 6.45, 'D': 3.87, 'N': 4.52, 'Y': 2.58, 'C': 1.94, 'I': 1.94, 'K': 5.16, 'Q': 3.87, 'A': 5.16}
- **Common reported functions (%):** 0.0
- **Common reported locations (%):** 20.0
- **Common reported processes (%):** 0.0

- **AF ID:** Q96CP6
- **Chain:** A
- **Protein length:** 724 aa
- **Resolution:** N/A
- **b-phipsi:** 0.036688
- **w-rdist:** 0.358499
- **t-alpha:** 0.047146
- **Chemical similarity (Tanimoto Index) (%):** 85.86
- **1D identity (%) [PDB]:** 2.67
- **1D identity (%) [Gaps excluded][PDB]:** 70.97
- **1D identity - Alignment Gaps [PDB]:** 1583
- **1D aligned content [PDB] (<aminoacid>:%):** {'F': 6.82, 'Q': 4.55, 'L': 18.18, 'P': 9.09, 'S': 9.09, 'R': 2.27, 'I': 2.27, 'E': 2.27, 'D': 4.55, 'K': 2.27, 'V': 4.55, 'T': 11.36, 'N': 2.27, 'G': 11.36, 'A': 9.09}
- **2D identity (%) [PDB]:** 32.79
- **2D identity (%) [Gaps excluded][PDB]:** 85.05
- **2D identity - Alignment Gaps [PDB]:** 757
- **2D aligned content [PDB] (<2D-fold>:%):** {'.': 24.75, 'E': 30.2, 'T': 8.17, 'H': 36.39, 'G': 0.5}
- **3D similarity (TM-Score) (%) [PDB]:** 14.73

- **Gene name:** GRAMD1A
- **Entrez ID:** 57655
- **RefSeq ID:** NM\_020895
- **Transcript sequence length:** 2659
- **5-UTR|CDS|3-UTR identity (%):** 25.93 | 30.3 | 35.62
- **5-UTR|CDS|3-UTR identity (%) [Gaps excluded]:** 62.1 | 72.19 | 75.84
- **5-UTR|CDS|3-UTR identity [Alignment Gaps]:** 173 | 2451 | 201
- **5-UTR aligned content (<base>:%):** {'C': 41.56, 'T': 20.78, 'G': 31.17, 'A': 6.49}
- **CDS aligned content (<base>:%):** {'A': 23.91, 'T': 23.91, 'C': 27.5, 'G': 24.69}
- **3-UTR aligned content (<base>:%):** {'G': 26.67, 'T': 23.7, 'A': 28.89, 'C': 20.74}

**Uniprot Description:**  
  
 Cholesterol transporter that mediates non-vesicular transport of cholesterol from the plasma membrane (PM) to the endoplasmic reticulum (ER) (By similarity). Contains unique domains for binding cholesterol and the PM, thereby serving as a molecular bridge for the transfer of cholesterol from the PM to the ER (By similarity). Plays a crucial role in cholesterol homeostasis and has the unique ability to localize to the PM based on the level of membrane cholesterol (By similarity). In lipid-poor conditions localizes to the ER membrane and in response to excess cholesterol in the PM is recruited to the endoplasmic reticulum-plasma membrane contact sites (EPCS) which is mediated by the GRAM domain (By similarity). At the EPCS, the sterol-binding VASt/ASTER domain binds to the cholesterol in the PM and facilitates its transfer from the PM to ER (By similarity). May play a role in tumor progression (By similarity). Plays a role in autophagy regulation and is required for biogenesis of the autophagosome (PubMed:31222192). This function in autophagy requires its cholesterol-transfer activity (PubMed:31222192). N/A   
  
 **Gene Ontology Information:**

Molecular Function

- cholesterol binding
- cholesterol transfer activity
- sterol binding
- sterol transfer activity

Location

- autophagosome
- cytoplasmic vesicle
- cytosol
- endoplasmic reticulum membrane
- endoplasmic reticulum-plasma membrane contact site
- extrinsic component of cytoplasmic side of plasma membrane
- organelle membrane contact site
- plasma membrane

Biological process

- autophagy
- cellular response to cholesterol
- intracellular sterol transport

---

92

- **Protein name:** Maltase-glucoamylase, intestinal
- **Organism:** Homo sapiens
- **Uniprot Accession Number:** O43451
- **Protein sequence length:** 1857 aa
- **1D identity (%):** 17.25
- **1D identity (%) [Gaps excluded]:** 28.2
- **1D identity - Alignment Gaps:** 754
- **1D aligned content (<aminoacid>:%):** {'M': 0.3, 'V': 8.96, 'L': 8.36, 'T': 8.06, 'R': 2.69, 'P': 8.06, 'N': 9.25, 'D': 5.67, 'W': 1.49, 'H': 2.69, 'G': 9.85, 'F': 7.76, 'S': 5.97, 'K': 2.99, 'Y': 3.88, 'Q': 3.58, 'E': 2.69, 'I': 2.99, 'A': 3.88, 'C': 0.9}
- **Common reported functions (%):** 0.0
- **Common reported locations (%):** 20.0
- **Common reported processes (%):** 0.0

- **AF ID:** O43451
- **Chain:** A
- **Protein length:** 1857 aa
- **Resolution:** N/A
- **b-phipsi:** 0.001582
- **w-rdist:** 0.790128
- **t-alpha:** 0.09512
- **Chemical similarity (Tanimoto Index) (%):** 86.01
- **1D identity (%) [PDB]:** 3.0
- **1D identity (%) [Gaps excluded][PDB]:** 79.61
- **1D identity - Alignment Gaps [PDB]:** 2634
- **1D aligned content [PDB] (<aminoacid>:%):** {'S': 15.85, 'L': 14.63, 'T': 4.88, 'A': 10.98, 'G': 7.32, 'V': 7.32, 'N': 4.88, 'Q': 7.32, 'F': 3.66, 'I': 6.1, 'R': 4.88, 'D': 2.44, 'P': 1.22, 'E': 2.44, 'Y': 2.44, 'K': 3.66}
- **2D identity (%) [PDB]:** N/A
- **2D identity (%) [Gaps excluded][PDB]:** N/A
- **2D identity - Alignment Gaps [PDB]:** N/A
- **2D aligned content [PDB] (<2D-fold>:%):** N/A
- **3D similarity (TM-Score) (%) [PDB]:** 29.17

- **Gene name:** MGAM
- **Entrez ID:** 8972
- **RefSeq ID:** NM\_004668
- **Transcript sequence length:** 6484
- **5-UTR|CDS|3-UTR identity (%):** 13.19 | 42.5 | 21.49
- **5-UTR|CDS|3-UTR identity (%) [Gaps excluded]:** 78.26 | 75.18 | 82.59
- **5-UTR|CDS|3-UTR identity [Alignment Gaps]:** 227 | 2610 | 637
- **5-UTR aligned content (<base>:%):** {'T': 16.67, 'G': 33.33, 'C': 22.22, 'A': 27.78}
- **CDS aligned content (<base>:%):** {'A': 28.69, 'T': 31.63, 'G': 19.4, 'C': 20.27}
- **3-UTR aligned content (<base>:%):** {'A': 44.32, 'T': 22.7, 'G': 20.54, 'C': 12.43}

**Uniprot Description:**  
  
 May serve as an alternate pathway for starch digestion when luminal alpha-amylase activity is reduced because of immaturity or malnutrition. May play a unique role in the digestion of malted dietary oligosaccharides used in food manufacturing.   
  
Monomer.   
  
 **Gene Ontology Information:**

Molecular Function

- alpha-1,4-glucosidase activity
- amylase activity
- carbohydrate binding
- catalytic activity
- glucan 1,4-alpha-glucosidase activity
- hydrolase activity, hydrolyzing O-glycosyl compounds
- maltose alpha-glucosidase activity

Location

- apical plasma membrane
- extracellular exosome
- ficolin-1-rich granule membrane
- plasma membrane
- tertiary granule membrane

Biological process

- dextrin catabolic process
- maltose catabolic process
- starch catabolic process

---

93

- **Protein name:** SKI2 subunit of superkiller complex protein
- **Organism:** Homo sapiens
- **Uniprot Accession Number:** Q15477
- **Protein sequence length:** 1246 aa
- **1D identity (%):** 14.76
- **1D identity (%) [Gaps excluded]:** 22.87
- **1D identity - Alignment Gaps:** 543
- **1D aligned content (<aminoacid>:%):** {'M': 0.44, 'L': 12.39, 'V': 7.52, 'P': 7.96, 'C': 0.44, 'A': 9.73, 'S': 8.41, 'G': 8.85, 'D': 7.52, 'F': 4.42, 'W': 0.88, 'H': 0.44, 'Q': 3.54, 'T': 7.08, 'E': 5.31, 'N': 3.1, 'I': 2.21, 'K': 3.1, 'R': 4.87, 'Y': 1.77}
- **Common reported functions (%):** 0.0
- **Common reported locations (%):** 0.0
- **Common reported processes (%):** 0.0

- **AF ID:** Q15477
- **Chain:** A
- **Protein length:** 1246 aa
- **Resolution:** N/A
- **b-phipsi:** 0.035933
- **w-rdist:** 0.365141
- **t-alpha:** 0.176178
- **Chemical similarity (Tanimoto Index) (%):** 83.97
- **1D identity (%) [PDB]:** 3.13
- **1D identity (%) [Gaps excluded][PDB]:** 77.91
- **1D identity - Alignment Gaps [PDB]:** 2057
- **1D aligned content [PDB] (<aminoacid>:%):** {'A': 8.96, 'V': 5.97, 'E': 5.97, 'D': 7.46, 'N': 1.49, 'T': 4.48, 'F': 8.96, 'K': 7.46, 'Q': 4.48, 'I': 5.97, 'Y': 2.99, 'P': 8.96, 'G': 5.97, 'S': 5.97, 'L': 13.43, 'R': 1.49}
- **2D identity (%) [PDB]:** 29.47
- **2D identity (%) [Gaps excluded][PDB]:** 83.79
- **2D identity - Alignment Gaps [PDB]:** 1069
- **2D aligned content [PDB] (<2D-fold>:%):** {'.': 21.81, 'E': 29.01, 'T': 13.17, 'H': 35.39, 'G': 0.62}
- **3D similarity (TM-Score) (%) [PDB]:** 20.36

- **Gene name:** SKIC2
- **Entrez ID:** 378722
- **RefSeq ID:** NM\_006929
- **Transcript sequence length:** 3795
- **5-UTR|CDS|3-UTR identity (%):** 5.26 | 43.27 | 11.74
- **5-UTR|CDS|3-UTR identity (%) [Gaps excluded]:** 82.35 | 72.85 | 77.14
- **5-UTR|CDS|3-UTR identity [Alignment Gaps]:** 249 | 1927 | 195
- **5-UTR aligned content (<base>:%):** {'A': 7.14, 'C': 21.43, 'T': 28.57, 'G': 42.86}
- **CDS aligned content (<base>:%):** {'A': 24.5, 'T': 27.72, 'G': 24.26, 'C': 23.53}
- **3-UTR aligned content (<base>:%):** {'A': 59.26, 'T': 14.81, 'C': 18.52, 'G': 7.41}

**Uniprot Description:**  
  
 Helicase; has ATPase activity (PubMed:7610041). Component of the SKI complex which is thought to be involved in exosome-mediated RNA decay and associates with transcriptionally active genes in a manner dependent on PAF1 complex (PAF1C) (PubMed:16024656).   
  
Component of the SKI complex which consists of SKIC2, SKIC3 and SKIC8 (PubMed:16024656). Interacts with HBS1L isoform 2 (PubMed:28204585).   
  
 **Gene Ontology Information:**

Molecular Function

- NADP-retinol dehydrogenase activity
- oxidoreductase activity, acting on the CH-OH group of donors, NAD or NADP as acceptor

Location

- endoplasmic reticulum membrane
- lipid droplet

Biological process   
  
N/A

---

94

- **Protein name:** Zinc finger ZZ-type and EF-hand domain-containing protein 1
- **Organism:** Homo sapiens
- **Uniprot Accession Number:** O43149
- **Protein sequence length:** 2961 aa
- **1D identity (%):** 12.03
- **1D identity (%) [Gaps excluded]:** 28.74
- **1D identity - Alignment Gaps:** 1736
- **1D aligned content (<aminoacid>:%):** {'P': 5.57, 'S': 7.8, 'V': 5.01, 'T': 6.96, 'L': 13.65, 'A': 5.57, 'F': 5.85, 'G': 9.19, 'D': 5.57, 'E': 4.18, 'I': 5.29, 'R': 3.06, 'N': 4.46, 'C': 5.85, 'K': 4.74, 'Y': 2.51, 'Q': 2.23, 'H': 1.67, 'W': 0.84}
- **Common reported functions (%):** 0.0
- **Common reported locations (%):** 0.0
- **Common reported processes (%):** 0.0

- **AF ID:** O43149
- **Chain:** A
- **Protein length:** 1400 aa
- **Resolution:** N/A
- **b-phipsi:** 0.027425
- **w-rdist:** 0.385784
- **t-alpha:** 0.054591
- **Chemical similarity (Tanimoto Index) (%):** 83.97
- **1D identity (%) [PDB]:** 3.02
- **1D identity (%) [Gaps excluded][PDB]:** 67.65
- **1D identity - Alignment Gaps [PDB]:** 2179
- **1D aligned content [PDB] (<aminoacid>:%):** {'V': 8.7, 'T': 5.8, 'L': 18.84, 'E': 4.35, 'N': 5.8, 'A': 8.7, 'Q': 11.59, 'S': 11.59, 'I': 4.35, 'K': 4.35, 'G': 4.35, 'D': 5.8, 'P': 1.45, 'R': 2.9, 'Y': 1.45}
- **2D identity (%) [PDB]:** 27.83
- **2D identity (%) [Gaps excluded][PDB]:** 86.7
- **2D identity - Alignment Gaps [PDB]:** 1225
- **2D aligned content [PDB] (<2D-fold>:%):** {'.': 16.14, 'T': 11.95, 'E': 35.66, 'H': 35.06, 'G': 1.2}
- **3D similarity (TM-Score) (%) [PDB]:** 23.99

- **Gene name:** ZZEF1
- **Entrez ID:** 23140
- **RefSeq ID:** NM\_015113
- **Transcript sequence length:** 11466
- **5-UTR|CDS|3-UTR identity (%):** 30.66 | 31.99 | 8.67
- **5-UTR|CDS|3-UTR identity (%) [Gaps excluded]:** 66.67 | 80.19 | 92.58
- **5-UTR|CDS|3-UTR identity [Alignment Gaps]:** 148 | 5460 | 2216
- **5-UTR aligned content (<base>:%):** {'A': 15.48, 'G': 38.1, 'C': 34.52, 'T': 11.9}
- **CDS aligned content (<base>:%):** {'A': 28.87, 'T': 31.18, 'G': 19.89, 'C': 20.06}
- **3-UTR aligned content (<base>:%):** {'C': 16.51, 'A': 38.21, 'T': 23.58, 'G': 21.7}

**Uniprot Description:**  
  
 Histone H3 reader which may act as a transcriptional coactivator for KLF6 and KLF9 transcription factors.   
  
Interacts with KLF6 and KLF9 (PubMed:33227311). Interacts via (ZZ-type 2 zinc finger) with histone H3 trimethylated at 'Lys-4' (H3K4me3) and histone H3 acetylated at 'Lys-4' (H3K4ac) (PubMed:33227311).   
  
 **Gene Ontology Information:**

Molecular Function

- calcium ion binding
- histone reader activity
- lysine-acetylated histone binding
- methylated histone binding
- zinc ion binding

Location   
  
N/A

Biological process   
  
N/A

---

95

- **Protein name:** Protein SLX4IP
- **Organism:** Homo sapiens
- **Uniprot Accession Number:** Q5VYV7
- **Protein sequence length:** 408 aa
- **1D identity (%):** 6.11
- **1D identity (%) [Gaps excluded]:** 25.78
- **1D identity - Alignment Gaps:** 1037
- **1D aligned content (<aminoacid>:%):** {'F': 7.23, 'K': 7.23, 'G': 7.23, 'V': 3.61, 'D': 6.02, 'P': 10.84, 'S': 6.02, 'E': 3.61, 'C': 6.02, 'L': 13.25, 'T': 7.23, 'R': 4.82, 'Q': 9.64, 'Y': 2.41, 'N': 1.2, 'I': 1.2, 'A': 2.41}
- **Common reported functions (%):** 0.0
- **Common reported locations (%):** 0.0
- **Common reported processes (%):** 0.0

- **AF ID:** Q5VYV7
- **Chain:** A
- **Protein length:** 408 aa
- **Resolution:** N/A
- **b-phipsi:** 0.068632
- **w-rdist:** 0.269974
- **t-alpha:** 0.125699
- **Chemical similarity (Tanimoto Index) (%):** 85.69
- **1D identity (%) [PDB]:** 1.09
- **1D identity (%) [Gaps excluded][PDB]:** 75.0
- **1D identity - Alignment Gaps [PDB]:** 1351
- **1D aligned content [PDB] (<aminoacid>:%):** {'L': 20.0, 'V': 6.67, 'D': 6.67, 'I': 13.33, 'G': 6.67, 'T': 13.33, 'R': 6.67, 'F': 6.67, 'Q': 6.67, 'A': 6.67, 'H': 6.67}
- **2D identity (%) [PDB]:** 10.53
- **2D identity (%) [Gaps excluded][PDB]:** 82.8
- **2D identity - Alignment Gaps [PDB]:** 1077
- **2D aligned content [PDB] (<2D-fold>:%):** {'.': 30.0, 'E': 31.54, 'T': 12.31, 'H': 25.38, 'B': 0.77}
- **3D similarity (TM-Score) (%) [PDB]:** 9.55

- **Gene name:** SLX4IP
- **Entrez ID:** 128710
- **RefSeq ID:** NM\_001009608
- **Transcript sequence length:** 6056
- **5-UTR|CDS|3-UTR identity (%):** 34.11 | 20.55 | 4.62
- **5-UTR|CDS|3-UTR identity (%) [Gaps excluded]:** 73.05 | 74.82 | 93.89
- **5-UTR|CDS|3-UTR identity [Alignment Gaps]:** 161 | 2873 | 4422
- **5-UTR aligned content (<base>:%):** {'G': 21.36, 'A': 24.27, 'T': 28.16, 'C': 26.21}
- **CDS aligned content (<base>:%):** {'A': 34.52, 'T': 25.92, 'C': 19.41, 'G': 20.15}
- **3-UTR aligned content (<base>:%):** {'C': 15.35, 'A': 40.0, 'T': 23.72, 'G': 20.93}

**Uniprot Description:**  
  
 N/A   
  
Interacts with SLX4/BTBD12; subunit of different structure-specific endonucleases.   
  
 **Gene Ontology Information:**

Molecular Function   
  
N/A

Location   
  
N/A

Biological process   
  
N/A

---

96

- **Protein name:** Zinc finger protein 398
- **Organism:** Homo sapiens
- **Uniprot Accession Number:** Q8TD17
- **Protein sequence length:** 642 aa
- **1D identity (%):** 10.36
- **1D identity (%) [Gaps excluded]:** 26.1
- **1D identity - Alignment Gaps:** 827
- **1D aligned content (<aminoacid>:%):** {'A': 4.23, 'P': 10.56, 'S': 9.86, 'E': 3.52, 'C': 4.93, 'L': 9.15, 'Q': 3.52, 'T': 9.86, 'V': 3.52, 'G': 13.38, 'K': 4.23, 'N': 4.23, 'Y': 2.11, 'R': 4.93, 'I': 2.82, 'F': 3.52, 'W': 1.41, 'D': 2.82, 'H': 0.7, 'M': 0.7}
- **Common reported functions (%):** 0.0
- **Common reported locations (%):** 0.0
- **Common reported processes (%):** 0.0

- **AF ID:** Q8TD17
- **Chain:** A
- **Protein length:** 642 aa
- **Resolution:** N/A
- **b-phipsi:** 0.031137
- **w-rdist:** 0.377948
- **t-alpha:** 0.039553
- **Chemical similarity (Tanimoto Index) (%):** 84.09
- **1D identity (%) [PDB]:** 1.9
- **1D identity (%) [Gaps excluded][PDB]:** 63.83
- **1D identity - Alignment Gaps [PDB]:** 1531
- **1D aligned content [PDB] (<aminoacid>:%):** {'A': 10.0, 'L': 16.67, 'T': 6.67, 'V': 6.67, 'K': 3.33, 'S': 6.67, 'N': 3.33, 'F': 3.33, 'G': 6.67, 'I': 10.0, 'D': 10.0, 'P': 3.33, 'E': 6.67, 'Q': 3.33, 'R': 3.33}
- **2D identity (%) [PDB]:** 16.75
- **2D identity (%) [Gaps excluded][PDB]:** 88.76
- **2D identity - Alignment Gaps [PDB]:** 1109
- **2D aligned content [PDB] (<2D-fold>:%):** {'.': 13.1, 'T': 8.73, 'H': 73.8, 'E': 4.37}
- **3D similarity (TM-Score) (%) [PDB]:** 13.39

- **Gene name:** ZNF398
- **Entrez ID:** 57541
- **RefSeq ID:** N/A
- **Sequence length:** N/A
- **5-UTR|CDS|3-UTR identity (%):** N/A | N/A | N/A
- **5-UTR|CDS|3-UTR identity (%) [Gaps excluded]:** N/A | N/A | N/A
- **5-UTR|CDS|3-UTR identity [Alignment Gaps]:** N/A | N/A | N/A
- **5-UTR aligned content (<base>:%):** N/A
- **CDS aligned content (<base>:%):** N/A
- **3-UTR aligned content (<base>:%):** N/A

**Uniprot Description:**  
  
 Functions as a transcriptional activator. N/A   
  
 **Gene Ontology Information:**

Molecular Function

- DNA-binding transcription factor activity, RNA polymerase II-specific
- DNA-binding transcription repressor activity, RNA polymerase II-specific
- metal ion binding
- RNA polymerase II transcription regulatory region sequence-specific DNA binding

Location

- nucleus

Biological process

- negative regulation of transcription by RNA polymerase II
- positive regulation of transcription, DNA-templated
- regulation of transcription, DNA-templated
- regulation of transcription by RNA polymerase II

---

97

- **Protein name:** Ubiquitin carboxyl-terminal hydrolase 48
- **Organism:** Homo sapiens
- **Uniprot Accession Number:** Q86UV5
- **Protein sequence length:** 1035 aa
- **1D identity (%):** 15.13
- **1D identity (%) [Gaps excluded]:** 22.02
- **1D identity - Alignment Gaps:** 428
- **1D aligned content (<aminoacid>:%):** {'A': 2.9, 'V': 5.31, 'E': 5.8, 'R': 3.86, 'C': 2.9, 'P': 7.73, 'G': 10.63, 'F': 4.35, 'N': 6.76, 'I': 3.38, 'D': 5.8, 'L': 14.01, 'Y': 4.83, 'S': 6.76, 'T': 4.35, 'K': 5.8, 'Q': 4.35, 'W': 0.48}
- **Common reported functions (%):** 0.0
- **Common reported locations (%):** 0.0
- **Common reported processes (%):** 0.0

- **AF ID:** Q86UV5
- **Chain:** A
- **Protein length:** 1035 aa
- **Resolution:** N/A
- **b-phipsi:** 0.01934
- **w-rdist:** 0.592057
- **t-alpha:** 0.006661
- **Chemical similarity (Tanimoto Index) (%):** 85.62
- **1D identity (%) [PDB]:** 3.09
- **1D identity (%) [Gaps excluded][PDB]:** 75.95
- **1D identity - Alignment Gaps [PDB]:** 1860
- **1D aligned content [PDB] (<aminoacid>:%):** {'V': 8.33, 'R': 8.33, 'F': 8.33, 'N': 6.67, 'I': 8.33, 'T': 8.33, 'L': 3.33, 'C': 1.67, 'P': 1.67, 'G': 1.67, 'E': 3.33, 'A': 10.0, 'S': 10.0, 'Y': 6.67, 'K': 5.0, 'D': 5.0, 'Q': 3.33}
- **2D identity (%) [PDB]:** 29.89
- **2D identity (%) [Gaps excluded][PDB]:** 84.12
- **2D identity - Alignment Gaps [PDB]:** 960
- **2D aligned content [PDB] (<2D-fold>:%):** {'.': 16.63, 'H': 37.08, 'G': 2.02, 'T': 14.61, 'E': 29.44, 'B': 0.22}
- **3D similarity (TM-Score) (%) [PDB]:** 18.47

- **Gene name:** USP48
- **Entrez ID:** 84196
- **RefSeq ID:** NM\_032236
- **Transcript sequence length:** 4419
- **5-UTR|CDS|3-UTR identity (%):** 33.33 | 43.97 | 17.16
- **5-UTR|CDS|3-UTR identity (%) [Gaps excluded]:** 65.58 | 75.53 | 86.55
- **5-UTR|CDS|3-UTR identity [Alignment Gaps]:** 149 | 1830 | 902
- **5-UTR aligned content (<base>:%):** {'G': 39.6, 'C': 28.71, 'T': 18.81, 'A': 12.87}
- **CDS aligned content (<base>:%):** {'A': 33.8, 'T': 30.06, 'G': 20.51, 'C': 15.63}
- **3-UTR aligned content (<base>:%):** {'T': 21.76, 'C': 11.92, 'A': 45.08, 'G': 21.24}

**Uniprot Description:**  
  
 Recognizes and hydrolyzes the peptide bond at the C-terminal Gly of ubiquitin. Involved in the processing of poly-ubiquitin precursors as well as that of ubiquitinated proteins. May be involved in the regulation of NF-kappa-B activation by TNF receptor superfamily via its interactions with RELA and TRAF2. May also play a regulatory role at postsynaptic sites.   
  
Interacts with TRAF2 and RELA.   
  
 **Gene Ontology Information:**

Molecular Function

- thiol-dependent ubiquitin-specific protease activity
- cysteine-type endopeptidase activity

Location

- cytosol
- mitochondrion
- nucleoplasm
- nucleus

Biological process

- protein deubiquitination
- ubiquitin-dependent protein catabolic process

---

98

- **Protein name:** von Willebrand factor
- **Organism:** Homo sapiens
- **Uniprot Accession Number:** P04275
- **Protein sequence length:** 2813 aa
- **1D identity (%):** 13.88
- **1D identity (%) [Gaps excluded]:** 32.14
- **1D identity - Alignment Gaps:** 1622
- **1D aligned content (<aminoacid>:%):** {'M': 0.51, 'V': 6.31, 'L': 8.08, 'C': 6.06, 'T': 5.81, 'R': 4.8, 'N': 5.05, 'F': 5.3, 'Y': 4.29, 'S': 7.58, 'Q': 4.29, 'G': 11.36, 'P': 6.82, 'D': 5.81, 'E': 4.29, 'W': 0.76, 'K': 3.54, 'I': 2.53, 'A': 6.31, 'H': 0.51}
- **Common reported functions (%):** 0.0
- **Common reported locations (%):** 0.0
- **Common reported processes (%):** 0.0

- **AF ID:** P04275
- **Chain:** A
- **Protein length:** 1400 aa
- **Resolution:** N/A
- **b-phipsi:** 0.004414
- **w-rdist:** 0.782302
- **t-alpha:** 0.032258
- **Chemical similarity (Tanimoto Index) (%):** 86.13
- **1D identity (%) [PDB]:** 3.13
- **1D identity (%) [Gaps excluded][PDB]:** 63.39
- **1D identity - Alignment Gaps [PDB]:** 2159
- **1D aligned content [PDB] (<aminoacid>:%):** {'L': 16.9, 'K': 2.82, 'Q': 8.45, 'D': 5.63, 'V': 12.68, 'N': 7.04, 'A': 5.63, 'T': 2.82, 'S': 9.86, 'I': 7.04, 'R': 5.63, 'E': 5.63, 'Y': 1.41, 'G': 4.23, 'F': 2.82, 'P': 1.41}
- **2D identity (%) [PDB]:** 32.6
- **2D identity (%) [Gaps excluded][PDB]:** 85.28
- **2D identity - Alignment Gaps [PDB]:** 1065
- **2D aligned content [PDB] (<2D-fold>:%):** {'.': 17.79, 'E': 35.77, 'T': 14.23, 'H': 30.43, 'G': 1.6, 'B': 0.18}
- **3D similarity (TM-Score) (%) [PDB]:** 25.1

- **Gene name:** VWF
- **Entrez ID:** N/A
- **RefSeq ID:** NM\_000552
- **Transcript sequence length:** 8830
- **5-UTR|CDS|3-UTR identity (%):** 40.98 | 32.25 | 29.23
- **5-UTR|CDS|3-UTR identity (%) [Gaps excluded]:** 71.28 | 77.06 | 71.03
- **5-UTR|CDS|3-UTR identity [Alignment Gaps]:** 139 | 5028 | 153
- **5-UTR aligned content (<base>:%):** {'A': 23.88, 'C': 24.63, 'T': 23.88, 'G': 27.61}
- **CDS aligned content (<base>:%):** {'A': 27.4, 'T': 29.95, 'G': 20.84, 'C': 21.81}
- **3-UTR aligned content (<base>:%):** {'G': 34.21, 'T': 23.68, 'A': 18.42, 'C': 23.68}

**Uniprot Description:**  
  
 Important in the maintenance of hemostasis, it promotes adhesion of platelets to the sites of vascular injury by forming a molecular bridge between sub-endothelial collagen matrix and platelet-surface receptor complex GPIb-IX-V. Also acts as a chaperone for coagulation factor VIII, delivering it to the site of injury, stabilizing its heterodimeric structure and protecting it from premature clearance from plasma.   
  
Multimeric. Interacts with F8.   
  
 **Gene Ontology Information:**

Molecular Function

- ATP binding
- ATPase activity
- ATP-dependent DNA damage sensor activity
- mismatched DNA binding

Location

- mismatch repair complex

Biological process

- mismatch repair

---

99

- **Protein name:** DNA-directed RNA polymerase III subunit RPC4
- **Organism:** Homo sapiens
- **Uniprot Accession Number:** P05423
- **Protein sequence length:** 398 aa
- **1D identity (%):** 5.97
- **1D identity (%) [Gaps excluded]:** 22.7
- **1D identity - Alignment Gaps:** 975
- **1D aligned content (<aminoacid>:%):** {'N': 2.53, 'E': 8.86, 'P': 6.33, 'G': 15.19, 'R': 3.8, 'S': 3.8, 'L': 15.19, 'I': 2.53, 'V': 7.59, 'K': 8.86, 'D': 7.59, 'Q': 6.33, 'F': 1.27, 'A': 5.06, 'T': 3.8, 'C': 1.27}
- **Common reported functions (%):** 0.0
- **Common reported locations (%):** 0.0
- **Common reported processes (%):** 0.0

- **AF ID:** P05423
- **Chain:** A
- **Protein length:** 398 aa
- **Resolution:** N/A
- **b-phipsi:** 0.069115
- **w-rdist:** 0.275613
- **t-alpha:** 0.098093
- **Chemical similarity (Tanimoto Index) (%):** 82.6
- **1D identity (%) [PDB]:** 1.94
- **1D identity (%) [Gaps excluded][PDB]:** 63.41
- **1D identity - Alignment Gaps [PDB]:** 1299
- **1D aligned content [PDB] (<aminoacid>:%):** {'S': 7.69, 'L': 19.23, 'I': 7.69, 'R': 15.38, 'D': 7.69, 'P': 3.85, 'E': 7.69, 'A': 3.85, 'V': 7.69, 'Q': 11.54, 'G': 3.85, 'T': 3.85}
- **2D identity (%) [PDB]:** 19.91
- **2D identity (%) [Gaps excluded][PDB]:** 79.71
- **2D identity - Alignment Gaps [PDB]:** 829
- **2D aligned content [PDB] (<2D-fold>:%):** {'.': 54.09, 'T': 14.55, 'H': 6.82, 'G': 1.36, 'E': 23.18}
- **3D similarity (TM-Score) (%) [PDB]:** 11.14

- **Gene name:** POLR3D
- **Entrez ID:** 508049
- **RefSeq ID:** NM\_001722
- **Transcript sequence length:** 5336
- **5-UTR|CDS|3-UTR identity (%):** 10.28 | 18.36 | 5.14
- **5-UTR|CDS|3-UTR identity (%) [Gaps excluded]:** 74.36 | 73.41 | 92.51
- **5-UTR|CDS|3-UTR identity [Alignment Gaps]:** 243 | 3011 | 3858
- **5-UTR aligned content (<base>:%):** {'A': 13.79, 'C': 27.59, 'T': 20.69, 'G': 37.93}
- **CDS aligned content (<base>:%):** {'A': 32.7, 'T': 20.22, 'G': 23.88, 'C': 23.2}
- **3-UTR aligned content (<base>:%):** {'A': 41.43, 'T': 22.38, 'C': 15.24, 'G': 20.95}

**Uniprot Description:**  
  
 DNA-dependent RNA polymerase catalyzes the transcription of DNA into RNA using the four ribonucleoside triphosphates as substrates. Specific peripheric component of RNA polymerase III which synthesizes small RNAs, such as 5S rRNA and tRNAs. Plays a key role in sensing and limiting infection by intracellular bacteria and DNA viruses. Acts as nuclear and cytosolic DNA sensor involved in innate immune response. Can sense non-self dsDNA that serves as template for transcription into dsRNA. The non-self RNA polymerase III transcripts, such as Epstein-Barr virus-encoded RNAs (EBERs) induce type I interferon and NF- Kappa-B through the RIG-I pathway (By similarity).   
  
Component of the RNA polymerase III (Pol III) complex consisting of 17 subunits (By similarity). Interacts with POLR3E/RPC5.   
  
 **Gene Ontology Information:**

Molecular Function

- DNA binding

Location

- RNA polymerase III complex

Biological process

- defense response to virus
- innate immune response
- positive regulation of innate immune response
- positive regulation of interferon-beta production
- transcription by RNA polymerase III

---

100

- **Protein name:** Rho GTPase-activating protein 24
- **Organism:** Homo sapiens
- **Uniprot Accession Number:** Q8N264
- **Protein sequence length:** 748 aa
- **1D identity (%):** 10.63
- **1D identity (%) [Gaps excluded]:** 25.17
- **1D identity - Alignment Gaps:** 821
- **1D aligned content (<aminoacid>:%):** {'N': 6.62, 'E': 4.64, 'Q': 5.96, 'K': 6.62, 'G': 5.3, 'F': 7.95, 'R': 3.97, 'V': 6.62, 'Y': 2.65, 'P': 8.61, 'L': 10.6, 'A': 4.64, 'H': 0.66, 'S': 11.92, 'W': 0.66, 'C': 3.31, 'I': 2.65, 'D': 1.32, 'T': 4.64, 'M': 0.66}
- **Common reported functions (%):** 0.0
- **Common reported locations (%):** 0.0
- **Common reported processes (%):** 0.0

- **AF ID:** Q8N264
- **Chain:** A
- **Protein length:** 748 aa
- **Resolution:** N/A
- **b-phipsi:** 0.069007
- **w-rdist:** 0.280584
- **t-alpha:** 0.169562
- **Chemical similarity (Tanimoto Index) (%):** 84.13
- **1D identity (%) [PDB]:** 1.78
- **1D identity (%) [Gaps excluded][PDB]:** 71.43
- **1D identity - Alignment Gaps [PDB]:** 1647
- **1D aligned content [PDB] (<aminoacid>:%):** {'Y': 3.33, 'F': 6.67, 'A': 3.33, 'S': 13.33, 'T': 10.0, 'K': 6.67, 'I': 13.33, 'R': 3.33, 'G': 6.67, 'L': 10.0, 'D': 3.33, 'V': 6.67, 'N': 6.67, 'E': 3.33, 'Q': 3.33}
- **2D identity (%) [PDB]:** 19.56
- **2D identity (%) [Gaps excluded][PDB]:** 89.68
- **2D identity - Alignment Gaps [PDB]:** 1111
- **2D aligned content [PDB] (<2D-fold>:%):** {'.': 13.31, 'T': 11.15, 'E': 14.75, 'H': 59.71, 'G': 1.08}
- **3D similarity (TM-Score) (%) [PDB]:** 16.78

- **Gene name:** ARHGAP24
- **Entrez ID:** 83478
- **RefSeq ID:** N/A
- **Sequence length:** N/A
- **5-UTR|CDS|3-UTR identity (%):** N/A | N/A | N/A
- **5-UTR|CDS|3-UTR identity (%) [Gaps excluded]:** N/A | N/A | N/A
- **5-UTR|CDS|3-UTR identity [Alignment Gaps]:** N/A | N/A | N/A
- **5-UTR aligned content (<base>:%):** N/A
- **CDS aligned content (<base>:%):** N/A
- **3-UTR aligned content (<base>:%):** N/A

**Uniprot Description:**  
  
 Rho GTPase-activating protein involved in cell polarity, cell morphology and cytoskeletal organization. Acts as a GTPase activator for the Rac-type GTPase by converting it to an inactive GDP-bound state. Controls actin remodeling by inactivating Rac downstream of Rho leading to suppress leading edge protrusion and promotes cell retraction to achieve cellular polarity. Able to suppress RAC1 and CDC42 activity in vitro. Overexpression induces cell rounding with partial or complete disruption of actin stress fibers and formation of membrane ruffles, lamellipodia, and filopodia. Isoform 2 is a vascular cell-specific GAP involved in modulation of angiogenesis.   
  
Interacts with FLNA.   
  
 **Gene Ontology Information:**

Molecular Function

- GTPase activator activity

Location

- adherens junction
- cell projection
- cytoplasm
- cytoskeleton
- focal adhesion

Biological process

- activation of GTPase activity
- angiogenesis
- cell differentiation
- negative regulation of Rac protein signal transduction
- negative regulation of ruffle assembly
- signal transduction
- wound healing, spreading of epidermal cells

---
