# Supplementary material for: Identifying and profiling structural similarities between Spike of SARS-CoV-2 and other viral or host proteins with Machaon
Source: Commun Biol. 2023 Jul 19;6:752. doi: 10.1038/s42003-023-05076-7 (PMC10356814; doi:10.1038/s42003-023-05076-7)
Supplement: Supplementary file 9 — Supplementary Data 6 [file 42003_2023_5076_MOESM9_ESM.zip › 6VXX_A_whole_alphafold4_dataset/candidates/6VXX_A-merged-notenriched_report.html]

 

# Structural Comparison Report for 6VXX\_A - whole structures (total: 398)

---

1

- **AF ID:** AF-Q9BZ76-F1-model-v4 | **Chain:** A
- **b-phipsi:** 0.0128986881964576
- **w-rdist:** 0.2630641160311902
- **t-alpha:** 0.0033195156791741

---

---

2

- **PDB ID:** 6VXX | **Chain:** B
- **b-phipsi:** 5.894373919306382e-09
- **w-rdist:** 7.617386263618201e-05
- **t-alpha:** 0.0254452761871732

---

---

3

- **AF ID:** AF-P22897-F1-model-v4 | **Chain:** A
- **b-phipsi:** 0.0074002078230839
- **w-rdist:** 0.2318476018340813
- **t-alpha:** 0.0075000236060698

---

---

4

- **AF ID:** AF-P01266-F8-model-v4 | **Chain:** A
- **b-phipsi:** 0.0060370436228148
- **w-rdist:** 0.2588650225464293
- **t-alpha:** 0.0193931278866674

---

---

5

- **PDB ID:** 6VXX | **Chain:** C
- **b-phipsi:** 2.204205180302208e-08
- **w-rdist:** 7.653592189866652e-05
- **t-alpha:** 0.0689655430268945

---

---

6

- **AF ID:** AF-Q96NU0-F1-model-v4 | **Chain:** A
- **b-phipsi:** 0.0130645908653005
- **w-rdist:** 0.2735636332462324
- **t-alpha:** 0.0330849069147651

---

---

7

- **AF ID:** AF-Q9Y485-F4-model-v4 | **Chain:** A
- **b-phipsi:** 0.0092444607544551
- **w-rdist:** 0.3180826644451995
- **t-alpha:** 0.0280611759977658

---

---

8

- **AF ID:** AF-Q99575-F1-model-v4 | **Chain:** A
- **b-phipsi:** 0.0135148609119822
- **w-rdist:** 0.4765704272041375
- **t-alpha:** 0.0024811957952362

---

---

9

- **AF ID:** AF-P20742-F1-model-v4 | **Chain:** A
- **b-phipsi:** 0.0069266262025374
- **w-rdist:** 0.3028358782324738
- **t-alpha:** 0.077540150779898

---

---

10

- **AF ID:** AF-Q9Y485-F3-model-v4 | **Chain:** A
- **b-phipsi:** 0.010180955518257
- **w-rdist:** 0.6467256589500925
- **t-alpha:** 0.0016540840807526

---

---

11

- **AF ID:** AF-P17020-F1-model-v4 | **Chain:** A
- **b-phipsi:** 0.0283377408714307
- **w-rdist:** 0.3931799238931455
- **t-alpha:** 0.0008271775551371

---

---

12

- **AF ID:** AF-P08F94-F12-model-v4 | **Chain:** A
- **b-phipsi:** 0.0199741444969198
- **w-rdist:** 0.2669733435690185
- **t-alpha:** 0.0605264241229863

---

---

13

- **AF ID:** AF-A8K2U0-F1-model-v4 | **Chain:** A
- **b-phipsi:** 0.0107187282478843
- **w-rdist:** 0.3194601777679055
- **t-alpha:** 0.0504550203401277

---

---

14

- **AF ID:** AF-Q8NB90-F1-model-v4 | **Chain:** A
- **b-phipsi:** 0.0277978857313025
- **w-rdist:** 0.2426201923276998
- **t-alpha:** 0.0386600222545225

---

---

15

- **AF ID:** AF-O75592-F2-model-v4 | **Chain:** A
- **b-phipsi:** 0.0043859520417955
- **w-rdist:** 0.6292645654035877
- **t-alpha:** 0.0033195156791741

---

---

16

- **AF ID:** AF-Q8NI77-F1-model-v4 | **Chain:** A
- **b-phipsi:** 0.0258205362135034
- **w-rdist:** 0.4199841488961673
- **t-alpha:** 0.0024811957952362

---

---

17

- **AF ID:** AF-P01023-F1-model-v4 | **Chain:** A
- **b-phipsi:** 0.0089790634563145
- **w-rdist:** 0.2888758039135159
- **t-alpha:** 0.0990908911987924

---

---

18

- **AF ID:** AF-Q6PJI9-F1-model-v4 | **Chain:** A
- **b-phipsi:** 0.0073493809499895
- **w-rdist:** 0.7003569145929152
- **t-alpha:** 0.0024878881015579

---

---

19

- **AF ID:** AF-P12111-F1-model-v4 | **Chain:** A
- **b-phipsi:** 0.0167106615295809
- **w-rdist:** 0.7415731685352024
- **t-alpha:** 0.0

---

---

20

- **AF ID:** AF-O14727-F1-model-v4 | **Chain:** A
- **b-phipsi:** 0.008269411547982
- **w-rdist:** 0.9244646095723614
- **t-alpha:** 0.0008271775551371

---

---

21

- **AF ID:** AF-Q6YHK3-F1-model-v4 | **Chain:** A
- **b-phipsi:** 0.0111769060421033
- **w-rdist:** 0.2893456619410336
- **t-alpha:** 0.1051191692070554

---

---

22

- **AF ID:** AF-Q9NQ66-F1-model-v4 | **Chain:** A
- **b-phipsi:** 0.021894092499219
- **w-rdist:** 0.2933403203265313
- **t-alpha:** 0.0694789568304672

---

---

23

- **AF ID:** AF-Q9Y4D8-F8-model-v4 | **Chain:** A
- **b-phipsi:** 0.0161728030047133
- **w-rdist:** 0.2791306470394897
- **t-alpha:** 0.0931283345367748

---

---

24

- **AF ID:** AF-Q9UBG0-F1-model-v4 | **Chain:** A
- **b-phipsi:** 0.0088864282121855
- **w-rdist:** 0.6382517611760044
- **t-alpha:** 0.0041355535019986

---

---

25

- **AF ID:** AF-Q76MJ5-F1-model-v4 | **Chain:** A
- **b-phipsi:** 0.0093457400212479
- **w-rdist:** 0.9201996120726438
- **t-alpha:** 0.0008271775551371

---

---

26

- **AF ID:** AF-Q6ZRI0-F1-model-v4 | **Chain:** A
- **b-phipsi:** 0.0081128077876909
- **w-rdist:** 0.3419492517614985
- **t-alpha:** 0.068651600081727

---

---

27

- **AF ID:** AF-P63132-F1-model-v4 | **Chain:** A
- **b-phipsi:** 0.0098134070014597
- **w-rdist:** 0.4516810519195361
- **t-alpha:** 0.0057897637424946

---

---

28

- **AF ID:** AF-Q92598-F1-model-v4 | **Chain:** A
- **b-phipsi:** 0.009200338197898
- **w-rdist:** 0.7542687803720698
- **t-alpha:** 0.0024878881015579

---

---

29

- **AF ID:** AF-Q9H3P2-F1-model-v4 | **Chain:** A
- **b-phipsi:** 0.0347676334842491
- **w-rdist:** 0.5122998240292116
- **t-alpha:** 0.0008271775551371

---

---

30

- **AF ID:** AF-Q53GL0-F1-model-v4 | **Chain:** A
- **b-phipsi:** 0.0217367148005146
- **w-rdist:** 0.2806283953542755
- **t-alpha:** 0.0921410228992365

---

---

31

- **AF ID:** AF-Q9HDB9-F1-model-v4 | **Chain:** A
- **b-phipsi:** 0.036095432075401
- **w-rdist:** 0.3206520118890295
- **t-alpha:** 0.0058238157721357

---

---

32

- **AF ID:** AF-O00499-F1-model-v4 | **Chain:** A
- **b-phipsi:** 0.0358075354831282
- **w-rdist:** 0.4260374721491091
- **t-alpha:** 0.0008281320187666

---

---

33

- **AF ID:** AF-P08648-F1-model-v4 | **Chain:** A
- **b-phipsi:** 0.0247429531384978
- **w-rdist:** 0.313121848352471
- **t-alpha:** 0.0540540988034132

---

---

34

- **AF ID:** AF-Q15311-F1-model-v4 | **Chain:** A
- **b-phipsi:** 0.0357231537751166
- **w-rdist:** 0.4478029048804142
- **t-alpha:** 0.0008281320187666

---

---

35

- **AF ID:** AF-Q9NR09-F7-model-v4 | **Chain:** A
- **b-phipsi:** 0.0308871945124606
- **w-rdist:** 0.5567455779461193
- **t-alpha:** 0.0008281320187666

---

---

36

- **AF ID:** AF-P08F94-F11-model-v4 | **Chain:** A
- **b-phipsi:** 0.0215423535118639
- **w-rdist:** 0.3511072950040151
- **t-alpha:** 0.0159666566861913

---

---

37

- **AF ID:** AF-Q9UGP8-F1-model-v4 | **Chain:** A
- **b-phipsi:** 0.0352520650130213
- **w-rdist:** 0.3129500257038799
- **t-alpha:** 0.0083406172007201

---

---

38

- **AF ID:** AF-Q7Z5R6-F1-model-v4 | **Chain:** A
- **b-phipsi:** 0.01805598575724
- **w-rdist:** 0.703595477208572
- **t-alpha:** 0.0024811957952362

---

---

39

- **AF ID:** AF-P08F94-F10-model-v4 | **Chain:** A
- **b-phipsi:** 0.0240880708236146
- **w-rdist:** 0.3415212112411531
- **t-alpha:** 0.0263160129948751

---

---

40

- **AF ID:** AF-P01031-F1-model-v4 | **Chain:** A
- **b-phipsi:** 0.0076500121859307
- **w-rdist:** 0.3436953726557255
- **t-alpha:** 0.082363784576418

---

---

41

- **AF ID:** AF-Q06732-F1-model-v4 | **Chain:** A
- **b-phipsi:** 0.0273294382966873
- **w-rdist:** 0.6407243515539173
- **t-alpha:** 0.0016540840807526

---

---

42

- **AF ID:** AF-Q7Z5H3-F1-model-v4 | **Chain:** A
- **b-phipsi:** 0.0424970408772335
- **w-rdist:** 0.2612880979691155
- **t-alpha:** 0.0202533741750159

---

---

43

- **AF ID:** AF-P20701-F1-model-v4 | **Chain:** A
- **b-phipsi:** 0.007099131595458
- **w-rdist:** 0.4714976927266274
- **t-alpha:** 0.0066610907906692

---

---

44

- **AF ID:** AF-Q9Y4C8-F1-model-v4 | **Chain:** A
- **b-phipsi:** 0.0319781405929951
- **w-rdist:** 0.6239888173088579
- **t-alpha:** 0.0008271775551371

---

---

45

- **AF ID:** AF-P22607-F1-model-v4 | **Chain:** A
- **b-phipsi:** 0.016824433019424
- **w-rdist:** 0.7657289250462624
- **t-alpha:** 0.0016569498401739

---

---

46

- **AF ID:** AF-Q6IE37-F1-model-v4 | **Chain:** A
- **b-phipsi:** 0.0095827417049574
- **w-rdist:** 0.3588675568726708
- **t-alpha:** 0.0531362262599823

---

---

47

- **AF ID:** AF-Q96K21-F1-model-v4 | **Chain:** A
- **b-phipsi:** 0.0246497061027511
- **w-rdist:** 0.5754919101682674
- **t-alpha:** 0.0033085120789013

---

---

48

- **AF ID:** AF-Q9Y4L1-F1-model-v4 | **Chain:** A
- **b-phipsi:** 0.0120041183031168
- **w-rdist:** 0.2928167007682592
- **t-alpha:** 0.1472291561109899

---

---

49

- **AF ID:** AF-Q8TDJ6-F4-model-v4 | **Chain:** A
- **b-phipsi:** 0.0111326467069971
- **w-rdist:** 0.3715768529734896
- **t-alpha:** 0.0271879789729934

---

---

50

- **AF ID:** AF-O95714-F16-model-v4 | **Chain:** A
- **b-phipsi:** 0.0087589972892165
- **w-rdist:** 0.3620938237740017
- **t-alpha:** 0.0645157779328724

---

---

51

- **AF ID:** AF-Q9NR09-F15-model-v4 | **Chain:** A
- **b-phipsi:** 0.0391335891046547
- **w-rdist:** 0.4701504458663506
- **t-alpha:** 0.0008281320187666

---

---

52

- **AF ID:** AF-P55884-F1-model-v4 | **Chain:** A
- **b-phipsi:** 0.0212348607081577
- **w-rdist:** 0.3172898775688429
- **t-alpha:** 0.083540124827504

---

---

53

- **AF ID:** AF-Q9UHD8-F1-model-v4 | **Chain:** A
- **b-phipsi:** 0.0463493245040567
- **w-rdist:** 0.4495919453976698
- **t-alpha:** 0.0

---

---

54

- **AF ID:** AF-O95714-F15-model-v4 | **Chain:** A
- **b-phipsi:** 0.0095668799946601
- **w-rdist:** 0.3858489610900717
- **t-alpha:** 0.0091819121329137

---

---

55

- **AF ID:** AF-O75976-F1-model-v4 | **Chain:** A
- **b-phipsi:** 0.0257594679110686
- **w-rdist:** 0.2938559201213908
- **t-alpha:** 0.0934656315674258

---

---

56

- **AF ID:** AF-Q13585-F1-model-v4 | **Chain:** A
- **b-phipsi:** 0.0470982411284897
- **w-rdist:** 0.2649874139927372
- **t-alpha:** 0.0237089470211593

---

---

57

- **AF ID:** AF-Q76KP1-F1-model-v4 | **Chain:** A
- **b-phipsi:** 0.0312639189606043
- **w-rdist:** 0.294876459620073
- **t-alpha:** 0.0678243742037618

---

---

58

- **AF ID:** AF-Q9HCU4-F6-model-v4 | **Chain:** A
- **b-phipsi:** 0.0085538699389184
- **w-rdist:** 0.7471471072443138
- **t-alpha:** 0.0049627974633414

---

---

59

- **AF ID:** AF-Q9Y6R7-F22-model-v4 | **Chain:** A
- **b-phipsi:** 0.0138684216446042
- **w-rdist:** 0.91543877882685
- **t-alpha:** 0.0024811957952362

---

---

60

- **AF ID:** AF-Q9Y485-F10-model-v4 | **Chain:** A
- **b-phipsi:** 0.0220783973454086
- **w-rdist:** 0.3071513840552214
- **t-alpha:** 0.1112133623477085

---

---

61

- **AF ID:** AF-O60486-F1-model-v4 | **Chain:** A
- **b-phipsi:** 0.0049767053005447
- **w-rdist:** 0.606514777678621
- **t-alpha:** 0.0066610907906692

---

---

62

- **AF ID:** AF-O95071-F2-model-v4 | **Chain:** A
- **b-phipsi:** 0.0153010937588536
- **w-rdist:** 0.6395922852941501
- **t-alpha:** 0.0049627974633414

---

---

63

- **AF ID:** AF-Q6ZS81-F10-model-v4 | **Chain:** A
- **b-phipsi:** 0.0135581477895966
- **w-rdist:** 0.6158688209589748
- **t-alpha:** 0.0057897637424946

---

---

64

- **AF ID:** AF-P53708-F1-model-v4 | **Chain:** A
- **b-phipsi:** 0.025270374752751
- **w-rdist:** 0.3163175779027693
- **t-alpha:** 0.082363784576418

---

---

65

- **AF ID:** AF-A0A0G2JMS6-F1-model-v4 | **Chain:** A
- **b-phipsi:** 0.0143210586882208
- **w-rdist:** 0.3597615256225064
- **t-alpha:** 0.0628617784103293

---

---

66

- **AF ID:** AF-Q16832-F1-model-v4 | **Chain:** A
- **b-phipsi:** 0.0036218160494508
- **w-rdist:** 1.0668868694094509
- **t-alpha:** 0.0033085120789013

---

---

67

- **AF ID:** AF-Q9P2H5-F1-model-v4 | **Chain:** A
- **b-phipsi:** 0.0508859174903886
- **w-rdist:** 0.2564178591889064
- **t-alpha:** 0.0333332138395487

---

---

68

- **AF ID:** AF-Q9UJY5-F1-model-v4 | **Chain:** A
- **b-phipsi:** 0.0356440069852946
- **w-rdist:** 0.7241635650585256
- **t-alpha:** 0.0

---

---

69

- **AF ID:** AF-P43354-F1-model-v4 | **Chain:** A
- **b-phipsi:** 0.054018607368821
- **w-rdist:** 0.4668956484917175
- **t-alpha:** 0.0

---

---

70

- **AF ID:** AF-Q86WI1-F15-model-v4 | **Chain:** A
- **b-phipsi:** 0.0151585034922566
- **w-rdist:** 0.3305520820611856
- **t-alpha:** 0.1132600047230165

---

---

71

- **AF ID:** AF-P15918-F1-model-v4 | **Chain:** A
- **b-phipsi:** 0.0245165456239836
- **w-rdist:** 0.895356353522929
- **t-alpha:** 0.0016540840807526

---

---

72

- **AF ID:** AF-Q6U841-F1-model-v4 | **Chain:** A
- **b-phipsi:** 0.0275278871116634
- **w-rdist:** 0.3466219420455617
- **t-alpha:** 0.0446646519250406

---

---

73

- **AF ID:** AF-O43296-F1-model-v4 | **Chain:** A
- **b-phipsi:** 0.0275927455195377
- **w-rdist:** 0.6168777796354625
- **t-alpha:** 0.0041355535019986

---

---

74

- **AF ID:** AF-Q92736-F7-model-v4 | **Chain:** A
- **b-phipsi:** 0.0294981585080987
- **w-rdist:** 0.828324955243523
- **t-alpha:** 0.0008271775551371

---

---

75

- **AF ID:** AF-Q8IZQ1-F3-model-v4 | **Chain:** A
- **b-phipsi:** 0.0359349446816221
- **w-rdist:** 0.6620910103038253
- **t-alpha:** 0.0008281320187666

---

---

76

- **AF ID:** AF-Q8TC27-F1-model-v4 | **Chain:** A
- **b-phipsi:** 0.0257220939782292
- **w-rdist:** 0.7331834048683609
- **t-alpha:** 0.0033085120789013

---

---

77

- **AF ID:** AF-Q15751-F1-model-v4 | **Chain:** A
- **b-phipsi:** 0.0365055450984083
- **w-rdist:** 0.2869518259129966
- **t-alpha:** 0.076922726695275

---

---

78

- **AF ID:** AF-Q96PQ6-F1-model-v4 | **Chain:** A
- **b-phipsi:** 0.0174451133186787
- **w-rdist:** 0.3539474113832183
- **t-alpha:** 0.0777503200474987

---

---

79

- **AF ID:** AF-Q15928-F1-model-v4 | **Chain:** A
- **b-phipsi:** 0.034463856665786
- **w-rdist:** 0.6717267913583621
- **t-alpha:** 0.0016569498401739

---

---

80

- **AF ID:** AF-Q9Y2L9-F1-model-v4 | **Chain:** A
- **b-phipsi:** 0.0249399678441987
- **w-rdist:** 0.6049658536599348
- **t-alpha:** 0.0049877163597098

---

---

81

- **AF ID:** AF-Q6ZN44-F1-model-v4 | **Chain:** A
- **b-phipsi:** 0.0255064340221229
- **w-rdist:** 0.3262368099064757
- **t-alpha:** 0.0951197353930601

---

---

82

- **AF ID:** AF-Q8NEG5-F1-model-v4 | **Chain:** A
- **b-phipsi:** 0.0416148228295413
- **w-rdist:** 0.5566117069508817
- **t-alpha:** 0.0024811957952362

---

---

83

- **AF ID:** AF-Q8WXG9-F13-model-v4 | **Chain:** A
- **b-phipsi:** 0.0224779590496765
- **w-rdist:** 1.2659190224483043
- **t-alpha:** 0.0

---

---

84

- **AF ID:** AF-Q86YV0-F1-model-v4 | **Chain:** A
- **b-phipsi:** 0.0218392785887321
- **w-rdist:** 0.4794925365188709
- **t-alpha:** 0.0066610907906692

---

---

85

- **AF ID:** AF-Q9H6S3-F1-model-v4 | **Chain:** A
- **b-phipsi:** 0.0161573699047697
- **w-rdist:** 0.9608226806443692
- **t-alpha:** 0.0024878881015579

---

---

86

- **AF ID:** AF-O75592-F3-model-v4 | **Chain:** A
- **b-phipsi:** 0.003705236811139
- **w-rdist:** 0.6573592094022884
- **t-alpha:** 0.0165423471063261

---

---

87

- **AF ID:** AF-P24043-F10-model-v4 | **Chain:** A
- **b-phipsi:** 0.002912180216064
- **w-rdist:** 0.4330939207329795
- **t-alpha:** 0.0736146078897646

---

---

88

- **AF ID:** AF-P26012-F1-model-v4 | **Chain:** A
- **b-phipsi:** 0.0137547976985173
- **w-rdist:** 0.3152037144095001
- **t-alpha:** 0.2077922859940879

---

---

89

- **AF ID:** AF-Q9Y5B9-F1-model-v4 | **Chain:** A
- **b-phipsi:** 0.0077721852007063
- **w-rdist:** 0.361745273608913
- **t-alpha:** 0.1194444620618173

---

---

90

- **AF ID:** AF-Q6P996-F1-model-v4 | **Chain:** A
- **b-phipsi:** 0.032127934943809
- **w-rdist:** 0.6693616315650265
- **t-alpha:** 0.0024878881015579

---

---

91

- **AF ID:** AF-Q9HCG1-F1-model-v4 | **Chain:** A
- **b-phipsi:** 0.0228619234662153
- **w-rdist:** 1.0836160318288428
- **t-alpha:** 0.0008281320187666

---

---

92

- **AF ID:** AF-Q4ADV7-F1-model-v4 | **Chain:** A
- **b-phipsi:** 0.0146440757556432
- **w-rdist:** 0.819849755601865
- **t-alpha:** 0.0041527907791232

---

---

93

- **AF ID:** AF-P0CJ89-F1-model-v4 | **Chain:** A
- **b-phipsi:** 0.0313542508214103
- **w-rdist:** 0.7695094922800007
- **t-alpha:** 0.0016569498401739

---

---

94

- **AF ID:** AF-Q7L2R6-F1-model-v4 | **Chain:** A
- **b-phipsi:** 0.0322750882707468
- **w-rdist:** 0.8673642135326054
- **t-alpha:** 0.0008271775551371

---

---

95

- **AF ID:** AF-O75030-F1-model-v4 | **Chain:** A
- **b-phipsi:** 0.0366492172098593
- **w-rdist:** 0.7962808474677763
- **t-alpha:** 0.0

---

---

96

- **AF ID:** AF-Q8NFF5-F1-model-v4 | **Chain:** A
- **b-phipsi:** 0.0156339556084307
- **w-rdist:** 1.199007094590475
- **t-alpha:** 0.0024811957952362

---

---

97

- **AF ID:** AF-P14410-F1-model-v4 | **Chain:** A
- **b-phipsi:** 0.0015596343554912
- **w-rdist:** 0.5206094769287
- **t-alpha:** 0.0727871210352775

---

---

98

- **AF ID:** AF-Q9NPF5-F1-model-v4 | **Chain:** A
- **b-phipsi:** 0.0287881544091671
- **w-rdist:** 0.3299583787406172
- **t-alpha:** 0.0901716732019928

---

---

99

- **AF ID:** AF-Q86UV5-F1-model-v4 | **Chain:** A
- **b-phipsi:** 0.0193396167880061
- **w-rdist:** 0.5920566506115279
- **t-alpha:** 0.0066610907906692

---

---

100

- **AF ID:** AF-P0CJ85-F1-model-v4 | **Chain:** A
- **b-phipsi:** 0.0440100656056751
- **w-rdist:** 0.5650982099289734
- **t-alpha:** 0.0024878881015579

---

---

101

- **AF ID:** AF-Q16625-F1-model-v4 | **Chain:** A
- **b-phipsi:** 0.0493115345354437
- **w-rdist:** 0.5253655975264151
- **t-alpha:** 0.0024811957952362

---

---

102

- **AF ID:** AF-Q99593-F1-model-v4 | **Chain:** A
- **b-phipsi:** 0.0872159701663811
- **w-rdist:** 0.3368265440860529
- **t-alpha:** 0.0024811957952362

---

---

103

- **AF ID:** AF-Q9Y692-F1-model-v4 | **Chain:** A
- **b-phipsi:** 0.0408797563417883
- **w-rdist:** 0.3476959471543356
- **t-alpha:** 0.0132341605941959

---

---

104

- **AF ID:** AF-Q9H4H8-F1-model-v4 | **Chain:** A
- **b-phipsi:** 0.0150533642264815
- **w-rdist:** 0.772763060682599
- **t-alpha:** 0.0057897637424946

---

---

105

- **AF ID:** AF-Q6ZNJ1-F7-model-v4 | **Chain:** A
- **b-phipsi:** 0.0275904304880709
- **w-rdist:** 1.2244341537862955
- **t-alpha:** 0.0

---

---

106

- **AF ID:** AF-Q86V59-F1-model-v4 | **Chain:** A
- **b-phipsi:** 0.054676724680589
- **w-rdist:** 0.3090528027889282
- **t-alpha:** 0.0272952510798727

---

---

107

- **AF ID:** AF-O60353-F1-model-v4 | **Chain:** A
- **b-phipsi:** 0.0351503610723197
- **w-rdist:** 0.3365316949648148
- **t-alpha:** 0.066137781369111

---

---

108

- **AF ID:** AF-Q9NRL3-F1-model-v4 | **Chain:** A
- **b-phipsi:** 0.0072177300147438
- **w-rdist:** 0.8691180653517001
- **t-alpha:** 0.0066169305252397

---

---

109

- **AF ID:** AF-Q9NT68-F7-model-v4 | **Chain:** A
- **b-phipsi:** 0.0083762062763749
- **w-rdist:** 0.7644682916847294
- **t-alpha:** 0.0074438168005386

---

---

110

- **AF ID:** AF-Q6IE36-F1-model-v4 | **Chain:** A
- **b-phipsi:** 0.013966124207162
- **w-rdist:** 0.3858501860792445
- **t-alpha:** 0.0678243742037618

---

---

111

- **AF ID:** AF-Q53LP3-F1-model-v4 | **Chain:** A
- **b-phipsi:** 0.0863312878724029
- **w-rdist:** 0.2898271895063253
- **t-alpha:** 0.0049877163597098

---

---

112

- **AF ID:** AF-Q8TDJ6-F2-model-v4 | **Chain:** A
- **b-phipsi:** 0.0291578798775404
- **w-rdist:** 0.4984439427103395
- **t-alpha:** 0.0066169305252397

---

---

113

- **AF ID:** AF-O43309-F1-model-v4 | **Chain:** A
- **b-phipsi:** 0.0310553317318161
- **w-rdist:** 0.3804856108693507
- **t-alpha:** 0.0091819121329137

---

---

114

- **AF ID:** AF-Q0VGE8-F1-model-v4 | **Chain:** A
- **b-phipsi:** 0.0207753193512944
- **w-rdist:** 0.3526071117434504
- **t-alpha:** 0.117375261057294

---

---

115

- **AF ID:** AF-P08621-F1-model-v4 | **Chain:** A
- **b-phipsi:** 0.0502430067883358
- **w-rdist:** 0.2716418385391683
- **t-alpha:** 0.0813954771139466

---

---

116

- **AF ID:** AF-P32314-F1-model-v4 | **Chain:** A
- **b-phipsi:** 0.0505783658388147
- **w-rdist:** 0.4835367398638488
- **t-alpha:** 0.0033085120789013

---

---

117

- **AF ID:** AF-Q8N184-F1-model-v4 | **Chain:** A
- **b-phipsi:** 0.0313187344326802
- **w-rdist:** 0.3694213695578927
- **t-alpha:** 0.034739440546361

---

---

118

- **AF ID:** AF-Q96M86-F8-model-v4 | **Chain:** A
- **b-phipsi:** 0.040138825820451
- **w-rdist:** 0.3040073444671038
- **t-alpha:** 0.0941175821499755

---

---

119

- **AF ID:** AF-Q6ZWH5-F1-model-v4 | **Chain:** A
- **b-phipsi:** 0.0435799667271972
- **w-rdist:** 0.324967205266481
- **t-alpha:** 0.0595534013336387

---

---

120

- **AF ID:** AF-Q6ZR08-F8-model-v4 | **Chain:** A
- **b-phipsi:** 0.066479305273992
- **w-rdist:** 0.4905553215743937
- **t-alpha:** 0.0008271775551371

---

---

121

- **AF ID:** AF-P56730-F1-model-v4 | **Chain:** A
- **b-phipsi:** 0.0288357434130113
- **w-rdist:** 0.700722494290007
- **t-alpha:** 0.0049627974633414

---

---

122

- **AF ID:** AF-P78509-F3-model-v4 | **Chain:** A
- **b-phipsi:** 0.0292964120721646
- **w-rdist:** 0.9581280163728376
- **t-alpha:** 0.0024811957952362

---

---

123

- **AF ID:** AF-Q99835-F1-model-v4 | **Chain:** A
- **b-phipsi:** 0.0300621009782494
- **w-rdist:** 0.3823259161485555
- **t-alpha:** 0.0185340926063368

---

---

124

- **AF ID:** AF-P25391-F8-model-v4 | **Chain:** A
- **b-phipsi:** 0.0088080116407543
- **w-rdist:** 1.2981576292715162
- **t-alpha:** 0.0041527907791232

---

---

125

- **AF ID:** AF-Q5T4S7-F6-model-v4 | **Chain:** A
- **b-phipsi:** 0.0188451579303458
- **w-rdist:** 0.8329892273683652
- **t-alpha:** 0.0049877163597098

---

---

126

- **AF ID:** AF-P01024-F1-model-v4 | **Chain:** A
- **b-phipsi:** 0.0035767644886528
- **w-rdist:** 0.4442821330229915
- **t-alpha:** 0.1132600047230165

---

---

127

- **AF ID:** AF-O96013-F1-model-v4 | **Chain:** A
- **b-phipsi:** 0.0388072829456872
- **w-rdist:** 0.9065908264025212
- **t-alpha:** 0.0008271775551371

---

---

128

- **AF ID:** AF-Q8TDW7-F14-model-v4 | **Chain:** A
- **b-phipsi:** 0.0243298020528436
- **w-rdist:** 1.3467344419132676
- **t-alpha:** 0.0016540840807526

---

---

129

- **AF ID:** AF-P35606-F1-model-v4 | **Chain:** A
- **b-phipsi:** 0.0035833563875058
- **w-rdist:** 0.6519663339986222
- **t-alpha:** 0.0651982118967349

---

---

130

- **AF ID:** AF-P11362-F1-model-v4 | **Chain:** A
- **b-phipsi:** 0.0447557381597913
- **w-rdist:** 0.2397668180838327
- **t-alpha:** 0.1267475121071146

---

---

131

- **AF ID:** AF-Q96RN1-F1-model-v4 | **Chain:** A
- **b-phipsi:** 0.0258969775657145
- **w-rdist:** 0.3686348372400443
- **t-alpha:** 0.0760957688434487

---

---

132

- **AF ID:** AF-Q8IZ52-F1-model-v4 | **Chain:** A
- **b-phipsi:** 0.0243023679143861
- **w-rdist:** 0.8722049906925802
- **t-alpha:** 0.0041527907791232

---

---

133

- **AF ID:** AF-Q12849-F1-model-v4 | **Chain:** A
- **b-phipsi:** 0.0398137474836367
- **w-rdist:** 0.8230993755635947
- **t-alpha:** 0.0008281320187666

---

---

134

- **AF ID:** AF-P08514-F1-model-v4 | **Chain:** A
- **b-phipsi:** 0.0259407031305296
- **w-rdist:** 0.3667757398931032
- **t-alpha:** 0.0794645485926308

---

---

135

- **AF ID:** AF-Q99457-F1-model-v4 | **Chain:** A
- **b-phipsi:** 0.0363276601256349
- **w-rdist:** 0.6265768452227467
- **t-alpha:** 0.0041527907791232

---

---

136

- **AF ID:** AF-Q5TKA1-F1-model-v4 | **Chain:** A
- **b-phipsi:** 0.0291782028939856
- **w-rdist:** 0.9341860318782028
- **t-alpha:** 0.0024878881015579

---

---

137

- **AF ID:** AF-Q8NI36-F1-model-v4 | **Chain:** A
- **b-phipsi:** 0.0019992048879244
- **w-rdist:** 0.5082195573869013
- **t-alpha:** 0.1081575850073603

---

---

138

- **AF ID:** AF-Q8N9M1-F1-model-v4 | **Chain:** A
- **b-phipsi:** 0.0634959938237104
- **w-rdist:** 0.5697028917946396
- **t-alpha:** 0.0008271775551371

---

---

139

- **AF ID:** AF-Q96PU4-F1-model-v4 | **Chain:** A
- **b-phipsi:** 0.0126537034357605
- **w-rdist:** 1.063534947503714
- **t-alpha:** 0.0049877163597098

---

---

140

- **AF ID:** AF-P09874-F1-model-v4 | **Chain:** A
- **b-phipsi:** 0.0099940048936372
- **w-rdist:** 1.0436875193239026
- **t-alpha:** 0.0057897637424946

---

---

141

- **AF ID:** AF-P04275-F3-model-v4 | **Chain:** A
- **b-phipsi:** 0.0044143518596265
- **w-rdist:** 0.7823021407129234
- **t-alpha:** 0.0322581402352546

---

---

142

- **AF ID:** AF-A6NHJ4-F1-model-v4 | **Chain:** A
- **b-phipsi:** 0.0302658335709262
- **w-rdist:** 0.567093609805526
- **t-alpha:** 0.0066169305252397

---

---

143

- **AF ID:** AF-Q04206-F1-model-v4 | **Chain:** A
- **b-phipsi:** 0.0337186347974787
- **w-rdist:** 0.6557727613079884
- **t-alpha:** 0.0049627974633414

---

---

144

- **AF ID:** AF-Q86WI1-F1-model-v4 | **Chain:** A
- **b-phipsi:** 0.0154271806381127
- **w-rdist:** 1.1166159443786716
- **t-alpha:** 0.0041527907791232

---

---

145

- **AF ID:** AF-Q9H6R4-F1-model-v4 | **Chain:** A
- **b-phipsi:** 0.0279804202173802
- **w-rdist:** 0.905970785703324
- **t-alpha:** 0.0033195156791741

---

---

146

- **AF ID:** AF-Q96GD3-F1-model-v4 | **Chain:** A
- **b-phipsi:** 0.0637663731257583
- **w-rdist:** 0.5170208701383436
- **t-alpha:** 0.0016540840807526

---

---

147

- **AF ID:** AF-Q96CP6-F1-model-v4 | **Chain:** A
- **b-phipsi:** 0.0366884076680553
- **w-rdist:** 0.358498679569155
- **t-alpha:** 0.0471463143139867

---

---

148

- **AF ID:** AF-Q9UQB8-F1-model-v4 | **Chain:** A
- **b-phipsi:** 0.0337502468977118
- **w-rdist:** 0.3792429343696762
- **t-alpha:** 0.0223323354003182

---

---

149

- **AF ID:** AF-O75925-F1-model-v4 | **Chain:** A
- **b-phipsi:** 0.0534164656822248
- **w-rdist:** 0.3540805703936062
- **t-alpha:** 0.0066610907906692

---

---

150

- **AF ID:** AF-Q9HBA0-F1-model-v4 | **Chain:** A
- **b-phipsi:** 0.0484055202874076
- **w-rdist:** 0.2574873198597717
- **t-alpha:** 0.117375261057294

---

---

151

- **AF ID:** AF-Q8TD17-F1-model-v4 | **Chain:** A
- **b-phipsi:** 0.0311374679801386
- **w-rdist:** 0.3779476051227446
- **t-alpha:** 0.039552885392045

---

---

152

- **AF ID:** AF-O14936-F1-model-v4 | **Chain:** A
- **b-phipsi:** 0.0130774234810325
- **w-rdist:** 0.9384684349813726
- **t-alpha:** 0.0058238157721357

---

---

153

- **AF ID:** AF-P98164-F8-model-v4 | **Chain:** A
- **b-phipsi:** 0.0146424077273261
- **w-rdist:** 0.3708829192899338
- **t-alpha:** 0.1267475121071146

---

---

154

- **AF ID:** AF-O95714-F19-model-v4 | **Chain:** A
- **b-phipsi:** 0.0116632708865024
- **w-rdist:** 0.9594588187596732
- **t-alpha:** 0.0058238157721357

---

---

155

- **AF ID:** AF-Q9H7R0-F1-model-v4 | **Chain:** A
- **b-phipsi:** 0.0241083839209943
- **w-rdist:** 0.3727808620813482
- **t-alpha:** 0.084367115769673

---

---

156

- **AF ID:** AF-Q9Y6R7-F6-model-v4 | **Chain:** A
- **b-phipsi:** 0.010774405740333
- **w-rdist:** 0.3732691593037676
- **t-alpha:** 0.1536259748810304

---

---

157

- **AF ID:** AF-Q6ZR08-F10-model-v4 | **Chain:** A
- **b-phipsi:** 0.0518692747750412
- **w-rdist:** 0.3039579539989679
- **t-alpha:** 0.0813954771139466

---

---

158

- **AF ID:** AF-P29317-F1-model-v4 | **Chain:** A
- **b-phipsi:** 0.0037587597302468
- **w-rdist:** 1.2645346999041032
- **t-alpha:** 0.0058238157721357

---

---

159

- **AF ID:** AF-Q8IZQ1-F5-model-v4 | **Chain:** A
- **b-phipsi:** 0.0277877633063367
- **w-rdist:** 1.1145383567883185
- **t-alpha:** 0.0024878881015579

---

---

160

- **AF ID:** AF-Q02846-F1-model-v4 | **Chain:** A
- **b-phipsi:** 0.0233295773582529
- **w-rdist:** 0.7920170805111694
- **t-alpha:** 0.0058238157721357

---

---

161

- **AF ID:** AF-Q8TBC5-F1-model-v4 | **Chain:** A
- **b-phipsi:** 0.0811766318298614
- **w-rdist:** 0.2638542802400018
- **t-alpha:** 0.0168211022886111

---

---

162

- **AF ID:** AF-O75022-F1-model-v4 | **Chain:** A
- **b-phipsi:** 0.0323706939563352
- **w-rdist:** 0.6167836600818378
- **t-alpha:** 0.0058238157721357

---

---

163

- **AF ID:** AF-Q9P2D7-F9-model-v4 | **Chain:** A
- **b-phipsi:** 0.0628853288558954
- **w-rdist:** 0.2814371848711425
- **t-alpha:** 0.0651982118967349

---

---

164

- **AF ID:** AF-Q16666-F1-model-v4 | **Chain:** A
- **b-phipsi:** 0.0532182185471713
- **w-rdist:** 0.3347169716213123
- **t-alpha:** 0.045491883252869

---

---

165

- **AF ID:** AF-O43149-F2-model-v4 | **Chain:** A
- **b-phipsi:** 0.0274253736436436
- **w-rdist:** 0.3857842660015048
- **t-alpha:** 0.0545905224299128

---

---

166

- **AF ID:** AF-Q9UK13-F1-model-v4 | **Chain:** A
- **b-phipsi:** 0.0375635000673447
- **w-rdist:** 0.3186931473965754
- **t-alpha:** 0.1163436922189591

---

---

167

- **AF ID:** AF-Q6NX49-F1-model-v4 | **Chain:** A
- **b-phipsi:** 0.0404477802624453
- **w-rdist:** 0.3144045963974531
- **t-alpha:** 0.1166253219249171

---

---

168

- **AF ID:** AF-P0C0L5-F1-model-v4 | **Chain:** A
- **b-phipsi:** 0.0012404587503702
- **w-rdist:** 0.9058690347437244
- **t-alpha:** 0.0438375773921617

---

---

169

- **AF ID:** AF-Q06278-F1-model-v4 | **Chain:** A
- **b-phipsi:** 0.0091426516543397
- **w-rdist:** 1.1717934316445282
- **t-alpha:** 0.0058238157721357

---

---

170

- **AF ID:** AF-Q13075-F1-model-v4 | **Chain:** A
- **b-phipsi:** 0.0480467225971429
- **w-rdist:** 0.3654909793492327
- **t-alpha:** 0.0142618534263445

---

---

171

- **AF ID:** AF-Q6N022-F4-model-v4 | **Chain:** A
- **b-phipsi:** 0.0148170467350264
- **w-rdist:** 0.8317488594957352
- **t-alpha:** 0.0066610907906692

---

---

172

- **AF ID:** AF-O95049-F1-model-v4 | **Chain:** A
- **b-phipsi:** 0.0306899523514365
- **w-rdist:** 0.3098515894464984
- **t-alpha:** 0.2415221382376902

---

---

173

- **AF ID:** AF-Q9Y4X4-F1-model-v4 | **Chain:** A
- **b-phipsi:** 0.0531636172044943
- **w-rdist:** 0.768707365951864
- **t-alpha:** 0.0008281320187666

---

---

174

- **AF ID:** AF-Q8NER1-F1-model-v4 | **Chain:** A
- **b-phipsi:** 0.0457588709127311
- **w-rdist:** 0.2321422749274005
- **t-alpha:** 0.1795123039771107

---

---

175

- **AF ID:** AF-Q9P2D7-F6-model-v4 | **Chain:** A
- **b-phipsi:** 0.0683985969588791
- **w-rdist:** 0.2972202106177342
- **t-alpha:** 0.0422415493952179

---

---

176

- **AF ID:** AF-P0C0L4-F1-model-v4 | **Chain:** A
- **b-phipsi:** 0.0011838928030006
- **w-rdist:** 0.9131842813683676
- **t-alpha:** 0.0494792275614968

---

---

177

- **AF ID:** AF-Q9Y4D8-F1-model-v4 | **Chain:** A
- **b-phipsi:** 0.0478722924375902
- **w-rdist:** 0.3441732491136102
- **t-alpha:** 0.0537631265310847

---

---

178

- **AF ID:** AF-Q6P5Q4-F1-model-v4 | **Chain:** A
- **b-phipsi:** 0.0460263155544669
- **w-rdist:** 0.6704759147460249
- **t-alpha:** 0.0033195156791741

---

---

179

- **AF ID:** AF-A2A3N6-F1-model-v4 | **Chain:** A
- **b-phipsi:** 0.0263025178080162
- **w-rdist:** 0.8309159257676184
- **t-alpha:** 0.0057897637424946

---

---

180

- **AF ID:** AF-Q9UQQ2-F1-model-v4 | **Chain:** A
- **b-phipsi:** 0.0480071177633897
- **w-rdist:** 0.3071300590196318
- **t-alpha:** 0.1091741848281244

---

---

181

- **AF ID:** AF-Q8WXR4-F1-model-v4 | **Chain:** A
- **b-phipsi:** 0.02406992349948
- **w-rdist:** 0.377978698744832
- **t-alpha:** 0.1058724543546798

---

---

182

- **AF ID:** AF-Q6PG37-F1-model-v4 | **Chain:** A
- **b-phipsi:** 0.0427217006324674
- **w-rdist:** 0.3666096590267295
- **t-alpha:** 0.0413563080873329

---

---

183

- **AF ID:** AF-Q96JS3-F1-model-v4 | **Chain:** A
- **b-phipsi:** 0.0141144562780006
- **w-rdist:** 0.928432265163665
- **t-alpha:** 0.0066610907906692

---

---

184

- **AF ID:** AF-Q86UW9-F1-model-v4 | **Chain:** A
- **b-phipsi:** 0.0288051065009446
- **w-rdist:** 0.9839059286779438
- **t-alpha:** 0.0041355535019986

---

---

185

- **AF ID:** AF-Q02386-F1-model-v4 | **Chain:** A
- **b-phipsi:** 0.0321396794089021
- **w-rdist:** 0.3169096667263927
- **t-alpha:** 0.212637982875945

---

---

186

- **AF ID:** AF-Q92736-F2-model-v4 | **Chain:** A
- **b-phipsi:** 0.0030866165420115
- **w-rdist:** 0.7268633750598787
- **t-alpha:** 0.0885027242012517

---

---

187

- **AF ID:** AF-Q2M3X9-F1-model-v4 | **Chain:** A
- **b-phipsi:** 0.0354436259914386
- **w-rdist:** 0.3178998232987378
- **t-alpha:** 0.1772153079970764

---

---

188

- **AF ID:** AF-O43897-F1-model-v4 | **Chain:** A
- **b-phipsi:** 0.0271905208995204
- **w-rdist:** 0.3798754883559828
- **t-alpha:** 0.0862534621133408

---

---

189

- **AF ID:** AF-Q63HM2-F1-model-v4 | **Chain:** A
- **b-phipsi:** 0.0562098962799436
- **w-rdist:** 0.8024185667806379
- **t-alpha:** 0.0008271775551371

---

---

190

- **AF ID:** AF-Q8TE73-F9-model-v4 | **Chain:** A
- **b-phipsi:** 0.0553659662361326
- **w-rdist:** 0.5521416173389201
- **t-alpha:** 0.0041355535019986

---

---

191

- **AF ID:** AF-Q8IVF4-F10-model-v4 | **Chain:** A
- **b-phipsi:** 0.0624956619943081
- **w-rdist:** 0.3195011342455353
- **t-alpha:** 0.0503909076104127

---

---

192

- **AF ID:** AF-Q63HN8-F13-model-v4 | **Chain:** A
- **b-phipsi:** 0.0451724717769773
- **w-rdist:** 0.793678586959579
- **t-alpha:** 0.0033085120789013

---

---

193

- **AF ID:** AF-Q8NB14-F1-model-v4 | **Chain:** A
- **b-phipsi:** 0.0363901454956394
- **w-rdist:** 0.3420590184688334
- **t-alpha:** 0.1149711661434758

---

---

194

- **AF ID:** AF-Q16787-F3-model-v4 | **Chain:** A
- **b-phipsi:** 0.0224571134139416
- **w-rdist:** 0.3740132850283472
- **t-alpha:** 0.1348220006399627

---

---

195

- **AF ID:** AF-Q08211-F1-model-v4 | **Chain:** A
- **b-phipsi:** 0.0159111680038275
- **w-rdist:** 0.9565716414514622
- **t-alpha:** 0.0066610907906692

---

---

196

- **AF ID:** AF-Q9NZV8-F1-model-v4 | **Chain:** A
- **b-phipsi:** 0.0697957779915078
- **w-rdist:** 0.2977417016613315
- **t-alpha:** 0.0531362262599823

---

---

197

- **AF ID:** AF-Q8IY82-F1-model-v4 | **Chain:** A
- **b-phipsi:** 0.045583859561719
- **w-rdist:** 0.3074639187846928
- **t-alpha:** 0.1569378712838169

---

---

198

- **AF ID:** AF-Q86VF2-F1-model-v4 | **Chain:** A
- **b-phipsi:** 0.0272583903531706
- **w-rdist:** 1.2195532435698315
- **t-alpha:** 0.0041355535019986

---

---

199

- **AF ID:** AF-Q9NR09-F9-model-v4 | **Chain:** A
- **b-phipsi:** 0.0450904460416208
- **w-rdist:** 0.9645995861205148
- **t-alpha:** 0.0016569498401739

---

---

200

- **AF ID:** AF-Q9NZJ4-F2-model-v4 | **Chain:** A
- **b-phipsi:** 0.0310401748168027
- **w-rdist:** 1.3132843946348685
- **t-alpha:** 0.0024878881015579

---

---

201

- **AF ID:** AF-O43149-F9-model-v4 | **Chain:** A
- **b-phipsi:** 0.0406147292611492
- **w-rdist:** 0.3443794259416308
- **t-alpha:** 0.0959466802142319

---

---

202

- **AF ID:** AF-Q70CQ2-F7-model-v4 | **Chain:** A
- **b-phipsi:** 0.0494713435575128
- **w-rdist:** 0.6288929564319865
- **t-alpha:** 0.0049627974633414

---

---

203

- **AF ID:** AF-O14503-F1-model-v4 | **Chain:** A
- **b-phipsi:** 0.0614395780714905
- **w-rdist:** 0.7486306697822966
- **t-alpha:** 0.0016540840807526

---

---

204

- **AF ID:** AF-Q9C0I9-F1-model-v4 | **Chain:** A
- **b-phipsi:** 0.0419818712716039
- **w-rdist:** 0.317385324705187
- **t-alpha:** 0.1669885884326432

---

---

205

- **AF ID:** AF-Q9BQG1-F1-model-v4 | **Chain:** A
- **b-phipsi:** 0.054724069166553
- **w-rdist:** 0.503035622006045
- **t-alpha:** 0.0049877163597098

---

---

206

- **AF ID:** AF-Q8N398-F1-model-v4 | **Chain:** A
- **b-phipsi:** 0.0300265087497315
- **w-rdist:** 0.3402846086915141
- **t-alpha:** 0.2109178108554219

---

---

207

- **AF ID:** AF-Q13202-F1-model-v4 | **Chain:** A
- **b-phipsi:** 0.0690777718146669
- **w-rdist:** 0.3919436417936494
- **t-alpha:** 0.0041355535019986

---

---

208

- **AF ID:** AF-Q9UN86-F1-model-v4 | **Chain:** A
- **b-phipsi:** 0.0951297413704216
- **w-rdist:** 0.2528484495984731
- **t-alpha:** 0.0368785736255408

---

---

209

- **AF ID:** AF-O43451-F1-model-v4 | **Chain:** A
- **b-phipsi:** 0.0015824492736229
- **w-rdist:** 0.7901280804608657
- **t-alpha:** 0.0951197353930601

---

---

210

- **AF ID:** AF-Q8N8U3-F1-model-v4 | **Chain:** A
- **b-phipsi:** 0.0375345831191947
- **w-rdist:** 0.3123436710348204
- **t-alpha:** 0.2399998462757295

---

---

211

- **AF ID:** AF-O60494-F2-model-v4 | **Chain:** A
- **b-phipsi:** 0.0323015402275729
- **w-rdist:** 1.209670303797573
- **t-alpha:** 0.0033085120789013

---

---

212

- **AF ID:** AF-A6PVC2-F1-model-v4 | **Chain:** A
- **b-phipsi:** 0.0225538489993672
- **w-rdist:** 0.902674678314001
- **t-alpha:** 0.0074438168005386

---

---

213

- **AF ID:** AF-Q96T49-F1-model-v4 | **Chain:** A
- **b-phipsi:** 0.0517103476178799
- **w-rdist:** 0.3074615943834919
- **t-alpha:** 0.1320224305126105

---

---

214

- **AF ID:** AF-A6NCL1-F1-model-v4 | **Chain:** A
- **b-phipsi:** 0.0752085182134985
- **w-rdist:** 0.6407060143043376
- **t-alpha:** 0.0008281320187666

---

---

215

- **AF ID:** AF-Q92560-F1-model-v4 | **Chain:** A
- **b-phipsi:** 0.0237023363775839
- **w-rdist:** 0.3820874760516023
- **t-alpha:** 0.1290321697790815

---

---

216

- **AF ID:** AF-Q16787-F11-model-v4 | **Chain:** A
- **b-phipsi:** 0.0027576352355688
- **w-rdist:** 0.7583220475748009
- **t-alpha:** 0.1112133623477085

---

---

217

- **AF ID:** AF-Q96M69-F1-model-v4 | **Chain:** A
- **b-phipsi:** 0.0332121705808368
- **w-rdist:** 0.3850428974358847
- **t-alpha:** 0.070305933400208

---

---

218

- **AF ID:** AF-Q2TAL5-F1-model-v4 | **Chain:** A
- **b-phipsi:** 0.0633592838404979
- **w-rdist:** 0.6122543247949392
- **t-alpha:** 0.0033195156791741

---

---

219

- **AF ID:** AF-Q15262-F1-model-v4 | **Chain:** A
- **b-phipsi:** 0.0018237618574584
- **w-rdist:** 1.3270282610547093
- **t-alpha:** 0.0297764065873182

---

---

220

- **AF ID:** AF-Q9HCR9-F1-model-v4 | **Chain:** A
- **b-phipsi:** 0.0547613951291841
- **w-rdist:** 0.6750551064268614
- **t-alpha:** 0.0033195156791741

---

---

221

- **AF ID:** AF-Q9UQ90-F1-model-v4 | **Chain:** A
- **b-phipsi:** 0.0337420186905668
- **w-rdist:** 0.7149094163609225
- **t-alpha:** 0.0066610907906692

---

---

222

- **AF ID:** AF-O14628-F1-model-v4 | **Chain:** A
- **b-phipsi:** 0.0399355865203917
- **w-rdist:** 0.7709624192345453
- **t-alpha:** 0.0049877163597098

---

---

223

- **AF ID:** AF-P53621-F1-model-v4 | **Chain:** A
- **b-phipsi:** 0.0042405592884209
- **w-rdist:** 1.222287415063216
- **t-alpha:** 0.0339123357766464

---

---

224

- **AF ID:** AF-P05423-F1-model-v4 | **Chain:** A
- **b-phipsi:** 0.069115242131767
- **w-rdist:** 0.2756127795328486
- **t-alpha:** 0.0980929580625606

---

---

225

- **AF ID:** AF-Q5T3U5-F1-model-v4 | **Chain:** A
- **b-phipsi:** 0.0884884201267201
- **w-rdist:** 0.2334867341458542
- **t-alpha:** 0.0699114965509424

---

---

226

- **AF ID:** AF-Q6UXK2-F1-model-v4 | **Chain:** A
- **b-phipsi:** 0.0812262841764514
- **w-rdist:** 0.3327004984319998
- **t-alpha:** 0.0115797655430185

---

---

227

- **AF ID:** AF-O00566-F1-model-v4 | **Chain:** A
- **b-phipsi:** 0.0357908345387154
- **w-rdist:** 1.219119458327352
- **t-alpha:** 0.0033085120789013

---

---

228

- **AF ID:** AF-P19634-F1-model-v4 | **Chain:** A
- **b-phipsi:** 0.0696065472448716
- **w-rdist:** 0.7328474997170961
- **t-alpha:** 0.0008281320187666

---

---

229

- **AF ID:** AF-Q9BUJ2-F1-model-v4 | **Chain:** A
- **b-phipsi:** 0.0621838784682572
- **w-rdist:** 0.2567917576668907
- **t-alpha:** 0.1695617260830908

---

---

230

- **AF ID:** AF-Q8WZ42-F42-model-v4 | **Chain:** A
- **b-phipsi:** 0.0371802444357674
- **w-rdist:** 1.3836748156777092
- **t-alpha:** 0.0024811957952362

---

---

231

- **AF ID:** AF-Q8NFZ4-F1-model-v4 | **Chain:** A
- **b-phipsi:** 0.0309878337169467
- **w-rdist:** 0.9613637617950684
- **t-alpha:** 0.0057897637424946

---

---

232

- **AF ID:** AF-O14522-F1-model-v4 | **Chain:** A
- **b-phipsi:** 0.0016024778586173
- **w-rdist:** 1.3109522273308714
- **t-alpha:** 0.0488003627713593

---

---

233

- **AF ID:** AF-O14709-F1-model-v4 | **Chain:** A
- **b-phipsi:** 0.0270088577881377
- **w-rdist:** 1.2812154531201343
- **t-alpha:** 0.0049877163597098

---

---

234

- **AF ID:** AF-Q96C45-F1-model-v4 | **Chain:** A
- **b-phipsi:** 0.0826335396526795
- **w-rdist:** 0.3350962457445806
- **t-alpha:** 0.0100253538396266

---

---

235

- **AF ID:** AF-Q63HQ2-F1-model-v4 | **Chain:** A
- **b-phipsi:** 0.0305100641905469
- **w-rdist:** 0.3641008861040751
- **t-alpha:** 0.1772153079970764

---

---

236

- **AF ID:** AF-Q9C009-F1-model-v4 | **Chain:** A
- **b-phipsi:** 0.10940409823997
- **w-rdist:** 0.5464375621214794
- **t-alpha:** 0.0008271775551371

---

---

237

- **AF ID:** AF-Q8IX90-F1-model-v4 | **Chain:** A
- **b-phipsi:** 0.0422927556476348
- **w-rdist:** 0.7171476110073711
- **t-alpha:** 0.0058238157721357

---

---

238

- **AF ID:** AF-Q8IXW5-F1-model-v4 | **Chain:** A
- **b-phipsi:** 0.0608593768758301
- **w-rdist:** 0.3651544005295576
- **t-alpha:** 0.0359899834238583

---

---

239

- **AF ID:** AF-Q96I24-F1-model-v4 | **Chain:** A
- **b-phipsi:** 0.0566401179724806
- **w-rdist:** 0.769898521994992
- **t-alpha:** 0.0033085120789013

---

---

240

- **AF ID:** AF-Q9ULK0-F1-model-v4 | **Chain:** A
- **b-phipsi:** 0.0208027942818804
- **w-rdist:** 1.2002853407688123
- **t-alpha:** 0.0066610907906692

---

---

241

- **AF ID:** AF-Q16587-F1-model-v4 | **Chain:** A
- **b-phipsi:** 0.0333960277018335
- **w-rdist:** 0.3796638590661433
- **t-alpha:** 0.101093154311656

---

---

242

- **AF ID:** AF-Q9UBP5-F1-model-v4 | **Chain:** A
- **b-phipsi:** 0.0651472676904873
- **w-rdist:** 0.2364085806048867
- **t-alpha:** 0.1749272671970156

---

---

243

- **AF ID:** AF-Q49AA0-F1-model-v4 | **Chain:** A
- **b-phipsi:** 0.0381391743585205
- **w-rdist:** 0.9747795939061812
- **t-alpha:** 0.0041527907791232

---

---

244

- **AF ID:** AF-O60494-F5-model-v4 | **Chain:** A
- **b-phipsi:** 0.0268090229910779
- **w-rdist:** 1.1890740220357527
- **t-alpha:** 0.0058238157721357

---

---

245

- **AF ID:** AF-Q5VYV7-F1-model-v4 | **Chain:** A
- **b-phipsi:** 0.0686318593143246
- **w-rdist:** 0.2699740378563165
- **t-alpha:** 0.1256987023072455

---

---

246

- **AF ID:** AF-Q9BXK5-F1-model-v4 | **Chain:** A
- **b-phipsi:** 0.0618360731199071
- **w-rdist:** 0.3485683692258532
- **t-alpha:** 0.0623900411658351

---

---

247

- **AF ID:** AF-Q15742-F1-model-v4 | **Chain:** A
- **b-phipsi:** 0.0817831681044421
- **w-rdist:** 0.3412521226771614
- **t-alpha:** 0.014888357714047

---

---

248

- **AF ID:** AF-Q15915-F1-model-v4 | **Chain:** A
- **b-phipsi:** 0.0724453712004048
- **w-rdist:** 0.3525462396215563
- **t-alpha:** 0.0223323354003182

---

---

249

- **AF ID:** AF-Q7Z6Z7-F1-model-v4 | **Chain:** A
- **b-phipsi:** 0.0838724717165843
- **w-rdist:** 0.4887846292810388
- **t-alpha:** 0.0033085120789013

---

---

250

- **AF ID:** AF-Q8N365-F1-model-v4 | **Chain:** A
- **b-phipsi:** 0.0614352885425813
- **w-rdist:** 0.3402498277302257
- **t-alpha:** 0.0833334756581622

---

---

251

- **AF ID:** AF-P98088-F3-model-v4 | **Chain:** A
- **b-phipsi:** 0.0161706475376137
- **w-rdist:** 0.3789136899598012
- **t-alpha:** 0.3515297598020153

---

---

252

- **AF ID:** AF-Q9NU22-F9-model-v4 | **Chain:** A
- **b-phipsi:** 0.0843444650956196
- **w-rdist:** 0.3309926981254287
- **t-alpha:** 0.0280611759977658

---

---

253

- **AF ID:** AF-Q8N3S3-F1-model-v4 | **Chain:** A
- **b-phipsi:** 0.0570801222170751
- **w-rdist:** 0.378253238521964
- **t-alpha:** 0.0289493438129448

---

---

254

- **AF ID:** AF-Q7Z572-F1-model-v4 | **Chain:** A
- **b-phipsi:** 0.054354651039538
- **w-rdist:** 0.3454085908084441
- **t-alpha:** 0.0970964541482639

---

---

255

- **AF ID:** AF-Q99704-F1-model-v4 | **Chain:** A
- **b-phipsi:** 0.0268863054769772
- **w-rdist:** 0.3802122807704232
- **t-alpha:** 0.1864573740074866

---

---

256

- **AF ID:** AF-Q96BF6-F1-model-v4 | **Chain:** A
- **b-phipsi:** 0.0672369593575027
- **w-rdist:** 0.6734290943855078
- **t-alpha:** 0.0033195156791741

---

---

257

- **AF ID:** AF-Q8NEM1-F1-model-v4 | **Chain:** A
- **b-phipsi:** 0.0463374551221811
- **w-rdist:** 0.8189269327103891
- **t-alpha:** 0.0049877163597098

---

---

258

- **AF ID:** AF-P20333-F1-model-v4 | **Chain:** A
- **b-phipsi:** 0.0494284427804645
- **w-rdist:** 0.3704673003097569
- **t-alpha:** 0.0794645485926308

---

---

259

- **AF ID:** AF-P48165-F1-model-v4 | **Chain:** A
- **b-phipsi:** 0.081442976381264
- **w-rdist:** 0.2612708555722334
- **t-alpha:** 0.1061298169835074

---

---

260

- **AF ID:** AF-Q8TD57-F5-model-v4 | **Chain:** A
- **b-phipsi:** 0.0730540967274421
- **w-rdist:** 0.3413048393741548
- **t-alpha:** 0.0531362262599823

---

---

261

- **AF ID:** AF-Q9BRS8-F1-model-v4 | **Chain:** A
- **b-phipsi:** 0.0754996358870287
- **w-rdist:** 0.6437970864031776
- **t-alpha:** 0.0033085120789013

---

---

262

- **AF ID:** AF-P21127-F1-model-v4 | **Chain:** A
- **b-phipsi:** 0.0624189967140911
- **w-rdist:** 0.6551587764062623
- **t-alpha:** 0.0049627974633414

---

---

263

- **AF ID:** AF-P41226-F1-model-v4 | **Chain:** A
- **b-phipsi:** 0.0283421304851463
- **w-rdist:** 1.1263612586417378
- **t-alpha:** 0.0066169305252397

---

---

264

- **AF ID:** AF-Q8WU79-F1-model-v4 | **Chain:** A
- **b-phipsi:** 0.0866481061808175
- **w-rdist:** 0.603335435435654
- **t-alpha:** 0.0024878881015579

---

---

265

- **AF ID:** AF-Q15477-F1-model-v4 | **Chain:** A
- **b-phipsi:** 0.0359329106824994
- **w-rdist:** 0.3651405017541945
- **t-alpha:** 0.1761784085956881

---

---

266

- **AF ID:** AF-Q9NRH2-F1-model-v4 | **Chain:** A
- **b-phipsi:** 0.0748441560879999
- **w-rdist:** 0.2660406336472113
- **t-alpha:** 0.1306861591014545

---

---

267

- **AF ID:** AF-Q9UPZ9-F1-model-v4 | **Chain:** A
- **b-phipsi:** 0.0616794578628582
- **w-rdist:** 0.5244217410594092
- **t-alpha:** 0.0066169305252397

---

---

268

- **AF ID:** AF-Q9BYV6-F1-model-v4 | **Chain:** A
- **b-phipsi:** 0.0544227229305665
- **w-rdist:** 1.0412422584038057
- **t-alpha:** 0.0024878881015579

---

---

269

- **AF ID:** AF-Q9NY43-F1-model-v4 | **Chain:** A
- **b-phipsi:** 0.1084828186484679
- **w-rdist:** 0.5779294439192195
- **t-alpha:** 0.0016569498401739

---

---

270

- **AF ID:** AF-Q9Y2J0-F1-model-v4 | **Chain:** A
- **b-phipsi:** 0.054692938725874
- **w-rdist:** 0.3874302199229951
- **t-alpha:** 0.0289493438129448

---

---

271

- **AF ID:** AF-P86452-F1-model-v4 | **Chain:** A
- **b-phipsi:** 0.0473928738235684
- **w-rdist:** 0.3452605260786196
- **t-alpha:** 0.1604628912780943

---

---

272

- **AF ID:** AF-P41212-F1-model-v4 | **Chain:** A
- **b-phipsi:** 0.0549621591169959
- **w-rdist:** 0.3782283218426196
- **t-alpha:** 0.0522196547392181

---

---

273

- **AF ID:** AF-Q8N264-F1-model-v4 | **Chain:** A
- **b-phipsi:** 0.0690070010106364
- **w-rdist:** 0.2805840926126036
- **t-alpha:** 0.1695617260830908

---

---

274

- **AF ID:** AF-Q8N4H0-F1-model-v4 | **Chain:** A
- **b-phipsi:** 0.099329616978502
- **w-rdist:** 0.2815319306312095
- **t-alpha:** 0.077540150779898

---

---

275

- **AF ID:** AF-Q8TE73-F8-model-v4 | **Chain:** A
- **b-phipsi:** 0.0767898958494012
- **w-rdist:** 0.2967755497062173
- **t-alpha:** 0.1051191692070554

---

---

276

- **AF ID:** AF-Q9Y222-F1-model-v4 | **Chain:** A
- **b-phipsi:** 0.065099797405085
- **w-rdist:** 0.3715622606592948
- **t-alpha:** 0.0380480467994648

---

---

277

- **AF ID:** AF-Q2VWP7-F1-model-v4 | **Chain:** A
- **b-phipsi:** 0.0619889118449584
- **w-rdist:** 0.9411778025411772
- **t-alpha:** 0.0024878881015579

---

---

278

- **AF ID:** AF-Q9NW68-F1-model-v4 | **Chain:** A
- **b-phipsi:** 0.0691670708465966
- **w-rdist:** 0.7225043896696204
- **t-alpha:** 0.0033195156791741

---

---

279

- **AF ID:** AF-P33527-F1-model-v4 | **Chain:** A
- **b-phipsi:** 0.0749977431932022
- **w-rdist:** 0.3205997792157542
- **t-alpha:** 0.0901716732019928

---

---

280

- **AF ID:** AF-Q8TF68-F1-model-v4 | **Chain:** A
- **b-phipsi:** 0.0459109322974558
- **w-rdist:** 0.7982873700622969
- **t-alpha:** 0.0066169305252397

---

---

281

- **AF ID:** AF-Q8TE73-F11-model-v4 | **Chain:** A
- **b-phipsi:** 0.0673387875157331
- **w-rdist:** 0.6987885831207027
- **t-alpha:** 0.0049627974633414

---

---

282

- **AF ID:** AF-Q9UEW3-F1-model-v4 | **Chain:** A
- **b-phipsi:** 0.0521795288916114
- **w-rdist:** 1.0530988835376225
- **t-alpha:** 0.0041355535019986

---

---

283

- **AF ID:** AF-Q7Z494-F1-model-v4 | **Chain:** A
- **b-phipsi:** 0.0773087771348871
- **w-rdist:** 0.3352517728679739
- **t-alpha:** 0.0711330386749458

---

---

284

- **AF ID:** AF-Q9BXL6-F1-model-v4 | **Chain:** A
- **b-phipsi:** 0.03500042762767
- **w-rdist:** 1.2065782169340602
- **t-alpha:** 0.0058238157721357

---

---

285

- **AF ID:** AF-Q8N2C7-F6-model-v4 | **Chain:** A
- **b-phipsi:** 0.0540786198384452
- **w-rdist:** 0.7641726012450258
- **t-alpha:** 0.0058238157721357

---

---

286

- **AF ID:** AF-A8MTY0-F1-model-v4 | **Chain:** A
- **b-phipsi:** 0.0505910989005765
- **w-rdist:** 0.3565725145313404
- **t-alpha:** 0.1513645667819287

---

---

287

- **AF ID:** AF-Q15751-F4-model-v4 | **Chain:** A
- **b-phipsi:** 0.053968781535505
- **w-rdist:** 0.875406479267659
- **t-alpha:** 0.0049877163597098

---

---

288

- **AF ID:** AF-D6RGH6-F1-model-v4 | **Chain:** A
- **b-phipsi:** 0.0395284489261036
- **w-rdist:** 0.3659686778957591
- **t-alpha:** 0.2053838882928957

---

---

289

- **AF ID:** AF-Q6ZUT9-F1-model-v4 | **Chain:** A
- **b-phipsi:** 0.0469077428295238
- **w-rdist:** 0.3671548800437539
- **t-alpha:** 0.1330836156472754

---

---

290

- **AF ID:** AF-Q86VP3-F1-model-v4 | **Chain:** A
- **b-phipsi:** 0.0591286582575233
- **w-rdist:** 0.3205572772078959
- **t-alpha:** 0.2026464892168518

---

---

291

- **AF ID:** AF-Q03933-F1-model-v4 | **Chain:** A
- **b-phipsi:** 0.1043474754386342
- **w-rdist:** 0.6374286147154538
- **t-alpha:** 0.0024878881015579

---

---

292

- **AF ID:** AF-Q9NS91-F1-model-v4 | **Chain:** A
- **b-phipsi:** 0.0773787131323602
- **w-rdist:** 0.2604862335127247
- **t-alpha:** 0.1772153079970764

---

---

293

- **AF ID:** AF-Q8IY67-F1-model-v4 | **Chain:** A
- **b-phipsi:** 0.085254194633641
- **w-rdist:** 0.319087165734884
- **t-alpha:** 0.083540124827504

---

---

294

- **AF ID:** AF-Q9P232-F1-model-v4 | **Chain:** A
- **b-phipsi:** 0.035570608058533
- **w-rdist:** 1.2659420429509198
- **t-alpha:** 0.0058238157721357

---

---

295

- **AF ID:** AF-Q8NHW3-F1-model-v4 | **Chain:** A
- **b-phipsi:** 0.0855082735112745
- **w-rdist:** 0.6182744271908834
- **t-alpha:** 0.0041527907791232

---

---

296

- **AF ID:** AF-Q96DT5-F10-model-v4 | **Chain:** A
- **b-phipsi:** 0.0688631584207134
- **w-rdist:** 0.3721196030013586
- **t-alpha:** 0.0558952654697204

---

---

297

- **AF ID:** AF-Q9NZJ4-F5-model-v4 | **Chain:** A
- **b-phipsi:** 0.0412581559612182
- **w-rdist:** 1.2493308620446908
- **t-alpha:** 0.0057897637424946

---

---

298

- **AF ID:** AF-Q92879-F1-model-v4 | **Chain:** A
- **b-phipsi:** 0.0642554507667077
- **w-rdist:** 1.0187490466434306
- **t-alpha:** 0.0033085120789013

---

---

299

- **AF ID:** AF-Q96MA1-F1-model-v4 | **Chain:** A
- **b-phipsi:** 0.0643341785351668
- **w-rdist:** 0.3131240385590096
- **t-alpha:** 0.2399998462757295

---

---

300

- **AF ID:** AF-Q9UHI6-F1-model-v4 | **Chain:** A
- **b-phipsi:** 0.0712858602187219
- **w-rdist:** 0.3177536508369842
- **t-alpha:** 0.163771663162388

---

---

301

- **AF ID:** AF-Q8N9Z9-F1-model-v4 | **Chain:** A
- **b-phipsi:** 0.0454422461813886
- **w-rdist:** 0.3565243120215235
- **t-alpha:** 0.2902882543062948

---

---

302

- **AF ID:** AF-Q9BVR0-F1-model-v4 | **Chain:** A
- **b-phipsi:** 0.0530433849251781
- **w-rdist:** 0.3625197892253897
- **t-alpha:** 0.169245558317908

---

---

303

- **AF ID:** AF-O94906-F1-model-v4 | **Chain:** A
- **b-phipsi:** 0.1233740015790687
- **w-rdist:** 0.8154689003922699
- **t-alpha:** 0.0008271775551371

---

---

304

- **AF ID:** AF-O00408-F1-model-v4 | **Chain:** A
- **b-phipsi:** 0.0553289106969365
- **w-rdist:** 0.3754721431961095
- **t-alpha:** 0.0951087693785319

---

---

305

- **AF ID:** AF-P78314-F1-model-v4 | **Chain:** A
- **b-phipsi:** 0.0658058440533383
- **w-rdist:** 0.3353486899253522
- **t-alpha:** 0.1662531754750364

---

---

306

- **AF ID:** AF-Q8IU81-F1-model-v4 | **Chain:** A
- **b-phipsi:** 0.0857845357695359
- **w-rdist:** 0.3697001567860911
- **t-alpha:** 0.0272952510798727

---

---

307

- **AF ID:** AF-P0CJ86-F1-model-v4 | **Chain:** A
- **b-phipsi:** 0.0749445335397203
- **w-rdist:** 0.5138958973384268
- **t-alpha:** 0.0066610907906692

---

---

308

- **AF ID:** AF-Q86VR2-F1-model-v4 | **Chain:** A
- **b-phipsi:** 0.0693534225898539
- **w-rdist:** 0.5351338667534058
- **t-alpha:** 0.0074438168005386

---

---

309

- **AF ID:** AF-Q9UN30-F1-model-v4 | **Chain:** A
- **b-phipsi:** 0.0464944481681263
- **w-rdist:** 0.3485311107819599
- **t-alpha:** 0.3285715562805054

---

---

310

- **AF ID:** AF-Q12796-F1-model-v4 | **Chain:** A
- **b-phipsi:** 0.099797347147152
- **w-rdist:** 0.7201121465025573
- **t-alpha:** 0.0024878881015579

---

---

311

- **AF ID:** AF-Q96N23-F2-model-v4 | **Chain:** A
- **b-phipsi:** 0.0851749587913519
- **w-rdist:** 0.5562931874239813
- **t-alpha:** 0.0057897637424946

---

---

312

- **AF ID:** AF-Q99501-F1-model-v4 | **Chain:** A
- **b-phipsi:** 0.0519141185714239
- **w-rdist:** 0.369196138198493
- **t-alpha:** 0.1563273326768577

---

---

313

- **AF ID:** AF-Q8WUQ7-F1-model-v4 | **Chain:** A
- **b-phipsi:** 0.0421594643536525
- **w-rdist:** 1.325674151289554
- **t-alpha:** 0.0057897637424946

---

---

314

- **AF ID:** AF-P21359-F9-model-v4 | **Chain:** A
- **b-phipsi:** 0.0392236848553044
- **w-rdist:** 0.3825572363630156
- **t-alpha:** 0.1761784085956881

---

---

315

- **AF ID:** AF-Q9NS37-F1-model-v4 | **Chain:** A
- **b-phipsi:** 0.0763783184858867
- **w-rdist:** 0.2921483559125469
- **t-alpha:** 0.2041832135621017

---

---

316

- **AF ID:** AF-Q05DH4-F1-model-v4 | **Chain:** A
- **b-phipsi:** 0.0519472125122347
- **w-rdist:** 0.3736277148202603
- **t-alpha:** 0.1248965096909142

---

---

317

- **AF ID:** AF-Q86YH2-F1-model-v4 | **Chain:** A
- **b-phipsi:** 0.0674510599630012
- **w-rdist:** 0.8156503036995131
- **t-alpha:** 0.0049627974633414

---

---

318

- **AF ID:** AF-Q92738-F1-model-v4 | **Chain:** A
- **b-phipsi:** 0.0575100133147795
- **w-rdist:** 0.3271079250709838
- **t-alpha:** 0.3143097396357464

---

---

319

- **AF ID:** AF-Q09428-F1-model-v4 | **Chain:** A
- **b-phipsi:** 0.0901574986906015
- **w-rdist:** 0.3643556298227621
- **t-alpha:** 0.0377684208359867

---

---

320

- **AF ID:** AF-P22105-F3-model-v4 | **Chain:** A
- **b-phipsi:** 0.0679828875217731
- **w-rdist:** 1.1623228500058689
- **t-alpha:** 0.0024878881015579

---

---

321

- **AF ID:** AF-Q5TBA9-F5-model-v4 | **Chain:** A
- **b-phipsi:** 0.0584709952368344
- **w-rdist:** 1.0687669322171305
- **t-alpha:** 0.0041527907791232

---

---

322

- **AF ID:** AF-Q8WZ42-F74-model-v4 | **Chain:** A
- **b-phipsi:** 0.0428101614860389
- **w-rdist:** 1.3485129605128778
- **t-alpha:** 0.0057897637424946

---

---

323

- **AF ID:** AF-Q5T953-F1-model-v4 | **Chain:** A
- **b-phipsi:** 0.0883545019024665
- **w-rdist:** 0.4582346642252015
- **t-alpha:** 0.0066169305252397

---

---

324

- **AF ID:** AF-Q6ZN84-F1-model-v4 | **Chain:** A
- **b-phipsi:** 0.0567783289426331
- **w-rdist:** 0.9410079552567716
- **t-alpha:** 0.0057897637424946

---

---

325

- **AF ID:** AF-Q14D04-F1-model-v4 | **Chain:** A
- **b-phipsi:** 0.0461556610603566
- **w-rdist:** 0.966133297994019
- **t-alpha:** 0.0066610907906692

---

---

326

- **AF ID:** AF-Q8N612-F1-model-v4 | **Chain:** A
- **b-phipsi:** 0.0666995439462259
- **w-rdist:** 0.7159852487448138
- **t-alpha:** 0.0066169305252397

---

---

327

- **AF ID:** AF-Q8WXX0-F4-model-v4 | **Chain:** A
- **b-phipsi:** 0.1052410266695568
- **w-rdist:** 0.7974669715381643
- **t-alpha:** 0.0024811957952362

---

---

328

- **AF ID:** AF-Q99558-F1-model-v4 | **Chain:** A
- **b-phipsi:** 0.0560473780812883
- **w-rdist:** 0.3465303729964361
- **t-alpha:** 0.2332499254674165

---

---

329

- **AF ID:** AF-Q63HN8-F17-model-v4 | **Chain:** A
- **b-phipsi:** 0.0687676413148594
- **w-rdist:** 1.095120111461146
- **t-alpha:** 0.0033085120789013

---

---

330

- **AF ID:** AF-Q9ULE3-F1-model-v4 | **Chain:** A
- **b-phipsi:** 0.0539935989490796
- **w-rdist:** 0.3471831746612487
- **t-alpha:** 0.2928034136839046

---

---

331

- **AF ID:** AF-A0AV02-F1-model-v4 | **Chain:** A
- **b-phipsi:** 0.0760706421054629
- **w-rdist:** 0.8244823060640286
- **t-alpha:** 0.0041355535019986

---

---

332

- **AF ID:** AF-Q8TE82-F1-model-v4 | **Chain:** A
- **b-phipsi:** 0.0772389611248473
- **w-rdist:** 0.6592947690184605
- **t-alpha:** 0.0057897637424946

---

---

333

- **AF ID:** AF-Q9BQ39-F1-model-v4 | **Chain:** A
- **b-phipsi:** 0.0754398124806158
- **w-rdist:** 0.9240566119893916
- **t-alpha:** 0.0033195156791741

---

---

334

- **AF ID:** AF-Q13283-F1-model-v4 | **Chain:** A
- **b-phipsi:** 0.0696792593396906
- **w-rdist:** 0.3201451384945373
- **t-alpha:** 0.2077922859940879

---

---

335

- **AF ID:** AF-Q5GH73-F1-model-v4 | **Chain:** A
- **b-phipsi:** 0.0615999846708485
- **w-rdist:** 1.1453549301262855
- **t-alpha:** 0.0041527907791232

---

---

336

- **AF ID:** AF-Q9Y210-F1-model-v4 | **Chain:** A
- **b-phipsi:** 0.1006803060509453
- **w-rdist:** 0.2868365825364242
- **t-alpha:** 0.1703779119274109

---

---

337

- **AF ID:** AF-P33981-F1-model-v4 | **Chain:** A
- **b-phipsi:** 0.0452193545700787
- **w-rdist:** 0.3747555281363712
- **t-alpha:** 0.2415221382376902

---

---

338

- **AF ID:** AF-Q15050-F1-model-v4 | **Chain:** A
- **b-phipsi:** 0.0481585246551523
- **w-rdist:** 0.3672846717447562
- **t-alpha:** 0.2793653269832095

---

---

339

- **AF ID:** AF-Q9NRP7-F1-model-v4 | **Chain:** A
- **b-phipsi:** 0.0760710743855098
- **w-rdist:** 0.3366778523257824
- **t-alpha:** 0.1414391590857324

---

---

340

- **AF ID:** AF-Q9H2C1-F1-model-v4 | **Chain:** A
- **b-phipsi:** 0.0784882332627712
- **w-rdist:** 0.3108981409588898
- **t-alpha:** 0.2175228597157934

---

---

341

- **AF ID:** AF-Q5T4T6-F1-model-v4 | **Chain:** A
- **b-phipsi:** 0.078897293521134
- **w-rdist:** 0.5788818199221428
- **t-alpha:** 0.0066610907906692

---

---

342

- **AF ID:** AF-P21817-F8-model-v4 | **Chain:** A
- **b-phipsi:** 0.0573094170871786
- **w-rdist:** 1.0678889841290409
- **t-alpha:** 0.0057897637424946

---

---

343

- **AF ID:** AF-Q53FE4-F1-model-v4 | **Chain:** A
- **b-phipsi:** 0.1069502897733881
- **w-rdist:** 0.2787573767906491
- **t-alpha:** 0.1946640646099655

---

---

344

- **AF ID:** AF-A6NFA0-F1-model-v4 | **Chain:** A
- **b-phipsi:** 0.0639896262230951
- **w-rdist:** 0.3282725083544531
- **t-alpha:** 0.4009272814583038

---

---

345

- **AF ID:** AF-Q05925-F1-model-v4 | **Chain:** A
- **b-phipsi:** 0.1272619018116654
- **w-rdist:** 0.6546835934197436
- **t-alpha:** 0.0041527907791232

---

---

346

- **AF ID:** AF-P78423-F1-model-v4 | **Chain:** A
- **b-phipsi:** 0.1035070629552051
- **w-rdist:** 0.719128379437984
- **t-alpha:** 0.0041527907791232

---

---

347

- **AF ID:** AF-Q9BZR9-F1-model-v4 | **Chain:** A
- **b-phipsi:** 0.0499590628105304
- **w-rdist:** 1.3411651705379497
- **t-alpha:** 0.0058238157721357

---

---

348

- **AF ID:** AF-Q64ET8-F1-model-v4 | **Chain:** A
- **b-phipsi:** 0.0756848831428486
- **w-rdist:** 0.3133875341667211
- **t-alpha:** 0.3373894058104945

---

---

349

- **AF ID:** AF-O95155-F1-model-v4 | **Chain:** A
- **b-phipsi:** 0.0888361506618955
- **w-rdist:** 0.3375019805057514
- **t-alpha:** 0.1207610347074268

---

---

350

- **AF ID:** AF-Q13671-F1-model-v4 | **Chain:** A
- **b-phipsi:** 0.0664042628233532
- **w-rdist:** 0.8092005822125765
- **t-alpha:** 0.0066610907906692

---

---

351

- **AF ID:** AF-Q96M86-F1-model-v4 | **Chain:** A
- **b-phipsi:** 0.0769952685889632
- **w-rdist:** 1.3565870747044313
- **t-alpha:** 0.0024811957952362

---

---

352

- **AF ID:** AF-O94851-F1-model-v4 | **Chain:** A
- **b-phipsi:** 0.059460557315089
- **w-rdist:** 0.3488667643917342
- **t-alpha:** 0.3631097586902247

---

---

353

- **AF ID:** AF-Q9H2E6-F1-model-v4 | **Chain:** A
- **b-phipsi:** 0.0721322264205129
- **w-rdist:** 0.3299537029924264
- **t-alpha:** 0.3242352879686216

---

---

354

- **AF ID:** AF-Q9C0G6-F4-model-v4 | **Chain:** A
- **b-phipsi:** 0.1207710845324876
- **w-rdist:** 1.143213095911
- **t-alpha:** 0.0008281320187666

---

---

355

- **AF ID:** AF-Q7KZI7-F1-model-v4 | **Chain:** A
- **b-phipsi:** 0.0695540364830912
- **w-rdist:** 0.3499445811762098
- **t-alpha:** 0.1803140466310127

---

---

356

- **AF ID:** AF-Q9UBD0-F1-model-v4 | **Chain:** A
- **b-phipsi:** 0.1012494159742148
- **w-rdist:** 0.310004233269775
- **t-alpha:** 0.1876228190312141

---

---

357

- **AF ID:** AF-A6NGY1-F1-model-v4 | **Chain:** A
- **b-phipsi:** 0.0757280279691294
- **w-rdist:** 0.3287649156153092
- **t-alpha:** 0.2515531018284478

---

---

358

- **AF ID:** AF-Q04724-F1-model-v4 | **Chain:** A
- **b-phipsi:** 0.0728124831454486
- **w-rdist:** 0.3214795726684198
- **t-alpha:** 0.3482212934945294

---

---

359

- **AF ID:** AF-P35453-F1-model-v4 | **Chain:** A
- **b-phipsi:** 0.1255693366015176
- **w-rdist:** 0.5297441130278786
- **t-alpha:** 0.0066169305252397

---

---

360

- **AF ID:** AF-Q8N9T8-F1-model-v4 | **Chain:** A
- **b-phipsi:** 0.0627424031685514
- **w-rdist:** 1.0533500308845118
- **t-alpha:** 0.0058238157721357

---

---

361

- **AF ID:** AF-Q96KN3-F1-model-v4 | **Chain:** A
- **b-phipsi:** 0.0949063258963917
- **w-rdist:** 0.3647767870172799
- **t-alpha:** 0.082363784576418

---

---

362

- **AF ID:** AF-O43186-F1-model-v4 | **Chain:** A
- **b-phipsi:** 0.1212875982653783
- **w-rdist:** 0.3284562567176031
- **t-alpha:** 0.1204821019187498

---

---

363

- **AF ID:** AF-P21359-F5-model-v4 | **Chain:** A
- **b-phipsi:** 0.1208708616871685
- **w-rdist:** 0.3871319678764483
- **t-alpha:** 0.0193931278866674

---

---

364

- **AF ID:** AF-Q96HA4-F1-model-v4 | **Chain:** A
- **b-phipsi:** 0.0953575839470928
- **w-rdist:** 0.372536681093666
- **t-alpha:** 0.0651982118967349

---

---

365

- **AF ID:** AF-E9PJI5-F1-model-v4 | **Chain:** A
- **b-phipsi:** 0.0695012194142338
- **w-rdist:** 0.3457189196707509
- **t-alpha:** 0.2620044642769714

---

---

366

- **AF ID:** AF-Q504T8-F1-model-v4 | **Chain:** A
- **b-phipsi:** 0.061209732336061
- **w-rdist:** 0.3817630667154885
- **t-alpha:** 0.1737865940938503

---

---

367

- **AF ID:** AF-Q0VDD8-F11-model-v4 | **Chain:** A
- **b-phipsi:** 0.0885944690618827
- **w-rdist:** 1.073050626300215
- **t-alpha:** 0.0033085120789013

---

---

368

- **AF ID:** AF-Q96EP5-F1-model-v4 | **Chain:** A
- **b-phipsi:** 0.1196050092322652
- **w-rdist:** 0.3678527693048058
- **t-alpha:** 0.0699114965509424

---

---

369

- **AF ID:** AF-Q5T4S7-F19-model-v4 | **Chain:** A
- **b-phipsi:** 0.0756509613469371
- **w-rdist:** 1.0082780992507685
- **t-alpha:** 0.0049877163597098

---

---

370

- **AF ID:** AF-Q2M3G0-F1-model-v4 | **Chain:** A
- **b-phipsi:** 0.102819548649335
- **w-rdist:** 0.649349418812404
- **t-alpha:** 0.0066169305252397

---

---

371

- **AF ID:** AF-Q9P0L2-F1-model-v4 | **Chain:** A
- **b-phipsi:** 0.0823882027387524
- **w-rdist:** 0.3357273600204238
- **t-alpha:** 0.2299418317226287

---

---

372

- **AF ID:** AF-Q9BW85-F1-model-v4 | **Chain:** A
- **b-phipsi:** 0.05414272601086
- **w-rdist:** 0.3717690243898914
- **t-alpha:** 0.3864677674458421

---

---

373

- **AF ID:** AF-Q9NQ60-F1-model-v4 | **Chain:** A
- **b-phipsi:** 0.0883704391629386
- **w-rdist:** 0.3129647316565612
- **t-alpha:** 0.3754269743943546

---

---

374

- **AF ID:** AF-Q8NFJ8-F1-model-v4 | **Chain:** A
- **b-phipsi:** 0.1372228954798187
- **w-rdist:** 0.6484848628996668
- **t-alpha:** 0.0058238157721357

---

---

375

- **AF ID:** AF-Q8NC51-F1-model-v4 | **Chain:** A
- **b-phipsi:** 0.0930350577995497
- **w-rdist:** 0.9499929433101134
- **t-alpha:** 0.0041527907791232

---

---

376

- **AF ID:** AF-Q96II8-F1-model-v4 | **Chain:** A
- **b-phipsi:** 0.0545678901696202
- **w-rdist:** 0.3822697761597838
- **t-alpha:** 0.2572378371925954

---

---

377

- **AF ID:** AF-Q14106-F1-model-v4 | **Chain:** A
- **b-phipsi:** 0.1268258880115951
- **w-rdist:** 0.3404048115625168
- **t-alpha:** 0.1277983746314754

---

---

378

- **AF ID:** AF-Q8TD57-F8-model-v4 | **Chain:** A
- **b-phipsi:** 0.0707808641362598
- **w-rdist:** 0.3829049155421711
- **t-alpha:** 0.1184088497246806

---

---

379

- **AF ID:** AF-Q9Y6X4-F1-model-v4 | **Chain:** A
- **b-phipsi:** 0.0815752778636078
- **w-rdist:** 0.3568574230956411
- **t-alpha:** 0.1778329439666597

---

---

380

- **AF ID:** AF-Q66K74-F1-model-v4 | **Chain:** A
- **b-phipsi:** 0.0623015231503077
- **w-rdist:** 0.3708190327088517
- **t-alpha:** 0.352357499310703

---

---

381

- **AF ID:** AF-Q96Q15-F3-model-v4 | **Chain:** A
- **b-phipsi:** 0.1534824135331442
- **w-rdist:** 0.3452092805290229
- **t-alpha:** 0.1215212383717661

---

---

382

- **AF ID:** AF-Q8TF76-F1-model-v4 | **Chain:** A
- **b-phipsi:** 0.0769151618273478
- **w-rdist:** 0.353081606935222
- **t-alpha:** 0.2795697096973535

---

---

383

- **AF ID:** AF-Q8NC54-F1-model-v4 | **Chain:** A
- **b-phipsi:** 0.093051632619254
- **w-rdist:** 0.3305788201100362
- **t-alpha:** 0.3198691164988583

---

---

384

- **AF ID:** AF-A0A1B0GV85-F1-model-v4 | **Chain:** A
- **b-phipsi:** 0.0925068841025771
- **w-rdist:** 0.3822091418474385
- **t-alpha:** 0.0921410228992365

---

---

385

- **AF ID:** AF-Q7RTW8-F1-model-v4 | **Chain:** A
- **b-phipsi:** 0.1076609759919529
- **w-rdist:** 1.028378965600228
- **t-alpha:** 0.0041527907791232

---

---

386

- **AF ID:** AF-Q6IEG0-F1-model-v4 | **Chain:** A
- **b-phipsi:** 0.0679468372385674
- **w-rdist:** 0.3672356340662343
- **t-alpha:** 0.4190139578055414

---

---

387

- **AF ID:** AF-A0A2U3TZM8-F1-model-v4 | **Chain:** A
- **b-phipsi:** 0.0786274219975468
- **w-rdist:** 0.3589663675919274
- **t-alpha:** 0.3098594904788534

---

---

388

- **AF ID:** AF-Q9P286-F1-model-v4 | **Chain:** A
- **b-phipsi:** 0.0724673814084918
- **w-rdist:** 0.3714292953584385
- **t-alpha:** 0.3457407678719879

---

---

389

- **AF ID:** AF-Q8IV33-F1-model-v4 | **Chain:** A
- **b-phipsi:** 0.1303793913391732
- **w-rdist:** 0.342280840142777
- **t-alpha:** 0.2361966776299009

---

---

390

- **AF ID:** AF-Q13761-F1-model-v4 | **Chain:** A
- **b-phipsi:** 0.1417038678830518
- **w-rdist:** 0.3784675608962691
- **t-alpha:** 0.106699602988707

---

---

391

- **AF ID:** AF-Q9HC52-F1-model-v4 | **Chain:** A
- **b-phipsi:** 0.0756968848291718
- **w-rdist:** 0.3830139655768608
- **t-alpha:** 0.2053838882928957

---

---

392

- **AF ID:** AF-Q6ZR08-F1-model-v4 | **Chain:** A
- **b-phipsi:** 0.1123361853135307
- **w-rdist:** 1.3477217084339104
- **t-alpha:** 0.0049627974633414

---

---

393

- **AF ID:** AF-Q69YZ2-F1-model-v4 | **Chain:** A
- **b-phipsi:** 0.0818291549491718
- **w-rdist:** 0.3701093052863921
- **t-alpha:** 0.3801368422924622

---

---

394

- **AF ID:** AF-O14836-F1-model-v4 | **Chain:** A
- **b-phipsi:** 0.0932998788799027
- **w-rdist:** 0.3607730626354259
- **t-alpha:** 0.412383552495942

---

---

395

- **AF ID:** AF-Q32MK0-F1-model-v4 | **Chain:** A
- **b-phipsi:** 0.0950909109426533
- **w-rdist:** 0.383818217429919
- **t-alpha:** 0.1761784085956881

---

---

396

- **AF ID:** AF-P48431-F1-model-v4 | **Chain:** A
- **b-phipsi:** 0.1267735525699477
- **w-rdist:** 0.3566084663979826
- **t-alpha:** 0.3478259790250211

---

---

397

- **AF ID:** AF-P0DM63-F1-model-v4 | **Chain:** A
- **b-phipsi:** 0.0937188393364475
- **w-rdist:** 0.3749648109699327
- **t-alpha:** 0.2593750625806321

---

---

398

- **AF ID:** AF-Q96BT1-F1-model-v4 | **Chain:** A
- **b-phipsi:** 0.0919749655375989
- **w-rdist:** 0.3803634785880536
- **t-alpha:** 0.3098594904788534

---

---
