# Supplementary material for: Identifying and profiling structural similarities between Spike of SARS-CoV-2 and other viral or host proteins with Machaon
Source: Commun Biol. 2023 Jul 19;6:752. doi: 10.1038/s42003-023-05076-7 (PMC10356814; doi:10.1038/s42003-023-05076-7)
Supplement: Supplementary file 9 — Supplementary Data 6 [file 42003_2023_5076_MOESM9_ESM.zip › 6VXX_A_whole_alphafold4_dataset/candidates/6VXX_A-merged-h-enriched_report.html]

 

# Structural Comparison Report for 6VXX\_A - whole structures (total: 100)

---

0

- **AF ID:** AF-Q9BZ76-F1-model-v4 | **Chain:** A
- **b-phipsi:** 0.0128986881964576
- **w-rdist:** 0.2630641160311902
- **t-alpha:** 0.0033195156791741

---

---

1

- **AF ID:** AF-P22897-F1-model-v4 | **Chain:** A
- **b-phipsi:** 0.0074002078230839
- **w-rdist:** 0.2318476018340813
- **t-alpha:** 0.0075000236060698

---

---

2

- **AF ID:** AF-Q9Y485-F4-model-v4 | **Chain:** A
- **b-phipsi:** 0.0092444607544551
- **w-rdist:** 0.3180826644451995
- **t-alpha:** 0.0280611759977658

---

---

3

- **AF ID:** AF-Q99575-F1-model-v4 | **Chain:** A
- **b-phipsi:** 0.0135148609119822
- **w-rdist:** 0.4765704272041375
- **t-alpha:** 0.0024811957952362

---

---

4

- **AF ID:** AF-P20742-F1-model-v4 | **Chain:** A
- **b-phipsi:** 0.0069266262025374
- **w-rdist:** 0.3028358782324738
- **t-alpha:** 0.077540150779898

---

---

5

- **AF ID:** AF-Q8NB90-F1-model-v4 | **Chain:** A
- **b-phipsi:** 0.0277978857313025
- **w-rdist:** 0.2426201923276998
- **t-alpha:** 0.0386600222545225

---

---

6

- **AF ID:** AF-A8K2U0-F1-model-v4 | **Chain:** A
- **b-phipsi:** 0.0107187282478843
- **w-rdist:** 0.3194601777679055
- **t-alpha:** 0.0504550203401277

---

---

7

- **AF ID:** AF-Q8NI77-F1-model-v4 | **Chain:** A
- **b-phipsi:** 0.0258205362135034
- **w-rdist:** 0.4199841488961673
- **t-alpha:** 0.0024811957952362

---

---

8

- **AF ID:** AF-Q6PJI9-F1-model-v4 | **Chain:** A
- **b-phipsi:** 0.0073493809499895
- **w-rdist:** 0.7003569145929152
- **t-alpha:** 0.0024878881015579

---

---

9

- **AF ID:** AF-Q6YHK3-F1-model-v4 | **Chain:** A
- **b-phipsi:** 0.0111769060421033
- **w-rdist:** 0.2893456619410336
- **t-alpha:** 0.1051191692070554

---

---

10

- **AF ID:** AF-Q9NQ66-F1-model-v4 | **Chain:** A
- **b-phipsi:** 0.021894092499219
- **w-rdist:** 0.2933403203265313
- **t-alpha:** 0.0694789568304672

---

---

11

- **AF ID:** AF-Q9Y4D8-F8-model-v4 | **Chain:** A
- **b-phipsi:** 0.0161728030047133
- **w-rdist:** 0.2791306470394897
- **t-alpha:** 0.0931283345367748

---

---

12

- **AF ID:** AF-Q6ZRI0-F1-model-v4 | **Chain:** A
- **b-phipsi:** 0.0081128077876909
- **w-rdist:** 0.3419492517614985
- **t-alpha:** 0.068651600081727

---

---

13

- **AF ID:** AF-Q53GL0-F1-model-v4 | **Chain:** A
- **b-phipsi:** 0.0217367148005146
- **w-rdist:** 0.2806283953542755
- **t-alpha:** 0.0921410228992365

---

---

14

- **AF ID:** AF-P08648-F1-model-v4 | **Chain:** A
- **b-phipsi:** 0.0247429531384978
- **w-rdist:** 0.313121848352471
- **t-alpha:** 0.0540540988034132

---

---

15

- **AF ID:** AF-P63132-F1-model-v4 | **Chain:** A
- **b-phipsi:** 0.0098134070014597
- **w-rdist:** 0.4516810519195361
- **t-alpha:** 0.0057897637424946

---

---

16

- **AF ID:** AF-Q9UBG0-F1-model-v4 | **Chain:** A
- **b-phipsi:** 0.0088864282121855
- **w-rdist:** 0.6382517611760044
- **t-alpha:** 0.0041355535019986

---

---

17

- **AF ID:** AF-Q76MJ5-F1-model-v4 | **Chain:** A
- **b-phipsi:** 0.0093457400212479
- **w-rdist:** 0.9201996120726438
- **t-alpha:** 0.0008271775551371

---

---

18

- **AF ID:** AF-Q9HDB9-F1-model-v4 | **Chain:** A
- **b-phipsi:** 0.036095432075401
- **w-rdist:** 0.3206520118890295
- **t-alpha:** 0.0058238157721357

---

---

19

- **AF ID:** AF-Q9H3P2-F1-model-v4 | **Chain:** A
- **b-phipsi:** 0.0347676334842491
- **w-rdist:** 0.5122998240292116
- **t-alpha:** 0.0008271775551371

---

---

20

- **AF ID:** AF-Q9UGP8-F1-model-v4 | **Chain:** A
- **b-phipsi:** 0.0352520650130213
- **w-rdist:** 0.3129500257038799
- **t-alpha:** 0.0083406172007201

---

---

21

- **AF ID:** AF-Q9NR09-F7-model-v4 | **Chain:** A
- **b-phipsi:** 0.0308871945124606
- **w-rdist:** 0.5567455779461193
- **t-alpha:** 0.0008281320187666

---

---

22

- **AF ID:** AF-O00499-F1-model-v4 | **Chain:** A
- **b-phipsi:** 0.0358075354831282
- **w-rdist:** 0.4260374721491091
- **t-alpha:** 0.0008281320187666

---

---

23

- **AF ID:** AF-Q15311-F1-model-v4 | **Chain:** A
- **b-phipsi:** 0.0357231537751166
- **w-rdist:** 0.4478029048804142
- **t-alpha:** 0.0008281320187666

---

---

24

- **AF ID:** AF-P01031-F1-model-v4 | **Chain:** A
- **b-phipsi:** 0.0076500121859307
- **w-rdist:** 0.3436953726557255
- **t-alpha:** 0.082363784576418

---

---

25

- **AF ID:** AF-Q7Z5R6-F1-model-v4 | **Chain:** A
- **b-phipsi:** 0.01805598575724
- **w-rdist:** 0.703595477208572
- **t-alpha:** 0.0024811957952362

---

---

26

- **AF ID:** AF-Q7Z5H3-F1-model-v4 | **Chain:** A
- **b-phipsi:** 0.0424970408772335
- **w-rdist:** 0.2612880979691155
- **t-alpha:** 0.0202533741750159

---

---

27

- **AF ID:** AF-Q6IE37-F1-model-v4 | **Chain:** A
- **b-phipsi:** 0.0095827417049574
- **w-rdist:** 0.3588675568726708
- **t-alpha:** 0.0531362262599823

---

---

28

- **AF ID:** AF-P22607-F1-model-v4 | **Chain:** A
- **b-phipsi:** 0.016824433019424
- **w-rdist:** 0.7657289250462624
- **t-alpha:** 0.0016569498401739

---

---

29

- **AF ID:** AF-Q8TDJ6-F4-model-v4 | **Chain:** A
- **b-phipsi:** 0.0111326467069971
- **w-rdist:** 0.3715768529734896
- **t-alpha:** 0.0271879789729934

---

---

30

- **AF ID:** AF-Q9Y4C8-F1-model-v4 | **Chain:** A
- **b-phipsi:** 0.0319781405929951
- **w-rdist:** 0.6239888173088579
- **t-alpha:** 0.0008271775551371

---

---

31

- **AF ID:** AF-Q96K21-F1-model-v4 | **Chain:** A
- **b-phipsi:** 0.0246497061027511
- **w-rdist:** 0.5754919101682674
- **t-alpha:** 0.0033085120789013

---

---

32

- **AF ID:** AF-P55884-F1-model-v4 | **Chain:** A
- **b-phipsi:** 0.0212348607081577
- **w-rdist:** 0.3172898775688429
- **t-alpha:** 0.083540124827504

---

---

33

- **AF ID:** AF-Q9UHD8-F1-model-v4 | **Chain:** A
- **b-phipsi:** 0.0463493245040567
- **w-rdist:** 0.4495919453976698
- **t-alpha:** 0.0

---

---

34

- **AF ID:** AF-O75976-F1-model-v4 | **Chain:** A
- **b-phipsi:** 0.0257594679110686
- **w-rdist:** 0.2938559201213908
- **t-alpha:** 0.0934656315674258

---

---

35

- **AF ID:** AF-Q76KP1-F1-model-v4 | **Chain:** A
- **b-phipsi:** 0.0312639189606043
- **w-rdist:** 0.294876459620073
- **t-alpha:** 0.0678243742037618

---

---

36

- **AF ID:** AF-Q13585-F1-model-v4 | **Chain:** A
- **b-phipsi:** 0.0470982411284897
- **w-rdist:** 0.2649874139927372
- **t-alpha:** 0.0237089470211593

---

---

37

- **AF ID:** AF-Q6ZS81-F10-model-v4 | **Chain:** A
- **b-phipsi:** 0.0135581477895966
- **w-rdist:** 0.6158688209589748
- **t-alpha:** 0.0057897637424946

---

---

38

- **AF ID:** AF-O60486-F1-model-v4 | **Chain:** A
- **b-phipsi:** 0.0049767053005447
- **w-rdist:** 0.606514777678621
- **t-alpha:** 0.0066610907906692

---

---

39

- **AF ID:** AF-Q9Y6R7-F22-model-v4 | **Chain:** A
- **b-phipsi:** 0.0138684216446042
- **w-rdist:** 0.91543877882685
- **t-alpha:** 0.0024811957952362

---

---

40

- **AF ID:** AF-Q9HCU4-F6-model-v4 | **Chain:** A
- **b-phipsi:** 0.0085538699389184
- **w-rdist:** 0.7471471072443138
- **t-alpha:** 0.0049627974633414

---

---

41

- **AF ID:** AF-O95071-F2-model-v4 | **Chain:** A
- **b-phipsi:** 0.0153010937588536
- **w-rdist:** 0.6395922852941501
- **t-alpha:** 0.0049627974633414

---

---

42

- **AF ID:** AF-Q9P2H5-F1-model-v4 | **Chain:** A
- **b-phipsi:** 0.0508859174903886
- **w-rdist:** 0.2564178591889064
- **t-alpha:** 0.0333332138395487

---

---

43

- **AF ID:** AF-Q86WI1-F15-model-v4 | **Chain:** A
- **b-phipsi:** 0.0151585034922566
- **w-rdist:** 0.3305520820611856
- **t-alpha:** 0.1132600047230165

---

---

44

- **AF ID:** AF-Q16832-F1-model-v4 | **Chain:** A
- **b-phipsi:** 0.0036218160494508
- **w-rdist:** 1.0668868694094509
- **t-alpha:** 0.0033085120789013

---

---

45

- **AF ID:** AF-Q6U841-F1-model-v4 | **Chain:** A
- **b-phipsi:** 0.0275278871116634
- **w-rdist:** 0.3466219420455617
- **t-alpha:** 0.0446646519250406

---

---

46

- **AF ID:** AF-Q9UJY5-F1-model-v4 | **Chain:** A
- **b-phipsi:** 0.0356440069852946
- **w-rdist:** 0.7241635650585256
- **t-alpha:** 0.0

---

---

47

- **AF ID:** AF-P15918-F1-model-v4 | **Chain:** A
- **b-phipsi:** 0.0245165456239836
- **w-rdist:** 0.895356353522929
- **t-alpha:** 0.0016540840807526

---

---

48

- **AF ID:** AF-Q96PQ6-F1-model-v4 | **Chain:** A
- **b-phipsi:** 0.0174451133186787
- **w-rdist:** 0.3539474113832183
- **t-alpha:** 0.0777503200474987

---

---

49

- **AF ID:** AF-Q15751-F1-model-v4 | **Chain:** A
- **b-phipsi:** 0.0365055450984083
- **w-rdist:** 0.2869518259129966
- **t-alpha:** 0.076922726695275

---

---

50

- **AF ID:** AF-Q92736-F7-model-v4 | **Chain:** A
- **b-phipsi:** 0.0294981585080987
- **w-rdist:** 0.828324955243523
- **t-alpha:** 0.0008271775551371

---

---

51

- **AF ID:** AF-Q6ZN44-F1-model-v4 | **Chain:** A
- **b-phipsi:** 0.0255064340221229
- **w-rdist:** 0.3262368099064757
- **t-alpha:** 0.0951197353930601

---

---

52

- **AF ID:** AF-P26012-F1-model-v4 | **Chain:** A
- **b-phipsi:** 0.0137547976985173
- **w-rdist:** 0.3152037144095001
- **t-alpha:** 0.2077922859940879

---

---

53

- **AF ID:** AF-Q8TC27-F1-model-v4 | **Chain:** A
- **b-phipsi:** 0.0257220939782292
- **w-rdist:** 0.7331834048683609
- **t-alpha:** 0.0033085120789013

---

---

54

- **AF ID:** AF-Q8IZQ1-F3-model-v4 | **Chain:** A
- **b-phipsi:** 0.0359349446816221
- **w-rdist:** 0.6620910103038253
- **t-alpha:** 0.0008281320187666

---

---

55

- **AF ID:** AF-Q9Y5B9-F1-model-v4 | **Chain:** A
- **b-phipsi:** 0.0077721852007063
- **w-rdist:** 0.361745273608913
- **t-alpha:** 0.1194444620618173

---

---

56

- **AF ID:** AF-Q15928-F1-model-v4 | **Chain:** A
- **b-phipsi:** 0.034463856665786
- **w-rdist:** 0.6717267913583621
- **t-alpha:** 0.0016569498401739

---

---

57

- **AF ID:** AF-Q8WXG9-F13-model-v4 | **Chain:** A
- **b-phipsi:** 0.0224779590496765
- **w-rdist:** 1.2659190224483043
- **t-alpha:** 0.0

---

---

58

- **AF ID:** AF-P24043-F10-model-v4 | **Chain:** A
- **b-phipsi:** 0.002912180216064
- **w-rdist:** 0.4330939207329795
- **t-alpha:** 0.0736146078897646

---

---

59

- **AF ID:** AF-Q8NEG5-F1-model-v4 | **Chain:** A
- **b-phipsi:** 0.0416148228295413
- **w-rdist:** 0.5566117069508817
- **t-alpha:** 0.0024811957952362

---

---

60

- **AF ID:** AF-Q9H6S3-F1-model-v4 | **Chain:** A
- **b-phipsi:** 0.0161573699047697
- **w-rdist:** 0.9608226806443692
- **t-alpha:** 0.0024878881015579

---

---

61

- **AF ID:** AF-Q9HCG1-F1-model-v4 | **Chain:** A
- **b-phipsi:** 0.0228619234662153
- **w-rdist:** 1.0836160318288428
- **t-alpha:** 0.0008281320187666

---

---

62

- **AF ID:** AF-P0CJ89-F1-model-v4 | **Chain:** A
- **b-phipsi:** 0.0313542508214103
- **w-rdist:** 0.7695094922800007
- **t-alpha:** 0.0016569498401739

---

---

63

- **AF ID:** AF-Q4ADV7-F1-model-v4 | **Chain:** A
- **b-phipsi:** 0.0146440757556432
- **w-rdist:** 0.819849755601865
- **t-alpha:** 0.0041527907791232

---

---

64

- **AF ID:** AF-Q9NPF5-F1-model-v4 | **Chain:** A
- **b-phipsi:** 0.0287881544091671
- **w-rdist:** 0.3299583787406172
- **t-alpha:** 0.0901716732019928

---

---

65

- **AF ID:** AF-Q8NFF5-F1-model-v4 | **Chain:** A
- **b-phipsi:** 0.0156339556084307
- **w-rdist:** 1.199007094590475
- **t-alpha:** 0.0024811957952362

---

---

66

- **AF ID:** AF-P14410-F1-model-v4 | **Chain:** A
- **b-phipsi:** 0.0015596343554912
- **w-rdist:** 0.5206094769287
- **t-alpha:** 0.0727871210352775

---

---

67

- **AF ID:** AF-O75030-F1-model-v4 | **Chain:** A
- **b-phipsi:** 0.0366492172098593
- **w-rdist:** 0.7962808474677763
- **t-alpha:** 0.0

---

---

68

- **AF ID:** AF-Q7L2R6-F1-model-v4 | **Chain:** A
- **b-phipsi:** 0.0322750882707468
- **w-rdist:** 0.8673642135326054
- **t-alpha:** 0.0008271775551371

---

---

69

- **AF ID:** AF-Q86UV5-F1-model-v4 | **Chain:** A
- **b-phipsi:** 0.0193396167880061
- **w-rdist:** 0.5920566506115279
- **t-alpha:** 0.0066610907906692

---

---

70

- **AF ID:** AF-P0CJ85-F1-model-v4 | **Chain:** A
- **b-phipsi:** 0.0440100656056751
- **w-rdist:** 0.5650982099289734
- **t-alpha:** 0.0024878881015579

---

---

71

- **AF ID:** AF-Q6ZNJ1-F7-model-v4 | **Chain:** A
- **b-phipsi:** 0.0275904304880709
- **w-rdist:** 1.2244341537862955
- **t-alpha:** 0.0

---

---

72

- **AF ID:** AF-Q6IE36-F1-model-v4 | **Chain:** A
- **b-phipsi:** 0.013966124207162
- **w-rdist:** 0.3858501860792445
- **t-alpha:** 0.0678243742037618

---

---

73

- **AF ID:** AF-Q0VGE8-F1-model-v4 | **Chain:** A
- **b-phipsi:** 0.0207753193512944
- **w-rdist:** 0.3526071117434504
- **t-alpha:** 0.117375261057294

---

---

74

- **AF ID:** AF-Q9NT68-F7-model-v4 | **Chain:** A
- **b-phipsi:** 0.0083762062763749
- **w-rdist:** 0.7644682916847294
- **t-alpha:** 0.0074438168005386

---

---

75

- **AF ID:** AF-Q9NRL3-F1-model-v4 | **Chain:** A
- **b-phipsi:** 0.0072177300147438
- **w-rdist:** 0.8691180653517001
- **t-alpha:** 0.0066169305252397

---

---

76

- **AF ID:** AF-Q53LP3-F1-model-v4 | **Chain:** A
- **b-phipsi:** 0.0863312878724029
- **w-rdist:** 0.2898271895063253
- **t-alpha:** 0.0049877163597098

---

---

77

- **AF ID:** AF-Q96M86-F8-model-v4 | **Chain:** A
- **b-phipsi:** 0.040138825820451
- **w-rdist:** 0.3040073444671038
- **t-alpha:** 0.0941175821499755

---

---

78

- **AF ID:** AF-Q6ZWH5-F1-model-v4 | **Chain:** A
- **b-phipsi:** 0.0435799667271972
- **w-rdist:** 0.324967205266481
- **t-alpha:** 0.0595534013336387

---

---

79

- **AF ID:** AF-Q8N184-F1-model-v4 | **Chain:** A
- **b-phipsi:** 0.0313187344326802
- **w-rdist:** 0.3694213695578927
- **t-alpha:** 0.034739440546361

---

---

80

- **AF ID:** AF-P11362-F1-model-v4 | **Chain:** A
- **b-phipsi:** 0.0447557381597913
- **w-rdist:** 0.2397668180838327
- **t-alpha:** 0.1267475121071146

---

---

81

- **AF ID:** AF-P32314-F1-model-v4 | **Chain:** A
- **b-phipsi:** 0.0505783658388147
- **w-rdist:** 0.4835367398638488
- **t-alpha:** 0.0033085120789013

---

---

82

- **AF ID:** AF-P56730-F1-model-v4 | **Chain:** A
- **b-phipsi:** 0.0288357434130113
- **w-rdist:** 0.700722494290007
- **t-alpha:** 0.0049627974633414

---

---

83

- **AF ID:** AF-Q8TDW7-F14-model-v4 | **Chain:** A
- **b-phipsi:** 0.0243298020528436
- **w-rdist:** 1.3467344419132676
- **t-alpha:** 0.0016540840807526

---

---

84

- **AF ID:** AF-Q6ZR08-F8-model-v4 | **Chain:** A
- **b-phipsi:** 0.066479305273992
- **w-rdist:** 0.4905553215743937
- **t-alpha:** 0.0008271775551371

---

---

85

- **AF ID:** AF-P78509-F3-model-v4 | **Chain:** A
- **b-phipsi:** 0.0292964120721646
- **w-rdist:** 0.9581280163728376
- **t-alpha:** 0.0024811957952362

---

---

86

- **AF ID:** AF-Q5T4S7-F6-model-v4 | **Chain:** A
- **b-phipsi:** 0.0188451579303458
- **w-rdist:** 0.8329892273683652
- **t-alpha:** 0.0049877163597098

---

---

87

- **AF ID:** AF-P25391-F8-model-v4 | **Chain:** A
- **b-phipsi:** 0.0088080116407543
- **w-rdist:** 1.2981576292715162
- **t-alpha:** 0.0041527907791232

---

---

88

- **AF ID:** AF-Q8NI36-F1-model-v4 | **Chain:** A
- **b-phipsi:** 0.0019992048879244
- **w-rdist:** 0.5082195573869013
- **t-alpha:** 0.1081575850073603

---

---

89

- **AF ID:** AF-Q8IZ52-F1-model-v4 | **Chain:** A
- **b-phipsi:** 0.0243023679143861
- **w-rdist:** 0.8722049906925802
- **t-alpha:** 0.0041527907791232

---

---

90

- **AF ID:** AF-Q5TKA1-F1-model-v4 | **Chain:** A
- **b-phipsi:** 0.0291782028939856
- **w-rdist:** 0.9341860318782028
- **t-alpha:** 0.0024878881015579

---

---

91

- **AF ID:** AF-O96013-F1-model-v4 | **Chain:** A
- **b-phipsi:** 0.0388072829456872
- **w-rdist:** 0.9065908264025212
- **t-alpha:** 0.0008271775551371

---

---

92

- **AF ID:** AF-Q12849-F1-model-v4 | **Chain:** A
- **b-phipsi:** 0.0398137474836367
- **w-rdist:** 0.8230993755635947
- **t-alpha:** 0.0008281320187666

---

---

93

- **AF ID:** AF-Q9HBA0-F1-model-v4 | **Chain:** A
- **b-phipsi:** 0.0484055202874076
- **w-rdist:** 0.2574873198597717
- **t-alpha:** 0.117375261057294

---

---

94

- **AF ID:** AF-A6NHJ4-F1-model-v4 | **Chain:** A
- **b-phipsi:** 0.0302658335709262
- **w-rdist:** 0.567093609805526
- **t-alpha:** 0.0066169305252397

---

---

95

- **AF ID:** AF-Q99457-F1-model-v4 | **Chain:** A
- **b-phipsi:** 0.0363276601256349
- **w-rdist:** 0.6265768452227467
- **t-alpha:** 0.0041527907791232

---

---

96

- **AF ID:** AF-Q8N9M1-F1-model-v4 | **Chain:** A
- **b-phipsi:** 0.0634959938237104
- **w-rdist:** 0.5697028917946396
- **t-alpha:** 0.0008271775551371

---

---

97

- **AF ID:** AF-Q96CP6-F1-model-v4 | **Chain:** A
- **b-phipsi:** 0.0366884076680553
- **w-rdist:** 0.358498679569155
- **t-alpha:** 0.0471463143139867

---

---

98

- **AF ID:** AF-P98164-F8-model-v4 | **Chain:** A
- **b-phipsi:** 0.0146424077273261
- **w-rdist:** 0.3708829192899338
- **t-alpha:** 0.1267475121071146

---

---

99

- **AF ID:** AF-Q8TD17-F1-model-v4 | **Chain:** A
- **b-phipsi:** 0.0311374679801386
- **w-rdist:** 0.3779476051227446
- **t-alpha:** 0.039552885392045

---

---
