# Supplementary material for: Identifying and profiling structural similarities between Spike of SARS-CoV-2 and other viral or host proteins with Machaon
Source: Commun Biol. 2023 Jul 19;6:752. doi: 10.1038/s42003-023-05076-7 (PMC10356814; doi:10.1038/s42003-023-05076-7)
Supplement: Supplementary file 12 — Reporting Summary [file 42003_2023_5076_MOESM12_ESM.pdf]

nature portfolio

Corresponding author(s): Ema Anastasiadou

Last updated by author(s): Jun 26, 2023

Reporting Summary

Nature Portfolio wishes to improve the reproducibility of the work that we publish. This form provides structure for consistency and transparency in reporting. For further information on Nature Portfolio policies, see our Editorial Policies and the Editorial Policy Checklist.

Statistics

For all statistical analyses, confirm that the following items are present in the figure legend, table legend, main text, or Methods section.

n/a	Confirmed	
☐	☒	The exact sample size (*n*) for each experimental group/condition, given as a discrete number and unit of measurement
☐	☒	A statement on whether measurements were taken from distinct samples or whether the same sample was measured repeatedly
☒	☐	The statistical test(s) used AND whether they are one- or two-sided<br>*Only common tests should be described solely by name; describe more complex techniques in the Methods section.*
☐	☒	A description of all covariates tested
☒	☐	A description of any assumptions or corrections, such as tests of normality and adjustment for multiple comparisons
☐	☒	A full description of the statistical parameters including central tendency (e.g. means) or other basic estimates (e.g. regression coefficient) AND variation (e.g. standard deviation) or associated estimates of uncertainty (e.g. confidence intervals)
☒	☐	For null hypothesis testing, the test statistic (e.g. *F*, *t*, *r*) with confidence intervals, effect sizes, degrees of freedom and *P* value noted<br>*Give P values as exact values whenever suitable.*
☒	☐	For Bayesian analysis, information on the choice of priors and Markov chain Monte Carlo settings
☒	☐	For hierarchical and complex designs, identification of the appropriate level for tests and full reporting of outcomes
☒	☐	Estimates of effect sizes (e.g. Cohen's *d*, Pearson's *r*), indicating how they were calculated

Our web collection on statistics for biologists contains articles on many of the points above.

Software and code

Policy information about availability of computer code

Data collection

Machaon's implementation and evaluation scripts are available to the community at: https://github.com/anastasiadoulab/machaon .
MachaonWeb's implementation is also available at: https://github.com/anastasiadoulab/machaonweb .
The computed features on the PDB files in Spike protein structural comparisons for the viral dataset are available at https://zenodo.org/record/6654658 .

Data analysis

Machaon was implemented in Python v.3.8 (https://www.python.org/) and uses the following packages: NumPy (https://github.com/numpy/numpy), pandas (https://github.com/pandas-dev/pandas), SciPy (https://www.scipy.org/), scikit-learn (https://github.com/scikit-learn/scikit-learn), Matplotlib (https://github.com/matplotlib/matplotlib), seaborn (https://github.com/mwaskom/seaborn), BioPython (https://github.com/biopython/biopython), RDKit (https://github.com/rdkit/rdkit), Open3D (https://github.com/isl-org/Open3D), HDBScan (https://github.com/scikit-learn-contrib/hdbscan), ranky (https://github.com/Didayolo/ranky), DSSPParser (https://github.com/neolei/DSSPparser), Pebble (https://github.com/noxdafox/pebble), protobuf (https://github.com/protocolbuffers/protobuf), lxml (https://github.com/lxml/lxml), networkx (https://github.com/networkx/networkx), Plotly (https://github.com/plotly/plotly.py), UMAP (https://github.com/lmcinnes/umap), word_cloud (https://github.com/amueller/word_cloud), tqdm (https://github.com/tqdm/tqdm), beautiful soup (https://code.launchpad.net/beautifulsoup). A modified version of TM-align (https://zhanglab.dcmb.med.umich.edu/TM-align/, source code retrieved at 10/2/2021) is used by the evaluation model for 3D structure similarity computation. Secondary structures are determined by DSSP (https://github.com/PDB-REDO/dssp).
The distributed computing platform MachaonWeb was implemented in Rust v.1.68 (https://www.rust-lang.org), React Framework v.18.2.0 (https://github.com/facebook/react) and its storage depends on MariaDB v.10.11.2 (https://mariadb.org). It is deployed via Docker (https://www.docker.com) and uses the following packages:
• Rust-based: async-stream (https://github.com/tokio-rs/async-stream), anyhow (https://github.com/dtolnay/anyhow), axum(https://github.com/tokio-rs/axum), axum-server (https://github.com/programatik29/axum-server), chrono (https://github.com/chronotope/chrono),

diesel (https://github.com/diesel-rs/diesel), dotenvy (https://github.com/allan2/dotenvy), futures (https://github.com/rust-lang/futures-rs), glob(https://github.com/rust-lang/glob), prost (https://github.com/tokio-rs/prost), rand(https://github.com/rust-random/rand), regex(https://github.com/rust-lang/regex), reqwest (https://github.com/seanmonstar/reqwest), rustls (https://github.com/rustls/rustls), serde (https://github.com/serde-rs/serde), serde_json(https://github.com/serde-rs/json), sha2 (https://github.com/RustCrypto), tokio (https://github.com/tokio-rs/tokio), tonic (https://github.com/hyperium/tonic), tower (https://github.com/tower-rs/tower), tracing (https://github.com/tokio-rs/tracing), unicode-segmentation (https://github.com/unicode-rs/unicode-segmentation), uuid (https://github.com/uuid-rs/uuid), walkdir (https://github.com/BurntSushi/walkdir)
• Javascript-based: axios (https://github.com/axios/axios), bootstrap (https://github.com/twbs/bootstrap), react-bootstrap (https://github.com/react-bootstrap/react-bootstrap), react-copy-to-clipboard (https://github.com/nkbt/react-copy-to-clipboard), react-ga4 (https://github.com/codler/react-ga4), react-google-recaptcha-v3 (https://github.com/t49tran/react-google-recaptcha-v3), react-router-bootstrap (https://github.com/react-bootstrap/react-router-bootstrap), react-router-dom (https://github.com/remix-run/react-router), react-scripts (https://github.com/facebook/create-react-app), web-vitals (https://github.com/GoogleChrome/web-vitals)
Machaon communicates with MachaonWeb via an mTLS-based gRPC module implemented in Python v.3.8 and uses the Python implementation of gRPC library (https://github.com/grpc/grpc)

For manuscripts utilizing custom algorithms or software that are central to the research but not yet described in published literature, software must be made available to editors and reviewers. We strongly encourage code deposition in a community repository (e.g. GitHub). See the Nature Portfolio guidelines for submitting code & software for further information.

Data

Policy information about availability of data

All manuscripts must include a data availability statement. This statement should provide the following information, where applicable:

- Accession codes, unique identifiers, or web links for publicly available datasets
- A description of any restrictions on data availability
- For clinical datasets or third party data, please ensure that the statement adheres to our policy

Source data from all figures are available in Supplementary Data 1.

The utilized UniProt ID mapping resources were retrieved in 21/9/2021 at: https://ftp.uniprot.org/pub/databases/uniprot/current_release/knowledgebase/idmapping and RefSeq resources were retrieved in 6/11/2021 at: ftp://ftp.ncbi.nlm.nih.gov/refseq/ .
RCSB PDB GraphQL (https://data.rcsb.org/graphql), UniProt (https://www.uniprot.org/uploadlists/, https://www.uniprot.org/uniprot/ACCESSION.xml/<ACCESSION-NUMBER>) and EBI QuickGo (https://www.ebi.ac.uk/QuickGO/services/ontology/go/terms/GO:<termid>) online services are used as a fallback method to retrieve required data which are not present in the local static data sources.

The viral PDB dataset for the comparisons to Spike was retrieved in December 2020 and it was the query result for viral proteins in RCSB (https://www.rcsb.org/docs/programmatic-access/batch-downloads-with-shell-script). The human PDB dataset was assembled in a similar way in 19/6/2022 by querying RCSB PDB for all the available PDBs that contain human proteins. The dataset containing PDB files of the Spike protein was obtained from the same source (RCSB). The predicted human protein dataset corresponds to the predicted human protein by AlphaFold v4 (https://ftp.ebi.ac.uk/pub/databases/alphafold/latest/UP000005640_9606_HUMAN_v4.tar).

Benchmark datasets were obtained from https://github.com/rcsb/biozernike-validation for Task 1, http://shrec2018.drugdesign.fr/shape_retrieval_shrec2018_pdb_files.tar.gz , http://shrec2018.drugdesign.fr/SHREC2018_ref.cla for Task 2 and http://ekhidna2.biocenter.helsinki.fi/dali/pdb_and_scope.tar for Task 3.

Human research participants

Policy information about studies involving human research participants and Sex and Gender in Research.

Reporting on sex and gender	Not applicable because the study does not involve human research participants.
Population characteristics	Not applicable because the study does not involve human research participants.
Recruitment	Not applicable because the study does not involve human research participants.
Ethics oversight	Not applicable because the study does not involve human research participants.

Note that full information on the approval of the study protocol must also be provided in the manuscript.

Field-specific reporting

Please select the one below that is the best fit for your research. If you are not sure, read the appropriate sections before making your selection.

☒ Life sciences ☐ Behavioural & social sciences ☐ Ecological, evolutionary & environmental sciences

For a reference copy of the document with all sections, see nature.com/documents/nr-reporting-summary-flat.pdf

Life sciences study design

All studies must disclose on these points even when the disclosure is negative.

Sample size	The target dataset for Spike protein structure comparisons is a sample from the RCSB PDB that was retrieved as a result of a query in the database for viral protein structures. There are several user-defined presets that affect the size of the target protein structures sample throughout the workflow of the method to support different case studies (see reference about exclusions below and Methods for full details).
Data exclusions	PDB chains in the search space that are malformed (parsing failure), miss a type of atoms (no angle information), contain non-protein data or have a single residue (no distance information) are discarded. They are filtered by organism or gene name according to the preferences of the user. If the total search space size exceeds a threshold then it is reduced to the intersection of the top x% (e.g. 1%) entries by each metric value. If the resulting sample size is more than a preset size, a clustering method fragments the search space and the cluster including the entry with the highest rank is chosen. A minimum resulting sample size is enforced by limited hyperparameter tuning on clustering. The set is further reduced by a threshold before data enrichment for performance. Finally, the remaining entries are filtered by their protein and coding gene identifiers (redundant sets will be severely reduced, hence a looser truncation threshold is preferable before data enrichment, like a limit of maximum 800 entries) and truncated to maximum 100 entries for user convenience. Intermediate snapshots of the samples are stored during the selection process for review. On segment level searches, the search space is initially pruned by mixed representation alignments (see Methods for full details). Figure 5d displays information about a subset of the initial search space sampled by a modulo operation (see figure legend for details).
Replication	Not applicable, no experimental work is described in this study. The results are the output of a computational method which is available to the community.
Randomization	Not applicable, we are not making a comparison between groups.
Blinding	Not applicable, we are not making a comparison between groups.

Reporting for specific materials, systems and methods

We require information from authors about some types of materials, experimental systems and methods used in many studies. Here, indicate whether each material, system or method listed is relevant to your study. If you are not sure if a list item applies to your research, read the appropriate section before selecting a response.

Materials & experimental systems

n/a	Involved in the study	
☒	☐	Antibodies
☒	☐	Eukaryotic cell lines
☒	☐	Palaeontology and archaeology
☒	☐	Animals and other organisms
☒	☐	Clinical data
☒	☐	Dual use research of concern

Methods

n/a	Involved in the study	
☒	☐	ChIP-seq
☒	☐	Flow cytometry
☒	☐	MRI-based neuroimaging
